# ‘Mainland-island’ population structure of a terrestrial salamander in a forest-bocage landscape with little evidence for *in situ* ecological speciation

**DOI:** 10.1038/s41598-020-58551-0

**Published:** 2020-02-03

**Authors:** Jan W. Arntzen, Joep van Belkom

**Affiliations:** Naturalis Biodiversity Center, Leiden, The Netherlands

**Keywords:** Molecular ecology, Population genetics, Speciation, Taxonomy

## Abstract

Adaptation to different ecological environments can, through divergent selection, generate phenotypic and genetic differences between populations, and eventually give rise to new species. The fire salamander (*Salamandra salamandra*) has been proposed to represent an early stage of ecological speciation, driven by differential habitat adaptation through the deposition and development of larvae in streams versus ponds in the Kottenforst near Bonn (Germany). We set out to test this hypothesis of ecological speciation in an area different from the one where it was raised and we took the opportunity to explore for drivers of genetic differentiation at a landscape scale. A survey over 640 localities demonstrated the species’ presence in ponds and streams across forests, hilly terrain and areas with hedgerows (‘bocage’). Genetic variation at 14 microsatellite loci across 41 localities in and around two small deciduous forests showed that salamander effective population sizes were higher in forests than in the bocage, with panmixia in the forests (*F*_st_ < 0.010) versus genetic drift or founder effects in several of the small and more or less isolated bocage populations (*F*_st_ > 0.025). The system fits the ‘mainland-island’ metapopulation model rather than indicating adaptive genetic divergence in pond versus stream larval habitats. A reanalysis of the Kottenforst data indicated that microsatellite genetic variation fitted a geographical rather than an environmental axis, with a sharp transition from a western pond-breeding to an eastern, more frequently stream-breeding group of populations. A parallel changeover in mitochondrial DNA exists but remains to be well documented. The data support the existence of a hybrid zone following secondary contact of differentiated lineages, more so than speciation *in situ*.

## Introduction

Adaptation to different ecological environments can, through divergent selection, generate phenotypic and genetic differences between populations. These changes may eventually give rise to new species. The speciation process is often quantitative in nature, as illustrated by numerous studies showing that divergence during speciation varies continuously, and the sequence of genetically-based changes that occur as two lineages on the pathway to reproductive isolation diverge from one another has been coined the ‘speciation continuum’^1,2^. Divergent evolution and reproductive isolation are the primary elements of the speciation continuum, but many have recognized that reproductive isolation is usually a signature effect rather than a primary cause of speciation. Whereas the mechanisms underlying reproductive isolation are by now mostly well understood (such as natural and sexual selection and genetic drift due to founder events, etc.), biologists continue to struggle with understanding how and why these evolutionary processes cause the disjoined genetic connections that are integral to the emergence of new species, in particular in conditions of sympatry^3–6^. Organisms that are organized in deme-structured metapopulations, with good population sizes and small to moderate dispersal capabilities, such as many amphibian species, are good models for population genetic research and convincing examples involving the early stages of ecological speciation in this group would be particularly welcome. Unfortunately, amphibians also appear particularly prone to severe losses in recent times e.g.^7,8^ and this includes the fire salamander *Salamandra salamandra* Linnaeus, 1758^9,10^.

The fire salamander represents a remarkable study system because from studies in the Kottenforst, near Bonn in Germany, it figures as an example of local ecological and genetic differentiation and adaptation, with pond- and stream-breeding populations possibly representing the first step in the speciation process^11–13^. It would be important to determine whether similar processes can be uncovered in other regions where the species is distributed today. Other regions may serve as replicates despite the fact that each region may have had its own contingent evolutionary specificities. To perform a replicate study, we choose the ‘département’ (department) Mayenne in the west of France where we found the fire salamanders to deposit their larvae in streams as well as in ponds.

The standing genetic variability of populations represents the adaptive potential to changing environmental conditions and acts as a buffer against stochastic and catastrophic events^14,15^. The protection of genetic diversity, along with that of habitats and species *per se* is thus a pillar to nature conservation^16,17^. The landscapes of the French coastal zone represent the archetypal dense hedgerow configuration known as ‘bocage’. From the perspective of biodiversity conservation prime assets of hedgerows are that they act as shelter areas for species unable to exist elsewhere in farmland and that they are usually interconnected into networks which facilitates dispersal across the landscape^18^. The network of hedgerows possibly supports the presence and dispersal of the fire salamander and that of other primarily forest-dwelling species. The Mayenne study area therewith affords the opportunity to explore other landscape processes in this system, such as supported by continuous forest versus a mosaic of hedgerows. It is important to document if species are reliant on the bocage because a decline through the thinning of the hedgerow network could fragment otherwise continuous species distributions. Accordingly, in the present study we aimed to determine whether adaptive divergence in pond- and stream-breeding populations of the fire salamander could be identified in the west of France. We expect genetic differentiation to be more strongly associated with a pond- versus stream-breeding habitat than with the wider geography. A second aim was to quantify the importance of the bocage as a constituent to the fire salamander habitat and to evaluate what role it plays in the population dynamics of the species. Finally, informed by our new findings, we reanalyzed the Kottenforst data in an explicit geographical context.

## Results

### Forest and bocage populations in the west of France

**Figure 1:**
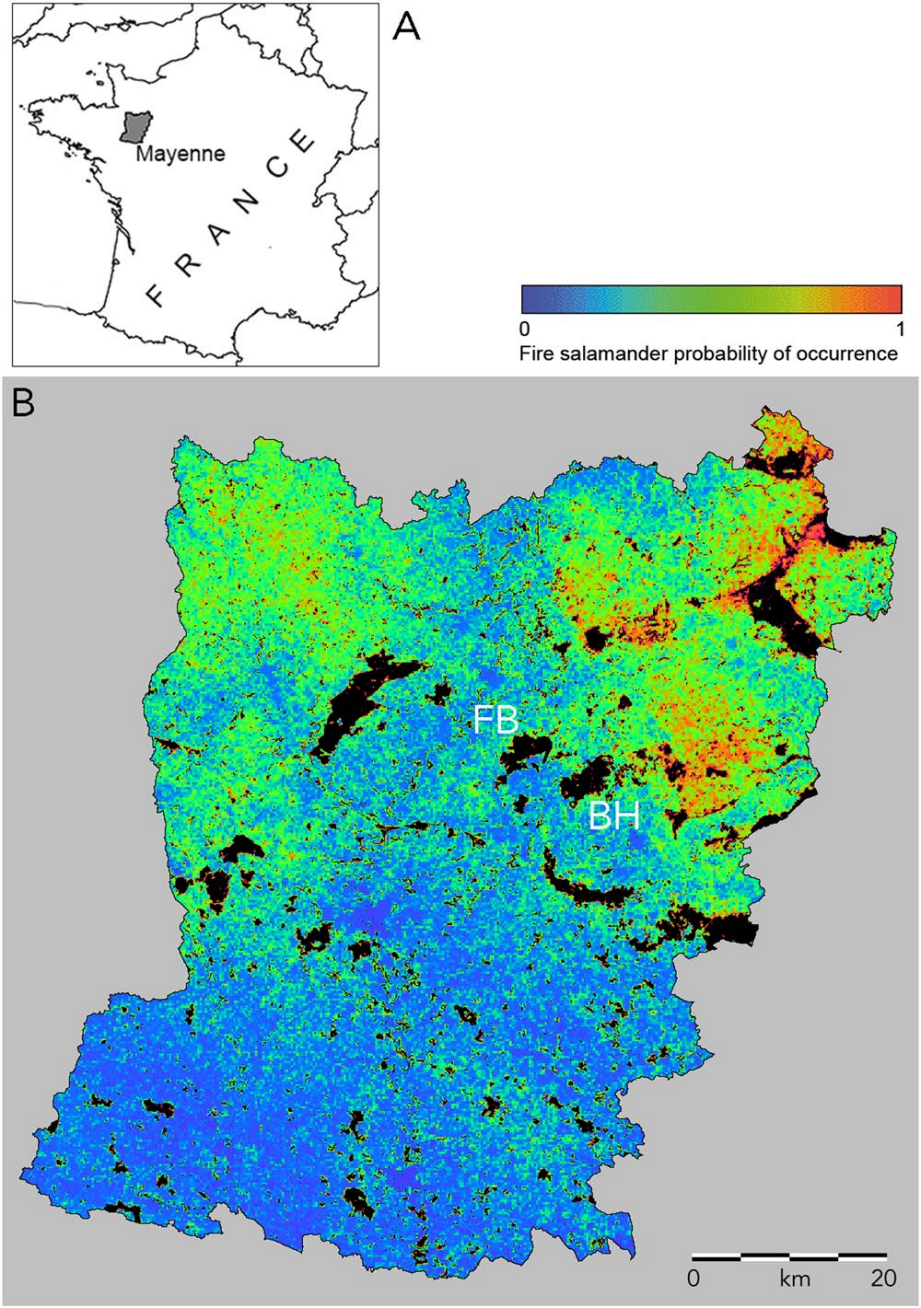
Continental France with the department Mayenne highlighted (**A**) and habitat model for the Fire salamander in department Mayenne (**B**). The map represents the habitat suitability model *P*_s_ = (1/(1 + exp(−0.0303*percent_forest_cover-0.00562*altitude-0.0299*percent_hedgerow_cover + 1.769))) and was visualized with ILWIS 3.6 software^58^, available at https://52north.org/software/software-projects/ilwis/. Habitat suitability increases from deep blue with a probability of occurrence of zero to deep red with a probability of occurrence at unity (see colour bar). Prime fire salamander habitats are found at higher altitudes and are forested (in black) or with a dense hedgerow cover. Populations genetically investigated are located in and around the largely deciduous forests Forêt de Bourgon (FB) and Bois de Hermet (BH) and listed in Table 1.The outer geographical coordinates of the department are 1.239–0.049W and 47.733–48.568N.

Larvae of *S. salamandra* were present in 251 localities (39%) and absent in 389 localities (61%). Localities are listed in a .kml file for use with e.g. Google Earth (Supplementary Information I). In logistic regression analysis the presence of the fire salamander is positively associated with forestation (P < 0.001), altitude (P < 0.001) and hedgerows (P < 0.05). At any amphibian breeding site, the probability for the occurrence of the fire salamander (*P*_s_) is estimated by the equation (1/(1 + exp(−0.0303*percent_forest_cover-0.00562*altitude-0.0299*percent_hedgerow_cover + 1.769))). The fit of the model is expressed by the ‘area under the curve’ statistic AUC = 0.721 ± 0.020, indicating that we achieved a good description of favourable versus unfavourable fire salamander terrestrial habitats. When habitat parameter values are standardized the formula is (1/(1 + exp(a*forest_cover + b*altitude + c*hedgerow_cover + 0.0210))), indicating the relative contributions to the model in the order forest (a = −0.910), altitude (b = −0.288) and hedgerows (c = −0.275). Extrapolating the model over the entire department suggests that habitats for the fire salamander outside forests are widespread in the hilly bocage landscape in the northeastern and northwestern corners of the department Mayenne. Low habitat suitability is found in the flat and deforested southern part of Mayenne, in particular the southwest (Fig. 1). The area studied for population genetic characteristics shows low habitat suitability in between and south of the forests Forêt de Bourgon and Bois de Hermet, intermediate habitat suitability surrounding the forests in the other cardinal directions and high habitat suitability further east of Bois de Hermet.

Localities studied for population genetic differentiation included 25 ponds and 16 streams and were 23 times from inside and 18 times from outside the forest. For the subdivision pond inside – outside and streams inside – outside the forests see Table 1. A high frequency of inferred null alleles (11.7%) and a substantial amount of missing data (5.0%) were observed for the microsatellite locus C2. After exclusion of this marker and the subsequent removal of seven individuals that had data missing for more than one locus (N = 734 remaining) the frequency of null alleles was estimated at 1.6% and 0.11% of data was missing. The genotypic data are presented in Supplementary Information II. The analyses with Colony software suggested that among individuals sampled from the same locality many were full siblings. With just one representative per family group population sample sizes went down by 357 under monogamy (48.6%) and by 85 under polygamy (11.6%). This approach also revealed a high potential for false positives since inferred siblings were frequent among larvae from different localities (N = 315 under monogamy, 42.9% and N = 108 under polygamy, 14.7%). Analytical results for the three data sets are summarized in Table 2.

**Table 1:** Populations of the fire salamander in Mayenne, France with samples subjected to genetic analyses, with locality number, geographical coordinates, sample size with and without all but one of the inferred siblings excluded, classification of the habitat in which the larval salamanders were found (pond versus stream and forest versus bocage) and estimates of the effective population size Ň_e_. The microsatellite genetic profiles are summarized as the loadings on the first and second principal component axis.

Locality	Eastern longitude	Northern latitude	Sample size		Habitat classification		Effective population size (95% confidence interval)		Principal components	
			Total	Siblings excluded #	Aquatic	Terrestrial			Axis 1	Axis 2
1	−0.691	48.276	8	4/6	Stream	Forest	9	(4–28)	−0.003	0.151
2	−0.599	48.197	15	7/13	Pond	Bocage	16	(8–38)	1.137	0.454
3	−0.592	48.206	22	12/21	Stream	Forest	39	(22–82)	0.123	−0.895
4	−0.588	48.219	16	9/15	Stream	Bocage	34	(16–118)	−0.510	0.016
5	−0.583	48.211	23	12/21	Pond	Forest	34	(18–70)	−0.360	0.174
6	−0.581	48.209	18	11/17	Pond	Bocage	38	(21–107)	−0.839	−0.248
7	−0.579	48.235	23	14/21	Pond	Forest	56	(30–135)	0.532	−0.592
8	−0.577	48.224	17	10/15	Stream	Forest	39	(19–115)	−0.011	−0.897
9	−0.571	48.230	9	5/9	Pond	Forest	18	(8–92)	0.422	−0.340
10	−0.570	48.242	14	8/12	Stream	Forest	20	(10–55)	−0.012	0.039
11	−0.568	48.186	7	4/7	Pond	Bocage	11	(5–73)	−0.177	−1.031
12	−0.567	48.233	15	10/15	Stream	Forest	26	(13–65)	−0.490	0.202
13	−0.564	48.220	26	16/23	Pond	Forest	54	(32–109)	0.008	−0.096
14	−0.561	48.236	25	12/24	Stream	Forest	32	(18–59)	−0.062	0.270
15	−0.561	48.189	24	13/20	Pond	Forest	37	(21–74)	−0.565	0.194
16	−0.556	48.165	19	9/16	Pond	Bocage	24	(13–52)	0.287	−0.048
17	−0.555	48.249	22	12/19	Stream	Forest	33	(18–66)	0.149	0.447
18	−0.555	48.243	15	7/13	Pond	Forest	26	(13–68)	−0.136	−0.289
19	−0.555	48.243	7	3/5	Pond	Bocage	7	(3–30)	−0.220	−0.291
20	−0.535	48.233	22	14/22	Stream	Forest	46	(24–92)	−0.724	−0.165
21	−0.532	48.245	27	12/24	Stream	Forest	33	(20–60)	0.138	0.361
22	−0.530	48.266	19	9/17	Pond	Forest	26	(14–53)	0.536	−0.081
23	−0.528	48.328	19	8/18	Stream	Bocage	34	(18–85)	0.831	−0.337
24	−0.526	48.234	24	12/20	Pond	Bocage	32	(18–61)	−1.251	0.326
25	−0.525	48.235	20	10/17	Stream	Bocage	29	(16–57)	−0.098	0.395
26	−0.521	48.330	18	9/18	Pond	Forest	31	(16–73)	0.268	−0.269
27	−0.509	48.212	17	3/8	Pond	Bocage	7	(4–21)	1.221	2.479
28	−0.505	48.229	13	6/11	Pond	Bocage	12	(6–32)	2.156	−0.385
29	−0.500	48.198	21	11/18	Pond	Forest	32	(18–65)	0.628	−0.001
30	−0.495	48.215	22	11/20	Pond	Bocage	29	(16–57)	0.001	1.258
31	−0.488	48.220	21	11/17	Pond	Bocage	32	(18–66)	0.323	0.560
32	−0.483	48.205	17	8/16	Pond	Forest	21	(11–47)	−0.722	−0.846
33	−0.478	48.275	11	6/9	Stream	Bocage	22	(10–75)	0.558	−1.162
34	−0.475	48.270	15	8/15	Pond	Bocage	23	(12–50)	0.110	−0.319
35	−0.474	48.269	25	10/20	Stream	Bocage	29	(16–54)	−0.324	−1.723
36	−0.462	48.226	22	12/20	Pond	Forest	39	(22–80)	−0.815	0.463
37	−0.461	48.221	14	8/13	Pond	Forest	26	(13–68)	−0.413	−0.007
38	−0.452	48.221	17	10/17	Stream	Forest	34	(18–90)	−0.149	−0.173
39	−0.430	48.284	20	10/19	Pond	Forest	25	(14–52)	0.594	0.572
40	−0.428	48.264	5	2/4	Stream	Bocage	5	(2–32)	−1.908	−0.266
41	−0.405	48.237	20	9/14	Pond	Bocage	19	(10–38)	−0.222	0.409

^#^All but one of the full siblings per inferred family group taken out under a monogamous/polygamous mating system.

**Table 2:** Summary of results for full and sibling reduced data sets in the study of population genetic variation in the fire salamander in Mayenne, France and the Kottenforst, Germany. Note that the genetic differentiation of pond- versus stream-breeding populations is subordinate to a forest/non-forest differentiation in France and to a longitudinal differentiation in Germany.

Study area	Mayenne, France			Kottenforst, Germany		
Data set	Full	Reduced-M	Reduced-P	Full	Reduced-M	Reduced-P
Sample size (a)	734	377	649	2563	935	1369
Global *F*st	0.024	0.010	0.018	0.036	0.019	0.026
Number of cases significantly different from random expectations						
Hardy-Weinberg equilibrium	1	0	2	72	0	0
Linkage disequilibrium	4	0	1	941	4	5
Genetic bottleneck effect	0	0	0	7	0	0
Isolation by distance	ρ = −0.033 NS	ρ = 0.014 NS	ρ = −0.016 NS	ρ = 0.070 NS	ρ = 0.191***	ρ = 0.119*
Genetic subdivision in UPGMA-tree						
Two groups defined at *F*st	0.01	0.01	0.01	<0.04	<0.015	<0.03
Forest versus non-forest	*G* = 11.34***	*G* = 6.70**	*G* = 8.64**	Not applicable		
Pond versus stream	*G* = 1.35 NS	*G* = 2.23 NS	*G* = 3.03 NS	*G* = 4.60 *	*F*, NS	*G* = 5.82 *
West versus East	Not relevant			*G* = 23.54***	*F*,**	*G* = 13.38***
Genetic differentiation in PC-plot						
Forest versus non-forest	*t* = 0.46 NS	*t* = 1.88 NS	*t* = 1.38 NS	Not applicable		
Idem, spread over PC1 and PC2 b)	*t* = 3.18**	*t* = 2.26*	*t* = 2.13*	Not applicable		
Pond versus stream	*t* = 0.58 NS	*t* = 0.29 NS	*t* = 1.31 NS	*t* = 2.81**	*t* = 3.30**	*t* = 3.45**
West versus East	Not relevant			*t* = 8.58***	*t* = 6.65***	*t* = 7.97***
Structure Bayesian assignment						
*K* selected	2	3	3	2	2	2
*Q*min - *Q*max, individuals	0.490–0.507	0.235–0.484	0.320–0.347	0–1 c)	0.02–0.98	0.01–0.99
*Q*min - *Q*max, populations	0.496–0.503	0.296–0.369	0.330–0.336	0.02–0.98 d)	0.05–0.95	0.04–0.96

(a) All but one of the full siblings taken out as inferred under a monogamous (reduced-M) and polygamous mating system (reduced-P).

(b) See Fig. 2B.

(c) As in Hendrix *et al*.^25^.

(d) See Fig. 4B.

Statistical tests referred to are matrix correlations (ρ - rho), G-test of independence (*G*), Fisher’s exact test (*F*) and Student’s *t*-test (*t*); NS - not significant, *P < 0.05, **P < 0.01 and ***P < 0.001. PC - principle component analysis.

The number of alleles observed per locus ranged from four in locus SalE5 to 15 in locus Sal3. A single locus significantly deviated from Hardy-Weinberg expectations and there were four instances of pairs of loci showing linkage disequilibrium. The overall *F*_st_ was 0.0244. The statistical power to be able to detect the mixture of genetically differentiated populations with genetic clustering methods is dependent on sample size, the number of loci studied (14), the number of alleles per locus (average 9.4) and the proportioning of the samples over habitats, in our case sample size in streams and ponds (285/449 = 0.63) or forest versus outside forest (294/440 = 0.67). Under equation 1 of Jorde *et al*. (2018: 4021; see Materials and methods) the threshold for a power of 10% is *F*_st_~0.004, which is well below the observed *F*_st_-value of 0.024.

**Figure 2:**
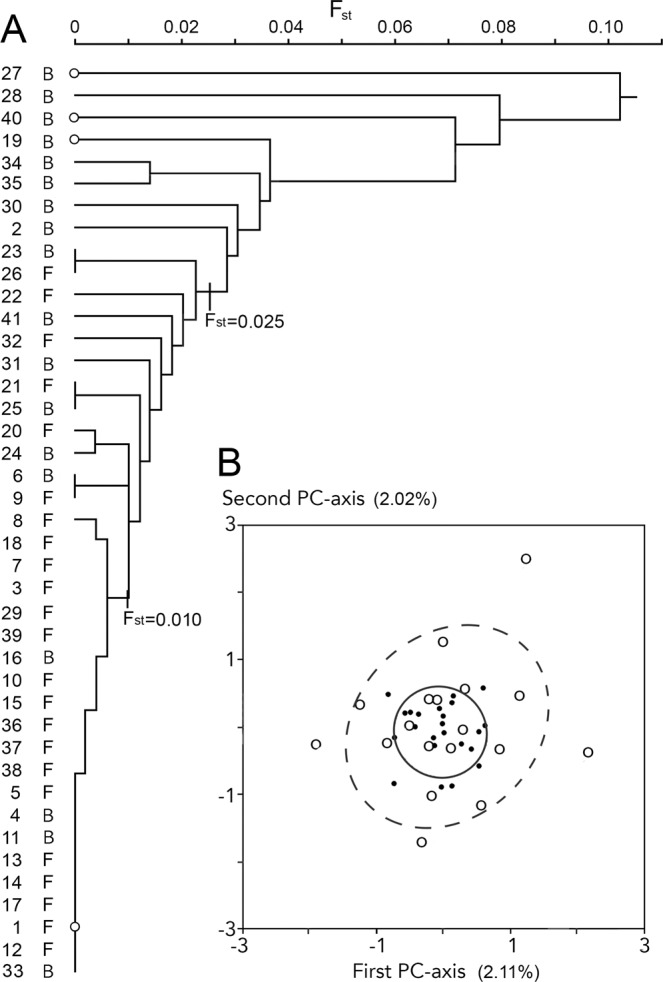
(**A**) Clustering of pairwise *F*_st_-values of fire salamander populations (Mayenne localities 1–41) with the UPGMA-method. The basal cluster at *F*_st_ < 0.010 is mostly composed of forest populations (F, 17/21 = 81%) whereas populations that join the dendrogram at higher *F*_st_-values are mostly from the bocage (B, 14/20 = 70%). At *F*_st_ > 0.025 the contribution of the bocage populations is eight out of eight. Note that populations that join the dendrogram at the highest *F*_st_-values are characterized by mostly small effective population sizes (Ň_e_ ≤ 10, indicated by small open dots). (**B**) Populations plotted along the first and second axis of a principal component analysis. The 23 forest populations are shown by small solid round symbols and the solid ellipse represents the mean ± standard deviation. Eighteen populations from the bocage are shown by large open round symbols, with the mean ± standard deviation shown by the wider ellipse with the interrupted line.

No association was observed between the pairwise distance matrices for genetic differentiation (*F*_st_/(1-*F*_st_)) and the logarithm of geographical distance (non-parametric matrix correlation, *ρ* = −0.033, *P* > 0.05), indicating the absence of isolation by distance over the study area. The observed amount of genetic differentiation is substantial but does not have a bimodal distribution as is illustrated by the UPGMA-dendrogram on *F*_st_ (Fig. 2A). A tight cluster at *F*_st_ < 0.010 is mostly composed of forest populations. Populations that join the dendrogram at higher *F*_st_-values are mostly from outside the forests, which reflects a non-random distribution (*G*-test for independence, *G* = 11.34, df = 1, P < 0.001). At *F*_st_ > 0.025 just non-forest populations are added. No significant genetic differentiation was found for pond- versus stream inhabiting population (*G* = 1.35, P > 0.05). Analyses with principal components revealed a wide overlap for the forest and non-forest groups. However, the forest populations form a tight group whereas the non-forest populations are more widely scattered over the bivariate plot (Fig. 2B). Accordingly, average distance to their nadir in the PC-plots is smaller for forest than for non-forest populations (Student’s *t*-test, *t* = 3.18, df = 21.1, P < 0.01). No significant difference was found for the pond- versus stream-breeding classification (*t* = 0.58, df = 39, P > 0.05). The analysis with Structure yielded flat distributions with undifferentiated Q-values (Table 2). It is now realized that the methods employed frequently identify K = 2 as the top level of hierarchical structure, even when more subpopulations are actually present^19^.

Estimates for effective population size N_e_ (Ň_e_) were significantly higher for forest than for non-forest populations (Ň_e/forested_ = 32.0, Ň_e/non-forested_ = 22.4; Mann-Whitney *U*-test, *U* = 301, P < 0.05) whereas they were not significantly different for pond and stream populations (Ň_e/pond_ = 27.0, Ň_e/stream_ = 29.0; *U* = 240.5, P > 0.05). Among just pond populations, those from the forest were larger than those from outside the forest (Ň_e/forested_ = 32.7, Ň_e/non-forested_ = 20.8; Kruskal-Wallis test statistic 5.25, P < 0.05) (Table 1; see also Supplementary Information III). No significant indications for population genetic bottleneck effects were observed.

Mitochondrial DNA sequences were 755–756 bp. The shorter sequence (Genbank accession number MK395359, N = 40) conforms to ‘type 1b’ and the longer one (Genbank accession number MK395358, N = 89) to ‘type 1a’^11^. Sequences are equivalent to Genbank accession number KU249989^20^ with an indel at position 599. The distribution of the two mtDNA types bears no correspondence to the forest/non-forest inhabiting or to the stream- versus pond-breeding habitat classification (see Supplementary Information IV). For a compilation of mtDNA data from across northern Europe see Supplementary Information V.

### Forest populations in the Kottenforst, Germany

Microsatellite genetic data for the Kottenforst were taken from Hendrix *et al*.^21^. All the investigated populations are situated inside the forest. Fire salamander larvae were observed in a variety of aquatic habitats (Table 3), with streams less frequent in the western section of the forest (one stream locality out of 27, 4%) than in the eastern section of the forest (six stream localities out of 20, 30%).

**Table 3:** Populations of the fire salamander in the Kottenforst, Germany with samples subjected to genetic analyses, with locality number, geographical coordinates at the Universal Transverse Mercator grid system, sample size with and without all but one of the inferred siblings excluded, classification of the aquatic habitat in which the larval salamanders were found (pond, stream, etc.), and estimates of the effective population size Ňe. The microsatellite genetic profiles are summarized as the loadings on the first and second principal component axis.

Locality	Coordinates		Aquatic habitat type	Sample size		Effective population size (95% confidence interval)		Principal components	
	UTM_x	UTM_y		Total	Siblings excluded #			Axis 1	Axis 2
K01	361.713	5616.952	Ditch	33	19/30	62	(39–113)	−0.651	−0.016
K02	361.806	5615.876	Ditch	142	40/61	67	(49–96)	−1.494	−0.024
K03	361.775	5615.610	Ditch	122	33/49	43	(29–68)	−1.396	−0.307
K04	361.819	5615.443	Ditch	117	50/73	104	(79–138)	−1.419	−0.287
K05	361.796	5615.444	Ditch	62	12/18	21	(12–39)	−1.282	−0.474
K06	362.255	5615.445	Pond	12	5/8	10	(5–26)	−0.454	0.230
K07	362.122	5615.034	Ditch	22	8/12	18	(10–38)	−0.957	0.059
K08	361.095	5613.985	Pond	157	42/54	52	(36–77)	−1.747	−0.225
K09	361.067	5614.004	Pond	93	29/39	39	(26–62)	−1.577	−0.145
K10	361.855	5613.555	Pond	51	20/37	44	(29–71)	−1.520	−0.500
K11	362.024	5613.471	Pond	85	30/40	48	(32–76)	−0.798	−0.320
K12	362.561	5614.092	Pond	3	2/3	4	(2–20)	−1.831	−0.719
K13	361.753	5612.671	Pond	5	3/5	10	(4–7158)	−1.080	−0.594
K14	361.457	5612.442	Ditch	65	34/48	95	(66–137)	−1.055	−0.107
K15	361.499	5613.341	Pond	26	11/20	31	(18–58)	−1.319	−0.034
K16	363.390	5614.655	Ditch	46	12/22	13	(7–30)	−1.445	−0.112
K17	363.492	5614.288	Pond	4	2/3	4	(2-undet.)	−2.035	−0.115
K18	363.267	5613.985	Ditch	4	3/3	12	(2-undet.)	−2.085	−0.813
K19	362.341	5612.463	Pond	3	2/3	4	(2–20)	−0.188	0.166
K20	363.290	5613.128	Pond	8	3/5	6	(2–23)	−1.263	−0.464
K21	364.219	5613.332	Pond	21	3/5	5	(2–20)	−1.098	−0.559
K22	364.107	5613.656	Ditch	4	4/4	undet.	0.562	−0.580	
K23	364.580	5614.403	Intermittent stream	151	43/61	46	(32–71)	−0.780	−0.220
K24 $	364.583	5614.562	Pond	34	16/18	31	(18–55)	−0.768	−0.255
K25	364.865	5613.702	Pond	4	3/4	12	(4-undet.)	−1.045	0.663
K26	364.687	5614.347	Pond	8	5/7	19	(8–150)	−0.913	−0.425
K27	364.873	5614.266	Tire rut	4	3/3	12	(4-undet.)	−0.430	0.745
K28	365.482	5613.251	Puddle	10	1/7	2	(2-undet.)	1.157	−0.373
K29	365.762	5613.586	Ditch	4	2/4	4	(2-undet.)	−1.323	0.150
K30	366.814	5613.873	Pond	7	2/5	5	(2–20)	0.219	0.318
K31	366.177	5615.348	Stream	20	1/7	2	(2-undet.)	2.509	−1.351
K32	366.592	5614.994	Pond	4	2/4	6	(2-undet.)	0.536	0.726
K33	366.793	5615.225	Pond	32	4/5	7	(4–21)	0.708	−0.289
K34 $	366.666	5613.432	Ditch	6	4/6	15	(6-undet.)	0.017	−0.304
K35	367.798	5614.881	Stream	258	123/181	272	(224–329)	−0.031	0.750
K36	368.179	5615.553	Stream	54	31/49	99	(70–151)	0.595	0.607
K37	368.216	5615.677	Pond	40	17/26	40	(26–65)	0.656	1.035
K38	367.542	5615.687	Tire rut	4	2/4	6	(2-undet.)	0.091	−1.177
K39	367.067	5615.553	Pond	109	20/24	14	(8–30)	2.240	−1.026
K40	367.033	5615.805	Tire rut	184	29/35	24	(15–43)	2.292	0.014
K41	367.200	5616.164	Pond	79	38/55	96	(71–133)	1.466	0.074
K42	366.964	5615.909	Ditch	4	2/4	4	(2-undet.)	2.217	−0.558
K43	367.873	5616.909	Stream	248	117/161	260	(215–316)	1.658	0.118
K44	367.146	5616.881	Pond	37	18/31	36	(22–61)	1.348	0.941
K45	366.960	5616.458	Stream	127	58/86	142	(108–186)	1.425	0.519
K46	366.416	5616.939	Stream	46	24/36	65	(44–100)	1.299	0.653
K47	366.566	5617.580	Puddle	4	3/4	12	(4-undet.)	0.427	−0.122

Undet. - not determined.

^#^All but one of the full siblings per inferred family group taken out, as estimated under the assumption of a monogamous/polygamous breeding system.

^$^Coordinates taken from Hendrix *et al*. (2017*b*: Supplementary Fig. 1)^25^.

Analyses with Colony software suggested that many of the sampled individuals were full siblings. With only one representative per family group population sample sizes went down by 1628 under monogamy (63.5%) and by 1194 under polygamy (46.6%). Colony also revealed a high potential for false positives since inferred siblings were frequent among larvae from different localities (N = 624 under monogamy, 24.3% and N = 210 under polygamy, 8.2%). Results for genetic (dis)equilibria, genetic bottleneck effects and isolation by distance were markedly different for full and the sibling-excluded data sets. In the former we found 72 cases of significant deviation from Hardy-Weinberg expectations and 941 pairwise locus combinations with significant linkage disequilibrium (Table 2). Numbers were by two orders of magnitude lower in the reduced data sets, suggesting that the significant signals for genetic equilibria are largely to be attributed to the sampling of family groups. In the sibling reduced data set we identified no significant signal for population genetic bottlenecks whereas there were seven in the full data set. Finally, the signal for isolation by distance was not significant in the full data set (non-parametric matrix correlation, *ρ* = 0.070, P > 0.05) and significant in the reduced data sets (*ρ* = 0.191, P < 0.001 under monogamy and *ρ* = 0.119, P < 0.05 under polygamy).

Estimates for effective population size varied widely and were not significantly different for forest sections (Ň_e/west_ = 31.2, range 4–104; Ň_e/east_ = 55.6, range 2–272; Mann-Whitney *U*-test, *U* = 249, P > 0.05). Average effective population sizes for the Kottenforst were not significantly different from those in Mayenne (Mann-Whitney *U*-test, *U* = 1037, P > 0.05), but showed a wider range in the Kottenforst than in Mayenne (5 < Ň_e_ < 56, see also Supplementary Information III).

**Figure 3:**
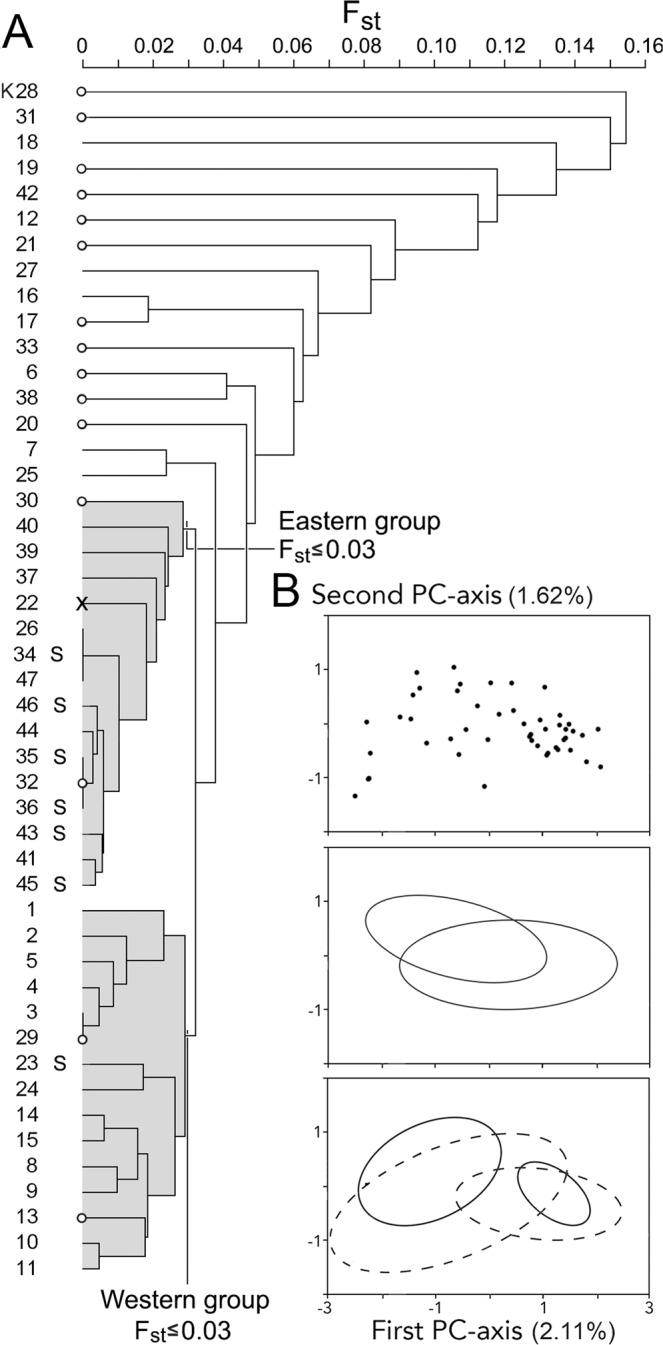
(**A**) Clustering of pairwise *F*_st_-values of Kottenforst fire salamander populations (localities K01-K47) with the UPGMA-method. Numbers K01-K27 represent populations in the western section of the forest and K28-K47 represent populations in the eastern section of the forest. The basal cluster at *F*_st_ < 0.04 is composed of two groups (shaded) composed of mostly eastern (14/16 = 88%) or mostly western localities (14/15 = 93%). Populations breeding in streams are shown by the letter S. Note that populations that join the dendrogram at higher *F*_st_-values are characterized by mostly small effective population sizes (Ň_e_ ≤ 10, indicated by small open dots; X – Ň_e_ not determined). **B** top panel - Populations plotted along the first and second axis of a principal component analysis. Middle panel - Ellipses represent means ± standard deviation for seven stream populations (left ellipse) and 40 non-stream populations (right ellipse). Lower panel - Ellipses represent means ± standard deviation for the western (left) and eastern (right) section of the Kottenforst, for small populations (Ň_e_ ≤ 10) shown by interrupted lines and for larger populations (Ň_e_ > 10) shown by uninterrupted lines. Note that for the larger populations the ellipses for western and eastern localities do not overlap.

**Figure 4:**
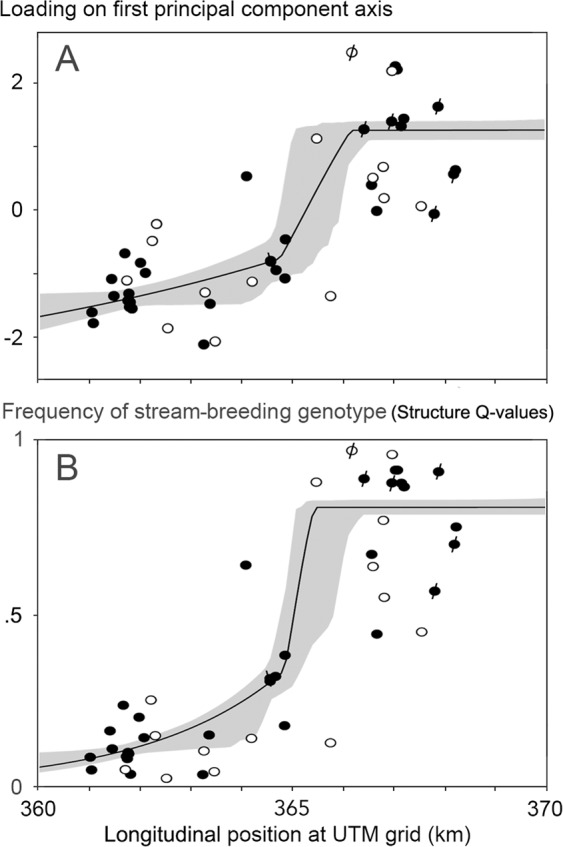
Microsatellite population genetic data for the fire salamander in the Kottenforst, Germany^21,24^ analyzed in the framework of allopatric speciation, i.e. a secondary spatial contact of a western pond-breeding lineage and an eastern stream-breeding lineage. The 95% credible cline regions are shown by grey shading. Solid and open round symbols represent larger (Ň_e_ > 10) and small populations (Ň_e_ ≤ 10), respectively. Note that the stream-breeding populations that gave the composite genotype its name are all located in the eastern section of the Kottenforst (six data points indicated with a forward slash (/). One ‘intermittent stream’ in the western section is indicated by a backward slash. Also note the paucity of data at and around the steepest part of the clines. **A** – loadings on the first PC axis versus geographical distance. The cline centre is at km 365.3 of the Universal Transverse Mercator (UTM) grid. Cline width is 3952 m. **B** – frequency of the stream-breeding genotype versus distance (after^21^). The cline centre is at UTM km 365.1 and the cline width is 1108 m. For model details see Supplementary Information VI.

The overall *F*_st_ for the studied fire salamander populations was 0.0360, which value widely surpassed the threshold for 10% analytical power of 0.0016. The UPGMA-dendrogram of pairwise *F*_st_-values reveals two clusters that are differentiated at *F*_st_ = 0.04 (Fig. 3A). One cluster is made up of one eastern plus 14 western populations and the other cluster is made up of two western and 14 eastern populations, which reflects a non-random distribution (*G*-test for independence, *G* = 23.54, df = 1, P < 0.001). This signal is stronger than the parallel separation in pond- and stream-breeding populations (*G* = 4.60, P < 0.05). Populations joining the dendrogram at higher *F*_st_ levels are from either forest section. This set of populations is characterized by particularly low Ň_e_ (average Ň_e_ = 7.6 versus Ň_e_ = 60.1 for the remainder; Mann-Whitney *U*-test, *U* = 439.5, P < 0.001), suggesting that a founder effect or genetic drift underlies the genetic differentiation of small populations. Analyses with principal components supports the population allocation of two spatial groups, in which the western and eastern group show no overlap along the first PC-axis if indeed small populations are excluded (Fig. 3B). The support for separation of a western versus an eastern group is several orders of magnitude stronger than that for the (more or less parallel) separation of pond- and stream-breeding salamander populations (Student’s *t*-test, *t* = 8.58, df = 29.0, P < 0.000001 versus *t* = 2.81, df = 45, P = 0.007401). In line with these results, the plot of the PC1-scores over a west to east axis describes a genetic transition in a sigmoid curve of the type reminiscent to those analyzed in the classical hybrid zone literature e.g.^22^. The cline that best fitted the data has a central position at km 365.3 of the longitudinal axis of the Universal Transverse Mercator coordinate system grid (UTM) and a width of 3.95 km (Fig. 4A). Because the PC-analysis extracted only a small proportion of the total variance in the data (2.1% along the first axis) we repeated the cline fitting procedure with the ‘proportion of pond- versus stream-breeding genotypes’ or ‘assignment probabilities’ of Hendrix *et al*.^21^ under an inferred optimal number of two genetic clusters (K = 2), derived with Structure software^23,24^. The cline description that best accommodates these values has a central position at km 365.1 and a width of 1.11 km (Fig. 4B). The full model descriptions are presented in Supplementary Information VI.

## Discussion

Adult fire salamanders are terrestrial amphibians that deposit larvae in ponds or streams, in where these develop until metamorphosis. The species figures as an example of local ecological and genetic differentiation and adaptation, possibly representing the first step in the speciation process^12,25^. We set out to replicate the observation of genetic differentiation of stream- and pond-breeding populations and we sought the opportunity to explore for additional drivers of genetic differentiation at a landscape scale. However, the microsatellite data we obtained for the fire salamander in Mayenne did not yield a signal for genetic differentiation of pond- and stream-breeding populations. Instead we found genetic differences for populations from inside and outside forests.

### Fire salamander population structure in Mayenne, France

The results of our habitat suitability modelling are in line with the general habitat preference of the fire salamander in Central Europe, namely mixed deciduous forest at 200–400 m a.s.l.^26,27^. In addition, we document the regular presence of fire salamanders outside forests, in particular in hilly areas where the network of hedges is dense. Such a pastural landscape is known as ‘bocage’. Our data suggest that forests are the primary fire salamander habitat. Firstly, forestation represents the most prominent contribution to the habitat model. Secondly, the estimates for effective population sizes are larger for forest than for bocage populations. Thirdly, forest populations are genetically most similar suggesting ongoing gene flow, presumably covering historical times. In contrast, the bocage populations are frequently genetically differentiated from one another (*F*_st_ > 0.025) indicating that they are more or less isolated and that genetic drift and founder effect operate more effectively in these small populations. In metapopulation terminology, the fire salamander system in Mayenne fits the ‘mainland-island’ model^28^ in which forests are the mainland and the bocage represents an archipelago of islands. In Spain, genetic differentiation was observed to increase from *F*_st_ < 0.05 among most continental *S. salamandra* populations to *F*_st_~0.10 for peninsular populations separated for at least 2000 years, to *F*_st_~0.19 for insular populations separated for 6–13 Kybp (thousands of years before present) (geological data^29,30^, genetic data^31,32^). This system, literally fitting the mainland-island model and at a comparable spatial scale to Mayenne, confirms the propensity for genetic drift in small and isolated fire salamander populations.

The two forests in Mayenne may be interconnected by dispersal through the dense but patchy and declining bocage. While population connectivity operating through the network of hedgerows is likely, the study area does not offer a good setting for testing this hypothesis. First, we noted no consistent spatial genetic signal and it will be difficult to assess if this can be attributed to the counteracting forces of isolation by distance and connectivity by habitat. Second, to be able to disentangle these effects requires an explicit spatial configuration. For example, to test for fire salamander dispersal along hedgerows, the hypothesized corridor is to deviate from a straight line. If not, genetic differentiation will, by default, be associated to geographical, not ecological distance.

We found no strong evidence for population genetic bottlenecking, suggesting that fire salamander populations may be stable over time. Another line of evidence for the long-term persistence of fire salamander populations are several occurrences in the northwest of Mayenne (Supplementary Information I)^33^. This area is currently devoid of forests (Fig. 2B) and also 18^th^ century maps by the Cassini family (accessible at https://www.geoportail.gouv.fr/donnees/carte-de-cassini) show no forest at the localities where fire salamanders were actually observed, suggesting that extant populations persisted in the bocage over the centuries and providing ample opportunity for genetic drift to operate. While we could not detect significant departures from panmixia in forests, the bocage populations are more or less isolated. Yet the bocage localities may serve as ‘islands’ or ‘stepping stones’. Even a loose network may help to preserve population connectivity at a wider spatial scale and eventually promote the genetic exchange between forest populations, such as those of Forêt de Bourgon and Bois de Hermet (Fig. 2B). However, the pastural landscape in western France rapidly deteriorates from the perspective of wildlife, mostly through field size enlargements and agricultural reform, with the concomitant loss of small landscape elements such as hedgerows, spinneys and ponds^18,33–36^. A further deterioration of the bocage is likely to isolate the forest populations from one another. To illustrate this point, in the flat, southern part of Mayenne the bocage has largely disappeared since the Second World War^37^ and the fire salamander is locally rare with some scattered occurrences^33^ (Supplementary Information I).

### Fire salamander population structure in the Kottenforst

The German Kottenforst supports two fire salamander population groups that are differentiated at *F*_st_ = 0.030 and that are proposed to represent pond- versus stream-breeders. However, the ecological data in support of this interpretation are meagre, simply because stream-breeding populations are a minority in the Kottenforst system. Moreover, focusing on large and presumably healthy populations that are locally more frequently found in streams than in ponds (Table 3) strengthens the eco-geographical distinction more so than it sharpens the genetic differentiation.

The west to east distinction is to some degree obscured by a suite of 16 populations that are more deeply genetically differentiated (0.030 < *F*_st_ < 0.153; Fig. 3). These latter populations are characterized by significantly smaller effective population sizes than average and 11 of them have Ň_e_ ≤ 10. The small population sizes suggest that genetic drift might be prominent, but given the more or less unhampered dispersal of adult fire salamanders across the forest, populations are unlikely to be isolated and a genetic founder effect is a more plausible explanation.

With an average minimum distance of neighbouring populations of ca. 400 m the grid of localities studied in the Kottenforst is tighter than in Mayenne. Yet, unlike Mayenne, the pond- and stream-breeding populations appear to be spatially, behaviourally and genetically separated. This separation takes effect along a roughly longitudinal axis. The by approximation sigmoid shaped transition is 1.11–3.95 km wide, with inflection points that are 730–1440 m apart (Fig. 4). A cline this narrow cannot result from neutral processes exclusively. In the absence of selection, the width (*w*) of the cline can be predicted from a diffusion model as a function of dispersal distance (*d*) and the length of time since contact (*t*), as *w* = 2.51*d* √(*t*)^38^. Generation time is reported as six years for both sexes^39^ and also as three years for males and four years for females^40^. At an average dispersal of 200 m per generation, which is at the lower limit because it just covers the regular home range, cline widths would exceed the measured width in a couple of generations and at higher dispersal, such as distances of up to 2000 m^24^, the collapse of the cline would be near-instantaneous. However, the Kottenforst genetic transition is sharp, suggesting that intrinsic selection operates against the two lineages’ mixed offspring. The center of the transition is further characterized by a paucity of material studied, presumably caused by lack of aquatic habitat suitable for the deposition of larvae. This observation fits classical theory where a hybrid zone may be ‘captured’ where an area of low population density acts as a barrier to further movement^41,42^. If dispersal is high, selection against hybrids is to be strong. Evidence for genetic incompatibility of the lineages may have been overlooked by Caspers *et al*.^13^. These authors were surprised to find that offspring numbers in the Kottenforst *increased* with the degree of genetic relatedness between females and their sires. Puzzling as this observation might be for within-lineage data, in the context of selection against hybrids it makes direct sense.

The postglacial range expansion of *S. salamandra* has been linked to the spread of the beech, *Fagus sylvatica* L^11,12,43^. The contact zone, cq. intra-specific hybrid zone in the Kottenforst may have originated ca. 8 Kybp, by colonization from southern European glacial refuge areas, possibly involving the south of France and the northern Balkans, but more northerly locations cannot be excluded^44,45^. Regardless of the location of the glacial refugia, the hybrid zone in the Kottenforst appears to have been kept in check over hundreds or thousands of years, in which selection against hybrids counteracted dispersal into the zone. As an alternative explanation to *in situ* ecological speciation, we suggest that the Kottenforst is an area of secondary contact of a pond-breeding western lineage and a stream-breeding eastern lineage. Pond-breeding in the Kottenforst is considered a recent adaptation^25,46^, but we consider this unlikely because the condition is widespread in western Europe^26,28,35,47,48^.

An argument against the secondary contact scenario may be that the Kottenforst fire salamander populations are more similar to one another than to other, nearby populations for mitochondrial^11^ and nuclear genetic markers^49^. The fire salamander in northern Europe is characterized by two mitochondrial DNA lineages with ‘type 1’ in the west and ‘type 2’ in the east. According to Weitere *et al*.^11^ the pond- and stream-breeding populations in the Kottenforst are all descendants of the western mitochondrial DNA lineage. However, a sharp and complete transition from type 1 to type 2 is found not far away (at 70–80 km north, in between the localities Felderbachtal and Bochum; Supplementary Information V). We propose that the mtDNA cline is displaced relative to the cline from the nuclear genetic markers, a phenomenon regularly observed, especially for uniparentally transmitted markers^50–52^. Unfortunately, a wide sampling gap (>350 km) precludes measuring position, width and shape of the mtDNA transition east of the Kottenforst. With microsatellite data Steinfartz *et al*.^49^ also found that the Kottenforst populations are the most closely related in the wider area. This conclusion, however, depends on the position of the ‘root’ of the graph (which is not provided) and the supporting evidence is not unambiguous given the graph’s short internal branches. Future research might reveal a continuation of the fire salamander contact zone outside the Kottenforst and possibly show that the western and eastern lineages represent the currently described subspecies *S. s. terrestris* Lacépède, 1788 and *S. s. salamandra*. A wider phylogeographic survey is required to solve these issues.

### Concluding remarks

The fire salamander is a species prone to population genetic differentiation. Substantial genetic drift has been reported for small and isolated populations across its range^31,53–56^. We analyzed microsatellite genetic data in two metapopulations of the fire salamander in the northwest of Europe, with contrasting results. In France we documented small, isolated, persisting and genetically differentiated populations in the bocage versus panmixia in two small deciduous forests. This population structure fits the classical mainland-island model. A mainland-island population structure may have been paramount to the fire salamander prior to the recent intensification of agriculture, the field size enlargements that came with it and the widespread conversion from pasture for cattle-breeding to arable for growing crops. We did not find a bimodal distribution coinciding with stream- and pond-reproduction as detected in the Kottenforst^12,25,57^. Accordingly, the fire salamander system in France offers no support for habitat driven genetic differentiation other than through drift and founder effects operating in more or less isolated bocage populations. In Germany we found small and genetically differentiated populations scattered over the Kottenforst, in addition to larger populations occupying ponds in the western section and ponds and streams in the eastern section of the forest. The finding is somewhat puzzling, given the unrestricted gene flow that we documented for the French forest populations and the rampant migration documented for the Kottenforst *per se*, with large home ranges and frequent long-distance dispersal^24^. An *ad hoc* explanation, supported by the high frequencies of full-siblings and low effective population sizes, is that these localities represent a particularly intensive sampling scheme, with larvae included from unusual aquatic habitats such as puddles and wheel ruts (Table 3). These small and frequently impermanent water bodies may represent sub-optimal conditions for larval growth, survival and metamorphosis and be transient satellites to the core fire salamander populations. Finally, to explain the significant spatial component in the remaining genetic variation, we propose that secondary contact between a western and an eastern fire salamander lineage better explains the available data than an ecological sympatric speciation scenario.

## Materials and Methods

We recorded the presence or absence of fire salamander larvae in 640 amphibian pond and stream breeding sites across the department Mayenne. Ponds represent focal points of amphibian presence and harbour more or less isolated populations (or ‘demes’) that together constitute a metapopulation. The deme structure in streams is less obvious, but clearly not all sections of a stream are equally suitable for reproduction, for example in sections with and without predatory fish, or sections falling dry. Yet, for consistency, we consider the amphibian sites that we study to represent local populations. Locality data on altitude (m above sea level, a.s.l.), forestation and hedgerows (percent cover) were extracted from digital IGN maps (Institute Géographique Nationale) with a 25 m spatial resolution, provided by department officials. The area considered around each pond or stream sampling locality had a radius of 200 m. This scale should represent a typical fire salamander home range because 200 m is the median distance travelled by adults in a capture-recapture study in the Kottenforst^24^. The biological and environmental data were analyzed by logistic regression with a weight parameter, so that in analysis the number of fire salamander presences equaled the number of absences. Habitat models were visualized with ILWIS 3.6^58^. We did not observe a disease-born population decline that was reported ca. 500 km to the northwest, in The Netherlands^10^.

The area selected for population genetic research is situated around two small, largely deciduous forests and positioned at the transition from flat and deforested in the south of Mayenne, to hilly with a bocage landscape in the north (Fig. 1). Larvae were captured with dip nets in 41 ponds and streams which were located inside as well as outside the forests. The larvae were released on the spot directly after tail tip tissue sampling. Altogether 741 salamander larvae were genotyped for 15 polymorphic microsatellite loci. The loci Sal3, Sal29, SalE11, SalE5, SalE6, SalE7 and SalE8 were studied following Steinfartz *et al*.^59^ and the loci B11, C2, C3, E11, G6, G9, IA6, IIA6 were studied following Hendrix *et al*.^60^. Locus SalE5 is a dinucleotide microsatellite locus and the others are composed of tetramer motifs. After a quality check (see Results) data for the locus C2 were excluded so that 14 markers remained. A total of 129 individuals from 14 localities was sequenced for the mitochondrial D-loop (control region, 756 bp) as described in Steinfartz *et al*.^61^. Nuclear genetic data from the German Kottenforst involved 2563 larval fire salamanders studied at 17 polymorphic microsatellite loci and were retrieved from Hendrix *et al*.^21^. The 47 Kottenforst sampling localities were classified in two spatial groups (‘west’ with localities K01-K27 and ‘east’ with localities K28-K47) that represent opposite sections of a sharp genetic transition (see Results).

Computer programs employed for the genetic data were as follows. FreeNA^62^ to analyze for the presence of null-alleles. FreeNA was run with 1000 replicates using the EM algorithm. GenePop version 4.2^63^ to estimate *F*_st_-values and analyze for Hardy-Weinberg and linkage (dis)equilibria. GenePop was run with dememorization number 1000, 100 batches and 1000 iterations per batch. Linkage disequilibrium was determined using the log likelihood ratio statistic. Hardy-Weinberg equilibrium and linkage disequilibrium results were interpreted under the Benjamini-Hochberg correction for multiple comparisons. Statistical power for the detection of genetically differentiated yet sympatric populations was estimated following Jorde *et al*.^64^. The genetic diversity among populations was summarized by clustering with the unweighted pair group method with arithmetic mean with Primer-e software (UPGMA^65^) and by principal component analysis (PC) with Adegenet version 2.0.0 following the manuals^66,67^. We also carried out a Bayesian assignment analysis with Structure^68^, to which we adopted the program settings for the similar analyses by Hendrix *et al*.^21^. The parameter K (the number of genetic groups suggested by the data) was evaluated under the ‘Evanno-criterion’ that is implemented in StructureHarvester^69^. We used Colony version 2.0.6.2^70^ to analyze family groups and to estimate effective population size (Ň_e_) from the frequency of siblings. Settings for Colony were both sexes either monogamous or polygamous (see below), no allelic dropout or typing errors, diploid, codominant markers, no inbreeding, scaled full sibship, unknown population allele frequency, ten ‘medium long’ or ‘very long’ runs under full-likelihood with ‘medium’ precision and a ‘strong/optimal’ sibship prior for effective population size. Results for Ň_e_ reported are under α is zero, assuming that deviations from Hardy-Weinberg equilibrium are negligible. We used Bottleneck^71^ to test for reductions in effective population size by considering that alleles are generally lost faster than heterozygosity and thus, populations that have experienced a reduction in effective population size are expected to have excess heterozygosity relative to that expected under mutation-drift equilibrium. Samples smaller than for ten individuals were ignored. Statistical relevance of the results were determined with the Wilcoxon signed-rank test. We applied the two phase model with 93.6% single step mutations, variance 30 and 10,000 replications. The proportion of single-step mutations was determined with Misat version 1.0^72^ with dimer code 2, tetramer code 4, gridsize 40, moments estimated under the one-step model, 100,000 runs through the Markov chain, with continuously updating the value of *θ*_0_. We used HZAR^73^ to formalize the geographical cline suggested by the first PC-axis for the Kottenforst. The chain length was 100,000 with a burn-in of 10%, randomized seeds and model selection based on AICc scores. For details on the cline fitting procedure and model selection see e.g.^74^. Mitochondrial DNA sequences were aligned with ClustalW version 2^75^ under default settings. Matrix correlations were done with Primer-e^65^ under 10,000 permutations. Other statistical analyses were with SPSS v. 20^76^.

Reproduction in the fire salamander may be monogamous or polygamous. The available data indicate that both systems occur in nature about equally frequent^49^ and we explored both possibilities. Including siblings in analyses of population diversity and structure can introduce a variety of biases (^77^ and references therein). Clutch sizes in the fire salamander are ca. 30^27^ and the more or less frequent sampling of full- and half-siblings in our study cannot be excluded. However, excluding all but one members of a family group is not exempt from problems neither and will cause other biases^78,79^. Considering the moderate number of markers and alleles in either of the two data sets we tested for the presence of full-siblings in the material at two levels. Firstly, we reconstructed family groups per population. The numbers of inferred full-siblings were substantial. Secondly, we compared all individuals across localities. Although female fire salamanders may deposit larvae in more than one water body^13^ this phenomenon will be rare in our sampling of disparate localities with average minimum distances of ca. 1.1 km in Mayenne and 400 m in the Kottenforst. Consequently, inferred full-siblings from different localities possibly qualify as false positives. It is altogether not straightforward to determine the balance between redundant information from siblings included versus independent information from false positives excluded. We here present results from the full data set not to hamper comparability with published results^24^, with the parallel results for the siblings excluded data set in Table 2. The mitochondrial DNA data considered include^80^.

### Ethics statement

Methods were carried out in accordance with the relevant guidelines and regulations. The tissue sampling protocol was approved by the Research Coordination Office of Naturalis Biodiversity Center. Fieldwork was carried under out under license from the ‘Service Aménagement Environnement-Chasse, Direction départementale de l’Agriculture et de la Forêt, Mayenne arrêt no. 2003-A-207.

## Supplementary information

Suplementary information.

## Data Availability

The genotypic data for fire salamanders from Mayenne, France are presented in Supplementary Information II. The data for the Kottenforst, Germany are accessible at https://doi.org/10.5061/dryad.h0r6q.
