# Publisher Correction: ‘Mainland-island’ population structure of a terrestrial salamander in a forest-bocage landscape with little evidence for *in situ* ecological speciation

**DOI:** 10.1038/s41598-020-64343-3

**Published:** 2020-04-24

**Authors:** Jan W. Arntzen, Joep van Belkom

**Affiliations:** Naturalis Biodiversity Center, Leiden, The Netherlands

Correction to: *Scientific Reports* 10.1038/s41598-020-58551-0, published online 03 February 2020

This Article contains a typographical error in the Author Contributions section where,

“J.W.A. conceived and designed the study, and organized the species inventory. The files SI I and SI V are also available at https://www.repository.naturalis.nl/record/707616. J.v.B. collected the tissue samples and performed the laboratory work in the laboratory of S. Steinfartz, Braunschweig. J.W.A. analyzed the data and wrote the manuscript with the help of J.v.B.”

should read:

“J.W.A. conceived and designed the study, and organized the species inventory. J.v.B. collected the tissue samples and performed the laboratory work in the laboratory of S. Steinfartz, Braunschweig. J.W.A. analyzed the data and wrote the manuscript with the help of J.v.B.”

As a result, the ‘Data Availability’ section,

“The genotypic data for fire salamanders from Mayenne, France are presented in Supplementary Information II. The data for the Kottenforst, Germany are accessible at 10.5061/dryad.h0r6q.”

should read:

“The genotypic data for fire salamanders from Mayenne, France are presented in Supplementary Information II. The data for the Kottenforst, Germany are accessible at 10.5061/dryad.h0r6q. The files SI I and SI V are also available at https://www.repository.naturalis.nl/record/707616”

