# Supplementary material for: ‘Mainland-island’ population structure of a terrestrial salamander in a forest-bocage landscape with little evidence for in situ ecological speciation
Source: Sci Rep. 2020 Feb 3;10:1700. doi: 10.1038/s41598-020-58551-0 (PMC6997349; doi:10.1038/s41598-020-58551-0)
Supplement: Supplementary file 1 — Suplementary information. [file 41598_2020_58551_MOESM1_ESM.pdf]

Supplementary Information I

[change document extention from .txt to .kml for use with e.g. Google Earth]


```
<?xml version="1.0" encoding="UTF-8"?><kml xmlns="http://earth.google.com/kml/2.1">
<!-- Comments here! -->

<Document>
	<name>pies_05-18-18_07:46:18.kml</name>  <!-- generates a random name for the file based on the timestamp -->
	<description>
	<![CDATA[ Pie charts of haplotype distributions.  Generated through <a href="https://www.nescent.org/wg_phyloinformatics/PhyloSoC:phylogeoviz">Phylogeoviz</a>.  Document created 05-18-18 at 07:46:18 UTC.]]>
	</description>
	<Style id="sn_ylw-circle">  <!-- style for just the placemarks -->
		<IconStyle>
			<Icon>
				<href>http://maps.google.com/mapfiles/kml/paddle/ylw-circle.png</href>
			</Icon>
		</IconStyle>
		<LineStyle>
			<color>ffff0000</color>
		</LineStyle>
		<PolyStyle>
			<color>d9003dff</color>
		</PolyStyle>
	</Style>

	<!-- styles for pie slices, i.e. hap colors -->
	<Style id="stylehap1">
		<IconStyle>
			<Icon>
				<href>http://maps.google.com/mapfiles/kml/pal2/icon7.png</href>
			</Icon>
		</IconStyle>
		<LineStyle>
			<color>ff000000</color>
			<width>1.0</width>
		</LineStyle>
		<PolyStyle>
			<color>ffA63125</color>
			<outline>0</outline>
		</PolyStyle>
	</Style>
	<Style id="stylehap2">
		<IconStyle>
			<Icon>
				<href>http://maps.google.com/mapfiles/kml/pal2/icon7.png</href>
			</Icon>
		</IconStyle>
		<LineStyle>
			<color>ff000000</color>
			<width>1.0</width>
		</LineStyle>
		<PolyStyle>
```

```
			<color>ffA604E9</color>
			<outline>0</outline>
		</PolyStyle>
	</Style>

	<!-- Generate placemarks at all the pop centers -->
	<Folder>
		<name>population labels</name>
		<Placemark>
			<name>1</name>
				<description>Further info on the population would go here</description>
				<styleUrl>#msn_ylw-circle</styleUrl>
				<Point>
				<coordinates>-0.69100000000003,48.276,0</coordinates>
				</Point>
			</Placemark>
		<Placemark>
			<name>2</name>
				<description>Further info on the population would go here</description>
				<styleUrl>#msn_ylw-circle</styleUrl>
				<Point>
				<coordinates>-0.59899999999993,48.197,0</coordinates>
				</Point>
			</Placemark>
		<Placemark>
			<name>3</name>
				<description>Further info on the population would go here</description>
				<styleUrl>#msn_ylw-circle</styleUrl>
				<Point>
				<coordinates>-0.59199999999998,48.206,0</coordinates>
				</Point>
			</Placemark>
		<Placemark>
			<name>4</name>
				<description>Further info on the population would go here</description>
				<styleUrl>#msn_ylw-circle</styleUrl>
				<Point>
				<coordinates>-0.58799999999997,48.219,0</coordinates>
				</Point>
			</Placemark>
		<Placemark>
			<name>5</name>
				<description>Further info on the population would go here</description>
				<styleUrl>#msn_ylw-circle</styleUrl>
				<Point>
				<coordinates>-0.58299999999997,48.211,0</coordinates>
				</Point>
			</Placemark>
		<Placemark>
			<name>6</name>
				<description>Further info on the population would go here</description>
				<styleUrl>#msn_ylw-circle</styleUrl>
				<Point>
				<coordinates>-0.58100000000002,48.209,0</coordinates>
				</Point>
			</Placemark>
		<Placemark>
```

<name>7</name>
<description>Further info on the population would go here</description>
<styleUrl>#msn_ylw-circle</styleUrl>
<Point>
<coordinates>-0.57899999999995,48.235,0</coordinates>
</Point>
</Placemark>
<Placemark>
<name>8</name>
<description>Further info on the population would go here</description>
<styleUrl>#msn_ylw-circle</styleUrl>
<Point>
<coordinates>-0.577,48.224,0</coordinates>
</Point>
</Placemark>
<Placemark>
<name>9</name>
<description>Further info on the population would go here</description>
<styleUrl>#msn_ylw-circle</styleUrl>
<Point>
<coordinates>-0.57100000000003,48.23,0</coordinates>
</Point>
</Placemark>
<Placemark>
<name>10</name>
<description>Further info on the population would go here</description>
<styleUrl>#msn_ylw-circle</styleUrl>
<Point>
<coordinates>-0.56999999999994,48.242,0</coordinates>
</Point>
</Placemark>
<Placemark>
<name>11</name>
<description>Further info on the population would go here</description>
<styleUrl>#msn_ylw-circle</styleUrl>
<Point>
<coordinates>-0.56799999999998,48.186,0</coordinates>
</Point>
</Placemark>
<Placemark>
<name>12</name>
<description>Further info on the population would go here</description>
<styleUrl>#msn_ylw-circle</styleUrl>
<Point>
<coordinates>-0.56700000000001,48.233,0</coordinates>
</Point>
</Placemark>
<Placemark>
<name>13</name>
<description>Further info on the population would go here</description>
<styleUrl>#msn_ylw-circle</styleUrl>
<Point>
<coordinates>-0.56399999999996,48.22,0</coordinates>
</Point>
</Placemark>
<Placemark>
<name>14</name>

<description>Further info on the population would go here</description>
<styleUrl>#msn_ylw-circle</styleUrl>
<Point>
<coordinates>-0.56100000000004,48.236,0</coordinates>
</Point>
</Placemark>
<Placemark>
<name>15</name>
<description>Further info on the population would go here</description>
<styleUrl>#msn_ylw-circle</styleUrl>
<Point>
<coordinates>-0.56100000000004,48.189,0</coordinates>
</Point>
</Placemark>
<Placemark>
<name>16</name>
<description>Further info on the population would go here</description>
<styleUrl>#msn_ylw-circle</styleUrl>
<Point>
<coordinates>-0.55600000000004,48.165,0</coordinates>
</Point>
</Placemark>
<Placemark>
<name>17</name>
<description>Further info on the population would go here</description>
<styleUrl>#msn_ylw-circle</styleUrl>
<Point>
<coordinates>-0.55500000000006,48.243,0</coordinates>
</Point>
</Placemark>
<Placemark>
<name>18</name>
<description>Further info on the population would go here</description>
<styleUrl>#msn_ylw-circle</styleUrl>
<Point>
<coordinates>-0.55500000000006,48.243,0</coordinates>
</Point>
</Placemark>
<Placemark>
<name>19</name>
<description>Further info on the population would go here</description>
<styleUrl>#msn_ylw-circle</styleUrl>
<Point>
<coordinates>-0.55500000000006,48.249,0</coordinates>
</Point>
</Placemark>
<Placemark>
<name>20</name>
<description>Further info on the population would go here</description>
<styleUrl>#msn_ylw-circle</styleUrl>
<Point>
<coordinates>-0.53499999999997,48.233,0</coordinates>
</Point>
</Placemark>
<Placemark>
<name>21</name>
<description>Further info on the population would go here</description>

<styleUrl>#msn_ylw-circle</styleUrl>
<Point>
<coordinates>-0.53200000000004,48.245,0</coordinates>
</Point>
</Placemark>
<Placemark>
<name>22</name>
<description>Further info on the population would go here</description>
<styleUrl>#msn_ylw-circle</styleUrl>
<Point>
<coordinates>-0.52999999999997,48.266,0</coordinates>
</Point>
</Placemark>
<Placemark>
<name>23</name>
<description>Further info on the population would go here</description>
<styleUrl>#msn_ylw-circle</styleUrl>
<Point>
<coordinates>-0.52800000000002,48.328,0</coordinates>
</Point>
</Placemark>
<Placemark>
<name>24</name>
<description>Further info on the population would go here</description>
<styleUrl>#msn_ylw-circle</styleUrl>
<Point>
<coordinates>-0.52600000000007,48.234,0</coordinates>
</Point>
</Placemark>
<Placemark>
<name>25</name>
<description>Further info on the population would go here</description>
<styleUrl>#msn_ylw-circle</styleUrl>
<Point>
<coordinates>-0.52499999999998,48.235,0</coordinates>
</Point>
</Placemark>
<Placemark>
<name>26</name>
<description>Further info on the population would go here</description>
<styleUrl>#msn_ylw-circle</styleUrl>
<Point>
<coordinates>-0.52099999999996,48.33,0</coordinates>
</Point>
</Placemark>
<Placemark>
<name>27</name>
<description>Further info on the population would go here</description>
<styleUrl>#msn_ylw-circle</styleUrl>
<Point>
<coordinates>-0.50900000000001,48.212,0</coordinates>
</Point>
</Placemark>
<Placemark>
<name>28</name>
<description>Further info on the population would go here</description>
<styleUrl>#msn_ylw-circle</styleUrl>

<Point>
<coordinates>-0.505,48.229,0</coordinates>
</Point>
</Placemark>
<Placemark>
<name>29</name>
<description>Further info on the population would go here</description>
<styleUrl>#msn_ylw-circle</styleUrl>
<Point>
<coordinates>-0.5,48.198,0</coordinates>
</Point>
</Placemark>
<Placemark>
<name>30</name>
<description>Further info on the population would go here</description>
<styleUrl>#msn_ylw-circle</styleUrl>
<Point>
<coordinates>-0.495,48.215,0</coordinates>
</Point>
</Placemark>
<Placemark>
<name>31</name>
<description>Further info on the population would go here</description>
<styleUrl>#msn_ylw-circle</styleUrl>
<Point>
<coordinates>-0.48800000000006,48.219,0</coordinates>
</Point>
</Placemark>
<Placemark>
<name>32</name>
<description>Further info on the population would go here</description>
<styleUrl>#msn_ylw-circle</styleUrl>
<Point>
<coordinates>-0.48299999999995,48.205,0</coordinates>
</Point>
</Placemark>
<Placemark>
<name>33</name>
<description>Further info on the population would go here</description>
<styleUrl>#msn_ylw-circle</styleUrl>
<Point>
<coordinates>-0.47800000000007,48.275,0</coordinates>
</Point>
</Placemark>
<Placemark>
<name>34</name>
<description>Further info on the population would go here</description>
<styleUrl>#msn_ylw-circle</styleUrl>
<Point>
<coordinates>-0.47500000000002,48.27,0</coordinates>
</Point>
</Placemark>
<Placemark>
<name>35</name>
<description>Further info on the population would go here</description>
<styleUrl>#msn_ylw-circle</styleUrl>
<Point>

<coordinates>-0.473999999999993,48.269,0</coordinates>
</Point>
</Placemark>
<Placemark>
<name>36</name>
<description>Further info on the population would go here</description>
<styleUrl>#msn_ylw-circle</styleUrl>
<Point>
<coordinates>-0.461999999999999,48.226,0</coordinates>
</Point>
</Placemark>
<Placemark>
<name>37</name>
<description>Further info on the population would go here</description>
<styleUrl>#msn_ylw-circle</styleUrl>
<Point>
<coordinates>-0.461000000000001,48.221,0</coordinates>
</Point>
</Placemark>
<Placemark>
<name>38</name>
<description>Further info on the population would go here</description>
<styleUrl>#msn_ylw-circle</styleUrl>
<Point>
<coordinates>-0.452,48.221,0</coordinates>
</Point>
</Placemark>
<Placemark>
<name>39</name>
<description>Further info on the population would go here</description>
<styleUrl>#msn_ylw-circle</styleUrl>
<Point>
<coordinates>-0.430000000000006,48.284,0</coordinates>
</Point>
</Placemark>
<Placemark>
<name>40</name>
<description>Further info on the population would go here</description>
<styleUrl>#msn_ylw-circle</styleUrl>
<Point>
<coordinates>-0.428,48.264,0</coordinates>
</Point>
</Placemark>
<Placemark>
<name>41</name>
<description>Further info on the population would go here</description>
<styleUrl>#msn_ylw-circle</styleUrl>
<Point>
<coordinates>-0.404999999999997,48.237,0</coordinates>
</Point>
</Placemark>
<Placemark>
<name>42</name>
<description>Further info on the population would go here</description>
<styleUrl>#msn_ylw-circle</styleUrl>
<Point>
<coordinates>-1.158,47.844,0</coordinates>

</Point>
</Placemark>
<Placemark>
<name>43</name>
<description>Further info on the population would go here</description>
<styleUrl>#msn_ylw-circle</styleUrl>
<Point>
<coordinates>-1.139,47.834,0</coordinates>
</Point>
</Placemark>
<Placemark>
<name>44</name>
<description>Further info on the population would go here</description>
<styleUrl>#msn_ylw-circle</styleUrl>
<Point>
<coordinates>-1.081,47.895,0</coordinates>
</Point>
</Placemark>
<Placemark>
<name>45</name>
<description>Further info on the population would go here</description>
<styleUrl>#msn_ylw-circle</styleUrl>
<Point>
<coordinates>-1.066,48.448,0</coordinates>
</Point>
</Placemark>
<Placemark>
<name>46</name>
<description>Further info on the population would go here</description>
<styleUrl>#msn_ylw-circle</styleUrl>
<Point>
<coordinates>-1.064,48.18,0</coordinates>
</Point>
</Placemark>
<Placemark>
<name>47</name>
<description>Further info on the population would go here</description>
<styleUrl>#msn_ylw-circle</styleUrl>
<Point>
<coordinates>-1.057,48.141,0</coordinates>
</Point>
</Placemark>
<Placemark>
<name>48</name>
<description>Further info on the population would go here</description>
<styleUrl>#msn_ylw-circle</styleUrl>
<Point>
<coordinates>-1.057,48.142,0</coordinates>
</Point>
</Placemark>
<Placemark>
<name>49</name>
<description>Further info on the population would go here</description>
<styleUrl>#msn_ylw-circle</styleUrl>
<Point>
<coordinates>-1.056,48.145,0</coordinates>
</Point>

</Placemark>
<Placemark>
<name>50</name>
<description>Further info on the population would go here</description>
<styleUrl>#msn_ylw-circle</styleUrl>
<Point>
<coordinates>-1.0509999999999,48.236,0</coordinates>
</Point>
</Placemark>
<Placemark>
<name>51</name>
<description>Further info on the population would go here</description>
<styleUrl>#msn_ylw-circle</styleUrl>
<Point>
<coordinates>-1.049,47.961,0</coordinates>
</Point>
</Placemark>
<Placemark>
<name>52</name>
<description>Further info on the population would go here</description>
<styleUrl>#msn_ylw-circle</styleUrl>
<Point>
<coordinates>-1.048,47.873,0</coordinates>
</Point>
</Placemark>
<Placemark>
<name>53</name>
<description>Further info on the population would go here</description>
<styleUrl>#msn_ylw-circle</styleUrl>
<Point>
<coordinates>-1.048,48.14,0</coordinates>
</Point>
</Placemark>
<Placemark>
<name>54</name>
<description>Further info on the population would go here</description>
<styleUrl>#msn_ylw-circle</styleUrl>
<Point>
<coordinates>-1.048,47.962,0</coordinates>
</Point>
</Placemark>
<Placemark>
<name>55</name>
<description>Further info on the population would go here</description>
<styleUrl>#msn_ylw-circle</styleUrl>
<Point>
<coordinates>-1.046,48.233,0</coordinates>
</Point>
</Placemark>
<Placemark>
<name>56</name>
<description>Further info on the population would go here</description>
<styleUrl>#msn_ylw-circle</styleUrl>
<Point>
<coordinates>-1.046,47.874,0</coordinates>
</Point>
</Placemark>

<Placemark>
<name>57</name>
<description>Further info on the population would go here</description>
<styleUrl>#msn_ylw-circle</styleUrl>
<Point>
<coordinates>-1.0409999999999,47.859,0</coordinates>
</Point>
</Placemark>
<Placemark>
<name>58</name>
<description>Further info on the population would go here</description>
<styleUrl>#msn_ylw-circle</styleUrl>
<Point>
<coordinates>-1.04,47.966,0</coordinates>
</Point>
</Placemark>
<Placemark>
<name>59</name>
<description>Further info on the population would go here</description>
<styleUrl>#msn_ylw-circle</styleUrl>
<Point>
<coordinates>-1.037,48.408,0</coordinates>
</Point>
</Placemark>
<Placemark>
<name>60</name>
<description>Further info on the population would go here</description>
<styleUrl>#msn_ylw-circle</styleUrl>
<Point>
<coordinates>-1.029,47.959,0</coordinates>
</Point>
</Placemark>
<Placemark>
<name>61</name>
<description>Further info on the population would go here</description>
<styleUrl>#msn_ylw-circle</styleUrl>
<Point>
<coordinates>-1.023,47.908,0</coordinates>
</Point>
</Placemark>
<Placemark>
<name>62</name>
<description>Further info on the population would go here</description>
<styleUrl>#msn_ylw-circle</styleUrl>
<Point>
<coordinates>-1.015,48.164,0</coordinates>
</Point>
</Placemark>
<Placemark>
<name>63</name>
<description>Further info on the population would go here</description>
<styleUrl>#msn_ylw-circle</styleUrl>
<Point>
<coordinates>-1.009,48.268,0</coordinates>
</Point>
</Placemark>
<Placemark>

<name>64</name>
<description>Further info on the population would go here</description>
<styleUrl>#msn_ylw-circle</styleUrl>
<Point>
<coordinates>-1,48.113,0</coordinates>
</Point>
</Placemark>
<Placemark>
<name>65</name>
<description>Further info on the population would go here</description>
<styleUrl>#msn_ylw-circle</styleUrl>
<Point>
<coordinates>-0.99900000000002,48.452,0</coordinates>
</Point>
</Placemark>
<Placemark>
<name>66</name>
<description>Further info on the population would go here</description>
<styleUrl>#msn_ylw-circle</styleUrl>
<Point>
<coordinates>-0.99800000000005,48.051,0</coordinates>
</Point>
</Placemark>
<Placemark>
<name>67</name>
<description>Further info on the population would go here</description>
<styleUrl>#msn_ylw-circle</styleUrl>
<Point>
<coordinates>-0.99700000000007,48.447,0</coordinates>
</Point>
</Placemark>
<Placemark>
<name>68</name>
<description>Further info on the population would go here</description>
<styleUrl>#msn_ylw-circle</styleUrl>
<Point>
<coordinates>-0.98500000000001,48.262,0</coordinates>
</Point>
</Placemark>
<Placemark>
<name>69</name>
<description>Further info on the population would go here</description>
<styleUrl>#msn_ylw-circle</styleUrl>
<Point>
<coordinates>-0.98299999999995,48.238,0</coordinates>
</Point>
</Placemark>
<Placemark>
<name>70</name>
<description>Further info on the population would go here</description>
<styleUrl>#msn_ylw-circle</styleUrl>
<Point>
<coordinates>-0.97800000000007,48.026,0</coordinates>
</Point>
</Placemark>
<Placemark>
<name>71</name>

<description>Further info on the population would go here</description>
<styleUrl>#msn_ylw-circle</styleUrl>
<Point>
<coordinates>-0.97299999999996,48.443,0</coordinates>
</Point>
</Placemark>
<Placemark>
<name>72</name>
<description>Further info on the population would go here</description>
<styleUrl>#msn_ylw-circle</styleUrl>
<Point>
<coordinates>-0.96699999999998,48.144,0</coordinates>
</Point>
</Placemark>
<Placemark>
<name>73</name>
<description>Further info on the population would go here</description>
<styleUrl>#msn_ylw-circle</styleUrl>
<Point>
<coordinates>-0.96500000000003,48.228,0</coordinates>
</Point>
</Placemark>
<Placemark>
<name>74</name>
<description>Further info on the population would go here</description>
<styleUrl>#msn_ylw-circle</styleUrl>
<Point>
<coordinates>-0.96100000000001,48.279,0</coordinates>
</Point>
</Placemark>
<Placemark>
<name>75</name>
<description>Further info on the population would go here</description>
<styleUrl>#msn_ylw-circle</styleUrl>
<Point>
<coordinates>-0.94799999999998,47.823,0</coordinates>
</Point>
</Placemark>
<Placemark>
<name>76</name>
<description>Further info on the population would go here</description>
<styleUrl>#msn_ylw-circle</styleUrl>
<Point>
<coordinates>-0.947,48.176,0</coordinates>
</Point>
</Placemark>
<Placemark>
<name>77</name>
<description>Further info on the population would go here</description>
<styleUrl>#msn_ylw-circle</styleUrl>
<Point>
<coordinates>-0.94600000000003,48.291,0</coordinates>
</Point>
</Placemark>
<Placemark>
<name>78</name>
<description>Further info on the population would go here</description>

<styleUrl>#msn_ylw-circle</styleUrl>
<Point>
<coordinates>-0.943999999999996,47.803,0</coordinates>
</Point>
</Placemark>
<Placemark>
<name>79</name>
<description>Further info on the population would go here</description>
<styleUrl>#msn_ylw-circle</styleUrl>
<Point>
<coordinates>-0.933999999999997,48.227,0</coordinates>
</Point>
</Placemark>
<Placemark>
<name>80</name>
<description>Further info on the population would go here</description>
<styleUrl>#msn_ylw-circle</styleUrl>
<Point>
<coordinates>-0.932000000000002,47.945,0</coordinates>
</Point>
</Placemark>
<Placemark>
<name>81</name>
<description>Further info on the population would go here</description>
<styleUrl>#msn_ylw-circle</styleUrl>
<Point>
<coordinates>-0.927000000000002,48.017,0</coordinates>
</Point>
</Placemark>
<Placemark>
<name>82</name>
<description>Further info on the population would go here</description>
<styleUrl>#msn_ylw-circle</styleUrl>
<Point>
<coordinates>-0.923999999999998,48.016,0</coordinates>
</Point>
</Placemark>
<Placemark>
<name>83</name>
<description>Further info on the population would go here</description>
<styleUrl>#msn_ylw-circle</styleUrl>
<Point>
<coordinates>-0.918999999999998,47.864,0</coordinates>
</Point>
</Placemark>
<Placemark>
<name>84</name>
<description>Further info on the population would go here</description>
<styleUrl>#msn_ylw-circle</styleUrl>
<Point>
<coordinates>-0.918000000000001,48.097,0</coordinates>
</Point>
</Placemark>
<Placemark>
<name>85</name>
<description>Further info on the population would go here</description>
<styleUrl>#msn_ylw-circle</styleUrl>

<Point>
<coordinates>-0.91700000000003,48.219,0</coordinates>
</Point>
</Placemark>
<Placemark>
<name>86</name>
<description>Further info on the population would go here</description>
<styleUrl>#msn_ylw-circle</styleUrl>
<Point>
<coordinates>-0.90100000000007,48.034,0</coordinates>
</Point>
</Placemark>
<Placemark>
<name>87</name>
<description>Further info on the population would go here</description>
<styleUrl>#msn_ylw-circle</styleUrl>
<Point>
<coordinates>-0.89999999999998,48.396,0</coordinates>
</Point>
</Placemark>
<Placemark>
<name>88</name>
<description>Further info on the population would go here</description>
<styleUrl>#msn_ylw-circle</styleUrl>
<Point>
<coordinates>-0.89999999999998,48.396,0</coordinates>
</Point>
</Placemark>
<Placemark>
<name>89</name>
<description>Further info on the population would go here</description>
<styleUrl>#msn_ylw-circle</styleUrl>
<Point>
<coordinates>-0.89599999999996,48.037,0</coordinates>
</Point>
</Placemark>
<Placemark>
<name>90</name>
<description>Further info on the population would go here</description>
<styleUrl>#msn_ylw-circle</styleUrl>
<Point>
<coordinates>-0.88099999999997,48.193,0</coordinates>
</Point>
</Placemark>
<Placemark>
<name>91</name>
<description>Further info on the population would go here</description>
<styleUrl>#msn_ylw-circle</styleUrl>
<Point>
<coordinates>-0.88,48.11,0</coordinates>
</Point>
</Placemark>
<Placemark>
<name>92</name>
<description>Further info on the population would go here</description>
<styleUrl>#msn_ylw-circle</styleUrl>
<Point>

<coordinates>-0.87900000000002,48.189,0</coordinates>
</Point>
</Placemark>
<Placemark>
<name>93</name>
<description>Further info on the population would go here</description>
<styleUrl>#msn_ylw-circle</styleUrl>
<Point>
<coordinates>-0.87900000000002,48.045,0</coordinates>
</Point>
</Placemark>
<Placemark>
<name>94</name>
<description>Further info on the population would go here</description>
<styleUrl>#msn_ylw-circle</styleUrl>
<Point>
<coordinates>-0.87699999999995,48.188,0</coordinates>
</Point>
</Placemark>
<Placemark>
<name>95</name>
<description>Further info on the population would go here</description>
<styleUrl>#msn_ylw-circle</styleUrl>
<Point>
<coordinates>-0.87599999999998,47.989,0</coordinates>
</Point>
</Placemark>
<Placemark>
<name>96</name>
<description>Further info on the population would go here</description>
<styleUrl>#msn_ylw-circle</styleUrl>
<Point>
<coordinates>-0.87599999999998,48.452,0</coordinates>
</Point>
</Placemark>
<Placemark>
<name>97</name>
<description>Further info on the population would go here</description>
<styleUrl>#msn_ylw-circle</styleUrl>
<Point>
<coordinates>-0.875,48.188,0</coordinates>
</Point>
</Placemark>
<Placemark>
<name>98</name>
<description>Further info on the population would go here</description>
<styleUrl>#msn_ylw-circle</styleUrl>
<Point>
<coordinates>-0.875,48.048,0</coordinates>
</Point>
</Placemark>
<Placemark>
<name>99</name>
<description>Further info on the population would go here</description>
<styleUrl>#msn_ylw-circle</styleUrl>
<Point>
<coordinates>-0.87200000000007,48.192,0</coordinates>

</Point>
</Placemark>
<Placemark>
<name>100</name>
<description>Further info on the population would go here</description>
<styleUrl>#msn_ylw-circle</styleUrl>
<Point>
<coordinates>-0.87200000000007,48.148,0</coordinates>
</Point>
</Placemark>
<Placemark>
<name>101</name>
<description>Further info on the population would go here</description>
<styleUrl>#msn_ylw-circle</styleUrl>
<Point>
<coordinates>-0.86900000000003,48.148,0</coordinates>
</Point>
</Placemark>
<Placemark>
<name>102</name>
<description>Further info on the population would go here</description>
<styleUrl>#msn_ylw-circle</styleUrl>
<Point>
<coordinates>-0.86900000000003,48.447,0</coordinates>
</Point>
</Placemark>
<Placemark>
<name>103</name>
<description>Further info on the population would go here</description>
<styleUrl>#msn_ylw-circle</styleUrl>
<Point>
<coordinates>-0.86699999999996,47.91,0</coordinates>
</Point>
</Placemark>
<Placemark>
<name>104</name>
<description>Further info on the population would go here</description>
<styleUrl>#msn_ylw-circle</styleUrl>
<Point>
<coordinates>-0.86400000000003,48.076,0</coordinates>
</Point>
</Placemark>
<Placemark>
<name>105</name>
<description>Further info on the population would go here</description>
<styleUrl>#msn_ylw-circle</styleUrl>
<Point>
<coordinates>-0.86400000000003,48.279,0</coordinates>
</Point>
</Placemark>
<Placemark>
<name>106</name>
<description>Further info on the population would go here</description>
<styleUrl>#msn_ylw-circle</styleUrl>
<Point>
<coordinates>-0.85400000000004,48.056,0</coordinates>
</Point>

</Placemark>
<Placemark>
<name>107</name>
<description>Further info on the population would go here</description>
<styleUrl>#msn_ylw-circle</styleUrl>
<Point>
<coordinates>-0.85300000000007,48.15,0</coordinates>
</Point>
</Placemark>
<Placemark>
<name>108</name>
<description>Further info on the population would go here</description>
<styleUrl>#msn_ylw-circle</styleUrl>
<Point>
<coordinates>-0.85300000000007,48.151,0</coordinates>
</Point>
</Placemark>
<Placemark>
<name>109</name>
<description>Further info on the population would go here</description>
<styleUrl>#msn_ylw-circle</styleUrl>
<Point>
<coordinates>-0.85000000000002,48.15,0</coordinates>
</Point>
</Placemark>
<Placemark>
<name>110</name>
<description>Further info on the population would go here</description>
<styleUrl>#msn_ylw-circle</styleUrl>
<Point>
<coordinates>-0.84899999999993,48.15,0</coordinates>
</Point>
</Placemark>
<Placemark>
<name>111</name>
<description>Further info on the population would go here</description>
<styleUrl>#msn_ylw-circle</styleUrl>
<Point>
<coordinates>-0.84899999999993,48.191,0</coordinates>
</Point>
</Placemark>
<Placemark>
<name>112</name>
<description>Further info on the population would go here</description>
<styleUrl>#msn_ylw-circle</styleUrl>
<Point>
<coordinates>-0.84799999999996,48.197,0</coordinates>
</Point>
</Placemark>
<Placemark>
<name>113</name>
<description>Further info on the population would go here</description>
<styleUrl>#msn_ylw-circle</styleUrl>
<Point>
<coordinates>-0.84799999999996,48.151,0</coordinates>
</Point>
</Placemark>

<Placemark>
<name>114</name>
<description>Further info on the population would go here</description>
<styleUrl>#msn_ylw-circle</styleUrl>
<Point>
<coordinates>-0.84699999999998,48.191,0</coordinates>
</Point>
</Placemark>
<Placemark>
<name>115</name>
<description>Further info on the population would go here</description>
<styleUrl>#msn_ylw-circle</styleUrl>
<Point>
<coordinates>-0.84500000000003,48.114,0</coordinates>
</Point>
</Placemark>
<Placemark>
<name>116</name>
<description>Further info on the population would go here</description>
<styleUrl>#msn_ylw-circle</styleUrl>
<Point>
<coordinates>-0.84500000000003,48.114,0</coordinates>
</Point>
</Placemark>
<Placemark>
<name>117</name>
<description>Further info on the population would go here</description>
<styleUrl>#msn_ylw-circle</styleUrl>
<Point>
<coordinates>-0.84299999999996,48.26,0</coordinates>
</Point>
</Placemark>
<Placemark>
<name>118</name>
<description>Further info on the population would go here</description>
<styleUrl>#msn_ylw-circle</styleUrl>
<Point>
<coordinates>-0.83799999999997,48.222,0</coordinates>
</Point>
</Placemark>
<Placemark>
<name>119</name>
<description>Further info on the population would go here</description>
<styleUrl>#msn_ylw-circle</styleUrl>
<Point>
<coordinates>-0.83799999999997,48.22,0</coordinates>
</Point>
</Placemark>
<Placemark>
<name>120</name>
<description>Further info on the population would go here</description>
<styleUrl>#msn_ylw-circle</styleUrl>
<Point>
<coordinates>-0.83600000000001,48.156,0</coordinates>
</Point>
</Placemark>
<Placemark>

<name>121</name>
<description>Further info on the population would go here</description>
<styleUrl>#msn_ylw-circle</styleUrl>
<Point>
<coordinates>-0.83400000000006,47.967,0</coordinates>
</Point>
</Placemark>
<Placemark>
<name>122</name>
<description>Further info on the population would go here</description>
<styleUrl>#msn_ylw-circle</styleUrl>
<Point>
<coordinates>-0.83400000000006,48.155,0</coordinates>
</Point>
</Placemark>
<Placemark>
<name>123</name>
<description>Further info on the population would go here</description>
<styleUrl>#msn_ylw-circle</styleUrl>
<Point>
<coordinates>-0.83100000000002,48.463,0</coordinates>
</Point>
</Placemark>
<Placemark>
<name>124</name>
<description>Further info on the population would go here</description>
<styleUrl>#msn_ylw-circle</styleUrl>
<Point>
<coordinates>-0.83000000000004,48.203,0</coordinates>
</Point>
</Placemark>
<Placemark>
<name>125</name>
<description>Further info on the population would go here</description>
<styleUrl>#msn_ylw-circle</styleUrl>
<Point>
<coordinates>-0.82899999999995,48.203,0</coordinates>
</Point>
</Placemark>
<Placemark>
<name>126</name>
<description>Further info on the population would go here</description>
<styleUrl>#msn_ylw-circle</styleUrl>
<Point>
<coordinates>-0.81299999999999,48.163,0</coordinates>
</Point>
</Placemark>
<Placemark>
<name>127</name>
<description>Further info on the population would go here</description>
<styleUrl>#msn_ylw-circle</styleUrl>
<Point>
<coordinates>-0.81200000000001,48.163,0</coordinates>
</Point>
</Placemark>
<Placemark>
<name>128</name>

<description>Further info on the population would go here</description>
<styleUrl>#msn_ylw-circle</styleUrl>
<Point>
<coordinates>-0.80999999999995,48.059,0</coordinates>
</Point>
</Placemark>
<Placemark>
<name>129</name>
<description>Further info on the population would go here</description>
<styleUrl>#msn_ylw-circle</styleUrl>
<Point>
<coordinates>-0.80600000000004,47.946,0</coordinates>
</Point>
</Placemark>
<Placemark>
<name>130</name>
<description>Further info on the population would go here</description>
<styleUrl>#msn_ylw-circle</styleUrl>
<Point>
<coordinates>-0.798,47.906,0</coordinates>
</Point>
</Placemark>
<Placemark>
<name>131</name>
<description>Further info on the population would go here</description>
<styleUrl>#msn_ylw-circle</styleUrl>
<Point>
<coordinates>-0.79600000000005,47.877,0</coordinates>
</Point>
</Placemark>
<Placemark>
<name>132</name>
<description>Further info on the population would go here</description>
<styleUrl>#msn_ylw-circle</styleUrl>
<Point>
<coordinates>-0.79399999999998,47.916,0</coordinates>
</Point>
</Placemark>
<Placemark>
<name>133</name>
<description>Further info on the population would go here</description>
<styleUrl>#msn_ylw-circle</styleUrl>
<Point>
<coordinates>-0.79200000000003,47.811,0</coordinates>
</Point>
</Placemark>
<Placemark>
<name>134</name>
<description>Further info on the population would go here</description>
<styleUrl>#msn_ylw-circle</styleUrl>
<Point>
<coordinates>-0.78800000000001,48.055,0</coordinates>
</Point>
</Placemark>
<Placemark>
<name>135</name>
<description>Further info on the population would go here</description>

<styleUrl>#msn_ylw-circle</styleUrl>
<Point>
<coordinates>-0.78800000000001,48.034,0</coordinates>
</Point>
</Placemark>
<Placemark>
<name>136</name>
<description>Further info on the population would go here</description>
<styleUrl>#msn_ylw-circle</styleUrl>
<Point>
<coordinates>-0.78800000000001,48.037,0</coordinates>
</Point>
</Placemark>
<Placemark>
<name>137</name>
<description>Further info on the population would go here</description>
<styleUrl>#msn_ylw-circle</styleUrl>
<Point>
<coordinates>-0.78700000000003,48.047,0</coordinates>
</Point>
</Placemark>
<Placemark>
<name>138</name>
<description>Further info on the population would go here</description>
<styleUrl>#msn_ylw-circle</styleUrl>
<Point>
<coordinates>-0.78700000000003,47.814,0</coordinates>
</Point>
</Placemark>
<Placemark>
<name>139</name>
<description>Further info on the population would go here</description>
<styleUrl>#msn_ylw-circle</styleUrl>
<Point>
<coordinates>-0.78700000000003,48.289,0</coordinates>
</Point>
</Placemark>
<Placemark>
<name>140</name>
<description>Further info on the population would go here</description>
<styleUrl>#msn_ylw-circle</styleUrl>
<Point>
<coordinates>-0.78600000000006,48.289,0</coordinates>
</Point>
</Placemark>
<Placemark>
<name>141</name>
<description>Further info on the population would go here</description>
<styleUrl>#msn_ylw-circle</styleUrl>
<Point>
<coordinates>-0.78600000000006,47.8,0</coordinates>
</Point>
</Placemark>
<Placemark>
<name>142</name>
<description>Further info on the population would go here</description>
<styleUrl>#msn_ylw-circle</styleUrl>

<Point>
<coordinates>-0.78600000000006,48.287,0</coordinates>
</Point>
</Placemark>
<Placemark>
<name>143</name>
<description>Further info on the population would go here</description>
<styleUrl>#msn_ylw-circle</styleUrl>
<Point>
<coordinates>-0.78300000000002,47.863,0</coordinates>
</Point>
</Placemark>
<Placemark>
<name>144</name>
<description>Further info on the population would go here</description>
<styleUrl>#msn_ylw-circle</styleUrl>
<Point>
<coordinates>-0.78300000000002,48.048,0</coordinates>
</Point>
</Placemark>
<Placemark>
<name>145</name>
<description>Further info on the population would go here</description>
<styleUrl>#msn_ylw-circle</styleUrl>
<Point>
<coordinates>-0.78200000000004,48.393,0</coordinates>
</Point>
</Placemark>
<Placemark>
<name>146</name>
<description>Further info on the population would go here</description>
<styleUrl>#msn_ylw-circle</styleUrl>
<Point>
<coordinates>-0.78200000000004,48.097,0</coordinates>
</Point>
</Placemark>
<Placemark>
<name>147</name>
<description>Further info on the population would go here</description>
<styleUrl>#msn_ylw-circle</styleUrl>
<Point>
<coordinates>-0.78099999999995,48.245,0</coordinates>
</Point>
</Placemark>
<Placemark>
<name>148</name>
<description>Further info on the population would go here</description>
<styleUrl>#msn_ylw-circle</styleUrl>
<Point>
<coordinates>-0.779,48.008,0</coordinates>
</Point>
</Placemark>
<Placemark>
<name>149</name>
<description>Further info on the population would go here</description>
<styleUrl>#msn_ylw-circle</styleUrl>
<Point>

<coordinates>-0.77800000000002,48.253,0</coordinates>
</Point>
</Placemark>
<Placemark>
<name>150</name>
<description>Further info on the population would go here</description>
<styleUrl>#msn_ylw-circle</styleUrl>
<Point>
<coordinates>-0.77700000000004,48.011,0</coordinates>
</Point>
</Placemark>
<Placemark>
<name>151</name>
<description>Further info on the population would go here</description>
<styleUrl>#msn_ylw-circle</styleUrl>
<Point>
<coordinates>-0.77700000000004,47.763,0</coordinates>
</Point>
</Placemark>
<Placemark>
<name>152</name>
<description>Further info on the population would go here</description>
<styleUrl>#msn_ylw-circle</styleUrl>
<Point>
<coordinates>-0.77700000000004,48.005,0</coordinates>
</Point>
</Placemark>
<Placemark>
<name>153</name>
<description>Further info on the population would go here</description>
<styleUrl>#msn_ylw-circle</styleUrl>
<Point>
<coordinates>-0.77700000000004,47.788,0</coordinates>
</Point>
</Placemark>
<Placemark>
<name>154</name>
<description>Further info on the population would go here</description>
<styleUrl>#msn_ylw-circle</styleUrl>
<Point>
<coordinates>-0.77600000000007,48.229,0</coordinates>
</Point>
</Placemark>
<Placemark>
<name>155</name>
<description>Further info on the population would go here</description>
<styleUrl>#msn_ylw-circle</styleUrl>
<Point>
<coordinates>-0.77300000000002,48.2,0</coordinates>
</Point>
</Placemark>
<Placemark>
<name>156</name>
<description>Further info on the population would go here</description>
<styleUrl>#msn_ylw-circle</styleUrl>
<Point>
<coordinates>-0.77300000000002,47.886,0</coordinates>

</Point>
</Placemark>
<Placemark>
<name>157</name>
<description>Further info on the population would go here</description>
<styleUrl>#msn_ylw-circle</styleUrl>
<Point>
<coordinates>-0.77300000000002,47.887,0</coordinates>
</Point>
</Placemark>
<Placemark>
<name>158</name>
<description>Further info on the population would go here</description>
<styleUrl>#msn_ylw-circle</styleUrl>
<Point>
<coordinates>-0.77300000000002,48.101,0</coordinates>
</Point>
</Placemark>
<Placemark>
<name>159</name>
<description>Further info on the population would go here</description>
<styleUrl>#msn_ylw-circle</styleUrl>
<Point>
<coordinates>-0.77199999999993,48.031,0</coordinates>
</Point>
</Placemark>
<Placemark>
<name>160</name>
<description>Further info on the population would go here</description>
<styleUrl>#msn_ylw-circle</styleUrl>
<Point>
<coordinates>-0.77199999999993,48.101,0</coordinates>
</Point>
</Placemark>
<Placemark>
<name>161</name>
<description>Further info on the population would go here</description>
<styleUrl>#msn_ylw-circle</styleUrl>
<Point>
<coordinates>-0.77099999999996,47.792,0</coordinates>
</Point>
</Placemark>
<Placemark>
<name>162</name>
<description>Further info on the population would go here</description>
<styleUrl>#msn_ylw-circle</styleUrl>
<Point>
<coordinates>-0.76999999999998,48.214,0</coordinates>
</Point>
</Placemark>
<Placemark>
<name>163</name>
<description>Further info on the population would go here</description>
<styleUrl>#msn_ylw-circle</styleUrl>
<Point>
<coordinates>-0.76900000000001,48.034,0</coordinates>
</Point>

</Placemark>
<Placemark>
<name>164</name>
<description>Further info on the population would go here</description>
<styleUrl>#msn_ylw-circle</styleUrl>
<Point>
<coordinates>-0.76800000000003,48.306,0</coordinates>
</Point>
</Placemark>
<Placemark>
<name>165</name>
<description>Further info on the population would go here</description>
<styleUrl>#msn_ylw-circle</styleUrl>
<Point>
<coordinates>-0.76700000000005,48.102,0</coordinates>
</Point>
</Placemark>
<Placemark>
<name>166</name>
<description>Further info on the population would go here</description>
<styleUrl>#msn_ylw-circle</styleUrl>
<Point>
<coordinates>-0.76599999999996,48.181,0</coordinates>
</Point>
</Placemark>
<Placemark>
<name>167</name>
<description>Further info on the population would go here</description>
<styleUrl>#msn_ylw-circle</styleUrl>
<Point>
<coordinates>-0.76599999999996,48.181,0</coordinates>
</Point>
</Placemark>
<Placemark>
<name>168</name>
<description>Further info on the population would go here</description>
<styleUrl>#msn_ylw-circle</styleUrl>
<Point>
<coordinates>-0.76499999999999,48.179,0</coordinates>
</Point>
</Placemark>
<Placemark>
<name>169</name>
<description>Further info on the population would go here</description>
<styleUrl>#msn_ylw-circle</styleUrl>
<Point>
<coordinates>-0.76400000000001,48.308,0</coordinates>
</Point>
</Placemark>
<Placemark>
<name>170</name>
<description>Further info on the population would go here</description>
<styleUrl>#msn_ylw-circle</styleUrl>
<Point>
<coordinates>-0.76300000000003,47.816,0</coordinates>
</Point>
</Placemark>

<Placemark>
<name>171</name>
<description>Further info on the population would go here</description>
<styleUrl>#msn_ylw-circle</styleUrl>
<Point>
<coordinates>-0.76300000000003,48.163,0</coordinates>
</Point>
</Placemark>
<Placemark>
<name>172</name>
<description>Further info on the population would go here</description>
<styleUrl>#msn_ylw-circle</styleUrl>
<Point>
<coordinates>-0.76099999999997,48.026,0</coordinates>
</Point>
</Placemark>
<Placemark>
<name>173</name>
<description>Further info on the population would go here</description>
<styleUrl>#msn_ylw-circle</styleUrl>
<Point>
<coordinates>-0.75999999999999,48.224,0</coordinates>
</Point>
</Placemark>
<Placemark>
<name>174</name>
<description>Further info on the population would go here</description>
<styleUrl>#msn_ylw-circle</styleUrl>
<Point>
<coordinates>-0.75900000000001,48.255,0</coordinates>
</Point>
</Placemark>
<Placemark>
<name>175</name>
<description>Further info on the population would go here</description>
<styleUrl>#msn_ylw-circle</styleUrl>
<Point>
<coordinates>-0.75700000000006,48.185,0</coordinates>
</Point>
</Placemark>
<Placemark>
<name>176</name>
<description>Further info on the population would go here</description>
<styleUrl>#msn_ylw-circle</styleUrl>
<Point>
<coordinates>-0.75599999999997,48.184,0</coordinates>
</Point>
</Placemark>
<Placemark>
<name>177</name>
<description>Further info on the population would go here</description>
<styleUrl>#msn_ylw-circle</styleUrl>
<Point>
<coordinates>-0.75299999999993,48.259,0</coordinates>
</Point>
</Placemark>
<Placemark>

<name>178</name>
<description>Further info on the population would go here</description>
<styleUrl>#msn_ylw-circle</styleUrl>
<Point>
<coordinates>-0.75199999999995,48.179,0</coordinates>
</Point>
</Placemark>
<Placemark>
<name>179</name>
<description>Further info on the population would go here</description>
<styleUrl>#msn_ylw-circle</styleUrl>
<Point>
<coordinates>-0.75099999999998,48.095,0</coordinates>
</Point>
</Placemark>
<Placemark>
<name>180</name>
<description>Further info on the population would go here</description>
<styleUrl>#msn_ylw-circle</styleUrl>
<Point>
<coordinates>-0.74900000000002,48.147,0</coordinates>
</Point>
</Placemark>
<Placemark>
<name>181</name>
<description>Further info on the population would go here</description>
<styleUrl>#msn_ylw-circle</styleUrl>
<Point>
<coordinates>-0.74900000000002,48.148,0</coordinates>
</Point>
</Placemark>
<Placemark>
<name>182</name>
<description>Further info on the population would go here</description>
<styleUrl>#msn_ylw-circle</styleUrl>
<Point>
<coordinates>-0.74700000000007,47.996,0</coordinates>
</Point>
</Placemark>
<Placemark>
<name>183</name>
<description>Further info on the population would go here</description>
<styleUrl>#msn_ylw-circle</styleUrl>
<Point>
<coordinates>-0.74599999999998,47.996,0</coordinates>
</Point>
</Placemark>
<Placemark>
<name>184</name>
<description>Further info on the population would go here</description>
<styleUrl>#msn_ylw-circle</styleUrl>
<Point>
<coordinates>-0.745,48.173,0</coordinates>
</Point>
</Placemark>
<Placemark>
<name>185</name>

<description>Further info on the population would go here</description>
<styleUrl>#msn_ylw-circle</styleUrl>
<Point>
<coordinates>-0.74400000000003,48.131,0</coordinates>
</Point>
</Placemark>
<Placemark>
<name>186</name>
<description>Further info on the population would go here</description>
<styleUrl>#msn_ylw-circle</styleUrl>
<Point>
<coordinates>-0.74400000000003,48.309,0</coordinates>
</Point>
</Placemark>
<Placemark>
<name>187</name>
<description>Further info on the population would go here</description>
<styleUrl>#msn_ylw-circle</styleUrl>
<Point>
<coordinates>-0.74299999999994,48.112,0</coordinates>
</Point>
</Placemark>
<Placemark>
<name>188</name>
<description>Further info on the population would go here</description>
<styleUrl>#msn_ylw-circle</styleUrl>
<Point>
<coordinates>-0.74199999999996,48.173,0</coordinates>
</Point>
</Placemark>
<Placemark>
<name>189</name>
<description>Further info on the population would go here</description>
<styleUrl>#msn_ylw-circle</styleUrl>
<Point>
<coordinates>-0.74099999999999,47.996,0</coordinates>
</Point>
</Placemark>
<Placemark>
<name>190</name>
<description>Further info on the population would go here</description>
<styleUrl>#msn_ylw-circle</styleUrl>
<Point>
<coordinates>-0.74099999999999,48.172,0</coordinates>
</Point>
</Placemark>
<Placemark>
<name>191</name>
<description>Further info on the population would go here</description>
<styleUrl>#msn_ylw-circle</styleUrl>
<Point>
<coordinates>-0.74000000000001,48.199,0</coordinates>
</Point>
</Placemark>
<Placemark>
<name>192</name>
<description>Further info on the population would go here</description>

<styleUrl>#msn_ylw-circle</styleUrl>
<Point>
<coordinates>-0.73900000000003,48.152,0</coordinates>
</Point>
</Placemark>
<Placemark>
<name>193</name>
<description>Further info on the population would go here</description>
<styleUrl>#msn_ylw-circle</styleUrl>
<Point>
<coordinates>-0.73900000000003,48.324,0</coordinates>
</Point>
</Placemark>
<Placemark>
<name>194</name>
<description>Further info on the population would go here</description>
<styleUrl>#msn_ylw-circle</styleUrl>
<Point>
<coordinates>-0.73500000000001,48.049,0</coordinates>
</Point>
</Placemark>
<Placemark>
<name>195</name>
<description>Further info on the population would go here</description>
<styleUrl>#msn_ylw-circle</styleUrl>
<Point>
<coordinates>-0.73400000000004,48.049,0</coordinates>
</Point>
</Placemark>
<Placemark>
<name>196</name>
<description>Further info on the population would go here</description>
<styleUrl>#msn_ylw-circle</styleUrl>
<Point>
<coordinates>-0.73400000000004,48.206,0</coordinates>
</Point>
</Placemark>
<Placemark>
<name>197</name>
<description>Further info on the population would go here</description>
<styleUrl>#msn_ylw-circle</styleUrl>
<Point>
<coordinates>-0.73400000000004,48.049,0</coordinates>
</Point>
</Placemark>
<Placemark>
<name>198</name>
<description>Further info on the population would go here</description>
<styleUrl>#msn_ylw-circle</styleUrl>
<Point>
<coordinates>-0.73099999999999,48.206,0</coordinates>
</Point>
</Placemark>
<Placemark>
<name>199</name>
<description>Further info on the population would go here</description>
<styleUrl>#msn_ylw-circle</styleUrl>

<Point>
<coordinates>-0.72800000000007,48.123,0</coordinates>
</Point>
</Placemark>
<Placemark>
<name>200</name>
<description>Further info on the population would go here</description>
<styleUrl>#msn_ylw-circle</styleUrl>
<Point>
<coordinates>-0.726,48.376,0</coordinates>
</Point>
</Placemark>
<Placemark>
<name>201</name>
<description>Further info on the population would go here</description>
<styleUrl>#msn_ylw-circle</styleUrl>
<Point>
<coordinates>-0.72500000000002,48,0</coordinates>
</Point>
</Placemark>
<Placemark>
<name>202</name>
<description>Further info on the population would go here</description>
<styleUrl>#msn_ylw-circle</styleUrl>
<Point>
<coordinates>-0.72299999999996,48.227,0</coordinates>
</Point>
</Placemark>
<Placemark>
<name>203</name>
<description>Further info on the population would go here</description>
<styleUrl>#msn_ylw-circle</styleUrl>
<Point>
<coordinates>-0.71900000000005,48.359,0</coordinates>
</Point>
</Placemark>
<Placemark>
<name>204</name>
<description>Further info on the population would go here</description>
<styleUrl>#msn_ylw-circle</styleUrl>
<Point>
<coordinates>-0.71799999999996,48.165,0</coordinates>
</Point>
</Placemark>
<Placemark>
<name>205</name>
<description>Further info on the population would go here</description>
<styleUrl>#msn_ylw-circle</styleUrl>
<Point>
<coordinates>-0.71799999999996,48.068,0</coordinates>
</Point>
</Placemark>
<Placemark>
<name>206</name>
<description>Further info on the population would go here</description>
<styleUrl>#msn_ylw-circle</styleUrl>
<Point>

<coordinates>-0.71600000000001,48.231,0</coordinates>
</Point>
</Placemark>
<Placemark>
<name>207</name>
<description>Further info on the population would go here</description>
<styleUrl>#msn_ylw-circle</styleUrl>
<Point>
<coordinates>-0.711999999999999,48.08,0</coordinates>
</Point>
</Placemark>
<Placemark>
<name>208</name>
<description>Further info on the population would go here</description>
<styleUrl>#msn_ylw-circle</styleUrl>
<Point>
<coordinates>-0.711999999999999,48.08,0</coordinates>
</Point>
</Placemark>
<Placemark>
<name>209</name>
<description>Further info on the population would go here</description>
<styleUrl>#msn_ylw-circle</styleUrl>
<Point>
<coordinates>-0.71100000000001,47.753,0</coordinates>
</Point>
</Placemark>
<Placemark>
<name>210</name>
<description>Further info on the population would go here</description>
<styleUrl>#msn_ylw-circle</styleUrl>
<Point>
<coordinates>-0.70900000000006,48.136,0</coordinates>
</Point>
</Placemark>
<Placemark>
<name>211</name>
<description>Further info on the population would go here</description>
<styleUrl>#msn_ylw-circle</styleUrl>
<Point>
<coordinates>-0.70900000000006,47.807,0</coordinates>
</Point>
</Placemark>
<Placemark>
<name>212</name>
<description>Further info on the population would go here</description>
<styleUrl>#msn_ylw-circle</styleUrl>
<Point>
<coordinates>-0.70900000000006,47.807,0</coordinates>
</Point>
</Placemark>
<Placemark>
<name>213</name>
<description>Further info on the population would go here</description>
<styleUrl>#msn_ylw-circle</styleUrl>
<Point>
<coordinates>-0.706999999999999,47.807,0</coordinates>

</Point>
</Placemark>
<Placemark>
<name>214</name>
<description>Further info on the population would go here</description>
<styleUrl>#msn_ylw-circle</styleUrl>
<Point>
<coordinates>-0.70699999999999,48.003,0</coordinates>
</Point>
</Placemark>
<Placemark>
<name>215</name>
<description>Further info on the population would go here</description>
<styleUrl>#msn_ylw-circle</styleUrl>
<Point>
<coordinates>-0.70699999999999,47.966,0</coordinates>
</Point>
</Placemark>
<Placemark>
<name>216</name>
<description>Further info on the population would go here</description>
<styleUrl>#msn_ylw-circle</styleUrl>
<Point>
<coordinates>-0.70600000000002,48.258,0</coordinates>
</Point>
</Placemark>
<Placemark>
<name>217</name>
<description>Further info on the population would go here</description>
<styleUrl>#msn_ylw-circle</styleUrl>
<Point>
<coordinates>-0.70500000000004,48.234,0</coordinates>
</Point>
</Placemark>
<Placemark>
<name>218</name>
<description>Further info on the population would go here</description>
<styleUrl>#msn_ylw-circle</styleUrl>
<Point>
<coordinates>-0.70399999999995,48.009,0</coordinates>
</Point>
</Placemark>
<Placemark>
<name>219</name>
<description>Further info on the population would go here</description>
<styleUrl>#msn_ylw-circle</styleUrl>
<Point>
<coordinates>-0.702,48.154,0</coordinates>
</Point>
</Placemark>
<Placemark>
<name>220</name>
<description>Further info on the population would go here</description>
<styleUrl>#msn_ylw-circle</styleUrl>
<Point>
<coordinates>-0.70000000000005,47.973,0</coordinates>
</Point>

</Placemark>
<Placemark>
<name>221</name>
<description>Further info on the population would go here</description>
<styleUrl>#msn_ylw-circle</styleUrl>
<Point>
<coordinates>-0.70000000000005,48.373,0</coordinates>
</Point>
</Placemark>
<Placemark>
<name>222</name>
<description>Further info on the population would go here</description>
<styleUrl>#msn_ylw-circle</styleUrl>
<Point>
<coordinates>-0.70000000000005,48.261,0</coordinates>
</Point>
</Placemark>
<Placemark>
<name>223</name>
<description>Further info on the population would go here</description>
<styleUrl>#msn_ylw-circle</styleUrl>
<Point>
<coordinates>-0.69799999999998,48.383,0</coordinates>
</Point>
</Placemark>
<Placemark>
<name>224</name>
<description>Further info on the population would go here</description>
<styleUrl>#msn_ylw-circle</styleUrl>
<Point>
<coordinates>-0.697,48.152,0</coordinates>
</Point>
</Placemark>
<Placemark>
<name>225</name>
<description>Further info on the population would go here</description>
<styleUrl>#msn_ylw-circle</styleUrl>
<Point>
<coordinates>-0.69499999999994,48.261,0</coordinates>
</Point>
</Placemark>
<Placemark>
<name>226</name>
<description>Further info on the population would go here</description>
<styleUrl>#msn_ylw-circle</styleUrl>
<Point>
<coordinates>-0.69399999999996,48.373,0</coordinates>
</Point>
</Placemark>
<Placemark>
<name>227</name>
<description>Further info on the population would go here</description>
<styleUrl>#msn_ylw-circle</styleUrl>
<Point>
<coordinates>-0.69200000000001,48.179,0</coordinates>
</Point>
</Placemark>

<Placemark>
<name>228</name>
<description>Further info on the population would go here</description>
<styleUrl>#msn_ylw-circle</styleUrl>
<Point>
<coordinates>-0.68799999999999,48.152,0</coordinates>
</Point>
</Placemark>
<Placemark>
<name>229</name>
<description>Further info on the population would go here</description>
<styleUrl>#msn_ylw-circle</styleUrl>
<Point>
<coordinates>-0.68799999999999,48.356,0</coordinates>
</Point>
</Placemark>
<Placemark>
<name>230</name>
<description>Further info on the population would go here</description>
<styleUrl>#msn_ylw-circle</styleUrl>
<Point>
<coordinates>-0.68700000000001,48.177,0</coordinates>
</Point>
</Placemark>
<Placemark>
<name>231</name>
<description>Further info on the population would go here</description>
<styleUrl>#msn_ylw-circle</styleUrl>
<Point>
<coordinates>-0.68499999999995,48.176,0</coordinates>
</Point>
</Placemark>
<Placemark>
<name>232</name>
<description>Further info on the population would go here</description>
<styleUrl>#msn_ylw-circle</styleUrl>
<Point>
<coordinates>-0.68299999999999,47.822,0</coordinates>
</Point>
</Placemark>
<Placemark>
<name>233</name>
<description>Further info on the population would go here</description>
<styleUrl>#msn_ylw-circle</styleUrl>
<Point>
<coordinates>-0.68200000000002,47.823,0</coordinates>
</Point>
</Placemark>
<Placemark>
<name>234</name>
<description>Further info on the population would go here</description>
<styleUrl>#msn_ylw-circle</styleUrl>
<Point>
<coordinates>-0.68200000000002,48.35,0</coordinates>
</Point>
</Placemark>
<Placemark>

<name>235</name>
<description>Further info on the population would go here</description>
<styleUrl>#msn_ylw-circle</styleUrl>
<Point>
<coordinates>-0.678999999999997,48.175,0</coordinates>
</Point>
</Placemark>
<Placemark>
<name>236</name>
<description>Further info on the population would go here</description>
<styleUrl>#msn_ylw-circle</styleUrl>
<Point>
<coordinates>-0.678999999999997,48.202,0</coordinates>
</Point>
</Placemark>
<Placemark>
<name>237</name>
<description>Further info on the population would go here</description>
<styleUrl>#msn_ylw-circle</styleUrl>
<Point>
<coordinates>-0.677000000000002,48.032,0</coordinates>
</Point>
</Placemark>
<Placemark>
<name>238</name>
<description>Further info on the population would go here</description>
<styleUrl>#msn_ylw-circle</styleUrl>
<Point>
<coordinates>-0.675999999999993,48.201,0</coordinates>
</Point>
</Placemark>
<Placemark>
<name>239</name>
<description>Further info on the population would go here</description>
<styleUrl>#msn_ylw-circle</styleUrl>
<Point>
<coordinates>-0.673999999999998,48.033,0</coordinates>
</Point>
</Placemark>
<Placemark>
<name>240</name>
<description>Further info on the population would go here</description>
<styleUrl>#msn_ylw-circle</styleUrl>
<Point>
<coordinates>-0.673,48.203,0</coordinates>
</Point>
</Placemark>
<Placemark>
<name>241</name>
<description>Further info on the population would go here</description>
<styleUrl>#msn_ylw-circle</styleUrl>
<Point>
<coordinates>-0.672000000000003,48.139,0</coordinates>
</Point>
</Placemark>
<Placemark>
<name>242</name>

<description>Further info on the population would go here</description>
<styleUrl>#msn_ylw-circle</styleUrl>
<Point>
<coordinates>-0.66999999999996,48.199,0</coordinates>
</Point>
</Placemark>
<Placemark>
<name>243</name>
<description>Further info on the population would go here</description>
<styleUrl>#msn_ylw-circle</styleUrl>
<Point>
<coordinates>-0.66700000000003,48.486,0</coordinates>
</Point>
</Placemark>
<Placemark>
<name>244</name>
<description>Further info on the population would go here</description>
<styleUrl>#msn_ylw-circle</styleUrl>
<Point>
<coordinates>-0.66399999999999,48.419,0</coordinates>
</Point>
</Placemark>
<Placemark>
<name>245</name>
<description>Further info on the population would go here</description>
<styleUrl>#msn_ylw-circle</styleUrl>
<Point>
<coordinates>-0.66200000000003,48.116,0</coordinates>
</Point>
</Placemark>
<Placemark>
<name>246</name>
<description>Further info on the population would go here</description>
<styleUrl>#msn_ylw-circle</styleUrl>
<Point>
<coordinates>-0.65999999999997,48.276,0</coordinates>
</Point>
</Placemark>
<Placemark>
<name>247</name>
<description>Further info on the population would go here</description>
<styleUrl>#msn_ylw-circle</styleUrl>
<Point>
<coordinates>-0.65599999999995,48.283,0</coordinates>
</Point>
</Placemark>
<Placemark>
<name>248</name>
<description>Further info on the population would go here</description>
<styleUrl>#msn_ylw-circle</styleUrl>
<Point>
<coordinates>-0.65599999999995,48.155,0</coordinates>
</Point>
</Placemark>
<Placemark>
<name>249</name>
<description>Further info on the population would go here</description>

<styleUrl>#msn_ylw-circle</styleUrl>
<Point>
<coordinates>-0.654,48.167,0</coordinates>
</Point>
</Placemark>
<Placemark>
<name>250</name>
<description>Further info on the population would go here</description>
<styleUrl>#msn_ylw-circle</styleUrl>
<Point>
<coordinates>-0.65200000000004,48.334,0</coordinates>
</Point>
</Placemark>
<Placemark>
<name>251</name>
<description>Further info on the population would go here</description>
<styleUrl>#msn_ylw-circle</styleUrl>
<Point>
<coordinates>-0.65200000000004,48.269,0</coordinates>
</Point>
</Placemark>
<Placemark>
<name>252</name>
<description>Further info on the population would go here</description>
<styleUrl>#msn_ylw-circle</styleUrl>
<Point>
<coordinates>-0.65100000000007,48.391,0</coordinates>
</Point>
</Placemark>
<Placemark>
<name>253</name>
<description>Further info on the population would go here</description>
<styleUrl>#msn_ylw-circle</styleUrl>
<Point>
<coordinates>-0.65100000000007,48.389,0</coordinates>
</Point>
</Placemark>
<Placemark>
<name>254</name>
<description>Further info on the population would go here</description>
<styleUrl>#msn_ylw-circle</styleUrl>
<Point>
<coordinates>-0.64999999999998,47.963,0</coordinates>
</Point>
</Placemark>
<Placemark>
<name>255</name>
<description>Further info on the population would go here</description>
<styleUrl>#msn_ylw-circle</styleUrl>
<Point>
<coordinates>-0.649,48.191,0</coordinates>
</Point>
</Placemark>
<Placemark>
<name>256</name>
<description>Further info on the population would go here</description>
<styleUrl>#msn_ylw-circle</styleUrl>

<Point>
<coordinates>-0.64099999999996,48.192,0</coordinates>
</Point>
</Placemark>
<Placemark>
<name>257</name>
<description>Further info on the population would go here</description>
<styleUrl>#msn_ylw-circle</styleUrl>
<Point>
<coordinates>-0.63699999999994,48.189,0</coordinates>
</Point>
</Placemark>
<Placemark>
<name>258</name>
<description>Further info on the population would go here</description>
<styleUrl>#msn_ylw-circle</styleUrl>
<Point>
<coordinates>-0.63599999999997,48.088,0</coordinates>
</Point>
</Placemark>
<Placemark>
<name>259</name>
<description>Further info on the population would go here</description>
<styleUrl>#msn_ylw-circle</styleUrl>
<Point>
<coordinates>-0.63499999999999,48.028,0</coordinates>
</Point>
</Placemark>
<Placemark>
<name>260</name>
<description>Further info on the population would go here</description>
<styleUrl>#msn_ylw-circle</styleUrl>
<Point>
<coordinates>-0.63499999999999,48.088,0</coordinates>
</Point>
</Placemark>
<Placemark>
<name>261</name>
<description>Further info on the population would go here</description>
<styleUrl>#msn_ylw-circle</styleUrl>
<Point>
<coordinates>-0.63499999999999,48.089,0</coordinates>
</Point>
</Placemark>
<Placemark>
<name>262</name>
<description>Further info on the population would go here</description>
<styleUrl>#msn_ylw-circle</styleUrl>
<Point>
<coordinates>-0.63499999999999,48.059,0</coordinates>
</Point>
</Placemark>
<Placemark>
<name>263</name>
<description>Further info on the population would go here</description>
<styleUrl>#msn_ylw-circle</styleUrl>
<Point>

<coordinates>-0.63200000000006,48.053,0</coordinates>
</Point>
</Placemark>
<Placemark>
<name>264</name>
<description>Further info on the population would go here</description>
<styleUrl>#msn_ylw-circle</styleUrl>
<Point>
<coordinates>-0.63099999999997,48.027,0</coordinates>
</Point>
</Placemark>
<Placemark>
<name>265</name>
<description>Further info on the population would go here</description>
<styleUrl>#msn_ylw-circle</styleUrl>
<Point>
<coordinates>-0.625,48.067,0</coordinates>
</Point>
</Placemark>
<Placemark>
<name>266</name>
<description>Further info on the population would go here</description>
<styleUrl>#msn_ylw-circle</styleUrl>
<Point>
<coordinates>-0.62099999999998,48.103,0</coordinates>
</Point>
</Placemark>
<Placemark>
<name>267</name>
<description>Further info on the population would go here</description>
<styleUrl>#msn_ylw-circle</styleUrl>
<Point>
<coordinates>-0.62,48.056,0</coordinates>
</Point>
</Placemark>
<Placemark>
<name>268</name>
<description>Further info on the population would go here</description>
<styleUrl>#msn_ylw-circle</styleUrl>
<Point>
<coordinates>-0.61699999999996,48.018,0</coordinates>
</Point>
</Placemark>
<Placemark>
<name>269</name>
<description>Further info on the population would go here</description>
<styleUrl>#msn_ylw-circle</styleUrl>
<Point>
<coordinates>-0.61500000000001,48.017,0</coordinates>
</Point>
</Placemark>
<Placemark>
<name>270</name>
<description>Further info on the population would go here</description>
<styleUrl>#msn_ylw-circle</styleUrl>
<Point>
<coordinates>-0.61500000000001,48.228,0</coordinates>

</Point>
</Placemark>
<Placemark>
<name>271</name>
<description>Further info on the population would go here</description>
<styleUrl>#msn_ylw-circle</styleUrl>
<Point>
<coordinates>-0.61500000000001,48.32,0</coordinates>
</Point>
</Placemark>
<Placemark>
<name>272</name>
<description>Further info on the population would go here</description>
<styleUrl>#msn_ylw-circle</styleUrl>
<Point>
<coordinates>-0.61300000000006,48.225,0</coordinates>
</Point>
</Placemark>
<Placemark>
<name>273</name>
<description>Further info on the population would go here</description>
<styleUrl>#msn_ylw-circle</styleUrl>
<Point>
<coordinates>-0.60900000000004,47.834,0</coordinates>
</Point>
</Placemark>
<Placemark>
<name>274</name>
<description>Further info on the population would go here</description>
<styleUrl>#msn_ylw-circle</styleUrl>
<Point>
<coordinates>-0.60599999999999,48.26,0</coordinates>
</Point>
</Placemark>
<Placemark>
<name>275</name>
<description>Further info on the population would go here</description>
<styleUrl>#msn_ylw-circle</styleUrl>
<Point>
<coordinates>-0.60500000000002,48.313,0</coordinates>
</Point>
</Placemark>
<Placemark>
<name>276</name>
<description>Further info on the population would go here</description>
<styleUrl>#msn_ylw-circle</styleUrl>
<Point>
<coordinates>-0.60500000000002,48.04,0</coordinates>
</Point>
</Placemark>
<Placemark>
<name>277</name>
<description>Further info on the population would go here</description>
<styleUrl>#msn_ylw-circle</styleUrl>
<Point>
<coordinates>-0.60300000000007,48.243,0</coordinates>
</Point>

</Placemark>
<Placemark>
<name>278</name>
<description>Further info on the population would go here</description>
<styleUrl>#msn_ylw-circle</styleUrl>
<Point>
<coordinates>-0.60300000000007,48.243,0</coordinates>
</Point>
</Placemark>
<Placemark>
<name>279</name>
<description>Further info on the population would go here</description>
<styleUrl>#msn_ylw-circle</styleUrl>
<Point>
<coordinates>-0.60199999999998,48.253,0</coordinates>
</Point>
</Placemark>
<Placemark>
<name>280</name>
<description>Further info on the population would go here</description>
<styleUrl>#msn_ylw-circle</styleUrl>
<Point>
<coordinates>-0.60199999999998,48.106,0</coordinates>
</Point>
</Placemark>
<Placemark>
<name>281</name>
<description>Further info on the population would go here</description>
<styleUrl>#msn_ylw-circle</styleUrl>
<Point>
<coordinates>-0.601,48.438,0</coordinates>
</Point>
</Placemark>
<Placemark>
<name>282</name>
<description>Further info on the population would go here</description>
<styleUrl>#msn_ylw-circle</styleUrl>
<Point>
<coordinates>-0.601,48.429,0</coordinates>
</Point>
</Placemark>
<Placemark>
<name>283</name>
<description>Further info on the population would go here</description>
<styleUrl>#msn_ylw-circle</styleUrl>
<Point>
<coordinates>-0.59899999999993,48.277,0</coordinates>
</Point>
</Placemark>
<Placemark>
<name>284</name>
<description>Further info on the population would go here</description>
<styleUrl>#msn_ylw-circle</styleUrl>
<Point>
<coordinates>-0.59899999999993,48.255,0</coordinates>
</Point>
</Placemark>

<Placemark>
<name>285</name>
<description>Further info on the population would go here</description>
<styleUrl>#msn_ylw-circle</styleUrl>
<Point>
<coordinates>-0.597999999999996,48.278,0</coordinates>
</Point>
</Placemark>
<Placemark>
<name>286</name>
<description>Further info on the population would go here</description>
<styleUrl>#msn_ylw-circle</styleUrl>
<Point>
<coordinates>-0.597999999999996,48.456,0</coordinates>
</Point>
</Placemark>
<Placemark>
<name>287</name>
<description>Further info on the population would go here</description>
<styleUrl>#msn_ylw-circle</styleUrl>
<Point>
<coordinates>-0.596999999999998,48.205,0</coordinates>
</Point>
</Placemark>
<Placemark>
<name>288</name>
<description>Further info on the population would go here</description>
<styleUrl>#msn_ylw-circle</styleUrl>
<Point>
<coordinates>-0.596999999999998,48.193,0</coordinates>
</Point>
</Placemark>
<Placemark>
<name>289</name>
<description>Further info on the population would go here</description>
<styleUrl>#msn_ylw-circle</styleUrl>
<Point>
<coordinates>-0.596,47.802,0</coordinates>
</Point>
</Placemark>
<Placemark>
<name>290</name>
<description>Further info on the population would go here</description>
<styleUrl>#msn_ylw-circle</styleUrl>
<Point>
<coordinates>-0.596,47.802,0</coordinates>
</Point>
</Placemark>
<Placemark>
<name>291</name>
<description>Further info on the population would go here</description>
<styleUrl>#msn_ylw-circle</styleUrl>
<Point>
<coordinates>-0.596,48.193,0</coordinates>
</Point>
</Placemark>
<Placemark>

<name>292</name>
<description>Further info on the population would go here</description>
<styleUrl>#msn_ylw-circle</styleUrl>
<Point>
<coordinates>-0.59500000000003,48.27,0</coordinates>
</Point>
</Placemark>
<Placemark>
<name>293</name>
<description>Further info on the population would go here</description>
<styleUrl>#msn_ylw-circle</styleUrl>
<Point>
<coordinates>-0.59299999999996,48.267,0</coordinates>
</Point>
</Placemark>
<Placemark>
<name>294</name>
<description>Further info on the population would go here</description>
<styleUrl>#msn_ylw-circle</styleUrl>
<Point>
<coordinates>-0.58899999999994,48.311,0</coordinates>
</Point>
</Placemark>
<Placemark>
<name>295</name>
<description>Further info on the population would go here</description>
<styleUrl>#msn_ylw-circle</styleUrl>
<Point>
<coordinates>-0.58799999999997,48.46,0</coordinates>
</Point>
</Placemark>
<Placemark>
<name>296</name>
<description>Further info on the population would go here</description>
<styleUrl>#msn_ylw-circle</styleUrl>
<Point>
<coordinates>-0.58600000000001,48.272,0</coordinates>
</Point>
</Placemark>
<Placemark>
<name>297</name>
<description>Further info on the population would go here</description>
<styleUrl>#msn_ylw-circle</styleUrl>
<Point>
<coordinates>-0.58600000000001,48.23,0</coordinates>
</Point>
</Placemark>
<Placemark>
<name>298</name>
<description>Further info on the population would go here</description>
<styleUrl>#msn_ylw-circle</styleUrl>
<Point>
<coordinates>-0.58600000000001,48.236,0</coordinates>
</Point>
</Placemark>
<Placemark>
<name>299</name>

<description>Further info on the population would go here</description>
<styleUrl>#msn_ylw-circle</styleUrl>
<Point>
<coordinates>-0.58500000000004,48.12,0</coordinates>
</Point>
</Placemark>
<Placemark>
<name>300</name>
<description>Further info on the population would go here</description>
<styleUrl>#msn_ylw-circle</styleUrl>
<Point>
<coordinates>-0.58500000000004,47.886,0</coordinates>
</Point>
</Placemark>
<Placemark>
<name>301</name>
<description>Further info on the population would go here</description>
<styleUrl>#msn_ylw-circle</styleUrl>
<Point>
<coordinates>-0.58500000000004,48.223,0</coordinates>
</Point>
</Placemark>
<Placemark>
<name>302</name>
<description>Further info on the population would go here</description>
<styleUrl>#msn_ylw-circle</styleUrl>
<Point>
<coordinates>-0.58500000000004,48.215,0</coordinates>
</Point>
</Placemark>
<Placemark>
<name>303</name>
<description>Further info on the population would go here</description>
<styleUrl>#msn_ylw-circle</styleUrl>
<Point>
<coordinates>-0.58500000000004,48.223,0</coordinates>
</Point>
</Placemark>
<Placemark>
<name>304</name>
<description>Further info on the population would go here</description>
<styleUrl>#msn_ylw-circle</styleUrl>
<Point>
<coordinates>-0.58400000000006,48.214,0</coordinates>
</Point>
</Placemark>
<Placemark>
<name>305</name>
<description>Further info on the population would go here</description>
<styleUrl>#msn_ylw-circle</styleUrl>
<Point>
<coordinates>-0.58299999999997,48.384,0</coordinates>
</Point>
</Placemark>
<Placemark>
<name>306</name>
<description>Further info on the population would go here</description>

<styleUrl>#msn_ylw-circle</styleUrl>
<Point>
<coordinates>-0.582999999999997,48.208,0</coordinates>
</Point>
</Placemark>
<Placemark>
<name>307</name>
<description>Further info on the population would go here</description>
<styleUrl>#msn_ylw-circle</styleUrl>
<Point>
<coordinates>-0.581999999999999,48.248,0</coordinates>
</Point>
</Placemark>
<Placemark>
<name>308</name>
<description>Further info on the population would go here</description>
<styleUrl>#msn_ylw-circle</styleUrl>
<Point>
<coordinates>-0.581999999999999,48.209,0</coordinates>
</Point>
</Placemark>
<Placemark>
<name>309</name>
<description>Further info on the population would go here</description>
<styleUrl>#msn_ylw-circle</styleUrl>
<Point>
<coordinates>-0.581999999999999,48.381,0</coordinates>
</Point>
</Placemark>
<Placemark>
<name>310</name>
<description>Further info on the population would go here</description>
<styleUrl>#msn_ylw-circle</styleUrl>
<Point>
<coordinates>-0.581999999999999,48.209,0</coordinates>
</Point>
</Placemark>
<Placemark>
<name>311</name>
<description>Further info on the population would go here</description>
<styleUrl>#msn_ylw-circle</styleUrl>
<Point>
<coordinates>-0.58100000000002,48.12,0</coordinates>
</Point>
</Placemark>
<Placemark>
<name>312</name>
<description>Further info on the population would go here</description>
<styleUrl>#msn_ylw-circle</styleUrl>
<Point>
<coordinates>-0.58000000000004,48.39,0</coordinates>
</Point>
</Placemark>
<Placemark>
<name>313</name>
<description>Further info on the population would go here</description>
<styleUrl>#msn_ylw-circle</styleUrl>

<Point>
<coordinates>-0.58000000000004,48.183,0</coordinates>
</Point>
</Placemark>
<Placemark>
<name>314</name>
<description>Further info on the population would go here</description>
<styleUrl>#msn_ylw-circle</styleUrl>
<Point>
<coordinates>-0.58000000000004,48.247,0</coordinates>
</Point>
</Placemark>
<Placemark>
<name>315</name>
<description>Further info on the population would go here</description>
<styleUrl>#msn_ylw-circle</styleUrl>
<Point>
<coordinates>-0.57899999999995,48.45,0</coordinates>
</Point>
</Placemark>
<Placemark>
<name>316</name>
<description>Further info on the population would go here</description>
<styleUrl>#msn_ylw-circle</styleUrl>
<Point>
<coordinates>-0.57799999999997,48.247,0</coordinates>
</Point>
</Placemark>
<Placemark>
<name>317</name>
<description>Further info on the population would go here</description>
<styleUrl>#msn_ylw-circle</styleUrl>
<Point>
<coordinates>-0.57600000000002,47.89,0</coordinates>
</Point>
</Placemark>
<Placemark>
<name>318</name>
<description>Further info on the population would go here</description>
<styleUrl>#msn_ylw-circle</styleUrl>
<Point>
<coordinates>-0.57500000000005,48.442,0</coordinates>
</Point>
</Placemark>
<Placemark>
<name>319</name>
<description>Further info on the population would go here</description>
<styleUrl>#msn_ylw-circle</styleUrl>
<Point>
<coordinates>-0.57500000000005,48.014,0</coordinates>
</Point>
</Placemark>
<Placemark>
<name>320</name>
<description>Further info on the population would go here</description>
<styleUrl>#msn_ylw-circle</styleUrl>
<Point>

<coordinates>-0.57500000000005,48.013,0</coordinates>
</Point>
</Placemark>
<Placemark>
<name>321</name>
<description>Further info on the population would go here</description>
<styleUrl>#msn_ylw-circle</styleUrl>
<Point>
<coordinates>-0.57400000000007,48.44,0</coordinates>
</Point>
</Placemark>
<Placemark>
<name>322</name>
<description>Further info on the population would go here</description>
<styleUrl>#msn_ylw-circle</styleUrl>
<Point>
<coordinates>-0.57400000000007,48.259,0</coordinates>
</Point>
</Placemark>
<Placemark>
<name>323</name>
<description>Further info on the population would go here</description>
<styleUrl>#msn_ylw-circle</styleUrl>
<Point>
<coordinates>-0.57299999999998,48.262,0</coordinates>
</Point>
</Placemark>
<Placemark>
<name>324</name>
<description>Further info on the population would go here</description>
<styleUrl>#msn_ylw-circle</styleUrl>
<Point>
<coordinates>-0.572,48.446,0</coordinates>
</Point>
</Placemark>
<Placemark>
<name>325</name>
<description>Further info on the population would go here</description>
<styleUrl>#msn_ylw-circle</styleUrl>
<Point>
<coordinates>-0.572,48.452,0</coordinates>
</Point>
</Placemark>
<Placemark>
<name>326</name>
<description>Further info on the population would go here</description>
<styleUrl>#msn_ylw-circle</styleUrl>
<Point>
<coordinates>-0.57100000000003,47.952,0</coordinates>
</Point>
</Placemark>
<Placemark>
<name>327</name>
<description>Further info on the population would go here</description>
<styleUrl>#msn_ylw-circle</styleUrl>
<Point>
<coordinates>-0.56999999999994,48.42,0</coordinates>

</Point>
</Placemark>
<Placemark>
<name>328</name>
<description>Further info on the population would go here</description>
<styleUrl>#msn_ylw-circle</styleUrl>
<Point>
<coordinates>-0.567999999999998,48.186,0</coordinates>
</Point>
</Placemark>
<Placemark>
<name>329</name>
<description>Further info on the population would go here</description>
<styleUrl>#msn_ylw-circle</styleUrl>
<Point>
<coordinates>-0.567999999999998,48.278,0</coordinates>
</Point>
</Placemark>
<Placemark>
<name>330</name>
<description>Further info on the population would go here</description>
<styleUrl>#msn_ylw-circle</styleUrl>
<Point>
<coordinates>-0.567999999999998,48.187,0</coordinates>
</Point>
</Placemark>
<Placemark>
<name>331</name>
<description>Further info on the population would go here</description>
<styleUrl>#msn_ylw-circle</styleUrl>
<Point>
<coordinates>-0.567000000000001,48.186,0</coordinates>
</Point>
</Placemark>
<Placemark>
<name>332</name>
<description>Further info on the population would go here</description>
<styleUrl>#msn_ylw-circle</styleUrl>
<Point>
<coordinates>-0.567000000000001,48.2,0</coordinates>
</Point>
</Placemark>
<Placemark>
<name>333</name>
<description>Further info on the population would go here</description>
<styleUrl>#msn_ylw-circle</styleUrl>
<Point>
<coordinates>-0.562999999999999,48.228,0</coordinates>
</Point>
</Placemark>
<Placemark>
<name>334</name>
<description>Further info on the population would go here</description>
<styleUrl>#msn_ylw-circle</styleUrl>
<Point>
<coordinates>-0.562000000000001,48.2,0</coordinates>
</Point>

</Placemark>
<Placemark>
<name>335</name>
<description>Further info on the population would go here</description>
<styleUrl>#msn_ylw-circle</styleUrl>
<Point>
<coordinates>-0.56200000000001,48.2,0</coordinates>
</Point>
</Placemark>
<Placemark>
<name>336</name>
<description>Further info on the population would go here</description>
<styleUrl>#msn_ylw-circle</styleUrl>
<Point>
<coordinates>-0.56100000000004,48.2,0</coordinates>
</Point>
</Placemark>
<Placemark>
<name>337</name>
<description>Further info on the population would go here</description>
<styleUrl>#msn_ylw-circle</styleUrl>
<Point>
<coordinates>-0.56100000000004,48.418,0</coordinates>
</Point>
</Placemark>
<Placemark>
<name>338</name>
<description>Further info on the population would go here</description>
<styleUrl>#msn_ylw-circle</styleUrl>
<Point>
<coordinates>-0.55899999999997,48.222,0</coordinates>
</Point>
</Placemark>
<Placemark>
<name>339</name>
<description>Further info on the population would go here</description>
<styleUrl>#msn_ylw-circle</styleUrl>
<Point>
<coordinates>-0.55500000000006,48.243,0</coordinates>
</Point>
</Placemark>
<Placemark>
<name>340</name>
<description>Further info on the population would go here</description>
<styleUrl>#msn_ylw-circle</styleUrl>
<Point>
<coordinates>-0.55500000000006,47.998,0</coordinates>
</Point>
</Placemark>
<Placemark>
<name>341</name>
<description>Further info on the population would go here</description>
<styleUrl>#msn_ylw-circle</styleUrl>
<Point>
<coordinates>-0.55500000000006,48.25,0</coordinates>
</Point>
</Placemark>

<Placemark>
<name>342</name>
<description>Further info on the population would go here</description>
<styleUrl>#msn_ylw-circle</styleUrl>
<Point>
<coordinates>-0.55200000000002,48.082,0</coordinates>
</Point>
</Placemark>
<Placemark>
<name>343</name>
<description>Further info on the population would go here</description>
<styleUrl>#msn_ylw-circle</styleUrl>
<Point>
<coordinates>-0.55200000000002,48.207,0</coordinates>
</Point>
</Placemark>
<Placemark>
<name>344</name>
<description>Further info on the population would go here</description>
<styleUrl>#msn_ylw-circle</styleUrl>
<Point>
<coordinates>-0.55099999999993,48.239,0</coordinates>
</Point>
</Placemark>
<Placemark>
<name>345</name>
<description>Further info on the population would go here</description>
<styleUrl>#msn_ylw-circle</styleUrl>
<Point>
<coordinates>-0.55099999999993,48.239,0</coordinates>
</Point>
</Placemark>
<Placemark>
<name>346</name>
<description>Further info on the population would go here</description>
<styleUrl>#msn_ylw-circle</styleUrl>
<Point>
<coordinates>-0.55099999999993,48.366,0</coordinates>
</Point>
</Placemark>
<Placemark>
<name>347</name>
<description>Further info on the population would go here</description>
<styleUrl>#msn_ylw-circle</styleUrl>
<Point>
<coordinates>-0.55099999999993,48.293,0</coordinates>
</Point>
</Placemark>
<Placemark>
<name>348</name>
<description>Further info on the population would go here</description>
<styleUrl>#msn_ylw-circle</styleUrl>
<Point>
<coordinates>-0.54999999999995,47.939,0</coordinates>
</Point>
</Placemark>
<Placemark>

<name>349</name>
<description>Further info on the population would go here</description>
<styleUrl>#msn_ylw-circle</styleUrl>
<Point>
<coordinates>-0.548,48.27,0</coordinates>
</Point>
</Placemark>
<Placemark>
<name>350</name>
<description>Further info on the population would go here</description>
<styleUrl>#msn_ylw-circle</styleUrl>
<Point>
<coordinates>-0.54600000000005,48.278,0</coordinates>
</Point>
</Placemark>
<Placemark>
<name>351</name>
<description>Further info on the population would go here</description>
<styleUrl>#msn_ylw-circle</styleUrl>
<Point>
<coordinates>-0.54499999999996,48.169,0</coordinates>
</Point>
</Placemark>
<Placemark>
<name>352</name>
<description>Further info on the population would go here</description>
<styleUrl>#msn_ylw-circle</styleUrl>
<Point>
<coordinates>-0.54499999999996,48.205,0</coordinates>
</Point>
</Placemark>
<Placemark>
<name>353</name>
<description>Further info on the population would go here</description>
<styleUrl>#msn_ylw-circle</styleUrl>
<Point>
<coordinates>-0.54499999999996,48.351,0</coordinates>
</Point>
</Placemark>
<Placemark>
<name>354</name>
<description>Further info on the population would go here</description>
<styleUrl>#msn_ylw-circle</styleUrl>
<Point>
<coordinates>-0.54200000000003,47.817,0</coordinates>
</Point>
</Placemark>
<Placemark>
<name>355</name>
<description>Further info on the population would go here</description>
<styleUrl>#msn_ylw-circle</styleUrl>
<Point>
<coordinates>-0.54099999999994,48.171,0</coordinates>
</Point>
</Placemark>
<Placemark>
<name>356</name>

<description>Further info on the population would go here</description>
<styleUrl>#msn_ylw-circle</styleUrl>
<Point>
<coordinates>-0.54099999999994,48.24,0</coordinates>
</Point>
</Placemark>
<Placemark>
<name>357</name>
<description>Further info on the population would go here</description>
<styleUrl>#msn_ylw-circle</styleUrl>
<Point>
<coordinates>-0.53999999999996,48.172,0</coordinates>
</Point>
</Placemark>
<Placemark>
<name>358</name>
<description>Further info on the population would go here</description>
<styleUrl>#msn_ylw-circle</styleUrl>
<Point>
<coordinates>-0.53999999999996,48.118,0</coordinates>
</Point>
</Placemark>
<Placemark>
<name>359</name>
<description>Further info on the population would go here</description>
<styleUrl>#msn_ylw-circle</styleUrl>
<Point>
<coordinates>-0.53899999999999,48.353,0</coordinates>
</Point>
</Placemark>
<Placemark>
<name>360</name>
<description>Further info on the population would go here</description>
<styleUrl>#msn_ylw-circle</styleUrl>
<Point>
<coordinates>-0.53899999999999,48.176,0</coordinates>
</Point>
</Placemark>
<Placemark>
<name>361</name>
<description>Further info on the population would go here</description>
<styleUrl>#msn_ylw-circle</styleUrl>
<Point>
<coordinates>-0.53800000000001,48.118,0</coordinates>
</Point>
</Placemark>
<Placemark>
<name>362</name>
<description>Further info on the population would go here</description>
<styleUrl>#msn_ylw-circle</styleUrl>
<Point>
<coordinates>-0.53800000000001,48.031,0</coordinates>
</Point>
</Placemark>
<Placemark>
<name>363</name>
<description>Further info on the population would go here</description>

<styleUrl>#msn_ylw-circle</styleUrl>
<Point>
<coordinates>-0.53800000000001,48.117,0</coordinates>
</Point>
</Placemark>
<Placemark>
<name>364</name>
<description>Further info on the population would go here</description>
<styleUrl>#msn_ylw-circle</styleUrl>
<Point>
<coordinates>-0.53700000000003,48.166,0</coordinates>
</Point>
</Placemark>
<Placemark>
<name>365</name>
<description>Further info on the population would go here</description>
<styleUrl>#msn_ylw-circle</styleUrl>
<Point>
<coordinates>-0.53700000000003,48.245,0</coordinates>
</Point>
</Placemark>
<Placemark>
<name>366</name>
<description>Further info on the population would go here</description>
<styleUrl>#msn_ylw-circle</styleUrl>
<Point>
<coordinates>-0.53600000000006,48.448,0</coordinates>
</Point>
</Placemark>
<Placemark>
<name>367</name>
<description>Further info on the population would go here</description>
<styleUrl>#msn_ylw-circle</styleUrl>
<Point>
<coordinates>-0.53399999999999,48.243,0</coordinates>
</Point>
</Placemark>
<Placemark>
<name>368</name>
<description>Further info on the population would go here</description>
<styleUrl>#msn_ylw-circle</styleUrl>
<Point>
<coordinates>-0.53399999999999,48.269,0</coordinates>
</Point>
</Placemark>
<Placemark>
<name>369</name>
<description>Further info on the population would go here</description>
<styleUrl>#msn_ylw-circle</styleUrl>
<Point>
<coordinates>-0.53399999999999,48.221,0</coordinates>
</Point>
</Placemark>
<Placemark>
<name>370</name>
<description>Further info on the population would go here</description>
<styleUrl>#msn_ylw-circle</styleUrl>

<Point>
<coordinates>-0.53399999999999,48.246,0</coordinates>
</Point>
</Placemark>
<Placemark>
<name>371</name>
<description>Further info on the population would go here</description>
<styleUrl>#msn_ylw-circle</styleUrl>
<Point>
<coordinates>-0.53300000000002,48.26,0</coordinates>
</Point>
</Placemark>
<Placemark>
<name>372</name>
<description>Further info on the population would go here</description>
<styleUrl>#msn_ylw-circle</styleUrl>
<Point>
<coordinates>-0.53300000000002,48.159,0</coordinates>
</Point>
</Placemark>
<Placemark>
<name>373</name>
<description>Further info on the population would go here</description>
<styleUrl>#msn_ylw-circle</styleUrl>
<Point>
<coordinates>-0.53200000000004,48.246,0</coordinates>
</Point>
</Placemark>
<Placemark>
<name>374</name>
<description>Further info on the population would go here</description>
<styleUrl>#msn_ylw-circle</styleUrl>
<Point>
<coordinates>-0.52999999999997,48.23,0</coordinates>
</Point>
</Placemark>
<Placemark>
<name>375</name>
<description>Further info on the population would go here</description>
<styleUrl>#msn_ylw-circle</styleUrl>
<Point>
<coordinates>-0.52800000000002,48.239,0</coordinates>
</Point>
</Placemark>
<Placemark>
<name>376</name>
<description>Further info on the population would go here</description>
<styleUrl>#msn_ylw-circle</styleUrl>
<Point>
<coordinates>-0.52800000000002,48.238,0</coordinates>
</Point>
</Placemark>
<Placemark>
<name>377</name>
<description>Further info on the population would go here</description>
<styleUrl>#msn_ylw-circle</styleUrl>
<Point>

<coordinates>-0.52800000000002,48.196,0</coordinates>
</Point>
</Placemark>
<Placemark>
<name>378</name>
<description>Further info on the population would go here</description>
<styleUrl>#msn_ylw-circle</styleUrl>
<Point>
<coordinates>-0.52700000000004,48.245,0</coordinates>
</Point>
</Placemark>
<Placemark>
<name>379</name>
<description>Further info on the population would go here</description>
<styleUrl>#msn_ylw-circle</styleUrl>
<Point>
<coordinates>-0.52700000000004,48.237,0</coordinates>
</Point>
</Placemark>
<Placemark>
<name>380</name>
<description>Further info on the population would go here</description>
<styleUrl>#msn_ylw-circle</styleUrl>
<Point>
<coordinates>-0.52600000000007,48.328,0</coordinates>
</Point>
</Placemark>
<Placemark>
<name>381</name>
<description>Further info on the population would go here</description>
<styleUrl>#msn_ylw-circle</styleUrl>
<Point>
<coordinates>-0.52600000000007,48.22,0</coordinates>
</Point>
</Placemark>
<Placemark>
<name>382</name>
<description>Further info on the population would go here</description>
<styleUrl>#msn_ylw-circle</styleUrl>
<Point>
<coordinates>-0.52600000000007,48.234,0</coordinates>
</Point>
</Placemark>
<Placemark>
<name>383</name>
<description>Further info on the population would go here</description>
<styleUrl>#msn_ylw-circle</styleUrl>
<Point>
<coordinates>-0.52499999999998,48.214,0</coordinates>
</Point>
</Placemark>
<Placemark>
<name>384</name>
<description>Further info on the population would go here</description>
<styleUrl>#msn_ylw-circle</styleUrl>
<Point>
<coordinates>-0.524,48.243,0</coordinates>

</Point>
</Placemark>
<Placemark>
<name>385</name>
<description>Further info on the population would go here</description>
<styleUrl>#msn_ylw-circle</styleUrl>
<Point>
<coordinates>-0.524,48.17,0</coordinates>
</Point>
</Placemark>
<Placemark>
<name>386</name>
<description>Further info on the population would go here</description>
<styleUrl>#msn_ylw-circle</styleUrl>
<Point>
<coordinates>-0.52300000000002,48.17,0</coordinates>
</Point>
</Placemark>
<Placemark>
<name>387</name>
<description>Further info on the population would go here</description>
<styleUrl>#msn_ylw-circle</styleUrl>
<Point>
<coordinates>-0.52300000000002,48.241,0</coordinates>
</Point>
</Placemark>
<Placemark>
<name>388</name>
<description>Further info on the population would go here</description>
<styleUrl>#msn_ylw-circle</styleUrl>
<Point>
<coordinates>-0.52300000000002,48.36,0</coordinates>
</Point>
</Placemark>
<Placemark>
<name>389</name>
<description>Further info on the population would go here</description>
<styleUrl>#msn_ylw-circle</styleUrl>
<Point>
<coordinates>-0.52199999999993,48.151,0</coordinates>
</Point>
</Placemark>
<Placemark>
<name>390</name>
<description>Further info on the population would go here</description>
<styleUrl>#msn_ylw-circle</styleUrl>
<Point>
<coordinates>-0.52199999999993,48.328,0</coordinates>
</Point>
</Placemark>
<Placemark>
<name>391</name>
<description>Further info on the population would go here</description>
<styleUrl>#msn_ylw-circle</styleUrl>
<Point>
<coordinates>-0.52199999999993,48.324,0</coordinates>
</Point>

</Placemark>
<Placemark>
<name>392</name>
<description>Further info on the population would go here</description>
<styleUrl>#msn_ylw-circle</styleUrl>
<Point>
<coordinates>-0.52099999999996,48.158,0</coordinates>
</Point>
</Placemark>
<Placemark>
<name>393</name>
<description>Further info on the population would go here</description>
<styleUrl>#msn_ylw-circle</styleUrl>
<Point>
<coordinates>-0.52099999999996,48.249,0</coordinates>
</Point>
</Placemark>
<Placemark>
<name>394</name>
<description>Further info on the population would go here</description>
<styleUrl>#msn_ylw-circle</styleUrl>
<Point>
<coordinates>-0.52099999999996,48.233,0</coordinates>
</Point>
</Placemark>
<Placemark>
<name>395</name>
<description>Further info on the population would go here</description>
<styleUrl>#msn_ylw-circle</styleUrl>
<Point>
<coordinates>-0.52099999999996,48.327,0</coordinates>
</Point>
</Placemark>
<Placemark>
<name>396</name>
<description>Further info on the population would go here</description>
<styleUrl>#msn_ylw-circle</styleUrl>
<Point>
<coordinates>-0.52099999999996,48.232,0</coordinates>
</Point>
</Placemark>
<Placemark>
<name>397</name>
<description>Further info on the population would go here</description>
<styleUrl>#msn_ylw-circle</styleUrl>
<Point>
<coordinates>-0.52099999999996,48.314,0</coordinates>
</Point>
</Placemark>
<Placemark>
<name>398</name>
<description>Further info on the population would go here</description>
<styleUrl>#msn_ylw-circle</styleUrl>
<Point>
<coordinates>-0.51999999999998,48.233,0</coordinates>
</Point>
</Placemark>

<Placemark>
<name>399</name>
<description>Further info on the population would go here</description>
<styleUrl>#msn_ylw-circle</styleUrl>
<Point>
<coordinates>-0.51999999999998,48.206,0</coordinates>
</Point>
</Placemark>
<Placemark>
<name>400</name>
<description>Further info on the population would go here</description>
<styleUrl>#msn_ylw-circle</styleUrl>
<Point>
<coordinates>-0.51999999999998,48.206,0</coordinates>
</Point>
</Placemark>
<Placemark>
<name>401</name>
<description>Further info on the population would go here</description>
<styleUrl>#msn_ylw-circle</styleUrl>
<Point>
<coordinates>-0.51900000000001,48.163,0</coordinates>
</Point>
</Placemark>
<Placemark>
<name>402</name>
<description>Further info on the population would go here</description>
<styleUrl>#msn_ylw-circle</styleUrl>
<Point>
<coordinates>-0.51800000000003,48.312,0</coordinates>
</Point>
</Placemark>
<Placemark>
<name>403</name>
<description>Further info on the population would go here</description>
<styleUrl>#msn_ylw-circle</styleUrl>
<Point>
<coordinates>-0.51800000000003,48.312,0</coordinates>
</Point>
</Placemark>
<Placemark>
<name>404</name>
<description>Further info on the population would go here</description>
<styleUrl>#msn_ylw-circle</styleUrl>
<Point>
<coordinates>-0.51800000000003,48.151,0</coordinates>
</Point>
</Placemark>
<Placemark>
<name>405</name>
<description>Further info on the population would go here</description>
<styleUrl>#msn_ylw-circle</styleUrl>
<Point>
<coordinates>-0.51800000000003,48.321,0</coordinates>
</Point>
</Placemark>
<Placemark>

<name>406</name>
<description>Further info on the population would go here</description>
<styleUrl>#msn_ylw-circle</styleUrl>
<Point>
<coordinates>-0.51800000000003,48.22,0</coordinates>
</Point>
</Placemark>
<Placemark>
<name>407</name>
<description>Further info on the population would go here</description>
<styleUrl>#msn_ylw-circle</styleUrl>
<Point>
<coordinates>-0.51700000000005,48.308,0</coordinates>
</Point>
</Placemark>
<Placemark>
<name>408</name>
<description>Further info on the population would go here</description>
<styleUrl>#msn_ylw-circle</styleUrl>
<Point>
<coordinates>-0.51700000000005,48.22,0</coordinates>
</Point>
</Placemark>
<Placemark>
<name>409</name>
<description>Further info on the population would go here</description>
<styleUrl>#msn_ylw-circle</styleUrl>
<Point>
<coordinates>-0.51700000000005,48.319,0</coordinates>
</Point>
</Placemark>
<Placemark>
<name>410</name>
<description>Further info on the population would go here</description>
<styleUrl>#msn_ylw-circle</styleUrl>
<Point>
<coordinates>-0.51700000000005,48.216,0</coordinates>
</Point>
</Placemark>
<Placemark>
<name>411</name>
<description>Further info on the population would go here</description>
<styleUrl>#msn_ylw-circle</styleUrl>
<Point>
<coordinates>-0.51700000000005,48.312,0</coordinates>
</Point>
</Placemark>
<Placemark>
<name>412</name>
<description>Further info on the population would go here</description>
<styleUrl>#msn_ylw-circle</styleUrl>
<Point>
<coordinates>-0.51599999999996,48.233,0</coordinates>
</Point>
</Placemark>
<Placemark>
<name>413</name>

<description>Further info on the population would go here</description>
<styleUrl>#msn_ylw-circle</styleUrl>
<Point>
<coordinates>-0.51599999999996,48.213,0</coordinates>
</Point>
</Placemark>
<Placemark>
<name>414</name>
<description>Further info on the population would go here</description>
<styleUrl>#msn_ylw-circle</styleUrl>
<Point>
<coordinates>-0.51400000000001,47.963,0</coordinates>
</Point>
</Placemark>
<Placemark>
<name>415</name>
<description>Further info on the population would go here</description>
<styleUrl>#msn_ylw-circle</styleUrl>
<Point>
<coordinates>-0.51300000000003,48.231,0</coordinates>
</Point>
</Placemark>
<Placemark>
<name>416</name>
<description>Further info on the population would go here</description>
<styleUrl>#msn_ylw-circle</styleUrl>
<Point>
<coordinates>-0.51300000000003,48.312,0</coordinates>
</Point>
</Placemark>
<Placemark>
<name>417</name>
<description>Further info on the population would go here</description>
<styleUrl>#msn_ylw-circle</styleUrl>
<Point>
<coordinates>-0.51300000000003,48.312,0</coordinates>
</Point>
</Placemark>
<Placemark>
<name>418</name>
<description>Further info on the population would go here</description>
<styleUrl>#msn_ylw-circle</styleUrl>
<Point>
<coordinates>-0.51300000000003,48.26,0</coordinates>
</Point>
</Placemark>
<Placemark>
<name>419</name>
<description>Further info on the population would go here</description>
<styleUrl>#msn_ylw-circle</styleUrl>
<Point>
<coordinates>-0.51300000000003,48.239,0</coordinates>
</Point>
</Placemark>
<Placemark>
<name>420</name>
<description>Further info on the population would go here</description>

<styleUrl>#msn_ylw-circle</styleUrl>
<Point>
<coordinates>-0.51300000000003,48.251,0</coordinates>
</Point>
</Placemark>
<Placemark>
<name>421</name>
<description>Further info on the population would go here</description>
<styleUrl>#msn_ylw-circle</styleUrl>
<Point>
<coordinates>-0.511999999999994,48.324,0</coordinates>
</Point>
</Placemark>
<Placemark>
<name>422</name>
<description>Further info on the population would go here</description>
<styleUrl>#msn_ylw-circle</styleUrl>
<Point>
<coordinates>-0.510999999999997,48.324,0</coordinates>
</Point>
</Placemark>
<Placemark>
<name>423</name>
<description>Further info on the population would go here</description>
<styleUrl>#msn_ylw-circle</styleUrl>
<Point>
<coordinates>-0.509999999999999,48.252,0</coordinates>
</Point>
</Placemark>
<Placemark>
<name>424</name>
<description>Further info on the population would go here</description>
<styleUrl>#msn_ylw-circle</styleUrl>
<Point>
<coordinates>-0.509999999999999,48.31,0</coordinates>
</Point>
</Placemark>
<Placemark>
<name>425</name>
<description>Further info on the population would go here</description>
<styleUrl>#msn_ylw-circle</styleUrl>
<Point>
<coordinates>-0.509999999999999,48.31,0</coordinates>
</Point>
</Placemark>
<Placemark>
<name>426</name>
<description>Further info on the population would go here</description>
<styleUrl>#msn_ylw-circle</styleUrl>
<Point>
<coordinates>-0.509999999999999,48.313,0</coordinates>
</Point>
</Placemark>
<Placemark>
<name>427</name>
<description>Further info on the population would go here</description>
<styleUrl>#msn_ylw-circle</styleUrl>

<Point>
<coordinates>-0.50999999999999,48.322,0</coordinates>
</Point>
</Placemark>
<Placemark>
<name>428</name>
<description>Further info on the population would go here</description>
<styleUrl>#msn_ylw-circle</styleUrl>
<Point>
<coordinates>-0.50900000000001,48.251,0</coordinates>
</Point>
</Placemark>
<Placemark>
<name>429</name>
<description>Further info on the population would go here</description>
<styleUrl>#msn_ylw-circle</styleUrl>
<Point>
<coordinates>-0.50900000000001,48.32,0</coordinates>
</Point>
</Placemark>
<Placemark>
<name>430</name>
<description>Further info on the population would go here</description>
<styleUrl>#msn_ylw-circle</styleUrl>
<Point>
<coordinates>-0.50900000000001,48.319,0</coordinates>
</Point>
</Placemark>
<Placemark>
<name>431</name>
<description>Further info on the population would go here</description>
<styleUrl>#msn_ylw-circle</styleUrl>
<Point>
<coordinates>-0.50900000000001,48.319,0</coordinates>
</Point>
</Placemark>
<Placemark>
<name>432</name>
<description>Further info on the population would go here</description>
<styleUrl>#msn_ylw-circle</styleUrl>
<Point>
<coordinates>-0.50900000000001,48.101,0</coordinates>
</Point>
</Placemark>
<Placemark>
<name>433</name>
<description>Further info on the population would go here</description>
<styleUrl>#msn_ylw-circle</styleUrl>
<Point>
<coordinates>-0.50900000000001,48.238,0</coordinates>
</Point>
</Placemark>
<Placemark>
<name>434</name>
<description>Further info on the population would go here</description>
<styleUrl>#msn_ylw-circle</styleUrl>
<Point>

<coordinates>-0.50900000000001,48.2,0</coordinates>
</Point>
</Placemark>
<Placemark>
<name>435</name>
<description>Further info on the population would go here</description>
<styleUrl>#msn_ylw-circle</styleUrl>
<Point>
<coordinates>-0.50900000000001,48.1,0</coordinates>
</Point>
</Placemark>
<Placemark>
<name>436</name>
<description>Further info on the population would go here</description>
<styleUrl>#msn_ylw-circle</styleUrl>
<Point>
<coordinates>-0.50800000000004,48.15,0</coordinates>
</Point>
</Placemark>
<Placemark>
<name>437</name>
<description>Further info on the population would go here</description>
<styleUrl>#msn_ylw-circle</styleUrl>
<Point>
<coordinates>-0.50800000000004,48.119,0</coordinates>
</Point>
</Placemark>
<Placemark>
<name>438</name>
<description>Further info on the population would go here</description>
<styleUrl>#msn_ylw-circle</styleUrl>
<Point>
<coordinates>-0.50700000000006,48.182,0</coordinates>
</Point>
</Placemark>
<Placemark>
<name>439</name>
<description>Further info on the population would go here</description>
<styleUrl>#msn_ylw-circle</styleUrl>
<Point>
<coordinates>-0.50700000000006,48.229,0</coordinates>
</Point>
</Placemark>
<Placemark>
<name>440</name>
<description>Further info on the population would go here</description>
<styleUrl>#msn_ylw-circle</styleUrl>
<Point>
<coordinates>-0.50599999999997,48.297,0</coordinates>
</Point>
</Placemark>
<Placemark>
<name>441</name>
<description>Further info on the population would go here</description>
<styleUrl>#msn_ylw-circle</styleUrl>
<Point>
<coordinates>-0.50599999999997,48.28,0</coordinates>

</Point>
</Placemark>
<Placemark>
<name>442</name>
<description>Further info on the population would go here</description>
<styleUrl>#msn_ylw-circle</styleUrl>
<Point>
<coordinates>-0.50599999999997,48.255,0</coordinates>
</Point>
</Placemark>
<Placemark>
<name>443</name>
<description>Further info on the population would go here</description>
<styleUrl>#msn_ylw-circle</styleUrl>
<Point>
<coordinates>-0.505,48.297,0</coordinates>
</Point>
</Placemark>
<Placemark>
<name>444</name>
<description>Further info on the population would go here</description>
<styleUrl>#msn_ylw-circle</styleUrl>
<Point>
<coordinates>-0.50400000000002,48.102,0</coordinates>
</Point>
</Placemark>
<Placemark>
<name>445</name>
<description>Further info on the population would go here</description>
<styleUrl>#msn_ylw-circle</styleUrl>
<Point>
<coordinates>-0.50299999999993,48.241,0</coordinates>
</Point>
</Placemark>
<Placemark>
<name>446</name>
<description>Further info on the population would go here</description>
<styleUrl>#msn_ylw-circle</styleUrl>
<Point>
<coordinates>-0.50299999999993,48.267,0</coordinates>
</Point>
</Placemark>
<Placemark>
<name>447</name>
<description>Further info on the population would go here</description>
<styleUrl>#msn_ylw-circle</styleUrl>
<Point>
<coordinates>-0.50199999999995,48.243,0</coordinates>
</Point>
</Placemark>
<Placemark>
<name>448</name>
<description>Further info on the population would go here</description>
<styleUrl>#msn_ylw-circle</styleUrl>
<Point>
<coordinates>-0.50199999999995,48.235,0</coordinates>
</Point>

</Placemark>
<Placemark>
<name>449</name>
<description>Further info on the population would go here</description>
<styleUrl>#msn_ylw-circle</styleUrl>
<Point>
<coordinates>-0.50199999999995,48.147,0</coordinates>
</Point>
</Placemark>
<Placemark>
<name>450</name>
<description>Further info on the population would go here</description>
<styleUrl>#msn_ylw-circle</styleUrl>
<Point>
<coordinates>-0.50099999999998,48.294,0</coordinates>
</Point>
</Placemark>
<Placemark>
<name>451</name>
<description>Further info on the population would go here</description>
<styleUrl>#msn_ylw-circle</styleUrl>
<Point>
<coordinates>-0.50099999999998,48.195,0</coordinates>
</Point>
</Placemark>
<Placemark>
<name>452</name>
<description>Further info on the population would go here</description>
<styleUrl>#msn_ylw-circle</styleUrl>
<Point>
<coordinates>-0.5,48.317,0</coordinates>
</Point>
</Placemark>
<Placemark>
<name>453</name>
<description>Further info on the population would go here</description>
<styleUrl>#msn_ylw-circle</styleUrl>
<Point>
<coordinates>-0.49900000000002,48.196,0</coordinates>
</Point>
</Placemark>
<Placemark>
<name>454</name>
<description>Further info on the population would go here</description>
<styleUrl>#msn_ylw-circle</styleUrl>
<Point>
<coordinates>-0.49800000000005,48.257,0</coordinates>
</Point>
</Placemark>
<Placemark>
<name>455</name>
<description>Further info on the population would go here</description>
<styleUrl>#msn_ylw-circle</styleUrl>
<Point>
<coordinates>-0.49800000000005,48.218,0</coordinates>
</Point>
</Placemark>

<Placemark>
<name>456</name>
<description>Further info on the population would go here</description>
<styleUrl>#msn_ylw-circle</styleUrl>
<Point>
<coordinates>-0.49700000000007,48.233,0</coordinates>
</Point>
</Placemark>
<Placemark>
<name>457</name>
<description>Further info on the population would go here</description>
<styleUrl>#msn_ylw-circle</styleUrl>
<Point>
<coordinates>-0.49700000000007,48.256,0</coordinates>
</Point>
</Placemark>
<Placemark>
<name>458</name>
<description>Further info on the population would go here</description>
<styleUrl>#msn_ylw-circle</styleUrl>
<Point>
<coordinates>-0.49599999999998,48.253,0</coordinates>
</Point>
</Placemark>
<Placemark>
<name>459</name>
<description>Further info on the population would go here</description>
<styleUrl>#msn_ylw-circle</styleUrl>
<Point>
<coordinates>-0.49599999999998,48.223,0</coordinates>
</Point>
</Placemark>
<Placemark>
<name>460</name>
<description>Further info on the population would go here</description>
<styleUrl>#msn_ylw-circle</styleUrl>
<Point>
<coordinates>-0.495,48.259,0</coordinates>
</Point>
</Placemark>
<Placemark>
<name>461</name>
<description>Further info on the population would go here</description>
<styleUrl>#msn_ylw-circle</styleUrl>
<Point>
<coordinates>-0.495,48.261,0</coordinates>
</Point>
</Placemark>
<Placemark>
<name>462</name>
<description>Further info on the population would go here</description>
<styleUrl>#msn_ylw-circle</styleUrl>
<Point>
<coordinates>-0.49400000000003,48.236,0</coordinates>
</Point>
</Placemark>
<Placemark>

<name>463</name>
<description>Further info on the population would go here</description>
<styleUrl>#msn_ylw-circle</styleUrl>
<Point>
<coordinates>-0.49299999999994,48.248,0</coordinates>
</Point>
</Placemark>
<Placemark>
<name>464</name>
<description>Further info on the population would go here</description>
<styleUrl>#msn_ylw-circle</styleUrl>
<Point>
<coordinates>-0.49299999999994,48.21,0</coordinates>
</Point>
</Placemark>
<Placemark>
<name>465</name>
<description>Further info on the population would go here</description>
<styleUrl>#msn_ylw-circle</styleUrl>
<Point>
<coordinates>-0.49199999999996,48.231,0</coordinates>
</Point>
</Placemark>
<Placemark>
<name>466</name>
<description>Further info on the population would go here</description>
<styleUrl>#msn_ylw-circle</styleUrl>
<Point>
<coordinates>-0.49099999999999,48.253,0</coordinates>
</Point>
</Placemark>
<Placemark>
<name>467</name>
<description>Further info on the population would go here</description>
<styleUrl>#msn_ylw-circle</styleUrl>
<Point>
<coordinates>-0.49000000000001,48.111,0</coordinates>
</Point>
</Placemark>
<Placemark>
<name>468</name>
<description>Further info on the population would go here</description>
<styleUrl>#msn_ylw-circle</styleUrl>
<Point>
<coordinates>-0.49000000000001,48.111,0</coordinates>
</Point>
</Placemark>
<Placemark>
<name>469</name>
<description>Further info on the population would go here</description>
<styleUrl>#msn_ylw-circle</styleUrl>
<Point>
<coordinates>-0.48900000000003,48.108,0</coordinates>
</Point>
</Placemark>
<Placemark>
<name>470</name>

<description>Further info on the population would go here</description>
<styleUrl>#msn_ylw-circle</styleUrl>
<Point>
<coordinates>-0.48900000000003,48.111,0</coordinates>
</Point>
</Placemark>
<Placemark>
<name>471</name>
<description>Further info on the population would go here</description>
<styleUrl>#msn_ylw-circle</styleUrl>
<Point>
<coordinates>-0.48800000000006,48.28,0</coordinates>
</Point>
</Placemark>
<Placemark>
<name>472</name>
<description>Further info on the population would go here</description>
<styleUrl>#msn_ylw-circle</styleUrl>
<Point>
<coordinates>-0.48800000000006,48.219,0</coordinates>
</Point>
</Placemark>
<Placemark>
<name>473</name>
<description>Further info on the population would go here</description>
<styleUrl>#msn_ylw-circle</styleUrl>
<Point>
<coordinates>-0.48800000000006,48.21,0</coordinates>
</Point>
</Placemark>
<Placemark>
<name>474</name>
<description>Further info on the population would go here</description>
<styleUrl>#msn_ylw-circle</styleUrl>
<Point>
<coordinates>-0.48800000000006,48.108,0</coordinates>
</Point>
</Placemark>
<Placemark>
<name>475</name>
<description>Further info on the population would go here</description>
<styleUrl>#msn_ylw-circle</styleUrl>
<Point>
<coordinates>-0.48699999999997,48.235,0</coordinates>
</Point>
</Placemark>
<Placemark>
<name>476</name>
<description>Further info on the population would go here</description>
<styleUrl>#msn_ylw-circle</styleUrl>
<Point>
<coordinates>-0.48699999999997,48.042,0</coordinates>
</Point>
</Placemark>
<Placemark>
<name>477</name>
<description>Further info on the population would go here</description>

<styleUrl>#msn_ylw-circle</styleUrl>
<Point>
<coordinates>-0.485999999999999,47.961,0</coordinates>
</Point>
</Placemark>
<Placemark>
<name>478</name>
<description>Further info on the population would go here</description>
<styleUrl>#msn_ylw-circle</styleUrl>
<Point>
<coordinates>-0.485999999999999,48.274,0</coordinates>
</Point>
</Placemark>
<Placemark>
<name>479</name>
<description>Further info on the population would go here</description>
<styleUrl>#msn_ylw-circle</styleUrl>
<Point>
<coordinates>-0.485999999999999,48.165,0</coordinates>
</Point>
</Placemark>
<Placemark>
<name>480</name>
<description>Further info on the population would go here</description>
<styleUrl>#msn_ylw-circle</styleUrl>
<Point>
<coordinates>-0.485999999999999,48.209,0</coordinates>
</Point>
</Placemark>
<Placemark>
<name>481</name>
<description>Further info on the population would go here</description>
<styleUrl>#msn_ylw-circle</styleUrl>
<Point>
<coordinates>-0.481999999999997,48.113,0</coordinates>
</Point>
</Placemark>
<Placemark>
<name>482</name>
<description>Further info on the population would go here</description>
<styleUrl>#msn_ylw-circle</styleUrl>
<Point>
<coordinates>-0.481999999999997,48.243,0</coordinates>
</Point>
</Placemark>
<Placemark>
<name>483</name>
<description>Further info on the population would go here</description>
<styleUrl>#msn_ylw-circle</styleUrl>
<Point>
<coordinates>-0.481999999999997,48.112,0</coordinates>
</Point>
</Placemark>
<Placemark>
<name>484</name>
<description>Further info on the population would go here</description>
<styleUrl>#msn_ylw-circle</styleUrl>

<Point>
<coordinates>-0.48099999999999,48.238,0</coordinates>
</Point>
</Placemark>
<Placemark>
<name>485</name>
<description>Further info on the population would go here</description>
<styleUrl>#msn_ylw-circle</styleUrl>
<Point>
<coordinates>-0.47800000000007,48.294,0</coordinates>
</Point>
</Placemark>
<Placemark>
<name>486</name>
<description>Further info on the population would go here</description>
<styleUrl>#msn_ylw-circle</styleUrl>
<Point>
<coordinates>-0.47699999999998,48.249,0</coordinates>
</Point>
</Placemark>
<Placemark>
<name>487</name>
<description>Further info on the population would go here</description>
<styleUrl>#msn_ylw-circle</styleUrl>
<Point>
<coordinates>-0.47699999999998,48.209,0</coordinates>
</Point>
</Placemark>
<Placemark>
<name>488</name>
<description>Further info on the population would go here</description>
<styleUrl>#msn_ylw-circle</styleUrl>
<Point>
<coordinates>-0.47699999999998,48.253,0</coordinates>
</Point>
</Placemark>
<Placemark>
<name>489</name>
<description>Further info on the population would go here</description>
<styleUrl>#msn_ylw-circle</styleUrl>
<Point>
<coordinates>-0.476,48.191,0</coordinates>
</Point>
</Placemark>
<Placemark>
<name>490</name>
<description>Further info on the population would go here</description>
<styleUrl>#msn_ylw-circle</styleUrl>
<Point>
<coordinates>-0.47500000000002,48.447,0</coordinates>
</Point>
</Placemark>
<Placemark>
<name>491</name>
<description>Further info on the population would go here</description>
<styleUrl>#msn_ylw-circle</styleUrl>
<Point>

<coordinates>-0.47399999999993,48.432,0</coordinates>
</Point>
</Placemark>
<Placemark>
<name>492</name>
<description>Further info on the population would go here</description>
<styleUrl>#msn_ylw-circle</styleUrl>
<Point>
<coordinates>-0.47399999999993,48.28,0</coordinates>
</Point>
</Placemark>
<Placemark>
<name>493</name>
<description>Further info on the population would go here</description>
<styleUrl>#msn_ylw-circle</styleUrl>
<Point>
<coordinates>-0.47199999999998,48.222,0</coordinates>
</Point>
</Placemark>
<Placemark>
<name>494</name>
<description>Further info on the population would go here</description>
<styleUrl>#msn_ylw-circle</styleUrl>
<Point>
<coordinates>-0.471,48.252,0</coordinates>
</Point>
</Placemark>
<Placemark>
<name>495</name>
<description>Further info on the population would go here</description>
<styleUrl>#msn_ylw-circle</styleUrl>
<Point>
<coordinates>-0.46900000000005,48.188,0</coordinates>
</Point>
</Placemark>
<Placemark>
<name>496</name>
<description>Further info on the population would go here</description>
<styleUrl>#msn_ylw-circle</styleUrl>
<Point>
<coordinates>-0.46900000000005,48.187,0</coordinates>
</Point>
</Placemark>
<Placemark>
<name>497</name>
<description>Further info on the population would go here</description>
<styleUrl>#msn_ylw-circle</styleUrl>
<Point>
<coordinates>-0.46900000000005,48.251,0</coordinates>
</Point>
</Placemark>
<Placemark>
<name>498</name>
<description>Further info on the population would go here</description>
<styleUrl>#msn_ylw-circle</styleUrl>
<Point>
<coordinates>-0.46799999999996,48.22,0</coordinates>

</Point>
</Placemark>
<Placemark>
<name>499</name>
<description>Further info on the population would go here</description>
<styleUrl>#msn_ylw-circle</styleUrl>
<Point>
<coordinates>-0.467999999999996,48.242,0</coordinates>
</Point>
</Placemark>
<Placemark>
<name>500</name>
<description>Further info on the population would go here</description>
<styleUrl>#msn_ylw-circle</styleUrl>
<Point>
<coordinates>-0.465000000000003,47.837,0</coordinates>
</Point>
</Placemark>
<Placemark>
<name>501</name>
<description>Further info on the population would go here</description>
<styleUrl>#msn_ylw-circle</styleUrl>
<Point>
<coordinates>-0.465000000000003,48.246,0</coordinates>
</Point>
</Placemark>
<Placemark>
<name>502</name>
<description>Further info on the population would go here</description>
<styleUrl>#msn_ylw-circle</styleUrl>
<Point>
<coordinates>-0.463999999999994,48.204,0</coordinates>
</Point>
</Placemark>
<Placemark>
<name>503</name>
<description>Further info on the population would go here</description>
<styleUrl>#msn_ylw-circle</styleUrl>
<Point>
<coordinates>-0.462999999999997,48.223,0</coordinates>
</Point>
</Placemark>
<Placemark>
<name>504</name>
<description>Further info on the population would go here</description>
<styleUrl>#msn_ylw-circle</styleUrl>
<Point>
<coordinates>-0.461999999999999,47.781,0</coordinates>
</Point>
</Placemark>
<Placemark>
<name>505</name>
<description>Further info on the population would go here</description>
<styleUrl>#msn_ylw-circle</styleUrl>
<Point>
<coordinates>-0.461999999999999,48.223,0</coordinates>
</Point>

</Placemark>
<Placemark>
<name>506</name>
<description>Further info on the population would go here</description>
<styleUrl>#msn_ylw-circle</styleUrl>
<Point>
<coordinates>-0.461999999999999,47.782,0</coordinates>
</Point>
</Placemark>
<Placemark>
<name>507</name>
<description>Further info on the population would go here</description>
<styleUrl>#msn_ylw-circle</styleUrl>
<Point>
<coordinates>-0.460000000000004,48.223,0</coordinates>
</Point>
</Placemark>
<Placemark>
<name>508</name>
<description>Further info on the population would go here</description>
<styleUrl>#msn_ylw-circle</styleUrl>
<Point>
<coordinates>-0.460000000000004,48.229,0</coordinates>
</Point>
</Placemark>
<Placemark>
<name>509</name>
<description>Further info on the population would go here</description>
<styleUrl>#msn_ylw-circle</styleUrl>
<Point>
<coordinates>-0.452999999999997,48.218,0</coordinates>
</Point>
</Placemark>
<Placemark>
<name>510</name>
<description>Further info on the population would go here</description>
<styleUrl>#msn_ylw-circle</styleUrl>
<Point>
<coordinates>-0.452,48.228,0</coordinates>
</Point>
</Placemark>
<Placemark>
<name>511</name>
<description>Further info on the population would go here</description>
<styleUrl>#msn_ylw-circle</styleUrl>
<Point>
<coordinates>-0.452,48.218,0</coordinates>
</Point>
</Placemark>
<Placemark>
<name>512</name>
<description>Further info on the population would go here</description>
<styleUrl>#msn_ylw-circle</styleUrl>
<Point>
<coordinates>-0.451000000000002,48.255,0</coordinates>
</Point>
</Placemark>

<Placemark>
<name>513</name>
<description>Further info on the population would go here</description>
<styleUrl>#msn_ylw-circle</styleUrl>
<Point>
<coordinates>-0.44900000000007,47.81,0</coordinates>
</Point>
</Placemark>
<Placemark>
<name>514</name>
<description>Further info on the population would go here</description>
<styleUrl>#msn_ylw-circle</styleUrl>
<Point>
<coordinates>-0.44600000000003,48.203,0</coordinates>
</Point>
</Placemark>
<Placemark>
<name>515</name>
<description>Further info on the population would go here</description>
<styleUrl>#msn_ylw-circle</styleUrl>
<Point>
<coordinates>-0.44600000000003,48.219,0</coordinates>
</Point>
</Placemark>
<Placemark>
<name>516</name>
<description>Further info on the population would go here</description>
<styleUrl>#msn_ylw-circle</styleUrl>
<Point>
<coordinates>-0.44399999999996,48.218,0</coordinates>
</Point>
</Placemark>
<Placemark>
<name>517</name>
<description>Further info on the population would go here</description>
<styleUrl>#msn_ylw-circle</styleUrl>
<Point>
<coordinates>-0.44399999999996,48.182,0</coordinates>
</Point>
</Placemark>
<Placemark>
<name>518</name>
<description>Further info on the population would go here</description>
<styleUrl>#msn_ylw-circle</styleUrl>
<Point>
<coordinates>-0.44000000000005,48.128,0</coordinates>
</Point>
</Placemark>
<Placemark>
<name>519</name>
<description>Further info on the population would go here</description>
<styleUrl>#msn_ylw-circle</styleUrl>
<Point>
<coordinates>-0.43899999999996,48.187,0</coordinates>
</Point>
</Placemark>
<Placemark>

<name>520</name>
<description>Further info on the population would go here</description>
<styleUrl>#msn_ylw-circle</styleUrl>
<Point>
<coordinates>-0.437999999999999,48.302,0</coordinates>
</Point>
</Placemark>
<Placemark>
<name>521</name>
<description>Further info on the population would go here</description>
<styleUrl>#msn_ylw-circle</styleUrl>
<Point>
<coordinates>-0.437999999999999,48.299,0</coordinates>
</Point>
</Placemark>
<Placemark>
<name>522</name>
<description>Further info on the population would go here</description>
<styleUrl>#msn_ylw-circle</styleUrl>
<Point>
<coordinates>-0.437000000000001,48.303,0</coordinates>
</Point>
</Placemark>
<Placemark>
<name>523</name>
<description>Further info on the population would go here</description>
<styleUrl>#msn_ylw-circle</styleUrl>
<Point>
<coordinates>-0.431000000000004,48.193,0</coordinates>
</Point>
</Placemark>
<Placemark>
<name>524</name>
<description>Further info on the population would go here</description>
<styleUrl>#msn_ylw-circle</styleUrl>
<Point>
<coordinates>-0.428999999999997,48.283,0</coordinates>
</Point>
</Placemark>
<Placemark>
<name>525</name>
<description>Further info on the population would go here</description>
<styleUrl>#msn_ylw-circle</styleUrl>
<Point>
<coordinates>-0.428999999999997,48.138,0</coordinates>
</Point>
</Placemark>
<Placemark>
<name>526</name>
<description>Further info on the population would go here</description>
<styleUrl>#msn_ylw-circle</styleUrl>
<Point>
<coordinates>-0.428,48.253,0</coordinates>
</Point>
</Placemark>
<Placemark>
<name>527</name>

<description>Further info on the population would go here</description>
<styleUrl>#msn_ylw-circle</styleUrl>
<Point>
<coordinates>-0.41800000000001,48.194,0</coordinates>
</Point>
</Placemark>
<Placemark>
<name>528</name>
<description>Further info on the population would go here</description>
<styleUrl>#msn_ylw-circle</styleUrl>
<Point>
<coordinates>-0.41700000000003,48.207,0</coordinates>
</Point>
</Placemark>
<Placemark>
<name>529</name>
<description>Further info on the population would go here</description>
<styleUrl>#msn_ylw-circle</styleUrl>
<Point>
<coordinates>-0.41100000000006,48.456,0</coordinates>
</Point>
</Placemark>
<Placemark>
<name>530</name>
<description>Further info on the population would go here</description>
<styleUrl>#msn_ylw-circle</styleUrl>
<Point>
<coordinates>-0.40899999999999,48.374,0</coordinates>
</Point>
</Placemark>
<Placemark>
<name>531</name>
<description>Further info on the population would go here</description>
<styleUrl>#msn_ylw-circle</styleUrl>
<Point>
<coordinates>-0.40700000000004,48.046,0</coordinates>
</Point>
</Placemark>
<Placemark>
<name>532</name>
<description>Further info on the population would go here</description>
<styleUrl>#msn_ylw-circle</styleUrl>
<Point>
<coordinates>-0.404,48.358,0</coordinates>
</Point>
</Placemark>
<Placemark>
<name>533</name>
<description>Further info on the population would go here</description>
<styleUrl>#msn_ylw-circle</styleUrl>
<Point>
<coordinates>-0.40300000000002,47.974,0</coordinates>
</Point>
</Placemark>
<Placemark>
<name>534</name>
<description>Further info on the population would go here</description>

<styleUrl>#msn_ylw-circle</styleUrl>
<Point>
<coordinates>-0.40300000000002,47.974,0</coordinates>
</Point>
</Placemark>
<Placemark>
<name>535</name>
<description>Further info on the population would go here</description>
<styleUrl>#msn_ylw-circle</styleUrl>
<Point>
<coordinates>-0.40100000000007,48.106,0</coordinates>
</Point>
</Placemark>
<Placemark>
<name>536</name>
<description>Further info on the population would go here</description>
<styleUrl>#msn_ylw-circle</styleUrl>
<Point>
<coordinates>-0.39999999999998,48.105,0</coordinates>
</Point>
</Placemark>
<Placemark>
<name>537</name>
<description>Further info on the population would go here</description>
<styleUrl>#msn_ylw-circle</styleUrl>
<Point>
<coordinates>-0.399,48.29,0</coordinates>
</Point>
</Placemark>
<Placemark>
<name>538</name>
<description>Further info on the population would go here</description>
<styleUrl>#msn_ylw-circle</styleUrl>
<Point>
<coordinates>-0.399,48.289,0</coordinates>
</Point>
</Placemark>
<Placemark>
<name>539</name>
<description>Further info on the population would go here</description>
<styleUrl>#msn_ylw-circle</styleUrl>
<Point>
<coordinates>-0.399,48.106,0</coordinates>
</Point>
</Placemark>
<Placemark>
<name>540</name>
<description>Further info on the population would go here</description>
<styleUrl>#msn_ylw-circle</styleUrl>
<Point>
<coordinates>-0.39800000000002,48.42,0</coordinates>
</Point>
</Placemark>
<Placemark>
<name>541</name>
<description>Further info on the population would go here</description>
<styleUrl>#msn_ylw-circle</styleUrl>

<Point>
<coordinates>-0.39699999999993,48.296,0</coordinates>
</Point>
</Placemark>
<Placemark>
<name>542</name>
<description>Further info on the population would go here</description>
<styleUrl>#msn_ylw-circle</styleUrl>
<Point>
<coordinates>-0.39300000000003,48.077,0</coordinates>
</Point>
</Placemark>
<Placemark>
<name>543</name>
<description>Further info on the population would go here</description>
<styleUrl>#msn_ylw-circle</styleUrl>
<Point>
<coordinates>-0.39300000000003,48.354,0</coordinates>
</Point>
</Placemark>
<Placemark>
<name>544</name>
<description>Further info on the population would go here</description>
<styleUrl>#msn_ylw-circle</styleUrl>
<Point>
<coordinates>-0.39200000000005,48.282,0</coordinates>
</Point>
</Placemark>
<Placemark>
<name>545</name>
<description>Further info on the population would go here</description>
<styleUrl>#msn_ylw-circle</styleUrl>
<Point>
<coordinates>-0.39200000000005,48.241,0</coordinates>
</Point>
</Placemark>
<Placemark>
<name>546</name>
<description>Further info on the population would go here</description>
<styleUrl>#msn_ylw-circle</styleUrl>
<Point>
<coordinates>-0.39200000000005,47.968,0</coordinates>
</Point>
</Placemark>
<Placemark>
<name>547</name>
<description>Further info on the population would go here</description>
<styleUrl>#msn_ylw-circle</styleUrl>
<Point>
<coordinates>-0.38900000000001,48.333,0</coordinates>
</Point>
</Placemark>
<Placemark>
<name>548</name>
<description>Further info on the population would go here</description>
<styleUrl>#msn_ylw-circle</styleUrl>
<Point>

<coordinates>-0.38900000000001,47.967,0</coordinates>
</Point>
</Placemark>
<Placemark>
<name>549</name>
<description>Further info on the population would go here</description>
<styleUrl>#msn_ylw-circle</styleUrl>
<Point>
<coordinates>-0.38200000000006,48.442,0</coordinates>
</Point>
</Placemark>
<Placemark>
<name>550</name>
<description>Further info on the population would go here</description>
<styleUrl>#msn_ylw-circle</styleUrl>
<Point>
<coordinates>-0.38,48.186,0</coordinates>
</Point>
</Placemark>
<Placemark>
<name>551</name>
<description>Further info on the population would go here</description>
<styleUrl>#msn_ylw-circle</styleUrl>
<Point>
<coordinates>-0.37400000000002,48.047,0</coordinates>
</Point>
</Placemark>
<Placemark>
<name>552</name>
<description>Further info on the population would go here</description>
<styleUrl>#msn_ylw-circle</styleUrl>
<Point>
<coordinates>-0.37200000000007,48.464,0</coordinates>
</Point>
</Placemark>
<Placemark>
<name>553</name>
<description>Further info on the population would go here</description>
<styleUrl>#msn_ylw-circle</styleUrl>
<Point>
<coordinates>-0.36799999999994,48.418,0</coordinates>
</Point>
</Placemark>
<Placemark>
<name>554</name>
<description>Further info on the population would go here</description>
<styleUrl>#msn_ylw-circle</styleUrl>
<Point>
<coordinates>-0.36699999999996,48.244,0</coordinates>
</Point>
</Placemark>
<Placemark>
<name>555</name>
<description>Further info on the population would go here</description>
<styleUrl>#msn_ylw-circle</styleUrl>
<Point>
<coordinates>-0.36699999999996,48.233,0</coordinates>

</Point>
</Placemark>
<Placemark>
<name>556</name>
<description>Further info on the population would go here</description>
<styleUrl>#msn_ylw-circle</styleUrl>
<Point>
<coordinates>-0.36400000000003,48.443,0</coordinates>
</Point>
</Placemark>
<Placemark>
<name>557</name>
<description>Further info on the population would go here</description>
<styleUrl>#msn_ylw-circle</styleUrl>
<Point>
<coordinates>-0.36300000000006,48.1,0</coordinates>
</Point>
</Placemark>
<Placemark>
<name>558</name>
<description>Further info on the population would go here</description>
<styleUrl>#msn_ylw-circle</styleUrl>
<Point>
<coordinates>-0.36300000000006,48.145,0</coordinates>
</Point>
</Placemark>
<Placemark>
<name>559</name>
<description>Further info on the population would go here</description>
<styleUrl>#msn_ylw-circle</styleUrl>
<Point>
<coordinates>-0.36300000000006,48.401,0</coordinates>
</Point>
</Placemark>
<Placemark>
<name>560</name>
<description>Further info on the population would go here</description>
<styleUrl>#msn_ylw-circle</styleUrl>
<Point>
<coordinates>-0.36300000000006,48.1,0</coordinates>
</Point>
</Placemark>
<Placemark>
<name>561</name>
<description>Further info on the population would go here</description>
<styleUrl>#msn_ylw-circle</styleUrl>
<Point>
<coordinates>-0.36199999999997,48.375,0</coordinates>
</Point>
</Placemark>
<Placemark>
<name>562</name>
<description>Further info on the population would go here</description>
<styleUrl>#msn_ylw-circle</styleUrl>
<Point>
<coordinates>-0.36099999999999,48.376,0</coordinates>
</Point>

</Placemark>
<Placemark>
<name>563</name>
<description>Further info on the population would go here</description>
<styleUrl>#msn_ylw-circle</styleUrl>
<Point>
<coordinates>-0.35900000000004,48.432,0</coordinates>
</Point>
</Placemark>
<Placemark>
<name>564</name>
<description>Further info on the population would go here</description>
<styleUrl>#msn_ylw-circle</styleUrl>
<Point>
<coordinates>-0.35500000000002,48.316,0</coordinates>
</Point>
</Placemark>
<Placemark>
<name>565</name>
<description>Further info on the population would go here</description>
<styleUrl>#msn_ylw-circle</styleUrl>
<Point>
<coordinates>-0.35500000000002,48.373,0</coordinates>
</Point>
</Placemark>
<Placemark>
<name>566</name>
<description>Further info on the population would go here</description>
<styleUrl>#msn_ylw-circle</styleUrl>
<Point>
<coordinates>-0.34899999999993,48.38,0</coordinates>
</Point>
</Placemark>
<Placemark>
<name>567</name>
<description>Further info on the population would go here</description>
<styleUrl>#msn_ylw-circle</styleUrl>
<Point>
<coordinates>-0.34799999999996,48.14,0</coordinates>
</Point>
</Placemark>
<Placemark>
<name>568</name>
<description>Further info on the population would go here</description>
<styleUrl>#msn_ylw-circle</styleUrl>
<Point>
<coordinates>-0.346,48.39,0</coordinates>
</Point>
</Placemark>
<Placemark>
<name>569</name>
<description>Further info on the population would go here</description>
<styleUrl>#msn_ylw-circle</styleUrl>
<Point>
<coordinates>-0.34400000000005,48.339,0</coordinates>
</Point>
</Placemark>

<Placemark>
<name>570</name>
<description>Further info on the population would go here</description>
<styleUrl>#msn_ylw-circle</styleUrl>
<Point>
<coordinates>-0.34400000000005,48.376,0</coordinates>
</Point>
</Placemark>
<Placemark>
<name>571</name>
<description>Further info on the population would go here</description>
<styleUrl>#msn_ylw-circle</styleUrl>
<Point>
<coordinates>-0.34299999999996,48.302,0</coordinates>
</Point>
</Placemark>
<Placemark>
<name>572</name>
<description>Further info on the population would go here</description>
<styleUrl>#msn_ylw-circle</styleUrl>
<Point>
<coordinates>-0.34199999999998,48.137,0</coordinates>
</Point>
</Placemark>
<Placemark>
<name>573</name>
<description>Further info on the population would go here</description>
<styleUrl>#msn_ylw-circle</styleUrl>
<Point>
<coordinates>-0.34199999999998,48.423,0</coordinates>
</Point>
</Placemark>
<Placemark>
<name>574</name>
<description>Further info on the population would go here</description>
<styleUrl>#msn_ylw-circle</styleUrl>
<Point>
<coordinates>-0.33100000000002,48.133,0</coordinates>
</Point>
</Placemark>
<Placemark>
<name>575</name>
<description>Further info on the population would go here</description>
<styleUrl>#msn_ylw-circle</styleUrl>
<Point>
<coordinates>-0.327,48.365,0</coordinates>
</Point>
</Placemark>
<Placemark>
<name>576</name>
<description>Further info on the population would go here</description>
<styleUrl>#msn_ylw-circle</styleUrl>
<Point>
<coordinates>-0.32500000000005,48.265,0</coordinates>
</Point>
</Placemark>
<Placemark>

<name>577</name>
<description>Further info on the population would go here</description>
<styleUrl>#msn_ylw-circle</styleUrl>
<Point>
<coordinates>-0.32500000000005,48.265,0</coordinates>
</Point>
</Placemark>
<Placemark>
<name>578</name>
<description>Further info on the population would go here</description>
<styleUrl>#msn_ylw-circle</styleUrl>
<Point>
<coordinates>-0.322,48.446,0</coordinates>
</Point>
</Placemark>
<Placemark>
<name>579</name>
<description>Further info on the population would go here</description>
<styleUrl>#msn_ylw-circle</styleUrl>
<Point>
<coordinates>-0.32100000000003,48.397,0</coordinates>
</Point>
</Placemark>
<Placemark>
<name>580</name>
<description>Further info on the population would go here</description>
<styleUrl>#msn_ylw-circle</styleUrl>
<Point>
<coordinates>-0.31799999999998,48.459,0</coordinates>
</Point>
</Placemark>
<Placemark>
<name>581</name>
<description>Further info on the population would go here</description>
<styleUrl>#msn_ylw-circle</styleUrl>
<Point>
<coordinates>-0.31799999999998,48.378,0</coordinates>
</Point>
</Placemark>
<Placemark>
<name>582</name>
<description>Further info on the population would go here</description>
<styleUrl>#msn_ylw-circle</styleUrl>
<Point>
<coordinates>-0.31700000000001,48.184,0</coordinates>
</Point>
</Placemark>
<Placemark>
<name>583</name>
<description>Further info on the population would go here</description>
<styleUrl>#msn_ylw-circle</styleUrl>
<Point>
<coordinates>-0.31700000000001,48.21,0</coordinates>
</Point>
</Placemark>
<Placemark>
<name>584</name>

<description>Further info on the population would go here</description>
<styleUrl>#msn_ylw-circle</styleUrl>
<Point>
<coordinates>-0.31700000000001,48.463,0</coordinates>
</Point>
</Placemark>
<Placemark>
<name>585</name>
<description>Further info on the population would go here</description>
<styleUrl>#msn_ylw-circle</styleUrl>
<Point>
<coordinates>-0.31600000000003,48.437,0</coordinates>
</Point>
</Placemark>
<Placemark>
<name>586</name>
<description>Further info on the population would go here</description>
<styleUrl>#msn_ylw-circle</styleUrl>
<Point>
<coordinates>-0.31399999999996,48.337,0</coordinates>
</Point>
</Placemark>
<Placemark>
<name>587</name>
<description>Further info on the population would go here</description>
<styleUrl>#msn_ylw-circle</styleUrl>
<Point>
<coordinates>-0.31299999999999,48.447,0</coordinates>
</Point>
</Placemark>
<Placemark>
<name>588</name>
<description>Further info on the population would go here</description>
<styleUrl>#msn_ylw-circle</styleUrl>
<Point>
<coordinates>-0.30600000000004,48.471,0</coordinates>
</Point>
</Placemark>
<Placemark>
<name>589</name>
<description>Further info on the population would go here</description>
<styleUrl>#msn_ylw-circle</styleUrl>
<Point>
<coordinates>-0.28999999999996,48.433,0</coordinates>
</Point>
</Placemark>
<Placemark>
<name>590</name>
<description>Further info on the population would go here</description>
<styleUrl>#msn_ylw-circle</styleUrl>
<Point>
<coordinates>-0.28899999999999,48.427,0</coordinates>
</Point>
</Placemark>
<Placemark>
<name>591</name>
<description>Further info on the population would go here</description>

<styleUrl>#msn_ylw-circle</styleUrl>
<Point>
<coordinates>-0.28800000000001,48.425,0</coordinates>
</Point>
</Placemark>
<Placemark>
<name>592</name>
<description>Further info on the population would go here</description>
<styleUrl>#msn_ylw-circle</styleUrl>
<Point>
<coordinates>-0.28800000000001,48.425,0</coordinates>
</Point>
</Placemark>
<Placemark>
<name>593</name>
<description>Further info on the population would go here</description>
<styleUrl>#msn_ylw-circle</styleUrl>
<Point>
<coordinates>-0.28800000000001,48.468,0</coordinates>
</Point>
</Placemark>
<Placemark>
<name>594</name>
<description>Further info on the population would go here</description>
<styleUrl>#msn_ylw-circle</styleUrl>
<Point>
<coordinates>-0.28600000000006,48.388,0</coordinates>
</Point>
</Placemark>
<Placemark>
<name>595</name>
<description>Further info on the population would go here</description>
<styleUrl>#msn_ylw-circle</styleUrl>
<Point>
<coordinates>-0.28300000000002,48.501,0</coordinates>
</Point>
</Placemark>
<Placemark>
<name>596</name>
<description>Further info on the population would go here</description>
<styleUrl>#msn_ylw-circle</styleUrl>
<Point>
<coordinates>-0.28200000000004,48.429,0</coordinates>
</Point>
</Placemark>
<Placemark>
<name>597</name>
<description>Further info on the population would go here</description>
<styleUrl>#msn_ylw-circle</styleUrl>
<Point>
<coordinates>-0.28200000000004,48.389,0</coordinates>
</Point>
</Placemark>
<Placemark>
<name>598</name>
<description>Further info on the population would go here</description>
<styleUrl>#msn_ylw-circle</styleUrl>

<Point>
<coordinates>-0.28099999999995,48.429,0</coordinates>
</Point>
</Placemark>
<Placemark>
<name>599</name>
<description>Further info on the population would go here</description>
<styleUrl>#msn_ylw-circle</styleUrl>
<Point>
<coordinates>-0.27700000000004,48.39,0</coordinates>
</Point>
</Placemark>
<Placemark>
<name>600</name>
<description>Further info on the population would go here</description>
<styleUrl>#msn_ylw-circle</styleUrl>
<Point>
<coordinates>-0.27300000000002,48.449,0</coordinates>
</Point>
</Placemark>
<Placemark>
<name>601</name>
<description>Further info on the population would go here</description>
<styleUrl>#msn_ylw-circle</styleUrl>
<Point>
<coordinates>-0.27199999999993,48.448,0</coordinates>
</Point>
</Placemark>
<Placemark>
<name>602</name>
<description>Further info on the population would go here</description>
<styleUrl>#msn_ylw-circle</styleUrl>
<Point>
<coordinates>-0.27099999999996,48.448,0</coordinates>
</Point>
</Placemark>
<Placemark>
<name>603</name>
<description>Further info on the population would go here</description>
<styleUrl>#msn_ylw-circle</styleUrl>
<Point>
<coordinates>-0.26900000000001,48.431,0</coordinates>
</Point>
</Placemark>
<Placemark>
<name>604</name>
<description>Further info on the population would go here</description>
<styleUrl>#msn_ylw-circle</styleUrl>
<Point>
<coordinates>-0.26700000000005,48.205,0</coordinates>
</Point>
</Placemark>
<Placemark>
<name>605</name>
<description>Further info on the population would go here</description>
<styleUrl>#msn_ylw-circle</styleUrl>
<Point>

<coordinates>-0.264999999999999,48.443,0</coordinates>
</Point>
</Placemark>
<Placemark>
<name>606</name>
<description>Further info on the population would go here</description>
<styleUrl>#msn_ylw-circle</styleUrl>
<Point>
<coordinates>-0.260999999999997,48.443,0</coordinates>
</Point>
</Placemark>
<Placemark>
<name>607</name>
<description>Further info on the population would go here</description>
<styleUrl>#msn_ylw-circle</styleUrl>
<Point>
<coordinates>-0.260999999999997,48.433,0</coordinates>
</Point>
</Placemark>
<Placemark>
<name>608</name>
<description>Further info on the population would go here</description>
<styleUrl>#msn_ylw-circle</styleUrl>
<Point>
<coordinates>-0.259999999999999,48.443,0</coordinates>
</Point>
</Placemark>
<Placemark>
<name>609</name>
<description>Further info on the population would go here</description>
<styleUrl>#msn_ylw-circle</styleUrl>
<Point>
<coordinates>-0.257000000000006,48.44,0</coordinates>
</Point>
</Placemark>
<Placemark>
<name>610</name>
<description>Further info on the population would go here</description>
<styleUrl>#msn_ylw-circle</styleUrl>
<Point>
<coordinates>-0.255999999999997,48.43,0</coordinates>
</Point>
</Placemark>
<Placemark>
<name>611</name>
<description>Further info on the population would go here</description>
<styleUrl>#msn_ylw-circle</styleUrl>
<Point>
<coordinates>-0.255999999999997,48.431,0</coordinates>
</Point>
</Placemark>
<Placemark>
<name>612</name>
<description>Further info on the population would go here</description>
<styleUrl>#msn_ylw-circle</styleUrl>
<Point>
<coordinates>-0.255,48.436,0</coordinates>

</Point>
</Placemark>
<Placemark>
<name>613</name>
<description>Further info on the population would go here</description>
<styleUrl>#msn_ylw-circle</styleUrl>
<Point>
<coordinates>-0.25400000000002,48.102,0</coordinates>
</Point>
</Placemark>
<Placemark>
<name>614</name>
<description>Further info on the population would go here</description>
<styleUrl>#msn_ylw-circle</styleUrl>
<Point>
<coordinates>-0.25400000000002,48.471,0</coordinates>
</Point>
</Placemark>
<Placemark>
<name>615</name>
<description>Further info on the population would go here</description>
<styleUrl>#msn_ylw-circle</styleUrl>
<Point>
<coordinates>-0.25199999999995,48.471,0</coordinates>
</Point>
</Placemark>
<Placemark>
<name>616</name>
<description>Further info on the population would go here</description>
<styleUrl>#msn_ylw-circle</styleUrl>
<Point>
<coordinates>-0.25199999999995,48.441,0</coordinates>
</Point>
</Placemark>
<Placemark>
<name>617</name>
<description>Further info on the population would go here</description>
<styleUrl>#msn_ylw-circle</styleUrl>
<Point>
<coordinates>-0.25099999999998,48.447,0</coordinates>
</Point>
</Placemark>
<Placemark>
<name>618</name>
<description>Further info on the population would go here</description>
<styleUrl>#msn_ylw-circle</styleUrl>
<Point>
<coordinates>-0.25099999999998,48.4,0</coordinates>
</Point>
</Placemark>
<Placemark>
<name>619</name>
<description>Further info on the population would go here</description>
<styleUrl>#msn_ylw-circle</styleUrl>
<Point>
<coordinates>-0.24800000000005,48.124,0</coordinates>
</Point>

</Placemark>
<Placemark>
<name>620</name>
<description>Further info on the population would go here</description>
<styleUrl>#msn_ylw-circle</styleUrl>
<Point>
<coordinates>-0.24700000000007,48.406,0</coordinates>
</Point>
</Placemark>
<Placemark>
<name>621</name>
<description>Further info on the population would go here</description>
<styleUrl>#msn_ylw-circle</styleUrl>
<Point>
<coordinates>-0.245,48.352,0</coordinates>
</Point>
</Placemark>
<Placemark>
<name>622</name>
<description>Further info on the population would go here</description>
<styleUrl>#msn_ylw-circle</styleUrl>
<Point>
<coordinates>-0.24400000000003,48.455,0</coordinates>
</Point>
</Placemark>
<Placemark>
<name>623</name>
<description>Further info on the population would go here</description>
<styleUrl>#msn_ylw-circle</styleUrl>
<Point>
<coordinates>-0.24199999999996,48.408,0</coordinates>
</Point>
</Placemark>
<Placemark>
<name>624</name>
<description>Further info on the population would go here</description>
<styleUrl>#msn_ylw-circle</styleUrl>
<Point>
<coordinates>-0.24099999999999,48.431,0</coordinates>
</Point>
</Placemark>
<Placemark>
<name>625</name>
<description>Further info on the population would go here</description>
<styleUrl>#msn_ylw-circle</styleUrl>
<Point>
<coordinates>-0.23199999999997,48.427,0</coordinates>
</Point>
</Placemark>
<Placemark>
<name>626</name>
<description>Further info on the population would go here</description>
<styleUrl>#msn_ylw-circle</styleUrl>
<Point>
<coordinates>-0.23099999999999,48.416,0</coordinates>
</Point>
</Placemark>

<Placemark>
<name>627</name>
<description>Further info on the population would go here</description>
<styleUrl>#msn_ylw-circle</styleUrl>
<Point>
<coordinates>-0.23000000000002,48.44,0</coordinates>
</Point>
</Placemark>
<Placemark>
<name>628</name>
<description>Further info on the population would go here</description>
<styleUrl>#msn_ylw-circle</styleUrl>
<Point>
<coordinates>-0.22699999999998,48.418,0</coordinates>
</Point>
</Placemark>
<Placemark>
<name>629</name>
<description>Further info on the population would go here</description>
<styleUrl>#msn_ylw-circle</styleUrl>
<Point>
<coordinates>-0.22000000000003,48.401,0</coordinates>
</Point>
</Placemark>
<Placemark>
<name>630</name>
<description>Further info on the population would go here</description>
<styleUrl>#msn_ylw-circle</styleUrl>
<Point>
<coordinates>-0.21199999999999,48.438,0</coordinates>
</Point>
</Placemark>
<Placemark>
<name>631</name>
<description>Further info on the population would go here</description>
<styleUrl>#msn_ylw-circle</styleUrl>
<Point>
<coordinates>-0.20500000000004,48.48,0</coordinates>
</Point>
</Placemark>
<Placemark>
<name>632</name>
<description>Further info on the population would go here</description>
<styleUrl>#msn_ylw-circle</styleUrl>
<Point>
<coordinates>-0.197,48.152,0</coordinates>
</Point>
</Placemark>
<Placemark>
<name>633</name>
<description>Further info on the population would go here</description>
<styleUrl>#msn_ylw-circle</styleUrl>
<Point>
<coordinates>-0.19000000000005,48.377,0</coordinates>
</Point>
</Placemark>
<Placemark>

<name>634</name>
<description>Further info on the population would go here</description>
<styleUrl>#msn_ylw-circle</styleUrl>
<Point>
<coordinates>-0.18700000000001,48.384,0</coordinates>
</Point>
</Placemark>
<Placemark>
<name>635</name>
<description>Further info on the population would go here</description>
<styleUrl>#msn_ylw-circle</styleUrl>
<Point>
<coordinates>-0.18600000000004,48.409,0</coordinates>
</Point>
</Placemark>
<Placemark>
<name>636</name>
<description>Further info on the population would go here</description>
<styleUrl>#msn_ylw-circle</styleUrl>
<Point>
<coordinates>-0.18499999999995,48.388,0</coordinates>
</Point>
</Placemark>
<Placemark>
<name>637</name>
<description>Further info on the population would go here</description>
<styleUrl>#msn_ylw-circle</styleUrl>
<Point>
<coordinates>-0.18299999999999,48.372,0</coordinates>
</Point>
</Placemark>
<Placemark>
<name>638</name>
<description>Further info on the population would go here</description>
<styleUrl>#msn_ylw-circle</styleUrl>
<Point>
<coordinates>-0.16499999999996,48.448,0</coordinates>
</Point>
</Placemark>
<Placemark>
<name>639</name>
<description>Further info on the population would go here</description>
<styleUrl>#msn_ylw-circle</styleUrl>
<Point>
<coordinates>-0.13099999999997,48.443,0</coordinates>
</Point>
</Placemark>
<Placemark>
<name>640</name>
<description>Further info on the population would go here</description>
<styleUrl>#msn_ylw-circle</styleUrl>
<Point>
<coordinates>-0.13,48.389,0</coordinates>
</Point>
</Placemark>
</Folder>

<Folder>
<name>population pies</name>
<description>Each locality has a pie where each slice represents the proportion of a haplotype in the population. Pie slices are organized by haplotype (instead of grouped together by population) to facilitate changing colors and turning on and off pie pieces.</description>
<Folder>
<name>hapgroup0</name>
<Folder>
<name>hap0</name>
<Placemark>
<name>42</name>
<styleUrl>#stylehap1</styleUrl>
<Polygon>
<outerBoundaryIs>
<LinearRing>
<coordinates>
-1.158,47.844,0
-1.156370680565,47.843999988474,0
-1.1563768776352,47.844095295124,0
-1.1563954277145,47.844189876607,0
-1.1564261896915,47.844283013093,0
-1.1564689295109,47.844373995748,0
-1.1565233219525,47.844462132127,0
-1.1565889531053,47.844546751442,0
-1.1566653235157,47.84462720967,0
-1.1567518519876,47.844702894454,0
-1.1568478800047,47.844773229767,0
-1.1569526767417,47.84483768029,0
-1.1570654446263,47.844895755493,0
-1.1571853254092,47.844947013368,0
-1.1573114066963,47.84499106379,0
-1.1574427288939,47.845027571489,0
-1.1575782925125,47.845056258605,0
-1.1577170657753,47.845076906797,0
-1.1578579924722,47.845089358912,0
-1.158,47.845093520173,0
-1.1581420075278,47.845089358912,0
-1.1582829342247,47.845076906797,0
-1.1584217074875,47.845056258605,0
-1.1585572711061,47.845027571489,0
-1.1586885933037,47.84499106379,0
-1.1588146745909,47.844947013368,0
-1.1589345553737,47.844895755493,0
-1.1590473232583,47.84483768029,0
-1.1591521199954,47.844773229767,0
-1.1592481480125,47.844702894454,0
-1.1593346764844,47.84462720967,0
-1.1594110468948,47.844546751442,0
-1.1594766780476,47.844462132127,0
-1.1595310704892,47.844373995748,0
-1.1595738103085,47.844283013093,0
-1.1596045722855,47.844189876607,0
-1.1596231223648,47.844095295124,0
-1.159629319435,47.843999988474,0
-1.1596231164005,47.843904681998,0
-1.159604560538,47.843810101036,0
-1.1595737931347,47.843716965399,0

-1.159531048411,47.843625983896,0
-1.1594766517358,47.843537848938,0
-1.1594110171489,47.843453231269,0
-1.1593346442082,47.843372774862,0
-1.1592481141868,47.843297092018,0
-1.1591520856478,47.843226758707,0
-1.1590472894326,47.843162310185,0
-1.1589345230976,47.843104236922,0
-1.158814644845,47.843052980869,0
-1.158688566992,47.843008932093,0
-1.158557249028,47.842972425814,0
-1.1584216903137,47.842943739851,0
-1.1582829224771,47.842923092508,0
-1.1581420015634,47.842910640913,0
-1.158,47.842906479826,0
-1.1578579984366,47.842910640913,0
-1.1577170775229,47.842923092508,0
-1.1575783096863,47.842943739851,0
-1.1574427509721,47.842972425814,0
-1.157311433008,47.843008932093,0
-1.157185355155,47.843052980869,0
-1.1570654769024,47.843104236922,0
-1.1569527105674,47.843162310185,0
-1.1568479143522,47.843226758707,0
-1.1567518858133,47.843297092018,0
-1.1566653557918,47.843372774862,0
-1.1565889828511,47.843453231269,0
-1.1565233482642,47.843537848938,0
-1.156468951589,47.843625983896,0
-1.1564262068653,47.843716965399,0
-1.156395439462,47.843810101036,0
-1.1563768835996,47.843904681998,0
-1.156370680565,47.843999988474,0
-1.158,47.844,0
</coordinates>
</LinearRing>
</outerBoundaryIs>
</Polygon>
</Placemark>
<Placemark>
<name>43</name>
<styleUrl>#stylehap1</styleUrl>
<Polygon>
<outerBoundaryIs>
<LinearRing>
<coordinates>
-1.139,47.834,0
-1.1373709945804,47.833999988478,0
-1.1373771904572,47.834095295128,0
-1.1373957369624,47.834189876611,0
-1.1374264930117,47.834283013097,0
-1.1374692245947,47.834373995752,0
-1.1375236065542,47.834462132131,0
-1.1375892250586,47.834546751445,0
-1.1376655807507,47.834627209673,0
-1.1377520925465,47.834702894457,0
-1.1378481020563,47.834773229769,0

-1.137952878596,47.834837680292,0
-1.1380656247469,47.834895755495,0
-1.1381854824249,47.834947013369,0
-1.1383115394121,47.834991063791,0
-1.1384428362995,47.83502757149,0
-1.1385783737903,47.835056258605,0
-1.1387171203067,47.835076906797,0
-1.138858019842,47.835089358912,0
-1.139,47.835093520174,0
-1.139141980158,47.835089358912,0
-1.1392828796933,47.835076906797,0
-1.1394216262097,47.835056258605,0
-1.1395571637006,47.83502757149,0
-1.1396884605879,47.834991063791,0
-1.1398145175751,47.834947013369,0
-1.1399343752531,47.834895755495,0
-1.140047121404,47.834837680292,0
-1.1401518979437,47.834773229769,0
-1.1402479074536,47.834702894457,0
-1.1403344192493,47.834627209673,0
-1.1404107749414,47.834546751445,0
-1.1404763934459,47.834462132131,0
-1.1405307754053,47.834373995752,0
-1.1405735069883,47.834283013097,0
-1.1406042630376,47.834189876611,0
-1.1406228095428,47.834095295128,0
-1.1406290054196,47.833999988478,0
-1.1406228035816,47.833904682002,0
-1.1406042512964,47.83381010104,0
-1.1405734898238,47.833716965403,0
-1.1405307533391,47.833625983899,0
-1.1404763671484,47.833537848941,0
-1.1404107452117,47.833453231272,0
-1.1403343869907,47.833372774864,0
-1.1402478736462,47.83329709202,0
-1.1401518636148,47.833226758709,0
-1.1400470875966,47.833162310187,0
-1.1399343429946,47.833104236924,0
-1.1398144878454,47.83305298087,0
-1.1396884342905,47.833008932094,0
-1.1395571416344,47.832972425814,0
-1.1394216090452,47.832943739851,0
-1.1392828679521,47.832923092508,0
-1.1391419741968,47.832910640913,0
-1.139,47.832906479826,0
-1.1388580258032,47.832910640913,0
-1.1387171320479,47.832923092508,0
-1.1385783909548,47.832943739851,0
-1.1384428583657,47.832972425814,0
-1.1383115657095,47.833008932094,0
-1.1381855121546,47.83305298087,0
-1.1380656570055,47.833104236924,0
-1.1379529124034,47.833162310187,0
-1.1378481363852,47.833226758709,0
-1.1377521263538,47.83329709202,0
-1.1376656130094,47.833372774864,0
-1.1375892547883,47.833453231272,0

-1.1375236328516,47.833537848941,0
-1.1374692466609,47.833625983899,0
-1.1374265101762,47.833716965403,0
-1.1373957487036,47.83381010104,0
-1.1373771964184,47.833904682002,0
-1.1373709945804,47.833999988478,0
-1.139,47.834,0
</coordinates>
</LinearRing>
</outerBoundaryIs>
</Polygon>
</Placemark>
<Placemark>
<name>44</name>
<styleUrl>#stylehap1</styleUrl>
<Polygon>
<outerBoundaryIs>
<LinearRing>
<coordinates>
-1.081,47.895,0
-1.0793690764285,47.894999988453,0
-1.0793752795946,47.895095295104,0
-1.0793938479321,47.895189876587,0
-1.0794246401907,47.895283013074,0
-1.0794674220849,47.89537399573,0
-1.0795218680745,47.89546213211,0
-1.079587563841,47.895546751427,0
-1.0796640094391,47.895627209656,0
-1.0797506231007,47.895702894442,0
-1.0798467456611,47.895773229757,0
-1.0799516455756,47.895837680281,0
-1.0800645244866,47.895895755487,0
-1.0801845232996,47.895947013363,0
-1.0803107287224,47.895991063786,0
-1.0804421802162,47.896027571487,0
-1.0805778773076,47.896056258604,0
-1.0807167872036,47.896076906797,0
-1.0808578526542,47.896089358911,0
-1.081,47.896093520174,0
-1.0811421473458,47.896089358911,0
-1.0812832127964,47.896076906797,0
-1.0814221226924,47.896056258604,0
-1.0815578197838,47.896027571487,0
-1.0816892712777,47.895991063786,0
-1.0818154767005,47.895947013363,0
-1.0819354755135,47.895895755487,0
-1.0820483544244,47.895837680281,0
-1.0821532543389,47.895773229757,0
-1.0822493768993,47.895702894442,0
-1.0823359905609,47.895627209656,0
-1.0824124361591,47.895546751427,0
-1.0824781319256,47.89546213211,0
-1.0825325779151,47.89537399573,0
-1.0825753598093,47.895283013074,0
-1.082606152068,47.895189876587,0
-1.0826247204055,47.895095295104,0
-1.0826309235716,47.894999988453,0

-1.0826247144245,47.894904681978,0
-1.0826061402878,47.894810101016,0
-1.0825753425878,47.89471696538,0
-1.0825325557756,47.894625983877,0
-1.0824781055407,47.894537848921,0
-1.0824124063306,47.894453231253,0
-1.0823359581952,47.894372774848,0
-1.0822493429797,47.894297092006,0
-1.082153219896,47.894226758697,0
-1.0820483205048,47.894162310177,0
-1.0819354431477,47.894104236916,0
-1.081815446872,47.894052980864,0
-1.0816892448929,47.894008932089,0
-1.0815577976443,47.893972425812,0
-1.081422105471,47.893943739849,0
-1.0812832010162,47.893923092507,0
-1.0811421413649,47.893910640913,0
-1.081,47.893906479826,0
-1.0808578586352,47.893910640913,0
-1.0807167989838,47.893923092507,0
-1.080577894529,47.893943739849,0
-1.0804422023557,47.893972425812,0
-1.0803107551072,47.894008932089,0
-1.080184553128,47.894052980864,0
-1.0800645568523,47.894104236916,0
-1.0799516794953,47.894162310177,0
-1.079846780104,47.894226758697,0
-1.0797506570203,47.894297092006,0
-1.0796640418049,47.894372774848,0
-1.0795875936694,47.894453231253,0
-1.0795218944593,47.894537848921,0
-1.0794674442244,47.894625983877,0
-1.0794246574122,47.89471696538,0
-1.0793938597123,47.894810101016,0
-1.0793752855755,47.894904681978,0
-1.0793690764285,47.894999988453,0
-1.081,47.895,0
</coordinates>
</LinearRing>
</outerBoundaryIs>
</Polygon>
</Placemark>
<Placemark>
<name>45</name>
<styleUrl>#stylehap1</styleUrl>
<Polygon>
<outerBoundaryIs>
<LinearRing>
<coordinates>
-1.066,48.448,0
-1.064351393053,48.447999988227,0
-1.064357663418,48.448095294879,0
-1.0643764330262,48.448189876367,0
-1.0644075590976,48.448283012862,0
-1.064450804808,48.44837399553,0
-1.064505841089,48.448462131924,0
-1.0645722491312,48.448546751257,0

-1.0646495235697,48.448627209504,0
-1.0647370763291,48.448702894309,0
-1.0648342410982,48.448773229643,0
-1.0649402784008,48.448837680188,0
-1.0650543812228,48.448895755412,0
-1.0651756811545,48.448947013306,0
-1.0653032550004,48.448991063746,0
-1.0654361318061,48.44902757146,0
-1.0655733002492,48.449056258588,0
-1.0657137163371,48.44907690679,0
-1.0658563113548,48.44908935891,0
-1.066,48.449093520173,0
-1.0661436886453,48.44908935891,0
-1.066286283663,48.44907690679,0
-1.0664266997509,48.449056258588,0
-1.0665638681939,48.44902757146,0
-1.0666967449997,48.448991063746,0
-1.0668243188456,48.448947013306,0
-1.0669456187773,48.448895755412,0
-1.0670597215992,48.448837680188,0
-1.0671657589018,48.448773229643,0
-1.067262923671,48.448702894309,0
-1.0673504764304,48.448627209504,0
-1.0674277508689,48.448546751257,0
-1.067494158911,48.448462131924,0
-1.0675491951921,48.44837399553,0
-1.0675924409024,48.448283012862,0
-1.0676235669739,48.448189876367,0
-1.0676423365821,48.448095294879,0
-1.067648606947,48.447999988227,0
-1.0676423304177,48.447904681753,0
-1.0676235548324,48.447810100796,0
-1.0675924231528,48.447716965168,0
-1.0675491723736,48.447625983678,0
-1.0674941317171,48.447537848735,0
-1.0674277201257,48.447453231083,0
-1.067350443072,48.447372774696,0
-1.0672628887111,48.447297091873,0
-1.0671657234026,48.447226758583,0
-1.0670596866393,48.447162310083,0
-1.0669455854189,48.447104236841,0
-1.0668242881024,48.447052980807,0
-1.0666967178057,48.447008932049,0
-1.0665638453755,48.446972425785,0
-1.0664266820013,48.446943739834,0
-1.0662862715215,48.4469230925,0
-1.0661436824809,48.446910640911,0
-1.066,48.446906479826,0
-1.0658563175191,48.446910640911,0
-1.0657137284785,48.4469230925,0
-1.0655733179988,48.446943739834,0
-1.0654361546246,48.446972425785,0
-1.0653032821943,48.447008932049,0
-1.0651757118977,48.447052980807,0
-1.0650544145811,48.447104236841,0
-1.0649403133607,48.447162310083,0
-1.0648342765975,48.447226758583,0

-1.0647371112889,48.447297091873,0
-1.064649556928,48.447372774696,0
-1.0645722798744,48.447453231083,0
-1.064505868283,48.447537848735,0
-1.0644508276265,48.447625983678,0
-1.0644075768472,48.447716965168,0
-1.0643764451676,48.447810100796,0
-1.0643576695824,48.447904681753,0
-1.064351393053,48.447999988227,0
-1.066,48.448,0
</coordinates>
</LinearRing>
</outerBoundaryIs>
</Polygon>
</Placemark>
<Placemark>
<name>46</name>
<styleUrl>#stylehap1</styleUrl>
<Polygon>
<outerBoundaryIs>
<LinearRing>
<coordinates>
-1.064,48.18,0
-1.062360029653,48.179999988337,0
-1.062366267198,48.180095294989,0
-1.0623849385052,48.180189876474,0
-1.0624159015418,48.180283012965,0
-1.0624589207234,48.180373995628,0
-1.0625136687048,48.180462132015,0
-1.06257972887,48.180546751339,0
-1.0626565985011,48.180627209578,0
-1.062743692603,48.180702894374,0
-1.0628403483551,48.180773229698,0
-1.0629458301548,48.180837680233,0
-1.0630593352159,48.180895755449,0
-1.0631799996783,48.180947013334,0
-1.0633069051832,48.180991063765,0
-1.0634390858634,48.181027571473,0
-1.0635755356949,48.181056258596,0
-1.0637152161553,48.181076906793,0
-1.0638570641289,48.18108935891,0
-1.064,48.181093520174,0
-1.064142935871,48.18108935891,0
-1.0642847838447,48.181076906793,0
-1.064424464305,48.181056258596,0
-1.0645609141365,48.181027571473,0
-1.0646930948167,48.180991063765,0
-1.0648200003217,48.180947013334,0
-1.064940664784,48.180895755449,0
-1.0650541698451,48.180837680233,0
-1.0651596516448,48.180773229698,0
-1.0652563073969,48.180702894374,0
-1.0653434014989,48.180627209578,0
-1.0654202711299,48.180546751339,0
-1.0654863312951,48.180462132015,0
-1.0655410792765,48.180373995628,0
-1.0655840984581,48.180283012965,0

-1.0656150614947,48.180189876474,0
-1.065633732802,48.180095294989,0
-1.0656399703469,48.179999988337,0
-1.0656337267274,48.179904681863,0
-1.0656150495301,48.179810100903,0
-1.065584080967,48.179716965271,0
-1.0655410567904,48.179625983775,0
-1.0654863044972,48.179537848825,0
-1.0654202408344,48.179453231166,0
-1.0653433686263,48.17937277477,0
-1.0652562729461,48.179297091938,0
-1.0651596166626,48.179226758639,0
-1.0650541353943,48.179162310129,0
-1.0649406319115,48.179104236877,0
-1.0648199700262,48.179052980835,0
-1.0646930680187,48.179008932068,0
-1.0645608916504,48.178972425798,0
-1.0644244468139,48.178943739842,0
-1.06428477188,48.178923092503,0
-1.0641429297964,48.178910640912,0
-1.064,48.178906479826,0
-1.0638570702035,48.178910640912,0
-1.0637152281199,48.178923092503,0
-1.063575553186,48.178943739842,0
-1.0634391083496,48.178972425798,0
-1.0633069319812,48.179008932068,0
-1.0631800299738,48.179052980835,0
-1.0630593680884,48.179104236877,0
-1.0629458646056,48.179162310129,0
-1.0628403833374,48.179226758639,0
-1.0627437270538,48.179297091938,0
-1.0626566313736,48.17937277477,0
-1.0625797591655,48.179453231166,0
-1.0625136955028,48.179537848825,0
-1.0624589432095,48.179625983775,0
-1.0624159190329,48.179716965271,0
-1.0623849504698,48.179810100903,0
-1.0623662732726,48.179904681863,0
-1.062360029653,48.179999988337,0
-1.064,48.18,0
</coordinates>
</LinearRing>
</outerBoundaryIs>
</Polygon>
</Placemark>
<Placemark>
<name>47</name>
<styleUrl>#stylehap1</styleUrl>
<Polygon>
<outerBoundaryIs>
<LinearRing>
<coordinates>
-1.057,48.141,0
-1.0553612759535,48.140999988353,0
-1.0553675087624,48.141095295004,0
-1.0553861658844,48.14118987649,0
-1.0554171053943,48.14128301298,0

-1.0554600918868,48.141373995642,0
-1.0555147982652,48.141462132028,0
-1.0555808082302,48.141546751351,0
-1.0556576194457,48.141627209589,0
-1.0557446473613,48.141702894384,0
-1.0558412296599,48.141773229706,0
-1.0559466312981,48.14183768024,0
-1.0560600500995,48.141895755454,0
-1.0561806228608,48.141947013338,0
-1.0563074319211,48.141991063768,0
-1.0564395121473,48.142027571475,0
-1.0565758582799,48.142056258597,0
-1.0567154325859,48.142076906794,0
-1.0568571727577,48.142089358911,0
-1.057,48.142093520173,0
-1.0571428272424,48.142089358911,0
-1.0572845674142,48.142076906794,0
-1.0574241417202,48.142056258597,0
-1.0575604878528,48.142027571475,0
-1.0576925680789,48.141991063768,0
-1.0578193771392,48.141947013338,0
-1.0579399499005,48.141895755454,0
-1.058053368702,48.14183768024,0
-1.0581587703401,48.141773229706,0
-1.0582553526387,48.141702894384,0
-1.0583423805543,48.141627209589,0
-1.0584191917698,48.141546751351,0
-1.0584852017348,48.141462132028,0
-1.0585399081133,48.141373995642,0
-1.0585828946057,48.14128301298,0
-1.0586138341157,48.14118987649,0
-1.0586324912376,48.141095295004,0
-1.0586387240465,48.140999988353,0
-1.0586324851759,48.140904681879,0
-1.0586138221765,48.140810100919,0
-1.0585828771518,48.140716965286,0
-1.058539885675,48.140625983789,0
-1.0584851749939,48.140537848838,0
-1.0584191615388,48.140453231178,0
-1.0583423477517,48.140372774781,0
-1.0582553182613,48.140297091947,0
-1.0581587354323,48.140226758647,0
-1.0580533343245,48.140162310135,0
-1.0579399170979,48.140104236883,0
-1.0578193469082,48.140052980839,0
-1.057692541338,48.140008932071,0
-1.0575604654145,48.1399724258,0
-1.0574241242663,48.139943739843,0
-1.057284555475,48.139923092504,0
-1.0571428211807,48.139910640912,0
-1.057,48.139906479826,0
-1.0568571788193,48.139910640912,0
-1.056715444525,48.139923092504,0
-1.0565758757338,48.139943739843,0
-1.0564395345856,48.1399724258,0
-1.0563074586621,48.140008932071,0
-1.0561806530919,48.140052980839,0

-1.0560600829021,48.140104236883,0
-1.0559466656755,48.140162310135,0
-1.0558412645677,48.140226758647,0
-1.0557446817388,48.140297091947,0
-1.0556576522483,48.140372774781,0
-1.0555808384612,48.140453231178,0
-1.0555148250061,48.140537848838,0
-1.0554601143251,48.140625983789,0
-1.0554171228482,48.140716965286,0
-1.0553861778236,48.140810100919,0
-1.0553675148241,48.140904681879,0
-1.0553612759535,48.140999988353,0
-1.057,48.141,0
</coordinates>
</LinearRing>
</outerBoundaryIs>
</Polygon>
</Placemark>
<Placemark>
<name>48</name>
<styleUrl>#stylehap1</styleUrl>
<Polygon>
<outerBoundaryIs>
<LinearRing>
<coordinates>
-1.057,48.142,0
-1.0553612440303,48.141999988353,0
-1.0553674769605,48.142095295004,0
-1.0553861344458,48.142189876489,0
-1.0554170745583,48.14228301298,0
-1.0554600618881,48.142373995641,0
-1.0555147693322,48.142462132028,0
-1.055580780583,48.142546751351,0
-1.0556575932948,48.142627209589,0
-1.0557446229057,48.142702894383,0
-1.0558412070858,48.142773229706,0
-1.0559466107772,48.14283768024,0
-1.0560600317882,48.142895755454,0
-1.0561806068984,48.142947013338,0
-1.0563074184291,48.142991063768,0
-1.0564395012282,48.143027571475,0
-1.0565758500171,48.143056258597,0
-1.0567154270421,48.143076906794,0
-1.0568571699752,48.143089358911,0
-1.057,48.143093520174,0
-1.0571428300249,48.143089358911,0
-1.0572845729579,48.143076906794,0
-1.057424149983,48.143056258597,0
-1.0575604987718,48.143027571475,0
-1.057692581571,48.142991063768,0
-1.0578193931017,48.142947013338,0
-1.0579399682118,48.142895755454,0
-1.0580533892228,48.14283768024,0
-1.0581587929142,48.142773229706,0
-1.0582553770943,48.142702894383,0
-1.0583424067053,48.142627209589,0
-1.058419219417,48.142546751351,0

-1.0584852306678,48.142462132028,0
-1.058539938112,48.142373995641,0
-1.0585829254417,48.14228301298,0
-1.0586138655543,48.142189876489,0
-1.0586325230396,48.142095295004,0
-1.0586387559698,48.141999988353,0
-1.0586325169776,48.141904681878,0
-1.0586138536145,48.141810100918,0
-1.0585829079868,48.141716965286,0
-1.0585399156724,48.141625983789,0
-1.0584852039255,48.141537848838,0
-1.0584191891843,48.141453231178,0
-1.0583423739009,48.14137277478,0
-1.058255342715,48.141297091947,0
-1.0581587580045,48.141226758646,0
-1.0580533548435,48.141162310135,0
-1.0579399354075,48.141104236883,0
-1.057819362869,48.141052980838,0
-1.0576925548286,48.141008932071,0
-1.0575604763323,48.1409724258,0
-1.0574241325281,48.140943739843,0
-1.0572845610181,48.140923092504,0
-1.0571428239628,48.140910640912,0
-1.057,48.140906479826,0
-1.0568571760372,48.140910640912,0
-1.0567154389819,48.140923092504,0
-1.0565758674719,48.140943739843,0
-1.0564395236678,48.1409724258,0
-1.0563074451714,48.141008932071,0
-1.056180637131,48.141052980838,0
-1.0560600645926,48.141104236883,0
-1.0559466451566,48.141162310135,0
-1.0558412419955,48.141226758646,0
-1.0557446572851,48.141297091947,0
-1.0556576260992,48.14137277478,0
-1.0555808108157,48.141453231178,0
-1.0555147960746,48.141537848838,0
-1.0554600843276,48.141625983789,0
-1.0554170920132,48.141716965286,0
-1.0553861463856,48.141810100918,0
-1.0553674830225,48.141904681878,0
-1.0553612440303,48.141999988353,0
-1.057,48.142,0
</coordinates>
</LinearRing>
</outerBoundaryIs>
</Polygon>
</Placemark>
<Placemark>
<name>52</name>
<styleUrl>#stylehap1</styleUrl>
<Polygon>
<outerBoundaryIs>
<LinearRing>
<coordinates>
-1.048,47.873,0
-1.0463697689546,47.872999988462,0

-1.046375969489,47.873095295113,0
-1.0463945299442,47.873189876595,0
-1.0464253091299,47.873283013082,0
-1.0464680728598,47.873373995738,0
-1.0465224957321,47.873462132118,0
-1.046588163604,47.873546751433,0
-1.0466645767427,47.873627209662,0
-1.0467511536268,47.873702894448,0
-1.0468472353716,47.873773229761,0
-1.0469520907431,47.873837680285,0
-1.0470649217226,47.87389575549,0
-1.0471848695805,47.873947013365,0
-1.0473110214123,47.873991063788,0
-1.0474424170873,47.874027571488,0
-1.0475780565568,47.874056258604,0
-1.0477169074666,47.874076906797,0
-1.0478579130154,47.874089358911,0
-1.048,47.874093520174,0
-1.0481420869846,47.874089358911,0
-1.0482830925334,47.874076906797,0
-1.0484219434432,47.874056258604,0
-1.0485575829127,47.874027571488,0
-1.0486889785877,47.873991063788,0
-1.0488151304195,47.873947013365,0
-1.0489350782774,47.87389575549,0
-1.0490479092569,47.873837680285,0
-1.0491527646284,47.873773229761,0
-1.0492488463732,47.873702894448,0
-1.0493354232574,47.873627209662,0
-1.049411836396,47.873546751433,0
-1.0494775042679,47.873462132118,0
-1.0495319271402,47.873373995738,0
-1.0495746908701,47.873283013082,0
-1.0496054700558,47.873189876595,0
-1.049624030511,47.873095295113,0
-1.0496302310454,47.872999988462,0
-1.0496240245372,47.872904681987,0
-1.0496054582897,47.872810101025,0
-1.0495746736692,47.872716965388,0
-1.0495319050272,47.872625983885,0
-1.0494774779147,47.872537848928,0
-1.0494118066032,47.87245323126,0
-1.0493353909303,47.872372774854,0
-1.0492488124942,47.872297092011,0
-1.0491527302266,47.872226758701,0
-1.0490478753778,47.872162310181,0
-1.0489350459504,47.872104236919,0
-1.0488151006268,47.872052980866,0
-1.0486889522344,47.872008932091,0
-1.0485575607997,47.871972425813,0
-1.0484219262423,47.87194373985,0
-1.0482830807674,47.871923092507,0
-1.0481420810108,47.871910640913,0
-1.048,47.871906479826,0
-1.0478579189892,47.871910640913,0
-1.0477169192326,47.871923092507,0
-1.0475780737577,47.87194373985,0

-1.0474424392004,47.871972425813,0
-1.0473110477656,47.872008932091,0
-1.0471848993733,47.872052980866,0
-1.0470649540496,47.872104236919,0
-1.0469521246222,47.872162310181,0
-1.0468472697734,47.872226758701,0
-1.0467511875058,47.872297092011,0
-1.0466646090697,47.872372774854,0
-1.0465881933968,47.87245323126,0
-1.0465225220853,47.872537848928,0
-1.0464680949728,47.872625983885,0
-1.0464253263308,47.872716965388,0
-1.0463945417103,47.872810101025,0
-1.0463759754628,47.872904681987,0
-1.0463697689546,47.872999988462,0
-1.048,47.873,0
</coordinates>
</LinearRing>
</outerBoundaryIs>
</Polygon>
</Placemark>
<Placemark>
<name>53</name>
<styleUrl>#stylehap1</styleUrl>
<Polygon>
<outerBoundaryIs>
<LinearRing>
<coordinates>
-1.048,48.14,0
-1.046361307875,48.139999988353,0
-1.0463675405626,48.140095295005,0
-1.0463861973213,48.14018987649,0
-1.0464171362286,48.140283012981,0
-1.0464601218838,48.140373995642,0
-1.0465148271967,48.140462132028,0
-1.0465808358759,48.140546751352,0
-1.0466576455952,48.140627209589,0
-1.0467446718155,48.140702894384,0
-1.0468412522328,48.140773229707,0
-1.0469466518178,48.14083768024,0
-1.0470600684098,48.140895755454,0
-1.0471806388224,48.140947013338,0
-1.0473074454125,48.140991063768,0
-1.0474395230657,48.141027571475,0
-1.0475758665422,48.141056258597,0
-1.0477154381293,48.141076906794,0
-1.0478571755399,48.141089358911,0
-1.048,48.141093520173,0
-1.0481428244601,48.141089358911,0
-1.0482845618707,48.141076906794,0
-1.0484241334578,48.141056258597,0
-1.0485604769343,48.141027571475,0
-1.0486925545875,48.140991063768,0
-1.0488193611776,48.140947013338,0
-1.0489399315902,48.140895755454,0
-1.0490533481822,48.14083768024,0
-1.0491587477672,48.140773229707,0

-1.0492553281845,48.140702894384,0
-1.0493423544048,48.140627209589,0
-1.0494191641241,48.140546751352,0
-1.0494851728033,48.140462132028,0
-1.0495398781162,48.140373995642,0
-1.0495828637714,48.140283012981,0
-1.0496138026787,48.14018987649,0
-1.0496324594374,48.140095295005,0
-1.0496386921249,48.139999988353,0
-1.049632453376,48.139904681879,0
-1.0496137907402,48.139810100919,0
-1.0495828463184,48.139716965287,0
-1.0495398556791,48.139625983789,0
-1.0494851460639,48.139537848839,0
-1.0494191338947,48.139453231178,0
-1.049342321604,48.139372774781,0
-1.0492552938089,48.139297091947,0
-1.0491587128613,48.139226758647,0
-1.0490533138067,48.139162310136,0
-1.0489398987893,48.139104236883,0
-1.0488193309482,48.139052980839,0
-1.0486925278481,48.139008932071,0
-1.0485604544973,48.1389724258,0
-1.0484241160048,48.138943739843,0
-1.0482845499322,48.138923092504,0
-1.0481428183987,48.138910640912,0
-1.048,48.138906479826,0
-1.0478571816013,48.138910640912,0
-1.0477154500678,48.138923092504,0
-1.0475758839952,48.138943739843,0
-1.0474395455027,48.1389724258,0
-1.0473074721519,48.139008932071,0
-1.0471806690518,48.139052980839,0
-1.0470601012106,48.139104236883,0
-1.0469466861933,48.139162310136,0
-1.0468412871387,48.139226758647,0
-1.0467447061911,48.139297091947,0
-1.046657678396,48.139372774781,0
-1.0465808661053,48.139453231178,0
-1.0465148539362,48.139537848839,0
-1.0464601443209,48.139625983789,0
-1.0464171536816,48.139716965287,0
-1.0463862092598,48.139810100919,0
-1.046367546624,48.139904681879,0
-1.046361307875,48.139999988353,0
-1.048,48.14,0
</coordinates>
</LinearRing>
</outerBoundaryIs>
</Polygon>
</Placemark>
<Placemark>
<name>55</name>
<styleUrl>#stylehap1</styleUrl>
<Polygon>
<outerBoundaryIs>
<LinearRing>

<coordinates>
-1.046,48.233,0
-1.0443583317005,48.232999988315,0
-1.0443645756979,48.233095294967,0
-1.0443832663311,48.233189876453,0
-1.0444142614204,48.233283012945,0
-1.0444573251374,48.233373995609,0
-1.0445121297985,48.233462131997,0
-1.0445782583561,48.233546751323,0
-1.0446552075722,48.233627209564,0
-1.0447423918461,48.233702894361,0
-1.0448391476707,48.233773229688,0
-1.0449447386821,48.233837680224,0
-1.0450583612627,48.233895755441,0
-1.0451791506581,48.233947013329,0
-1.0453061875587,48.233991063762,0
-1.0454385050969,48.234027571471,0
-1.0455750962072,48.234056258594,0
-1.0457149212916,48.234076906793,0
-1.0458569161339,48.23408935891,0
-1.046,48.234093520174,0
-1.0461430838662,48.23408935891,0
-1.0462850787085,48.234076906793,0
-1.0464249037929,48.234056258594,0
-1.0465614949032,48.234027571471,0
-1.0466938124414,48.233991063762,0
-1.0468208493419,48.233947013329,0
-1.0469416387374,48.233895755441,0
-1.047055261318,48.233837680224,0
-1.0471608523294,48.233773229688,0
-1.0472576081541,48.233702894361,0
-1.0473447924279,48.233627209564,0
-1.0474217416439,48.233546751323,0
-1.0474878702016,48.233462131997,0
-1.0475426748627,48.233373995609,0
-1.0475857385797,48.233283012945,0
-1.047616733669,48.233189876453,0
-1.0476354243021,48.233095294967,0
-1.0476416682995,48.232999988315,0
-1.0476354182099,48.232904681841,0
-1.0476167216696,48.232810100882,0
-1.0475857210379,48.232716965251,0
-1.0475426523113,48.232625983756,0
-1.047487843326,48.232537848807,0
-1.0474217112606,48.23245323115,0
-1.04734475946,48.232372774755,0
-1.0472575736033,48.232297091925,0
-1.0471608172457,48.232226758628,0
-1.0470552267673,48.23216231012,0
-1.0469416057695,48.23210423687,0
-1.0468208189586,48.232052980829,0
-1.0466937855657,48.232008932065,0
-1.0465614723518,48.231972425795,0
-1.0464248862511,48.23194373984,0
-1.0462850667091,48.231923092503,0
-1.046143077774,48.231910640912,0
-1.046,48.231906479826,0

-1.0458569222261,48.231910640912,0
-1.045714933291,48.231923092503,0
-1.045575113749,48.23194373984,0
-1.0454385276483,48.231972425795,0
-1.0453062144344,48.232008932065,0
-1.0451791810415,48.232052980829,0
-1.0450583942306,48.23210423687,0
-1.0449447732328,48.23216231012,0
-1.0448391827544,48.232226758628,0
-1.0447424263967,48.232297091925,0
-1.0446552405401,48.232372774755,0
-1.0445782887395,48.23245323115,0
-1.0445121566741,48.232537848807,0
-1.0444573476888,48.232625983756,0
-1.0444142789622,48.232716965251,0
-1.0443832783305,48.232810100882,0
-1.0443645817902,48.232904681841,0
-1.0443583317005,48.232999988315,0
-1.046,48.233,0
</coordinates>
</LinearRing>
</outerBoundaryIs>
</Polygon>
</Placemark>
<Placemark>
<name>56</name>
<styleUrl>#stylehap1</styleUrl>
<Polygon>
<outerBoundaryIs>
<LinearRing>
<coordinates>
-1.046,47.874,0
-1.0443697374942,47.873999988462,0
-1.0443759381481,47.874095295112,0
-1.0443944989614,47.874189876595,0
-1.044425278741,47.874283013082,0
-1.0444680432961,47.874373995738,0
-1.0445224672185,47.874462132117,0
-1.0445881363577,47.874546751433,0
-1.0446645509709,47.874627209662,0
-1.0447511295257,47.874702894447,0
-1.0448472131248,47.874773229761,0
-1.0449520705198,47.874837680285,0
-1.0450649036767,47.87489575549,0
-1.0451848538495,47.874947013365,0
-1.0453110081159,47.874991063788,0
-1.0454424063267,47.875027571488,0
-1.0455780484138,47.875056258604,0
-1.0457169020032,47.875076906797,0
-1.0458579102733,47.875089358911,0
-1.046,47.875093520174,0
-1.0461420897268,47.875089358911,0
-1.0462830979969,47.875076906797,0
-1.0464219515863,47.875056258604,0
-1.0465575936734,47.875027571488,0
-1.0466889918842,47.874991063788,0
-1.0468151461506,47.874947013365,0

-1.0469350963234,47.87489575549,0
-1.0470479294803,47.874837680285,0
-1.0471527868753,47.874773229761,0
-1.0472488704744,47.874702894447,0
-1.0473354490292,47.874627209662,0
-1.0474118636425,47.874546751433,0
-1.0474775327816,47.874462132117,0
-1.047531956704,47.874373995738,0
-1.0475747212591,47.874283013082,0
-1.0476055010387,47.874189876595,0
-1.047624061852,47.874095295112,0
-1.0476302625059,47.873999988462,0
-1.0476240558779,47.873904681986,0
-1.047605489272,47.873810101024,0
-1.0475747040573,47.873716965388,0
-1.0475319345898,47.873625983885,0
-1.0474775064269,47.873537848928,0
-1.047411833848,47.87345323126,0
-1.0473354167004,47.873372774854,0
-1.0472488365934,47.873297092011,0
-1.0471527524717,47.873226758701,0
-1.0470478955994,47.87316231018,0
-1.0469350639945,47.873104236918,0
-1.0468151163562,47.873052980866,0
-1.0466889655295,47.873008932091,0
-1.0465575715592,47.872972425813,0
-1.0464219343845,47.87294373985,0
-1.0462830862302,47.872923092507,0
-1.0461420837527,47.872910640913,0
-1.046,47.872906479827,0
-1.0458579162474,47.872910640913,0
-1.0457169137699,47.872923092507,0
-1.0455780656156,47.87294373985,0
-1.0454424284409,47.872972425813,0
-1.0453110344706,47.873008932091,0
-1.0451848836439,47.873052980866,0
-1.0450649360056,47.873104236918,0
-1.0449521044008,47.87316231018,0
-1.0448472475284,47.873226758701,0
-1.0447511634067,47.873297092011,0
-1.0446645832997,47.873372774854,0
-1.0445881661521,47.87345323126,0
-1.0445224935732,47.873537848928,0
-1.0444680654103,47.873625983885,0
-1.0444252959428,47.873716965388,0
-1.0443945107281,47.873810101024,0
-1.0443759441222,47.873904681986,0
-1.0443697374942,47.873999988462,0
-1.046,47.874,0
</coordinates>
</LinearRing>
</outerBoundaryIs>
</Polygon>
</Placemark>
<Placemark>
<name>57</name>
<styleUrl>#stylehap1</styleUrl>

<Polygon>
<outerBoundaryIs>
<LinearRing>
<coordinates>
-1.0409999999999,47.859,0
-1.0393702092218,47.858999988468,0
-1.0393764080831,47.859095295118,0
-1.0393949635272,47.859189876601,0
-1.0394257344019,47.859283013087,0
-1.039468486584,47.859373995743,0
-1.0395228947597,47.859462132122,0
-1.0395885448979,47.859546751437,0
-1.0396649374007,47.859627209666,0
-1.0397514909039,47.859702894451,0
-1.0398475467006,47.859773229764,0
-1.0399523737543,47.859837680287,0
-1.0400651742617,47.859895755492,0
-1.0401850897254,47.859947013367,0
-1.0403112074873,47.859991063789,0
-1.040442567676,47.860027571489,0
-1.0405781705129,47.860056258605,0
-1.0407169839226,47.860076906797,0
-1.0408579513894,47.860089358912,0
-1.0409999999999,47.860093520174,0
-1.0411420486105,47.860089358912,0
-1.0412830160773,47.860076906797,0
-1.041421829487,47.860056258605,0
-1.0415574323239,47.860027571489,0
-1.0416887925126,47.859991063789,0
-1.0418149102745,47.859947013367,0
-1.0419348257382,47.859895755492,0
-1.0420476262456,47.859837680287,0
-1.0421524532993,47.859773229764,0
-1.042248509096,47.859702894451,0
-1.0423350625992,47.859627209666,0
-1.042411455102,47.859546751437,0
-1.0424771052402,47.859462132122,0
-1.0425315134158,47.859373995743,0
-1.042574265598,47.859283013087,0
-1.0426050364726,47.859189876601,0
-1.0426235919168,47.859095295118,0
-1.0426297907781,47.858999988468,0
-1.0426235859475,47.858904681992,0
-1.0426050247155,47.85881010103,0
-1.0425742484102,47.858716965393,0
-1.0425314913196,47.85862598389,0
-1.042477078907,47.858537848933,0
-1.0424114253319,47.858453231264,0
-1.0423350302967,47.858372774858,0
-1.0422484752427,47.858297092014,0
-1.0421524189237,47.858226758704,0
-1.0420475923923,47.858162310183,0
-1.0419347934357,47.85810423692,0
-1.0418148805044,47.858052980867,0
-1.0416887661794,47.858008932092,0
-1.0415574102277,47.857972425813,0
-1.0414218122992,47.85794373985,0

-1.0412830043202,47.857923092507,0
-1.0411420426412,47.857910640913,0
-1.0409999999999,47.857906479827,0
-1.0408579573587,47.857910640913,0
-1.0407169956797,47.857923092507,0
-1.0405781877006,47.85794373985,0
-1.0404425897721,47.857972425813,0
-1.0403112338205,47.858008932092,0
-1.0401851194955,47.858052980867,0
-1.0400652065642,47.85810423692,0
-1.0399524076076,47.858162310183,0
-1.0398475810762,47.858226758704,0
-1.0397515247572,47.858297092014,0
-1.0396649697032,47.858372774858,0
-1.039588574668,47.858453231264,0
-1.0395229210929,47.858537848933,0
-1.0394685086803,47.85862598389,0
-1.0394257515897,47.858716965393,0
-1.0393949752844,47.85881010103,0
-1.0393764140524,47.858904681992,0
-1.0393702092218,47.858999988468,0
-1.0409999999999,47.859,0
</coordinates>
</LinearRing>
</outerBoundaryIs>
</Polygon>
</Placemark>
<Placemark>
<name>60</name>
<styleUrl>#stylehap1</styleUrl>
<Polygon>
<outerBoundaryIs>
<LinearRing>
<coordinates>
-1.029,47.959,0
-1.0273670570871,47.958999988427,0
-1.027373267927,47.959095295078,0
-1.0273918592485,47.959189876561,0
-1.0274226896268,47.959283013049,0
-1.0274655244861,47.959373995707,0
-1.0275200378837,47.959462132089,0
-1.027585814988,47.959546751407,0
-1.0276623552351,47.959627209639,0
-1.0277490761363,47.959702894427,0
-1.0278453177111,47.959773229744,0
-1.0279503475089,47.959837680271,0
-1.0280633661835,47.959895755478,0
-1.0281835135769,47.959947013357,0
-1.0283098752658,47.959991063782,0
-1.0284414895222,47.960027571484,0
-1.0285773546335,47.960056258602,0
-1.0287164365281,47.960076906796,0
-1.0288576766465,47.960089358911,0
-1.029,47.960093520174,0
-1.0291423233534,47.960089358911,0
-1.0292835634719,47.960076906796,0
-1.0294226453665,47.960056258602,0

-1.0295585104778,47.960027571484,0
-1.0296901247342,47.959991063782,0
-1.0298164864231,47.959947013357,0
-1.0299366338165,47.959895755478,0
-1.0300496524912,47.959837680271,0
-1.0301546822889,47.959773229744,0
-1.0302509238637,47.959702894427,0
-1.0303376447649,47.959627209639,0
-1.030414185012,47.959546751407,0
-1.0304799621163,47.959462132089,0
-1.0305344755139,47.959373995707,0
-1.0305773103732,47.959283013049,0
-1.0306081407515,47.959189876561,0
-1.030626732073,47.959095295078,0
-1.0306329429128,47.958999988427,0
-1.0306267260712,47.958904681952,0
-1.0306081289302,47.958810100991,0
-1.0305772930917,47.958716965355,0
-1.0305344532971,47.958625983855,0
-1.0304799356394,47.958537848899,0
-1.0304141550794,47.958453231234,0
-1.0303376122862,47.95837277483,0
-1.0302508898257,47.95829709199,0
-1.0301546477258,47.958226758684,0
-1.0300496184531,47.958162310166,0
-1.0299366013378,47.958104236907,0
-1.0298164564906,47.958052980857,0
-1.0296900982573,47.958008932085,0
-1.0295584882611,47.957972425809,0
-1.0294226280849,47.957943739848,0
-1.0292835516506,47.957923092506,0
-1.0291423173516,47.957910640913,0
-1.029,47.957906479826,0
-1.0288576826484,47.957910640913,0
-1.0287164483494,47.957923092506,0
-1.0285773719151,47.957943739848,0
-1.0284415117389,47.957972425809,0
-1.0283099017427,47.958008932085,0
-1.0281835435094,47.958052980857,0
-1.0280633986622,47.958104236907,0
-1.0279503815469,47.958162310166,0
-1.0278453522742,47.958226758684,0
-1.0277491101743,47.95829709199,0
-1.0276623877138,47.95837277483,0
-1.0275858449206,47.958453231234,0
-1.0275200643605,47.958537848899,0
-1.0274655467029,47.958625983855,0
-1.0274227069083,47.958716965355,0
-1.0273918710698,47.958810100991,0
-1.0273732739288,47.958904681952,0
-1.0273670570871,47.958999988427,0
-1.029,47.959,0
</coordinates>
</LinearRing>
</outerBoundaryIs>
</Polygon>
</Placemark>

<Placemark>
<name>61</name>
<styleUrl>#stylehap1</styleUrl>
<Polygon>
<outerBoundaryIs>
<LinearRing>
<coordinates>
-1.023,47.908,0
-1.0213686668188,47.907999988448,0
-1.0213748715414,47.908095295099,0
-1.0213934445411,47.908189876582,0
-1.0214242445321,47.908283013069,0
-1.0214670371699,47.908373995726,0
-1.0215214968327,47.908462132106,0
-1.021587209098,47.908546751423,0
-1.021663673895,47.908627209653,0
-1.0217503093095,47.908702894439,0
-1.0218464560111,47.908773229754,0
-1.0219513822716,47.908837680279,0
-1.0220642895326,47.908895755485,0
-1.0221843184842,47.908947013362,0
-1.0223105556045,47.908991063785,0
-1.0224420401136,47.909027571486,0
-1.0225777712867,47.909056258603,0
-1.0227167160715,47.909076906797,0
-1.0228578169522,47.909089358911,0
-1.023,47.909093520174,0
-1.0231421830478,47.909089358911,0
-1.0232832839286,47.909076906797,0
-1.0234222287133,47.909056258603,0
-1.0235579598864,47.909027571486,0
-1.0236894443955,47.908991063785,0
-1.0238156815159,47.908947013362,0
-1.0239357104674,47.908895755485,0
-1.0240486177285,47.908837680279,0
-1.0241535439889,47.908773229754,0
-1.0242496906906,47.908702894439,0
-1.024336326105,47.908627209653,0
-1.024412790902,47.908546751423,0
-1.0244785031674,47.908462132106,0
-1.0245329628302,47.908373995726,0
-1.024575755468,47.908283013069,0
-1.0246065554589,47.908189876582,0
-1.0246251284586,47.908095295099,0
-1.0246313331813,47.907999988448,0
-1.0246251224734,47.907904681973,0
-1.0246065436704,47.907810101011,0
-1.0245757382343,47.907716965375,0
-1.024532940675,47.907625983873,0
-1.0244784767639,47.907537848916,0
-1.0244127610525,47.907453231249,0
-1.0243362937163,47.907372774844,0
-1.0242496567469,47.907297092003,0
-1.0241535095216,47.907226758694,0
-1.0240485837848,47.907162310175,0
-1.0239356780787,47.907104236914,0
-1.0238156516663,47.907052980862,0

-1.0236894179921,47.907008932088,0
-1.0235579377313,47.906972425811,0
-1.0234222114797,47.906943739849,0
-1.0232832721401,47.906923092507,0
-1.0231421770626,47.906910640913,0
-1.023,47.906906479827,0
-1.0228578229374,47.906910640913,0
-1.02271672786,47.906923092507,0
-1.0225777885203,47.906943739849,0
-1.0224420622688,47.906972425811,0
-1.022310582008,47.907008932088,0
-1.0221843483337,47.907052980862,0
-1.0220643219213,47.907104236914,0
-1.0219514162152,47.907162310175,0
-1.0218464904784,47.907226758694,0
-1.0217503432531,47.907297092003,0
-1.0216637062837,47.907372774844,0
-1.0215872389476,47.907453231249,0
-1.0215215232362,47.907537848916,0
-1.021467059325,47.907625983873,0
-1.0214242617657,47.907716965375,0
-1.0213934563296,47.907810101011,0
-1.0213748775266,47.907904681973,0
-1.0213686668188,47.907999988448,0
-1.023,47.908,0
</coordinates>
</LinearRing>
</outerBoundaryIs>
</Polygon>
</Placemark>
<Placemark>
<name>62</name>
<styleUrl>#stylehap1</styleUrl>
<Polygon>
<outerBoundaryIs>
<LinearRing>
<coordinates>
-1.015,48.164,0
-1.0133605412769,48.163999988344,0
-1.0133667768777,48.164095294995,0
-1.0133854423617,48.16418987648,0
-1.0134163957402,48.164283012971,0
-1.0134594015025,48.164373995634,0
-1.0135141324053,48.16446213202,0
-1.0135801719626,48.164546751344,0
-1.0136570176132,48.164627209583,0
-1.0137440845447,48.164702894378,0
-1.0138407101432,48.164773229702,0
-1.0139461590354,48.164837680236,0
-1.0140596286857,48.164895755451,0
-1.0141802555035,48.164947013336,0
-1.0143071214166,48.164991063767,0
-1.014439260859,48.165027571474,0
-1.0145756681206,48.165056258596,0
-1.0147153050031,48.165076906793,0
-1.0148571087225,48.165089358911,0
-1.015,48.165093520174,0

-1.0151428912774,48.165089358911,0
-1.0152846949969,48.165076906793,0
-1.0154243318794,48.165056258596,0
-1.015560739141,48.165027571474,0
-1.0156928785834,48.164991063767,0
-1.0158197444965,48.164947013336,0
-1.0159403713143,48.164895755451,0
-1.0160538409645,48.164837680236,0
-1.0161592898568,48.164773229702,0
-1.0162559154552,48.164702894378,0
-1.0163429823867,48.164627209583,0
-1.0164198280374,48.164546751344,0
-1.0164858675947,48.16446213202,0
-1.0165405984975,48.164373995634,0
-1.0165836042597,48.164283012971,0
-1.0166145576383,48.16418987648,0
-1.0166332231223,48.164095294995,0
-1.016639458723,48.163999988344,0
-1.016633217053,48.163904681869,0
-1.0166145456841,48.16381010091,0
-1.0165835867839,48.163716965277,0
-1.016540576031,48.163625983781,0
-1.0164858408201,48.163537848831,0
-1.0164197977684,48.163453231171,0
-1.0163429495429,48.163372774774,0
-1.0162558810346,48.163297091941,0
-1.0161592549051,48.163226758642,0
-1.0160538065439,48.163162310132,0
-1.0159403384704,48.16310423688,0
-1.0158197142274,48.163052980836,0
-1.0156928518089,48.16300893207,0
-1.0155607166745,48.162972425799,0
-1.0154243144035,48.162943739842,0
-1.0152846830427,48.162923092504,0
-1.0151428852082,48.162910640912,0
-1.015,48.162906479826,0
-1.0148571147918,48.162910640912,0
-1.0147153169572,48.162923092504,0
-1.0145756855964,48.162943739842,0
-1.0144392833254,48.162972425799,0
-1.0143071481911,48.16300893207,0
-1.0141802857725,48.163052980836,0
-1.0140596615295,48.16310423688,0
-1.0139461934561,48.163162310132,0
-1.0138407450948,48.163226758642,0
-1.0137441189654,48.163297091941,0
-1.0136570504571,48.163372774774,0
-1.0135802022316,48.163453231171,0
-1.0135141591798,48.163537848831,0
-1.013459423969,48.163625983781,0
-1.0134164132161,48.163716965277,0
-1.0133854543158,48.16381010091,0
-1.013366782947,48.163904681869,0
-1.0133605412769,48.163999988344,0
-1.015,48.164,0
</coordinates>
</LinearRing>

</outerBoundaryIs>
</Polygon>
</Placemark>
<Placemark>
<name>63</name>
<styleUrl>#stylehap1</styleUrl>
<Polygon>
<outerBoundaryIs>
<LinearRing>
<coordinates>
-1.009,48.268,0
-1.0073572077109,48.267999988301,0
-1.0073634559796,48.268095294953,0
-1.0073821594059,48.268189876439,0
-1.007413175713,48.268283012932,0
-1.0074562689111,48.268373995596,0
-1.0075111110922,48.268462131985,0
-1.0075772849235,48.268546751312,0
-1.0076542868222,48.268627209554,0
-1.0077415307869,48.268702894353,0
-1.0078383528565,48.26877322968,0
-1.0079440161624,48.268837680218,0
-1.0080577165372,48.268895755437,0
-1.0081785886342,48.268947013325,0
-1.0083057125144,48.268991063759,0
-1.0084381206482,48.269027571469,0
-1.0085748052804,48.269056258593,0
-1.0087147261014,48.269076906792,0
-1.0088568181658,48.26908935891,0
-1.009,48.269093520174,0
-1.0091431818342,48.26908935891,0
-1.0092852738987,48.269076906792,0
-1.0094251947196,48.269056258593,0
-1.0095618793518,48.269027571469,0
-1.0096942874856,48.268991063759,0
-1.0098214113658,48.268947013325,0
-1.0099422834629,48.268895755437,0
-1.0100559838376,48.268837680218,0
-1.0101616471436,48.26877322968,0
-1.0102584692131,48.268702894353,0
-1.0103457131778,48.268627209554,0
-1.0104227150766,48.268546751312,0
-1.0104888889079,48.268462131985,0
-1.010543731089,48.268373995596,0
-1.0105868242871,48.268283012932,0
-1.0106178405941,48.268189876439,0
-1.0106365440204,48.268095294953,0
-1.0106427922891,48.267999988301,0
-1.0106365379165,48.267904681827,0
-1.0106178285718,48.267810100868,0
-1.0105868067116,48.267716965238,0
-1.0105437084944,48.267625983743,0
-1.0104888619807,48.267537848796,0
-1.010422684635,48.267453231139,0
-1.0103456801468,48.267372774746,0
-1.0102584345962,48.267297091916,0
-1.0101616119927,48.267226758621,0

-1.0100559492207,48.267162310114,0
-1.0099422504318,48.267104236866,0
-1.0098213809242,48.267052980825,0
-1.0096942605585,48.267008932062,0
-1.0095618567573,48.266972425794,0
-1.0094251771441,48.266943739839,0
-1.0092852618763,48.266923092502,0
-1.0091431757303,48.266910640912,0
-1.009,48.266906479826,0
-1.0088568242697,48.266910640912,0
-1.0087147381237,48.266923092502,0
-1.0085748228559,48.266943739839,0
-1.0084381432428,48.266972425794,0
-1.0083057394416,48.267008932062,0
-1.0081786190758,48.267052980825,0
-1.0080577495682,48.267104236866,0
-1.0079440507793,48.267162310114,0
-1.0078383880074,48.267226758621,0
-1.0077415654038,48.267297091916,0
-1.0076543198533,48.267372774746,0
-1.0075773153651,48.267453231139,0
-1.0075111380193,48.267537848796,0
-1.0074562915056,48.267625983743,0
-1.0074131932884,48.267716965238,0
-1.0073821714283,48.267810100868,0
-1.0073634620835,48.267904681827,0
-1.0073572077109,48.267999988301,0
-1.009,48.268,0
</coordinates>
</LinearRing>
</outerBoundaryIs>
</Polygon>
</Placemark>
<Placemark>
<name>64</name>
<styleUrl>#stylehap1</styleUrl>
<Polygon>
<outerBoundaryIs>
<LinearRing>
<coordinates>
-1,48.113,0
-0.99836216909813,48.112999988364,0
-0.99836839851297,48.113095295016,0
-0.99838704546921,48.113189876501,0
-0.99841796811908,48.113283012991,0
-0.99846093118527,48.113373995652,0
-0.99851560774954,48.113462132037,0
-0.99858158173918,48.11354675136,0
-0.99865835109197,48.113627209597,0
-0.99874533157599,48.11370289439,0
-0.99884186123519,48.113773229712,0
-0.99894720542658,48.113837680245,0
-0.99906056241126,48.113895755458,0
-0.99918106945617,48.113947013341,0
-0.99930780940078,48.11399106377,0
-0.99943981763782,48.114027571477,0
-0.99957608945602,48.114056258598,0

-0.99971558768789,48.114076906794,0
-0.99985725060494,48.114089358911,0
-0.99999999999999,48.114093520174,0
-1.0001427493951,48.114089358911,0
-1.0002844123121,48.114076906794,0
-1.000423910544,48.114056258598,0
-1.0005601823622,48.114027571477,0
-1.0006921905992,48.11399106377,0
-1.0008189305438,48.113947013341,0
-1.0009394375887,48.113895755458,0
-1.0010527945734,48.113837680245,0
-1.0011581387648,48.113773229712,0
-1.001254668424,48.11370289439,0
-1.001341648908,48.113627209597,0
-1.0014184182608,48.11354675136,0
-1.0014843922505,48.113462132037,0
-1.0015390688147,48.113373995652,0
-1.0015820318809,48.113283012991,0
-1.0016129545308,48.113189876501,0
-1.001631601487,48.113095295016,0
-1.0016378309019,48.112999988364,0
-1.0016315954346,48.11290468189,0
-1.0016129426098,48.11281010093,0
-1.0015820144537,48.112716965297,0
-1.0015390464107,48.112625983799,0
-1.0014843655504,48.112537848848,0
-1.0014183880759,48.112453231187,0
-1.0013416161555,48.112372774788,0
-1.001254634099,48.112297091954,0
-1.0011581039103,48.112226758652,0
-1.0010527602484,48.11216231014,0
-1.0009394048363,48.112104236887,0
-1.0008189003589,48.112052980841,0
-1.0006921638992,48.112008932073,0
-1.0005601599582,48.111972425801,0
-1.0004238931167,48.111943739843,0
-1.0002844003912,48.111923092504,0
-1.0001427433426,48.111910640913,0
-0.99999999999999,48.111906479827,0
-0.99985725665736,48.111910640913,0
-0.99971559960884,48.111923092504,0
-0.99957610688327,48.111943739843,0
-0.99943984004185,48.111972425801,0
-0.99930783610085,48.112008932073,0
-0.99918109964106,48.112052980841,0
-0.99906059516375,48.112104236887,0
-0.99894723975156,48.11216231014,0
-0.99884189608967,48.112226758652,0
-0.99874536590095,48.112297091954,0
-0.99865838384445,48.112372774788,0
-0.99858161192404,48.112453231187,0
-0.99851563444964,48.112537848848,0
-0.99846095358931,48.112625983799,0
-0.99841798554633,48.112716965297,0
-0.99838705739016,48.11281010093,0
-0.9983684045653 8,48.11290468189,0
-0.99836216909813,48.112999988364,0

-1,48.113,0
</coordinates>
</LinearRing>
</outerBoundaryIs>
</Polygon>
</Placemark>
<Placemark>
<name>65</name>
<styleUrl>#stylehap1</styleUrl>
<Polygon>
<outerBoundaryIs>
<LinearRing>
<coordinates>
-0.99900000000002,48.452,0
-0.99735126318578,48.451999988225,0
-0.99735753404426,48.452095294877,0
-0.9973763051306,48.452189876365,0
-0.99740743365358,48.452283012861,0
-0.99745068277022,48.452373995529,0
-0.99750572338634,48.452462131923,0
-0.99757213665948,48.452546751255,0
-0.99764941718499,48.452627209503,0
-0.9977369768411З,48.452702894309,0
-0.99783414926435,48.452773229642,0
-0.99794019491995,48.452837680187,0
-0.99805430673033,48.452895755412,0
-0.99817561621746,48.452947013306,0
-0.99830320011312,48.452991063745,0
-0.99843608738641,48.45302757146,0
-0.9985732666351,48.453056258588,0
-0.99871369378451,48.45307690679,0
-0.99885630003541,48.45308935891,0
-0.99900000000003,48.453093520174,0
-0.99914369996463,48.45308935891,0
-0.99928630621553,48.45307690679,0
-0.99942673336495,48.453056258588,0
-0.99956391261364,48.45302757146,0
-0.99969679988692,48.452991063745,0
-0.99982438378258,48.452947013306,0
-0.99994569326973,48.452895755412,0
-1.0000598050801,48.452837680187,0
-1.0001658507357,48.452773229642,0
-1.0002630231589,48.452702894309,0
-1.0003505828151,48.452627209503,0
-1.0004278633405,48.452546751255,0
-1.0004942766137,48.452462131923,0
-1.0005493172298,48.452373995529,0
-1.0005925663465,48.452283012861,0
-1.0006236948695,48.452189876365,0
-1.0006424659558,48.452095294877,0
-1.0006487368143,48.451999988225,0
-1.0006424597901,48.451904681752,0
-1.0006236827253,48.451810100795,0
-1.000592548593,48.451716965167,0
-1.0005492944064,48.451625983676,0
-1.0004942494138,48.451537848733,0
-1.0004278325906,48.451453231082,0

-1.0003505494494,48.451372774695,0
-1.0002629881913,48.451297091872,0
-1.0001658152287,48.451226758583,0
-1.0000597701125,48.451162310083,0
-0.99994565990406,48.451104236841,0
-0.99982435303262,48.451052980807,0
-0.99969677268696,48.451008932048,0
-0.99956388979016,48.450972425785,0
-0.99942671561145,48.450943739834,0
-0.99928629407143,48.4509230925,0
-0.99914369379891,48.450910640911,0
-0.99900000000003,48.450906479827,0
-0.99885630620115,48.450910640911,0
-0.99871370592863,48.4509230925,0
-0.9985732843886,48.450943739834,0
-0.99843611020987,48.450972425785,0
-0.99830322731307,48.451008932048,0
-0.99817564696744,48.451052980807,0
-0.998054340096,48.451104236841,0
-0.99794022988753,48.451162310083,0
-0.99783418477135,48.451226758583,0
-0.99773701180871,48.451297091872,0
-0.99764945055066,48.451372774695,0
-0.99757216740947,48.451453231082,0
-0.9975057505863,48.451537848733,0
-0.99745070559367,48.451625983676,0
-0.99740745140708,48.451716965167,0
-0.9973763172747,48.451810100795,0
-0.99735754020998,48.451904681752,0
-0.99735126318578,48.451999988225,0
-0.99900000000002,48.452,0
</coordinates>
</LinearRing>
</outerBoundaryIs>
</Polygon>
</Placemark>
<Placemark>
<name>66</name>
<styleUrl>#stylehap1</styleUrl>
<Polygon>
<outerBoundaryIs>
<LinearRing>
<coordinates>
-0.99800000000005,48.051,0
-0.99636414192616,48.05099998839,0
-0.99637036384399,48.051095295041,0
-0.99638898834567,48.051189876525,0
-0.99641987375405,48.051283013015,0
-0.99646278507503,48.051373995674,0
-0.99651739578403,48.051462132058,0
-0.99658329030934,48.051546751379,0
-0.99665996719338,48.051627209613,0
-0.9967468429079,48.051702894405,0
-0.99684325629411,48.051773229725,0
-0.99694847359396,48.051837680255,0
-0.99706169403423,48.051895755466,0
-0.99718205592119,48.051947013347,0

-0.99730864319902,48.051991063775,0
-0.9974404924226,48.05202757148,0
-0.99757660009095,48.052056258599,0
-0.99771593028605,48.052076906795,0
-0.99785742255848,48.052089358911,0
-0.99800000000004,48.052093520174,0
-0.99814257744161,48.052089358911,0
-0.99828406971404,48.052076906795,0
-0.99842339990914,48.052056258599,0
-0.99855950757749,48.05202757148,0
-0.99869135680107,48.051991063775,0
-0.99881794407892,48.051947013347,0
-0.99893830596586,48.051895755466,0
-0.99905152640613,48.051837680255,0
-0.99915674370598,48.051773229725,0
-0.99925315709219,48.051702894405,0
-0.99934003280673,48.051627209613,0
-0.99941670969075,48.051546751379,0
-0.99948260421606,48.051462132058,0
-0.99953721492506,48.051373995674,0
-0.99958012624604,48.051283013015,0
-0.99961101165442,48.051189876525,0
-0.9996296361561,48.051095295041,0
-0.99963585807396,48.05099998839,0
-0.99962963012411,48.050904681915,0
-0.99961099977375,48.050810100955,0
-0.99958010887765,48.05071696532,0
-0.99953719259667,48.050625983822,0
-0.99948257760614,48.050537848869,0
-0.99941667960782,48.050453231206,0
-0.9993400001648,48.050372774805,0
-0.99925312288313,48.050297091968,0
-0.99915670896918,48.050226758665,0
-0.99905149219707,48.050162310151,0
-0.99893827332396,48.050104236895,0
-0.99881791399597,48.050052980848,0
-0.99869133019115,48.050008932078,0
-0.9985594852491,48.049972425804,0
-0.99842338254074,48.049943739845,0
-0.99828405783335,48.049923092505,0
-0.99814257140962,48.049910640913,0
-0.99800000000004,48.049906479827,0
-0.99785742859047,48.049910640913,0
-0.99771594216674,48.049923092505,0
-0.99757661745935,48.049943739845,0
-0.99744051475099,48.049972425804,0
-0.99730866980897,48.050008932078,0
-0.99718208600412,48.050052980848,0
-0.99706172667616,48.050104236895,0
-0.99694850780304,48.050162310151,0
-0.99684329103093,48.050226758665,0
-0.99674687711698,48.050297091968,0
-0.99665999983529,48.050372774805,0
-0.9965833203923,48.050453231206,0
-0.99651742239395,48.050537848869,0
-0.99646280740342,48.050625983822,0
-0.99641989112244,48.05071696532,0

-0.99638900022636,48.050810100955,0
-0.99637036987598,48.050904681915,0
-0.99636414192616,48.05099998839,0
-0.99800000000005,48.051,0
</coordinates>
</LinearRing>
</outerBoundaryIs>
</Polygon>
</Placemark>
<Placemark>
<name>67</name>
<styleUrl>#stylehap1</styleUrl>
<Polygon>
<outerBoundaryIs>
<LinearRing>
<coordinates>
-0.99700000000007,48.447,0
-0.99535142551542,48.446999988227,0
-0.99535769575702,48.447095294879,0
-0.99537646499575,48.447189876367,0
-0.9954075904544,48.447283012863,0
-0.99545083531329,48.447373995531,0
-0.99550587051069,48.447462131925,0
-0.9955722772453,48.447546751257,0
-0.99564955016221,48.447627209504,0
-0.99573710119767,48.44770289431,0
-0.99583426405362,48.447773229643,0
-0.99594029926823,48.447837680188,0
-0.99605439984336,48.447895755412,0
-0.99617569738651,48.447947013307,0
-0.99630326872036,48.447991063746,0
-0.99643614290957,48.448027571461,0
-0.99657330865156,48.448056258589,0
-0.99671372197446,48.44807690679,0
-0.99685631418427,48.44808935891,0
-0.99700000000006,48.448093520174,0
-0.99714368581587,48.44808935891,0
-0.99728627802566,48.44807690679,0
-0.99742669134859,48.448056258589,0
-0.99756385709055,48.448027571461,0
-0.99769673127979,48.447991063746,0
-0.99782430261363,48.447947013307,0
-0.99794560015676,48.447895755412,0
-0.99805970073189,48.447837680188,0
-0.99816573594652,48.447773229643,0
-0.99826289880248,48.44770289431,0
-0.99835044983791,48.447627209504,0
-0.99842772275485,48.447546751257,0
-0.99849412948945,48.447462131925,0
-0.99854916468686,48.447373995531,0
-0.99859240954575,48.447283012863,0
-0.9986235350044,48.447189876367,0
-0.99864230424312,48.447095294879,0
-0.99864857448472,48.446999988227,0
-0.99864229807908,48.446904681754,0
-0.99862352286361,48.446810100797,0
-0.99859239179713,48.446716965169,0

-0.99854914186964,48.446625983678,0
-0.99849410229698,48.446537848735,0
-0.99842769201331,48.446453231084,0
-0.9983504164814,48.446372774696,0
-0.99826286384449,48.446297091873,0
-0.99816570044927,48.446226758584,0
-0.99805966577393,48.446162310083,0
-0.99794556680024,48.446104236841,0
-0.99782427187209,48.446052980807,0
-0.99769670408732,48.446008932049,0
-0.99756383427336,48.445972425785,0
-0.99742667359995,48.445943739834,0
-0.9972862658849,48.4459230925,0
-0.99714367965185,48.445910640911,0
-0.99700000000006,48.445906479826,0
-0.99685632034829,48.445910640911,0
-0.99671373411524,48.4459230925,0
-0.9965733264002,48.445943739834,0
-0.99643616572679,48.445972425785,0
-0.99630329591283,48.446008932049,0
-0.99617572812805,48.446052980807,0
-0.9960544331999,48.446104236841,0
-0.99594033422621,48.446162310083,0
-0.99583429955088,48.446226758584,0
-0.99573713615563,48.446297091873,0
-0.99564958351875,48.446372774696,0
-0.99557230798684,48.446453231084,0
-0.99550589770316,48.446537848735,0
-0.99545085813048,48.446625983678,0
-0.99540760820301,48.446716965169,0
-0.99537647713651,48.446810100797,0
-0.99535770192107,48.446904681754,0
-0.99535142551542,48.446999988227,0
-0.99700000000007,48.447,0
</coordinates>
</LinearRing>
</outerBoundaryIs>
</Polygon>
</Placemark>
<Placemark>
<name>68</name>
<styleUrl>#stylehap1</styleUrl>
<Polygon>
<outerBoundaryIs>
<LinearRing>
<coordinates>
-0.98500000000001,48.262,0
-0.98335740054769,48.261999988303,0
-0.98336364808357,48.262095294955,0
-0.98338234931506,48.262189876441,0
-0.98341336198185,48.262283012934,0
-0.98345645012204,48.262373995598,0
-0.98351128586605,48.262462131987,0
-0.98357745193001,48.262546751314,0
-0.98365444479028,48.262627209556,0
-0.98374167851414,48.262702894355,0
-0.98383848921839,48.262773229682,0

-0.98394414012116,48.262837680219,0
-0.98405782714922,48.262895755437,0
-0.98417868505761,48.262947013326,0
-0.98430579401516,48.262991063759,0
-0.98443818660597,48.263027571469,0
-0.98457485519315,48.263056258594,0
-0.98471475958908,48.263076906792,0
-0.98485683497367,48.26308935891,0
-0.98500000000002,48.263093520173,0
-0.98514316502636,48.26308935891,0
-0.98528524041092,48.263076906792,0
-0.98542514480688,48.263056258594,0
-0.98556181339406,48.263027571469,0
-0.98569420598487,48.262991063759,0
-0.98582131494242,48.262947013326,0
-0.98594217285081,48.262895755437,0
-0.98605585987887,48.262837680219,0
-0.98616151078164,48.262773229682,0
-0.98625832148589,48.262702894355,0
-0.98634555520975,48.262627209556,0
-0.98642254807002,48.262546751314,0
-0.98648871413398,48.262462131987,0
-0.98654354987797,48.262373995598,0
-0.98658663801818,48.262283012934,0
-0.98661765068497,48.262189876441,0
-0.98663635191646,48.262095294955,0
-0.98664259945234,48.261999988303,0
-0.98663634581456,48.261904681829,0
-0.9866176386666,48.261810100871,0
-0.98658662044849,48.26171696524,0
-0.98654352729081,48.261625983745,0
-0.98648868721565,48.261537848798,0
-0.98642251763842,48.261453231141,0
-0.98634552218954,48.261372774747,0
-0.98625828688033,48.261297091918,0
-0.98616147564226,48.261226758622,0
-0.98605582527332,48.261162310115,0
-0.98594213983059,48.261104236866,0
-0.98582128451082,48.261052980826,0
-0.98569417906654,48.261008932062,0
-0.9855617908069,48.260972425794,0
-0.98542512723719,48.260943739839,0
-0.98528522839255,48.260923092502,0
-0.98514315892448,48.260910640912,0
-0.98500000000002,48.260906479826,0
-0.98485684107555,48.260910640912,0
-0.98471477160745,48.260923092502,0
-0.98457487276284,48.260943739839,0
-0.98443820919313,48.260972425794,0
-0.98430582093349,48.261008932062,0
-0.98417871548921,48.261052980826,0
-0.98405786016944,48.261104236866,0
-0.98394417472671,48.261162310115,0
-0.98383852435777,48.261226758622,0
-0.98374171311967,48.261297091918,0
-0.98365447781049,48.261372774747,0
-0.98357748236161,48.261453231141,0

-0.98351131278435,48.261537848798,0
-0.98345647270922,48.261625983745,0
-0.98341337955154,48.26171696524,0
-0.98338236133343,48.261810100871,0
-0.98336365418545,48.261904681829,0
-0.98335740054769,48.261999988303,0
-0.98500000000001,48.262,0
</coordinates>
</LinearRing>
</outerBoundaryIs>
</Polygon>
</Placemark>
<Placemark>
<name>72</name>
<styleUrl>#stylehap1</styleUrl>
<Polygon>
<outerBoundaryIs>
<LinearRing>
<coordinates>
-0.96699999999998,48.144,0
-0.96536118017844,48.143999988352,0
-0.96536741335129,48.144095295003,0
-0.96538607156334,48.144189876488,0
-0.96541701288126,48.144283012979,0
-0.96546000188577,48.144373995641,0
-0.96551471146132,48.144462132027,0
-0.96558072528404,48.14454675135,0
-0.96565754098863,48.144627209588,0
-0.96574457399049,48.144702894383,0
-0.96584116193385,48.144773229706,0
-0.96594656973219,48.144837680239,0
-0.96605999516249,48.144895755453,0
-0.96618057497078,48.144947013338,0
-0.96630739144264,48.144991063768,0
-0.9664394793884,48.145027571475,0
-0.96657583349001,48.145056258597,0
-0.96671541595368,48.145076906794,0
-0.96685716440977,48.145089358911,0
-0.96699999999999,48.145093520174,0
-0.9671428355902,48.145089358911,0
-0.96728458404629,48.145076906794,0
-0.96742416650996,48.145056258597,0
-0.96756052061157,48.145027571475,0
-0.96769260855733,48.144991063768,0
-0.96781942502919,48.144947013338,0
-0.96794000483748,48.144895755453,0
-0.96805343026778,48.144837680239,0
-0.96815883806612,48.144773229706,0
-0.96825542600949,48.144702894383,0
-0.96834245901132,48.144627209588,0
-0.96841927471591,48.14454675135,0
-0.96848528853863,48.144462132027,0
-0.9685399981142,48.144373995641,0
-0.96858298711869,48.144283012979,0
-0.96861392843664,48.144189876488,0
-0.96863258664868,48.144095295003,0
-0.96863881982153,48.143999988352,0

-0.96863258058601,48.143904681877,0
-0.96861391649551,48.143810100918,0
-0.96858296966195,48.143716965285,0
-0.96853997567224,48.143625983788,0
-0.96848526179334,48.143537848837,0
-0.96841924447992,48.143453231177,0
-0.96834242620336,48.14337277478,0
-0.96825539162637,48.143297091946,0
-0.96815880315261,48.143226758646,0
-0.96805339588469,48.143162310135,0
-0.96793997202953,48.143104236882,0
-0.96781939479321,48.143052980838,0
-0.96769258181202,48.143008932071,0
-0.9675604981696,48.1429724258,0
-0.96742414905319,48.142943739843,0
-0.96728457210516,48.142923092504,0
-0.96714282952753,48.142910640912,0
-0.96699999999999,48.142906479826,0
-0.96685717047244,48.142910640912,0
-0.96671542789481,48.142923092504,0
-0.96657585094678,48.142943739843,0
-0.96643950183037,48.1429724258,0
-0.96630741818795,48.143008932071,0
-0.96618060520676,48.143052980838,0
-0.96606002797044,48.143104236882,0
-0.96594660411529,48.143162310135,0
-0.96584119684736,48.143226758646,0
-0.96574460837358,48.143297091946,0
-0.96565757379661,48.14337277478,0
-0.96558075552005,48.143453231177,0
-0.96551473820663,48.143537848837,0
-0.96546002432774,48.143625983788,0
-0.96541703033802,48.143716965285,0
-0.96538608350446,48.143810100918,0
-0.96536741941396,48.143904681877,0
-0.96536118017844,48.143999988352,0
-0.96699999999998,48.144,0
</coordinates>
</LinearRing>
</outerBoundaryIs>
</Polygon>
</Placemark>
<Placemark>
<name>74</name>
<styleUrl>#stylehap1</styleUrl>
<Polygon>
<outerBoundaryIs>
<LinearRing>
<coordinates>
-0.96100000000001,48.279,0
-0.95935685401243,48.278999988296,0
-0.95936310362521,48.279095294948,0
-0.95938181107731,48.279189876435,0
-0.95941283406116,48.279283012927,0
-0.95945593653641,48.279373995592,0
-0.95951079052436,48.279462131982,0
-0.95957697860245,48.279546751309,0

-0.95965399707943,48.279627209551,0
-0.95974125982777,48.27970289435,0
-0.95983810274325,48.279773229678,0
-0.95994378879898,48.279837680217,0
-0.96005751365412,48.279895755435,0
-0.96017841177581,48.279947013324,0
-0.96030556302685,48.279991063758,0
-0.96043799966939,48.280027571469,0
-0.96057471373122,48.280056258593,0
-0.96071466467864,48.280076906792,0
-0.96085678733714,48.28008935891,0
-0.961,48.280093520173,0
-0.96114321266289,48.28008935891,0
-0.96128533532139,48.280076906792,0
-0.96142528626878,48.280056258593,0
-0.96156200033063,48.280027571469,0
-0.96169443697317,48.279991063758,0
-0.96182158822422,48.279947013324,0
-0.9619424863459,48.279895755435,0
-0.96205621120105,48.279837680217,0
-0.96216189725678,48.279773229678,0
-0.96225874017226,48.27970289435,0
-0.96234600292059,48.279627209551,0
-0.96242302139758,48.279546751309,0
-0.96248920947567,48.279462131982,0
-0.96254406346362,48.279373995592,0
-0.96258716593887,48.279283012927,0
-0.96261818892272,48.279189876435,0
-0.96263689637482,48.279095294948,0
-0.96264314598757,48.278999988296,0
-0.96263689026724,48.278904681822,0
-0.96261817689315,48.278810100864,0
-0.96258714835282,48.278716965233,0
-0.96254404085545,48.278625983739,0
-0.9624891825323,48.278537848792,0
-0.96242299093769,48.278453231136,0
-0.96234596986964,48.278372774743,0
-0.96225870553455,48.278297091914,0
-0.9621618620847,48.278226758618,0
-0.96205617656331,48.278162310112,0
-0.96194245329495,48.278104236864,0
-0.9618215577643,48.278052980824,0
-0.9616944100298,48.278008932061,0
-0.96156197772246,48.277972425793,0
-0.96142526868275,48.277943739839,0
-0.96128532329181,48.277923092502,0
-0.96114320655533,48.277910640912,0
-0.961,48.277906479826,0
-0.9608567934447,48.277910640912,0
-0.96071467670821,48.277923092502,0
-0.96057473131727,48.277943739839,0
-0.96043802227756,48.277972425793,0
-0.96030558997022,48.278008932061,0
-0.96017844223573,48.278052980824,0
-0.96005754670507,48.278104236864,0
-0.9599438234367,48.278162310112,0
-0.95983813791533,48.278226758618,0

-0.95974129446548,48.278297091914,0
-0.95965403013038,48.278372774743,0
-0.95957700906234,48.278453231136,0
-0.95951081746773,48.278537848792,0
-0.95945595914458,48.278625983739,0
-0.95941285164719,48.278716965233,0
-0.95938182310688,48.278810100864,0
-0.95936310973279,48.278904681822,0
-0.95935685401243,48.278999988296,0
-0.96100000000001,48.279,0
</coordinates>
</LinearRing>
</outerBoundaryIs>
</Polygon>
</Placemark>
<Placemark>
<name>75</name>
<styleUrl>#stylehap1</styleUrl>
<Polygon>
<outerBoundaryIs>
<LinearRing>
<coordinates>
-0.94799999999998,47.823,0
-0.94637133980015,47.822999988482,0
-0.94637753436514,47.823095295133,0
-0.94639607694107,47.823189876615,0
-0.94642682647355,47.823283013101,0
-0.94646954900183,47.823373995756,0
-0.9465239194374,47.823462132134,0
-0.9465895240366,47.823546751448,0
-0.94666586354788,47.823627209676,0
-0.94675235701024,47.823702894459,0
-0.94684834617386,47.823773229771,0
-0.94695310050915,47.823837680293,0
-0.94706582276643,47.823895755496,0
-0.94718565504366,47.82394701337,0
-0.9473116853161,47.823991063791,0
-0.94744295437816,47.82402757149,0
-0.9475784631449,47.824056258606,0
-0.94771718025697,47.824076906798,0
-0.94785804993168,47.824089358912,0
-0.94799999999997,47.824093520174,0
-0.94814195006827,47.824089358912,0
-0.94828281974298,47.824076906798,0
-0.94842153685507,47.824056258606,0
-0.94855704562179,47.82402757149,0
-0.94868831468387,47.823991063791,0
-0.94881434495628,47.82394701337,0
-0.94893417723354,47.823895755496,0
-0.94904689949082,47.823837680293,0
-0.94915165382611,47.823773229771,0
-0.94924764298973,47.823702894459,0
-0.94933413645209,47.823627209676,0
-0.94941047596337,47.823546751448,0
-0.94947608056257,47.823462132134,0
-0.94953045099814,47.823373995756,0
-0.94957317352639,47.823283013101,0

-0.9496039230589,47.823189876615,0
-0.94962246563483,47.823095295133,0
-0.94962866019979,47.822999988482,0
-0.94962245967725,47.822904682007,0
-0.94960391132474,47.822810101044,0
-0.94957315637221,47.822716965407,0
-0.94953042894514,47.822625983903,0
-0.94947605428083,47.822537848945,0
-0.94941044625144,47.822453231275,0
-0.94933410421276,47.822372774867,0
-0.94924760920257,47.822297092023,0
-0.94915161951775,47.822226758711,0
-0.94904686570369,47.822162310189,0
-0.94893414499421,47.822104236925,0
-0.94881431524438,47.822052980871,0
-0.94868828840213,47.822008932094,0
-0.94855702356881,47.821972425815,0
-0.94842151970089,47.821943739851,0
-0.94828280800884,47.821923092508,0
-0.94814194411068,47.821910640913,0
-0.94799999999997,47.821906479826,0
-0.94785805588926,47.821910640913,0
-0.94771719199113,47.821923092508,0
-0.94757848029908,47.821943739851,0
-0.94744297643116,47.821972425815,0
-0.94731171159783,47.822008932094,0
-0.94718568475559,47.822052980871,0
-0.94706585500576,47.822104236925,0
-0.94695313429628,47.822162310189,0
-0.94684838048222,47.822226758711,0
-0.94675239079737,47.822297092023,0
-0.94666589578718,47.822372774867,0
-0.9465895537485,47.822453231275,0
-0.94652394571911,47.822537848945,0
-0.9464695710548,47.822625983903,0
-0.94642684362773,47.822716965407,0
-0.94639608867523,47.822810101044,0
-0.94637754032272,47.822904682007,0
-0.94637133980015,47.822999988482,0
-0.94799999999998,47.823,0
</coordinates>
</LinearRing>
</outerBoundaryIs>
</Polygon>
</Placemark>
<Placemark>
<name>76</name>
<styleUrl>#stylehap1</styleUrl>
<Polygon>
<outerBoundaryIs>
<LinearRing>
<coordinates>
-0.947,48.176,0
-0.94536015760093,48.175999988339,0
-0.9453663946597,48.17609529499,0
-0.94538506451064,48.176189876476,0
-0.94541602513196,48.176283012967,0

-0.94545904095758,48.176373995629,0
-0.94551378466796,48.176462132016,0
-0.94557983967951,48.176546751341,0
-0.94565670331348,48.176627209579,0
-0.94574379062061,48.176702894375,0
-0.94584043883182,48.176773229699,0
-0.94594591240197,48.176837680234,0
-0.94605940860742,48.176895755449,0
-0.94618006365557,48.176947013334,0
-0.94630695925933,48.176991063766,0
-0.94643912962667,48.177027571474,0
-0.94657556881222,48.177056258596,0
-0.94671523837454,48.177076906793,0
-0.94685707528099,48.177089358911,0
-0.94700000000001,48.177093520174,0
-0.94714292471901,48.177089358911,0
-0.94728476162546,48.177076906793,0
-0.94742443118778,48.177056258596,0
-0.94756087037333,48.177027571474,0
-0.9476930407407,48.176991063766,0
-0.94781993634443,48.176947013334,0
-0.94794059139258,48.176895755449,0
-0.94805408759803,48.176837680234,0
-0.94815956116818,48.176773229699,0
-0.94825620937939,48.176702894375,0
-0.94834329668652,48.176627209579,0
-0.94842016032049,48.176546751341,0
-0.94848621533204,48.176462132016,0
-0.94854095904242,48.176373995629,0
-0.94858397486804,48.176283012967,0
-0.94861493548938,48.176189876476,0
-0.94863360534032,48.17609529499,0
-0.94863984239907,48.175999988339,0
-0.94863359926704,48.175904681864,0
-0.94861492352737,48.175810100905,0
-0.94858395738074,48.175716965273,0
-0.94854093656119,48.175625983776,0
-0.94848618853996,48.175537848827,0
-0.94842013003164,48.175453231167,0
-0.94834326382116,48.175372774771,0
-0.94825617493617,48.175297091939,0
-0.9481595261936,48.175226758639,0
-0.94805405315481,48.17516231013,0
-0.94794055852722,48.175104236878,0
-0.94781990605555,48.175052980835,0
-0.94769301394859,48.175008932069,0
-0.9475608478921,48.174972425798,0
-0.94742441370048,48.174943739842,0
-0.94728474966345,48.174923092503,0
-0.94714291864575,48.174910640912,0
-0.94700000000001,48.174906479827,0
-0.94685708135424,48.174910640912,0
-0.94671525033655,48.174923092503,0
-0.94657558629952,48.174943739842,0
-0.9464391521079,48.174972425798,0
-0.94630698605141,48.175008932069,0
-0.94618009394445,48.175052980835,0

-0.94605944147278,48.175104236878,0
-0.94594594684521,48.17516231013,0
-0.9458404738064,48.175226758639,0
-0.94574382506385,48.175297091939,0
-0.94565673617884,48.175372274771,0
-0.94557986996839,48.175453231167,0
-0.94551381146007,48.175537848827,0
-0.94545906343881,48.175625983776,0
-0.94541604261926,48.175716965273,0
-0.94538507647263,48.175810100905,0
-0.94536640073296,48.175904681864,0
-0.94536015760093,48.175999988339,0
-0.947,48.176,0
</coordinates>
</LinearRing>
</outerBoundaryIs>
</Polygon>
</Placemark>
<Placemark>
<name>77</name>
<styleUrl>#stylehap1</styleUrl>
<Polygon>
<outerBoundaryIs>
<LinearRing>
<coordinates>
-0.94600000000003,48.291,0
-0.94435646791671,48.290999988291,0
-0.94436271899669,48.291095294943,0
-0.94438143084329,48.29118987643,0
-0.94441246111555,48.291283012923,0
-0.94445557371766,48.291373995587,0
-0.94451044059393,48.291462131977,0
-0.94457664422371,48.291546751305,0
-0.94465368079747,48.291627209548,0
-0.9447409640499,48.291702894348,0
-0.9448378297208,48.291773229676,0
-0.94494354061005,48.291837680215,0
-0.94505729218785,48.291895755434,0
-0.94517821871795,48.291947013323,0
-0.94530539984692,48.291991063757,0
-0.94543786760946,48.292027571468,0
-0.94557461379654,48.292056258593,0
-0.94571459762988,48.292076906792,0
-0.94585675368469,48.29208935891,0
-0.94600000000003,48.292093520174,0
-0.94614324631537,48.29208935891,0
-0.94628540237018,48.292076906792,0
-0.94642538620351,48.292056258593,0
-0.9465621323906,48.292027571468,0
-0.94669460015314,48.291991063757,0
-0.9468217812821,48.291947013323,0
-0.9469427078122,48.291895755434,0
-0.94705645939001,48.291837680215,0
-0.94716217027926,48.291773229676,0
-0.94725903595016,48.291702894348,0
-0.94734631920259,48.291627209548,0
-0.94742335577634,48.291546751305,0

-0.94748955940612,48.291462131977,0
-0.94754442628239,48.291373995587,0
-0.9475875388845,48.291283012923,0
-0.94761856915676,48.29118987643,0
-0.94763728100337,48.291095294943,0
-0.94764353208335,48.290999988291,0
-0.94763727489179,48.290904681818,0
-0.9476185571193,48.290810100859,0
-0.94758752128693,48.290716965229,0
-0.94754440365936,48.290625983735,0
-0.94748953244504,48.290537848788,0
-0.94742332529643,48.290453231132,0
-0.94734628612993,48.290372774739,0
-0.94725900128967,48.290297091911,0
-0.94716213508408,48.290226758616,0
-0.94705642472952,48.29016231011,0
-0.94694267473955,48.290104236863,0
-0.94682175080219,48.290052980823,0
-0.94669457319208,48.29000893206,0
-0.94656210976757,48.289972425793,0
-0.94642536860591,48.289943739839,0
-0.94628539033269,48.289923092502,0
-0.94614324020379,48.289910640912,0
-0.94600000000003,48.289906479826,0
-0.94585675979626,48.289910640912,0
-0.94571460966734,48.289923092502,0
-0.94557463139412,48.289943739839,0
-0.94543789023249,48.289972425793,0
-0.94530542680797,48.29000893206,0
-0.94517824919787,48.290052980823,0
-0.94505732526051,48.290104236863,0
-0.94494357527053,48.29016231011,0
-0.94483786491598,48.290226758616,0
-0.94474099871039,48.290297091911,0
-0.94465371387012,48.290372774739,0
-0.94457667470363,48.290453231132,0
-0.94451046755501,48.290537848788,0
-0.9444555963407,48.290625983735,0
-0.94441247871313,48.290716965229,0
-0.94438144288075,48.290810100859,0
-0.94436272510826,48.290904681818,0
-0.94435646791671,48.290999988291,0
-0.94600000000003,48.291,0
</coordinates>
</LinearRing>
</outerBoundaryIs>
</Polygon>
</Placemark>
<Placemark>
<name>78</name>
<styleUrl>#stylehap1</styleUrl>
<Polygon>
<outerBoundaryIs>
<LinearRing>
<coordinates>
-0.94399999999996,47.803,0
-0.94237196694412,47.80299998849,0

-0.94237815912585,47.803095295141,0
-0.94239669456368,47.803189876623,0
-0.94242743225743,47.803283013108,0
-0.94247013833636,47.803373995763,0
-0.94252448783711,47.803462132141,0
-0.94259006717533,47.803546751454,0
-0.94266637729164,47.803627209681,0
-0.94275283744873,47.803702894464,0
-0.94284878965021,47.803773229775,0
-0.94295350364785,47.803837680297,0
-0.94306618249893,47.803895755499,0
-0.94318596863179,47.803947013372,0
-0.94331195037285,47.803991063793,0
-0.943443168886,47.804027571491,0
-0.94357862547101,47.804056258606,0
-0.94371728916573,47.804076906798,0
-0.9438581045941,47.804089358912,0
-0.94399999999996,47.804093520174,0
-0.94414189540581,47.804089358912,0
-0.94428271083418,47.804076906798,0
-0.94442137452893,47.804056258606,0
-0.94455683111392,47.804027571491,0
-0.94468804962706,47.803991063793,0
-0.94481403136813,47.803947013372,0
-0.94493381750098,47.803895755499,0
-0.94504649635207,47.803837680297,0
-0.9451512103497,47.803773229775,0
-0.94524716255118,47.803702894464,0
-0.94533362270827,47.803627209681,0
-0.94540993282458,47.803546751454,0
-0.9454755121628,47.803462132141,0
-0.94552986166358,47.803373995763,0
-0.94557256774249,47.803283013108,0
-0.94560330543623,47.803189876623,0
-0.94562184087406,47.803095295141,0
-0.94562803305579,47.80299998849,0
-0.94562183492296,47.802904682015,0
-0.94560329371485,47.802810101052,0
-0.94557255060693,47.802716965414,0
-0.94552983963453,47.80262598391,0
-0.94547548590961,47.802537848951,0
-0.94540990314494,47.802453231281,0
-0.94533359050398,47.802372774873,0
-0.94524712880074,47.802297092028,0
-0.94515117607859,47.802226758715,0
-0.94504646260162,47.802162310192,0
-0.94493378529669,47.802104236928,0
-0.94481400168849,47.802052980873,0
-0.94468802337388,47.802008932096,0
-0.94455680908489,47.801972425816,0
-0.94442135739338,47.801943739852,0
-0.94428269911277,47.801923092508,0
-0.94414188945469,47.801910640913,0
-0.94399999999996,47.801906479826,0
-0.94385811054522,47.801910640913,0
-0.94371730088715,47.801923092508,0
-0.94357864260654,47.801943739852,0

-0.94344319091503,47.801972425816,0
-0.94331197662604,47.802008932096,0
-0.94318599831143,47.802052980873,0
-0.94306621470325,47.802104236928,0
-0.94295353739829,47.802162310192,0
-0.94284882392132,47.802226758715,0
-0.94275287119918,47.802297092028,0
-0.94266640949593,47.802372774873,0
-0.94259009685497,47.802453231281,0
-0.9425245140903,47.802537848951,0
-0.94247016036539,47.80262598391,0
-0.94242744939298,47.802716965414,0
-0.94239670628509,47.802810101052,0
-0.94237816507698,47.802904682015,0
-0.94237196694412,47.80299998849,0
-0.94399999999996,47.803,0
</coordinates>
</LinearRing>
</outerBoundaryIs>
</Polygon>
</Placemark>
<Placemark>
<name>79</name>
<styleUrl>#stylehap1</styleUrl>
<Polygon>
<outerBoundaryIs>
<LinearRing>
<coordinates>
-0.93399999999997,48.227,0
-0.93235852416849,48.226999988318,0
-0.93236476743449,48.227095294969,0
-0.93238345587702,48.227189876455,0
-0.932414447333,48.227283012947,0
-0.93245750600181,48.227373995611,0
-0.93251230423805,48.227462131999,0
-0.93257842504326,48.227546751325,0
-0.93265536523811,48.227627209565,0
-0.9327425392907,48.227702894363,0
-0.93283928377186,48.227773229689,0
-0.93294486240377,48.227837680225,0
-0.9330584716632,48.227895755442,0
-0.93317924689709,48.227947013329,0
-0.93330626890354,48.227991063762,0
-0.93343857092847,48.228027571471,0
-0.93357514602436,48.228056258595,0
-0.93371495471524,48.228076906793,0
-0.93385693290946,48.22808935891,0
-0.93399999999996,48.228093520174,0
-0.93414306709048,48.22808935891,0
-0.93428504528468,48.228076906793,0
-0.93442485397558,48.228056258595,0
-0.93456142907147,48.228027571471,0
-0.9346937310964,48.227991063762,0
-0.93482075310285,48.227947013329,0
-0.93494152833674,48.227895755442,0
-0.93505513759617,48.227837680225,0
-0.93516071622808,48.227773229689,0

-0.93525746070924,48.227702894363,0
-0.93534463476183,48.227627209565,0
-0.93542157495668,48.227546751325,0
-0.93548769576189,48.227462131999,0
-0.93554249399813,48.227373995611,0
-0.93558555266694,48.227283012947,0
-0.93561654412292,48.227189876455,0
-0.93563523256545,48.227095294969,0
-0.93564147583145,48.226999988318,0
-0.93563522647522,48.226904681844,0
-0.93561653212752,48.226810100885,0
-0.93558553513086,48.226716965253,0
-0.93554247145415,48.226625983758,0
-0.93548766889504,48.226537848809,0
-0.93542154458328,48.226453231152,0
-0.93534460180477,48.226372774757,0
-0.93525742616989,48.226297091926,0
-0.93516068115587,48.226226758629,0
-0.9350551030568,48.226162310121,0
-0.93494149537967,48.226104236871,0
-0.93482072272944,48.22605298083,0
-0.93469370422954,48.226008932065,0
-0.93456140652749,48.225972425796,0
-0.93442483643948,48.22594373984,0
-0.9342850332893,48.225923092503,0
-0.93414306100025,48.225910640912,0
-0.93399999999996,48.225906479826,0
-0.93385693899969,48.225910640912,0
-0.93371496671064,48.225923092503,0
-0.93357516356046,48.22594373984,0
-0.93343859347245,48.225972425796,0
-0.9333062957704,48.226008932065,0
-0.9331792772705,48.22605298083,0
-0.93305850462026,48.226104236871,0
-0.93294489694312,48.226162310121,0
-0.93283931884404,48.226226758629,0
-0.93274257383005,48.226297091926,0
-0.93265539819517,48.226372774757,0
-0.93257845541666,48.226453231152,0
-0.9325123311049,48.226537848809,0
-0.93245752854579,48.226625983758,0
-0.93241446486908,48.226716965253,0
-0.93238346787242,48.226810100885,0
-0.93236477352472,48.226904681844,0
-0.93235852416849,48.226999988318,0
-0.93399999999997,48.227,0
</coordinates>
</LinearRing>
</outerBoundaryIs>
</Polygon>
</Placemark>
<Placemark>
<name>80</name>
<styleUrl>#stylehap1</styleUrl>
<Polygon>
<outerBoundaryIs>
<LinearRing>

<coordinates>
-0.93200000000002,47.945,0
-0.93036749941934,47.944999988433,0
-0.93037370857829,47.945095295084,0
-0.9303922948652,47.945189876567,0
-0.93042311689344,47.945283013055,0
-0.9304659401509,47.945373995712,0
-0.93052043878287,47.945462132094,0
-0.93058619807036,47.945546751411,0
-0.93066271758478,47.945627209642,0
-0.93074941499539,47.94570289443,0
-0.93084563050034,47.945773229746,0
-0.93095063184748,47.945837680273,0
-0.93106361990718,47.94589575548,0
-0.93118373475438,47.945947013358,0
-0.93131006221357,47.945991063783,0
-0.93144164081718,47.946027571485,0
-0.93157746912413,47.946056258602,0
-0.93171651334281,47.946076906796,0
-0.93185771520065,47.946089358911,0
-0.93200000000001,47.946093520173,0
-0.93214228479937,47.946089358911,0
-0.93228348665722,47.946076906796,0
-0.9324225308759,47.946056258602,0
-0.93255835918285,47.946027571485,0
-0.93268993778646,47.945991063783,0
-0.93281626524565,47.945947013358,0
-0.93293638009285,47.94589575548,0
-0.93304936815255,47.945837680273,0
-0.93315436949969,47.945773229746,0
-0.93325058500463,47.94570289443,0
-0.93333728241527,47.945627209642,0
-0.93341380192969,47.945546751411,0
-0.93347956121715,47.945462132094,0
-0.93353405984913,47.945373995712,0
-0.93357688310659,47.945283013055,0
-0.93360770513483,47.945189876567,0
-0.93362629142174,47.945095295084,0
-0.93363250058069,47.944999988433,0
-0.93362628542448,47.944904681958,0
-0.93360769332256,47.944810100996,0
-0.93357686583819,47.944716965361,0
-0.93353403764932,47.94462598386,0
-0.93347953476046,47.944537848904,0
-0.93341377201996,47.944453231238,0
-0.93333724996133,47.944372774834,0
-0.93325055099254,47.944297091994,0
-0.93315433496294,47.944226758686,0
-0.93304933414049,47.944162310168,0
-0.9329363476389,47.944104236909,0
-0.93281623533594,47.944052980858,0
-0.93268991132977,47.944008932086,0
-0.93255833698306,47.943972425809,0
-0.93242251360752,47.943943739848,0
-0.93228347484494,47.943923092506,0
-0.93214227880215,47.943910640913,0
-0.93200000000001,47.943906479826,0

-0.93185772119791,47.943910640913,0
-0.93171652515508,47.943923092506,0
-0.9315774863925,47.943943739848,0
-0.931441663017,47.943972425809,0
-0.93131008867026,47.944008932086,0
-0.93118376466411,47.944052980858,0
-0.93106365236115,47.944104236909,0
-0.93095066585956,47.944162310168,0
-0.93084566503712,47.944226758686,0
-0.93074944900748,47.944297091994,0
-0.93066275003873,47.944372774834,0
-0.93058622798007,47.944453231238,0
-0.93052046523957,47.944537848904,0
-0.93046596235071,47.94462598386,0
-0.93042313416184,47.944716965361,0
-0.93039230667747,47.944810100996,0
-0.93037371457554,47.944904681958,0
-0.93036749941934,47.944999988433,0
-0.93200000000002,47.945,0
</coordinates>
</LinearRing>
</outerBoundaryIs>
</Polygon>
</Placemark>
<Placemark>
<name>81</name>
<styleUrl>#stylehap1</styleUrl>
<Polygon>
<outerBoundaryIs>
<LinearRing>
<coordinates>
-0.92700000000002,48.017,0
-0.92536522097106,48.016999988404,0
-0.92537143878839,48.017095295055,0
-0.92539005100845,48.017189876539,0
-0.92542091604746,48.017283013027,0
-0.92546379906624,48.017373995687,0
-0.92551837375546,48.01746213207,0
-0.92558422481747,48.017546751389,0
-0.92566085112542,48.017627209623,0
-0.92574766953592,48.017702894413,0
-0.92584401932624,48.017773229732,0
-0.92594916722232,48.017837680261,0
-0.92606231297917,48.01789575547,0
-0.92618259547147,48.017947013351,0
-0.92630909924774,48.017991063777,0
-0.92644086149836,48.018027571481,0
-0.92657687938438,48.0180562586,0
-0.92671611767122,48.018076906795,0
-0.92685751660901,48.018089358911,0
-0.92700000000001,48.018093520174,0
-0.92714248339102,48.018089358911,0
-0.92728388232883,48.018076906795,0
-0.92742312061565,48.0180562586,0
-0.92755913850169,48.018027571481,0
-0.92769090075232,48.017991063777,0
-0.92781740452858,48.017947013351,0

-0.92793768702088,48.01789575547,0
-0.92805083277773,48.017837680261,0
-0.92815598067381,48.017773229732,0
-0.92825233046414,48.017702894413,0
-0.92833914887463,48.017627209623,0
-0.92841577518256,48.017546751389,0
-0.92848162624459,48.01746213207,0
-0.92853620093381,48.017373995687,0
-0.92857908395257,48.017283013027,0
-0.92860994899161,48.017189876539,0
-0.92862856121166,48.017095295055,0
-0.92863477902899,48.016999988404,0
-0.92862855519084,48.016904681929,0
-0.92860993713289,48.016810100968,0
-0.92857906661633,48.016716965333,0
-0.92853617864678,48.016625983834,0
-0.92848159968393,48.01653784888,0
-0.92841574515532,48.016453231216,0
-0.92833911629316,48.016372774815,0
-0.92825229631839,48.016297091977,0
-0.92815594600134,48.016226758672,0
-0.92805079863201,48.016162310156,0
-0.92793765443941,48.016104236899,0
-0.92781737450132,48.016052980851,0
-0.92769087419166,48.0160089320

8,0
-0.92755911621465,48.015972425806,0
-0.92742310327941,48.015943739846,0
-0.92728387047015,48.015923092505,0
-0.92714247737021,48.015910640913,0
-0.92700000000001,48.015906479826,0
-0.92685752262982,48.015910640913,0
-0.92671612952991,48.015923092505,0
-0.92657689672062,48.015943739846,0
-0.9264408837854,48.015972425806,0
-0.9263091258084,48.01600893208,0
-0.92618262549871,48.016052980851,0
-0.92606234556064,48.016104236899,0
-0.92594920136805,48.016162310156,0
-0.92584405399872,48.016226758672,0
-0.92574770368164,48.016297091977,0
-0.9256608837069,48.016372774815,0
-0.92558425484473,48.016453231216,0
-0.92551840031612,48.01653784888,0
-0.92546382135328,48.016625983834,0
-0.92542093338369,48.016716965333,0
-0.92539006286713,48.016810100968,0
-0.92537144480921,48.016904681929,0
-0.92536522097106,48.016999988404,0
-0.92700000000002,48.017,0
</coordinates>
</LinearRing>
</outerBoundaryIs>
</Polygon>
</Placemark>
<Placemark>
<name>83</name>
<styleUrl>#stylehap1</styleUrl>

<Polygon>
<outerBoundaryIs>
<LinearRing>
<coordinates>
-0.91899999999998,47.864,0
-0.91737005202199,47.863999988466,0
-0.91737625148071,47.864095295116,0
-0.91739480871406,47.864189876599,0
-0.91742558255624,47.864283013085,0
-0.91746833886157,47.864373995741,0
-0.91752275228468,47.864462132121,0
-0.91758840875484,47.864546751436,0
-0.91766480862579,47.864627209664,0
-0.91775137047725,47.86470289445,0
-0.9178474355389,47.864773229763,0
-0.9179522727036,47.864837680287,0
-0.91806508409127,47.864895755491,0
-0.91818501112145,47.864947013366,0
-0.91831114104822,47.864991063788,0
-0.91844251390748,47.865027571488,0
-0.91857812982426,47.865056258604,0
-0.91871695662355,47.865076906797,0
-0.91885793768778,47.865089358911,0
-0.91899999999998,47.865093520174,0
-0.91914206231219,47.865089358911,0
-0.91928304337641,47.865076906797,0
-0.91942187017571,47.865056258604,0
-0.91955748609246,47.865027571488,0
-0.91968885895175,47.864991063788,0
-0.91981498887852,47.864947013366,0
-0.9199349159087,47.864895755491,0
-0.92004772729637,47.864837680287,0
-0.92015256446106,47.864773229763,0
-0.92024862952272,47.86470289445,0
-0.92033519137417,47.864627209664,0
-0.92041159124513,47.864546751436,0
-0.92047724771526,47.864462132121,0
-0.9205316611384,47.864373995741,0
-0.92057441744373,47.864283013085,0
-0.92060519128591,47.864189876599,0
-0.92062374851926,47.864095295116,0
-0.92062994797797,47.863999988466,0
-0.92062374254839,47.86390468199,0
-0.92060517952559,47.863810101028,0
-0.92057440025128,47.863716965391,0
-0.92053163903619,47.863625983889,0
-0.9204772213749,47.863537848931,0
-0.92041156146692,47.863453231263,0
-0.92033515906292,47.863372774856,0
-0.92024859566019,47.863297092013,0
-0.92015253007616,47.863226758703,0
-0.92004769343385,47.863162310182,0
-0.91993488359747,47.86310423692,0
-0.91981495910031,47.863052980867,0
-0.91968883261139,47.863008932091,0
-0.91955746399028,47.862972425813,0
-0.91942185298326,47.86294373985,0

-0.91928303161607,47.862923092507,0
-0.9191420563413,47.862910640913,0
-0.91899999999998,47.862906479826,0
-0.91885794365867,47.862910640913,0
-0.91871696838387,47.862923092507,0
-0.91857814701671,47.86294373985,0
-0.91844253600969,47.862972425813,0
-0.91831116738858,47.863008932091,0
-0.91818504089966,47.863052980867,0
-0.9180651164025,47.86310423692,0
-0.91795230656612,47.863162310182,0
-0.9178474699238,47.863226758703,0
-0.91775140433978,47.863297092013,0
-0.91766484093705,47.863372774856,0
-0.91758843853305,47.863453231263,0
-0.91752277862507,47.863537848931,0
-0.91746836096378,47.863625983889,0
-0.91742559974869,47.863716965391,0
-0.91739482047438,47.863810101028,0
-0.91737625745158,47.86390468199,0
-0.91737005202199,47.863999988466,0
-0.91899999999998,47.864,0
</coordinates>
</LinearRing>
</outerBoundaryIs>
</Polygon>
</Placemark>
<Placemark>
<name>85</name>
<styleUrl>#stylehap1</styleUrl>
<Polygon>
<outerBoundaryIs>
<LinearRing>
<coordinates>
-0.91700000000003,48.219,0
-0.91535878069441,48.218999988321,0
-0.91536502298558,48.219095294973,0
-0.91538370850836,48.219189876459,0
-0.91541469512183,48.21928301295,0
-0.91545774706227,48.219373995614,0
-0.91551253673535,48.219462132002,0
-0.91557864720786,48.219546751327,0
-0.91565557537905,48.219627209567,0
-0.9157427358085,48.219702894365,0
-0.91583946517076,48.21977322969,0
-0.91594502730302,48.219837680227,0
-0.91605861880765,48.219895755443,0
-0.9161793751667,48.21994701333,0
-0.91630637732199,48.219991063763,0
-0.91643865867046,48.220027571472,0
-0.91657521242203,48.220056258595,0
-0.91671499926319,48.220076906793,0
-0.91685695526856,48.22008935891,0
-0.91700000000004,48.220093520174,0
-0.91714304473152,48.22008935891,0
-0.91728500073689,48.220076906793,0
-0.91742478757802,48.220056258595,0

-0.91756134132959,48.220027571472,0
-0.91769362267809,48.219991063763,0
-0.91782062483335,48.21994701333,0
-0.91794138119242,48.219895755443,0
-0.91805497269704,48.219837680227,0
-0.9181605348293,48.21977322969,0
-0.91825726419155,48.219702894365,0
-0.91834442462101,48.219627209567,0
-0.9184213527922,48.219546751327,0
-0.9184874632647,48.219462132002,0
-0.91854225293781,48.219373995614,0
-0.91858530487822,48.21928301295,0
-0.91861629149172,48.219189876459,0
-0.91863497701448,48.219095294973,0
-0.91864121930565,48.218999988321,0
-0.91863497092692,48.218904681847,0
-0.91861627950156,48.218810100888,0
-0.9185852873498,48.218716965256,0
-0.91854223040369,48.218625983761,0
-0.9184874364096,48.218537848812,0
-0.91842132243207,48.218453231154,0
-0.91834439167835,48.218372774759,0
-0.91825722966729,48.218297091928,0
-0.91816049977244,48.218226758631,0
-0.91805493817278,48.218162310122,0
-0.91794134824974,48.218104236872,0
-0.91782059447323,48.218052980831,0
-0.91769359582296,48.218008932066,0
-0.91756131879546,48.217972425796,0
-0.9174247700496,48.217943739841,0
-0.91728498874674,48.217923092503,0
-0.91714303864394,48.217910640912,0
-0.91700000000004,48.217906479827,0
-0.91685696135612,48.217910640912,0
-0.91671501125334,48.217923092503,0
-0.91657522995048,48.217943739841,0
-0.91643868120459,48.217972425796,0
-0.9163064041771,48.218008932066,0
-0.91617940552683,48.218052980831,0
-0.91605865175031,48.218104236872,0
-0.91594506182728,48.218162310122,0
-0.91583950022762,48.218226758631,0
-0.91574277033276,48.218297091928,0
-0.91565560832171,48.218372774759,0
-0.91557867756798,48.218453231154,0
-0.91551256359048,48.218537848812,0
-0.91545776959637,48.218625983761,0
-0.91541471265027,48.218716965256,0
-0.91538372049851,48.218810100888,0
-0.91536502907313,48.218904681847,0
-0.91535878069441,48.218999988321,0
-0.91700000000003,48.219,0
</coordinates>
</LinearRing>
</outerBoundaryIs>
</Polygon>
</Placemark>

<Placemark>
<name>87</name>
<styleUrl>#stylehap1</styleUrl>
<Polygon>
<outerBoundaryIs>
<LinearRing>
<coordinates>
-0.89999999999998,48.396,0
-0.89835307873715,48.395999988248,0
-0.89835934269634,48.3960952949,0
-0.89837809311826,48.396189876388,0
-0.89840918736873,48.396283012882,0
-0.89845238886538,48.396373995549,0
-0.89850736887635,48.396462131942,0
-0.89857370902022,48.396546751273,0
-0.89865090444871,48.396627209518,0
-0.89873836768776,48.396702894322,0
-0.89883543310745,48.396773229654,0
-0.89894136198746,48.396837680197,0
-0.89905534813893,48.396895755419,0
-0.89917652404024,48.396947013312,0
-0.89930396743997,48.39699106375,0
-0.8994367083765,48.397027571463,0
-0.89957373656154,48.39705625859,0
-0.89971400907029,48.397076906791,0
-0.89985645828056,48.39708935891,0
-0.89999999999997,48.397093520174,0
-0.9001435417194,48.39708935891,0
-0.90028599092967,48.397076906791,0
-0.90042626343842,48.39705625859,0
-0.90056329162346,48.397027571463,0
-0.90069603256,48.39699106375,0
-0.9008234759597,48.396947013312,0
-0.90094465186103,48.396895755419,0
-0.90105863801251,48.396837680197,0
-0.90116456689252,48.396773229654,0
-0.9012616323122,48.396702894322,0
-0.90134909555123,48.396627209518,0
-0.90142629097975,48.396546751273,0
-0.90149263112359,48.396462131942,0
-0.90154761113455,48.396373995549,0
-0.90159081263123,48.396283012882,0
-0.9016219068817,48.396189876388,0
-0.90164065730362,48.3960952949,0
-0.90164692126282,48.395999988248,0
-0.90164065115678,48.395904681775,0
-0.90162189477483,48.395810100817,0
-0.90159079493216,48.395716965188,0
-0.90154758838107,48.395625983697,0
-0.90149260400704,48.395537848752,0
-0.90142626032406,48.3954532311,0
-0.90134906228787,48.39537277471,0
-0.90126159745182,48.395297091885,0
-0.90116453149437,48.395226758594,0
-0.90105860315213,48.395162310092,0
-0.90094461859764,48.395104236848,0
-0.90082344530401,48.395052980812,0

-0.90069600544345,48.395008932053,0
-0.90056326886995,48.394972425788,0
-0.90042624573935,48.394943739836,0
-0.9002859788228,48.394923092501,0
-0.90014353557259,48.394910640912,0
-0.89999999999997,48.394906479826,0
-0.89985646442738,48.394910640912,0
-0.89971402117716,48.394923092501,0
-0.89957375426062,48.394943739836,0
-0.89943673112999,48.394972425788,0
-0.89930399455651,48.395008932053,0
-0.89917655469596,48.395052980812,0
-0.89905538140229,48.395104236848,0
-0.89894139684781,48.395162310092,0
-0.89883546850557,48.395226758594,0
-0.89873840254812,48.395297091885,0
-0.8986509377121,48.39537277471,0
-0.8985737396759,48.3954532311,0
-0.89850739599289,48.395537848752,0
-0.89845241161887,48.395625983697,0
-0.89840920506778,48.395716965188,0
-0.89837810522513,48.395810100817,0
-0.89835934884316,48.395904681775,0
-0.89835307873715,48.395999988248,0
-0.89999999999998,48.396,0
</coordinates>
</LinearRing>
</outerBoundaryIs>
</Polygon>
</Placemark>
<Placemark>
<name>89</name>
<styleUrl>#stylehap1</styleUrl>
<Polygon>
<outerBoundaryIs>
<LinearRing>
<coordinates>
-0.89599999999996,48.037,0
-0.8943645864809,48.036999988395,0
-0.89437080670937,48.037095295047,0
-0.89438942615114,48.037189876531,0
-0.89442030316759,48.03728301302,0
-0.89446320282836,48.037373995679,0
-0.8945177986976,48.037462132063,0
-0.89458367531649,48.037546751383,0
-0.89466033136372,48.037627209617,0
-0.89474718346962,48.037702894409,0
-0.894843570655,48.037773229728,0
-0.89494875936127,48.037837680258,0
-0.89506194903277,48.037895755468,0
-0.89518227820999,48.037947013349,0
-0.89530883108608,48.037991063776,0
-0.89544064447773,48.03802757148,0
-0.89557671515674,48.0380562586,0
-0.89571600748662,48.038076906795,0
-0.8958574613062,48.038089358911,0
-0.89599999999995,48.038093520174,0

-0.89614253869371,48.038089358911,0
-0.89628399251329,48.038076906795,0
-0.89642328484317,48.0380562586,0
-0.89655935552218,48.03802757148,0
-0.89669116891383,48.037991063776,0
-0.89681772178994,48.037947013349,0
-0.89693805096714,48.037895755468,0
-0.89705124063867,48.037837680258,0
-0.8971564293449,48.037773229728,0
-0.89725281653031,48.037702894409,0
-0.89733966863619,48.037627209617,0
-0.89741632468342,48.037546751383,0
-0.89748220130233,48.037462132063,0
-0.89753679717158,48.037373995679,0
-0.8975796968323,48.03728301302,0
-0.89761057384876,48.037189876531,0
-0.89762919329054,48.037095295047,0
-0.89763541351904,48.036999988395,0
-0.89762918726316,48.036904681921,0
-0.89761056197715,48.03681010096,0
-0.89757967947718,48.036716965326,0
-0.89753677486021,48.036625983827,0
-0.89748217471269,48.036537848873,0
-0.89741629462341,48.03645323121,0
-0.89733963601917,48.036372774809,0
-0.89725278234734,48.036297091972,0
-0.8971563946346,48.036226758668,0
-0.8970512064557,48.036162310153,0
-0.89693801835015,48.036104236897,0
-0.89681769172994,48.036052980849,0
-0.89669114232421,48.036008932079,0
-0.89655933321084,48.035972425805,0
-0.89642326748803,48.035943739846,0
-0.89628398064168,48.035923092505,0
-0.89614253266633,48.035910640913,0
-0.89599999999995,48.035906479827,0
-0.89585746733358,48.035910640913,0
-0.89571601935826,48.035923092505,0
-0.8955767325119,48.035943739846,0
-0.8954406667891,48.035972425805,0
-0.89530885767572,48.036008932079,0
-0.89518230826997,48.036052980849,0
-0.89506198164979,48.036104236897,0
-0.89494879354424,48.036162310153,0
-0.89484360536531,48.036226758668,0
-0.8947472176526,48.036297091972,0
-0.89466036398074,48.036372774809,0
-0.8945837053765,48.03645323121,0
-0.89451782528722,48.036537848873,0
-0.89446322513969,48.036625983827,0
-0.89442032052273,48.036716965326,0
-0.89438943802278,48.03681010096,0
-0.89437081273675,48.036904681921,0
-0.8943645864809,48.036999988395,0
-0.89599999999996,48.037,0
</coordinates>
</LinearRing>

</outerBoundaryIs>
</Polygon>
</Placemark>
<Placemark>
<name>91</name>
<styleUrl>#stylehap1</styleUrl>
<Polygon>
<outerBoundaryIs>
<LinearRing>
<coordinates>
-0.88,48.11,0
-0.87836226471126,48.109999988366,0
-0.87836849376275,48.110095295017,0
-0.87838713963073,48.110189876502,0
-0.8784180604757,48.110283012992,0
-0.87846102103404,48.110373995653,0
-0.87851569440666,48.110462132038,0
-0.87858166454505,48.110546751361,0
-0.87865842941634,48.110627209597,0
-0.8787454048227,48.110702894391,0
-0.87884192884674,48.110773229713,0
-0.87894726688831,48.110837680245,0
-0.87906061725536,48.110895755458,0
-0.87918111726521,48.110947013341,0
-0.87930784981081,48.110991063771,0
-0.87943985034126,48.111027571477,0
-0.87957611420395,48.111056258598,0
-0.87971560429192,48.111076906794,0
-0.87985725893867,48.111089358911,0
-0.88,48.111093520174,0
-0.88014274106133,48.111089358911,0
-0.88028439570807,48.111076906794,0
-0.88042388579605,48.111056258598,0
-0.88056014965871,48.111027571477,0
-0.88069215018919,48.110991063771,0
-0.88081888273478,48.110947013341,0
-0.88093938274464,48.110895755458,0
-0.88105273311165,48.110837680245,0
-0.88115807115325,48.110773229713,0
-0.88125459517729,48.110702894391,0
-0.88134157058365,48.110627209597,0
-0.88141833545494,48.110546751361,0
-0.88148430559333,48.110462132038,0
-0.88153897896595,48.110373995653,0
-0.88158193952429,48.110283012992,0
-0.88161286036926,48.110189876502,0
-0.88163150623724,48.110095295017,0
-0.88163773528873,48.109999988366,0
-0.88163150018582,48.109904681891,0
-0.88161284845027,48.109810100931,0
-0.88158192209989,48.109716965298,0
-0.88153895656558,48.1096259838,0
-0.88148427889764,48.109537848849,0
-0.88141830527501,48.109453231188,0
-0.88134153783654,48.109372774789,0
-0.88125456085794,48.109297091954,0
-0.88115803630447,48.109226758653,0

-0.88105269879232,48.109162310141,0
-0.88093934999749,48.109104236887,0
-0.88081885255486,48.109052980842,0
-0.88069212349347,48.109008932074,0
-0.88056012725837,48.108972425801,0
-0.88042386837164,48.108943739844,0
-0.88028438378909,48.108923092504,0
-0.8801427350099,48.108910640913,0
-0.88,48.108906479827,0
-0.87985726499009,48.108910640913,0
-0.87971561621091,48.108923092504,0
-0.87957613162835,48.108943739844,0
-0.87943987274163,48.108972425801,0
-0.87930787650652,48.109008932074,0
-0.87918114744514,48.109052980842,0
-0.8790606500025,48.109104236887,0
-0.87894730120767,48.109162310141,0
-0.87884196369552,48.109226758653,0
-0.87874543914206,48.109297091954,0
-0.87865846216346,48.109372774789,0
-0.87858169472498,48.109453231188,0
-0.87851572110236,48.109537848849,0
-0.87846104343441,48.1096259838,0
-0.87841807790008,48.109716965298,0
-0.87838715154972,48.109810100931,0
-0.87836849981417,48.109904681891,0
-0.87836226471126,48.109999988366,0
-0.88,48.11,0
</coordinates>
</LinearRing>
</outerBoundaryIs>
</Polygon>
</Placemark>
<Placemark>
<name>93</name>
<styleUrl>#stylehap1</styleUrl>
<Polygon>
<outerBoundaryIs>
<LinearRing>
<coordinates>
-0.87900000000002,48.045,0
-0.87736433249117,48.044999988392,0
-0.87737055368486,48.045095295043,0
-0.87738917601752,48.045189876528,0
-0.87742005782856,48.045283013017,0
-0.87746296415122,48.045373995676,0
-0.87751756849893,48.04546213206,0
-0.87758345534839,48.045546751381,0
-0.87766012330044,48.045627209615,0
-0.87774698889477,48.045702894407,0
-0.8778433910496,48.045773229726,0
-0.87794859609238,48.045837680256,0
-0.87806180334317,48.045895755467,0
-0.87818215120859,48.045947013348,0
-0.87830872373962,48.045991063775,0
-0.87844055760331,48.04602757148,0
-0.87857664941562,48.0460562586,0

-0.87871596337923,48.046076906795,0
-0.8788574391683,48.046089358911,0
-0.87900000000001,48.046093520174,0
-0.87914256083175,48.046089358911,0
-0.87928403662079,48.046076906795,0
-0.87942335058441,48.0460562586,0
-0.87955944239672,48.04602757148,0
-0.87969127626043,48.045991063775,0
-0.87981784879143,48.045947013348,0
-0.87993819665688,48.045895755467,0
-0.88005140390767,48.045837680256,0
-0.88015660895043,48.045773229726,0
-0.88025301110528,48.045702894407,0
-0.88033987669961,48.045627209615,0
-0.88041654465163,48.045546751381,0
-0.88048243150109,48.04546213206,0
-0.88053703584883,48.045373995676,0
-0.88057994217149,48.045283013017,0
-0.88061082398253,48.045189876528,0
-0.88062944631519,48.045095295043,0
-0.88063566750888,48.044999988392,0
-0.88062944028519,48.044904681917,0
-0.88061081210572,48.044810100957,0
-0.88057992480876,48.044716965323,0
-0.88053701352772,48.044625983824,0
-0.88048240489987,48.044537848871,0
-0.88041651457852,48.044453231208,0
-0.88033984406836,48.044372774807,0
-0.8802529769074,48.04429709197,0
-0.880156574225,48.044226758666,0
-0.88005136970978,48.044162310152,0
-0.87993816402564,48.044104236896,0
-0.87981781871832,48.044052980848,0
-0.87969124965919,48.044008932078,0
-0.87955942007564,48.043972425804,0
-0.87942333322168,48.043943739845,0
-0.87928402474399,48.043923092505,0
-0.87914255480175,48.043910640913,0
-0.87900000000001,48.043906479827,0
-0.8788574451983,48.043910640913,0
-0.87871597525603,48.043923092505,0
-0.87857666677834,48.043943739845,0
-0.87844057992439,48.043972425804,0
-0.87830875034086,48.044008932078,0
-0.8781821812817,48.044052980848,0
-0.87806183597441,48.044104236896,0
-0.87794863029027,48.044162310152,0
-0.87784342577505,48.044226758666,0
-0.87774702309265,48.04429709197,0
-0.87766015593166,48.044372774807,0
-0.8775834854215,48.044453231208,0
-0.87751759510018,48.044537848871,0
-0.8774629864723,48.044625983824,0
-0.87742007519129,48.044716965323,0
-0.87738918789432,48.044810100957,0
-0.87737055971486,48.044904681917,0
-0.87736433249117,48.044999988392,0

-0.87900000000002,48.045,0
</coordinates>
</LinearRing>
</outerBoundaryIs>
</Polygon>
</Placemark>
<Placemark>
<name>96</name>
<styleUrl>#stylehap1</styleUrl>
<Polygon>
<outerBoundaryIs>
<LinearRing>
<coordinates>
-0.87599999999998,48.452,0
-0.87435126318573,48.451999988225,0
-0.87435753404421,48.452095294877,0
-0.87437630513056,48.452189876365,0
-0.87440743365353,48.452283012861,0
-0.87445068277017,48.452373995529,0
-0.8745057233863,48.452462131923,0
-0.87457213665944,48.452546751255,0
-0.87464941718494,48.452627209503,0
-0.87473697684108,48.452702894309,0
-0.8748341492643,48.452773229642,0
-0.8749401949199,48.452837680187,0
-0.87505430673028,48.452895755412,0
-0.87517561621741,48.452947013306,0
-0.87530320011309,48.452991063745,0
-0.87543608738637,48.45302757146,0
-0.87557326663506,48.453056258588,0
-0.87571369378446,48.45307690679,0
-0.87585630003536,48.45308935891,0
-0.87599999999998,48.453093520174,0
-0.87614369996458,48.45308935891,0
-0.87628630621548,48.45307690679,0
-0.87642673336491,48.453056258588,0
-0.8765639126136,48.45302757146,0
-0.87669679988687,48.452991063745,0
-0.87682438378253,48.452947013306,0
-0.87694569326968,48.452895755412,0
-0.87705980508004,48.452837680187,0
-0.87716585073564,48.452773229642,0
-0.87726302315885,48.452702894309,0
-0.87735058281502,48.452627209503,0
-0.8774278633405,48.452546751255,0
-0.87749427661367,48.452462131923,0
-0.8775493172298,48.452373995529,0
-0.87759256634643,48.452283012861,0
-0.87762369486941,48.452189876365,0
-0.87764246595572,48.452095294877,0
-0.8776487368142,48.451999988225,0
-0.87764245979,48.451904681752,0
-0.87762368272528,48.451810100795,0
-0.87759254859293,48.451716965167,0
-0.87754929440634,48.451625983676,0
-0.87749424941371,48.451537848733,0
-0.87742783259054,48.451453231082,0

-0.87735054944935,48.451372774695,0
-0.87726298819128,48.451297091872,0
-0.87716581522864,48.451226758583,0
-0.87705977011248,48.451162310083,0
-0.87694565990401,48.451104236841,0
-0.87682435303257,48.451052980807,0
-0.87669677268692,48.451008932048,0
-0.87656388979011,48.450972425785,0
-0.87642671561141,48.450943739834,0
-0.87628629407138,48.4509230925,0
-0.87614369379886,48.450910640911,0
-0.87599999999998,48.450906479827,0
-0.8758563062011,48.450910640911,0
-0.87571370592858,48.4509230925,0
-0.87557328438856,48.450943739834,0
-0.87543611020982,48.450972425785,0
-0.87530322731302,48.451008932048,0
-0.87517564696739,48.451052980807,0
-0.87505434009596,48.451104236841,0
-0.87494022988748,48.451162310083,0
-0.8748341847713,48.451226758583,0
-0.87473701180866,48.451297091872,0
-0.87464945055062,48.451372774695,0
-0.87457216740942,48.451453231082,0
-0.87450575058625,48.451537848733,0
-0.87445070559362,48.451625983676,0
-0.87440745140704,48.451716965167,0
-0.87437631727465,48.451810100795,0
-0.87435754020993,48.451904681752,0
-0.87435126318573,48.451999988225,0
-0.87599999999998,48.452,0
</coordinates>
</LinearRing>
</outerBoundaryIs>
</Polygon>
</Placemark>
<Placemark>
<name>97</name>
<styleUrl>#stylehap1</styleUrl>
<Polygon>
<outerBoundaryIs>
<LinearRing>
<coordinates>
-0.875,48.188,0
-0.87335977367331,48.187999988334,0
-0.87336601219104,48.188095294985,0
-0.87338468641183,48.188189876471,0
-0.87341565428065,48.188283012962,0
-0.87345868017628,48.188373995625,0
-0.87351343670259,48.188462132012,0
-0.8735795071785,48.188546751337,0
-0.87365638880762,48.188627209576,0
-0.87374349650368,48.188702894372,0
-0.87384016734253,48.188773229697,0
-0.87394566560676,48.188837680232,0
-0.87405918838483,48.188895755447,0
-0.87417987168183,48.188947013333,0

-0.87430679699571,48.188991063765,0
-0.87443899830832,48.189027571473,0
-0.8745754694387,48.189056258596,0
-0.87471517170228,48.189076906793,0
-0.87485704181749,48.18908935891,0
-0.875,48.189093520174,0
-0.8751429581825,48.18908935891,0
-0.87528482829771,48.189076906793,0
-0.8754245305613,48.189056258596,0
-0.87556100169167,48.189027571473,0
-0.87569320300429,48.188991063765,0
-0.87582012831816,48.188947013333,0
-0.87594081161519,48.188895755447,0
-0.87605433439323,48.188837680232,0
-0.87615983265746,48.188773229697,0
-0.87625650349631,48.188702894372,0
-0.87634361119237,48.188627209576,0
-0.87642049282149,48.188546751337,0
-0.8764865632974,48.188462132012,0
-0.87654131982371,48.188373995625,0
-0.87658434571935,48.188283012962,0
-0.87661531358818,48.188189876471,0
-0.87663398780895,48.188095294985,0
-0.87664022632669,48.187999988334,0
-0.8766339817317,48.18790468186,0
-0.87661530161833,48.1878101009,0
-0.8765843282206,48.187716965268,0
-0.87654129732775,48.187625983772,0
-0.87648653648774,48.187537848823,0
-0.87642046251276,48.187453231164,0
-0.87634357830546,48.187372774768,0
-0.87625646903047,48.187297091936,0
-0.87615979765993,48.187226758637,0
-0.87605429992742,48.187162310128,0
-0.87594077872828,48.187104236876,0
-0.87582009800941,48.187052980834,0
-0.87569317619463,48.187008932068,0
-0.87556097919571,48.186972425798,0
-0.87542451306252,48.186943739841,0
-0.87528481632786,48.186923092503,0
-0.87514295210525,48.186910640912,0
-0.875,48.186906479826,0
-0.87485704789474,48.186910640912,0
-0.87471518367214,48.186923092503,0
-0.87457548693747,48.186943739841,0
-0.87443902080428,48.186972425798,0
-0.87430682380537,48.187008932068,0
-0.87417990199058,48.187052980834,0
-0.87405922127174,48.187104236876,0
-0.8739457000726,48.187162310128,0
-0.87384020234006,48.187226758637,0
-0.87374353096952,48.187297091936,0
-0.87365642169453,48.187372774768,0
-0.87357953748725,48.187453231164,0
-0.87351346351225,48.187537848823,0
-0.87345870267224,48.187625983772,0
-0.8734156717794,48.187716965268,0

-0.87338469838169,48.1878101009,0
-0.87336601826832,48.18790468186,0
-0.87335977367331,48.187999988334,0
-0.875,48.188,0
</coordinates>
</LinearRing>
</outerBoundaryIs>
</Polygon>
</Placemark>
<Placemark>
<name>98</name>
<styleUrl>#stylehap1</styleUrl>
<Polygon>
<outerBoundaryIs>
<LinearRing>
<coordinates>
-0.875,48.048,0
-0.87336423721642,48.047999988391,0
-0.87337045877215,48.048095295042,0
-0.87338908218924,48.048189876526,0
-0.87341996579879,48.048283013016,0
-0.87346287462042,48.048373995675,0
-0.87351748214851,48.048462132059,0
-0.87358337283558,48.04854675138,0
-0.87366004525325,48.048627209614,0
-0.87374691590726,48.048702894406,0
-0.87384332367734,48.048773229725,0
-0.87394853484814,48.048837680256,0
-0.87406174869313,48.048895755466,0
-0.87418210356875,48.048947013348,0
-0.87430868347258,48.048991063775,0
-0.87444052501558,48.04902757148,0
-0.87457662475526,48.049056258599,0
-0.87471594683397,48.049076906795,0
-0.87485743086403,48.049089358911,0
-0.875,48.049093520174,0
-0.87514256913596,48.049089358911,0
-0.87528405316602,48.049076906795,0
-0.87542337524473,48.049056258599,0
-0.87555947498441,48.04902757148,0
-0.87569131652741,48.048991063775,0
-0.87581789643124,48.048947013348,0
-0.87593825130686,48.048895755466,0
-0.87605146515185,48.048837680256,0
-0.87615667632265,48.048773229725,0
-0.87625308409274,48.048702894406,0
-0.87633995474674,48.048627209614,0
-0.87641662716442,48.04854675138,0
-0.87648251785148,48.048462132059,0
-0.8765371253796,48.048373995675,0
-0.8765800342012,48.048283013016,0
-0.87661091781076,48.048189876526,0
-0.87662954122784,48.048095295042,0
-0.87663576278357,48.047999988391,0
-0.87662953519685,48.047904681916,0
-0.87661090593202,48.047810100956,0
-0.87658001683563,48.047716965322,0

-0.87653710305485,48.047625983823,0
-0.87648249124591,48.04753784887,0
-0.87641659708639,48.047453231207,0
-0.87633992211018,48.047372774806,0
-0.87625304988925,48.047297091969,0
-0.87615664159153,48.047226758666,0
-0.87605143094837,48.047162310151,0
-0.8759382186703,48.047104236895,0
-0.87581786635322,48.047052980848,0
-0.87569128992184,48.047008932078,0
-0.87555945265967,48.046972425804,0
-0.87542335787916,48.046943739845,0
-0.87528404128729,48.046923092505,0
-0.87514256310497,48.046910640913,0
-0.875,48.046906479827,0
-0.87485743689503,48.046910640913,0
-0.8747159587127,48.046923092505,0
-0.87457664212084,48.046943739845,0
-0.87444054734033,48.046972425804,0
-0.87430871007815,48.047008932078,0
-0.87418213364677,48.047052980848,0
-0.87406178132972,48.047104236895,0
-0.87394856905162,48.047162310151,0
-0.87384335840846,48.047226758666,0
-0.87374695011074,48.047297091969,0
-0.87366007788982,48.047372774806,0
-0.87358340291363,48.047453231207,0
-0.87351750875411,48.04753784887,0
-0.8734628969451,48.047625983823,0
-0.87341998316437,48.047716965322,0
-0.87338909406797,48.047810100956,0
-0.87337046480315,48.047904681916,0
-0.87336423721642,48.047999988391,0
-0.875,48.048,0
</coordinates>
</LinearRing>
</outerBoundaryIs>
</Polygon>
</Placemark>
<Placemark>
<name>99</name>
<styleUrl>#stylehap1</styleUrl>
<Polygon>
<outerBoundaryIs>
<LinearRing>
<coordinates>
-0.87200000000007,48.192,0
-0.87035964564157,48.191999988332,0
-0.87036588464586,48.192095294984,0
-0.87038456032385,48.192189876469,0
-0.87041553060956,48.192283012961,0
-0.87045855986335,48.192373995623,0
-0.87051332066351,48.192462132011,0
-0.87057939629647,48.192546751336,0
-0.87065628392659,48.192627209575,0
-0.87074339842194,48.192702894371,0
-0.87084007680661,48.192773229696,0

-0.87094558330581,48.192837680231,0
-0.87105911494526,48.192895755447,0
-0.8711798076627,48.192947013333,0
-0.87130674288426,48.192991063765,0
-0.87143895451648,48.193027571473,0
-0.87157543629981,48.193056258596,0
-0.87171514946855,48.193076906793,0
-0.87185703065819,48.19308935891,0
-0.87200000000007,48.193093520174,0
-0.87214296934194,48.19308935891,0
-0.87228485053159,48.193076906793,0
-0.87242456370036,48.193056258596,0
-0.87256104548368,48.193027571473,0
-0.87269325711587,48.192991063765,0
-0.87282019233746,48.192947013333,0
-0.87294088505488,48.192895755447,0
-0.87305441669433,48.192837680231,0
-0.87315992319352,48.192773229696,0
-0.8732566015782,48.192702894371,0
-0.87334371607355,48.192627209575,0
-0.87342060370369,48.192546751336,0
-0.87348667933662,48.192462132011,0
-0.87354144013679,48.192373995623,0
-0.87358446939058,48.192283012961,0
-0.87361543967628,48.192189876469,0
-0.87363411535431,48.192095294984,0
-0.87364035435856,48.191999988332,0
-0.87363410927571,48.191904681858,0
-0.87361542770381,48.191810100899,0
-0.87358445188799,48.191716965267,0
-0.87354141763592,48.191625983771,0
-0.87348665252111,48.191537848821,0
-0.87342057338832,48.191453231162,0
-0.87334368317947,48.191372774767,0
-0.87325656710483,48.191297091935,0
-0.87315988818836,48.191226758636,0
-0.87305438222098,48.191162310127,0
-0.87294085216079,48.191104236876,0
-0.87282016202209,48.191052980833,0
-0.87269323030036,48.191008932068,0
-0.87256102298279,48.190972425797,0
-0.87242454619776,48.190943739841,0
-0.87228483855911,48.190923092503,0
-0.87214296326337,48.190910640912,0
-0.87200000000007,48.190906479826,0
-0.87185703673677,48.190910640912,0
-0.87171516144102,48.190923092503,0
-0.87157545380237,48.190943739841,0
-0.87143897701735,48.190972425797,0
-0.87130676969978,48.191008932068,0
-0.87117983797805,48.191052980833,0
-0.87105914783937,48.191104236876,0
-0.87094561777915,48.191162310127,0
-0.8708401118118,48.191226758636,0
-0.87074343289531,48.191297091935,0
-0.87065631682067,48.191372774767,0
-0.87057942661182,48.191453231162,0

-0.87051334747902,48.191537848821,0
-0.87045858236422,48.191625983771,0
-0.87041554811215,48.191716965267,0
-0.87038457229633,48.191810100899,0
-0.87036589072443,48.191904681858,0
-0.87035964564157,48.191999988332,0
-0.87200000000007,48.192,0
</coordinates>
</LinearRing>
</outerBoundaryIs>
</Polygon>
</Placemark>
<Placemark>
<name>100</name>
<styleUrl>#stylehap1</styleUrl>
<Polygon>
<outerBoundaryIs>
<LinearRing>
<coordinates>
-0.87200000000007,48.148,0
-0.87036105245404,48.14799998835,0
-0.87036728611226,48.148095295002,0
-0.87038594577805,48.148189876487,0
-0.87041688950706,48.148283012978,0
-0.87045988186164,48.148373995639,0
-0.87051459570079,48.148462132026,0
-0.8705806146682,48.148546751349,0
-0.87065743635938,48.148627209587,0
-0.87074447614419,48.148702894382,0
-0.87084107161527,48.148773229705,0
-0.8709464876288,48.148837680239,0
-0.87105992189923,48.148895755453,0
-0.87118051110532,48.148947013337,0
-0.8713073374611,48.148991063768,0
-0.87143943570168,48.149027571475,0
-0.87157580043067,48.149056258597,0
-0.87171539377334,48.149076906794,0
-0.87185715327728,48.149089358911,0
-0.87200000000007,48.149093520174,0
-0.87214284672286,48.149089358911,0
-0.8722846062268,48.149076906794,0
-0.87242419956949,48.149056258597,0
-0.87256056429846,48.149027571475,0
-0.87269266253904,48.148991063768,0
-0.87281948889484,48.148947013337,0
-0.87294007810091,48.148895755453,0
-0.87305351237134,48.148837680239,0
-0.87315892838487,48.148773229705,0
-0.87325552385595,48.148702894382,0
-0.87334256364078,48.148627209587,0
-0.87341938533196,48.148546751349,0
-0.87348540429935,48.148462132026,0
-0.8735401181385,48.148373995639,0
-0.87358311049307,48.148283012978,0
-0.87361405422208,48.148189876487,0
-0.87363271388787,48.148095295002,0
-0.8736389475461,48.14799998835,0

-0.87363270782388,48.147904681876,0
-0.87361404227836,48.147810100916,0
-0.87358309303251,48.147716965284,0
-0.87354009569162,48.147625983787,0
-0.87348537754821,48.147537848836,0
-0.87341935508937,48.147453231176,0
-0.87334253082563,48.147372774779,0
-0.87325548946535,48.147297091945,0
-0.87315889346373,48.147226758645,0
-0.87305347798074,48.147162310134,0
-0.87294004528577,48.147104236882,0
-0.87281945865224,48.147052980838,0
-0.8726926357879,48.147008932071,0
-0.87256054185158,48.1469724258,0
-0.87242418210891,48.146943739842,0
-0.87228459428308,48.146923092504,0
-0.87214284065886,48.146910640912,0
-0.87200000000007,48.146906479826,0
-0.87185715934127,48.146910640912,0
-0.87171540571709,48.146923092504,0
-0.87157581789123,48.146943739842,0
-0.87143945814856,48.1469724258,0
-0.87130736421224,48.147008932071,0
-0.87118054134792,48.147052980838,0
-0.87105995471436,48.147104236882,0
-0.87094652201942,48.147162310134,0
-0.87084110653641,48.147226758645,0
-0.87074451053479,48.147297091945,0
-0.87065746917451,48.147372774779,0
-0.87058064491077,48.147453231176,0
-0.87051462245195,48.147537848836,0
-0.87045990430852,48.147625983787,0
-0.87041690696765,48.147716965284,0
-0.8703859577218,48.147810100916,0
-0.87036729217626,48.147904681876,0
-0.87036105245404,48.14799998835,0
-0.87200000000007,48.148,0
</coordinates>
</LinearRing>
</outerBoundaryIs>
</Polygon>
</Placemark>
<Placemark>
<name>102</name>
<styleUrl>#stylehap1</styleUrl>
<Polygon>
<outerBoundaryIs>
<LinearRing>
<coordinates>
-0.86900000000003,48.447,0
-0.86735142551537,48.446999988227,0
-0.86735769575698,48.447095294879,0
-0.8673764649957,48.447189876367,0
-0.86740759045435,48.447283012863,0
-0.86745083531324,48.447373995531,0
-0.86750587051064,48.447462131925,0
-0.86757227724525,48.447546751257,0

-0.86764955016218,48.447627209504,0
-0.86773710119762,48.44770289431,0
-0.86783426405358,48.447773229643,0
-0.86794029926821,48.447837680188,0
-0.86805439984334,48.447895755412,0
-0.86817569738647,48.447947013307,0
-0.86830326872031,48.447991063746,0
-0.86843614290954,48.448027571461,0
-0.86857330865151,48.448056258589,0
-0.86871372197443,48.44807690679,0
-0.86885631418423,48.44808935891,0
-0.86900000000004,48.448093520174,0
-0.86914368581585,48.44808935891,0
-0.86928627802564,48.44807690679,0
-0.86942669134854,48.448056258589,0
-0.86956385709053,48.448027571461,0
-0.86969673127974,48.447991063746,0
-0.86982430261358,48.447947013307,0
-0.86994560015673,48.447895755412,0
-0.87005970073187,48.447837680188,0
-0.87016573594647,48.447773229643,0
-0.87026289880245,48.44770289431,0
-0.87035044983789,48.447627209504,0
-0.8704277227548,48.447546751257,0
-0.87049412948943,48.447462131925,0
-0.87054916468681,48.447373995531,0
-0.87059240954572,48.447283012863,0
-0.87062353500435,48.447189876367,0
-0.87064230424307,48.447095294879,0
-0.87064857448468,48.446999988227,0
-0.87064229807903,48.446904681754,0
-0.87062352286359,48.446810100797,0
-0.87059239179708,48.446716965169,0
-0.87054914186961,48.446625983678,0
-0.87049410229693,48.446537848735,0
-0.87042769201326,48.446453231084,0
-0.87035041648135,48.446372774696,0
-0.87026286384447,48.446297091873,0
-0.87016570044922,48.446226758584,0
-0.87005966577388,48.446162310083,0
-0.8699455668002,48.446104236841,0
-0.86982427187205,48.446052980807,0
-0.86969670408727,48.446008932049,0
-0.86956383427331,48.445972425785,0
-0.8694266735999,48.445943739834,0
-0.86928626588485,48.4459230925,0
-0.86914367965181,48.445910640911,0
-0.86900000000004,48.445906479826,0
-0.86885632034824,48.445910640911,0
-0.8687137341152,48.4459230925,0
-0.86857332640015,48.445943739834,0
-0.86843616572674,48.445972425785,0
-0.86830329591278,48.446008932049,0
-0.868175728128,48.446052980807,0
-0.86805443319985,48.446104236841,0
-0.86794033422619,48.446162310083,0
-0.86783429955083,48.446226758584,0

-0.86773713615561,48.446297091873,0
-0.8676495835187,48.446372774696,0
-0.86757230798679,48.446453231084,0
-0.86750589770312,48.446537848735,0
-0.86745085813046,48.446625983678,0
-0.86740760820297,48.446716965169,0
-0.86737647713649,48.446810100797,0
-0.86735770192102,48.446904681754,0
-0.86735142551537,48.446999988227,0
-0.86900000000003,48.447,0
</coordinates>
</LinearRing>
</outerBoundaryIs>
</Polygon>
</Placemark>
<Placemark>
<name>103</name>
<styleUrl>#stylehap1</styleUrl>
<Polygon>
<outerBoundaryIs>
<LinearRing>
<coordinates>
-0.86699999999996,47.91,0
-0.86536860377612,47.909999988447,0
-0.86537480873837,47.910095295098,0
-0.86539338245557,47.910189876581,0
-0.86542418363662,47.910283013068,0
-0.86546697792793,47.910373995725,0
-0.86552143969519,47.910462132105,0
-0.86558715449982,47.910546751422,0
-0.86566362225176,47.910627209652,0
-0.86575026101411,47.910702894439,0
-0.86584641143135,47.910773229753,0
-0.86595134174667,47.910837680279,0
-0.86606425337108,47.910895755485,0
-0.86618428696119,47.910947013362,0
-0.86631052896005,47.910991063785,0
-0.86644201855052,47.911027571486,0
-0.86657775496908,47.911056258603,0
-0.86671670512354,47.911076906797,0
-0.86685781145734,47.911089358911,0
-0.86699999999997,47.911093520174,0
-0.86714218854259,47.911089358911,0
-0.86728329487637,47.911076906797,0
-0.86742224503086,47.911056258603,0
-0.86755798144939,47.911027571486,0
-0.86768947103986,47.910991063785,0
-0.86781571303872,47.910947013362,0
-0.86793574662885,47.910895755485,0
-0.86804865825326,47.910837680279,0
-0.86815358856859,47.910773229753,0
-0.86824973898579,47.910702894439,0
-0.86833637774818,47.910627209652,0
-0.86841284550011,47.910546751422,0
-0.86847856030474,47.910462132105,0
-0.86853302207198,47.910373995725,0
-0.86857581636331,47.910283013068,0

-0.86860661754437,47.910189876581,0
-0.86862519126157,47.910095295098,0
-0.86863139622379,47.909999988447,0
-0.86862518527574,47.909904681972,0
-0.86860660575456,47.90981010101,0
-0.86857579912778,47.909716965374,0
-0.86853299991443,47.909625983872,0
-0.86847853389838,47.909537848916,0
-0.8684128156473,47.909453231249,0
-0.86833634535598,47.909372774844,0
-0.86824970503846,47.909297092002,0
-0.86815355409753,47.909226758694,0
-0.8680486243059,47.909162310174,0
-0.86793571423666,47.909104236914,0
-0.86781568318593,47.909052980862,0
-0.86768944463352,47.909008932088,0
-0.86755795929184,47.908972425811,0
-0.86742222779533,47.908943739849,0
-0.86728328308658,47.908923092507,0
-0.86714218255676,47.908910640913,0
-0.86699999999997,47.908906479827,0
-0.86685781744317,47.908910640913,0
-0.86671671691335,47.908923092507,0
-0.86657777220461,47.908943739849,0
-0.8664420407081,47.908972425811,0
-0.86631055536641,47.909008932088,0
-0.86618431681401,47.909052980862,0
-0.86606428576325,47.909104236914,0
-0.86595137569403,47.909162310174,0
-0.8658464459024,47.909226758694,0
-0.86575029496147,47.909297092002,0
-0.86566365464395,47.909372774844,0
-0.86558718435264,47.909453231249,0
-0.86552146610156,47.909537848916,0
-0.86546700008551,47.909625983872,0
-0.86542420087212,47.909716965374,0
-0.86539339424535,47.90981010101,0
-0.8653748147242,47.909904681972,0
-0.86536860377612,47.909999988447,0
-0.86699999999996,47.91,0
</coordinates>
</LinearRing>
</outerBoundaryIs>
</Polygon>
</Placemark>
<Placemark>
<name>104</name>
<styleUrl>#stylehap1</styleUrl>
<Polygon>
<outerBoundaryIs>
<LinearRing>
<coordinates>
-0.86400000000003,48.076,0
-0.86236334723365,48.07599998838,0
-0.86236957217143,48.076095295031,0
-0.86238820571823,48.076189876515,0
-0.86241910612821,48.076283013005,0

-0.86246203829313,48.076373995665,0
-0.86251667552989,48.07646213205,0
-0.86258260206493,48.076546751371,0
-0.86265931619711,48.076627209607,0
-0.86274623411479,48.076702894399,0
-0.86284269433798,48.07677322972,0
-0.86294796275212,48.076837680251,0
-0.86306123819506,48.076895755463,0
-0.86318165855441,48.076947013345,0
-0.86330830732931,48.076991063773,0
-0.86344022060652,48.077027571478,0
-0.86357639439755,48.077056258599,0
-0.86371579228104,48.077076906795,0
-0.86385735329236,48.077089358911,0
-0.86400000000004,48.077093520173,0
-0.86414264670772,48.077089358911,0
-0.86428420771904,48.077076906795,0
-0.86442360560253,48.077056258599,0
-0.86455977939353,48.077027571478,0
-0.86469169267076,48.076991063773,0
-0.86481834144566,48.076947013345,0
-0.86493876180499,48.076895755463,0
-0.86505203724793,48.076837680251,0
-0.8651573056621,48.07677322972,0
-0.86525376588528,48.076702894399,0
-0.86534068380294,48.076627209607,0
-0.86541739793514,48.076546751371,0
-0.86548332447019,48.07646213205,0
-0.86553796170695,48.076373995665,0
-0.86558089387187,48.076283013005,0
-0.86561179428182,48.076189876515,0
-0.86563042782862,48.076095295031,0
-0.8656366527664,48.07599998838,0
-0.86563042178841,48.075904681905,0
-0.86561178238495,48.075810100945,0
-0.86558087647978,48.075716965311,0
-0.86553793934808,48.075625983813,0
-0.86548329782396,48.07553784886,0
-0.86541736781114,48.075453231198,0
-0.86534065111651,48.075372774798,0
-0.86525373162954,48.075297091962,0
-0.8651572708779,48.07522675866,0
-0.86505200299219,48.075162310146,0
-0.86493872911855,48.075104236891,0
-0.86481831132166,48.075052980845,0
-0.86469166602453,48.075008932076,0
-0.86455975703469,48.074972425803,0
-0.86442358821044,48.074943739844,0
-0.86428419582214,48.074923092505,0
-0.86414264066751,48.074910640913,0
-0.86400000000004,48.074906479826,0
-0.86385735933256,48.074910640913,0
-0.86371580417791,48.074923092505,0
-0.86357641178964,48.074943739844,0
-0.86344024296536,48.074972425803,0
-0.86330833397554,48.075008932076,0
-0.86318168867839,48.075052980845,0

-0.86306127088152,48.075104236891,0
-0.86294799700786,48.075162310146,0
-0.86284272912215,48.07522675866,0
-0.86274626837054,48.075297091962,0
-0.86265934888357,48.075372774798,0
-0.86258263218891,48.075453231198,0
-0.86251670217612,48.07553784886,0
-0.86246206065197,48.075625983813,0
-0.8624191235203,48.075716965311,0
-0.86238821761513,48.075810100945,0
-0.86236957821164,48.075904681905,0
-0.86236334723365,48.07599998838,0
-0.86400000000003,48.076,0
</coordinates>
</LinearRing>
</outerBoundaryIs>
</Polygon>
</Placemark>
<Placemark>
<name>106</name>
<styleUrl>#stylehap1</styleUrl>
<Polygon>
<outerBoundaryIs>
<LinearRing>
<coordinates>
-0.85400000000004,48.056,0
-0.85236398307432,48.055999988388,0
-0.85237020559582,48.056095295039,0
-0.85238883190553,48.056189876523,0
-0.85241972031258,48.056283013013,0
-0.85246263580009,48.056373995672,0
-0.85251725181174,48.056462132057,0
-0.85258315273551,48.056546751377,0
-0.8526598370651,48.056627209612,0
-0.85274672121565,48.056702894404,0
-0.85284314396416,48.056773229724,0
-0.85294837148131,48.056837680254,0
-0.85306160291611,48.056895755465,0
-0.85318197649116,48.056947013347,0
-0.85330857606171,48.056991063774,0
-0.85344043808903,48.057027571479,0
-0.85357655897469,48.057056258599,0
-0.8537159027001,48.057076906795,0
-0.85385740871281,48.057089358911,0
-0.85400000000004,48.057093520174,0
-0.85414259128727,48.057089358911,0
-0.85428409729998,48.057076906795,0
-0.85442344102539,48.057056258599,0
-0.85455956191105,48.057027571479,0
-0.85469142393837,48.056991063774,0
-0.85481802350892,48.056947013347,0
-0.85493839708396,48.056895755465,0
-0.8550516285188,48.056837680254,0
-0.85515685603592,48.056773229724,0
-0.85525327878443,48.056702894404,0
-0.85534016293498,48.056627209612,0
-0.85541684726459,48.056546751377,0

-0.85548274818834,48.056462132057,0
-0.85553736420001,48.056373995672,0
-0.8555802796875,48.056283013013,0
-0.85561116809455,48.056189876523,0
-0.85562979440426,48.056095295039,0
-0.85563601692579,48.055999988388,0
-0.85562978837064,48.055904681913,0
-0.85561115621062,48.055810100953,0
-0.85558026231437,48.055716965319,0
-0.85553734186552,48.05562598382,0
-0.85548272157116,48.055537848867,0
-0.85541681717341,48.055453231204,0
-0.85534013028416,48.055372774804,0
-0.85525324456601,48.055297091967,0
-0.85515682128965,48.055226758664,0
-0.85505159430038,48.05516231015,0
-0.85493836443315,48.055104236894,0
-0.85481799341777,48.055052980847,0
-0.8546913973212,48.055008932077,0
-0.85455953957658,48.054972425804,0
-0.85442342365226,48.054943739845,0
-0.85428408541605,48.054923092505,0
-0.85414258525365,48.054910640913,0
-0.85400000000004,48.054906479826,0
-0.85385741474645,48.054910640913,0
-0.85371591458403,48.054923092505,0
-0.85357657634785,48.054943739845,0
-0.85344046042349,48.054972425804,0
-0.8533086026789,48.055008932077,0
-0.85318200658231,48.055052980847,0
-0.85306163556692,48.055104236894,0
-0.8529484056997,48.05516231015,0
-0.85284317871045,48.055226758664,0
-0.85274675543407,48.055297091967,0
-0.85265986971594,48.055372774804,0
-0.85258318282666,48.055453231204,0
-0.85251727842894,48.055537848867,0
-0.85246265813456,48.05562598382,0
-0.85241973768571,48.055716965319,0
-0.85238884378945,48.055810100953,0
-0.85237021162943,48.055904681913,0
-0.85236398307432,48.055999988388,0
-0.85400000000004,48.056,0
</coordinates>
</LinearRing>
</outerBoundaryIs>
</Polygon>
</Placemark>
<Placemark>
<name>107</name>
<styleUrl>#stylehap1</styleUrl>
<Polygon>
<outerBoundaryIs>
<LinearRing>
<coordinates>
-0.85300000000007,48.15,0
-0.85136098858134,48.149999988349,0

-0.85136722248228,48.150095295001,0
-0.85138588287506,48.150189876486,0
-0.85141682780981,48.150283012977,0
-0.8514598218397,48.150373995639,0
-0.85151453781099,48.150462132025,0
-0.85158055935114,48.150546751349,0
-0.85165738403611,48.150627209586,0
-0.85174442721297,48.150702894381,0
-0.85184102644853,48.150773229705,0
-0.85194644657034,48.150837680238,0
-0.85205988526154,48.150895755453,0
-0.8521804791673,48.150947013337,0
-0.85230731046586,48.150991063768,0
-0.85243941385469,48.151027571475,0
-0.85257578389824,48.151056258597,0
-0.85271538268131,48.151076906794,0
-0.85285714771007,48.151089358911,0
-0.85300000000005,48.151093520174,0
-0.85314285229006,48.151089358911,0
-0.85328461731883,48.151076906794,0
-0.8534242161019,48.151056258597,0
-0.85356058614544,48.151027571475,0
-0.85369268953427,48.150991063768,0
-0.85381952083284,48.150947013337,0
-0.85394011473859,48.150895755453,0
-0.85405355342979,48.150837680238,0
-0.85415897355158,48.150773229705,0
-0.85425557278716,48.150702894381,0
-0.85434261596402,48.150627209586,0
-0.854419440649,48.150546751349,0
-0.85448546218915,48.150462132025,0
-0.85454017816044,48.150373995639,0
-0.85458317219032,48.150283012977,0
-0.85461411712507,48.150189876486,0
-0.85463277751786,48.150095295001,0
-0.8546390114188,48.149999988349,0
-0.8546327714532,48.149904681875,0
-0.85461410518003,48.149810100915,0
-0.85458315472786,48.149716965283,0
-0.85454015571111,48.149625983786,0
-0.85448543543506,48.149537848835,0
-0.85441941040309,48.149453231175,0
-0.8543425831453,48.149372774778,0
-0.8542555383928,48.149297091945,0
-0.85415893862665,48.149226758645,0
-0.85405351903542,48.149162310134,0
-0.85394008191987,48.149104236882,0
-0.85381949058693,48.149052980838,0
-0.85369266278021,48.149008932071,0
-0.85356056369612,48.148972425799,0
-0.85342419863943,48.148943739842,0
-0.85328460537378,48.148923092504,0
-0.85314284622541,48.148910640912,0
-0.85300000000005,48.148906479826,0
-0.85285715377473,48.148910640912,0
-0.85271539462635,48.148923092504,0
-0.8525758013607,48.148943739842,0

-0.85243943630401,48.148972425799,0
-0.85230733721992,48.149008932071,0
-0.8521805094132,48.149052980838,0
-0.85205991808026,48.149104236882,0
-0.85194648096471,48.149162310134,0
-0.85184106137348,48.149226758645,0
-0.85174446160733,48.149297091945,0
-0.85165741685483,48.149372774778,0
-0.85158058959704,48.149453231175,0
-0.85151456456508,48.149537848835,0
-0.85145984428902,48.149625983786,0
-0.85141684527228,48.149716965283,0
-0.85138589482011,48.149810100915,0
-0.85136722854693,48.149904681875,0
-0.85136098858134,48.149999988349,0
-0.85300000000007,48.15,0
</coordinates>
</LinearRing>
</outerBoundaryIs>
</Polygon>
</Placemark>
<Placemark>
<name>111</name>
<styleUrl>#stylehap1</styleUrl>
<Polygon>
<outerBoundaryIs>
<LinearRing>
<coordinates>
-0.84899999999993,48.191,0
-0.84735967765202,48.190999988333,0
-0.84736591653463,48.191095294984,0
-0.84738459184831,48.19118987647,0
-0.84741556152976,48.191283012961,0
-0.84745858994391,48.191373995624,0
-0.84751334967554,48.191462132011,0
-0.84757942401913,48.191546751336,0
-0.84765631014888,48.191627209575,0
-0.84774342294426,48.191702894372,0
-0.84784009944234,48.191773229696,0
-0.84794560388262,48.191837680232,0
-0.84805913330655,48.191895755447,0
-0.84817982366868,48.191947013333,0
-0.84830675641312,48.191991063765,0
-0.84843896546522,48.192027571473,0
-0.84857544458507,48.192056258596,0
-0.84871515502731,48.192076906793,0
-0.84885703344812,48.19208935891,0
-0.84899999999994,48.192093520174,0
-0.84914296655173,48.19208935891,0
-0.84928484497253,48.192076906793,0
-0.84942455541478,48.192056258596,0
-0.84956103453466,48.192027571473,0
-0.84969324358676,48.191991063765,0
-0.8498201763312,48.191947013333,0
-0.84994086669333,48.191895755447,0
-0.85005439611725,48.191837680232,0
-0.85015990055754,48.191773229696,0

-0.85025657705559,48.191702894372,0
-0.85034368985099,48.191627209575,0
-0.85042057598075,48.191546751336,0
-0.85048665032431,48.191462132011,0
-0.85054141005594,48.191373995624,0
-0.85058443847012,48.191283012961,0
-0.85061540815154,48.19118987647,0
-0.85063408346522,48.191095294984,0
-0.85064032234785,48.190999988333,0
-0.85063407738698,48.190904681858,0
-0.85061539617973,48.190810100899,0
-0.85058442096849,48.190716965267,0
-0.85054138755629,48.190625983771,0
-0.85048662351028,48.190537848822,0
-0.85042054566703,48.190453231163,0
-0.85034365695869,48.190372774767,0
-0.85025654258412,48.190297091935,0
-0.85015986555428,48.190226758636,0
-0.85005436164579,48.190162310127,0
-0.84994083380102,48.190104236876,0
-0.84982014601748,48.190052980833,0
-0.84969321677269,48.190008932068,0
-0.84956101203501,48.189972425797,0
-0.84942453791315,48.189943739841,0
-0.84928483300072,48.189923092503,0
-0.84914296047348,48.189910640912,0
-0.84899999999994,48.189906479827,0
-0.84885703952639,48.189910640912,0
-0.84871516699913,48.189923092503,0
-0.84857546208672,48.189943739841,0
-0.84843898796487,48.189972425797,0
-0.84830678322718,48.190008932068,0
-0.84817985398239,48.190052980833,0
-0.84805916619882,48.190104236876,0
-0.84794563835408,48.190162310127,0
-0.84784013444559,48.190226758636,0
-0.84774345741575,48.190297091935,0
-0.84765634304116,48.190372774767,0
-0.84757945433284,48.190453231163,0
-0.8475133764896,48.190537848822,0
-0.84745861244359,48.190625983771,0
-0.84741557903138,48.190716965267,0
-0.84738460382012,48.190810100899,0
-0.8473659226129,48.190904681858,0
-0.84735967765202,48.190999988333,0
-0.84899999999993,48.191,0
</coordinates>
</LinearRing>
</outerBoundaryIs>
</Polygon>
</Placemark>
<Placemark>
<name>112</name>
<styleUrl>#stylehap1</styleUrl>
<Polygon>
<outerBoundaryIs>
<LinearRing>

<coordinates>
-0.84799999999996,48.197,0
-0.84635948556234,48.19699998833,0
-0.84636572517492,48.197095294982,0
-0.84638440267497,48.197189876467,0
-0.8464153759825,48.197283012959,0
-0.84645840943498,48.197373995622,0
-0.84651317557878,48.197462132009,0
-0.84657925765962,48.197546751334,0
-0.84665615279284,48.197627209573,0
-0.8467432757894,48.19770289437,0
-0.8468399636087,48.197773229695,0
-0.84694548040411,48.197837680231,0
-0.84705902312304,48.197895755446,0
-0.84717972761887,48.197947013332,0
-0.84730667522813,48.197991063764,0
-0.84743889976299,48.198027571473,0
-0.84757539486576,48.198056258595,0
-0.84771512166934,48.198076906793,0
-0.84785701670544,48.19808935891,0
-0.84799999999995,48.198093520174,0
-0.84814298329449,48.19808935891,0
-0.84828487833056,48.198076906793,0
-0.84842460513414,48.198056258595,0
-0.84856110023692,48.198027571473,0
-0.84869332477178,48.197991063764,0
-0.84882027238104,48.197947013332,0
-0.84894097687687,48.197895755446,0
-0.84905451959582,48.197837680231,0
-0.84916003639123,48.197773229695,0
-0.84925672421053,48.19770289437,0
-0.84934384720709,48.197627209573,0
-0.84942074234029,48.197546751334,0
-0.84948682442112,48.197462132009,0
-0.84954159056493,48.197373995622,0
-0.84958462401741,48.197283012959,0
-0.84961559732496,48.197189876467,0
-0.84963427482499,48.197095294982,0
-0.84964051443757,48.19699998833,0
-0.84963426874473,48.196904681856,0
-0.84961558534921,48.196810100897,0
-0.84958460651006,48.196716965265,0
-0.8495415680579,48.196625983769,0
-0.84948679759828,48.19653784882,0
-0.84942071201665,48.196453231161,0
-0.849343814304,48.196372774765,0
-0.84925668972774,48.196297091933,0
-0.84916000137648,48.196226758635,0
-0.84905448511303,48.196162310126,0
-0.84894094397377,48.196104236875,0
-0.8488202420574,48.196052980833,0
-0.84869329794894,48.196008932067,0
-0.84856107772989,48.195972425797,0
-0.84842458762679,48.195943739841,0
-0.84828486635481,48.195923092503,0
-0.84814297721424,48.195910640912,0
-0.84799999999995,48.195906479826,0

-0.84785702278567,48.195910640912,0
-0.8477151336451,48.195923092503,0
-0.84757541237314,48.195943739841,0
-0.84743892227004,48.195972425797,0
-0.84730670205097,48.196008932067,0
-0.84717975794253,48.196052980833,0
-0.84705905602613,48.196104236875,0
-0.84694551488687,48.196162310126,0
-0.84683999862343,48.196226758635,0
-0.84674331027216,48.196297091933,0
-0.84665618569593,48.196372774765,0
-0.84657928798328,48.196453231161,0
-0.84651320240162,48.19653784882,0
-0.84645843194201,48.196625983769,0
-0.84641539348987,48.196716965265,0
-0.8463844146507,48.196810100897,0
-0.84636573125517,48.196904681856,0
-0.84635948556234,48.19699998833,0
-0.84799999999996,48.197,0
</coordinates>
</LinearRing>
</outerBoundaryIs>
</Polygon>
</Placemark>
<Placemark>
<name>116</name>
<styleUrl>#stylehap1</styleUrl>
<Polygon>
<outerBoundaryIs>
<LinearRing>
<coordinates>
-0.84500000000003,48.114,0
-0.84336213722364,48.113999988364,0
-0.84336836675959,48.114095295015,0
-0.84338701407864,48.1141898765,0
-0.84341793733023,48.114283012991,0
-0.84346090123242,48.114373995652,0
-0.84351557886073,48.114462132037,0
-0.84358155413424,48.11454675136,0
-0.84365832498102,48.114627209596,0
-0.84374530715779,48.11470289439,0
-0.84384183869557,48.114773229712,0
-0.84394718493713,48.114837680245,0
-0.84406054412792,48.114895755457,0
-0.84418105351812,48.114947013341,0
-0.84430779592931,48.11499106377,0
-0.8444398067355,48.115027571477,0
-0.84457608120585,48.115056258598,0
-0.84471558215264,48.115076906794,0
-0.84485724782675,48.115089358911,0
-0.84500000000002,48.115093520173,0
-0.8451427521733,48.115089358911,0
-0.84528441784741,48.115076906794,0
-0.8454239187942,48.115056258598,0
-0.84556019326455,48.115027571477,0
-0.84569220407074,48.11499106377,0
-0.84581894648193,48.114947013341,0

-0.84593945587216,48.114895755457,0
-0.84605281506292,48.114837680245,0
-0.84615816130447,48.114773229712,0
-0.84625469284228,48.11470289439,0
-0.84634167501905,48.114627209596,0
-0.84641844586581,48.11454675136,0
-0.84648442113935,48.114462132037,0
-0.84653909876762,48.114373995652,0
-0.84658206266985,48.114283012991,0
-0.84661298592143,48.1141898765,0
-0.84663163324046,48.114095295015,0
-0.84663786277643,48.113999988364,0
-0.84663162718771,48.11390468189,0
-0.84661297399985,48.11381010093,0
-0.84658204524165,48.113716965296,0
-0.84653907636237,48.113625983799,0
-0.8464843944378,48.113537848847,0
-0.84641841567929,48.113453231186,0
-0.84634164226476,48.113372774788,0
-0.84625465851545,48.113297091953,0
-0.84615812644809,48.113226758652,0
-0.84605278073609,48.11316231014,0
-0.84593942311786,48.113104236886,0
-0.84581891629541,48.113052980841,0
-0.84569217736919,48.113008932073,0
-0.84556017085929,48.112972425801,0
-0.84542390136601,48.112943739843,0
-0.84528440592582,48.112923092504,0
-0.84514274612055,48.112910640913,0
-0.84500000000002,48.112906479826,0
-0.8448572538795,48.112910640913,0
-0.84471559407422,48.112923092504,0
-0.84457609863404,48.112943739843,0
-0.84443982914075,48.112972425801,0
-0.84430782263086,48.113008932073,0
-0.84418108370463,48.113052980841,0
-0.84406057688219,48.113104236886,0
-0.84394721926396,48.11316231014,0
-0.84384187355196,48.113226758652,0
-0.84374534148463,48.113297091953,0
-0.84365835773529,48.113372774788,0
-0.84358158432075,48.113453231186,0
-0.84351560556225,48.113537848847,0
-0.84346092363768,48.113625983799,0
-0.84341795475842,48.113716965296,0
-0.84338702600023,48.11381010093,0
-0.84336837281234,48.11390468189,0
-0.84336213722364,48.113999988364,0
-0.84500000000003,48.114,0
</coordinates>
</LinearRing>
</outerBoundaryIs>
</Polygon>
</Placemark>
<Placemark>
<name>119</name>
<styleUrl>#stylehap1</styleUrl>

<Polygon>
<outerBoundaryIs>
<LinearRing>
<coordinates>
-0.83799999999997,48.22,0
-0.83635874863474,48.219999988321,0
-0.83636499104773,48.220095294972,0
-0.83638367693541,48.220189876458,0
-0.83641466415411,48.22028301295,0
-0.8364577169354,48.220373995613,0
-0.83651250767871,48.220462132001,0
-0.83657861944254,48.220546751327,0
-0.83665554911641,48.220627209567,0
-0.83674271124843,48.220702894365,0
-0.83683944250018,48.22077322969,0
-0.83694500669449,48.220837680227,0
-0.83705860041806,48.220895755443,0
-0.83717935913601,48.22094701333,0
-0.83730636377221,48.220991063762,0
-0.83743864770477,48.221027571471,0
-0.83757520412385,48.221056258595,0
-0.8377149936957,48.221076906793,0
-0.83785695247414,48.22108935891,0
-0.83799999999995,48.221093520174,0
-0.83814304752579,48.22108935891,0
-0.83828500630423,48.221076906793,0
-0.83842479587608,48.221056258595,0
-0.83856135229517,48.221027571471,0
-0.83869363622772,48.220991063762,0
-0.83882064086392,48.22094701333,0
-0.83894139958187,48.220895755443,0
-0.83905499330544,48.220837680227,0
-0.83916055749975,48.22077322969,0
-0.8392572887515,48.220702894365,0
-0.83934445088352,48.220627209567,0
-0.83942138055739,48.220546751327,0
-0.83948749232122,48.220462132001,0
-0.83954228306453,48.220373995613,0
-0.83958533584582,48.22028301295,0
-0.83961632306452,48.220189876458,0
-0.8396350089522,48.220095294972,0
-0.83964125136519,48.219999988321,0
-0.83963500286429,48.219904681846,0
-0.8396163110737,48.219810100888,0
-0.83958531831644,48.219716965256,0
-0.83954226052918,48.219625983761,0
-0.83948746546464,48.219537848812,0
-0.83942135019561,48.219453231154,0
-0.83934441793906,48.219372774759,0
-0.83925725422536,48.219297091928,0
-0.83916052244098,48.21922675863,0
-0.83905495877928,48.219162310122,0
-0.83894136663741,48.219104236872,0
-0.83882061050212,48.21905298083,0
-0.83869360937114,48.219008932066,0
-0.83856132975982,48.218972425796,0
-0.83842477834669,48.218943739841,0

-0.83828499431343,48.218923092503,0
-0.8381430414379,48.218910640912,0
-0.83799999999995,48.218906479827,0
-0.83785695856203,48.218910640912,0
-0.8377150056865,48.218923092503,0
-0.83757522165324,48.218943739841,0
-0.83743867024012,48.218972425796,0
-0.83730639062879,48.219008932066,0
-0.83717938949781,48.21905298083,0
-0.83705863336252,48.219104236872,0
-0.83694504122066,48.219162310122,0
-0.83683947755896,48.21922675863,0
-0.83674274577457,48.219297091928,0
-0.83665558206087,48.219372774759,0
-0.83657864980432,48.219453231154,0
-0.83651253453529,48.219537848812,0
-0.83645773947075,48.219625983761,0
-0.83641468168349,48.219716965256,0
-0.83638368892623,48.219810100888,0
-0.83636499713565,48.219904681846,0
-0.83635874863474,48.219999988321,0
-0.83799999999997,48.22,0
</coordinates>
</LinearRing>
</outerBoundaryIs>
</Polygon>
</Placemark>
<Placemark>
<name>121</name>
<styleUrl>#stylehap1</styleUrl>
<Polygon>
<outerBoundaryIs>
<LinearRing>
<coordinates>
-0.83400000000006,47.967,0
-0.83236680417454,47.966999988424,0
-0.83237301597551,47.967095295075,0
-0.83239161017566,47.967189876558,0
-0.8324224453282,47.967283013046,0
-0.83246528682118,47.967373995704,0
-0.83251980866123,47.967462132086,0
-0.83258559595276,47.967546751405,0
-0.83266214805412,47.967627209636,0
-0.8327488823866,47.967702894425,0
-0.83284513886732,47.967773229742,0
-0.83295018493237,47.967837680269,0
-0.83306322111173,47.967895755477,0
-0.83318338711408,47.967947013356,0
-0.83330976837459,47.967991063781,0
-0.83344140301617,47.968027571484,0
-0.83357728917122,47.968056258602,0
-0.83371639260778,47.968076906796,0
-0.83385765460251,47.968089358911,0
-0.83400000000007,47.968093520174,0
-0.83414234539759,47.968089358911,0
-0.83428360739235,47.968076906796,0
-0.83442271082891,47.968056258602,0

-0.83455859698394,47.968027571484,0
-0.83469023162554,47.967991063781,0
-0.83481661288605,47.967947013356,0
-0.8349367788884,47.967895755477,0
-0.83504981506776,47.967837680269,0
-0.83515486113279,47.967773229742,0
-0.83525111761353,47.967702894425,0
-0.83533785194601,47.967627209636,0
-0.83541440404734,47.967546751405,0
-0.83548019133888,47.967462132086,0
-0.83553471317893,47.967373995704,0
-0.83557755467193,47.967283013046,0
-0.83560838982447,47.967189876558,0
-0.83562698402462,47.967095295075,0
-0.83563319582559,47.966999988424,0
-0.83562697802019,47.966904681949,0
-0.83560837799804,47.966810100988,0
-0.83557753738285,47.966716965352,0
-0.83553469095253,47.966625983852,0
-0.83548016485048,47.966537848897,0
-0.83541437410179,47.966453231231,0
-0.83533781945315,47.966372774828,0
-0.83525108356068,47.966297091989,0
-0.83515482655461,47.966226758682,0
-0.83504978101491,47.966162310165,0
-0.83493674639555,47.966104236906,0
-0.83481658294047,47.966052980856,0
-0.83469020513712,47.966008932084,0
-0.83455857475753,47.965972425808,0
-0.83442269353982,47.965943739847,0
-0.83428359556593,47.965923092506,0
-0.83414233939316,47.965910640913,0
-0.83400000000007,47.965906479826,0
-0.83385766060694,47.965910640913,0
-0.8337164044342,47.965923092506,0
-0.83357730646028,47.965943739847,0
-0.8334414252426,47.965972425808,0
-0.83330979486298,47.966008932084,0
-0.83318341705964,47.966052980856,0
-0.83306325360458,47.966104236906,0
-0.83295021898519,47.966162310165,0
-0.8328451734455,47.966226758682,0
-0.83274891643945,47.966297091989,0
-0.83266218054698,47.966372774828,0
-0.83258562589834,47.966453231231,0
-0.83251983514965,47.966537848897,0
-0.8324653090476,47.966625983852,0
-0.83242246261726,47.966716965352,0
-0.83239162200209,47.966810100988,0
-0.83237302197994,47.966904681949,0
-0.83236680417454,47.966999988424,0
-0.83400000000006,47.967,0
</coordinates>
</LinearRing>
</outerBoundaryIs>
</Polygon>
</Placemark>

<Placemark>
<name>123</name>
<styleUrl>#stylehap1</styleUrl>
<Polygon>
<outerBoundaryIs>
<LinearRing>
<coordinates>
-0.83100000000002,48.463,0
-0.82935090590395,48.46299998822,0
-0.82935717812012,48.463095294873,0
-0.829375953273,48.463189876361,0
-0.82940708854045,48.463283012857,0
-0.8294503470282,48.463373995525,0
-0.82950539957082,48.463462131919,0
-0.82957182723505,48.463546751252,0
-0.82964912450677,48.4636272095,0
-0.82973670313682,48.463702894306,0
-0.82983389661722,48.46377322964,0
-0.82993996525304,48.463837680185,0
-0.8300541017918,48.46389575541,0
-0.83017543756726,48.463947013305,0
-0.83030304911107,48.463991063745,0
-0.83043596518193,48.46402757146,0
-0.83057317415837,48.464056258588,0
-0.8307136317395,48.46407690679,0
-0.83085626889437,48.46408935891,0
-0.83100000000001,48.464093520174,0
-0.83114373110565,48.46408935891,0
-0.83128636826052,48.46407690679,0
-0.83142682584165,48.464056258588,0
-0.83156403481809,48.46402757146,0
-0.83169695088895,48.463991063745,0
-0.83182456243279,48.463947013305,0
-0.83194589820821,48.46389575541,0
-0.83206003474698,48.463837680185,0
-0.83216610338282,48.46377322964,0
-0.83226329686322,48.463702894306,0
-0.83235087549328,48.4636272095,0
-0.83242817276499,48.463546751252,0
-0.83249460042922,48.463462131919,0
-0.83254965297182,48.463373995525,0
-0.83259291145959,48.463283012857,0
-0.83262404672702,48.463189876361,0
-0.83264282187992,48.463095294873,0
-0.83264909409609,48.46299998822,0
-0.83264281571046,48.462904681747,0
-0.8326240345756,48.46281010079,0
-0.83259289369538,48.462716965163,0
-0.8325496301346,48.462625983672,0
-0.83249457321286,48.46253784873,0
-0.83242814199646,48.462453231079,0
-0.83235084210748,48.462372774692,0
-0.83226326187455,48.462297091869,0
-0.83216606785437,48.46222675858,0
-0.83205999975831,48.462162310081,0
-0.83194586482242,48.462104236839,0
-0.83182453166425,48.462052980805,0

-0.83169692367258,48.462008932048,0
-0.83156401198087,48.461972425784,0
-0.83142680807744,48.461943739834,0
-0.8312863561091,48.4619230925,0
-0.83114372493621,48.461910640911,0
-0.83100000000001,48.461906479827,0
-0.83085627506383,48.461910640911,0
-0.83071364389095,48.4619230925,0
-0.83057319192261,48.461943739834,0
-0.83043598801917,48.461972425784,0
-0.83030307632744,48.462008932048,0
-0.83017546833579,48.462052980805,0
-0.83005413517763,48.462104236839,0
-0.82994000024174,48.462162310081,0
-0.82983393214564,48.46222675858,0
-0.8297367381255,48.462297091869,0
-0.82964915789257,48.462372774692,0
-0.82957185800359,48.462453231079,0
-0.82950542678718,48.46253784873,0
-0.82945036986544,48.462625983672,0
-0.82940710630467,48.462716965163,0
-0.82937596542444,48.46281010079,0
-0.82935718428956,48.462904681747,0
-0.82935090590395,48.46299998822,0
-0.83100000000002,48.463,0
</coordinates>
</LinearRing>
</outerBoundaryIs>
</Polygon>
</Placemark>
<Placemark>
<name>124</name>
<styleUrl>#stylehap1</styleUrl>
<Polygon>
<outerBoundaryIs>
<LinearRing>
<coordinates>
-0.83000000000004,48.203,0
-0.82835929340973,48.202999988328,0
-0.82836553375252,48.203095294979,0
-0.82838421343962,48.203189876465,0
-0.82841519037446,48.203283012957,0
-0.82845822886692,48.203373995619,0
-0.82851300142499,48.203462132007,0
-0.82857909124561,48.203546751332,0
-0.82865599538525,48.203627209572,0
-0.82874312858633,48.203702894369,0
-0.82883982773057,48.203773229694,0
-0.82894535688516,48.20383768023,0
-0.82905891290348,48.203895755445,0
-0.82917963153764,48.203947013332,0
-0.82930659401658,48.203991063764,0
-0.82943883403929,48.204027571472,0
-0.82957534513021,48.204056258595,0
-0.82971508830051,48.204076906793,0
-0.8298569999573,48.20408935891,0
-0.83000000000005,48.204093520174,0

-0.83014300004278,48.20408935891,0
-0.83028491169956,48.204076906793,0
-0.83042465486987,48.204056258595,0
-0.83056116596079,48.204027571472,0
-0.8306934059835,48.203991063764,0
-0.83082036846244,48.203947013332,0
-0.83094108709659,48.203895755445,0
-0.83105464311492,48.20383768023,0
-0.83116017226951,48.203773229694,0
-0.83125687141374,48.203702894369,0
-0.83134400461483,48.203627209572,0
-0.83142090875446,48.203546751332,0
-0.83148699857509,48.203462132007,0
-0.83154177113318,48.203373995619,0
-0.83158480962562,48.203283012957,0
-0.83161578656046,48.203189876465,0
-0.83163446624756,48.203095294979,0
-0.83164070659034,48.202999988328,0
-0.83163446016532,48.202904681853,0
-0.83161577458078,48.202810100894,0
-0.83158479211252,48.202716965263,0
-0.83154174861874,48.202625983767,0
-0.83148697174344,48.202537848818,0
-0.83142087842087,48.202453231159,0
-0.83134397170095,48.202372774764,0
-0.83125683691966,48.202297091932,0
-0.83116013724331,48.202226758634,0
-0.83105460862084,48.202162310125,0
-0.83094105418274,48.202104236874,0
-0.83082033812885,48.202052980832,0
-0.83069337915185,48.202008932067,0
-0.83056114344635,48.201972425797,0
-0.83042463735677,48.201943739841,0
-0.83028489971989,48.201923092503,0
-0.83014299396054,48.201910640912,0
-0.83000000000005,48.201906479826,0
-0.82985700603954,48.201910640912,0
-0.82971510028018,48.201923092503,0
-0.82957536264331,48.201943739841,0
-0.82943885655372,48.201972425797,0
-0.82930662084822,48.202008932067,0
-0.82917966187123,48.202052980832,0
-0.82905894581736,48.202104236874,0
-0.82894539137926,48.202162310125,0
-0.82883986275679,48.202226758634,0
-0.82874316308041,48.202297091932,0
-0.82865602829913,48.202372774764,0
-0.82857912157923,48.202453231159,0
-0.82851302825663,48.202537848818,0
-0.82845825138133,48.202625983767,0
-0.82841520788758,48.202716965263,0
-0.82838422541929,48.202810100894,0
-0.82836553983475,48.202904681853,0
-0.82835929340973,48.202999988328,0
-0.83000000000004,48.203,0
</coordinates>
</LinearRing>

</outerBoundaryIs>
</Polygon>
</Placemark>
<Placemark>
<name>126</name>
<styleUrl>#stylehap1</styleUrl>
<Polygon>
<outerBoundaryIs>
<LinearRing>
<coordinates>
-0.81299999999999,48.163,0
-0.81136057323858,48.162999988344,0
-0.81136680871787,48.163095294995,0
-0.81138547383808,48.163189876481,0
-0.81141642661332,48.163283012972,0
-0.81145943153722,48.163373995634,0
-0.81151416137313,48.163462132021,0
-0.81158019964302,48.163546751345,0
-0.81165704379559,48.163627209583,0
-0.81174410902975,48.163702894378,0
-0.81184073274443,48.163773229702,0
-0.81194617958092,48.163837680236,0
-0.81205964701904,48.163895755451,0
-0.81218027148514,48.163947013336,0
-0.81230713492489,48.163991063767,0
-0.8124392717911,48.164027571474,0
-0.81257567639335,48.164056258596,0
-0.81271531055348,48.164076906793,0
-0.81285711150834,48.164089358911,0
-0.81299999999998,48.164093520174,0
-0.81314288849165,48.164089358911,0
-0.81328468944648,48.164076906793,0
-0.81342432360661,48.164056258596,0
-0.81356072820889,48.164027571474,0
-0.81369286507509,48.163991063767,0
-0.81381972851481,48.163947013336,0
-0.81394035298092,48.163895755451,0
-0.81405382041906,48.163837680236,0
-0.81415926725553,48.163773229702,0
-0.81425589097024,48.163702894378,0
-0.8143429562044,48.163627209583,0
-0.81441980035696,48.163546751345,0
-0.81448583862685,48.163462132021,0
-0.81454056846277,48.163373995634,0
-0.81458357338666,48.163283012972,0
-0.81461452616191,48.163189876481,0
-0.81463319128209,48.163095294995,0
-0.81463942676141,48.162999988344,0
-0.81463318521314,48.16290468187,0
-0.81461451420839,48.16281010091,0
-0.81458355591178,48.162716965278,0
-0.81454054599749,48.162625983781,0
-0.81448581185378,48.162537848831,0
-0.81441977008958,48.162453231171,0
-0.81434292336237,48.162372774775,0
-0.81425585655145,48.162297091942,0
-0.81415923230579,48.162226758642,0

-0.81405378600027,48.162162310132,0
-0.81394032013889,48.16210423688,0
-0.81381969824746,48.162052980836,0
-0.81369283830202,48.16200893207,0
-0.81356070574361,48.161972425799,0
-0.81342430613175,48.161943739842,0
-0.81328467749296,48.161923092504,0
-0.81314288242269,48.161910640912,0
-0.81299999999998,48.161906479826,0
-0.81285711757729,48.161910640912,0
-0.81271532250702,48.161923092504,0
-0.81257569386824,48.161943739842,0
-0.81243929425635,48.161972425799,0
-0.81230716169796,48.16200893207,0
-0.81218030175252,48.162052980836,0
-0.81205967986107,48.16210423688,0
-0.81194621399971,48.162162310132,0
-0.8118407676942,48.162226758642,0
-0.81174414344854,48.162297091942,0
-0.81165707663762,48.162372774775,0
-0.8115802299104,48.162453231171,0
-0.81151418814618,48.162537848831,0
-0.81145945400247,48.162625983781,0
-0.81141644408821,48.162716965278,0
-0.81138548579159,48.16281010091,0
-0.81136681478685,48.16290468187,0
-0.81136057323858,48.162999988344,0
-0.81299999999999,48.163,0
</coordinates>
</LinearRing>
</outerBoundaryIs>
</Polygon>
</Placemark>
<Placemark>
<name>127</name>
<styleUrl>#stylehap1</styleUrl>
<Polygon>
<outerBoundaryIs>
<LinearRing>
<coordinates>
-0.81200000000001,48.163,0
-0.8103605732386,48.162999988344,0
-0.81036680871791,48.163095294995,0
-0.81038547383809,48.163189876481,0
-0.81041642661334,48.163283012972,0
-0.81045943153724,48.163373995634,0
-0.81051416137315,48.163462132021,0
-0.81058019964304,48.163546751345,0
-0.8106570437956,48.163627209583,0
-0.81074410902976,48.163702894378,0
-0.81084073274447,48.163773229702,0
-0.81094617958094,48.163837680236,0
-0.81105964701908,48.163895755451,0
-0.81118027148516,48.163947013336,0
-0.81130713492491,48.163991063767,0
-0.81143927179111,48.164027571474,0
-0.81157567639339,48.164056258596,0

-0.81171531055352,48.164076906793,0
-0.81185711150835,48.164089358911,0
-0.81200000000002,48.164093520174,0
-0.81214288849166,48.164089358911,0
-0.81228468944652,48.164076906793,0
-0.81242432360665,48.164056258596,0
-0.81256072820891,48.164027571474,0
-0.81269286507511,48.163991063767,0
-0.81281972851486,48.163947013336,0
-0.81294035298096,48.163895755451,0
-0.81305382041908,48.163837680236,0
-0.81315926725557,48.163773229702,0
-0.81325589097025,48.163702894378,0
-0.81334295620441,48.163627209583,0
-0.81341980035698,48.163546751345,0
-0.81348583862687,48.163462132021,0
-0.81354056846278,48.163373995634,0
-0.81358357338668,48.163283012972,0
-0.81361452616192,48.163189876481,0
-0.81363319128214,48.163095294995,0
-0.81363942676142,48.162999988344,0
-0.81363318521315,48.16290468187,0
-0.81361451420841,48.16281010091,0
-0.81358355591179,48.162716965278,0
-0.81354054599753,48.162625983781,0
-0.81348581185382,48.162537848831,0
-0.8134197700896,48.162453231171,0
-0.81334292336239,48.162372774775,0
-0.81325585655146,48.162297091942,0
-0.8131592323058,48.162226758642,0
-0.81305378600029,48.162162310132,0
-0.81294032013893,48.16210423688,0
-0.81281969824748,48.162052980836,0
-0.81269283830204,48.16200893207,0
-0.81256070574366,48.161972425799,0
-0.81242430613176,48.161943739842,0
-0.81228467749298,48.161923092504,0
-0.81214288242271,48.161910640912,0
-0.81200000000002,48.161906479826,0
-0.81185711757731,48.161910640912,0
-0.81171532250704,48.161923092504,0
-0.81157569386825,48.161943739842,0
-0.81143929425639,48.161972425799,0
-0.81130716169798,48.16200893207,0
-0.81118030175254,48.162052980836,0
-0.81105967986111,48.16210423688,0
-0.81094621399973,48.162162310132,0
-0.81084076769421,48.162226758642,0
-0.81074414344855,48.162297091942,0
-0.81065707663763,48.162372774775,0
-0.81058022991042,48.162453231171,0
-0.81051418814622,48.162537848831,0
-0.81045945400251,48.162625983781,0
-0.81041644408822,48.162716965278,0
-0.81038548579161,48.16281010091,0
-0.81036681478686,48.16290468187,0
-0.8103605732386,48.162999988344,0

-0.81200000000001,48.163,0
</coordinates>
</LinearRing>
</outerBoundaryIs>
</Polygon>
</Placemark>
<Placemark>
<name>128</name>
<styleUrl>#stylehap1</styleUrl>
<Polygon>
<outerBoundaryIs>
<LinearRing>
<coordinates>
-0.80999999999995,48.059,0
-0.80836388774232,48.058999988386,0
-0.8083701106261,48.059095295038,0
-0.80838873802086,48.059189876522,0
-0.80841962822752,48.059283013011,0
-0.80846254621548,48.059373995671,0
-0.80851716540944,48.059462132056,0
-0.80858307017311,48.059546751377,0
-0.80865975897102,48.059627209611,0
-0.8087466481843,48.059702894403,0
-0.8088430765514,48.059773229723,0
-0.80894831020025,48.059837680254,0
-0.80906154823321,48.059895755465,0
-0.80918192882264,48.059947013346,0
-0.80930853577043,48.059991063774,0
-0.80944040548166,48.060027571479,0
-0.80957653429948,48.060056258599,0
-0.80971588614482,48.060076906795,0
-0.80985740040351,48.060089358911,0
-0.80999999999995,48.060093520173,0
-0.81014259959639,48.060089358911,0
-0.81028411385508,48.060076906795,0
-0.81042346570042,48.060056258599,0
-0.81055959451821,48.060027571479,0
-0.81069146422945,48.059991063774,0
-0.81081807117723,48.059947013346,0
-0.81093845176667,48.059895755465,0
-0.81105168979965,48.059837680254,0
-0.8111569234485,48.059773229723,0
-0.8112533518156,48.059702894403,0
-0.81134024102885,48.059627209611,0
-0.81141692982679,48.059546751377,0
-0.81148283459046,48.059462132056,0
-0.81153745378441,48.059373995671,0
-0.81158037177235,48.059283013011,0
-0.81161126197902,48.059189876522,0
-0.81162988937378,48.059095295038,0
-0.81163611225756,48.058999988386,0
-0.81162988333917,48.058904681912,0
-0.81161125009316,48.058810100951,0
-0.81158035439637,48.058716965317,0
-0.81153743144628,48.058625983819,0
-0.81148280796891,48.058537848866,0
-0.8114168997307,48.058453231203,0

-0.8113402083727,48.058372774803,0
-0.81125331759158,48.058297091967,0
-0.81115688869654,48.058226758663,0
-0.81105165557566,48.058162310149,0
-0.81093841911051,48.058104236894,0
-0.81081804108117,48.058052980847,0
-0.81069143760792,48.058008932077,0
-0.81055957218008,48.057972425804,0
-0.81042344832444,48.057943739845,0
-0.8102841019692,48.057923092505,0
-0.81014259356178,48.057910640913,0
-0.80999999999995,48.057906479826,0
-0.80985740643812,48.057910640913,0
-0.80971589803068,48.057923092505,0
-0.80957655167546,48.057943739845,0
-0.80944042781979,48.057972425804,0
-0.80930856239198,48.058008932077,0
-0.80918195891873,48.058052980847,0
-0.80906158088936,48.058104236894,0
-0.80894834442424,48.058162310149,0
-0.80884311130336,48.058226758663,0
-0.80874668240829,48.058297091967,0
-0.80865979162718,48.058372774803,0
-0.8085831002692,48.058453231203,0
-0.80851719203096,48.058537848866,0
-0.80846256855361,48.058625983819,0
-0.8084196456035,48.058716965317,0
-0.80838874990674,48.058810100951,0
-0.80837011666073,48.058904681912,0
-0.80836388774232,48.058999988386,0
-0.80999999999995,48.059,0
</coordinates>
</LinearRing>
</outerBoundaryIs>
</Polygon>
</Placemark>
<Placemark>
<name>129</name>
<styleUrl>#stylehap1</styleUrl>
<Polygon>
<outerBoundaryIs>
<LinearRing>
<coordinates>
-0.80600000000004,47.946,0
-0.80436746783539,47.945999988432,0
-0.80437367711436,47.946095295083,0
-0.80439226376077,47.946189876567,0
-0.80442308638522,47.946283013054,0
-0.80446591047108,47.946373995712,0
-0.80452041015737,47.946462132093,0
-0.80458617071703,47.946546751411,0
-0.80466269171183,47.946627209642,0
-0.80474939079977,47.94670289443,0
-0.80484560816617,47.946773229746,0
-0.80495061154477,47.946837680273,0
-0.80506360179051,47.94689575548,0
-0.8051837189616,47.946947013358,0

-0.8053100488649,47.946991063782,0
-0.80544163001423,47.947027571485,0
-0.80557746094913,47.947056258602,0
-0.805716507858,47.947076906796,0
-0.80585771244779,47.947089358911,0
-0.80600000000004,47.947093520174,0
-0.80614228755231,47.947089358911,0
-0.80628349214207,47.947076906796,0
-0.80642253905094,47.947056258602,0
-0.80655836998584,47.947027571485,0
-0.80668995113517,47.946991063782,0
-0.80681628103847,47.946947013358,0
-0.80693639820957,47.94689575548,0
-0.8070493884553,47.946837680273,0
-0.8071543918339,47.946773229746,0
-0.80725060920031,47.94670289443,0
-0.80733730828824,47.946627209642,0
-0.80741382928304,47.946546751411,0
-0.8074795898427,47.946462132093,0
-0.807534089529,47.946373995712,0
-0.80757691361485,47.946283013054,0
-0.80760773623931,47.946189876567,0
-0.80762632288572,47.946095295083,0
-0.80763253216469,47.945999988432,0
-0.80762631688816,47.945904681957,0
-0.80760772442639,47.945810100996,0
-0.80757689634553,47.94571696536,0
-0.80753406732796,47.945625983859,0
-0.80747956338456,47.945537848904,0
-0.80741379937168,47.945453231238,0
-0.80733727583254,47.945372774834,0
-0.80725057518639,47.945297091994,0
-0.80715435729526,47.945226758686,0
-0.80704935444136,47.945162310168,0
-0.80693636575386,47.945104236909,0
-0.80681625112714,47.945052980858,0
-0.80668992467703,47.945008932085,0
-0.80655834778483,47.944972425809,0
-0.80642252178162,47.944943739848,0
-0.80628348032916,47.944923092506,0
-0.80614228155472,47.944910640913,0
-0.80600000000004,47.944906479826,0
-0.80585771844535,47.944910640913,0
-0.80571651967094,47.944923092506,0
-0.80557747821847,47.944943739848,0
-0.80544165221527,47.944972425809,0
-0.80531007532305,47.945008932085,0
-0.80518374887296,47.945052980858,0
-0.80506363424621,47.945104236909,0
-0.80495064555872,47.945162310168,0
-0.80484564270484,47.945226758686,0
-0.80474942481369,47.945297091994,0
-0.80466272416756,47.945372774834,0
-0.80458620062839,47.945453231238,0
-0.80452043661551,47.945537848904,0
-0.80446593267211,47.945625983859,0
-0.80442310365454,47.94571696536,0

-0.80439227557368,47.945810100996,0
-0.80437368311194,47.945904681957,0
-0.80436746783539,47.945999988432,0
-0.80600000000004,47.946,0
</coordinates>
</LinearRing>
</outerBoundaryIs>
</Polygon>
</Placemark>
<Placemark>
<name>130</name>
<styleUrl>#stylehap1</styleUrl>
<Polygon>
<outerBoundaryIs>
<LinearRing>
<coordinates>
-0.798,47.906,0
-0.79636872985449,47.905999988449,0
-0.79637493433762,47.906095295099,0
-0.79639350661981,47.906189876582,0
-0.79642430542084,47.90628301307,0
-0.79646709640526,47.906373995726,0
-0.79652155396389,47.906462132107,0
-0.79658726369016,47.906546751423,0
-0.79666372553263,47.906627209653,0
-0.79675035759946,47.90670289444,0
-0.79684650058597,47.906773229754,0
-0.79695142279201,47.90683768028,0
-0.79706432569019,47.906895755485,0
-0.79718435000367,47.906947013362,0
-0.79731058224598,47.906991063785,0
-0.7974420616743,47.907027571486,0
-0.79757778760247,47.907056258603,0
-0.79771672701814,47.907076906797,0
-0.79785782244648,47.907089358911,0
-0.79800000000001,47.907093520174,0
-0.79814217755353,47.907089358911,0
-0.79828327298187,47.907076906797,0
-0.79842221239754,47.907056258603,0
-0.79855793832569,47.907027571486,0
-0.79868941775403,47.906991063785,0
-0.79881564999634,47.906947013362,0
-0.79893567430982,47.906895755485,0
-0.79904857720801,47.90683768028,0
-0.79915349941401,47.906773229754,0
-0.79924964240056,47.90670289444,0
-0.79933627446736,47.906627209653,0
-0.79941273630985,47.906546751423,0
-0.79947844603613,47.906462132107,0
-0.79953290359475,47.906373995726,0
-0.79957569457917,47.90628301307,0
-0.79960649338021,47.906189876582,0
-0.79962506566239,47.906095295099,0
-0.79963127014552,47.905999988449,0
-0.79962505967786,47.905904681974,0
-0.79960648159297,47.905810101012,0
-0.79957567734739,47.905716965375,0

-0.79953288144201,47.905625983874,0
-0.79947841963551,47.905537848917,0
-0.79941270646355,47.90545323125,0
-0.79933624208222,47.905372774845,0
-0.79924960846058,47.905297092003,0
-0.79915346495047,47.905226758694,0
-0.79904854326803,47.905162310175,0
-0.79893564192467,47.905104236914,0
-0.79881562015005,47.905052980862,0
-0.79868939135342,47.905008932088,0
-0.79855791617294,47.904972425811,0
-0.79842219516575,47.904943739849,0
-0.79828326119464,47.904923092507,0
-0.798142171569,47.904910640913,0
-0.79800000000001,47.904906479826,0
-0.79785782843101,47.904910640913,0
-0.79771673880538,47.904923092507,0
-0.79757780483424,47.904943739849,0
-0.79744208382704,47.904972425811,0
-0.79731060864657,47.905008932088,0
-0.79718437984997,47.905052980862,0
-0.79706435807534,47.905104236914,0
-0.79695145673196,47.905162310175,0
-0.79684653504952,47.905226758694,0
-0.79675039153944,47.905297092003,0
-0.79666375791777,47.905372774845,0
-0.79658729353646,47.90545323125,0
-0.79652158036447,47.905537848917,0
-0.796467118558,47.905625983874,0
-0.79642432265263,47.905716965375,0
-0.79639351840702,47.905810101012,0
-0.79637494032215,47.905904681974,0
-0.79636872985449,47.905999988449,0
-0.798,47.906,0
</coordinates>
</LinearRing>
</outerBoundaryIs>
</Polygon>
</Placemark>
<Placemark>
<name>132</name>
<styleUrl>#stylehap1</styleUrl>
<Polygon>
<outerBoundaryIs>
<LinearRing>
<coordinates>
-0.79399999999998,47.916,0
-0.79236841460719,47.915999988445,0
-0.79237462028829,47.916095295095,0
-0.7923931961586,47.916189876578,0
-0.79242400091065,47.916283013066,0
-0.79246680016367,47.916373995723,0
-0.79252126824561,47.916462132103,0
-0.79258699066986,47.91654675142,0
-0.79266346728834,47.91662720965,0
-0.79275011609677,47.916702894437,0
-0.79284627766309,47.916773229752,0

-0.7929512201457,47.916837680278,0
-0.79306414486295,47.916895755484,0
-0.79318419237188,47.916947013361,0
-0.7933104490095,47.916991063785,0
-0.79344195384735,47.917027571486,0
-0.79357770600576,47.917056258603,0
-0.79371667227279,47.917076906797,0
-0.79385779496922,47.917089358911,0
-0.79399999999999,47.917093520174,0
-0.79414220503074,47.917089358911,0
-0.79428332772717,47.917076906797,0
-0.7944222939942,47.917056258603,0
-0.79455804615263,47.917027571486,0
-0.79468955099046,47.916991063785,0
-0.79481580762808,47.916947013361,0
-0.79493585513701,47.916895755484,0
-0.79504877985429,47.916837680278,0
-0.79515372233686,47.916773229752,0
-0.79524988390319,47.916702894437,0
-0.79533653271162,47.91662720965,0
-0.7954130093301,47.91654675142,0
-0.79547873175437,47.916462132103,0
-0.79553319983631,47.916373995723,0
-0.79557599908933,47.916283013066,0
-0.79560680384136,47.916189876578,0
-0.79562537971167,47.916095295095,0
-0.79563158539277,47.915999988445,0
-0.79562537372388,47.91590468197,0
-0.79560679204771,47.915810101008,0
-0.79557598184818,47.915716965372,0
-0.79553317767151,47.91562598387,0
-0.79547870533938,47.915537848914,0
-0.79541297946757,47.915453231247,0
-0.79533650030884,47.915372774842,0
-0.79524984994476,47.915297092001,0
-0.79515368785456,47.915226758692,0
-0.79504874589583,47.915162310173,0
-0.79493582273426,47.915104236913,0
-0.79481577776555,47.915052980861,0
-0.79468952457549,47.915008932088,0
-0.79455802398783,47.914972425811,0
-0.79442227675305,47.914943739849,0
-0.79428331593352,47.914923092507,0
-0.79414219904295,47.914910640913,0
-0.79399999999999,47.914906479826,0
-0.79385780095701,47.914910640913,0
-0.79371668406644,47.914923092507,0
-0.79357772324691,47.914943739849,0
-0.79344197601213,47.914972425811,0
-0.79331047542449,47.915008932088,0
-0.79318422223444,47.915052980861,0
-0.79306417726573,47.915104236913,0
-0.79295125410412,47.915162310173,0
-0.7928463121454,47.915226758692,0
-0.79275015005522,47.915297092001,0
-0.79266349969112,47.915372774842,0
-0.79258702053242,47.915453231247,0

-0.79252129466058,47.915537848914,0
-0.79246682232848,47.91562598387,0
-0.7924240181518,47.915716965372,0
-0.79239320795225,47.915810101008,0
-0.79237462627608,47.91590468197,0
-0.79236841460719,47.915999988445,0
-0.79399999999998,47.916,0
</coordinates>
</LinearRing>
</outerBoundaryIs>
</Polygon>
</Placemark>
<Placemark>
<name>133</name>
<styleUrl>#stylehap1</styleUrl>
<Polygon>
<outerBoundaryIs>
<LinearRing>
<coordinates>
-0.79200000000003,47.811,0
-0.79037171616837,47.810999988487,0
-0.79037790930309,47.811095295137,0
-0.79039644759523,47.81118987662,0
-0.79042719002294,47.811283013105,0
-0.79046990267946,47.81137399576,0
-0.79052426055141,47.811462132138,0
-0.79058984999071,47.811546751452,0
-0.79066617186118,47.811627209679,0
-0.79075264533604,47.811702894462,0
-0.79084861231756,47.811773229774,0
-0.79095334244498,47.811837680295,0
-0.79106603865289,47.811895755498,0
-0.79118584323749,47.811947013372,0
-0.79131184438477,47.811991063792,0
-0.7914430831109,47.812027571491,0
-0.79157856056178,47.812056258606,0
-0.7917172456165,47.812076906798,0
-0.79185808273632,47.812089358912,0
-0.79200000000002,47.812093520174,0
-0.79214191726372,47.812089358912,0
-0.79228275438357,47.812076906798,0
-0.79242143943827,47.812056258606,0
-0.79255691688917,47.812027571491,0
-0.7926881556153,47.811991063792,0
-0.79281415676258,47.811947013372,0
-0.79293396134715,47.811895755498,0
-0.79304665755506,47.811837680295,0
-0.79315138768251,47.811773229774,0
-0.79324735466403,47.811702894462,0
-0.79333382813888,47.811627209679,0
-0.79341015000933,47.811546751452,0
-0.79347573944866,47.811462132138,0
-0.79353009732061,47.81137399576,0
-0.79357280997713,47.811283013105,0
-0.79360355240484,47.81118987662,0
-0.79362209069698,47.811095295137,0
-0.79362828383168,47.810999988487,0

-0.79362208474326,47.810904682012,0
-0.79360354067834,47.810810101049,0
-0.79357279283412,47.810716965411,0
-0.79353007528199,47.810625983907,0
-0.79347571318405,47.810537848949,0
-0.7934101203168,47.810453231279,0
-0.79333379592058,47.810372774871,0
-0.7932473208989,47.810297092026,0
-0.79315135339649,47.810226758714,0
-0.79304662378994,47.810162310191,0
-0.79293392912885,47.810104236927,0
-0.79281412707001,47.810052980872,0
-0.79268812935069,47.810008932095,0
-0.79255689485054,47.809972425816,0
-0.79242142229528,47.809943739852,0
-0.79228274265707,47.809923092508,0
-0.79214191131003,47.809910640913,0
-0.79200000000002,47.809906479826,0
-0.79185808869002,47.809910640913,0
-0.791717257343,47.809923092508,0
-0.79157857770479,47.809943739852,0
-0.7914431051495,47.809972425816,0
-0.79131187064938,47.810008932095,0
-0.79118587293003,47.810052980872,0
-0.7910660708712,47.810104236927,0
-0.79095337621011,47.810162310191,0
-0.79084864660356,47.810226758714,0
-0.79075267910117,47.810297092026,0
-0.79066620407949,47.810372774871,0
-0.79058987968327,47.810453231279,0
-0.79052428681602,47.810537848949,0
-0.79046992471805,47.810625983907,0
-0.79042720716592,47.810716965411,0
-0.79039645932173,47.810810101049,0
-0.79037791525679,47.810904682012,0
-0.79037171616837,47.810999988487,0
-0.79200000000003,47.811,0
</coordinates>
</LinearRing>
</outerBoundaryIs>
</Polygon>
</Placemark>
<Placemark>
<name>134</name>
<styleUrl>#stylehap1</styleUrl>
<Polygon>
<outerBoundaryIs>
<LinearRing>
<coordinates>
-0.78800000000001,48.055,0
-0.7863640148481,48.054999988388,0
-0.78637023724887,48.055095295039,0
-0.78638886319694,48.055189876524,0
-0.78641975100419,48.055283013013,0
-0.7864626656583,48.055373995673,0
-0.78651728060932,48.055462132057,0
-0.78658318025323,48.055546751378,0

-0.78665986309356,48.055627209612,0
-0.78674674555673,48.055702894404,0
-0.78684316643259,48.055773229724,0
-0.78694839190603,48.055837680255,0
-0.78706162114168,48.055895755465,0
-0.78718199237886,48.055947013347,0
-0.78730858949061,48.055991063775,0
-0.7874404489569,48.056027571479,0
-0.78757656719881,48.056056258599,0
-0.78771590821785,48.056076906795,0
-0.78785741148223,48.056089358911,0
-0.78800000000001,48.056093520174,0
-0.78814258851781,48.056089358911,0
-0.78828409178217,48.056076906795,0
-0.7884234328012,48.056056258599,0
-0.78855955104314,48.056027571479,0
-0.78869141050943,48.055991063775,0
-0.78881800762115,48.055947013347,0
-0.78893837885834,48.055895755465,0
-0.78905160809401,48.055837680255,0
-0.78915683356745,48.055773229724,0
-0.78925325444328,48.055702894404,0
-0.78934013690645,48.055627209612,0
-0.78941681974678,48.055546751378,0
-0.78948271939072,48.055462132057,0
-0.78953733434172,48.055373995673,0
-0.78958024899582,48.055283013013,0
-0.78961113680308,48.055189876524,0
-0.78962976275114,48.055095295039,0
-0.78963598515191,48.054999988388,0
-0.78962975671786,48.054904681913,0
-0.78961112491981,48.054810100953,0
-0.78958023162364,48.054716965319,0
-0.78953731200847,48.05462598382,0
-0.78948269277498,48.054537848867,0
-0.78941678965726,48.054453231204,0
-0.78934010425742,48.054372774804,0
-0.78925322022675,48.054297091968,0
-0.78915679882307,48.054226758664,0
-0.78905157387747,48.05416231015,0
-0.78893834620931,48.054104236894,0
-0.78881797753166,48.054052980847,0
-0.78869138389368,48.054008932078,0
-0.78855952870987,48.053972425804,0
-0.78842341542901,48.053943739845,0
-0.78828407989888,48.053923092505,0
-0.7881425824845,48.053910640913,0
-0.78800000000001,48.053906479826,0
-0.78785741751551,48.053910640913,0
-0.78771592010114,48.053923092505,0
-0.787576584571,48.053943739845,0
-0.78744047129015,48.053972425804,0
-0.78730861610633,48.054008932078,0
-0.78718202246838,48.054052980847,0
-0.78706165379073,48.054104236894,0
-0.78694842612257,48.05416231015,0
-0.78684320117694,48.054226758664,0

-0.78674677977327,48.054297091968,0
-0.78665989574261,48.054372774804,0
-0.78658321034275,48.054453231204,0
-0.78651730722504,48.054537848867,0
-0.78646268799154,48.05462598382,0
-0.78641976837638,48.054716965319,0
-0.7863888750802,48.054810100953,0
-0.78637024328218,48.054904681913,0
-0.7863640148481,48.054999988388,0
-0.78800000000001,48.055,0
</coordinates>
</LinearRing>
</outerBoundaryIs>
</Polygon>
</Placemark>
<Placemark>
<name>137</name>
<styleUrl>#stylehap1</styleUrl>
<Polygon>
<outerBoundaryIs>
<LinearRing>
<coordinates>
-0.78700000000003,48.047,0
-0.78536426897642,48.046999988391,0
-0.78537049041147,48.047095295042,0
-0.78538911346707,48.047189876527,0
-0.7854199964771,48.047283013016,0
-0.78546290446568,48.047373995676,0
-0.78551751093358,48.04746213206,0
-0.78558340034137,48.04754675138,0
-0.78566007127042,48.047627209615,0
-0.78574694023779,48.047702894406,0
-0.785843346136,48.047773229726,0
-0.78594855526402,48.047837680256,0
-0.78606176691084,48.047895755466,0
-0.78618211944959,48.047947013348,0
-0.7863086968957,48.047991063775,0
-0.78644053587878,48.04802757148,0
-0.78657663297588,48.0480562586,0
-0.78671595234938,48.048076906795,0
-0.78685743363231,48.048089358911,0
-0.78700000000002,48.048093520174,0
-0.78714256636776,48.048089358911,0
-0.78728404765069,48.048076906795,0
-0.78742336702419,48.0480562586,0
-0.78755946412129,48.04802757148,0
-0.78769130310437,48.047991063775,0
-0.78781788055048,48.047947013348,0
-0.78793823308923,48.047895755466,0
-0.78805144473605,48.047837680256,0
-0.78815665386404,48.047773229726,0
-0.78825305976228,48.047702894406,0
-0.78833992872965,48.047627209615,0
-0.7884165996587,48.04754675138,0
-0.78848248906649,48.04746213206,0
-0.78853709553439,48.047373995676,0
-0.78858000352297,48.047283013016,0

-0.788610886533,48.047189876527,0
-0.7886295095886,48.047095295042,0
-0.78863573102365,48.046999988391,0
-0.78862950355794,48.046904681917,0
-0.78861087465491,48.046810100956,0
-0.78857998615836,48.046716965322,0
-0.78853707321089,48.046625983823,0
-0.78848246246235,48.04653784887,0
-0.7884165695823,48.046453231207,0
-0.78833989609484,48.046372774806,0
-0.78825302556068,48.046297091969,0
-0.78815661913483,48.046226758666,0
-0.78805141053443,48.046162310151,0
-0.78793820045445,48.046104236895,0
-0.78781785047408,48.046052980848,0
-0.78769127650026,48.046008932078,0
-0.78755944179777,48.045972425804,0
-0.78742334965959,48.045943739845,0
-0.78728403577259,48.045923092505,0
-0.78714256033709,48.045910640913,0
-0.78700000000002,48.045906479826,0
-0.78685743966298,48.045910640913,0
-0.78671596422748,48.045923092505,0
-0.78657665034048,48.045943739845,0
-0.7864405582023,48.045972425804,0
-0.78630872349981,48.046008932078,0
-0.78618214952599,48.046052980848,0
-0.78606179954562,48.046104236895,0
-0.78594858946564,48.046162310151,0
-0.78584338086524,48.046226758666,0
-0.78574697443939,48.046297091969,0
-0.78566010390523,48.046372774806,0
-0.78558343041776,48.046453231207,0
-0.78551753753772,48.04653784887,0
-0.78546292678918,48.046625983823,0
-0.78542001384171,48.046716965322,0
-0.78538912534516,48.046810100956,0
-0.78537049644213,48.046904681917,0
-0.78536426897642,48.046999988391,0
-0.78700000000003,48.047,0
</coordinates>
</LinearRing>
</outerBoundaryIs>
</Polygon>
</Placemark>
<Placemark>
<name>138</name>
<styleUrl>#stylehap1</styleUrl>
<Polygon>
<outerBoundaryIs>
<LinearRing>
<coordinates>
-0.78700000000003,47.814,0
-0.78537162209934,47.813999988486,0
-0.78537781559154,47.814095295136,0
-0.78539635495437,47.814189876618,0
-0.78542709915782,47.814283013104,0

-0.78546981428166,47.814373995759,0
-0.78552417529377,47.814462132137,0
-0.78558976852213,47.814546751451,0
-0.78566609480171,47.814627209678,0
-0.78575257327223,47.814702894462,0
-0.78584854579791,47.814773229773,0
-0.78595328197585,47.814837680295,0
-0.7860659846945,47.814895755497,0
-0.78618579620055,47.814947013371,0
-0.78631180462735,47.814991063792,0
-0.7864430509356,47.815027571491,0
-0.78657853621354,47.815056258606,0
-0.78671722928061,47.815076906798,0
-0.78685807453718,47.815089358912,0
-0.78700000000002,47.815093520174,0
-0.78714192546289,47.815089358912,0
-0.78728277071946,47.815076906798,0
-0.78742146378653,47.815056258606,0
-0.78755694906447,47.815027571491,0
-0.78768819537272,47.814991063792,0
-0.78781420379952,47.814947013371,0
-0.78793401530557,47.814895755497,0
-0.78804671802422,47.814837680295,0
-0.78815145420216,47.814773229773,0
-0.78824742672784,47.814702894462,0
-0.78833390519836,47.814627209678,0
-0.78841023147795,47.814546751451,0
-0.7884758247063,47.814462132137,0
-0.78853018571841,47.814373995759,0
-0.78857290084225,47.814283013104,0
-0.7886036450457,47.814189876618,0
-0.78862218440853,47.814095295136,0
-0.78862837790073,47.813999988486,0
-0.78862217845387,47.81390468201,0
-0.78860363331729,47.813810101048,0
-0.78857288369644,47.81371696541,0
-0.78853016367619,47.813625983906,0
-0.78847579843742,47.813537848948,0
-0.78841020178055,47.813453231278,0
-0.78833387297478,47.81337277487,0
-0.78824739295722,47.813297092025,0
-0.78815141991056,47.813226758713,0
-0.7880466842536,47.81316231019,0
-0.78793398308202,47.813104236926,0
-0.78781417410212,47.813052980872,0
-0.78768816910385,47.813008932095,0
-0.78755692702226,47.812972425815,0
-0.78742144664075,47.812943739852,0
-0.78728275899102,47.812923092508,0
-0.7871419195082,47.812910640913,0
-0.78700000000002,47.812906479826,0
-0.78685808049187,47.812910640913,0
-0.78671724100905,47.812923092508,0
-0.78657855335932,47.812943739852,0
-0.78644307297781,47.812972425815,0
-0.78631183089622,47.813008932095,0
-0.78618582589795,47.813052980872,0

-0.78606601691805,47.813104236926,0
-0.78595331574647,47.81316231019,0
-0.78584858008951,47.813226758713,0
-0.78575260704285,47.813297092025,0
-0.78566612702526,47.81337277487,0
-0.78558979821952,47.813453231278,0
-0.78552420156265,47.813537848948,0
-0.78546983632388,47.813625983906,0
-0.78542711630363,47.81371696541,0
-0.78539636668278,47.813810101048,0
-0.7853778215462,47.81390468201,0
-0.78537162209934,47.813999988486,0
-0.78700000000003,47.814,0
</coordinates>
</LinearRing>
</outerBoundaryIs>
</Polygon>
</Placemark>
<Placemark>
<name>141</name>
<styleUrl>#stylehap1</styleUrl>
<Polygon>
<outerBoundaryIs>
<LinearRing>
<coordinates>
-0.78600000000006,47.8,0
-0.78437206095707,47.799999988492,0
-0.78437825278151,47.800095295142,0
-0.7843967871493,47.800189876624,0
-0.78442752306835,47.80028301311,0
-0.78447022668141,47.800373995764,0
-0.78452457304392,47.800462132142,0
-0.78459014859535,47.800546751455,0
-0.78466645430517,47.800627209682,0
-0.7847529094696,47.800702894465,0
-0.78484885613022,47.800773229776,0
-0.78495356408099,47.800837680297,0
-0.78506623642522,47.800895755499,0
-0.78518601564072,47.800947013373,0
-0.78531199010663,47.800991063793,0
-0.78544320104217,47.801027571491,0
-0.7855786498048,47.801056258606,0
-0.78571730549193,47.801076906798,0
-0.78585811278843,47.801089358912,0
-0.78600000000006,47.801093520174,0
-0.78614188721167,47.801089358912,0
-0.78628269450817,47.801076906798,0
-0.7864213501953,47.801056258606,0
-0.78655679895795,47.801027571491,0
-0.78668800989349,47.800991063793,0
-0.78681398435941,47.800947013373,0
-0.78693376357491,47.800895755499,0
-0.78704643591914,47.800837680297,0
-0.78715114386988,47.800773229776,0
-0.78724709053053,47.800702894465,0
-0.78733354569496,47.800627209682,0
-0.78740985140475,47.800546751455,0

-0.7874754269562,47.800462132142,0
-0.78752977331869,47.800373995764,0
-0.78757247693178,47.80028301311,0
-0.78760321285083,47.800189876624,0
-0.78762174721859,47.800095295142,0
-0.78762793904305,47.799999988492,0
-0.78762174126845,47.799904682016,0
-0.78760320113132,47.799810101053,0
-0.78757245979902,47.799716965415,0
-0.78752975129325,47.799625983911,0
-0.78747540070729,47.799537848952,0
-0.78740982172995,47.799453231282,0
-0.78733351349589,47.799372774874,0
-0.78724705678558,47.799297092028,0
-0.78715110960437,47.799226758716,0
-0.78704640217419,47.799162310193,0
-0.78693373137586,47.799104236928,0
-0.78681395468458,47.799052980873,0
-0.78668798364458,47.799008932096,0
-0.78655677693249,47.798972425816,0
-0.78642133306254,47.798943739852,0
-0.78628268278866,47.798923092508,0
-0.78614188126154,47.798910640913,0
-0.78600000000006,47.798906479826,0
-0.78585811873859,47.798910640913,0
-0.78571731721144,47.798923092508,0
-0.78557866693756,47.798943739852,0
-0.78544322306762,47.798972425816,0
-0.78531201635555,47.799008932096,0
-0.78518604531553,47.799052980873,0
-0.78506626862426,47.799104236928,0
-0.78495359782593,47.799162310193,0
-0.78484889039574,47.799226758716,0
-0.78475294321455,47.799297092028,0
-0.78466648650421,47.799372774874,0
-0.78459017827018,47.799453231282,0
-0.78452459929284,47.799537848952,0
-0.78447024870685,47.799625983911,0
-0.78442754020111,47.799716965415,0
-0.78439679886881,47.799810101053,0
-0.78437825873167,47.799904682016,0
-0.78437206095707,47.799999988492,0
-0.78600000000006,47.8,0
</coordinates>
</LinearRing>
</outerBoundaryIs>
</Polygon>
</Placemark>
<Placemark>
<name>142</name>
<styleUrl>#stylehap1</styleUrl>
<Polygon>
<outerBoundaryIs>
<LinearRing>
<coordinates>
-0.78600000000006,48.287,0
-0.78435659664348,48.286999988293,0

-0.7843628472343,48.287095294945,0
-0.78438155761573,48.287189876431,0
-0.784412585458,48.287283012924,0
-0.78445569468376,48.287373995589,0
-0.78451055726297,48.287462131979,0
-0.78457675570774,48.287546751306,0
-0.7846537862479,48.287627209549,0
-0.78474106266412,48.287702894348,0
-0.78483792074823,48.287773229677,0
-0.78494362335783,48.287837680215,0
-0.78505736602614,48.287895755434,0
-0.7851782830847,48.287947013323,0
-0.78530545425218,48.287991063758,0
-0.78543791163911,48.288027571468,0
-0.78557464711543,48.288056258593,0
-0.78571461998441,48.288076906792,0
-0.78585676490465,48.28808935891,0
-0.78600000000006,48.288093520174,0
-0.78614323509545,48.28808935891,0
-0.78628538001572,48.288076906792,0
-0.78642535288469,48.288056258593,0
-0.78656208836102,48.288027571468,0
-0.78669454574795,48.287991063758,0
-0.78682171691542,48.287947013323,0
-0.78694263397398,48.287895755434,0
-0.78705637664227,48.287837680215,0
-0.78716207925187,48.287773229677,0
-0.78725893733598,48.287702894348,0
-0.78734621375222,48.287627209549,0
-0.78742324429239,48.287546751306,0
-0.78748944273713,48.287462131979,0
-0.78754430531636,48.287373995589,0
-0.78758741454213,48.287283012924,0
-0.78761844238437,48.287189876431,0
-0.78763715276583,48.287095294945,0
-0.78764340335662,48.286999988293,0
-0.78763714665558,48.286904681819,0
-0.78761843034955,48.286810100861,0
-0.78758739694839,48.28671696523,0
-0.7875442826983,48.286625983736,0
-0.78748941578198,48.286537848789,0
-0.78742321381914,48.286453231133,0
-0.78734618068679,48.286372774741,0
-0.7872589026831,48.286297091912,0
-0.7871620440644,48.286226758617,0
-0.78705634198939,48.286162310111,0
-0.78694260090855,48.286104236863,0
-0.78682168644217,48.286052980824,0
-0.78669451879278,48.286008932061,0
-0.78656206574295,48.285972425793,0
-0.78642533529093,48.285943739839,0
-0.78628536798088,48.285923092502,0
-0.78614322898522,48.285910640912,0
-0.78600000000006,48.285906479827,0
-0.7858567710149,48.285910640912,0
-0.78571463201922,48.285923092502,0
-0.78557466470917,48.285943739839,0

-0.78543793425718,48.285972425793,0
-0.78530548120733,48.286008932061,0
-0.78517831355795,48.286052980824,0
-0.78505739909155,48.286104236863,0
-0.78494365801074,48.286162310111,0
-0.78483795593573,48.286226758617,0
-0.78474109731703,48.286297091912,0
-0.78465381931331,48.286372774741,0
-0.78457678618096,48.286453231133,0
-0.78451058421815,48.286537848789,0
-0.78445571730183,48.286625983736,0
-0.78441260305173,48.28671696523,0
-0.78438156965057,48.286810100861,0
-0.78436285334452,48.286904681819,0
-0.78435659664348,48.286999988293,0
-0.78600000000006,48.287,0
</coordinates>
</LinearRing>
</outerBoundaryIs>
</Polygon>
</Placemark>
<Placemark>
<name>143</name>
<styleUrl>#stylehap1</styleUrl>
<Polygon>
<outerBoundaryIs>
<LinearRing>
<coordinates>
-0.78300000000002,47.863,0
-0.78137008346541,47.862999988466,0
-0.78137628280462,47.863095295117,0
-0.78139483968009,47.863189876599,0
-0.7814256129287,47.863283013086,0
-0.78146836840933,47.863373995742,0
-0.78152278078283,47.863462132121,0
-0.78158843598646,47.863546751436,0
-0.78166483438363,47.863627209665,0
-0.78175139456522,47.86370289445,0
-0.7818474577737,47.863773229763,0
-0.78195229291597,47.863837680287,0
-0.78206510212736,47.863895755491,0
-0.78218502684397,47.863947013366,0
-0.78231115433752,47.863991063788,0
-0.78244252466238,47.864027571488,0
-0.78257813796289,47.864056258605,0
-0.78271696208398,47.864076906797,0
-0.78285794042845,47.864089358911,0
-0.78300000000001,47.864093520174,0
-0.78314205957159,47.864089358911,0
-0.78328303791604,47.864076906797,0
-0.78342186203713,47.864056258605,0
-0.78355747533763,47.864027571488,0
-0.78368884566252,47.863991063788,0
-0.78381497315604,47.863947013366,0
-0.78393489787268,47.863895755491,0
-0.78404770708407,47.863837680287,0
-0.78415254222634,47.863773229763,0

-0.7842486054348,47.86370289445,0
-0.78433516561641,47.863627209665,0
-0.78441156401358,47.863546751436,0
-0.78447721921721,47.863462132121,0
-0.78453163159071,47.863373995742,0
-0.78457438707131,47.863283013086,0
-0.78460516031995,47.863189876599,0
-0.7846237171954,47.863095295117,0
-0.78462991653463,47.862999988466,0
-0.78462371122486,47.862904681991,0
-0.78460514856024,47.862810101029,0
-0.7845743698798,47.862716965392,0
-0.7845316094897,47.862625983889,0
-0.78447719287827,47.862537848931,0
-0.784411534237,47.862453231263,0
-0.78433513330691,47.862372774857,0
-0.78424857157413,47.862297092013,0
-0.7841525078433,47.862226758703,0
-0.78404767322338,47.862162310182,0
-0.78393486556318,47.86210423692,0
-0.78381494337946,47.862052980867,0
-0.78368881932359,47.862008932091,0
-0.78355745323665,47.861972425813,0
-0.78342184484562,47.86194373985,0
-0.78328302615635,47.861923092507,0
-0.78314205360103,47.861910640913,0
-0.78300000000001,47.861906479827,0
-0.78285794639901,47.861910640913,0
-0.78271697384369,47.861923092507,0
-0.78257815515442,47.86194373985,0
-0.78244254676339,47.861972425813,0
-0.78231118067645,47.862008932091,0
-0.78218505662058,47.862052980867,0
-0.78206513443686,47.86210423692,0
-0.78195232677666,47.862162310182,0
-0.78184749215674,47.862226758703,0
-0.78175142842591,47.862297092013,0
-0.7816648666931,47.862372774857,0
-0.78158846576304,47.862453231263,0
-0.78152280712177,47.862537848931,0
-0.78146839051032,47.862625983889,0
-0.78142563012024,47.862716965392,0
-0.7813948514398,47.862810101029,0
-0.78137628877518,47.862904681991,0
-0.78137008346541,47.862999988466,0
-0.78300000000002,47.863,0
</coordinates>
</LinearRing>
</outerBoundaryIs>
</Polygon>
</Placemark>
<Placemark>
<name>144</name>
<styleUrl>#stylehap1</styleUrl>
<Polygon>
<outerBoundaryIs>
<LinearRing>

<coordinates>
-0.78300000000002,48.048,0
-0.78136423721643,48.047999988391,0
-0.78137045877216,48.048095295042,0
-0.78138908218925,48.048189876526,0
-0.78141996579883,48.048283013016,0
-0.78146287462043,48.048373995675,0
-0.78151748214852,48.048462132059,0
-0.78158337283559,48.04854675138,0
-0.78166004525326,48.048627209614,0
-0.78174691590727,48.048702894406,0
-0.78184332367735,48.048773229725,0
-0.78194853484815,48.048837680256,0
-0.78206174869314,48.048895755466,0
-0.78218210356876,48.048947013348,0
-0.7823086834726,48.048991063775,0
-0.78244052501559,48.04902757148,0
-0.7825766247553,48.049056258599,0
-0.78271594683398,48.049076906795,0
-0.78285743086404,48.049089358911,0
-0.78300000000001,48.049093520174,0
-0.78314256913597,48.049089358911,0
-0.78328405316606,48.049076906795,0
-0.78342337524474,48.049056258599,0
-0.78355947498442,48.04902757148,0
-0.78369131652744,48.048991063775,0
-0.78381789643128,48.048947013348,0
-0.78393825130687,48.048895755466,0
-0.78405146515186,48.048837680256,0
-0.78415667632269,48.048773229725,0
-0.78425308409275,48.048702894406,0
-0.78433995474678,48.048627209614,0
-0.78441662716443,48.04854675138,0
-0.78448251785149,48.048462132059,0
-0.78453712537961,48.048373995675,0
-0.78458003420121,48.048283013016,0
-0.78461091781079,48.048189876526,0
-0.78462954122785,48.048095295042,0
-0.78463576278358,48.047999988391,0
-0.78462953519686,48.047904681916,0
-0.78461090593203,48.047810100956,0
-0.78458001683566,48.047716965322,0
-0.78453710305486,48.047625983823,0
-0.78448249124592,48.04753784887,0
-0.7844165970864,48.047453231207,0
-0.78433992211019,48.047372774806,0
-0.78425304988926,48.047297091969,0
-0.78415664159156,48.047226758666,0
-0.78405143094841,48.047162310151,0
-0.78393821867031,48.047104236895,0
-0.78381786635323,48.047052980848,0
-0.78369128992185,48.047008932078,0
-0.7835594526597,48.046972425804,0
-0.78342335787919,48.046943739845,0
-0.7832840412873,48.046923092505,0
-0.78314256310498,48.046910640913,0
-0.78300000000001,48.046906479827,0

-0.78285743689504,48.046910640913,0
-0.78271595871271,48.046923092505,0
-0.78257664212085,48.046943739845,0
-0.78244054734034,48.046972425804,0
-0.78230871007819,48.047008932078,0
-0.78218213364681,48.047052980848,0
-0.78206178132973,48.047104236895,0
-0.78194856905163,48.047162310151,0
-0.78184335840848,48.047226758666,0
-0.78174695011075,48.047297091969,0
-0.78166007788985,48.047372774806,0
-0.78158340291364,48.047453231207,0
-0.78151750875412,48.04753784887,0
-0.78146289694515,48.047625983823,0
-0.78141998316438,48.047716965322,0
-0.78138909406798,48.047810100956,0
-0.78137046480316,48.047904681916,0
-0.78136423721643,48.047999988391,0
-0.78300000000002,48.048,0
</coordinates>
</LinearRing>
</outerBoundaryIs>
</Polygon>
</Placemark>
<Placemark>
<name>146</name>
<styleUrl>#stylehap1</styleUrl>
<Polygon>
<outerBoundaryIs>
<LinearRing>
<coordinates>
-0.78200000000004,48.097,0
-0.78036267885404,48.096999988371,0
-0.78036890633173,48.097095295022,0
-0.78038754748598,48.097189876507,0
-0.7804184605131,48.097283012997,0
-0.78046141020893,48.097373995658,0
-0.78051606975695,48.097462132043,0
-0.78058202321394,48.097546751365,0
-0.78065876867387,48.097627209601,0
-0.78074572208667,48.097702894394,0
-0.78084222170227,48.097773229715,0
-0.78094753310636,48.097837680247,0
-0.78106085480954,48.09789575546,0
-0.78118132434733,48.097947013342,0
-0.78130802484459,48.097991063771,0
-0.78143999199441,48.098027571477,0
-0.78157622139821,48.098056258598,0
-0.78171567621131,48.098076906794,0
-0.78185729503578,48.098089358911,0
-0.78200000000005,48.098093520174,0
-0.7821427049643,48.098089358911,0
-0.78228432378876,48.098076906794,0
-0.78242377860186,48.098056258598,0
-0.78256000800566,48.098027571477,0
-0.78269197515548,48.097991063771,0
-0.78281867565274,48.097947013342,0

-0.78293914519054,48.09789575546,0
-0.78305246689371,48.097837680247,0
-0.7831577782978,48.097773229715,0
-0.7832542779134,48.097702894394,0
-0.78334123132623,48.097627209601,0
-0.78341797678616,48.097546751365,0
-0.78348393024312,48.097462132043,0
-0.78353858979117,48.097373995658,0
-0.78358153948697,48.097283012997,0
-0.78361245251409,48.097189876507,0
-0.78363109366834,48.097095295022,0
-0.78363732114603,48.096999988371,0
-0.78363108762119,48.096904681896,0
-0.78361244060355,48.096810100936,0
-0.78358152207496,48.096716965303,0
-0.78353856740668,48.096625983805,0
-0.78348390356633,48.096537848853,0
-0.78341794662763,48.096453231192,0
-0.78334119860231,48.096372774793,0
-0.7832542436184,48.096297091957,0
-0.78315774347373,48.096226758656,0
-0.7830524325987,48.096162310143,0
-0.78293911246662,48.096104236889,0
-0.78281864549421,48.096052980843,0
-0.78269194847869,48.096008932075,0
-0.78255998562119,48.095972425802,0
-0.78242376118985,48.095943739844,0
-0.78228431187822,48.095923092504,0
-0.78214269891717,48.095910640913,0
-0.78200000000005,48.095906479826,0
-0.7818573010829,48.095910640913,0
-0.78171568812185,48.095923092504,0
-0.78157623881024,48.095943739844,0
-0.7814400143789,48.095972425802,0
-0.78130805152138,48.096008932075,0
-0.78118135450586,48.096052980843,0
-0.78106088753345,48.096104236889,0
-0.78094756740137,48.096162310143,0
-0.78084225652634,48.096226758656,0
-0.78074575638167,48.096297091957,0
-0.78065880139778,48.096372774793,0
-0.78058205337244,48.096453231192,0
-0.78051609643374,48.096537848853,0
-0.78046143259339,48.096625983805,0
-0.78041847792513,48.096716965303,0
-0.78038755939652,48.096810100936,0
-0.78036891237888,48.096904681896,0
-0.78036267885404,48.096999988371,0
-0.78200000000004,48.097,0
</coordinates>
</LinearRing>
</outerBoundaryIs>
</Polygon>
</Placemark>
<Placemark>
<name>147</name>
<styleUrl>#stylehap1</styleUrl>

<Polygon>
<outerBoundaryIs>
<LinearRing>
<coordinates>
-0.780999999999995,48.245,0
-0.77935794657491,48.24499998831,0
-0.77936419203584,48.245095294962,0
-0.77938288705248,48.245189876448,0
-0.77941388941181,48.24528301294,0
-0.77945696323029,48.245373995604,0
-0.77951178074727,48.245462131993,0
-0.77957792481758,48.245546751319,0
-0.77965489208491,48.24562720956,0
-0.77974209681133,48.245702894359,0
-0.77983887533424,48.245773229685,0
-0.77994449111676,48.245837680222,0
-0.78005814035289,48.24589575544,0
-0.78017895808533,48.245947013327,0
-0.7803060247887,48.245991063761,0
-0.78043837336873,48.24602757147,0
-0.78057499652351,48.246056258594,0
-0.78071485441126,48.246076906792,0
-0.78085688256584,48.24608935891,0
-0.780999999999996,48.246093520174,0
-0.78114311743406,48.24608935891,0
-0.78128514558864,48.246076906792,0
-0.78142500347639,48.246056258594,0
-0.78156162663117,48.24602757147,0
-0.7816939752112,48.245991063761,0
-0.78182104191457,48.245947013327,0
-0.781941859647,48.24589575544,0
-0.78205550888314,48.245837680222,0
-0.78216112466566,48.245773229685,0
-0.78225790318857,48.245702894359,0
-0.78234510791499,48.24562720956,0
-0.78242207518232,48.245546751319,0
-0.78248821925262,48.245462131993,0
-0.78254303676961,48.245373995604,0
-0.78258611058809,48.24528301294,0
-0.78261711294742,48.245189876448,0
-0.78263580796406,48.245095294962,0
-0.78264205342499,48.24499998831,0
-0.78263580186785,48.244904681836,0
-0.78261710094021,48.244810100878,0
-0.78258609303473,48.244716965246,0
-0.78254301420342,48.244625983751,0
-0.78248819235933,48.244537848803,0
-0.78242204477899,48.244453231146,0
-0.78234507492546,48.244372774752,0
-0.78225786861518,48.244297091922,0
-0.78216108955892,48.244226758625,0
-0.78205547430977,48.244162310118,0
-0.78194182665748,48.244104236869,0
-0.78182101151124,48.244052980828,0
-0.78169394831788,48.244008932064,0
-0.78156160406501,48.243972425795,0
-0.78142498592303,48.24394373984,0

-0.78128513358143,48.243923092503,0
-0.78114311133783,48.243910640912,0
-0.78099999999996,48.243906479826,0
-0.78085688866207,48.243910640912,0
-0.78071486641846,48.243923092503,0
-0.78057501407687,48.24394373984,0
-0.78043839593489,48.243972425795,0
-0.78030605168202,48.244008932064,0
-0.78017898848865,48.244052980828,0
-0.78005817334242,48.244104236869,0
-0.77994452569013,48.244162310118,0
-0.77983891044098,48.244226758625,0
-0.77974213138472,48.244297091922,0
-0.77965492507444,48.244372774752,0
-0.77957795522091,48.244453231146,0
-0.77951180764057,48.244537848803,0
-0.77945698579648,48.244625983751,0
-0.77941390696516,48.244716965246,0
-0.77938289905968,48.244810100878,0
-0.77936419813205,48.244904681836,0
-0.77935794657491,48.24499998831,0
-0.78099999999995,48.245,0
</coordinates>
</LinearRing>
</outerBoundaryIs>
</Polygon>
</Placemark>
<Placemark>
<name>148</name>
<styleUrl>#stylehap1</styleUrl>
<Polygon>
<outerBoundaryIs>
<LinearRing>
<coordinates>
-0.779,48.008,0
-0.777365506266,48.007999988407,0
-0.7773717229992,48.008095295058,0
-0.77739033197206,48.008189876542,0
-0.7774211916255,48.008283013031,0
-0.77746406716125,48.00837399569,0
-0.77751863232703,48.008462132073,0
-0.77758447189752,48.008546751392,0
-0.77766108483335,48.008627209625,0
-0.7777478880929,48.008702894415,0
-0.77784422106873,48.008773229734,0
-0.77794935061474,48.008837680262,0
-0.77806247662561,48.008895755472,0
-0.7781827381263,48.008947013352,0
-0.77830921982509,48.008991063778,0
-0.77844095908043,48.009027571482,0
-0.77857695322839,48.009056258601,0
-0.77871616721506,48.009076906795,0
-0.77885754147555,48.009089358911,0
-0.77899999999999,48.009093520174,0
-0.77914245852443,48.009089358911,0
-0.77928383278495,48.009076906795,0
-0.77942304677159,48.009056258601,0

-0.77955904091958,48.009027571482,0
-0.7796907801749,48.008991063778,0
-0.77981726187368,48.008947013352,0
-0.77993752337438,48.008895755472,0
-0.78005064938524,48.008837680262,0
-0.78015577893126,48.008773229734,0
-0.78025211190708,48.008702894415,0
-0.78033891516664,48.008627209625,0
-0.78041552810247,48.008546751392,0
-0.78048136767298,48.008462132073,0
-0.78053593283873,48.00837399569,0
-0.78057880837451,48.008283013031,0
-0.78060966802795,48.008189876542,0
-0.78062827700078,48.008095295058,0
-0.78063449373398,48.007999988407,0
-0.78062827098292,48.007904681932,0
-0.78060965617506,48.007810100972,0
-0.78057879104677,48.007716965337,0
-0.78053591056261,48.007625983837,0
-0.78048134112535,48.007537848883,0
-0.78041549808994,48.007453231219,0
-0.78033888260114,48.007372774817,0
-0.7802520777781,48.007297091979,0
-0.78015574427578,48.007226758674,0
-0.78005061525626,48.007162310158,0
-0.77993749080888,48.007104236901,0
-0.77981723186116,48.007052980852,0
-0.77969075362726,48.007008932081,0
-0.77955901864346,48.006972425806,0
-0.77942302944386,48.006943739846,0
-0.77928382093207,48.006923092506,0
-0.77914245250657,48.006910640913,0
-0.77899999999999,48.006906479826,0
-0.77885754749342,48.006910640913,0
-0.77871617906791,48.006923092506,0
-0.77857697055613,48.006943739846,0
-0.77844098135653,48.006972425806,0
-0.77830924637272,48.007008932081,0
-0.77818276813883,48.007052980852,0
-0.77806250919113,48.007104236901,0
-0.77794938474372,48.007162310158,0
-0.77784425572421,48.007226758674,0
-0.77774792222188,48.007297091979,0
-0.77766111739887,48.007372774817,0
-0.77758450191004,48.007453231219,0
-0.77751865887466,48.007537848883,0
-0.77746408943738,48.007625983837,0
-0.77742120895324,48.007716965337,0
-0.77739034382492,48.007810100972,0
-0.77737172901706,48.007904681932,0
-0.777365506266,48.007999988407,0
-0.779,48.008,0
</coordinates>
</LinearRing>
</outerBoundaryIs>
</Polygon>
</Placemark>

<Placemark>
<name>149</name>
<styleUrl>#stylehap1</styleUrl>
<Polygon>
<outerBoundaryIs>
<LinearRing>
<coordinates>
-0.77800000000002,48.253,0
-0.7763576896842,48.252999988307,0
-0.77636393612133,48.253095294959,0
-0.77638263406189,48.253189876445,0
-0.7764136412706,48.253283012937,0
-0.77645672182705,48.253373995601,0
-0.77651154791934,48.25346213199,0
-0.77657770233706,48.253546751317,0
-0.77665468164516,48.253627209558,0
-0.77674190001409,48.253702894357,0
-0.77683869367742,48.253773229684,0
-0.77694432598304,48.253837680221,0
-0.77705799299924,48.253895755439,0
-0.77717882963335,48.253947013327,0
-0.77730591621616,48.25399106376,0
-0.77743828550203,48.25402757147,0
-0.77757493003152,48.254056258594,0
-0.77771480980008,48.254076906792,0
-0.77785686017508,48.25408935891,0
-0.77800000000001,48.254093520174,0
-0.77814313982496,48.25408935891,0
-0.77828519019996,48.254076906792,0
-0.77842506996852,48.254056258594,0
-0.77856171449801,48.25402757147,0
-0.77869408378388,48.25399106376,0
-0.77882117036669,48.253947013327,0
-0.7789420070008,48.253895755439,0
-0.779055674017,48.253837680221,0
-0.77916130632262,48.253773229684,0
-0.77925809998595,48.253702894357,0
-0.77934531835488,48.253627209558,0
-0.77942229766298,48.253546751317,0
-0.7794884520807,48.25346213199,0
-0.77954327817299,48.253373995601,0
-0.77958635872944,48.253283012937,0
-0.77961736593815,48.253189876445,0
-0.77963606387871,48.253095294959,0
-0.77964231031584,48.252999988307,0
-0.77963605777983,48.252904681833,0
-0.77961735392568,48.252810100874,0
-0.7795863411684,48.252716965243,0
-0.77954325559693,48.252625983749,0
-0.77948842517562,48.252537848801,0
-0.77942226724637,48.252453231144,0
-0.7793452853509,48.25237277475,0
-0.77925806539744,48.25229709192,0
-0.77916127120052,48.252226758624,0
-0.77905563942849,48.252162310117,0
-0.77894197399682,48.252104236868,0
-0.77882113995008,48.252052980827,0

-0.7786940568788,48.252008932063,0
-0.77856169192195,48.251972425795,0
-0.77842505240748,48.25194373984,0
-0.77828517818749,48.251923092502,0
-0.77814313372608,48.251910640912,0
-0.77800000000001,48.251906479826,0
-0.77785686627396,48.251910640912,0
-0.77771482181255,48.251923092502,0
-0.77757494759256,48.25194373984,0
-0.77743830807809,48.251972425795,0
-0.77730594312124,48.252008932063,0
-0.77717886004996,48.252052980827,0
-0.77705802600322,48.252104236868,0
-0.77694436057155,48.252162310117,0
-0.77683872879952,48.252226758624,0
-0.7767419346026,48.25229709192,0
-0.77665471464911,48.25237277475,0
-0.77657773275367,48.252453231144,0
-0.77651157482442,48.252537848801,0
-0.77645674440311,48.252625983749,0
-0.77641365883164,48.252716965243,0
-0.77638264607436,48.252810100874,0
-0.77636394222021,48.252904681833,0
-0.7763576896842,48.252999988307,0
-0.77800000000002,48.253,0
</coordinates>
</LinearRing>
</outerBoundaryIs>
</Polygon>
</Placemark>
<Placemark>
<name>150</name>
<styleUrl>#stylehap1</styleUrl>
<Polygon>
<outerBoundaryIs>
<LinearRing>
<coordinates>
-0.77700000000004,48.011,0
-0.77536541118328,48.010999988406,0
-0.77537162827778,48.011095295057,0
-0.77539023833287,48.011189876541,0
-0.77542109978121,48.01128301303,0
-0.7754639778109,48.011373995689,0
-0.77551854615062,48.011462132072,0
-0.775584389551,48.011546751391,0
-0.77566100694348,48.011627209624,0
-0.77574781525253,48.011702894415,0
-0.77584415383227,48.011773229733,0
-0.77594928949397,48.011837680262,0
-0.77606242208577,48.011895755471,0
-0.77618269058251,48.011947013351,0
-0.77630917963924,48.011991063778,0
-0.77644092655842,48.012027571481,0
-0.77657692861777,48.0120562586,0
-0.77671615070317,48.012076906795,0
-0.7768575331881,48.012089358911,0
-0.77700000000005,48.012093520174,0

-0.77714246681197,48.012089358911,0
-0.77728384929692,48.012076906795,0
-0.7774230713823,48.0120562586,0
-0.77755907344167,48.012027571481,0
-0.77769082036085,48.011991063778,0
-0.77781730941759,48.011947013351,0
-0.77793757791433,48.011895755471,0
-0.77805071050612,48.011837680262,0
-0.77815584616783,48.011773229733,0
-0.77825218474757,48.011702894415,0
-0.77833899305659,48.011627209624,0
-0.77841561044907,48.011546751391,0
-0.77848145384945,48.011462132072,0
-0.77853602218919,48.011373995689,0
-0.77857890021888,48.01128301303,0
-0.77860976166722,48.011189876541,0
-0.77862837172229,48.011095295057,0
-0.77863458881682,48.010999988406,0
-0.77862836570346,48.010904681931,0
-0.77860974981241,48.01081010097,0
-0.77857888288832,48.010716965336,0
-0.77853599990943,48.010625983836,0
-0.77848142729747,48.010537848882,0
-0.77841558043163,48.010453231218,0
-0.77833896048578,48.010372774816,0
-0.77825215061299,48.010297091978,0
-0.77815581150668,48.010226758673,0
-0.77805067637154,48.010162310157,0
-0.77793754534352,48.0101042369,0
-0.77781727940015,48.010052980852,0
-0.77769079380887,48.010008932081,0
-0.77755905116191,48.009972425806,0
-0.77742305405174,48.009943739846,0
-0.77728383744211,48.009923092505,0
-0.77714246079314,48.009910640913,0
-0.77700000000005,48.009906479826,0
-0.77685753920696,48.009910640913,0
-0.77671616255799,48.009923092505,0
-0.77657694594836,48.009943739846,0
-0.77644094883818,48.009972425806,0
-0.77630920619123,48.010008932081,0
-0.77618272059992,48.010052980852,0
-0.77606245465658,48.0101042369,0
-0.77594932362853,48.010162310157,0
-0.77584418849342,48.010226758673,0
-0.77574784938708,48.010297091978,0
-0.77566103951432,48.010372774816,0
-0.77558441956844,48.010453231218,0
-0.7755185727026,48.010537848882,0
-0.77546400009066,48.010625983836,0
-0.77542111711178,48.010716965336,0
-0.77539025018767,48.01081010097,0
-0.77537163429664,48.010904681931,0
-0.77536541118328,48.010999988406,0
-0.77700000000004,48.011,0
</coordinates>
</LinearRing>

</outerBoundaryIs>
</Polygon>
</Placemark>
<Placemark>
<name>151</name>
<styleUrl>#stylehap1</styleUrl>
<Polygon>
<outerBoundaryIs>
<LinearRing>
<coordinates>
-0.77700000000004,47.763,0
-0.77537321919006,47.762999988507,0
-0.77537940661308,47.763095295157,0
-0.7753979277979,47.763189876638,0
-0.77542864185272,47.763283013123,0
-0.77547131508652,47.763373995777,0
-0.77552562278585,47.763462132154,0
-0.77559115168434,47.763546751467,0
-0.77566740310642,47.763627209692,0
-0.77575379676142,47.763702894474,0
-0.77584967515901,47.763773229783,0
-0.77595430861266,47.763837680303,0
-0.77606690079267,47.763895755504,0
-0.77618659478699,47.763947013376,0
-0.7763124796234,47.763991063796,0
-0.7764435972033,47.764027571493,0
-0.77657894959476,47.764056258607,0
-0.77671750662875,47.764076906798,0
-0.77685821374106,47.764089358912,0
-0.77700000000005,47.764093520174,0
-0.77714178625901,47.764089358912,0
-0.77728249337135,47.764076906798,0
-0.77742105040534,47.764056258607,0
-0.7775564027968,47.764027571493,0
-0.7776875203767,47.763991063796,0
-0.77781340521308,47.763947013376,0
-0.7779330992074,47.763895755504,0
-0.77804569138741,47.763837680303,0
-0.77815032484106,47.763773229783,0
-0.77824620323868,47.763702894474,0
-0.77833259689368,47.763627209692,0
-0.77840884831576,47.763546751467,0
-0.77847437721422,47.763462132154,0
-0.77852868491355,47.763373995777,0
-0.77857135814738,47.763283013123,0
-0.77860207220218,47.763189876638,0
-0.77862059338702,47.763095295157,0
-0.77862678081003,47.762999988507,0
-0.77862058744882,47.762904682031,0
-0.77860206050621,47.762810101068,0
-0.778571341049,47.762716965429,0
-0.77852866293233,47.762625983925,0
-0.778474351018,47.762537848965,0
-0.77840881870052,47.762453231293,0
-0.77833256475926,47.762372774884,0
-0.77824616956146,47.762297092037,0
-0.77815029064432,47.762226758723,0

-0.77804565771022,47.762162310199,0
-0.77793306707298,47.762104236933,0
-0.77781337559784,47.762052980877,0
-0.77768749418048,47.762008932099,0
-0.77755638081555,47.761972425818,0
-0.77742103330698,47.761943739853,0
-0.77728248167536,47.761923092508,0
-0.77714178032082,47.761910640914,0
-0.77700000000005,47.761906479827,0
-0.77685821967925,47.761910640914,0
-0.77671751832471,47.761923092508,0
-0.77657896669312,47.761943739853,0
-0.77644361918452,47.761972425818,0
-0.77631250581962,47.762008932099,0
-0.77618662440223,47.762052980877,0
-0.77606693292709,47.762104236933,0
-0.77595434228988,47.762162310199,0
-0.77584970935575,47.762226758723,0
-0.77575383043863,47.762297092037,0
-0.77566743524084,47.762372774884,0
-0.77559118129958,47.762453231293,0
-0.77552564898207,47.762537848965,0
-0.77547133706776,47.762625983925,0
-0.77542865895107,47.762716965429,0
-0.77539793949386,47.762810101068,0
-0.77537941255127,47.762904682031,0
-0.77537321919006,47.762999988507,0
-0.77700000000004,47.763,0
</coordinates>
</LinearRing>
</outerBoundaryIs>
</Polygon>
</Placemark>
<Placemark>
<name>152</name>
<styleUrl>#stylehap1</styleUrl>
<Polygon>
<outerBoundaryIs>
<LinearRing>
<coordinates>
-0.77700000000004,48.005,0
-0.7753656013333,48.004999988408,0
-0.77537181770523,48.005095295059,0
-0.77539042559602,48.005189876543,0
-0.77542128345486,48.005283013032,0
-0.77546415649711,48.005373995691,0
-0.7755187184894,48.005462132074,0
-0.77558455423068,48.005546751393,0
-0.77566116271058,48.005627209626,0
-0.77574796092147,48.005702894416,0
-0.77584428829431,48.005773229734,0
-0.77594941172562,48.005837680263,0
-0.77606253115666,48.005895755472,0
-0.77618278566242,48.005947013352,0
-0.77630926000446,48.005991063778,0
-0.7764409915972,48.006027571482,0
-0.77657697783507,48.006056258601,0

-0.77671618372433,48.006076906795,0
-0.77685754976177,48.006089358911,0
-0.77700000000005,48.006093520174,0
-0.77714245023833,48.006089358911,0
-0.77728381627577,48.006076906795,0
-0.77742302216502,48.006056258601,0
-0.77755900840289,48.006027571482,0
-0.77769073999564,48.005991063778,0
-0.77781721433768,48.005947013352,0
-0.77793746884344,48.005895755472,0
-0.77805058827445,48.005837680263,0
-0.77815571170578,48.005773229734,0
-0.77825203907862,48.005702894416,0
-0.77833883728949,48.005627209626,0
-0.77841544576941,48.005546751393,0
-0.77848128151067,48.005462132074,0
-0.77853584350296,48.005373995691,0
-0.77857871654523,48.005283013032,0
-0.77860957440405,48.005189876543,0
-0.77862818229486,48.005095295059,0
-0.77863439866677,48.004999988408,0
-0.77862817627799,48.004904681934,0
-0.77860956255313,48.004810100973,0
-0.77857869922032,48.004716965338,0
-0.7785358212305,48.004625983838,0
-0.77848125496736,48.004537848884,0
-0.7784154157618,48.00445323122,0
-0.77833880472931,48.004372774818,0
-0.77825200495521,48.004297091979,0
-0.77815567705596,48.004226758674,0
-0.77805055415105,48.004162310158,0
-0.77793743628326,48.004104236901,0
-0.77781718433004,48.004052980852,0
-0.77769071345233,48.004008932081,0
-0.77755898613041,48.003972425806,0
-0.77742300484011,48.003943739846,0
-0.77728380442484,48.003923092506,0
-0.77714244422146,48.003910640913,0
-0.77700000000005,48.003906479826,0
-0.77685755577864,48.003910640913,0
-0.77671619557525,48.003923092506,0
-0.77657699515996,48.003943739846,0
-0.77644101386969,48.003972425806,0
-0.77630928654776,48.004008932081,0
-0.77618281567004,48.004052980852,0
-0.77606256371683,48.004104236901,0
-0.77594944584903,48.004162310158,0
-0.77584432294411,48.004226758674,0
-0.77574799504488,48.004297091979,0
-0.77566119527076,48.004372774818,0
-0.7755845842383,48.00445323122,0
-0.77551874503271,48.004537848884,0
-0.77546417876959,48.004625983838,0
-0.77542130077978,48.004716965338,0
-0.77539043744697,48.004810100973,0
-0.77537182372211,48.004904681934,0
-0.7753656013333,48.004999988408,0

-0.77700000000004,48.005,0
</coordinates>
</LinearRing>
</outerBoundaryIs>
</Polygon>
</Placemark>
<Placemark>
<name>153</name>
<styleUrl>#stylehap1</styleUrl>
<Polygon>
<outerBoundaryIs>
<LinearRing>
<coordinates>
-0.77700000000004,47.788,0
-0.77537243685524,47.787999988496,0
-0.77537862725123,47.788095295147,0
-0.77539715734056,47.788189876629,0
-0.77542788616369,47.788283013114,0
-0.77547057991733,47.788373995768,0
-0.77552491373192,47.788462132146,0
-0.77559047414238,47.788546751459,0
-0.7756667622334,47.788627209685,0
-0.77575319743528,47.788702894468,0
-0.77584912194153,47.788773229778,0
-0.77595380571466,47.788837680299,0
-0.77606645204203,47.788895755501,0
-0.77618620359945,47.788947013374,0
-0.7763121489766,47.788991063794,0
-0.77644332961407,47.789027571492,0
-0.77657874709999,47.789056258607,0
-0.77671737076978,47.789076906798,0
-0.77685814555205,47.789089358912,0
-0.77700000000005,47.789093520174,0
-0.77714185444802,47.789089358912,0
-0.7772826292303,47.789076906798,0
-0.77742125290011,47.789056258607,0
-0.777556670386,47.789027571492,0
-0.77768785102348,47.788991063794,0
-0.77781379640062,47.788947013374,0
-0.77793354795807,47.788895755501,0
-0.77804619428544,47.788837680299,0
-0.77815087805855,47.788773229778,0
-0.7782468025648,47.788702894468,0
-0.77833323776667,47.788627209685,0
-0.77840952585772,47.788546751459,0
-0.77847508626817,47.788462132146,0
-0.77852942008276,47.788373995768,0
-0.77857211383641,47.788283013114,0
-0.77860284265954,47.788189876629,0
-0.77862137274887,47.788095295147,0
-0.77862756314486,47.787999988496,0
-0.7786213668026,47.787904682021,0
-0.77860283094767,47.787810101058,0
-0.77857209671482,47.78771696542,0
-0.77852939807167,47.787625983916,0
-0.77847506003636,47.787537848956,0
-0.77840949620223,47.787453231286,0

-0.77833320558861,47.787372774877,0
-0.77824676884183,47.787297092031,0
-0.77815084381537,47.787226758718,0
-0.77804616056248,47.787162310195,0
-0.77793351577999,47.78710423693,0
-0.77781376674515,47.787052980874,0
-0.77768782479169,47.787008932097,0
-0.77755664837491,47.786972425817,0
-0.77742123577852,47.786943739852,0
-0.77728261751845,47.786923092508,0
-0.77714184850176,47.786910640914,0
-0.77700000000005,47.786906479826,0
-0.77685815149831,47.786910640914,0
-0.77671738248165,47.786923092508,0
-0.77657876422158,47.786943739852,0
-0.77644335162519,47.786972425817,0
-0.77631217520841,47.787008932097,0
-0.77618623325494,47.787052980874,0
-0.77606648422011,47.78710423693,0
-0.77595383943762,47.787162310195,0
-0.77584915618473,47.787226758718,0
-0.77575323115824,47.787297092031,0
-0.77566679441148,47.787372774877,0
-0.77559050379785,47.787453231286,0
-0.77552493996371,47.787537848956,0
-0.77547060192842,47.787625983916,0
-0.77542790328528,47.78771696542,0
-0.7753971690524,47.787810101058,0
-0.77537863319749,47.787904682021,0
-0.77537243685524,47.787999988496,0
-0.77700000000004,47.788,0
</coordinates>
</LinearRing>
</outerBoundaryIs>
</Polygon>
</Placemark>
<Placemark>
<name>154</name>
<styleUrl>#stylehap1</styleUrl>
<Polygon>
<outerBoundaryIs>
<LinearRing>
<coordinates>
-0.77600000000007,48.229,0
-0.77435846001959,48.228999988317,0
-0.77436470352936,48.229095294969,0
-0.77438339270202,48.229189876455,0
-0.77441438536896,48.229283012947,0
-0.77445744572036,48.22937399561,0
-0.77451224609794,48.229462131998,0
-0.77457836948704,48.229546751324,0
-0.77465531268861,48.229627209565,0
-0.77474249014793,48.229702894362,0
-0.77483923840985,48.229773229688,0
-0.77494482116778,48.229837680225,0
-0.77505843486714,48.229895755442,0
-0.77517921482102,48.229947013329,0

-0.77530624179164,48.229991063762,0
-0.7754385489871,48.230027571471,0
-0.77557512942052,48.230056258595,0
-0.77571494357534,48.230076906793,0
-0.77585692731828,48.23008935891,0
-0.77600000000006,48.230093520174,0
-0.77614307268185,48.23008935891,0
-0.77628505642479,48.230076906793,0
-0.77642487057961,48.230056258595,0
-0.77656145101305,48.230027571471,0
-0.77669375820852,48.229991063762,0
-0.77682078517911,48.229947013329,0
-0.77694156513298,48.229895755442,0
-0.77705517883235,48.229837680225,0
-0.77716076159028,48.229773229688,0
-0.77725750985222,48.229702894362,0
-0.77734468731151,48.229627209565,0
-0.77742163051312,48.229546751324,0
-0.77748775390218,48.229462131998,0
-0.7775425542798,48.22937399561,0
-0.77758561463117,48.229283012947,0
-0.7776166072981,48.229189876455,0
-0.77763529647076,48.229095294969,0
-0.77764153998054,48.228999988317,0
-0.77763529037987,48.228904681843,0
-0.77761659530138,48.228810100884,0
-0.77758559709316,48.228716965253,0
-0.77754253173335,48.228625983757,0
-0.7774877270324,48.228537848809,0
-0.77742160013638,48.228453231151,0
-0.77734465435084,48.228372774756,0
-0.77725747530906,48.228297091926,0
-0.77716072651425,48.228226758629,0
-0.77705514428921,48.228162310121,0
-0.77694153217231,48.228104236871,0
-0.77682075480239,48.228052980829,0
-0.77669373133871,48.228008932065,0
-0.77656142846661,48.227972425796,0
-0.77642485304159,48.22794373984,0
-0.77628504442809,48.227923092503,0
-0.77614306659099,48.227910640912,0
-0.77600000000006,48.227906479826,0
-0.77585693340917,48.227910640912,0
-0.77571495557203,48.227923092503,0
-0.77557514695853,48.22794373984,0
-0.77543857153352,48.227972425796,0
-0.77530626866142,48.228008932065,0
-0.77517924519774,48.228052980829,0
-0.77505846782782,48.228104236871,0
-0.77494485571095,48.228162310121,0
-0.77483927348587,48.228226758629,0
-0.77474252469107,48.228297091926,0
-0.77465534564931,48.228372774756,0
-0.77457839986375,48.228453231151,0
-0.77451227296773,48.228537848809,0
-0.77445746826677,48.228625983757,0
-0.77441440290697,48.228716965253,0

-0.77438340469875,48.228810100884,0
-0.77436470962025,48.228904681843,0
-0.77435846001959,48.228999988317,0
-0.77600000000007,48.229,0
</coordinates>
</LinearRing>
</outerBoundaryIs>
</Polygon>
</Placemark>
<Placemark>
<name>155</name>
<styleUrl>#stylehap1</styleUrl>
<Polygon>
<outerBoundaryIs>
<LinearRing>
<coordinates>
-0.77300000000002,48.2,0
-0.77135938949394,48.199999988329,0
-0.7713656294716,48.20009529498,0
-0.77138430806507,48.200189876466,0
-0.77141528318611,48.200283012958,0
-0.77145831915837,48.200373995621,0
-0.77151308850904,48.200462132008,0
-0.77157917445947,48.200546751333,0
-0.77165607409551,48.200627209573,0
-0.77174320219392,48.200702894369,0
-0.77183989567521,48.200773229694,0
-0.77194541864973,48.20083768023,0
-0.77205896801779,48.200895755446,0
-0.77217967958222,48.200947013332,0
-0.77230663462571,48.200991063764,0
-0.77243886690387,48.201027571472,0
-0.77257537000004,48.201056258595,0
-0.77271510498632,48.201076906793,0
-0.77285700833209,48.20108935891,0
-0.77300000000003,48.201093520174,0
-0.77314299166798,48.20108935891,0
-0.77328489501372,48.201076906793,0
-0.77342463,48.201056258595,0
-0.77356113309618,48.201027571472,0
-0.77369336537434,48.200991063764,0
-0.77382032041782,48.200947013332,0
-0.77394103198225,48.200895755446,0
-0.77405458135034,48.20083768023,0
-0.77416010432483,48.200773229694,0
-0.77425679780612,48.200702894369,0
-0.77434392590453,48.200627209573,0
-0.77442082554057,48.200546751333,0
-0.774486911491,48.200462132008,0
-0.77454168084167,48.200373995621,0
-0.77458471681394,48.200283012958,0
-0.77461569193497,48.200189876466,0
-0.77463437052844,48.20009529498,0
-0.7746406105061,48.199999988329,0
-0.77463436444722,48.199904681855,0
-0.77461567995728,48.199810100895,0
-0.77458469930368,48.199716965264,0

-0.77454165833095,48.199625983768,0
-0.77448688466376,48.199537848819,0
-0.77442079521195,48.19945323116,0
-0.77434389299604,48.199372774764,0
-0.77425676331769,48.199297091933,0
-0.77416006930435,48.199226758634,0
-0.7740545468619,48.199162310126,0
-0.77394099907376,48.199104236875,0
-0.7738202900892,48.199052980832,0
-0.77369333854709,48.199008932067,0
-0.77356111058546,48.198972425797,0
-0.77342461248978,48.198943739841,0
-0.77328488303601,48.198923092503,0
-0.77314298558673,48.198910640912,0
-0.77300000000003,48.198906479826,0
-0.77285701441333,48.198910640912,0
-0.77271511696403,48.198923092503,0
-0.77257538751029,48.198943739841,0
-0.77243888941459,48.198972425797,0
-0.77230666145295,48.199008932067,0
-0.77217970991084,48.199052980832,0
-0.77205900092628,48.199104236875,0
-0.77194545313816,48.199162310126,0
-0.77183993069569,48.199226758634,0
-0.77174323668235,48.199297091933,0
-0.771656107004,48.199372774764,0
-0.77157920478809,48.19945323116,0
-0.77151311533628,48.199537848819,0
-0.77145834166909,48.199625983768,0
-0.77141530069636,48.199716965264,0
-0.77138432004279,48.199810100895,0
-0.77136563555284,48.199904681855,0
-0.77135938949394,48.199999988329,0
-0.77300000000002,48.2,0
</coordinates>
</LinearRing>
</outerBoundaryIs>
</Polygon>
</Placemark>
<Placemark>
<name>156</name>
<styleUrl>#stylehap1</styleUrl>
<Polygon>
<outerBoundaryIs>
<LinearRing>
<coordinates>
-0.77300000000002,47.886,0
-0.7713693598348,47.885999988457,0
-0.77137556192391,47.886095295107,0
-0.7713941270357,47.88618987659,0
-0.77142491394446,47.886283013077,0
-0.77146768840514,47.886373995733,0
-0.77152212493429,47.886462132113,0
-0.77158780928531,47.886546751429,0
-0.77166424159989,47.886627209658,0
-0.77175084021082,47.886702894445,0
-0.77184694606808,47.886773229758,0

-0.77195182775399,47.886837680283,0
-0.77206468704968,47.886895755488,0
-0.77218466501005,47.886947013364,0
-0.77231084850151,47.886991063787,0
-0.77244227715233,47.887027571487,0
-0.77257795066275,47.887056258604,0
-0.7727168364195,47.887076906797,0
-0.77285787735616,47.887089358911,0
-0.77300000000003,47.887093520174,0
-0.7731421226439,47.887089358911,0
-0.77328316358054,47.887076906797,0
-0.7734220493373,47.887056258604,0
-0.77355772284774,47.887027571487,0
-0.77368915149853,47.886991063787,0
-0.77381533499001,47.886947013364,0
-0.77393531295039,47.886895755488,0
-0.77404817224607,47.886837680283,0
-0.77415305393199,47.886773229758,0
-0.77424915978922,47.886702894445,0
-0.77433575840015,47.886627209658,0
-0.77441219071473,47.886546751429,0
-0.77447787506578,47.886462132113,0
-0.77453231159491,47.886373995733,0
-0.77457508605561,47.886283013077,0
-0.77460587296437,47.88618987659,0
-0.77462443807613,47.886095295107,0
-0.77463064016524,47.885999988457,0
-0.77462443209812,47.885904681982,0
-0.77460586118995,47.88581010102,0
-0.77457506884257,47.885716965383,0
-0.77453228946626,47.885625983881,0
-0.77447784869386,47.885537848924,0
-0.77441216090087,47.885453231256,0
-0.77433572605022,47.88537277485,0
-0.77424912588616,47.885297092008,0
-0.77415301950592,47.885226758698,0
-0.77404813834299,47.885162310178,0
-0.77393528060046,47.885104236917,0
-0.77381530517615,47.885052980864,0
-0.77368912512664,47.88500893209,0
-0.77355770071907,47.884972425812,0
-0.77342203212426,47.88494373985,0
-0.77328315180613,47.884923092507,0
-0.77314211666586,47.884910640913,0
-0.77300000000003,47.884906479826,0
-0.77285788333418,47.884910640913,0
-0.77271684819391,47.884923092507,0
-0.77257796787578,47.88494373985,0
-0.77244229928097,47.884972425812,0
-0.77231087487342,47.88500893209,0
-0.77218469482389,47.885052980864,0
-0.77206471939958,47.885104236917,0
-0.77195186165705,47.885162310178,0
-0.77184698049415,47.885226758698,0
-0.77175087411388,47.885297092008,0
-0.77166427394982,47.88537277485,0
-0.77158783909917,47.885453231256,0

-0.77152215130618,47.885537848924,0
-0.77146771053381,47.885625983881,0
-0.77142493115749,47.885716965383,0
-0.77139413881011,47.88581010102,0
-0.77137556790193,47.885904681982,0
-0.7713693598348,47.885999988457,0
-0.77300000000002,47.886,0
</coordinates>
</LinearRing>
</outerBoundaryIs>
</Polygon>
</Placemark>
<Placemark>
<name>157</name>
<styleUrl>#stylehap1</styleUrl>
<Polygon>
<outerBoundaryIs>
<LinearRing>
<coordinates>
-0.77300000000002,47.887,0
-0.77136932835207,47.886999988456,0
-0.77137553056081,47.887095295107,0
-0.77139409603094,47.88718987659,0
-0.77142488353398,47.887283013077,0
-0.77146765882046,47.887373995733,0
-0.77152209640052,47.887462132113,0
-0.77158778201966,47.887546751429,0
-0.77166421580986,47.887627209658,0
-0.77175081609273,47.887702894444,0
-0.77184692380547,47.887773229758,0
-0.77195180751635,47.887837680283,0
-0.77206466899102,47.887895755488,0
-0.77218464926786,47.887947013364,0
-0.77231083519562,47.887991063787,0
-0.77244226638399,47.888027571487,0
-0.77257794251396,47.888056258604,0
-0.77271683095225,47.888076906797,0
-0.77285787461208,47.888089358911,0
-0.77300000000003,47.888093520174,0
-0.77314212538796,47.888089358911,0
-0.77328316904779,47.888076906797,0
-0.77342205748611,47.888056258604,0
-0.77355773361605,47.888027571487,0
-0.77368916480445,47.887991063787,0
-0.77381535073218,47.887947013364,0
-0.77393533100902,47.887895755488,0
-0.77404819248371,47.887837680283,0
-0.77415307619458,47.887773229758,0
-0.77424918390732,47.887702894444,0
-0.77433578419018,47.887627209658,0
-0.77441221798038,47.887546751429,0
-0.77447790359952,47.887462132113,0
-0.77453234117961,47.887373995733,0
-0.77457511646606,47.887283013077,0
-0.77460590396913,47.88718987659,0
-0.77462446943925,47.887095295107,0
-0.774630671648,47.886999988456,0

-0.7746244634609,47.886904681981,0
-0.77460589219408,47.886810101019,0
-0.77457509925209,47.886716965383,0
-0.77453231904974,47.88662598388,0
-0.77447787722619,47.886537848923,0
-0.77441218816492,47.886453231256,0
-0.77433575183849,47.88637277485,0
-0.77424915000242,47.886297092008,0
-0.77415304176662,47.886226758698,0
-0.7740481585788,47.886162310178,0
-0.77393529865733,47.886104236917,0
-0.77381532091672,47.886052980864,0
-0.77368913843111,47.88600893209,0
-0.77355771148621,47.885972425812,0
-0.77342204027213,47.88594373985,0
-0.77328315727274,47.885923092507,0
-0.77314211940961,47.885910640913,0
-0.77300000000003,47.885906479826,0
-0.77285788059043,47.885910640913,0
-0.7727168427273,47.885923092507,0
-0.77257795972794,47.88594373985,0
-0.77244228851386,47.885972425812,0
-0.77231086156896,47.88600893209,0
-0.77218467908335,47.886052980864,0
-0.77206470134271,47.886104236917,0
-0.77195184142124,47.886162310178,0
-0.77184695823342,47.886226758698,0
-0.77175084999764,47.886297092008,0
-0.77166424816155,47.88637277485,0
-0.77158781183513,47.886453231256,0
-0.77152212277385,47.886537848923,0
-0.7714676809503,47.88662598388,0
-0.77142490074796,47.886716965383,0
-0.77139410780599,47.886810101019,0
-0.77137553653916,47.886904681981,0
-0.77136932835207,47.886999988456,0
-0.77300000000002,47.887,0
</coordinates>
</LinearRing>
</outerBoundaryIs>
</Polygon>
</Placemark>
<Placemark>
<name>158</name>
<styleUrl>#stylehap1</styleUrl>
<Polygon>
<outerBoundaryIs>
<LinearRing>
<coordinates>
-0.77300000000002,48.101,0
-0.77136255145678,48.100999988369,0
-0.77136877941861,48.101095295021,0
-0.77138742202288,48.101189876505,0
-0.771418337454888,48.101283012996,0
-0.7714612904922,48.101373995656,0
-0.7715159542929,48.101462132041,0
-0.77158191288137,48.101546751364,0

-0.77165866431255,48.1016272096,0
-0.77174562449095,48.101702894393,0
-0.771842131615,48.101773229715,0
-0.77194745121322,48.101837680247,0
-0.77206078173389,48.101895755459,0
-0.77218126064541,48.101947013342,0
-0.77230797100127,48.101991063771,0
-0.77243994841953,48.102027571477,0
-0.77257618842345,48.102056258598,0
-0.77271565408771,48.102076906794,0
-0.77285728393171,48.102089358911,0
-0.77300000000003,48.102093520174,0
-0.77314271606836,48.102089358911,0
-0.77328434591235,48.102076906794,0
-0.77342381157659,48.102056258598,0
-0.77356005158051,48.102027571477,0
-0.77369202899877,48.101991063771,0
-0.77381873935465,48.101947013342,0
-0.77393921826616,48.101895755459,0
-0.77405254878682,48.101837680247,0
-0.77415786838504,48.101773229715,0
-0.77425437550909,48.101702894393,0
-0.77434133568749,48.1016272096,0
-0.77441808711867,48.101546751364,0
-0.77448404570714,48.101462132041,0
-0.77453870950784,48.101373995656,0
-0.77458166254516,48.101283012996,0
-0.77461257797719,48.101189876505,0
-0.77463122058144,48.101095295021,0
-0.77463744854326,48.100999988369,0
-0.77463121453299,48.100904681895,0
-0.77461256606405,48.100810100935,0
-0.77458164512934,48.100716965301,0
-0.77453868711847,48.100625983804,0
-0.77448401902452,48.100537848852,0
-0.77441805695357,48.10045323119,0
-0.77434130295643,48.100372774792,0
-0.7742543412066,48.100297091957,0
-0.77415783355339,48.100226758655,0
-0.77405251448433,48.100162310142,0
-0.77393918553509,48.100104236888,0
-0.77381870918953,48.100052980843,0
-0.77369200231618,48.100008932074,0
-0.77356002919114,48.099972425802,0
-0.77342379416077,48.099943739844,0
-0.77328433399922,48.099923092504,0
-0.77314271001989,48.099910640913,0
-0.77300000000003,48.099906479827,0
-0.77285728998015,48.099910640913,0
-0.77271566600082,48.099923092504,0
-0.77257620583928,48.099943739844,0
-0.7724399708089,48.099972425802,0
-0.77230799768389,48.100008932074,0
-0.77218129081051,48.100052980843,0
-0.77206081446495,48.100104236888,0
-0.77194748551573,48.100162310142,0
-0.77184216644665,48.100226758655,0

-0.77174565879344,48.100297091957,0
-0.77165869704362,48.100372774792,0
-0.77158194304647,48.10045323119,0
-0.77151598097552,48.100537848852,0
-0.77146131288157,48.100625983804,0
-0.77141835487073,48.100716965301,0
-0.77138743393602,48.100810100935,0
-0.77136878546705,48.100904681895,0
-0.77136255145678,48.100999988369,0
-0.77300000000002,48.101,0
</coordinates>
</LinearRing>
</outerBoundaryIs>
</Polygon>
</Placemark>
<Placemark>
<name>159</name>
<styleUrl>#stylehap1</styleUrl>
<Polygon>
<outerBoundaryIs>
<LinearRing>
<coordinates>
-0.77199999999993,48.031,0
-0.77036477690052,48.030999988398,0
-0.77037099640538,48.031095295049,0
-0.77038961367982,48.031189876533,0
-0.77042048710165,48.031283013022,0
-0.77046338176793,48.031373995681,0
-0.77051797128073,48.031462132065,0
-0.77058384022964,48.031546751385,0
-0.7706604873517,48.031627209619,0
-0.77074732934509,48.03170289441,0
-0.7708437053077,48.031773229729,0
-0.77094888176623,48.031837680259,0
-0.7710620582583,48.031895755469,0
-0.7711823734247,48.031947013349,0
-0.77130891156522,48.031991063776,0
-0.77144070960872,48.032027571481,0
-0.7715767644438,48.0320562586,0
-0.77171604055459,48.032076906795,0
-0.77185747790334,48.032089358911,0
-0.77199999999995,48.032093520174,0
-0.77214252209652,48.032089358911,0
-0.77228395944528,48.032076906795,0
-0.77242323555607,48.0320562586,0
-0.77255929039115,48.032027571481,0
-0.77269108843464,48.031991063776,0
-0.77281762657517,48.031947013349,0
-0.77293794174157,48.031895755469,0
-0.77305111823364,48.031837680259,0
-0.77315629469217,48.031773229729,0
-0.77325267065477,48.03170289441,0
-0.77333951264816,48.031627209619,0
-0.77341615977023,48.031546751385,0
-0.77348202871914,48.031462132065,0
-0.77353661823193,48.031373995681,0
-0.77357951289821,48.031283013022,0

-0.77361038632005,48.031189876533,0
-0.77362900359449,48.031095295049,0
-0.77363522309935,48.030999988398,0
-0.77362899756907,48.030904681923,0
-0.7736103744523,48.030810100962,0
-0.77357949554875,48.030716965328,0
-0.7735365959279,48.030625983829,0
-0.7734820021382,48.030537848875,0
-0.77341612972004,48.030453231212,0
-0.77333948004183,48.030372774811,0
-0.77325263648297,48.030297091973,0
-0.77315625999324,48.030226758669,0
-0.77305108406186,48.030162310154,0
-0.77293790913521,48.030104236898,0
-0.77281759652499,48.03005298085,0
-0.7726910618537,48.030008932079,0
-0.77255926808709,48.029972425805,0
-0.7724232182066,48.029943739846,0
-0.77228394757756,48.029923092505,0
-0.77214251607113,48.029910640913,0
-0.77199999999995,48.029906479827,0
-0.77185748392874,48.029910640913,0
-0.7717160524223,48.029923092505,0
-0.77157678179327,48.029943739846,0
-0.77144073191278,48.029972425805,0
-0.77130893814616,48.030008932079,0
-0.77118240347488,48.03005298085,0
-0.77106209086466,48.030104236898,0
-0.77094891593801,48.030162310154,0
-0.77084374000666,48.030226758669,0
-0.7707473635169,48.030297091973,0
-0.77066051995804,48.030372774811,0
-0.77058387027983,48.030453231212,0
-0.77051799786167,48.030537848875,0
-0.77046340407197,48.030625983829,0
-0.77042050445112,48.030716965328,0
-0.77038962554757,48.030810100962,0
-0.7703710024308,48.030904681923,0
-0.77036477690052,48.030999988398,0
-0.77199999999993,48.031,0
</coordinates>
</LinearRing>
</outerBoundaryIs>
</Polygon>
</Placemark>
<Placemark>
<name>160</name>
<styleUrl>#stylehap1</styleUrl>
<Polygon>
<outerBoundaryIs>
<LinearRing>
<coordinates>
-0.77199999999993,48.101,0
-0.77036255145669,48.100999988369,0
-0.77036877941852,48.101095295021,0
-0.77038742202279,48.101189876505,0
-0.77041833745479,48.101283012996,0

-0.77046129049211,48.101373995656,0
-0.77051595429282,48.101462132041,0
-0.77058191288129,48.101546751364,0
-0.77065866431247,48.1016272096,0
-0.77074562449087,48.101702894393,0
-0.77084213161491,48.101773229715,0
-0.77094745121314,48.101837680247,0
-0.7710607817338,48.101895755459,0
-0.7711812606453,48.101947013342,0
-0.77130797100119,48.101991063771,0
-0.77143994841944,48.102027571477,0
-0.77157618842336,48.102056258598,0
-0.7717156540876,48.102076906794,0
-0.77185728393159,48.102089358911,0
-0.77199999999995,48.102093520174,0
-0.77214271606827,48.102089358911,0
-0.77228434591227,48.102076906794,0
-0.77242381157651,48.102056258598,0
-0.77256005158043,48.102027571477,0
-0.77269202899868,48.101991063771,0
-0.77281873935457,48.101947013342,0
-0.77293921826607,48.101895755459,0
-0.77305254878673,48.101837680247,0
-0.77315786838495,48.101773229715,0
-0.773254375509,48.101702894393,0
-0.7733413356874,48.1016272096,0
-0.77341808711858,48.101546751364,0
-0.77348404570705,48.101462132041,0
-0.77353870950775,48.101373995656,0
-0.77358166254508,48.101283012996,0
-0.77361257797707,48.101189876505,0
-0.77363122058135,48.101095295021,0
-0.77363744854318,48.100999988369,0
-0.7736312145329,48.100904681895,0
-0.77361256606396,48.100810100935,0
-0.77358164512925,48.100716965301,0
-0.77353868711838,48.100625983804,0
-0.77348401902444,48.100537848852,0
-0.77341805695349,48.10045323119,0
-0.77334130295634,48.100372774792,0
-0.77325434120652,48.100297091957,0
-0.7731578335533,48.100226758655,0
-0.77305251448425,48.100162310142,0
-0.77293918553501,48.100104236888,0
-0.77281870918945,48.100052980843,0
-0.77269200231609,48.100008932074,0
-0.77256002919105,48.099972425802,0
-0.77242379416068,48.099943739844,0
-0.77228433399913,48.099923092504,0
-0.7721427100198,48.099910640913,0
-0.77199999999995,48.099906479827,0
-0.77185728998007,48.099910640913,0
-0.77171566600074,48.099923092504,0
-0.77157620583919,48.099943739844,0
-0.77143997080882,48.099972425802,0
-0.77130799768378,48.100008932074,0
-0.77118129081042,48.100052980843,0

-0.77106081446486,48.100104236888,0
-0.77094748551562,48.100162310142,0
-0.77084216644657,48.100226758655,0
-0.77074565879335,48.100297091957,0
-0.77065869704353,48.100372774792,0
-0.77058194304638,48.10045323119,0
-0.77051598097543,48.100537848852,0
-0.77046131288149,48.100625983804,0
-0.77041835487064,48.100716965301,0
-0.77038743393591,48.100810100935,0
-0.77036878546697,48.100904681895,0
-0.77036255145669,48.100999988369,0
-0.77199999999993,48.101,0
</coordinates>
</LinearRing>
</outerBoundaryIs>
</Polygon>
</Placemark>
<Placemark>
<name>162</name>
<styleUrl>#stylehap1</styleUrl>
<Polygon>
<outerBoundaryIs>
<LinearRing>
<coordinates>
-0.76999999999998,48.214,0
-0.76835894096606,48.213999988323,0
-0.76836518264819,48.214095294975,0
-0.76838386634676,48.214189876461,0
-0.76841484993476,48.214283012952,0
-0.76845789767142,48.214373995615,0
-0.76851268199448,48.214462132003,0
-0.76857878601132,48.214546751329,0
-0.7686557066704,48.214627209569,0
-0.76874285858841,48.214702894366,0
-0.7688395785047,48.214773229691,0
-0.76894513032835,48.214837680228,0
-0.76905871074018,48.214895755444,0
-0.76917945530664,48.214947013331,0
-0.76930644505935,48.214991063763,0
-0.76943871348965,48.215027571472,0
-0.76957525390577,48.215056258595,0
-0.76971502709565,48.215076906793,0
-0.76985696923793,48.21508935891,0
-0.76999999999998,48.215093520174,0
-0.77014303076203,48.21508935891,0
-0.77028497290431,48.215076906793,0
-0.77042474609421,48.215056258595,0
-0.7705612865103,48.215027571472,0
-0.77069355494061,48.214991063763,0
-0.77082054469331,48.214947013331,0
-0.7709412892598,48.214895755444,0
-0.77105486967161,48.214837680228,0
-0.77116042149526,48.214773229691,0
-0.77125714141154,48.214702894366,0
-0.77134429332958,48.214627209569,0
-0.77142121398863,48.214546751329,0

-0.7714873180055,48.214462132003,0
-0.77154210232854,48.214373995615,0
-0.7715851500652,48.214283012952,0
-0.7716161336532,48.214189876461,0
-0.77163481735179,48.214095294975,0
-0.7716410590339,48.213999988323,0
-0.77163481126589,48.213904681849,0
-0.77161612166632,48.21381010089,0
-0.77158513254156,48.213716965258,0
-0.77154207980059,48.213625983763,0
-0.77148729115773,48.213537848814,0
-0.7714211836368,48.213453231156,0
-0.7713442603959,48.213372774761,0
-0.7712571068967,48.213297091929,0
-0.77116038644799,48.213226758632,0
-0.77105483515679,48.213162310123,0
-0.77094125632613,48.213104236873,0
-0.77082051434148,48.213052980831,0
-0.77069352809283,48.213008932066,0
-0.77056126398236,48.212972425796,0
-0.77042472857058,48.212943739841,0
-0.77028496091743,48.212923092503,0
-0.77014302467613,48.212910640912,0
-0.76999999999998,48.212906479827,0
-0.76985697532383,48.212910640912,0
-0.76971503908252,48.212923092503,0
-0.7695752714294,48.212943739841,0
-0.76943873601762,48.212972425796,0
-0.76930647190712,48.213008932066,0
-0.7691794856585,48.213052980831,0
-0.76905874367383,48.213104236873,0
-0.76894516484319,48.213162310123,0
-0.76883961355199,48.213226758632,0
-0.76874289310326,48.213297091929,0
-0.76865573960405,48.213372774761,0
-0.76857881636315,48.213453231156,0
-0.76851270884225,48.213537848814,0
-0.76845792019939,48.213625983763,0
-0.76841486745839,48.213716965258,0
-0.76838387833363,48.21381010089,0
-0.76836518873407,48.213904681849,0
-0.76835894096606,48.213999988323,0
-0.76999999999998,48.214,0
</coordinates>
</LinearRing>
</outerBoundaryIs>
</Polygon>
</Placemark>
<Placemark>
<name>163</name>
<styleUrl>#stylehap1</styleUrl>
<Polygon>
<outerBoundaryIs>
<LinearRing>
<coordinates>
-0.76900000000001,48.034,0
-0.76736468169855,48.033999988397,0

-0.76737090156521,48.034095295048,0
-0.76738951992322,48.034189876532,0
-0.7674203951422,48.034283013021,0
-0.76746329230552,48.03437399568,0
-0.76751788499629,48.034462132064,0
-0.76758375777986,48.034546751384,0
-0.76766040936414,48.034627209618,0
-0.76774725641339,48.034702894409,0
-0.76784363798693,48.034773229728,0
-0.76794882056879,48.034837680258,0
-0.76806200365005,48.034895755468,0
-0.7681823258213,48.034947013349,0
-0.76830887132899,48.034991063776,0
-0.76844067704594,48.03502757148,0
-0.76857673980233,48.0350562586,0
-0.76871602402201,48.035076906795,0
-0.7688574696055,48.035089358911,0
-0.76899999999999,48.035093520174,0
-0.76914253039451,48.035089358911,0
-0.769283975978,48.035076906795,0
-0.76942326019768,48.0350562586,0
-0.76955932295407,48.03502757148,0
-0.76969112867102,48.034991063776,0
-0.76981767417871,48.034947013349,0
-0.76993799634996,48.034895755468,0
-0.77005117943122,48.034837680258,0
-0.77015636201308,48.034773229728,0
-0.77025274358662,48.034702894409,0
-0.77033959063587,48.034627209618,0
-0.77041624222015,48.034546751384,0
-0.77048211500372,48.034462132064,0
-0.7705367076945,48.03437399568,0
-0.77057960485782,48.034283013021,0
-0.77061048007679,48.034189876532,0
-0.77062909843481,48.034095295048,0
-0.77063531830146,48.033999988397,0
-0.77062909240842,48.033904681922,0
-0.77061046820711,48.033810100961,0
-0.7705795875055,48.033716965327,0
-0.7705366853868,48.033625983828,0
-0.77048208841845,48.033537848874,0
-0.77041621216505,48.033453231211,0
-0.77033955802419,48.03337277481,0
-0.77025270940924,48.033297091973,0
-0.77015632730847,48.033226758668,0
-0.77005114525382,48.033162310154,0
-0.76993796373828,48.033104236897,0
-0.76981764412364,48.033052980849,0
-0.76969110208573,48.033008932079,0
-0.76955930064635,48.032972425805,0
-0.76942324284536,48.032943739846,0
-0.76928396410833,48.032923092505,0
-0.7691425243681,48.032910640913,0
-0.76899999999999,48.032906479827,0
-0.76885747563192,48.032910640913,0
-0.76871603589168,48.032923092505,0
-0.76857675715465,48.032943739846,0

-0.76844069935366,48.032972425805,0
-0.76830889791428,48.033008932079,0
-0.76818235587638,48.033052980849,0
-0.76806203626173,48.033104236897,0
-0.76794885474619,48.033162310154,0
-0.76784367269154,48.033226758668,0
-0.76774729059077,48.033297091973,0
-0.76766044197582,48.03337277481,0
-0.76758378783496,48.033453231211,0
-0.76751791158156,48.033537848874,0
-0.76746331461321,48.033625983828,0
-0.76742041249451,48.033716965327,0
-0.7673895317929,48.033810100961,0
-0.76737090759159,48.033904681922,0
-0.76736468169855,48.033999988397,0
-0.76900000000001,48.034,0
</coordinates>
</LinearRing>
</outerBoundaryIs>
</Polygon>
</Placemark>
<Placemark>
<name>165</name>
<styleUrl>#stylehap1</styleUrl>
<Polygon>
<outerBoundaryIs>
<LinearRing>
<coordinates>
-0.76700000000005,48.102,0
-0.76536251960316,48.101999988369,0
-0.76536874768602,48.10209529502,0
-0.76538739065286,48.102189876505,0
-0.76541830668618,48.102283012995,0
-0.76546126055897,48.102373995656,0
-0.765515925423,48.102462132041,0
-0.76558188529451,48.102546751363,0
-0.76565863821871,48.1026272096,0
-0.76574560008873,48.102702894393,0
-0.76584210909015,48.102773229714,0
-0.76594743073718,48.102837680247,0
-0.76606076346253,48.102895755459,0
-0.76618124471777,48.102947013342,0
-0.76630795753864,48.102991063771,0
-0.76643993752436,48.103027571477,0
-0.76657618017868,48.103056258598,0
-0.76671564855608,48.103076906794,0
-0.76685728115533,48.103089358911,0
-0.76700000000005,48.103093520174,0
-0.76714271884477,48.103089358911,0
-0.76728435144404,48.103076906794,0
-0.76742381982142,48.103056258598,0
-0.76756006247574,48.103027571477,0
-0.76769204246146,48.102991063771,0
-0.76781875528233,48.102947013342,0
-0.76793923653759,48.102895755459,0
-0.76805256926291,48.102837680247,0
-0.76815789090997,48.102773229714,0

-0.76825439991139,48.102702894393,0
-0.76834136178141,48.1026272096,0
-0.76841811470559,48.102546751363,0
-0.7684840745771,48.102462132041,0
-0.76853873944113,48.102373995656,0
-0.76858169331394,48.102283012995,0
-0.76861260934727,48.102189876505,0
-0.76863125231407,48.10209529502,0
-0.76863748039697,48.101999988369,0
-0.7686312462653,48.101904681894,0
-0.76861259743347,48.101810100934,0
-0.76858167589715,48.101716965301,0
-0.76853871705056,48.101625983803,0
-0.76848404789306,48.101537848852,0
-0.76841808453885,48.10145323119,0
-0.76834132904857,48.101372774791,0
-0.76825436560702,48.101297091956,0
-0.76815785607641,48.101226758655,0
-0.76805253495854,48.101162310142,0
-0.76793920380475,48.101104236888,0
-0.76781872511558,48.101052980843,0
-0.76769201577742,48.101008932074,0
-0.76756004008517,48.100972425802,0
-0.76742380240465,48.100943739844,0
-0.76728433953024,48.100923092504,0
-0.76714271279599,48.100910640913,0
-0.76700000000005,48.100906479826,0
-0.76685728720411,48.100910640913,0
-0.76671566046985,48.100923092504,0
-0.76657619759545,48.100943739844,0
-0.76643995991493,48.100972425802,0
-0.7663079842227,48.101008932074,0
-0.76618127488454,48.101052980843,0
-0.76606079619537,48.101104236888,0
-0.76594746504155,48.101162310142,0
-0.76584214392371,48.101226758655,0
-0.7657456343931,48.101297091956,0
-0.76565867095156,48.101372774791,0
-0.76558191546125,48.10145323119,0
-0.76551595210706,48.101537848852,0
-0.76546128294957,48.101625983803,0
-0.76541832410295,48.101716965301,0
-0.76538740256663,48.101810100934,0
-0.7653687537348,48.101904681894,0
-0.76536251960316,48.101999988369,0
-0.76700000000005,48.102,0
</coordinates>
</LinearRing>
</outerBoundaryIs>
</Polygon>
</Placemark>
<Placemark>
<name>167</name>
<styleUrl>#stylehap1</styleUrl>
<Polygon>
<outerBoundaryIs>
<LinearRing>

<coordinates>
-0.76599999999996,48.181,0
-0.76435999766165,48.180999988337,0
-0.76436623532819,48.181095294988,0
-0.76438490699955,48.181189876474,0
-0.76441587064008,48.181283012965,0
-0.76445889066075,48.181373995627,0
-0.76451363971009,48.181462132015,0
-0.76457970116388,48.181546751339,0
-0.7646565722944,48.181627209578,0
-0.76474366809529,48.181702894374,0
-0.76484032573288,48.181773229698,0
-0.76494580959026,48.181837680233,0
-0.76505931686552,48.181895755448,0
-0.76517998368177,48.181947013334,0
-0.76530689166236,48.181991063765,0
-0.7654390749211,48.182027571473,0
-0.76557552741447,48.182056258596,0
-0.7657152105997,48.182076906793,0
-0.76585706134049,48.18208935891,0
-0.76599999999996,48.182093520173,0
-0.76614293865943,48.18208935891,0
-0.76628478940023,48.182076906793,0
-0.76642447258545,48.182056258596,0
-0.76656092507882,48.182027571473,0
-0.76669310833756,48.181991063765,0
-0.76682001631815,48.181947013334,0
-0.76694068313441,48.181895755448,0
-0.76705419040966,48.181837680233,0
-0.76715967426705,48.181773229698,0
-0.76725633190464,48.181702894374,0
-0.76734342770553,48.181627209578,0
-0.76742029883604,48.181546751339,0
-0.76748636028984,48.181462132015,0
-0.76754110933917,48.181373995627,0
-0.76758412935984,48.181283012965,0
-0.76761509300037,48.181189876474,0
-0.76763376467174,48.181095294988,0
-0.76764000233828,48.180999988337,0
-0.7676337585968,48.180904681862,0
-0.7676150810351,48.180810100903,0
-0.76758411186776,48.180716965271,0
-0.76754108685181,48.180625983775,0
-0.76748633349043,48.180537848825,0
-0.76742026853889,48.180453231166,0
-0.76734339483119,48.18037277477,0
-0.76725629745201,48.180297091937,0
-0.76715963928291,48.180226758638,0
-0.76705415595703,48.180162310129,0
-0.76694065026007,48.180104236877,0
-0.766819986021,48.180052980834,0
-0.76669308153816,48.180008932068,0
-0.76656090259146,48.179972425798,0
-0.76642445509339,48.179943739842,0
-0.76628477743496,48.179923092503,0
-0.7661429325845,48.179910640912,0
-0.76599999999996,48.179906479826,0

-0.76585706741543,48.179910640912,0
-0.76571522256499,48.179923092503,0
-0.76557554490653,48.179943739842,0
-0.76543909740849,48.179972425798,0
-0.76530691846177,48.180008932068,0
-0.76518001397892,48.180052980834,0
-0.76505934973986,48.180104236877,0
-0.76494584404289,48.180162310129,0
-0.76484036071702,48.180226758638,0
-0.76474370254794,48.180297091937,0
-0.76465660516874,48.18037277477,0
-0.76457973146103,48.180453231166,0
-0.76451366650949,48.180537848825,0
-0.76445891314811,48.180625983775,0
-0.76441588813217,48.180716965271,0
-0.76438491896482,48.180810100903,0
-0.76436624140312,48.180904681862,0
-0.76435999766165,48.180999988337,0
-0.76599999999996,48.181,0
</coordinates>
</LinearRing>
</outerBoundaryIs>
</Polygon>
</Placemark>
<Placemark>
<name>168</name>
<styleUrl>#stylehap1</styleUrl>
<Polygon>
<outerBoundaryIs>
<LinearRing>
<coordinates>
-0.76499999999999,48.179,0
-0.76336006164259,48.178999988337,0
-0.76336629906601,48.179095294989,0
-0.76338497000915,48.179189876474,0
-0.76341593244191,48.179283012966,0
-0.76345895078442,48.179373995628,0
-0.763513697698,48.179462132015,0
-0.76357975657468,48.17954675134,0
-0.76365662470634,48.179627209578,0
-0.76374371710943,48.179702894375,0
-0.76384037097618,48.179773229699,0
-0.76394585071831,48.179837680234,0
-0.76405935356529,48.179895755449,0
-0.76418001567392,48.179947013334,0
-0.76430691870338,48.179991063765,0
-0.76443909680514,48.180027571473,0
-0.76457554397493,48.180056258596,0
-0.76471522171056,48.180076906793,0
-0.76485706691716,48.180089358911,0
-0.76499999999998,48.180093520174,0
-0.76514293308282,48.180089358911,0
-0.76528477828942,48.180076906793,0
-0.76542445602503,48.180056258596,0
-0.76556090319484,48.180027571473,0
-0.7656930812966,48.179991063765,0
-0.76581998432604,48.179947013334,0

-0.76594064643469,48.179895755449,0
-0.76605414928165,48.179837680234,0
-0.7661596290238,48.179773229699,0
-0.76625628289055,48.179702894375,0
-0.76634337529364,48.179627209578,0
-0.7664202434253,48.17954675134,0
-0.76648630230198,48.179462132015,0
-0.76654104921556,48.179373995628,0
-0.76658406755807,48.179283012966,0
-0.76661502999083,48.179189876474,0
-0.76663370093395,48.179095294989,0
-0.76663993835736,48.178999988337,0
-0.7666336948597,48.178904681863,0
-0.76661501802685,48.178810100904,0
-0.76658405006789,48.178716965272,0
-0.76654102673064,48.178625983775,0
-0.7664862755055,48.178537848826,0
-0.76642021313146,48.178453231166,0
-0.76634334242289,48.17837277477,0
-0.76625624844166,48.178297091938,0
-0.76615959404348,48.178226758639,0
-0.76605411483278,48.178162310129,0
-0.76594061356394,48.178104236878,0
-0.7658199540322,48.178052980835,0
-0.76569305450012,48.178008932069,0
-0.76556088070992,48.177972425798,0
-0.76542443853488,48.177943739842,0
-0.76528476632545,48.177923092503,0
-0.76514292700854,48.177910640912,0
-0.76499999999998,48.177906479827,0
-0.76485707299141,48.177910640912,0
-0.76471523367453,48.177923092503,0
-0.7645755614651,48.177943739842,0
-0.76443911929004,48.177972425798,0
-0.76430694549986,48.178008932069,0
-0.76418004596776,48.178052980835,0
-0.76405938643604,48.178104236878,0
-0.7639458851672,48.178162310129,0
-0.76384040595648,48.178226758639,0
-0.76374375155832,48.178297091938,0
-0.76365665757707,48.17837277477,0
-0.76357978686852,48.178453231166,0
-0.76351372449448,48.178537848826,0
-0.76345897326934,48.178625983775,0
-0.76341594993206,48.178716965272,0
-0.76338498197313,48.178810100904,0
-0.76336630514028,48.178904681863,0
-0.76336006164259,48.178999988337,0
-0.76499999999999,48.179,0
</coordinates>
</LinearRing>
</outerBoundaryIs>
</Polygon>
</Placemark>
<Placemark>
<name>170</name>
<styleUrl>#stylehap1</styleUrl>

<Polygon>
<outerBoundaryIs>
<LinearRing>
<coordinates>
-0.76300000000003,47.816,0
-0.76137155937813,47.815999988485,0
-0.76137775310866,47.816095295135,0
-0.76139629318539,47.816189876618,0
-0.76142703857285,47.816283013103,0
-0.76146975534183,47.816373995758,0
-0.76152411844762,47.816462132137,0
-0.76158971420236,47.81654675145,0
-0.76166604342177,47.816627209677,0
-0.76175252522317,47.816702894461,0
-0.76184850144547,47.816773229772,0
-0.76195324165759,47.816837680295,0
-0.76206594871735,47.816895755497,0
-0.76218576483833,47.816947013371,0
-0.76231177811879,47.816991063792,0
-0.76244302948249,47.817027571491,0
-0.76257851997916,47.817056258606,0
-0.76271721838857,47.817076906798,0
-0.76285806907036,47.817089358912,0
-0.76300000000003,47.817093520174,0
-0.76314193092971,47.817089358912,0
-0.7632827816115,47.817076906798,0
-0.76342148002091,47.817056258606,0
-0.76355697051758,47.817027571491,0
-0.76368822188128,47.816991063792,0
-0.76381423516174,47.816947013371,0
-0.76393405128272,47.816895755497,0
-0.76404675834247,47.816837680295,0
-0.7641514985546,47.816773229772,0
-0.7642474747769,47.816702894461,0
-0.76433395657829,47.816627209677,0
-0.76441028579771,47.81654675145,0
-0.76447588155245,47.816462132137,0
-0.76453024465824,47.816373995758,0
-0.76457296142722,47.816283013103,0
-0.76460370681468,47.816189876618,0
-0.76462224689141,47.816095295135,0
-0.76462844062194,47.815999988485,0
-0.76462224093609,47.81590468201,0
-0.764603695085,47.815810101047,0
-0.76457294427955,47.815716965409,0
-0.76453022261364,47.815625983906,0
-0.76447585528071,47.815537848947,0
-0.7644102560971,47.815453231277,0
-0.76433392435126,47.815372774869,0
-0.76424744100261,47.815297092024,0
-0.76415146425929,47.815226758713,0
-0.76404672456819,47.81516231019,0
-0.76393401905568,47.815104236926,0
-0.76381420546111,47.815052980872,0
-0.76368819560954,47.815008932095,0
-0.76355694847298,47.814972425815,0
-0.76342146287324,47.814943739852,0

-0.76328276988181,47.814923092508,0
-0.76314192497439,47.814910640913,0
-0.76300000000003,47.814906479827,0
-0.76285807502568,47.814910640913,0
-0.76271723011825,47.814923092508,0
-0.76257853712683,47.814943739852,0
-0.76244305152709,47.814972425815,0
-0.76231180439053,47.815008932095,0
-0.76218579453896,47.815052980872,0
-0.76206598094439,47.815104236926,0
-0.76195327543188,47.81516231019,0
-0.76184853574078,47.815226758713,0
-0.76175255899746,47.815297092024,0
-0.76166607564881,47.815372774869,0
-0.76158974390296,47.815453231277,0
-0.76152414471936,47.815537848947,0
-0.76146977738643,47.815625983906,0
-0.76142705572052,47.815716965409,0
-0.76139630491507,47.815810101047,0
-0.76137775906398,47.81590468201,0
-0.76137155937813,47.815999988485,0
-0.76300000000003,47.816,0
</coordinates>
</LinearRing>
</outerBoundaryIs>
</Polygon>
</Placemark>
<Placemark>
<name>171</name>
<styleUrl>#stylehap1</styleUrl>
<Polygon>
<outerBoundaryIs>
<LinearRing>
<coordinates>
-0.76300000000003,48.163,0
-0.76136057323863,48.162999988344,0
-0.76136680871792,48.163095294995,0
-0.7613854738381,48.163189876481,0
-0.76141642661338,48.163283012972,0
-0.76145943153725,48.163373995634,0
-0.76151416137316,48.163462132021,0
-0.76158019964305,48.163546751345,0
-0.76165704379562,48.163627209583,0
-0.76174410902977,48.163702894378,0
-0.76184073274448,48.163773229702,0
-0.76194617958098,48.163837680236,0
-0.7620596470191,48.163895755451,0
-0.7621802714852,48.163947013336,0
-0.76230713492492,48.163991063767,0
-0.76243927179112,48.164027571474,0
-0.76257567639341,48.164056258596,0
-0.76271531055353,48.164076906793,0
-0.76285711150837,48.164089358911,0
-0.76300000000003,48.164093520174,0
-0.7631428884917,48.164089358911,0
-0.76328468944653,48.164076906793,0
-0.76342432360666,48.164056258596,0

-0.76356072820894,48.164027571474,0
-0.76369286507515,48.163991063767,0
-0.76381972851487,48.163947013336,0
-0.76394035298097,48.163895755451,0
-0.76405382041909,48.163837680236,0
-0.76415926725558,48.163773229702,0
-0.76425589097029,48.163702894378,0
-0.76434295620445,48.163627209583,0
-0.76441980035702,48.163546751345,0
-0.76448583862691,48.163462132021,0
-0.76454056846282,48.163373995634,0
-0.76458357338669,48.163283012972,0
-0.76461452616196,48.163189876481,0
-0.76463319128215,48.163095294995,0
-0.76463942676143,48.162999988344,0
-0.76463318521319,48.16290468187,0
-0.76461451420845,48.16281010091,0
-0.76458355591183,48.162716965278,0
-0.76454054599754,48.162625983781,0
-0.76448581185384,48.162537848831,0
-0.76441977008964,48.162453231171,0
-0.7643429233624,48.162372774775,0
-0.7642558565515,48.162297091942,0
-0.76415923230582,48.162226758642,0
-0.7640537860003,48.162162310132,0
-0.76394032013895,48.16210423688,0
-0.76381969824749,48.162052980836,0
-0.76369283830208,48.16200893207,0
-0.76356070574367,48.161972425799,0
-0.76342430613178,48.161943739842,0
-0.76328467749302,48.161923092504,0
-0.76314288242272,48.161910640912,0
-0.76300000000003,48.161906479826,0
-0.76285711757735,48.161910640912,0
-0.76271532250705,48.161923092504,0
-0.76257569386829,48.161943739842,0
-0.7624392942564,48.161972425799,0
-0.76230716169799,48.16200893207,0
-0.76218030175258,48.162052980836,0
-0.76205967986112,48.16210423688,0
-0.76194621399977,48.162162310132,0
-0.76184076769425,48.162226758642,0
-0.76174414344857,48.162297091942,0
-0.76165707663767,48.162372774775,0
-0.76158022991043,48.162453231171,0
-0.76151418814623,48.162537848831,0
-0.76145945400252,48.162625983781,0
-0.76141644408823,48.162716965278,0
-0.76138548579162,48.16281010091,0
-0.76136681478688,48.16290468187,0
-0.76136057323863,48.162999988344,0
-0.76300000000003,48.163,0
</coordinates>
</LinearRing>
</outerBoundaryIs>
</Polygon>
</Placemark>

<Placemark>
<name>172</name>
<styleUrl>#stylehap1</styleUrl>
<Polygon>
<outerBoundaryIs>
<LinearRing>
<coordinates>
-0.76099999999997,48.026,0
-0.75936493553605,48.0259999884,0
-0.75937115443808,48.026095295051,0
-0.75938976990693,48.026189876535,0
-0.75942064033415,48.026283013024,0
-0.75946353083958,48.026373995683,0
-0.75951811505698,48.026462132067,0
-0.75958397761613,48.026546751387,0
-0.75966061730275,48.02662720962,0
-0.75974745087161,48.026702894411,0
-0.75984381748468,48.02677322973,0
-0.75994898373984,48.026837680259,0
-0.76006214925236,48.026895755469,0
-0.76018245274658,48.02694701335,0
-0.76030897861113,48.026991063777,0
-0.76044076386833,48.027027571481,0
-0.76057680550406,48.0270562586,0
-0.76071606810299,48.027076906795,0
-0.7608574917302,48.027089358911,0
-0.76099999999996,48.027093520174,0
-0.76114250826975,48.027089358911,0
-0.76128393189694,48.027076906795,0
-0.76142319449586,48.0270562586,0
-0.76155923613162,48.027027571481,0
-0.7616910213888,48.026991063777,0
-0.76181754725338,48.02694701335,0
-0.76193785074757,48.026895755469,0
-0.76205101626011,48.026837680259,0
-0.76215618251524,48.02677322973,0
-0.76225254912832,48.026702894411,0
-0.76233938269718,48.02662720962,0
-0.76241602238379,48.026546751387,0
-0.76248188494297,48.026462132067,0
-0.76253646916034,48.026373995683,0
-0.76257935966577,48.026283013024,0
-0.762610230093,48.026189876535,0
-0.76262884556185,48.026095295051,0
-0.76263506446391,48.0259999884,0
-0.76262883953808,48.025904681925,0
-0.76261021822849,48.025810100964,0
-0.76257934232101,48.02571696533,0
-0.76253644686237,48.025625983831,0
-0.76248185836929,48.025537848877,0
-0.76241599234182,48.025453231213,0
-0.76233935009972,48.025372774812,0
-0.76225251496582,48.025297091974,0
-0.76215614782575,48.02522675867,0
-0.76205098209762,48.025162310155,0
-0.76193781815011,48.025104236898,0
-0.76181751721138,48.02505298085,0

-0.76169099481511,48.02500893208,0
-0.76155921383364,48.024972425805,0
-0.7614231771511,48.024943739846,0
-0.76128392003245,48.024923092505,0
-0.76114250224599,48.024910640913,0
-0.76099999999996,48.024906479826,0
-0.76085749775397,48.024910640913,0
-0.7607160799675,48.024923092505,0
-0.76057682284882,48.024943739846,0
-0.76044078616631,48.024972425805,0
-0.76030900518482,48.02500893208,0
-0.76018248278854,48.02505298085,0
-0.76006218184981,48.025104236898,0
-0.7599490179023,48.025162310155,0
-0.75984385217418,48.02522675867,0
-0.7597474850341,48.025297091974,0
-0.7596606499002,48.025372774812,0
-0.75958400765813,48.025453231213,0
-0.75951814163067,48.025537848877,0
-0.75946355313756,48.025625983831,0
-0.75942065767891,48.02571696533,0
-0.75938978177144,48.025810100964,0
-0.75937116046184,48.025904681925,0
-0.75936493553605,48.0259999884,0
-0.76099999999997,48.026,0
</coordinates>
</LinearRing>
</outerBoundaryIs>
</Polygon>
</Placemark>
<Placemark>
<name>176</name>
<styleUrl>#stylehap1</styleUrl>
<Polygon>
<outerBoundaryIs>
<LinearRing>
<coordinates>
-0.75599999999997,48.184,0
-0.75435990167711,48.183999988335,0
-0.75436613970842,48.184095294987,0
-0.75438481247227,48.184189876472,0
-0.75441577792473,48.184283012964,0
-0.75445880046297,48.184373995626,0
-0.75451355271637,48.184462132014,0
-0.75457961803637,48.184546751338,0
-0.75465649366579,48.184627209577,0
-0.75474359456407,48.184702894373,0
-0.75484025785872,48.184773229698,0
-0.75494574788979,48.184837680233,0
-0.75505926180836,48.184895755448,0
-0.75517993568705,48.184947013334,0
-0.75530685109538,48.184991063765,0
-0.75543904209063,48.185027571473,0
-0.75557550257042,48.185056258596,0
-0.7557151939312,48.185076906793,0
-0.75585705297439,48.18508935891,0
-0.75599999999996,48.185093520174,0

-0.75614294702554,48.18508935891,0
-0.75628480606875,48.185076906793,0
-0.75642449742953,48.185056258596,0
-0.75656095790932,48.185027571473,0
-0.75669314890457,48.184991063765,0
-0.7568200643129,48.184947013334,0
-0.75694073819157,48.184895755448,0
-0.75705425211016,48.184837680233,0
-0.75715974214123,48.184773229698,0
-0.75725640543588,48.184702894373,0
-0.75734350633416,48.184627209577,0
-0.75742038196358,48.184546751338,0
-0.75748644728355,48.184462132014,0
-0.75754119953698,48.184373995626,0
-0.7575842220752,48.184283012964,0
-0.75761518752768,48.184189876472,0
-0.75763386029153,48.184095294987,0
-0.75764009832281,48.183999988335,0
-0.7576338542156,48.183904681861,0
-0.75761517556042,48.183810100902,0
-0.75758420458027,48.18371696527,0
-0.75754117704591,48.183625983774,0
-0.75748642047977,48.183537848824,0
-0.75742035166147,48.183453231165,0
-0.75734347345442,48.183372774769,0
-0.75725637097758,48.183297091937,0
-0.75715970715136,48.183226758638,0
-0.75705421765188,48.183162310128,0
-0.75694070531186,48.183104236877,0
-0.75682003401079,48.183052980834,0
-0.75669312210076,48.183008932068,0
-0.75656093541825,48.182972425798,0
-0.75642447993459,48.182943739841,0
-0.75628479410152,48.182923092503,0
-0.75614294094961,48.182910640912,0
-0.75599999999996,48.182906479826,0
-0.75585705905031,48.182910640912,0
-0.75571520589843,48.182923092503,0
-0.75557552006536,48.182943739841,0
-0.75543906458168,48.182972425798,0
-0.75530687789916,48.183008932068,0
-0.75517996598916,48.183052980834,0
-0.75505929468809,48.183104236877,0
-0.75494578234807,48.183162310128,0
-0.75484029284859,48.183226758638,0
-0.75474362902235,48.183297091937,0
-0.75465652654553,48.183372774769,0
-0.75457964833848,48.183453231165,0
-0.75451357952018,48.183537848824,0
-0.75445882295402,48.183625983774,0
-0.75441579541966,48.18371696527,0
-0.7543848244395,48.183810100902,0
-0.75436614578435,48.183904681861,0
-0.75435990167711,48.183999988335,0
-0.75599999999997,48.184,0
</coordinates>
</LinearRing>

</outerBoundaryIs>
</Polygon>
</Placemark>
<Placemark>
<name>177</name>
<styleUrl>#stylehap1</styleUrl>
<Polygon>
<outerBoundaryIs>
<LinearRing>
<coordinates>
-0.75299999999993,48.259,0
-0.75135749694226,48.258999988305,0
-0.75136374411183,48.259095294956,0
-0.75138244424616,48.259189876443,0
-0.7514134550933,48.259283012935,0
-0.75145654070517,48.259373995599,0
-0.75151137323137,48.259462131988,0
-0.75157753541262,48.259546751315,0
-0.75165452375477,48.259627209556,0
-0.75174175235949,48.259702894355,0
-0.75183855738251,48.259773229682,0
-0.7519442020852,48.25983768022,0
-0.75205788244156,48.259895755438,0
-0.75217873325733,48.259947013326,0
-0.75230583475541,48.25999106376,0
-0.75243821957664,48.26002757147,0
-0.75257488014327,48.260056258594,0
-0.75271477632874,48.260076906792,0
-0.75285684337542,48.26008935891,0
-0.75299999999993,48.260093520173,0
-0.75314315662445,48.26008935891,0
-0.7532852236711,48.260076906792,0
-0.75342511985659,48.260056258594,0
-0.75356178042322,48.26002757147,0
-0.75369416524443,48.25999106376,0
-0.75382126674251,48.259947013326,0
-0.75394211755831,48.259895755438,0
-0.75405579791464,48.25983768022,0
-0.75416144261736,48.259773229682,0
-0.75425824764038,48.259702894355,0
-0.7543454762451,48.259627209556,0
-0.75442246458722,48.259546751315,0
-0.75448862676847,48.259462131988,0
-0.75454345929469,48.259373995599,0
-0.75458654490657,48.259283012935,0
-0.7546175557537,48.259189876443,0
-0.75463625588803,48.259095294956,0
-0.75464250305761,48.258999988305,0
-0.75463624978714,48.258904681831,0
-0.75461754373729,48.258810100872,0
-0.75458652733975,48.258716965241,0
-0.75454343671125,48.258625983746,0
-0.75448859985456,48.258537848799,0
-0.75442243416063,48.258453231142,0
-0.7543454432303,48.258372774748,0
-0.75425821304052,48.258297091919,0
-0.75416140748375,48.258226758622,0

-0.75405576331479,48.258162310116,0
-0.75394208454351,48.258104236867,0
-0.75382123631592,48.258052980826,0
-0.75369413833052,48.258008932063,0
-0.75356175783976,48.257972425794,0
-0.75342510228978,48.257943739839,0
-0.75328521165471,48.257923092502,0
-0.75314315052356,48.257910640912,0
-0.75299999999993,48.257906479826,0
-0.75285684947631,48.257910640912,0
-0.75271478834516,48.257923092502,0
-0.75257489771009,48.257943739839,0
-0.75243824216011,48.257972425794,0
-0.75230586166934,48.258008932063,0
-0.75217876368394,48.258052980826,0
-0.75205791545635,48.258104236867,0
-0.75194423668505,48.258162310116,0
-0.75183859251611,48.258226758622,0
-0.75174178695935,48.258297091919,0
-0.75165455676956,48.258372774748,0
-0.75157756583923,48.258453231142,0
-0.7515114001453,48.258537848799,0
-0.75145656328861,48.258625983746,0
-0.75141347266009,48.258716965241,0
-0.75138245626255,48.258810100872,0
-0.75136375021272,48.258904681831,0
-0.75135749694226,48.258999988305,0
-0.75299999999993,48.259,0
</coordinates>
</LinearRing>
</outerBoundaryIs>
</Polygon>
</Placemark>
<Placemark>
<name>178</name>
<styleUrl>#stylehap1</styleUrl>
<Polygon>
<outerBoundaryIs>
<LinearRing>
<coordinates>
-0.75199999999995,48.179,0
-0.75036006164256,48.178999988337,0
-0.75036629906598,48.179095294989,0
-0.75038497000912,48.179189876474,0
-0.75041593244188,48.179283012966,0
-0.75045895078439,48.179373995628,0
-0.75051369769797,48.179462132015,0
-0.75057975657463,48.17954675134,0
-0.75065662470631,48.179627209578,0
-0.7507437171094,48.179702894375,0
-0.75084037097615,48.179773229699,0
-0.75094585071828,48.179837680234,0
-0.75105935356526,48.179895755449,0
-0.75118001567389,48.179947013334,0
-0.75130691870335,48.179991063765,0
-0.75143909680509,48.180027571473,0
-0.7515755439749,48.180056258596,0

-0.75171522171053,48.180076906793,0
-0.75185706691711,48.180089358911,0
-0.75199999999995,48.180093520174,0
-0.75214293308279,48.180089358911,0
-0.75228477828939,48.180076906793,0
-0.752424456025,48.180056258596,0
-0.75256090319481,48.180027571473,0
-0.75269308129657,48.179991063765,0
-0.75281998432601,48.179947013334,0
-0.75294064643464,48.179895755449,0
-0.75305414928162,48.179837680234,0
-0.75315962902377,48.179773229699,0
-0.7532562828905,48.179702894375,0
-0.75334337529361,48.179627209578,0
-0.75342024342527,48.17954675134,0
-0.75348630230195,48.179462132015,0
-0.75354104921551,48.179373995628,0
-0.75358406755804,48.179283012966,0
-0.7536150299908,48.179189876474,0
-0.75363370093392,48.179095294989,0
-0.75363993835733,48.178999988337,0
-0.75363369485964,48.178904681863,0
-0.75361501802682,48.178810100904,0
-0.75358405006786,48.178716965272,0
-0.75354102673061,48.178625983775,0
-0.75348627550547,48.178537848826,0
-0.75342021313143,48.178453231166,0
-0.75334334242286,48.17837277477,0
-0.75325624844163,48.178297091938,0
-0.75315959404345,48.178226758639,0
-0.75305411483272,48.178162310129,0
-0.75294061356391,48.178104236878,0
-0.75281995403217,48.178052980835,0
-0.75269305450009,48.178008932069,0
-0.75256088070989,48.177972425798,0
-0.75242443853485,48.177943739842,0
-0.75228476632542,48.177923092503,0
-0.75214292700851,48.177910640912,0
-0.75199999999995,48.177906479827,0
-0.75185707299138,48.177910640912,0
-0.7517152336745,48.177923092503,0
-0.75157556146505,48.177943739842,0
-0.75143911929001,48.177972425798,0
-0.75130694549983,48.178008932069,0
-0.75118004596773,48.178052980835,0
-0.75105938643601,48.178104236878,0
-0.75094588516717,48.178162310129,0
-0.75084040595645,48.178226758639,0
-0.75074375155829,48.178297091938,0
-0.75065665757704,48.17837277477,0
-0.75057978686847,48.178453231166,0
-0.75051372449445,48.178537848826,0
-0.75045897326931,48.178625983775,0
-0.75041594993203,48.178716965272,0
-0.7503849819731,48.178810100904,0
-0.75036630514025,48.178904681863,0
-0.75036006164256,48.178999988337,0

-0.75199999999995,48.179,0
</coordinates>
</LinearRing>
</outerBoundaryIs>
</Polygon>
</Placemark>
<Placemark>
<name>179</name>
<styleUrl>#stylehap1</styleUrl>
<Polygon>
<outerBoundaryIs>
<LinearRing>
<coordinates>
-0.75099999999998,48.095,0
-0.74936274254217,48.094999988372,0
-0.74936896977786,48.095095295023,0
-0.7493876102072,48.095189876508,0
-0.74941852203207,48.095283012998,0
-0.74946147005742,48.095373995658,0
-0.74951612747945,48.095462132043,0
-0.74958207837111,48.095546751366,0
-0.7496588208459,48.095627209601,0
-0.74974577087647,48.095702894395,0
-0.74984226673846,48.095773229716,0
-0.74994757404615,48.095837680248,0
-0.7500608913413,48.09589575546,0
-0.75018135619302,48.095947013343,0
-0.7503080517618,48.095991063772,0
-0.75044001377823,48.096027571477,0
-0.75057623788281,48.096056258598,0
-0.75071568727123,48.096076906794,0
-0.75085730058683,48.096089358911,0
-0.75099999999999,48.096093520174,0
-0.75114269941312,48.096089358911,0
-0.75128431272872,48.096076906794,0
-0.75142376211714,48.096056258598,0
-0.75155998622172,48.096027571477,0
-0.75169194823815,48.095991063772,0
-0.75181864380693,48.095947013343,0
-0.75193910865865,48.09589575546,0
-0.7520524259538,48.095837680248,0
-0.7521577332615,48.095773229716,0
-0.75225422912348,48.095702894395,0
-0.75234117915405,48.095627209601,0
-0.75241792162884,48.095546751366,0
-0.7524838725205,48.095462132043,0
-0.75253852994253,48.095373995658,0
-0.75258147796789,48.095283012998,0
-0.75261238979275,48.095189876508,0
-0.75263103022209,48.095095295023,0
-0.75263725745778,48.094999988372,0
-0.75263102417563,48.094904681897,0
-0.75261237788351,48.094810100937,0
-0.75258146055776,48.094716965304,0
-0.75253850756051,48.094625983806,0
-0.75248384584661,48.094537848854,0
-0.75241789147362,48.094453231192,0

-0.7523411464337,48.094372774793,0
-0.75225419483222,48.094297091958,0
-0.75215769844122,48.094226758656,0
-0.75205239166254,48.094162310143,0
-0.7519390759383,48.094104236889,0
-0.75181861365171,48.094052980843,0
-0.75169192156429,48.094008932075,0
-0.7515599638397,48.093972425802,0
-0.75142374470701,48.093943739844,0
-0.75128430081947,48.093923092504,0
-0.75114269336664,48.093910640913,0
-0.75099999999999,48.093906479827,0
-0.75085730663332,48.093910640913,0
-0.75071569918048,48.093923092504,0
-0.75057625529294,48.093943739844,0
-0.75044003616025,48.093972425802,0
-0.75030807843566,48.094008932075,0
-0.75018138634824,48.094052980843,0
-0.75006092406165,48.094104236889,0
-0.74994760833742,48.094162310143,0
-0.74984230155874,48.094226758656,0
-0.74974580516774,48.094297091958,0
-0.74965885356625,48.094372774793,0
-0.74958210852636,48.094453231192,0
-0.74951615415334,48.094537848854,0
-0.74946149243944,48.094625983806,0
-0.74941853944219,48.094716965304,0
-0.74938762211644,48.094810100937,0
-0.74936897582433,48.094904681897,0
-0.74936274254217,48.094999988372,0
-0.75099999999998,48.095,0
</coordinates>
</LinearRing>
</outerBoundaryIs>
</Polygon>
</Placemark>
<Placemark>
<name>180</name>
<styleUrl>#stylehap1</styleUrl>
<Polygon>
<outerBoundaryIs>
<LinearRing>
<coordinates>
-0.74900000000002,48.147,0
-0.74736108438773,48.146999988351,0
-0.74736731792461,48.147095295002,0
-0.74738597722692,48.147189876487,0
-0.74741692035313,48.147283012978,0
-0.7474599118701,48.14737399564,0
-0.74751462464328,48.147462132026,0
-0.74758064232439,48.14754675135,0
-0.74765746251881,48.147627209587,0
-0.74774450060774,48.147702894382,0
-0.74784109419675,48.147773229705,0
-0.74794650815631,48.147837680239,0
-0.74805994021651,48.147895755453,0
-0.74818052707296,48.147947013337,0

-0.74830735095757,48.147991063768,0
-0.74843944662422,48.148027571475,0
-0.74857580869615,48.148056258597,0
-0.74871539931886,48.148076906794,0
-0.7488571560606,48.148089358911,0
-0.74900000000002,48.148093520174,0
-0.74914284393944,48.148089358911,0
-0.74928460068118,48.148076906794,0
-0.74942419130389,48.148056258597,0
-0.74956055337582,48.148027571475,0
-0.74969264904249,48.147991063768,0
-0.74981947292708,48.147947013337,0
-0.74994005978353,48.147895755453,0
-0.75005349184373,48.147837680239,0
-0.75015890580332,48.147773229705,0
-0.75025549939229,48.147702894382,0
-0.75034253748123,48.147627209587,0
-0.75041935767564,48.14754675135,0
-0.75048537535676,48.147462132026,0
-0.75054008812994,48.14737399564,0
-0.75058307964694,48.147283012978,0
-0.75061402277312,48.147189876487,0
-0.75063268207546,48.147095295002,0
-0.75063891561231,48.146999988351,0
-0.75063267601179,48.146904681876,0
-0.75061401083003,48.146810100917,0
-0.75058306218732,48.146716965284,0
-0.75054006568428,48.146625983787,0
-0.7504853486071,48.146537848836,0
-0.7504193274347,48.146453231176,0
-0.75034250466791,48.146372774779,0
-0.75025546500358,48.146297091945,0
-0.75015887088408,48.146226758645,0
-0.75005345745502,48.146162310134,0
-0.74994002697018,48.146104236882,0
-0.74981944268614,48.146052980838,0
-0.74969262229281,48.146008932071,0
-0.74956053093017,48.1459724258,0
-0.74942417384428,48.145943739843,0
-0.7492845887381,48.145923092504,0
-0.74914283787578,48.145910640912,0
-0.74900000000002,48.145906479827,0
-0.74885716212426,48.145910640912,0
-0.74871541126194,48.145923092504,0
-0.74857582615576,48.145943739843,0
-0.74843946906987,48.1459724258,0
-0.74830737770726,48.146008932071,0
-0.7481805573139,48.146052980838,0
-0.74805997302986,48.146104236882,0
-0.74794654254505,48.146162310134,0
-0.74784112911598,48.146226758645,0
-0.74774453499649,48.146297091945,0
-0.74765749533216,48.146372774779,0
-0.74758067256534,48.146453231176,0
-0.74751465139296,48.146537848836,0
-0.74745993431576,48.146625983787,0
-0.74741693781275,48.146716965284,0

-0.74738598917001,48.146810100917,0
-0.74736732398827,48.146904681876,0
-0.74736108438773,48.146999988351,0
-0.74900000000002,48.147,0
</coordinates>
</LinearRing>
</outerBoundaryIs>
</Polygon>
</Placemark>
<Placemark>
<name>181</name>
<styleUrl>#stylehap1</styleUrl>
<Polygon>
<outerBoundaryIs>
<LinearRing>
<coordinates>
-0.74900000000002,48.148,0
-0.74736105245399,48.14799998835,0
-0.74736728611222,48.148095295002,0
-0.747385945778,48.148189876487,0
-0.74741688950702,48.148283012978,0
-0.74745988186159,48.148373995639,0
-0.74751459570074,48.148462132026,0
-0.74758061466815,48.148546751349,0
-0.74765743635933,48.148627209587,0
-0.74774447614414,48.148702894382,0
-0.74784107161522,48.148773229705,0
-0.74794648762875,48.148837680239,0
-0.74805992189918,48.148895755453,0
-0.74818051110527,48.148947013337,0
-0.74830733746105,48.148991063768,0
-0.74843943570163,48.149027571475,0
-0.74857580043062,48.149056258597,0
-0.74871539377329,48.149076906794,0
-0.74885715327723,48.149089358911,0
-0.74900000000002,48.149093520174,0
-0.74914284672281,48.149089358911,0
-0.74928460622675,48.149076906794,0
-0.74942419956944,48.149056258597,0
-0.74956056429841,48.149027571475,0
-0.74969266253899,48.148991063768,0
-0.74981948889479,48.148947013337,0
-0.74994007810086,48.148895755453,0
-0.75005351237129,48.148837680239,0
-0.75015892838482,48.148773229705,0
-0.7502555238559,48.148702894382,0
-0.75034256364073,48.148627209587,0
-0.75041938533192,48.148546751349,0
-0.7504854042993,48.148462132026,0
-0.75054011813845,48.148373995639,0
-0.75058311049302,48.148283012978,0
-0.75061405422203,48.148189876487,0
-0.75063271388782,48.148095295002,0
-0.75063894754605,48.14799998835,0
-0.75063270782385,48.147904681876,0
-0.75061404227831,48.147810100916,0
-0.75058309303247,48.147716965284,0

-0.75054009569157,48.147625983787,0
-0.75048537754816,48.147537848836,0
-0.75041935508932,48.147453231176,0
-0.75034253082558,48.147372774779,0
-0.7502554894653,48.147297091945,0
-0.75015889346368,48.147226758645,0
-0.75005347798069,48.147162310134,0
-0.74994004528573,48.147104236882,0
-0.74981945865219,48.147052980838,0
-0.74969263578785,48.147008932071,0
-0.74956054185153,48.1469724258,0
-0.74942418210886,48.146943739842,0
-0.74928459428303,48.146923092504,0
-0.74914284065884,48.146910640912,0
-0.74900000000002,48.146906479826,0
-0.74885715934122,48.146910640912,0
-0.74871540571704,48.146923092504,0
-0.74857581789118,48.146943739842,0
-0.74843945814851,48.1469724258,0
-0.74830736421219,48.147008932071,0
-0.74818054134787,48.147052980838,0
-0.74805995471431,48.147104236882,0
-0.74794652201938,48.147162310134,0
-0.74784110653636,48.147226758645,0
-0.74774451053474,48.147297091945,0
-0.74765746917446,48.147372774779,0
-0.74758064491072,48.147453231176,0
-0.7475146224519,48.147537848836,0
-0.74745990430847,48.147625983787,0
-0.7474169069676,48.147716965284,0
-0.74738595772175,48.147810100916,0
-0.74736729217621,48.147904681876,0
-0.74736105245399,48.14799998835,0
-0.74900000000002,48.148,0
</coordinates>
</LinearRing>
</outerBoundaryIs>
</Polygon>
</Placemark>
<Placemark>
<name>182</name>
<styleUrl>#stylehap1</styleUrl>
<Polygon>
<outerBoundaryIs>
<LinearRing>
<coordinates>
-0.74700000000007,47.996,0
-0.74536588644183,47.995999988412,0
-0.74537210173032,47.996095295063,0
-0.74539070637602,47.996189876547,0
-0.74542155885281,47.996283013035,0
-0.74546442441696,47.996373995694,0
-0.74551897689202,47.996462132077,0
-0.74558480114926,47.996546751396,0
-0.74566139626581,47.996627209629,0
-0.74574817933566,47.996702894418,0
-0.74584448990499,47.996773229736,0

-0.74594959499824,47.996837680264,0
-0.7460626946962,47.996895755473,0
-0.74618292822407,47.996947013353,0
-0.74630938050305,47.996991063779,0
-0.74644108911553,47.997027571482,0
-0.74657705163084,47.997056258601,0
-0.74671623323582,47.997076906796,0
-0.74685757461211,47.997089358911,0
-0.74700000000008,47.997093520174,0
-0.74714242538805,47.997089358911,0
-0.74728376676433,47.997076906796,0
-0.74742294836929,47.997056258601,0
-0.74755891088459,47.997027571482,0
-0.7476906194971,47.996991063779,0
-0.74781707177608,47.996947013353,0
-0.74793730530395,47.996895755473,0
-0.74805040500191,47.996837680264,0
-0.74815551009516,47.996773229736,0
-0.74825182066449,47.996702894418,0
-0.74833860373431,47.996627209629,0
-0.74841519885087,47.996546751396,0
-0.74848102310813,47.996462132077,0
-0.74853557558316,47.996373995694,0
-0.74857844114734,47.996283013035,0
-0.74860929362413,47.996189876547,0
-0.74862789826983,47.996095295063,0
-0.74863411355829,47.995999988412,0
-0.74862789225591,47.995904681937,0
-0.74860928177898,47.995810100976,0
-0.74857842383093,47.995716965341,0
-0.74853555332162,47.995625983841,0
-0.74848099657785,47.995537848887,0
-0.74841516885796,47.995453231222,0
-0.74833857119011,47.99537277482,0
-0.7482517865578,47.995297091982,0
-0.74815547546233,47.995226758676,0
-0.74805037089522,47.99516231016,0
-0.74793727275973,47.995104236902,0
-0.74781704178317,47.995052980853,0
-0.74769059296679,47.995008932082,0
-0.74755888862305,47.994972425807,0
-0.74742293105287,47.994943739847,0
-0.74728375491918,47.994923092506,0
-0.7471424193741,47.994910640913,0
-0.74700000000008,47.994906479827,0
-0.74685758062603,47.994910640913,0
-0.74671624508094,47.994923092506,0
-0.74657706894726,47.994943739847,0
-0.7464411113771,47.994972425807,0
-0.74630940703333,47.995008932082,0
-0.74618295821698,47.995052980853,0
-0.7460627272404,47.995104236902,0
-0.74594962910493,47.99516231016,0
-0.74584452453782,47.995226758676,0
-0.74574821344233,47.995297091982,0
-0.74566142881004,47.99537277482,0
-0.74558483114217,47.995453231222,0

-0.7455190034223,47.995537848887,0
-0.74546444667853,47.995625983841,0
-0.74542157616923,47.995716965341,0
-0.74539071822115,47.995810100976,0
-0.74537210774424,47.995904681937,0
-0.74536588644183,47.995999988412,0
-0.74700000000007,47.996,0
</coordinates>
</LinearRing>
</outerBoundaryIs>
</Polygon>
</Placemark>
<Placemark>
<name>184</name>
<styleUrl>#stylehap1</styleUrl>
<Polygon>
<outerBoundaryIs>
<LinearRing>
<coordinates>
-0.745,48.173,0
-0.74336025354352,48.17299998834,0
-0.74336649023768,48.173095294991,0
-0.74338515899662,48.173189876477,0
-0.74341611780683,48.173283012968,0
-0.74345913111597,48.17337399563,0
-0.74351387162369,48.173462132017,0
-0.7435799227707,48.173546751342,0
-0.74365678190775,48.17362720958,0
-0.74374386411971,48.173702894376,0
-0.74384050667635,48.1737732297,0
-0.74394597407549,48.173837680235,0
-0.74405946364052,48.173895755449,0
-0.74418011162935,48.173947013335,0
-0.74430699980859,48.173991063766,0
-0.74443916244281,48.174027571474,0
-0.74457559364542,48.174056258596,0
-0.74471525503578,48.174076906793,0
-0.74485708364342,48.174089358911,0
-0.745,48.174093520174,0
-0.74514291635659,48.174089358911,0
-0.74528474496423,48.174076906793,0
-0.74542440635459,48.174056258596,0
-0.7455608375572,48.174027571474,0
-0.74569300019142,48.173991063766,0
-0.74581988837066,48.173947013335,0
-0.74594053635949,48.173895755449,0
-0.74605402592452,48.173837680235,0
-0.74615949332366,48.1737732297,0
-0.7462561358803,48.173702894376,0
-0.74634321809226,48.17362720958,0
-0.74642007722931,48.173546751342,0
-0.74648612837632,48.173462132017,0
-0.74654086888404,48.17337399563,0
-0.74658388219318,48.173283012968,0
-0.74661484100339,48.173189876477,0
-0.74663350976233,48.173095294991,0
-0.74663974645649,48.17299998834,0

-0.74663350369004,48.172904681866,0
-0.74661482904333,48.172810100906,0
-0.74658386470875,48.172716965274,0
-0.7465408464065,48.172625983778,0
-0.74648610158865,48.172537848828,0
-0.74642004694539,48.172453231168,0
-0.7463431852323,48.172372774772,0
-0.74625610144271,48.172297091939,0
-0.74615945835481,48.17222675864,0
-0.74605399148693,48.17216231013,0
-0.74594050349952,48.172104236878,0
-0.74581985808674,48.172052980835,0
-0.74569297340371,48.172008932069,0
-0.74556081507966,48.171972425798,0
-0.74542438887016,48.171943739842,0
-0.74528473300418,48.171923092503,0
-0.7451429102843,48.171910640912,0
-0.745,48.171906479827,0
-0.74485708971571,48.171910640912,0
-0.74471526699583,48.171923092503,0
-0.74457561112985,48.171943739842,0
-0.74443918492035,48.171972425798,0
-0.74430702659629,48.172008932069,0
-0.74418014191326,48.172052980835,0
-0.74405949650049,48.172104236878,0
-0.74394600851308,48.17216231013,0
-0.7438405416452,48.17222675864,0
-0.7437438985573,48.172297091939,0
-0.74365681476771,48.172372774772,0
-0.74357995305462,48.172453231168,0
-0.74351389841136,48.172537848828,0
-0.74345915359351,48.172625983778,0
-0.74341613529126,48.172716965274,0
-0.74338517095668,48.172810100906,0
-0.74336649630997,48.172904681866,0
-0.74336025354352,48.17299998834,0
-0.745,48.173,0
</coordinates>
</LinearRing>
</outerBoundaryIs>
</Polygon>
</Placemark>
<Placemark>
<name>185</name>
<styleUrl>#stylehap1</styleUrl>
<Polygon>
<outerBoundaryIs>
<LinearRing>
<coordinates>
-0.74400000000003,48.131,0
-0.74236159509049,48.130999988357,0
-0.74236782668662,48.131095295008,0
-0.74238648017618,48.131189876494,0
-0.74241741366172,48.131283012984,0
-0.74246039178351,48.131373995645,0
-0.74251508750884,48.131462132031,0
-0.74258108461915,48.131546751354,0

-0.74265788087633,48.131627209592,0
-0.74274489184376,48.131702894386,0
-0.74284145533337,48.131773229708,0
-0.74294683644471,48.131837680242,0
-0.74306023315791,48.131895755455,0
-0.74318078243755,48.131947013339,0
-0.74330756680156,48.131991063769,0
-0.74343962130466,48.132027571476,0
-0.74357594088338,48.132056258597,0
-0.74371548800669,48.132076906794,0
-0.74385720057393,48.132089358911,0
-0.74400000000002,48.132093520173,0
-0.74414279942613,48.132089358911,0
-0.74428451199338,48.132076906794,0
-0.74442405911668,48.132056258597,0
-0.74456037869541,48.132027571476,0
-0.7446924331985,48.131991063769,0
-0.74481921756251,48.131947013339,0
-0.74493976684216,48.131895755455,0
-0.74505316355533,48.131837680242,0
-0.74515854466667,48.131773229708,0
-0.74525510815628,48.131702894386,0
-0.74534211912371,48.131627209592,0
-0.74541891538089,48.131546751354,0
-0.74548491249122,48.131462132031,0
-0.74553960821653,48.131373995645,0
-0.74558258633832,48.131283012984,0
-0.74561351982389,48.131189876494,0
-0.74563217331342,48.131095295008,0
-0.74563840490958,48.130999988357,0
-0.74563216725508,48.130904681883,0
-0.74561350789124,48.130810100923,0
-0.74558256889397,48.13071696529,0
-0.74553958579049,48.130625983793,0
-0.7454848857649,48.130537848842,0
-0.74541888516636,48.130453231181,0
-0.74534208633901,48.130372774783,0
-0.74525507379759,48.130297091949,0
-0.74515850977795,48.130226758649,0
-0.74505312919664,48.130162310137,0
-0.74493973405746,48.130104236884,0
-0.74481918734798,48.13005298084,0
-0.74469240647217,48.130008932072,0
-0.74456035626934,48.1299724258,0
-0.74442404167231,48.129943739843,0
-0.74428450006072,48.129923092504,0
-0.74414279336776,48.129910640912,0
-0.74400000000002,48.129906479826,0
-0.74385720663231,48.129910640912,0
-0.74371549993934,48.129923092504,0
-0.74357595832776,48.129943739843,0
-0.7434396437307,48.1299724258,0
-0.74330759352789,48.130008932072,0
-0.74318081265209,48.13005298084,0
-0.74306026594261,48.130104236884,0
-0.74294687080343,48.130162310137,0
-0.74284149022212,48.130226758649,0

-0.74274492620247,48.130297091949,0
-0.74265791366102,48.130372774783,0
-0.74258111483368,48.130453231181,0
-0.74251511423517,48.130537848842,0
-0.74246041420958,48.130625983793,0
-0.74241743110609,48.13071696529,0
-0.74238649210883,48.130810100923,0
-0.74236783274499,48.130904681883,0
-0.74236159509049,48.130999988357,0
-0.74400000000003,48.131,0
</coordinates>
</LinearRing>
</outerBoundaryIs>
</Polygon>
</Placemark>
<Placemark>
<name>187</name>
<styleUrl>#stylehap1</styleUrl>
<Polygon>
<outerBoundaryIs>
<LinearRing>
<coordinates>
-0.74299999999994,48.112,0
-0.74136220097085,48.111999988365,0
-0.74136843026457,48.112095295016,0
-0.74138707685805,48.112189876501,0
-0.74141799890626,48.112283012991,0
-0.74146096113643,48.112373995652,0
-0.74151563663676,48.112462132038,0
-0.7415816093426,48.11254675136,0
-0.74165837720144,48.112627209597,0
-0.74174535599283,48.112702894391,0
-0.74184188377354,48.112773229712,0
-0.74194722591489,48.112837680245,0
-0.74206058069357,48.112895755458,0
-0.74218108539334,48.112947013341,0
-0.74230782287147,48.11299106377,0
-0.74243982853951,48.113027571477,0
-0.74257609770574,48.113056258598,0
-0.74271559322281,48.113076906794,0
-0.74285725338294,48.113089358911,0
-0.74299999999993,48.113093520174,0
-0.74314274661693,48.113089358911,0
-0.74328440677705,48.113076906794,0
-0.74342390229416,48.113056258598,0
-0.74356017146038,48.113027571477,0
-0.74369217712842,48.11299106377,0
-0.74381891460655,48.112947013341,0
-0.74393941930632,48.112895755458,0
-0.744052774085,48.112837680245,0
-0.74415811622633,48.112773229712,0
-0.74425464400704,48.112702894391,0
-0.74434162279843,48.112627209597,0
-0.7444183906573,48.11254675136,0
-0.74448436336311,48.112462132038,0
-0.74453903886344,48.112373995652,0
-0.74458200109363,48.112283012991,0

-0.74461292314185,48.112189876501,0
-0.7446315697353,48.112095295016,0
-0.74463779902902,48.111999988365,0
-0.74463156368321,48.11190468189,0
-0.74461291122156,48.11181010093,0
-0.74458198366735,48.111716965297,0
-0.74453901646063,48.1116259838,0
-0.74448433666449,48.111537848848,0
-0.74441836047406,48.111453231187,0
-0.74434159004772,48.111372774789,0
-0.74425460968394,48.111297091954,0
-0.74415808137376,48.111226758653,0
-0.7440527397619,48.11116231014,0
-0.74393938655562,48.111104236887,0
-0.74381888442332,48.111052980841,0
-0.7436921504298,48.111008932073,0
-0.74356014905757,48.110972425801,0
-0.74342388486787,48.110943739843,0
-0.74328439485679,48.110923092504,0
-0.74314274056484,48.110910640913,0
-0.74299999999993,48.110906479827,0
-0.74285725943503,48.110910640913,0
-0.7427156051431,48.110923092504,0
-0.74257611513202,48.110943739843,0
-0.74243985094232,48.110972425801,0
-0.74230784957009,48.111008932073,0
-0.74218111557655,48.111052980841,0
-0.74206061344428,48.111104236887,0
-0.74194726023799,48.11116231014,0
-0.74184191862613,48.111226758653,0
-0.74174539031592,48.111297091954,0
-0.74165840995217,48.111372774789,0
-0.74158163952581,48.111453231187,0
-0.74151566333538,48.111537848848,0
-0.74146098353924,48.1116259838,0
-0.74141801633254,48.111716965297,0
-0.74138708877833,48.11181010093,0
-0.74136843631665,48.11190468189,0
-0.74136220097085,48.111999988365,0
-0.74299999999994,48.112,0
</coordinates>
</LinearRing>
</outerBoundaryIs>
</Polygon>
</Placemark>
<Placemark>
<name>188</name>
<styleUrl>#stylehap1</styleUrl>
<Polygon>
<outerBoundaryIs>
<LinearRing>
<coordinates>
-0.74199999999996,48.173,0
-0.74036025354347,48.17299998834,0
-0.74036649023765,48.173095294991,0
-0.74038515899657,48.173189876477,0
-0.74041611780677,48.173283012968,0

-0.74045913111594,48.17337399563,0
-0.74051387162363,48.173462132017,0
-0.74057992277064,48.173546751342,0
-0.74065678190769,48.17362720958,0
-0.74074386411968,48.173702894376,0
-0.7408405066763,48.1737732297,0
-0.74094597407546,48.173837680235,0
-0.74105946364049,48.173895755449,0
-0.74118011162932,48.173947013335,0
-0.74130699980856,48.173991063766,0
-0.74143916244275,48.174027571474,0
-0.74157559364539,48.174056258596,0
-0.74171525503572,48.174076906793,0
-0.74185708364339,48.174089358911,0
-0.74199999999997,48.174093520174,0
-0.74214291635653,48.174089358911,0
-0.7422847449642,48.174076906793,0
-0.74242440635453,48.174056258596,0
-0.74256083755717,48.174027571474,0
-0.74269300019136,48.173991063766,0
-0.74281988837061,48.173947013335,0
-0.74294053635943,48.173895755449,0
-0.74305402592447,48.173837680235,0
-0.74315949332363,48.1737732297,0
-0.74325613588025,48.173702894376,0
-0.74334321809223,48.17362720958,0
-0.74342007722928,48.173546751342,0
-0.74348612837629,48.173462132017,0
-0.74354086888399,48.17337399563,0
-0.74358388219315,48.173283012968,0
-0.74361484100336,48.173189876477,0
-0.74363350976228,48.173095294991,0
-0.74363974645646,48.17299998834,0
-0.74363350369001,48.172904681866,0
-0.7436148290433,48.172810100906,0
-0.74358386470872,48.172716965274,0
-0.74354084640645,48.172625983778,0
-0.74348610158859,48.172537848828,0
-0.74342004694536,48.172453231168,0
-0.74334318523224,48.172372774772,0
-0.74325610144265,48.172297091939,0
-0.74315945835478,48.17222675864,0
-0.74305399148687,48.17216231013,0
-0.74294050349947,48.172104236878,0
-0.74281985808669,48.172052980835,0
-0.74269297340366,48.172008932069,0
-0.74256081507963,48.171972425798,0
-0.74242438887011,48.171943739842,0
-0.74228473300415,48.171923092503,0
-0.74214291028427,48.171910640912,0
-0.74199999999997,48.171906479827,0
-0.74185708971566,48.171910640912,0
-0.74171526699578,48.171923092503,0
-0.74157561112982,48.171943739842,0
-0.74143918492029,48.171972425798,0
-0.74130702659626,48.172008932069,0
-0.74118014191323,48.172052980835,0

-0.74105949650046,48.172104236878,0
-0.74094600851305,48.17216231013,0
-0.74084054164515,48.17222675864,0
-0.74074389855727,48.172297091939,0
-0.74065681476768,48.172372774772,0
-0.74057995305456,48.172453231168,0
-0.74051389841133,48.172537848828,0
-0.74045915359348,48.172625983778,0
-0.7404161352912,48.172716965274,0
-0.74038517095662,48.172810100906,0
-0.74036649630991,48.172904681866,0
-0.74036025354347,48.17299998834,0
-0.74199999999996,48.173,0
</coordinates>
</LinearRing>
</outerBoundaryIs>
</Polygon>
</Placemark>
<Placemark>
<name>189</name>
<styleUrl>#stylehap1</styleUrl>
<Polygon>
<outerBoundaryIs>
<LinearRing>
<coordinates>
-0.74099999999999,47.996,0
-0.73936588644175,47.995999988412,0
-0.73937210173023,47.996095295063,0
-0.73939070637594,47.996189876547,0
-0.73942155885272,47.996283013035,0
-0.73946442441688,47.996373995694,0
-0.73951897689193,47.996462132077,0
-0.73958480114917,47.996546751396,0
-0.73966139626573,47.996627209629,0
-0.73974817933558,47.996702894418,0
-0.73984448990491,47.996773229736,0
-0.73994959499815,47.996837680264,0
-0.74006269469611,47.996895755473,0
-0.74018292822398,47.996947013353,0
-0.74030938050297,47.996991063779,0
-0.74044108911545,47.997027571482,0
-0.74057705163075,47.997056258601,0
-0.74071623323574,47.997076906796,0
-0.74085757461202,47.997089358911,0
-0.74099999999999,47.997093520174,0
-0.74114242538796,47.997089358911,0
-0.74128376676424,47.997076906796,0
-0.7414229483692,47.997056258601,0
-0.74155891088451,47.997027571482,0
-0.74169061949701,47.996991063779,0
-0.741817071776,47.996947013353,0
-0.74193730530387,47.996895755473,0
-0.74205040500183,47.996837680264,0
-0.74215551009507,47.996773229736,0
-0.7422518206644,47.996702894418,0
-0.74233860373423,47.996627209629,0
-0.74241519885078,47.996546751396,0

-0.74248102310805,47.996462132077,0
-0.74253557558308,47.996373995694,0
-0.74257844114725,47.996283013035,0
-0.74260929362404,47.996189876547,0
-0.74262789826974,47.996095295063,0
-0.74263411355821,47.995999988412,0
-0.74262789225582,47.995904681937,0
-0.74260928177889,47.995810100976,0
-0.74257842383084,47.995716965341,0
-0.74253555332153,47.995625983841,0
-0.74248099657777,47.995537848887,0
-0.74241516885787,47.995453231222,0
-0.74233857119003,47.99537277482,0
-0.74225178655774,47.995297091982,0
-0.74215547546224,47.995226758676,0
-0.74205037089514,47.99516231016,0
-0.74193727275964,47.995104236902,0
-0.74181704178309,47.995052980853,0
-0.74169059296671,47.995008932082,0
-0.74155888862297,47.994972425807,0
-0.74142293105278,47.994943739847,0
-0.74128375491912,47.994923092506,0
-0.74114241937401,47.994910640913,0
-0.74099999999999,47.994906479827,0
-0.74085758062594,47.994910640913,0
-0.74071624508086,47.994923092506,0
-0.74057706894717,47.994943739847,0
-0.74044111137701,47.994972425807,0
-0.74030940703325,47.995008932082,0
-0.74018295821689,47.995052980853,0
-0.74006272724031,47.995104236902,0
-0.73994962910484,47.99516231016,0
-0.73984452453774,47.995226758676,0
-0.73974821344224,47.995297091982,0
-0.73966142880995,47.99537277482,0
-0.73958483114208,47.995453231222,0
-0.73951900342221,47.995537848887,0
-0.73946444667845,47.995625983841,0
-0.73942157616914,47.995716965341,0
-0.73939071822106,47.995810100976,0
-0.73937210774415,47.995904681937,0
-0.73936588644175,47.995999988412,0
-0.74099999999999,47.996,0
</coordinates>
</LinearRing>
</outerBoundaryIs>
</Polygon>
</Placemark>
<Placemark>
<name>190</name>
<styleUrl>#stylehap1</styleUrl>
<Polygon>
<outerBoundaryIs>
<LinearRing>
<coordinates>
-0.74099999999999,48.172,0
-0.73936028552086,48.17199998834,0

-0.73936652209351,48.172095294992,0
-0.73938519048847,48.172189876477,0
-0.73941614869504,48.172283012968,0
-0.73945916116548,48.172373995631,0
-0.73951390060572,48.172462132018,0
-0.73957995046472,48.172546751342,0
-0.73965680810296,48.17262720958,0
-0.73974388861671,48.172702894376,0
-0.73984052928868,48.1727732297,0
-0.73994599463107,48.172837680235,0
-0.74005948198287,48.17289575545,0
-0.74018012761884,48.172947013335,0
-0.74030701332354,48.172991063766,0
-0.74043917338029,48.173027571474,0
-0.74057560192223,48.173056258596,0
-0.74071526058888,48.173076906793,0
-0.74085708643059,48.173089358911,0
-0.74099999999999,48.173093520174,0
-0.74114291356939,48.173089358911,0
-0.74128473941107,48.173076906793,0
-0.74142439807773,48.173056258596,0
-0.74156082661966,48.173027571474,0
-0.74169298667643,48.172991063766,0
-0.74181987238113,48.172947013335,0
-0.74194051801711,48.17289575545,0
-0.74205400536889,48.172837680235,0
-0.74215947071127,48.1727732297,0
-0.74225611138325,48.172702894376,0
-0.74234319189702,48.17262720958,0
-0.74242004953526,48.172546751342,0
-0.74248609939426,48.172462132018,0
-0.7425408388345,48.172373995631,0
-0.74258385130491,48.172283012968,0
-0.74261480951148,48.172189876477,0
-0.74263347790647,48.172095294992,0
-0.74263971447912,48.17199998834,0
-0.74263347183451,48.171904681866,0
-0.74261479755209,48.171810100907,0
-0.74258383382146,48.171716965274,0
-0.74254081635818,48.171625983778,0
-0.74248607260801,48.171537848828,0
-0.742420019253,48.171453231169,0
-0.74234315903884,48.171372774772,0
-0.74225607694754,48.171297091939,0
-0.74215943574433,48.17122675864,0
-0.74205397093317,48.17116231013,0
-0.74194048515893,48.171104236879,0
-0.74181984209887,48.171052980835,0
-0.74169295989021,48.171008932069,0
-0.74156080414335,48.170972425798,0
-0.74142438059427,48.170943739842,0
-0.74128472745168,48.170923092503,0
-0.74114290749743,48.170910640912,0
-0.74099999999999,48.170906479826,0
-0.74085709250252,48.170910640912,0
-0.74071527254828,48.170923092503,0
-0.74057561940571,48.170943739842,0

-0.74043919585661,48.170972425798,0
-0.74030704010977,48.171008932069,0
-0.74018015790111,48.171052980835,0
-0.74005951484105,48.171104236879,0
-0.73994602906678,48.17116231013,0
-0.73984056425562,48.17122675864,0
-0.73974392305242,48.171297091939,0
-0.73965684096112,48.171372774772,0
-0.73957998074698,48.171453231169,0
-0.73951392739195,48.171537848828,0
-0.73945918364177,48.171625983778,0
-0.73941616617852,48.171716965274,0
-0.73938520244787,48.171810100907,0
-0.73936652816545,48.171904681866,0
-0.73936028552086,48.17199998834,0
-0.74099999999999,48.172,0
</coordinates>
</LinearRing>
</outerBoundaryIs>
</Polygon>
</Placemark>
<Placemark>
<name>192</name>
<styleUrl>#stylehap1</styleUrl>
<Polygon>
<outerBoundaryIs>
<LinearRing>
<coordinates>
-0.73900000000003,48.152,0
-0.73736092470162,48.151999988349,0
-0.7373671588453,48.152095295,0
-0.73738581996519,48.152189876485,0
-0.73741676610579,48.152283012976,0
-0.73745976181117,48.152373995638,0
-0.73751447991486,48.152462132024,0
-0.73758050402803,48.152546751348,0
-0.73765733170712,48.152627209586,0
-0.73774437827639,48.152702894381,0
-0.73784098127683,48.152773229704,0
-0.73794640550736,48.152837680238,0
-0.73805984861984,48.152895755452,0
-0.73818044722577,48.152947013337,0
-0.73830728346765,48.152991063767,0
-0.73843939200529,48.153027571475,0
-0.73857576736397,48.153056258597,0
-0.73871537158803,48.153076906794,0
-0.73885714214221,48.153089358911,0
-0.73900000000002,48.153093520174,0
-0.73914285785786,48.153089358911,0
-0.73928462841203,48.153076906794,0
-0.73942423263609,48.153056258597,0
-0.73956060799478,48.153027571475,0
-0.73969271653241,48.152991063767,0
-0.7398195527743,48.152947013337,0
-0.73994015138023,48.152895755452,0
-0.7400535944927,48.152837680238,0
-0.74015901872324,48.152773229704,0

-0.74025562172368,48.152702894381,0
-0.74034266829294,48.152627209586,0
-0.74041949597204,48.152546751348,0
-0.74048552008521,48.152462132024,0
-0.74054023818889,48.152373995638,0
-0.74058323389427,48.152283012976,0
-0.74061418003488,48.152189876485,0
-0.74063284115477,48.152095295,0
-0.74063907529845,48.151999988349,0
-0.74063283508945,48.151904681874,0
-0.74061416808854,48.151810100915,0
-0.7405832164299,48.151716965282,0
-0.7405402157371,48.151625983785,0
-0.74048549332822,48.151537848835,0
-0.74041946572283,48.151453231175,0
-0.74034263547064,48.151372774778,0
-0.74025558732555,48.151297091944,0
-0.74015898379446,48.151226758644,0
-0.7400535600946,48.151162310134,0
-0.73994011855792,48.151104236881,0
-0.73981952252509,48.151052980837,0
-0.73969268977543,48.151008932071,0
-0.73956058554299,48.150972425799,0
-0.73942421517172,48.150943739842,0
-0.73928461646569,48.150923092504,0
-0.73914285179254,48.150910640912,0
-0.73900000000002,48.150906479827,0
-0.73885714820753,48.150910640912,0
-0.73871538353437,48.150923092504,0
-0.73857578482835,48.150943739842,0
-0.73843941445708,48.150972425799,0
-0.73830731022464,48.151008932071,0
-0.73818047747498,48.151052980837,0
-0.73805988144215,48.151104236881,0
-0.73794643990547,48.151162310134,0
-0.73784101620561,48.151226758644,0
-0.73774441267452,48.151297091944,0
-0.73765736452943,48.151372774778,0
-0.73758053427724,48.151453231175,0
-0.73751450667185,48.151537848835,0
-0.73745978426296,48.151625983785,0
-0.73741678357017,48.151716965282,0
-0.73738583191153,48.151810100915,0
-0.73736716491062,48.151904681874,0
-0.73736092470162,48.151999988349,0
-0.73900000000003,48.152,0
</coordinates>
</LinearRing>
</outerBoundaryIs>
</Polygon>
</Placemark>
<Placemark>
<name>193</name>
<styleUrl>#stylehap1</styleUrl>
<Polygon>
<outerBoundaryIs>
<LinearRing>

<coordinates>
-0.73900000000003,48.324,0
-0.73735540484507,48.323999988278,0
-0.73736165996482,48.32409529493,0
-0.73738038391122,48.324189876417,0
-0.73741143425128,48.32428301291,0
-0.73745457473665,48.324373995575,0
-0.73750947709946,48.324462131966,0
-0.73757572354913,48.324546751295,0
-0.73765280995027,48.324627209538,0
-0.73774014965843,48.32470289434,0
-0.73783707798382,48.324773229669,0
-0.73794285724944,48.324837680209,0
-0.73805668240513,48.324895755429,0
-0.73817768715459,48.324947013319,0
-0.73830495054906,48.324991063755,0
-0.73843750399709,48.325027571466,0
-0.73857433863746,48.325056258592,0
-0.73871441301857,48.325076906791,0
-0.73885666102637,48.32508935891,0
-0.73900000000002,48.325093520174,0
-0.7391433389737,48.32508935891,0
-0.7392855869815,48.325076906791,0
-0.7394256613626,48.325056258592,0
-0.73956249600298,48.325027571466,0
-0.739695049451,48.324991063755,0
-0.73982231284548,48.324947013319,0
-0.73994331759493,48.324895755429,0
-0.74005714275062,48.324837680209,0
-0.74016292201625,48.324773229669,0
-0.74025985034164,48.32470289434,0
-0.7403471900498,48.324627209538,0
-0.74042427645094,48.324546751295,0
-0.7404905229006,48.324462131966,0
-0.74054542526341,48.324373995575,0
-0.74058856574879,48.32428301291,0
-0.74061961608885,48.324189876417,0
-0.74063834003525,48.32409529493,0
-0.74064459515499,48.323999988278,0
-0.7406383339126,48.323904681804,0
-0.74061960402963,48.323810100846,0
-0.74058854811938,48.323716965216,0
-0.74054540259949,48.323625983723,0
-0.7404904958908,48.323537848777,0
-0.74042424591591,48.323453231122,0
-0.74034715691735,48.32337277473,0
-0.74025981561848,48.323297091903,0
-0.74016288675743,48.323226758609,0
-0.74005710802746,48.323162310104,0
-0.73994328446251,48.323104236858,0
-0.73982228231045,48.32305298082,0
-0.7396950224412,48.323008932058,0
-0.73956247333906,48.322972425791,0
-0.73942564373322,48.322943739838,0
-0.73928557492228,48.322923092502,0
-0.73914333285108,48.322910640912,0
-0.73900000000002,48.322906479826,0

-0.73885666714899,48.322910640912,0
-0.73871442507778,48.322923092502,0
-0.73857435626685,48.322943739838,0
-0.73843752666101,48.322972425791,0
-0.73830497755887,48.323008932058,0
-0.73817771768962,48.32305298082,0
-0.73805671553756,48.323104236858,0
-0.7379428919726,48.323162310104,0
-0.73783711324263,48.323226758609,0
-0.73774018438159,48.323297091903,0
-0.73765284308272,48.32337277473,0
-0.73757575408416,48.323453231122,0
-0.73750950410927,48.323537848777,0
-0.73745459740057,48.323625983723,0
-0.73741145188069,48.323716965216,0
-0.73738039597043,48.323810100846,0
-0.73736166608746,48.323904681804,0
-0.73735540484507,48.323999988278,0
-0.73900000000003,48.324,0
</coordinates>
</LinearRing>
</outerBoundaryIs>
</Polygon>
</Placemark>
<Placemark>
<name>194</name>
<styleUrl>#stylehap1</styleUrl>
<Polygon>
<outerBoundaryIs>
<LinearRing>
<coordinates>
-0.73500000000001,48.049,0
-0.73336420545472,48.048999988391,0
-0.73337042713116,48.049095295042,0
-0.73338905090976,48.049189876526,0
-0.73341993511889,48.049283013015,0
-0.73346284477357,48.049373995675,0
-0.73351745336193,48.049462132059,0
-0.73358334532834,48.04954675138,0
-0.7336600192347,48.049627209614,0
-0.73374689157546,48.049702894406,0
-0.73384330121749,48.049773229725,0
-0.7339485144312,48.049837680256,0
-0.73406173047449,48.049895755466,0
-0.73418208768708,48.049947013347,0
-0.73430867004879,48.049991063775,0
-0.73444051415186,48.05002757148,0
-0.73457661653427,48.050056258599,0
-0.73471594131828,48.050076906795,0
-0.73485742809566,48.050089358911,0
-0.73500000000001,48.050093520174,0
-0.73514257190435,48.050089358911,0
-0.73528405868173,48.050076906795,0
-0.73542338346574,48.050056258599,0
-0.73555948584818,48.05002757148,0
-0.73569132995124,48.049991063775,0
-0.73581791231293,48.049947013347,0

-0.73593826952555,48.049895755466,0
-0.73605148556883,48.049837680256,0
-0.73615669878254,48.049773229725,0
-0.73625310842457,48.049702894406,0
-0.73633998076531,48.049627209614,0
-0.73641665467169,48.04954675138,0
-0.7364825466381,48.049462132059,0
-0.73653715522644,48.049373995675,0
-0.73658006488115,48.049283013015,0
-0.73661094909027,48.049189876526,0
-0.73662957286887,48.049095295042,0
-0.73663579454531,48.048999988391,0
-0.73662956683755,48.048904681916,0
-0.73661093721088,48.048810100955,0
-0.73658004751463,48.048716965321,0
-0.7365371329005,48.048625983822,0
-0.73648252003108,48.048537848869,0
-0.73641662459202,48.048453231206,0
-0.73633994812697,48.048372774806,0
-0.73625307421921,48.048297091969,0
-0.73615666404954,48.048226758665,0
-0.73605145136349,48.048162310151,0
-0.73593823688718,48.048104236895,0
-0.73581788223325,48.048052980848,0
-0.7356913033442,48.048008932078,0
-0.73555946352223,48.047972425804,0
-0.73542336609925,48.047943739845,0
-0.73528404680235,48.047923092505,0
-0.73514256587302,48.047910640913,0
-0.73500000000001,48.047906479827,0
-0.73485743412699,48.047910640913,0
-0.73471595319768,48.047923092505,0
-0.73457663390079,48.047943739845,0
-0.7344405364778,48.047972425804,0
-0.73430869665581,48.048008932078,0
-0.73418211776676,48.048052980848,0
-0.73406176311286,48.048104236895,0
-0.73394854863654,48.048162310151,0
-0.7338433359505,48.048226758665,0
-0.7337469257808,48.048297091969,0
-0.73366005187306,48.048372774806,0
-0.73358337540799,48.048453231206,0
-0.73351747996895,48.048537848869,0
-0.73346286709954,48.048625983822,0
-0.73341995248541,48.048716965321,0
-0.73338906278913,48.048810100955,0
-0.73337043316249,48.048904681916,0
-0.73336420545472,48.048999988391,0
-0.73500000000001,48.049,0
</coordinates>
</LinearRing>
</outerBoundaryIs>
</Polygon>
</Placemark>
<Placemark>
<name>195</name>
<styleUrl>#stylehap1</styleUrl>

<Polygon>
<outerBoundaryIs>
<LinearRing>
<coordinates>
-0.73400000000004,48.049,0
-0.73236420545474,48.048999988391,0
-0.73237042713118,48.049095295042,0
-0.73238905090978,48.049189876526,0
-0.73241993511891,48.049283013015,0
-0.73246284477361,48.049373995675,0
-0.73251745336195,48.049462132059,0
-0.73258334532836,48.04954675138,0
-0.73266001923474,48.049627209614,0
-0.73274689157548,48.049702894406,0
-0.73284330121751,48.049773229725,0
-0.73294851443122,48.049837680256,0
-0.7330617304745,48.049895755466,0
-0.73318208768712,48.049947013347,0
-0.73330867004881,48.049991063775,0
-0.73344051415187,48.05002757148,0
-0.73357661653431,48.050056258599,0
-0.73371594131833,48.050076906795,0
-0.7338574280957,48.050089358911,0
-0.73400000000005,48.050093520174,0
-0.73414257190439,48.050089358911,0
-0.73428405868177,48.050076906795,0
-0.73442338346578,48.050056258599,0
-0.73455948584819,48.05002757148,0
-0.73469132995126,48.049991063775,0
-0.73481791231297,48.049947013347,0
-0.73493826952556,48.049895755466,0
-0.73505148556885,48.049837680256,0
-0.73515669878256,48.049773229725,0
-0.73525310842459,48.049702894406,0
-0.73533998076535,48.049627209614,0
-0.73541665467171,48.04954675138,0
-0.73548254663812,48.049462132059,0
-0.73553715522648,48.049373995675,0
-0.73558006488116,48.049283013015,0
-0.73561094909029,48.049189876526,0
-0.73562957286889,48.049095295042,0
-0.73563579454533,48.048999988391,0
-0.73562956683756,48.048904681916,0
-0.73561093721092,48.048810100955,0
-0.73558004751465,48.048716965321,0
-0.73553713290051,48.048625983822,0
-0.7354825200311,48.048537848869,0
-0.73541662459206,48.048453231206,0
-0.73533994812699,48.048372774806,0
-0.73525307421925,48.048297091969,0
-0.73515666404955,48.048226758665,0
-0.73505145136351,48.048162310151,0
-0.73493823688719,48.048104236895,0
-0.73481788223329,48.048052980848,0
-0.73469130334424,48.048008932078,0
-0.73455946352225,48.047972425804,0
-0.73442336609926,48.047943739845,0

-0.73428404680237,48.047923092505,0
-0.73414256587306,48.047910640913,0
-0.73400000000005,48.047906479827,0
-0.73385743412703,48.047910640913,0
-0.7337159531977,48.047923092505,0
-0.7335766339008,48.047943739845,0
-0.73344053647782,48.047972425804,0
-0.73330869665585,48.048008932078,0
-0.7331821177668,48.048052980848,0
-0.73306176311287,48.048104236895,0
-0.73294854863656,48.048162310151,0
-0.73284333595052,48.048226758665,0
-0.73274692578084,48.048297091969,0
-0.73266005187308,48.048372774806,0
-0.73258337540803,48.048453231206,0
-0.73251747996897,48.048537848869,0
-0.73246286709955,48.048625983822,0
-0.73241995248542,48.048716965321,0
-0.73238906278917,48.048810100955,0
-0.7323704331625,48.048904681916,0
-0.73236420545474,48.048999988391,0
-0.73400000000004,48.049,0
</coordinates>
</LinearRing>
</outerBoundaryIs>
</Polygon>
</Placemark>
<Placemark>
<name>196</name>
<styleUrl>#stylehap1</styleUrl>
<Polygon>
<outerBoundaryIs>
<LinearRing>
<coordinates>
-0.73400000000004,48.206,0
-0.73235919730975,48.205999988326,0
-0.73236543801774,48.206095294978,0
-0.73238411879865,48.206189876464,0
-0.73241509754757,48.206283012955,0
-0.73245813856064,48.206373995618,0
-0.73251291432664,48.206462132006,0
-0.73257900801813,48.206546751331,0
-0.73265591666206,48.206627209571,0
-0.73274305496664,48.206702894368,0
-0.73283975977475,48.206773229693,0
-0.73294529511046,48.206837680229,0
-0.73305885778009,48.206895755445,0
-0.73317958348515,48.206947013331,0
-0.73330655340079,48.206991063764,0
-0.73343880116932,48.207027571472,0
-0.73357532025625,48.207056258595,0
-0.73371507161193,48.207076906793,0
-0.73385699158114,48.20708935891,0
-0.73400000000005,48.207093520174,0
-0.73414300841893,48.20708935891,0
-0.73428492838813,48.207076906793,0
-0.73442467974381,48.207056258595,0

-0.73456119883075,48.207027571472,0
-0.7346934465993,48.206991063764,0
-0.73482041651491,48.206947013331,0
-0.73494114221998,48.206895755445,0
-0.73505470488961,48.206837680229,0
-0.73516024022534,48.206773229693,0
-0.73525694503343,48.206702894368,0
-0.735344083338,48.206627209571,0
-0.73542099198196,48.206546751331,0
-0.73548708567343,48.206462132006,0
-0.73554186143945,48.206373995618,0
-0.73558490245249,48.206283012955,0
-0.73561588120145,48.206189876464,0
-0.73563456198233,48.206095294978,0
-0.73564080269032,48.205999988326,0
-0.7356345558991,48.205904681852,0
-0.73561586921981,48.205810100893,0
-0.73558488493652,48.205716965261,0
-0.73554183892133,48.205625983766,0
-0.73548705883741,48.205537848817,0
-0.73542096164339,48.205453231158,0
-0.73534405041873,48.205372774763,0
-0.7352569105337,48.205297091931,0
-0.73516020519336,48.205226758633,0
-0.73505467038988,48.205162310124,0
-0.7349411093007,48.205104236874,0
-0.73482038617634,48.205052980832,0
-0.73469341976326,48.205008932067,0
-0.73456117631265,48.204972425797,0
-0.73442466222784,48.204943739841,0
-0.7342849164065,48.204923092503,0
-0.7341430023357,48.204910640912,0
-0.73400000000005,48.204906479826,0
-0.73385699766437,48.204910640912,0
-0.73371508359356,48.204923092503,0
-0.73357533777223,48.204943739841,0
-0.73343882368744,48.204972425797,0
-0.73330658023681,48.205008932067,0
-0.73317961382373,48.205052980832,0
-0.73305889069936,48.205104236874,0
-0.73294532961022,48.205162310124,0
-0.73283979480671,48.205226758633,0
-0.73274308946639,48.205297091931,0
-0.73265594958134,48.205372774763,0
-0.73257903835667,48.205453231158,0
-0.73251294116268,48.205537848817,0
-0.73245816107874,48.205625983766,0
-0.73241511506358,48.205716965261,0
-0.73238413078028,48.205810100893,0
-0.73236544410097,48.205904681852,0
-0.73235919730975,48.205999988326,0
-0.73400000000004,48.206,0
</coordinates>
</LinearRing>
</outerBoundaryIs>
</Polygon>
</Placemark>

<Placemark>
<name>197</name>
<styleUrl>#stylehap1</styleUrl>
<Polygon>
<outerBoundaryIs>
<LinearRing>
<coordinates>
-0.73400000000004,48.049,0
-0.73236420545474,48.048999988391,0
-0.73237042713118,48.049095295042,0
-0.73238905090978,48.049189876526,0
-0.73241993511891,48.049283013015,0
-0.73246284477361,48.049373995675,0
-0.73251745336195,48.049462132059,0
-0.73258334532836,48.04954675138,0
-0.73266001923474,48.049627209614,0
-0.73274689157548,48.049702894406,0
-0.73284330121751,48.049773229725,0
-0.73294851443122,48.049837680256,0
-0.7330617304745,48.049895755466,0
-0.73318208768712,48.049947013347,0
-0.73330867004881,48.049991063775,0
-0.73344051415187,48.05002757148,0
-0.73357661653431,48.050056258599,0
-0.73371594131833,48.050076906795,0
-0.7338574280957,48.050089358911,0
-0.73400000000005,48.050093520174,0
-0.73414257190439,48.050089358911,0
-0.73428405868177,48.050076906795,0
-0.73442338346578,48.050056258599,0
-0.73455948584819,48.05002757148,0
-0.73469132995126,48.049991063775,0
-0.73481791231297,48.049947013347,0
-0.73493826952556,48.049895755466,0
-0.73505148556885,48.049837680256,0
-0.73515669878256,48.049773229725,0
-0.73525310842459,48.049702894406,0
-0.73533998076535,48.049627209614,0
-0.73541665467171,48.04954675138,0
-0.73548254663812,48.049462132059,0
-0.73553715522648,48.049373995675,0
-0.73558006488116,48.049283013015,0
-0.73561094909029,48.049189876526,0
-0.73562957286889,48.049095295042,0
-0.73563579454533,48.048999988391,0
-0.73562956683756,48.048904681916,0
-0.73561093721092,48.048810100955,0
-0.73558004751465,48.048716965321,0
-0.73553713290051,48.048625983822,0
-0.7354825200311,48.048537848869,0
-0.73541662459206,48.048453231206,0
-0.73533994812699,48.048372774806,0
-0.73525307421925,48.048297091969,0
-0.73515666404955,48.048226758665,0
-0.73505145136351,48.048162310151,0
-0.73493823688719,48.048104236895,0
-0.73481788223329,48.048052980848,0

-0.73469130334424,48.048008932078,0
-0.73455946352225,48.047972425804,0
-0.73442336609926,48.047943739845,0
-0.73428404680237,48.047923092505,0
-0.73414256587306,48.047910640913,0
-0.73400000000005,48.047906479827,0
-0.73385743412703,48.047910640913,0
-0.7337159531977,48.047923092505,0
-0.7335766339008,48.047943739845,0
-0.73344053647782,48.047972425804,0
-0.73330869665585,48.048008932078,0
-0.7331821177668,48.048052980848,0
-0.73306176311287,48.048104236895,0
-0.73294854863656,48.048162310151,0
-0.73284333595052,48.048226758665,0
-0.73274692578084,48.048297091969,0
-0.73266005187308,48.048372774806,0
-0.73258337540803,48.048453231206,0
-0.73251747996897,48.048537848869,0
-0.73246286709955,48.048625983822,0
-0.73241995248542,48.048716965321,0
-0.73238906278917,48.048810100955,0
-0.7323704331625,48.048904681916,0
-0.73236420545474,48.048999988391,0
-0.73400000000004,48.049,0
</coordinates>
</LinearRing>
</outerBoundaryIs>
</Polygon>
</Placemark>
<Placemark>
<name>198</name>
<styleUrl>#stylehap1</styleUrl>
<Polygon>
<outerBoundaryIs>
<LinearRing>
<coordinates>
-0.730999999999999,48.206,0
-0.72935919730972,48.205999988326,0
-0.72936543801768,48.206095294978,0
-0.72938411879859,48.206189876464,0
-0.72941509754754,48.206283012955,0
-0.72945813856058,48.206373995618,0
-0.72951291432658,48.206462132006,0
-0.72957900801807,48.206546751331,0
-0.72965591666201,48.206627209571,0
-0.72974305496661,48.206702894368,0
-0.7298397597747,48.206773229693,0
-0.72994529511043,48.206837680229,0
-0.73005885778006,48.206895755445,0
-0.73017958348512,48.206947013331,0
-0.73030655340073,48.206991063764,0
-0.73043880116929,48.207027571472,0
-0.73057532025622,48.207056258595,0
-0.7307150716119,48.207076906793,0
-0.73085699158111,48.20708935891,0
-0.730999999999999,48.207093520174,0

-0.7311430084189,48.20708935891,0
-0.7312849283881,48.207076906793,0
-0.73142467974378,48.207056258595,0
-0.73156119883072,48.207027571472,0
-0.73169344659925,48.206991063764,0
-0.73182041651486,48.206947013331,0
-0.73194114221995,48.206895755445,0
-0.73205470488958,48.206837680229,0
-0.73216024022528,48.206773229693,0
-0.7322569450334,48.206702894368,0
-0.73234408333797,48.206627209571,0
-0.73242099198191,48.206546751331,0
-0.7324870856734,48.206462132006,0
-0.7325418614394,48.206373995618,0
-0.73258490245244,48.206283012955,0
-0.73261588120139,48.206189876464,0
-0.7326345619823,48.206095294978,0
-0.73264080269029,48.205999988326,0
-0.73263455589907,48.205904681852,0
-0.73261586921976,48.205810100893,0
-0.73258488493646,48.205716965261,0
-0.7325418389213,48.205625983766,0
-0.73248705883735,48.205537848817,0
-0.73242096164336,48.205453231158,0
-0.7323440504187,48.205372774763,0
-0.73225691053364,48.205297091931,0
-0.73216020519333,48.205226758633,0
-0.73205467038982,48.205162310124,0
-0.73194110930067,48.205104236874,0
-0.73182038617631,48.205052980832,0
-0.73169341976323,48.205008932067,0
-0.73156117631259,48.204972425797,0
-0.73142466222778,48.204943739841,0
-0.73128491640645,48.204923092503,0
-0.73114300233564,48.204910640912,0
-0.73099999999999,48.204906479826,0
-0.73085699766434,48.204910640912,0
-0.73071508359353,48.204923092503,0
-0.7305753377722,48.204943739841,0
-0.73043882368739,48.204972425797,0
-0.73030658023678,48.205008932067,0
-0.7301796138237,48.205052980832,0
-0.73005889069933,48.205104236874,0
-0.72994532961016,48.205162310124,0
-0.72983979480668,48.205226758633,0
-0.72974308946634,48.205297091931,0
-0.72965594958128,48.205372774763,0
-0.72957903835664,48.205453231158,0
-0.72951294116263,48.205537848817,0
-0.72945816107871,48.205625983766,0
-0.72941511506352,48.205716965261,0
-0.72938413078022,48.205810100893,0
-0.72936544410094,48.205904681852,0
-0.72935919730972,48.205999988326,0
-0.73099999999999,48.206,0
</coordinates>
</LinearRing>

</outerBoundaryIs>
</Polygon>
</Placemark>
<Placemark>
<name>199</name>
<styleUrl>#stylehap1</styleUrl>
<Polygon>
<outerBoundaryIs>
<LinearRing>
<coordinates>
-0.72800000000007,48.123,0
-0.72636185027467,48.12299998836,0
-0.72636808090107,48.123095295012,0
-0.72638673148614,48.123189876497,0
-0.72641766015452,48.123283012987,0
-0.7264606315831,48.123373995648,0
-0.72651531879009,48.123462132034,0
-0.72658130562174,48.123546751357,0
-0.72665808991815,48.123627209594,0
-0.72674508733369,48.123702894388,0
-0.72684163578347,48.12377322971,0
-0.72694700048148,48.123837680243,0
-0.72706037953272,48.123895755456,0
-0.72718091003626,48.12394701334,0
-0.72730767465292,48.12399106377,0
-0.72743970858772,48.124027571476,0
-0.72757600693375,48.124056258597,0
-0.72771553232158,48.124076906794,0
-0.72785722281605,48.124089358911,0
-0.72800000000006,48.124093520173,0
-0.72814277718407,48.124089358911,0
-0.72828446767855,48.124076906794,0
-0.7284239930664,48.124056258597,0
-0.72856029141243,48.124027571476,0
-0.72869232534723,48.12399106377,0
-0.72881908996389,48.12394701334,0
-0.72893962046742,48.123895755456,0
-0.72905299951867,48.123837680243,0
-0.72915836421667,48.12377322971,0
-0.72925491266643,48.123702894388,0
-0.72934191008198,48.123627209594,0
-0.72941869437839,48.123546751357,0
-0.72948468121006,48.123462132034,0
-0.72953936841702,48.123373995648,0
-0.72958233984561,48.123283012987,0
-0.72961326851398,48.123189876497,0
-0.72963191909906,48.123095295012,0
-0.72963814972545,48.12299998836,0
-0.72963191304333,48.122904681886,0
-0.72961325658654,48.122810100926,0
-0.72958232240887,48.122716965293,0
-0.72953934600075,48.122625983796,0
-0.72948465449538,48.122537848844,0
-0.72941866417704,48.122453231184,0
-0.7293418773116,48.122372774786,0
-0.72925487832273,48.122297091951,0
-0.72915832934316,48.12222675865,0

-0.72905296517496,48.122162310139,0
-0.72893958769702,48.122104236885,0
-0.72881905976254,48.12205298084,0
-0.72869229863256,48.122008932073,0
-0.72856026899616,48.121972425801,0
-0.72842397562963,48.121943739843,0
-0.72828445575111,48.121923092504,0
-0.72814277112834,48.121910640912,0
-0.72800000000006,48.121906479826,0
-0.72785722887178,48.121910640912,0
-0.72771554424901,48.121923092504,0
-0.72757602437049,48.121943739843,0
-0.72743973100396,48.121972425801,0
-0.72730770136757,48.122008932073,0
-0.72718094023758,48.12205298084,0
-0.7270604123031,48.122104236885,0
-0.72694703482516,48.122162310139,0
-0.72684167065698,48.12222675865,0
-0.7267451216774,48.122297091951,0
-0.72665812268852,48.122372774786,0
-0.72658133582309,48.122453231184,0
-0.72651534550474,48.122537848844,0
-0.72646065399937,48.122625983796,0
-0.72641767759128,48.122716965293,0
-0.72638674341358,48.122810100926,0
-0.72636808695679,48.122904681886,0
-0.72636185027467,48.12299998836,0
-0.72800000000007,48.123,0
</coordinates>
</LinearRing>
</outerBoundaryIs>
</Polygon>
</Placemark>
<Placemark>
<name>200</name>
<styleUrl>#stylehap1</styleUrl>
<Polygon>
<outerBoundaryIs>
<LinearRing>
<coordinates>
-0.726,48.376,0
-0.72435372579883,48.375999988256,0
-0.72435998729915,48.376095294909,0
-0.72437873035628,48.376189876396,0
-0.72440981239202,48.37628301289,0
-0.72445299691695,48.376373995557,0
-0.72450795532826,48.376462131949,0
-0.72457426940889,48.376546751279,0
-0.72465143450886,48.376627209524,0
-0.72473886338489,48.376702894327,0
-0.72483589066855,48.376773229658,0
-0.72494177792981,48.3768376802,0
-0.72505571929651,48.376895755422,0
-0.72517684758791,48.376947013314,0
-0.72530424091492,48.376991063751,0
-0.72543692969712,48.377027571464,0
-0.72557390404313,48.37705625859,0

-0.72571412143801,48.377076906791,0
-0.72585651467907,48.37708935891,0
-0.72599999999999,48.377093520174,0
-0.72614348532091,48.37708935891,0
-0.72628587856197,48.377076906791,0
-0.72642609595685,48.37705625859,0
-0.72656307030288,48.377027571464,0
-0.72669575908509,48.376991063751,0
-0.72682315241209,48.376947013314,0
-0.7269442807035,48.376895755422,0
-0.7270582220702,48.3768376802,0
-0.72716410933145,48.376773229658,0
-0.72726113661512,48.376702894327,0
-0.72734856549112,48.376627209524,0
-0.72742573059112,48.376546751279,0
-0.72749204467172,48.376462131949,0
-0.72754700308303,48.376373995557,0
-0.72759018760796,48.37628301289,0
-0.72762126964372,48.376189876396,0
-0.72764001270086,48.376095294909,0
-0.72764627420115,48.375999988256,0
-0.72764000656076,48.375904681783,0
-0.72762125755011,48.375810100825,0
-0.72759016992828,48.375716965196,0
-0.72754698035448,48.375625983704,0
-0.72749201758489,48.375537848759,0
-0.727425699969,48.375453231106,0
-0.7273485322642,48.375372774716,0
-0.72726110179293,48.37529709189,0
-0.72716407397208,48.375226758598,0
-0.72705818724801,48.375162310096,0
-0.72694424747655,48.375104236851,0
-0.72682312178997,48.375052980814,0
-0.72669573199824,48.375008932054,0
-0.72656304757431,48.374972425789,0
-0.72642607827716,48.374943739836,0
-0.72628586646836,48.374923092501,0
-0.72614347918084,48.374910640912,0
-0.72599999999999,48.374906479826,0
-0.72585652081917,48.374910640912,0
-0.72571413353162,48.374923092501,0
-0.72557392172282,48.374943739836,0
-0.72543695242567,48.374972425789,0
-0.72530426800177,48.375008932054,0
-0.72517687821002,48.375052980814,0
-0.72505575252346,48.375104236851,0
-0.72494181275197,48.375162310096,0
-0.72483592602792,48.375226758598,0
-0.72473889820705,48.37529709189,0
-0.72465146773581,48.375372774716,0
-0.72457430003099,48.375453231106,0
-0.72450798241511,48.375537848759,0
-0.72445301964553,48.375625983704,0
-0.7244098300717,48.375716965196,0
-0.7243787424499,48.375810100825,0
-0.72435999343925,48.375904681783,0
-0.72435372579883,48.375999988256,0

-0.726,48.376,0
</coordinates>
</LinearRing>
</outerBoundaryIs>
</Polygon>
</Placemark>
<Placemark>
<name>204</name>
<styleUrl>#stylehap1</styleUrl>
<Polygon>
<outerBoundaryIs>
<LinearRing>
<coordinates>
-0.71799999999996,48.165,0
-0.71636050931351,48.164999988343,0
-0.71636674503571,48.165095294995,0
-0.71638541088351,48.16518987648,0
-0.71641636486548,48.165283012971,0
-0.71645937146606,48.165373995633,0
-0.71651410343588,48.16546213202,0
-0.71658014428062,48.165546751344,0
-0.71665699142942,48.165627209582,0
-0.7167440600584,48.165702894378,0
-0.71684068754066,48.165773229702,0
-0.7169461384888,48.165837680236,0
-0.71705961035132,48.165895755451,0
-0.71718023952093,48.165947013336,0
-0.71730710790751,48.165991063767,0
-0.71743924992619,48.166027571474,0
-0.71757565984735,48.166056258596,0
-0.71771529945233,48.166076906793,0
-0.71785710593655,48.166089358911,0
-0.71799999999996,48.166093520174,0
-0.71814289406337,48.166089358911,0
-0.71828470054759,48.166076906793,0
-0.71842434015257,48.166056258596,0
-0.71856075007373,48.166027571474,0
-0.71869289209241,48.165991063767,0
-0.71881976047899,48.165947013336,0
-0.7189403896486,48.165895755451,0
-0.71905386151115,48.165837680236,0
-0.71915931245926,48.165773229702,0
-0.71925593994153,48.165702894378,0
-0.7193430085705,48.165627209582,0
-0.7194198557193,48.165546751344,0
-0.71948589656407,48.16546213202,0
-0.71954062853386,48.165373995633,0
-0.71958363513444,48.165283012971,0
-0.71961458911642,48.16518987648,0
-0.71963325496421,48.165095294995,0
-0.71963949068641,48.164999988343,0
-0.71963324889457,48.164904681869,0
-0.7196145771616,48.164810100909,0
-0.71958361765765,48.164716965277,0
-0.71954060606614,48.16462598378,0
-0.71948586978808,48.16453784883,0
-0.71941982544862,48.164453231171,0

-0.71934297572487,48.164372774774,0
-0.71925590551897,48.164297091941,0
-0.71915927750567,48.164226758642,0
-0.71905382708859,48.164162310131,0
-0.71894035680298,48.16410423688,0
-0.7188197302083,48.164052980836,0
-0.71869286531644,48.16400893207,0
-0.71856072760602,48.163972425799,0
-0.71842432267578,48.163943739842,0
-0.71828468859275,48.163923092504,0
-0.71814288799376,48.163910640912,0
-0.71799999999996,48.163906479827,0
-0.71785711200616,48.163910640912,0
-0.71771531140717,48.163923092504,0
-0.71757567732414,48.163943739842,0
-0.71743927239391,48.163972425799,0
-0.71730713468348,48.16400893207,0
-0.71718026979162,48.164052980836,0
-0.71705964319694,48.16410423688,0
-0.71694617291133,48.164162310131,0
-0.71684072249425,48.164226758642,0
-0.71674409448095,48.164297091941,0
-0.71665702427506,48.164372774774,0
-0.7165801745513,48.164453231171,0
-0.71651413021185,48.16453784883,0
-0.71645939393378,48.16462598378,0
-0.71641638234227,48.164716965277,0
-0.71638542283832,48.164810100909,0
-0.71636675110535,48.164904681869,0
-0.71636050931351,48.164999988343,0
-0.71799999999996,48.165,0
</coordinates>
</LinearRing>
</outerBoundaryIs>
</Polygon>
</Placemark>
<Placemark>
<name>205</name>
<styleUrl>#stylehap1</styleUrl>
<Polygon>
<outerBoundaryIs>
<LinearRing>
<coordinates>
-0.71799999999996,48.068,0
-0.71636360165306,48.067999988383,0
-0.71636982562403,48.068095295034,0
-0.71638845627505,48.068189876518,0
-0.71641935188226,48.068283013008,0
-0.71646227737405,48.068373995668,0
-0.71651690611799,48.068462132053,0
-0.71658282240516,48.068546751374,0
-0.71665952461242,48.068627209609,0
-0.71674642901883,48.068702894401,0
-0.71684287424722,48.068773229721,0
-0.71694812629723,48.068837680252,0
-0.71706138413115,48.068895755464,0
-0.71718178577064,48.068947013345,0

-0.71730841485735,48.068991063774,0
-0.71744030762792,48.069027571479,0
-0.71757646024986,48.069056258599,0
-0.71771583646304,48.069076906795,0
-0.71785737546772,48.069089358911,0
-0.71799999999996,48.069093520173,0
-0.7181426245322,48.069089358911,0
-0.71828416353688,48.069076906795,0
-0.71842353975006,48.069056258599,0
-0.71855969237201,48.069027571479,0
-0.71869158514257,48.068991063774,0
-0.71881821422928,48.068947013345,0
-0.71893861586877,48.068895755464,0
-0.71905187370269,48.068837680252,0
-0.7191571257527,48.068773229721,0
-0.71925357098109,48.068702894401,0
-0.7193404753875,48.068627209609,0
-0.71941717759476,48.068546751374,0
-0.71948309388193,48.068462132053,0
-0.71953772262587,48.068373995668,0
-0.71958064811766,48.068283013008,0
-0.71961154372488,48.068189876518,0
-0.71963017437589,48.068095295034,0
-0.71963639834686,48.067999988383,0
-0.71963016833833,48.067904681908,0
-0.71961153183319,48.067810100948,0
-0.71958063073315,48.067716965314,0
-0.71953770027678,48.067625983815,0
-0.71948306724733,48.067537848863,0
-0.71941714748391,48.0674532312,0
-0.71934044271532,48.067372774801,0
-0.71925353674031,48.067297091964,0
-0.71915709098369,48.067226758661,0
-0.71905183946191,48.067162310148,0
-0.71893858319659,48.067104236893,0
-0.71881818411843,48.067052980846,0
-0.71869155850794,48.067008932077,0
-0.71855967002291,48.066972425803,0
-0.71842352236555,48.066943739845,0
-0.71828415164517,48.066923092505,0
-0.71814261849462,48.066910640913,0
-0.71799999999996,48.066906479826,0
-0.7178573815053,48.066910640913,0
-0.71771584835475,48.066923092505,0
-0.71757647763437,48.066943739845,0
-0.71744032997701,48.066972425803,0
-0.71730844149198,48.067008932077,0
-0.71718181588149,48.067052980846,0
-0.71706141680333,48.067104236893,0
-0.71694816053801,48.067162310148,0
-0.71684290901623,48.067226758661,0
-0.71674646325961,48.067297091964,0
-0.7166595572846,48.067372774801,0
-0.71658285251601,48.0674532312,0
-0.71651693275259,48.067537848863,0
-0.71646229972314,48.067625983815,0
-0.71641936926677,48.067716965314,0

-0.71638846816673,48.067810100948,0
-0.71636983166159,48.067904681908,0
-0.71636360165306,48.067999988383,0
-0.71799999999996,48.068,0
</coordinates>
</LinearRing>
</outerBoundaryIs>
</Polygon>
</Placemark>
<Placemark>
<name>206</name>
<styleUrl>#stylehap1</styleUrl>
<Polygon>
<outerBoundaryIs>
<LinearRing>
<coordinates>
-0.71600000000001,48.231,0
-0.71435839586353,48.230999988316,0
-0.71436463961709,48.231095294968,0
-0.71438332951998,48.231189876454,0
-0.71441432339799,48.231283012946,0
-0.71445738543213,48.231373995609,0
-0.71451218795133,48.231462131998,0
-0.71457831392458,48.231546751324,0
-0.71465526013323,48.231627209564,0
-0.71474244099961,48.231702894362,0
-0.71483919304273,48.231773229688,0
-0.71494477992716,48.231837680225,0
-0.71505839806691,48.231895755442,0
-0.71517918274129,48.231947013329,0
-0.7153062146766,48.231991063762,0
-0.71543852704316,48.232027571471,0
-0.71557511281472,48.232056258594,0
-0.71571493243404,48.232076906793,0
-0.71585692172631,48.23208935891,0
-0.71600000000002,48.232093520174,0
-0.7161430782737,48.23208935891,0
-0.71628506756597,48.232076906793,0
-0.71642488718531,48.232056258594,0
-0.71656147295687,48.232027571471,0
-0.71669378532341,48.231991063762,0
-0.71682081725872,48.231947013329,0
-0.71694160193313,48.231895755442,0
-0.71705522007285,48.231837680225,0
-0.7171608069573,48.231773229688,0
-0.7172575590004,48.231702894362,0
-0.71734473986677,48.231627209564,0
-0.71742168607545,48.231546751324,0
-0.71748781204868,48.231462131998,0
-0.7175426145679,48.231373995609,0
-0.71758567660202,48.231283012946,0
-0.71761667048003,48.231189876454,0
-0.71763536038292,48.231095294968,0
-0.71764160413648,48.230999988316,0
-0.71763535429137,48.230904681842,0
-0.71761665848201,48.230810100883,0
-0.7175856590621,48.230716965252,0

-0.71754259201899,48.230625983757,0
-0.71748778517594,48.230537848808,0
-0.71742165569538,48.23045323115,0
-0.71734470690249,48.230372774756,0
-0.71725752445347,48.230297091925,0
-0.71716077187744,48.230226758628,0
-0.71705518552594,48.23016231012,0
-0.71694156896884,48.230104236871,0
-0.71682078687867,48.230052980829,0
-0.71669375845068,48.230008932065,0
-0.71656145040796,48.229972425796,0
-0.71642486964537,48.22994373984,0
-0.71628505556795,48.229923092503,0
-0.71614307218215,48.229910640912,0
-0.71600000000002,48.229906479827,0
-0.71585692781786,48.229910640912,0
-0.71571494443205,48.229923092503,0
-0.71557513035464,48.22994373984,0
-0.71543854959205,48.229972425796,0
-0.71530624154933,48.230008932065,0
-0.71517921312134,48.230052980829,0
-0.71505843103119,48.230104236871,0
-0.71494481447406,48.23016231012,0
-0.71483922812259,48.230226758628,0
-0.71474247554654,48.230297091925,0
-0.71465529309752,48.230372774756,0
-0.71457834430463,48.23045323115,0
-0.71451221482406,48.230537848808,0
-0.71445740798102,48.230625983757,0
-0.71441434093794,48.230716965252,0
-0.714383341518,48.230810100883,0
-0.71436464570864,48.230904681842,0
-0.71435839586353,48.230999988316,0
-0.71600000000001,48.231,0
</coordinates>
</LinearRing>
</outerBoundaryIs>
</Polygon>
</Placemark>
<Placemark>
<name>207</name>
<styleUrl>#stylehap1</styleUrl>
<Polygon>
<outerBoundaryIs>
<LinearRing>
<coordinates>
-0.71199999999999,48.08,0
-0.71036321998224,48.079999988378,0
-0.7103694454036,48.080095295029,0
-0.71038808039874,48.080189876514,0
-0.71041898321085,48.080283013003,0
-0.71046191871347,48.080373995664,0
-0.710516560198,48.080462132048,0
-0.71058249185865,48.08054675137,0
-0.71065921195529,48.080627209606,0
-0.71074613663081,48.080702894398,0
-0.71084260435381,48.080773229719,0

-0.71094788095274,48.08083768025,0
-0.71106116520308,48.080895755462,0
-0.71118159492539,48.080947013344,0
-0.7113082535476,48.080991063773,0
-0.7114401770815,48.081027571478,0
-0.71157636146053,48.081056258599,0
-0.71171577018271,48.081076906794,0
-0.71185734220096,48.081089358911,0
-0.712,48.081093520173,0
-0.71214265779901,48.081089358911,0
-0.71228422981727,48.081076906794,0
-0.71242363853945,48.081056258599,0
-0.71255982291847,48.081027571478,0
-0.71269174645238,48.080991063773,0
-0.71281840507458,48.080947013344,0
-0.7129388347969,48.080895755462,0
-0.71305211904723,48.08083768025,0
-0.71315739564616,48.080773229719,0
-0.7132538633692,48.080702894398,0
-0.71334078804469,48.080627209606,0
-0.71341750814133,48.08054675137,0
-0.71348343980197,48.080462132048,0
-0.71353808128651,48.080373995664,0
-0.71358101678912,48.080283013003,0
-0.71361191960124,48.080189876514,0
-0.71363055459638,48.080095295029,0
-0.71363678001774,48.079999988378,0
-0.71363054855486,48.079904681903,0
-0.71361190770174,48.079810100943,0
-0.71358099939322,48.079716965309,0
-0.71353805892278,48.079625983811,0
-0.71348341314992,48.079537848859,0
-0.71341747801073,48.079453231197,0
-0.71334075535111,48.079372774797,0
-0.71325382910597,48.079297091962,0
-0.71315736085438,48.079226758659,0
-0.71305208478403,48.079162310146,0
-0.71293880210332,48.079104236891,0
-0.71281837494402,48.079052980845,0
-0.71269171980032,48.079008932076,0
-0.71255980055475,48.078972425803,0
-0.71242362114357,48.078943739844,0
-0.71228421791777,48.078923092505,0
-0.71214265175749,48.078910640913,0
-0.712,48.078906479826,0
-0.71185734824249,48.078910640913,0
-0.71171578208221,48.078923092505,0
-0.71157637885641,48.078943739844,0
-0.71144019944523,48.078972425803,0
-0.71130828019965,48.079008932076,0
-0.71118162505596,48.079052980845,0
-0.71106119789666,48.079104236891,0
-0.71094791521594,48.079162310146,0
-0.7108426391456,48.079226758659,0
-0.71074617089401,48.079297091962,0
-0.71065924464887,48.079372774797,0
-0.71058252198924,48.079453231197,0

-0.71051658685006,48.079537848859,0
-0.7104619410772,48.079625983811,0
-0.71041900060676,48.079716965309,0
-0.71038809229824,48.079810100943,0
-0.71036945144512,48.079904681903,0
-0.71036321998224,48.079999988378,0
-0.71199999999999,48.08,0
</coordinates>
</LinearRing>
</outerBoundaryIs>
</Polygon>
</Placemark>
<Placemark>
<name>208</name>
<styleUrl>#stylehap1</styleUrl>
<Polygon>
<outerBoundaryIs>
<LinearRing>
<coordinates>
-0.71199999999999,48.08,0
-0.71036321998224,48.079999988378,0
-0.7103694454036,48.080095295029,0
-0.71038808039874,48.080189876514,0
-0.71041898321085,48.080283013003,0
-0.71046191871347,48.080373995664,0
-0.710516560198,48.080462132048,0
-0.71058249185865,48.08054675137,0
-0.71065921195529,48.080627209606,0
-0.71074613663081,48.080702894398,0
-0.71084260435381,48.080773229719,0
-0.71094788095274,48.08083768025,0
-0.71106116520308,48.080895755462,0
-0.71118159492539,48.080947013344,0
-0.7113082535476,48.080991063773,0
-0.7114401770815,48.081027571478,0
-0.71157636146053,48.081056258599,0
-0.71171577018271,48.081076906794,0
-0.71185734220096,48.081089358911,0
-0.712,48.081093520173,0
-0.71214265779901,48.081089358911,0
-0.71228422981727,48.081076906794,0
-0.71242363853945,48.081056258599,0
-0.71255982291847,48.081027571478,0
-0.71269174645238,48.080991063773,0
-0.71281840507458,48.080947013344,0
-0.7129388347969,48.080895755462,0
-0.71305211904723,48.08083768025,0
-0.71315739564616,48.080773229719,0
-0.7132538633692,48.080702894398,0
-0.71334078804469,48.080627209606,0
-0.71341750814133,48.08054675137,0
-0.71348343980197,48.080462132048,0
-0.71353808128651,48.080373995664,0
-0.71358101678912,48.080283013003,0
-0.71361191960124,48.080189876514,0
-0.71363055459638,48.080095295029,0
-0.71363678001774,48.079999988378,0

-0.71363054855486,48.079904681903,0
-0.71361190770174,48.079810100943,0
-0.71358099939322,48.079716965309,0
-0.71353805892278,48.079625983811,0
-0.71348341314992,48.079537848859,0
-0.71341747801073,48.079453231197,0
-0.71334075535111,48.079372774797,0
-0.71325382910597,48.079297091962,0
-0.71315736085438,48.079226758659,0
-0.71305208478403,48.079162310146,0
-0.71293880210332,48.079104236891,0
-0.71281837494402,48.079052980845,0
-0.71269171980032,48.079008932076,0
-0.71255980055475,48.078972425803,0
-0.71242362114357,48.078943739844,0
-0.71228421791777,48.078923092505,0
-0.71214265175749,48.078910640913,0
-0.712,48.078906479826,0
-0.71185734824249,48.078910640913,0
-0.71171578208221,48.078923092505,0
-0.71157637885641,48.078943739844,0
-0.71144019944523,48.078972425803,0
-0.71130828019965,48.079008932076,0
-0.71118162505596,48.079052980845,0
-0.71106119789666,48.079104236891,0
-0.71094791521594,48.079162310146,0
-0.7108426391456,48.079226758659,0
-0.71074617089401,48.079297091962,0
-0.71065924464887,48.079372774797,0
-0.71058252198924,48.079453231197,0
-0.71051658685006,48.079537848859,0
-0.7104619410772,48.079625983811,0
-0.71041900060676,48.079716965309,0
-0.71038809229824,48.079810100943,0
-0.71036945144512,48.079904681903,0
-0.71036321998224,48.079999988378,0
-0.71199999999999,48.08,0
</coordinates>
</LinearRing>
</outerBoundaryIs>
</Polygon>
</Placemark>
<Placemark>
<name>209</name>
<styleUrl>#stylehap1</styleUrl>
<Polygon>
<outerBoundaryIs>
<LinearRing>
<coordinates>
-0.71100000000001,47.753,0
-0.70937353182673,47.752999988511,0
-0.70937971806169,47.753095295161,0
-0.7093982356881,47.753189876642,0
-0.70942894384119,47.753283013127,0
-0.70947160887486,47.753373995781,0
-0.70952590613803,47.753462132157,0
-0.70959142244368,47.75354675147,0

-0.70966765921211,47.753627209695,0
-0.70975403626413,47.753702894476,0
-0.7098498962358,47.753773229785,0
-0.70995450958079,47.753837680305,0
-0.71006708012241,47.753895755506,0
-0.71018675111336,47.753947013377,0
-0.71031261175646,47.753991063796,0
-0.71044370413728,47.754027571494,0
-0.7105790305157,47.754056258607,0
-0.71071756092068,47.754076906798,0
-0.71085824099072,47.754089358912,0
-0.71100000000002,47.754093520173,0
-0.71114175900929,47.754089358912,0
-0.71128243907935,47.754076906798,0
-0.71142096948434,47.754056258607,0
-0.71155629586276,47.754027571494,0
-0.71168738824358,47.753991063796,0
-0.71181324888667,47.753947013377,0
-0.71193291987762,47.753895755506,0
-0.71204549041924,47.753837680305,0
-0.71215010376421,47.753773229785,0
-0.71224596373588,47.753702894476,0
-0.71233234078793,47.753627209695,0
-0.71240857755635,47.75354675147,0
-0.712474093862,47.753462132157,0
-0.71252839112515,47.753373995781,0
-0.71257105615885,47.753283013127,0
-0.71260176431193,47.753189876642,0
-0.71262028193835,47.753095295161,0
-0.71262646817328,47.752999988511,0
-0.71262027600335,47.752904682035,0
-0.7126017526223,47.752810101072,0
-0.71257103906975,47.752716965433,0
-0.71252836915587,47.752625983928,0
-0.71247406768001,47.752537848968,0
-0.71240854795719,47.752453231296,0
-0.71233230867094,47.752372774886,0
-0.71224593007696,47.752297092039,0
-0.71215006958604,47.752226758725,0
-0.71204545676032,47.752162310201,0
-0.71193288776063,47.752104236935,0
-0.71181321928749,47.752052980878,0
-0.71168736206158,47.752008932099,0
-0.71155627389344,47.751972425818,0
-0.71142095239524,47.751943739853,0
-0.71128242738972,47.751923092509,0
-0.71114175307432,47.751910640914,0
-0.71100000000002,47.751906479826,0
-0.71085824692571,47.751910640914,0
-0.71071757261031,47.751923092509,0
-0.71057904760479,47.751943739853,0
-0.71044372610659,47.751972425818,0
-0.71031263793845,47.752008932099,0
-0.71018678071252,47.752052980878,0
-0.7100671122394,47.752104236935,0
-0.70995454323971,47.752162310201,0
-0.70984993041399,47.752226758725,0

-0.70975406992307,47.752297092039,0
-0.70966769132909,47.752372774886,0
-0.70959145204284,47.752453231296,0
-0.70952593232003,47.752537848968,0
-0.70947163084417,47.752625983928,0
-0.70942896093028,47.752716965433,0
-0.70939824737773,47.752810101072,0
-0.70937972399665,47.752904682035,0
-0.70937353182673,47.752999988511,0
-0.71100000000001,47.753,0
</coordinates>
</LinearRing>
</outerBoundaryIs>
</Polygon>
</Placemark>
<Placemark>
<name>210</name>
<styleUrl>#stylehap1</styleUrl>
<Polygon>
<outerBoundaryIs>
<LinearRing>
<coordinates>
-0.70900000000006,48.136,0
-0.70736143554384,48.135999988355,0
-0.70736766774626,48.136095295006,0
-0.70738632305176,48.136189876492,0
-0.70741725954911,48.136283012982,0
-0.70746024185565,48.136373995644,0
-0.70751494290681,48.13646213203,0
-0.70758094644356,48.136546751353,0
-0.70765775017889,48.13662720959,0
-0.70774476961924,48.136702894385,0
-0.7078413425121,48.136773229707,0
-0.70794673388544,48.136837680241,0
-0.70806014164124,48.136895755454,0
-0.70818070266011,48.136947013339,0
-0.7083074993706,48.136991063769,0
-0.70843956673344,48.137027571475,0
-0.70857589958729,48.137056258597,0
-0.70871546030009,48.137076906794,0
-0.70885718666771,48.137089358911,0
-0.70900000000005,48.137093520174,0
-0.70914281333241,48.137089358911,0
-0.70928453970003,48.137076906794,0
-0.70942410041283,48.137056258597,0
-0.70956043326668,48.137027571475,0
-0.70969250062952,48.136991063769,0
-0.70981929734001,48.136947013339,0
-0.70993985835888,48.136895755454,0
-0.71005326611468,48.136837680241,0
-0.71015865748802,48.136773229707,0
-0.71025523038085,48.136702894385,0
-0.71034224982123,48.13662720959,0
-0.71041905355656,48.136546751353,0
-0.71048505709331,48.13646213203,0
-0.71053975814447,48.136373995644,0
-0.71058274045101,48.136283012982,0

-0.71061367694836,48.136189876492,0
-0.71063233225386,48.136095295006,0
-0.71063856445628,48.135999988355,0
-0.71063232619383,48.135904681881,0
-0.71061366501245,48.135810100921,0
-0.71058272300188,48.135716965288,0
-0.7105397357123,48.135625983791,0
-0.71048503035968,48.13553784884,0
-0.71041902333375,48.13545323118,0
-0.71034221702758,48.135372774782,0
-0.71025519601277,48.135297091948,0
-0.71015862258976,48.135226758648,0
-0.7100532317466,48.135162310136,0
-0.70993982556523,48.135104236883,0
-0.70981926711723,48.135052980839,0
-0.70969247389592,48.135008932072,0
-0.70956041083451,48.1349724258,0
-0.70942408296369,48.134943739843,0
-0.70928452776412,48.134923092504,0
-0.70914280727238,48.134910640912,0
-0.70900000000005,48.134906479826,0
-0.70885719272774,48.134910640912,0
-0.708715472236,48.134923092504,0
-0.70857591703643,48.134943739843,0
-0.70843958916561,48.1349724258,0
-0.70830752610421,48.135008932072,0
-0.70818073288289,48.135052980839,0
-0.7080601744349,48.135104236883,0
-0.70794676825352,48.135162310136,0
-0.70784137741036,48.135226758648,0
-0.70774480398735,48.135297091948,0
-0.70765778297254,48.135372774782,0
-0.70758097666637,48.13545323118,0
-0.70751496964044,48.13553784884,0
-0.70746026428782,48.135625983791,0
-0.70741727699824,48.135716965288,0
-0.70738633498767,48.135810100921,0
-0.70736767380629,48.135904681881,0
-0.70736143554384,48.135999988355,0
-0.70900000000006,48.136,0
</coordinates>
</LinearRing>
</outerBoundaryIs>
</Polygon>
</Placemark>
<Placemark>
<name>211</name>
<styleUrl>#stylehap1</styleUrl>
<Polygon>
<outerBoundaryIs>
<LinearRing>
<coordinates>
-0.70900000000006,47.807,0
-0.70737184156995,47.806999988489,0
-0.70737803422811,47.807095295139,0
-0.70739657109293,47.807189876621,0
-0.7074273111534,47.807283013107,0

-0.70747002052076,47.807373995762,0
-0.70752437420667,47.80746213214,0
-0.7075899585949,47.807546751453,0
-0.70766627458763,47.80762720968,0
-0.70775274140289,47.807702894463,0
-0.70784870099359,47.807773229774,0
-0.70795342305525,47.807837680296,0
-0.70806611058381,47.807895755498,0
-0.70818590594152,47.807947013372,0
-0.70831189738464,47.807991063792,0
-0.70844312600318,47.808027571491,0
-0.70857859301997,47.808056258606,0
-0.70871726739355,47.808076906798,0
-0.70885809366647,47.808089358912,0
-0.70900000000005,47.808093520174,0
-0.70914190633366,47.808089358912,0
-0.70928273260657,47.808076906798,0
-0.70942140698015,47.808056258606,0
-0.70955687399694,47.808027571491,0
-0.70968810261548,47.807991063792,0
-0.7098140940586,47.807947013372,0
-0.70993388941631,47.807895755498,0
-0.71004657694487,47.807837680296,0
-0.71015129900653,47.807773229774,0
-0.71024725859723,47.807702894463,0
-0.71033372541249,47.80762720968,0
-0.71041004140522,47.807546751453,0
-0.71047562579345,47.80746213214,0
-0.71052997947936,47.807373995762,0
-0.71057268884672,47.807283013107,0
-0.71060342890719,47.807189876621,0
-0.71062196577201,47.807095295139,0
-0.71062815843017,47.806999988489,0
-0.71062195981959,47.806904682013,0
-0.71060341718323,47.806810101051,0
-0.71057267170743,47.806716965413,0
-0.71052995744552,47.806625983909,0
-0.71047559953456,47.80653784895,0
-0.71041001171912,47.80645323128,0
-0.71033369320118,47.806372774872,0
-0.71024722483946,47.806297092027,0
-0.71015126472797,47.806226758714,0
-0.7100465431871,47.806162310192,0
-0.70993385720503,47.806104236927,0
-0.7098140643725,47.806052980872,0
-0.70968807635659,47.806008932096,0
-0.70955685196313,47.805972425816,0
-0.70942138984085,47.805943739852,0
-0.70928272088261,47.805923092508,0
-0.70914190038123,47.805910640913,0
-0.70900000000005,47.805906479826,0
-0.70885809961889,47.805910640913,0
-0.70871727911749,47.805923092508,0
-0.70857861015927,47.805943739852,0
-0.70844314803699,47.805972425816,0
-0.70831192364353,47.806008932096,0
-0.70818593562762,47.806052980872,0

-0.70806614279509,47.806104236927,0
-0.70795345681302,47.806162310192,0
-0.70784873527215,47.806226758714,0
-0.70775277516066,47.806297092027,0
-0.70766630679894,47.806372774872,0
-0.707589988281,47.80645323128,0
-0.70752440046556,47.80653784895,0
-0.7074700425546,47.806625983909,0
-0.70742732829269,47.806716965413,0
-0.70739658281689,47.806810101051,0
-0.70737804018053,47.806904682013,0
-0.70737184156995,47.806999988489,0
-0.70900000000006,47.807,0
</coordinates>
</LinearRing>
</outerBoundaryIs>
</Polygon>
</Placemark>
<Placemark>
<name>212</name>
<styleUrl>#stylehap1</styleUrl>
<Polygon>
<outerBoundaryIs>
<LinearRing>
<coordinates>
-0.70900000000006,47.807,0
-0.70737184156995,47.806999988489,0
-0.70737803422811,47.807095295139,0
-0.70739657109293,47.807189876621,0
-0.7074273111534,47.807283013107,0
-0.70747002052076,47.807373995762,0
-0.70752437420667,47.80746213214,0
-0.7075899585949,47.807546751453,0
-0.70766627458763,47.80762720968,0
-0.70775274140289,47.807702894463,0
-0.70784870099359,47.807773229774,0
-0.70795342305525,47.807837680296,0
-0.70806611058381,47.807895755498,0
-0.70818590594152,47.807947013372,0
-0.70831189738464,47.807991063792,0
-0.70844312600318,47.808027571491,0
-0.70857859301997,47.808056258606,0
-0.70871726739355,47.808076906798,0
-0.70885809366647,47.808089358912,0
-0.70900000000005,47.808093520174,0
-0.70914190633366,47.808089358912,0
-0.70928273260657,47.808076906798,0
-0.70942140698015,47.808056258606,0
-0.70955687399694,47.808027571491,0
-0.70968810261548,47.807991063792,0
-0.7098140940586,47.807947013372,0
-0.70993388941631,47.807895755498,0
-0.71004657694487,47.807837680296,0
-0.71015129900653,47.807773229774,0
-0.71024725859723,47.807702894463,0
-0.71033372541249,47.80762720968,0
-0.71041004140522,47.807546751453,0

-0.71047562579345,47.80746213214,0
-0.71052997947936,47.807373995762,0
-0.71057268884672,47.807283013107,0
-0.71060342890719,47.807189876621,0
-0.71062196577201,47.807095295139,0
-0.71062815843017,47.806999988489,0
-0.71062195981959,47.806904682013,0
-0.71060341718323,47.806810101051,0
-0.71057267170743,47.806716965413,0
-0.71052995744552,47.806625983909,0
-0.71047559953456,47.80653784895,0
-0.71041001171912,47.80645323128,0
-0.71033369320118,47.806372774872,0
-0.71024722483946,47.806297092027,0
-0.71015126472797,47.806226758714,0
-0.7100465431871,47.806162310192,0
-0.70993385720503,47.806104236927,0
-0.7098140643725,47.806052980872,0
-0.70968807635659,47.806008932096,0
-0.70955685196313,47.805972425816,0
-0.70942138984085,47.805943739852,0
-0.70928272088261,47.805923092508,0
-0.70914190038123,47.805910640913,0
-0.70900000000005,47.805906479826,0
-0.70885809961889,47.805910640913,0
-0.70871727911749,47.805923092508,0
-0.70857861015927,47.805943739852,0
-0.70844314803699,47.805972425816,0
-0.70831192364353,47.806008932096,0
-0.70818593562762,47.806052980872,0
-0.70806614279509,47.806104236927,0
-0.70795345681302,47.806162310192,0
-0.70784873527215,47.806226758714,0
-0.70775277516066,47.806297092027,0
-0.70766630679894,47.806372774872,0
-0.707589988281,47.80645323128,0
-0.70752440046556,47.80653784895,0
-0.7074700425546,47.806625983909,0
-0.70742732829269,47.806716965413,0
-0.70739658281689,47.806810101051,0
-0.70737804018053,47.806904682013,0
-0.70737184156995,47.806999988489,0
-0.70900000000006,47.807,0
</coordinates>
</LinearRing>
</outerBoundaryIs>
</Polygon>
</Placemark>
<Placemark>
<name>214</name>
<styleUrl>#stylehap1</styleUrl>
<Polygon>
<outerBoundaryIs>
<LinearRing>
<coordinates>
-0.70699999999999,48.003,0
-0.70536566470277,48.002999988409,0

-0.70537188083388,48.00309529506,0
-0.70539048800342,48.003189876544,0
-0.70542134466602,48.003283013033,0
-0.70546421604616,48.003373995692,0
-0.7055187759231,48.003462132074,0
-0.70558460911187,48.003546751394,0
-0.70566121462159,48.003627209627,0
-0.70574800946715,48.003702894417,0
-0.70584433310518,48.003773229735,0
-0.70594945246059,48.003837680263,0
-0.70606256750564,48.003895755472,0
-0.70618281734876,48.003947013352,0
-0.70630928678697,48.003991063778,0
-0.70644101327203,48.004027571482,0
-0.7065769942372,48.004056258601,0
-0.70671619472892,48.004076906795,0
-0.70685755528505,48.004089358911,0
-0.707,48.004093520173,0
-0.70714244471493,48.004089358911,0
-0.70728380527108,48.004076906795,0
-0.70742300576278,48.004056258601,0
-0.70755898672798,48.004027571482,0
-0.70769071321301,48.003991063778,0
-0.70781718265122,48.003947013352,0
-0.70793743249434,48.003895755472,0
-0.70805054753942,48.003837680263,0
-0.70815566689483,48.003773229735,0
-0.70825199053283,48.003702894417,0
-0.70833878537839,48.003627209627,0
-0.70841539088811,48.003546751394,0
-0.70848122407688,48.003462132074,0
-0.70853578395382,48.003373995692,0
-0.70857865533396,48.003283013033,0
-0.70860951199656,48.003189876544,0
-0.7086281191661,48.00309529506,0
-0.7086343352972,48.002999988409,0
-0.70862811314989,48.002904681934,0
-0.70860950014691,48.002810100973,0
-0.70857863801096,48.002716965339,0
-0.70853576168378,48.002625983839,0
-0.70848119753647,48.002537848885,0
-0.70841536088375,48.00245323122,0
-0.70833875282177,48.002372774818,0
-0.70825195641313,48.00229709198,0
-0.70815563224879,48.002226758675,0
-0.70805051341972,48.002162310159,0
-0.70793739993772,48.002104236901,0
-0.70781715264686,48.002052980853,0
-0.7076906866726,48.002008932081,0
-0.70755896445791,48.001972425806,0
-0.70742298843977,48.001943739846,0
-0.70728379342143,48.001923092506,0
-0.70714243869872,48.001910640913,0
-0.707,48.001906479826,0
-0.70685756130128,48.001910640913,0
-0.70671620657855,48.001923092506,0
-0.70657701156023,48.001943739846,0

-0.70644103554207,48.001972425806,0
-0.70630931332738,48.002008932081,0
-0.70618284735312,48.002052980853,0
-0.70606260006228,48.002104236901,0
-0.70594948658028,48.002162310159,0
-0.70584436775121,48.002226758675,0
-0.70574804358684,48.00229709198,0
-0.70566124717823,48.002372774818,0
-0.70558463911623,48.00245323122,0
-0.70551880246351,48.002537848885,0
-0.7054642383162,48.002625983839,0
-0.70542136198905,48.002716965339,0
-0.70539049985307,48.002810100973,0
-0.70537188685012,48.002904681934,0
-0.70536566470277,48.002999988409,0
-0.70699999999999,48.003,0
</coordinates>
</LinearRing>
</outerBoundaryIs>
</Polygon>
</Placemark>
<Placemark>
<name>215</name>
<styleUrl>#stylehap1</styleUrl>
<Polygon>
<outerBoundaryIs>
<LinearRing>
<coordinates>
-0.70699999999999,47.966,0
-0.70536683579459,47.965999988424,0
-0.70537304747538,47.966095295075,0
-0.70539164131565,47.966189876559,0
-0.70542247587128,47.966283013047,0
-0.7054653165349,47.966373995705,0
-0.70551983731944,47.966462132087,0
-0.70558562333732,47.966546751405,0
-0.70566217395663,47.966627209637,0
-0.70574890660987,47.966702894425,0
-0.70584516122699,47.966773229742,0
-0.70595020525824,47.966837680269,0
-0.7060632392491,47.966895755477,0
-0.70618340292488,47.966947013356,0
-0.70630978173848,47.966991063781,0
-0.70644141383141,47.967027571484,0
-0.70657729735551,47.967056258602,0
-0.70671639809881,47.967076906796,0
-0.70685765735849,47.967089358911,0
-0.707,47.967093520174,0
-0.70714234264149,47.967089358911,0
-0.7072836019012,47.967076906796,0
-0.70742270264449,47.967056258602,0
-0.70755858616856,47.967027571484,0
-0.70769021826153,47.966991063781,0
-0.70781659707512,47.966947013356,0
-0.70793676075091,47.966895755477,0
-0.70804979474176,47.966837680269,0
-0.70815483877299,47.966773229742,0

-0.7082510933901,47.966702894425,0
-0.70833782604338,47.966627209637,0
-0.70841437666265,47.966546751405,0
-0.70848016268054,47.966462132087,0
-0.70853468346508,47.966373995705,0
-0.70857752412872,47.966283013047,0
-0.70860835868436,47.966189876559,0
-0.7086269525246,47.966095295075,0
-0.70863316420539,47.965999988424,0
-0.7086269465205,47.965904681949,0
-0.70860834685857,47.965810100988,0
-0.70857750684058,47.965716965353,0
-0.70853466123987,47.965625983852,0
-0.70848013619357,47.965537848897,0
-0.70841434671872,47.965453231232,0
-0.70833779355228,47.965372774829,0
-0.70825105933911,47.965297091989,0
-0.7081548041967,47.965226758682,0
-0.70804976069077,47.965162310165,0
-0.70793672825981,47.965104236906,0
-0.70781656713116,47.965052980856,0
-0.70769019177455,47.965008932084,0
-0.70755856394336,47.964972425808,0
-0.70742268535635,47.964943739847,0
-0.70728359007541,47.964923092506,0
-0.70714233663739,47.964910640913,0
-0.707,47.964906479826,0
-0.70685766336259,47.964910640913,0
-0.70671640992459,47.964923092506,0
-0.70657731464363,47.964943739847,0
-0.70644143605665,47.964972425808,0
-0.70630980822545,47.965008932084,0
-0.70618343286881,47.965052980856,0
-0.70606327174017,47.965104236906,0
-0.70595023930923,47.965162310165,0
-0.70584519580328,47.965226758682,0
-0.70574894066087,47.965297091989,0
-0.7056622064477,47.965372774829,0
-0.70558565328128,47.965453231232,0
-0.70551986380641,47.965537848897,0
-0.7054653387601,47.965625983852,0
-0.7054224931594,47.965716965353,0
-0.70539165314143,47.965810100988,0
-0.70537305347951,47.965904681949,0
-0.70536683579459,47.965999988424,0
-0.70699999999999,47.966,0
</coordinates>
</LinearRing>
</outerBoundaryIs>
</Polygon>
</Placemark>
<Placemark>
<name>218</name>
<styleUrl>#stylehap1</styleUrl>
<Polygon>
<outerBoundaryIs>
<LinearRing>

<coordinates>
-0.70399999999995,48.009,0
-0.70236547457343,48.008999988407,0
-0.70237169142706,48.009095295058,0
-0.70239030076062,48.009189876542,0
-0.70242116101234,48.00928301303,0
-0.70246403737938,48.009373995689,0
-0.70251860360306,48.009462132072,0
-0.70258444445012,48.009546751392,0
-0.70266105887143,48.009627209625,0
-0.70274786381403,48.009702894415,0
-0.70284419865773,48.009773229733,0
-0.7029493302422,48.009837680262,0
-0.70306245844661,48.009895755471,0
-0.70318272227918,48.009947013351,0
-0.70330920643048,48.009991063778,0
-0.70344094824029,48.010027571482,0
-0.70357694502524,48.010056258601,0
-0.70371616171133,48.010076906795,0
-0.70385753871316,48.010089358911,0
-0.70399999999995,48.010093520174,0
-0.70414246128673,48.010089358911,0
-0.70428383828857,48.010076906795,0
-0.70442305497466,48.010056258601,0
-0.70455905175963,48.010027571482,0
-0.70469079356944,48.009991063778,0
-0.70481727772074,48.009947013351,0
-0.70493754155331,48.009895755471,0
-0.70505066975769,48.009837680262,0
-0.70515580134216,48.009773229733,0
-0.70525213618587,48.009702894415,0
-0.70533894112849,48.009627209625,0
-0.70541555554977,48.009546751392,0
-0.70548139639686,48.009462132072,0
-0.70553596262053,48.009373995689,0
-0.70557883898756,48.00928301303,0
-0.70560969923927,48.009189876542,0
-0.70562830857286,48.009095295058,0
-0.70563452542646,48.008999988407,0
-0.70562830255466,48.008904681932,0
-0.70560968738575,48.008810100971,0
-0.70557882165888,48.008716965336,0
-0.70553594034319,48.008625983837,0
-0.70548136984775,48.008537848883,0
-0.70541552553561,48.008453231219,0
-0.70533890856121,48.008372774817,0
-0.70525210205503,48.008297091979,0
-0.7051557666848,48.008226758674,0
-0.70505063562686,48.008162310158,0
-0.70493750898604,48.0081042369,0
-0.70481724770658,48.008052980852,0
-0.70469076702033,48.008008932081,0
-0.70455902948229,48.007972425806,0
-0.70442303764598,48.007943739846,0
-0.70428382643504,48.007923092506,0
-0.70414245526854,48.007910640913,0
-0.70399999999995,48.007906479826,0

-0.70385754473135,48.007910640913,0
-0.70371617356485,48.007923092506,0
-0.70357696235392,48.007943739846,0
-0.70344097051761,48.007972425806,0
-0.70330923297956,48.008008932081,0
-0.70318275229334,48.008052980852,0
-0.70306249101386,48.0081042369,0
-0.70294936437304,48.008162310158,0
-0.70284423331512,48.008226758674,0
-0.70274789794489,48.008297091979,0
-0.7026610914387,48.008372774817,0
-0.70258447446428,48.008453231219,0
-0.70251863015214,48.008537848883,0
-0.7024640596567,48.008625983837,0
-0.70242117834102,48.008716965336,0
-0.70239031261414,48.008810100971,0
-0.70237169744525,48.008904681932,0
-0.70236547457343,48.008999988407,0
-0.70399999999995,48.009,0
</coordinates>
</LinearRing>
</outerBoundaryIs>
</Polygon>
</Placemark>
<Placemark>
<name>220</name>
<styleUrl>#stylehap1</styleUrl>
<Polygon>
<outerBoundaryIs>
<LinearRing>
<coordinates>
-0.70000000000005,47.973,0
-0.69836661441771,47.972999988422,0
-0.69837282693977,47.973095295072,0
-0.69839142329972,47.973189876556,0
-0.69842226203432,47.973283013044,0
-0.69846510850444,47.973373995702,0
-0.69851963667881,47.973462132084,0
-0.69858543161362,47.973546751403,0
-0.69866199260911,47.973627209635,0
-0.69874873701886,47.973702894424,0
-0.69884500468332,47.973773229741,0
-0.69895006295343,47.973837680268,0
-0.69906311226635,47.973895755476,0
-0.69918329223075,47.973947013355,0
-0.69930968817555,47.973991063781,0
-0.69944133811188,47.974027571483,0
-0.69957724005569,47.974056258602,0
-0.69971635965489,47.974076906796,0
-0.69985763806311,47.974089358911,0
-0.70000000000006,47.974093520174,0
-0.70014236193698,47.974089358911,0
-0.7002836403452,47.974076906796,0
-0.7004227599444,47.974056258602,0
-0.70055866188821,47.974027571483,0
-0.70069031182455,47.973991063781,0
-0.70081670776934,47.973947013355,0

-0.70093688773375,47.973895755476,0
-0.70104993704666,47.973837680268,0
-0.70115499531677,47.973773229741,0
-0.70125126298123,47.973702894424,0
-0.70133800739098,47.973627209635,0
-0.70141456838647,47.973546751403,0
-0.70148036332128,47.973462132084,0
-0.70153489149565,47.973373995702,0
-0.70157773796577,47.973283013044,0
-0.70160857670037,47.973189876556,0
-0.70162717306032,47.973095295072,0
-0.70163338558239,47.972999988422,0
-0.70162716705393,47.972904681947,0
-0.70160856487008,47.972810100985,0
-0.70157772067103,47.97271696535,0
-0.70153486926195,47.97262598385,0
-0.70148033682421,47.972537848895,0
-0.70141453843112,47.97245323123,0
-0.70133797488752,47.972372774827,0
-0.70125122891726,47.972297091987,0
-0.70115496072729,47.972226758681,0
-0.70104990298266,47.972162310164,0
-0.70093685523026,47.972104236905,0
-0.70081667781396,47.972052980856,0
-0.70069028532747,47.972008932084,0
-0.70055863965453,47.971972425808,0
-0.70042274264967,47.971943739847,0
-0.70028362851489,47.971923092506,0
-0.70014235593057,47.971910640913,0
-0.70000000000006,47.971906479827,0
-0.69985764406953,47.971910640913,0
-0.6997163714852,47.971923092506,0
-0.69957725735042,47.971943739847,0
-0.69944136034556,47.971972425808,0
-0.69930971467262,47.972008932084,0
-0.69918332218613,47.972052980856,0
-0.69906314476983,47.972104236905,0
-0.69895009701743,47.972162310164,0
-0.6988450392728,47.972226758681,0
-0.69874877108283,47.972297091987,0
-0.69866202511258,47.972372774827,0
-0.69858546156898,47.97245323123,0
-0.69851966317588,47.972537848895,0
-0.69846513073814,47.97262598385,0
-0.69842227932906,47.97271696535,0
-0.69839143513001,47.972810100985,0
-0.69837283294616,47.972904681947,0
-0.69836661441771,47.972999988422,0
-0.70000000000005,47.973,0
</coordinates>
</LinearRing>
</outerBoundaryIs>
</Polygon>
</Placemark>
<Placemark>
<name>224</name>
<styleUrl>#stylehap1</styleUrl>

<Polygon>
<outerBoundaryIs>
<LinearRing>
<coordinates>
-0.697,48.152,0
-0.69536092470157,48.151999988349,0
-0.69536715884528,48.152095295,0
-0.69538581996514,48.152189876485,0
-0.69541676610575,48.152283012976,0
-0.69545976181116,48.152373995638,0
-0.69551447991482,48.152462132024,0
-0.69558050402801,48.152546751348,0
-0.69565733170708,48.152627209586,0
-0.69574437827637,48.152702894381,0
-0.69584098127681,48.152773229704,0
-0.69594640550732,48.152837680238,0
-0.69605984861982,48.152895755452,0
-0.69618044722575,48.152947013337,0
-0.69630728346761,48.152991063767,0
-0.69643939200527,48.153027571475,0
-0.69657576736393,48.153056258597,0
-0.69671537158799,48.153076906794,0
-0.69685714214219,48.153089358911,0
-0.697,48.153093520174,0
-0.69714285785781,48.153089358911,0
-0.69728462841202,48.153076906794,0
-0.69742423263607,48.153056258597,0
-0.69756060799473,48.153027571475,0
-0.6976927165324,48.152991063767,0
-0.69781955277426,48.152947013337,0
-0.69794015138018,48.152895755452,0
-0.69805359449268,48.152837680238,0
-0.69815901872319,48.152773229704,0
-0.69825562172364,48.152702894381,0
-0.69834266829292,48.152627209586,0
-0.698419495972,48.152546751348,0
-0.69848552008519,48.152462132024,0
-0.69854023818885,48.152373995638,0
-0.69858323389425,48.152283012976,0
-0.69861418003486,48.152189876485,0
-0.69863284115472,48.152095295,0
-0.69863907529843,48.151999988349,0
-0.69863283508941,48.151904681874,0
-0.69861416808852,48.151810100915,0
-0.69858321642985,48.151716965282,0
-0.69854021573709,48.151625983785,0
-0.6984854933282,48.151537848835,0
-0.69841946572281,48.151453231175,0
-0.69834263547062,48.151372774778,0
-0.69825558732553,48.151297091944,0
-0.69815898379442,48.151226758644,0
-0.69805356009455,48.151162310134,0
-0.69794011855788,48.151104236881,0
-0.69781952252507,48.151052980837,0
-0.69769268977541,48.151008932071,0
-0.69756058554297,48.150972425799,0
-0.69742421517167,48.150943739842,0

-0.69728461646567,48.150923092504,0
-0.6971428517925,48.150910640912,0
-0.697,48.150906479827,0
-0.69685714820751,48.150910640912,0
-0.69671538353436,48.150923092504,0
-0.69657578482833,48.150943739842,0
-0.69643941445704,48.150972425799,0
-0.6963073102246,48.151008932071,0
-0.69618047747494,48.151052980837,0
-0.69605988144213,48.151104236881,0
-0.69594643990545,48.151162310134,0
-0.69584101620559,48.151226758644,0
-0.69574441267447,48.151297091944,0
-0.69565736452939,48.151372774778,0
-0.69558053427719,48.151453231175,0
-0.69551450667181,48.151537848835,0
-0.69545978426292,48.151625983785,0
-0.69541678357015,48.151716965282,0
-0.69538583191148,48.151810100915,0
-0.6953671649106,48.151904681874,0
-0.69536092470157,48.151999988349,0
-0.697,48.152,0
</coordinates>
</LinearRing>
</outerBoundaryIs>
</Polygon>
</Placemark>
<Placemark>
<name>227</name>
<styleUrl>#stylehap1</styleUrl>
<Polygon>
<outerBoundaryIs>
<LinearRing>
<coordinates>
-0.69200000000001,48.179,0
-0.69036006164262,48.178999988337,0
-0.69036629906603,48.179095294989,0
-0.69038497000918,48.179189876474,0
-0.69041593244194,48.179283012966,0
-0.69045895078444,48.179373995628,0
-0.69051369769803,48.179462132015,0
-0.69057975657468,48.17954675134,0
-0.69065662470636,48.179627209578,0
-0.69074371710946,48.179702894375,0
-0.6908403709762,48.179773229699,0
-0.69094585071833,48.179837680234,0
-0.69105935356531,48.179895755449,0
-0.69118001567394,48.179947013334,0
-0.6913069187034,48.179991063765,0
-0.69143909680514,48.180027571473,0
-0.69157554397495,48.180056258596,0
-0.69171522171058,48.180076906793,0
-0.69185706691716,48.180089358911,0
-0.692,48.180093520174,0
-0.69214293308284,48.180089358911,0
-0.69228477828945,48.180076906793,0
-0.69242445602505,48.180056258596,0

-0.69256090319486,48.180027571473,0
-0.69269308129663,48.179991063765,0
-0.69281998432606,48.179947013334,0
-0.69294064643469,48.179895755449,0
-0.69305414928167,48.179837680234,0
-0.69315962902383,48.179773229699,0
-0.69325628289055,48.179702894375,0
-0.69334337529367,48.179627209578,0
-0.69342024342532,48.17954675134,0
-0.693486302302,48.179462132015,0
-0.69354104921556,48.179373995628,0
-0.69358406755809,48.179283012966,0
-0.69361502999085,48.179189876474,0
-0.69363370093397,48.179095294989,0
-0.69363993835739,48.178999988337,0
-0.6936336948597,48.178904681863,0
-0.69361501802688,48.178810100904,0
-0.69358405006792,48.178716965272,0
-0.69354102673067,48.178625983775,0
-0.69348627550552,48.178537848826,0
-0.69342021313148,48.178453231166,0
-0.69334334242291,48.17837277477,0
-0.69325624844168,48.178297091938,0
-0.6931595940435,48.178226758639,0
-0.69305411483278,48.178162310129,0
-0.69294061356396,48.178104236878,0
-0.69281995403222,48.178052980835,0
-0.69269305450015,48.178008932069,0
-0.69256088070995,48.177972425798,0
-0.6924244385349,48.177943739842,0
-0.69228476632547,48.177923092503,0
-0.69214292700857,48.177910640912,0
-0.692,48.177906479827,0
-0.69185707299144,48.177910640912,0
-0.69171523367456,48.177923092503,0
-0.6915755614651,48.177943739842,0
-0.69143911929006,48.177972425798,0
-0.69130694549988,48.178008932069,0
-0.69118004596779,48.178052980835,0
-0.69105938643607,48.178104236878,0
-0.69094588516723,48.178162310129,0
-0.6908404059565,48.178226758639,0
-0.69074375155835,48.178297091938,0
-0.69065665757709,48.17837277477,0
-0.69057978686852,48.178453231166,0
-0.69051372449451,48.178537848826,0
-0.69045897326936,48.178625983775,0
-0.69041594993209,48.178716965272,0
-0.69038498197315,48.178810100904,0
-0.69036630514031,48.178904681863,0
-0.69036006164262,48.178999988337,0
-0.69200000000001,48.179,0
</coordinates>
</LinearRing>
</outerBoundaryIs>
</Polygon>
</Placemark>

<Placemark>
<name>228</name>
<styleUrl>#stylehap1</styleUrl>
<Polygon>
<outerBoundaryIs>
<LinearRing>
<coordinates>
-0.68799999999999,48.152,0
-0.68636092470156,48.151999988349,0
-0.68636715884527,48.152095295,0
-0.68638581996513,48.152189876485,0
-0.68641676610574,48.152283012976,0
-0.68645976181114,48.152373995638,0
-0.6865144799148,48.152462132024,0
-0.68658050402799,48.152546751348,0
-0.68665733170707,48.152627209586,0
-0.68674437827635,48.152702894381,0
-0.6868409812768,48.152773229704,0
-0.68694640550731,48.152837680238,0
-0.68705984861981,48.152895755452,0
-0.68718044722573,48.152947013337,0
-0.68730728346759,48.152991063767,0
-0.68743939200526,48.153027571475,0
-0.68757576736392,48.153056258597,0
-0.68771537158798,48.153076906794,0
-0.68785714214218,48.153089358911,0
-0.68799999999999,48.153093520174,0
-0.6881428578578,48.153089358911,0
-0.688284628412,48.153076906794,0
-0.68842423263606,48.153056258597,0
-0.68856060799472,48.153027571475,0
-0.6886927165323 8,48.152991063767,0
-0.68881955277424,48.152947013337,0
-0.68894015138017,48.152895755452,0
-0.68905359449267,48.152837680238,0
-0.68915901872318,48.152773229704,0
-0.68925562172362,48.152702894381,0
-0.68934266829291,48.152627209586,0
-0.68941949597198,48.152546751348,0
-0.68948552008517,48.152462132024,0
-0.68954023818884,48.152373995638,0
-0.68958323389424,48.152283012976,0
-0.68961418003485,48.152189876485,0
-0.68963284115471,48.152095295,0
-0.68963907529842,48.151999988349,0
-0.68963283508939,48.151904681874,0
-0.68961416808851,48.151810100915,0
-0.68958321642984,48.151716965282,0
-0.68954021573707,48.151625983785,0
-0.68948549332819,48.151537848835,0
-0.6894194657228,48.151453231175,0
-0.6893426354706,48.151372774778,0
-0.68925558732552,48.151297091944,0
-0.6891589837944,48.151226758644,0
-0.68905356009454,48.151162310134,0
-0.68894011855786,48.151104236881,0
-0.68881952252505,48.151052980837,0

-0.68869268977539,48.151008932071,0
-0.68856058554295,48.150972425799,0
-0.68842421517166,48.150943739842,0
-0.68828461646566,48.150923092504,0
-0.68814285179248,48.150910640912,0
-0.68799999999999,48.150906479827,0
-0.68785714820749,48.150910640912,0
-0.68771538353434,48.150923092504,0
-0.68757578482832,48.150943739842,0
-0.68743941445702,48.150972425799,0
-0.68730731022458,48.151008932071,0
-0.68718047747492,48.151052980837,0
-0.68705988144211,48.151104236881,0
-0.68694643990544,48.151162310134,0
-0.68684101620557,48.151226758644,0
-0.68674441267446,48.151297091944,0
-0.68665736452937,48.151372774778,0
-0.68658053427718,48.151453231175,0
-0.68651450667179,48.151537848835,0
-0.6864597842629,48.151625983785,0
-0.68641678357014,48.151716965282,0
-0.68638583191147,48.151810100915,0
-0.68636716491058,48.151904681874,0
-0.68636092470156,48.151999988349,0
-0.68799999999999,48.152,0
</coordinates>
</LinearRing>
</outerBoundaryIs>
</Polygon>
</Placemark>
<Placemark>
<name>231</name>
<styleUrl>#stylehap1</styleUrl>
<Polygon>
<outerBoundaryIs>
<LinearRing>
<coordinates>
-0.68499999999995,48.176,0
-0.68336015760087,48.175999988339,0
-0.68336639465962,48.17609529499,0
-0.68338506451056,48.176189876476,0
-0.68341602513191,48.176283012967,0
-0.68345904095753,48.176373995629,0
-0.68351378466791,48.176462132016,0
-0.68357983967945,48.176546751341,0
-0.68365670331342,48.176627209579,0
-0.68374379062055,48.176702894375,0
-0.68384043883177,48.176773229699,0
-0.68394591240191,48.176837680234,0
-0.68405940860736,48.176895755449,0
-0.68418006365552,48.176947013334,0
-0.68430695925927,48.176991063766,0
-0.68443912962661,48.177027571474,0
-0.68457556881216,48.177056258596,0
-0.68471523837448,48.177076906793,0
-0.68485707528093,48.177089358911,0
-0.68499999999996,48.177093520174,0

-0.68514292471896,48.177089358911,0
-0.68528476162541,48.177076906793,0
-0.68542443118773,48.177056258596,0
-0.68556087037328,48.177027571474,0
-0.68569304074064,48.176991063766,0
-0.68581993634437,48.176947013334,0
-0.68594059139253,48.176895755449,0
-0.68605408759797,48.176837680234,0
-0.68615956116812,48.176773229699,0
-0.68625620937934,48.176702894375,0
-0.68634329668647,48.176627209579,0
-0.68642016032044,48.176546751341,0
-0.68648621533198,48.176462132016,0
-0.68654095904236,48.176373995629,0
-0.68658397486798,48.176283012967,0
-0.68661493548933,48.176189876476,0
-0.68663360534027,48.17609529499,0
-0.68663984239902,48.175999988339,0
-0.68663359926699,48.175904681864,0
-0.68661492352731,48.175810100905,0
-0.68658395738068,48.175716965273,0
-0.68654093656113,48.175625983776,0
-0.6864861885399,48.175537848827,0
-0.68642013003156,48.175453231167,0
-0.68634326382111,48.175372774771,0
-0.68625617493612,48.175297091939,0
-0.68615952619355,48.175226758639,0
-0.68605405315473,48.17516231013,0
-0.68594055852717,48.175104236878,0
-0.68581990605549,48.175052980835,0
-0.68569301394854,48.175008932069,0
-0.68556084789205,48.174972425798,0
-0.68542441370043,48.174943739842,0
-0.68528474966339,48.174923092503,0
-0.6851429186457,48.174910640912,0
-0.68499999999996,48.174906479827,0
-0.68485708135419,48.174910640912,0
-0.6847152503365,48.174923092503,0
-0.68457558629946,48.174943739842,0
-0.68443915210784,48.174972425798,0
-0.68430698605135,48.175008932069,0
-0.6841800939444,48.175052980835,0
-0.68405944147272,48.175104236878,0
-0.68394594684516,48.17516231013,0
-0.68384047380634,48.175226758639,0
-0.68374382506377,48.175297091939,0
-0.68365673617878,48.175372774771,0
-0.68357986996833,48.175453231167,0
-0.68351381145999,48.175537848827,0
-0.68345906343875,48.175625983776,0
-0.68341604261921,48.175716965273,0
-0.68338507647258,48.175810100905,0
-0.6833664007329,48.175904681864,0
-0.68336015760087,48.175999988339,0
-0.68499999999995,48.176,0
</coordinates>
</LinearRing>

</outerBoundaryIs>
</Polygon>
</Placemark>
<Placemark>
<name>232</name>
<styleUrl>#stylehap1</styleUrl>
<Polygon>
<outerBoundaryIs>
<LinearRing>
<coordinates>
-0.682999999999999,47.822,0
-0.68137137117357,47.821999988483,0
-0.68137756561929,47.822095295133,0
-0.68139610783816,47.822189876615,0
-0.6814268567784,47.822283013101,0
-0.68146957848378,47.822373995756,0
-0.68152394787206,47.822462132135,0
-0.68158955120756,47.822546751449,0
-0.68166588924833,47.822627209676,0
-0.68175238104459,47.82270289446,0
-0.68184836835913,47.822773229771,0
-0.6819531206765,47.822837680294,0
-0.68206584076236,47.822895755496,0
-0.68218567073119,47.82294701337,0
-0.6823116985758,47.822991063791,0
-0.68244296510911,47.82302757149,0
-0.68257847126541,47.823056258606,0
-0.68271718570522,47.823076906798,0
-0.68285805266623,47.823089358912,0
-0.682999999999999,47.823093520174,0
-0.68314194733374,47.823089358912,0
-0.68328281429475,47.823076906798,0
-0.68342152873457,47.823056258606,0
-0.68355703489089,47.82302757149,0
-0.68368830142421,47.822991063791,0
-0.68381432926878,47.82294701337,0
-0.68393415923762,47.822895755496,0
-0.6840468793235,47.822837680294,0
-0.68415163164087,47.822773229771,0
-0.68424761895541,47.82270289446,0
-0.68433411075164,47.822627209676,0
-0.68441044879241,47.822546751449,0
-0.68447605212791,47.822462132135,0
-0.68453042151622,47.822373995756,0
-0.68457314322157,47.822283013101,0
-0.68460389216184,47.822189876615,0
-0.68462243438068,47.822095295133,0
-0.68462862882641,47.821999988483,0
-0.68462242842343,47.821904682007,0
-0.68460388042831,47.821810101045,0
-0.68457312606833,47.821716965407,0
-0.68453039946442,47.821625983904,0
-0.68447602584763,47.821537848945,0
-0.68441041908211,47.821453231275,0
-0.68433407851407,47.821372774868,0
-0.68424758517009,47.821297092023,0
-0.68415159733436,47.821226758711,0

-0.68404684553817,47.821162310189,0
-0.68393412700005,47.821104236925,0
-0.68381429955848,47.821052980871,0
-0.68368827514389,47.821008932094,0
-0.68355701283909,47.820972425815,0
-0.68342151158133,47.820943739851,0
-0.68328280256123,47.820923092508,0
-0.68314194137649,47.820910640913,0
-0.68299999999999,47.820906479826,0
-0.68285805862349,47.820910640913,0
-0.68271719743875,47.820923092508,0
-0.68257848841865,47.820943739851,0
-0.68244298716089,47.820972425815,0
-0.68231172485608,47.821008932094,0
-0.68218570044149,47.821052980871,0
-0.68206587299993,47.821104236925,0
-0.6819531544618,47.821162310189,0
-0.68184840266564,47.821226758711,0
-0.68175241482989,47.821297092023,0
-0.6816659214859,47.821372774868,0
-0.68158958091786,47.821453231275,0
-0.68152397415237,47.821537848945,0
-0.68146960053556,47.821625983904,0
-0.68142687393164,47.821716965407,0
-0.68139611957166,47.821810101045,0
-0.68137757157657,47.821904682007,0
-0.68137137117357,47.821999988483,0
-0.68299999999999,47.822,0
</coordinates>
</LinearRing>
</outerBoundaryIs>
</Polygon>
</Placemark>
<Placemark>
<name>233</name>
<styleUrl>#stylehap1</styleUrl>
<Polygon>
<outerBoundaryIs>
<LinearRing>
<coordinates>
-0.68200000000002,47.823,0
-0.68037133980021,47.822999988482,0
-0.68037753436517,47.823095295133,0
-0.6803960769411,47.823189876615,0
-0.68042682647359,47.823283013101,0
-0.68046954900186,47.823373995756,0
-0.68052391943743,47.823462132134,0
-0.68058952403663,47.823546751448,0
-0.68066586354792,47.823627209676,0
-0.68075235701027,47.823702894459,0
-0.68084834617389,47.823773229771,0
-0.68095310050918,47.823837680293,0
-0.68106582276647,47.823895755496,0
-0.68118565504372,47.82394701337,0
-0.68131168531613,47.823991063791,0
-0.68144295437819,47.82402757149,0
-0.68157846314493,47.824056258606,0

-0.681717180257,47.824076906798,0
-0.68185804993173,47.824089358912,0
-0.68200000000003,47.824093520174,0
-0.6821419500683,47.824089358912,0
-0.68228281974303,47.824076906798,0
-0.6824215368551,47.824056258606,0
-0.68255704562184,47.82402757149,0
-0.6826883146839,47.823991063791,0
-0.68281434495631,47.82394701337,0
-0.68293417723357,47.823895755496,0
-0.68304689949085,47.823837680293,0
-0.68315165382614,47.823773229771,0
-0.68324764298976,47.823702894459,0
-0.68333413645212,47.823627209676,0
-0.6834104759634,47.823546751448,0
-0.6834760805626,47.823462132134,0
-0.68353045099817,47.823373995756,0
-0.68357317352645,47.823283013101,0
-0.68360392305893,47.823189876615,0
-0.68362246563486,47.823095295133,0
-0.68362866019982,47.822999988482,0
-0.68362245967728,47.822904682007,0
-0.68360391132477,47.822810101044,0
-0.68357315637227,47.822716965407,0
-0.68353042894517,47.822625983903,0
-0.68347605428086,47.822537848945,0
-0.6834104462515,47.822453231275,0
-0.68333410421279,47.822372774867,0
-0.6832476092026,47.822297092023,0
-0.68315161951778,47.822226758711,0
-0.68304686570372,47.822162310189,0
-0.68293414499424,47.822104236925,0
-0.68281431524441,47.822052980871,0
-0.68268828840217,47.822008932094,0
-0.68255702356884,47.821972425815,0
-0.68242151970092,47.821943739851,0
-0.68228280800887,47.821923092508,0
-0.68214194411071,47.821910640913,0
-0.68200000000003,47.821906479826,0
-0.68185805588932,47.821910640913,0
-0.68171719199116,47.821923092508,0
-0.68157848029911,47.821943739851,0
-0.68144297643119,47.821972425815,0
-0.68131171159787,47.822008932094,0
-0.68118568475562,47.822052980871,0
-0.68106585500579,47.822104236925,0
-0.68095313429631,47.822162310189,0
-0.68084838048225,47.822226758711,0
-0.68075239079743,47.822297092023,0
-0.68066589578724,47.822372774867,0
-0.68058955374854,47.822453231275,0
-0.68052394571917,47.822537848945,0
-0.68046957105486,47.822625983903,0
-0.68042684362777,47.822716965407,0
-0.68039608867526,47.822810101044,0
-0.68037754032275,47.822904682007,0
-0.68037133980021,47.822999988482,0

-0.68200000000002,47.823,0
</coordinates>
</LinearRing>
</outerBoundaryIs>
</Polygon>
</Placemark>
<Placemark>
<name>235</name>
<styleUrl>#stylehap1</styleUrl>
<Polygon>
<outerBoundaryIs>
<LinearRing>
<coordinates>
-0.67899999999997,48.175,0
-0.6773601895835,48.174999988339,0
-0.67736642652073,48.175095294991,0
-0.67738509600764,48.175189876476,0
-0.67741605602524,48.175283012967,0
-0.677459071012,48.17537399563,0
-0.67751381365476,48.175462132017,0
-0.67757986737805,48.175546751341,0
-0.67765672951296,48.175627209579,0
-0.6777438151216,48.175702894375,0
-0.67784046144787,48.175773229699,0
-0.67794593296091,48.175837680234,0
-0.67805942695277,48.175895755449,0
-0.67818007964769,48.175947013335,0
-0.67830697277647,48.175991063766,0
-0.67843914056596,48.176027571474,0
-0.67857557709037,48.176056258596,0
-0.67871524392856,48.176076906793,0
-0.67885707806859,48.176089358911,0
-0.67899999999997,48.176093520173,0
-0.67914292193136,48.176089358911,0
-0.67928475607138,48.176076906793,0
-0.67942442290957,48.176056258596,0
-0.67956085943399,48.176027571474,0
-0.6796930272235,48.175991063766,0
-0.67981992035228,48.175947013335,0
-0.67994057304718,48.175895755449,0
-0.68005406703904,48.175837680234,0
-0.68015953855208,48.175773229699,0
-0.68025618487834,48.175702894375,0
-0.68034327048698,48.175627209579,0
-0.68042013262189,48.175546751341,0
-0.68048618634518,48.175462132017,0
-0.68054092898794,48.17537399563,0
-0.68058394397471,48.175283012967,0
-0.68061490399231,48.175189876476,0
-0.68063357347922,48.175095294991,0
-0.68063981041644,48.174999988339,0
-0.68063356740627,48.174904681865,0
-0.68061489203093,48.174810100905,0
-0.68058392648838,48.174716965273,0
-0.68054090650794,48.174625983777,0
-0.68048615955456,48.174537848827,0
-0.68042010233467,48.174453231168,0

-0.6803432376234,48.174372774771,0
-0.68025615043698,48.174297091939,0
-0.68015950357941,48.17422675864,0
-0.68005403259768,48.17416231013,0
-0.67994054018363,48.174104236878,0
-0.67981989006505,48.174052980835,0
-0.67969300043288,48.174008932069,0
-0.67956083695401,48.173972425798,0
-0.67942440542324,48.173943739842,0
-0.67928474411003,48.173923092503,0
-0.67914291585843,48.173910640912,0
-0.67899999999997,48.173906479827,0
-0.67885708414152,48.173910640912,0
-0.67871525588991,48.173923092503,0
-0.67857559457671,48.173943739842,0
-0.67843916304594,48.173972425798,0
-0.67830699956707,48.174008932069,0
-0.67818010993489,48.174052980835,0
-0.67805945981632,48.174104236878,0
-0.67794596740227,48.17416231013,0
-0.67784049642053,48.17422675864,0
-0.67774384956296,48.174297091939,0
-0.67765676237654,48.174372774771,0
-0.67757989766528,48.174453231168,0
-0.67751384044539,48.174537848827,0
-0.67745909349201,48.174625983777,0
-0.67741607351157,48.174716965273,0
-0.67738510796902,48.174810100905,0
-0.67736643259368,48.174904681865,0
-0.6773601895835,48.174999988339,0
-0.67899999999997,48.175,0
</coordinates>
</LinearRing>
</outerBoundaryIs>
</Polygon>
</Placemark>
<Placemark>
<name>236</name>
<styleUrl>#stylehap1</styleUrl>
<Polygon>
<outerBoundaryIs>
<LinearRing>
<coordinates>
-0.67899999999997,48.202,0
-0.67735932543949,48.201999988328,0
-0.67736556566055,48.20209529498,0
-0.67738424498308,48.202189876465,0
-0.67741522131332,48.202283012957,0
-0.67745825896566,48.20237399562,0
-0.67751303045455,48.202462132008,0
-0.67757911898504,48.202546751333,0
-0.67765602162337,48.202627209572,0
-0.67774315312347,48.202702894369,0
-0.67783985037996,48.202773229694,0
-0.67794537747441,48.20283768023,0
-0.67805893127586,48.202895755446,0
-0.67817964755331,48.202947013332,0

-0.67830660755363,48.202991063764,0
-0.67843884499469,48.203027571472,0
-0.67857535342054,48.203056258595,0
-0.67871509386269,48.203076906793,0
-0.67885700274898,48.20308935891,0
-0.67899999999997,48.203093520174,0
-0.67914299725096,48.20308935891,0
-0.67928490613726,48.203076906793,0
-0.67942464657941,48.203056258595,0
-0.67956115500525,48.203027571472,0
-0.67969339244632,48.202991063764,0
-0.67982035244664,48.202947013332,0
-0.67994106872408,48.202895755446,0
-0.68005462252553,48.20283768023,0
-0.68016014961999,48.202773229694,0
-0.68025684687648,48.202702894369,0
-0.68034397837657,48.202627209572,0
-0.68042088101491,48.202546751333,0
-0.6804869695454,48.202462132008,0
-0.68054174103429,48.20237399562,0
-0.68058477868663,48.202283012957,0
-0.68061575501686,48.202189876465,0
-0.68063443433939,48.20209529498,0
-0.68064067456045,48.201999988328,0
-0.68063442825749,48.201904681854,0
-0.68061574303782,48.201810100895,0
-0.68058476117447,48.201716965263,0
-0.6805417185211,48.201625983767,0
-0.68048694271523,48.201537848818,0
-0.68042085068298,48.201453231159,0
-0.6803439454645,48.201372774764,0
-0.68025681238428,48.201297091932,0
-0.6801601145957,48.201226758634,0
-0.68005458803333,48.201162310125,0
-0.67994103581201,48.201104236875,0
-0.6798203221147,48.201052980832,0
-0.67969336561612,48.201008932067,0
-0.67956113249206,48.200972425797,0
-0.67942462906725,48.200943739841,0
-0.67928489415825,48.200923092503,0
-0.67914299116905,48.200910640912,0
-0.67899999999997,48.200906479826,0
-0.67885700883089,48.200910640912,0
-0.6787151058417,48.200923092503,0
-0.6785753709327,48.200943739841,0
-0.67843886750788,48.200972425797,0
-0.67830663438382,48.201008932067,0
-0.67817967788524,48.201052980832,0
-0.67805896418794,48.201104236875,0
-0.67794541196661,48.201162310125,0
-0.67783988540428,48.201226758634,0
-0.67774318761567,48.201297091932,0
-0.67765605453545,48.201372774764,0
-0.67757914931697,48.201453231159,0
-0.67751305728471,48.201537848818,0
-0.67745828147884,48.201625983767,0
-0.67741523882548,48.201716965263,0

-0.67738425696212,48.201810100895,0
-0.67736557174246,48.201904681854,0
-0.67735932543949,48.201999988328,0
-0.67899999999997,48.202,0
</coordinates>
</LinearRing>
</outerBoundaryIs>
</Polygon>
</Placemark>
<Placemark>
<name>237</name>
<styleUrl>#stylehap1</styleUrl>
<Polygon>
<outerBoundaryIs>
<LinearRing>
<coordinates>
-0.67700000000002,48.032,0
-0.67536474516834,48.031999988398,0
-0.67537096479378,48.032095295049,0
-0.6753895824294,48.032189876533,0
-0.67542045645025,48.032283013022,0
-0.67546335194882,48.032373995681,0
-0.67551794252088,48.032462132065,0
-0.67558381274795,48.032546751385,0
-0.67566046135732,48.032627209619,0
-0.67574730503592,48.03270289441,0
-0.67584368286871,48.032773229729,0
-0.67594886136824,48.032837680258,0
-0.67606204005663,48.032895755468,0
-0.67618235755783,48.032947013349,0
-0.67630889815395,48.032991063776,0
-0.67644069875512,48.03302757148,0
-0.67657675623049,48.0330562586,0
-0.67671603504409,48.033076906795,0
-0.67685747513762,48.033089358911,0
-0.67700000000003,48.033093520174,0
-0.67714252486244,48.033089358911,0
-0.67728396495597,48.033076906795,0
-0.67742324376956,48.0330562586,0
-0.67755930124491,48.03302757148,0
-0.67769110184611,48.032991063776,0
-0.67781764244223,48.032947013349,0
-0.67793795994343,48.032895755468,0
-0.67805113863179,48.032837680258,0
-0.67815631713132,48.032773229729,0
-0.67825269496412,48.03270289441,0
-0.67833953864272,48.032627209619,0
-0.67841618725208,48.032546751385,0
-0.67848205747915,48.032462132065,0
-0.67853664805124,48.032373995681,0
-0.67857954354981,48.032283013022,0
-0.67861041757066,48.032189876533,0
-0.67862903520625,48.032095295049,0
-0.67863525483172,48.031999988398,0
-0.67862902918053,48.031904681923,0
-0.67861040570228,48.031810100962,0
-0.67857952619938,48.031716965328,0

-0.67853662574596,48.031625983828,0
-0.67848203089678,48.031537848875,0
-0.67841615720027,48.031453231211,0
-0.6783395060346,48.031372774811,0
-0.67825266079048,48.031297091973,0
-0.67815628243048,48.031226758669,0
-0.67805110445813,48.031162310154,0
-0.67793792733532,48.031104236897,0
-0.67781761239041,48.03105298085,0
-0.67769107526372,48.031008932079,0
-0.67755927893966,48.030972425805,0
-0.67742322641913,48.030943739846,0
-0.67728395308756,48.030923092505,0
-0.67714251883669,48.030910640913,0
-0.67700000000003,48.030906479826,0
-0.67685748116334,48.030910640913,0
-0.67671604691247,48.030923092505,0
-0.6765767735809,48.030943739846,0
-0.6764407210604,48.030972425805,0
-0.67630892473634,48.031008932079,0
-0.67618238760965,48.03105298085,0
-0.67606207266474,48.031104236897,0
-0.6759488955419,48.031162310154,0
-0.67584371756956,48.031226758669,0
-0.67574733920958,48.031297091973,0
-0.67566049396546,48.031372774811,0
-0.67558384279976,48.031453231211,0
-0.67551796910327,48.031537848875,0
-0.67546337425407,48.031625983828,0
-0.67542047380066,48.031716965328,0
-0.67538959429778,48.031810100962,0
-0.67537097081953,48.031904681923,0
-0.67536474516834,48.031999988398,0
-0.67700000000002,48.032,0
</coordinates>
</LinearRing>
</outerBoundaryIs>
</Polygon>
</Placemark>
<Placemark>
<name>238</name>
<styleUrl>#stylehap1</styleUrl>
<Polygon>
<outerBoundaryIs>
<LinearRing>
<coordinates>
-0.67599999999993,48.201,0
-0.67435935746752,48.200999988328,0
-0.67436559756688,48.20109529498,0
-0.67438427652487,48.201189876466,0
-0.67441525225049,48.201283012957,0
-0.67445828906276,48.20137399562,0
-0.67451305948252,48.201462132008,0
-0.67457914672293,48.201546751333,0
-0.67465604786009,48.201627209572,0
-0.67474317765931,48.201702894369,0
-0.67483987302815,48.201773229694,0

-0.67494539806257,48.20183768023,0
-0.67505894964728,48.201895755446,0
-0.67517966356813,48.201947013332,0
-0.67530662108999,48.201991063764,0
-0.67543885594951,48.202027571472,0
-0.67557536171046,48.202056258595,0
-0.67571509942458,48.202076906793,0
-0.67585700554054,48.20208935891,0
-0.67599999999994,48.202093520174,0
-0.67614299445932,48.20208935891,0
-0.67628490057528,48.202076906793,0
-0.6764246382894,48.202056258595,0
-0.67656114405035,48.202027571472,0
-0.67669337890987,48.201991063764,0
-0.67682033643173,48.201947013332,0
-0.67694105035261,48.201895755446,0
-0.67705460193729,48.20183768023,0
-0.67716012697171,48.201773229694,0
-0.67725682234055,48.201702894369,0
-0.67734395213977,48.201627209572,0
-0.67742085327693,48.201546751333,0
-0.67748694051734,48.201462132008,0
-0.6775417109371,48.20137399562,0
-0.67758474774937,48.201283012957,0
-0.67761572347499,48.201189876466,0
-0.67763440243298,48.20109529498,0
-0.67764064253234,48.200999988328,0
-0.6776343963514,48.200904681854,0
-0.67761571149661,48.200810100895,0
-0.67758473023818,48.200716965263,0
-0.6775416884251,48.200625983767,0
-0.67748691368865,48.200537848818,0
-0.67742082294665,48.20045323116,0
-0.67734391922947,48.200372774764,0
-0.67725678785024,48.200297091933,0
-0.67716009194933,48.200226758634,0
-0.67705456744697,48.200162310125,0
-0.67694101744231,48.200104236875,0
-0.67682030610145,48.200052980832,0
-0.67669335208118,48.200008932067,0
-0.67656112153841,48.199972425797,0
-0.67642462077821,48.199943739841,0
-0.6762848885969,48.199923092503,0
-0.67614298837774,48.199910640912,0
-0.67599999999994,48.199906479826,0
-0.67585701162212,48.199910640912,0
-0.67571511140296,48.199923092503,0
-0.67557537922165,48.199943739841,0
-0.67543887846148,48.199972425797,0
-0.67530664791868,48.200008932067,0
-0.67517969389841,48.200052980832,0
-0.67505898255755,48.200104236875,0
-0.67494543255289,48.200162310125,0
-0.67483990805053,48.200226758634,0
-0.67474321214962,48.200297091933,0
-0.67465608077039,48.200372774764,0
-0.67457917705321,48.20045323116,0

-0.67451308631121,48.200537848818,0
-0.67445831157472,48.200625983767,0
-0.67441526976168,48.200716965263,0
-0.67438428850325,48.200810100895,0
-0.67436560364846,48.200904681854,0
-0.67435935746752,48.200999988328,0
-0.67599999999993,48.201,0
</coordinates>
</LinearRing>
</outerBoundaryIs>
</Polygon>
</Placemark>
<Placemark>
<name>239</name>
<styleUrl>#stylehap1</styleUrl>
<Polygon>
<outerBoundaryIs>
<LinearRing>
<coordinates>
-0.67399999999998,48.033,0
-0.67236471343426,48.032999988397,0
-0.67237093318031,48.033095295048,0
-0.67238955117711,48.033189876532,0
-0.67242042579702,48.033283013021,0
-0.67246332212793,48.033373995681,0
-0.67251791375933,48.033462132064,0
-0.67258378526462,48.033546751384,0
-0.67266043536142,48.033627209618,0
-0.67274728072528,48.03370289441,0
-0.67284366042839,48.033773229729,0
-0.67294884096905,48.033837680258,0
-0.6730620218538,48.033895755468,0
-0.67318234168996,48.033947013349,0
-0.6733088847418,48.033991063776,0
-0.6734406879008,48.03402757148,0
-0.67357674801662,48.0340562586,0
-0.67371602953318,48.034076906795,0
-0.6738574723716,48.034089358911,0
-0.67399999999997,48.034093520174,0
-0.67414252762835,48.034089358911,0
-0.6742839704668,48.034076906795,0
-0.67442325198336,48.0340562586,0
-0.67455931209915,48.03402757148,0
-0.67469111525814,48.033991063776,0
-0.67481765831001,48.033947013349,0
-0.67493797814615,48.033895755468,0
-0.6750511590309,48.033837680258,0
-0.67515633957155,48.033773229729,0
-0.67525271927467,48.03370289441,0
-0.67533956463856,48.033627209618,0
-0.67541621473533,48.033546751384,0
-0.67548208624062,48.033462132064,0
-0.67553667787202,48.033373995681,0
-0.67557957420295,48.033283013021,0
-0.67561044882283,48.033189876532,0
-0.67562906681964,48.033095295048,0
-0.67563528656569,48.032999988397,0

-0.67562906079358,48.032904681922,0
-0.67561043695379,48.032810100962,0
-0.67557955685157,48.032716965327,0
-0.67553665556554,48.032625983828,0
-0.67548205965678,48.032537848875,0
-0.67541618468188,48.032453231211,0
-0.67533953202863,48.03237277481,0
-0.67525268509915,48.032297091973,0
-0.67515630486883,48.032226758669,0
-0.67505112485538,48.032162310154,0
-0.67493794553625,48.032104236897,0
-0.67481762825654,48.03205298085,0
-0.67469108867433,48.032008932079,0
-0.67455928979267,48.031972425805,0
-0.67442323463198,48.031943739846,0
-0.67428395859776,48.031923092505,0
-0.67414252160229,48.031910640913,0
-0.67399999999997,48.031906479827,0
-0.67385747839768,48.031910640913,0
-0.67371604140219,48.031923092505,0
-0.67357676536797,48.031943739846,0
-0.67344071020727,48.031972425805,0
-0.67330891132564,48.032008932079,0
-0.67318237174341,48.03205298085,0
-0.6730620544637,48.032104236897,0
-0.67294887514457,48.032162310154,0
-0.67284369513112,48.032226758669,0
-0.6727473149008,48.032297091973,0
-0.67266046797131,48.03237277481,0
-0.67258381531806,48.032453231211,0
-0.67251794034317,48.032537848875,0
-0.67246334443443,48.032625983828,0
-0.67242044314837,48.032716965327,0
-0.67238956304616,48.032810100962,0
-0.67237093920639,48.032904681922,0
-0.67236471343426,48.032999988397,0
-0.67399999999998,48.033,0
</coordinates>
</LinearRing>
</outerBoundaryIs>
</Polygon>
</Placemark>
<Placemark>
<name>240</name>
<styleUrl>#stylehap1</styleUrl>
<Polygon>
<outerBoundaryIs>
<LinearRing>
<coordinates>
-0.673,48.203,0
-0.67135929340969,48.202999988328,0
-0.67136553375248,48.203095294979,0
-0.67138421343958,48.203189876465,0
-0.67141519037442,48.203283012957,0
-0.67145822886689,48.203373995619,0
-0.67151300142495,48.203462132007,0
-0.67157909124558,48.203546751332,0

-0.67165599538521,48.203627209572,0
-0.67174312858629,48.203702894369,0
-0.67183982773053,48.203773229694,0
-0.67194535688512,48.20383768023,0
-0.67205891290344,48.203895755445,0
-0.6721796315376,48.203947013332,0
-0.67230659401654,48.203991063764,0
-0.67243883403925,48.204027571472,0
-0.67257534513017,48.204056258595,0
-0.67271508830047,48.204076906793,0
-0.67285699995726,48.20408935891,0
-0.67300000000001,48.204093520174,0
-0.67314300004274,48.20408935891,0
-0.67328491169953,48.204076906793,0
-0.67342465486983,48.204056258595,0
-0.67356116596075,48.204027571472,0
-0.67369340598346,48.203991063764,0
-0.6738203684624,48.203947013332,0
-0.67394108709656,48.203895755445,0
-0.67405464311488,48.20383768023,0
-0.67416017226947,48.203773229694,0
-0.67425687141371,48.203702894369,0
-0.67434400461479,48.203627209572,0
-0.67442090875442,48.203546751332,0
-0.67448699857505,48.203462132007,0
-0.67454177113312,48.203373995619,0
-0.67458480962558,48.203283012957,0
-0.67461578656042,48.203189876465,0
-0.67463446624752,48.203095294979,0
-0.67464070659031,48.202999988328,0
-0.67463446016528,48.202904681853,0
-0.67461577458075,48.202810100894,0
-0.67458479211245,48.202716965263,0
-0.67454174861871,48.202625983767,0
-0.67448697174341,48.202537848818,0
-0.67442087842084,48.202453231159,0
-0.67434397170091,48.202372774764,0
-0.67425683691963,48.202297091932,0
-0.67416013724327,48.202226758634,0
-0.6740546086208,48.202162310125,0
-0.67394105418268,48.202104236874,0
-0.67382033812881,48.202052980832,0
-0.67369337915182,48.202008932067,0
-0.67356114344632,48.201972425797,0
-0.67342463735673,48.201943739841,0
-0.67328489971985,48.201923092503,0
-0.6731429939605,48.201910640912,0
-0.67300000000001,48.201906479826,0
-0.6728570060395,48.201910640912,0
-0.67271510028015,48.201923092503,0
-0.67257536264327,48.201943739841,0
-0.67243885655369,48.201972425797,0
-0.67230662084818,48.202008932067,0
-0.67217966187119,48.202052980832,0
-0.67205894581733,48.202104236874,0
-0.6719453913792,48.202162310125,0
-0.67183986275673,48.202226758634,0

-0.67174316308038,48.202297091932,0
-0.67165602829909,48.202372774764,0
-0.67157912157919,48.202453231159,0
-0.67151302825659,48.202537848818,0
-0.6714582513813,48.202625983767,0
-0.67141520788755,48.202716965263,0
-0.67138422541925,48.202810100894,0
-0.67136553983472,48.202904681853,0
-0.67135929340969,48.202999988328,0
-0.673,48.203,0
</coordinates>
</LinearRing>
</outerBoundaryIs>
</Polygon>
</Placemark>
<Placemark>
<name>242</name>
<styleUrl>#stylehap1</styleUrl>
<Polygon>
<outerBoundaryIs>
<LinearRing>
<coordinates>
-0.66999999999996,48.199,0
-0.66835942151844,48.198999988329,0
-0.66836566137439,48.199095294981,0
-0.66838433960338,48.199189876467,0
-0.6684153141199,48.199283012958,0
-0.66845834925217,48.199373995621,0
-0.66851311753384,48.199462132009,0
-0.66857920219434,48.199546751334,0
-0.66865610032934,48.199627209573,0
-0.66874322672704,48.19970289437,0
-0.66883991832092,48.199773229695,0
-0.6689454392356,48.19983768023,0
-0.66905898638718,48.199895755446,0
-0.66917969559527,48.199947013332,0
-0.66930664816054,48.199991063764,0
-0.66943887785747,48.200027571472,0
-0.66957537828902,48.200056258595,0
-0.66971511054758,48.200076906793,0
-0.66985701112332,48.20008935891,0
-0.66999999999996,48.200093520173,0
-0.6701429888766,48.20008935891,0
-0.67028488945233,48.200076906793,0
-0.67042462171089,48.200056258595,0
-0.67056112214245,48.200027571472,0
-0.67069335183937,48.199991063764,0
-0.67082030440465,48.199947013332,0
-0.67094101361274,48.199895755446,0
-0.67105456076431,48.19983768023,0
-0.671160081679,48.199773229695,0
-0.67125677327288,48.19970289437,0
-0.67134389967058,48.199627209573,0
-0.6714207978056,48.199546751334,0
-0.67148688246607,48.199462132009,0
-0.67154165074774,48.199373995621,0
-0.67158468588001,48.199283012958,0

-0.67161566039654,48.199189876467,0
-0.67163433862552,48.199095294981,0
-0.67164057848148,48.198999988329,0
-0.67163433254461,48.198904681855,0
-0.67161564841949,48.198810100896,0
-0.67158466837076,48.198716965264,0
-0.67154162823824,48.198625983768,0
-0.67148685564031,48.198537848819,0
-0.67142076747863,48.19845323116,0
-0.6713438667639,48.198372774765,0
-0.67125673878633,48.198297091933,0
-0.67116004666046,48.198226758635,0
-0.67105452627776,48.198162310126,0
-0.67094098070606,48.198104236875,0
-0.67082027407768,48.198052980833,0
-0.67069332501361,48.198008932067,0
-0.67056109963295,48.197972425797,0
-0.67042460420161,48.197943739841,0
-0.67028487747528,48.197923092503,0
-0.67014298279571,48.197910640912,0
-0.66999999999996,48.197906479826,0
-0.66985701720421,48.197910640912,0
-0.66971512252464,48.197923092503,0
-0.66957539579831,48.197943739841,0
-0.66943890036697,48.197972425797,0
-0.66930667498633,48.198008932067,0
-0.66917972592224,48.198052980833,0
-0.66905901929386,48.198104236875,0
-0.66894547372216,48.198162310126,0
-0.66883995333946,48.198226758635,0
-0.66874326121359,48.198297091933,0
-0.66865613323602,48.198372774765,0
-0.66857923252128,48.19845323116,0
-0.66851314435961,48.198537848819,0
-0.66845837176167,48.198625983768,0
-0.66841533162916,48.198716965264,0
-0.66838435158043,48.198810100896,0
-0.66836566745531,48.198904681855,0
-0.66835942151844,48.198999988329,0
-0.66999999999996,48.199,0
</coordinates>
</LinearRing>
</outerBoundaryIs>
</Polygon>
</Placemark>
<Placemark>
<name>243</name>
<styleUrl>#stylehap1</styleUrl>
<Polygon>
<outerBoundaryIs>
<LinearRing>
<coordinates>
-0.66700000000003,48.486,0
-0.66535015816292,48.485999988211,0
-0.6653564332206,48.486095294863,0
-0.66537521688418,48.486189876352,0
-0.66540636626686,48.486283012848,0

-0.66544964436707,48.486373995516,0
-0.66550472187013,48.486462131911,0
-0.66557117965292,48.486546751245,0
-0.66564851197211,48.486627209494,0
-0.66573613031187,48.4867028943,0
-0.66583336786203,48.486773229635,0
-0.6659394845923,48.486837680181,0
-0.6660536728841,48.486895755407,0
-0.66617506367732,48.486947013302,0
-0.6663027330849,48.486991063743,0
-0.66643570942502,48.487027571459,0
-0.66657298061757,48.487056258587,0
-0.66671350188798,48.487076906789,0
-0.66685620372058,48.48708935891,0
-0.66700000000003,48.487093520174,0
-0.66714379627948,48.48708935891,0
-0.66728649811208,48.487076906789,0
-0.66742701938249,48.487056258587,0
-0.66756429057504,48.487027571459,0
-0.66769726691516,48.486991063743,0
-0.66782493632273,48.486947013302,0
-0.66794632711596,48.486895755407,0
-0.66806051540776,48.486837680181,0
-0.66816663213803,48.486773229635,0
-0.66826386968819,48.4867028943,0
-0.66835148802795,48.486627209494,0
-0.66842882034714,48.486546751245,0
-0.66849527812993,48.486462131911,0
-0.66855035563298,48.486373995516,0
-0.6685936337332,48.486283012848,0
-0.66862478311588,48.486189876352,0
-0.66864356677946,48.486095294863,0
-0.66864984183714,48.485999988211,0
-0.66864356060224,48.485904681738,0
-0.66862477094909,48.485810100781,0
-0.66859361594654,48.485716965154,0
-0.66855033276691,48.485625983664,0
-0.66849525087921,48.485537848722,0
-0.66842878953975,48.485453231072,0
-0.66835145459999,48.485372774685,0
-0.66826383465532,48.485297091864,0
-0.66816659656472,48.485226758576,0
-0.6680604803749,48.485162310077,0
-0.66794629368797,48.485104236836,0
-0.66782490551535,48.485052980803,0
-0.66769723966443,48.485008932046,0
-0.66756426770896,48.484972425783,0
-0.66742700159584,48.484943739833,0
-0.66728648594529,48.4849230925,0
-0.66714379010223,48.484910640911,0
-0.66700000000003,48.484906479826,0
-0.66685620989783,48.484910640911,0
-0.66671351405477,48.4849230925,0
-0.66657299840422,48.484943739833,0
-0.66643573229112,48.484972425783,0
-0.66630276033563,48.485008932046,0
-0.66617509448471,48.485052980803,0

-0.66605370631209,48.485104236836,0
-0.66593951962516,48.485162310077,0
-0.66583340343534,48.485226758576,0
-0.66573616534474,48.485297091864,0
-0.66564854540007,48.485372774685,0
-0.66557121046031,48.485453231072,0
-0.66550474912085,48.485537848722,0
-0.66544966723315,48.485625983664,0
-0.66540638405352,48.485716965154,0
-0.66537522905097,48.485810100781,0
-0.66535643939782,48.485904681738,0
-0.66535015816292,48.485999988211,0
-0.66700000000003,48.486,0
</coordinates>
</LinearRing>
</outerBoundaryIs>
</Polygon>
</Placemark>
<Placemark>
<name>244</name>
<styleUrl>#stylehap1</styleUrl>
<Polygon>
<outerBoundaryIs>
<LinearRing>
<coordinates>
-0.66399999999999,48.419,0
-0.66235233373881,48.418999988239,0
-0.66235860052907,48.419095294891,0
-0.66237735943048,48.419189876379,0
-0.66240846774442,48.419283012874,0
-0.66245168878162,48.419373995541,0
-0.66250669366147,48.419462131934,0
-0.66257306381341,48.419546751266,0
-0.66265029416083,48.419627209512,0
-0.66273779696395,48.419702894317,0
-0.66283490629175,48.419773229649,0
-0.66294088308977,48.419837680193,0
-0.66305492080447,48.419895755416,0
-0.66317615152175,48.419947013309,0
-0.66330365257294,48.419991063748,0
-0.66343645355772,48.420027571462,0
-0.66357354373062,48.420056258589,0
-0.66371387969505,48.42007690679,0
-0.6638563933458,48.42008935891,0
-0.664,48.420093520173,0
-0.66414360665417,48.42008935891,0
-0.66428612030492,48.42007690679,0
-0.66442645626935,48.420056258589,0
-0.66456354644226,48.420027571462,0
-0.66469634742703,48.419991063748,0
-0.66482384847822,48.419947013309,0
-0.6649450791955,48.419895755416,0
-0.66505911691021,48.419837680193,0
-0.66516509370822,48.419773229649,0
-0.66526220303603,48.419702894317,0
-0.66534970583914,48.419627209512,0
-0.66542693618657,48.419546751266,0

-0.66549330633851,48.419462131934,0
-0.66554831121836,48.419373995541,0
-0.66559153225555,48.419283012874,0
-0.66562264056949,48.419189876379,0
-0.6656413994709,48.419095294891,0
-0.66564766626117,48.418999988239,0
-0.66564139331632,48.418904681765,0
-0.66562262844733,48.418810100808,0
-0.66559151453414,48.41871696518,0
-0.66554828843617,48.418625983688,0
-0.66549327918774,48.418537848745,0
-0.6654269054922,48.418453231092,0
-0.6653496725338,48.418372774704,0
-0.66526216813168,48.41829709188,0
-0.66516505826542,48.418226758589,0
-0.66505908200586,48.418162310088,0
-0.66494504589016,48.418104236845,0
-0.66482381778386,48.41805298081,0
-0.66469632027626,48.418008932051,0
-0.66456352366007,48.417972425786,0
-0.66442643854794,48.417943739835,0
-0.66428610818276,48.4179230925,0
-0.66414360049959,48.417910640912,0
-0.664,48.417906479826,0
-0.66385639950038,48.417910640912,0
-0.66371389181721,48.4179230925,0
-0.66357356145204,48.417943739835,0
-0.6634364763399,48.417972425786,0
-0.66330367972371,48.418008932051,0
-0.66317618221611,48.41805298081,0
-0.66305495410982,48.418104236845,0
-0.66294091799412,48.418162310088,0
-0.66283494173455,48.418226758589,0
-0.66273783186829,48.41829709188,0
-0.66265032746617,48.418372774704,0
-0.66257309450777,48.418453231092,0
-0.66250672081223,48.418537848745,0
-0.6624517115638,48.418625983688,0
-0.66240848546584,48.41871696518,0
-0.66237737155265,48.418810100808,0
-0.66235860668365,48.418904681765,0
-0.66235233373881,48.418999988239,0
-0.66399999999999,48.419,0
</coordinates>
</LinearRing>
</outerBoundaryIs>
</Polygon>
</Placemark>
<Placemark>
<name>245</name>
<styleUrl>#stylehap1</styleUrl>
<Polygon>
<outerBoundaryIs>
<LinearRing>
<coordinates>
-0.66200000000003,48.116,0
-0.66036207346938,48.115999988363,0

-0.66036830324761,48.116095295015,0
-0.66038695129229,48.116189876499,0
-0.66041787574739,48.11628301299,0
-0.6604608413218,48.116373995651,0
-0.66051552107827,48.116462132036,0
-0.66058149891979,48.116546751359,0
-0.66065827275479,48.116627209596,0
-0.66074525831733,48.11670289439,0
-0.66084179361262,48.116773229712,0
-0.66094714395482,48.116837680244,0
-0.66106050755819,48.116895755457,0
-0.66118102163935,48.116947013341,0
-0.66130776898414,48.11699106377,0
-0.66143978492906,48.117027571476,0
-0.66157606470409,48.117056258598,0
-0.66171557108119,48.117076906794,0
-0.66185724226987,48.117089358911,0
-0.66200000000003,48.117093520174,0
-0.66214275773019,48.117089358911,0
-0.66228442891887,48.117076906794,0
-0.66242393529599,48.117056258598,0
-0.66256021507102,48.117027571476,0
-0.66269223101592,48.11699106377,0
-0.66281897836071,48.116947013341,0
-0.66293949244187,48.116895755457,0
-0.66305285604527,48.116837680244,0
-0.66315820638747,48.116773229712,0
-0.66325474168276,48.11670289439,0
-0.66334172724527,48.116627209596,0
-0.66341850108027,48.116546751359,0
-0.66348447892179,48.116462132036,0
-0.66353915867826,48.116373995651,0
-0.66358212425269,48.11628301299,0
-0.66361304870777,48.116189876499,0
-0.66363169675245,48.116095295015,0
-0.66363792653071,48.115999988363,0
-0.66363169069904,48.115904681889,0
-0.66361303678489,48.115810100929,0
-0.66358210682259,48.115716965296,0
-0.66353913627057,48.115625983798,0
-0.66348445221734,48.115537848847,0
-0.66341847089047,48.115453231186,0
-0.66334169448741,48.115372774788,0
-0.66325470735216,48.115297091953,0
-0.66315817152727,48.115226758652,0
-0.66305282171466,48.11516231014,0
-0.66293945968402,48.115104236886,0
-0.66281894817091,48.115052980841,0
-0.66269220431147,48.115008932073,0
-0.66256019266333,48.114972425801,0
-0.66242391786589,48.114943739843,0
-0.66228441699599,48.114923092504,0
-0.66214275167678,48.114910640913,0
-0.66200000000003,48.114906479827,0
-0.66185724832328,48.114910640913,0
-0.66171558300407,48.114923092504,0
-0.66157608213419,48.114943739843,0

-0.66143980733676,48.114972425801,0
-0.66130779568861,48.115008932073,0
-0.66118105182917,48.115052980841,0
-0.66106054031604,48.115104236886,0
-0.6609471782854,48.11516231014,0
-0.66084182847279,48.115226758652,0
-0.6607452926479,48.115297091953,0
-0.66065830551265,48.115372774788,0
-0.66058152910959,48.115453231186,0
-0.66051554778275,48.115537848847,0
-0.66046086372949,48.115625983798,0
-0.66041789317747,48.115716965296,0
-0.66038696321519,48.115810100929,0
-0.66036830930102,48.115904681889,0
-0.66036207346938,48.115999988363,0
-0.66200000000003,48.116,0
</coordinates>
</LinearRing>
</outerBoundaryIs>
</Polygon>
</Placemark>
<Placemark>
<name>246</name>
<styleUrl>#stylehap1</styleUrl>
<Polygon>
<outerBoundaryIs>
<LinearRing>
<coordinates>
-0.65999999999997,48.276,0
-0.65835695049673,48.275999988298,0
-0.65836319974285,48.276095294949,0
-0.65838190609677,48.276189876436,0
-0.65841292725926,48.276283012929,0
-0.65845602720383,48.276373995593,0
-0.65851087797102,48.276462131983,0
-0.65857706216278,48.27654675131,0
-0.65865407611745,48.276627209552,0
-0.65874133374183,48.276702894351,0
-0.65883817097084,48.276773229679,0
-0.65894385082072,48.276837680217,0
-0.65905756899792,48.276895755436,0
-0.65917846002043,48.276947013324,0
-0.65930560380506,48.276991063758,0
-0.65943803267079,48.277027571469,0
-0.65957473870462,48.277056258593,0
-0.65971468143392,48.277076906792,0
-0.65985679574677,48.27708935891,0
-0.65999999999996,48.277093520174,0
-0.66014320425318,48.27708935891,0
-0.66028531856603,48.277076906792,0
-0.66042526129533,48.277056258593,0
-0.66056196732916,48.277027571469,0
-0.66069439619488,48.276991063758,0
-0.66082153997948,48.276947013324,0
-0.660942431002,48.276895755436,0
-0.66105614917922,48.276837680217,0
-0.6611618290291,48.276773229679,0

-0.66125866625809,48.276702894351,0
-0.6613459238825,48.276627209552,0
-0.66142293783717,48.27654675131,0
-0.6614891220289,48.276462131983,0
-0.66154397279611,48.276373995593,0
-0.66158707274066,48.276283012929,0
-0.66161809390318,48.276189876436,0
-0.66163680025709,48.276095294949,0
-0.66164304950321,48.275999988298,0
-0.66163679415053,48.275904681824,0
-0.66161808187559,48.275810100865,0
-0.66158705515751,48.275716965235,0
-0.66154395019166,48.27562598374,0
-0.66148909508995,48.275537848793,0
-0.66142290738226,48.275453231137,0
-0.66134589083699,48.275372774744,0
-0.66125863162605,48.275297091914,0
-0.6611617938628,48.275226758619,0
-0.66105611454718,48.275162310113,0
-0.66094239795649,48.275104236865,0
-0.66082150952458,48.275052980825,0
-0.66069436925592,48.275008932061,0
-0.66056194472468,48.274972425793,0
-0.66042524371218,48.274943739839,0
-0.66028530653844,48.274923092502,0
-0.66014319814661,48.274910640912,0
-0.65999999999996,48.274906479826,0
-0.65985680185333,48.274910640912,0
-0.6597146934615,48.274923092502,0
-0.65957475628777,48.274943739839,0
-0.65943805527524,48.274972425793,0
-0.65930563074403,48.275008932061,0
-0.65917849047536,48.275052980825,0
-0.65905760204345,48.275104236865,0
-0.65894388545276,48.275162310113,0
-0.65883820613714,48.275226758619,0
-0.6587413683739,48.275297091914,0
-0.65865410916295,48.275372774744,0
-0.65857709261768,48.275453231137,0
-0.65851090490999,48.275537848793,0
-0.65845604980829,48.27562598374,0
-0.65841294484241,48.275716965235,0
-0.65838191812435,48.275810100865,0
-0.65836320584942,48.275904681824,0
-0.65835695049673,48.275999988298,0
-0.65999999999997,48.276,0
</coordinates>
</LinearRing>
</outerBoundaryIs>
</Polygon>
</Placemark>
<Placemark>
<name>247</name>
<styleUrl>#stylehap1</styleUrl>
<Polygon>
<outerBoundaryIs>
<LinearRing>

<coordinates>
-0.65599999999995,48.283,0
-0.65435672534197,48.282999988295,0
-0.65436297544369,48.283095294947,0
-0.65438168436031,48.283189876433,0
-0.65441270977309,48.283283012926,0
-0.65445581562322,48.28337399559,0
-0.65451067390634,48.28346213198,0
-0.65457686716718,48.283546751308,0
-0.65465389167513,48.28362720955,0
-0.65474116125665,48.283702894349,0
-0.65483801175562,48.283773229677,0
-0.65494370608738,48.283837680216,0
-0.65505743984812,48.283895755435,0
-0.6551783474372,48.283947013323,0
-0.65530550864537,48.283991063758,0
-0.65543795565898,48.284027571468,0
-0.65557468042689,48.284056258593,0
-0.65571464233389,48.284076906792,0
-0.65585677612204,48.28408935891,0
-0.65599999999994,48.284093520174,0
-0.65614322387787,48.28408935891,0
-0.65628535766602,48.284076906792,0
-0.656425319573,48.284056258593,0
-0.65656204434093,48.284027571468,0
-0.65669449135452,48.283991063758,0
-0.65682165256268,48.283947013323,0
-0.65694256015176,48.283895755435,0
-0.65705629391254,48.283837680216,0
-0.65716198824427,48.283773229677,0
-0.65725883874324,48.283702894349,0
-0.65734610832478,48.28362720955,0
-0.65742313283271,48.283546751308,0
-0.65748932609355,48.28346213198,0
-0.65754418437667,48.28337399559,0
-0.6575872902268,48.283283012926,0
-0.6576183156396,48.283189876433,0
-0.6576370245562,48.283095294947,0
-0.65764327465795,48.282999988295,0
-0.6576370184473,48.282904681821,0
-0.65761830360741,48.282810100862,0
-0.65758727263693,48.282716965232,0
-0.65754416176356,48.282625983738,0
-0.65748929914428,48.282537848791,0
-0.65742310236613,48.282453231134,0
-0.6573460752666,48.282372774742,0
-0.65725880409794,48.282297091913,0
-0.65716195306451,48.282226758617,0
-0.65705625926721,48.282162310111,0
-0.65694252709359,48.282104236864,0
-0.6568216220961,48.282052980824,0
-0.65669446440525,48.282008932061,0
-0.6565620217278,48.281972425793,0
-0.65642530198313,48.281943739839,0
-0.65628534563383,48.281923092502,0
-0.65614321776897,48.281910640912,0
-0.65599999999994,48.281906479827,0

-0.65585678223095,48.281910640912,0
-0.65571465436608,48.281923092502,0
-0.65557469801678,48.281943739839,0
-0.65543797827209,48.281972425793,0
-0.65530553559464,48.282008932061,0
-0.65517837790379,48.282052980824,0
-0.6550574729063,48.282104236864,0
-0.65494374073268,48.282162310111,0
-0.6548380469354,48.282226758617,0
-0.65474119590194,48.282297091913,0
-0.65465392473328,48.282372774742,0
-0.65457689763376,48.282453231134,0
-0.65451070085561,48.282537848791,0
-0.65445583823635,48.282625983738,0
-0.65441272736298,48.282716965232,0
-0.6543816963925,48.282810100862,0
-0.65436298155259,48.282904681821,0
-0.65435672534197,48.282999988295,0
-0.65599999999995,48.283,0
</coordinates>
</LinearRing>
</outerBoundaryIs>
</Polygon>
</Placemark>
<Placemark>
<name>249</name>
<styleUrl>#stylehap1</styleUrl>
<Polygon>
<outerBoundaryIs>
<LinearRing>
<coordinates>
-0.654,48.167,0
-0.6523604453815,48.166999988342,0
-0.65236668134667,48.167095294994,0
-0.65238534792212,48.167189876479,0
-0.65241630311095,48.16728301297,0
-0.6524593113884,48.167373995632,0
-0.65251404549234,48.167462132019,0
-0.65258008891224,48.167546751343,0
-0.6526569390576,48.167627209582,0
-0.65274401108179,48.167702894377,0
-0.65284064233202,48.167773229701,0
-0.65294609739223,48.167837680236,0
-0.65305957367967,48.16789575545,0
-0.65318020755332,48.167947013335,0
-0.65330708088724,48.167991063766,0
-0.65343922805895,48.168027571474,0
-0.6535756432996,48.168056258596,0
-0.65371528835005,48.168076906793,0
-0.6538571003642,48.168089358911,0
-0.654,48.168093520174,0
-0.6541428996358,48.168089358911,0
-0.65428471164995,48.168076906793,0
-0.65442435670038,48.168056258596,0
-0.65456077194105,48.168027571474,0
-0.65469291911276,48.167991063766,0
-0.65481979244668,48.167947013335,0

-0.65494042632033,48.16789575545,0
-0.65505390260777,48.167837680236,0
-0.65515935766798,48.167773229701,0
-0.65525598891821,48.167702894377,0
-0.65534306094237,48.167627209582,0
-0.65541991108776,48.167546751343,0
-0.65548595450766,48.167462132019,0
-0.6555406886116,48.167373995632,0
-0.65558369688902,48.16728301297,0
-0.65561465207788,48.167189876479,0
-0.65563331865333,48.167095294994,0
-0.65563955461848,48.166999988342,0
-0.65563331258303,48.166904681868,0
-0.65561464012174,48.166810100909,0
-0.65558367941035,48.166716965276,0
-0.65554066614141,48.16662598378,0
-0.65548592772874,48.16653784883,0
-0.65541988081377,48.16645323117,0
-0.65534302809317,48.166372774774,0
-0.65525595449189,48.166297091941,0
-0.65515932271058,48.166226758641,0
-0.65505386818145,48.166162310131,0
-0.65494039347113,48.166104236879,0
-0.65481976217269,48.166052980836,0
-0.65469289233384,48.166008932069,0
-0.65456074947087,48.165972425799,0
-0.65442433922168,48.165943739842,0
-0.65428469969382,48.165923092504,0
-0.65414289356552,48.165910640912,0
-0.654,48.165906479826,0
-0.65385710643448,48.165910640912,0
-0.65371530030618,48.165923092504,0
-0.6535756607783,48.165943739842,0
-0.65343925052913,48.165972425799,0
-0.65330710766613,48.166008932069,0
-0.65318023782729,48.166052980836,0
-0.65305960652887,48.166104236879,0
-0.65294613181853,48.166162310131,0
-0.65284067728942,48.166226758641,0
-0.65274404550808,48.166297091941,0
-0.65265697190683,48.166372774774,0
-0.65258011918623,48.16645323117,0
-0.65251407227126,48.16653784883,0
-0.65245933385859,48.16662598378,0
-0.65241632058965,48.166716965276,0
-0.65238535987826,48.166810100909,0
-0.65236668741695,48.166904681868,0
-0.6523604453815,48.166999988342,0
-0.654,48.167,0
</coordinates>
</LinearRing>
</outerBoundaryIs>
</Polygon>
</Placemark>
<Placemark>
<name>252</name>
<styleUrl>#stylehap1</styleUrl>

<Polygon>
<outerBoundaryIs>
<LinearRing>
<coordinates>
-0.65100000000007,48.391,0
-0.64935324056914,48.39099998825,0
-0.64935950391336,48.391095294902,0
-0.64937825249334,48.39118987639,0
-0.64940934368887,48.391283012884,0
-0.64945254094086,48.391373995551,0
-0.64950751554969,48.391462131944,0
-0.64957384917505,48.391546751274,0
-0.6496510370183,48.39162720952,0
-0.64973849166306,48.391702894323,0
-0.64983554754482,48.391773229655,0
-0.64994146601587,48.391837680198,0
-0.65005544096655,48.39189575542,0
-0.6501766049605,48.391947013312,0
-0.65030403583688,48.39199106375,0
-0.65043676372948,48.392027571463,0
-0.65057377844924,48.39205625859,0
-0.65071403717385,48.392076906791,0
-0.65085647238607,48.39208935891,0
-0.65100000000007,48.392093520174,0
-0.65114352761407,48.39208935891,0
-0.65128596282629,48.392076906791,0
-0.6514262215509,48.39205625859,0
-0.65156323627064,48.392027571463,0
-0.65169596416326,48.39199106375,0
-0.65182339503964,48.391947013312,0
-0.65194455903359,48.39189575542,0
-0.65205853398427,48.391837680198,0
-0.65216445245532,48.391773229655,0
-0.65226150833708,48.391702894323,0
-0.65234896298182,48.39162720952,0
-0.65242615082507,48.391546751274,0
-0.65249248445045,48.391462131944,0
-0.65254745905928,48.391373995551,0
-0.65259065631127,48.391283012884,0
-0.6526217475068,48.39118987639,0
-0.65264049608678,48.391095294902,0
-0.65264675943101,48.39099998825,0
-0.65264048994164,48.390904681777,0
-0.65262173540324,48.390810100819,0
-0.65259063861706,48.39071696519,0
-0.65254743631203,48.390625983698,0
-0.65249245734134,48.390537848754,0
-0.65242612017778,48.390453231101,0
-0.65234892972756,48.390372774712,0
-0.65226147348627,48.390297091887,0
-0.65216441706687,48.390226758595,0
-0.65205849913346,48.390162310093,0
-0.65194452577931,48.390104236849,0
-0.65182336439235,48.390052980813,0
-0.65169593705412,48.390008932053,0
-0.65156321352339,48.389972425788,0
-0.65142620385669,48.389943739836,0

-0.65128595072273,48.389923092501,0
-0.65114352146894,48.389910640912,0
-0.65100000000007,48.389906479827,0
-0.65085647853121,48.389910640912,0
-0.65071404927741,48.389923092501,0
-0.65057379614346,48.389943739836,0
-0.65043678647673,48.389972425788,0
-0.65030406294599,48.390008932053,0
-0.65017663560779,48.390052980813,0
-0.6500554742208,48.390104236849,0
-0.64994150086668,48.390162310093,0
-0.64983558293328,48.390226758595,0
-0.64973852651387,48.390297091887,0
-0.64965107027258,48.390372774712,0
-0.64957387982234,48.390453231101,0
-0.6495075426588,48.390537848754,0
-0.64945256368811,48.390625983698,0
-0.64940936138309,48.39071696519,0
-0.64937826459688,48.390810100819,0
-0.6493595100585,48.390904681777,0
-0.64935324056914,48.39099998825,0
-0.65100000000007,48.391,0
</coordinates>
</LinearRing>
</outerBoundaryIs>
</Polygon>
</Placemark>
<Placemark>
<name>253</name>
<styleUrl>#stylehap1</styleUrl>
<Polygon>
<outerBoundaryIs>
<LinearRing>
<coordinates>
-0.65100000000007,48.389,0
-0.64935330528948,48.388999988251,0
-0.64935956838776,48.389095294903,0
-0.6493783162311,48.389189876391,0
-0.64940940620489,48.389283012885,0
-0.64945260175935,48.389373995552,0
-0.64950757420772,48.389462131944,0
-0.64957390522619,48.389546751275,0
-0.64965109003594,48.38962720952,0
-0.64973854124363,48.389702894324,0
-0.64983559331097,48.389773229655,0
-0.6499415076192,48.389837680198,0
-0.65005547809044,48.38989575542,0
-0.65017663732237,48.389947013313,0
-0.65030406319036,48.38999106375,0
-0.65043678586639,48.390027571463,0
-0.65057379520107,48.39005625859,0
-0.65071404841309,48.390076906791,0
-0.65085647802715,48.39008935891,0
-0.65100000000007,48.390093520174,0
-0.65114352197299,48.39008935891,0
-0.65128595158705,48.390076906791,0
-0.65142620479907,48.39005625859,0

-0.65156321413373,48.390027571463,0
-0.65169593680976,48.38999106375,0
-0.65182336267778,48.389947013313,0
-0.6519445219097,48.38989575542,0
-0.65205849238091,48.389837680198,0
-0.65216440668917,48.389773229655,0
-0.65226145875651,48.389702894324,0
-0.6523489099641 8,48.38962720952,0
-0.65242609477392,48.389546751275,0
-0.6524924257924,48.389462131944,0
-0.65254739824079,48.389373995552,0
-0.65259059379526,48.389283012885,0
-0.65262168376905,48.389189876391,0
-0.65264043161236,48.389095294903,0
-0.65264669471064,48.388999988251,0
-0.65264042546791,48.388904681777,0
-0.65262167166681,48.38881010082,0
-0.65259057610298,48.388716965191,0
-0.65254737549604,48.388625983699,0
-0.65249239868626,48.388537848755,0
-0.65242606412999,48.388453231102,0
-0.65234887671357,48.388372774712,0
-0.65226142390951,48.388297091887,0
-0.65216437130461,48.388226758596,0
-0.65205845753392,48.388162310093,0
-0.65194448865909,48.388104236849,0
-0.65182333203385,48.388052980813,0
-0.65169590970362,48.388008932053,0
-0.65156319138897,48.387972425788,0
-0.65142618710679,48.387943739836,0
-0.65128593948481,48.387923092501,0
-0.65114351582852,48.387910640912,0
-0.65100000000007,48.387906479826,0
-0.65085648417163,48.387910640912,0
-0.6507140605153,48.387923092501,0
-0.65057381289335,48.387943739836,0
-0.65043680861115,48.387972425788,0
-0.65030409029652,48.388008932053,0
-0.6501766679663,48.388052980813,0
-0.65005551134106,48.388104236849,0
-0.6499415424662,48.388162310093,0
-0.64983562869553,48.388226758596,0
-0.64973857609063,48.388297091887,0
-0.64965112328655,48.388372774712,0
-0.64957393587013,48.388453231102,0
-0.64950760131388,48.388537848755,0
-0.64945262450411,48.388625983699,0
-0.64940942389717,48.388716965191,0
-0.64937832833334,48.38881010082,0
-0.64935957453224,48.388904681777,0
-0.64935330528948,48.388999988251,0
-0.65100000000007,48.389,0
</coordinates>
</LinearRing>
</outerBoundaryIs>
</Polygon>
</Placemark>

<Placemark>
<name>254</name>
<styleUrl>#stylehap1</styleUrl>
<Polygon>
<outerBoundaryIs>
<LinearRing>
<coordinates>
-0.64999999999998,47.963,0
-0.64836693064456,47.962999988426,0
-0.64837314196494,47.963095295076,0
-0.6483917347256,47.96318987656,0
-0.64842256749073,47.963283013048,0
-0.64846540566654,47.963373995706,0
-0.64851992328488,47.963462132088,0
-0.64858570548228,47.963546751406,0
-0.64866225165583,47.963627209638,0
-0.64874897927194,47.963702894426,0
-0.64884522829889,47.963773229743,0
-0.6489502662294,47.96383768027,0
-0.64906329365544,47.963895755478,0
-0.64918345035231,47.963947013356,0
-0.64930982182597,47.963991063781,0
-0.64944144627384,47.964027571484,0
-0.64957732190589,47.964056258602,0
-0.64971641457027,47.964076906796,0
-0.64985766562567,47.964089358911,0
-0.64999999999999,47.964093520174,0
-0.65014233437427,47.964089358911,0
-0.65028358542967,47.964076906796,0
-0.65042267809408,47.964056258602,0
-0.65055855372613,47.964027571484,0
-0.650690178174,47.963991063781,0
-0.65081654964764,47.963947013356,0
-0.6509367063445,47.963895755478,0
-0.65104973377057,47.96383768027,0
-0.65115477170108,47.963773229743,0
-0.651251020728,47.963702894426,0
-0.65133774834414,47.963627209638,0
-0.65141429451769,47.963546751406,0
-0.65148007671507,47.963462132088,0
-0.6515345943334,47.963373995706,0
-0.65157743250922,47.963283013048,0
-0.65160826527435,47.96318987656,0
-0.65162685803503,47.963095295076,0
-0.65163306935538,47.962999988426,0
-0.6516268520319,47.962904681951,0
-0.6516082534505,47.962810100989,0
-0.65157741522392,47.962716965354,0
-0.65153457211184,47.962625983853,0
-0.65148005023242,47.962537848898,0
-0.65141426457864,47.962453231233,0
-0.65133771585836,47.962372774829,0
-0.65125098668258,47.962297091989,0
-0.65115473713044,47.962226758683,0
-0.65104969972512,47.962162310165,0
-0.65093667385872,47.962104236907,0
-0.65081651970859,47.962052980857,0

-0.65069015169136,47.962008932084,0
-0.65055853150453,47.961972425808,0
-0.65042266080876,47.961943739848,0
-0.65028357360582,47.961923092506,0
-0.65014232837114,47.961910640913,0
-0.64999999999999,47.961906479826,0
-0.64985767162881,47.961910640913,0
-0.64971642639412,47.961923092506,0
-0.64957733919119,47.961943739848,0
-0.64944146849541,47.961972425808,0
-0.64930984830861,47.962008932084,0
-0.64918348029135,47.962052980857,0
-0.64906332614122,47.962104236907,0
-0.64895030027483,47.962162310165,0
-0.64884526286953,47.962226758683,0
-0.64874901331739,47.962297091989,0
-0.64866228414161,47.962372774829,0
-0.64858573542133,47.962453231233,0
-0.64851994976753,47.962537848898,0
-0.64846542788813,47.962625983853,0
-0.64842258477605,47.962716965354,0
-0.64839174654945,47.962810100989,0
-0.64837314796804,47.962904681951,0
-0.64836693064456,47.962999988426,0
-0.64999999999998,47.963,0
</coordinates>
</LinearRing>
</outerBoundaryIs>
</Polygon>
</Placemark>
<Placemark>
<name>255</name>
<styleUrl>#stylehap1</styleUrl>
<Polygon>
<outerBoundaryIs>
<LinearRing>
<coordinates>
-0.649,48.191,0
-0.64735967765208,48.190999988333,0
-0.64736591653472,48.191095294984,0
-0.64738459184838,48.19118987647,0
-0.64741556152982,48.191283012961,0
-0.647458589944,48.191373995624,0
-0.64751334967563,48.191462132011,0
-0.64757942401919,48.191546751336,0
-0.64765631014894,48.191627209575,0
-0.64774342294433,48.191702894372,0
-0.6478400994424,48.191773229696,0
-0.64794560388268,48.191837680232,0
-0.64805913330661,48.191895755447,0
-0.64817982366874,48.191947013333,0
-0.64830675641318,48.191991063765,0
-0.64843896546528,48.192027571473,0
-0.64857544458516,48.192056258596,0
-0.64871515502738,48.192076906793,0
-0.64885703344818,48.19208935891,0
-0.649,48.192093520174,0

-0.64914296655182,48.19208935891,0
-0.64928484497262,48.192076906793,0
-0.64942455541484,48.192056258596,0
-0.64956103453472,48.192027571473,0
-0.64969324358682,48.191991063765,0
-0.64982017633126,48.191947013333,0
-0.64994086669339,48.191895755447,0
-0.65005439611732,48.191837680232,0
-0.6501599005576,48.191773229696,0
-0.65025657705567,48.191702894372,0
-0.65034368985106,48.191627209575,0
-0.65042057598081,48.191546751336,0
-0.6504866503244,48.191462132011,0
-0.650541410056,48.191373995624,0
-0.65058443847018,48.191283012961,0
-0.65061540815163,48.19118987647,0
-0.65063408346528,48.191095294984,0
-0.65064032234792,48.190999988333,0
-0.65063407738704,48.190904681858,0
-0.65061539617979,48.190810100899,0
-0.65058442096855,48.190716965267,0
-0.65054138755635,48.190625983771,0
-0.65048662351034,48.190537848822,0
-0.6504205456671,48.190453231163,0
-0.65034365695875,48.190372774767,0
-0.65025654258419,48.190297091935,0
-0.65015986555435,48.190226758636,0
-0.65005436164586,48.190162310127,0
-0.64994083380109,48.190104236876,0
-0.64982014601755,48.190052980833,0
-0.64969321677276,48.190008932068,0
-0.64956101203507,48.189972425797,0
-0.64942453791322,48.189943739841,0
-0.64928483300079,48.189923092503,0
-0.64914296047355,48.189910640912,0
-0.649,48.189906479827,0
-0.64885703952645,48.189910640912,0
-0.64871516699921,48.189923092503,0
-0.64857546208678,48.189943739841,0
-0.64843898796493,48.189972425797,0
-0.64830678322724,48.190008932068,0
-0.64817985398245,48.190052980833,0
-0.64805916619891,48.190104236876,0
-0.64794563835415,48.190162310127,0
-0.64784013444565,48.190226758636,0
-0.64774345741581,48.190297091935,0
-0.64765634304125,48.190372774767,0
-0.6475794543329,48.190453231163,0
-0.64751337648966,48.190537848822,0
-0.64745861244365,48.190625983771,0
-0.64741557903145,48.190716965267,0
-0.64738460382021,48.190810100899,0
-0.64736592261296,48.190904681858,0
-0.64735967765208,48.190999988333,0
-0.649,48.191,0
</coordinates>
</LinearRing>

</outerBoundaryIs>
</Polygon>
</Placemark>
<Placemark>
<name>257</name>
<styleUrl>#stylehap1</styleUrl>
<Polygon>
<outerBoundaryIs>
<LinearRing>
<coordinates>
-0.63699999999994,48.189,0
-0.63535974166793,48.188999988333,0
-0.63536598030729,48.189095294985,0
-0.63538465489234,48.18918987647,0
-0.63541562336533,48.189283012962,0
-0.63545865010043,48.189373995624,0
-0.63551340769513,48.189462132012,0
-0.63557947946021,48.189546751337,0
-0.63565636258943,48.189627209576,0
-0.6357434719852,48.189702894372,0
-0.63584014471035,48.189773229697,0
-0.63594564503314,48.189837680232,0
-0.63605917002635,48.189895755447,0
-0.63617985567828,48.189947013333,0
-0.63630678346889,48.189991063765,0
-0.63643898736116,48.190027571473,0
-0.63657546115458,48.190056258596,0
-0.63671516614424,48.190076906793,0
-0.63685703902782,48.19008935891,0
-0.63699999999996,48.190093520174,0
-0.63714296097206,48.19008935891,0
-0.63728483385565,48.190076906793,0
-0.6374245388453,48.190056258596,0
-0.63756101263872,48.190027571473,0
-0.63769321653099,48.189991063765,0
-0.6378201443216,48.189947013333,0
-0.63794082997354,48.189895755447,0
-0.63805435496675,48.189837680232,0
-0.63815985528953,48.189773229697,0
-0.63825652801471,48.189702894372,0
-0.63834363741046,48.189627209576,0
-0.6384205205397,48.189546751337,0
-0.63848659230475,48.189462132012,0
-0.63854134989945,48.189373995624,0
-0.63858437663455,48.189283012962,0
-0.63861534510754,48.18918987647,0
-0.6386340196926,48.189095294985,0
-0.63864025833196,48.188999988333,0
-0.63863401361502,48.188904681859,0
-0.63861533313703,48.1888101009,0
-0.63858435913484,48.188716965268,0
-0.63854132740225,48.188625983772,0
-0.63848656549364,48.188537848822,0
-0.6384204902293,48.188453231163,0
-0.63834360452174,48.188372774768,0
-0.63825649354699,48.188297091935,0
-0.63815982029012,48.188226758637,0

-0.63805432049905,48.188162310127,0
-0.63794079708482,48.188104236876,0
-0.6378201140112,48.188052980834,0
-0.63769318971988,48.188008932068,0
-0.63756099014151,48.187972425798,0
-0.63742452134559,48.187943739841,0
-0.63728482188513,48.187923092503,0
-0.63714295489448,48.187910640912,0
-0.63699999999996,48.187906479827,0
-0.63685704510541,48.187910640912,0
-0.63671517811475,48.187923092503,0
-0.6365754786543,48.187943739841,0
-0.63643900985837,48.187972425798,0
-0.63630681028,48.188008932068,0
-0.63617988598869,48.188052980834,0
-0.63605920291506,48.188104236876,0
-0.63594567950083,48.188162310127,0
-0.63584017970976,48.188226758637,0
-0.6357435064529,48.188297091935,0
-0.63565639547814,48.188372774768,0
-0.63557950977059,48.188453231163,0
-0.63551343450627,48.188537848822,0
-0.63545867259764,48.188625983772,0
-0.63541564086505,48.188716965268,0
-0.63538466686286,48.1888101009,0
-0.63536598638487,48.188904681859,0
-0.63535974166793,48.188999988333,0
-0.63699999999994,48.189,0
</coordinates>
</LinearRing>
</outerBoundaryIs>
</Polygon>
</Placemark>
<Placemark>
<name>258</name>
<styleUrl>#stylehap1</styleUrl>
<Polygon>
<outerBoundaryIs>
<LinearRing>
<coordinates>
-0.63599999999997,48.088,0
-0.63436296539614,48.087999988375,0
-0.63436919178494,48.088095295026,0
-0.63438782967779,48.088189876511,0
-0.63441873729578,48.088283013,0
-0.63446167947591,48.088373995661,0
-0.63451632945883,48.088462132046,0
-0.63458227137407,48.088546751368,0
-0.63465900340343,48.088627209603,0
-0.63474594159906,48.088702894396,0
-0.63484242432665,48.088773229717,0
-0.63494771730048,48.088837680249,0
-0.63506101917134,48.088895755461,0
-0.63518146762575,48.088947013343,0
-0.63530814594905,48.088991063772,0
-0.63544009000303,48.089027571478,0
-0.63557629556498,48.089056258598,0

-0.6357157259717,48.089076906794,0
-0.63585732001099,48.089089358911,0
-0.63599999999997,48.089093520173,0
-0.63614267998895,48.089089358911,0
-0.63628427402825,48.089076906794,0
-0.63642370443497,48.089056258598,0
-0.63655990999689,48.089027571478,0
-0.63669185405089,48.088991063772,0
-0.63681853237419,48.088947013343,0
-0.6369389808286,48.088895755461,0
-0.63705228269946,48.088837680249,0
-0.63715757567326,48.088773229717,0
-0.63725405840088,48.088702894396,0
-0.63734099659651,48.088627209603,0
-0.63741772862587,48.088546751368,0
-0.63748367054111,48.088462132046,0
-0.63753832052403,48.088373995661,0
-0.63758126270416,48.088283013,0
-0.63761217032215,48.088189876511,0
-0.63763080821498,48.088095295026,0
-0.6376370346038,48.087999988375,0
-0.63763080217083,48.0879046819,0
-0.63761215841746,48.08781010094,0
-0.63758124530067,48.087716965306,0
-0.63753829815054,48.087625983808,0
-0.6374836438774,48.087537848856,0
-0.63741769848215,48.087453231194,0
-0.63734096388865,48.087372774795,0
-0.63725402412272,48.08729709196,0
-0.63715754086632,48.087226758657,0
-0.6370522484213,48.087162310144,0
-0.63693894812075,48.08710423689,0
-0.63681850223047,48.087052980844,0
-0.63669182738721,48.087008932075,0
-0.63655988762342,48.086972425802,0
-0.63642368703148,48.086943739844,0
-0.63628426212356,48.086923092505,0
-0.63614267394478,48.086910640913,0
-0.63599999999997,48.086906479826,0
-0.63585732605514,48.086910640913,0
-0.63571573787639,48.086923092505,0
-0.63557631296846,48.086943739844,0
-0.63544011237652,48.086972425802,0
-0.63530817261273,48.087008932075,0
-0.63518149776947,48.087052980844,0
-0.6350610518792,48.08710423689,0
-0.63494775157864,48.087162310144,0
-0.63484245913362,48.087226758657,0
-0.63474597587722,48.08729709196,0
-0.63465903611127,48.087372774795,0
-0.63458230151779,48.087453231194,0
-0.63451635612252,48.087537848856,0
-0.63446170184941,48.087625983808,0
-0.63441875469927,48.087716965306,0
-0.63438784158248,48.08781010094,0
-0.63436919782911,48.0879046819,0
-0.63436296539614,48.087999988375,0

-0.63599999999997,48.088,0
</coordinates>
</LinearRing>
</outerBoundaryIs>
</Polygon>
</Placemark>
<Placemark>
<name>259</name>
<styleUrl>#stylehap1</styleUrl>
<Polygon>
<outerBoundaryIs>
<LinearRing>
<coordinates>
-0.63499999999999,48.028,0
-0.63336487208706,48.027999988399,0
-0.6333710912302,48.02809529505,0
-0.63338970742124,48.028189876534,0
-0.63342057904621,48.028283013023,0
-0.63346347121583,48.028373995683,0
-0.63351805755122,48.028462132066,0
-0.63358392266607,48.028546751386,0
-0.6336605653266,48.02862720962,0
-0.63374740226502,48.028702894411,0
-0.63384377261759,48.02877322973,0
-0.63394894295375,48.028837680259,0
-0.63406211285775,48.028895755469,0
-0.63418242102046,48.02894701335,0
-0.63430895179501,48.028991063777,0
-0.63444074216629,48.029027571481,0
-0.63457678908133,48.0290562586,0
-0.63471605708455,48.029076906795,0
-0.63485748619995,48.029089358911,0
-0.63499999999999,48.029093520174,0
-0.63514251380005,48.029089358911,0
-0.63528394291542,48.029076906795,0
-0.63542321091864,48.0290562586,0
-0.63555925783368,48.029027571481,0
-0.63569104820499,48.028991063777,0
-0.63581757897953,48.02894701335,0
-0.63593788714222,48.028895755469,0
-0.63605105704622,48.028837680259,0
-0.63615622738239,48.02877322973,0
-0.63625259773495,48.028702894411,0
-0.63633943467337,48.02862720962,0
-0.6364160773339,48.028546751386,0
-0.63648194244877,48.028462132066,0
-0.63653652878414,48.028373995683,0
-0.63657942095376,48.028283013023,0
-0.63661029257876,48.028189876534,0
-0.63662890876977,48.02809529505,0
-0.63663512791292,48.027999988399,0
-0.63662890274534,48.027904681924,0
-0.63661028071295,48.027810100964,0
-0.63657940360714,48.027716965329,0
-0.63653650648374,48.02762598383,0
-0.63648191587218,48.027537848876,0
-0.63641604728865,48.027453231213,0

-0.63633940207235,48.027372774812,0
-0.63625256356874,48.027297091974,0
-0.63615619268912,48.02722675867,0
-0.63605102288001,48.027162310155,0
-0.6359378545412,48.027104236898,0
-0.63581754893426,48.02705298085,0
-0.6356910216284,48.02700893208,0
-0.63555923553329,48.026972425805,0
-0.635423193572,48.026943739846,0
-0.63528393104963,48.026923092505,0
-0.63514250777562,48.026910640913,0
-0.63499999999999,48.026906479826,0
-0.63485749222435,48.026910640913,0
-0.63471606895036,48.026923092505,0
-0.63457680642797,48.026943739846,0
-0.63444076446671,48.026972425805,0
-0.63430897837159,48.02700893208,0
-0.63418245106571,48.02705298085,0
-0.63406214545877,48.027104236898,0
-0.63394897711996,48.027162310155,0
-0.63384380731087,48.02722675867,0
-0.63374743643123,48.027297091974,0
-0.63366059792762,48.027372774812,0
-0.63358395271132,48.027453231213,0
-0.63351808412781,48.027537848876,0
-0.63346349351625,48.02762598383,0
-0.63342059639285,48.027716965329,0
-0.63338971928702,48.027810100964,0
-0.63337109725463,48.027904681924,0
-0.63336487208706,48.027999988399,0
-0.63499999999999,48.028,0
</coordinates>
</LinearRing>
</outerBoundaryIs>
</Polygon>
</Placemark>
<Placemark>
<name>260</name>
<styleUrl>#stylehap1</styleUrl>
<Polygon>
<outerBoundaryIs>
<LinearRing>
<coordinates>
-0.63499999999999,48.088,0
-0.63336296539615,48.087999988375,0
-0.63336919178498,48.088095295026,0
-0.63338782967781,48.088189876511,0
-0.6334187372958,48.088283013,0
-0.63346167947592,48.088373995661,0
-0.63351632945887,48.088462132046,0
-0.63358227137409,48.088546751368,0
-0.63365900340345,48.088627209603,0
-0.63374594159908,48.088702894396,0
-0.63384242432669,48.088773229717,0
-0.63394771730049,48.088837680249,0
-0.63406101917135,48.088895755461,0
-0.63418146762576,48.088947013343,0

-0.63430814594906,48.088991063772,0
-0.63444009000307,48.089027571478,0
-0.63457629556499,48.089056258598,0
-0.63471572597171,48.089076906794,0
-0.63485732001101,48.089089358911,0
-0.63499999999999,48.089093520173,0
-0.63514267998897,48.089089358911,0
-0.63528427402826,48.089076906794,0
-0.63542370443498,48.089056258598,0
-0.63555990999693,48.089027571478,0
-0.63569185405094,48.088991063772,0
-0.63581853237421,48.088947013343,0
-0.6359389808286,48.088895755461,0
-0.6360522826995,48.088837680249,0
-0.6361575756733,48.088773229717,0
-0.6362540584009,48.088702894396,0
-0.63634099659652,48.088627209603,0
-0.63641772862588,48.088546751368,0
-0.63648367054113,48.088462132046,0
-0.63653832052405,48.088373995661,0
-0.63658126270418,48.088283013,0
-0.63661217032216,48.088189876511,0
-0.63663080821502,48.088095295026,0
-0.63663703460382,48.087999988375,0
-0.63663080217084,48.0879046819,0
-0.63661215841747,48.08781010094,0
-0.63658124530069,48.087716965306,0
-0.63653829815058,48.087625983808,0
-0.63648364387744,48.087537848856,0
-0.63641769848216,48.087453231194,0
-0.63634096388869,48.087372774795,0
-0.63625402412273,48.08729709196,0
-0.63615754086633,48.087226758657,0
-0.63605224842132,48.087162310144,0
-0.63593894812076,48.08710423689,0
-0.63581850223049,48.087052980844,0
-0.63569182738723,48.087008932075,0
-0.63555988762343,48.086972425802,0
-0.63542368703149,48.086943739844,0
-0.63528426212357,48.086923092505,0
-0.63514267394482,48.086910640913,0
-0.63499999999999,48.086906479826,0
-0.63485732605518,48.086910640913,0
-0.6347157378764,48.086923092505,0
-0.63457631296848,48.086943739844,0
-0.63444011237654,48.086972425802,0
-0.63430817261275,48.087008932075,0
-0.63418149776948,48.087052980844,0
-0.63406105187921,48.08710423689,0
-0.63394775157865,48.087162310144,0
-0.63384245913367,48.087226758657,0
-0.63374597587724,48.08729709196,0
-0.63365903611131,48.087372774795,0
-0.63358230151781,48.087453231194,0
-0.63351635612256,48.087537848856,0
-0.63346170184942,48.087625983808,0
-0.63341875469928,48.087716965306,0

-0.6333878415825,48.08781010094,0
-0.63336919782915,48.0879046819,0
-0.63336296539615,48.087999988375,0
-0.634999999999999,48.088,0
</coordinates>
</LinearRing>
</outerBoundaryIs>
</Polygon>
</Placemark>
<Placemark>
<name>261</name>
<styleUrl>#stylehap1</styleUrl>
<Polygon>
<outerBoundaryIs>
<LinearRing>
<coordinates>
-0.634999999999999,48.089,0
-0.63336293356508,48.088999988374,0
-0.63336916007487,48.089095295025,0
-0.63338779833001,48.08918987651,0
-0.63341870654889,48.089283013,0
-0.6334616495639,48.089373995661,0
-0.63351630060938,48.089462132045,0
-0.63358224380674,48.089546751367,0
-0.63365897732807,48.089627209603,0
-0.63374591721412,48.089702894396,0
-0.63384240181778,48.089773229717,0
-0.63394769683894,48.089837680249,0
-0.63406100091291,48.089895755461,0
-0.6341814517094,48.089947013343,0
-0.63430813249593,48.089991063772,0
-0.63444007911557,48.090027571478,0
-0.63457628732603,48.090056258598,0
-0.63471572044398,48.090076906794,0
-0.63485731723657,48.090089358911,0
-0.634999999999999,48.090093520174,0
-0.6351426827634,48.090089358911,0
-0.63528427955599,48.090076906794,0
-0.63542371267394,48.090056258598,0
-0.6355599208844,48.090027571478,0
-0.63569186750405,48.089991063772,0
-0.63581854829057,48.089947013343,0
-0.63593899908706,48.089895755461,0
-0.63605230316106,48.089837680249,0
-0.63615759818222,48.089773229717,0
-0.63625408278585,48.089702894396,0
-0.63634102267191,48.089627209603,0
-0.63641775619323,48.089546751367,0
-0.63648369939059,48.089462132045,0
-0.6365383504361,48.089373995661,0
-0.63658129345111,48.089283013,0
-0.63661220166999,48.08918987651,0
-0.63663083992512,48.089095295025,0
-0.63663706643489,48.088999988374,0
-0.63663083388062,48.0889046819,0
-0.63661218976464,48.08881010094,0
-0.63658127604668,48.088716965306,0

-0.63653832806138,48.088625983808,0
-0.63648367272545,48.088537848856,0
-0.63641772604788,48.088453231194,0
-0.63634098996227,48.088372774795,0
-0.63625404850581,48.088297091959,0
-0.63615756337334,48.088226758657,0
-0.63605226888101,48.088162310144,0
-0.63593896637742,48.08810423689,0
-0.6358185181452,48.088052980844,0
-0.63569184083891,48.088008932075,0
-0.63555989850968,48.087972425802,0
-0.63542369526951,48.087943739844,0
-0.63528426765066,48.087923092505,0
-0.6351426767189,48.087910640913,0
-0.63499999999999,48.087906479826,0
-0.63485732328107,48.087910640913,0
-0.63471573234931,48.087923092505,0
-0.63457630473046,48.087943739844,0
-0.63444010149029,48.087972425802,0
-0.63430815916109,48.088008932075,0
-0.63418148185478,48.088052980844,0
-0.63406103362255,48.08810423689,0
-0.63394773111899,48.088162310144,0
-0.63384243662664,48.088226758657,0
-0.63374595149416,48.088297091959,0
-0.6336590100377,48.088372774795,0
-0.63358227395212,48.088453231194,0
-0.63351632727452,48.088537848856,0
-0.63346167193859,48.088625983808,0
-0.63341872395332,48.088716965306,0
-0.63338781023533,48.08881010094,0
-0.63336916611935,48.0889046819,0
-0.63336293356508,48.088999988374,0
-0.63499999999999,48.089,0
</coordinates>
</LinearRing>
</outerBoundaryIs>
</Polygon>
</Placemark>
<Placemark>
<name>262</name>
<styleUrl>#stylehap1</styleUrl>
<Polygon>
<outerBoundaryIs>
<LinearRing>
<coordinates>
-0.63499999999999,48.059,0
-0.63336388774238,48.058999988386,0
-0.63337011062616,48.059095295038,0
-0.63338873802092,48.059189876522,0
-0.63341962822758,48.059283013011,0
-0.63346254621552,48.059373995671,0
-0.63351716540948,48.059462132056,0
-0.63358307017314,48.059546751377,0
-0.63365975897108,48.059627209611,0
-0.63374664818434,48.059702894403,0
-0.63384307655144,48.059773229723,0

-0.63394831020029,48.059837680254,0
-0.63406154823327,48.059895755465,0
-0.63418192882271,48.059947013346,0
-0.63430853577049,48.059991063774,0
-0.63444040548173,48.060027571479,0
-0.63457653429951,48.060056258599,0
-0.63471588614485,48.060076906795,0
-0.63485740040355,48.060089358911,0
-0.63499999999999,48.060093520173,0
-0.63514259959643,48.060089358911,0
-0.63528411385512,48.060076906795,0
-0.63542346570046,48.060056258599,0
-0.63555959451827,48.060027571479,0
-0.63569146422951,48.059991063774,0
-0.63581807117729,48.059947013346,0
-0.63593845176673,48.059895755465,0
-0.63605168979969,48.059837680254,0
-0.63615692344853,48.059773229723,0
-0.63625335181564,48.059702894403,0
-0.63634024102891,48.059627209611,0
-0.63641692982683,48.059546751377,0
-0.63648283459049,48.059462132056,0
-0.63653745378445,48.059373995671,0
-0.63658037177242,48.059283013011,0
-0.63661126197908,48.059189876522,0
-0.63662988937384,48.059095295038,0
-0.63663611225762,48.058999988386,0
-0.63662988333923,48.058904681912,0
-0.63661125009319,48.058810100951,0
-0.63658035439643,48.058716965317,0
-0.63653743144632,48.058625983819,0
-0.63648280796897,48.058537848866,0
-0.63641689973077,48.058453231203,0
-0.63634020837276,48.058372774803,0
-0.63625331759165,48.058297091967,0
-0.63615688869657,48.058226758663,0
-0.6360516555757,48.058162310149,0
-0.63593841911058,48.058104236894,0
-0.6358180410812,48.058052980847,0
-0.63569143760796,48.058008932077,0
-0.63555957218014,48.057972425804,0
-0.63542344832448,48.057943739845,0
-0.63528410196926,48.057923092505,0
-0.63514259356182,48.057910640913,0
-0.63499999999999,48.057906479826,0
-0.63485740643815,48.057910640913,0
-0.63471589803074,48.057923092505,0
-0.63457655167549,48.057943739845,0
-0.63444042781986,48.057972425804,0
-0.63430856239201,48.058008932077,0
-0.63418195891877,48.058052980847,0
-0.63406158088942,48.058104236894,0
-0.63394834442428,48.058162310149,0
-0.6338431113034,48.058226758663,0
-0.63374668240835,48.058297091967,0
-0.63365979162724,48.058372774803,0
-0.63358310026923,48.058453231203,0

-0.63351719203103,48.058537848866,0
-0.63346256855365,48.058625983819,0
-0.63341964560356,48.058716965317,0
-0.63338874990678,48.058810100951,0
-0.63337011666076,48.058904681912,0
-0.63336388774238,48.058999988386,0
-0.63499999999999,48.059,0
</coordinates>
</LinearRing>
</outerBoundaryIs>
</Polygon>
</Placemark>
<Placemark>
<name>263</name>
<styleUrl>#stylehap1</styleUrl>
<Polygon>
<outerBoundaryIs>
<LinearRing>
<coordinates>
-0.63200000000006,48.053,0
-0.63036407839062,48.052999988389,0
-0.63037030054992,48.05309529504,0
-0.63038892577476,48.053189876524,0
-0.63041981238251,48.053283013014,0
-0.63046272536995,48.053373995674,0
-0.63051733819985,48.053462132058,0
-0.63058323528431,48.053546751378,0
-0.63065991514634,48.053627209613,0
-0.630746794235,48.053702894405,0
-0.63084321136583,48.053773229724,0
-0.63094843275224,48.053837680255,0
-0.63106165758997,48.053895755466,0
-0.63118202415179,48.053947013347,0
-0.6313086163463,48.053991063775,0
-0.63144047069095,48.054027571479,0
-0.63157658364581,48.054056258599,0
-0.63171591925259,48.054076906795,0
-0.63185741702069,48.054089358911,0
-0.63200000000006,48.054093520174,0
-0.63214258297942,48.054089358911,0
-0.63228408074753,48.054076906795,0
-0.6324234163543,48.054056258599,0
-0.63255952930916,48.054027571479,0
-0.63269138365381,48.053991063775,0
-0.63281797584833,48.053947013347,0
-0.63293834241015,48.053895755466,0
-0.63305156724787,48.053837680255,0
-0.63315678863431,48.053773229724,0
-0.63325320576512,48.053702894405,0
-0.63334008485378,48.053627209613,0
-0.63341676471581,48.053546751378,0
-0.63348266180029,48.053462132058,0
-0.63353727463016,48.053373995674,0
-0.63358018761763,48.053283013014,0
-0.63361107422538,48.053189876524,0
-0.63362969945019,48.05309529504,0
-0.63363592160949,48.052999988389,0

-0.63362969341757,48.052904681914,0
-0.63361106234339,48.052810100954,0
-0.63358017024733,48.05271696532,0
-0.63353725229936,48.052625983821,0
-0.63348263518744,48.052537848868,0
-0.63341673462957,48.052453231205,0
-0.63334005220831,48.052372774805,0
-0.63325317155233,48.052297091968,0
-0.63315675389372,48.052226758665,0
-0.63305153303508,48.05216231015,0
-0.63293830976468,48.052104236895,0
-0.63281794576211,48.052052980847,0
-0.63269135704097,48.052008932078,0
-0.63255950697833,48.051972425804,0
-0.63242339898402,48.051943739845,0
-0.63228406886556,48.051923092505,0
-0.6321425769468,48.051910640913,0
-0.63200000000006,48.051906479826,0
-0.63185742305334,48.051910640913,0
-0.63171593113456,48.051923092505,0
-0.63157660101612,48.051943739845,0
-0.63144049302178,48.051972425804,0
-0.63130864295915,48.052008932078,0
-0.63118205423803,48.052052980847,0
-0.63106169023546,48.052104236895,0
-0.63094846696506,48.05216231015,0
-0.63084324610642,48.052226758665,0
-0.63074682844782,48.052297091968,0
-0.63065994779181,48.052372774805,0
-0.63058326537055,48.052453231205,0
-0.63051736481267,48.052537848868,0
-0.63046274770078,48.052625983821,0
-0.63041982975279,48.05271696532,0
-0.63038893765672,48.052810100954,0
-0.63037030658257,48.052904681914,0
-0.63036407839062,48.052999988389,0
-0.63200000000006,48.053,0
</coordinates>
</LinearRing>
</outerBoundaryIs>
</Polygon>
</Placemark>
<Placemark>
<name>264</name>
<styleUrl>#stylehap1</styleUrl>
<Polygon>
<outerBoundaryIs>
<LinearRing>
<coordinates>
-0.63099999999997,48.027,0
-0.62936490381241,48.0269999884,0
-0.629371122835,48.027095295051,0
-0.62938973866493,48.027189876535,0
-0.62942060969102,48.027283013024,0
-0.62946350102853,48.027373995683,0
-0.62951808630487,48.027462132066,0
-0.62958395014185,48.027546751386,0

-0.62966059131538,48.02762720962,0
-0.62974742656897,48.027702894411,0
-0.62984379505175,48.02777322973,0
-0.62994896334735,48.027837680259,0
-0.63006213105555,48.027895755469,0
-0.63018243688394,48.02794701335,0
-0.63030896520342,48.027991063777,0
-0.63044075301759,48.028027571481,0
-0.63057679729292,48.0280562586,0
-0.63071606259392,48.028076906795,0
-0.63085748896513,48.028089358911,0
-0.63099999999997,48.028093520174,0
-0.63114251103481,48.028089358911,0
-0.63128393740602,48.028076906795,0
-0.63142320270702,48.0280562586,0
-0.63155924698235,48.028027571481,0
-0.63169103479652,48.027991063777,0
-0.63181756311601,48.02794701335,0
-0.63193786894439,48.027895755469,0
-0.63205103665259,48.027837680259,0
-0.63215620494819,48.02777322973,0
-0.63225257343097,48.027702894411,0
-0.63233940868456,48.02762720962,0
-0.63241604985809,48.027546751386,0
-0.63248191369507,48.027462132066,0
-0.63253649897144,48.027373995683,0
-0.63257939030892,48.027283013024,0
-0.63261026133501,48.027189876535,0
-0.63262887716494,48.027095295051,0
-0.63263509618753,48.0269999884,0
-0.63262887114084,48.026904681925,0
-0.63261024946986,48.026810100964,0
-0.63257937296325,48.02671696533,0
-0.63253647667224,48.02662598383,0
-0.63248188711993,48.026537848877,0
-0.63241601981447,48.026453231213,0
-0.63233937608532,48.026372774812,0
-0.63225253926662,48.026297091974,0
-0.63215617025681,48.02622675867,0
-0.63205100248827,48.026162310155,0
-0.63193783634516,48.026104236898,0
-0.63181753307238,48.02605298085,0
-0.63169100822138,48.02600893208,0
-0.63155922468315,48.025972425805,0
-0.63142318536132,48.025943739846,0
-0.63128392554087,48.025923092505,0
-0.63114250501071,48.025910640913,0
-0.63099999999997,48.025906479827,0
-0.63085749498923,48.025910640913,0
-0.63071607445907,48.025923092505,0
-0.63057681463862,48.025943739846,0
-0.63044077531679,48.025972425805,0
-0.63030899177856,48.02600893208,0
-0.63018246692756,48.02605298085,0
-0.63006216365478,48.026104236898,0
-0.62994899751167,48.026162310155,0
-0.62984382974313,48.02622675867,0

-0.62974746073332,48.026297091974,0
-0.62966062391462,48.026372774812,0
-0.62958398018547,48.026453231213,0
-0.62951811288001,48.026537848877,0
-0.6294635233277,48.02662598383,0
-0.62942062703669,48.02671696533,0
-0.62938975053008,48.026810100964,0
-0.6293711288591,48.026904681925,0
-0.62936490381241,48.0269999884,0
-0.63099999999997,48.027,0
</coordinates>
</LinearRing>
</outerBoundaryIs>
</Polygon>
</Placemark>
<Placemark>
<name>265</name>
<styleUrl>#stylehap1</styleUrl>
<Polygon>
<outerBoundaryIs>
<LinearRing>
<coordinates>
-0.625,48.067,0
-0.62336363344775,48.066999988383,0
-0.62336985729788,48.067095295034,0
-0.623388487587,48.067189876519,0
-0.62341938259403,48.067283013008,0
-0.62346230725187,48.067373995669,0
-0.62351693493448,48.067462132053,0
-0.62358284994098,48.067546751374,0
-0.62365955065798,48.067627209609,0
-0.6237464533759,48.067702894401,0
-0.62384289673041,48.067773229722,0
-0.6239481467354,48.067837680252,0
-0.62406140236873,48.067895755464,0
-0.62418180166881,48.067947013346,0
-0.62430842829512,48.067991063774,0
-0.62444031850297,48.068027571479,0
-0.62457646847944,48.068056258599,0
-0.62471584198448,48.068076906795,0
-0.624857378239,48.068089358911,0
-0.62500000000001,48.068093520174,0
-0.625142621761,48.068089358911,0
-0.62528415801552,48.068076906795,0
-0.62542353152056,48.068056258599,0
-0.62555968149703,48.068027571479,0
-0.62569157170488,48.067991063774,0
-0.62581819833119,48.067947013346,0
-0.62593859763127,48.067895755464,0
-0.6260518532646,48.067837680252,0
-0.62615710326959,48.067773229722,0
-0.6262535466241,48.067702894401,0
-0.62634044934202,48.067627209609,0
-0.62641715005902,48.067546751374,0
-0.62648306506552,48.067462132053,0
-0.62653769274812,48.067373995669,0
-0.62658061740597,48.067283013008,0

-0.626611512413,48.067189876519,0
-0.62663014270212,48.067095295034,0
-0.62663636655225,48.066999988383,0
-0.62663013666489,48.066904681909,0
-0.62661150052195,48.066810100948,0
-0.6265806000224,48.066716965314,0
-0.62653767040025,48.066625983816,0
-0.62648303843237,48.066537848863,0
-0.6264171199498,48.066453231201,0
-0.62634041667161,48.066372774801,0
-0.62625351238518,48.066297091965,0
-0.62615706850246,48.066226758662,0
-0.62605181902567,48.066162310148,0
-0.62593856496087,48.066104236893,0
-0.62581816822197,48.066052980846,0
-0.62569154507173,48.066008932077,0
-0.62555965914915,48.065972425803,0
-0.625423514137,48.065943739845,0
-0.62528414612447,48.065923092505,0
-0.62514261572375,48.065910640913,0
-0.62500000000001,48.065906479826,0
-0.62485738427625,48.065910640913,0
-0.62471585387553,48.065923092505,0
-0.624576485863,48.065943739845,0
-0.62444034085085,48.065972425803,0
-0.62430845492827,48.066008932077,0
-0.62418183177803,48.066052980846,0
-0.62406143503913,48.066104236893,0
-0.62394818097432,48.066162310148,0
-0.62384293149754,48.066226758662,0
-0.62374648761482,48.066297091965,0
-0.62365958332839,48.066372774801,0
-0.6235828800502,48.066453231201,0
-0.62351696156763,48.066537848863,0
-0.62346232959975,48.066625983816,0
-0.62341939997759,48.066716965314,0
-0.62338849947805,48.066810100948,0
-0.62336986333511,48.066904681909,0
-0.62336363344775,48.066999988383,0
-0.625,48.067,0
</coordinates>
</LinearRing>
</outerBoundaryIs>
</Polygon>
</Placemark>
<Placemark>
<name>266</name>
<styleUrl>#stylehap1</styleUrl>
<Polygon>
<outerBoundaryIs>
<LinearRing>
<coordinates>
-0.62099999999998,48.103,0
-0.61936248774769,48.102999988369,0
-0.61936871595162,48.10309529502,0
-0.61938735928101,48.103189876505,0
-0.61941827591569,48.103283012995,0

-0.61946123062402,48.103373995656,0
-0.6195158965514,48.103462132041,0
-0.61958185770602,48.103546751363,0
-0.61965861212333,48.103627209599,0
-0.61974557568508,48.103702894393,0
-0.61984208656396,48.103773229714,0
-0.61994741025993,48.103837680246,0
-0.62006074519006,48.103895755459,0
-0.6201812287892,48.103947013342,0
-0.62030794407517,48.103991063771,0
-0.62043992662849,48.104027571477,0
-0.62057617193336,48.104056258598,0
-0.62071564302405,48.104076906794,0
-0.6208572783787,48.104089358911,0
-0.62099999999997,48.104093520174,0
-0.62114272162124,48.104089358911,0
-0.62128435697592,48.104076906794,0
-0.62142382806661,48.104056258598,0
-0.62156007337148,48.104027571477,0
-0.6216920559248,48.103991063771,0
-0.62181877121077,48.103947013342,0
-0.6219392548099,48.103895755459,0
-0.62205258974004,48.103837680246,0
-0.62215791343601,48.103773229714,0
-0.62225442431489,48.103702894393,0
-0.62234138787664,48.103627209599,0
-0.62241814229393,48.103546751363,0
-0.62248410344855,48.103462132041,0
-0.62253876937595,48.103373995656,0
-0.62258172408428,48.103283012995,0
-0.62261264071896,48.103189876505,0
-0.62263128404835,48.10309529502,0
-0.62263751225228,48.102999988369,0
-0.62263127799924,48.102904681894,0
-0.62261262880452,48.102810100934,0
-0.62258170666654,48.102716965301,0
-0.62253874698413,48.102625983803,0
-0.62248407676303,48.102537848851,0
-0.62241811212553,48.10245323119,0
-0.62234135514201,48.102372774791,0
-0.62225439000864,48.102297091956,0
-0.62215787860054,48.102226758654,0
-0.62205255543378,48.102162310142,0
-0.62193922207528,48.102104236888,0
-0.62181874104237,48.102052980842,0
-0.62169202923928,48.102008932074,0
-0.62156005097966,48.101972425802,0
-0.62142381064888,48.101943739844,0
-0.62128434506148,48.101923092504,0
-0.62114271557213,48.101910640913,0
-0.62099999999997,48.101906479827,0
-0.62085728442784,48.101910640913,0
-0.62071565493849,48.101923092504,0
-0.62057618935107,48.101943739844,0
-0.62043994902028,48.101972425802,0
-0.62030797076069,48.102008932074,0
-0.6201812589576,48.102052980842,0

-0.62006077792469,48.102104236888,0
-0.61994744456616,48.102162310142,0
-0.61984212139943,48.102226758654,0
-0.6197456099913,48.102297091956,0
-0.61965864485795,48.102372774791,0
-0.61958188787442,48.10245323119,0
-0.61951592323691,48.102537848851,0
-0.61946125301582,48.102625983803,0
-0.6194182933334,48.102716965301,0
-0.61938737119544,48.102810100934,0
-0.61936872200073,48.102904681894,0
-0.61936248774769,48.102999988369,0
-0.62099999999998,48.103,0
</coordinates>
</LinearRing>
</outerBoundaryIs>
</Polygon>
</Placemark>
<Placemark>
<name>267</name>
<styleUrl>#stylehap1</styleUrl>
<Polygon>
<outerBoundaryIs>
<LinearRing>
<coordinates>
-0.62,48.056,0
-0.61836398307426,48.055999988388,0
-0.61837020559577,48.056095295039,0
-0.61838883190547,48.056189876523,0
-0.61841972031255,48.056283013013,0
-0.61846263580004,48.056373995672,0
-0.61851725181172,48.056462132057,0
-0.61858315273546,48.056546751377,0
-0.61865983706508,48.056627209612,0
-0.61874672121562,48.056702894404,0
-0.61884314396414,48.056773229724,0
-0.61894837148126,48.056837680254,0
-0.61906160291606,48.056895755465,0
-0.6191819764911,48.056947013347,0
-0.61930857606166,48.056991063774,0
-0.61944043808898,48.057027571479,0
-0.61957655897466,48.057056258599,0
-0.61971590270005,48.057076906795,0
-0.61985740871278,48.057089358911,0
-0.62000000000001,48.057093520174,0
-0.62014259128724,48.057089358911,0
-0.62028409729995,48.057076906795,0
-0.62042344102534,48.057056258599,0
-0.62055956191102,48.057027571479,0
-0.62069142393834,48.056991063774,0
-0.62081802350889,48.056947013347,0
-0.62093839708394,48.056895755465,0
-0.62105162851874,48.056837680254,0
-0.62115685603589,48.056773229724,0
-0.62125327878438,48.056702894404,0
-0.62134016293492,48.056627209612,0
-0.62141684726454,48.056546751377,0

-0.62148274818831,48.056462132057,0
-0.62153736419996,48.056373995672,0
-0.62158027968747,48.056283013013,0
-0.62161116809452,48.056189876523,0
-0.62162979440423,48.056095295039,0
-0.62163601692573,48.055999988388,0
-0.62162978837059,48.055904681913,0
-0.6216111562106,48.055810100953,0
-0.6215802623l431,48.055716965319,0
-0.62153734186549,48.05562598382,0
-0.62148272157111,48.055537848867,0
-0.62141681717339,48.055453231204,0
-0.62134013028411,48.055372774804,0
-0.62125324456598,48.055297091967,0
-0.6211568212896,48.055226758664,0
-0.62105159430035,48.05516231015,0
-0.62093836443313,48.055104236894,0
-0.62081799341774,48.055052980847,0
-0.62069139732115,48.055008932077,0
-0.62055953957656,48.054972425804,0
-0.6204234236522,48.054943739845,0
-0.62028408541603,48.054923092505,0
-0.6201425852536,48.054910640913,0
-0.62000000000001,48.054906479826,0
-0.6198574147464,48.054910640913,0
-0.61971591458397,48.054923092505,0
-0.61957657634779,48.054943739845,0
-0.61944046042347,48.054972425804,0
-0.61930860267885,48.055008932077,0
-0.61918200658228,48.055052980847,0
-0.6190616355669,48.055104236894,0
-0.61894840569967,48.05516231015,0
-0.6188431787104,48.055226758664,0
-0.61874675543402,48.055297091967,0
-0.61865986971589,48.055372774804,0
-0.61858318282661,48.055453231204,0
-0.61851727842889,48.055537848867,0
-0.61846265813451,48.05562598382,0
-0.61841973768569,48.055716965319,0
-0.6183888437894,48.055810100953,0
-0.61837021162941,48.055904681913,0
-0.61836398307426,48.055999988388,0
-0.62,48.056,0
</coordinates>
</LinearRing>
</outerBoundaryIs>
</Polygon>
</Placemark>
<Placemark>
<name>268</name>
<styleUrl>#stylehap1</styleUrl>
<Polygon>
<outerBoundaryIs>
<LinearRing>
<coordinates>
-0.61699999999996,48.018,0
-0.61536518926291,48.017999988403,0

-0.61537140720075,48.018095295054,0
-0.6153900197817,48.018189876538,0
-0.61542088541927,48.018283013027,0
-0.6154637692697,48.018373995686,0
-0.61551834501739,48.018462132069,0
-0.6155841973566,48.018546751389,0
-0.61566082515074,48.018627209623,0
-0.61574764524512,48.018702894413,0
-0.61584399690424,48.018773229732,0
-0.61594914683977,48.018837680261,0
-0.61606229479123,48.01889575547,0
-0.61618257961655,48.018947013351,0
-0.61630908584653,48.018991063777,0
-0.61644085065287,48.019027571481,0
-0.61657687117719,48.0190562586,0
-0.61671611216478,48.019076906795,0
-0.61685751384525,48.019089358911,0
-0.61699999999996,48.019093520173,0
-0.61714248615466,48.019089358911,0
-0.61728388783515,48.019076906795,0
-0.61742312882272,48.0190562586,0
-0.61755914934705,48.019027571481,0
-0.61769091415339,48.018991063777,0
-0.61781742038338,48.018947013351,0
-0.61793770520871,48.01889575547,0
-0.61805085316017,48.018837680261,0
-0.6181560030957,48.018773229732,0
-0.61825235475479,48.018702894413,0
-0.6183391748492,48.018627209623,0
-0.61841580264333,48.018546751389,0
-0.61848165498253,48.018462132069,0
-0.61853623073021,48.018373995686,0
-0.61857911458064,48.018283013027,0
-0.61860998021823,48.018189876538,0
-0.61862859279917,48.018095295054,0
-0.61863481073701,48.017999988403,0
-0.61862858677805,48.017904681928,0
-0.61860996835889,48.017810100968,0
-0.61857909724347,48.017716965333,0
-0.61853620844195,48.017625983833,0
-0.61848162842042,48.01753784888,0
-0.61841577261444,48.017453231216,0
-0.61833914226594,48.017372774814,0
-0.61825232060721,48.017297091976,0
-0.61815596842131,48.017226758672,0
-0.61805081901256,48.017162310156,0
-0.61793767262546,48.017104236899,0
-0.6178173903545,48.017052980851,0
-0.61769088759128,48.01700893208,0
-0.61755912705879,48.016972425806,0
-0.61742311148554,48.016943739846,0
-0.61728387597581,48.016923092505,0
-0.61714248013354,48.016910640913,0
-0.61699999999996,48.016906479826,0
-0.6168575198664,48.016910640913,0
-0.61671612402411,48.016923092505,0
-0.61657688851437,48.016943739846,0

-0.61644087294113,48.016972425806,0
-0.61630911240864,48.01700893208,0
-0.61618260964544,48.017052980851,0
-0.61606232737446,48.017104236899,0
-0.61594918098735,48.017162310156,0
-0.6158440315786,48.017226758672,0
-0.6157476793927,48.017297091976,0
-0.615660857734,48.017372774814,0
-0.61558422738549,48.017453231216,0
-0.6155183715795,48.01753784888,0
-0.61546379155796,48.017625983833,0
-0.61542090275647,48.017716965333,0
-0.61539003164103,48.017810100968,0
-0.61537141322189,48.017904681928,0
-0.61536518926291,48.017999988403,0
-0.61699999999996,48.018,0
</coordinates>
</LinearRing>
</outerBoundaryIs>
</Polygon>
</Placemark>
<Placemark>
<name>269</name>
<styleUrl>#stylehap1</styleUrl>
<Polygon>
<outerBoundaryIs>
<LinearRing>
<coordinates>
-0.61500000000001,48.017,0
-0.61336522097103,48.016999988404,0
-0.61337143878839,48.017095295055,0
-0.61339005100845,48.017189876539,0
-0.61342091604746,48.017283013027,0
-0.61346379906621,48.017373995687,0
-0.61351837375543,48.01746213207,0
-0.61358422481747,48.017546751389,0
-0.61366085112542,48.017627209623,0
-0.61374766953589,48.017702894413,0
-0.61384401932622,48.017773229732,0
-0.6139491672223,48.017837680261,0
-0.61406231297917,48.01789575547,0
-0.61418259547145,48.017947013351,0
-0.61430909924771,48.017991063777,0
-0.61444086149834,48.018027571481,0
-0.61457687938438,48.0180562586,0
-0.61471611767119,48.018076906795,0
-0.61485751660901,48.018089358911,0
-0.61500000000001,48.018093520174,0
-0.61514248339102,48.018089358911,0
-0.61528388232883,48.018076906795,0
-0.61542312061565,48.0180562586,0
-0.61555913850169,48.018027571481,0
-0.61569090075231,48.017991063777,0
-0.61581740452855,48.017947013351,0
-0.61593768702086,48.01789575547,0
-0.61605083277773,48.017837680261,0
-0.61615598067378,48.017773229732,0

-0.61625233046411,48.017702894413,0
-0.6163391488746,48.017627209623,0
-0.61641577518256,48.017546751389,0
-0.61648162624457,48.01746213207,0
-0.61653620093381,48.017373995687,0
-0.61657908395257,48.017283013027,0
-0.61660994899158,48.017189876539,0
-0.61662856121163,48.017095295055,0
-0.61663477902899,48.016999988404,0
-0.61662855519082,48.016904681929,0
-0.61660993713289,48.016810100968,0
-0.61657906661633,48.016716965333,0
-0.61653617864675,48.016625983834,0
-0.61648159968391,48.01653784888,0
-0.6164157451553,48.016453231216,0
-0.61633911629313,48.016372774815,0
-0.61625229631839,48.016297091977,0
-0.61615594600131,48.016226758672,0
-0.61605079863198,48.016162310156,0
-0.61593765443938,48.016104236899,0
-0.61581737450132,48.016052980851,0
-0.61569087419163,48.0160089320 8,0
-0.61555911621463,48.015972425806,0
-0.61542310327941,48.015943739846,0
-0.61528387047015,48.015923092505,0
-0.6151424773702,48.015910640913,0
-0.61500000000001,48.015906479826,0
-0.61485752262982,48.015910640913,0
-0.61471612952988,48.015923092505,0
-0.61457689672062,48.015943739846,0
-0.61444088378537,48.015972425806,0
-0.61430912580837,48.01600893208,0
-0.61418262549871,48.016052980851,0
-0.61406234556064,48.016104236899,0
-0.61394920136802,48.016162310156,0
-0.61384405399872,48.016226758672,0
-0.61374770368164,48.016297091977,0
-0.6136608837069,48.016372774815,0
-0.6135842548447,48.016453231216,0
-0.61351840031612,48.01653784888,0
-0.61346382135325,48.016625983834,0
-0.61342093338369,48.016716965333,0
-0.61339006286713,48.016810100968,0
-0.61337144480918,48.016904681929,0
-0.61336522097103,48.016999988404,0
-0.61500000000001,48.017,0
</coordinates>
</LinearRing>
</outerBoundaryIs>
</Polygon>
</Placemark>
<Placemark>
<name>270</name>
<styleUrl>#stylehap1</styleUrl>
<Polygon>
<outerBoundaryIs>
<LinearRing>

<coordinates>
-0.61500000000001,48.228,0
-0.61335849209492,48.227999988317,0
-0.61336473548279,48.228095294969,0
-0.61338342429038,48.228189876455,0
-0.61341441635181,48.228283012947,0
-0.61345747586189,48.22837399561,0
-0.61351227516877,48.228462131999,0
-0.6135783972659,48.228546751325,0
-0.61365533896407,48.228627209565,0
-0.61374251471998,48.228702894363,0
-0.61383926109146,48.228773229689,0
-0.61394484178632,48.228837680225,0
-0.61405845326566,48.228895755442,0
-0.61417923085947,48.228947013329,0
-0.61430625534795,48.228991063762,0
-0.61443855995808,48.229027571471,0
-0.61457513772266,48.229056258595,0
-0.61471494914543,48.229076906793,0
-0.61485693011393,48.22908935891,0
-0.61500000000001,48.229093520174,0
-0.61514306988609,48.22908935891,0
-0.61528505085457,48.229076906793,0
-0.61542486227734,48.229056258595,0
-0.61556144004195,48.229027571471,0
-0.61569374465208,48.228991063762,0
-0.61582076914053,48.228947013329,0
-0.61594154673437,48.228895755442,0
-0.61605515821368,48.228837680225,0
-0.61616073890854,48.228773229689,0
-0.61625748528005,48.228702894363,0
-0.61634466103596,48.228627209565,0
-0.61642160273413,48.228546751325,0
-0.61648772483123,48.228462131999,0
-0.61654252413811,48.22837399561,0
-0.61658558364822,48.228283012947,0
-0.61661657570965,48.228189876455,0
-0.61663526451723,48.228095294969,0
-0.61664150790511,48.227999988317,0
-0.61663525842667,48.227904681843,0
-0.61661656371359,48.227810100884,0
-0.61658556611115,48.227716965253,0
-0.61654250159292,48.227625983758,0
-0.61648769796292,48.227537848809,0
-0.61642157235907,48.227453231151,0
-0.61634462807707,48.227372774757,0
-0.61625745073879,48.227297091926,0
-0.61616070383443,48.227226758629,0
-0.61605512367242,48.227162310121,0
-0.61594151377547,48.227104236871,0
-0.61582073876547,48.22705298083,0
-0.61569371778374,48.227008932065,0
-0.61556141749675,48.226972425796,0
-0.6154248447403,48.22694373984,0
-0.61528503885854,48.226923092503,0
-0.61514306379553,48.226910640912,0
-0.61500000000001,48.226906479827,0

-0.61485693620449,48.226910640912,0
-0.61471496114149,48.226923092503,0
-0.61457515525973,48.22694373984,0
-0.61443858250328,48.226972425796,0
-0.61430628221628,48.227008932065,0
-0.61417926123455,48.22705298083,0
-0.61405848622455,48.227104236871,0
-0.61394487632758,48.227162310121,0
-0.61383929616557,48.227226758629,0
-0.6137425492612 3,48.227297091926,0
-0.61365537192296,48.227372774757,0
-0.61357842764096,48.227453231151,0
-0.61351230203711,48.227537848809,0
-0.61345749840711,48.227625983758,0
-0.61341443388888,48.227716965253,0
-0.61338343628644,48.227810100884,0
-0.61336474157335,48.227904681843,0
-0.61335849209492,48.227999988317,0
-0.61500000000001,48.228,0
</coordinates>
</LinearRing>
</outerBoundaryIs>
</Polygon>
</Placemark>
<Placemark>
<name>271</name>
<styleUrl>#stylehap1</styleUrl>
<Polygon>
<outerBoundaryIs>
<LinearRing>
<coordinates>
-0.61500000000001,48.32,0
-0.61335553380452,48.31999998828,0
-0.61336178843421,48.320095294931,0
-0.6133805109128,48.320189876418,0
-0.61341155881848,48.320283012912,0
-0.61345469592135,48.320373995577,0
-0.61350959397937,48.320462131968,0
-0.61357583523463,48.320546751296,0
-0.61365291559129,48.32062720954,0
-0.6137402484509,48.320702894341,0
-0.61383716917578,48.32077322967,0
-0.61394294014678,48.32083768021,0
-0.61405675637684,48.32089575543,0
-0.61417775163765,48.32094701332,0
-0.61430500505262,48.320991063755,0
-0.61443754810629,48.321027571467,0
-0.61457437201654,48.321056258592,0
-0.61471443541343,48.321076906792,0
-0.61485667226657,48.32108935891,0
-0.61500000000001,48.321093520173,0
-0.61514332773346,48.32108935891,0
-0.61528556458659,48.321076906792,0
-0.61542562798349,48.321056258592,0
-0.61556245189374,48.321027571467,0
-0.61569499494741,48.320991063755,0
-0.61582224836237,48.32094701332,0

-0.61594324362319,48.32089575543,0
-0.61605705985325,48.32083768021,0
-0.61616283082424,48.32077322967,0
-0.61625975154912,48.320702894341,0
-0.61634708440874,48.32062720954,0
-0.6164241647654,48.320546751296,0
-0.61649040602066,48.320462131968,0
-0.61654530407865,48.320373995577,0
-0.61658844118154,48.320283012912,0
-0.61661948908723,48.320189876418,0
-0.61663821156582,48.320095294931,0
-0.6166444661955,48.31999998828,0
-0.61663820544452,48.319904681806,0
-0.61661947703064,48.319810100848,0
-0.616588423556,48.319716965218,0
-0.61654528141969,48.319625983724,0
-0.61649037901675,48.319537848778,0
-0.61642413423706,48.319453231123,0
-0.61634705128354,48.319372774731,0
-0.61625971683357,48.319297091904,0
-0.61616279557316,48.31922675861,0
-0.6160570251377,48.319162310105,0
-0.61594321049799,48.319104236859,0
-0.61582221783403,48.31905298082,0
-0.61569496794351,48.319008932058,0
-0.61556242923478,48.318972425791,0
-0.61542561035795,48.318943739838,0
-0.61528555253,48.318923092502,0
-0.61514332161216,48.318910640912,0
-0.61500000000001,48.318906479826,0
-0.61485667838786,48.318910640912,0
-0.61471444747003,48.318923092502,0
-0.61457438964208,48.318943739838,0
-0.61443757076524,48.318972425791,0
-0.61430503205652,48.319008932058,0
-0.61417778216597,48.31905298082,0
-0.61405678950204,48.319104236859,0
-0.6139429748623,48.319162310105,0
-0.61383720442686,48.31922675861,0
-0.61374028316645,48.319297091904,0
-0.61365294871646,48.319372774731,0
-0.61357586576296,48.319453231123,0
-0.61350962098327,48.319537848778,0
-0.61345471858031,48.319625983724,0
-0.61341157644402,48.319716965218,0
-0.61338052296936,48.319810100848,0
-0.6133617945555,48.319904681806,0
-0.61335553380452,48.31999998828,0
-0.61500000000001,48.32,0
</coordinates>
</LinearRing>
</outerBoundaryIs>
</Polygon>
</Placemark>
<Placemark>
<name>273</name>
<styleUrl>#stylehap1</styleUrl>

<Polygon>
<outerBoundaryIs>
<LinearRing>
<coordinates>
-0.60900000000004,47.834,0
-0.60737099458041,47.833999988478,0
-0.60737719045726,47.834095295128,0
-0.60739573696248,47.834189876611,0
-0.60742649301176,47.834283013097,0
-0.60746922459477,47.834373995752,0
-0.60752360655418,47.834462132131,0
-0.60758922505864,47.834546751445,0
-0.60766558075078,47.834627209673,0
-0.60775209254649,47.834702894457,0
-0.60784810205637,47.834773229769,0
-0.60795287859608,47.834837680292,0
-0.60806562474691,47.834895755495,0
-0.60818548242494,47.834947013369,0
-0.6083115394121,47.834991063791,0
-0.60844283629949,47.83502757149,0
-0.60857837379035,47.835056258605,0
-0.60871712030672,47.835076906797,0
-0.60885801984208,47.835089358912,0
-0.60900000000003,47.835093520174,0
-0.609141980158,47.835089358912,0
-0.60928287969336,47.835076906797,0
-0.6094216262097,47.835056258605,0
-0.60955716370057,47.83502757149,0
-0.60968846058798,47.834991063791,0
-0.60981451757514,47.834947013369,0
-0.60993437525317,47.834895755495,0
-0.61004712140401,47.834837680292,0
-0.61015189794371,47.834773229769,0
-0.6102479074536,47.834702894457,0
-0.6103344192493,47.834627209673,0
-0.61041077494144,47.834546751445,0
-0.6104763934459,47.834462132131,0
-0.61053077540531,47.834373995752,0
-0.61057350698832,47.834283013097,0
-0.61060426303761,47.834189876611,0
-0.61062280954282,47.834095295128,0
-0.61062900541967,47.833999988478,0
-0.61062280358167,47.833904682002,0
-0.61060425129645,47.83381010104,0
-0.61057348982388,47.833716965403,0
-0.61053075333913,47.833625983899,0
-0.61047636714844,47.833537848941,0
-0.61041074521175,47.833453231272,0
-0.61033438699069,47.833372774864,0
-0.61024787364626,47.83329709202,0
-0.61015186361482,47.833226758709,0
-0.61004708759664,47.833162310187,0
-0.60993434299459,47.833104236924,0
-0.60981448784545,47.83305298087,0
-0.60968843429052,47.833008932094,0
-0.60955714163439,47.832972425814,0
-0.60942160904527,47.832943739851,0

-0.60928286795218,47.832923092508,0
-0.60914197419685,47.832910640913,0
-0.60900000000003,47.832906479826,0
-0.60885802580323,47.832910640913,0
-0.60871713204791,47.832923092508,0
-0.60857839095481,47.832943739851,0
-0.60844285836566,47.832972425814,0
-0.60831156570954,47.833008932094,0
-0.60818551215463,47.83305298087,0
-0.6080656570055,47.833104236924,0
-0.60795291240341,47.833162310187,0
-0.60784813638527,47.833226758709,0
-0.60775212635382,47.83329709202,0
-0.60766561300936,47.833372774864,0
-0.60758925478833,47.833453231272,0
-0.60752363285165,47.833537848941,0
-0.60746924666095,47.833625983899,0
-0.6074265101762,47.833716965403,0
-0.60739574870363,47.83381010104,0
-0.60737719641841,47.833904682002,0
-0.60737099458041,47.833999988478,0
-0.60900000000004,47.834,0
</coordinates>
</LinearRing>
</outerBoundaryIs>
</Polygon>
</Placemark>
<Placemark>
<name>274</name>
<styleUrl>#stylehap1</styleUrl>
<Polygon>
<outerBoundaryIs>
<LinearRing>
<coordinates>
-0.60599999999999,48.26,0
-0.60435746481254,48.259999988304,0
-0.6043637121042,48.260095294956,0
-0.60438241260421,48.260189876442,0
-0.60441342405787,48.260283012935,0
-0.60445651051249,48.260373995599,0
-0.60451134411122,48.260462131988,0
-0.60457750758665,48.260546751315,0
-0.60465449743475,48.260627209556,0
-0.60474172774575,48.260702894355,0
-0.60483853466242,48.260773229682,0
-0.60494418143168,48.26083768022,0
-0.60505786401184,48.260895755438,0
-0.60517871719168,48.260947013326,0
-0.60530582117611,48.26099106376,0
-0.60543820858705,48.26102757147,0
-0.60557487182705,48.261056258594,0
-0.6057147707492,48.261076906792,0
-0.60585684057502,48.26108935891,0
-0.606,48.261093520173,0
-0.60614315942495,48.26108935891,0
-0.60628522925077,48.261076906792,0
-0.60642512817294,48.261056258594,0

-0.60656179141295,48.26102757147,0
-0.60669417882388,48.26099106376,0
-0.60682128280832,48.260947013326,0
-0.60694213598816,48.260895755438,0
-0.60705581856829,48.26083768022,0
-0.60716146533757,48.260773229682,0
-0.60725827225424,48.260702894355,0
-0.60734550256524,48.260627209556,0
-0.60742249241335,48.260546751315,0
-0.60748865588875,48.260462131988,0
-0.60754348948751,48.260373995599,0
-0.6075865759421,48.260283012935,0
-0.60761758739576,48.260189876442,0
-0.6076362878958,48.260095294956,0
-0.60764253518746,48.259999988304,0
-0.60763628179458,48.25990468183,0
-0.60761757537871,48.259810100872,0
-0.60758655837435,48.259716965241,0
-0.60754346690282,48.259625983746,0
-0.60748862897337,48.259537848798,0
-0.60742246198508,48.259453231142,0
-0.60734546954864,48.259372774748,0
-0.60725823765248,48.259297091918,0
-0.60716143020204,48.259226758622,0
-0.60705578396655,48.259162310115,0
-0.60694210297156,48.259104236867,0
-0.60682125238003,48.259052980826,0
-0.6066941519085,48.259008932063,0
-0.60656176882823,48.258972425794,0
-0.60642511060516,48.258943739839,0
-0.60628521723372,48.258923092502,0
-0.60614315332373,48.258910640912,0
-0.606,48.258906479826,0
-0.60585684667624,48.258910640912,0
-0.60571478276627,48.258923092502,0
-0.60557488939483,48.258943739839,0
-0.60543823117174,48.258972425794,0
-0.60530584809149,48.259008932063,0
-0.60517874761994,48.259052980826,0
-0.60505789702844,48.259104236867,0
-0.60494421603345,48.259162310115,0
-0.60483856979796,48.259226758622,0
-0.60474176234751,48.259297091918,0
-0.60465453045135,48.259372774748,0
-0.60457753801491,48.259453231142,0
-0.6045113710266,48.259537848798,0
-0.60445653309717,48.259625983746,0
-0.60441344162565,48.259716965241,0
-0.60438242462129,48.259810100872,0
-0.60436371820542,48.25990468183,0
-0.60435746481254,48.259999988304,0
-0.60599999999999,48.26,0
</coordinates>
</LinearRing>
</outerBoundaryIs>
</Polygon>
</Placemark>

<Placemark>
<name>276</name>
<styleUrl>#stylehap1</styleUrl>
<Polygon>
<outerBoundaryIs>
<LinearRing>
<coordinates>
-0.60500000000002,48.04,0
-0.60336449124777,48.039999988394,0
-0.60337071183814,48.040095295045,0
-0.60338933236387,48.04018987653,0
-0.60342021117802,48.040283013019,0
-0.60346311333667,48.040373995678,0
-0.60351771238489,48.040462132062,0
-0.60358359283973,48.040546751382,0
-0.60366025335065,48.040627209617,0
-0.60374711051402,48.040702894408,0
-0.60384350331221,48.040773229727,0
-0.60394869814381,48.040837680257,0
-0.60406189440665,48.040895755467,0
-0.60418223059099,48.040947013348,0
-0.60430879083668,48.040991063776,0
-0.60444061190431,48.04102757148,0
-0.60457669050722,48.0410562586,0
-0.60471599094866,48.041076906795,0
-0.60485745300565,48.041089358911,0
-0.60500000000001,48.041093520174,0
-0.60514254699438,48.041089358911,0
-0.60528400905139,48.041076906795,0
-0.60542330949281,48.0410562586,0
-0.60555938809572,48.04102757148,0
-0.60569120916335,48.040991063776,0
-0.60581776940904,48.040947013348,0
-0.60593810559338,48.040895755467,0
-0.60605130185624,48.040837680257,0
-0.60615649668784,48.040773229727,0
-0.60625288948601,48.040702894408,0
-0.60633974664937,48.040627209617,0
-0.6064164071603,48.040546751382,0
-0.60648228761514,48.040462132062,0
-0.60653688666336,48.040373995678,0
-0.60657978882201,48.040283013019,0
-0.60661066763618,48.04018987653,0
-0.60662928816188,48.040095295045,0
-0.60663550875228,48.039999988394,0
-0.60662928213351,48.03990468192,0
-0.60661065576261,48.039810100959,0
-0.60657977146402,48.039716965325,0
-0.60653686434839,48.039625983825,0
-0.60648226102115,48.039537848872,0
-0.60641637709538,48.039453231209,0
-0.60633971402704,48.039372774808,0
-0.60625285529744,48.039297091971,0
-0.60615646197186,48.039226758667,0
-0.60605126766767,48.039162310153,0
-0.60593807297104,48.039104236896,0
-0.60581773934412,48.039052980849,0

-0.60569118256938,48.039008932079,0
-0.60555936578074,48.038972425805,0
-0.60542329213482,48.038943739845,0
-0.60528399717782,48.038923092505,0
-0.60514254096601,48.038910640913,0
-0.60500000000001,48.038906479827,0
-0.60485745903402,48.038910640913,0
-0.60471600282221,48.038923092505,0
-0.60457670786521,48.038943739845,0
-0.60444063421931,48.038972425805,0
-0.60430881743067,48.039008932079,0
-0.60418226065591,48.039052980849,0
-0.60406192702899,48.039104236896,0
-0.60394873233236,48.039162310153,0
-0.60384353802817,48.039226758667,0
-0.60374714470259,48.039297091971,0
-0.60366028597302,48.039372774808,0
-0.60358362290465,48.039453231209,0
-0.60351773897888,48.039537848872,0
-0.60346313565167,48.039625983825,0
-0.60342022853601,48.039716965325,0
-0.60338934423742,48.039810100959,0
-0.60337071786651,48.03990468192,0
-0.60336449124777,48.039999988394,0
-0.60500000000002,48.04,0
</coordinates>
</LinearRing>
</outerBoundaryIs>
</Polygon>
</Placemark>
<Placemark>
<name>279</name>
<styleUrl>#stylehap1</styleUrl>
<Polygon>
<outerBoundaryIs>
<LinearRing>
<coordinates>
-0.60199999999998,48.253,0
-0.60035768968417,48.252999988307,0
-0.60036393612128,48.253095294959,0
-0.60038263406184,48.253189876445,0
-0.60041364127055,48.253283012937,0
-0.60045672182702,48.253373995601,0
-0.60051154791929,48.25346213199,0
-0.60057770233701,48.253546751317,0
-0.60065468164511,48.253627209558,0
-0.60074190001404,48.253702894357,0
-0.60083869367739,48.253773229684,0
-0.60094432598302,48.253837680221,0
-0.60105799299922,48.253895755439,0
-0.6011788296333,48.253947013327,0
-0.60130591621611,48.25399106376,0
-0.60143828550198,48.25402757147,0
-0.60157493003147,48.254056258594,0
-0.60171480980003,48.254076906792,0
-0.60185686017503,48.25408935891,0
-0.60199999999998,48.254093520174,0

-0.60214313982491,48.25408935891,0
-0.60228519019991,48.254076906792,0
-0.6024250699685,48.254056258594,0
-0.60256171449796,48.25402757147,0
-0.60269408378383,48.25399106376,0
-0.60282117036664,48.253947013327,0
-0.60294200700075,48.253895755439,0
-0.60305567401695,48.253837680221,0
-0.60316130632257,48.253773229684,0
-0.60325809998592,48.253702894357,0
-0.60334531835483,48.253627209558,0
-0.60342229766293,48.253546751317,0
-0.60348845208067,48.25346213199,0
-0.60354327817294,48.253373995601,0
-0.60358635872939,48.253283012937,0
-0.6036173659381,48.253189876445,0
-0.60363606387866,48.253095294959,0
-0.60364231031579,48.252999988307,0
-0.60363605777978,48.252904681833,0
-0.60361735392565,48.252810100874,0
-0.60358634116835,48.252716965243,0
-0.60354325559688,48.252625983749,0
-0.60348842517557,48.252537848801,0
-0.60342226724632,48.252453231144,0
-0.60334528535088,48.25237277475,0
-0.60325806539742,48.25229709192,0
-0.60316127120047,48.252226758624,0
-0.60305563942844,48.252162310117,0
-0.60294197399677,48.252104236868,0
-0.60282113995003,48.252052980827,0
-0.60269405687875,48.252008932063,0
-0.60256169192192,48.251972425795,0
-0.60242505240743,48.25194373984,0
-0.60228517818746,48.251923092502,0
-0.60214313372603,48.251910640912,0
-0.60199999999998,48.251906479826,0
-0.60185686627391,48.251910640912,0
-0.6017148218125,48.251923092502,0
-0.60157494759251,48.25194373984,0
-0.60143830807804,48.251972425795,0
-0.60130594312119,48.252008932063,0
-0.60117886004994,48.252052980827,0
-0.60105802600317,48.252104236868,0
-0.6009443605715,48.252162310117,0
-0.60083872879947,48.252226758624,0
-0.60074193460255,48.25229709192,0
-0.60065471464909,48.25237277475,0
-0.60057773275365,48.252453231144,0
-0.60051157482437,48.252537848801,0
-0.60045674440306,48.252625983749,0
-0.60041365883159,48.252716965243,0
-0.60038264607431,48.252810100874,0
-0.60036394222019,48.252904681833,0
-0.60035768968417,48.252999988307,0
-0.60199999999998,48.253,0
</coordinates>
</LinearRing>

</outerBoundaryIs>
</Polygon>
</Placemark>
<Placemark>
<name>281</name>
<styleUrl>#stylehap1</styleUrl>
<Polygon>
<outerBoundaryIs>
<LinearRing>
<coordinates>
-0.601,48.438,0
-0.59935171759643,48.437999988231,0
-0.5993579867281,48.438095294883,0
-0.59937675264238,48.438189876371,0
-0.59940787258735,48.438283012866,0
-0.59945110978527,48.438373995534,0
-0.59950613523268,48.438462131928,0
-0.59957253020244,48.43854675126,0
-0.59964978942919,48.438627209507,0
-0.59973732495329,48.438702894312,0
-0.59983447059481,48.438773229645,0
-0.5999404870229,48.43883768019,0
-0.60005456738235,48.438895755414,0
-0.60017584343457,48.438947013307,0
-0.60030339216579,48.438991063746,0
-0.60043624281274,48.439027571461,0
-0.60057338425201,48.439056258589,0
-0.60071377269674,48.43907690679,0
-0.60085633964226,48.43908935891,0
-0.601,48.439093520174,0
-0.60114366035773,48.43908935891,0
-0.60128622730326,48.43907690679,0
-0.60142661574799,48.439056258589,0
-0.60156375718726,48.439027571461,0
-0.6016966078342,48.438991063746,0
-0.60182415656542,48.438947013307,0
-0.60194543261764,48.438895755414,0
-0.6020595129771,48.43883768019,0
-0.60216552940518,48.438773229645,0
-0.6022626750467,48.438702894312,0
-0.6023502105708,48.438627209507,0
-0.60242746979756,48.43854675126,0
-0.60249386476732,48.438462131928,0
-0.60254889021472,48.438373995534,0
-0.60259212741265,48.438283012866,0
-0.60262324735762,48.438189876371,0
-0.6026420132719,48.438095294883,0
-0.60264828240356,48.437999988231,0
-0.60264200711091,48.437904681757,0
-0.60262323522284,48.4378101008,0
-0.60259210967276,48.437716965172,0
-0.60254886740877,48.437625983681,0
-0.60249383758826,48.437537848738,0
-0.60242743907119,48.437453231086,0
-0.60235017723075,48.437372774699,0
-0.60226264010597,48.437297091875,0
-0.60216549392543,48.437226758585,0

-0.60205947803636,48.437162310085,0
-0.60194539927759,48.437104236843,0
-0.60182412583905,48.437052980808,0
-0.60169658065514,48.437008932049,0
-0.60156373438131,48.436972425786,0
-0.6014265980081,48.436943739834,0
-0.60128621516848,48.4369230925,0
-0.60114365419674,48.436910640911,0
-0.601,48.436906479826,0
-0.60085634580325,48.436910640911,0
-0.60071378483152,48.4369230925,0
-0.6005734019919,48.436943739834,0
-0.60043626561869,48.436972425786,0
-0.60030341934485,48.437008932049,0
-0.60017587416094,48.437052980808,0
-0.60005460072243,48.437104236843,0
-0.59994052196363,48.437162310085,0
-0.59983450607456,48.437226758585,0
-0.59973735989403,48.437297091875,0
-0.59964982276924,48.437372774699,0
-0.5995725609288,48.437453231086,0
-0.59950616241174,48.437537848738,0
-0.59945113259122,48.437625983681,0
-0.59940789032724,48.437716965172,0
-0.59937676477716,48.4378101008,0
-0.59935799288909,48.437904681757,0
-0.59935171759643,48.437999988231,0
-0.601,48.438,0
</coordinates>
</LinearRing>
</outerBoundaryIs>
</Polygon>
</Placemark>
<Placemark>
<name>283</name>
<styleUrl>#stylehap1</styleUrl>
<Polygon>
<outerBoundaryIs>
<LinearRing>
<coordinates>
-0.598999999999993,48.277,0
-0.59735691833701,48.276999988297,0
-0.59736316770535,48.277095294949,0
-0.59738187442531,48.277189876435,0
-0.59741289619488,48.277283012928,0
-0.59745599698296,48.277373995593,0
-0.59751084882368,48.277462131982,0
-0.59757703431081,48.27754675131,0
-0.59765404977283,48.277627209551,0
-0.59774130910512,48.277702894351,0
-0.59783814822951,48.277773229679,0
-0.59794383014787,48.277837680217,0
-0.59805755055095,48.277895755435,0
-0.59817844393972,48.277947013324,0
-0.59830559021302,48.277991063758,0
-0.59843802167087,48.278027571469,0
-0.59857473038057,48.278056258593,0

-0.59871467584908,48.278076906792,0
-0.59885679294365,48.27808935891,0
-0.59899999999993,48.278093520174,0
-0.59914320705621,48.27808935891,0
-0.59928532415077,48.278076906792,0
-0.59942526961931,48.278056258593,0
-0.59956197832898,48.278027571469,0
-0.59969440978684,48.277991063758,0
-0.59982155606013,48.277947013324,0
-0.5999424494489,48.277895755435,0
-0.60005616985198,48.277837680217,0
-0.60016185177037,48.277773229679,0
-0.60025869089476,48.277702894351,0
-0.60034595022702,48.277627209551,0
-0.60042296568907,48.27754675131,0
-0.60048915117618,48.277462131982,0
-0.6005440030169,48.277373995593,0
-0.60058710380498,48.277283012928,0
-0.60061812557457,48.277189876435,0
-0.60063683229453,48.277095294949,0
-0.60064308166286,48.276999988297,0
-0.60063682618764,48.276904681823,0
-0.60061811354633,48.276810100865,0
-0.60058708622086,48.276716965234,0
-0.60054398041122,48.27662598374,0
-0.60048912423576,48.276537848793,0
-0.60042293523249,48.276453231136,0
-0.60034591717971,48.276372774743,0
-0.60025865626081,48.276297091914,0
-0.60016181660214,48.276226758619,0
-0.60005613521803,48.276162310112,0
-0.59994241640159,48.276104236864,0
-0.59982152560355,48.276052980825,0
-0.59969438284642,48.276008932061,0
-0.59956195572328,48.275972425793,0
-0.5994252520352,48.275943739839,0
-0.59928531212255,48.275923092502,0
-0.59914320094931,48.275910640912,0
-0.59899999999993,48.275906479827,0
-0.59885679905054,48.275910640912,0
-0.59871468787733,48.275923092502,0
-0.59857474796468,48.275943739839,0
-0.59843804427657,48.275972425793,0
-0.59830561715343,48.276008932061,0
-0.59817847439631,48.276052980825,0
-0.59805758359829,48.276104236864,0
-0.59794386478182,48.276162310112,0
-0.59783818339772,48.276226758619,0
-0.59774134373904,48.276297091914,0
-0.59765408282017,48.276372774743,0
-0.59757706476739,48.276453231136,0
-0.59751087576412,48.276537848793,0
-0.59745601958866,48.27662598374,0
-0.59741291377899,48.276716965234,0
-0.59738188645353,48.276810100865,0
-0.59736317381224,48.276904681823,0
-0.59735691833701,48.276999988297,0

-0.59899999999993,48.277,0
</coordinates>
</LinearRing>
</outerBoundaryIs>
</Polygon>
</Placemark>
<Placemark>
<name>285</name>
<styleUrl>#stylehap1</styleUrl>
<Polygon>
<outerBoundaryIs>
<LinearRing>
<coordinates>
-0.59799999999996,48.278,0
-0.59635688617559,48.277999988297,0
-0.59636313566614,48.278095294949,0
-0.59638184275216,48.278189876435,0
-0.59641286512886,48.278283012928,0
-0.59645596676048,48.278373995592,0
-0.5965108196748,48.278462131982,0
-0.59657700645736,48.278546751309,0
-0.59665402342684,48.278627209551,0
-0.59674128446708,48.278702894351,0
-0.59683812548698,48.278773229678,0
-0.59694380947398,48.278837680217,0
-0.59705753210304,48.278895755435,0
-0.59717842785819,48.278947013324,0
-0.59730557662028,48.278991063758,0
-0.59743801067042,48.279027571469,0
-0.5975747220561,48.279056258593,0
-0.59771467026399,48.279076906792,0
-0.59785679014045,48.27908935891,0
-0.59799999999997,48.279093520174,0
-0.59814320985946,48.27908935891,0
-0.59828532973592,48.279076906792,0
-0.59842527794381,48.279056258593,0
-0.5985619893295,48.279027571469,0
-0.59869442337963,48.278991063758,0
-0.59882157214172,48.278947013324,0
-0.59894246789687,48.278895755435,0
-0.59905619052593,48.278837680217,0
-0.59916187451293,48.278773229678,0
-0.59925871553283,48.278702894351,0
-0.59934597657307,48.278627209551,0
-0.59942299354255,48.278546751309,0
-0.59948918032511,48.278462131982,0
-0.59954403323943,48.278373995592,0
-0.59958713487105,48.278283012928,0
-0.59961815724775,48.278189876435,0
-0.59963686433377,48.278095294949,0
-0.59964311382432,48.277999988297,0
-0.59963685822655,48.277904681823,0
-0.59961814521886,48.277810100864,0
-0.59958711728599,48.277716965234,0
-0.59954401063248,48.277625983739,0
-0.59948915338322,48.277537848792,0
-0.59942296308432,48.277453231136,0

-0.59934594352393,48.277372774743,0
-0.59925868089699,48.277297091914,0
-0.59916183934279,48.277226758618,0
-0.5990561558901,48.277162310112,0
-0.59894243484775,48.277104236864,0
-0.59882154168346,48.277052980824,0
-0.59869439643771,48.277008932061,0
-0.59856196672257,48.276972425793,0
-0.59842526035872,48.276943739839,0
-0.59828531770701,48.276923092502,0
-0.59814320375223,48.276910640912,0
-0.59799999999997,48.276906479826,0
-0.59785679624768,48.276910640912,0
-0.5977146822929,48.276923092502,0
-0.59757473964119,48.276943739839,0
-0.59743803327734,48.276972425793,0
-0.5973056035622,48.277008932061,0
-0.59717845831645,48.277052980824,0
-0.59705756515216,48.277104236864,0
-0.59694384410981,48.277162310112,0
-0.59683816065712,48.277226758618,0
-0.59674131910292,48.277297091914,0
-0.59665405647598,48.277372774743,0
-0.59657703691562,48.277453231136,0
-0.59651084661669,48.277537848792,0
-0.59645598936743,48.277625983739,0
-0.59641288271392,48.277716965234,0
-0.59638185478105,48.277810100864,0
-0.59636314177336,48.277904681823,0
-0.59635688617559,48.277999988297,0
-0.59799999999996,48.278,0
</coordinates>
</LinearRing>
</outerBoundaryIs>
</Polygon>
</Placemark>
<Placemark>
<name>286</name>
<styleUrl>#stylehap1</styleUrl>
<Polygon>
<outerBoundaryIs>
<LinearRing>
<coordinates>
-0.59799999999996,48.456,0
-0.59635113329001,48.455999988223,0
-0.59635740464209,48.456095294876,0
-0.59637617720688,48.456189876364,0
-0.59640730818191,48.456283012859,0
-0.59645056070558,48.456373995527,0
-0.59650560565777,48.456462131922,0
-0.59657202416305,48.456546751254,0
-0.5966493107769,48.456627209502,0
-0.59673687733134,48.456702894308,0
-0.59683405741025,48.456773229642,0
-0.59694011142068,48.456837680186,0
-0.59705423222148,48.456895755411,0
-0.59717555126617,48.456947013306,0

-0.59730314521378,48.456991063745,0
-0.59743604295688,48.45702757146,0
-0.59757323301358,48.457056258588,0
-0.59771367122696,48.45707690679,0
-0.59785628871351,48.45708935891,0
-0.59799999999997,48.457093520174,0
-0.5981437112864,48.45708935891,0
-0.59828632877295,48.45707690679,0
-0.59842676698633,48.457056258588,0
-0.59856395704303,48.45702757146,0
-0.59869685478613,48.456991063745,0
-0.59882444873374,48.456947013306,0
-0.59894576777843,48.456895755411,0
-0.59905988857923,48.456837680186,0
-0.59916594258966,48.456773229642,0
-0.59926312266857,48.456702894308,0
-0.59935068922301,48.456627209502,0
-0.59942797583686,48.456546751254,0
-0.59949439434214,48.456462131922,0
-0.59954943929433,48.456373995527,0
-0.599592691818,48.456283012859,0
-0.59962382279303,48.456189876364,0
-0.59964259535783,48.456095294876,0
-0.59964886670992,48.455999988223,0
-0.59964258919075,48.45590468175,0
-0.59962381064626,48.455810100793,0
-0.59959267406058,48.455716965165,0
-0.59954941646586,48.455625983675,0
-0.59949436713621,48.455537848732,0
-0.59942794508013,48.455453231081,0
-0.59935065585001,48.455372774694,0
-0.59926308769334,48.455297091871,0
-0.59916590707488,48.455226758582,0
-0.59905985360399,48.455162310082,0
-0.59894573440543,48.45510423684,0
-0.59882441797704,48.455052980806,0
-0.59869682758022,48.455008932048,0
-0.59856393421456,48.454972425785,0
-0.59842674922891,48.454943739834,0
-0.59828631662618,48.4549230925,0
-0.59814370511933,48.454910640911,0
-0.59799999999997,48.454906479826,0
-0.59785629488058,48.454910640911,0
-0.59771368337373,48.4549230925,0
-0.597573250771,48.454943739834,0
-0.59743606578535,48.454972425785,0
-0.59730317241969,48.455008932048,0
-0.59717558202287,48.455052980806,0
-0.59705426559448,48.45510423684,0
-0.59694014639595,48.455162310082,0
-0.59683409292503,48.455226758582,0
-0.5967369123066,48.455297091871,0
-0.5966493441499,48.455372774694,0
-0.59657205491978,48.455453231081,0
-0.5965056328637,48.455537848732,0
-0.59645058353405,48.455625983675,0
-0.59640732593933,48.455716965165,0

-0.59637618935365,48.455810100793,0
-0.59635741080916,48.45590468175,0
-0.59635113329001,48.455999988223,0
-0.59799999999996,48.456,0
</coordinates>
</LinearRing>
</outerBoundaryIs>
</Polygon>
</Placemark>
<Placemark>
<name>288</name>
<styleUrl>#stylehap1</styleUrl>
<Polygon>
<outerBoundaryIs>
<LinearRing>
<coordinates>
-0.59699999999998,48.193,0
-0.59535961362916,48.192999988332,0
-0.59536585275509,48.193095294983,0
-0.59538452879745,48.193189876469,0
-0.59541549968746,48.19328301296,0
-0.59545852978089,48.193373995623,0
-0.59551329164967,48.193462132011,0
-0.59557936857205,48.193546751335,0
-0.59565625770262,48.193627209575,0
-0.59574337389804,48.193702894371,0
-0.59584005416945,48.193773229696,0
-0.59594556272764,48.193837680231,0
-0.59605909658276,48.193895755447,0
-0.5961797916556,48.193947013333,0
-0.59630672935446,48.193991063764,0
-0.59643894356691,48.194027571473,0
-0.59657542801382,48.194056258596,0
-0.59671514390926,48.194076906793,0
-0.59685702786787,48.19408935891,0
-0.59699999999998,48.194093520173,0
-0.5971429721321,48.19408935891,0
-0.59728485609071,48.194076906793,0
-0.59742457198613,48.194056258596,0
-0.59756105643305,48.194027571473,0
-0.59769327064551,48.193991063764,0
-0.59782020834434,48.193947013333,0
-0.5979409034172,48.193895755447,0
-0.5980544372723,48.193837680231,0
-0.59815994583051,48.193773229696,0
-0.59825662610192,48.193702894371,0
-0.59834374229734,48.193627209575,0
-0.59842063142791,48.193546751335,0
-0.5984867083503,48.193462132011,0
-0.59854147021908,48.193373995623,0
-0.59858450031248,48.19328301296,0
-0.59861547120249,48.193189876469,0
-0.59863414724488,48.193095294983,0
-0.59864038637081,48.192999988332,0
-0.59863414116597,48.192904681857,0
-0.59861545922938,48.192810100898,0
-0.59858448280895,48.192716965266,0

-0.59854144771696,48.19262598377,0
-0.59848668153331,48.192537848821,0
-0.59842060111089,48.192453231162,0
-0.59834370940143,48.192372774766,0
-0.59825659162667,48.192297091934,0
-0.59815991082345,48.192226758636,0
-0.59805440279708,48.192162310127,0
-0.59794087052131,48.192104236876,0
-0.59782017802732,48.192052980833,0
-0.59769324382852,48.192008932067,0
-0.59756103393093,48.191972425797,0
-0.59742455448259,48.191943739841,0
-0.59728484411757,48.191923092503,0
-0.59714296605317,48.191910640912,0
-0.59699999999998,48.191906479826,0
-0.5968570339468,48.191910640912,0
-0.59671515588237,48.191923092503,0
-0.59657544551738,48.191943739841,0
-0.59643896606901,48.191972425797,0
-0.59630675617144,48.192008932067,0
-0.59617982197262,48.192052980833,0
-0.59605912947865,48.192104236876,0
-0.59594559720289,48.192162310127,0
-0.59584008917652,48.192226758636,0
-0.5957434083733,48.192297091934,0
-0.59565629059852,48.192372774766,0
-0.59557939888908,48.192453231162,0
-0.59551331846666,48.192537848821,0
-0.59545855228301,48.19262598377,0
-0.59541551719102,48.192716965266,0
-0.59538454077058,48.192810100898,0
-0.59536585883399,48.192904681857,0
-0.59535961362916,48.192999988332,0
-0.59699999999998,48.193,0
</coordinates>
</LinearRing>
</outerBoundaryIs>
</Polygon>
</Placemark>
<Placemark>
<name>289</name>
<styleUrl>#stylehap1</styleUrl>
<Polygon>
<outerBoundaryIs>
<LinearRing>
<coordinates>
-0.596,47.802,0
-0.59437199828349,47.801999988491,0
-0.59437819034612,47.802095295141,0
-0.59439672542724,47.802189876623,0
-0.59442746252938,47.802283013109,0
-0.59447016778632,47.802373995763,0
-0.59452451624093,47.802462132141,0
-0.59459009431683,47.802546751455,0
-0.59466640296423,47.802627209681,0
-0.594752861457,47.802702894464,0
-0.59484881181144,47.802773229775,0

-0.59495352379334,47.802837680297,0
-0.59506620047534,47.802895755499,0
-0.59518598430229,47.802947013372,0
-0.59531196361817,47.802991063793,0
-0.59544317960532,47.803027571491,0
-0.59557863358271,47.803056258606,0
-0.59571729460811,47.803076906798,0
-0.5958581073257,47.803089358912,0
-0.596,47.803093520174,0
-0.59614189267429,47.803089358912,0
-0.59628270539188,47.803076906798,0
-0.59642136641729,47.803056258606,0
-0.5965568203947,47.803027571491,0
-0.59668803638183,47.802991063793,0
-0.59681401569773,47.802947013372,0
-0.59693379952465,47.802895755499,0
-0.59704647620668,47.802837680297,0
-0.59715118818858,47.802773229775,0
-0.597247138543,47.802702894464,0
-0.59733359703579,47.802627209681,0
-0.59740990568317,47.802546751455,0
-0.59747548375906,47.802462132141,0
-0.5975298322137,47.802373995763,0
-0.59757253747062,47.802283013109,0
-0.59760327457276,47.802189876623,0
-0.59762180965388,47.802095295141,0
-0.59762800171653,47.801999988491,0
-0.59762180370309,47.801904682015,0
-0.59760326285201,47.801810101053,0
-0.597572520336,47.801716965415,0
-0.59752981018584,47.801625983911,0
-0.5974754575073,47.801537848952,0
-0.59740987600514,47.801453231281,0
-0.59733356483323,47.801372774873,0
-0.59724710479438,47.801297092028,0
-0.59715115391933,47.801226758715,0
-0.59704644245807,47.801162310192,0
-0.5969337673221,47.801104236928,0
-0.59681398601969,47.801052980873,0
-0.59668801013006,47.801008932096,0
-0.59655679836684,47.800972425816,0
-0.59642134928267,47.800943739852,0
-0.59628269367113,47.800923092508,0
-0.5961418867235,47.800910640913,0
-0.596,47.800906479827,0
-0.5958581132765,47.800910640913,0
-0.59571730632889,47.800923092508,0
-0.59557865071735,47.800943739852,0
-0.59544320163316,47.800972425816,0
-0.59531198986993,47.801008932096,0
-0.59518601398033,47.801052980873,0
-0.5950662326779,47.801104236928,0
-0.59495355754195,47.801162310192,0
-0.59484884608067,47.801226758715,0
-0.59475289520561,47.801297092028,0
-0.59466643516676,47.801372774873,0
-0.59459012399486,47.801453231281,0

-0.5945245424927,47.801537848952,0
-0.59447018981416,47.801625983911,0
-0.59442747966402,47.801716965415,0
-0.59439673714802,47.801810101053,0
-0.59437819629691,47.801904682015,0
-0.59437199828349,47.801999988491,0
-0.596,47.802,0
</coordinates>
</LinearRing>
</outerBoundaryIs>
</Polygon>
</Placemark>
<Placemark>
<name>290</name>
<styleUrl>#stylehap1</styleUrl>
<Polygon>
<outerBoundaryIs>
<LinearRing>
<coordinates>
-0.596,47.802,0
-0.59437199828349,47.801999988491,0
-0.59437819034612,47.802095295141,0
-0.59439672542724,47.802189876623,0
-0.59442746252938,47.802283013109,0
-0.59447016778632,47.802373995763,0
-0.59452451624093,47.802462132141,0
-0.59459009431683,47.802546751455,0
-0.59466640296423,47.802627209681,0
-0.594752861457,47.802702894464,0
-0.59484881181144,47.802773229775,0
-0.59495352379334,47.802837680297,0
-0.59506620047534,47.802895755499,0
-0.59518598430229,47.802947013372,0
-0.59531196361817,47.802991063793,0
-0.59544317960532,47.803027571491,0
-0.59557863358271,47.803056258606,0
-0.59571729460811,47.803076906798,0
-0.5958581073257,47.803089358912,0
-0.596,47.803093520174,0
-0.59614189267429,47.803089358912,0
-0.59628270539188,47.803076906798,0
-0.59642136641729,47.803056258606,0
-0.5965568203947,47.803027571491,0
-0.59668803638183,47.802991063793,0
-0.59681401569773,47.802947013372,0
-0.59693379952465,47.802895755499,0
-0.59704647620668,47.802837680297,0
-0.59715118818858,47.802773229775,0
-0.597247138543,47.802702894464,0
-0.59733359703579,47.802627209681,0
-0.59740990568317,47.802546751455,0
-0.59747548375906,47.802462132141,0
-0.5975298322137,47.802373995763,0
-0.59757253747062,47.802283013109,0
-0.59760327457276,47.802189876623,0
-0.59762180965388,47.802095295141,0
-0.59762800171653,47.801999988491,0

-0.59762180370309,47.801904682015,0
-0.59760326285201,47.801810101053,0
-0.597572520336,47.801716965415,0
-0.59752981018584,47.801625983911,0
-0.5974754575073,47.801537848952,0
-0.59740987600514,47.801453231281,0
-0.59733356483323,47.801372774873,0
-0.59724710479438,47.801297092028,0
-0.59715115391933,47.801226758715,0
-0.59704644245807,47.801162310192,0
-0.5969337673221,47.801104236928,0
-0.59681398601969,47.801052980873,0
-0.59668801013006,47.801008932096,0
-0.59655679836684,47.800972425816,0
-0.59642134928267,47.800943739852,0
-0.59628269367113,47.800923092508,0
-0.5961418867235,47.800910640913,0
-0.596,47.800906479827,0
-0.5958581132765,47.800910640913,0
-0.59571730632889,47.800923092508,0
-0.59557865071735,47.800943739852,0
-0.59544320163316,47.800972425816,0
-0.59531198986993,47.801008932096,0
-0.59518601398033,47.801052980873,0
-0.5950662326779,47.801104236928,0
-0.59495355754195,47.801162310192,0
-0.59484884608067,47.801226758715,0
-0.59475289520561,47.801297092028,0
-0.59466643516676,47.801372774873,0
-0.59459012399486,47.801453231281,0
-0.5945245424927,47.801537848952,0
-0.59447018981416,47.801625983911,0
-0.59442747966402,47.801716965415,0
-0.59439673714802,47.801810101053,0
-0.59437819629691,47.801904682015,0
-0.59437199828349,47.801999988491,0
-0.596,47.802,0
</coordinates>
</LinearRing>
</outerBoundaryIs>
</Polygon>
</Placemark>
<Placemark>
<name>291</name>
<styleUrl>#stylehap1</styleUrl>
<Polygon>
<outerBoundaryIs>
<LinearRing>
<coordinates>
-0.596,48.193,0
-0.59435961362917,48.192999988332,0
-0.5943658527551,48.193095294983,0
-0.59438452879749,48.193189876469,0
-0.5944154996875,48.19328301296,0
-0.5944585297809,48.193373995623,0
-0.59451329164969,48.193462132011,0
-0.59457936857207,48.193546751335,0

-0.59465625770264,48.193627209575,0
-0.59474337389806,48.193702894371,0
-0.59484005416947,48.193773229696,0
-0.59494556272768,48.193837680231,0
-0.59505909658278,48.193895755447,0
-0.59517979165564,48.193947013333,0
-0.59530672935447,48.193991063764,0
-0.59543894356693,48.194027571473,0
-0.59557542801386,48.194056258596,0
-0.59571514390928,48.194076906793,0
-0.59585702786791,48.19408935891,0
-0.596,48.194093520173,0
-0.59614297213211,48.19408935891,0
-0.59628485609072,48.194076906793,0
-0.59642457198617,48.194056258596,0
-0.59656105643309,48.194027571473,0
-0.59669327064553,48.193991063764,0
-0.59682020834438,48.193947013333,0
-0.59694090341722,48.193895755447,0
-0.59705443727235,48.193837680231,0
-0.59715994583055,48.193773229696,0
-0.59725662610194,48.193702894371,0
-0.59734374229736,48.193627209575,0
-0.59742063142793,48.193546751335,0
-0.59748670835031,48.193462132011,0
-0.59754147021909,48.193373995623,0
-0.59758450031253,48.19328301296,0
-0.59761547120253,48.193189876469,0
-0.5976341472449,48.193095294983,0
-0.59764038637083,48.192999988332,0
-0.59763414116599,48.192904681857,0
-0.5976154592294,48.192810100898,0
-0.59758448280899,48.192716965266,0
-0.59754144771697,48.19262598377,0
-0.59748668153332,48.192537848821,0
-0.59742060111091,48.192453231162,0
-0.59734370940147,48.192372774766,0
-0.59725659162668,48.192297091934,0
-0.59715991082346,48.192226758636,0
-0.59705440279709,48.192162310127,0
-0.59694087052133,48.192104236876,0
-0.59682017802736,48.192052980833,0
-0.59669324382854,48.192008932067,0
-0.59656103393097,48.191972425797,0
-0.59642455448261,48.191943739841,0
-0.59628484411761,48.191923092503,0
-0.59614296605318,48.191910640912,0
-0.596,48.191906479826,0
-0.59585703394682,48.191910640912,0
-0.59571515588241,48.191923092503,0
-0.59557544551739,48.191943739841,0
-0.59543896606905,48.191972425797,0
-0.59530675617146,48.192008932067,0
-0.59517982197266,48.192052980833,0
-0.59505912947867,48.192104236876,0
-0.59494559720291,48.192162310127,0
-0.59484008917654,48.192226758636,0

-0.59474340837331,48.192297091934,0
-0.59465629059856,48.192372774766,0
-0.59457939888909,48.192453231162,0
-0.59451331846667,48.192537848821,0
-0.59445855228302,48.19262598377,0
-0.59441551719103,48.192716965266,0
-0.5943845407706,48.192810100898,0
-0.59436585883401,48.192904681857,0
-0.59435961362917,48.192999988332,0
-0.596,48.193,0
</coordinates>
</LinearRing>
</outerBoundaryIs>
</Polygon>
</Placemark>
<Placemark>
<name>292</name>
<styleUrl>#stylehap1</styleUrl>
<Polygon>
<outerBoundaryIs>
<LinearRing>
<coordinates>
-0.59500000000003,48.27,0
-0.59335714341796,48.2699999883,0
-0.59336339193095,48.270095294952,0
-0.59338209608906,48.270189876438,0
-0.59341311360974,48.270283012931,0
-0.5934562084942,48.270373995595,0
-0.59351105282146,48.270462131985,0
-0.59357722924246,48.270546751312,0
-0.59365423415468,48.270627209553,0
-0.59374148153376,48.270702894353,0
-0.59383830739252,48.27077322968,0
-0.59394397483378,48.270837680218,0
-0.59405767965846,48.270895755436,0
-0.59417855648608,48.270947013325,0
-0.59430568534156,48.270991063759,0
-0.59443809865748,48.271027571469,0
-0.59457478863923,48.271056258593,0
-0.59471471493636,48.271076906792,0
-0.59485681256201,48.27108935891,0
-0.59500000000004,48.271093520174,0
-0.59514318743805,48.27108935891,0
-0.5952852850637,48.271076906792,0
-0.59542521136082,48.271056258593,0
-0.59556190134257,48.271027571469,0
-0.59569431465849,48.270991063759,0
-0.59582144351398,48.270947013325,0
-0.5959423203416,48.270895755436,0
-0.59605602516627,48.270837680218,0
-0.59616169260754,48.27077322968,0
-0.59625851846629,48.270702894353,0
-0.59634576584537,48.270627209553,0
-0.59642277075759,48.270546751312,0
-0.59648894717859,48.270462131985,0
-0.59654379150585,48.270373995595,0
-0.59658688639031,48.270283012931,0

-0.59661790391099,48.270189876438,0
-0.5966366080691,48.270095294952,0
-0.59664285658209,48.2699999883,0
-0.59663660196455,48.269904681826,0
-0.59661789188735,48.269810100868,0
-0.59658686881291,48.269716965237,0
-0.59654376890882,48.269625983742,0
-0.59648892024848,48.269537848795,0
-0.59642274031266,48.269453231138,0
-0.59634573281068,48.269372774745,0
-0.5962584838456,48.269297091916,0
-0.59616165745276,48.26922675862,0
-0.59605599054558,48.269162310114,0
-0.59594228730693,48.269104236865,0
-0.59582141306905,48.269052980825,0
-0.59569428772838,48.269008932062,0
-0.59556187874552,48.268972425794,0
-0.59542519378343,48.268943739839,0
-0.59528527304006,48.268923092502,0
-0.59514318133349,48.268910640912,0
-0.59500000000004,48.268906479826,0
-0.59485681866656,48.268910640912,0
-0.59471472696,48.268923092502,0
-0.59457480621663,48.268943739839,0
-0.59443812125453,48.268972425794,0
-0.59430571227167,48.269008932062,0
-0.59417858693101,48.269052980825,0
-0.59405771269312,48.269104236865,0
-0.59394400945448,48.269162310114,0
-0.59383834254729,48.26922675862,0
-0.59374151615446,48.269297091916,0
-0.59365426718938,48.269372774745,0
-0.59357725968739,48.269453231138,0
-0.59351107975157,48.269537848795,0
-0.59345623109123,48.269625983742,0
-0.59341313118714,48.269716965237,0
-0.59338210811271,48.269810100868,0
-0.59336339803551,48.269904681826,0
-0.59335714341796,48.2699999883,0
-0.59500000000003,48.27,0
</coordinates>
</LinearRing>
</outerBoundaryIs>
</Polygon>
</Placemark>
<Placemark>
<name>293</name>
<styleUrl>#stylehap1</styleUrl>
<Polygon>
<outerBoundaryIs>
<LinearRing>
<coordinates>
-0.59299999999996,48.267,0
-0.59135723985475,48.266999988301,0
-0.59136348800127,48.267095294953,0
-0.59138219106173,48.267189876439,0
-0.59141320676196,48.267283012932,0

-0.59145629911697,48.267373995596,0
-0.59151114022507,48.267462131986,0
-0.59157731276164,48.267546751313,0
-0.59165431315376,48.267627209554,0
-0.59174155541146,48.267702894353,0
-0.59183837558649,48.267773229681,0
-0.59194403682496,48.267837680219,0
-0.592057734975,48.267895755437,0
-0.59217860470693,48.267947013325,0
-0.59230572609966,48.267991063759,0
-0.59243813164261,48.268027571469,0
-0.5925748136003,48.268056258593,0
-0.59271473168336,48.268076906792,0
-0.59285682096746,48.26808935891,0
-0.59299999999997,48.268093520174,0
-0.59314317903246,48.26808935891,0
-0.59328526831656,48.268076906792,0
-0.59342518639961,48.268056258593,0
-0.5935618683573,48.268027571469,0
-0.59369427390025,48.267991063759,0
-0.59382139529298,48.267947013325,0
-0.59394226502494,48.267895755437,0
-0.59405596317495,48.267837680219,0
-0.59416162441345,48.267773229681,0
-0.59425844458847,48.267702894353,0
-0.59434568684615,48.267627209554,0
-0.59442268723827,48.267546751313,0
-0.59448885977484,48.267462131986,0
-0.59454370088295,48.267373995596,0
-0.59458679323795,48.267283012932,0
-0.59461780893818,48.267189876439,0
-0.59463651199867,48.267095294953,0
-0.59464276014519,48.266999988301,0
-0.59463650589511,48.266904681827,0
-0.59461779691652,48.266810100869,0
-0.59458677566345,48.266716965238,0
-0.59454367828961,48.266625983743,0
-0.59448883284916,48.266537848796,0
-0.59442265679835,48.266453231139,0
-0.5943456538169,48.266372774746,0
-0.59425840997348,48.266297091917,0
-0.59416158926445,48.266226758621,0
-0.59405592855995,48.266162310114,0
-0.59394223199567,48.266104236866,0
-0.59382136485306,48.266052980826,0
-0.59369424697456,48.266008932062,0
-0.59356184576396,48.265972425794,0
-0.59342516882513,48.265943739839,0
-0.5932852562949,48.265923092502,0
-0.59314317292889,48.265910640912,0
-0.59299999999997,48.265906479826,0
-0.59285682707102,48.265910640912,0
-0.59271474370504,48.265923092502,0
-0.5925748311748,48.265943739839,0
-0.59243815423595,48.265972425794,0
-0.59230575302535,48.266008932062,0
-0.59217863514685,48.266052980826,0

-0.59205776800424,48.266104236866,0
-0.59194407143998,48.266162310114,0
-0.59183841073549,48.266226758621,0
-0.59174159002646,48.266297091917,0
-0.59165434618304,48.266372774746,0
-0.59157734320156,48.266453231139,0
-0.59151116715075,48.266537848796,0
-0.5914563217103,48.266625983743,0
-0.59141322433646,48.266716965238,0
-0.59138220308341,48.266810100869,0
-0.59136349410483,48.266904681827,0
-0.59135723985475,48.266999988301,0
-0.59299999999996,48.267,0
</coordinates>
</LinearRing>
</outerBoundaryIs>
</Polygon>
</Placemark>
<Placemark>
<name>295</name>
<styleUrl>#stylehap1</styleUrl>
<Polygon>
<outerBoundaryIs>
<LinearRing>
<coordinates>
-0.58799999999997,48.46,0
-0.58635100336578,48.459999988222,0
-0.5863572752116,48.460095294874,0
-0.58637604925517,48.460189876362,0
-0.58640718268282,48.460283012858,0
-0.58645043861424,48.460373995526,0
-0.58650548790346,48.46046213192,0
-0.586571911642,48.460546751253,0
-0.58664920434554,48.460627209501,0
-0.58673677779977,48.460702894307,0
-0.58683396553606,48.460773229641,0
-0.58694002790319,48.460837680186,0
-0.58705415769635,48.460895755411,0
-0.58717548630069,48.460947013305,0
-0.58730309030245,48.460991063745,0
-0.58743599851769,48.46102757146,0
-0.58757319938479,48.461056258588,0
-0.58771364866454,48.46107690679,0
-0.58785627738921,48.46108935891,0
-0.58799999999997,48.461093520174,0
-0.58814372261073,48.46108935891,0
-0.5882863513354,48.46107690679,0
-0.58842680061515,48.461056258588,0
-0.58856400148225,48.46102757146,0
-0.58869690969749,48.460991063745,0
-0.58882451369924,48.460947013305,0
-0.58894584230359,48.460895755411,0
-0.58905997209675,48.460837680186,0
-0.58916603446388,48.460773229641,0
-0.58926322220017,48.460702894307,0
-0.58935079565437,48.460627209501,0
-0.58942808835791,48.460546751253,0

-0.58949451209648,48.46046213192,0
-0.5895495613857,48.460373995526,0
-0.58959281731711,48.460283012858,0
-0.58962395074476,48.460189876362,0
-0.58964272478834,48.460095294874,0
-0.58964899663416,48.459999988222,0
-0.58964271861992,48.459904681748,0
-0.58962393859532,48.459810100792,0
-0.58959279955583,48.459716965164,0
-0.58954953855222,48.459625983673,0
-0.58949448488459,48.459537848731,0
-0.58942805759447,48.4594532310 8,0
-0.58935076227406,48.459372774693,0
-0.58926318721725,48.45929709187,0
-0.58916599894128,48.459226758581,0
-0.58905993711383,48.459162310081,0
-0.58894580892326,48.45910423684,0
-0.58882448293577,48.459052980806,0
-0.58869688248561,48.459008932048,0
-0.58856397864877,48.458972425785,0
-0.58842678285384,48.458943739834,0
-0.58828633918596,48.4589230925,0
-0.58814371644231,48.458910640911,0
-0.58799999999997,48.458906479827,0
-0.58785628355763,48.458910640911,0
-0.58771366081398,48.4589230925,0
-0.58757321714607,48.458943739834,0
-0.58743602135117,48.458972425785,0
-0.58730311751433,48.459008932048,0
-0.58717551706414,48.459052980806,0
-0.58705419107665,48.45910423684,0
-0.58694006288611,48.459162310081,0
-0.58683400105863,48.459226758581,0
-0.58673681278269,48.45929709187,0
-0.58664923772587,48.459372774693,0
-0.58657194240547,48.4594532310 8,0
-0.58650551511534,48.459537848731,0
-0.58645046144772,48.459625983673,0
-0.58640720044411,48.459716965164,0
-0.58637606140461,48.459810100792,0
-0.58635728138002,48.459904681748,0
-0.58635100336578,48.459999988222,0
-0.58799999999997,48.46,0
</coordinates>
</LinearRing>
</outerBoundaryIs>
</Polygon>
</Placemark>
<Placemark>
<name>296</name>
<styleUrl>#stylehap1</styleUrl>
<Polygon>
<outerBoundaryIs>
<LinearRing>
<coordinates>
-0.58600000000001,48.272,0
-0.58435707911792,48.271999988299,0

-0.58436332787528,48.272095294951,0
-0.58438203276525,48.272189876437,0
-0.58441305149973,48.27228301293,0
-0.5844561480707,48.272373995594,0
-0.58451099454437,48.272462131984,0
-0.58457717355533,48.272546751311,0
-0.58465418148138,48.272627209553,0
-0.5847414322752,48.272702894352,0
-0.5848382619236,48.27277322968,0
-0.58494393350063,48.272837680218,0
-0.58505764277566,48.272895755436,0
-0.58517852433439,48.272947013325,0
-0.58530565816569,48.272991063759,0
-0.58543807666434,48.273027571469,0
-0.58557477199619,48.273056258593,0
-0.5857147037701,48.273076906792,0
-0.58585680695755,48.27308935891,0
-0.58600000000002,48.273093520174,0
-0.58614319304247,48.27308935891,0
-0.58628529622992,48.273076906792,0
-0.58642522800384,48.273056258593,0
-0.58656192333569,48.273027571469,0
-0.58669434183433,48.272991063759,0
-0.58682147566563,48.272947013325,0
-0.58694235722436,48.272895755436,0
-0.5870560664994,48.272837680218,0
-0.58716173807642,48.27277322968,0
-0.58725856772482,48.272702894352,0
-0.58734581851865,48.272627209553,0
-0.58742282644469,48.272546751311,0
-0.58748900545565,48.272462131984,0
-0.58754385192933,48.272373995594,0
-0.5875869485003,48.27228301293,0
-0.5876179672348,48.272189876437,0
-0.58763667212474,48.272095294951,0
-0.58764292088211,48.271999988299,0
-0.58763666601953,48.271904681825,0
-0.58761795520984,48.271810100867,0
-0.58758693092099,48.271716965236,0
-0.58754382932983,48.271625983742,0
-0.58748897852259,48.271537848794,0
-0.58742279599645,48.271453231138,0
-0.58734578548037,48.271372774745,0
-0.58725853310036,48.271297091915,0
-0.58716170291781,48.27122675862,0
-0.58705603187494,48.271162310113,0
-0.58694232418609,48.271104236865,0
-0.58682144521739,48.271052980825,0
-0.58669431490127,48.271008932062,0
-0.58656190073619,48.270972425794,0
-0.58642521042453,48.270943739839,0
-0.58628528420496,48.270923092502,0
-0.58614318693726,48.270910640912,0
-0.58600000000002,48.270906479827,0
-0.58585681306276,48.270910640912,0
-0.58571471579507,48.270923092502,0
-0.58557478957549,48.270943739839,0

-0.58543809926386,48.270972425794,0
-0.58530568509875,48.271008932062,0
-0.58517855478263,48.271052980825,0
-0.58505767581394,48.271104236865,0
-0.58494396812509,48.271162310113,0
-0.58483829708221,48.27122675862,0
-0.58474146689966,48.271297091915,0
-0.58465421451966,48.271372774745,0
-0.58457720400357,48.271453231138,0
-0.58451102147744,48.271537848794,0
-0.58445617067022,48.271625983742,0
-0.58441306907904,48.271716965236,0
-0.58438204479019,48.271810100867,0
-0.58436333398049,48.271904681825,0
-0.58435707911792,48.271999988299,0
-0.58600000000001,48.272,0
</coordinates>
</LinearRing>
</outerBoundaryIs>
</Polygon>
</Placemark>
<Placemark>
<name>299</name>
<styleUrl>#stylehap1</styleUrl>
<Polygon>
<outerBoundaryIs>
<LinearRing>
<coordinates>
-0.58500000000004,48.12,0
-0.58336194593996,48.119999988362,0
-0.58336817620283,48.120095295013,0
-0.58338682569904,48.120189876498,0
-0.58341775256154,48.120283012988,0
-0.58346072148092,48.120373995649,0
-0.58351540549448,48.120462132035,0
-0.5835813884728,48.120546751358,0
-0.58365816828524,48.120627209595,0
-0.58374516062037,48.120702894389,0
-0.58384170343189,48.120773229711,0
-0.58394706197674,48.120837680244,0
-0.58406043440674,48.120895755457,0
-0.58418095787135,48.12094701334,0
-0.58430771508497,48.12099106377,0
-0.58443974130899,48.121027571476,0
-0.58457603169514,48.121056258598,0
-0.58471554893464,48.121076906794,0
-0.5848572311543,48.121089358911,0
-0.58500000000004,48.121093520173,0
-0.58514276884578,48.121089358911,0
-0.58528445106544,48.121076906794,0
-0.58542396830492,48.121056258598,0
-0.58556025869109,48.121027571476,0
-0.58569228491508,48.12099106377,0
-0.58581904212871,48.12094701334,0
-0.58593956559332,48.120895755457,0
-0.58605293802334,48.120837680244,0
-0.58615829656819,48.120773229711,0

-0.58625483937969,48.120702894389,0
-0.58634183171482,48.120627209595,0
-0.58641861152728,48.120546751358,0
-0.5864845945056,48.120462132035,0
-0.58653927851916,48.120373995649,0
-0.58658224743854,48.120283012988,0
-0.58661317430102,48.120189876498,0
-0.58663182379725,48.120095295013,0
-0.58663805406009,48.119999988362,0
-0.58663181774252,48.119904681887,0
-0.58661316237554,48.119810100927,0
-0.58658223000465,48.119716965294,0
-0.58653925610658,48.119625983797,0
-0.58648456779532,48.119537848846,0
-0.58641858133089,48.119453231185,0
-0.58634179894981,48.119372774786,0
-0.58625480504161,48.119297091952,0
-0.58615826170038,48.119226758651,0
-0.58605290368525,48.119162310139,0
-0.58593953282831,48.119104236886,0
-0.58581901193232,48.119052980841,0
-0.5856922582048,48.119008932073,0
-0.58556023627851,48.118972425801,0
-0.58542395087103,48.118943739843,0
-0.58528443913994,48.118923092504,0
-0.58514276279105,48.118910640912,0
-0.58500000000004,48.118906479826,0
-0.58485723720903,48.118910640912,0
-0.58471556086014,48.118923092504,0
-0.58457604912905,48.118943739843,0
-0.58443976372157,48.118972425801,0
-0.58430774179527,48.119008932073,0
-0.58418098806776,48.119052980841,0
-0.58406046717177,48.119104236886,0
-0.58394709631483,48.119162310139,0
-0.5838417382997,48.119226758651,0
-0.58374519495845,48.119297091952,0
-0.58365820105027,48.119372774786,0
-0.58358141866919,48.119453231185,0
-0.58351543220476,48.119537848846,0
-0.5834607438935,48.119625983797,0
-0.58341776999543,48.119716965294,0
-0.58338683762454,48.119810100927,0
-0.58336818225756,48.119904681887,0
-0.58336194593996,48.119999988362,0
-0.58500000000004,48.12,0
</coordinates>
</LinearRing>
</outerBoundaryIs>
</Polygon>
</Placemark>
<Placemark>
<name>301</name>
<styleUrl>#stylehap1</styleUrl>
<Polygon>
<outerBoundaryIs>
<LinearRing>

<coordinates>
-0.58500000000004,48.223,0
-0.58335865244551,48.222999988319,0
-0.58336489522404,48.223095294971,0
-0.58338358220652,48.223189876457,0
-0.58341457124101,48.223283012949,0
-0.58345762654525,48.223373995612,0
-0.58351242049946,48.223462132,0
-0.58357853613775,48.223546751326,0
-0.58365547032011,48.223627209566,0
-0.58374263756038,48.223702894364,0
-0.58383937448126,48.22377322969,0
-0.58394494486243,48.223837680226,0
-0.58405854524351,48.223895755443,0
-0.58417931103894,48.22394701333,0
-0.58430632311873,48.223991063762,0
-0.5844386148043,48.224027571471,0
-0.58457517922686,48.224056258595,0
-0.5847149769917,48.224076906793,0
-0.58485694409026,48.22408935891,0
-0.58500000000004,48.224093520174,0
-0.58514305590979,48.22408935891,0
-0.58528502300838,48.224076906793,0
-0.58542482077319,48.224056258595,0
-0.58556138519578,48.224027571471,0
-0.58569367688135,48.223991063762,0
-0.58582068896114,48.22394701333,0
-0.58594145475657,48.223895755443,0
-0.58605505513763,48.223837680226,0
-0.58616062551882,48.22377322969,0
-0.5862573624397,48.223702894364,0
-0.58634452967997,48.223627209566,0
-0.58642146386233,48.223546751326,0
-0.58648757950062,48.223462132,0
-0.58654237345483,48.223373995612,0
-0.58658542875907,48.223283012949,0
-0.58661641779353,48.223189876457,0
-0.58663510477604,48.223095294971,0
-0.58664134755457,48.222999988319,0
-0.58663509868713,48.222904681845,0
-0.58661640580078,48.222810100886,0
-0.58658541122681,48.222716965255,0
-0.58654235091577,48.222625983759,0
-0.58648755263964,48.222537848811,0
-0.58642143349557,48.222453231153,0
-0.58634449673011,48.222372774758,0
-0.58625732790788,48.222297091927,0
-0.5861605904543,48.22222675863,0
-0.58605502060581,48.222162310122,0
-0.58594142180671,48.222104236872,0
-0.58582065859435,48.22205298083,0
-0.58569365002037,48.222008932065,0
-0.58556136265671,48.221972425796,0
-0.58542480324093,48.22194373984,0
-0.58528501101561,48.221923092503,0
-0.58514304982092,48.221910640912,0
-0.58500000000004,48.221906479827,0

-0.58485695017916,48.221910640912,0
-0.58471498898445,48.221923092503,0
-0.58457519675913,48.22194373984,0
-0.58443863734334,48.221972425796,0
-0.58430634997971,48.222008932065,0
-0.5841793414057,48.22205298083,0
-0.58405857819337,48.222104236872,0
-0.58394497939425,48.222162310122,0
-0.58383940954578,48.22222675863,0
-0.5837426720922,48.222297091927,0
-0.58365550326997,48.222372774758,0
-0.58357856650451,48.222453231153,0
-0.58351244736044,48.222537848811,0
-0.58345764908429,48.222625983759,0
-0.58341458877327,48.222716965255,0
-0.5833835941993,48.222810100886,0
-0.58336490131292,48.222904681845,0
-0.58335865244551,48.222999988319,0
-0.58500000000004,48.223,0
</coordinates>
</LinearRing>
</outerBoundaryIs>
</Polygon>
</Placemark>
<Placemark>
<name>303</name>
<styleUrl>#stylehap1</styleUrl>
<Polygon>
<outerBoundaryIs>
<LinearRing>
<coordinates>
-0.58500000000004,48.223,0
-0.58335865244551,48.222999988319,0
-0.58336489522404,48.223095294971,0
-0.58338358220652,48.223189876457,0
-0.58341457124101,48.223283012949,0
-0.58345762654525,48.223373995612,0
-0.58351242049946,48.223462132,0
-0.58357853613775,48.223546751326,0
-0.58365547032011,48.223627209566,0
-0.58374263756038,48.223702894364,0
-0.58383937448126,48.22377322969,0
-0.58394494486243,48.223837680226,0
-0.58405854524351,48.223895755443,0
-0.58417931103894,48.22394701333,0
-0.58430632311873,48.223991063762,0
-0.5844386148043,48.224027571471,0
-0.58457517922686,48.224056258595,0
-0.5847149769917,48.224076906793,0
-0.58485694409026,48.22408935891,0
-0.58500000000004,48.224093520174,0
-0.58514305590979,48.22408935891,0
-0.58528502300838,48.224076906793,0
-0.58542482077319,48.224056258595,0
-0.58556138519578,48.224027571471,0
-0.58569367688135,48.223991063762,0
-0.58582068896114,48.22394701333,0

-0.58594145475657,48.223895755443,0
-0.58605505513763,48.223837680226,0
-0.58616062551882,48.22377322969,0
-0.5862573624397,48.223702894364,0
-0.58634452967997,48.223627209566,0
-0.58642146386233,48.223546751326,0
-0.58648757950062,48.223462132,0
-0.58654237345483,48.223373995612,0
-0.58658542875907,48.223283012949,0
-0.58661641779353,48.223189876457,0
-0.58663510477604,48.223095294971,0
-0.58664134755457,48.222999988319,0
-0.58663509868713,48.222904681845,0
-0.58661640580078,48.222810100886,0
-0.58658541122681,48.222716965255,0
-0.58654235091577,48.222625983759,0
-0.58648755263964,48.222537848811,0
-0.58642143349557,48.222453231153,0
-0.58634449673011,48.222372774758,0
-0.58625732790788,48.222297091927,0
-0.5861605904543,48.22222675863,0
-0.58605502060581,48.222162310122,0
-0.58594142180671,48.222104236872,0
-0.58582065859435,48.22205298083,0
-0.58569365002037,48.222008932065,0
-0.58556136265671,48.221972425796,0
-0.58542480324093,48.22194373984,0
-0.58528501101561,48.221923092503,0
-0.58514304982092,48.221910640912,0
-0.58500000000004,48.221906479827,0
-0.58485695017916,48.221910640912,0
-0.58471498898445,48.221923092503,0
-0.58457519675913,48.22194373984,0
-0.58443863734334,48.221972425796,0
-0.58430634997971,48.222008932065,0
-0.5841793414057,48.22205298083,0
-0.58405857819337,48.222104236872,0
-0.58394497939425,48.222162310122,0
-0.58383940954578,48.22222675863,0
-0.5837426720922,48.222297091927,0
-0.58365550326997,48.222372774758,0
-0.58357856650451,48.222453231153,0
-0.58351244736044,48.222537848811,0
-0.58345764908429,48.222625983759,0
-0.58341458877327,48.222716965255,0
-0.5833835941993,48.222810100886,0
-0.58336490131292,48.222904681845,0
-0.58335865244551,48.222999988319,0
-0.58500000000004,48.223,0
</coordinates>
</LinearRing>
</outerBoundaryIs>
</Polygon>
</Placemark>
<Placemark>
<name>305</name>
<styleUrl>#stylehap1</styleUrl>

<Polygon>
<outerBoundaryIs>
<LinearRing>
<coordinates>
-0.58299999999997,48.384,0
-0.58135346705923,48.383999988253,0
-0.58135972954278,48.384095294905,0
-0.58137847554485,48.384189876393,0
-0.58140956246488,48.384283012887,0
-0.58145275377627,48.384373995554,0
-0.58150772082462,48.384462131946,0
-0.58157404532711,48.384546751277,0
-0.58165122255451,48.384627209522,0
-0.5817386651712,48.384702894325,0
-0.58183570770427,48.384773229657,0
-0.58194161160754,48.384837680199,0
-0.58205557088226,48.384895755421,0
-0.58217671821138,48.384947013313,0
-0.58230413156089,48.38499106375,0
-0.58243684119797,48.385027571464,0
-0.58257383707251,48.38505625859,0
-0.58271407650568,48.385076906791,0
-0.58285649212706,48.38508935891,0
-0.58299999999997,48.385093520173,0
-0.58314350787288,48.38508935891,0
-0.58328592349426,48.385076906791,0
-0.58342616292742,48.38505625859,0
-0.58356315880197,48.385027571464,0
-0.58369586843908,48.38499106375,0
-0.58382328178856,48.384947013313,0
-0.58394442911768,48.384895755421,0
-0.5840583883924,48.384837680199,0
-0.58416429229566,48.384773229657,0
-0.58426133482874,48.384702894325,0
-0.58434877744543,48.384627209522,0
-0.58442595467286,48.384546751277,0
-0.58449227917531,48.384462131946,0
-0.58454724622366,48.384373995554,0
-0.58459043753506,48.384283012887,0
-0.58462152445509,48.384189876393,0
-0.58464027045716,48.384095294905,0
-0.5846465329407,48.383999988253,0
-0.58464026431439,48.383904681779,0
-0.58462151235618,48.383810100822,0
-0.58459041984762,48.383716965193,0
-0.58454722348514,48.383625983701,0
-0.58449225207661,48.383537848756,0
-0.5844259240373,48.383453231103,0
-0.58434874420393,48.383372774714,0
-0.58426129999128,48.383297091888,0
-0.5841642569208,48.383226758597,0
-0.58405835355494,48.383162310094,0
-0.58394439587617,48.38310423685,0
-0.58382325115303,48.383052980814,0
-0.58369584134034,48.383008932053,0
-0.58356313606345,48.382972425788,0
-0.58342614524,48.382943739836,0

file:///H|/...dra_Joep_van_Belkom/___ScientificReports/REVISION%20STUFF/SupplementalInformation/SupplementaryInformation_I.txt[19/12/2019 11:32:01]

-0.58328591139535,48.382923092501,0
-0.58314350173008,48.382910640912,0
-0.58299999999997,48.382906479826,0
-0.58285649826985,48.382910640912,0
-0.58271408860459,48.382923092501,0
-0.58257385475993,48.382943739836,0
-0.58243686393649,48.382972425788,0
-0.58230415865959,48.383008932053,0
-0.58217674884691,48.383052980814,0
-0.58205560412377,48.38310423685,0
-0.581941646445,48.383162310094,0
-0.58183574307914,48.383226758597,0
-0.58173870000865,48.383297091888,0
-0.58165125579601,48.383372774714,0
-0.58157407596264,48.383453231103,0
-0.58150774792333,48.383537848756,0
-0.58145277651482,48.383625983701,0
-0.58140958015232,48.383716965193,0
-0.58137848764376,48.383810100822,0
-0.58135973568555,48.383904681779,0
-0.58135346705923,48.383999988253,0
-0.58299999999997,48.384,0
</coordinates>
</LinearRing>
</outerBoundaryIs>
</Polygon>
</Placemark>
<Placemark>
<name>307</name>
<styleUrl>#stylehap1</styleUrl>
<Polygon>
<outerBoundaryIs>
<LinearRing>
<coordinates>
-0.58199999999999,48.248,0
-0.5803578502541,48.247999988309,0
-0.58036409608102,48.248095294961,0
-0.58038279219401,48.248189876447,0
-0.5804137963716,48.248283012939,0
-0.58045687271648,48.248373995603,0
-0.58051169344875,48.248462131992,0
-0.5805778413988,48.248546751318,0
-0.58065481318082,48.248627209559,0
-0.58074202302248,48.248702894358,0
-0.58083880722228,48.248773229685,0
-0.58094442920011,48.248837680222,0
-0.58105808510284,48.248895755439,0
-0.58117890992244,48.248947013327,0
-0.58130598407958,48.24899106376,0
-0.58143834042324,48.24902757147,0
-0.58157497159244,48.249056258594,0
-0.58171483768436,48.249076906792,0
-0.58185687417047,48.24908935891,0
-0.58199999999998,48.249093520173,0
-0.58214312582953,48.24908935891,0
-0.5822851623156,48.249076906792,0
-0.58242502840755,48.249056258594,0

-0.58256165957675,48.24902757147,0
-0.58269401592041,48.24899106376,0
-0.58282109007756,48.248947013327,0
-0.58294191489713,48.248895755439,0
-0.58305557079988,48.248837680222,0
-0.58316119277771,48.248773229685,0
-0.58325797697751,48.248702894358,0
-0.58334518681918,48.248627209559,0
-0.58342215860117,48.248546751318,0
-0.58348830655124,48.248462131992,0
-0.58354312728351,48.248373995603,0
-0.58358620362839,48.248283012939,0
-0.58361720780598,48.248189876447,0
-0.58363590391894,48.248095294961,0
-0.5836421497459,48.247999988309,0
-0.58363589782174,48.247904681835,0
-0.58361719579682,48.247810100876,0
-0.58358618607214,48.247716965245,0
-0.58354310471363,48.24762598375,0
-0.58348827965352,48.247537848802,0
-0.58342212819288,48.247453231145,0
-0.58334515382423,48.247372774751,0
-0.58325794239847,48.247297091921,0
-0.58316115766523,48.247226758625,0
-0.58305553622084,48.247162310117,0
-0.58294188190218,48.247104236868,0
-0.58282105966924,48.247052980828,0
-0.58269398902269,48.247008932063,0
-0.58256163700687,48.246972425795,0
-0.5824250108513,48.24694373984,0
-0.58228515030644,48.246923092503,0
-0.5821431197323,48.246910640912,0
-0.58199999999998,48.246906479826,0
-0.58185688026769,48.246910640912,0
-0.58171484969355,48.246923092503,0
-0.58157498914867,48.24694373984,0
-0.58143836299312,48.246972425795,0
-0.5813060109773,48.247008932063,0
-0.58117894033075,48.247052980828,0
-0.58105811809779,48.247104236868,0
-0.58094446377915,48.247162310117,0
-0.58083884233476,48.247226758625,0
-0.58074205760152,48.247297091921,0
-0.58065484617576,48.247372774751,0
-0.58057787180712,48.247453231145,0
-0.58051172034648,48.247537848802,0
-0.58045689528636,48.24762598375,0
-0.58041381392783,48.247716965245,0
-0.58038280420317,48.247810100876,0
-0.58036410217825,48.247904681835,0
-0.5803578502541,48.247999988309,0
-0.58199999999999,48.248,0
</coordinates>
</LinearRing>
</outerBoundaryIs>
</Polygon>
</Placemark>

<Placemark>
<name>311</name>
<styleUrl>#stylehap1</styleUrl>
<Polygon>
<outerBoundaryIs>
<LinearRing>
<coordinates>
-0.58100000000002,48.12,0
-0.57936194593995,48.119999988362,0
-0.57936817620282,48.120095295013,0
-0.57938682569902,48.120189876498,0
-0.5794177525615,48.120283012988,0
-0.57946072148091,48.120373995649,0
-0.57951540549444,48.120462132035,0
-0.57958138847276,48.120546751358,0
-0.57965816828522,48.120627209595,0
-0.57974516062035,48.120702894389,0
-0.57984170343188,48.120773229711,0
-0.57994706197673,48.120837680244,0
-0.58006043440672,48.120895755457,0
-0.58018095787133,48.12094701334,0
-0.58030771508496,48.12099106377,0
-0.58043974130897,48.121027571476,0
-0.58057603169512,48.121056258598,0
-0.58071554893462,48.121076906794,0
-0.58085723115429,48.121089358911,0
-0.58100000000002,48.121093520173,0
-0.58114276884576,48.121089358911,0
-0.5812844510654,48.121076906794,0
-0.5814239683049,48.121056258598,0
-0.58156025869108,48.121027571476,0
-0.58169228491507,48.12099106377,0
-0.58181904212869,48.12094701334,0
-0.5819395655933,48.120895755457,0
-0.58205293802332,48.120837680244,0
-0.58215829656817,48.120773229711,0
-0.58225483937967,48.120702894389,0
-0.5823418317148,48.120627209595,0
-0.58241861152727,48.120546751358,0
-0.58248459450558,48.120462132035,0
-0.58253927851914,48.120373995649,0
-0.58258224743852,48.120283012988,0
-0.582613174301,48.120189876498,0
-0.58263182379721,48.120095295013,0
-0.58263805406008,48.119999988362,0
-0.58263181774248,48.119904681887,0
-0.58261316237553,48.119810100927,0
-0.58258223000463,48.119716965294,0
-0.58253925610654,48.119625983797,0
-0.58248456779528,48.119537848846,0
-0.58241858133088,48.119453231185,0
-0.5823417989498,48.119372774786,0
-0.58225480504159,48.119297091952,0
-0.58215826170036,48.119226758651,0
-0.58205290368524,48.119162310139,0
-0.5819395328283,48.119104236886,0
-0.5818190119323,48.119052980841,0

-0.5816922582048,48.119008932073,0
-0.58156023627847,48.118972425801,0
-0.58142395087101,48.118943739843,0
-0.58128443913993,48.118923092504,0
-0.58114276279103,48.118910640912,0
-0.58100000000002,48.118906479826,0
-0.58085723720902,48.118910640912,0
-0.5807155608601,48.118923092504,0
-0.58057604912904,48.118943739843,0
-0.58043976372155,48.118972425801,0
-0.58030774179525,48.119008932073,0
-0.58018098806775,48.119052980841,0
-0.58006046717175,48.119104236886,0
-0.57994709631481,48.119162310139,0
-0.57984173829966,48.119226758651,0
-0.57974519495843,48.119297091952,0
-0.57965820105025,48.119372774786,0
-0.57958141866917,48.119453231185,0
-0.57951543220474,48.119537848846,0
-0.57946074389349,48.119625983797,0
-0.57941776999542,48.119716965294,0
-0.57938683762452,48.119810100927,0
-0.57936818225755,48.119904681887,0
-0.57936194593995,48.119999988362,0
-0.58100000000002,48.12,0
</coordinates>
</LinearRing>
</outerBoundaryIs>
</Polygon>
</Placemark>
<Placemark>
<name>312</name>
<styleUrl>#stylehap1</styleUrl>
<Polygon>
<outerBoundaryIs>
<LinearRing>
<coordinates>
-0.58000000000004,48.39,0
-0.57835327293018,48.389999988251,0
-0.57835953615144,48.390095294903,0
-0.57837828436305,48.39018987639,0
-0.57840937494771,48.390283012885,0
-0.5784525713509,48.390373995551,0
-0.57850754487949,48.390462131944,0
-0.57857387720138,48.390546751275,0
-0.57865106352784,48.39062720952,0
-0.578738516454,48.390702894324,0
-0.5788355704285,48.390773229655,0
-0.57894148681809,48.390837680198,0
-0.57905545952898,48.39089575542,0
-0.57917662114183,48.390947013312,0
-0.57930404951397,48.39099106375,0
-0.57943677479821,48.391027571463,0
-0.57957378682534,48.39105625859,0
-0.5797140427936,48.391076906791,0
-0.57985647520665,48.39108935891,0
-0.58000000000004,48.391093520174,0

-0.58014352479343,48.39108935891,0
-0.5802859572065,48.391076906791,0
-0.58042621317474,48.39105625859,0
-0.58056322520187,48.391027571463,0
-0.58069595048611,48.39099106375,0
-0.58082337885825,48.390947013312,0
-0.5809445404711,48.39089575542,0
-0.58105851318199,48.390837680198,0
-0.58116442957158,48.390773229655,0
-0.58126148354608,48.390702894324,0
-0.58134893647224,48.39062720952,0
-0.5814261227987,48.390546751275,0
-0.58149245512059,48.390462131944,0
-0.58154742864918,48.390373995551,0
-0.58159062505237,48.390283012885,0
-0.58162171563703,48.39018987639,0
-0.58164046384865,48.390095294903,0
-0.5816467270699,48.389999988251,0
-0.58164045770386,48.389904681777,0
-0.58162170353413,48.38981010082,0
-0.58159060735912,48.389716965191,0
-0.58154740590318,48.389625983699,0
-0.58149242801295,48.389537848754,0
-0.58142609215309,48.389453231101,0
-0.58134890321982,48.389372774712,0
-0.58126144869717,48.389297091887,0
-0.58116439418508,48.389226758595,0
-0.58105847833308,48.389162310093,0
-0.58094450721866,48.389104236849,0
-0.58082334821263,48.389052980813,0
-0.58069592337847,48.389008932053,0
-0.58056320245587,48.388972425788,0
-0.58042619548147,48.388943739836,0
-0.5802859451036,48.388923092501,0
-0.58014351864862,48.388910640912,0
-0.58000000000004,48.388906479827,0
-0.57985648135146,48.388910640912,0
-0.57971405489648,48.388923092501,0
-0.57957380451862,48.388943739836,0
-0.57943679754421,48.388972425788,0
-0.57930407662161,48.389008932053,0
-0.57917665178747,48.389052980813,0
-0.57905549278142,48.389104236849,0
-0.578941521667,48.389162310093,0
-0.578835605815,48.389226758595,0
-0.57873855130291,48.389297091887,0
-0.57865109678026,48.389372774712,0
-0.57857390784699,48.389453231101,0
-0.57850757198713,48.389537848754,0
-0.5784525940969,48.389625983699,0
-0.57840939264096,48.389716965191,0
-0.57837829646595,48.38981010082,0
-0.57835954229624,48.389904681777,0
-0.57835327293018,48.389999988251,0
-0.58000000000004,48.39,0
</coordinates>
</LinearRing>

</outerBoundaryIs>
</Polygon>
</Placemark>
<Placemark>
<name>314</name>
<styleUrl>#stylehap1</styleUrl>
<Polygon>
<outerBoundaryIs>
<LinearRing>
<coordinates>
-0.58000000000004,48.247,0
-0.57835788236284,48.24699998831,0
-0.57836412806779,48.247095294961,0
-0.57838282381529,48.247189876447,0
-0.57841382738675,48.24728301294,0
-0.57845690288947,48.247373995603,0
-0.57851172254989,48.247462131992,0
-0.57857786920665,48.247546751319,0
-0.57865483948368,48.24762720956,0
-0.57874204762016,48.247702894358,0
-0.57883882992758,48.247773229685,0
-0.57894444984019,48.247837680222,0
-0.5790581035206,48.247895755439,0
-0.57917892597768,48.247947013327,0
-0.5793059976501,48.24799106376,0
-0.57943835140573,48.24802757147,0
-0.57957497990331,48.248056258594,0
-0.57971484326036,48.248076906792,0
-0.57985687696916,48.24808935891,0
-0.58000000000004,48.248093520173,0
-0.58014312303092,48.24808935891,0
-0.58028515673972,48.248076906792,0
-0.58042502009677,48.248056258594,0
-0.58056164859435,48.24802757147,0
-0.58069400234998,48.24799106376,0
-0.5808210740224,48.247947013327,0
-0.58094189647948,48.247895755439,0
-0.58105555015989,48.247837680222,0
-0.5811611700725,48.247773229685,0
-0.58125795237992,48.247702894358,0
-0.5813451605164,48.24762720956,0
-0.58142213079343,48.247546751319,0
-0.58148827745019,48.247462131992,0
-0.58154309711061,48.247373995603,0
-0.58158617261333,48.24728301294,0
-0.58161717618479,48.247189876447,0
-0.58163587193231,48.247095294961,0
-0.58164211763724,48.24699998831,0
-0.58163586583542,48.246904681835,0
-0.58161716417629,48.246810100877,0
-0.58158615505805,48.246716965246,0
-0.58154307454198,48.246625983751,0
-0.58148825055392,48.246537848803,0
-0.58142210038679,48.246453231146,0
-0.58134512752326,48.246372774752,0
-0.58125791780276,48.246297091921,0
-0.58116113496195,48.246226758625,0

-0.58105551558274,48.246162310118,0
-0.58094186348634,48.246104236868,0
-0.58082104361576,48.246052980828,0
-0.58069397545374,48.246008932064,0
-0.58056162602572,48.245972425795,0
-0.58042500254148,48.24594373984,0
-0.58028514473119,48.245923092503,0
-0.58014311693405,48.245910640912,0
-0.58000000000004,48.245906479826,0
-0.57985688306603,48.245910640912,0
-0.57971485526889,48.245923092503,0
-0.5795749974586,48.24594373984,0
-0.57943837397436,48.245972425795,0
-0.57930602454634,48.246008932064,0
-0.57917895638432,48.246052980828,0
-0.57905813651374,48.246104236868,0
-0.57894448441734,48.246162310118,0
-0.57883886503813,48.246226758625,0
-0.57874208219732,48.246297091921,0
-0.57865487247682,48.246372774752,0
-0.57857789961329,48.246453231146,0
-0.57851174944617,48.246537848803,0
-0.5784569254581,48.246625983751,0
-0.57841384494203,48.246716965246,0
-0.5783828358238,48.246810100877,0
-0.57836413416466,48.246904681835,0
-0.57835788236284,48.24699998831,0
-0.58000000000004,48.247,0
</coordinates>
</LinearRing>
</outerBoundaryIs>
</Polygon>
</Placemark>
<Placemark>
<name>315</name>
<styleUrl>#stylehap1</styleUrl>
<Polygon>
<outerBoundaryIs>
<LinearRing>
<coordinates>
-0.578999999999995,48.45,0
-0.57735132812289,48.449999988226,0
-0.57735759873461,48.450095294878,0
-0.57737636908181,48.450189876366,0
-0.57740749637896,48.450283012862,0
-0.57745074379236,48.45037399553,0
-0.57750578224083,48.450462131924,0
-0.57757219289836,48.450546751256,0
-0.57764947038018,48.450627209504,0
-0.57773702658776,48.450702894309,0
-0.57783419518375,48.450773229643,0
-0.57794023666262,48.450837680187,0
-0.57805434397852,48.450895755412,0
-0.57817564868766,48.450947013306,0
-0.57830322755818,48.450991063746,0
-0.57843610959742,48.45102757146,0
-0.57857328344296,48.451056258588,0

-0.57871370506133,48.45107690679,0
-0.57885630569532,48.45108935891,0
-0.57899999999995,48.451093520174,0
-0.57914369430456,48.45108935891,0
-0.57928629493858,48.45107690679,0
-0.57942671655692,48.451056258588,0
-0.57956389040248,48.45102757146,0
-0.57969677244171,48.450991063746,0
-0.57982435131224,48.450947013306,0
-0.57994565602139,48.450895755412,0
-0.58005976333729,48.450837680187,0
-0.58016580481616,48.450773229643,0
-0.58026297341215,48.450702894309,0
-0.58035052961973,48.450627209504,0
-0.58042780710155,48.450546751256,0
-0.58049421775908,48.450462131924,0
-0.58054925620754,48.45037399553,0
-0.58059250362093,48.450283012862,0
-0.58062363091807,48.450189876366,0
-0.5806424012653,48.450095294878,0
-0.58064867187702,48.449999988226,0
-0.58064239510024,48.449904681752,0
-0.58062361877529,48.449810100796,0
-0.58059248586938,48.449716965168,0
-0.58054923338658,48.449625983677,0
-0.5804941905621,48.449537848734,0
-0.58042777635495,48.449453231083,0
-0.58035049625772,48.449372774695,0
-0.58026293844842,48.449297091872,0
-0.58016576931306,48.449226758583,0
-0.58005972837355,48.449162310083,0
-0.57994562265938,48.449104236841,0
-0.57982432056564,48.449052980807,0
-0.57969674524476,48.449008932049,0
-0.57956386758152,48.448972425785,0
-0.57942669880538,48.448943739834,0
-0.57928628279578,48.4489230925,0
-0.57914368813953,48.448910640911,0
-0.57899999999995,48.448906479826,0
-0.57885631186038,48.448910640911,0
-0.5787137172041,48.4489230925,0
-0.57857330119453,48.448943739834,0
-0.57843613241836,48.448972425785,0
-0.57830325475515,48.449008932049,0
-0.57817567943426,48.449052980807,0
-0.57805437734053,48.449104236841,0
-0.57794027162635,48.449162310083,0
-0.57783423068685,48.449226758583,0
-0.57773706155149,48.449297091872,0
-0.57764950374216,48.449372774695,0
-0.57757222364493,48.449453231083,0
-0.57750580943778,48.449537848734,0
-0.57745076661333,48.449625983677,0
-0.57740751413052,48.449716965168,0
-0.57737638122461,48.449810100796,0
-0.57735760489964,48.449904681752,0
-0.57735132812289,48.449999988226,0

-0.57899999999995,48.45,0
</coordinates>
</LinearRing>
</outerBoundaryIs>
</Polygon>
</Placemark>
<Placemark>
<name>317</name>
<styleUrl>#stylehap1</styleUrl>
<Polygon>
<outerBoundaryIs>
<LinearRing>
<coordinates>
-0.57600000000002,47.89,0
-0.57436923389355,47.889999988455,0
-0.57437543646127,47.890095295106,0
-0.5743940030065,47.890189876589,0
-0.57442479229269,47.890283013076,0
-0.57446757005669,47.890373995732,0
-0.5745220107899,47.890462132112,0
-0.57458770021378,47.890546751428,0
-0.57466413843136,47.890627209657,0
-0.57475074373054,47.890702894444,0
-0.57484685701042,47.890773229758,0
-0.57495174679683,47.890837680282,0
-0.57506461480922,47.890895755487,0
-0.57518460203618,47.890947013364,0
-0.57531079527357,47.890991063787,0
-0.57544223407547,47.891027571487,0
-0.57557791806488,47.891056258604,0
-0.57571681454875,47.891076906797,0
-0.57585786637899,47.891089358911,0
-0.57600000000003,47.891093520173,0
-0.57614213362103,47.891089358911,0
-0.5762831854513,47.891076906797,0
-0.57642208193515,47.891056258604,0
-0.57655776592458,47.891027571487,0
-0.57668920472645,47.890991063787,0
-0.57681539796387,47.890947013364,0
-0.57693538519083,47.890895755487,0
-0.57704825320322,47.890837680282,0
-0.57715314298963,47.890773229758,0
-0.57724925626948,47.890702894444,0
-0.57733586156869,47.890627209657,0
-0.57741229978627,47.890546751428,0
-0.57747798921015,47.890462132112,0
-0.57753242994336,47.890373995732,0
-0.57757520770736,47.890283013076,0
-0.57760599699352,47.890189876589,0
-0.57762456353878,47.890095295106,0
-0.57763076610647,47.889999988455,0
-0.57762455755946,47.88990468198,0
-0.57760598521657,47.889810101018,0
-0.57757519049056,47.889716965382,0
-0.57753240780988,47.889625983879,0
-0.57747796283251,47.889537848922,0
-0.57741226996592,47.889453231255,0

-0.57733582921171,47.889372774849,0
-0.57724922235904,47.889297092007,0
-0.57715310855605,47.889226758698,0
-0.57704821929278,47.889162310178,0
-0.57693535283385,47.889104236916,0
-0.57681536814352,47.889052980864,0
-0.57668917834882,47.88900893209,0
-0.5765577437911,47.888972425812,0
-0.57642206471837,47.88894373985,0
-0.57628317367431,47.888923092507,0
-0.57614212764172,47.888910640913,0
-0.57600000000003,47.888906479827,0
-0.57585787235833,47.888910640913,0
-0.57571682632574,47.888923092507,0
-0.57557793528168,47.88894373985,0
-0.57544225620895,47.888972425812,0
-0.57531082165121,47.88900893209,0
-0.57518463185653,47.889052980864,0
-0.5750646471662,47.889104236916,0
-0.57495178070727,47.889162310178,0
-0.57484689144397,47.889226758698,0
-0.57475077764098,47.889297092007,0
-0.57466417078832,47.889372774849,0
-0.57458773003413,47.889453231255,0
-0.57452203716754,47.889537848922,0
-0.57446759219017,47.889625983879,0
-0.57442480950946,47.889716965382,0
-0.57439401478348,47.889810101018,0
-0.57437544244059,47.88990468198,0
-0.57436923389355,47.889999988455,0
-0.57600000000002,47.89,0
</coordinates>
</LinearRing>
</outerBoundaryIs>
</Polygon>
</Placemark>
<Placemark>
<name>318</name>
<styleUrl>#stylehap1</styleUrl>
<Polygon>
<outerBoundaryIs>
<LinearRing>
<coordinates>
-0.57500000000005,48.442,0
-0.57335158780046,48.441999988229,0
-0.57335785742536,48.442095294881,0
-0.57337662481697,48.442189876369,0
-0.57340774721214,48.442283012865,0
-0.57345098781448,48.442373995532,0
-0.57350601759461,48.442462131926,0
-0.57357241779249,48.442546751259,0
-0.57364968310291,48.442627209506,0
-0.57373722551999,48.442702894311,0
-0.57383437881132,48.442773229645,0
-0.57394040358783,48.442837680189,0
-0.5740544929308,48.442895755413,0
-0.57417577853323,48.442947013307,0

-0.5743033373087,48.442991063746,0
-0.57443619841745,48.443027571461,0
-0.57457335065644,48.443056258589,0
-0.57471375015662,48.44307690679,0
-0.57485632832915,48.44308935891,0
-0.57500000000004,48.443093520173,0
-0.57514367167093,48.44308935891,0
-0.57528624984349,48.44307690679,0
-0.57542664934367,48.443056258589,0
-0.57556380158265,48.443027571461,0
-0.57569666269141,48.442991063746,0
-0.57582422146685,48.442947013307,0
-0.57594550706928,48.442895755413,0
-0.57605959641225,48.442837680189,0
-0.57616562118876,48.442773229645,0
-0.5762627744801,48.442702894311,0
-0.57635031689717,48.442627209506,0
-0.57642758220762,48.442546751259,0
-0.5764939824055,48.442462131926,0
-0.57654901218563,48.442373995532,0
-0.57659225278797,48.442283012865,0
-0.57662337518311,48.442189876369,0
-0.57664214257472,48.442095294881,0
-0.57664841219962,48.441999988229,0
-0.57664213641238,48.441904681756,0
-0.57662336304568,48.441810100799,0
-0.57659223504419,48.441716965171,0
-0.57654898937467,48.44162598368,0
-0.57649395522046,48.441537848737,0
-0.5764275514745,48.441453231085,0
-0.57635028354982,48.441372774698,0
-0.5762627395317,48.441297091874,0
-0.57616558570122,48.441226758585,0
-0.57605956146386,48.441162310084,0
-0.5759454737219,48.441104236842,0
-0.57582419073373,48.441052980808,0
-0.57569663550637,48.441008932049,0
-0.57556377877169,48.440972425785,0
-0.57542663159989,48.440943739834,0
-0.57528623770603,48.4409230925,0
-0.57514366550859,48.440910640911,0
-0.57500000000004,48.440906479826,0
-0.57485633449151,48.440910640911,0
-0.57471376229407,48.4409230925,0
-0.57457336840019,48.440943739834,0
-0.57443622122839,48.440972425785,0
-0.57430336449371,48.441008932049,0
-0.57417580926635,48.441052980808,0
-0.57405452627818,48.441104236842,0
-0.57394043853622,48.441162310084,0
-0.57383441429886,48.441226758585,0
-0.5737372604684,48.441297091874,0
-0.57364971645029,48.441372774698,0
-0.57357244852558,48.441453231085,0
-0.57350604477963,48.441537848737,0
-0.57345101062542,48.44162598368,0
-0.57340776495589,48.441716965171,0

-0.57337663695443,48.441810100799,0
-0.5733578635877,48.441904681756,0
-0.57335158780046,48.441999988229,0
-0.57500000000005,48.442,0
</coordinates>
</LinearRing>
</outerBoundaryIs>
</Polygon>
</Placemark>
<Placemark>
<name>319</name>
<styleUrl>#stylehap1</styleUrl>
<Polygon>
<outerBoundaryIs>
<LinearRing>
<coordinates>
-0.57500000000005,48.014,0
-0.57336531608496,48.013999988405,0
-0.57337153354086,48.014095295056,0
-0.57339014467833,48.01418987654,0
-0.57342100792185,48.014283013029,0
-0.57346388844588,48.014373995688,0
-0.5735184599601,48.014462132071,0
-0.57358430719099,48.01454675139,0
-0.57366092904085,48.014627209624,0
-0.57374774240019,48.014702894414,0
-0.57384408658477,48.014773229732,0
-0.5739492283615,48.014837680261,0
-0.57406236753693,48.014895755471,0
-0.57418264303089,48.014947013351,0
-0.57430913944679,48.014991063778,0
-0.57444089403106,48.015027571481,0
-0.5745769040031,48.0150562586,0
-0.57471613418856,48.015076906795,0
-0.57485752489926,48.015089358911,0
-0.57500000000004,48.015093520174,0
-0.57514247510084,48.015089358911,0
-0.57528386581153,48.015076906795,0
-0.57542309599699,48.0150562586,0
-0.57555910596905,48.015027571481,0
-0.57569086055331,48.014991063778,0
-0.57581735696921,48.014947013351,0
-0.57593763246315,48.014895755471,0
-0.57605077163694,48.014837680261,0
-0.57615591341533,48.014773229732,0
-0.5762522575999,48.014702894414,0
-0.57633907095926,48.014627209624,0
-0.57641569280909,48.01454675139,0
-0.57648154003999,48.014462132071,0
-0.5765361115542,48.014373995688,0
-0.57657899207823,48.014283013029,0
-0.57660985532175,48.01418987654,0
-0.57662846645925,48.014095295056,0
-0.57663468391514,48.013999988405,0
-0.57662846043943,48.01390468193,0
-0.57660984346503,48.013810100969,0
-0.57657897474482,48.013716965335,0

-0.5765360892708,48.013625983835,0
-0.57648151348368,48.013537848881,0
-0.57641566278677,48.013453231217,0
-0.5763390383831,48.013372774815,0
-0.57625222345977,48.013297091977,0
-0.57615587874851,48.013226758672,0
-0.57605073749678,48.013162310157,0
-0.57593759988699,48.0131042369,0
-0.57581732694686,48.013052980851,0
-0.57569083399698,48.013008932081,0
-0.57555908368565,48.012972425806,0
-0.57542307866357,48.012943739846,0
-0.5752838539548,48.012923092505,0
-0.57514246908102,48.012910640913,0
-0.57500000000004,48.012906479826,0
-0.57485753091909,48.012910640913,0
-0.57471614604531,48.012923092505,0
-0.57457692133651,48.012943739846,0
-0.57444091631446,48.012972425806,0
-0.5743091660031,48.013008932081,0
-0.57418267305322,48.013052980851,0
-0.57406240011309,48.0131042369,0
-0.5739492625033,48.013162310157,0
-0.57384412125157,48.013226758672,0
-0.57374777654034,48.013297091977,0
-0.57366096161698,48.013372774815,0
-0.57358433721334,48.013453231217,0
-0.57351848651643,48.013537848881,0
-0.57346391072928,48.013625983835,0
-0.57342102525526,48.013716965335,0
-0.57339015653508,48.013810100969,0
-0.57337153956068,48.01390468193,0
-0.57336531608496,48.013999988405,0
-0.57500000000005,48.014,0
</coordinates>
</LinearRing>
</outerBoundaryIs>
</Polygon>
</Placemark>
<Placemark>
<name>320</name>
<styleUrl>#stylehap1</styleUrl>
<Polygon>
<outerBoundaryIs>
<LinearRing>
<coordinates>
-0.57500000000005,48.013,0
-0.57336534778611,48.012999988405,0
-0.57337156512155,48.013095295056,0
-0.5733901758982,48.01318987654,0
-0.57342103854332,48.013283013029,0
-0.57346391823585,48.013373995688,0
-0.57351848869185,48.013462132071,0
-0.57358433464582,48.013546751391,0
-0.57366095500979,48.013627209624,0
-0.57374776668563,48.013702894414,0
-0.57384410900181,48.013773229733,0

-0.57394924874119,48.013837680262,0
-0.57406238572087,48.013895755471,0
-0.5741826588823,48.013947013351,0
-0.574309152845,48.013991063778,0
-0.57444090487411,48.014027571481,0
-0.57457691220844,48.0140562586,0
-0.57471613969374,48.014076906795,0
-0.57485752766235,48.014089358911,0
-0.57500000000004,48.014093520174,0
-0.57514247233773,48.014089358911,0
-0.57528386030637,48.014076906795,0
-0.57542308779164,48.0140562586,0
-0.575559095126,48.014027571481,0
-0.57569084715509,48.013991063778,0
-0.57581734111781,48.013947013351,0
-0.57593761427923,48.013895755471,0
-0.57605075125889,48.013837680262,0
-0.57615589099828,48.013773229733,0
-0.57625223331448,48.013702894414,0
-0.57633904499029,48.013627209624,0
-0.57641566535427,48.013546751391,0
-0.57648151130825,48.013462132071,0
-0.57653608176423,48.013373995688,0
-0.57657896145679,48.013283013029,0
-0.57660982410189,48.01318987654,0
-0.57662843487853,48.013095295056,0
-0.57663465221397,48.012999988405,0
-0.57662842885904,48.01290468193,0
-0.57660981224577,48.01281010097,0
-0.57657894412432,48.012716965335,0
-0.57653605948205,48.012625983835,0
-0.57648148475337,48.012537848881,0
-0.57641563533357,48.012453231217,0
-0.57633901241591,48.012372774816,0
-0.57625219917618,48.012297091978,0
-0.57615585633336,48.012226758673,0
-0.5760507171206,48.012162310157,0
-0.57593758170486,48.0121042369,0
-0.57581731109709,48.012052980852,0
-0.57569082060023,48.012008932081,0
-0.5755590728438,48.011972425806,0
-0.57542307045919,48.011943739846,0
-0.57528384845025,48.011923092505,0
-0.57514246631824,48.011910640913,0
-0.57500000000004,48.011906479826,0
-0.57485753368184,48.011910640913,0
-0.57471615154983,48.011923092505,0
-0.57457692954091,48.011943739846,0
-0.57444092715628,48.011972425806,0
-0.57430917939988,48.012008932081,0
-0.57418268890299,48.012052980852,0
-0.57406241829525,48.0121042369,0
-0.57394928287948,48.012162310157,0
-0.57384414366675,48.012226758673,0
-0.5737478008239,48.012297091978,0
-0.57366098758417,48.012372774816,0
-0.57358436466654,48.012453231217,0

-0.57351851524671,48.012537848881,0
-0.57346394051803,48.012625983835,0
-0.57342105587576,48.012716965335,0
-0.57339018775431,48.01281010097,0
-0.57337157114104,48.01290468193,0
-0.57336534778611,48.012999988405,0
-0.57500000000005,48.013,0
</coordinates>
</LinearRing>
</outerBoundaryIs>
</Polygon>
</Placemark>
<Placemark>
<name>321</name>
<styleUrl>#stylehap1</styleUrl>
<Polygon>
<outerBoundaryIs>
<LinearRing>
<coordinates>
-0.57400000000007,48.44,0
-0.57235165270206,48.43999998823,0
-0.57235792208033,48.440095294882,0
-0.57237668873323,48.44018987637,0
-0.57240780990323,48.440283012865,0
-0.57245104880326,48.440373995533,0
-0.57250607641692,48.440462131927,0
-0.57257247400059,48.440546751259,0
-0.572649736269,48.440627209506,0
-0.57273727523942,48.440702894311,0
-0.57283442470565,48.440773229645,0
-0.57294044530769,48.440837680189,0
-0.57305453015868,48.440895755413,0
-0.57317581098575,48.440947013307,0
-0.57330336473878,48.440991063746,0
-0.57343622061637,48.441027571461,0
-0.5735733674552,48.441056258589,0
-0.57371376142735,48.44107690679,0
-0.57385633398607,48.44108935891,0
-0.57400000000008,48.441093520174,0
-0.57414366601406,48.44108935891,0
-0.57428623857279,48.44107690679,0
-0.57442663254493,48.441056258589,0
-0.57456377938377,48.441027571461,0
-0.57469663526136,48.440991063746,0
-0.57482418901439,48.440947013307,0
-0.57494546984146,48.440895755413,0
-0.57505955469245,48.440837680189,0
-0.57516557529451,48.440773229645,0
-0.57526272476071,48.440702894311,0
-0.57535026373114,48.440627209506,0
-0.57542752599955,48.440546751259,0
-0.57549392358321,48.440462131927,0
-0.57554895119688,48.440373995533,0
-0.57559219009691,48.440283012865,0
-0.5756233112669,48.44018987637,0
-0.57564207791981,48.440095294882,0
-0.57564834729808,48.43999998823,0

-0.57564207175816,48.439904681757,0
-0.5756232991308,48.4398101008,0
-0.57559217235509,48.439716965171,0
-0.57554892838843,48.43962598368,0
-0.57549389640118,48.439537848737,0
-0.57542749526982,48.439453231086,0
-0.57535023038742,48.439372774698,0
-0.57526268981616,48.439297091875,0
-0.57516553981087,48.439226758585,0
-0.57505951974787,48.439162310085,0
-0.57494543649775,48.439104236842,0
-0.57482415828466,48.439052980808,0
-0.57469660807929,48.439008932049,0
-0.57456375657533,48.438972425785,0
-0.57442661480311,48.438943739834,0
-0.57428622643668,48.4389230925,0
-0.57414365985241,48.438910640911,0
-0.57400000000008,48.438906479826,0
-0.57385634014772,48.438910640911,0
-0.57371377356346,48.4389230925,0
-0.57357338519702,48.438943739834,0
-0.57343624342481,48.438972425785,0
-0.57330339192085,48.439008932049,0
-0.57317584171548,48.439052980808,0
-0.57305456350239,48.439104236842,0
-0.57294048025227,48.439162310085,0
-0.57283446018927,48.439226758585,0
-0.57273731018398,48.439297091875,0
-0.57264976961272,48.439372774698,0
-0.57257250473032,48.439453231086,0
-0.57250610359896,48.439537848737,0
-0.5724510716117,48.43962598368,0
-0.57240782764505,48.439716965171,0
-0.57237670086934,48.4398101008,0
-0.57235792824198,48.439904681757,0
-0.57235165270206,48.43999998823,0
-0.57400000000007,48.44,0
</coordinates>
</LinearRing>
</outerBoundaryIs>
</Polygon>
</Placemark>
<Placemark>
<name>322</name>
<styleUrl>#stylehap1</styleUrl>
<Polygon>
<outerBoundaryIs>
<LinearRing>
<coordinates>
-0.57400000000007,48.259,0
-0.5723574969424,48.258999988305,0
-0.57236374411196,48.259095294956,0
-0.57238244424628,48.259189876443,0
-0.57241345509342,48.259283012935,0
-0.57245654070529,48.259373995599,0
-0.57251137323152,48.259462131988,0
-0.57257753541277,48.259546751315,0

-0.57265452375492,48.259627209556,0
-0.57274175235964,48.259702894355,0
-0.57283855738263,48.259773229682,0
-0.57294420208535,48.25983768022,0
-0.57305788244168,48.259895755438,0
-0.57317873325748,48.259947013326,0
-0.57330583475556,48.25999106376,0
-0.57343821957679,48.26002757147,0
-0.57357488014342,48.260056258594,0
-0.57371477632889,48.260076906792,0
-0.57385684337557,48.26008935891,0
-0.57400000000008,48.260093520173,0
-0.57414315662457,48.26008935891,0
-0.57428522367125,48.260076906792,0
-0.57442511985672,48.260056258594,0
-0.57456178042335,48.26002757147,0
-0.57469416524457,48.25999106376,0
-0.57482126674266,48.259947013326,0
-0.57494211755846,48.259895755438,0
-0.57505579791479,48.25983768022,0
-0.57516144261751,48.259773229682,0
-0.5752582476405,48.259702894355,0
-0.57534547624522,48.259627209556,0
-0.57542246458737,48.259546751315,0
-0.57548862676861,48.259462131988,0
-0.57554345929484,48.259373995599,0
-0.57558654490672,48.259283012935,0
-0.57561755575385,48.259189876443,0
-0.57563625588818,48.259095294956,0
-0.57564250305773,48.258999988305,0
-0.57563624978729,48.258904681831,0
-0.57561754373744,48.258810100872,0
-0.5755865273399,48.258716965241,0
-0.57554343671138,48.258625983746,0
-0.57548859985471,48.258537848799,0
-0.57542243416076,48.258453231142,0
-0.57534544323043,48.258372774748,0
-0.57525821304064,48.258297091919,0
-0.57516140748388,48.258226758622,0
-0.57505576331493,48.258162310116,0
-0.57494208454364,48.258104236867,0
-0.57482123631605,48.258052980826,0
-0.57469413833067,48.258008932063,0
-0.57456175783991,48.257972425794,0
-0.57442510228993,48.257943739839,0
-0.57428521165486,48.257923092502,0
-0.57414315052368,48.257910640912,0
-0.57400000000008,48.257906479826,0
-0.57385684947646,48.257910640912,0
-0.5737147883453,48.257923092502,0
-0.57357489771021,48.257943739839,0
-0.57343824216023,48.257972425794,0
-0.57330586166947,48.258008932063,0
-0.57317876368409,48.258052980826,0
-0.5730579154565,48.258104236867,0
-0.5729442366852,48.258162310116,0
-0.57283859251626,48.258226758622,0

-0.57274178695949,48.258297091919,0
-0.57265455676971,48.258372774748,0
-0.57257756583938,48.258453231142,0
-0.57251140014543,48.258537848799,0
-0.57245656328876,48.258625983746,0
-0.57241347266024,48.258716965241,0
-0.5723824562627,48.258810100872,0
-0.57236375021285,48.258904681831,0
-0.5723574969424,48.258999988305,0
-0.57400000000007,48.259,0
</coordinates>
</LinearRing>
</outerBoundaryIs>
</Polygon>
</Placemark>
<Placemark>
<name>323</name>
<styleUrl>#stylehap1</styleUrl>
<Polygon>
<outerBoundaryIs>
<LinearRing>
<coordinates>
-0.57299999999998,48.262,0
-0.57135740054767,48.261999988303,0
-0.57136364808355,48.262095294955,0
-0.57138234931501,48.262189876441,0
-0.57141336198181,48.262283012934,0
-0.57145645012202,48.262373995598,0
-0.571511285866,48.262462131987,0
-0.57157745192997,48.262546751314,0
-0.57165444479023,48.262627209556,0
-0.57174167851412,48.262702894355,0
-0.57183848921835,48.262773229682,0
-0.57194414012114,48.262837680219,0
-0.5720578271492,48.262895755437,0
-0.57217868505756,48.262947013326,0
-0.57230579401512,48.262991063759,0
-0.57243818660593,48.263027571469,0
-0.5725748551931,48.263056258594,0
-0.57271475958906,48.263076906792,0
-0.57285683497363,48.26308935891,0
-0.57299999999997,48.263093520173,0
-0.57314316502634,48.26308935891,0
-0.5732852404109,48.263076906792,0
-0.57342514480686,48.263056258594,0
-0.57356181339404,48.263027571469,0
-0.57369420598482,48.262991063759,0
-0.57382131494238,48.262947013326,0
-0.57394217285076,48.262895755437,0
-0.57405585987883,48.262837680219,0
-0.57416151078159,48.262773229682,0
-0.57425832148584,48.262702894355,0
-0.57434555520973,48.262627209556,0
-0.57442254807,48.262546751314,0
-0.57448871413396,48.262462131987,0
-0.57454354987795,48.262373995598,0
-0.57458663801816,48.262283012934,0

-0.57461765068495,48.262189876441,0
-0.57463635191642,48.262095294955,0
-0.57464259945229,48.261999988303,0
-0.57463634581454,48.261904681829,0
-0.57461763866655,48.261810100871,0
-0.57458662044847,48.26171696524,0
-0.57454352729079,48.261625983745,0
-0.57448868721563,48.261537848798,0
-0.5744225176384,48.261453231141,0
-0.57434552218952,48.261372774747,0
-0.57425828688031,48.261297091918,0
-0.57416147564221,48.261226758622,0
-0.5740558252733,48.261162310115,0
-0.57394213983055,48.261104236866,0
-0.57382128451078,48.261052980826,0
-0.57369417906652,48.261008932062,0
-0.57356179080688,48.260972425794,0
-0.57342512723717,48.260943739839,0
-0.57328522839253,48.260923092502,0
-0.57314315892443,48.260910640912,0
-0.57299999999997,48.260906479826,0
-0.57285684107551,48.260910640912,0
-0.57271477160743,48.260923092502,0
-0.57257487276279,48.260943739839,0
-0.57243820919308,48.260972425794,0
-0.57230582093345,48.261008932062,0
-0.57217871548916,48.261052980826,0
-0.57205786016942,48.261104236866,0
-0.57194417472666,48.261162310115,0
-0.57183852435773,48.261226758622,0
-0.57174171311965,48.261297091918,0
-0.57165447781045,48.261372774747,0
-0.57157748236157,48.261453231141,0
-0.57151131278433,48.261537848798,0
-0.57145647270917,48.261625983745,0
-0.5714133795515,48.26171696524,0
-0.57138236133339,48.261810100871,0
-0.57136365418543,48.261904681829,0
-0.57135740054767,48.261999988303,0
-0.57299999999998,48.262,0
</coordinates>
</LinearRing>
</outerBoundaryIs>
</Polygon>
</Placemark>
<Placemark>
<name>325</name>
<styleUrl>#stylehap1</styleUrl>
<Polygon>
<outerBoundaryIs>
<LinearRing>
<coordinates>
-0.572,48.452,0
-0.57035126318576,48.451999988225,0
-0.57035753404424,48.452095294877,0
-0.57037630513059,48.452189876365,0
-0.57040743365356,48.452283012861,0

-0.5704506827702,48.452373995529,0
-0.57050572338633,48.452462131923,0
-0.57057213665947,48.452546751255,0
-0.57064941718497,48.452627209503,0
-0.57073697684111,48.452702894309,0
-0.57083414926433,48.452773229642,0
-0.57094019491993,48.452837680187,0
-0.57105430673031,48.452895755412,0
-0.57117561621744,48.452947013306,0
-0.5713032001131,48.452991063745,0
-0.5714360873864,48.45302757146,0
-0.57157326663506,48.453056258588,0
-0.57171369378449,48.45307690679,0
-0.57185630003539,48.45308935891,0
-0.57200000000001,48.453093520174,0
-0.57214369996461,48.45308935891,0
-0.57228630621551,48.45307690679,0
-0.57242673336493,48.453056258588,0
-0.5725639126136,48.45302757146,0
-0.5726967998869,48.452991063745,0
-0.57282438378256,48.452947013306,0
-0.57294569326968,48.452895755412,0
-0.57305980508007,48.452837680187,0
-0.57316585073567,48.452773229642,0
-0.57326302315888,48.452702894309,0
-0.57335058281505,48.452627209503,0
-0.57342786334053,48.452546751255,0
-0.57349427661369,48.452462131923,0
-0.57354931722982,48.452373995529,0
-0.57359256634646,48.452283012861,0
-0.57362369486944,48.452189876365,0
-0.57364246595575,48.452095294877,0
-0.57364873681423,48.451999988225,0
-0.57364245979003,48.451904681752,0
-0.57362368272531,48.451810100795,0
-0.57359254859296,48.451716965167,0
-0.57354929440637,48.451625983676,0
-0.57349424941374,48.451537848733,0
-0.57342783259057,48.451453231082,0
-0.57335054944938,48.451372774695,0
-0.5732629881913,48.451297091872,0
-0.57316581522867,48.451226758583,0
-0.57305977011249,48.451162310083,0
-0.57294565990404,48.451104236841,0
-0.5728243530326,48.451052980807,0
-0.57269677268695,48.451008932048,0
-0.57256388979014,48.450972425785,0
-0.57242671561143,48.450943739834,0
-0.57228629407138,48.4509230925,0
-0.57214369379889,48.450910640911,0
-0.57200000000001,48.450906479827,0
-0.57185630620111,48.450910640911,0
-0.57171370592861,48.4509230925,0
-0.57157328438859,48.450943739834,0
-0.57143611020985,48.450972425785,0
-0.57130322731305,48.451008932048,0
-0.57117564696742,48.451052980807,0

-0.57105434009598,48.451104236841,0
-0.57094022988751,48.451162310083,0
-0.57083418477133,48.451226758583,0
-0.57073701180869,48.451297091872,0
-0.57064945055065,48.451372774695,0
-0.57057216740945,48.451453231082,0
-0.57050575058625,48.451537848733,0
-0.57045070559365,48.451625983676,0
-0.57040745140706,48.451716965167,0
-0.57037631727468,48.451810100795,0
-0.57035754020996,48.451904681752,0
-0.57035126318576,48.451999988225,0
-0.572,48.452,0
</coordinates>
</LinearRing>
</outerBoundaryIs>
</Polygon>
</Placemark>
<Placemark>
<name>326</name>
<styleUrl>#stylehap1</styleUrl>
<Polygon>
<outerBoundaryIs>
<LinearRing>
<coordinates>
-0.57100000000003,47.952,0
-0.56936727829541,47.95199998843,0
-0.56937348829466,47.952095295081,0
-0.56939207709838,47.952189876564,0
-0.56942290330083,47.952283013052,0
-0.56946573235815,47.95237399571,0
-0.56952023837148,47.952462132091,0
-0.56958600656572,47.952546751409,0
-0.56966253644447,47.952627209641,0
-0.56974924559815,47.952702894429,0
-0.56984547413553,47.952773229745,0
-0.56995048970527,47.952837680272,0
-0.57006349306952,47.952895755479,0
-0.57018362418671,47.952947013357,0
-0.57030996875751,47.952991063782,0
-0.57044156518411,47.953027571484,0
-0.57057741188975,47.953056258602,0
-0.57071647494279,47.953076906796,0
-0.57085769592729,47.953089358911,0
-0.57100000000003,47.953093520174,0
-0.57114230407276,47.953089358911,0
-0.57128352505726,47.953076906796,0
-0.5714225881103,47.953056258602,0
-0.57155843481594,47.953027571484,0
-0.57169003124254,47.952991063782,0
-0.57181637581334,47.952947013357,0
-0.57193650693053,47.952895755479,0
-0.57204951029478,47.952837680272,0
-0.57215452586452,47.952773229745,0
-0.5722507544019,47.952702894429,0
-0.57233746355558,47.952627209641,0
-0.57241399343434,47.952546751409,0

-0.57247976162857,47.952462132091,0
-0.57253426764191,47.95237399571,0
-0.57257709669922,47.952283013052,0
-0.57260792290167,47.952189876564,0
-0.57262651170539,47.952095295081,0
-0.57263272170464,47.95199998843,0
-0.57262650570587,47.951904681955,0
-0.57260791108489,47.951810100994,0
-0.57257707942425,47.951716965358,0
-0.57253424543364,47.951625983857,0
-0.57247973516178,47.951537848902,0
-0.57241396351323,47.951453231236,0
-0.57233743108927,47.951372774832,0
-0.57225072037686,47.951297091992,0
-0.57215449131459,47.951226758685,0
-0.57204947626974,47.951162310167,0
-0.57193647446419,47.951104236908,0
-0.57181634589221,47.951052980858,0
-0.57169000477575,47.951008932085,0
-0.57155841260768,47.950972425809,0
-0.57142257083533,47.950943739848,0
-0.57128351324049,47.950923092506,0
-0.57114229807322,47.950910640913,0
-0.57100000000003,47.950906479826,0
-0.57085770192684,47.950910640913,0
-0.57071648675957,47.950923092506,0
-0.57057742916472,47.950943739848,0
-0.57044158739237,47.950972425809,0
-0.5703099952243,47.951008932085,0
-0.57018365410784,47.951052980858,0
-0.57006352553586,47.951104236908,0
-0.56995052373031,47.951162310167,0
-0.56984550868546,47.951226758685,0
-0.56974927962319,47.951297091992,0
-0.56966256891078,47.951372774832,0
-0.56958603648682,47.951453231236,0
-0.56952026483827,47.951537848902,0
-0.56946575456641,47.951625983857,0
-0.5694229205758,47.951716965358,0
-0.56939208891516,47.951810100994,0
-0.56937349429418,47.951904681955,0
-0.56936727829541,47.95199998843,0
-0.57100000000003,47.952,0
</coordinates>
</LinearRing>
</outerBoundaryIs>
</Polygon>
</Placemark>
<Placemark>
<name>327</name>
<styleUrl>#stylehap1</styleUrl>
<Polygon>
<outerBoundaryIs>
<LinearRing>
<coordinates>
-0.56999999999994,48.42,0
-0.56835230132622,48.419999988238,0

-0.56835856823964,48.420095294891,0
-0.56837732750997,48.420189876378,0
-0.56840843643577,48.420283012873,0
-0.56845165832311,48.420373995541,0
-0.56850666428494,48.420462131934,0
-0.56857303574241,48.420546751265,0
-0.56865026760907,48.420627209512,0
-0.56873777213347,48.420702894316,0
-0.56883488337159,48.420773229649,0
-0.56894086225438,48.420837680193,0
-0.56905490221244,48.420895755416,0
-0.5691761353146,48.420947013309,0
-0.56930363887403,48.420991063748,0
-0.56943644247131,48.421027571462,0
-0.5695735353411,48.421056258589,0
-0.5697138740663,48.42107690679,0
-0.56985639052065,48.42108935891,0
-0.56999999999994,48.421093520174,0
-0.57014360947923,48.42108935891,0
-0.57028612593358,48.42107690679,0
-0.57042646465875,48.421056258589,0
-0.57056355752857,48.421027571462,0
-0.57069636112585,48.420991063748,0
-0.57082386468528,48.420947013309,0
-0.57094509778744,48.420895755416,0
-0.5710591377455,48.420837680193,0
-0.57116511662828,48.420773229649,0
-0.57126222786641,48.420702894316,0
-0.57134973239081,48.420627209512,0
-0.57142696425744,48.420546751265,0
-0.57149333571494,48.420462131934,0
-0.57154834167676,48.420373995541,0
-0.57159156356411,48.420283012873,0
-0.57162267248988,48.420189876378,0
-0.57164143176021,48.420095294891,0
-0.57164769867366,48.419999988238,0
-0.5716414256053,48.419904681765,0
-0.57162266036708,48.419810100808,0
-0.57159154584173,48.419716965179,0
-0.57154831889333,48.419625983688,0
-0.5714933085627,48.419537848744,0
-0.5714269335614,48.419453231092,0
-0.57134969908364,48.419372774704,0
-0.57126219296012,48.41929709188,0
-0.57116508118355,48.419226758589,0
-0.57105910283925,48.419162310088,0
-0.57094506448029,48.419104236845,0
-0.57082383398923,48.41905298081,0
-0.5706963339736,48.419008932051,0
-0.57056353474513,48.418972425786,0
-0.5704264469364,48.418943739835,0
-0.57028611381078,48.4189230925,0
-0.57014360332432,48.418910640912,0
-0.56999999999994,48.418906479827,0
-0.56985639667555,48.418910640912,0
-0.5697138861891,48.4189230925,0
-0.56957355306348,48.418943739835,0

-0.56943646525474,48.418972425786,0
-0.56930366602627,48.419008932051,0
-0.56917616601064,48.41905298081,0
-0.56905493551959,48.419104236845,0
-0.56894089716063,48.419162310088,0
-0.56883491881633,48.419226758589,0
-0.56873780703973,48.41929709188,0
-0.56865030091624,48.419372774704,0
-0.56857306643848,48.419453231092,0
-0.56850669143718,48.419537848744,0
-0.56845168110655,48.419625983688,0
-0.56840845415815,48.419716965179,0
-0.56837733963279,48.419810100808,0
-0.56835857439457,48.419904681765,0
-0.56835230132622,48.419999988238,0
-0.56999999999994,48.42,0
</coordinates>
</LinearRing>
</outerBoundaryIs>
</Polygon>
</Placemark>
<Placemark>
<name>329</name>
<styleUrl>#stylehap1</styleUrl>
<Polygon>
<outerBoundaryIs>
<LinearRing>
<coordinates>
-0.56799999999998,48.278,0
-0.56635688617562,48.277999988297,0
-0.56636313566616,48.278095294949,0
-0.56638184275219,48.278189876435,0
-0.56641286512889,48.278283012928,0
-0.5664559667605,48.278373995592,0
-0.56651081967482,48.278462131982,0
-0.56657700645738,48.278546751309,0
-0.56665402342686,48.278627209551,0
-0.56674128446711,48.278702894351,0
-0.56683812548701,48.278773229678,0
-0.566943809474,48.278837680217,0
-0.56705753210306,48.278895755435,0
-0.56717842785822,48.278947013324,0
-0.56730557662031,48.278991063758,0
-0.56743801067044,48.279027571469,0
-0.56757472205613,48.279056258593,0
-0.56771467026402,48.279076906792,0
-0.56785679014048,48.27908935891,0
-0.568,48.279093520174,0
-0.56814320985949,48.27908935891,0
-0.56828532973594,48.279076906792,0
-0.56842527794384,48.279056258593,0
-0.56856198932952,48.279027571469,0
-0.56869442337965,48.278991063758,0
-0.56882157214175,48.278947013324,0
-0.5689424678969,48.278895755435,0
-0.56905619052596,48.278837680217,0
-0.56916187451295,48.278773229678,0

-0.56925871553285,48.278702894351,0
-0.5693459765731,48.278627209551,0
-0.56942299354258,48.278546751309,0
-0.56948918032514,48.278462131982,0
-0.56954403323946,48.278373995592,0
-0.56958713487108,48.278283012928,0
-0.56961815724777,48.278189876435,0
-0.5696368643338,48.278095294949,0
-0.56964311382435,48.277999988297,0
-0.56963685822658,48.277904681823,0
-0.56961814521889,48.277810100864,0
-0.56958711728602,48.277716965234,0
-0.56954401063251,48.277625983739,0
-0.56948915338325,48.277537848792,0
-0.56942296308434,48.277453231136,0
-0.56934594352396,48.277372774743,0
-0.56925868089702,48.277297091914,0
-0.56916183934281,48.277226758618,0
-0.56905615589013,48.277162310112,0
-0.56894243484778,48.277104236864,0
-0.56882154168348,48.277052980824,0
-0.56869439643774,48.277008932061,0
-0.5685619667226,48.276972425793,0
-0.56842526035875,48.276943739839,0
-0.56828531770703,48.276923092502,0
-0.56814320375226,48.276910640912,0
-0.568,48.276906479826,0
-0.5678567962477,48.276910640912,0
-0.56771468229293,48.276923092502,0
-0.56757473964121,48.276943739839,0
-0.56743803327737,48.276972425793,0
-0.56730560356223,48.277008932061,0
-0.56717845831648,48.277052980824,0
-0.56705756515218,48.277104236864,0
-0.56694384410984,48.277162310112,0
-0.56683816065715,48.277226758618,0
-0.56674131910294,48.277297091914,0
-0.56665405647601,48.277372774743,0
-0.56657703691565,48.277453231136,0
-0.56651084661672,48.277537848792,0
-0.56645598936745,48.277625983739,0
-0.56641288271395,48.277716965234,0
-0.56638185478107,48.277810100864,0
-0.56636314177339,48.277904681823,0
-0.56635688617562,48.277999988297,0
-0.56799999999998,48.278,0
</coordinates>
</LinearRing>
</outerBoundaryIs>
</Polygon>
</Placemark>
<Placemark>
<name>333</name>
<styleUrl>#stylehap1</styleUrl>
<Polygon>
<outerBoundaryIs>
<LinearRing>

<coordinates>
-0.56299999999999,48.228,0
-0.5613584920949,48.227999988317,0
-0.56136473548278,48.228095294969,0
-0.56138342429036,48.228189876455,0
-0.56141441635179,48.228283012947,0
-0.56145747586187,48.22837399561,0
-0.56151227516876,48.228462131999,0
-0.56157839726586,48.228546751325,0
-0.56165533896405,48.228627209565,0
-0.56174251471994,48.228702894363,0
-0.56183926109144,48.228773229689,0
-0.56194484178631,48.228837680225,0
-0.56205845326564,48.228895755442,0
-0.56217923085945,48.228947013329,0
-0.56230625534793,48.228991063762,0
-0.56243855995806,48.229027571471,0
-0.56257513772264,48.229056258595,0
-0.56271494914541,48.229076906793,0
-0.56285693011392,48.22908935891,0
-0.563,48.229093520174,0
-0.56314306988607,48.22908935891,0
-0.56328505085455,48.229076906793,0
-0.56342486227732,48.229056258595,0
-0.56356144004193,48.229027571471,0
-0.56369374465206,48.228991063762,0
-0.56382076914052,48.228947013329,0
-0.56394154673435,48.228895755442,0
-0.56405515821366,48.228837680225,0
-0.56416073890853,48.228773229689,0
-0.56425748528003,48.228702894363,0
-0.56434466103592,48.228627209565,0
-0.56442160273411,48.228546751325,0
-0.56448772483121,48.228462131999,0
-0.5645425241381,48.22837399561,0
-0.56458558364817,48.228283012947,0
-0.56461657570961,48.228189876455,0
-0.56463526451721,48.228095294969,0
-0.56464150790509,48.227999988317,0
-0.56463525842666,48.227904681843,0
-0.56461656371357,48.227810100884,0
-0.56458556611113,48.227716965253,0
-0.5645425015929,48.227625983758,0
-0.5644876979629,48.227537848809,0
-0.56442157235905,48.227453231151,0
-0.56434462807705,48.227372774757,0
-0.56425745073877,48.227297091926,0
-0.56416070383441,48.227226758629,0
-0.5640551236724,48.227162310121,0
-0.56394151377546,48.227104236871,0
-0.56382073876546,48.22705298083,0
-0.56369371778373,48.227008932065,0
-0.56356141749673,48.226972425796,0
-0.56342484474028,48.22694373984,0
-0.56328503885852,48.226923092503,0
-0.56314306379552,48.226910640912,0
-0.563,48.226906479827,0

-0.56285693620447,48.226910640912,0
-0.56271496114147,48.226923092503,0
-0.56257515525971,48.22694373984,0
-0.56243858250326,48.226972425796,0
-0.56230628221624,48.227008932065,0
-0.56217926123453,48.22705298083,0
-0.56205848622451,48.227104236871,0
-0.56194487632756,48.227162310121,0
-0.56183929616555,48.227226758629,0
-0.56174254926119,48.227297091926,0
-0.56165537192294,48.227372774757,0
-0.56157842764094,48.227453231151,0
-0.56151230203709,48.227537848809,0
-0.56145749840707,48.227625983758,0
-0.56141443388884,48.227716965253,0
-0.56138343628642,48.227810100884,0
-0.56136474157334,48.227904681843,0
-0.5613584920949,48.227999988317,0
-0.56299999999999,48.228,0
</coordinates>
</LinearRing>
</outerBoundaryIs>
</Polygon>
</Placemark>
<Placemark>
<name>334</name>
<styleUrl>#stylehap1</styleUrl>
<Polygon>
<outerBoundaryIs>
<LinearRing>
<coordinates>
-0.56200000000001,48.2,0
-0.56035938949392,48.199999988329,0
-0.56036562947158,48.20009529498,0
-0.56038430806505,48.200189876466,0
-0.56041528318611,48.200283012958,0
-0.56045831915835,48.200373995621,0
-0.56051308850904,48.200462132008,0
-0.56057917445944,48.200546751333,0
-0.56065607409551,48.200627209573,0
-0.56074320219389,48.200702894369,0
-0.56083989567522,48.200773229694,0
-0.56094541864971,48.20083768023,0
-0.5610589680178,48.200895755446,0
-0.56117967958222,48.200947013332,0
-0.56130663462571,48.200991063764,0
-0.56143886690384,48.201027571472,0
-0.56157537000004,48.201056258595,0
-0.56171510498632,48.201076906793,0
-0.56185700833207,48.20108935891,0
-0.56200000000001,48.201093520174,0
-0.56214299166796,48.20108935891,0
-0.5622848950137,48.201076906793,0
-0.56242462999998,48.201056258595,0
-0.56256113309618,48.201027571472,0
-0.56269336537431,48.200991063764,0
-0.5628203204178,48.200947013332,0

-0.56294103198222,48.200895755446,0
-0.56305458135031,48.20083768023,0
-0.56316010432481,48.200773229694,0
-0.56325679780613,48.200702894369,0
-0.56334392590451,48.200627209573,0
-0.56342082554058,48.200546751333,0
-0.56348691149098,48.200462132008,0
-0.56354168084167,48.200373995621,0
-0.56358471681391,48.200283012958,0
-0.56361569193497,48.200189876466,0
-0.56363437052845,48.20009529498,0
-0.5636406105061,48.199999988329,0
-0.5636343644472,48.199904681855,0
-0.56361567995726,48.199810100895,0
-0.56358469930369,48.199716965264,0
-0.56354165833092,48.199625983768,0
-0.56348688466374,48.199537848819,0
-0.56342079521195,48.19945323116,0
-0.56334389299605,48.199372774764,0
-0.56325676331769,48.199297091933,0
-0.56316006930433,48.199226758634,0
-0.56305454686188,48.199162310126,0
-0.56294099907376,48.199104236875,0
-0.5628202900892,48.199052980832,0
-0.56269333854707,48.199008932067,0
-0.56256111058543,48.198972425797,0
-0.56242461248975,48.198943739841,0
-0.56228488303601,48.198923092503,0
-0.56214298558671,48.198910640912,0
-0.56200000000001,48.198906479826,0
-0.56185701441331,48.198910640912,0
-0.56171511696401,48.198923092503,0
-0.56157538751027,48.198943739841,0
-0.56143888941459,48.198972425797,0
-0.56130666145295,48.199008932067,0
-0.56117970991082,48.199052980832,0
-0.56105900092626,48.199104236875,0
-0.56094545313814,48.199162310126,0
-0.56083993069569,48.199226758634,0
-0.56074323668233,48.199297091933,0
-0.56065610700398,48.199372774764,0
-0.56057920478807,48.19945323116,0
-0.56051311533629,48.199537848819,0
-0.5604583416691,48.199625983768,0
-0.56041530069633,48.199716965264,0
-0.56038432004276,48.199810100895,0
-0.56036563555282,48.199904681855,0
-0.56035938949392,48.199999988329,0
-0.56200000000001,48.2,0
</coordinates>
</LinearRing>
</outerBoundaryIs>
</Polygon>
</Placemark>
<Placemark>
<name>336</name>
<styleUrl>#stylehap1</styleUrl>

<Polygon>
<outerBoundaryIs>
<LinearRing>
<coordinates>
-0.56100000000004,48.2,0
-0.55935938949396,48.199999988329,0
-0.55936562947162,48.20009529498,0
-0.55938430806509,48.200189876466,0
-0.55941528318612,48.200283012958,0
-0.55945831915839,48.200373995621,0
-0.55951308850906,48.200462132008,0
-0.55957917445949,48.200546751333,0
-0.55965607409553,48.200627209573,0
-0.55974320219393,48.200702894369,0
-0.55983989567523,48.200773229694,0
-0.55994541864972,48.20083768023,0
-0.56005896801781,48.200895755446,0
-0.56017967958223,48.200947013332,0
-0.56030663462572,48.200991063764,0
-0.56043886690388,48.201027571472,0
-0.56057537000006,48.201056258595,0
-0.56071510498634,48.201076906793,0
-0.56085700833208,48.20108935891,0
-0.56100000000003,48.201093520174,0
-0.56114299166797,48.20108935891,0
-0.56128489501374,48.201076906793,0
-0.56142463000002,48.201056258595,0
-0.56156113309619,48.201027571472,0
-0.56169336537435,48.200991063764,0
-0.56182032041784,48.200947013332,0
-0.56194103198226,48.200895755446,0
-0.56205458135033,48.20083768023,0
-0.56216010432485,48.200773229694,0
-0.56225679780614,48.200702894369,0
-0.56234392590455,48.200627209573,0
-0.56242082554059,48.200546751333,0
-0.56248691149102,48.200462132008,0
-0.56254168084168,48.200373995621,0
-0.56258471681395,48.200283012958,0
-0.56261569193499,48.200189876466,0
-0.56263437052846,48.20009529498,0
-0.56264061050612,48.199999988329,0
-0.56263436444722,48.199904681855,0
-0.56261567995727,48.199810100895,0
-0.5625846993037,48.199716965264,0
-0.56254165833097,48.199625983768,0
-0.56248688466378,48.199537848819,0
-0.56242079521197,48.19945323116,0
-0.56234389299606,48.199372774764,0
-0.56225676331771,48.199297091933,0
-0.56216006930437,48.199226758634,0
-0.5620545468619,48.199162310126,0
-0.56194099907378,48.199104236875,0
-0.56182029008922,48.199052980832,0
-0.56169333854711,48.199008932067,0
-0.56156111058547,48.198972425797,0
-0.56142461248977,48.198943739841,0

-0.56128488303603,48.198923092503,0
-0.56114298558672,48.198910640912,0
-0.56100000000003,48.198906479826,0
-0.56085701441333,48.198910640912,0
-0.56071511696405,48.198923092503,0
-0.56057538751028,48.198943739841,0
-0.5604388894146,48.198972425797,0
-0.56030666145296,48.199008932067,0
-0.56017970991086,48.199052980832,0
-0.5600590009263,48.199104236875,0
-0.55994545313816,48.199162310126,0
-0.55983993069571,48.199226758634,0
-0.55974323668237,48.199297091933,0
-0.55965610700402,48.199372774764,0
-0.55957920478811,48.19945323116,0
-0.5595131153363,48.199537848819,0
-0.55945834166911,48.199625983768,0
-0.55941530069638,48.199716965264,0
-0.55938432004278,48.199810100895,0
-0.55936563555284,48.199904681855,0
-0.55935938949396,48.199999988329,0
-0.56100000000004,48.2,0
</coordinates>
</LinearRing>
</outerBoundaryIs>
</Polygon>
</Placemark>
<Placemark>
<name>337</name>
<styleUrl>#stylehap1</styleUrl>
<Polygon>
<outerBoundaryIs>
<LinearRing>
<coordinates>
-0.56100000000004,48.418,0
-0.55935236614963,48.417999988239,0
-0.55935863281672,48.418095294891,0
-0.55937739134923,48.418189876379,0
-0.55940849905136,48.418283012874,0
-0.55945171923843,48.418373995541,0
-0.55950672303638,48.418462131934,0
-0.55957309188285,48.418546751266,0
-0.55965032071114,48.418627209512,0
-0.55973782179304,48.418702894317,0
-0.55983492921065,48.418773229649,0
-0.559940903924,48.418837680193,0
-0.56005493939548,48.418895755416,0
-0.56017616772803,48.41894701331,0
-0.56030366627114,48.418991063748,0
-0.56043646464353,48.419027571462,0
-0.56057355211968,48.419056258589,0
-0.56071388532351,48.41907690679,0
-0.5608563961708,48.41908935891,0
-0.56100000000003,48.419093520174,0
-0.56114360382928,48.41908935891,0
-0.56128611467656,48.41907690679,0
-0.5614264478804,48.419056258589,0

-0.56156353535655,48.419027571462,0
-0.56169633372894,48.418991063748,0
-0.56182383227205,48.41894701331,0
-0.5619450606046,48.418895755416,0
-0.56205909607605,48.418837680193,0
-0.56216507078943,48.418773229649,0
-0.56226217820704,48.418702894317,0
-0.56234967928892,48.418627209512,0
-0.56242690811723,48.418546751266,0
-0.56249327696367,48.418462131934,0
-0.56254828076162,48.418373995541,0
-0.56259150094872,48.418283012874,0
-0.56262260865082,48.418189876379,0
-0.56264136718333,48.418095294891,0
-0.56264763385045,48.417999988239,0
-0.56264136102911,48.417904681766,0
-0.56262259652934,48.417810100808,0
-0.56259148322827,48.4177169651 8,0
-0.56254825798068,48.417625983688,0
-0.5624932498144,48.417537848745,0
-0.56242687742455,48.417453231093,0
-0.5623496459854,48.417372774704,0
-0.5622621433046,48.41729709188,0
-0.56216503534858,48.41722675859,0
-0.56205906117363,48.417162310088,0
-0.56194502730109,48.417104236845,0
-0.56182380157937,48.41705298081,0
-0.56169630657968,48.417008932051,0
-0.56156351257558,48.416972425787,0
-0.56142643015995,48.416943739835,0
-0.56128610255509,48.4169230925,0
-0.56114359767503,48.416910640912,0
-0.56100000000003,48.416906479827,0
-0.56085640232504,48.416910640912,0
-0.56071389744499,48.4169230925,0
-0.5605735698401,48.416943739835,0
-0.56043648742447,48.416972425787,0
-0.5603036934204,48.417008932051,0
-0.56017619842071,48.41705298081,0
-0.56005497269899,48.417104236845,0
-0.55994093882644,48.417162310088,0
-0.55983496465149,48.41722675859,0
-0.55973785669548,48.41729709188,0
-0.55965035401465,48.417372774704,0
-0.55957312257553,48.417453231093,0
-0.55950675018567,48.417537848745,0
-0.5594517420194,48.417625983688,0
-0.55940851677178,48.41771696518,0
-0.55937740347074,48.417810100808,0
-0.55935863897096,48.417904681766,0
-0.55935236614963,48.417999988239,0
-0.56100000000004,48.418,0
</coordinates>
</LinearRing>
</outerBoundaryIs>
</Polygon>
</Placemark>

<Placemark>
<name>340</name>
<styleUrl>#stylehap1</styleUrl>
<Polygon>
<outerBoundaryIs>
<LinearRing>
<coordinates>
-0.55500000000006,47.998,0
-0.55336582309647,47.997999988411,0
-0.55337203862567,47.998095295062,0
-0.55339064399236,47.998189876546,0
-0.55342149766493,47.998283013035,0
-0.55346436489058,47.998373995693,0
-0.55351891948015,47.998462132076,0
-0.55358474628893,47.998546751395,0
-0.55366134437455,47.998627209628,0
-0.55374813080843,47.998702894418,0
-0.55384444511115,47.998773229736,0
-0.55394955427876,47.998837680264,0
-0.554062658361,47.998895755473,0
-0.55418289654973,47.998947013353,0
-0.55430935373069,47.998991063779,0
-0.55444106744892,47.999027571482,0
-0.55457703523492,47.999056258601,0
-0.55471622223537,47.999076906795,0
-0.55485756909086,47.999089358911,0
-0.55500000000007,47.999093520173,0
-0.55514243090928,47.999089358911,0
-0.55528377776477,47.999076906795,0
-0.55542296476521,47.999056258601,0
-0.55555893255119,47.999027571482,0
-0.55569064626945,47.998991063779,0
-0.55581710345038,47.998947013353,0
-0.55593734163913,47.998895755473,0
-0.55605044572138,47.998837680264,0
-0.55615555488896,47.998773229736,0
-0.5562518691917,47.998702894418,0
-0.55633865562556,47.998627209628,0
-0.55641525371121,47.998546751395,0
-0.55648108051996,47.998462132076,0
-0.55653563510955,47.998373995693,0
-0.5565785023352,47.998283013035,0
-0.55660935600777,47.998189876546,0
-0.55662796137447,47.998095295062,0
-0.55663417690366,47.997999988411,0
-0.55662795535989,47.997904681936,0
-0.55660934416135,47.997810100975,0
-0.5565784850169,47.997716965341,0
-0.55653561284556,47.997625983841,0
-0.55648105398678,47.997537848886,0
-0.55641522371501,47.997453231222,0
-0.5563386230778,47.99737277482,0
-0.5562518350813,47.997297091981,0
-0.55615552025237,47.997226758676,0
-0.55605041161097,47.99716231016,0
-0.55593730909137,47.997104236902,0
-0.55581707345421,47.997052980853,0

-0.55569061973627,47.997008932082,0
-0.55555891028723,47.996972425807,0
-0.55542294744692,47.996943739847,0
-0.55528376591835,47.996923092506,0
-0.55514242489469,47.996910640913,0
-0.55500000000007,47.996906479826,0
-0.55485757510544,47.996910640913,0
-0.55471623408179,47.996923092506,0
-0.55457705255322,47.996943739847,0
-0.55444108971291,47.996972425807,0
-0.55430938026387,47.997008932082,0
-0.55418292654592,47.997052980853,0
-0.55406269090876,47.997104236902,0
-0.55394958838914,47.99716231016,0
-0.55384447974777,47.997226758676,0
-0.55374816491881,47.997297091981,0
-0.55366137692231,47.99737277482,0
-0.5535847762851,47.997453231222,0
-0.55351894601333,47.997537848886,0
-0.55346438715457,47.997625983841,0
-0.55342151498323,47.997716965341,0
-0.55339065583878,47.997810100975,0
-0.55337204464025,47.997904681936,0
-0.55336582309647,47.997999988411,0
-0.55500000000006,47.998,0
</coordinates>
</LinearRing>
</outerBoundaryIs>
</Polygon>
</Placemark>
<Placemark>
<name>342</name>
<styleUrl>#stylehap1</styleUrl>
<Polygon>
<outerBoundaryIs>
<LinearRing>
<coordinates>
-0.55200000000002,48.082,0
-0.55036315634617,48.081999988377,0
-0.55036938200935,48.082095295028,0
-0.55038801772879,48.082189876513,0
-0.55041892174219,48.082283013003,0
-0.55046185891389,48.082373995663,0
-0.5505165025227,48.082462132048,0
-0.55058243674657,48.082546751369,0
-0.55065915982588,48.082627209605,0
-0.55074608788088,48.082702894398,0
-0.55084255935442,48.082773229718,0
-0.55094784004639,48.08283768025,0
-0.55106112870116,48.082895755462,0
-0.55118156310571,48.082947013344,0
-0.55130822665241,48.082991063773,0
-0.55144015531548,48.083027571478,0
-0.55157634498936,48.083056258599,0
-0.55171575913182,48.083076906794,0
-0.55185733665442,48.083089358911,0
-0.55200000000001,48.083093520173,0

-0.55214266334563,48.083089358911,0
-0.55228424086823,48.083076906794,0
-0.55242365501069,48.083056258599,0
-0.55255984468455,48.083027571478,0
-0.55269177334764,48.082991063773,0
-0.55281843689431,48.082947013344,0
-0.55293887129889,48.082895755462,0
-0.55305215995363,48.08283768025,0
-0.5531574406456,48.082773229718,0
-0.55325391211917,48.082702894398,0
-0.55334084017415,48.082627209605,0
-0.55341756325348,48.082546751369,0
-0.55348349747735,48.082462132048,0
-0.55353814108613,48.082373995663,0
-0.55358107825786,48.082283013003,0
-0.55361198227123,48.082189876513,0
-0.5536306179907,48.082095295028,0
-0.55363684365388,48.081999988377,0
-0.55363061194851,48.081904681903,0
-0.55361197037046,48.081810100942,0
-0.55358106086007,48.081716965309,0
-0.55353811871999,48.08162598381,0
-0.55348347082239,48.081537848858,0
-0.55341753311961,48.081453231196,0
-0.553340807477,48.081372774797,0
-0.55325387785221,48.081297091961,0
-0.55315740585003,48.081226758659,0
-0.55305212568669,48.081162310145,0
-0.55293883860174,48.081104236891,0
-0.55281840676047,48.081052980845,0
-0.55269174669269,48.081008932076,0
-0.5525598223184,48.080972425803,0
-0.55242363761288,48.080943739844,0
-0.55228422896743,48.080923092505,0
-0.55214265730344,48.080910640913,0
-0.55200000000001,48.080906479826,0
-0.55185734269661,48.080910640913,0
-0.55171577103259,48.080923092505,0
-0.55157636238715,48.080943739844,0
-0.55144017768165,48.080972425803,0
-0.55130825330736,48.081008932076,0
-0.55118159323958,48.081052980845,0
-0.5510611613983,48.081104236891,0
-0.55094787431336,48.081162310145,0
-0.55084259415002,48.081226758659,0
-0.55074612214784,48.081297091961,0
-0.55065919252305,48.081372774797,0
-0.55058246688042,48.081453231196,0
-0.55051652917766,48.081537848858,0
-0.55046188128006,48.08162598381,0
-0.55041893913998,48.081716965309,0
-0.55038802962959,48.081810100942,0
-0.55036938805154,48.081904681903,0
-0.55036315634617,48.081999988377,0
-0.55200000000002,48.082,0
</coordinates>
</LinearRing>

</outerBoundaryIs>
</Polygon>
</Placemark>
<Placemark>
<name>343</name>
<styleUrl>#stylehap1</styleUrl>
<Polygon>
<outerBoundaryIs>
<LinearRing>
<coordinates>
-0.55200000000002,48.207,0
-0.5503591652729,48.206999988326,0
-0.55036540610265,48.207095294978,0
-0.55038408724817,48.207189876463,0
-0.55041506660189,48.207283012955,0
-0.55045810845523,48.207373995618,0
-0.55051288529066,48.207462132006,0
-0.55057898027256,48.207546751331,0
-0.55065589041813,48.207627209571,0
-0.55074303042404,48.207702894368,0
-0.55083973712032,48.207773229693,0
-0.55094527451664,48.207837680229,0
-0.55105883940362,48.207895755445,0
-0.55117956746591,48.207947013331,0
-0.55130653986068,48.207991063763,0
-0.55143879021146,48.208027571472,0
-0.55157531196403,48.208056258595,0
-0.55171506604847,48.208076906793,0
-0.55185698878875,48.20808935891,0
-0.55200000000001,48.208093520174,0
-0.55214301121127,48.20808935891,0
-0.55228493395158,48.208076906793,0
-0.55242468803602,48.208056258595,0
-0.55256120978859,48.208027571472,0
-0.55269346013937,48.207991063763,0
-0.55282043253414,48.207947013331,0
-0.55294116059643,48.207895755445,0
-0.55305472548341,48.207837680229,0
-0.55316026287973,48.207773229693,0
-0.55325696957598,48.207702894368,0
-0.55334410958192,48.207627209571,0
-0.55342101972749,48.207546751331,0
-0.55348711470939,48.207462132006,0
-0.55354189154482,48.207373995618,0
-0.55358493339816,48.207283012955,0
-0.55361591275188,48.207189876463,0
-0.5536345938974,48.207095294978,0
-0.55364083472712,48.206999988326,0
-0.55363458781384,48.206904681852,0
-0.55361590076958,48.206810100893,0
-0.55358491588122,48.206716965261,0
-0.55354186902547,48.206625983765,0
-0.55348708787187,48.206537848816,0
-0.55342098938726,48.206453231158,0
-0.55334407666087,48.206372774763,0
-0.55325693507436,48.206297091931,0
-0.55316022784585,48.206226758633,0

-0.5530546909818,48.206162310124,0
-0.55294112767537,48.206104236874,0
-0.55282040219391,48.206052980832,0
-0.55269343330185,48.206008932066,0
-0.55256118726924,48.205972425797,0
-0.55242467051908,48.205943739841,0
-0.55228492196928,48.205923092503,0
-0.55214300512771,48.20591064091 2,0
-0.55200000000001,48.205906479826,0
-0.55185699487234,48.205910640912,0
-0.55171507803074,48.205923092503,0
-0.55157532948097,48.205943739841,0
-0.55143881273078,48.205972425797,0
-0.5513065666982,48.206008932066,0
-0.55117959780614,48.206052980832,0
-0.55105887232468,48.206104236874,0
-0.55094530901825,48.206162310124,0
-0.5508397721542,48.206226758633,0
-0.55074306492568,48.206297091931,0
-0.55065592333918,48.206372774763,0
-0.55057901061279,48.206453231158,0
-0.55051291212815,48.206537848816,0
-0.55045813097458,48.206625983765,0
-0.55041508411883,48.206716965261,0
-0.55038409923046,48.206810100893,0
-0.55036541218621,48.206904681852,0
-0.5503591652729,48.206999988326,0
-0.55200000000002,48.207,0
</coordinates>
</LinearRing>
</outerBoundaryIs>
</Polygon>
</Placemark>
<Placemark>
<name>344</name>
<styleUrl>#stylehap1</styleUrl>
<Polygon>
<outerBoundaryIs>
<LinearRing>
<coordinates>
-0.55099999999993,48.239,0
-0.54935813916926,48.238999988313,0
-0.54936438389829,48.239095294965,0
-0.54938307672284,48.239189876451,0
-0.54941407544654,48.239283012943,0
-0.54945714421348,48.239373995606,0
-0.54951195530142,48.239462131995,0
-0.54957809161415,48.239546751321,0
-0.54965504985436,48.239627209562,0
-0.54974224435282,48.23970289436,0
-0.54983901152477,48.239773229686,0
-0.54994461491966,48.239837680223,0
-0.55005825082586,48.239895755441,0
-0.55017905438747,48.239947013328,0
-0.550306106187,48.239991063761,0
-0.55043843924355,48.240027571471,0
-0.55057504637345,48.240056258594,0

-0.55071488785687,48.240076906793,0
-0.55085689935254,48.24008935891,0
-0.55099999999992,48.240093520174,0
-0.55114310064734,48.24008935891,0
-0.55128511214301,48.240076906793,0
-0.55142495362642,48.240056258594,0
-0.5515615607563,48.240027571471,0
-0.55169389381288,48.239991063761,0
-0.5518209456124,48.239947013328,0
-0.55194174917399,48.239895755441,0
-0.55205538508019,48.239837680223,0
-0.55216098847511,48.239773229686,0
-0.55225775564706,48.23970289436,0
-0.55234495014549,48.239627209562,0
-0.5524219083857,48.239546751321,0
-0.55248804469845,48.239462131995,0
-0.55254285578639,48.239373995606,0
-0.55258592455334,48.239283012943,0
-0.55261692327701,48.239189876451,0
-0.55263561610159,48.239095294965,0
-0.55264186083061,48.238999988313,0
-0.55263561000736,48.238904681839,0
-0.55261691127375,48.23881010088,0
-0.55258590700573,48.238716965249,0
-0.55254283322764,48.238625983754,0
-0.55248801781396,48.238537848805,0
-0.55242187799237,48.238453231148,0
-0.55234491716678,48.238372774754,0
-0.55225772108501,48.238297091923,0
-0.5521609533799,48.238226758626,0
-0.55205535051817,48.238162310119,0
-0.55194171619527,48.23810423687,0
-0.55182091521905,48.238052980828,0
-0.55169386692839,48.238008932064,0
-0.55156153819754,48.237972425795,0
-0.55142493607882,48.23794373984,0
-0.55128510013972,48.237923092503,0
-0.55114309455311,48.237910640912,0
-0.55099999999992,48.237906479826,0
-0.55085690544676,48.237910640912,0
-0.55071489986013,48.237923092503,0
-0.55057506392106,48.23794373984,0
-0.55043846180233,48.237972425795,0
-0.55030613307149,48.238008932064,0
-0.5501790847808,48.238052980828,0
-0.55005828380458,48.23810423687,0
-0.54994464948171,48.238162310119,0
-0.54983904661998,48.238226758626,0
-0.54974227891486,48.238297091923,0
-0.54965508283307,48.238372774754,0
-0.5495781220075,48.238453231148,0
-0.54951198218591,48.238537848805,0
-0.54945716677224,48.238625983754,0
-0.54941409299414,48.238716965249,0
-0.54938308872612,48.23881010088,0
-0.54936438999251,48.238904681839,0
-0.54935813916926,48.238999988313,0

-0.55099999999993,48.239,0
</coordinates>
</LinearRing>
</outerBoundaryIs>
</Polygon>
</Placemark>
<Placemark>
<name>346</name>
<styleUrl>#stylehap1</styleUrl>
<Polygon>
<outerBoundaryIs>
<LinearRing>
<coordinates>
-0.55099999999993,48.366,0
-0.5493540490638,48.365999988261,0
-0.54936030933566,48.366095294913,0
-0.5493790487134,48.3661898764,0
-0.54941012464683,48.366283012894,0
-0.54945330069289,48.36637399556,0
-0.54950824831324,48.366462131952,0
-0.54957454937292,48.366546751282,0
-0.54965169932111,48.366627209527,0
-0.54973911102973,48.366702894329,0
-0.54983611926106,48.36677322966,0
-0.54994198573001,48.366837680202,0
-0.55005590472273,48.366895755423,0
-0.55017700922873,48.366947013315,0
-0.55030437753995,48.366991063752,0
-0.55043704026642,48.367027571464,0
-0.55057398771507,48.367056258591,0
-0.55071417757565,48.367076906791,0
-0.55085654285511,48.36708935891,0
-0.55099999999992,48.367093520174,0
-0.55114345714477,48.36708935891,0
-0.55128582242423,48.367076906791,0
-0.55142601228481,48.367056258591,0
-0.55156295973343,48.367027571464,0
-0.5516956224599,48.366991063752,0
-0.55182299077112,48.366947013315,0
-0.55194409527712,48.366895755423,0
-0.55205801426984,48.366837680202,0
-0.55216388073879,48.36677322966,0
-0.55226088897014,48.366702894329,0
-0.55234830067876,48.366627209527,0
-0.55242545062693,48.366546751282,0
-0.55249175168664,48.366462131952,0
-0.55254669930699,48.36637399556,0
-0.55258987535302,48.366283012894,0
-0.55262095128645,48.3661898764,0
-0.55263969066421,48.366095294913,0
-0.55264595093607,48.365999988261,0
-0.55263968452747,48.365904681787,0
-0.55262093919945,48.365810100829,0
-0.55258985768303,48.3657169652,0
-0.55254667659088,48.365625983707,0
-0.55249172461462,48.365537848763,0
-0.5524254200216,48.365453231109,0

-0.55234826747003,48.365372774719,0
-0.55226085416704,48.365297091893,0
-0.55216384539881,48.3652267586,0
-0.55205797946673,48.365162310097,0
-0.55194406206839,48.365104236852,0
-0.55182296016579,48.365052980815,0
-0.55169559538788,48.365008932055,0
-0.55156293701732,48.364972425789,0
-0.55142599461479,48.364943739836,0
-0.55128581033723,48.364923092501,0
-0.55114345100803,48.364910640912,0
-0.55099999999992,48.364906479826,0
-0.55085654899182,48.364910640912,0
-0.55071418966262,48.364923092501,0
-0.55057400538506,48.364943739836,0
-0.55043706298253,48.364972425789,0
-0.55030440461197,48.365008932055,0
-0.55017703983409,48.365052980815,0
-0.55005593793148,48.365104236852,0
-0.54994202053312,48.365162310097,0
-0.54983615460107,48.3652267586,0
-0.54973914583284,48.365297091893,0
-0.54965173252984,48.365372774719,0
-0.54957457997825,48.365453231109,0
-0.54950827538523,48.365537848763,0
-0.54945332340899,48.365625983707,0
-0.54941014231684,48.3657169652,0
-0.5493790608004,48.365810100829,0
-0.54936031547238,48.365904681787,0
-0.5493540490638,48.365999988261,0
-0.55099999999993,48.366,0
</coordinates>
</LinearRing>
</outerBoundaryIs>
</Polygon>
</Placemark>
<Placemark>
<name>347</name>
<styleUrl>#stylehap1</styleUrl>
<Polygon>
<outerBoundaryIs>
<LinearRing>
<coordinates>
-0.55099999999993,48.293,0
-0.54935640354266,48.292999988291,0
-0.54936265486727,48.293095294943,0
-0.54938136744657,48.293189876429,0
-0.54941239893403,48.293283012922,0
-0.54945551322461,48.293373995587,0
-0.54951038224976,48.293462131977,0
-0.54957658847248,48.293546751305,0
-0.54965362806352,48.293627209547,0
-0.54974091473461,48.293702894347,0
-0.54983778419953,48.293773229675,0
-0.5499434992293,48.293837680214,0
-0.55005725526258,48.293895755433,0
-0.55017818652924,48.293947013322,0

-0.55030537263974,48.293991063757,0
-0.55043784559093,48.294027571468,0
-0.55057459713428,48.294056258593,0
-0.55071458645071,48.294076906792,0
-0.5508567480737,48.29408935891,0
-0.55099999999992,48.294093520174,0
-0.55114325192617,48.29408935891,0
-0.55128541354914,48.294076906792,0
-0.55142540286557,48.294056258593,0
-0.55156215440892,48.294027571468,0
-0.55169462736013,48.293991063757,0
-0.55182181347064,48.293947013322,0
-0.55194274473727,48.293895755433,0
-0.55205650077058,48.293837680214,0
-0.55216221580035,48.293773229675,0
-0.55225908526527,48.293702894347,0
-0.55234637193633,48.293627209547,0
-0.55242341152737,48.293546751305,0
-0.55248961775009,48.293462131977,0
-0.55254448677524,48.293373995587,0
-0.55258760106582,48.293283012922,0
-0.55261863255328,48.293189876429,0
-0.55263734513258,48.293095294943,0
-0.55264359645719,48.292999988291,0
-0.55263733902035,48.292904681817,0
-0.5526186205145,48.292810100858,0
-0.55258758346631,48.292716965228,0
-0.55254446414974,48.292625983734,0
-0.55248959078608,48.292537848787,0
-0.55242338104412,48.292453231131,0
-0.55234633886007,48.292372774739,0
-0.55225905060099,48.29229709191,0
-0.5521621806013,48.292226758615,0
-0.5520564661063,48.29216231011,0
-0.551942711661,48.292104236862,0
-0.55182178298739,48.292052980823,0
-0.5516946003961,48.29200893206,0
-0.55156213178342,48.291972425793,0
-0.55142538526606,48.291943739838,0
-0.55128540151036,48.291923092502,0
-0.55114324581393,48.291910640912,0
-0.55099999999992,48.291906479826,0
-0.55085675418594,48.291910640912,0
-0.55071459848949,48.291923092502,0
-0.55057461473379,48.291943739838,0
-0.55043786821643,48.291972425793,0
-0.55030539960375,48.29200893206,0
-0.55017821701249,48.292052980823,0
-0.55005728833885,48.292104236862,0
-0.54994353389355,48.29216231011,0
-0.54983781939855,48.292226758615,0
-0.54974094939889,48.29229709191,0
-0.54965366113978,48.292372774739,0
-0.54957661895573,48.292453231131,0
-0.54951040921379,48.292537848787,0
-0.54945553585011,48.292625983734,0
-0.54941241653354,48.292716965228,0

-0.54938137948535,48.292810100858,0
-0.54936266097953,48.292904681817,0
-0.54935640354266,48.292999988291,0
-0.55099999999993,48.293,0
</coordinates>
</LinearRing>
</outerBoundaryIs>
</Polygon>
</Placemark>
<Placemark>
<name>348</name>
<styleUrl>#stylehap1</styleUrl>
<Polygon>
<outerBoundaryIs>
<LinearRing>
<coordinates>
-0.54999999999995,47.939,0
-0.54836768888705,47.938999988435,0
-0.54837389732597,47.939095295086,0
-0.54839248145638,47.939189876569,0
-0.548423299908,47.939283013057,0
-0.54846611819591,47.939373995715,0
-0.54852061050324,47.939462132096,0
-0.54858636215905,47.939546751413,0
-0.5486628727929,47.939627209644,0
-0.54874956014161,47.939702894432,0
-0.54884576447983,47.939773229748,0
-0.54895075364048,47.939837680274,0
-0.54906372858666,47.939895755481,0
-0.54918382949308,47.939947013359,0
-0.54931014229034,47.939991063783,0
-0.54944170562254,47.940027571485,0
-0.54957751816475,47.940056258602,0
-0.54971654624539,47.940076906796,0
-0.54985773171476,47.940089358911,0
-0.54999999999997,47.940093520173,0
-0.55014226828515,47.940089358911,0
-0.55028345375451,47.940076906796,0
-0.55042248183516,47.940056258602,0
-0.55055829437739,47.940027571485,0
-0.55068985770956,47.939991063783,0
-0.55081617050683,47.939947013359,0
-0.55093627141325,47.939895755481,0
-0.55104924635942,47.939837680274,0
-0.55115423552007,47.939773229748,0
-0.55125043985829,47.939702894432,0
-0.55133712720701,47.939627209644,0
-0.55141363784086,47.939546751413,0
-0.55147938949667,47.939462132096,0
-0.551533881804,47.939373995715,0
-0.5515767000919,47.939283013057,0
-0.55160751854352,47.939189876569,0
-0.55162610267394,47.939095295086,0
-0.55163231111286,47.938999988435,0
-0.55162609667864,47.93890468196,0
-0.55160750673512,47.938810100999,0
-0.55157668282915,47.938716965363,0

-0.55153385961144,47.938625983862,0
-0.55147936304862,47.938537848906,0
-0.5514136079409,47.93845323124,0
-0.55133709476365,47.938372774836,0
-0.55125040585732,47.938297091995,0
-0.55115420099457,47.938226758688,0
-0.55104921235845,47.938162310169,0
-0.55093623896988,47.93810423691,0
-0.55081614060686,47.938052980859,0
-0.5506898312615,47.938008932086,0
-0.55055827218483,47.93797242581,0
-0.55042246457241,47.937943739848,0
-0.55028344194608,47.937923092506,0
-0.55014226228985,47.937910640913,0
-0.54999999999997,47.937906479826,0
-0.54985773771005,47.937910640913,0
-0.54971655805383,47.937923092506,0
-0.5495775354275,47.937943739848,0
-0.5494417278151,47.93797242581,0
-0.54931016873841,47.938008932086,0
-0.54918385939304,47.938052980859,0
-0.54906376103002,47.93810423691,0
-0.54895078764145,47.938162310169,0
-0.54884579900534,47.938226758688,0
-0.54874959414258,47.938297091995,0
-0.54866290523626,47.938372774836,0
-0.54858639205901,47.93845323124,0
-0.54852063695131,47.938537848906,0
-0.54846614038847,47.938625983862,0
-0.54842331717075,47.938716965363,0
-0.54839249326479,47.938810100999,0
-0.54837390332126,47.93890468196,0
-0.54836768888705,47.938999988435,0
-0.54999999999995,47.939,0
</coordinates>
</LinearRing>
</outerBoundaryIs>
</Polygon>
</Placemark>
<Placemark>
<name>349</name>
<styleUrl>#stylehap1</styleUrl>
<Polygon>
<outerBoundaryIs>
<LinearRing>
<coordinates>
-0.548,48.27,0
-0.54635714341794,48.2699999883,0
-0.54636339193094,48.270095294952,0
-0.54638209608905,48.270189876438,0
-0.54641311360972,48.270283012931,0
-0.54645620849416,48.270373995595,0
-0.54651105282144,48.270462131985,0
-0.54657722924244,48.270546751312,0
-0.54665423415467,48.270627209553,0
-0.54674148153375,48.270702894353,0
-0.5468383073925,48.27077322968,0

-0.54694397483377,48.270837680218,0
-0.54705767965844,48.270895755436,0
-0.54717855648606,48.270947013325,0
-0.54730568534152,48.270991063759,0
-0.54743809865746,48.271027571469,0
-0.54757478863921,48.271056258593,0
-0.54771471493634,48.271076906792,0
-0.54785681256199,48.27108935891,0
-0.548,48.271093520174,0
-0.54814318743803,48.27108935891,0
-0.54828528506365,48.271076906792,0
-0.54842521136078,48.271056258593,0
-0.54856190134256,48.271027571469,0
-0.54869431465848,48.270991063759,0
-0.54882144351393,48.270947013325,0
-0.54894232034158,48.270895755436,0
-0.54905602516625,48.270837680218,0
-0.54916169260749,48.27077322968,0
-0.54925851846625,48.270702894353,0
-0.54934576584533,48.270627209553,0
-0.54942277075755,48.270546751312,0
-0.54948894717855,48.270462131985,0
-0.54954379150583,48.270373995595,0
-0.54958688639027,48.270283012931,0
-0.54961790391097,48.270189876438,0
-0.54963660806908,48.270095294952,0
-0.54964285658208,48.2699999883,0
-0.5496366019645,48.269904681826,0
-0.54961789188733,48.269810100868,0
-0.54958686881289,48.269716965237,0
-0.54954376890878,48.269625983742,0
-0.54948892024846,48.269537848795,0
-0.54942274031265,48.269453231138,0
-0.54934573281066,48.269372774745,0
-0.54925848384558,48.269297091916,0
-0.54916165745275,48.26922675862,0
-0.54905599054556,48.269162310114,0
-0.54894228730691,48.269104236865,0
-0.54882141306903,48.269052980825,0
-0.54869428772836,48.269008932062,0
-0.54856187874551,48.268972425794,0
-0.54842519378341,48.268943739839,0
-0.54828527304004,48.268923092502,0
-0.54814318133347,48.268910640912,0
-0.548,48.268906479826,0
-0.54785681866654,48.268910640912,0
-0.54771472695998,48.268923092502,0
-0.54757480621658,48.268943739839,0
-0.54743812125449,48.268972425794,0
-0.54730571227163,48.269008932062,0
-0.54717858693096,48.269052980825,0
-0.5470577126931,48.269104236865,0
-0.54694400945443,48.269162310114,0
-0.54683834254727,48.26922675862,0
-0.54674151615444,48.269297091916,0
-0.54665426718933,48.269372774745,0
-0.54657725968735,48.269453231138,0

-0.54651107975156,48.269537848795,0
-0.54645623109121,48.269625983742,0
-0.5464131311871,48.269716965237,0
-0.54638210811269,48.269810100868,0
-0.54636339803549,48.269904681826,0
-0.54635714341794,48.2699999883,0
-0.548,48.27,0
</coordinates>
</LinearRing>
</outerBoundaryIs>
</Polygon>
</Placemark>
<Placemark>
<name>350</name>
<styleUrl>#stylehap1</styleUrl>
<Polygon>
<outerBoundaryIs>
<LinearRing>
<coordinates>
-0.54600000000005,48.278,0
-0.54435688617568,48.277999988297,0
-0.54436313566622,48.278095294949,0
-0.54438184275225,48.278189876435,0
-0.54441286512895,48.278283012928,0
-0.54445596676059,48.278373995592,0
-0.54451081967491,48.278462131982,0
-0.54457700645747,48.278546751309,0
-0.54465402342692,48.278627209551,0
-0.54474128446719,48.278702894351,0
-0.54483812548707,48.278773229678,0
-0.54494380947406,48.278837680217,0
-0.54505753210312,48.278895755435,0
-0.5451784278583,48.278947013324,0
-0.54530557662039,48.278991063758,0
-0.5454380106705,48.279027571469,0
-0.54557472205621,48.279056258593,0
-0.5457146702641,48.279076906792,0
-0.54585679014054,48.27908935891,0
-0.54600000000005,48.279093520174,0
-0.54614320985954,48.27908935891,0
-0.546285329736,48.279076906792,0
-0.54642527794389,48.279056258593,0
-0.54656198932958,48.279027571469,0
-0.54669442337971,48.278991063758,0
-0.5468215721418,48.278947013324,0
-0.54694246789698,48.278895755435,0
-0.54705619052602,48.278837680217,0
-0.54716187451301,48.278773229678,0
-0.54725871553291,48.278702894351,0
-0.54734597657316,48.278627209551,0
-0.54742299354264,48.278546751309,0
-0.5474891803252,48.278462131982,0
-0.54754403323952,48.278373995592,0
-0.54758713487116,48.278283012928,0
-0.54761815724786,48.278189876435,0
-0.54763686433386,48.278095294949,0
-0.5476431138244,48.277999988297,0

-0.54763685822663,48.277904681823,0
-0.54761814521895,48.277810100864,0
-0.54758711728608,48.277716965234,0
-0.54754401063257,48.277625983739,0
-0.5474891533833,48.277537848792,0
-0.5474229630844,48.277453231136,0
-0.54734594352404,48.277372774743,0
-0.54725868089708,48.277297091914,0
-0.54716183934287,48.277226758618,0
-0.54705615589018,48.277162310112,0
-0.54694243484784,48.277104236864,0
-0.54682154168357,48.277052980824,0
-0.54669439643782,48.277008932061,0
-0.54656196672266,48.276972425793,0
-0.54642526035881,48.276943739839,0
-0.54628531770709,48.276923092502,0
-0.54614320375232,48.276910640912,0
-0.54600000000005,48.276906479826,0
-0.54585679624779,48.276910640912,0
-0.54571468229299,48.276923092502,0
-0.54557473964127,48.276943739839,0
-0.54543803327745,48.276972425793,0
-0.54530560356229,48.277008932061,0
-0.54517845831654,48.277052980824,0
-0.54505756515224,48.277104236864,0
-0.54494384410989,48.277162310112,0
-0.54483816065723,48.277226758618,0
-0.54474131910303,48.277297091914,0
-0.54465405647606,48.277372774743,0
-0.5445770369157,48.277453231136,0
-0.5445108466168,48.277537848792,0
-0.54445598936751,48.277625983739,0
-0.54441288271403,48.277716965234,0
-0.54438185478116,48.277810100864,0
-0.54436314177347,48.277904681823,0
-0.54435688617568,48.277999988297,0
-0.54600000000005,48.278,0
</coordinates>
</LinearRing>
</outerBoundaryIs>
</Polygon>
</Placemark>
<Placemark>
<name>351</name>
<styleUrl>#stylehap1</styleUrl>
<Polygon>
<outerBoundaryIs>
<LinearRing>
<coordinates>
-0.54499999999996,48.169,0
-0.54336038144244,48.168999988342,0
-0.54336661765058,48.169095294993,0
-0.5433852849538,48.169189876478,0
-0.54341624134961,48.16928301297,0
-0.54345925130413,48.169373995632,0
-0.5435139875424,48.169462132019,0
-0.54358003353773,48.169546751343,0

-0.54365688668002,48.169627209581,0
-0.54374396209973,48.169702894377,0
-0.54384059711836,48.169773229701,0
-0.54394605629111,48.169837680235,0
-0.54405953700392,48.16989575545,0
-0.54418017558212,48.169947013335,0
-0.54430705386394,48.169991063766,0
-0.54443920618924,48.170027571474,0
-0.54457562674999,48.170056258596,0
-0.54471527724647,48.170076906793,0
-0.54485709479118,48.170089358911,0
-0.54499999999997,48.170093520174,0
-0.54514290520873,48.170089358911,0
-0.54528472275346,48.170076906793,0
-0.54542437324994,48.170056258596,0
-0.54556079381067,48.170027571474,0
-0.54569294613599,48.169991063766,0
-0.54581982441781,48.169947013335,0
-0.5459404629960l,48.16989575545,0
-0.54605394370882,48.169837680235,0
-0.54615940288157,48.169773229701,0
-0.54625603790017,48.169702894377,0
-0.54634311331992,48.169627209581,0
-0.54641996646218,48.169546751343,0
-0.54648601245751,48.169462132019,0
-0.5465407486958,48.169373995632,0
-0.5465837586503,48.16928301297,0
-0.54661471504613,48.169189876478,0
-0.54663338234932,48.169095294993,0
-0.54663961855746,48.168999988342,0
-0.54663337627838,48.168904681867,0
-0.5466147030887,48.168810100908,0
-0.54658374116969,48.168716965275,0
-0.54654072622317,48.168625983779,0
-0.54648598567566,48.168537848829,0
-0.54641993618488,48.16845323117,0
-0.54634308046713,48.168372774773,0
-0.54625600347011,48.16829709194,0
-0.54615936792035,48.168226758641,0
-0.54605390927876,48.168162310131,0
-0.54594043014322,48.168104236879,0
-0.54581979414051,48.168052980836,0
-0.54569291935414,48.168008932069,0
-0.54556077133804,48.167972425799,0
-0.54542435576933,48.167943739842,0
-0.54528471079603,48.167923092504,0
-0.54514289913779,48.167910640912,0
-0.54499999999997,48.167906479826,0
-0.54485710086212,48.167910640912,0
-0.5447152892039,48.167923092504,0
-0.54457564423058,48.167943739842,0
-0.54443922866187,48.167972425799,0
-0.54430708064579,48.168008932069,0
-0.5441802058594,48.168052980836,0
-0.54405956985671,48.168104236879,0
-0.54394609072117,48.168162310131,0
-0.54384063207958,48.168226758641,0

-0.5437439965298,48.16829709194,0
-0.54365691953281,48.168372774773,0
-0.54358006381503,48.16845323117,0
-0.54351401432424,48.168537848829,0
-0.54345927377676,48.168625983779,0
-0.54341625883022,48.168716965275,0
-0.54338529691123,48.168810100908,0
-0.54336662372155,48.168904681867,0
-0.54336038144244,48.168999988342,0
-0.54499999999996,48.169,0
</coordinates>
</LinearRing>
</outerBoundaryIs>
</Polygon>
</Placemark>
<Placemark>
<name>352</name>
<styleUrl>#stylehap1</styleUrl>
<Polygon>
<outerBoundaryIs>
<LinearRing>
<coordinates>
-0.54499999999996,48.205,0
-0.54335922934475,48.204999988327,0
-0.54336546993099,48.205095294978,0
-0.54338415034728,48.205189876464,0
-0.54341512849149,48.205283012956,0
-0.54345816866428,48.205373995619,0
-0.54351294336093,48.205462132006,0
-0.54357903576205,48.205546751332,0
-0.54365594290448,48.205627209571,0
-0.54374307950779,48.205702894368,0
-0.54383978242786,48.205773229693,0
-0.54394531570307,48.205837680229,0
-0.54405887615547,48.205895755445,0
-0.54417959950347,48.205947013331,0
-0.54430656694005,48.205991063764,0
-0.5444388121265,48.206027571472,0
-0.54457532854796,48.206056258595,0
-0.54471507717502,48.206076906793,0
-0.54485699437328,48.20608935891,0
-0.54499999999997,48.206093520173,0
-0.54514300562665,48.20608935891,0
-0.54528492282491,48.206076906793,0
-0.54542467145198,48.206056258595,0
-0.54556118787343,48.206027571472,0
-0.54569343305988,48.205991063764,0
-0.54582040049647,48.205947013331,0
-0.54594112384444,48.205895755445,0
-0.54605468429683,48.205837680229,0
-0.54616021757208,48.205773229693,0
-0.54625692049211,48.205702894368,0
-0.54634405709545,48.205627209571,0
-0.54642096423786,48.205546751332,0
-0.54648705663898,48.205462132006,0
-0.54654183133563,48.205373995619,0
-0.54658487150841,48.205283012956,0

-0.54661584965266,48.205189876464,0
-0.54663453006892,48.205095294978,0
-0.54664077065516,48.204999988327,0
-0.54663452398602,48.204904681853,0
-0.54661583767166,48.204810100893,0
-0.54658485399341,48.204716965262,0
-0.54654180881875,48.204625983766,0
-0.54648702980441,48.204537848817,0
-0.54642093390096,48.204453231158,0
-0.54634402417795,48.204372774763,0
-0.54625688599426,48.204297091932,0
-0.54616018254203,48.204226758633,0
-0.54605464979899,48.204162310125,0
-0.54594109092697,48.204104236874,0
-0.54582037015955,48.204052980832,0
-0.54569340622531,48.204008932067,0
-0.54556116535655,48.203972425797,0
-0.54542465393694,48.203943739841,0
-0.54528491084391,48.203923092503,0
-0.54514299954375,48.203910640912,0
-0.54499999999997,48.203906479826,0
-0.54485700045618,48.203910640912,0
-0.544715089156,48.203923092503,0
-0.54457534606297,48.203943739841,0
-0.54443883464338,48.203972425797,0
-0.54430659377462,48.204008932067,0
-0.54417962984036,48.204052980832,0
-0.54405890907297,48.204104236874,0
-0.54394535020095,48.204162310125,0
-0.5438398174579,48.204226758633,0
-0.54374311400567,48.204297091932,0
-0.54365597582195,48.204372774763,0
-0.54357906609897,48.204453231158,0
-0.5435129701955,48.204537848817,0
-0.54345819118116,48.204625983766,0
-0.5434151460065,48.204716965262,0
-0.54338416232825,48.204810100893,0
-0.54336547601391,48.204904681853,0
-0.54335922934475,48.204999988327,0
-0.54499999999996,48.205,0
</coordinates>
</LinearRing>
</outerBoundaryIs>
</Polygon>
</Placemark>
<Placemark>
<name>360</name>
<styleUrl>#stylehap1</styleUrl>
<Polygon>
<outerBoundaryIs>
<LinearRing>
<coordinates>
-0.53899999999999,48.176,0
-0.53736015760092,48.175999988339,0
-0.53736639465967,48.17609529499,0
-0.53738506451061,48.176189876476,0
-0.53741602513196,48.176283012967,0

-0.53745904095758,48.176373995629,0
-0.53751378466796,48.176462132016,0
-0.53757983967948,48.176546751341,0
-0.53765670331347,48.176627209579,0
-0.53774379062058,48.176702894375,0
-0.53784043883182,48.176773229699,0
-0.53794591240196,48.176837680234,0
-0.53805940860741,48.176895755449,0
-0.53818006365557,48.176947013334,0
-0.5383069592593,48.176991063766,0
-0.53843912962664,48.177027571474,0
-0.53857556881221,48.177056258596,0
-0.53871523837451,48.177076906793,0
-0.53885707528096,48.177089358911,0
-0.53899999999998,48.177093520174,0
-0.53914292471901,48.177089358911,0
-0.53928476162546,48.177076906793,0
-0.53942443118778,48.177056258596,0
-0.53956087037333,48.177027571474,0
-0.53969304074067,48.176991063766,0
-0.53981993634442,48.176947013334,0
-0.53994059139258,48.176895755449,0
-0.54005408759802,48.176837680234,0
-0.54015956116817,48.176773229699,0
-0.54025620937939,48.176702894375,0
-0.54034329668649,48.176627209579,0
-0.54042016032049,48.176546751341,0
-0.54048621533203,48.176462132016,0
-0.54054095904239,48.176373995629,0
-0.54058397486803,48.176283012967,0
-0.54061493548935,48.176189876476,0
-0.54063360534029,48.17609529499,0
-0.54063984239907,48.175999988339,0
-0.54063359926704,48.175904681864,0
-0.54061492352733,48.175810100905,0
-0.54058395738073,48.175716965273,0
-0.54054093656116,48.175625983776,0
-0.54048618853993,48.175537848827,0
-0.54042013003161,48.175453231167,0
-0.54034326382116,48.175372774771,0
-0.54025617493614,48.175297091939,0
-0.54015952619357,48.175226758639,0
-0.54005405315478,48.17516231013,0
-0.53994055852722,48.175104236878,0
-0.53981990605554,48.175052980835,0
-0.53969301394859,48.175008932069,0
-0.5395608478921,48.174972425798,0
-0.53942441370048,48.174943739842,0
-0.53928474966344,48.174923092503,0
-0.53914291864572,48.174910640912,0
-0.53899999999998,48.174906479827,0
-0.53885708135424,48.174910640912,0
-0.53871525033652,48.174923092503,0
-0.53857558629949,48.174943739842,0
-0.53843915210787,48.174972425798,0
-0.5383069860514,48.175008932069,0
-0.53818009394445,48.175052980835,0

-0.53805944147277,48.175104236878,0
-0.53794594684518,48.17516231013,0
-0.53784047380639,48.175226758639,0
-0.53774382506382,48.175297091939,0
-0.53765673617883,48.175372774771,0
-0.53757986996836,48.175453231167,0
-0.53751381146004,48.175537848827,0
-0.5374590634388,48.175625983776,0
-0.53741604261923,48.17571696527,0
-0.53738507647263,48.175810100905,0
-0.53736640073295,48.175904681864,0
-0.53736015760092,48.175999988339,0
-0.53899999999999,48.176,0
</coordinates>
</LinearRing>
</outerBoundaryIs>
</Polygon>
</Placemark>
<Placemark>
<name>361</name>
<styleUrl>#stylehap1</styleUrl>
<Polygon>
<outerBoundaryIs>
<LinearRing>
<coordinates>
-0.53800000000001,48.118,0
-0.53636200970813,48.117999988362,0
-0.53636823972867,48.118095295014,0
-0.53638688849907,48.118189876499,0
-0.5364178141578,48.118283012989,0
-0.53646078140462,48.11837399565,0
-0.53651546328952,48.118462132036,0
-0.53658144369927,48.118546751358,0
-0.53665822052285,48.118627209595,0
-0.53674520947148,48.118702894389,0
-0.53684174852468,48.118773229711,0
-0.536947102968,48.118837680244,0
-0.53706047098445,48.118895755457,0
-0.53718098975708,48.11894701334,0
-0.53730774203601,48.11899106377,0
-0.53743976312017,48.119027571476,0
-0.53757604820049,48.119056258598,0
-0.5377155600085,48.119076906794,0
-0.53785723671237,48.119089358911,0
-0.53800000000002,48.119093520174,0
-0.53814276328765,48.119089358911,0
-0.53828443999152,48.119076906794,0
-0.53842395179953,48.119056258598,0
-0.53856023687985,48.119027571476,0
-0.53869225796401,48.11899106377,0
-0.53881901024294,48.11894701334,0
-0.53893952901557,48.118895755457,0
-0.53905289703202,48.118837680244,0
-0.53915825147534,48.118773229711,0
-0.53925479052854,48.118702894389,0
-0.53934177947717,48.118627209595,0
-0.53941855630075,48.118546751358,0

-0.53948453671052,48.118462132036,0
-0.53953921859543,48.11837399565,0
-0.53958218584222,48.118283012989,0
-0.53961311150095,48.118189876499,0
-0.53963176027135,48.118095295014,0
-0.53963799029189,48.117999988362,0
-0.53963175421728,48.117904681888,0
-0.53961309957677,48.117810100928,0
-0.53958216841024,48.117716965295,0
-0.53953919618526,48.117625983797,0
-0.53948451000315,48.117537848846,0
-0.53941852610764,48.117453231185,0
-0.53934174671572,48.117372774787,0
-0.5392547561942,48.117297091952,0
-0.53915821661134,48.117226758651,0
-0.53905286269771,48.117162310139,0
-0.53893949625416,48.117104236886,0
-0.53881898004983,48.117052980841,0
-0.53869223125664,48.117008932073,0
-0.53856021446969,48.116972425801,0
-0.53842393436754,48.116943739843,0
-0.53828442806734,48.116923092504,0
-0.53814275723358,48.116910640913,0
-0.53800000000002,48.116906479827,0
-0.53785724276644,48.116910640913,0
-0.53771557193268,48.116923092504,0
-0.5375760656325,48.116943739843,0
-0.53743978553033,48.116972425801,0
-0.53730776874338,48.117008932073,0
-0.53718101995018,48.117052980841,0
-0.53706050374589,48.117104236886,0
-0.53694713730231,48.117162310139,0
-0.53684178338868,48.117226758651,0
-0.53674524380582,48.117297091952,0
-0.53665825328429,48.117372774787,0
-0.53658147389238,48.117453231185,0
-0.53651548999687,48.117537848846,0
-0.53646080381475,48.117625983797,0
-0.53641783158978,48.117716965295,0
-0.53638690042328,48.117810100928,0
-0.53636824578274,48.117904681888,0
-0.53636200970813,48.117999988362,0
-0.53800000000001,48.118,0
</coordinates>
</LinearRing>
</outerBoundaryIs>
</Polygon>
</Placemark>
<Placemark>
<name>362</name>
<styleUrl>#stylehap1</styleUrl>
<Polygon>
<outerBoundaryIs>
<LinearRing>
<coordinates>
-0.53800000000001,48.031,0
-0.5363647769006,48.030999988398,0

-0.53637099640546,48.031095295049,0
-0.5363896136799,48.031189876533,0
-0.53642048710173,48.031283013022,0
-0.53646338176801,48.031373995681,0
-0.53651797128083,48.031462132065,0
-0.53658384022972,48.031546751385,0
-0.53666048735178,48.031627209619,0
-0.53674732934517,48.03170289441,0
-0.53684370530777,48.031773229729,0
-0.5369488817663,48.031837680259,0
-0.53706205825838,48.031895755469,0
-0.53718237342477,48.031947013349,0
-0.5373089115653,48.031991063776,0
-0.53744070960879,48.032027571481,0
-0.53757676444387,48.0320562586,0
-0.53771604055466,48.032076906795,0
-0.53785747790342,48.032089358911,0
-0.53800000000002,48.032093520174,0
-0.5381425220966,48.032089358911,0
-0.53828395944536,48.032076906795,0
-0.53842323555614,48.0320562586,0
-0.53855929039123,48.032027571481,0
-0.53869108843472,48.031991063776,0
-0.53881762657525,48.031947013349,0
-0.53893794174164,48.031895755469,0
-0.53905111823372,48.031837680259,0
-0.53915629469225,48.031773229729,0
-0.53925267065485,48.03170289441,0
-0.53933951264824,48.031627209619,0
-0.5394161597703,48.031546751385,0
-0.53948202871922,48.031462132065,0
-0.53953661823201,48.031373995681,0
-0.53957951289829,48.031283013022,0
-0.53961038632012,48.031189876533,0
-0.53962900359456,48.031095295049,0
-0.53963522309942,48.030999988398,0
-0.53962899756914,48.030904681923,0
-0.53961037445238,48.030810100962,0
-0.53957949554882,48.030716965328,0
-0.53953659592798,48.030625983829,0
-0.53948200213828,48.030537848875,0
-0.53941612972012,48.030453231212,0
-0.53933948004191,48.030372774811,0
-0.53925263648305,48.030297091973,0
-0.53915625999331,48.030226758669,0
-0.53905108406194,48.030162310154,0
-0.53893790913528,48.030104236898,0
-0.53881759652506,48.03005298085,0
-0.53869106185378,48.030008932079,0
-0.53855926808717,48.029972425805,0
-0.53842321820668,48.029943739846,0
-0.53828394757764,48.029923092505,0
-0.5381425160712,48.029910640913,0
-0.53800000000002,48.029906479827,0
-0.53785748392882,48.029910640913,0
-0.53771605242238,48.029923092505,0
-0.53757678179334,48.029943739846,0

-0.53744073191285,48.029972425805,0
-0.53730893814624,48.030008932079,0
-0.53718240347496,48.03005298085,0
-0.53706209086474,48.030104236898,0
-0.53694891593808,48.030162310154,0
-0.53684374000673,48.030226758669,0
-0.53674736351697,48.030297091973,0
-0.53666051995811,48.030372774811,0
-0.5365838702799,48.030453231212,0
-0.53651799786174,48.030537848875,0
-0.53646340407204,48.030625983829,0
-0.5364205044512,48.030716965328,0
-0.53638962554764,48.030810100962,0
-0.53637100243088,48.030904681923,0
-0.5363647769006,48.030999988398,0
-0.53800000000001,48.031,0
</coordinates>
</LinearRing>
</outerBoundaryIs>
</Polygon>
</Placemark>
<Placemark>
<name>363</name>
<styleUrl>#stylehap1</styleUrl>
<Polygon>
<outerBoundaryIs>
<LinearRing>
<coordinates>
-0.53800000000001,48.117,0
-0.5363620415896,48.116999988363,0
-0.536368271489,48.117095295014,0
-0.53638691989654,48.117189876499,0
-0.53641784495341,48.117283012989,0
-0.53646081136402,48.11737399565,0
-0.53651549218468,48.117462132036,0
-0.53658147131028,48.117546751359,0
-0.53665824663953,48.117627209595,0
-0.53674523389506,48.117702894389,0
-0.53684177106924,48.117773229711,0
-0.53694712346194,48.117837680244,0
-0.5370604892718,48.117895755457,0
-0.53718100569863,48.11794701334,0
-0.53730775551044,48.11799106377,0
-0.53743977402489,48.118027571476,0
-0.5375760564525,48.118056258598,0
-0.53771556554498,48.118076906794,0
-0.53785723949118,48.118089358911,0
-0.53800000000002,48.118093520174,0
-0.53814276050884,48.118089358911,0
-0.53828443445504,48.118076906794,0
-0.53842394354751,48.118056258598,0
-0.53856022597513,48.118027571476,0
-0.53869224448958,48.11799106377,0
-0.53881899430139,48.11794701334,0
-0.53893951072822,48.117895755457,0
-0.53905287653808,48.117837680244,0
-0.53915822893077,48.117773229711,0

-0.53925476610496,48.117702894389,0
-0.53934175336049,48.117627209595,0
-0.53941852868974,48.117546751359,0
-0.53948450781534,48.117462132036,0
-0.539539188636,48.11737399565,0
-0.53958215504661,48.117283012989,0
-0.53961308010348,48.117189876499,0
-0.53963172851102,48.117095295014,0
-0.53963795841042,48.116999988363,0
-0.53963172245728,48.116904681888,0
-0.53961306817996,48.116810100928,0
-0.53958213761557,48.116716965295,0
-0.53953916622709,48.116625983798,0
-0.53948448110944,48.116537848847,0
-0.53941849849829,48.116453231185,0
-0.53934172060085,48.116372774787,0
-0.5392547317725,48.116297091953,0
-0.53915819406869,48.116226758651,0
-0.53905284220562,48.11616231014,0
-0.53893947796856,48.116104236886,0
-0.53881896410994,48.116052980841,0
-0.53869221778368,48.116008932073,0
-0.53856020356621,48.115972425801,0
-0.53842392611647,48.115943739843,0
-0.5382844225315,48.115923092504,0
-0.5381427544551,48.115910640913,0
-0.53800000000002,48.115906479826,0
-0.53785724554492,48.115910640913,0
-0.53771557746852,48.115923092504,0
-0.53757607388355,48.115943739843,0
-0.53743979643384,48.115972425801,0
-0.53730778221634,48.116008932073,0
-0.53718103589008,48.116052980841,0
-0.53706052203146,48.116104236886,0
-0.5369471577944,48.11616231014,0
-0.53684180593133,48.116226758651,0
-0.53674526822752,48.116297091953,0
-0.53665827939917,48.116372774787,0
-0.53658150150173,48.116453231185,0
-0.53651551889058,48.116537848847,0
-0.53646083377293,48.116625983798,0
-0.53641786238447,48.116716965295,0
-0.53638693182006,48.116810100928,0
-0.53636827754274,48.116904681888,0
-0.5363620415896,48.116999988363,0
-0.53800000000001,48.117,0
</coordinates>
</LinearRing>
</outerBoundaryIs>
</Polygon>
</Placemark>
<Placemark>
<name>367</name>
<styleUrl>#stylehap1</styleUrl>
<Polygon>
<outerBoundaryIs>
<LinearRing>

<coordinates>
-0.53399999999999,48.243,0
-0.5323580107801,48.242999988311,0
-0.53236425599702,48.243095294963,0
-0.53238295028289,48.243189876449,0
-0.5324139514302,48.243283012941,0
-0.53245702356467,48.243373995605,0
-0.53251183893839,48.243462131994,0
-0.53257798042257,48.24354675132,0
-0.53265494468054,48.243627209561,0
-0.53274214599725,48.243702894359,0
-0.5328389207361,48.243773229686,0
-0.53294453238896,48.243837680223,0
-0.53305817718129,48.24389575544,0
-0.53317899018961,48.243947013328,0
-0.53330605192447,48.243991063761,0
-0.53343839532946,48.24402757147,0
-0.53357501314201,48.244056258594,0
-0.53371486556106,48.244076906792,0
-0.53385688816208,48.24408935891,0
-0.53399999999998,48.244093520174,0
-0.53414311183791,48.24408935891,0
-0.53428513443893,48.244076906792,0
-0.53442498685796,48.244056258594,0
-0.53456160467053,48.24402757147,0
-0.53469394807549,48.243991063761,0
-0.53482100981038,48.243947013328,0
-0.53494182281869,48.24389575544,0
-0.53505546761103,48.243837680223,0
-0.53516107926387,48.243773229686,0
-0.53525785400274,48.243702894359,0
-0.53534505531945,48.243627209561,0
-0.53542201957742,48.24354675132,0
-0.5354881610616,48.243462131994,0
-0.53554297643532,48.243373995605,0
-0.53558604856976,48.243283012941,0
-0.5356170497171,48.243189876449,0
-0.53563574400297,48.243095294963,0
-0.53564198921989,48.242999988311,0
-0.5356357379074,48.242904681837,0
-0.53561703771119,48.242810100878,0
-0.53558603101834,48.242716965247,0
-0.5355429538716,48.242625983752,0
-0.53548813417123,48.242537848804,0
-0.53542198917742,48.242453231147,0
-0.53534502233353,48.242372774753,0
-0.53525781943314,48.242297091922,0
-0.535161044161,48.242226758626,0
-0.53505543304143,48.242162310118,0
-0.53494178983276,48.242104236869,0
-0.53482097941038,48.242052980828,0
-0.53469392118512,48.242008932064,0
-0.53456158210683,48.241972425795,0
-0.53442496930651,48.24194373984,0
-0.53428512243302,48.241923092503,0
-0.53414310574236,48.241910640912,0
-0.53399999999998,48.241906479826,0

-0.53385689425763,48.241910640912,0
-0.53371487756696,48.241923092503,0
-0.53357503069345,48.24194373984,0
-0.53343841789315,48.241972425795,0
-0.53330607881484,48.242008932064,0
-0.53317902058958,48.242052980828,0
-0.53305821016721,48.242104236869,0
-0.53294456695856,48.242162310118,0
-0.53283895583899,48.242226758626,0
-0.53274218056685,48.242297091922,0
-0.53265497766646,48.242372774753,0
-0.53257801082257,48.242453231147,0
-0.53251186582876,48.242537848804,0
-0.53245704612836,48.242625983752,0
-0.53241396898165,48.242716965247,0
-0.5323829622888,48.242810100878,0
-0.53236426209259,48.242904681837,0
-0.5323580107801,48.242999988311,0
-0.53399999999999,48.243,0
</coordinates>
</LinearRing>
</outerBoundaryIs>
</Polygon>
</Placemark>
<Placemark>
<name>368</name>
<styleUrl>#stylehap1</styleUrl>
<Polygon>
<outerBoundaryIs>
<LinearRing>
<coordinates>
-0.53399999999999,48.269,0
-0.53235717556529,48.268999988301,0
-0.53236342395613,48.269095294952,0
-0.53238212774835,48.269189876439,0
-0.53241314466218,48.269283012931,0
-0.53245623870344,48.269373995595,0
-0.53251108195757,48.269462131985,0
-0.5325772570837,48.269546751312,0
-0.53265426048914,48.269627209554,0
-0.53274150616099,48.269702894353,0
-0.53283833012508,48.26977322968,0
-0.53294399549863,48.269837680218,0
-0.53305769809829,48.269895755437,0
-0.53317857256057,48.269947013325,0
-0.53330569892832,48.269991063759,0
-0.53343810965311,48.270027571469,0
-0.53357479696004,48.270056258593,0
-0.53371472051899,48.270076906792,0
-0.53385681536397,48.27008935891,0
-0.53399999999998,48.270093520174,0
-0.53414318463602,48.27008935891,0
-0.534285279481,48.270076906792,0
-0.53442520303995,48.270056258593,0
-0.53456189034688,48.270027571469,0
-0.53469430107167,48.269991063759,0
-0.53482142743942,48.269947013325,0

-0.53494230190168,48.269895755437,0
-0.53505600450136,48.269837680218,0
-0.53516166987491,48.26977322968,0
-0.53525849383899,48.269702894353,0
-0.53534573951083,48.269627209554,0
-0.53542274291628,48.269546751312,0
-0.53548891804239,48.269462131985,0
-0.53554376129655,48.269373995595,0
-0.53558685533781,48.269283012931,0
-0.53561787225164,48.269189876439,0
-0.53563657604386,48.269095294952,0
-0.53564282443469,48.268999988301,0
-0.53563656993964,48.268904681827,0
-0.53561786022866,48.268810100868,0
-0.53558683776138,48.268716965237,0
-0.53554373870075,48.268625983743,0
-0.53548889111375,48.268537848795,0
-0.53542271247303,48.268453231139,0
-0.53534570647797,48.268372774745,0
-0.53525845922018,48.268297091916,0
-0.53516163472207,48.26822675862,0
-0.53505596988257,48.268162310114,0
-0.53494226886882,48.268104236865,0
-0.53482139699617,48.268052980825,0
-0.53469427414303,48.268008932062,0
-0.53456186775105,48.267972425794,0
-0.53442518546352,48.267943739839,0
-0.53428526745802,48.267923092502,0
-0.5341431785318,48.267910640912,0
-0.53399999999998,48.267906479827,0
-0.53385682146819,48.267910640912,0
-0.53371473254197,48.267923092502,0
-0.53357481453644,48.267943739839,0
-0.53343813224891,48.267972425794,0
-0.53330572585696,48.268008932062,0
-0.53317860300382,48.268052980825,0
-0.53305773113115,48.268104236865,0
-0.53294403011742,48.268162310114,0
-0.53283836527792,48.26822675862,0
-0.53274154077978,48.268297091916,0
-0.53265429352202,48.268372774745,0
-0.53257728752696,48.268453231139,0
-0.53251110888621,48.268537848795,0
-0.53245626129924,48.268625983743,0
-0.53241316223858,48.268716965237,0
-0.53238213977133,48.268810100868,0
-0.53236343006035,48.268904681827,0
-0.53235717556529,48.268999988301,0
-0.53399999999999,48.269,0
</coordinates>
</LinearRing>
</outerBoundaryIs>
</Polygon>
</Placemark>
<Placemark>
<name>371</name>
<styleUrl>#stylehap1</styleUrl>

<Polygon>
<outerBoundaryIs>
<LinearRing>
<coordinates>
-0.53300000000002,48.26,0
-0.53135746481256,48.259999988304,0
-0.53136371210422,48.260095294956,0
-0.53138241260424,48.260189876442,0
-0.53141342405789,48.260283012935,0
-0.53145651051251,48.260373995599,0
-0.53151134411125,48.260462131988,0
-0.53157750758667,48.260546751315,0
-0.53165449743478,48.260627209556,0
-0.53174172774578,48.260702894355,0
-0.53183853466242,48.260773229682,0
-0.53194418143171,48.26083768022,0
-0.53205786401184,48.260895755438,0
-0.5321787171917,48.260947013326,0
-0.53230582117614,48.26099106376,0
-0.53243820858708,48.26102757147,0
-0.53257487182708,48.261056258594,0
-0.53271477074922,48.261076906792,0
-0.53285684057504,48.26108935891,0
-0.53300000000002,48.261093520173,0
-0.53314315942498,48.26108935891,0
-0.5332852292508,48.261076906792,0
-0.53342512817297,48.261056258594,0
-0.53356179141295,48.26102757147,0
-0.53369417882391,48.26099106376,0
-0.53382128280832,48.260947013326,0
-0.53394213598818,48.260895755438,0
-0.53405581856831,48.26083768022,0
-0.5341614653376,48.260773229682,0
-0.53425827225424,48.260702894355,0
-0.53434550256527,48.260627209556,0
-0.53442249241337,48.260546751315,0
-0.53448865588877,48.260462131988,0
-0.53454348948754,48.260373995599,0
-0.53458657594213,48.260283012935,0
-0.53461758739578,48.260189876442,0
-0.53463628789582,48.260095294956,0
-0.53464253518749,48.259999988304,0
-0.5346362817946,48.25990468183,0
-0.53461757537873,48.259810100872,0
-0.53458655837437,48.259716965241,0
-0.53454346690285,48.259625983746,0
-0.53448862897339,48.259537848798,0
-0.53442246198508,48.259453231142,0
-0.53434546954867,48.259372774748,0
-0.53425823765251,48.259297091918,0
-0.53416143020206,48.259226758622,0
-0.53405578396657,48.259162310115,0
-0.53394210297158,48.259104236867,0
-0.53382125238005,48.259052980826,0
-0.5336941519085,48.259008932063,0
-0.53356176882826,48.258972425794,0
-0.53342511060519,48.258943739839,0

-0.53328521723375,48.258923092502,0
-0.53314315332376,48.258910640912,0
-0.53300000000002,48.258906479826,0
-0.53285684667626,48.258910640912,0
-0.5327147827663,48.258923092502,0
-0.53257488939483,48.258943739839,0
-0.53243823117176,48.258972425794,0
-0.53230584809152,48.259008932063,0
-0.53217874761997,48.259052980826,0
-0.53205789702846,48.259104236867,0
-0.53194421603347,48.259162310115,0
-0.53183856979796,48.259226758622,0
-0.53174176234754,48.259297091918,0
-0.53165453045138,48.259372774748,0
-0.53157753801494,48.259453231142,0
-0.53151137102663,48.259537848798,0
-0.5314565330972,48.259625983746,0
-0.53141344162567,48.259716965241,0
-0.53138242462131,48.259810100872,0
-0.53136371820544,48.25990468183,0
-0.53135746481256,48.259999988304,0
-0.53300000000002,48.26,0
</coordinates>
</LinearRing>
</outerBoundaryIs>
</Polygon>
</Placemark>
<Placemark>
<name>372</name>
<styleUrl>#stylehap1</styleUrl>
<Polygon>
<outerBoundaryIs>
<LinearRing>
<coordinates>
-0.53300000000002,48.159,0
-0.53136070106776,48.158999988346,0
-0.53136693606129,48.159095294997,0
-0.53138559972653,48.159189876482,0
-0.53141655008873,48.159283012973,0
-0.53145955165979,48.159373995635,0
-0.53151427722862,48.159462132022,0
-0.53158031034965,48.159546751346,0
-0.53165714851071,48.159627209584,0
-0.53174420695635,48.159702894379,0
-0.53184082313713,48.159773229703,0
-0.53194626175171,48.159837680237,0
-0.53205972034245,48.159895755451,0
-0.53218033540306,48.159947013336,0
-0.53230718895078,48.159991063767,0
-0.53243931551371,48.160027571474,0
-0.53257570947992,48.160056258596,0
-0.53271533275211,48.160076906794,0
-0.53285712265007,48.160089358911,0
-0.53300000000002,48.160093520174,0
-0.53314287734995,48.160089358911,0
-0.53328466724791,48.160076906794,0
-0.53342429052013,48.160056258596,0

-0.53356068448633,48.160027571474,0
-0.53369281104927,48.159991063767,0
-0.53381966459698,48.159947013336,0
-0.53394027965757,48.159895755451,0
-0.53405373824833,48.159837680237,0
-0.53415917686289,48.159773229703,0
-0.5342557930437,48.159702894379,0
-0.53434285148933,48.159627209584,0
-0.53441968965039,48.159546751346,0
-0.5344857227714,48.159462132022,0
-0.53454044834023,48.159373995635,0
-0.53458344991129,48.159283012973,0
-0.53461440027349,48.159189876482,0
-0.53463306393873,48.159095294997,0
-0.53463929893226,48.158999988346,0
-0.5346330578711,48.158904681871,0
-0.53461438832259,48.158810100912,0
-0.53458343244025,48.158716965279,0
-0.53454042587986,48.158625983783,0
-0.53448569600418,48.158537848832,0
-0.53441965938963,48.158453231173,0
-0.53434281865446,48.158372774776,0
-0.53425575863241,48.158297091943,0
-0.53415914192078,48.158226758643,0
-0.53405370383705,48.158162310132,0
-0.53394024682272,48.15810423688,0
-0.53381963433622,48.158052980837,0
-0.53369278428205,48.15800893207,0
-0.53356066202597,48.157972425799,0
-0.53342427304906,48.157943739842,0
-0.53328465529701,48.157923092504,0
-0.53314287128232,48.157910640912,0
-0.53300000000002,48.157906479827,0
-0.5328571287177,48.157910640912,0
-0.53271534470304,48.157923092504,0
-0.53257572695098,48.157943739842,0
-0.53243933797408,48.157972425799,0
-0.532307215718,48.15800893207,0
-0.53218036566383,48.158052980837,0
-0.5320597531773,48.15810423688,0
-0.53194629616298,48.158162310132,0
-0.53184085807927,48.158226758643,0
-0.53174424136761,48.158297091943,0
-0.53165718134556,48.158372774776,0
-0.53158034061042,48.158453231173,0
-0.53151430399584,48.158537848832,0
-0.53145957412016,48.158625983783,0
-0.5314165675598,48.158716965279,0
-0.53138561167746,48.158810100912,0
-0.53136694212892,48.158904681871,0
-0.53136070106776,48.158999988346,0
-0.53300000000002,48.159,0
</coordinates>
</LinearRing>
</outerBoundaryIs>
</Polygon>
</Placemark>

<Placemark>
<name>377</name>
<styleUrl>#stylehap1</styleUrl>
<Polygon>
<outerBoundaryIs>
<LinearRing>
<coordinates>
-0.52800000000002,48.196,0
-0.52635951758174,48.19599998833,0
-0.52636575707264,48.196095294982,0
-0.52638443420823,48.196189876468,0
-0.52641540691133,48.196283012959,0
-0.52645843952399,48.196373995622,0
-0.52651320459895,48.19646213201,0
-0.52657928539005,48.196546751335,0
-0.52665617902248,48.196627209574,0
-0.52674330031862,48.19670289437,0
-0.52683998625078,48.196773229695,0
-0.52694550098672,48.196837680231,0
-0.52705904148954,48.196895755446,0
-0.52717974362942,48.196947013332,0
-0.52730668876087,48.196991063764,0
-0.52743891071493,48.197027571473,0
-0.52757540315352,48.197056258595,0
-0.52771512722984,48.197076906793,0
-0.52785701949633,48.19708935891,0
-0.52800000000002,48.197093520174,0
-0.52814298050371,48.19708935891,0
-0.52828487277021,48.197076906793,0
-0.52842459684653,48.197056258595,0
-0.52856108928512,48.197027571473,0
-0.52869331123915,48.196991063764,0
-0.5288202563706,48.196947013332,0
-0.52894095851051,48.196895755446,0
-0.5290544990133,48.196837680231,0
-0.52916001374924,48.196773229695,0
-0.52925669968142,48.19670289437,0
-0.52934382097756,48.196627209574,0
-0.52942071460999,48.196546751335,0
-0.5294867954011,48.19646213201,0
-0.52954156047606,48.196373995622,0
-0.52958459308869,48.196283012959,0
-0.52961556579182,48.196189876468,0
-0.5296342429274,48.196095294982,0
-0.52964048241831,48.19599998833,0
-0.52963423684748,48.195904681856,0
-0.52961555381673,48.195810100897,0
-0.52958457558229,48.195716965265,0
-0.52954153797025,48.195625983769,0
-0.52948676857971,48.19553784882,0
-0.52942068428798,48.195453231161,0
-0.52934378807628,48.195372774766,0
-0.52925666520052,48.195297091934,0
-0.52915997873642,48.195226758635,0
-0.52905446453242,48.195162310126,0
-0.52894092560922,48.195104236875,0
-0.52882022604862,48.195052980833,0

-0.52869328441778,48.195008932067,0
-0.52856106677931,48.194972425797,0
-0.52842457934012,48.194943739841,0
-0.52828486079512,48.194923092503,0
-0.52814297442379,48.194910640912,0
-0.52800000000002,48.194906479826,0
-0.52785702557623,48.194910640912,0
-0.52771513920493,48.194923092503,0
-0.52757542065993,48.194943739841,0
-0.52743893322074,48.194972425797,0
-0.52730671558226,48.195008932067,0
-0.52717977395143,48.195052980833,0
-0.52705907439082,48.195104236875,0
-0.52694553546763,48.195162310126,0
-0.5268400212636,48.195226758635,0
-0.52674333479953,48.195297091934,0
-0.52665621192377,48.195372774766,0
-0.52657931571206,48.195453231161,0
-0.52651323142031,48.19553784882,0
-0.5264584620298,48.195625983769,0
-0.52641542441774,48.195716965265,0
-0.52638444618332,48.195810100897,0
-0.52636576315254,48.195904681856,0
-0.52635951758174,48.19599998833,0
-0.52800000000002,48.196,0
</coordinates>
</LinearRing>
</outerBoundaryIs>
</Polygon>
</Placemark>
<Placemark>
<name>380</name>
<styleUrl>#stylehap1</styleUrl>
<Polygon>
<outerBoundaryIs>
<LinearRing>
<coordinates>
-0.52600000000007,48.328,0
-0.52435527585738,48.327999988276,0
-0.52436153146731,48.328095294928,0
-0.52438025688183,48.328189876415,0
-0.52441130965682,48.328283012909,0
-0.52445445352539,48.328373995574,0
-0.52450936019399,48.328462131965,0
-0.52457561183917,48.328546751294,0
-0.52465270428614,48.328627209537,0
-0.52474005084436,48.328702894339,0
-0.5248369867719,48.328773229668,0
-0.52494277433397,48.328837680208,0
-0.52505660841722,48.328895755429,0
-0.52517762265742,48.328947013319,0
-0.52530489603357,48.328991063755,0
-0.52543745987825,48.329027571466,0
-0.52557430525109,48.329056258592,0
-0.5257143906188,48.329076906791,0
-0.5258566497837,48.32908935891,0
-0.52600000000008,48.329093520174,0

-0.52614335021643,48.32908935891,0
-0.52628560938134,48.329076906791,0
-0.52642569474905,48.329056258592,0
-0.52656254012188,48.329027571466,0
-0.52669510396656,48.328991063755,0
-0.52682237734271,48.328947013319,0
-0.52694339158291,48.328895755429,0
-0.52705722566617,48.328837680208,0
-0.52716301322823,48.328773229668,0
-0.52725994915578,48.328702894339,0
-0.52734729571399,48.328627209537,0
-0.52742438816096,48.328546751294,0
-0.52749063980615,48.328462131965,0
-0.52754554647474,48.328373995574,0
-0.52758869034331,48.328283012909,0
-0.5276197431183,48.328189876415,0
-0.52763846853282,48.328095294928,0
-0.52764472414275,48.327999988276,0
-0.52763846240885,48.327904681802,0
-0.52761973105644,48.327810100844,0
-0.52758867271003,48.327716965215,0
-0.52754552380586,48.327625983721,0
-0.52749061279044,48.327537848775,0
-0.52742435761924,48.327453231121,0
-0.52734726257432,48.327372774729,0
-0.52725991442504,48.327297091902,0
-0.5271629779617,48.327226758608,0
-0.5270571909354,48.327162310104,0
-0.52694335844321,48.327104236857,0
-0.52682234680101,48.327052980819,0
-0.52669507695085,48.327008932058,0
-0.526562517453,48.326972425791,0
-0.5264256771158,48.326943739838,0
-0.52628559731948,48.326923092502,0
-0.52614334409246,48.326910640912,0
-0.52600000000008,48.326906479826,0
-0.52585665590767,48.326910640912,0
-0.52571440268066,48.326923092502,0
-0.52557432288434,48.326943739838,0
-0.52543748254713,48.326972425791,0
-0.52530492304928,48.327008932058,0
-0.52517765319912,48.327052980819,0
-0.52505664155692,48.327104236857,0
-0.52494280906473,48.327162310104,0
-0.52483702203843,48.327226758608,0
-0.5247400855751,48.327297091902,0
-0.52465273742582,48.327372774729,0
-0.5245756423809,48.327453231121,0
-0.52450938720969,48.327537848775,0
-0.52445447619427,48.327625983721,0
-0.5244113272901,48.327716965215,0
-0.52438026894369,48.327810100844,0
-0.52436153759128,48.327904681802,0
-0.52435527585738,48.327999988276,0
-0.52600000000007,48.328,0
</coordinates>
</LinearRing>

</outerBoundaryIs>
</Polygon>
</Placemark>
<Placemark>
<name>381</name>
<styleUrl>#stylehap1</styleUrl>
<Polygon>
<outerBoundaryIs>
<LinearRing>
<coordinates>
-0.52600000000007,48.22,0
-0.52435874863484,48.219999988321,0
-0.52436499104783,48.220095294972,0
-0.52438367693551,48.220189876458,0
-0.52441466415421,48.22028301295,0
-0.52445771693551,48.220373995613,0
-0.52451250767881,48.220462132001,0
-0.52457861944265,48.220546751327,0
-0.52465554911651,48.220627209567,0
-0.52474271124853,48.220702894365,0
-0.52483944250029,48.22077322969,0
-0.52494500669459,48.220837680227,0
-0.52505860041816,48.220895755443,0
-0.52517935913611,48.22094701333,0
-0.52530636377231,48.220991063762,0
-0.52543864770487,48.221027571471,0
-0.52557520412396,48.221056258595,0
-0.52571499369581,48.221076906793,0
-0.52585695247424,48.22108935891,0
-0.52600000000008,48.221093520174,0
-0.52614304752589,48.22108935891,0
-0.52628500630433,48.221076906793,0
-0.52642479587618,48.221056258595,0
-0.52656135229527,48.221027571471,0
-0.52669363622782,48.220991063762,0
-0.52682064086402,48.22094701333,0
-0.52694139958197,48.220895755443,0
-0.52705499330554,48.220837680227,0
-0.52716055749985,48.22077322969,0
-0.5272572887516,48.220702894365,0
-0.52734445088362,48.220627209567,0
-0.52742138055749,48.220546751327,0
-0.52748749232132,48.220462132001,0
-0.52754228306463,48.220373995613,0
-0.52758533584592,48.22028301295,0
-0.52761632306462,48.220189876458,0
-0.5276350089523,48.220095294972,0
-0.5276412513653,48.219999988321,0
-0.52763500286439,48.219904681846,0
-0.5276163110738,48.219810100888,0
-0.52758531831654,48.219716965256,0
-0.52754226052928,48.219625983761,0
-0.52748746546474,48.219537848812,0
-0.52742135019571,48.219453231154,0
-0.52734441793916,48.219372774759,0
-0.52725725422546,48.219297091928,0
-0.52716052244108,48.21922675863,0

-0.52705495877938,48.219162310122,0
-0.52694136663751,48.219104236872,0
-0.52682061050224,48.21905298083,0
-0.52669360937124,48.219008932066,0
-0.52656132975992,48.218972425796,0
-0.52642477834679,48.218943739841,0
-0.52628499431353,48.218923092503,0
-0.52614304143801,48.218910640912,0
-0.52600000000008,48.218906479827,0
-0.52585695856213,48.218910640912,0
-0.5257150056866,48.218923092503,0
-0.52557522165334,48.218943739841,0
-0.52543867024022,48.218972425796,0
-0.52530639062889,48.219008932066,0
-0.52517938949792,48.21905298083,0
-0.52505863336262,48.219104236872,0
-0.52494504122076,48.219162310122,0
-0.52483947755906,48.21922675863,0
-0.52474274577467,48.219297091928,0
-0.52465558206097,48.219372774759,0
-0.52457864980442,48.219453231154,0
-0.52451253453539,48.219537848812,0
-0.52445773947086,48.219625983761,0
-0.52441468168359,48.219716965256,0
-0.52438368892633,48.219810100888,0
-0.52436499713575,48.219904681846,0
-0.52435874863484,48.219999988321,0
-0.52600000000007,48.22,0
</coordinates>
</LinearRing>
</outerBoundaryIs>
</Polygon>
</Placemark>
<Placemark>
<name>383</name>
<styleUrl>#stylehap1</styleUrl>
<Polygon>
<outerBoundaryIs>
<LinearRing>
<coordinates>
-0.52499999999998,48.214,0
-0.52335894096605,48.213999988323,0
-0.52336518264818,48.214095294975,0
-0.52338386634675,48.214189876461,0
-0.52341484993475,48.214283012952,0
-0.52345789767143,48.214373995615,0
-0.52351268199447,48.214462132003,0
-0.52357878601131,48.214546751329,0
-0.52365570667039,48.214627209569,0
-0.52374285858843,48.214702894366,0
-0.52383957850471,48.214773229691,0
-0.52394513032834,48.214837680228,0
-0.52405871074017,48.214895755444,0
-0.52417945530666,48.214947013331,0
-0.52430644505934,48.214991063763,0
-0.52443871348964,48.215027571472,0
-0.52457525390576,48.215056258595,0

-0.52471502709567,48.215076906793,0
-0.52485696923794,48.21508935891,0
-0.52499999999997,48.215093520174,0
-0.52514303076202,48.21508935891,0
-0.52528497290429,48.215076906793,0
-0.5254247460942,48.215056258595,0
-0.52556128651029,48.215027571472,0
-0.5256935549406,48.214991063763,0
-0.5258205446933,48.214947013331,0
-0.52594128925979,48.214895755444,0
-0.52605486967162,48.214837680228,0
-0.52616042149525,48.214773229691,0
-0.52625714141153,48.214702894366,0
-0.52634429332957,48.214627209569,0
-0.52642121398865,48.214546751329,0
-0.52648731800549,48.214462132003,0
-0.52654210232853,48.214373995615,0
-0.52658515006521,48.214283012952,0
-0.52661613365321,48.214189876461,0
-0.52663481735178,48.214095294975,0
-0.52664105903389,48.213999988323,0
-0.52663481126588,48.213904681849,0
-0.52661612166634,48.21381010089,0
-0.52658513254155,48.213716965258,0
-0.52654207980058,48.213625983763,0
-0.52648729115772,48.213537848814,0
-0.52642118363679,48.213453231156,0
-0.52634426039589,48.213372774761,0
-0.52625710689671,48.213297091929,0
-0.52616038644798,48.213226758632,0
-0.52605483515678,48.213162310123,0
-0.52594125632612,48.213104236873,0
-0.52582051434147,48.213052980831,0
-0.52569352809285,48.213008932066,0
-0.52556126398235,48.212972425796,0
-0.52542472857057,48.212943739841,0
-0.52528496091742,48.212923092503,0
-0.52514302467612,48.212910640912,0
-0.52499999999997,48.212906479827,0
-0.52485697532382,48.212910640912,0
-0.52471503908254,48.212923092503,0
-0.52457527142939,48.212943739841,0
-0.52443873601761,48.212972425796,0
-0.52430647190711,48.213008932066,0
-0.52417948565849,48.213052980831,0
-0.52405874367384,48.213104236873,0
-0.52394516484318,48.213162310123,0
-0.52383961355198,48.213226758632,0
-0.52374289310325,48.213297091929,0
-0.52365573960407,48.213372774761,0
-0.52357881636314,48.213453231156,0
-0.52351270884224,48.213537848814,0
-0.52345792019938,48.213625983763,0
-0.52341486745838,48.213716965258,0
-0.52338387833362,48.21381010089,0
-0.52336518873408,48.213904681849,0
-0.52335894096605,48.213999988323,0

-0.52499999999998,48.214,0
</coordinates>
</LinearRing>
</outerBoundaryIs>
</Polygon>
</Placemark>
<Placemark>
<name>386</name>
<styleUrl>#stylehap1</styleUrl>
<Polygon>
<outerBoundaryIs>
<LinearRing>
<coordinates>
-0.52300000000002,48.17,0
-0.52136034947039,48.169999988341,0
-0.52136658580003,48.170095294993,0
-0.52138525346713,48.170189876478,0
-0.52141621046651,48.170283012969,0
-0.5214592212596,48.170373995631,0
-0.52151395856516,48.170462132018,0
-0.52158000584831,48.170546751343,0
-0.52165686048915,48.170627209581,0
-0.5217439376068,48.170702894377,0
-0.52184057450977,48.170773229701,0
-0.52194603573893,48.170837680235,0
-0.52205951866462,48.17089575545,0
-0.52218015959528,48.170947013335,0
-0.52230704035127,48.170991063766,0
-0.52243919525358,48.171027571474,0
-0.52257561847458,48.171056258596,0
-0.52271527169429,48.171076906793,0
-0.52285709200453,48.171089358911,0
-0.52300000000002,48.171093520174,0
-0.52314290799551,48.171089358911,0
-0.52328472830575,48.171076906793,0
-0.52342438152547,48.171056258596,0
-0.52356080474646,48.171027571474,0
-0.52369295964878,48.170991063766,0
-0.52381984040476,48.170947013335,0
-0.52394048133543,48.17089575545,0
-0.52405396426112,48.170837680235,0
-0.52415942549028,48.170773229701,0
-0.52425606239325,48.170702894377,0
-0.5243431395109,48.170627209581,0
-0.52441999415174,48.170546751343,0
-0.52448604143489,48.170462132018,0
-0.52454077874045,48.170373995631,0
-0.52458378953354,48.170283012969,0
-0.52461474653292,48.170189876478,0
-0.52463341420002,48.170095294993,0
-0.52463965052965,48.169999988341,0
-0.52463340812874,48.169904681867,0
-0.52461473457482,48.169810100907,0
-0.52458377205199,48.169716965275,0
-0.5245407562666,48.169625983779,0
-0.52448601465159,48.169537848829,0
-0.52441996387281,48.169453231169,0

-0.52434310665633,48.169372774773,0
-0.5242560279613,48.16929709194,0
-0.52415939052716,48.169226758641,0
-0.52405392982917,48.169162310131,0
-0.52394044848083,48.169104236879,0
-0.52381981012581,48.169052980836,0
-0.52369293286548,48.169008932069,0
-0.52356078227261,48.168972425798,0
-0.52342436404392,48.168943739842,0
-0.52328471634766,48.168923092504,0
-0.52314290192424,48.168910640912,0
-0.52300000000002,48.168906479827,0
-0.52285709807581,48.168910640912,0
-0.52271528365239,48.168923092504,0
-0.52257563595613,48.168943739842,0
-0.52243921772743,48.168972425798,0
-0.52230706713457,48.169008932069,0
-0.52218018987424,48.169052980836,0
-0.52205955151922,48.169104236879,0
-0.52194607017088,48.169162310131,0
-0.52184060947289,48.169226758641,0
-0.52174397203874,48.16929709194,0
-0.52165689334372,48.169372774773,0
-0.52158003612724,48.169453231169,0
-0.52151398534846,48.169537848829,0
-0.52145924373345,48.169625983779,0
-0.52141622794806,48.169716965275,0
-0.52138526542523,48.169810100907,0
-0.5213665918713,48.169904681867,0
-0.52136034947039,48.169999988341,0
-0.52300000000002,48.17,0
</coordinates>
</LinearRing>
</outerBoundaryIs>
</Polygon>
</Placemark>
<Placemark>
<name>388</name>
<styleUrl>#stylehap1</styleUrl>
<Polygon>
<outerBoundaryIs>
<LinearRing>
<coordinates>
-0.52300000000002,48.36,0
-0.52135424293792,48.359999988263,0
-0.52136050247304,48.360095294915,0
-0.52137923964413,48.360189876402,0
-0.52141031191776,48.360283012896,0
-0.52145348287869,48.360373995562,0
-0.5215084240273,48.360462131954,0
-0.52157471727789,48.360546751284,0
-0.52165185813895,48.360627209528,0
-0.52173925955166,48.360702894331,0
-0.5218362563566,48.360773229661,0
-0.52194211035564,48.360837680203,0
-0.52205601592985,48.360895755424,0
-0.52217710617089,48.360947013315,0

-0.52230445947921,48.360991063752,0
-0.52243710657908,48.361027571465,0
-0.52257403789635,48.361056258591,0
-0.5227142112436,48.361076906791,0
-0.52285655975344,48.36108935891,0
-0.52300000000002,48.361093520174,0
-0.52314344024661,48.36108935891,0
-0.52328578875645,48.361076906791,0
-0.52342596210369,48.361056258591,0
-0.52356289342097,48.361027571465,0
-0.52369554052084,48.360991063752,0
-0.52382289382919,48.360947013315,0
-0.52394398407019,48.360895755424,0
-0.5240578896444,48.360837680203,0
-0.52416374364345,48.360773229661,0
-0.52426074044839,48.360702894331,0
-0.52434814186109,48.360627209528,0
-0.52442528272219,48.360546751284,0
-0.52449157597274,48.360462131954,0
-0.52454651712135,48.360373995562,0
-0.52458968808229,48.360283012896,0
-0.52462076035591,48.360189876402,0
-0.52463949752701,48.360095294915,0
-0.52464575706213,48.359999988263,0
-0.5246394913923,48.359904681789,0
-0.52462074827288,48.359810100832,0
-0.5245896704181,48.359716965202,0
-0.5245464944127,48.35962598371,0
-0.52449154890963,48.359537848765,0
-0.5244252521269,48.359453231111,0
-0.52434810866328,48.35937277472,0
-0.52426070565673,48.359297091894,0
-0.52416370831507,48.359226758602,0
-0.52405785485272,48.359162310098,0
-0.52394395087235,48.359104236853,0
-0.5238228632339,48.359052980816,0
-0.52369551345773,48.359008932055,0
-0.52356287071232,48.358972425789,0
-0.5234259444395,48.358943739837,0
-0.52328577667344,48.358923092501,0
-0.5231434341119,48.358910640912,0
-0.52300000000002,48.358906479827,0
-0.52285656588814,48.358910640912,0
-0.5227142233266,48.358923092501,0
-0.52257405556054,48.358943739837,0
-0.52243712928773,48.358972425789,0
-0.52230448654232,48.359008932055,0
-0.52217713676614,48.359052980816,0
-0.52205604912769,48.359104236853,0
-0.52194214514732,48.359162310098,0
-0.52183629168498,48.359226758602,0
-0.52173929434331,48.359297091894,0
-0.52165189133677,48.35937277472,0
-0.52157474787314,48.359453231111,0
-0.52150845109041,48.359537848765,0
-0.52145350558735,48.35962598371,0
-0.52141032958195,48.359716965202,0

-0.52137925172716,48.359810100832,0
-0.52136050860774,48.359904681789,0
-0.52135424293792,48.359999988263,0
-0.52300000000002,48.36,0
</coordinates>
</LinearRing>
</outerBoundaryIs>
</Polygon>
</Placemark>
<Placemark>
<name>389</name>
<styleUrl>#stylehap1</styleUrl>
<Polygon>
<outerBoundaryIs>
<LinearRing>
<coordinates>
-0.52199999999993,48.151,0
-0.52036095664224,48.150999988349,0
-0.52036719066455,48.151095295,0
-0.52038585142086,48.151189876486,0
-0.52041679695852,48.151283012976,0
-0.52045979182614,48.151373995638,0
-0.5205145088636,48.151462132025,0
-0.52058053169022,48.151546751348,0
-0.52065735787222,48.151627209586,0
-0.52074440274523,48.151702894381,0
-0.52084100386319,48.151773229704,0
-0.52094642603929,48.151837680238,0
-0.52105986694107,48.151895755452,0
-0.52118046319685,48.151947013337,0
-0.52130729696701,48.151991063768,0
-0.52143940293018,48.152027571475,0
-0.52157577563122,48.152056258597,0
-0.52171537713471,48.152076906794,0
-0.5218571449261,48.152089358911,0
-0.52199999999994,48.152093520174,0
-0.52214285507377,48.152089358911,0
-0.52228462286516,48.152076906794,0
-0.52242422436865,48.152056258597,0
-0.52256059706969,48.152027571475,0
-0.52269270303286,48.151991063768,0
-0.522819536803,48.151947013337,0
-0.52294013305877,48.151895755452,0
-0.52305357396059,48.151837680238,0
-0.52315899613669,48.151773229704,0
-0.52325559725462,48.151702894381,0
-0.52334264212766,48.151627209586,0
-0.52341946830963,48.151546751348,0
-0.52348549113627,48.151462132025,0
-0.52354020817374,48.151373995638,0
-0.52358320304133,48.151283012976,0
-0.52361414857901,48.151189876486,0
-0.52363280933532,48.151095295,0
-0.52363904335763,48.150999988349,0
-0.52363280327033,48.150904681875,0
-0.5236141366333,48.150810100915,0
-0.5235831855779,48.150716965282,0

-0.52354018572317,48.150625983785,0
-0.52348546438074,48.150537848835,0
-0.5234194380621,48.150453231175,0
-0.52334260930713,48.150372774778,0
-0.5232555628584,48.150297091945,0
-0.52315896120982,48.150226758645,0
-0.52305353956434,48.150162310134,0
-0.52294010023827,48.150104236881,0
-0.52281950655546,48.150052980838,0
-0.52269267627732,48.150008932071,0
-0.52256057461915,48.149972425799,0
-0.52242420690522,48.149943739842,0
-0.52228461091948,48.149923092504,0
-0.52214284900878,48.149910640912,0
-0.52199999999994,48.149906479826,0
-0.52185715099109,48.149910640912,0
-0.52171538908039,48.149923092504,0
-0.52157579309463,48.149943739842,0
-0.52143942538072,48.149972425799,0
-0.52130732372253,48.150008932071,0
-0.52118049344441,48.150052980838,0
-0.5210598997616,48.150104236881,0
-0.52094646043554,48.150162310134,0
-0.52084103879006,48.150226758645,0
-0.52074443714147,48.150297091945,0
-0.52065739069272,48.150372774778,0
-0.52058056193778,48.150453231175,0
-0.52051453561914,48.150537848835,0
-0.52045981427671,48.150625983785,0
-0.52041681442195,48.150716965282,0
-0.52038586336654,48.150810100915,0
-0.52036719672954,48.150904681875,0
-0.52036095664224,48.150999988349,0
-0.52199999999993,48.151,0
</coordinates>
</LinearRing>
</outerBoundaryIs>
</Polygon>
</Placemark>
<Placemark>
<name>391</name>
<styleUrl>#stylehap1</styleUrl>
<Polygon>
<outerBoundaryIs>
<LinearRing>
<coordinates>
-0.52199999999993,48.324,0
-0.52035540484497,48.323999988278,0
-0.52036165996474,48.32409529493,0
-0.52038038391111,48.324189876417,0
-0.5204114342512,48.32428301291,0
-0.52045457473654,48.324373995575,0
-0.52050947709938,48.324462131966,0
-0.52057572354902,48.324546751295,0
-0.52065280995018,48.324627209538,0
-0.52074014965835,48.32470289434,0
-0.52083707798374,48.324773229669,0

-0.52094285724936,48.324837680209,0
-0.52105668240502,48.324895755429,0
-0.52117768715448,48.324947013319,0
-0.52130495054895,48.324991063755,0
-0.52143750399701,48.325027571466,0
-0.52157433863735,48.325056258592,0
-0.52171441301846,48.325076906791,0
-0.52185666102626,48.32508935891,0
-0.52199999999994,48.325093520174,0
-0.52214333897361,48.32508935891,0
-0.52228558698141,48.325076906791,0
-0.52242566136252,48.325056258592,0
-0.52256249600287,48.325027571466,0
-0.52269504945092,48.324991063755,0
-0.52282231284537,48.324947013319,0
-0.52294331759485,48.324895755429,0
-0.52305714275051,48.324837680209,0
-0.52316292201614,48.324773229669,0
-0.52325985034153,48.32470289434,0
-0.52334719004969,48.324627209538,0
-0.52342427645083,48.324546751295,0
-0.52349052290049,48.324462131966,0
-0.52354542526333,48.324373995575,0
-0.52358856574868,48.32428301291,0
-0.52361961608876,48.324189876417,0
-0.52363834003514,48.32409529493,0
-0.52364459515491,48.323999988278,0
-0.52363833391252,48.323904681804,0
-0.52361960402952,48.323810100846,0
-0.52358854811927,48.323716965216,0
-0.52354540259941,48.323625983723,0
-0.52349049589069,48.323537848777,0
-0.52342424591583,48.323453231122,0
-0.52334715691727,48.32337277473,0
-0.52325981561839,48.323297091903,0
-0.52316288675735,48.323226758609,0
-0.52305710802738,48.323162310104,0
-0.5229432844624,48.323104236858,0
-0.52282228231037,48.32305298082,0
-0.52269502244112,48.323008932058,0
-0.52256247333895,48.322972425791,0
-0.52242564373311,48.322943739838,0
-0.52228557492217,48.322923092502,0
-0.52214333285097,48.322910640912,0
-0.52199999999994,48.322906479826,0
-0.5218566671489,48.322910640912,0
-0.52171442507767,48.322923092502,0
-0.52157435626676,48.322943739838,0
-0.52143752666093,48.322972425791,0
-0.52130497755876,48.323008932058,0
-0.52117771768951,48.32305298082,0
-0.52105671553747,48.323104236858,0
-0.52094289197249,48.323162310104,0
-0.52083711324252,48.323226758609,0
-0.52074018438148,48.323297091903,0
-0.52065284308261,48.32337277473,0
-0.52057575408405,48.323453231122,0

-0.52050950410919,48.323537848777,0
-0.52045459740046,48.323625983723,0
-0.52041145188058,48.323716965216,0
-0.52038039597032,48.323810100846,0
-0.52036166608735,48.323904681804,0
-0.52035540484497,48.323999988278,0
-0.52199999999993,48.324,0
</coordinates>
</LinearRing>
</outerBoundaryIs>
</Polygon>
</Placemark>
<Placemark>
<name>392</name>
<styleUrl>#stylehap1</styleUrl>
<Polygon>
<outerBoundaryIs>
<LinearRing>
<coordinates>
-0.52099999999996,48.158,0
-0.51936073302064,48.157999988346,0
-0.51936696789274,48.158095294998,0
-0.51938563119429,48.158189876483,0
-0.5194165809533,48.158283012974,0
-0.51945958168629,48.158373995636,0
-0.51951430618849,48.158462132022,0
-0.51958033802247,48.158546751346,0
-0.51965717468584,48.158627209584,0
-0.51974423143459,48.15870289438,0
-0.51984084573218,48.158773229703,0
-0.51994628229154,48.158837680237,0
-0.52005973867074,48.158895755451,0
-0.52018035138029,48.158947013336,0
-0.52030720245534,48.158991063767,0
-0.52043932644282,48.159027571474,0
-0.52057571775036,48.159056258596,0
-0.52071533830097,48.159076906794,0
-0.52085712543507,48.159089358911,0
-0.52099999999995,48.159093520174,0
-0.52114287456486,48.159089358911,0
-0.52128466169896,48.159076906794,0
-0.52142428224955,48.159056258596,0
-0.52156067355711,48.159027571474,0
-0.52169279754459,48.158991063767,0
-0.52181964861964,48.158947013336,0
-0.52194026132919,48.158895755451,0
-0.52205371770839,48.158837680237,0
-0.52215915426775,48.158773229703,0
-0.52225576856534,48.15870289438,0
-0.52234282531406,48.158627209584,0
-0.52241966197746,48.158546751346,0
-0.52248569381144,48.158462132022,0
-0.52254041831364,48.158373995636,0
-0.52258341904661,48.158283012974,0
-0.52261436880562,48.158189876483,0
-0.52263303210719,48.158095294998,0
-0.52263926697929,48.157999988346,0

-0.52263302603989,48.157904681872,0
-0.52261435685536,48.157810100912,0
-0.52258340157651,48.15771696528,0
-0.52254039585449,48.157625983783,0
-0.52248566704567,48.157537848833,0
-0.52241963171835,48.157453231173,0
-0.52234279248099,48.157372774776,0
-0.52225573415594,48.157297091943,0
-0.52215911932752,48.157226758643,0
-0.52205368329899,48.157162310133,0
-0.52194022849612,48.15710423688,0
-0.52181961836053,48.157052980837,0
-0.52169277077882,48.15700893207,0
-0.52156065109797,48.156972425799,0
-0.52142426477945,48.156943739842,0
-0.5212846497487,48.156923092504,0
-0.52114286849756,48.156910640912,0
-0.52099999999995,48.156906479827,0
-0.52085713150237,48.156910640912,0
-0.52071535025123,48.156923092504,0
-0.52057573522046,48.156943739842,0
-0.52043934890194,48.156972425799,0
-0.52030722922109,48.15700893207,0
-0.5201803816394,48.157052980837,0
-0.52005977150379,48.15710423688,0
-0.51994631670092,48.157162310133,0
-0.51984088067238,48.157226758643,0
-0.51974426584396,48.157297091943,0
-0.51965720751891,48.157372774776,0
-0.51958036828158,48.157453231173,0
-0.51951433295423,48.157537848833,0
-0.51945960414541,48.157625983783,0
-0.51941659842342,48.15771696528,0
-0.51938564314455,48.157810100912,0
-0.51936697396004,48.157904681872,0
-0.51936073302064,48.157999988346,0
-0.52099999999996,48.158,0
</coordinates>
</LinearRing>
</outerBoundaryIs>
</Polygon>
</Placemark>
<Placemark>
<name>394</name>
<styleUrl>#stylehap1</styleUrl>
<Polygon>
<outerBoundaryIs>
<LinearRing>
<coordinates>
-0.52099999999996,48.233,0
-0.51935833170045,48.232999988315,0
-0.51936457569785,48.233095294967,0
-0.51938326633102,48.233189876453,0
-0.51941426142026,48.233283012945,0
-0.51945732513733,48.233373995609,0
-0.51951212979836,48.233462131997,0
-0.51957825835605,48.233546751323,0

-0.51965520757211,48.233627209564,0
-0.51974239184596,48.233702894361,0
-0.51983914767065,48.233773229688,0
-0.51994473868202,48.233837680224,0
-0.52005836126266,48.233895755441,0
-0.52017915065808,48.233947013329,0
-0.52030618755861,48.233991063762,0
-0.52043850509684,48.234027571471,0
-0.5205750962071,48.234056258594,0
-0.52071492129155,48.234076906793,0
-0.52085691613376,48.23408935891,0
-0.52099999999995,48.234093520174,0
-0.52114308386617,48.23408935891,0
-0.52128507870836,48.234076906793,0
-0.52142490379283,48.234056258594,0
-0.52156149490309,48.234027571471,0
-0.52169381244129,48.233991063762,0
-0.52182084934185,48.233947013329,0
-0.52194163873727,48.233895755441,0
-0.52205526131789,48.233837680224,0
-0.52216085232928,48.233773229688,0
-0.52225760815397,48.233702894361,0
-0.52234479242782,48.233627209564,0
-0.52242174164385,48.233546751323,0
-0.52248787020155,48.233462131997,0
-0.5225426748626,48.233373995609,0
-0.52258573857964,48.233283012945,0
-0.52261673366889,48.233189876453,0
-0.52263542430206,48.233095294967,0
-0.52264166829945,48.232999988315,0
-0.52263541820984,48.232904681841,0
-0.52261672166954,48.232810100882,0
-0.52258572103781,48.232716965251,0
-0.52254265231123,48.232625983756,0
-0.52248784332589,48.232537848807,0
-0.5224217112605,48.23245323115,0
-0.52234475945992,48.232372774755,0
-0.52225757360328,48.232297091925,0
-0.52216081724557,48.232226758628,0
-0.52205522676719,48.23216231012,0
-0.52194160576937,48.23210423687,0
-0.52182081895847,48.232052980829,0
-0.52169378556563,48.232008932065,0
-0.52156147235172,48.231972425795,0
-0.52142488625098,48.23194373984,0
-0.52128506670904,48.231923092503,0
-0.52114307777393,48.231910640912,0
-0.52099999999995,48.231906479826,0
-0.52085692222597,48.231910640912,0
-0.52071493329089,48.231923092503,0
-0.52057511374895,48.23194373984,0
-0.52043852764819,48.231972425795,0
-0.52030621443428,48.232008932065,0
-0.52017918104144,48.232052980829,0
-0.52005839423053,48.23210423687,0
-0.51994477323272,48.23216231012,0
-0.51983918275433,48.232226758628,0

-0.51974242639665,48.232297091925,0
-0.51965524054001,48.232372774755,0
-0.51957828873943,48.23245323115,0
-0.51951215667402,48.232537848807,0
-0.51945734768868,48.232625983756,0
-0.51941427896212,48.232716965251,0
-0.51938327833036,48.232810100882,0
-0.51936458179006,48.232904681841,0
-0.51935833170045,48.232999988315,0
-0.52099999999996,48.233,0
</coordinates>
</LinearRing>
</outerBoundaryIs>
</Polygon>
</Placemark>
<Placemark>
<name>397</name>
<styleUrl>#stylehap1</styleUrl>
<Polygon>
<outerBoundaryIs>
<LinearRing>
<coordinates>
-0.52099999999996,48.314,0
-0.51935572719071,48.313999988282,0
-0.51936198108554,48.314095294934,0
-0.519380701363,48.314189876421,0
-0.5194117456181,48.314283012914,0
-0.51945487764867,48.314373995579,0
-0.51950976925123,48.31446213197,0
-0.519576002717,48.314546751298,0
-0.51965307400942,48.314627209541,0
-0.51974039659902,48.314702894342,0
-0.51983730592624,48.314773229671,0
-0.51994306445872,48.314837680211,0
-0.52005686730404,48.31489575543,0
-0.52017784833574,48.31494701332,0
-0.52030508678556,48.314991063756,0
-0.52043761425194,48.315027571467,0
-0.52057442207141,48.315056258592,0
-0.52071446899654,48.315076906792,0
-0.52085668912225,48.31508935891,0
-0.52099999999995,48.315093520174,0
-0.52114331087768,48.31508935891,0
-0.52128553100339,48.315076906792,0
-0.52142557792849,48.315056258592,0
-0.52156238574796,48.315027571467,0
-0.52169491321437,48.314991063756,0
-0.52182215166419,48.31494701332,0
-0.52194313269589,48.31489575543,0
-0.52205693554121,48.314837680211,0
-0.52216269407366,48.314773229671,0
-0.52225960340088,48.314702894342,0
-0.52234692599049,48.314627209541,0
-0.52242399728293,48.314546751298,0
-0.5224902307487,48.31446213197,0
-0.52254512235124,48.314373995579,0
-0.52258825438183,48.314283012914,0

-0.5226192986369,48.314189876421,0
-0.52263801891439,48.314095294934,0
-0.52264427280919,48.313999988282,0
-0.52263801279511,48.313904681808,0
-0.52261928658428,48.31381010085,0
-0.52258823676207,48.31371696522,0
-0.52254509969973,48.313625983726,0
-0.52249020375365,48.31353784878,0
-0.52242396676461,48.313453231125,0
-0.52234689287618,48.313372774733,0
-0.52225956869676,48.313297091905,0
-0.52216265883416,48.313226758611,0
-0.52205690083706,48.313162310106,0
-0.52194309958158,48.313104236859,0
-0.52182212114587,48.313052980821,0
-0.52169488621932,48.313008932059,0
-0.52156236309643,48.312972425792,0
-0.52142556030876,48.312943739838,0
-0.52128551895076,48.312923092502,0
-0.52114330475839,48.312910640912,0
-0.52099999999995,48.312906479827,0
-0.52085669524151,48.312910640912,0
-0.52071448104914,48.312923092502,0
-0.52057443969117,48.312943739838,0
-0.52043763690347,48.312972425792,0
-0.52030511378058,48.313008932059,0
-0.52017787885403,48.313052980821,0
-0.52005690041835,48.313104236859,0
-0.51994309916285,48.313162310106,0
-0.51983734116574,48.313226758611,0
-0.51974043130317,48.313297091905,0
-0.51965310712372,48.313372774733,0
-0.51957603323529,48.313453231125,0
-0.51950979624625,48.31353784878,0
-0.5194549003002,48.313625983726,0
-0.51941176323786,48.31371696522,0
-0.51938071341563,48.31381010085,0
-0.51936198720482,48.313904681808,0
-0.51935572719071,48.313999988282,0
-0.52099999999996,48.314,0
</coordinates>
</LinearRing>
</outerBoundaryIs>
</Polygon>
</Placemark>
<Placemark>
<name>399</name>
<styleUrl>#stylehap1</styleUrl>
<Polygon>
<outerBoundaryIs>
<LinearRing>
<coordinates>
-0.51999999999998,48.206,0
-0.5183591973097,48.205999988326,0
-0.51836543801769,48.206095294978,0
-0.51838411879857,48.206189876464,0
-0.51841509754752,48.206283012955,0

-0.51845813856056,48.206373995618,0
-0.51851291432659,48.206462132006,0
-0.51857900801805,48.206546751331,0
-0.51865591666201,48.206627209571,0
-0.51874305496659,48.206702894368,0
-0.5188397597747,48.206773229693,0
-0.51894529511041,48.206837680229,0
-0.51905885778003,48.206895755445,0
-0.5191795834851,48.206947013331,0
-0.51930655340071,48.206991063764,0
-0.51943880116927,48.207027571472,0
-0.5195753202562,48.207056258595,0
-0.51971507161188,48.207076906793,0
-0.51985699158108,48.20708935891,0
-0.51999999999999,48.207093520174,0
-0.52014300841888,48.20708935891,0
-0.52028492838808,48.207076906793,0
-0.52042467974376,48.207056258595,0
-0.52056119883069,48.207027571472,0
-0.52069344659925,48.206991063764,0
-0.52082041651486,48.206947013331,0
-0.52094114221993,48.206895755445,0
-0.52105470488955,48.206837680229,0
-0.52116024022526,48.206773229693,0
-0.52125694503338,48.206702894368,0
-0.52134408333795,48.206627209571,0
-0.52142099198191,48.206546751331,0
-0.52148708567338,48.206462132006,0
-0.5215418614394,48.206373995618,0
-0.52158490245244,48.206283012955,0
-0.52161588120139,48.206189876464,0
-0.52163456198227,48.206095294978,0
-0.52164080269026,48.205999988326,0
-0.52163455589904,48.205904681852,0
-0.52161586921976,48.205810100893,0
-0.52158488493646,48.205716965261,0
-0.52154183892128,48.205625983766,0
-0.52148705883736,48.205537848817,0
-0.52142096164334,48.205453231158,0
-0.52134405041868,48.205372774763,0
-0.52125691053362,48.205297091931,0
-0.52116020519331,48.205226758633,0
-0.52105467038982,48.205162310124,0
-0.52094110930065,48.205104236874,0
-0.52082038617629,48.205052980832,0
-0.52069341976321,48.205008932067,0
-0.5205611763126,48.204972425797,0
-0.52042466222778,48.204943739841,0
-0.52028491640645,48.204923092503,0
-0.52014300233565,48.204910640912,0
-0.51999999999999,48.204906479826,0
-0.51985699766432,48.204910640912,0
-0.51971508359351,48.204923092503,0
-0.51957533777218,48.204943739841,0
-0.51943882368736,48.204972425797,0
-0.51930658023676,48.205008932067,0
-0.51917961382367,48.205052980832,0

-0.51905889069931,48.205104236874,0
-0.51894532961014,48.205162310124,0
-0.51883979480665,48.205226758633,0
-0.51874308946634,48.205297091931,0
-0.51865594958128,48.205372774763,0
-0.51857903835662,48.205453231158,0
-0.5185129411626,48.205537848817,0
-0.51845816107869,48.205625983766,0
-0.5184151150635,48.205716965261,0
-0.51838413078023,48.205810100893,0
-0.51836544410092,48.205904681852,0
-0.5183591973097,48.205999988326,0
-0.51999999999998,48.206,0
</coordinates>
</LinearRing>
</outerBoundaryIs>
</Polygon>
</Placemark>
<Placemark>
<name>400</name>
<styleUrl>#stylehap1</styleUrl>
<Polygon>
<outerBoundaryIs>
<LinearRing>
<coordinates>
-0.51999999999998,48.206,0
-0.5183591973097,48.205999988326,0
-0.51836543801769,48.206095294978,0
-0.51838411879857,48.206189876464,0
-0.51841509754752,48.206283012955,0
-0.51845813856056,48.206373995618,0
-0.51851291432659,48.206462132006,0
-0.51857900801805,48.206546751331,0
-0.51865591666201,48.206627209571,0
-0.51874305496659,48.206702894368,0
-0.5188397597747,48.206773229693,0
-0.51894529511041,48.206837680229,0
-0.51905885778003,48.206895755445,0
-0.5191795834851,48.206947013331,0
-0.51930655340071,48.206991063764,0
-0.51943880116927,48.207027571472,0
-0.5195753202562,48.207056258595,0
-0.51971507161188,48.207076906793,0
-0.51985699158108,48.20708935891,0
-0.51999999999999,48.207093520174,0
-0.52014300841888,48.20708935891,0
-0.52028492838808,48.207076906793,0
-0.52042467974376,48.207056258595,0
-0.52056119883069,48.207027571472,0
-0.52069344659925,48.206991063764,0
-0.52082041651486,48.206947013331,0
-0.52094114221993,48.206895755445,0
-0.52105470488955,48.206837680229,0
-0.52116024022526,48.206773229693,0
-0.52125694503338,48.206702894368,0
-0.52134408333795,48.206627209571,0
-0.52142099198191,48.206546751331,0

-0.52148708567338,48.206462132006,0
-0.5215418614394,48.206373995618,0
-0.52158490245244,48.206283012955,0
-0.52161588120139,48.206189876464,0
-0.52163456198227,48.206095294978,0
-0.52164080269026,48.205999988326,0
-0.52163455589904,48.205904681852,0
-0.52161586921976,48.205810100893,0
-0.52158488493646,48.205716965261,0
-0.52154183892128,48.205625983766,0
-0.52148705883736,48.205537848817,0
-0.52142096164334,48.205453231158,0
-0.52134405041868,48.205372774763,0
-0.52125691053362,48.205297091931,0
-0.52116020519331,48.205226758633,0
-0.52105467038982,48.205162310124,0
-0.52094110930065,48.205104236874,0
-0.52082038617629,48.205052980832,0
-0.52069341976321,48.205008932067,0
-0.5205611763126,48.204972425797,0
-0.52042466222778,48.204943739841,0
-0.52028491640645,48.204923092503,0
-0.52014300233565,48.204910640912,0
-0.51999999999999,48.204906479826,0
-0.51985699766432,48.204910640912,0
-0.51971508359351,48.204923092503,0
-0.51957533777218,48.204943739841,0
-0.51943882368736,48.204972425797,0
-0.51930658023676,48.205008932067,0
-0.51917961382367,48.205052980832,0
-0.51905889069931,48.205104236874,0
-0.51894532961014,48.205162310124,0
-0.51883979480665,48.205226758633,0
-0.51874308946634,48.205297091931,0
-0.51865594958128,48.205372774763,0
-0.51857903835662,48.205453231158,0
-0.5185129411626,48.205537848817,0
-0.51845816107869,48.205625983766,0
-0.5184151150635,48.205716965261,0
-0.51838413078023,48.205810100893,0
-0.51836544410092,48.205904681852,0
-0.5183591973097,48.205999988326,0
-0.51999999999998,48.206,0
</coordinates>
</LinearRing>
</outerBoundaryIs>
</Polygon>
</Placemark>
<Placemark>
<name>401</name>
<styleUrl>#stylehap1</styleUrl>
<Polygon>
<outerBoundaryIs>
<LinearRing>
<coordinates>
-0.51900000000001,48.163,0
-0.51736057323861,48.162999988344,0

-0.51736680871789,48.163095294995,0
-0.51738547383808,48.163189876481,0
-0.51741642661333,48.163283012972,0
-0.51745943153722,48.163373995634,0
-0.51751416137314,48.163462132021,0
-0.51758019964303,48.163546751345,0
-0.51765704379559,48.163627209583,0
-0.51774410902975,48.163702894378,0
-0.51784073274446,48.163773229702,0
-0.51794617958095,48.163837680236,0
-0.51805964701907,48.163895755451,0
-0.51818027148517,48.163947013336,0
-0.5183071349249,48.163991063767,0
-0.5184392717911,48.164027571474,0
-0.51857567639338,48.164056258596,0
-0.51871531055351,48.164076906793,0
-0.51885711150834,48.164089358911,0
-0.51900000000001,48.164093520174,0
-0.51914288849165,48.164089358911,0
-0.51928468944651,48.164076906793,0
-0.51942432360664,48.164056258596,0
-0.51956072820892,48.164027571474,0
-0.51969286507512,48.163991063767,0
-0.51981972851484,48.163947013336,0
-0.51994035298095,48.163895755451,0
-0.52005382041907,48.163837680236,0
-0.52015926725556,48.163773229702,0
-0.52025589097024,48.163702894378,0
-0.5203429562044,48.163627209583,0
-0.52041980035696,48.163546751345,0
-0.52048583862688,48.163462132021,0
-0.52054056846279,48.163373995634,0
-0.52058357338667,48.163283012972,0
-0.52061452616194,48.163189876481,0
-0.52063319128212,48.163095294995,0
-0.52063942676141,48.162999988344,0
-0.52063318521317,48.16290468187,0
-0.52061451420842,48.16281010091,0
-0.52058355591181,48.162716965278,0
-0.52054054599752,48.162625983781,0
-0.52048581185381,48.162537848831,0
-0.52041977008961,48.162453231171,0
-0.52034292336237,48.162372774775,0
-0.52025585655148,48.162297091942,0
-0.52015923230579,48.162226758642,0
-0.52005378600027,48.162162310132,0
-0.51994032013892,48.16210423688,0
-0.51981969824746,48.162052980836,0
-0.51969283830205,48.16200893207,0
-0.51956070574364,48.161972425799,0
-0.51942430613175,48.161943739842,0
-0.51928467749299,48.161923092504,0
-0.51914288242269,48.161910640912,0
-0.51900000000001,48.161906479826,0
-0.51885711757732,48.161910640912,0
-0.51871532250702,48.161923092504,0
-0.51857569386826,48.161943739842,0

-0.51843929425637,48.161972425799,0
-0.51830716169797,48.16200893207,0
-0.51818030175255,48.162052980836,0
-0.5180596798611,48.16210423688,0
-0.51794621399974,48.162162310132,0
-0.5178407676942,48.162226758642,0
-0.51774414344854,48.162297091942,0
-0.51765707663762,48.162372774775,0
-0.51758022991041,48.162453231171,0
-0.51751418814621,48.162537848831,0
-0.5174594540025,48.162625983781,0
-0.51741644408821,48.162716965278,0
-0.5173854857916,48.16281010091,0
-0.51736681478685,48.16290468187,0
-0.51736057323861,48.162999988344,0
-0.51900000000001,48.163,0
</coordinates>
</LinearRing>
</outerBoundaryIs>
</Polygon>
</Placemark>
<Placemark>
<name>402</name>
<styleUrl>#stylehap1</styleUrl>
<Polygon>
<outerBoundaryIs>
<LinearRing>
<coordinates>
-0.51800000000003,48.312,0
-0.51635579163877,48.311999988283,0
-0.51636204528867,48.312095294935,0
-0.51638076483259,48.312189876421,0
-0.51641180787109,48.312283012915,0
-0.51645493821126,48.31237399558,0
-0.51650982766247,48.31246213197,0
-0.51657605853232,48.312546751299,0
-0.516653126804,48.312627209542,0
-0.51674044597099,48.312702894342,0
-0.51683735149985,48.312773229671,0
-0.51694310588704,48.312837680211,0
-0.51705690427175,48.312895755431,0
-0.51717788056145,48.31294701332,0
-0.517305114024,48.312991063756,0
-0.51743763629578,48.313027571467,0
-0.5175744387528,48.313056258592,0
-0.51771448018853,48.313076906792,0
-0.51785669473967,48.31308935891,0
-0.51800000000002,48.313093520173,0
-0.51814330526041,48.31308935891,0
-0.51828551981152,48.313076906792,0
-0.51842556124724,48.313056258592,0
-0.5185623637043,48.313027571467,0
-0.51869488597607,48.312991063756,0
-0.5188221194386,48.31294701332,0
-0.5189430957283,48.312895755431,0
-0.51905689411303,48.312837680211,0
-0.5191626485002,48.312773229671,0

-0.51925955402905,48.312702894342,0
-0.51934687319605,48.312627209542,0
-0.51942394146775,48.312546751299,0
-0.51949017233758,48.31246213197,0
-0.51954506178879,48.31237399558,0
-0.51958819212896,48.312283012915,0
-0.51961923516745,48.312189876421,0
-0.51963795471138,48.312095294935,0
-0.5196442083613,48.311999988283,0
-0.51963794859278,48.311904681809,0
-0.51961922311615,48.311810100851,0
-0.51958817451115,48.311716965221,0
-0.51954503913975,48.311625983727,0
-0.51949014534551,48.311537848781,0
-0.51942391095277,48.311453231125,0
-0.51934684008538,48.311372774734,0
-0.51925951932872,48.311297091906,0
-0.51916261326457,48.311226758611,0
-0.51905685941267,48.311162310106,0
-0.51894306261763,48.31110423686,0
-0.51882208892362,48.311052980821,0
-0.51869485898397,48.311008932059,0
-0.51856234105526,48.310972425792,0
-0.51842554362941,48.310943739838,0
-0.51828550776022,48.310923092502,0
-0.51814329914178,48.310910640912,0
-0.51800000000002,48.310906479826,0
-0.51785670085827,48.310910640912,0
-0.51771449223983,48.310923092502,0
-0.51757445637063,48.310943739838,0
-0.51743765894481,48.310972425792,0
-0.51730514101607,48.311008932059,0
-0.51717791107643,48.311052980821,0
-0.51705693738245,48.31110423686,0
-0.51694314058738,48.311162310106,0
-0.51683738673551,48.311226758611,0
-0.51674048067135,48.311297091906,0
-0.51665315991469,48.311372774734,0
-0.51657608904728,48.311453231125,0
-0.51650985465454,48.311537848781,0
-0.51645496086029,48.311625983727,0
-0.51641182548892,48.311716965221,0
-0.51638077688389,48.311810100851,0
-0.51636205140726,48.311904681809,0
-0.51635579163877,48.311999988283,0
-0.51800000000003,48.312,0
</coordinates>
</LinearRing>
</outerBoundaryIs>
</Polygon>
</Placemark>
<Placemark>
<name>404</name>
<styleUrl>#stylehap1</styleUrl>
<Polygon>
<outerBoundaryIs>
<LinearRing>

<coordinates>
-0.51800000000003,48.151,0
-0.51636095664233,48.150999988349,0
-0.51636719066464,48.151095295,0
-0.51638585142095,48.151189876486,0
-0.51641679695863,48.151283012976,0
-0.51645979182625,48.151373995638,0
-0.51651450886369,48.151462132025,0
-0.51658053169033,48.151546751348,0
-0.5166573578723,48.151627209586,0
-0.51674440274534,48.151702894381,0
-0.51684100386328,48.151773229704,0
-0.51694642603937,48.151837680238,0
-0.51705986694119,48.151895755452,0
-0.51718046319696,48.151947013337,0
-0.5173072969671,48.151991063768,0
-0.51743940293027,48.152027571475,0
-0.51757577563131,48.152056258597,0
-0.5177153771348,48.152076906794,0
-0.51785714492619,48.152089358911,0
-0.51800000000002,48.152093520174,0
-0.51814285507386,48.152089358911,0
-0.51828462286525,48.152076906794,0
-0.51842422436877,48.152056258597,0
-0.51856059706978,48.152027571475,0
-0.51869270303295,48.151991063768,0
-0.51881953680311,48.151947013337,0
-0.51894013305889,48.151895755452,0
-0.51905357396067,48.151837680238,0
-0.51915899613677,48.151773229704,0
-0.51925559725473,48.151702894381,0
-0.51934264212774,48.151627209586,0
-0.51941946830974,48.151546751348,0
-0.51948549113636,48.151462132025,0
-0.51954020817382,48.151373995638,0
-0.51958320304144,48.151283012976,0
-0.5196141485791,48.151189876486,0
-0.51963280933541,48.151095295,0
-0.51963904335774,48.150999988349,0
-0.51963280327042,48.150904681875,0
-0.51961413663342,48.150810100915,0
-0.51958318557801,48.150716965282,0
-0.51954018572326,48.150625983785,0
-0.51948546438085,48.150537848835,0
-0.51941943806218,48.150453231175,0
-0.51934260930724,48.150372774778,0
-0.51925556285849,48.150297091945,0
-0.51915896120991,48.150226758645,0
-0.51905353956442,48.150162310134,0
-0.51894010023836,48.150104236881,0
-0.51881950655555,48.150052980838,0
-0.51869267627744,48.150008932071,0
-0.51856057461924,48.149972425799,0
-0.51842420690533,48.149943739842,0
-0.51828461091957,48.149923092504,0
-0.51814284900887,48.149910640912,0
-0.51800000000002,48.149906479826,0

-0.51785715099118,48.149910640912,0
-0.5177153890805,48.149923092504,0
-0.51757579309474,48.149943739842,0
-0.51743942538084,48.149972425799,0
-0.51730732372264,48.150008932071,0
-0.5171804934445,48.150052980838,0
-0.51705989976169,48.150104236881,0
-0.51694646043562,48.150162310134,0
-0.51684103879014,48.150226758645,0
-0.51674443714156,48.150297091945,0
-0.51665739069283,48.150372774778,0
-0.51658056193786,48.150453231175,0
-0.51651453561923,48.150537848835,0
-0.51645981427679,48.150625983785,0
-0.51641681442206,48.150716965282,0
-0.51638586336666,48.150810100915,0
-0.51636719672963,48.150904681875,0
-0.51636095664233,48.150999988349,0
-0.51800000000003,48.151,0
</coordinates>
</LinearRing>
</outerBoundaryIs>
</Polygon>
</Placemark>
<Placemark>
<name>406</name>
<styleUrl>#stylehap1</styleUrl>
<Polygon>
<outerBoundaryIs>
<LinearRing>
<coordinates>
-0.51800000000003,48.22,0
-0.51635874863481,48.219999988321,0
-0.5163649910478,48.220095294972,0
-0.51638367693548,48.220189876458,0
-0.51641466415415,48.22028301295,0
-0.51645771693548,48.220373995613,0
-0.51651250767876,48.220462132001,0
-0.51657861944259,48.220546751327,0
-0.51665554911645,48.220627209567,0
-0.5167427112485,48.220702894365,0
-0.51683944250023,48.22077322969,0
-0.51694500669456,48.220837680227,0
-0.5170586004181,48.220895755443,0
-0.51717935913608,48.22094701333,0
-0.51730636377228,48.220991063762,0
-0.51743864770484,48.221027571471,0
-0.51757520412393,48.221056258595,0
-0.51771499369575,48.221076906793,0
-0.51785695247421,48.22108935891,0
-0.51800000000002,48.221093520174,0
-0.51814304752586,48.22108935891,0
-0.5182850063043,48.221076906793,0
-0.51842479587612,48.221056258595,0
-0.51856135229524,48.221027571471,0
-0.51869363622779,48.220991063762,0
-0.51882064086399,48.22094701333,0

-0.51894139958194,48.220895755443,0
-0.51905499330549,48.220837680227,0
-0.51916055749982,48.22077322969,0
-0.51925728875157,48.220702894365,0
-0.51934445088359,48.220627209567,0
-0.51942138055746,48.220546751327,0
-0.51948749232129,48.220462132001,0
-0.51954228306457,48.220373995613,0
-0.51958533584589,48.22028301295,0
-0.51961632306459,48.220189876458,0
-0.51963500895224,48.220095294972,0
-0.51964125136524,48.219999988321,0
-0.51963500286436,48.219904681846,0
-0.51961631107377,48.219810100888,0
-0.51958531831651,48.219716965256,0
-0.51954226052925,48.219625983761,0
-0.51948746546471,48.219537848812,0
-0.51942135019568,48.219453231154,0
-0.51934441793913,48.219372774759,0
-0.51925725422541,48.219297091928,0
-0.51916052244105,48.21922675863,0
-0.51905495877935,48.219162310122,0
-0.51894136663748,48.219104236872,0
-0.51882061050219,48.21905298083,0
-0.51869360937121,48.219008932066,0
-0.51856132975989,48.218972425796,0
-0.51842477834674,48.218943739841,0
-0.51828499431348,48.218923092503,0
-0.51814304143795,48.218910640912,0
-0.51800000000002,48.218906479827,0
-0.5178569585621,48.218910640912,0
-0.51771500568657,48.218923092503,0
-0.51757522165331,48.218943739841,0
-0.51743867024019,48.218972425796,0
-0.51730639062886,48.219008932066,0
-0.51717938949786,48.21905298083,0
-0.51705863336259,48.219104236872,0
-0.5169450412207,48.219162310122,0
-0.51683947755903,48.21922675863,0
-0.51674274577464,48.219297091928,0
-0.51665558206094,48.219372774759,0
-0.51657864980439,48.219453231154,0
-0.51651253453533,48.219537848812,0
-0.51645773947083,48.219625983761,0
-0.51641468168354,48.219716965256,0
-0.51638368892628,48.219810100888,0
-0.51636499713569,48.219904681846,0
-0.51635874863481,48.219999988321,0
-0.51800000000003,48.22,0
</coordinates>
</LinearRing>
</outerBoundaryIs>
</Polygon>
</Placemark>
<Placemark>
<name>408</name>
<styleUrl>#stylehap1</styleUrl>

<Polygon>
<outerBoundaryIs>
<LinearRing>
<coordinates>
-0.51700000000005,48.22,0
-0.51535874863482,48.219999988321,0
-0.51536499104784,48.220095294972,0
-0.5153836769355,48.220189876458,0
-0.51541466415419,48.22028301295,0
-0.51545771693549,48.220373995613,0
-0.5155125076788,48.220462132001,0
-0.51557861944263,48.220546751327,0
-0.51565554911649,48.220627209567,0
-0.51574271124851,48.220702894365,0
-0.51583944250027,48.22077322969,0
-0.51594500669457,48.220837680227,0
-0.51605860041814,48.220895755443,0
-0.5161793591361,48.22094701333,0
-0.5163063637723,48.220991063762,0
-0.51643864770485,48.221027571471,0
-0.51657520412394,48.221056258595,0
-0.51671499369579,48.221076906793,0
-0.51685695247423,48.22108935891,0
-0.51700000000006,48.221093520174,0
-0.51714304752588,48.22108935891,0
-0.51728500630431,48.221076906793,0
-0.51742479587616,48.221056258595,0
-0.51756135229525,48.221027571471,0
-0.51769363622781,48.220991063762,0
-0.51782064086401,48.22094701333,0
-0.51794139958196,48.220895755443,0
-0.51805499330553,48.220837680227,0
-0.51816055749983,48.22077322969,0
-0.51825728875159,48.220702894365,0
-0.51834445088361,48.220627209567,0
-0.51842138055747,48.220546751327,0
-0.51848749232131,48.220462132001,0
-0.51854228306461,48.220373995613,0
-0.51858533584591,48.22028301295,0
-0.5186163230646,48.220189876458,0
-0.51863500895228,48.220095294972,0
-0.51864125136528,48.219999988321,0
-0.51863500286437,48.219904681846,0
-0.51861631107379,48.219810100888,0
-0.51858531831652,48.219716965256,0
-0.51854226052926,48.219625983761,0
-0.51848746546473,48.219537848812,0
-0.51842135019569,48.219453231154,0
-0.51834441793914,48.219372774759,0
-0.51825725422545,48.219297091928,0
-0.51816052244106,48.21922675863,0
-0.51805495877936,48.219162310122,0
-0.51794136663749,48.219104236872,0
-0.51782061050223,48.21905298083,0
-0.51769360937123,48.219008932066,0
-0.5175613297599,48.218972425796,0
-0.51742477834678,48.218943739841,0

-0.51728499431352,48.218923092503,0
-0.51714304143799,48.218910640912,0
-0.51700000000006,48.218906479827,0
-0.51685695856211,48.218910640912,0
-0.51671500568658,48.218923092503,0
-0.51657522165333,48.218943739841,0
-0.5164386702402,48.218972425796,0
-0.51630639062887,48.219008932066,0
-0.5161793894979,48.21905298083,0
-0.51605863336261,48.219104236872,0
-0.51594504122074,48.219162310122,0
-0.51583947755904,48.21922675863,0
-0.51574274577466,48.219297091928,0
-0.51565558206096,48.219372774759,0
-0.51557864980441,48.219453231154,0
-0.51551253453538,48.219537848812,0
-0.51545773947084,48.219625983761,0
-0.51541468168358,48.219716965256,0
-0.51538368892632,48.219810100888,0
-0.51536499713573,48.219904681846,0
-0.51535874863482,48.219999988321,0
-0.51700000000005,48.22,0
</coordinates>
</LinearRing>
</outerBoundaryIs>
</Polygon>
</Placemark>
<Placemark>
<name>409</name>
<styleUrl>#stylehap1</styleUrl>
<Polygon>
<outerBoundaryIs>
<LinearRing>
<coordinates>
-0.51700000000005,48.319,0
-0.51535556604001,48.31899998828,0
-0.5153618205472,48.319095294932,0
-0.51538054265888,48.319189876419,0
-0.51541158995607,48.319283012912,0
-0.51545472621344,48.319373995577,0
-0.51550962319539,48.319462131968,0
-0.51557586315221,48.319546751297,0
-0.51565294199799,48.31962720954,0
-0.51574027314567,48.319702894341,0
-0.51583719197069,48.31977322967,0
-0.51594296086832,48.31983768021,0
-0.51605677486729,48.31989575543,0
-0.51617776775625,48.31994701332,0
-0.51630501867669,48.319991063755,0
-0.51643755913213,48.320027571467,0
-0.5165743803602,48.320056258592,0
-0.51671444101144,48.320076906792,0
-0.5168566750763,48.32008935891,0
-0.51700000000006,48.320093520174,0
-0.51714332492383,48.32008935891,0
-0.51728555898866,48.320076906792,0
-0.5174256196399,48.320056258592,0

-0.51756244086798,48.320027571467,0
-0.51769498132341,48.319991063755,0
-0.51782223224386,48.31994701332,0
-0.51794322513282,48.31989575543,0
-0.51805703913178,48.31983768021,0
-0.51816280802941,48.31977322967,0
-0.51825972685443,48.319702894341,0
-0.51834705800212,48.31962720954,0
-0.51842413684789,48.319546751297,0
-0.51849037680472,48.319462131968,0
-0.51854527378667,48.319373995577,0
-0.51858841004404,48.319283012912,0
-0.51861945734122,48.319189876419,0
-0.5186381794529,48.319095294932,0
-0.5186444339601,48.31899998828,0
-0.51863817333194,48.318904681806,0
-0.51861944528529,48.318810100848,0
-0.51858839241947,48.318716965218,0
-0.51854525112895,48.318625983725,0
-0.51849034980229,48.318537848778,0
-0.51842410632123,48.318453231123,0
-0.51834702487875,48.318372774732,0
-0.51825969214079,48.318297091904,0
-0.51816277278026,48.31822675861,0
-0.51805700441814,48.318162310105,0
-0.51794319200945,48.318104236859,0
-0.5178222017172,48.31805298082,0
-0.51769495432099,48.318008932058,0
-0.51756241821027,48.317972425791,0
-0.51742560201533,48.317943739838,0
-0.51728554693276,48.317923092502,0
-0.51714331880287,48.317910640912,0
-0.51700000000006,48.317906479826,0
-0.51685668119724,48.317910640912,0
-0.51671445306735,48.317923092502,0
-0.51657439798478,48.317943739838,0
-0.51643758178984,48.317972425791,0
-0.51630504567911,48.318008932058,0
-0.51617779828291,48.31805298082,0
-0.51605680799065,48.318104236859,0
-0.51594299558196,48.318162310105,0
-0.51583722721984,48.31822675861,0
-0.51574030785932,48.318297091904,0
-0.51565297512135,48.318372774732,0
-0.51557589367887,48.318453231123,0
-0.51550965019781,48.318537848778,0
-0.51545474887115,48.318625983725,0
-0.51541160758064,48.318716965218,0
-0.51538055471481,48.318810100848,0
-0.51536182666817,48.318904681806,0
-0.51535556604001,48.31899998828,0
-0.51700000000005,48.319,0
</coordinates>
</LinearRing>
</outerBoundaryIs>
</Polygon>
</Placemark>

<Placemark>
<name>410</name>
<styleUrl>#stylehap1</styleUrl>
<Polygon>
<outerBoundaryIs>
<LinearRing>
<coordinates>
-0.51700000000005,48.216,0
-0.5153588768627,48.215999988322,0
-0.51536511878844,48.216095294974,0
-0.51538380321662,48.21618987646,0
-0.51541478801471,48.216283012952,0
-0.51545783743274,48.216373995615,0
-0.51551262389565,48.216462132003,0
-0.51557873049455,48.216546751328,0
-0.51565565415821,48.216627209568,0
-0.51574280948053,48.216702894366,0
-0.51583953317491,48.216773229691,0
-0.51594508912165,48.216837680227,0
-0.51605867397023,48.216895755444,0
-0.51617942325336,48.21694701333,0
-0.51630641796668,48.216991063763,0
-0.51643869156384,48.217027571472,0
-0.51657523731369,48.217056258595,0
-0.51671501596364,48.217076906793,0
-0.5168569636507,48.21708935891,0
-0.51700000000006,48.217093520174,0
-0.51714303634941,48.21708935891,0
-0.51728498403649,48.217076906793,0
-0.51742476268641,48.217056258595,0
-0.51756130843627,48.217027571472,0
-0.51769358203342,48.216991063763,0
-0.51782057674675,48.21694701333,0
-0.51794132602988,48.216895755444,0
-0.51805491087845,48.216837680227,0
-0.5181604668252,48.216773229691,0
-0.51825719051957,48.216702894366,0
-0.5183443458419,48.216627209568,0
-0.51842126950556,48.216546751328,0
-0.51848737610445,48.216462132003,0
-0.51854216256736,48.216373995615,0
-0.51858521198539,48.216283012952,0
-0.51861619678348,48.21618987646,0
-0.51863488121167,48.216095294974,0
-0.5186411231374,48.215999988322,0
-0.51863487512513,48.215904681848,0
-0.51861618479531,48.215810100889,0
-0.51858519445982,48.215716965258,0
-0.51854214003693,48.215625983762,0
-0.51848734925375,48.215537848813,0
-0.51842123915042,48.215453231155,0
-0.51834431290463,48.21537277476,0
-0.51825715600096,48.215297091929,0
-0.51816043177411,48.215226758631,0
-0.51805487635987,48.215162310123,0
-0.51794129309261,48.215104236873,0
-0.51782054639161,48.215052980831,0

-0.51769355518272,48.215008932066,0
-0.51756128590585,48.214972425796,0
-0.51742474516087,48.214943739841,0
-0.51728497204829,48.214923092503,0
-0.51714303026284,48.214910640912,0
-0.51700000000006,48.214906479827,0
-0.51685696973726,48.214910640912,0
-0.51671502795182,48.214923092503,0
-0.51657525483923,48.214943739841,0
-0.51643871409425,48.214972425796,0
-0.51630644481738,48.215008932066,0
-0.51617945360852,48.215052980831,0
-0.51605870690749,48.215104236873,0
-0.51594512364026,48.215162310123,0
-0.51583956822602,48.215226758631,0
-0.51574284399914,48.215297091929,0
-0.51565568709547,48.21537277476,0
-0.51557876084968,48.215453231155,0
-0.51551265074636,48.215537848813,0
-0.51545785996318,48.215625983762,0
-0.51541480554028,48.215716965258,0
-0.5153838152048,48.215810100889,0
-0.51536512487498,48.215904681848,0
-0.5153588768627,48.215999988322,0
-0.51700000000005,48.216,0
</coordinates>
</LinearRing>
</outerBoundaryIs>
</Polygon>
</Placemark>
<Placemark>
<name>412</name>
<styleUrl>#stylehap1</styleUrl>
<Polygon>
<outerBoundaryIs>
<LinearRing>
<coordinates>
-0.51599999999996,48.233,0
-0.51435833170045,48.232999988315,0
-0.51436457569785,48.233095294967,0
-0.51438326633105,48.233189876453,0
-0.51441426142026,48.233283012945,0
-0.51445732513733,48.233373995609,0
-0.51451212979836,48.233462131997,0
-0.51457825835605,48.233546751323,0
-0.51465520757211,48.233627209564,0
-0.51474239184596,48.233702894361,0
-0.51483914767065,48.233773229688,0
-0.51494473868202,48.233837680224,0
-0.51505836126266,48.233895755441,0
-0.51517915065808,48.233947013329,0
-0.51530618755861,48.233991063762,0
-0.51543850509684,48.234027571471,0
-0.5155750962071,48.234056258594,0
-0.51571492129155,48.234076906793,0
-0.51585691613376,48.23408935891,0
-0.51599999999995,48.234093520174,0

-0.51614308386617,48.23408935891,0
-0.51628507870838,48.234076906793,0
-0.51642490379283,48.234056258594,0
-0.51656149490309,48.234027571471,0
-0.51669381244132,48.233991063762,0
-0.51682084934185,48.233947013329,0
-0.51694163873727,48.233895755441,0
-0.51705526131791,48.233837680224,0
-0.51716085232928,48.233773229688,0
-0.51725760815397,48.233702894361,0
-0.51734479242782,48.233627209564,0
-0.51742174164388,48.233546751323,0
-0.51748787020157,48.233462131997,0
-0.5175426748626,48.233373995609,0
-0.51758573857967,48.233283012945,0
-0.51761673366889,48.233189876453,0
-0.51763542430208,48.233095294967,0
-0.51764166829948,48.232999988315,0
-0.51763541820984,48.232904681841,0
-0.51761672166957,48.232810100882,0
-0.51758572103781,48.232716965251,0
-0.51754265231123,48.232625983756,0
-0.51748784332589,48.232537848807,0
-0.5174217112605,48.23245323115,0
-0.51734475945992,48.232372774755,0
-0.51725757360328,48.232297091925,0
-0.51716081724557,48.232226758628,0
-0.51705522676719,48.23216231012,0
-0.51694160576937,48.23210423687,0
-0.51682081895847,48.232052980829,0
-0.51669378556563,48.232008932065,0
-0.51656147235174,48.231972425795,0
-0.51642488625098,48.23194373984,0
-0.51628506670904,48.231923092503,0
-0.51614307777393,48.231910640912,0
-0.51599999999995,48.231906479826,0
-0.51585692222597,48.231910640912,0
-0.51571493329089,48.231923092503,0
-0.51557511374895,48.23194373984,0
-0.51543852764819,48.231972425795,0
-0.5153062144343,48.232008932065,0
-0.51517918104146,48.232052980829,0
-0.51505839423056,48.23210423687,0
-0.51494477323272,48.23216231012,0
-0.51483918275436,48.232226758628,0
-0.51474242639665,48.232297091925,0
-0.51465524054001,48.232372774755,0
-0.51457828873943,48.23245323115,0
-0.51451215667404,48.232537848807,0
-0.51445734768871,48.232625983756,0
-0.51441427896212,48.232716965251,0
-0.51438327833036,48.232810100882,0
-0.51436458179006,48.232904681841,0
-0.51435833170045,48.232999988315,0
-0.51599999999996,48.233,0
</coordinates>
</LinearRing>

</outerBoundaryIs>
</Polygon>
</Placemark>
<Placemark>
<name>413</name>
<styleUrl>#stylehap1</styleUrl>
<Polygon>
<outerBoundaryIs>
<LinearRing>
<coordinates>
-0.51599999999996,48.213,0
-0.51435897301513,48.212999988324,0
-0.51436521457546,48.213095294975,0
-0.51438389790926,48.213189876461,0
-0.51441488089227,48.213283012953,0
-0.51445792778832,48.213373995616,0
-0.51451271104152,48.213462132004,0
-0.51457881376746,48.213546751329,0
-0.51465573292435,48.213627209569,0
-0.51474288314036,48.213702894366,0
-0.51483960116778,48.213773229692,0
-0.51494515093002,48.213837680228,0
-0.51505872912366,48.213895755444,0
-0.51517947133201,48.213947013331,0
-0.51530645860459,48.213991063763,0
-0.51543872445168,48.214027571472,0
-0.5155752622011,48.214056258595,0
-0.51571503266124,48.214076906793,0
-0.51585697203135,48.21408935891,0
-0.51599999999995,48.214093520174,0
-0.51614302796859,48.21408935891,0
-0.51628496733869,48.214076906793,0
-0.51642473779879,48.214056258595,0
-0.51656127554823,48.214027571472,0
-0.51669354139534,48.213991063763,0
-0.51682052866792,48.213947013331,0
-0.51694127087627,48.213895755444,0
-0.51705484906991,48.213837680228,0
-0.51716039883215,48.213773229692,0
-0.51725711685955,48.213702894366,0
-0.51734426707558,48.213627209569,0
-0.51742118623247,48.213546751329,0
-0.51748728895841,48.213462132004,0
-0.51754207221161,48.213373995616,0
-0.51758511910766,48.213283012953,0
-0.51761610209067,48.213189876461,0
-0.51763478542447,48.213095294975,0
-0.5176410269848,48.212999988324,0
-0.5176347793389,48.212904681849,0
-0.51761609010446,48.21281010089,0
-0.51758510158499,48.212716965259,0
-0.51754204968489,48.212625983763,0
-0.51748726211209,48.212537848814,0
-0.51742115588229,48.212453231156,0
-0.51734423414371,48.212372774761,0
-0.51725708234661,48.21229709193,0
-0.51716036378679,48.212226758632,0

-0.51705481455695,48.212162310123,0
-0.5169412379444,48.212104236873,0
-0.51682049831774,48.212052980831,0
-0.51669351454904,48.212008932066,0
-0.5165612530215,48.211972425796,0
-0.51642472027612,48.211943739841,0
-0.51628495535248,48.211923092503,0
-0.51614302188301,48.211910640912,0
-0.51599999999995,48.211906479827,0
-0.51585697811692,48.211910640912,0
-0.51571504464745,48.211923092503,0
-0.51557527972381,48.211943739841,0
-0.5154387469784,48.211972425796,0
-0.51530648545089,48.212008932066,0
-0.51517950168219,48.212052980831,0
-0.51505876205553,48.212104236873,0
-0.51494518544298,48.212162310123,0
-0.51483963621314,48.212226758632,0
-0.51474291765332,48.21229709193,0
-0.51465576585622,48.212372774761,0
-0.51457884411764,48.212453231156,0
-0.51451273788782,48.212537848814,0
-0.51445795031504,48.212625983763,0
-0.51441489841494,48.212716965259,0
-0.51438390989547,48.21281010089,0
-0.51436522066101,48.212904681849,0
-0.51435897301513,48.212999988324,0
-0.51599999999996,48.213,0
</coordinates>
</LinearRing>
</outerBoundaryIs>
</Polygon>
</Placemark>
<Placemark>
<name>414</name>
<styleUrl>#stylehap1</styleUrl>
<Polygon>
<outerBoundaryIs>
<LinearRing>
<coordinates>
-0.51400000000001,47.963,0
-0.51236693064461,47.962999988426,0
-0.51237314196496,47.963095295076,0
-0.51239173472562,47.96318987656,0
-0.51242256749075,47.963283013048,0
-0.51246540566659,47.963373995706,0
-0.51251992328493,47.963462132088,0
-0.51258570548231,47.963546751406,0
-0.51266225165585,47.963627209638,0
-0.51274897927199,47.963702894426,0
-0.51284522829891,47.963773229743,0
-0.51295026622943,47.96383768027,0
-0.51306329365549,47.963895755478,0
-0.51318345035233,47.963947013356,0
-0.51330982182599,47.963991063781,0
-0.51344144627387,47.964027571484,0
-0.51357732190591,47.964056258602,0

-0.51371641457032,47.964076906796,0
-0.51385766562572,47.964089358911,0
-0.51400000000001,47.964093520174,0
-0.51414233437429,47.964089358911,0
-0.5142835854297,47.964076906796,0
-0.5144226780941,47.964056258602,0
-0.51455855372615,47.964027571484,0
-0.51469017817403,47.963991063781,0
-0.51481654964769,47.963947013356,0
-0.51493670634455,47.963895755478,0
-0.51504973377059,47.96383768027,0
-0.5151547717011,47.963773229743,0
-0.51525102072805,47.963702894426,0
-0.51533774834416,47.963627209638,0
-0.51541429451771,47.963546751406,0
-0.51548007671511,47.963462132088,0
-0.51553459433343,47.963373995706,0
-0.51557743250927,47.963283013048,0
-0.5156082652744,47.96318987656,0
-0.51562685803506,47.963095295076,0
-0.51563306935543,47.962999988426,0
-0.51562685203192,47.962904681951,0
-0.51560825345052,47.962810100989,0
-0.51557741522394,47.962716965354,0
-0.51553457211186,47.962625983853,0
-0.51548005023247,47.962537848898,0
-0.51541426457867,47.962453231233,0
-0.51533771585839,47.962372774829,0
-0.5152509866826,47.962297091989,0
-0.51515473713046,47.962226758683,0
-0.51504969972514,47.962162310165,0
-0.51493667385877,47.962104236907,0
-0.51481651970862,47.962052980857,0
-0.51469015169138,47.962008932084,0
-0.51455853150458,47.961972425808,0
-0.51442266080878,47.961943739848,0
-0.51428357360584,47.961923092506,0
-0.51414232837119,47.961910640913,0
-0.51400000000001,47.961906479826,0
-0.51385767162885,47.961910640913,0
-0.51371642639417,47.961923092506,0
-0.51357733919124,47.961943739848,0
-0.51344146849544,47.961972425808,0
-0.51330984830864,47.962008932084,0
-0.5131834802914,47.962052980857,0
-0.51306332614125,47.962104236907,0
-0.51295030027487,47.962162310165,0
-0.51284526286956,47.962226758683,0
-0.51274901331741,47.962297091989,0
-0.51266228414163,47.962372774829,0
-0.51258573542135,47.962453231233,0
-0.51251994976758,47.962537848898,0
-0.51246542788816,47.962625983853,0
-0.51242258477607,47.962716965354,0
-0.5123917465495,47.962810100989,0
-0.51237314796809,47.962904681951,0
-0.51236693064461,47.962999988426,0

-0.51400000000001,47.963,0
</coordinates>
</LinearRing>
</outerBoundaryIs>
</Polygon>
</Placemark>
<Placemark>
<name>415</name>
<styleUrl>#stylehap1</styleUrl>
<Polygon>
<outerBoundaryIs>
<LinearRing>
<coordinates>
-0.51300000000003,48.231,0
-0.51135839586356,48.230999988316,0
-0.51136463961712,48.231095294968,0
-0.51138332952001,48.231189876454,0
-0.51141432339802,48.231283012946,0
-0.51145738543214,48.231373995609,0
-0.51151218795136,48.231462131998,0
-0.51157831392459,48.231546751324,0
-0.51165526013327,48.231627209564,0
-0.51174244099964,48.231702894362,0
-0.51183919304276,48.231773229688,0
-0.51194477992719,48.231837680225,0
-0.51205839806692,48.231895755442,0
-0.51217918274132,48.231947013329,0
-0.51230621467663,48.231991063762,0
-0.51243852704317,48.232027571471,0
-0.51257511281473,48.232056258594,0
-0.51271493243407,48.232076906793,0
-0.51285692172634,48.23208935891,0
-0.51300000000002,48.232093520174,0
-0.51314307827373,48.23208935891,0
-0.513285067566,48.232076906793,0
-0.51342488718532,48.232056258594,0
-0.51356147295688,48.232027571471,0
-0.51369378532344,48.231991063762,0
-0.51382081725875,48.231947013329,0
-0.51394160193313,48.231895755442,0
-0.51405522007288,48.231837680225,0
-0.51416080695731,48.231773229688,0
-0.51425755900043,48.231702894362,0
-0.51434473986681,48.231627209564,0
-0.51442168607546,48.231546751324,0
-0.51448781204871,48.231462131998,0
-0.51454261456791,48.231373995609,0
-0.51458567660205,48.231283012946,0
-0.51461667048006,48.231189876454,0
-0.51463536038295,48.231095294968,0
-0.51464160413651,48.230999988316,0
-0.5146353542914,48.230904681842,0
-0.51461665848204,48.230810100883,0
-0.51458565906211,48.230716965252,0
-0.51454259201902,48.230625983757,0
-0.51448778517598,48.230537848808,0
-0.51442165569541,48.23045323115,0

-0.51434470690252,48.230372774756,0
-0.5142575244535,48.230297091925,0
-0.51416077187745,48.230226758628,0
-0.51405518552598,48.23016231012,0
-0.51394156896885,48.230104236871,0
-0.5138207868787,48.230052980829,0
-0.51369375845071,48.230008932065,0
-0.513561450408,48.229972425796,0
-0.5134248696454,48.22994373984,0
-0.51328505556799,48.229923092503,0
-0.51314307218218,48.229910640912,0
-0.51300000000002,48.229906479827,0
-0.51285692781789,48.229910640912,0
-0.51271494443209,48.229923092503,0
-0.51257513035467,48.22994373984,0
-0.51243854959208,48.229972425796,0
-0.51230624154937,48.230008932065,0
-0.51217921312137,48.230052980829,0
-0.51205843103123,48.230104236871,0
-0.5119448144741,48.23016231012,0
-0.5118392281226,48.230226758628,0
-0.51174247554657,48.230297091925,0
-0.51165529309755,48.230372774756,0
-0.51157834430466,48.23045323115,0
-0.5115122148241,48.230537848808,0
-0.51145740798105,48.230625983757,0
-0.51141434093794,48.230716965252,0
-0.51138334151803,48.230810100883,0
-0.51136464570867,48.230904681842,0
-0.51135839586356,48.230999988316,0
-0.51300000000003,48.231,0
</coordinates>
</LinearRing>
</outerBoundaryIs>
</Polygon>
</Placemark>
<Placemark>
<name>418</name>
<styleUrl>#stylehap1</styleUrl>
<Polygon>
<outerBoundaryIs>
<LinearRing>
<coordinates>
-0.51300000000003,48.26,0
-0.51135746481256,48.259999988304,0
-0.51136371210422,48.260095294956,0
-0.51138241260426,48.260189876442,0
-0.51141342405792,48.260283012935,0
-0.51145651051251,48.260373995599,0
-0.51151134411127,48.260462131988,0
-0.51157750758668,48.260546751315,0
-0.51165449743478,48.260627209556,0
-0.5117417277458,48.260702894355,0
-0.51183853466245,48.260773229682,0
-0.51194418143174,48.26083768022,0
-0.51205786401186,48.260895755438,0
-0.51217871719173,48.260947013326,0

-0.51230582117614,48.26099106376,0
-0.5124382085871,48.26102757147,0
-0.5125748718271,48.261056258594,0
-0.51271477074925,48.261076906792,0
-0.51285684057507,48.26108935891,0
-0.51300000000002,48.261093520173,0
-0.513143159425,48.26108935891,0
-0.51328522925083,48.261076906792,0
-0.51342512817297,48.261056258594,0
-0.51356179141297,48.26102757147,0
-0.51369417882391,48.26099106376,0
-0.51382128280835,48.260947013326,0
-0.51394213598821,48.260895755438,0
-0.51405581856834,48.26083768022,0
-0.51416146533763,48.260773229682,0
-0.51425827225427,48.260702894355,0
-0.5143455025653,48.260627209556,0
-0.51442249241337,48.260546751315,0
-0.5144886558888,48.260462131988,0
-0.51454348948754,48.260373995599,0
-0.51458657594215,48.260283012935,0
-0.51461758739581,48.260189876442,0
-0.51463628789582,48.260095294956,0
-0.51464253518749,48.259999988304,0
-0.5146362817946,48.25990468183,0
-0.51461757537873,48.259810100872,0
-0.51458655837437,48.259716965241,0
-0.51454346690285,48.259625983746,0
-0.51448862897342,48.259537848798,0
-0.51442246198511,48.259453231142,0
-0.51434546954867,48.259372774748,0
-0.51425823765253,48.259297091918,0
-0.51416143020209,48.259226758622,0
-0.51405578396657,48.259162310115,0
-0.51394210297161,48.259104236867,0
-0.51382125238008,48.259052980826,0
-0.51369415190853,48.259008932063,0
-0.51356176882829,48.258972425794,0
-0.51342511060521,48.258943739839,0
-0.51328521723377,48.258923092502,0
-0.51314315332378,48.258910640912,0
-0.51300000000002,48.258906479826,0
-0.51285684667629,48.258910640912,0
-0.5127147827663,48.258923092502,0
-0.51257488939486,48.258943739839,0
-0.51243823117179,48.258972425794,0
-0.51230584809155,48.259008932063,0
-0.51217874761999,48.259052980826,0
-0.51205789702846,48.259104236867,0
-0.51194421603347,48.259162310115,0
-0.51183856979798,48.259226758622,0
-0.51174176234754,48.259297091918,0
-0.51165453045138,48.259372774748,0
-0.51157753801497,48.259453231142,0
-0.51151137102665,48.259537848798,0
-0.5114565330972,48.259625983746,0
-0.51141344162567,48.259716965241,0

-0.51138242462131,48.259810100872,0
-0.51136371820547,48.25990468183,0
-0.51135746481256,48.259999988304,0
-0.51300000000003,48.26,0
</coordinates>
</LinearRing>
</outerBoundaryIs>
</Polygon>
</Placemark>
<Placemark>
<name>419</name>
<styleUrl>#stylehap1</styleUrl>
<Polygon>
<outerBoundaryIs>
<LinearRing>
<coordinates>
-0.51300000000003,48.239,0
-0.51135813916936,48.238999988313,0
-0.51136438389839,48.239095294965,0
-0.51138307672293,48.239189876451,0
-0.51141407544664,48.239283012943,0
-0.51145714421358,48.239373995606,0
-0.51151195530152,48.239462131995,0
-0.51157809161425,48.239546751321,0
-0.51165504985446,48.239627209562,0
-0.51174224435292,48.23970289436,0
-0.51183901152487,48.239773229686,0
-0.51194461491976,48.239837680223,0
-0.51205825082596,48.239895755441,0
-0.51217905438757,48.239947013328,0
-0.5123061061871,48.239991063761,0
-0.51243843924365,48.240027571471,0
-0.51257504637355,48.240056258594,0
-0.51271488785697,48.240076906793,0
-0.51285689935264,48.24008935891,0
-0.51300000000002,48.240093520174,0
-0.51314310064744,48.24008935891,0
-0.51328511214311,48.240076906793,0
-0.51342495362652,48.240056258594,0
-0.5135615607564,48.240027571471,0
-0.51369389381298,48.239991063761,0
-0.5138209456125,48.239947013328,0
-0.51394174917409,48.239895755441,0
-0.51405538508029,48.239837680223,0
-0.51416098847521,48.239773229686,0
-0.51425775564715,48.23970289436,0
-0.51434495014559,48.239627209562,0
-0.5144219083858,48.239546751321,0
-0.51448804469855,48.239462131995,0
-0.51454285578649,48.239373995606,0
-0.51458592455344,48.239283012943,0
-0.51461692327711,48.239189876451,0
-0.51463561610169,48.239095294965,0
-0.51464186083071,48.238999988313,0
-0.51463561000746,48.238904681839,0
-0.51461691127385,48.23881010088,0
-0.51458590700583,48.238716965249,0

-0.51454283322774,48.238625983754,0
-0.51448801781406,48.238537848805,0
-0.51442187799247,48.238453231148,0
-0.51434491716688,48.238372774754,0
-0.51425772108511,48.238297091923,0
-0.51416095338,48.238226758626,0
-0.51405535051827,48.238162310119,0
-0.51394171619537,48.23810423687,0
-0.51382091521915,48.238052980828,0
-0.51369386692849,48.238008932064,0
-0.51356153819764,48.237972425795,0
-0.51342493607892,48.23794373984,0
-0.51328510013982,48.237923092503,0
-0.51314309455321,48.237910640912,0
-0.51300000000002,48.237906479826,0
-0.51285690544686,48.237910640912,0
-0.51271489986023,48.237923092503,0
-0.51257506392116,48.23794373984,0
-0.51243846180243,48.237972425795,0
-0.51230613307159,48.238008932064,0
-0.51217908478093,48.238052980828,0
-0.51205828380467,48.23810423687,0
-0.51194464948181,48.238162310119,0
-0.51183904662008,48.238226758626,0
-0.51174227891496,48.238297091923,0
-0.51165508283317,48.238372774754,0
-0.5115781220076,48.238453231148,0
-0.51151198218601,48.238537848805,0
-0.51145716677234,48.238625983754,0
-0.51141409299424,48.238716965249,0
-0.51138308872622,48.23881010088,0
-0.51136438999261,48.238904681839,0
-0.51135813916936,48.238999988313,0
-0.51300000000003,48.239,0
</coordinates>
</LinearRing>
</outerBoundaryIs>
</Polygon>
</Placemark>
<Placemark>
<name>423</name>
<styleUrl>#stylehap1</styleUrl>
<Polygon>
<outerBoundaryIs>
<LinearRing>
<coordinates>
-0.50999999999999,48.252,0
-0.50835772180168,48.251999988308,0
-0.50836396811676,48.252095294959,0
-0.50838266569176,48.252189876445,0
-0.50841367229418,48.252283012938,0
-0.50845675200821,48.252373995602,0
-0.50851157702838,48.252462131991,0
-0.50857773015242,48.252546751317,0
-0.50865470795515,48.252627209558,0
-0.50874192461842,48.252702894357,0
-0.50883871638885,48.252773229684,0

-0.5089443466287,48.252837680221,0
-0.50905801142197,48.252895755439,0
-0.5091788456929,48.252947013327,0
-0.5093059297903,48.25299106376,0
-0.50943829648745,48.25302757147,0
-0.50957493834458,48.253056258594,0
-0.50971481537751,48.253076906792,0
-0.50985686297444,48.25308935891,0
-0.50999999999999,48.253093520174,0
-0.51014313702555,48.25308935891,0
-0.51028518462245,48.253076906792,0
-0.5104250616554,48.253056258594,0
-0.51056170351254,48.25302757147,0
-0.51069407020969,48.25299106376,0
-0.51082115430709,48.252947013327,0
-0.51094198857802,48.252895755439,0
-0.51105565337129,48.252837680221,0
-0.51116128361113,48.252773229684,0
-0.51125807538154,48.252702894357,0
-0.51134529204484,48.252627209558,0
-0.51142226984756,48.252546751317,0
-0.51148842297161,48.252462131991,0
-0.51154324799175,48.252373995602,0
-0.51158632770581,48.252283012938,0
-0.51161733430823,48.252189876445,0
-0.51163603188323,48.252095294959,0
-0.51164227819831,48.251999988308,0
-0.51163602578468,48.251904681833,0
-0.51161732229642,48.251810100875,0
-0.51158631014574,48.251716965244,0
-0.51154322541694,48.251625983749,0
-0.51148839606799,48.251537848801,0
-0.51142223943261,48.251453231144,0
-0.51134525904269,48.25137277475,0
-0.51125804079495,48.25129709192,0
-0.51116124849097,48.251226758624,0
-0.51105561878467,48.251162310117,0
-0.51094195557585,48.251104236868,0
-0.51082112389213,48.251052980827,0
-0.51069404330607,48.251008932063,0
-0.51056168093772,48.250972425795,0
-0.51042504409533,48.25094373984,0
-0.51028517261066,48.250923092503,0
-0.510143130927,48.250910640912,0
-0.50999999999999,48.250906479826,0
-0.50985686907299,48.250910640912,0
-0.50971482738932,48.250923092503,0
-0.50957495590466,48.25094373984,0
-0.50943831906226,48.250972425795,0
-0.50930595669392,48.251008932063,0
-0.50917887610786,48.251052980827,0
-0.50905804442411,48.251104236868,0
-0.50894438121532,48.251162310117,0
-0.50883875150902,48.251226758624,0
-0.50874195920504,48.25129709192,0
-0.5086547409573,48.25137277475,0
-0.50857776056738,48.251453231144,0

-0.50851160393198,48.251537848801,0
-0.50845677458302,48.251625983749,0
-0.50841368985425,48.251716965244,0
-0.50838267770354,48.251810100875,0
-0.50836397421531,48.251904681833,0
-0.50835772180168,48.251999988308,0
-0.50999999999999,48.252,0
</coordinates>
</LinearRing>
</outerBoundaryIs>
</Polygon>
</Placemark>
<Placemark>
<name>424</name>
<styleUrl>#stylehap1</styleUrl>
<Polygon>
<outerBoundaryIs>
<LinearRing>
<coordinates>
-0.50999999999999,48.31,0
-0.50835585607965,48.309999988284,0
-0.50836210948467,48.310095294936,0
-0.50838082829514,48.310189876422,0
-0.50841187011716,48.310283012916,0
-0.50845499876713,48.310373995581,0
-0.50850988606722,48.310462131971,0
-0.50857611434141,48.310546751299,0
-0.50865317959268,48.310627209542,0
-0.50874049533747,48.310702894343,0
-0.50883739706833,48.310773229672,0
-0.50894314731074,48.310837680211,0
-0.50905694123531,48.310895755431,0
-0.50917791278354,48.310947013321,0
-0.50930514125934,48.310991063756,0
-0.50943765833707,48.311027571467,0
-0.50957445543226,48.311056258592,0
-0.50971449137922,48.311076906792,0
-0.50985670035637,48.31108935891,0
-0.50999999999999,48.311093520174,0
-0.51014329964362,48.31108935891,0
-0.51028550862077,48.311076906792,0
-0.5104255445677,48.311056258592,0
-0.51056234166292,48.311027571467,0
-0.51069485874064,48.310991063756,0
-0.51082208721642,48.310947013321,0
-0.51094305876467,48.310895755431,0
-0.51105685268925,48.310837680211,0
-0.51116260293163,48.310773229672,0
-0.51125950466251,48.310702894343,0
-0.51134682040731,48.310627209542,0
-0.51142388565855,48.310546751299,0
-0.51149011393277,48.310462131971,0
-0.51154500123286,48.310373995581,0
-0.51158812988282,48.310283012916,0
-0.51161917170485,48.310189876422,0
-0.51163789051532,48.310095294936,0
-0.51164414392033,48.309999988284,0

-0.51163788439738,48.30990468181,0
-0.51161915965485,48.309810100852,0
-0.51158811226693,48.309716965221,0
-0.51154497858631,48.309625983728,0
-0.51149008694365,48.309537848781,0
-0.51142385514693,48.309453231126,0
-0.51134678730025,48.309372774734,0
-0.51125946996597,48.309297091906,0
-0.51116256769983,48.309226758612,0
-0.51105681799271,48.309162310107,0
-0.51094302565762,48.30910423686,0
-0.5108220567048,48.309052980821,0
-0.51069483175153,48.309008932059,0
-0.51056231901635,48.308972425792,0
-0.5104255269518,48.308943739838,0
-0.51028549657079,48.308923092502,0
-0.51014329352568,48.308910640912,0
-0.50999999999999,48.308906479826,0
-0.50985670647428,48.308910640912,0
-0.50971450342919,48.308923092502,0
-0.50957447304816,48.308943739838,0
-0.50943768098363,48.308972425792,0
-0.50930516824846,48.309008932059,0
-0.50917794329519,48.309052980821,0
-0.50905697434237,48.30910423686,0
-0.50894318200728,48.309162310107,0
-0.50883743230013,48.309226758612,0
-0.50874053003402,48.309297091906,0
-0.50865321269973,48.309372774734,0
-0.50857614485306,48.309453231126,0
-0.50850991305634,48.309537848781,0
-0.50845502141367,48.309625983728,0
-0.50841188773306,48.309716965221,0
-0.50838084034512,48.309810100852,0
-0.50836211560261,48.30990468181,0
-0.50835585607965,48.309999988284,0
-0.50999999999999,48.31,0
</coordinates>
</LinearRing>
</outerBoundaryIs>
</Polygon>
</Placemark>
<Placemark>
<name>425</name>
<styleUrl>#stylehap1</styleUrl>
<Polygon>
<outerBoundaryIs>
<LinearRing>
<coordinates>
-0.50999999999999,48.31,0
-0.50835585607965,48.309999988284,0
-0.50836210948467,48.310095294936,0
-0.50838082829514,48.310189876422,0
-0.50841187011716,48.310283012916,0
-0.50845499876713,48.310373995581,0
-0.50850988606722,48.310462131971,0
-0.50857611434141,48.310546751299,0

-0.50865317959268,48.310627209542,0
-0.50874049533747,48.310702894343,0
-0.50883739706833,48.310773229672,0
-0.50894314731074,48.310837680211,0
-0.50905694123531,48.310895755431,0
-0.50917791278354,48.310947013321,0
-0.50930514125934,48.310991063756,0
-0.50943765833707,48.311027571467,0
-0.50957445543226,48.311056258592,0
-0.50971449137922,48.311076906792,0
-0.50985670035637,48.31108935891,0
-0.50999999999999,48.311093520174,0
-0.51014329964362,48.31108935891,0
-0.51028550862077,48.311076906792,0
-0.5104255445677,48.311056258592,0
-0.51056234166292,48.311027571467,0
-0.51069485874064,48.310991063756,0
-0.51082208721642,48.310947013321,0
-0.51094305876467,48.310895755431,0
-0.51105685268925,48.310837680211,0
-0.51116260293163,48.310773229672,0
-0.51125950466251,48.310702894343,0
-0.51134682040731,48.310627209542,0
-0.51142388565855,48.310546751299,0
-0.51149011393277,48.310462131971,0
-0.51154500123286,48.310373995581,0
-0.51158812988282,48.310283012916,0
-0.51161917170485,48.310189876422,0
-0.51163789051532,48.310095294936,0
-0.51164414392033,48.309999988284,0
-0.51163788439738,48.30990468181,0
-0.51161915965485,48.309810100852,0
-0.51158811226693,48.309716965221,0
-0.51154497858631,48.309625983728,0
-0.51149008694365,48.309537848781,0
-0.51142385514693,48.309453231126,0
-0.51134678730025,48.309372774734,0
-0.51125946996597,48.309297091906,0
-0.51116256769983,48.309226758612,0
-0.51105681799271,48.309162310107,0
-0.51094302565762,48.30910423686,0
-0.5108220567048,48.309052980821,0
-0.51069483175153,48.309008932059,0
-0.51056231901635,48.308972425792,0
-0.5104255269518,48.308943739838,0
-0.51028549657079,48.308923092502,0
-0.51014329352568,48.308910640912,0
-0.50999999999999,48.308906479826,0
-0.50985670647428,48.308910640912,0
-0.50971450342919,48.308923092502,0
-0.50957447304816,48.308943739838,0
-0.50943768098363,48.308972425792,0
-0.50930516824846,48.309008932059,0
-0.50917794329519,48.309052980821,0
-0.50905697434237,48.30910423686,0
-0.50894318200728,48.309162310107,0
-0.50883743230013,48.309226758612,0

-0.50874053003402,48.309297091906,0
-0.50865321269973,48.309372774734,0
-0.50857614485306,48.309453231126,0
-0.50850991305634,48.309537848781,0
-0.50845502141367,48.309625983728,0
-0.50841188773306,48.309716965221,0
-0.50838084034512,48.309810100852,0
-0.50836211560261,48.30990468181,0
-0.50835585607965,48.309999988284,0
-0.50999999999999,48.31,0
</coordinates>
</LinearRing>
</outerBoundaryIs>
</Polygon>
</Placemark>
<Placemark>
<name>429</name>
<styleUrl>#stylehap1</styleUrl>
<Polygon>
<outerBoundaryIs>
<LinearRing>
<coordinates>
-0.50900000000001,48.32,0
-0.50735553380452,48.31999998828,0
-0.50736178843423,48.320095294931,0
-0.50738051091279,48.320189876418,0
-0.50741155881848,48.320283012912,0
-0.50745469592137,48.320373995577,0
-0.50750959397937,48.320462131968,0
-0.50757583523462,48.320546751296,0
-0.50765291559129,48.32062720954,0
-0.5077402484509,48.320702894341,0
-0.50783716917578,48.32077322967,0
-0.50794294014677,48.32083768021,0
-0.50805675637683,48.32089575543,0
-0.50817775163765,48.32094701332,0
-0.50830500505261,48.320991063755,0
-0.50843754810628,48.321027571467,0
-0.50857437201653,48.321056258592,0
-0.50871443541343,48.321076906792,0
-0.50885667226657,48.32108935891,0
-0.50900000000001,48.321093520173,0
-0.50914332773345,48.32108935891,0
-0.50928556458659,48.321076906792,0
-0.50942562798351,48.321056258592,0
-0.50956245189374,48.321027571467,0
-0.50969499494741,48.320991063755,0
-0.50982224836239,48.32094701332,0
-0.50994324362318,48.32089575543,0
-0.51005705985325,48.32083768021,0
-0.51016283082424,48.32077322967,0
-0.51025975154912,48.320702894341,0
-0.51034708440873,48.32062720954,0
-0.51042416476539,48.320546751296,0
-0.51049040602065,48.320462131968,0
-0.51054530407867,48.320373995577,0
-0.51058844118156,48.320283012912,0

-0.51061948908722,48.320189876418,0
-0.51063821156581,48.320095294931,0
-0.5106444661955,48.31999998828,0
-0.51063820544452,48.319904681806,0
-0.51061947703066,48.319810100848,0
-0.510588423556,48.319716965218,0
-0.51054528141971,48.319625983724,0
-0.51049037901675,48.319537848778,0
-0.51042413423708,48.319453231123,0
-0.51034705128356,48.319372774731,0
-0.51025971683359,48.319297091904,0
-0.51016279557316,48.31922675861,0
-0.51005702513772,48.319162310105,0
-0.50994321049799,48.319104236859,0
-0.50982221783406,48.31905298082,0
-0.5096949679435,48.319008932058,0
-0.50956242923478,48.318972425791,0
-0.50942561035794,48.318943739838,0
-0.50928555253002,48.318923092502,0
-0.50914332161216,48.318910640912,0
-0.50900000000001,48.318906479826,0
-0.50885667838786,48.318910640912,0
-0.50871444747002,48.318923092502,0
-0.50857438964207,48.318943739838,0
-0.50843757076524,48.318972425791,0
-0.50830503205651,48.319008932058,0
-0.50817778216599,48.31905298082,0
-0.50805678950203,48.319104236859,0
-0.50794297486232,48.319162310105,0
-0.50783720442686,48.31922675861,0
-0.50774028316645,48.319297091904,0
-0.50765294871648,48.319372774731,0
-0.50757586576296,48.319453231123,0
-0.50750962098327,48.319537848778,0
-0.50745471858033,48.319625983724,0
-0.50741157644402,48.319716965218,0
-0.50738052296939,48.319810100848,0
-0.5073617945555,48.319904681806,0
-0.50735553380452,48.31999998828,0
-0.50900000000001,48.32,0
</coordinates>
</LinearRing>
</outerBoundaryIs>
</Polygon>
</Placemark>
<Placemark>
<name>430</name>
<styleUrl>#stylehap1</styleUrl>
<Polygon>
<outerBoundaryIs>
<LinearRing>
<coordinates>
-0.50900000000001,48.319,0
-0.50735556603998,48.31899998828,0
-0.50736182054717,48.319095294932,0
-0.50738054265885,48.319189876419,0
-0.50741158995601,48.319283012912,0

-0.50745472621341,48.319373995577,0
-0.50750962319536,48.319462131968,0
-0.50757586315218,48.319546751297,0
-0.50765294199793,48.31962720954,0
-0.50774027314564,48.319702894341,0
-0.50783719197066,48.31977322967,0
-0.50794296086829,48.31983768021,0
-0.50805677486726,48.31989575543,0
-0.50817776775622,48.31994701332,0
-0.50830501867666,48.319991063755,0
-0.5084375591321,48.320027571467,0
-0.50857438036017,48.320056258592,0
-0.50871444101138,48.320076906792,0
-0.50885667507624,48.32008935891,0
-0.50900000000001,48.320093520174,0
-0.50914332492378,48.32008935891,0
-0.50928555898863,48.320076906792,0
-0.50942561963987,48.320056258592,0
-0.50956244086795,48.320027571467,0
-0.50969498132338,48.319991063755,0
-0.50982223224383,48.31994701332,0
-0.50994322513279,48.31989575543,0
-0.51005703913175,48.31983768021,0
-0.51016280802938,48.31977322967,0
-0.51025972685438,48.319702894341,0
-0.51034705800209,48.31962720954,0
-0.51042413684786,48.319546751297,0
-0.51049037680466,48.319462131968,0
-0.51054527378664,48.319373995577,0
-0.51058841004401,48.319283012912,0
-0.51061945734117,48.319189876419,0
-0.51063817945285,48.319095294932,0
-0.51064443396004,48.31899998828,0
-0.51063817333191,48.318904681806,0
-0.51061944528526,48.318810100848,0
-0.51058839241944,48.318716965218,0
-0.5105452511289,48.318625983725,0
-0.51049034980226,48.318537848778,0
-0.51042410632118,48.318453231123,0
-0.51034702487872,48.318372774732,0
-0.51025969214073,48.318297091904,0
-0.51016277278021,48.31822675861,0
-0.51005700441811,48.318162310105,0
-0.5099431920094,48.318104236859,0
-0.50982220171717,48.31805298082,0
-0.50969495432096,48.318008932058,0
-0.50956241821021,48.317972425791,0
-0.5094256020153,48.317943739838,0
-0.5092855469327,48.317923092502,0
-0.50914331880284,48.317910640912,0
-0.50900000000001,48.317906479826,0
-0.50885668119721,48.317910640912,0
-0.50871445306731,48.317923092502,0
-0.50857439798475,48.317943739838,0
-0.50843758178981,48.317972425791,0
-0.50830504567906,48.318008932058,0
-0.50817779828288,48.31805298082,0

-0.50805680799062,48.318104236859,0
-0.50794299558191,48.318162310105,0
-0.50783722721981,48.31822675861,0
-0.50774030785929,48.318297091904,0
-0.50765297512132,48.318372774732,0
-0.50757589367884,48.318453231123,0
-0.50750965019778,48.318537848778,0
-0.50745474887112,48.318625983725,0
-0.50741160758061,48.318716965218,0
-0.50738055471478,48.318810100848,0
-0.50736182666814,48.318904681806,0
-0.50735556603998,48.31899998828,0
-0.50900000000001,48.319,0
</coordinates>
</LinearRing>
</outerBoundaryIs>
</Polygon>
</Placemark>
<Placemark>
<name>431</name>
<styleUrl>#stylehap1</styleUrl>
<Polygon>
<outerBoundaryIs>
<LinearRing>
<coordinates>
-0.50900000000001,48.319,0
-0.50735556603998,48.31899998828,0
-0.50736182054717,48.319095294932,0
-0.50738054265885,48.319189876419,0
-0.50741158995601,48.319283012912,0
-0.50745472621341,48.319373995577,0
-0.50750962319536,48.319462131968,0
-0.50757586315218,48.319546751297,0
-0.50765294199793,48.31962720954,0
-0.50774027314564,48.319702894341,0
-0.50783719197066,48.31977322967,0
-0.50794296086829,48.31983768021,0
-0.50805677486726,48.31989575543,0
-0.50817776775622,48.31994701332,0
-0.50830501867666,48.319991063755,0
-0.5084375591321,48.320027571467,0
-0.50857438036017,48.320056258592,0
-0.50871444101138,48.320076906792,0
-0.50885667507624,48.32008935891,0
-0.50900000000001,48.320093520174,0
-0.50914332492378,48.32008935891,0
-0.50928555898863,48.320076906792,0
-0.50942561963987,48.320056258592,0
-0.50956244086795,48.320027571467,0
-0.50969498132338,48.319991063755,0
-0.50982223224383,48.31994701332,0
-0.50994322513279,48.31989575543,0
-0.51005703913175,48.31983768021,0
-0.51016280802938,48.31977322967,0
-0.51025972685438,48.319702894341,0
-0.51034705800209,48.31962720954,0
-0.51042413684786,48.319546751297,0

-0.51049037680466,48.319462131968,0
-0.51054527378664,48.319373995577,0
-0.51058841004401,48.319283012912,0
-0.51061945734117,48.319189876419,0
-0.51063817945285,48.319095294932,0
-0.51064443396004,48.31899998828,0
-0.51063817333191,48.318904681806,0
-0.51061944528526,48.318810100848,0
-0.51058839241944,48.318716965218,0
-0.5105452511289,48.318625983725,0
-0.51049034980226,48.318537848778,0
-0.51042410632118,48.318453231123,0
-0.51034702487872,48.318372774732,0
-0.51025969214073,48.318297091904,0
-0.51016277278021,48.31822675861,0
-0.51005700441811,48.318162310105,0
-0.5099431920094,48.318104236859,0
-0.50982220171717,48.31805298082,0
-0.50969495432096,48.318008932058,0
-0.50956241821021,48.317972425791,0
-0.5094256020153,48.317943739838,0
-0.5092855469327,48.317923092502,0
-0.50914331880284,48.317910640912,0
-0.50900000000001,48.317906479826,0
-0.50885668119721,48.317910640912,0
-0.50871445306731,48.317923092502,0
-0.50857439798475,48.317943739838,0
-0.50843758178981,48.317972425791,0
-0.50830504567906,48.318008932058,0
-0.50817779828288,48.31805298082,0
-0.50805680799062,48.318104236859,0
-0.50794299558191,48.318162310105,0
-0.50783722721981,48.31822675861,0
-0.50774030785929,48.318297091904,0
-0.50765297512132,48.318372774732,0
-0.50757589367884,48.318453231123,0
-0.50750965019778,48.318537848778,0
-0.50745474887112,48.318625983725,0
-0.50741160758061,48.318716965218,0
-0.50738055471478,48.318810100848,0
-0.50736182666814,48.318904681806,0
-0.50735556603998,48.31899998828,0
-0.50900000000001,48.319,0
</coordinates>
</LinearRing>
</outerBoundaryIs>
</Polygon>
</Placemark>
<Placemark>
<name>432</name>
<styleUrl>#stylehap1</styleUrl>
<Polygon>
<outerBoundaryIs>
<LinearRing>
<coordinates>
-0.50900000000001,48.101,0
-0.50736255145678,48.100999988369,0

-0.50736877941861,48.101095295021,0
-0.50738742202286,48.101189876505,0
-0.50741833745488,48.101283012996,0
-0.5074612904922,48.101373995656,0
-0.5075159542929,48.101462132041,0
-0.50758191288135,48.101546751364,0
-0.50765866431255,48.1016272096,0
-0.50774562449095,48.101702894393,0
-0.50784213161498,48.101773229715,0
-0.50794745121322,48.101837680247,0
-0.50806078173389,48.101895755459,0
-0.50818126064539,48.101947013342,0
-0.50830797100128,48.101991063771,0
-0.50843994841953,48.102027571477,0
-0.50857618842345,48.102056258598,0
-0.50871565408769,48.102076906794,0
-0.50885728393168,48.102089358911,0
-0.50900000000001,48.102093520174,0
-0.50914271606834,48.102089358911,0
-0.50928434591233,48.102076906794,0
-0.50942381157659,48.102056258598,0
-0.50956005158052,48.102027571477,0
-0.50969202899877,48.101991063771,0
-0.50981873935463,48.101947013342,0
-0.50993921826616,48.101895755459,0
-0.51005254878682,48.101837680247,0
-0.51015786838504,48.101773229715,0
-0.51025437550909,48.101702894393,0
-0.51034133568749,48.1016272096,0
-0.51041808711867,48.101546751364,0
-0.51048404570711,48.101462132041,0
-0.51053870950784,48.101373995656,0
-0.51058166254514,48.101283012996,0
-0.51061257797716,48.101189876505,0
-0.51063122058144,48.101095295021,0
-0.51063744854326,48.100999988369,0
-0.51063121453299,48.100904681895,0
-0.51061256606402,48.100810100935,0
-0.51058164512931,48.100716965301,0
-0.51053868711847,48.100625983804,0
-0.51048401902453,48.100537848852,0
-0.51041805695355,48.10045323119,0
-0.51034130295643,48.100372774792,0
-0.51025434120658,48.100297091957,0
-0.51015783355336,48.100226758655,0
-0.51005251448431,48.100162310142,0
-0.50993918553509,48.100104236888,0
-0.50981870918954,48.100052980843,0
-0.50969200231615,48.100008932074,0
-0.50956002919114,48.099972425802,0
-0.50942379416074,48.099943739844,0
-0.50928433399922,48.099923092504,0
-0.50914271001989,48.099910640913,0
-0.50900000000001,48.099906479827,0
-0.50885728998013,48.099910640913,0
-0.50871566600082,48.099923092504,0
-0.50857620583928,48.099943739844,0

-0.50843997080888,48.099972425802,0
-0.50830799768386,48.100008932074,0
-0.50818129081051,48.100052980843,0
-0.50806081446495,48.100104236888,0
-0.50794748551571,48.100162310142,0
-0.50784216644666,48.100226758655,0
-0.50774565879344,48.100297091957,0
-0.50765869704362,48.100372774792,0
-0.50758194304647,48.10045323119,0
-0.50751598097552,48.100537848852,0
-0.50746131288155,48.100625983804,0
-0.50741835487071,48.100716965301,0
-0.50738743393599,48.100810100935,0
-0.50736878546706,48.100904681895,0
-0.50736255145678,48.100999988369,0
-0.50900000000001,48.101,0
</coordinates>
</LinearRing>
</outerBoundaryIs>
</Polygon>
</Placemark>
<Placemark>
<name>433</name>
<styleUrl>#stylehap1</styleUrl>
<Polygon>
<outerBoundaryIs>
<LinearRing>
<coordinates>
-0.50900000000001,48.238,0
-0.50735817126224,48.237999988313,0
-0.50736441586933,48.238095294965,0
-0.5073831083286,48.238189876451,0
-0.50741410644647,48.238283012943,0
-0.50745717437164,48.238373995607,0
-0.50751198438829,48.238462131995,0
-0.50757811940834,48.238546751322,0
-0.50765507614432,48.238627209562,0
-0.50774226893844,48.23870289436,0
-0.50783903421892,48.238773229687,0
-0.50794463554964,48.238837680224,0
-0.50805826923459,48.238895755441,0
-0.50817907043485,48.238947013328,0
-0.50830611975085,48.238991063761,0
-0.50843845022067,48.239027571471,0
-0.50857505468028,48.239056258594,0
-0.50871489343015,48.239076906793,0
-0.50885690214986,48.23908935891,0
-0.50900000000001,48.239093520174,0
-0.50914309785016,48.23908935891,0
-0.50928510656987,48.239076906793,0
-0.50942494531976,48.239056258594,0
-0.50956154977935,48.239027571471,0
-0.50969388024917,48.238991063761,0
-0.50982092956519,48.238947013328,0
-0.50994173076543,48.238895755441,0
-0.51005536445041,48.238837680224,0
-0.51016096578109,48.238773229687,0

-0.51025773106158,48.23870289436,0
-0.5103449238557,48.238627209562,0
-0.51042188059168,48.238546751322,0
-0.51048801561173,48.238462131995,0
-0.51054282562838,48.238373995607,0
-0.51058589355357,48.238283012943,0
-0.51061689167142,48.238189876451,0
-0.51063558413071,48.238095294965,0
-0.51064182873778,48.237999988313,0
-0.51063557803682,48.237904681839,0
-0.51061687966882,48.23781010088,0
-0.51058587600691,48.237716965249,0
-0.51054280307085,48.237625983754,0
-0.51048798872871,48.237537848806,0
-0.5104218502,48.237453231148,0
-0.51034489087879,48.237372774754,0
-0.51025769650144,48.237297091924,0
-0.51016093068782,48.237226758627,0
-0.51005532989024,48.237162310119,0
-0.50994169778852,48.23710423687,0
-0.50982089917351,48.237052980829,0
-0.50969385336615,48.237008932064,0
-0.50956152722181,48.236972425795,0
-0.5094249277731,48.23694373984,0
-0.50928509456727,48.236923092503,0
-0.50914309175627,48.236910640912,0
-0.50900000000001,48.236906479827,0
-0.50885690824375,48.236910640912,0
-0.50871490543277,48.236923092503,0
-0.50857507222692,48.23694373984,0
-0.50843847277821,48.236972425795,0
-0.50830614663389,48.237008932064,0
-0.50817910082653,48.237052980829,0
-0.5080583022115,48.23710423687,0
-0.50794467010977,48.237162310119,0
-0.50783906931222,48.237226758627,0
-0.5077423034986,48.237297091924,0
-0.50765510912123,48.237372774754,0
-0.50757814980004,48.237453231148,0
-0.5075120112713,48.237537848806,0
-0.50745719692917,48.237625983754,0
-0.50741412399311,48.237716965249,0
-0.50738312033122,48.23781010088,0
-0.50736442196323,48.237904681839,0
-0.50735817126224,48.237999988313,0
-0.50900000000001,48.238,0
</coordinates>
</LinearRing>
</outerBoundaryIs>
</Polygon>
</Placemark>
<Placemark>
<name>435</name>
<styleUrl>#stylehap1</styleUrl>
<Polygon>
<outerBoundaryIs>
<LinearRing>

<coordinates>
-0.50900000000001,48.1,0
-0.50736258330869,48.09999998837,0
-0.50736881114947,48.100095295021,0
-0.50738745339122,48.100189876506,0
-0.50741836822194,48.100283012996,0
-0.50746132042382,48.100373995657,0
-0.50751598316127,48.100462132042,0
-0.50758194046676,48.100546751364,0
-0.50765869040501,48.1006272096,0
-0.50774564889186,48.100702894393,0
-0.50784215413865,48.100773229715,0
-0.50794747168819,48.100837680247,0
-0.50806080000429,48.100895755459,0
-0.50818127657218,48.100947013342,0
-0.50830798446319,48.100991063771,0
-0.50843995931412,48.101027571477,0
-0.5085761966678,48.101056258598,0
-0.50871565961905,48.101076906794,0
-0.50885728670792,48.101089358911,0
-0.50900000000001,48.101093520174,0
-0.50914271329209,48.101089358911,0
-0.50928434038099,48.101076906794,0
-0.50942380333224,48.101056258598,0
-0.50956004068589,48.101027571477,0
-0.50969201553683,48.100991063771,0
-0.50981872342786,48.100947013342,0
-0.50993919999575,48.100895755459,0
-0.51005252831186,48.100837680247,0
-0.51015784586137,48.100773229715,0
-0.51025435110815,48.100702894393,0
-0.51034130959503,48.1006272096,0
-0.51041805953328,48.100546751364,0
-0.51048401683875,48.100462132042,0
-0.5105386795762,48.100373995657,0
-0.51058163177808,48.100283012996,0
-0.51061254660883,48.100189876506,0
-0.51063118885054,48.100095295021,0
-0.51063741669133,48.09999998837,0
-0.51063118280243,48.099904681895,0
-0.51061253469635,48.099810100935,0
-0.51058161436319,48.099716965302,0
-0.51053865718807,48.099625983804,0
-0.51048399015761,48.099537848852,0
-0.51041802936982,48.099453231191,0
-0.51034127686575,48.099372774792,0
-0.51025431680755,48.099297091957,0
-0.51015781103163,48.099226758655,0
-0.51005249401123,48.099162310142,0
-0.50993916726647,48.099104236888,0
-0.5098186932644,48.099052980843,0
-0.50969198885569,48.099008932074,0
-0.50956001829777,48.098972425802,0
-0.50942378591735,48.098943739844,0
-0.50928432846851,48.098923092504,0
-0.50914270724398,48.098910640913,0
-0.50900000000001,48.098906479827,0

-0.50885729275604,48.098910640913,0
-0.50871567153153,48.098923092504,0
-0.50857621408269,48.098943739844,0
-0.50843998170228,48.098972425802,0
-0.50830801114435,48.099008932074,0
-0.50818130673565,48.099052980843,0
-0.50806083273357,48.099104236888,0
-0.50794750598879,48.099162310142,0
-0.50784218896842,48.099226758655,0
-0.50774568319249,48.099297091957,0
-0.50765872313427,48.099372774792,0
-0.50758197063022,48.099453231191,0
-0.50751600984241,48.099537848852,0
-0.50746134281197,48.099625983804,0
-0.50741838563682,48.099716965302,0
-0.50738746530369,48.099810100935,0
-0.50736881719759,48.099904681895,0
-0.50736258330869,48.09999998837,0
-0.50900000000001,48.1,0
</coordinates>
</LinearRing>
</outerBoundaryIs>
</Polygon>
</Placemark>
<Placemark>
<name>436</name>
<styleUrl>#stylehap1</styleUrl>
<Polygon>
<outerBoundaryIs>
<LinearRing>
<coordinates>
-0.50800000000004,48.15,0
-0.50636098858131,48.149999988349,0
-0.50636722248225,48.150095295001,0
-0.50638588287503,48.150189876486,0
-0.50641682780978,48.150283012977,0
-0.50645982183967,48.150373995639,0
-0.50651453781096,48.150462132025,0
-0.50658055935111,48.150546751349,0
-0.50665738403608,48.150627209586,0
-0.50674442721294,48.150702894381,0
-0.50684102644852,48.150773229705,0
-0.50694644657031,48.150837680238,0
-0.50705988526151,48.150895755453,0
-0.50718047916727,48.150947013337,0
-0.50730731046583,48.150991063768,0
-0.50743941385466,48.151027571475,0
-0.50757578389821,48.151056258597,0
-0.50771538268128,48.151076906794,0
-0.50785714771004,48.151089358911,0
-0.50800000000005,48.151093520174,0
-0.50814285229003,48.151089358911,0
-0.5082846173188,48.151076906794,0
-0.50842421610187,48.151056258597,0
-0.50856058614541,48.151027571475,0
-0.50869268953424,48.150991063768,0
-0.50881952083281,48.150947013337,0

-0.50894011473856,48.150895755453,0
-0.50905355342976,48.150837680238,0
-0.50915897355158,48.150773229705,0
-0.50925557278714,48.150702894381,0
-0.50934261596399,48.150627209586,0
-0.50941944064897,48.150546751349,0
-0.50948546218912,48.150462132025,0
-0.50954017816041,48.150373995639,0
-0.50958317219029,48.150283012977,0
-0.50961411712504,48.150189876486,0
-0.50963277751783,48.150095295001,0
-0.50963901141877,48.149999988349,0
-0.50963277145317,48.149904681875,0
-0.50961410518,48.149810100915,0
-0.50958315472783,48.149716965283,0
-0.50954015571108,48.149625983786,0
-0.50948543543503,48.149537848835,0
-0.50941941040306,48.149453231175,0
-0.50934258314527,48.149372774778,0
-0.50925553839277,48.149297091945,0
-0.50915893862662,48.149226758645,0
-0.5090535190354,48.149162310134,0
-0.50894008191984,48.149104236882,0
-0.5088194905869,48.149052980838,0
-0.50869266278018,48.149008932071,0
-0.50856056369609,48.148972425799,0
-0.5084241986394,48.148943739842,0
-0.50828460537375,48.148923092504,0
-0.50814284622538,48.148910640912,0
-0.50800000000005,48.148906479826,0
-0.5078571537747,48.148910640912,0
-0.50771539462632,48.148923092504,0
-0.50757580136067,48.148943739842,0
-0.50743943630398,48.148972425799,0
-0.50730733721989,48.149008932071,0
-0.50718050941317,48.149052980838,0
-0.50705991808023,48.149104236882,0
-0.50694648096468,48.149162310134,0
-0.50684106137346,48.149226758645,0
-0.5067444616073,48.149297091945,0
-0.5066574168548,48.149372774778,0
-0.50658058959701,48.149453231175,0
-0.50651456456505,48.149537848835,0
-0.50645984428899,48.149625983786,0
-0.50641684527225,48.149716965283,0
-0.50638589482008,48.149810100915,0
-0.5063672285469,48.149904681875,0
-0.50636098858131,48.149999988349,0
-0.50800000000004,48.15,0
</coordinates>
</LinearRing>
</outerBoundaryIs>
</Polygon>
</Placemark>
<Placemark>
<name>437</name>
<styleUrl>#stylehap1</styleUrl>

<Polygon>
<outerBoundaryIs>
<LinearRing>
<coordinates>
-0.50800000000004,48.119,0
-0.50636197782493,48.118999988362,0
-0.50636820796663,48.119095295013,0
-0.50638685709992,48.119189876498,0
-0.50641778336052,48.119283012989,0
-0.50646075144359,48.11937399565,0
-0.50651543439278,48.119462132035,0
-0.50658141608679,48.119546751358,0
-0.50665819440477,48.119627209595,0
-0.5067451850466,48.119702894389,0
-0.50684172597893,48.119773229711,0
-0.50694708247294,48.119837680244,0
-0.50706045269611,48.119895755457,0
-0.50718097381466,48.11994701334,0
-0.50730772856086,48.11999106377,0
-0.50743975221489,48.120027571476,0
-0.50757603994805,48.120056258598,0
-0.50771555447174,48.120076906794,0
-0.50785723393343,48.120089358911,0
-0.50800000000005,48.120093520173,0
-0.50814276606665,48.120089358911,0
-0.50828444552834,48.120076906794,0
-0.50842396005203,48.120056258598,0
-0.5085602477851‍8,48.120027571476,0
-0.50869227143921,48.11999106377,0
-0.50881902618541,48.11994701334,0
-0.50893954730397,48.119895755457,0
-0.50905291752714,48.119837680244,0
-0.50915827402117,48.119773229711,0
-0.50925481495347,48.119702894389,0
-0.5093418055953,48.119627209595,0
-0.50941858391328,48.119546751358,0
-0.50948456560729,48.119462132035,0
-0.50953924855648,48.11937399565,0
-0.50958221663956,48.119283012989,0
-0.50961314290015,48.119189876498,0
-0.50963179203344,48.119095295013,0
-0.50963802217515,48.118999988362,0
-0.50963178597904,48.118904681888,0
-0.50961313097531,48.118810100928,0
-0.50958219920661,48.118716965295,0
-0.50953922614513,48.118625983797,0
-0.50948453889844,48.118537848846,0
-0.50941855371852,48.118453231185,0
-0.50934177283208,48.118372774787,0
-0.50925478061725,48.118297091952,0
-0.50915823915527,48.118226758651,0
-0.50905288319094,48.118162310139,0
-0.50893951454074,48.118104236886,0
-0.50881899599065,48.118052980841,0
-0.50869224473036,48.118008932073,0
-0.50856022537382,48.117972425801,0
-0.50842394261907,48.117943739843,0

-0.5082844336035,48.117923092504,0
-0.50814276001224,48.117910640913,0
-0.50800000000005,48.117906479826,0
-0.50785723998783,48.117910640913,0
-0.50771556639658,48.117923092504,0
-0.507576057381,48.117943739843,0
-0.50743977462625,48.117972425801,0
-0.50730775526971,48.118008932073,0
-0.50718100400942,48.118052980841,0
-0.50706048545933,48.118104236886,0
-0.50694711680914,48.118162310139,0
-0.50684176084481,48.118226758651,0
-0.50674521938283,48.118297091952,0
-0.506658227168,48.118372774787,0
-0.50658144628155,48.118453231185,0
-0.50651546110163,48.118537848846,0
-0.50646077385495,48.118625983797,0
-0.50641780079347,48.118716965295,0
-0.50638686902477,48.118810100928,0
-0.50636821402103,48.118904681888,0
-0.50636197782493,48.118999988362,0
-0.50800000000004,48.119,0
</coordinates>
</LinearRing>
</outerBoundaryIs>
</Polygon>
</Placemark>
<Placemark>
<name>438</name>
<styleUrl>#stylehap1</styleUrl>
<Polygon>
<outerBoundaryIs>
<LinearRing>
<coordinates>
-0.50700000000006,48.182,0
-0.50535996566865,48.181999988336,0
-0.50536620345677,48.182095294988,0
-0.50538487549227,48.182189876473,0
-0.50541583973675,48.182283012965,0
-0.50545886059657,48.182373995627,0
-0.50551361071386,48.182462132014,0
-0.50557967345632,48.182546751339,0
-0.5056565460864,48.182627209578,0
-0.50574364358631,48.182702894374,0
-0.50584030310949,48.182773229698,0
-0.50594578902464,48.182837680233,0
-0.50605929851422,48.182895755448,0
-0.5061799676845,48.182947013334,0
-0.50630687814089,48.182991063765,0
-0.50643906397832,48.183027571473,0
-0.50657551913366,48.183056258596,0
-0.50671520504393,48.183076906793,0
-0.50685705855204,48.183089358911,0
-0.50700000000007,48.183093520174,0
-0.50714294144809,48.183089358911,0
-0.5072847949562,48.183076906793,0
-0.50742448086645,48.183056258596,0

-0.50756093602181,48.183027571473,0
-0.50769312185924,48.182991063765,0
-0.50782003231563,48.182947013334,0
-0.50794070148591,48.182895755448,0
-0.50805421097546,48.182837680233,0
-0.50815969689064,48.182773229698,0
-0.50825635641382,48.182702894374,0
-0.50834345391373,48.182627209578,0
-0.50842032654381,48.182546751339,0
-0.50848638928627,48.182462132014,0
-0.50854113940356,48.182373995627,0
-0.50858416026336,48.182283012965,0
-0.50861512450784,48.182189876473,0
-0.50863379654334,48.182095294988,0
-0.50864003433148,48.181999988336,0
-0.5086337904681,48.181904681862,0
-0.50861511254191,48.181810100903,0
-0.50858414277033,48.181716965271,0
-0.50854111691495,48.181625983774,0
-0.50848636248539,48.181537848825,0
-0.50842029624501,48.181453231166,0
-0.50834342103761,48.181372774769,0
-0.50825632195927,48.181297091937,0
-0.50815966190459,48.181226758638,0
-0.50805417652095,48.181162310129,0
-0.50794066860976,48.181104236877,0
-0.50782000201683,48.181052980834,0
-0.50769309505839,48.181008932068,0
-0.5075609135332,48.180972425798,0
-0.50742446337342,48.180943739842,0
-0.50728478299027,48.180923092503,0
-0.50714293537283,48.180910640912,0
-0.50700000000007,48.180906479826,0
-0.5068570646273,48.180910640912,0
-0.50671521700986,48.180923092503,0
-0.50657553662668,48.180943739842,0
-0.5064390864669,48.180972425798,0
-0.50630690494174,48.181008932068,0
-0.5061799979833,48.181052980834,0
-0.50605933139037,48.181104236877,0
-0.50594582347918,48.181162310129,0
-0.50584033809554,48.181226758638,0
-0.50574367804083,48.181297091937,0
-0.50565657896252,48.181372774769,0
-0.50557970375512,48.181453231166,0
-0.50551363751472,48.181537848825,0
-0.50545888308515,48.181625983774,0
-0.50541585722977,48.181716965271,0
-0.5053848874582,48.181810100903,0
-0.50536620953203,48.181904681862,0
-0.50535996566865,48.181999988336,0
-0.50700000000006,48.182,0
</coordinates>
</LinearRing>
</outerBoundaryIs>
</Polygon>
</Placemark>

<Placemark>
<name>440</name>
<styleUrl>#stylehap1</styleUrl>
<Polygon>
<outerBoundaryIs>
<LinearRing>
<coordinates>
-0.50599999999997,48.297,0
-0.50435627477368,48.296999988289,0
-0.50436252658764,48.297095294941,0
-0.50438124063256,48.297189876427,0
-0.50441227455083,48.297283012921,0
-0.50445539221888,48.297373995585,0
-0.50451026554249,48.297462131975,0
-0.50457647695196,48.297546751303,0
-0.50465352257854,48.297627209546,0
-0.50474081608805,48.297702894346,0
-0.50483769314226,48.297773229674,0
-0.5049434164544,48.297837680214,0
-0.50505718140013,48.297895755433,0
-0.50517812214143,48.297947013322,0
-0.50530531821669,48.297991063757,0
-0.50543780154689,48.298027571468,0
-0.50557456380451,48.298056258593,0
-0.50571456408892,48.298076906792,0
-0.50585673685012,48.29808935891,0
-0.50599999999998,48.298093520173,0
-0.50614326314984,48.29808935891,0
-0.50628543591101,48.298076906792,0
-0.50642543619542,48.298056258593,0
-0.50656219845304,48.298027571468,0
-0.50669468178327,48.297991063757,0
-0.50682187785853,48.297947013322,0
-0.50694281859983,48.297895755433,0
-0.50705658354553,48.297837680214,0
-0.50716230685767,48.297773229674,0
-0.50725918391191,48.297702894346,0
-0.50734647742142,48.297627209546,0
-0.507423523048,48.297546751303,0
-0.50748973445747,48.297462131975,0
-0.50754460778108,48.297373995585,0
-0.50758772544912,48.297283012921,0
-0.50761875936737,48.297189876427,0
-0.50763747341232,48.297095294941,0
-0.50764372522625,48.296999988289,0
-0.50763746729874,48.296904681815,0
-0.50761874732597,48.296810100857,0
-0.50758770784575,48.296716965226,0
-0.50754458515062,48.296625983733,0
-0.50748970748753,48.296537848786,0
-0.50742349255808,48.29645323113,0
-0.5073464443379,48.296372774738,0
-0.50725914924002,48.296297091909,0
-0.50716227165094,48.296226758615,0
-0.50705654887367,48.296162310109,0
-0.50694278551631,48.296104236862,0
-0.50682184736861,48.296052980823,0

-0.50669465481334,48.29600893206,0
-0.5065621758226,48.295972425792,0
-0.50642541859207,48.295943739838,0
-0.50628542386961,48.295923092502,0
-0.50614325703626,48.295910640912,0
-0.50599999999998,48.295906479826,0
-0.5058567429637,48.295910640912,0
-0.50571457613035,48.295923092502,0
-0.50557458140789,48.295943739838,0
-0.50543782417736,48.295972425792,0
-0.5053053451866,48.29600893206,0
-0.50517815263134,48.296052980823,0
-0.50505721448362,48.296104236862,0
-0.50494345112626,48.296162310109,0
-0.50483772834899,48.296226758615,0
-0.50474085075991,48.296297091909,0
-0.50465355566203,48.296372774738,0
-0.50457650744187,48.29645323113,0
-0.5045102925124,48.296537848786,0
-0.50445541484932,48.296625983733,0
-0.50441229215419,48.296716965226,0
-0.50438125267397,48.296810100857,0
-0.50436253270122,48.296904681815,0
-0.50435627477368,48.296999988289,0
-0.50599999999997,48.297,0
</coordinates>
</LinearRing>
</outerBoundaryIs>
</Polygon>
</Placemark>
<Placemark>
<name>441</name>
<styleUrl>#stylehap1</styleUrl>
<Polygon>
<outerBoundaryIs>
<LinearRing>
<coordinates>
-0.50599999999997,48.28,0
-0.50435682184743,48.279999988296,0
-0.50436307158245,48.280095294948,0
-0.50438177940064,48.280189876434,0
-0.50441280299167,48.280283012927,0
-0.50445590631056,48.280373995591,0
-0.50451076137221,48.280462131981,0
-0.50457695074588,48.280546751309,0
-0.5046539707305,48.280627209551,0
-0.50474123518697,48.28070289435,0
-0.50483807999819,48.280773229678,0
-0.50494376812276,48.280837680216,0
-0.50505749520411,48.280895755435,0
-0.50517839569246,48.280947013324,0
-0.50530554943258,48.280991063758,0
-0.50543798866765,48.281027571469,0
-0.50557470540581,48.281056258593,0
-0.50571465909288,48.281076906792,0
-0.50585678453358,48.28108935891,0
-0.50599999999998,48.281093520174,0

-0.50614321546638,48.28108935891,0
-0.50628534090705,48.281076906792,0
-0.50642529459412,48.281056258593,0
-0.50656201133228,48.281027571469,0
-0.50669445056738,48.280991063758,0
-0.50682160430747,48.280947013324,0
-0.50694250479582,48.280895755435,0
-0.5070562318772,48.280837680216,0
-0.50716192000174,48.280773229678,0
-0.50725876481296,48.28070289435,0
-0.50734602926946,48.280627209551,0
-0.50742304925405,48.280546751309,0
-0.50748923862772,48.280462131981,0
-0.50754409368937,48.280373995591,0
-0.50758719700828,48.280283012927,0
-0.50761822059932,48.280189876434,0
-0.50763692841751,48.280095294948,0
-0.5076431781525,48.279999988296,0
-0.5076369223096,48.279904681822,0
-0.50761820856909,48.279810100864,0
-0.50758717942127,48.279716965233,0
-0.50754407107998,48.279625983739,0
-0.50748921168287,48.279537848792,0
-0.50742301879248,48.279453231135,0
-0.5073459962167,48.279372774742,0
-0.50725873017334,48.279297091913,0
-0.50716188482776,48.279226758618,0
-0.50705619723758,48.279162310112,0
-0.50694247174309,48.279104236864,0
-0.5068215738459,48.279052980824,0
-0.50669442362253,48.279008932061,0
-0.50656198872286,48.278972425793,0
-0.50642527700713,48.278943739839,0
-0.50628532887685,48.278923092502,0
-0.50614320935847,48.278910640912,0
-0.50599999999998,48.278906479826,0
-0.50585679064146,48.278910640912,0
-0.50571467112311,48.278923092502,0
-0.5055747229928,48.278943739839,0
-0.50543801127707,48.278972425793,0
-0.50530557637743,48.279008932061,0
-0.50517842615403,48.279052980824,0
-0.50505752825687,48.279104236864,0
-0.50494380276238,48.279162310112,0
-0.5048381151722,48.279226758618,0
-0.50474126982659,48.279297091913,0
-0.50465400378323,48.279372774742,0
-0.50457698120745,48.279453231135,0
-0.50451078831706,48.279537848792,0
-0.50445592891998,48.279625983739,0
-0.50441282057866,48.279716965233,0
-0.50438179143085,48.279810100864,0
-0.50436307769033,48.279904681822,0
-0.50435682184743,48.279999988296,0
-0.50599999999997,48.28,0
</coordinates>
</LinearRing>

</outerBoundaryIs>
</Polygon>
</Placemark>
<Placemark>
<name>442</name>
<styleUrl>#stylehap1</styleUrl>
<Polygon>
<outerBoundaryIs>
<LinearRing>
<coordinates>
-0.50599999999997,48.255,0
-0.50435762544391,48.254999988306,0
-0.50436387212516,48.255095294958,0
-0.50438257079689,48.255189876444,0
-0.50441357921826,48.255283012937,0
-0.50445666145968,48.255373995601,0
-0.50451148969636,48.25546213199,0
-0.50457764670165,48.255546751316,0
-0.50465462902077,48.255627209557,0
-0.50474185080124,48.255702894356,0
-0.50483864825075,48.255773229683,0
-0.50494428468827,48.255837680221,0
-0.5050579561507,48.255895755438,0
-0.5051787975115,48.255947013326,0
-0.50530588906552,48.25599106376,0
-0.50543826352928,48.25602757147,0
-0.50557491340391,48.256056258594,0
-0.50571479864417,48.256076906792,0
-0.50585685457578,48.25608935891,0
-0.50599999999998,48.256093520174,0
-0.50614314542416,48.25608935891,0
-0.50628520135576,48.256076906792,0
-0.50642508659605,48.256056258594,0
-0.50656173647065,48.25602757147,0
-0.50669411093441,48.25599106376,0
-0.50682120248843,48.255947013326,0
-0.50694204384925,48.255895755438,0
-0.50705571531169,48.255837680221,0
-0.50716135174921,48.255773229683,0
-0.50725814919869,48.255702894356,0
-0.50734537097916,48.255627209557,0
-0.50742235329828,48.255546751316,0
-0.50748851030357,48.25546213199,0
-0.50754333854027,48.255373995601,0
-0.50758642078168,48.255283012937,0
-0.50761742920307,48.255189876444,0
-0.5076361278748,48.255095294958,0
-0.50764237455605,48.254999988306,0
-0.50763612177523,48.254904681832,0
-0.5076174171893,48.254810100874,0
-0.5075864032187,48.254716965243,0
-0.50754331596175,48.254625983748,0
-0.50748848339554,48.2545378488,0
-0.50742232287834,48.254453231143,0
-0.5073453379716,48.254372774749,0
-0.50725811460639,48.254297091919,0
-0.50716131662327,48.254226758623,0

-0.50705568071939,48.254162310116,0
-0.50694201084166,48.254104236867,0
-0.50682117206849,48.254052980827,0
-0.50669408402639,48.254008932063,0
-0.50656171389215,48.253972425794,0
-0.50642506903308,48.25394373984,0
-0.50628518934199,48.253923092502,0
-0.50614313932461,48.253910640912,0
-0.50599999999998,48.253906479826,0
-0.50585686067534,48.253910640912,0
-0.50571481065794,48.253923092502,0
-0.50557493096685,48.25394373984,0
-0.50543828610781,48.253972425794,0
-0.50530591597354,48.254008932063,0
-0.50517882793147,48.254052980827,0
-0.50505798915827,48.254104236867,0
-0.50494431928057,48.254162310116,0
-0.50483868337667,48.254226758623,0
-0.50474188539354,48.254297091919,0
-0.50465466202833,48.254372774749,0
-0.50457767712159,48.254453231143,0
-0.50451151660439,48.2545378488,0
-0.50445668403819,48.254625983748,0
-0.50441359678123,48.254716965243,0
-0.50438258281066,48.254810100874,0
-0.5043638782247,48.254904681832,0
-0.50435762544391,48.254999988306,0
-0.50599999999997,48.255,0
</coordinates>
</LinearRing>
</outerBoundaryIs>
</Polygon>
</Placemark>
<Placemark>
<name>443</name>
<styleUrl>#stylehap1</styleUrl>
<Polygon>
<outerBoundaryIs>
<LinearRing>
<coordinates>
-0.505,48.297,0
-0.50335627477372,48.296999988289,0
-0.50336252658765,48.297095294941,0
-0.50338124063258,48.297189876427,0
-0.50341227455085,48.297283012921,0
-0.5034553922189,48.297373995585,0
-0.50351026554251,48.297462131975,0
-0.50357647695197,48.297546751303,0
-0.50365352257855,48.297627209546,0
-0.50374081608807,48.297702894346,0
-0.5038376931423,48.297773229674,0
-0.50394341645441,48.297837680214,0
-0.50405718140014,48.297895755433,0
-0.50417812214144,48.297947013322,0
-0.5043053182167,48.297991063757,0
-0.50443780154694,48.298027571468,0
-0.50457456380455,48.298056258593,0

-0.50471456408896,48.298076906792,0
-0.50485673685013,48.29808935891,0
-0.50499999999999,48.298093520173,0
-0.50514326314986,48.29808935891,0
-0.50528543591103,48.298076906792,0
-0.50542543619544,48.298056258593,0
-0.50556219845308,48.298027571468,0
-0.50569468178328,48.297991063757,0
-0.50582187785855,48.297947013322,0
-0.50594281859984,48.297895755433,0
-0.50605658354557,48.297837680214,0
-0.50616230685771,48.297773229674,0
-0.50625918391192,48.297702894346,0
-0.50634647742143,48.297627209546,0
-0.50642352304802,48.297546751303,0
-0.50648973445748,48.297462131975,0
-0.50654460778109,48.297373995585,0
-0.50658772544914,48.297283012921,0
-0.50661875936741,48.297189876427,0
-0.50663747341234,48.297095294941,0
-0.50664372522629,48.296999988289,0
-0.50663746729875,48.296904681815,0
-0.50661874732598,48.296810100857,0
-0.50658770784579,48.296716965226,0
-0.50654458515063,48.296625983733,0
-0.50648970748755,48.296537848786,0
-0.5064234925581,48.29645323113,0
-0.50634644433791,48.296372774738,0
-0.50625914924006,48.296297091909,0
-0.50616227165095,48.296226758615,0
-0.50605654887371,48.296162310109,0
-0.50594278551633,48.296104236862,0
-0.50582184736863,48.296052980823,0
-0.50569465481335,48.29600893206,0
-0.50556217582262,48.295972425792,0
-0.50542541859208,48.295943739838,0
-0.50528542386962,48.295923092502,0
-0.50514325703627,48.295910640912,0
-0.50499999999999,48.295906479826,0
-0.50485674296372,48.295910640912,0
-0.50471457613036,48.295923092502,0
-0.5045745814079,48.295943739838,0
-0.50443782417737,48.295972425792,0
-0.50430534518664,48.29600893206,0
-0.50417815263136,48.296052980823,0
-0.50405721448366,48.296104236862,0
-0.5039434511263,48.296162310109,0
-0.50383772834903,48.296226758615,0
-0.50374085075993,48.296297091909,0
-0.50365355566207,48.296372774738,0
-0.50357650744189,48.29645323113,0
-0.50351029251244,48.296537848786,0
-0.50345541484936,48.296625983733,0
-0.5034122921542,48.296716965226,0
-0.50338125267401,48.296810100857,0
-0.50336253270124,48.296904681815,0
-0.50335627477372,48.296999988289,0

-0.505,48.297,0
</coordinates>
</LinearRing>
</outerBoundaryIs>
</Polygon>
</Placemark>
<Placemark>
<name>444</name>
<styleUrl>#stylehap1</styleUrl>
<Polygon>
<outerBoundaryIs>
<LinearRing>
<coordinates>
-0.50400000000002,48.102,0
-0.50236251960312,48.101999988369,0
-0.50236874768598,48.10209529502,0
-0.50238739065282,48.102189876505,0
-0.50241830668614,48.102283012995,0
-0.50246126055893,48.102373995656,0
-0.50251592542296,48.102462132041,0
-0.50258188529447,48.102546751363,0
-0.50265863821867,48.1026272096,0
-0.5027456000887,48.102702894393,0
-0.50284210909011,48.102773229714,0
-0.50294743073714,48.102837680247,0
-0.50306076346249,48.102895755459,0
-0.50318124471776,48.102947013342,0
-0.5033079575386,48.102991063771,0
-0.50343993752432,48.103027571477,0
-0.50357618017864,48.103056258598,0
-0.50371564855604,48.103076906794,0
-0.50385728115529,48.103089358911,0
-0.50400000000001,48.103093520174,0
-0.50414271884473,48.103089358911,0
-0.504284351444,48.103076906794,0
-0.50442381982141,48.103056258598,0
-0.50456006247572,48.103027571477,0
-0.50469204246145,48.102991063771,0
-0.50481875528229,48.102947013342,0
-0.50493923653755,48.102895755459,0
-0.50505256926287,48.102837680247,0
-0.50515789090993,48.102773229714,0
-0.50525439991135,48.102702894393,0
-0.50534136178137,48.1026272096,0
-0.50541811470558,48.102546751363,0
-0.50548407457709,48.102462132041,0
-0.50553873944109,48.102373995656,0
-0.5055816933139,48.102283012995,0
-0.50561260934723,48.102189876505,0
-0.50563125231403,48.10209529502,0
-0.50563748039693,48.101999988369,0
-0.50563124626526,48.101904681894,0
-0.50561259743343,48.101810100934,0
-0.50558167589711,48.101716965301,0
-0.50553871705052,48.101625983803,0
-0.50548404789302,48.101537848852,0
-0.50541808453881,48.10145323119,0

-0.50534132904853,48.101372774791,0
-0.50525436560698,48.101297091956,0
-0.50515785607637,48.101226758655,0
-0.50505253495853,48.101162310142,0
-0.50493920380471,48.101104236888,0
-0.50481872511554,48.101052980843,0
-0.50469201577738,48.101008932074,0
-0.50456004008513,48.100972425802,0
-0.50442380240461,48.100943739844,0
-0.5042843395302,48.100923092504,0
-0.50414271279595,48.100910640913,0
-0.50400000000001,48.100906479826,0
-0.50385728720409,48.100910640913,0
-0.50371566046981,48.100923092504,0
-0.50357619759543,48.100943739844,0
-0.50343995991492,48.100972425802,0
-0.50330798422266,48.101008932074,0
-0.5031812748845,48.101052980843,0
-0.50306079619533,48.101104236888,0
-0.50294746504151,48.101162310142,0
-0.50284214392367,48.101226758655,0
-0.50274563439306,48.101297091956,0
-0.50265867095152,48.101372774791,0
-0.50258191546121,48.10145323119,0
-0.50251595210702,48.101537848852,0
-0.50246128294953,48.101625983803,0
-0.50241832410291,48.101716965301,0
-0.50238740256659,48.101810100934,0
-0.50236875373479,48.101904681894,0
-0.50236251960312,48.101999988369,0
-0.50400000000002,48.102,0
</coordinates>
</LinearRing>
</outerBoundaryIs>
</Polygon>
</Placemark>
<Placemark>
<name>445</name>
<styleUrl>#stylehap1</styleUrl>
<Polygon>
<outerBoundaryIs>
<LinearRing>
<coordinates>
-0.50299999999993,48.241,0
-0.50135807497816,48.240999988312,0
-0.50136431995112,48.241095294964,0
-0.50138301350631,48.24118987645,0
-0.50141401344172,48.241283012942,0
-0.50145708389233,48.241373995606,0
-0.50151189712304,48.241462131994,0
-0.50157803602136,48.241546751321,0
-0.50165499727029,48.241627209561,0
-0.5017421951777,48.24170289436,0
-0.50183896613288,48.241773229686,0
-0.50194457365654,48.241837680223,0
-0.50205821400557,48.24189575544,0
-0.50217902229025,48.241947013328,0

-0.50230607905719,48.241991063761,0
-0.50243841728767,48.24202757147,0
-0.50257502975862,48.242056258594,0
-0.50271487670953,48.242076906792,0
-0.50285689375758,48.24208935891,0
-0.50299999999992,48.242093520174,0
-0.50314310624229,48.24208935891,0
-0.50328512329032,48.242076906792,0
-0.50342497024125,48.242056258594,0
-0.50356158271217,48.24202757147,0
-0.50369392094268,48.241991063761,0
-0.50382097770959,48.241947013328,0
-0.5039417859943,48.2418957544,0
-0.50405542634333,48.241837680223,0
-0.50416103386696,48.241773229686,0
-0.50425780482217,48.24170289436,0
-0.50434500272958,48.241627209561,0
-0.50442196397849,48.241546751321,0
-0.5044881028768,48.241462131994,0
-0.50454291610752,48.241373995606,0
-0.50458598655812,48.241283012942,0
-0.50461698649356,48.24118987645,0
-0.50463568004875,48.241095294964,0
-0.50464192502171,48.240999988312,0
-0.50463567395386,48.240904681838,0
-0.50461697448898,48.240810100879,0
-0.50458596900861,48.240716965248,0
-0.50454289354629,48.240625983753,0
-0.50448807598939,48.240537848805,0
-0.50442193358182,48.240453231147,0
-0.50434496974725,48.240372774753,0
-0.50425777025636,48.240297091923,0
-0.50416099876791,48.240226758626,0
-0.50405539177752,48.240162310119,0
-0.50394175301197,48.240104236869,0
-0.50382094731293,48.240052980828,0
-0.50369389405524,48.240008932064,0
-0.50356156015095,48.239972425795,0
-0.50342495269172,48.23994373984,0
-0.50328511128573,48.239923092503,0
-0.5031431001474,48.239910640912,0
-0.50299999999992,48.239906479826,0
-0.50285689985247,48.239910640912,0
-0.50271488871411,48.239923092503,0
-0.50257504730813,48.23994373984,0
-0.5024384398489,48.239972425795,0
-0.50230610594461,48.240008932064,0
-0.50217905268692,48.240052980828,0
-0.50205824698787,48.240104236869,0
-0.50194460822235,48.240162310119,0
-0.50183900123193,48.240226758626,0
-0.50174222974351,48.240297091923,0
-0.5016550302526,48.240372774753,0
-0.50157806641805,48.240453231147,0
-0.50151192401049,48.240537848805,0
-0.50145710645358,48.240625983753,0
-0.50141403099126,48.240716965248,0

-0.50138302551087,48.240810100879,0
-0.50136432604601,48.240904681838,0
-0.50135807497816,48.240999988312,0
-0.50299999999993,48.241,0
</coordinates>
</LinearRing>
</outerBoundaryIs>
</Polygon>
</Placemark>
<Placemark>
<name>446</name>
<styleUrl>#stylehap1</styleUrl>
<Polygon>
<outerBoundaryIs>
<LinearRing>
<coordinates>
-0.50299999999993,48.267,0
-0.5013572398547,48.266999988301,0
-0.50136348800122,48.267095294953,0
-0.50138219106171,48.267189876439,0
-0.50141320676194,48.267283012932,0
-0.50145629911695,48.267373995596,0
-0.50151114022505,48.267462131986,0
-0.50157731276162,48.267546751313,0
-0.50165431315374,48.267627209554,0
-0.50174155541142,48.267702894353,0
-0.50183837558645,48.267773229681,0
-0.50194403682494,48.267837680219,0
-0.50205773497495,48.267895755437,0
-0.50217860470691,48.267947013325,0
-0.50230572609964,48.267991063759,0
-0.50243813164259,48.268027571469,0
-0.50257481360028,48.268056258593,0
-0.50271473168334,48.268076906792,0
-0.50285682096744,48.26808935891,0
-0.50299999999992,48.268093520174,0
-0.50314317903244,48.26808935891,0
-0.50328526831654,48.268076906792,0
-0.50342518639959,48.268056258593,0
-0.50356186835728,48.268027571469,0
-0.50369427390023,48.267991063759,0
-0.50382139529296,48.267947013325,0
-0.50394226502489,48.267895755437,0
-0.50405596317493,48.267837680219,0
-0.5041616244134,48.267773229681,0
-0.50425844458843,48.267702894353,0
-0.50434568684613,48.267627209554,0
-0.50442268723825,48.267546751313,0
-0.50448885977482,48.267462131986,0
-0.50454370088293,48.267373995596,0
-0.50458679323793,48.267283012932,0
-0.50461780893816,48.267189876439,0
-0.50463651199862,48.267095294953,0
-0.50464276014514,48.266999988301,0
-0.50463650589506,48.266904681827,0
-0.50461779691648,48.266810100869,0
-0.50458677566343,48.266716965238,0

-0.50454367828959,48.266625983743,0
-0.50448883284914,48.266537848796,0
-0.50442265679831,48.266453231139,0
-0.50434565381685,48.266372774746,0
-0.50425840997343,48.266297091917,0
-0.5041615892644,48.266226758621,0
-0.50405592855991,48.266162310114,0
-0.50394223199565,48.266104236866,0
-0.50382136485304,48.266052980826,0
-0.50369424697454,48.266008932062,0
-0.50356184576394,48.265972425794,0
-0.50342516882509,48.265943739839,0
-0.50328525629485,48.265923092502,0
-0.50314317292887,48.265910640912,0
-0.50299999999992,48.265906479826,0
-0.502856827071,48.265910640912,0
-0.50271474370499,48.265923092502,0
-0.50257483117476,48.265943739839,0
-0.50243815423593,48.265972425794,0
-0.50230575302533,48.266008932062,0
-0.50217863514683,48.266052980826,0
-0.50205776800422,48.266104236866,0
-0.50194407143994,48.266162310114,0
-0.50183841073544,48.266226758621,0
-0.50174159002641,48.266297091917,0
-0.50165434618299,48.266372774746,0
-0.50157734320154,48.266453231139,0
-0.50151116715073,48.266537848796,0
-0.50145632171028,48.266625983743,0
-0.50141322433644,48.266716965238,0
-0.50138220308337,48.266810100869,0
-0.50136349410478,48.266904681827,0
-0.5013572398547,48.266999988301,0
-0.50299999999993,48.267,0
</coordinates>
</LinearRing>
</outerBoundaryIs>
</Polygon>
</Placemark>
<Placemark>
<name>447</name>
<styleUrl>#stylehap1</styleUrl>
<Polygon>
<outerBoundaryIs>
<LinearRing>
<coordinates>
-0.50199999999995,48.243,0
-0.50035801078006,48.242999988311,0
-0.50036425599698,48.243095294963,0
-0.50038295028285,48.243189876449,0
-0.50041395143018,48.243283012941,0
-0.50045702356462,48.243373995605,0
-0.50051183893835,48.243462131994,0
-0.50057798042253,48.24354675132,0
-0.5006549446805,48.243627209561,0
-0.50074214599721,48.243702894359,0
-0.50083892073605,48.243773229686,0

-0.50094453238891,48.243837680223,0
-0.50105817718125,48.24389575544,0
-0.50117899018957,48.243947013328,0
-0.50130605192443,48.243991063761,0
-0.50143839532942,48.24402757147,0
-0.50157501314199,48.244056258594,0
-0.50171486556101,48.244076906792,0
-0.50185688816204,48.24408935891,0
-0.50199999999996,48.244093520174,0
-0.50214311183786,48.24408935891,0
-0.50228513443889,48.244076906792,0
-0.50242498685791,48.244056258594,0
-0.50256160467049,48.24402757147,0
-0.50269394807547,48.243991063761,0
-0.50282100981034,48.243947013328,0
-0.50294182281865,48.24389575544,0
-0.50305546761099,48.243837680223,0
-0.50316107926385,48.243773229686,0
-0.50325785400269,48.243702894359,0
-0.5033450553194,48.243627209561,0
-0.50342201957737,48.24354675132,0
-0.50348816106155,48.243462131994,0
-0.50354297643528,48.243373995605,0
-0.50358604856974,48.243283012941,0
-0.50361704971705,48.243189876449,0
-0.50363574400292,48.243095294963,0
-0.50364198921985,48.242999988311,0
-0.50363573790735,48.242904681837,0
-0.50361703771115,48.242810100878,0
-0.5035860310183,48.242716965247,0
-0.50354295387158,48.242625983752,0
-0.50348813417119,48.242537848804,0
-0.50342198917738,48.242453231147,0
-0.50334502233349,48.242372774753,0
-0.50325781943309,48.242297091922,0
-0.50316104416096,48.242226758626,0
-0.50305543304139,48.242162310118,0
-0.50294178983274,48.242104236869,0
-0.50282097941034,48.242052980828,0
-0.5026939211851,48.242008932064,0
-0.50256158210679,48.241972425795,0
-0.50242496930649,48.24194373984,0
-0.50228512243298,48.241923092503,0
-0.50214310574232,48.241910640912,0
-0.50199999999996,48.241906479826,0
-0.50185689425758,48.241910640912,0
-0.50171487756692,48.241923092503,0
-0.50157503069343,48.24194373984,0
-0.50143841789311,48.241972425795,0
-0.50130607881483,48.242008932064,0
-0.50117902058956,48.242052980828,0
-0.50105821016719,48.242104236869,0
-0.50094456695851,48.242162310118,0
-0.50083895583894,48.242226758626,0
-0.50074218056681,48.242297091922,0
-0.50065497766641,48.242372774753,0
-0.50057801082253,48.242453231147,0

-0.50051186582872,48.242537848804,0
-0.50045704612832,48.242625983752,0
-0.50041396898161,48.242716965247,0
-0.50038296228876,48.242810100878,0
-0.50036426209255,48.242904681837,0
-0.50035801078006,48.242999988311,0
-0.50199999999995,48.243,0
</coordinates>
</LinearRing>
</outerBoundaryIs>
</Polygon>
</Placemark>
<Placemark>
<name>449</name>
<styleUrl>#stylehap1</styleUrl>
<Polygon>
<outerBoundaryIs>
<LinearRing>
<coordinates>
-0.50199999999995,48.147,0
-0.50036108438767,48.146999988351,0
-0.50036731792453,48.147095295002,0
-0.50038597722687,48.147189876487,0
-0.50041692035305,48.147283012978,0
-0.50045991187004,48.14737399564,0
-0.5005146246432,48.147462132026,0
-0.50058064232431,48.14754675135,0
-0.50065746251873,48.147627209587,0
-0.50074450060766,48.147702894382,0
-0.50084109419667,48.147773229705,0
-0.50094650815622,48.147837680239,0
-0.50105994021645,48.147895755453,0
-0.5011805270729,48.147947013337,0
-0.50130735095749,48.147991063768,0
-0.50143944662416,48.148027571475,0
-0.50157580869609,48.148056258597,0
-0.50171539931877,48.148076906794,0
-0.50185715606054,48.148089358911,0
-0.50199999999996,48.148093520174,0
-0.50214284393939,48.148089358911,0
-0.50228460068113,48.148076906794,0
-0.50242419130384,48.148056258597,0
-0.50256055337574,48.148027571475,0
-0.50269264904241,48.147991063768,0
-0.50281947292703,48.147947013337,0
-0.50294005978345,48.147895755453,0
-0.50305349184368,48.147837680239,0
-0.50315890580323,48.147773229705,0
-0.50325549939224,48.147702894382,0
-0.50334253748118,48.147627209587,0
-0.50341935767559,48.14754675135,0
-0.50348537535671,48.147462132026,0
-0.50354008812986,48.14737399564,0
-0.50358307964685,48.147283012978,0
-0.50361402277304,48.147189876487,0
-0.50363268207538,48.147095295002,0
-0.50363891561223,48.146999988351,0

-0.50363267601171,48.146904681876,0
-0.50361401082998,48.146810100917,0
-0.50358306218724,48.146716965284,0
-0.50354006568423,48.146625983787,0
-0.50348534860702,48.146537848836,0
-0.50341932743465,48.146453231176,0
-0.50334250466783,48.146372774779,0
-0.5032554650035,48.146297091945,0
-0.503158870884,48.146226758645,0
-0.50305345745494,48.146162310134,0
-0.50294002697013,48.146104236882,0
-0.50281944268608,48.146052980838,0
-0.50269262229273,48.146008932071,0
-0.50256053093011,48.1459724258,0
-0.50242417384422,48.145943739843,0
-0.50228458873804,48.145923092504,0
-0.50214283787572,48.145910640912,0
-0.50199999999996,48.145906479827,0
-0.50185716212418,48.145910640912,0
-0.50171541126186,48.145923092504,0
-0.50157582615571,48.145943739843,0
-0.50143946906979,48.1459724258,0
-0.50130737770718,48.146008932071,0
-0.50118055731384,48.146052980838,0
-0.5010599730298,48.146104236882,0
-0.50094654254497,48.146162310134,0
-0.5008411291159,48.146226758645,0
-0.50074453499641,48.146297091945,0
-0.50065749533208,48.146372774779,0
-0.50058067256526,48.146453231176,0
-0.50051465139288,48.146537848836,0
-0.50045993431567,48.146625983787,0
-0.50041693781266,48.146716965284,0
-0.50038598916995,48.146810100917,0
-0.50036732398819,48.146904681876,0
-0.50036108438767,48.146999988351,0
-0.50199999999995,48.147,0
</coordinates>
</LinearRing>
</outerBoundaryIs>
</Polygon>
</Placemark>
<Placemark>
<name>450</name>
<styleUrl>#stylehap1</styleUrl>
<Polygon>
<outerBoundaryIs>
<LinearRing>
<coordinates>
-0.50099999999998,48.294,0
-0.49935637135311,48.29399998829,0
-0.49936262280003,48.294095294942,0
-0.4993813357457,48.294189876429,0
-0.49941236784083,48.294283012922,0
-0.4994554829757,48.294373995586,0
-0.49951035307538,48.294462131976,0
-0.49957656059467,48.294546751304,0

-0.49965360169448,48.294627209547,0
-0.49974089007503,48.294702894347,0
-0.49983776143712,48.294773229675,0
-0.4999434785373,48.294837680214,0
-0.50005723679852,48.294895755433,0
-0.50017817043362,48.294947013322,0
-0.50030535903511,48.294991063757,0
-0.50043783458087,48.295027571468,0
-0.50057458880255,48.295056258593,0
-0.50071458086078,48.295076906792,0
-0.50085674526805,48.29508935891,0
-0.50099999999998,48.295093520174,0
-0.50114325473188,48.29508935891,0
-0.50128541913918,48.295076906792,0
-0.50142541119738,48.295056258593,0
-0.50156216541908,48.295027571468,0
-0.50169464096482,48.294991063757,0
-0.50182182956634,48.294947013322,0
-0.50194276320144,48.294895755433,0
-0.50205652146266,48.294837680214,0
-0.50216223856284,48.294773229675,0
-0.50225910992493,48.294702894347,0
-0.50234639830548,48.294627209547,0
-0.50242343940527,48.294546751304,0
-0.50248964692458,48.294462131976,0
-0.50254451702425,48.294373995586,0
-0.50258763215913,48.294283012922,0
-0.50261866425426,48.294189876429,0
-0.50263737719993,48.294095294942,0
-0.50264362864685,48.29399998829,0
-0.50263737108734,48.293904681816,0
-0.50261865221481,48.293810100858,0
-0.50258761455865,48.293716965228,0
-0.50254449439751,48.293625983734,0
-0.50248961995908,48.293537848787,0
-0.50242340892036,48.293453231131,0
-0.5023463652274,48.293372774739,0
-0.50225907525876,48.293297709191,0
-0.50216220336189,48.293226758615,0
-0.50205648679647,48.29316231011,0
-0.50194273012336,48.293104236862,0
-0.50182179908141,48.293052980823,0
-0.50169461399934,48.29300893206,0
-0.50156214279234,48.292972425793,0
-0.5014253935969,48.292943739838,0
-0.50128540709976,48.292923092502,0
-0.50114324861931,48.292910640912,0
-0.50099999999998,48.292906479826,0
-0.50085675138064,48.292910640912,0
-0.50071459290019,48.292923092502,0
-0.50057460640303,48.292943739838,0
-0.50043785720759,48.292972425793,0
-0.50030538600062,48.29300893206,0
-0.50017820091855,48.293052980823,0
-0.50005726987659,48.293104236862,0
-0.49994351320346,48.29316231011,0
-0.49983779663807,48.293226758615,0

-0.49974092474119,48.29329709191,0
-0.49965363477256,48.293372774739,0
-0.4995765910796,48.293453231131,0
-0.49951038004088,48.293537848787,0
-0.49945550560242,48.293625983734,0
-0.49941238544131,48.293716965228,0
-0.49938134778515,48.293810100858,0
-0.49936262891262,48.293904681816,0
-0.49935637135311,48.29399998829,0
-0.50099999999998,48.294,0
</coordinates>
</LinearRing>
</outerBoundaryIs>
</Polygon>
</Placemark>
<Placemark>
<name>451</name>
<styleUrl>#stylehap1</styleUrl>
<Polygon>
<outerBoundaryIs>
<LinearRing>
<coordinates>
-0.50099999999998,48.195,0
-0.49935954959924,48.194999988331,0
-0.4993657889685,48.195095294982,0
-0.49938446573967,48.195189876468,0
-0.49941543783836,48.19528301296,0
-0.49945846961123,48.195373995622,0
-0.49951323361742,48.19546213201,0
-0.49957931311887,48.195546751335,0
-0.49965620525059,48.195627209574,0
-0.49974332484641,48.195702894371,0
-0.49984000889156,48.195773229695,0
-0.4999455215681,48.195837680231,0
-0.50005905985491,48.195895755446,0
-0.50017975963899,48.195947013332,0
-0.50030670229279,48.195991063764,0
-0.50043892166614,48.196027571473,0
-0.5005754114407,48.196056258595,0
-0.50071513278991,48.196076906793,0
-0.50085702228696,48.19608935891,0
-0.50099999999998,48.196093520173,0
-0.50114297771297,48.19608935891,0
-0.50128486721002,48.196076906793,0
-0.50142458855925,48.196056258595,0
-0.50156107833379,48.196027571473,0
-0.50169329770717,48.195991063764,0
-0.50182024036094,48.195947013332,0
-0.50194094014505,48.195895755446,0
-0.50205447843183,48.195837680231,0
-0.5021599911084,48.195773229695,0
-0.50225667515355,48.195702894371,0
-0.50234379474934,48.195627209574,0
-0.50242068688108,48.195546751335,0
-0.50248676638254,48.19546213201,0
-0.50254153038873,48.195373995622,0
-0.50258456216157,48.19528301296,0

-0.50261553426029,48.195189876468,0
-0.50263421103146,48.195095294982,0
-0.50264045040069,48.194999988331,0
-0.50263420495187,48.194904681857,0
-0.50261552228586,48.194810100897,0
-0.50258454465613,48.194716965266,0
-0.50254150788414,48.19462598377,0
-0.50248673956263,48.19453784882,0
-0.50242065656075,48.194453231162,0
-0.50234376184986,48.194372774766,0
-0.50225664067453,48.194297091934,0
-0.50215995609749,48.194226758636,0
-0.50205444395281,48.194162310126,0
-0.50194090724554,48.194104236875,0
-0.50182021004062,48.194052980833,0
-0.50169327088725,48.194008932067,0
-0.50156105582923,48.193972425797,0
-0.50142457105379,48.193943739841,0
-0.50128485523559,48.193923092503,0
-0.50114297163341,48.193910640912,0
-0.50099999999998,48.193906479826,0
-0.50085702836655,48.193910640912,0
-0.50071514476434,48.193923092503,0
-0.50057542894617,48.193943739841,0
-0.50043894417073,48.193972425797,0
-0.50030672911268,48.194008932067,0
-0.50017978995934,48.194052980833,0
-0.50005909275441,48.194104236875,0
-0.49994555604714,48.194162310126,0
-0.49984004390247,48.194226758636,0
-0.49974335932543,48.194297091934,0
-0.4996562381501,48.194372774766,0
-0.4995793434392,48.194453231162,0
-0.49951326043731,48.19453784882,0
-0.49945849211582,48.19462598377,0
-0.49941545534383,48.194716965266,0
-0.4993844777141,48.194810100897,0
-0.49936579504807,48.194904681857,0
-0.49935954959924,48.194999988331,0
-0.50099999999998,48.195,0
</coordinates>
</LinearRing>
</outerBoundaryIs>
</Polygon>
</Placemark>
<Placemark>
<name>452</name>
<styleUrl>#stylehap1</styleUrl>
<Polygon>
<outerBoundaryIs>
<LinearRing>
<coordinates>
-0.5,48.317,0
-0.49835563050558,48.316999988281,0
-0.4983618847678,48.317095294933,0
-0.49838060614574,48.317189876419,0
-0.49841165222596,48.317283012913,0

-0.4984547867925,48.317373995578,0
-0.49850968162251,48.317462131969,0
-0.49857591898271,48.317546751297,0
-0.49865299480688,48.31762720954,0
-0.49874032253107,48.317702894341,0
-0.49883723755665,48.31777322967,0
-0.49894300230785,48.31783768021,0
-0.49905681184501,48.31789575543,0
-0.49917779999067,48.31794701332,0
-0.49930504592245,48.317991063755,0
-0.49943758118185,48.318027571467,0
-0.49957439704603,48.318056258592,0
-0.49971445220639,48.318076906792,0
-0.49985668069512,48.31808935891,0
-0.49999999999999,48.318093520173,0
-0.50014331930489,48.31808935891,0
-0.50028554779363,48.318076906792,0
-0.50042560295395,48.318056258592,0
-0.50056241881814,48.318027571467,0
-0.50069495407754,48.317991063755,0
-0.50082220000932,48.31794701332,0
-0.50094318815498,48.31789575543,0
-0.50105699769214,48.31783768021,0
-0.50116276244337,48.31777322967,0
-0.50125967746895,48.317702894341,0
-0.50134700519311,48.31762720954,0
-0.5014240810173,48.317546751297,0
-0.5014903183775,48.317462131969,0
-0.50154521320751,48.317373995578,0
-0.50158834777403,48.317283012913,0
-0.50161939385425,48.317189876419,0
-0.50163811523219,48.317095294933,0
-0.50164436949443,48.316999988281,0
-0.50163810911191,48.316904681807,0
-0.50161938179967,48.316810100849,0
-0.50158833015139,48.316716965219,0
-0.50154519055227,48.316625983725,0
-0.50149029137803,48.316537848779,0
-0.50142405049398,48.316453231124,0
-0.50134697207338,48.316372774732,0
-0.5012596427591,48.316297091905,0
-0.50116272719806,48.31622675861,0
-0.50105696298229,48.316162310106,0
-0.50094315503526,48.316104236859,0
-0.500822169486,48.31605298082,0
-0.50069492707807,48.316008932058,0
-0.5005623961629,48.315972425791,0
-0.50042558533132,48.315943739838,0
-0.50028553573902,48.315923092502,0
-0.50014331318461,48.315910640912,0
-0.49999999999999,48.315906479826,0
-0.4998566868154,48.315910640912,0
-0.49971446426097,48.315923092502,0
-0.4995744146687,48.315943739838,0
-0.49943760383709,48.315972425791,0
-0.49930507292192,48.316008932058,0
-0.49917783051399,48.31605298082,0

-0.49905684496476,48.316104236859,0
-0.4989430370177,48.316162310106,0
-0.49883727280193,48.31622675861,0
-0.49874035724089,48.316297091905,0
-0.49865302792663,48.316372774732,0
-0.49857594950601,48.316453231124,0
-0.49850970862196,48.316537848779,0
-0.49845480944772,48.316625983725,0
-0.49841166984863,48.316716965219,0
-0.49838061820034,48.316810100849,0
-0.49836189088808,48.316904681807,0
-0.49835563050558,48.316999988281,0
-0.5,48.317,0
</coordinates>
</LinearRing>
</outerBoundaryIs>
</Polygon>
</Placemark>
<Placemark>
<name>456</name>
<styleUrl>#stylehap1</styleUrl>
<Polygon>
<outerBoundaryIs>
<LinearRing>
<coordinates>
-0.49700000000007,48.233,0
-0.49535833170057,48.232999988315,0
-0.49536457569796,48.233095294967,0
-0.49538326633113,48.233189876453,0
-0.49541426142038,48.233283012945,0
-0.49545732513744,48.233373995609,0
-0.49551212979847,48.233462131997,0
-0.49557825835617,48.233546751323,0
-0.49565520757222,48.233627209564,0
-0.49574239184607,48.233702894361,0
-0.49583914767077,48.233773229688,0
-0.49594473868213,48.233837680224,0
-0.49605836126277,48.233895755441,0
-0.49617915065819,48.233947013329,0
-0.49630618755873,48.233991063762,0
-0.49643850509692,48.234027571471,0
-0.49657509620721,48.234056258594,0
-0.49671492129166,48.234076906793,0
-0.49685691613387,48.23408935891,0
-0.49700000000007,48.234093520174,0
-0.49714308386629,48.23408935891,0
-0.49728507870847,48.234076906793,0
-0.49742490379294,48.234056258594,0
-0.49756149490321,48.234027571471,0
-0.4976938124414,48.233991063762,0
-0.49782084934196,48.233947013329,0
-0.49794163873738,48.233895755441,0
-0.498055261318,48.233837680224,0
-0.49816085232939,48.233773229688,0
-0.49825760815409,48.233702894361,0
-0.49834479242793,48.233627209564,0
-0.49842174164396,48.233546751323,0

-0.49848787020166,48.233462131997,0
-0.49854267486272,48.233373995609,0
-0.49858573857975,48.233283012945,0
-0.498616733669,48.233189876453,0
-0.49863542430217,48.233095294967,0
-0.49864166829956,48.232999988315,0
-0.49863541820996,48.232904681841,0
-0.49861672166966,48.232810100882,0
-0.49858572103793,48.232716965251,0
-0.49854265231134,48.232625983756,0
-0.498487843326,48.232537848807,0
-0.49842171126061,48.23245323115,0
-0.49834475946003,48.232372774755,0
-0.49825757360339,48.232297091925,0
-0.49816081724568,48.232226758628,0
-0.4980552267673,48.23216231012,0
-0.49794160576948,48.23210423687,0
-0.49782081895858,48.232052980829,0
-0.49769378556574,48.232008932065,0
-0.49756147235183,48.231972425795,0
-0.49742488625109,48.23194373984,0
-0.49728506670915,48.231923092503,0
-0.49714307777405,48.231910640912,0
-0.49700000000007,48.231906479826,0
-0.49685692222608,48.231910640912,0
-0.496714933291,48.231923092503,0
-0.49657511374907,48.23194373984,0
-0.4964385276483,48.231972425795,0
-0.49630621443439,48.232008932065,0
-0.49617918104155,48.232052980829,0
-0.49605839423065,48.23210423687,0
-0.49594477323283,48.23216231012,0
-0.49583918275445,48.232226758628,0
-0.49574242639677,48.232297091925,0
-0.49565524054012,48.232372774755,0
-0.49557828873955,48.23245323115,0
-0.49551215667413,48.232537848807,0
-0.49545734768879,48.232625983756,0
-0.49541427896223,48.232716965251,0
-0.49538327833048,48.232810100882,0
-0.49536458179018,48.232904681841,0
-0.49535833170057,48.232999988315,0
-0.49700000000007,48.233,0
</coordinates>
</LinearRing>
</outerBoundaryIs>
</Polygon>
</Placemark>
<Placemark>
<name>458</name>
<styleUrl>#stylehap1</styleUrl>
<Polygon>
<outerBoundaryIs>
<LinearRing>
<coordinates>
-0.49599999999998,48.253,0
-0.49435768968417,48.252999988307,0

-0.49436393612131,48.253095294959,0
-0.49438263406186,48.253189876445,0
-0.49441364127055,48.253283012937,0
-0.49445672182702,48.253373995601,0
-0.49451154791929,48.25346213199,0
-0.49457770233703,48.253546751317,0
-0.49465468164513,48.253627209558,0
-0.49474190001404,48.253702894357,0
-0.49483869367739,48.253773229684,0
-0.49494432598302,48.253837680221,0
-0.49505799299921,48.253895755439,0
-0.49517882963332,48.253947013327,0
-0.49530591621611,48.25399106376,0
-0.495438285502,48.25402757147,0
-0.49557493003146,48.254056258594,0
-0.49571480980005,48.254076906792,0
-0.49585686017503,48.25408935891,0
-0.49599999999998,48.254093520174,0
-0.49614313982493,48.25408935891,0
-0.49628519019993,48.254076906792,0
-0.49642506996849,48.254056258594,0
-0.49656171449795,48.25402757147,0
-0.49669408378385,48.25399106376,0
-0.49682117036663,48.253947013327,0
-0.49694200700075,48.253895755439,0
-0.49705567401694,48.253837680221,0
-0.49716130632257,48.253773229684,0
-0.49725809998592,48.253702894357,0
-0.49734531835485,48.253627209558,0
-0.49742229766295,48.253546751317,0
-0.49748845208067,48.25346213199,0
-0.49754327817294,48.253373995601,0
-0.49758635872941,48.253283012937,0
-0.49761736593812,48.253189876445,0
-0.49763606387865,48.253095294959,0
-0.49764231031579,48.252999988307,0
-0.49763605777977,48.252904681833,0
-0.49761735392565,48.252810100874,0
-0.49758634116835,48.252716965243,0
-0.4975432555969,48.252625983749,0
-0.49748842517559,48.252537848801,0
-0.49742226724631,48.252453231144,0
-0.49734528535087,48.25237277475,0
-0.49725806539741,48.25229709192,0
-0.49716127120049,48.252226758624,0
-0.49705563942844,48.252162310117,0
-0.49694197399679,48.252104236868,0
-0.49682113995002,48.252052980827,0
-0.49669405687878,48.252008932063,0
-0.49656169192192,48.251972425795,0
-0.49642505240746,48.25194373984,0
-0.49628517818746,48.251923092502,0
-0.49614313372605,48.251910640912,0
-0.49599999999998,48.251906479826,0
-0.49585686627393,48.251910640912,0
-0.4957148218125,48.251923092502,0
-0.4955749475925,48.25194373984,0

-0.49543830807804,48.251972425795,0
-0.49530594312121,48.252008932063,0
-0.49517886004994,48.252052980827,0
-0.49505802600317,48.252104236868,0
-0.49494436057152,48.252162310117,0
-0.49483872879949,48.252226758624,0
-0.49474193460255,48.25229709192,0
-0.49465471464909,48.2523727475,0
-0.49457773275364,48.252453231144,0
-0.49451157482437,48.252537848801,0
-0.49445674440305,48.252625983749,0
-0.49441365883161,48.252716965243,0
-0.49438264607431,48.252810100874,0
-0.49436394222019,48.252904681833,0
-0.49435768968417,48.252999988307,0
-0.49599999999998,48.253,0
</coordinates>
</LinearRing>
</outerBoundaryIs>
</Polygon>
</Placemark>
<Placemark>
<name>459</name>
<styleUrl>#stylehap1</styleUrl>
<Polygon>
<outerBoundaryIs>
<LinearRing>
<coordinates>
-0.49599999999998,48.223,0
-0.49435865244545,48.222999988319,0
-0.49436489522398,48.223095294971,0
-0.49438358220649,48.223189876457,0
-0.49441457124095,48.223283012949,0
-0.49445762654519,48.223373995612,0
-0.4945124204994,48.223462132,0
-0.49457853613769,48.223546751326,0
-0.49465547032005,48.223627209566,0
-0.49474263756032,48.223702894364,0
-0.4948393744812,48.22377322969,0
-0.49494494486239,48.223837680226,0
-0.49505854524345,48.223895755443,0
-0.49517931103887,48.22394701333,0
-0.49530632311867,48.223991063762,0
-0.49543861480424,48.224027571471,0
-0.4955751792268,48.224056258595,0
-0.49571497699164,48.224076906793,0
-0.4958569440902,48.22408935891,0
-0.49599999999998,48.224093520174,0
-0.49614305590976,48.22408935891,0
-0.49628502300832,48.224076906793,0
-0.49642482077315,48.224056258595,0
-0.49656138519571,48.224027571471,0
-0.49669367688129,48.223991063762,0
-0.49682068896108,48.22394701333,0
-0.49694145475651,48.223895755443,0
-0.49705505513759,48.223837680226,0
-0.49716062551876,48.22377322969,0

-0.49725736243964,48.223702894364,0
-0.49734452967991,48.223627209566,0
-0.49742146386227,48.223546751326,0
-0.49748757950056,48.223462132,0
-0.49754237345477,48.223373995612,0
-0.49758542875901,48.223283012949,0
-0.4976164177935,48.223189876457,0
-0.49763510477598,48.223095294971,0
-0.49764134755451,48.222999988319,0
-0.4976350986871,48.222904681845,0
-0.49761640580072,48.222810100886,0
-0.49758541122675,48.222716965255,0
-0.49754235091573,48.222625983759,0
-0.49748755263958,48.222537848811,0
-0.49742143349551,48.222453231153,0
-0.49734449673005,48.222372774758,0
-0.49725732790782,48.222297091927,0
-0.49716059045424,48.22222675863,0
-0.49705502060577,48.222162310122,0
-0.49694142180665,48.222104236872,0
-0.49682065859432,48.22205298083,0
-0.49669365002031,48.222008932065,0
-0.49656136265668,48.221972425796,0
-0.49642480324089,48.22194373984,0
-0.49628501101555,48.221923092503,0
-0.49614304982086,48.221910640912,0
-0.49599999999998,48.221906479827,0
-0.4958569501791,48.221910640912,0
-0.49571498898441,48.221923092503,0
-0.49557519675909,48.22194373984,0
-0.49543863734328,48.221972425796,0
-0.49530634997965,48.222008932065,0
-0.49517934140567,48.22205298083,0
-0.49505857819331,48.222104236872,0
-0.49494497939421,48.222162310122,0
-0.49483940954572,48.22222675863,0
-0.49474267209214,48.222297091927,0
-0.49465550326991,48.222372774758,0
-0.49457856650445,48.222453231153,0
-0.49451244736038,48.222537848811,0
-0.49445764908423,48.222625983759,0
-0.49441458877321,48.222716965255,0
-0.49438359419924,48.222810100886,0
-0.49436490131286,48.222904681845,0
-0.49435865244545,48.222999988319,0
-0.49599999999998,48.223,0
</coordinates>
</LinearRing>
</outerBoundaryIs>
</Polygon>
</Placemark>
<Placemark>
<name>462</name>
<styleUrl>#stylehap1</styleUrl>
<Polygon>
<outerBoundaryIs>
<LinearRing>

<coordinates>
-0.49400000000003,48.236,0
-0.49235823544283,48.235999988314,0
-0.49236447980602,48.236095294966,0
-0.4923831715348,48.236189876452,0
-0.49241416844111,48.236283012944,0
-0.49245723468289,48.236373995607,0
-0.49251204255712,48.236462131996,0
-0.49257817499202,48.236546751322,0
-0.49265512871978,48.236627209563,0
-0.49274231810551,48.236702894361,0
-0.49283907960338,48.236773229687,0
-0.49294467680601,48.236837680224,0
-0.49305830604887,48.236895755441,0
-0.4931791025268,48.236947013328,0
-0.49330614687622,48.236991063761,0
-0.49343847217296,48.237027571471,0
-0.4935750712924,48.237056258594,0
-0.49371490457565,48.237076906793,0
-0.49385690774392,48.23708935891,0
-0.49400000000004,48.237093520174,0
-0.49414309225615,48.23708935891,0
-0.49428509542439,48.237076906793,0
-0.49442492870765,48.237056258594,0
-0.49456152782708,48.237027571471,0
-0.49469385312382,48.236991063761,0
-0.49482089747327,48.236947013328,0
-0.4949416939512,48.236895755441,0
-0.49505532319406,48.236837680224,0
-0.49516092039669,48.236773229687,0
-0.49525768189453,48.236702894361,0
-0.49534487128027,48.236627209563,0
-0.49542182500803,48.236546751322,0
-0.49548795744292,48.236462131996,0
-0.49554276531715,48.236373995607,0
-0.49558583155894,48.236283012944,0
-0.49561682846527,48.236189876452,0
-0.49563552019403,48.236095294966,0
-0.49564176455721,48.235999988314,0
-0.49563551410082,48.23590468184,0
-0.49561681646397,48.235810100881,0
-0.49558581401421,48.23571696525,0
-0.49554274276209,48.235625983755,0
-0.49548793056286,48.235537848806,0
-0.49542179461969,48.235453231149,0
-0.49534483830697,48.235372774755,0
-0.49525764733819,48.235297091924,0
-0.49516088530723,48.235226758627,0
-0.49505528863769,48.235162310119,0
-0.49494166097788,48.23510423687,0
-0.4948208670849,48.235052980829,0
-0.49469382624376,48.235008932064,0
-0.49456150527201,48.234972425795,0
-0.49442491116292,48.23494373984,0
-0.49428508342309,48.234923092503,0
-0.49414308616292,48.234910640912,0
-0.49400000000004,48.234906479826,0

-0.49385691383712,48.234910640912,0
-0.49371491657695,48.234923092503,0
-0.49357508883712,48.23494373984,0
-0.49343849472803,48.234972425795,0
-0.49330617375631,48.235008932064,0
-0.49317913291517,48.235052980829,0
-0.49305833902217,48.23510423687,0
-0.49294471136236,48.235162310119,0
-0.49283911469284,48.235226758627,0
-0.49274235266189,48.235297091924,0
-0.4926551616931,48.235372774755,0
-0.49257820538039,48.235453231149,0
-0.49251206943721,48.235537848806,0
-0.49245725723796,48.235625983755,0
-0.49241418598583,48.23571696525,0
-0.4923831835361,48.235810100881,0
-0.49236448589925,48.23590468184,0
-0.49235823544283,48.235999988314,0
-0.49400000000003,48.236,0
</coordinates>
</LinearRing>
</outerBoundaryIs>
</Polygon>
</Placemark>
<Placemark>
<name>463</name>
<styleUrl>#stylehap1</styleUrl>
<Polygon>
<outerBoundaryIs>
<LinearRing>
<coordinates>
-0.49299999999994,48.248,0
-0.49135785025404,48.247999988309,0
-0.49136409608099,48.248095294961,0
-0.49138279219395,48.248189876447,0
-0.49141379637154,48.248283012939,0
-0.49145687271642,48.248373995603,0
-0.49151169344869,48.248462131992,0
-0.49157784139877,48.248546751318,0
-0.49165481318076,48.248627209559,0
-0.49174202302242,48.248702894358,0
-0.49183880722222,48.248773229685,0
-0.49194442920005,48.248837680222,0
-0.4920580851028,48.248895755439,0
-0.49217890992238,48.248947013327,0
-0.49230598407952,48.24899106376,0
-0.49243834042318,48.24902757147,0
-0.49257497159238,48.249056258594,0
-0.4927148376843,48.249076906792,0
-0.49285687417041,48.24908935891,0
-0.49299999999995,48.249093520173,0
-0.49314312582947,48.24908935891,0
-0.49328516231557,48.249076906792,0
-0.49342502840749,48.249056258594,0
-0.49356165957669,48.24902757147,0
-0.49369401592035,48.24899106376,0
-0.4938210900775,48.248947013327,0

-0.49394191489709,48.248895755439,0
-0.49405557079982,48.248837680222,0
-0.49416119277765,48.248773229685,0
-0.49425797697745,48.248702894358,0
-0.49434518681912,48.248627209559,0
-0.49442215860113,48.248546751318,0
-0.49448830655118,48.248462131992,0
-0.49454312728345,48.248373995603,0
-0.49458620362833,48.248283012939,0
-0.49461720780592,48.248189876447,0
-0.49463590391891,48.248095294961,0
-0.49464214974584,48.247999988309,0
-0.49463589782168,48.247904681835,0
-0.49461719579676,48.247810100876,0
-0.4945861860721,48.247716965245,0
-0.49454310471357,48.24762598375,0
-0.49448827965346,48.247537848802,0
-0.49442212819282,48.247453231145,0
-0.49434515382417,48.247372774751,0
-0.49425794239841,48.247297091921,0
-0.49416115766517,48.247226758625,0
-0.49405553622078,48.247162310117,0
-0.49394188190215,48.247104236868,0
-0.49382105966918,48.247052980828,0
-0.49369398902263,48.247008932063,0
-0.49356163700681,48.246972425795,0
-0.49342501085126,48.24694373984,0
-0.49328515030638,48.246923092503,0
-0.49314311973224,48.246910640912,0
-0.49299999999995,48.246906479826,0
-0.49285688026763,48.246910640912,0
-0.49271484969349,48.246923092503,0
-0.49257498914861,48.24694373984,0
-0.49243836299306,48.246972425795,0
-0.49230601097724,48.247008932063,0
-0.49217894033069,48.247052980828,0
-0.49205811809775,48.247104236868,0
-0.49194446377909,48.247162310117,0
-0.4918388423347,48.247226758625,0
-0.49174205760146,48.247297091921,0
-0.4916548461757,48.247372774751,0
-0.49157787180706,48.247453231145,0
-0.49151172034642,48.247537848802,0
-0.4914568952863,48.24762598375,0
-0.49141381392779,48.247716965245,0
-0.49138280420311,48.247810100876,0
-0.49136410217819,48.247904681835,0
-0.49135785025404,48.247999988309,0
-0.49299999999994,48.248,0
</coordinates>
</LinearRing>
</outerBoundaryIs>
</Polygon>
</Placemark>
<Placemark>
<name>464</name>
<styleUrl>#stylehap1</styleUrl>

<Polygon>
<outerBoundaryIs>
<LinearRing>
<coordinates>
-0.49299999999994,48.21,0
-0.49135906915185,48.209999988325,0
-0.49136531034684,48.210095294976,0
-0.49138399258643,48.210189876462,0
-0.49141497375464,48.210283012954,0
-0.49145801812912,48.210373995617,0
-0.49151279817319,48.210462132005,0
-0.49157889702677,48.21054675133,0
-0.49165581167763,48.21062720957,0
-0.49174295678819,48.210702894367,0
-0.49183966914956,48.210773229692,0
-0.49194521272835,48.210837680228,0
-0.49205878426811,48.210895755444,0
-0.49217951940283,48.210947013331,0
-0.49230649923592,48.210991063763,0
-0.49243875733421,48.211027571472,0
-0.49257528708457,48.211056258595,0
-0.49271504935617,48.211076906793,0
-0.49285698041068,48.21108935891,0
-0.49299999999995,48.211093520174,0
-0.49314301958919,48.21108935891,0
-0.49328495064373,48.211076906793,0
-0.49342471291532,48.211056258595,0
-0.49356124266567,48.211027571472,0
-0.49369350076395,48.210991063763,0
-0.49382048059704,48.210947013331,0
-0.49394121573179,48.210895755444,0
-0.49405478727152,48.210837680228,0
-0.49416033085031,48.210773229692,0
-0.49425704321169,48.210702894367,0
-0.49434418832224,48.21062720957,0
-0.4944211029731,48.21054675133,0
-0.4944872018267,48.210462132005,0
-0.49454198187075,48.210373995617,0
-0.49458502624523,48.210283012954,0
-0.49461600741344,48.210189876462,0
-0.49463468965303,48.210095294976,0
-0.49464093084802,48.209999988325,0
-0.49463468356847,48.209904681851,0
-0.49461599542919,48.209810100891,0
-0.49458500872544,48.20971696526,0
-0.49454195934772,48.209625983764,0
-0.49448717498478,48.209537848815,0
-0.49442107262789,48.209453231157,0
-0.49434415539577,48.209372774762,0
-0.4942570087044,48.20929709193,0
-0.4941602958107,48.209226758632,0
-0.49405475276426,48.209162310124,0
-0.49394118280531,48.209104236873,0
-0.49382045025182,48.209052980831,0
-0.49369347392205,48.209008932066,0
-0.49356122014263,48.208972425797,0
-0.49342469539551,48.208943739841,0

-0.49328493865945,48.208923092503,0
-0.49314301350462,48.208910640912,0
-0.49299999999995,48.208906479826,0
-0.49285698649526,48.208910640912,0
-0.49271506134042,48.208923092503,0
-0.49257530460437,48.208943739841,0
-0.49243877985724,48.208972425797,0
-0.49230652607782,48.209008932066,0
-0.49217954974805,48.209052980831,0
-0.49205881719456,48.209104236873,0
-0.49194524723564,48.209162310124,0
-0.49183970418917,48.209226758632,0
-0.49174299129547,48.20929709193,0
-0.49165584460411,48.209372774762,0
-0.49157892737199,48.209453231157,0
-0.49151282501509,48.209537848815,0
-0.49145804065215,48.209625983764,0
-0.49141499127443,48.20971696526,0
-0.49138400457068,48.209810100891,0
-0.4913653164314,48.209904681851,0
-0.49135906915185,48.209999988325,0
-0.49299999999994,48.21,0
</coordinates>
</LinearRing>
</outerBoundaryIs>
</Polygon>
</Placemark>
<Placemark>
<name>465</name>
<styleUrl>#stylehap1</styleUrl>
<Polygon>
<outerBoundaryIs>
<LinearRing>
<coordinates>
-0.49199999999996,48.231,0
-0.49035839586347,48.230999988316,0
-0.49036463961706,48.231095294968,0
-0.49038332951993,48.231189876454,0
-0.49041432339794,48.231283012946,0
-0.49045738543208,48.231373995609,0
-0.49051218795128,48.231462131998,0
-0.49057831392453,48.231546751324,0
-0.49065526013318,48.231627209564,0
-0.49074244099958,48.231702894362,0
-0.49083919304268,48.231773229688,0
-0.49094477992711,48.231837680225,0
-0.49105839806686,48.231895755442,0
-0.49117918274124,48.231947013329,0
-0.49130621467654,48.231991063762,0
-0.49143852704311,48.232027571471,0
-0.49157511281467,48.232056258594,0
-0.49171493243401,48.232076906793,0
-0.49185692172628,48.23208935891,0
-0.49199999999996,48.232093520174,0
-0.49214307827365,48.23208935891,0
-0.49228506756592,48.232076906793,0
-0.49242488718526,48.232056258594,0

-0.49256147295682,48.232027571471,0
-0.49269378532336,48.231991063762,0
-0.49282081725867,48.231947013329,0
-0.49294160193307,48.231895755442,0
-0.49305522007282,48.231837680225,0
-0.49316080695725,48.231773229688,0
-0.49325755900035,48.231702894362,0
-0.49334473986675,48.231627209564,0
-0.4934216860754,48.231546751324,0
-0.49348781204865,48.231462131998,0
-0.49354261456785,48.231373995609,0
-0.49358567660196,48.231283012946,0
-0.49361667047998,48.231189876454,0
-0.49363536038287,48.231095294968,0
-0.49364160413643,48.230999988316,0
-0.49363535429132,48.230904681842,0
-0.49361665848196,48.230810100883,0
-0.49358565906205,48.230716965252,0
-0.49354259201894,48.230625983757,0
-0.49348778517592,48.230537848808,0
-0.49342165569535,48.23045323115,0
-0.49334470690246,48.230372774756,0
-0.49325752445344,48.230297091925,0
-0.49316077187739,48.230226758628,0
-0.49305518552589,48.23016231012,0
-0.49294156896879,48.230104236871,0
-0.49282078687862,48.230052980829,0
-0.49269375845065,48.230008932065,0
-0.49256145040791,48.229972425796,0
-0.49242486964531,48.22994373984,0
-0.4922850555679,48.229923092503,0
-0.4921430721821,48.229910640912,0
-0.49199999999996,48.229906479827,0
-0.49185692781783,48.229910640912,0
-0.49171494443203,48.229923092503,0
-0.49157513035459,48.22994373984,0
-0.49143854959202,48.229972425796,0
-0.49130624154928,48.230008932065,0
-0.49117921312131,48.230052980829,0
-0.49105843103114,48.230104236871,0
-0.49094481447404,48.23016231012,0
-0.49083922812254,48.230226758628,0
-0.49074247554649,48.230297091925,0
-0.49065529309747,48.230372774756,0
-0.49057834430458,48.23045323115,0
-0.49051221482401,48.230537848808,0
-0.49045740798099,48.230625983757,0
-0.49041434093788,48.230716965252,0
-0.49038334151794,48.230810100883,0
-0.49036464570861,48.230904681842,0
-0.49035839586347,48.230999988316,0
-0.49199999999996,48.231,0
</coordinates>
</LinearRing>
</outerBoundaryIs>
</Polygon>
</Placemark>

<Placemark>
<name>466</name>
<styleUrl>#stylehap1</styleUrl>
<Polygon>
<outerBoundaryIs>
<LinearRing>
<coordinates>
-0.49099999999999,48.253,0
-0.48935768968417,48.252999988307,0
-0.48936393612131,48.253095294959,0
-0.48938263406186,48.253189876445,0
-0.48941364127057,48.253283012937,0
-0.48945672182702,48.253373995601,0
-0.48951154791929,48.25346213199,0
-0.48957770233703,48.253546751317,0
-0.48965468164513,48.253627209558,0
-0.48974190001404,48.253702894357,0
-0.48983869367739,48.253773229684,0
-0.48994432598302,48.253837680221,0
-0.49005799299921,48.253895755439,0
-0.49017882963332,48.253947013327,0
-0.49030591621613,48.25399106376,0
-0.490438285502,48.25402757147,0
-0.49057493003147,48.254056258594,0
-0.49071480980005,48.254076906792,0
-0.49085686017503,48.25408935891,0
-0.49099999999998,48.254093520174,0
-0.49114313982493,48.25408935891,0
-0.49128519019993,48.254076906792,0
-0.49142506996849,48.254056258594,0
-0.49156171449798,48.25402757147,0
-0.49169408378385,48.25399106376,0
-0.49182117036666,48.253947013327,0
-0.49194200700075,48.253895755439,0
-0.49205567401694,48.253837680221,0
-0.49216130632257,48.253773229684,0
-0.49225809998592,48.253702894357,0
-0.49234531835485,48.253627209558,0
-0.49242229766295,48.253546751317,0
-0.49248845208067,48.25346213199,0
-0.49254327817294,48.253373995601,0
-0.49258635872941,48.253283012937,0
-0.49261736593812,48.253189876445,0
-0.49263606387868,48.253095294959,0
-0.49264231031579,48.252999988307,0
-0.49263605777977,48.252904681833,0
-0.49261735392565,48.252810100874,0
-0.49258634116837,48.252716965243,0
-0.49254325559691,48.252625983749,0
-0.49248842517559,48.252537848801,0
-0.49242226724632,48.252453231144,0
-0.49234528535087,48.25237277475,0
-0.49225806539741,48.25229709192,0
-0.49216127120049,48.252226758624,0
-0.49205563942846,48.252162310117,0
-0.49194197399679,48.252104236868,0
-0.49182113995002,48.252052980827,0

-0.49169405687878,48.252008932063,0
-0.49156169192192,48.251972425795,0
-0.49142505240746,48.25194373984,0
-0.49128517818746,48.251923092502,0
-0.49114313372605,48.251910640912,0
-0.49099999999998,48.251906479826,0
-0.49085686627393,48.251910640912,0
-0.4907148218125,48.251923092502,0
-0.49057494759253,48.25194373984,0
-0.49043830807804,48.251972425795,0
-0.49030594312121,48.252008932063,0
-0.49017886004994,48.252052980827,0
-0.49005802600319,48.252104236868,0
-0.48994436057152,48.252162310117,0
-0.48983872879949,48.252226758624,0
-0.48974193460255,48.25229709192,0
-0.48965471464909,48.25237277475,0
-0.48957773275364,48.252453231144,0
-0.48951157482439,48.252537848801,0
-0.48945674440308,48.252625983749,0
-0.48941365883161,48.252716965243,0
-0.48938264607431,48.252810100874,0
-0.48936394222019,48.252904681833,0
-0.48935768968417,48.252999988307,0
-0.49099999999999,48.253,0
</coordinates>
</LinearRing>
</outerBoundaryIs>
</Polygon>
</Placemark>
<Placemark>
<name>467</name>
<styleUrl>#stylehap1</styleUrl>
<Polygon>
<outerBoundaryIs>
<LinearRing>
<coordinates>
-0.49000000000001,48.111,0
-0.48836223284195,48.110999988365,0
-0.48836846201458,48.111095295017,0
-0.48838710824527,48.111189876501,0
-0.48841802969185,48.111283012992,0
-0.48846099108609,48.111373995653,0
-0.48851566552254,48.111462132038,0
-0.48858163694461,48.111546751361,0
-0.48865840330963,48.111627209597,0
-0.48874538040848,48.111702894391,0
-0.48884190631078,48.111773229713,0
-0.48894724640221,48.111837680245,0
-0.489060598975,48.111895755458,0
-0.48918110132976,48.111947013341,0
-0.48930783634154,48.11199106377,0
-0.48943983944073,48.112027571477,0
-0.48957610595511,48.112056258598,0
-0.48971559875755,48.112076906794,0
-0.48985725616092,48.112089358911,0
-0.49000000000002,48.112093520174,0

-0.49014274383909,48.112089358911,0
-0.49028440124247,48.112076906794,0
-0.4904238940449,48.112056258598,0
-0.49056016055928,48.112027571477,0
-0.49069216365848,48.11199106377,0
-0.49081889867025,48.111947013341,0
-0.49093940102501,48.111895755458,0
-0.49105275359781,48.111837680245,0
-0.49115809368924,48.111773229713,0
-0.49125461959153,48.111702894391,0
-0.49134159669038,48.111627209597,0
-0.49141836305541,48.111546751361,0
-0.49148433447747,48.111462132038,0
-0.49153900891392,48.111373995653,0
-0.49158197030816,48.111283012992,0
-0.49161289175474,48.111189876501,0
-0.49163153798546,48.111095295017,0
-0.49163776715806,48.110999988365,0
-0.49163153193371,48.110904681891,0
-0.49161287983509,48.110810100931,0
-0.49158195288282,48.110716965298,0
-0.49153898651234,48.1106259838,0
-0.4914843077803,48.110537848849,0
-0.49141833287383,48.110453231187,0
-0.49134156394146,48.110372774789,0
-0.49125458527032,48.110297091954,0
-0.49115805883855,48.110226758653,0
-0.4910527192766,48.110162310141,0
-0.49093936827609,48.110104236887,0
-0.4908188684887,48.110052980842,0
-0.49069213696131,48.110008932073,0
-0.49056013815769,48.109972425801,0
-0.49042387661958,48.109943739843,0
-0.49028438932284,48.109923092504,0
-0.49014273778734,48.109910640913,0
-0.49000000000002,48.109906479826,0
-0.48985726221268,48.109910640913,0
-0.4897156106772,48.109923092504,0
-0.48957612338046,48.109943739843,0
-0.48943986184232,48.109972425801,0
-0.48930786303871,48.110008932073,0
-0.48918113151132,48.110052980842,0
-0.48906063172393,48.110104236887,0
-0.48894728072342,48.110162310141,0
-0.48884194116146,48.110226758653,0
-0.48874541472969,48.110297091954,0
-0.48865843605856,48.110372774789,0
-0.48858166712619,48.110453231187,0
-0.48851569221971,48.110537848849,0
-0.48846101348768,48.1106259838,0
-0.48841804711719,48.110716965298,0
-0.48838712016492,48.110810100931,0
-0.48836846806633,48.110904681891,0
-0.48836223284195,48.110999988365,0
-0.49000000000001,48.111,0
</coordinates>
</LinearRing>

</outerBoundaryIs>
</Polygon>
</Placemark>
<Placemark>
<name>468</name>
<styleUrl>#stylehap1</styleUrl>
<Polygon>
<outerBoundaryIs>
<LinearRing>
<coordinates>
-0.49000000000001,48.111,0
-0.48836223284195,48.110999988365,0
-0.48836846201458,48.111095295017,0
-0.48838710824527,48.111189876501,0
-0.48841802969185,48.111283012992,0
-0.48846099108609,48.111373995653,0
-0.48851566552254,48.111462132038,0
-0.48858163694461,48.111546751361,0
-0.48865840330963,48.111627209597,0
-0.48874538040848,48.111702894391,0
-0.48884190631078,48.111773229713,0
-0.48894724640221,48.111837680245,0
-0.489060598975,48.111895755458,0
-0.48918110132976,48.111947013341,0
-0.48930783634154,48.11199106377,0
-0.48943983944073,48.112027571477,0
-0.48957610595511,48.112056258598,0
-0.48971559875755,48.112076906794,0
-0.48985725616092,48.112089358911,0
-0.49000000000002,48.112093520174,0
-0.49014274383909,48.112089358911,0
-0.49028440124247,48.112076906794,0
-0.4904238940449,48.112056258598,0
-0.49056016055928,48.112027571477,0
-0.49069216365848,48.11199106377,0
-0.49081889867025,48.111947013341,0
-0.49093940102501,48.111895755458,0
-0.49105275359781,48.111837680245,0
-0.49115809368924,48.111773229713,0
-0.49125461959153,48.111702894391,0
-0.49134159669038,48.111627209597,0
-0.49141836305541,48.111546751361,0
-0.49148433447747,48.111462132038,0
-0.49153900891392,48.111373995653,0
-0.49158197030816,48.111283012992,0
-0.49161289175474,48.111189876501,0
-0.49163153798546,48.111095295017,0
-0.49163776715806,48.110999988365,0
-0.49163153193371,48.110904681891,0
-0.49161287983509,48.110810100931,0
-0.49158195288282,48.110716965298,0
-0.49153898651234,48.1106259838,0
-0.4914843077803,48.110537848849,0
-0.49141833287383,48.110453231187,0
-0.49134156394146,48.110372774789,0
-0.49125458527032,48.110297091954,0
-0.49115805883855,48.110226758653,0

-0.4910527192766,48.110162310141,0
-0.49093936827609,48.110104236887,0
-0.4908188684887,48.110052980842,0
-0.49069213696131,48.110008932073,0
-0.49056013815769,48.109972425801,0
-0.49042387661958,48.109943739843,0
-0.49028438932284,48.109923092504,0
-0.49014273778734,48.109910640913,0
-0.49000000000002,48.109906479826,0
-0.48985726221268,48.109910640913,0
-0.4897156106772,48.109923092504,0
-0.48957612338046,48.109943739843,0
-0.48943986184232,48.109972425801,0
-0.48930786303871,48.110008932073,0
-0.48918113151132,48.110052980842,0
-0.48906063172393,48.110104236887,0
-0.48894728072342,48.110162310141,0
-0.48884194116146,48.110226758653,0
-0.48874541472969,48.110297091954,0
-0.48865843605856,48.110372774789,0
-0.48858166712619,48.110453231187,0
-0.48851569221971,48.110537848849,0
-0.48846101348768,48.1106259838,0
-0.48841804711719,48.110716965298,0
-0.48838712016492,48.110810100931,0
-0.48836846806633,48.110904681891,0
-0.48836223284195,48.110999988365,0
-0.49000000000001,48.111,0
</coordinates>
</LinearRing>
</outerBoundaryIs>
</Polygon>
</Placemark>
<Placemark>
<name>469</name>
<styleUrl>#stylehap1</styleUrl>
<Polygon>
<outerBoundaryIs>
<LinearRing>
<coordinates>
-0.48900000000003,48.108,0
-0.48736232844468,48.107999988367,0
-0.48736855725399,48.108095295018,0
-0.48738720239655,48.108189876503,0
-0.48741812203843,48.108283012993,0
-0.4874610809251,48.108373995654,0
-0.48751575217021,48.108462132039,0
-0.48758171974148,48.108546751362,0
-0.48765848162549,48.108627209598,0
-0.48774545364723,48.108702894392,0
-0.48784197391498,48.108773229713,0
-0.48794730785728,48.108837680246,0
-0.48806065381317,48.108895755458,0
-0.48818114913359,48.108947013341,0
-0.48830787674719,48.108991063771,0
-0.48843987214064,48.109027571477,0
-0.48857613070035,48.109056258598,0

-0.48871561535978,48.109076906794,0
-0.48885726449376,48.109089358911,0
-0.48900000000004,48.109093520174,0
-0.48914273550631,48.109089358911,0
-0.48928438464027,48.109076906794,0
-0.48942386929972,48.109056258598,0
-0.48956012785943,48.109027571477,0
-0.48969212325288,48.108991063771,0
-0.48981885086648,48.108947013341,0
-0.4899393461869,48.108895755458,0
-0.49005269214279,48.108837680246,0
-0.49015802608509,48.108773229713,0
-0.49025454635284,48.108702894392,0
-0.49034151837458,48.108627209598,0
-0.49041828025859,48.108546751362,0
-0.49048424782984,48.108462132039,0
-0.49053891907495,48.108373995654,0
-0.49058187796164,48.108283012993,0
-0.4906127976035,48.108189876503,0
-0.49063144274608,48.108095295018,0
-0.49063767155539,48.107999988367,0
-0.49063143669532,48.107904681892,0
-0.49061278568583,48.107810100932,0
-0.49058186053915,48.107716965299,0
-0.49053889667705,48.107625983801,0
-0.49048422113705,48.10753784885,0
-0.49041825008197,48.107453231188,0
-0.490341485631,48.10737277479,0
-0.49025451203725,48.107297091955,0
-0.4901579912401,48.107226758653,0
-0.4900526578272,48.107162310141,0
-0.48993931344334,48.107104236887,0
-0.48981882068984,48.107052980842,0
-0.48969209656006,48.107008932074,0
-0.4895601054615,48.106972425801,0
-0.48942385187722,48.106943739844,0
-0.4892843727226,48.106923092504,0
-0.48914272945555,48.106910640913,0
-0.48900000000004,48.106906479826,0
-0.48885727054453,48.106910640913,0
-0.48871562727747,48.106923092504,0
-0.48857614812285,48.106943739844,0
-0.48843989453854,48.106972425801,0
-0.48830790343998,48.107008932074,0
-0.48818117931024,48.107052980842,0
-0.48806068655673,48.107104236887,0
-0.48794734217287,48.107162310141,0
-0.48784200875995,48.107226758653,0
-0.48774548796282,48.107297091955,0
-0.48765851436904,48.10737277479,0
-0.4875817499181,48.107453231188,0
-0.48751577886302,48.10753784885,0
-0.48746110332302,48.107625983801,0
-0.4874181394609,48.107716965299,0
-0.48738721431424,48.107810100932,0
-0.48736856330475,48.107904681892,0
-0.48736232844468,48.107999988367,0

-0.48900000000003,48.108,0
</coordinates>
</LinearRing>
</outerBoundaryIs>
</Polygon>
</Placemark>
<Placemark>
<name>470</name>
<styleUrl>#stylehap1</styleUrl>
<Polygon>
<outerBoundaryIs>
<LinearRing>
<coordinates>
-0.48900000000003,48.111,0
-0.48736223284199,48.110999988365,0
-0.48736846201459,48.111095295017,0
-0.48738710824531,48.111189876501,0
-0.48741802969189,48.111283012992,0
-0.48746099108613,48.111373995653,0
-0.48751566552256,48.111462132038,0
-0.48758163694465,48.111546751361,0
-0.48765840330967,48.111627209597,0
-0.4877453804085,48.111702894391,0
-0.48784190631082,48.111773229713,0
-0.48794724640222,48.111837680245,0
-0.48806059897502,48.111895755458,0
-0.48818110132978,48.111947013341,0
-0.48830783634158,48.11199106377,0
-0.48843983944075,48.112027571477,0
-0.48857610595513,48.112056258598,0
-0.48871559875759,48.112076906794,0
-0.48885725616094,48.112089358911,0
-0.48900000000004,48.112093520174,0
-0.48914274383911,48.112089358911,0
-0.48928440124248,48.112076906794,0
-0.48942389404494,48.112056258598,0
-0.48956016055932,48.112027571477,0
-0.48969216365849,48.11199106377,0
-0.48981889867029,48.111947013341,0
-0.48993940102503,48.111895755458,0
-0.49005275359782,48.111837680245,0
-0.49015809368925,48.111773229713,0
-0.49025461959155,48.111702894391,0
-0.4903415966904,48.111627209597,0
-0.49041836305543,48.111546751361,0
-0.49048433447751,48.111462132038,0
-0.49053900891394,48.111373995653,0
-0.49058197030818,48.111283012992,0
-0.49061289175476,48.111189876501,0
-0.49063153798548,48.111095295017,0
-0.49063776715808,48.110999988365,0
-0.49063153193372,48.110904681891,0
-0.49061287983513,48.110810100931,0
-0.49058195288284,48.110716965298,0
-0.49053898651235,48.1106259838,0
-0.49048430778034,48.110537848849,0
-0.49041833287384,48.110453231187,0

-0.49034156394147,48.110372774789,0
-0.49025458527034,48.110297091954,0
-0.49015805883857,48.110226758653,0
-0.49005271927661,48.110162310141,0
-0.4899393682761,48.110104236887,0
-0.48981886848871,48.110052980842,0
-0.48969213696132,48.110008932073,0
-0.48956013815773,48.109972425801,0
-0.4894238766196,48.109943739843,0
-0.48928438932286,48.109923092504,0
-0.48914273778735,48.109910640913,0
-0.48900000000004,48.109906479826,0
-0.48885726221269,48.109910640913,0
-0.48871561067721,48.109923092504,0
-0.48857612338047,48.109943739843,0
-0.48843986184234,48.109972425801,0
-0.48830786303875,48.110008932073,0
-0.48818113151136,48.110052980842,0
-0.48806063172394,48.110104236887,0
-0.48794728072346,48.110162310141,0
-0.48784194116148,48.110226758653,0
-0.48774541472971,48.110297091954,0
-0.4876584360586,48.110372774789,0
-0.4875816671262,48.110453231187,0
-0.48751569221973,48.110537848849,0
-0.48746101348772,48.1106259838,0
-0.48741804711721,48.110716965298,0
-0.48738712016494,48.110810100931,0
-0.48736846806635,48.110904681891,0
-0.48736223284199,48.110999988365,0
-0.48900000000003,48.111,0
</coordinates>
</LinearRing>
</outerBoundaryIs>
</Polygon>
</Placemark>
<Placemark>
<name>476</name>
<styleUrl>#stylehap1</styleUrl>
<Polygon>
<outerBoundaryIs>
<LinearRing>
<coordinates>
-0.48699999999997,48.042,0
-0.48536442775026,48.041999988393,0
-0.48537064858195,48.042095295045,0
-0.48538926983038,48.042189876529,0
-0.48542014984321,48.042283013018,0
-0.48546305366733,48.042373995678,0
-0.48551765483517,48.042462132061,0
-0.48558353784766,48.042546751382,0
-0.48566020133477,48.042627209616,0
-0.48574706187023,48.042702894407,0
-0.4858434584108,48.042773229727,0
-0.48594865732651,48.042837680257,0
-0.48606185798418,48.042895755467,0
-0.48618219884058,48.042947013348,0

-0.486308764,48.042991063775,0
-0.48644059018562,48.04302757148,0
-0.48657667407187,48.0430562586,0
-0.48671597992173,48.043076906795,0
-0.4868574474711,48.043089358911,0
-0.48699999999996,48.043093520174,0
-0.48714255252883,48.043089358911,0
-0.4872840200782,48.043076906795,0
-0.48742332592806,48.0430562586,0
-0.4875594098143,48.04302757148,0
-0.48769123599993,48.042991063775,0
-0.48781780115935,48.042947013348,0
-0.48793814201575,48.042895755467,0
-0.48805134267342,48.042837680257,0
-0.48815654158916,48.042773229727,0
-0.4882529381297,48.042702894407,0
-0.48833979866516,48.042627209616,0
-0.4884164621523,48.042546751382,0
-0.48848234516476,48.042462132061,0
-0.48853694633263,48.042373995678,0
-0.48857985015672,48.042283013018,0
-0.48861073016955,48.042189876529,0
-0.48862935141798,48.042095295045,0
-0.48863557224966,48.041999988393,0
-0.48862934538897,48.041904681919,0
-0.48861071829468,48.041810100958,0
-0.48857983279684,48.041716965324,0
-0.48853692401519,48.041625983825,0
-0.48848231856789,48.041537848872,0
-0.4884164320841,48.041453231208,0
-0.48833976603926,48.041372774808,0
-0.48825290393741,48.041297091971,0
-0.48815650686938,48.041226758667,0
-0.48805130848113,48.041162310152,0
-0.48793810938984,48.041104236896,0
-0.48781777109117,48.041052980849,0
-0.48769120940306,48.041008932079,0
-0.48755938749686,48.040972425805,0
-0.48742330856819,48.040943739845,0
-0.48728400820333,48.040923092505,0
-0.48714254649979,48.040910640913,0
-0.48699999999996,48.040906479827,0
-0.48685745350014,48.040910640913,0
-0.4867159917966,48.040923092505,0
-0.48657669143177,48.040943739845,0
-0.48644061250307,48.040972425805,0
-0.48630879059687,48.041008932079,0
-0.48618222890878,48.041052980849,0
-0.48606189061008,48.041104236896,0
-0.48594869151882,48.041162310152,0
-0.48584349313055,48.041226758667,0
-0.48574709606254,48.041297091971,0
-0.48566023396067,48.041372774808,0
-0.48558356791583,48.041453231208,0
-0.48551768143206,48.041537848872,0
-0.48546307598474,48.041625983825,0
-0.48542016720308,48.041716965324,0

-0.48538928170525,48.041810100958,0
-0.48537065461098,48.041904681919,0
-0.48536442775026,48.041999988393,0
-0.48699999999997,48.042,0
</coordinates>
</LinearRing>
</outerBoundaryIs>
</Polygon>
</Placemark>
<Placemark>
<name>477</name>
<styleUrl>#stylehap1</styleUrl>
<Polygon>
<outerBoundaryIs>
<LinearRing>
<coordinates>
-0.48599999999999,47.961,0
-0.4843669938693,47.960999988426,0
-0.48437320494941,47.961095295077,0
-0.48439179699044,47.961189876561,0
-0.48442262856208,47.961283013049,0
-0.48446546507958,47.961373995707,0
-0.4845199805874,47.961462132088,0
-0.48458576023812,47.961546751406,0
-0.48466230344826,47.961627209638,0
-0.48474902770677,47.961702894427,0
-0.48484527300741,47.961773229743,0
-0.48495030687134,47.96183768027,0
-0.48506332992145,47.961895755478,0
-0.4851834819663,47.961947013356,0
-0.48530984854733,47.961991063781,0
-0.48544146789919,47.962027571484,0
-0.4855773382706,47.962056258602,0
-0.4857164255498,47.962076906796,0
-0.48585767113643,47.962089358911,0
-0.48599999999998,47.962093520174,0
-0.48614232886356,47.962089358911,0
-0.48628357445019,47.962076906796,0
-0.48642266172938,47.962056258602,0
-0.48655853210079,47.962027571484,0
-0.48669015145263,47.961991063781,0
-0.48681651803366,47.961947013356,0
-0.48693667007854,47.961895755478,0
-0.48704969312864,47.96183768027,0
-0.48715472699258,47.961773229743,0
-0.48725097229321,47.961702894427,0
-0.4873376965517,47.961627209638,0
-0.48741423976184,47.961546751406,0
-0.48748001941258,47.961462132088,0
-0.48753453492041,47.961373995707,0
-0.4875773714379,47.961283013049,0
-0.48760820300952,47.961189876561,0
-0.48762679505058,47.961095295077,0
-0.48763300613068,47.960999988426,0
-0.48762678904811,47.960904681951,0
-0.48760819118696,47.96081010099,0
-0.48757735415447,47.960716965355,0

-0.48753451270126,47.960625983854,0
-0.48747999293281,47.960537848899,0
-0.48741420982605,47.960453231233,0
-0.48733766406949,47.96037277483,0
-0.48725093825148,47.96029709199,0
-0.4871546924257,47.960226758683,0
-0.48704965908691,47.960162310166,0
-0.4869366375963,47.960104236907,0
-0.48681648809787,47.960052980857,0
-0.48669012497288,47.960008932084,0
-0.48655850988164,47.959972425808,0
-0.48642264444594,47.959943739848,0
-0.48628356262761,47.959923092506,0
-0.48614232286109,47.959910640913,0
-0.48599999999998,47.959906479826,0
-0.4858576771389,47.959910640913,0
-0.48571643737235,47.959923092506,0
-0.48557735555404,47.959943739848,0
-0.48544149011835,47.959972425808,0
-0.4853098750271,47.960008932084,0
-0.48518351190209,47.960052980857,0
-0.48506336240369,47.960104236907,0
-0.48495034091307,47.960162310166,0
-0.48484530757429,47.960226758683,0
-0.48474906174848,47.96029709199,0
-0.4846623359305,47.96037277483,0
-0.48458579017391,47.960453231233,0
-0.48452000706717,47.960537848899,0
-0.48446548729873,47.960625983854,0
-0.48442264584552,47.960716965355,0
-0.48439180881302,47.96081010099,0
-0.48437321095188,47.960904681951,0
-0.4843669938693,47.960999988426,0
-0.48599999999999,47.961,0
</coordinates>
</LinearRing>
</outerBoundaryIs>
</Polygon>
</Placemark>
<Placemark>
<name>479</name>
<styleUrl>#stylehap1</styleUrl>
<Polygon>
<outerBoundaryIs>
<LinearRing>
<coordinates>
-0.48599999999999,48.165,0
-0.48436050931353,48.164999988343,0
-0.48436674503576,48.165095294995,0
-0.48438541088352,48.16518987648,0
-0.4844163648655,48.165283012971,0
-0.48445937146608,48.165373995633,0
-0.48451410343589,48.16546213202,0
-0.48458014428066,48.165546751344,0
-0.48465699142946,48.165627209582,0
-0.48474406005844,48.165702894378,0
-0.48484068754071,48.165773229702,0

-0.48494613848882,48.165837680236,0
-0.48505961035134,48.165895755451,0
-0.48518023952098,48.165947013336,0
-0.48530710790753,48.165991063767,0
-0.48543924992621,48.166027571474,0
-0.48557565984739,48.166056258596,0
-0.48571529945238,48.166076906793,0
-0.48585710593657,48.166089358911,0
-0.48599999999998,48.166093520174,0
-0.48614289406342,48.166089358911,0
-0.48628470054761,48.166076906793,0
-0.48642434015259,48.166056258596,0
-0.48656075007378,48.166027571474,0
-0.48669289209245,48.165991063767,0
-0.48681976047901,48.165947013336,0
-0.48694038964862,48.165895755451,0
-0.48705386151117,48.165837680236,0
-0.48715931245928,48.165773229702,0
-0.48725593994155,48.165702894378,0
-0.48734300857052,48.165627209582,0
-0.48741985571932,48.165546751344,0
-0.48748589656409,48.16546213202,0
-0.4875406285339,48.165373995633,0
-0.48758363513446,48.165283012971,0
-0.48761458911646,48.16518987648,0
-0.48763325496423,48.165095294995,0
-0.48763949068646,48.164999988343,0
-0.48763324889461,48.164904681869,0
-0.48761457716162,48.164810100909,0
-0.48758361765769,48.164716965277,0
-0.48754060606616,48.16462598378,0
-0.48748586978809,48.16453784883,0
-0.48741982544864,48.164453231171,0
-0.48734297572491,48.164372774774,0
-0.48725590551899,48.164297091941,0
-0.48715927750572,48.164226758642,0
-0.48705382708861,48.164162310131,0
-0.486940356803,48.16410423688,0
-0.48681973020832,48.164052980836,0
-0.48669286531646,48.16400893207,0
-0.48656072760606,48.163972425799,0
-0.4864243226758,48.163943739842,0
-0.48628468859279,48.163923092504,0
-0.48614288799378,48.163910640912,0
-0.48599999999998,48.163906479827,0
-0.48585711200618,48.163910640912,0
-0.48571531140719,48.163923092504,0
-0.48557567732416,48.163943739842,0
-0.48543927239392,48.163972425799,0
-0.48530713468353,48.16400893207,0
-0.48518026979167,48.164052980836,0
-0.48505964319698,48.16410423688,0
-0.48494617291137,48.164162310131,0
-0.48484072249427,48.164226758642,0
-0.48474409448097,48.164297091941,0
-0.48465702427508,48.164372774774,0
-0.48458017455132,48.164453231171,0

-0.48451413021189,48.16453784883,0
-0.4844593939338,48.16462598378,0
-0.48441638234229,48.164716965277,0
-0.48438542283836,48.164810100909,0
-0.48436675110537,48.164904681869,0
-0.48436050931353,48.164999988343,0
-0.48599999999999,48.165,0
</coordinates>
</LinearRing>
</outerBoundaryIs>
</Polygon>
</Placemark>
<Placemark>
<name>481</name>
<styleUrl>#stylehap1</styleUrl>
<Polygon>
<outerBoundaryIs>
<LinearRing>
<coordinates>
-0.48199999999997,48.113,0
-0.48036216909811,48.112999988364,0
-0.48036839851294,48.113095295016,0
-0.48038704546918,48.113189876501,0
-0.48041796811906,48.113283012991,0
-0.48046093118525,48.113373995652,0
-0.48051560774952,48.113462132037,0
-0.48058158173915,48.11354675136,0
-0.48065835109194,48.113627209597,0
-0.48074533157597,48.11370289439,0
-0.48084186123516,48.113773229712,0
-0.48094720542656,48.113837680245,0
-0.48106056241121,48.113895755458,0
-0.48118106945615,48.113947013341,0
-0.48130780940073,48.11399106377,0
-0.4814398176378,48.114027571477,0
-0.48157608945599,48.114056258598,0
-0.48171558768786,48.114076906794,0
-0.48185725060492,48.114089358911,0
-0.48199999999996,48.114093520174,0
-0.48214274939503,48.114089358911,0
-0.48228441231206,48.114076906794,0
-0.48242391054394,48.114056258598,0
-0.48256018236216,48.114027571477,0
-0.4826921905992,48.11399106377,0
-0.48281893054378,48.113947013341,0
-0.48293943758872,48.113895755458,0
-0.4830527945734,48.113837680245,0
-0.48315813876479,48.113773229712,0
-0.48325466842398,48.11370289439,0
-0.48334164890801,48.113627209597,0
-0.48341841826078,48.11354675136,0
-0.48348439225041,48.113462132037,0
-0.4835390688147,48.113373995652,0
-0.48358203188087,48.113283012991,0
-0.48361295453077,48.113189876501,0
-0.48363160148701,48.113095295016,0
-0.48363783090185,48.112999988364,0

-0.4836315954346,48.11290468189,0
-0.48361294260982,48.11281010093,0
-0.48358201445365,48.112716965297,0
-0.48353904641067,48.112625983799,0
-0.48348436555034,48.112537848848,0
-0.48341838807591,48.112453231187,0
-0.4833416161555,48.112372774788,0
-0.48325463409901,48.112297091954,0
-0.48315810391031,48.112226758652,0
-0.48305276024842,48.11216231014,0
-0.48293940483623,48.112104236887,0
-0.48281890035892,48.112052980841,0
-0.48269216389913,48.112008932073,0
-0.48256015995813,48.111972425801,0
-0.48242389311671,48.111943739843,0
-0.48228440039114,48.111923092504,0
-0.48214274334262,48.111910640913,0
-0.48199999999996,48.111906479827,0
-0.48185725665734,48.111910640913,0
-0.48171559960881,48.111923092504,0
-0.48157610688325,48.111943739843,0
-0.48143984004183,48.111972425801,0
-0.48130783610083,48.112008932073,0
-0.48118109964101,48.112052980841,0
-0.48106059516373,48.112104236887,0
-0.48094723975151,48.11216231014,0
-0.48084189608964,48.112226758652,0
-0.48074536590092,48.112297091954,0
-0.48065838384443,48.112372774788,0
-0.48058161192402,48.112453231187,0
-0.48051563444961,48.112537848848,0
-0.48046095358928,48.112625983799,0
-0.48041798554631,48.112716965297,0
-0.48038705739011,48.11281010093,0
-0.48036840456536,48.11290468189,0
-0.48036216909811,48.112999988364,0
-0.48199999999997,48.113,0
</coordinates>
</LinearRing>
</outerBoundaryIs>
</Polygon>
</Placemark>
<Placemark>
<name>483</name>
<styleUrl>#stylehap1</styleUrl>
<Polygon>
<outerBoundaryIs>
<LinearRing>
<coordinates>
-0.48199999999997,48.112,0
-0.48036220097088,48.111999988365,0
-0.4803684302646,48.112095295016,0
-0.48038707685808,48.112189876501,0
-0.48041799890629,48.112283012991,0
-0.48046096113646,48.112373995652,0
-0.48051563663679,48.112462132038,0
-0.48058160934263,48.11254675136,0

-0.48065837720147,48.112627209597,0
-0.48074535599286,48.112702894391,0
-0.48084188377357,48.112773229712,0
-0.48094722591493,48.112837680245,0
-0.4810605806936,48.112895755458,0
-0.48118108539337,48.112947013341,0
-0.4813078228715,48.11299106377,0
-0.48143982853954,48.113027571477,0
-0.48157609770577,48.113056258598,0
-0.48171559322284,48.113076906794,0
-0.48185725338297,48.113089358911,0
-0.48199999999996,48.113093520174,0
-0.48214274661696,48.113089358911,0
-0.48228440677711,48.113076906794,0
-0.48242390229419,48.113056258598,0
-0.48256017146041,48.113027571477,0
-0.48269217712845,48.11299106377,0
-0.48281891460658,48.112947013341,0
-0.48293941930635,48.112895755458,0
-0.48305277408503,48.112837680245,0
-0.48315811622636,48.112773229712,0
-0.48325464400707,48.112702894391,0
-0.48334162279846,48.112627209597,0
-0.48341839065733,48.11254675136,0
-0.48348436336314,48.112462132038,0
-0.48353903886347,48.112373995652,0
-0.48358200109366,48.112283012991,0
-0.48361292314188,48.112189876501,0
-0.48363156973533,48.112095295016,0
-0.48363779902905,48.111999988365,0
-0.48363156368325,48.11190468189,0
-0.48361291122159,48.11181010093,0
-0.48358198366738,48.111716965297,0
-0.48353901646066,48.1116259838,0
-0.48348433666452,48.111537848848,0
-0.48341836047409,48.111453231187,0
-0.48334159004775,48.111372774789,0
-0.48325460968397,48.111297091954,0
-0.48315808137379,48.111226758653,0
-0.48305273976193,48.11116231014,0
-0.48293938655565,48.111104236887,0
-0.48281888442335,48.111052980841,0
-0.48269215042983,48.111008932073,0
-0.4825601490576,48.110972425801,0
-0.4824238848679,48.110943739843,0
-0.48228439485682,48.110923092504,0
-0.48214274056487,48.110910640913,0
-0.48199999999996,48.110906479827,0
-0.48185725943506,48.110910640913,0
-0.48171560514313,48.110923092504,0
-0.48157611513205,48.110943739843,0
-0.48143985094235,48.110972425801,0
-0.48130784957012,48.111008932073,0
-0.48118111557658,48.111052980841,0
-0.48106061344431,48.111104236887,0
-0.48094726023802,48.11116231014,0
-0.48084191862616,48.111226758653,0

-0.48074539031596,48.111297091954,0
-0.4806584099522,48.111372774789,0
-0.48058163952584,48.111453231187,0
-0.48051566333544,48.111537848848,0
-0.48046098353927,48.1116259838,0
-0.48041801633258,48.111716965297,0
-0.48038708877836,48.11181010093,0
-0.48036843631668,48.11190468189,0
-0.48036220097088,48.111999988365,0
-0.48199999999997,48.112,0
</coordinates>
</LinearRing>
</outerBoundaryIs>
</Polygon>
</Placemark>
<Placemark>
<name>484</name>
<styleUrl>#stylehap1</styleUrl>
<Polygon>
<outerBoundaryIs>
<LinearRing>
<coordinates>
-0.48099999999999,48.238,0
-0.47935817126224,48.237999988313,0
-0.47936441586931,48.238095294965,0
-0.4793831083286,48.238189876451,0
-0.47941410644644,48.238283012943,0
-0.47945717437163,48.238373995607,0
-0.47951198438829,48.238462131995,0
-0.47957811940833,48.238546751322,0
-0.47965507614432,48.238627209562,0
-0.47974226893844,48.23870289436,0
-0.4798390342189,48.238773229687,0
-0.47994463554961,48.238837680224,0
-0.48005826923459,48.238895755441,0
-0.48017907043482,48.238947013328,0
-0.48030611975084,48.238991063761,0
-0.48043845022067,48.239027571471,0
-0.48057505468025,48.239056258594,0
-0.48071489343015,48.239076906793,0
-0.48085690214985,48.23908935891,0
-0.48100000000001,48.239093520174,0
-0.48114309785016,48.23908935891,0
-0.48128510656987,48.239076906793,0
-0.48142494531973,48.239056258594,0
-0.48156154977932,48.239027571471,0
-0.48169388024914,48.238991063761,0
-0.48182092956516,48.238947013328,0
-0.48194173076543,48.238895755441,0
-0.48205536445038,48.238837680224,0
-0.48216096578109,48.238773229687,0
-0.48225773106158,48.23870289436,0
-0.48234492385569,48.238627209562,0
-0.48242188059165,48.238546751322,0
-0.48248801561173,48.238462131995,0
-0.48254282562835,48.238373995607,0
-0.48258589355354,48.238283012943,0

-0.48261689167141,48.238189876451,0
-0.48263558413068,48.238095294965,0
-0.48264182873775,48.237999988313,0
-0.48263557803679,48.237904681839,0
-0.48261687966879,48.23781010088,0
-0.48258587600691,48.237716965249,0
-0.48254280307084,48.237625983754,0
-0.48248798872869,48.237537848806,0
-0.48242185019998,48.237453231148,0
-0.48234489087879,48.237372774754,0
-0.48225769650141,48.237297091924,0
-0.48216093068779,48.237226758627,0
-0.48205532989024,48.237162310119,0
-0.48194169778852,48.23710423687,0
-0.48182089917349,48.237052980829,0
-0.48169385336613,48.237008932064,0
-0.48156152722181,48.236972425795,0
-0.4814249277731,48.23694373984,0
-0.48128509456724,48.236923092503,0
-0.48114309175627,48.236910640912,0
-0.48100000000001,48.236906479827,0
-0.48085690824372,48.236910640912,0
-0.48071490543274,48.236923092503,0
-0.48057507222689,48.23694373984,0
-0.4804384727782,48.236972425795,0
-0.48030614663386,48.237008932064,0
-0.4801791008265,48.237052980829,0
-0.48005830221149,48.23710423687,0
-0.47994467010975,48.237162310119,0
-0.47983906931219,48.237226758627,0
-0.47974230349857,48.237297091924,0
-0.47965510912122,48.237372774754,0
-0.47957814980001,48.237453231148,0
-0.4795120112713,48.237537848806,0
-0.47945719692917,48.237625983754,0
-0.47941412399308,48.237716965249,0
-0.4793831203312,48.23781010088,0
-0.4793644219632,48.237904681839,0
-0.47935817126224,48.237999988313,0
-0.48099999999999,48.238,0
</coordinates>
</LinearRing>
</outerBoundaryIs>
</Polygon>
</Placemark>
<Placemark>
<name>485</name>
<styleUrl>#stylehap1</styleUrl>
<Polygon>
<outerBoundaryIs>
<LinearRing>
<coordinates>
-0.47800000000007,48.294,0
-0.47635637135318,48.29399998829,0
-0.47636262280012,48.294095294942,0
-0.4763813357458,48.294189876429,0
-0.4764123678409,48.294283012922,0

-0.47645548297578,48.294373995586,0
-0.47651035307547,48.294462131976,0
-0.47657656059477,48.294546751304,0
-0.47665360169455,48.294627209547,0
-0.4767408900751,48.294702894347,0
-0.47683776143722,48.294773229675,0
-0.4769434785374,48.294837680214,0
-0.47705723679859,48.294895755433,0
-0.47717817043372,48.294947013322,0
-0.47730535903521,48.294991063757,0
-0.47743783458095,48.295027571468,0
-0.47757458880265,48.295056258593,0
-0.47771458086085,48.295076906792,0
-0.47785674526815,48.29508935891,0
-0.47800000000008,48.295093520174,0
-0.47814325473198,48.29508935891,0
-0.47828541913928,48.295076906792,0
-0.47842541119748,48.295056258593,0
-0.47856216541918,48.295027571468,0
-0.47869464096492,48.294991063757,0
-0.47882182956641,48.294947013322,0
-0.47894276320154,48.294895755433,0
-0.47905652146273,48.294837680214,0
-0.47916223856291,48.294773229675,0
-0.47925910992503,48.294702894347,0
-0.47934639830557,48.294627209547,0
-0.47942343940536,48.294546751304,0
-0.47948964692465,48.294462131976,0
-0.47954451702435,48.294373995586,0
-0.47958763215923,48.294283012922,0
-0.47961866425433,48.294189876429,0
-0.4796373772,48.294095294942,0
-0.47964362864695,48.29399998829,0
-0.47963737108744,48.293904681816,0
-0.47961865221489,48.293810100858,0
-0.47958761455875,48.293716965228,0
-0.47954449439761,48.293625983734,0
-0.47948961995917,48.293537848787,0
-0.47942340892043,48.293453231131,0
-0.4793463652275,48.293372774739,0
-0.47925907525884,48.293297709191,0
-0.47916220336196,48.293226758615,0
-0.47905648679657,48.29316231011,0
-0.47894273012344,48.293104236862,0
-0.47882179908151,48.293052980823,0
-0.47869461399941,48.29300893206,0
-0.47856214279244,48.292972425793,0
-0.478425393597,48.292943739838,0
-0.47828540709984,48.292923092502,0
-0.47814324861939,48.292910640912,0
-0.47800000000008,48.292906479826,0
-0.47785675138074,48.292910640912,0
-0.47771459290029,48.292923092502,0
-0.47757460640313,48.292943739838,0
-0.47743785720769,48.292972425793,0
-0.47730538600072,48.29300893206,0
-0.47717820091865,48.293052980823,0

-0.47705726987669,48.293104236862,0
-0.47694351320356,48.29316231011,0
-0.47683779663817,48.293226758615,0
-0.47674092474129,48.29329709191,0
-0.47665363477265,48.293372774739,0
-0.4765765910797,48.293453231131,0
-0.47651038004095,48.293537848787,0
-0.47645550560252,48.293625983734,0
-0.47641238544138,48.293716965228,0
-0.47638134778524,48.293810100858,0
-0.47636262891269,48.293904681816,0
-0.47635637135318,48.29399998829,0
-0.47800000000007,48.294,0
</coordinates>
</LinearRing>
</outerBoundaryIs>
</Polygon>
</Placemark>
<Placemark>
<name>486</name>
<styleUrl>#stylehap1</styleUrl>
<Polygon>
<outerBoundaryIs>
<LinearRing>
<coordinates>
-0.47699999999998,48.249,0
-0.4753578181436,48.248999988309,0
-0.47536406409256,48.24909529496,0
-0.47538276057101,48.249189876447,0
-0.47541376535476,48.249283012939,0
-0.47545684254188,48.249373995603,0
-0.47551166434602,48.249462131992,0
-0.47557781358949,48.249546751318,0
-0.47565478687654,48.249627209559,0
-0.47574199842347,48.249702894358,0
-0.47583878451577,48.249773229684,0
-0.47594440855893,48.249837680222,0
-0.47605806668413,48.249895755439,0
-0.47617889386637,48.249947013327,0
-0.47630597050836,48.24999106376,0
-0.47643832944018,48.25002757147,0
-0.47657496328112,48.250056258594,0
-0.47671483210809,48.250076906792,0
-0.47685687137169,48.25008935891,0
-0.47699999999997,48.250093520174,0
-0.47714312862827,48.25008935891,0
-0.47728516789186,48.250076906792,0
-0.47742503671881,48.250056258594,0
-0.47756167055977,48.25002757147,0
-0.47769402949159,48.24999106376,0
-0.47782110613359,48.249947013327,0
-0.47794193331583,48.249895755439,0
-0.47805559144103,48.249837680222,0
-0.47816121548418,48.249773229684,0
-0.47825800157649,48.249702894358,0
-0.47834521312341,48.249627209559,0
-0.47842218641047,48.249546751318,0

-0.47848833565393,48.249462131992,0
-0.47854315745808,48.249373995603,0
-0.4785862346452,48.249283012939,0
-0.47861723942895,48.249189876447,0
-0.47863593590739,48.24909529496,0
-0.47864218185635,48.248999988309,0
-0.47863592980984,48.248904681835,0
-0.4786172274191,48.248810100876,0
-0.47858621708797,48.248716965245,0
-0.47854313488698,48.24862598375,0
-0.47848830875473,48.248537848802,0
-0.4784221560005,48.248453231145,0
-0.47834518012666,48.248372774751,0
-0.47825796699554,48.248297091921,0
-0.47816118036977,48.248226758624,0
-0.47805555686008,48.248162310117,0
-0.47794190031908,48.248104236868,0
-0.47782107572362,48.248052980827,0
-0.47769400259239,48.248008932063,0
-0.47756164798867,48.247972425795,0
-0.47742501916161,48.24794373984,0
-0.47728515588201,48.247923092503,0
-0.47714312253071,48.247910640912,0
-0.47699999999997,48.247906479826,0
-0.47685687746922,48.247910640912,0
-0.47671484411795,48.247923092503,0
-0.47657498083834,48.24794373984,0
-0.47643835201128,48.247972425795,0
-0.47630599740756,48.248008932063,0
-0.47617892427631,48.248052980827,0
-0.47605809968088,48.248104236868,0
-0.47594444313985,48.248162310117,0
-0.47583881963016,48.248226758624,0
-0.47574203300439,48.248297091921,0
-0.4756548198733,48.248372774751,0
-0.47557784399946,48.248453231145,0
-0.47551169124522,48.248537848802,0
-0.47545686511298,48.24862598375,0
-0.47541378291196,48.248716965245,0
-0.47538277258086,48.248810100876,0
-0.47536407019012,48.248904681835,0
-0.4753578181436,48.248999988309,0
-0.47699999999998,48.249,0
</coordinates>
</LinearRing>
</outerBoundaryIs>
</Polygon>
</Placemark>
<Placemark>
<name>487</name>
<styleUrl>#stylehap1</styleUrl>
<Polygon>
<outerBoundaryIs>
<LinearRing>
<coordinates>
-0.47699999999998,48.209,0
-0.47535910119397,48.208999988325,0

-0.47536534226719,48.209095294977,0
-0.47538402414208,48.209189876463,0
-0.47541500470542,48.209283012954,0
-0.47545804823947,48.209373995617,0
-0.47551282721394,48.209462132005,0
-0.4755789247769,48.209546751331,0
-0.4756558379259,48.20962720957,0
-0.47574298133483,48.209702894367,0
-0.47583969180772,48.209773229693,0
-0.47594523332556,48.209837680228,0
-0.47605880264761,48.209895755445,0
-0.47617953542475,48.209947013331,0
-0.47630651277826,48.209991063763,0
-0.47643876829391,48.210027571472,0
-0.47657529537818,48.210056258595,0
-0.47671505492058,48.210076906793,0
-0.47685698320355,48.21008935891,0
-0.47699999999997,48.210093520174,0
-0.4771430167964,48.21008935891,0
-0.47728494507938,48.210076906793,0
-0.47742470462178,48.210056258595,0
-0.47756123170605,48.210027571472,0
-0.4776934872217,48.209991063763,0
-0.4778204645752,48.209947013331,0
-0.47794119735235,48.209895755445,0
-0.47805476667437,48.209837680228,0
-0.47816030819221,48.209773229693,0
-0.47825701866512,48.209702894367,0
-0.47834416207406,48.20962720957,0
-0.47842107522306,48.209546751331,0
-0.47848717278602,48.209462132005,0
-0.47854195176048,48.209373995617,0
-0.47858499529453,48.209283012954,0
-0.47861597585788,48.209189876463,0
-0.47863465773274,48.209095294977,0
-0.47864089880599,48.208999988325,0
-0.47863465164852,48.208904681851,0
-0.47861596387429,48.208810100892,0
-0.47858497777568,48.20871696526,0
-0.47854192923867,48.208625983764,0
-0.47848714594557,48.208537848816,0
-0.47842104487952,48.208453231157,0
-0.47834412914939,48.208372774762,0
-0.47825698415972,48.208297091931,0
-0.47816027315451,48.208226758633,0
-0.47805473216899,48.208162310124,0
-0.47794116442768,48.208104236874,0
-0.47782043423167,48.208052980832,0
-0.47769346038125,48.208008932066,0
-0.47756120918426,48.207972425797,0
-0.47742468710293,48.207943739841,0
-0.47728493309578,48.207923092503,0
-0.47714301071218,48.207910640912,0
-0.47699999999997,48.207906479826,0
-0.47685698928778,48.207910640912,0
-0.47671506690417,48.207923092503,0
-0.47657531289703,48.207943739841,0

-0.47643879081569,48.207972425797,0
-0.47630653961868,48.208008932066,0
-0.47617956576829,48.208052980832,0
-0.47605883557228,48.208104236874,0
-0.47594526783097,48.208162310124,0
-0.47583972684542,48.208226758633,0
-0.47574301584024,48.208297091931,0
-0.47565587085057,48.208372774762,0
-0.47557895512043,48.208453231157,0
-0.47551285405438,48.208537848816,0
-0.47545807076129,48.208625983764,0
-0.47541502222427,48.20871696526,0
-0.47538403612567,48.208810100892,0
-0.47536534835144,48.208904681851,0
-0.47535910119397,48.208999988325,0
-0.47699999999998,48.209,0
</coordinates>
</LinearRing>
</outerBoundaryIs>
</Polygon>
</Placemark>
<Placemark>
<name>488</name>
<styleUrl>#stylehap1</styleUrl>
<Polygon>
<outerBoundaryIs>
<LinearRing>
<coordinates>
-0.47699999999998,48.253,0
-0.47535768968416,48.252999988307,0
-0.47536393612129,48.253095294959,0
-0.47538263406185,48.253189876445,0
-0.47541364127056,48.253283012937,0
-0.475456721827,48.253373995601,0
-0.4755115479193,48.25346213199,0
-0.47557770233702,48.253546751317,0
-0.47565468164512,48.253627209558,0
-0.47574190001405,48.253702894357,0
-0.47583869367737,48.253773229684,0
-0.475944325983,48.253837680221,0
-0.4760579929992,48.253895755439,0
-0.47617882963331,48.253947013327,0
-0.47630591621612,48.25399106376,0
-0.47643828550199,48.25402757147,0
-0.47657493003145,48.254056258594,0
-0.47671480980004,48.254076906792,0
-0.47685686017504,48.25408935891,0
-0.47699999999997,48.254093520174,0
-0.47714313982492,48.25408935891,0
-0.47728519019992,48.254076906792,0
-0.47742506996848,48.254056258594,0
-0.47756171449797,48.25402757147,0
-0.47769408378384,48.25399106376,0
-0.47782117036665,48.253947013327,0
-0.47794200700076,48.253895755439,0
-0.47805567401695,48.253837680221,0
-0.47816130632256,48.253773229684,0

-0.4782580999859,48.253702894357,0
-0.47834531835484,48.253627209558,0
-0.47842229766294,48.253546751317,0
-0.47848845208065,48.25346213199,0
-0.47854327817293,48.253373995601,0
-0.4785863587294,48.253283012937,0
-0.47861736593811,48.253189876445,0
-0.47863606387866,48.253095294959,0
-0.47864231031577,48.252999988307,0
-0.47863605777978,48.252904681833,0
-0.47861735392563,48.252810100874,0
-0.47858634116836,48.252716965243,0
-0.47854325559689,48.252625983749,0
-0.47848842517558,48.252537848801,0
-0.47842226724633,48.252453231144,0
-0.47834528535086,48.25237277475,0
-0.4782580653974,48.25229709192,0
-0.47816127120048,48.252226758624,0
-0.47805563942845,48.252162310117,0
-0.47794197399678,48.252104236868,0
-0.47782113995001,48.252052980827,0
-0.47769405687876,48.252008932063,0
-0.47756169192191,48.251972425795,0
-0.47742505240744,48.25194373984,0
-0.47728517818744,48.251923092502,0
-0.47714313372604,48.251910640912,0
-0.47699999999997,48.251906479826,0
-0.47685686627392,48.251910640912,0
-0.47671482181251,48.251923092502,0
-0.47657494759251,48.25194373984,0
-0.47643830807802,48.251972425795,0
-0.47630594312119,48.252008932063,0
-0.47617886004992,48.252052980827,0
-0.47605802600318,48.252104236868,0
-0.47594436057151,48.252162310117,0
-0.47583872879948,48.252226758624,0
-0.47574193460253,48.25229709192,0
-0.47565471464907,48.25237277475,0
-0.47557773275363,48.252453231144,0
-0.47551157482438,48.252537848801,0
-0.47545674440307,48.252625983749,0
-0.4754136588316,48.252716965243,0
-0.4753826460743,48.252810100874,0
-0.47536394222017,48.252904681833,0
-0.47535768968416,48.252999988307,0
-0.47699999999998,48.253,0
</coordinates>
</LinearRing>
</outerBoundaryIs>
</Polygon>
</Placemark>
<Placemark>
<name>490</name>
<styleUrl>#stylehap1</styleUrl>
<Polygon>
<outerBoundaryIs>
<LinearRing>

<coordinates>
-0.47500000000002,48.447,0
-0.47335142551536,48.446999988227,0
-0.47335769575699,48.447095294879,0
-0.47337646499568,48.447189876367,0
-0.47340759045434,48.447283012863,0
-0.47345083531325,48.447373995531,0
-0.47350587051063,48.447462131925,0
-0.47357227724526,48.447546751257,0
-0.47364955016217,48.447627209504,0
-0.47373710119761,48.44770289431,0
-0.47383426405356,48.447773229643,0
-0.47394029926819,48.447837680188,0
-0.47405439984333,48.447895755412,0
-0.47417569738648,48.447947013307,0
-0.47430326872029,48.447991063746,0
-0.47443614290953,48.448027571461,0
-0.47457330865152,48.448056258589,0
-0.47471372197442,48.44807690679,0
-0.47485631418421,48.44808935891,0
-0.47500000000002,48.448093520174,0
-0.47514368581583,48.44808935891,0
-0.47528627802562,48.44807690679,0
-0.47542669134852,48.448056258589,0
-0.47556385709052,48.448027571461,0
-0.47569673127975,48.447991063746,0
-0.47582430261357,48.447947013307,0
-0.47594560015672,48.447895755412,0
-0.47605970073185,48.447837680188,0
-0.47616573594648,48.447773229643,0
-0.47626289880244,48.44770289431,0
-0.47635044983787,48.447627209504,0
-0.47642772275478,48.447546751257,0
-0.47649412948941,48.447462131925,0
-0.47654916468679,48.447373995531,0
-0.47659240954571,48.447283012863,0
-0.47662353500436,48.447189876367,0
-0.47664230424308,48.447095294879,0
-0.47664857448469,48.446999988227,0
-0.47664229807904,48.446904681754,0
-0.47662352286357,48.446810100797,0
-0.47659239179707,48.446716965169,0
-0.4765491418696,48.446625983678,0
-0.47649410229692,48.446537848735,0
-0.47642769201327,48.446453231084,0
-0.47635041648136,48.446372774696,0
-0.47626286384445,48.446297091873,0
-0.4761657004492,48.446226758584,0
-0.47605966577387,48.446162310083,0
-0.4759455668002,48.446104236841,0
-0.47582427187203,48.446052980807,0
-0.47569670408725,48.446008932049,0
-0.47556383427332,48.445972425785,0
-0.47542667359991,48.445943739834,0
-0.47528626588484,48.4459230925,0
-0.47514367965179,48.445910640911,0
-0.47500000000002,48.445906479826,0

-0.47485632034825,48.445910640911,0
-0.47471373411521,48.4459230925,0
-0.47457332640014,48.445943739834,0
-0.47443616572673,48.445972425785,0
-0.47430329591279,48.446008932049,0
-0.47417572812801,48.446052980807,0
-0.47405443319984,48.446104236841,0
-0.47394033422617,48.446162310083,0
-0.47383429955084,48.446226758584,0
-0.47373713615559,48.446297091873,0
-0.47364958351868,48.446372774696,0
-0.4735723079868,48.446453231084,0
-0.47350589770312,48.446537848735,0
-0.47345085813045,48.446625983678,0
-0.47340760820298,48.446716965169,0
-0.47337647713647,48.446810100797,0
-0.473357701921,48.446904681754,0
-0.47335142551536,48.446999988227,0
-0.47500000000002,48.447,0
</coordinates>
</LinearRing>
</outerBoundaryIs>
</Polygon>
</Placemark>
<Placemark>
<name>495</name>
<styleUrl>#stylehap1</styleUrl>
<Polygon>
<outerBoundaryIs>
<LinearRing>
<coordinates>
-0.46900000000005,48.188,0
-0.46735977367337,48.187999988334,0
-0.46736601219111,48.188095294985,0
-0.46738468641188,48.188189876471,0
-0.46741565428069,48.188283012962,0
-0.46745868017632,48.188373995625,0
-0.46751343670266,48.188462132012,0
-0.46757950717857,48.188546751337,0
-0.46765638880769,48.188627209576,0
-0.46774349650375,48.188702894372,0
-0.4678401673426,48.188773229697,0
-0.4679456656068,48.188837680232,0
-0.46805918838487,48.188895755447,0
-0.4681798716819,48.188947013333,0
-0.46830679699577,48.188991063765,0
-0.46843899830836,48.189027571473,0
-0.46857546943876,48.189056258596,0
-0.46871517170233,48.189076906793,0
-0.46885704181753,48.18908935891,0
-0.46900000000006,48.189093520174,0
-0.46914295818257,48.18908935891,0
-0.46928482829777,48.189076906793,0
-0.46942453056134,48.189056258596,0
-0.46956100169174,48.189027571473,0
-0.46969320300433,48.188991063765,0
-0.4698201283182,48.188947013333,0

-0.46994081161523,48.188895755447,0
-0.4700543343933,48.188837680232,0
-0.4701598326575,48.188773229697,0
-0.47025650349635,48.188702894372,0
-0.47034361119244,48.188627209576,0
-0.47042049282153,48.188546751337,0
-0.47048656329744,48.188462132012,0
-0.47054131982378,48.188373995625,0
-0.47058434571941,48.188283012962,0
-0.47061531358822,48.188189876471,0
-0.47063398780899,48.188095294985,0
-0.47064022632673,48.187999988334,0
-0.47063398173174,48.18790468186,0
-0.47061530161837,48.1878101009,0
-0.47058432822066,48.187716965268,0
-0.47054129732779,48.187625983772,0
-0.47048653648778,48.187537848823,0
-0.4704204625128,48.187453231164,0
-0.47034357830553,48.187372774768,0
-0.47025646903054,48.187297091936,0
-0.47015979766,48.187226758637,0
-0.47005429992746,48.187162310128,0
-0.46994077872832,48.187104236876,0
-0.46982009800948,48.187052980834,0
-0.46969317619469,48.187008932068,0
-0.46956097919575,48.186972425798,0
-0.46942451306259,48.186943739841,0
-0.46928481632792,48.186923092503,0
-0.46914295210529,48.186910640912,0
-0.46900000000006,48.186906479826,0
-0.46885704789481,48.186910640912,0
-0.46871518367218,48.186923092503,0
-0.46857548693751,48.186943739841,0
-0.46843902080435,48.186972425798,0
-0.46830682380541,48.187008932068,0
-0.46817990199062,48.187052980834,0
-0.46805922127178,48.187104236876,0
-0.46794570007264,48.187162310128,0
-0.4678402023401,48.187226758637,0
-0.46774353096956,48.187297091936,0
-0.4676564216946,48.187372774768,0
-0.4675795374873,48.187453231164,0
-0.46751346351232,48.187537848823,0
-0.46745870267231,48.187625983772,0
-0.46741567177946,48.187716965268,0
-0.46738469838173,48.1878101009,0
-0.46736601826836,48.18790468186,0
-0.46735977367337,48.187999988334,0
-0.46900000000005,48.188,0
</coordinates>
</LinearRing>
</outerBoundaryIs>
</Polygon>
</Placemark>
<Placemark>
<name>498</name>
<styleUrl>#stylehap1</styleUrl>

<Polygon>
<outerBoundaryIs>
<LinearRing>
<coordinates>
-0.46799999999996,48.22,0
-0.46635874863473,48.219999988321,0
-0.46636499104773,48.220095294972,0
-0.46638367693541,48.220189876458,0
-0.46641466415411,48.22028301295,0
-0.4664577169354,48.220373995613,0
-0.46651250767868,48.220462132001,0
-0.46657861944254,48.220546751327,0
-0.46665554911638,48.220627209567,0
-0.46674271124843,48.220702894365,0
-0.46683944250018,48.22077322969,0
-0.46694500669449,48.220837680227,0
-0.46705860041806,48.220895755443,0
-0.46717935913601,48.22094701333,0
-0.46730636377221,48.220991063762,0
-0.46743864770476,48.221027571471,0
-0.46757520412385,48.221056258595,0
-0.4677149936957,48.221076906793,0
-0.46785695247414,48.22108935891,0
-0.46799999999995,48.221093520174,0
-0.46814304752579,48.22108935891,0
-0.46828500630422,48.221076906793,0
-0.46842479587607,48.221056258595,0
-0.46856135229516,48.221027571471,0
-0.46869363622772,48.220991063762,0
-0.46882064086392,48.22094701333,0
-0.46894139958187,48.220895755443,0
-0.46905499330544,48.220837680227,0
-0.46916055749974,48.22077322969,0
-0.4692572887515,48.220702894365,0
-0.46934445088352,48.220627209567,0
-0.46942138055738,48.220546751327,0
-0.46948749232122,48.220462132001,0
-0.46954228306452,48.220373995613,0
-0.46958533584582,48.22028301295,0
-0.46961632306452,48.220189876458,0
-0.46963500895217,48.220095294972,0
-0.46964125136517,48.219999988321,0
-0.46963500286428,48.219904681846,0
-0.4696163110737,48.219810100888,0
-0.46958531831644,48.219716965256,0
-0.46954226052917,48.219625983761,0
-0.46948746546464,48.219537848812,0
-0.46942135019561,48.219453231154,0
-0.46934441793906,48.219372774759,0
-0.46925725422536,48.219297091928,0
-0.46916052244097,48.21922675863,0
-0.46905495877927,48.219162310122,0
-0.4689413666374,48.219104236872,0
-0.46882061050211,48.21905298083,0
-0.46869360937114,48.219008932066,0
-0.46856132975981,48.218972425796,0
-0.46842477834666,48.218943739841,0

-0.46828499431343,48.218923092503,0
-0.4681430414379,48.218910640912,0
-0.46799999999995,48.218906479827,0
-0.46785695856202,48.218910640912,0
-0.4677150056865,48.218923092503,0
-0.46757522165324,48.218943739841,0
-0.46743867024011,48.218972425796,0
-0.46730639062878,48.219008932066,0
-0.46717938949779,48.21905298083,0
-0.46705863336252,48.219104236872,0
-0.46694504122065,48.219162310122,0
-0.46683947755895,48.21922675863,0
-0.46674274577457,48.219297091928,0
-0.46665558206087,48.219372774759,0
-0.46657864980432,48.219453231154,0
-0.46651253453529,48.219537848812,0
-0.46645773947075,48.219625983761,0
-0.46641468168349,48.21971696525,0
-0.46638368892623,48.219810100888,0
-0.46636499713564,48.219904681846,0
-0.46635874863473,48.219999988321,0
-0.46799999999996,48.22,0
</coordinates>
</LinearRing>
</outerBoundaryIs>
</Polygon>
</Placemark>
<Placemark>
<name>500</name>
<styleUrl>#stylehap1</styleUrl>
<Polygon>
<outerBoundaryIs>
<LinearRing>
<coordinates>
-0.46500000000003,47.837,0
-0.46337090039372,47.836999988477,0
-0.4633770966285,47.837095295127,0
-0.46339564420574,47.837189876609,0
-0.46342640203301,47.837283013096,0
-0.46346913608644,47.837373995751,0
-0.46352352118992,47.83746213213,0
-0.46358914348814,47.837546751444,0
-0.46366550359492,47.837627209672,0
-0.46375202039252,47.837702894456,0
-0.46384803545354,47.837773229768,0
-0.46395281805128,47.837837680291,0
-0.46406557072101,47.837895755494,0
-0.46418543532917,47.837947013369,0
-0.46431149960493,47.83799106379,0
-0.46444280408395,47.83802757149,0
-0.46457834941165,47.838056258605,0
-0.46471710395042,47.838076906797,0
-0.46485801163268,47.838089358912,0
-0.46500000000002,47.838093520174,0
-0.46514198836739,47.838089358912,0
-0.46528289604965,47.838076906797,0
-0.46542165058842,47.838056258605,0

-0.46555719591612,47.83802757149,0
-0.46568850039514,47.83799106379,0
-0.46581456467088,47.837947013369,0
-0.46593442927906,47.837895755494,0
-0.46604718194878,47.837837680291,0
-0.46615196454653,47.837773229768,0
-0.46624797960753,47.837702894456,0
-0.46633449640513,47.837627209672,0
-0.46641085651193,47.837546751444,0
-0.46647647881015,47.83746213213,0
-0.46653086391363,47.837373995751,0
-0.46657359796706,47.837283013096,0
-0.46660435579433,47.837189876609,0
-0.46662290337157,47.837095295127,0
-0.46662909960635,47.836999988477,0
-0.46662289740945,47.836904682001,0
-0.46660434405126,47.836810101039,0
-0.46657358079983,47.836716965402,0
-0.46653084184386,47.836625983898,0
-0.46647645250841,47.83653784894,0
-0.46641082677738,47.836453231271,0
-0.46633446414127,47.836372774864,0
-0.46624794579467,47.836297092019,0
-0.46615193021206,47.836226758708,0
-0.46604714813593,47.836162310187,0
-0.46593439701521,47.836104236923,0
-0.46581453493636,47.836052980869,0
-0.4656884740934,47.836008932093,0
-0.46555717384633,47.835972425814,0
-0.46542163342119,47.835943739851,0
-0.46528288430656,47.835923092508,0
-0.46514198240527,47.835910640913,0
-0.46500000000002,47.835906479826,0
-0.4648580175948,47.835910640913,0
-0.46471711569351,47.835923092508,0
-0.46457836657888,47.835943739851,0
-0.46444282615371,47.835972425814,0
-0.46431152590667,47.836008932093,0
-0.46418546506371,47.836052980869,0
-0.46406560298486,47.836104236923,0
-0.46395285186414,47.836162310187,0
-0.46384806978801,47.836226758708,0
-0.46375205420538,47.836297092019,0
-0.46366553585879,47.836372774864,0
-0.46358917322269,47.836453231271,0
-0.46352354749166,47.83653784894,0
-0.46346915815621,47.836625983898,0
-0.46342641920024,47.836716965402,0
-0.46339565594881,47.836810101039,0
-0.46337710259061,47.836904682001,0
-0.46337090039372,47.836999988477,0
-0.46500000000003,47.837,0
</coordinates>
</LinearRing>
</outerBoundaryIs>
</Polygon>
</Placemark>

<Placemark>
<name>504</name>
<styleUrl>#stylehap1</styleUrl>
<Polygon>
<outerBoundaryIs>
<LinearRing>
<coordinates>
-0.46199999999999,47.781,0
-0.46037265601601,47.780999988499,0
-0.46037884557915,47.78109529515,0
-0.46039737317399,47.781189876631,0
-0.46042809785998,47.781283013117,0
-0.46047078586526,47.781373995771,0
-0.460525112364,47.781462132148,0
-0.46059066394681,47.781546751461,0
-0.46066694176551,47.781627209687,0
-0.46075336532858,47.781702894469,0
-0.46084927691809,47.78177322978,0
-0.46095394659488,47.7818376803,0
-0.46106657775357,47.781895755502,0
-0.46118631318546,47.781947013375,0
-0.46131224160289,47.781991063794,0
-0.46144340457562,47.782027571492,0
-0.46157880382617,47.782056258607,0
-0.46171740882882,47.782076906798,0
-0.46185816465425,47.782089358912,0
-0.46199999999999,47.782093520174,0
-0.46214183534573,47.782089358912,0
-0.46228259117114,47.782076906798,0
-0.46242119617379,47.782056258607,0
-0.46255659542434,47.782027571492,0
-0.46268775839707,47.781991063794,0
-0.46281368681453,47.781947013375,0
-0.46293342224641,47.781895755502,0
-0.46304605340511,47.7818376803,0
-0.46315072308189,47.78177322978,0
-0.4632466346714,47.781702894469,0
-0.46333305823448,47.781627209687,0
-0.46340933605317,47.781546751461,0
-0.46347488763596,47.781462132148,0
-0.46352921413472,47.781373995771,0
-0.46357190214001,47.781283013117,0
-0.46360262682597,47.781189876631,0
-0.46362115442083,47.78109529515,0
-0.46362734398397,47.780999988499,0
-0.46362114847681,47.780904682024,0
-0.46360261511858,47.780810101061,0
-0.46357188502491,47.780716965423,0
-0.463529192132,47.780625983918,0
-0.46347486141415,47.780537848959,0
-0.46340930640898,47.780453231288,0
-0.46333302606864,47.780372774879,0
-0.46324660096126,47.780297092033,0
-0.46315068885172,47.78022675872,0
-0.46304601969497,47.780162310196,0
-0.46293339008057,47.780104236931,0
-0.46281365717033,47.780052980875,0

-0.46268773217525,47.780008932097,0
-0.46255657342162,47.779972425817,0
-0.46242117905872,47.779943739852,0
-0.46228257946374,47.779923092508,0
-0.46214182940171,47.779910640914,0
-0.46199999999999,47.779906479827,0
-0.46185817059825,47.779910640914,0
-0.46171742053624,47.779923092508,0
-0.46157882094127,47.779943739852,0
-0.46144342657836,47.779972425817,0
-0.46131226782473,47.780008932097,0
-0.46118634282965,47.780052980875,0
-0.46106660991941,47.780104236931,0
-0.46095398030502,47.780162310196,0
-0.46084931114827,47.78022675872,0
-0.46075339903872,47.780297092033,0
-0.46066697393135,47.780372774879,0
-0.460590693591,47.780453231288,0
-0.46052513858584,47.780537848959,0
-0.46047080786799,47.780625983918,0
-0.46042811497508,47.780716965423,0
-0.4603973848814,47.780810101061,0
-0.46037885152315,47.780904682024,0
-0.46037265601601,47.780999988499,0
-0.46199999999999,47.781,0
</coordinates>
</LinearRing>
</outerBoundaryIs>
</Polygon>
</Placemark>
<Placemark>
<name>510</name>
<styleUrl>#stylehap1</styleUrl>
<Polygon>
<outerBoundaryIs>
<LinearRing>
<coordinates>
-0.452,48.228,0
-0.4503584920949,48.227999988317,0
-0.45036473548277,48.228095294969,0
-0.45038342429036,48.228189876455,0
-0.45041441635181,48.228283012947,0
-0.45045747586189,48.22837399561,0
-0.45051227516878,48.228462131999,0
-0.45057839726588,48.228546751325,0
-0.45065533896407,48.228627209565,0
-0.45074251471996,48.228702894363,0
-0.45083926109146,48.228773229689,0
-0.45094484178633,48.228837680225,0
-0.45105845326564,48.228895755442,0
-0.45117923085947,48.228947013329,0
-0.45130625534793,48.228991063762,0
-0.45143855995806,48.229027571471,0
-0.45157513772267,48.229056258595,0
-0.45171494914543,48.229076906793,0
-0.45185693011391,48.22908935891,0
-0.45199999999999,48.229093520174,0

-0.45214306988607,48.22908935891,0
-0.45228505085458,48.229076906793,0
-0.45242486227734,48.229056258595,0
-0.45256144004192,48.229027571471,0
-0.45269374465206,48.228991063762,0
-0.45282076914054,48.228947013329,0
-0.45294154673435,48.228895755442,0
-0.45305515821368,48.228837680225,0
-0.45316073890855,48.228773229689,0
-0.45325748528005,48.228702894363,0
-0.45334466103594,48.228627209565,0
-0.45342160273411,48.228546751325,0
-0.45348772483123,48.228462131999,0
-0.45354252413812,48.22837399561,0
-0.4535855836482,48.228283012947,0
-0.45361657570963,48.228189876455,0
-0.45363526451721,48.228095294969,0
-0.45364150790508,48.227999988317,0
-0.45363525842665,48.227904681843,0
-0.45361656371357,48.227810100884,0
-0.45358556611113,48.227716965253,0
-0.45354250159292,48.227625983758,0
-0.4534876979629,48.227537848809,0
-0.45342157235905,48.227453231151,0
-0.45334462807705,48.227372774757,0
-0.4532574507388,48.227297091926,0
-0.45316070383444,48.227226758629,0
-0.45305512367242,48.227162310121,0
-0.45294151377545,48.227104236871,0
-0.45282073876545,48.22705298083,0
-0.45269371778375,48.227008932065,0
-0.45256141749673,48.226972425796,0
-0.45242484474028,48.22694373984,0
-0.45228503885852,48.226923092503,0
-0.45214306379551,48.226910640912,0
-0.45199999999999,48.226906479827,0
-0.45185693620447,48.226910640912,0
-0.45171496114147,48.226923092503,0
-0.45157515525971,48.22694373984,0
-0.45143858250326,48.226972425796,0
-0.45130628221626,48.227008932065,0
-0.45117926123453,48.22705298083,0
-0.45105848622453,48.227104236871,0
-0.45094487632759,48.227162310121,0
-0.45083929616557,48.227226758629,0
-0.45074254926121,48.227297091926,0
-0.45065537192294,48.227372774757,0
-0.45057842764094,48.227453231151,0
-0.45051230203709,48.227537848809,0
-0.45045749840709,48.227625983758,0
-0.45041443388886,48.227716965253,0
-0.45038343628642,48.227810100884,0
-0.45036474157333,48.227904681843,0
-0.4503584920949,48.227999988317,0
-0.452,48.228,0
</coordinates>
</LinearRing>

</outerBoundaryIs>
</Polygon>
</Placemark>
<Placemark>
<name>513</name>
<styleUrl>#stylehap1</styleUrl>
<Polygon>
<outerBoundaryIs>
<LinearRing>
<coordinates>
-0.44900000000007,47.81,0
-0.44737174752135,47.809999988488,0
-0.44737794053692,47.810095295138,0
-0.44739647847219,47.81018987662,0
-0.44742722030804,47.810283013106,0
-0.4474699321422,47.810373995761,0
-0.44752428896757,47.810462132139,0
-0.44758987714401,47.810546751452,0
-0.44766619754492,47.810627209679,0
-0.44775266935474,47.810702894463,0
-0.44784863448841,47.810773229774,0
-0.44795336259923,47.810837680296,0
-0.44806605663711,47.810895755498,0
-0.44818585891482,47.810947013372,0
-0.44831185763584,47.810991063792,0
-0.44844309383486,47.811027571491,0
-0.44857856867702,47.811056258606,0
-0.44871725106122,47.811076906798,0
-0.44885808546912,47.811089358912,0
-0.44900000000006,47.811093520174,0
-0.44914191453103,47.811089358912,0
-0.44928274893891,47.811076906798,0
-0.44942143132311,47.811056258606,0
-0.44955690616527,47.811027571491,0
-0.44968814236428,47.810991063792,0
-0.44981414108533,47.810947013372,0
-0.44993394336301,47.810895755498,0
-0.45004663740092,47.810837680296,0
-0.45015136551172,47.810773229774,0
-0.45024733064538,47.810702894463,0
-0.45033380245521,47.810627209679,0
-0.45041012285614,47.810546751452,0
-0.45047571103256,47.810462132139,0
-0.45053006785793,47.810373995761,0
-0.45057277969208,47.810283013106,0
-0.45060352152793,47.81018987662,0
-0.45062205946321,47.810095295138,0
-0.45062825247878,47.809999988488,0
-0.45062205350984,47.809904682012,0
-0.45060350980207,47.809810101049,0
-0.45057276255001,47.809716965412,0
-0.4505300458205,47.809625983908,0
-0.4504756847694,47.809537848949,0
-0.4504100931652,47.809453231279,0
-0.45033377023866,47.809372774871,0
-0.45024729688212,47.809297092026,0
-0.4501513312276,47.809226758714,0

-0.45004660363763,47.809162310191,0
-0.44993391114646,47.809104236927,0
-0.4498141113944,47.809052980872,0
-0.44968811610112,47.809008932095,0
-0.44955688412784,47.808972425816,0
-0.44942141418104,47.808943739852,0
-0.44928273721304,47.808923092508,0
-0.44914190857764,47.808910640913,0
-0.44900000000006,47.808906479827,0
-0.44885809142249,47.808910640913,0
-0.44871726278708,47.808923092508,0
-0.44857858581909,47.808943739852,0
-0.44844311587229,47.808972425816,0
-0.44831188389903,47.809008932095,0
-0.44818588860575,47.809052980872,0
-0.44806608885369,47.809104236927,0
-0.44795339636253,47.809162310191,0
-0.44784866877255,47.809226758714,0
-0.44775270311804,47.809297092026,0
-0.44766622976147,47.809372774871,0
-0.44758990683495,47.809453231279,0
-0.44752431523076,47.809537848949,0
-0.44746995417963,47.809625983908,0
-0.44742723745011,47.809716965412,0
-0.44739649019806,47.809810101049,0
-0.44737794649031,47.809904682012,0
-0.44737174752135,47.809999988488,0
-0.44900000000007,47.81,0
</coordinates>
</LinearRing>
</outerBoundaryIs>
</Polygon>
</Placemark>
<Placemark>
<name>514</name>
<styleUrl>#stylehap1</styleUrl>
<Polygon>
<outerBoundaryIs>
<LinearRing>
<coordinates>
-0.44600000000003,48.203,0
-0.44435929340971,48.202999988328,0
-0.4443655337525,48.203095294979,0
-0.4443842134396,48.203189876465,0
-0.44441519037447,48.203283012957,0
-0.44445822886691,48.203373995619,0
-0.44451300142497,48.203462132007,0
-0.4445790912456,48.203546751332,0
-0.44465599538523,48.203627209572,0
-0.44474312858631,48.203702894369,0
-0.44483982773055,48.203773229694,0
-0.44494535688514,48.20383768023,0
-0.44505891290346,48.203895755445,0
-0.44517963153762,48.203947013332,0
-0.44530659401656,48.203991063764,0
-0.4454388340393,48.204027571472,0
-0.44557534513019,48.204056258595,0

-0.44571508830049,48.204076906793,0
-0.44585699995731,48.20408935891,0
-0.44600000000003,48.204093520174,0
-0.44614300004276,48.20408935891,0
-0.44628491169955,48.204076906793,0
-0.44642465486985,48.204056258595,0
-0.44656116596077,48.204027571472,0
-0.44669340598348,48.203991063764,0
-0.44682036846242,48.203947013332,0
-0.44694108709658,48.203895755445,0
-0.4470546431149,48.20383768023,0
-0.44716017226949,48.203773229694,0
-0.44725687141375,48.203702894369,0
-0.44734400461481,48.203627209572,0
-0.44742090875444,48.203546751332,0
-0.44748699857507,48.203462132007,0
-0.44754177113316,48.203373995619,0
-0.4475848096256,48.203283012957,0
-0.44761578656044,48.203189876465,0
-0.44763446624754,48.203095294979,0
-0.44764070659033,48.202999988328,0
-0.4476344601653,48.202904681853,0
-0.44761577458077,48.202810100894,0
-0.4475847921125,48.202716965263,0
-0.44754174861873,48.202625983767,0
-0.44748697174343,48.202537848818,0
-0.44742087842086,48.202453231159,0
-0.44734397170093,48.202372774764,0
-0.44725683691964,48.202297091932,0
-0.44716013724329,48.202226758634,0
-0.44705460862082,48.202162310125,0
-0.44694105418272,48.202104236874,0
-0.44682033812883,48.202052980832,0
-0.44669337915184,48.202008932067,0
-0.44656114344636,48.201972425797,0
-0.44642463735675,48.201943739841,0
-0.44628489971987,48.201923092503,0
-0.44614299396052,48.201910640912,0
-0.44600000000003,48.201906479826,0
-0.44585700603954,48.201910640912,0
-0.44571510028017,48.201923092503,0
-0.44557536264329,48.201943739841,0
-0.4454388565537,48.201972425797,0
-0.4453066208482,48.202008932067,0
-0.44517966187121,48.202052980832,0
-0.44505894581734,48.202104236874,0
-0.44494539137925,48.202162310125,0
-0.44483986275678,48.202226758634,0
-0.4447431630804,48.202297091932,0
-0.44465602829911,48.202372774764,0
-0.44457912157921,48.202453231159,0
-0.44451302825661,48.202537848818,0
-0.44445825138131,48.202625983767,0
-0.44441520788757,48.202716965263,0
-0.44438422541927,48.202810100894,0
-0.44436553983474,48.202904681853,0
-0.44435929340971,48.202999988328,0

-0.44600000000003,48.203,0
</coordinates>
</LinearRing>
</outerBoundaryIs>
</Polygon>
</Placemark>
<Placemark>
<name>515</name>
<styleUrl>#stylehap1</styleUrl>
<Polygon>
<outerBoundaryIs>
<LinearRing>
<coordinates>
-0.44600000000003,48.219,0
-0.4443587806944,48.218999988321,0
-0.44436502298557,48.219095294973,0
-0.44438370850835,48.219189876459,0
-0.44441469512182,48.21928301295,0
-0.44445774706226,48.219373995614,0
-0.44451253673535,48.219462132002,0
-0.44457864720785,48.219546751327,0
-0.44465557537904,48.219627209567,0
-0.4447427358085,48.219702894365,0
-0.44483946517075,48.21977322969,0
-0.44494502730301,48.219837680227,0
-0.44505861880765,48.219895755443,0
-0.4451793751667,48.21994701333,0
-0.44530637732199,48.219991063763,0
-0.44543865867048,48.220027571472,0
-0.44557521242203,48.220056258595,0
-0.44571499926318,48.220076906793,0
-0.44585695526855,48.22008935891,0
-0.44600000000003,48.220093520174,0
-0.44614304473151,48.22008935891,0
-0.44628500073689,48.220076906793,0
-0.44642478757801,48.220056258595,0
-0.44656134132958,48.220027571472,0
-0.44669362267808,48.219991063763,0
-0.44682062483334,48.21994701333,0
-0.44694138119242,48.219895755443,0
-0.44705497269703,48.219837680227,0
-0.44716053482929,48.21977322969,0
-0.44725726419154,48.219702894365,0
-0.447344424621,48.219627209567,0
-0.44742135279219,48.219546751327,0
-0.44748746326469,48.219462132002,0
-0.44754225293781,48.219373995614,0
-0.44758530487822,48.21928301295,0
-0.44761629149172,48.219189876459,0
-0.44763497701447,48.219095294973,0
-0.44764121930564,48.218999988321,0
-0.44763497092692,48.218904681847,0
-0.44761627950156,48.218810100888,0
-0.4475852873498,48.218716965256,0
-0.44754223040368,48.218625983761,0
-0.44748743640959,48.218537848812,0
-0.44742132243207,48.218453231154,0

-0.44734439167834,48.218372774759,0
-0.44725722966728,48.218297091928,0
-0.44716049977243,48.218226758631,0
-0.44705493817277,48.218162310122,0
-0.44694134824973,48.218104236872,0
-0.44682059447322,48.218052980831,0
-0.44669359582295,48.218008932066,0
-0.44656131879546,48.217972425796,0
-0.44642477004959,48.217943739841,0
-0.44628498874673,48.217923092503,0
-0.44614303864393,48.217910640912,0
-0.44600000000003,48.217906479827,0
-0.44585696135611,48.217910640912,0
-0.44571501125334,48.217923092503,0
-0.44557522995047,48.217943739841,0
-0.44543868120459,48.217972425796,0
-0.44530640417709,48.218008932066,0
-0.44517940552682,48.218052980831,0
-0.44505865175031,48.218104236872,0
-0.44494506182727,48.218162310122,0
-0.44483950022761,48.218226758631,0
-0.44474277033278,48.218297091928,0
-0.44465560832173,48.218372774759,0
-0.44457867756797,48.218453231154,0
-0.44451256359047,48.218537848812,0
-0.44445776959636,48.218625983761,0
-0.44441471265027,48.218716965256,0
-0.44438372049851,48.218810100888,0
-0.44436502907313,48.218904681847,0
-0.4443587806944,48.218999988321,0
-0.44600000000003,48.219,0
</coordinates>
</LinearRing>
</outerBoundaryIs>
</Polygon>
</Placemark>
<Placemark>
<name>516</name>
<styleUrl>#stylehap1</styleUrl>
<Polygon>
<outerBoundaryIs>
<LinearRing>
<coordinates>
-0.44399999999996,48.218,0
-0.44235881275218,48.217999988321,0
-0.44236505492152,48.218095294973,0
-0.44238374007943,48.218189876459,0
-0.44241472608774,48.218283012951,0
-0.44245777718733,48.218373995614,0
-0.4425125657903,48.218462132002,0
-0.44257867497153,48.218546751328,0
-0.44265560164012,48.218627209568,0
-0.44274276036712,48.218702894365,0
-0.44283948783997,48.218773229691,0
-0.44294504791026,48.218837680227,0
-0.44305863719609,48.218895755443,0
-0.44317939119639,48.21894701333,0

-0.44330639087088,48.218991063763,0
-0.44343866963544,48.219027571472,0
-0.44357522071964,48.219056258595,0
-0.44371500483023,48.219076906793,0
-0.44385695806266,48.21908935891,0
-0.44399999999996,48.219093520174,0
-0.44414304193724,48.21908935891,0
-0.4442849951697,48.219076906793,0
-0.44442477928028,48.219056258595,0
-0.44456133036446,48.219027571472,0
-0.44469360912905,48.218991063763,0
-0.44482060880353,48.21894701333,0
-0.44494136280383,48.218895755443,0
-0.44505495208964,48.218837680227,0
-0.44516051215996,48.218773229691,0
-0.44525723963281,48.218702894365,0
-0.4453443983598,48.218627209568,0
-0.4454213250284,48.218546751328,0
-0.44548743420962,48.218462132002,0
-0.44554222281259,48.218373995614,0
-0.44558527391216,48.218283012951,0
-0.44561625992049,48.218189876459,0
-0.4456349450784,48.218095294973,0
-0.44564118724774,48.217999988321,0
-0.44563493899117,48.217904681847,0
-0.445616247931,48.217810100888,0
-0.44558525638471,48.217716965257,0
-0.44554220027971,48.217625983761,0
-0.44548740735597,48.217537848813,0
-0.44542129466993,48.217453231154,0
-0.44534436541892,48.21737277476,0
-0.44525720511043,48.217297091928,0
-0.445160477105,48.217226758631,0
-0.44505491756726,48.217162310122,0
-0.44494132986295,48.217104236872,0
-0.44482057844506,48.217052980831,0
-0.4446935822754,48.217008932066,0
-0.44456130783158,48.216972425796,0
-0.44442476175281,48.216943739841,0
-0.4442849831802,48.216923092503,0
-0.44414303585001,48.216910640912,0
-0.44399999999996,48.216906479826,0
-0.44385696414991,48.216910640912,0
-0.44371501681972,48.216923092503,0
-0.44357523824709,48.216943739841,0
-0.44343869216832,48.216972425796,0
-0.44330641772453,48.217008932066,0
-0.44317942155486,48.217052980831,0
-0.44305867013697,48.217104236872,0
-0.44294508243266,48.217162310122,0
-0.4428395228949,48.217226758631,0
-0.44274279488949,48.217297091928,0
-0.442655634581,48.21737277476,0
-0.44257870533,48.217453231154,0
-0.44251259264393,48.217537848813,0
-0.44245779972021,48.217625983761,0
-0.44241474361521,48.217716965257,0

-0.44238375206893,48.217810100888,0
-0.44236506100875,48.217904681847,0
-0.44235881275218,48.217999988321,0
-0.44399999999996,48.218,0
</coordinates>
</LinearRing>
</outerBoundaryIs>
</Polygon>
</Placemark>
<Placemark>
<name>517</name>
<styleUrl>#stylehap1</styleUrl>
<Polygon>
<outerBoundaryIs>
<LinearRing>
<coordinates>
-0.44399999999996,48.182,0
-0.44235996566855,48.181999988336,0
-0.44236620345666,48.182095294988,0
-0.44238487549216,48.182189876473,0
-0.44241583973664,48.182283012965,0
-0.44245886059647,48.182373995627,0
-0.44251361071376,48.182462132014,0
-0.44257967345621,48.182546751339,0
-0.44265654608629,48.182627209578,0
-0.44274364358621,48.182702894374,0
-0.44284030310939,48.182773229698,0
-0.44294578902454,48.182837680233,0
-0.44305929851412,48.182895755448,0
-0.4431799676844,48.182947013334,0
-0.44330687814078,48.182991063765,0
-0.44343906397821,48.183027571473,0
-0.44357551913356,48.183056258596,0
-0.44371520504383,48.183076906793,0
-0.44385705855193,48.183089358911,0
-0.44399999999996,48.183093520174,0
-0.44414294144799,48.183089358911,0
-0.4442847949561,48.183076906793,0
-0.44442448086634,48.183056258596,0
-0.44456093602171,48.183027571473,0
-0.44469312185914,48.182991063765,0
-0.44482003231553,48.182947013334,0
-0.4449407014858,48.182895755448,0
-0.44505421097536,48.182837680233,0
-0.44515969689054,48.182773229698,0
-0.44525635641371,48.182702894374,0
-0.44534345391363,48.182627209578,0
-0.44542032654371,48.182546751339,0
-0.44548638928616,48.182462132014,0
-0.44554113940346,48.182373995627,0
-0.44558416026326,48.182283012965,0
-0.44561512450776,48.182189876473,0
-0.44563379654324,48.182095294988,0
-0.44564003433137,48.181999988336,0
-0.44563379046799,48.181904681862,0
-0.44561511254183,48.181810100903,0
-0.44558414277023,48.181716965271,0

-0.44554111691487,48.181625983774,0
-0.44548636248528,48.181537848825,0
-0.44542029624491,48.181453231166,0
-0.44534342103751,48.181372774769,0
-0.44525632195917,48.181297091937,0
-0.44515966190449,48.181226758638,0
-0.44505417652084,48.181162310129,0
-0.44494066860966,48.181104236877,0
-0.44482000201672,48.181052980834,0
-0.44469309505829,48.181008932068,0
-0.4445609135331,48.180972425798,0
-0.44442446337332,48.180943739842,0
-0.44428478299016,48.180923092503,0
-0.44414293537273,48.180910640912,0
-0.44399999999996,48.180906479826,0
-0.4438570646272,48.180910640912,0
-0.44371521700976,48.180923092503,0
-0.44357553662658,48.180943739842,0
-0.4434390864668,48.180972425798,0
-0.44330690494164,48.181008932068,0
-0.4431799979832,48.181052980834,0
-0.44305933139027,48.181104236877,0
-0.44294582347908,48.181162310129,0
-0.44284033809544,48.181226758638,0
-0.44274367804073,48.181297091937,0
-0.44265657896242,48.181372774769,0
-0.44257970375502,48.181453231166,0
-0.44251363751464,48.181537848825,0
-0.44245888308505,48.181625983774,0
-0.44241585722967,48.181716965271,0
-0.4423848874581,48.181810100903,0
-0.44236620953193,48.181904681862,0
-0.44235996566855,48.181999988336,0
-0.44399999999996,48.182,0
</coordinates>
</LinearRing>
</outerBoundaryIs>
</Polygon>
</Placemark>
<Placemark>
<name>519</name>
<styleUrl>#stylehap1</styleUrl>
<Polygon>
<outerBoundaryIs>
<LinearRing>
<coordinates>
-0.43899999999996,48.187,0
-0.43735980567685,48.186999988334,0
-0.43736604407299,48.187095294986,0
-0.43738471792949,48.187189876471,0
-0.43741568519418,48.187283012963,0
-0.4374587102504,48.187373995625,0
-0.43751346570841,48.187462132013,0
-0.43757953489522,48.187546751337,0
-0.43765641502429,48.187627209576,0
-0.43774352102076,48.187702894373,0
-0.43784018997342,48.187773229697,0

-0.43794568617917,48.187837680232,0
-0.43805920674217,48.187895755448,0
-0.43817988768441,48.187947013333,0
-0.43830681052171,48.187991063765,0
-0.43843900925476,48.188027571473,0
-0.43857547772228,48.188056258596,0
-0.43871517725993,48.188076906793,0
-0.43885704460693,48.18808935891,0
-0.43899999999996,48.188093520173,0
-0.43914295539302,48.18808935891,0
-0.43928482274,48.188076906793,0
-0.43942452227765,48.188056258596,0
-0.43956099074516,48.188027571473,0
-0.43969318947822,48.187991063765,0
-0.43982011231551,48.187947013333,0
-0.43994079325776,48.187895755448,0
-0.44005431382076,48.187837680232,0
-0.44015981002651,48.187773229697,0
-0.44025647897916,48.187702894373,0
-0.44034358497564,48.187627209576,0
-0.4404204651047,48.187546751337,0
-0.44048653429155,48.187462132013,0
-0.44054128974955,48.187373995625,0
-0.44058431480575,48.187283012963,0
-0.44061528207043,48.187189876471,0
-0.44063395592694,48.187095294986,0
-0.44064019432307,48.186999988334,0
-0.44063394985002,48.18690468186,0
-0.44061527010124,48.186810100901,0
-0.44058429730796,48.186716965269,0
-0.44054126725481,48.186625983772,0
-0.44048650748334,48.186537848823,0
-0.44042043479763,48.186453231164,0
-0.44034355209053,48.186372774768,0
-0.44025644451521,48.186297091936,0
-0.44015977503091,48.186226758637,0
-0.4400542793568,48.186162310128,0
-0.43994076037263,48.186104236877,0
-0.43982008200841,48.186052980834,0
-0.43969316267003,48.186008932068,0
-0.43956096825042,48.185972425798,0
-0.43942450477984,48.185943739841,0
-0.43928481077078,48.185923092503,0
-0.4391429493161,48.185910640912,0
-0.43899999999996,48.185906479826,0
-0.43885705068385,48.185910640912,0
-0.43871518922914,48.185923092503,0
-0.43857549522009,48.185943739841,0
-0.4384390317495,48.185972425798,0
-0.43830683732989,48.186008932068,0
-0.43817991799151,48.186052980834,0
-0.4380592396273,48.186104236877,0
-0.43794572064312,48.186162310128,0
-0.43784022496901,48.186226758637,0
-0.43774355548472,48.186297091936,0
-0.43765644790939,48.186372774768,0
-0.43757956520229,48.186453231164,0

-0.43751349251659,48.186537848823,0
-0.43745873274514,48.186625983772,0
-0.43741570269196,48.186716965269,0
-0.43738472989868,48.186810100901,0
-0.43736605014991,48.18690468186,0
-0.43735980567685,48.186999988334,0
-0.43899999999996,48.187,0
</coordinates>
</LinearRing>
</outerBoundaryIs>
</Polygon>
</Placemark>
<Placemark>
<name>521</name>
<styleUrl>#stylehap1</styleUrl>
<Polygon>
<outerBoundaryIs>
<LinearRing>
<coordinates>
-0.437999999999999,48.299,0
-0.43635621037862,48.298999988288,0
-0.43636246243727,48.29909529494,0
-0.43638117721515,48.299189876427,0
-0.436412212349,48.29928301292,0
-0.43645533170608,48.299373995585,0
-0.43651020717926,48.299462131975,0
-0.43657642118251,48.299546751303,0
-0.43665346982739,48.299627209545,0
-0.43674076675668,48.299702894346,0
-0.43683764760616,48.299773229674,0
-0.43694337506016,48.299837680213,0
-0.43705714446282,48.299895755432,0
-0.43717808994223,48.299947013322,0
-0.43730529100068,48.299991063757,0
-0.43743777952127,48.300027571468,0
-0.4375745471369,48.300056258593,0
-0.43771455290619,48.300076906792,0
-0.43785673123738,48.30008935891,0
-0.43799999999998,48.300093520174,0
-0.43814326876258,48.30008935891,0
-0.43828544709376,48.300076906792,0
-0.43842545286308,48.300056258593,0
-0.43856222047871,48.300027571468,0
-0.4386947089993,48.299991063757,0
-0.43882191005775,48.299947013322,0
-0.43894285553716,48.299895755432,0
-0.43905662493982,48.299837680213,0
-0.43916235239382,48.299773229674,0
-0.4392592332433,48.299702894346,0
-0.4393465301726,48.299627209545,0
-0.43942357881745,48.299546751303,0
-0.43948979282072,48.299462131975,0
-0.4395446682939,48.299373995585,0
-0.43958778765098,48.29928301292,0
-0.43961882278483,48.299189876427,0
-0.43963753756271,48.29909529494,0
-0.43964378962136,48.298999988288,0

-0.43963753144846,48.298904681814,0
-0.4396188107421,48.298810100856,0
-0.43958777004569,48.298716965226,0
-0.43954464566097,48.298625983732,0
-0.43948976584784,48.298537848785,0
-0.4394235483242,48.29845323113,0
-0.43934649708546,48.298372774737,0
-0.43925919856765,48.298297091909,0
-0.43916231718323,48.298226758614,0
-0.43905659026416,48.298162310109,0
-0.43894282245003,48.298104236861,0
-0.4388218795645,48.298052980822,0
-0.43869468202642,48.29800893206,0
-0.43856219784578,48.297972425792,0
-0.43842543525779,48.297943739838,0
-0.43828543505104,48.297923092502,0
-0.43814326264833,48.297910640912,0
-0.43799999999998,48.297906479826,0
-0.43785673735165,48.297910640912,0
-0.43771456494894,48.297923092502,0
-0.43757456474219,48.297943739838,0
-0.4374378021542,48.297972425792,0
-0.43730531797357,48.29800893206,0
-0.43717812043548,48.298052980822,0
-0.43705717754995,48.298104236861,0
-0.43694340973582,48.298162310109,0
-0.43683768281676,48.298226758614,0
-0.43674080143233,48.298297091909,0
-0.43665350291452,48.298372774737,0
-0.43657645167578,48.29845323113,0
-0.43651023415215,48.298537848785,0
-0.43645535433901,48.298625983732,0
-0.43641222995429,48.298716965226,0
-0.43638118925788,48.298810100856,0
-0.43636246855152,48.298904681814,0
-0.43635621037862,48.298999988288,0
-0.43799999999999,48.299,0
</coordinates>
</LinearRing>
</outerBoundaryIs>
</Polygon>
</Placemark>
<Placemark>
<name>522</name>
<styleUrl>#stylehap1</styleUrl>
<Polygon>
<outerBoundaryIs>
<LinearRing>
<coordinates>
-0.43700000000001,48.303,0
-0.43535608156734,48.302999988287,0
-0.43536233411547,48.303095294938,0
-0.43538105035948,48.303189876425,0
-0.43541208792493,48.303283012918,0
-0.43545521066057,48.303373995583,0
-0.43551009043363,48.303462131973,0
-0.43557630962533,48.303546751302,0

-0.43565336430772,48.303627209544,0
-0.4357406680777,48.303702894345,0
-0.43583755651898,48.303773229673,0
-0.43594329225808,48.303837680213,0
-0.43605707057608,48.303895755432,0
-0.43617802553324,48.303947013321,0
-0.43630523655975,48.303991063756,0
-0.43643773546274,48.304027571467,0
-0.43657451379618,48.304056258592,0
-0.43671453053706,48.304076906792,0
-0.43685672001008,48.30408935891,0
-0.43700000000002,48.304093520174,0
-0.43714327998993,48.30408935891,0
-0.43728546946298,48.304076906792,0
-0.43742548620386,48.304056258592,0
-0.43756226453727,48.304027571467,0
-0.43769476344028,48.303991063756,0
-0.43782197446677,48.303947013321,0
-0.43794292942393,48.303895755432,0
-0.43805670774196,48.303837680213,0
-0.43816244348106,48.303773229673,0
-0.43825933192231,48.303702894345,0
-0.4383466356923,48.303627209544,0
-0.43842369037468,48.303546751302,0
-0.43848990956638,48.303462131973,0
-0.43854478933944,48.303373995583,0
-0.43858791207511,48.303283012918,0
-0.43861894964056,48.303189876425,0
-0.43863766588454,48.303095294938,0
-0.4386439184327,48.302999988287,0
-0.43863765976895,48.302904681813,0
-0.43861893759518,48.302810100854,0
-0.43858789446595,48.302716965224,0
-0.43854476670155,48.30262598373,0
-0.43848988258762,48.302537848784,0
-0.43842365987474,48.302453231128,0
-0.43834660259791,48.302372774736,0
-0.43825929723905,48.302297091908,0
-0.43816240826275,48.302226758613,0
-0.4380566730587,48.302162310108,0
-0.43794289632955,48.302104236861,0
-0.43782194396683,48.302052980822,0
-0.4376947364615,48.302008932059,0
-0.4375622418994,48.301972425792,0
-0.4374254685947,48.301943739838,0
-0.4372854574176,48.301923092502,0
-0.43714327387433,48.301910640912,0
-0.43700000000002,48.301906479826,0
-0.43685672612568,48.301910640912,0
-0.43671454258243,48.301923092502,0
-0.43657453140533,48.301943739838,0
-0.43643775810063,48.301972425792,0
-0.43630526353851,48.302008932059,0
-0.43617805603318,48.302052980822,0
-0.43605710367046,48.302104236861,0
-0.43594332694131,48.302162310108,0
-0.43583759173726,48.302226758613,0

-0.43574070276096,48.302297091908,0
-0.4356533974021,48.302372774736,0
-0.43557634012527,48.302453231128,0
-0.43551011741242,48.302537848784,0
-0.43545523329846,48.30262598373,0
-0.43541210553406,48.302716965224,0
-0.43538106240483,48.302810100854,0
-0.43536234023106,48.302904681813,0
-0.43535608156734,48.302999988287,0
-0.43700000000001,48.303,0
</coordinates>
</LinearRing>
</outerBoundaryIs>
</Polygon>
</Placemark>
<Placemark>
<name>523</name>
<styleUrl>#stylehap1</styleUrl>
<Polygon>
<outerBoundaryIs>
<LinearRing>
<coordinates>
-0.43100000000004,48.193,0
-0.42935961362921,48.192999988332,0
-0.42936585275514,48.193095294983,0
-0.42938452879753,48.193189876469,0
-0.42941549968753,48.19328301296,0
-0.42945852978094,48.193373995623,0
-0.42951329164972,48.193462132011,0
-0.4295793685721,48.193546751335,0
-0.4296562577027,48.193627209575,0
-0.4297433738981,48.193702894371,0
-0.4298400541695,48.193773229696,0
-0.42994556272771,48.193837680231,0
-0.43005909658281,48.193895755447,0
-0.43017979165568,48.193947013333,0
-0.43030672935451,48.193991063764,0
-0.43043894356696,48.194027571473,0
-0.43057542801389,48.194056258596,0
-0.43071514390931,48.194076906793,0
-0.43085702786795,48.19408935891,0
-0.43100000000003,48.194093520173,0
-0.43114297213215,48.19408935891,0
-0.43128485609076,48.194076906793,0
-0.4314245719862,48.194056258596,0
-0.43156105643313,48.194027571473,0
-0.43169327064556,48.193991063764,0
-0.43182020834442,48.193947013333,0
-0.43194090341726,48.193895755447,0
-0.43205443727238,48.193837680231,0
-0.43215994583059,48.193773229696,0
-0.43225662610197,48.193702894371,0
-0.43234374229739,48.193627209575,0
-0.43242063142796,48.193546751335,0
-0.43248670835035,48.193462132011,0
-0.43254147021913,48.193373995623,0
-0.43258450031256,48.19328301296,0

-0.43261547120257,48.193189876469,0
-0.43263414724493,48.193095294983,0
-0.43264038637086,48.192999988332,0
-0.43263414116603,48.192904681857,0
-0.43261545922943,48.192810100898,0
-0.43258448280903,48.192716965266,0
-0.43254144771701,48.19262598377,0
-0.43248668153336,48.192537848821,0
-0.43242060111094,48.192453231162,0
-0.4323437094015,48.192372774766,0
-0.43225659162675,48.192297091934,0
-0.4321599108235,48.192226758636,0
-0.43205440279713,48.192162310127,0
-0.43194087052136,48.192104236876,0
-0.4318201780274,48.192052980833,0
-0.43169324382857,48.192008932067,0
-0.43156103393101,48.191972425797,0
-0.43142455448264,48.191943739841,0
-0.43128484411765,48.191923092503,0
-0.43114296605322,48.191910640912,0
-0.43100000000003,48.191906479826,0
-0.43085703394685,48.191910640912,0
-0.43071515588245,48.191923092503,0
-0.43057544551743,48.191943739841,0
-0.43043896606908,48.191972425797,0
-0.43030675617149,48.192008932067,0
-0.4301798219727,48.192052980833,0
-0.4300591294787,48.192104236876,0
-0.42994559720294,48.192162310127,0
-0.42984008917657,48.192226758636,0
-0.42974340837335,48.192297091934,0
-0.42965629059859,48.192372774766,0
-0.42957939888913,48.192453231162,0
-0.42951331846671,48.192537848821,0
-0.42945855228306,48.19262598377,0
-0.42941551719107,48.192716965266,0
-0.42938454077063,48.192810100898,0
-0.42936585883404,48.192904681857,0
-0.42935961362921,48.192999988332,0
-0.43100000000004,48.193,0
</coordinates>
</LinearRing>
</outerBoundaryIs>
</Polygon>
</Placemark>
<Placemark>
<name>525</name>
<styleUrl>#stylehap1</styleUrl>
<Polygon>
<outerBoundaryIs>
<LinearRing>
<coordinates>
-0.42899999999997,48.138,0
-0.42736137171287,48.137999988354,0
-0.42736760415786,48.138095295006,0
-0.4273862601899,48.138189876491,0
-0.42741719789217,48.138283012981,0

-0.42746018187293,48.138373995643,0
-0.42751488505485,48.138462132029,0
-0.42758089116269,48.138546751352,0
-0.42765769788982,48.13862720959,0
-0.42774472072001,48.138702894384,0
-0.42784129737485,48.138773229707,0
-0.42794669285378,48.138837680241,0
-0.42806010502747,48.138895755454,0
-0.42818067074293,48.138947013338,0
-0.42830747239295,48.138991063768,0
-0.42843954490068,48.139027571475,0
-0.42857588306563,48.139056258597,0
-0.42871544921524,48.139076906794,0
-0.42885718110407,48.139089358911,0
-0.42899999999996,48.139093520174,0
-0.42914281889588,48.139089358911,0
-0.42928455078471,48.139076906794,0
-0.42942411693433,48.139056258597,0
-0.42956045509925,48.139027571475,0
-0.429692527607,48.138991063768,0
-0.42981932925702,48.138947013338,0
-0.42993989497248,48.138895755454,0
-0.43005330714617,48.138837680241,0
-0.4301587026251,48.138773229707,0
-0.43025527927994,48.138702894384,0
-0.43034230211013,48.13862720959,0
-0.43041910883726,48.138546751352,0
-0.4304851149451,48.138462132029,0
-0.43053981812703,48.138373995643,0
-0.43058280210776,48.138283012981,0
-0.43061373981005,48.138189876491,0
-0.43063239584207,48.138095295006,0
-0.43063862828708,48.137999988354,0
-0.43063238978141,48.13790468188,0
-0.43061372787284,48.13781010092,0
-0.43058278465674,48.137716965287,0
-0.43053979569238,48.13762598379,0
-0.43048508820854,48.137537848839,0
-0.43041907861118,48.137453231179,0
-0.43034226931288,48.137372774782,0
-0.43025524490809,48.137297091948,0
-0.43015866772302,48.137226758647,0
-0.43005327277432,48.137162310136,0
-0.42993986217523,48.137104236883,0
-0.42981929903094,48.137052980839,0
-0.42969250087045,48.137008932072,0
-0.42956043266463,48.1369724258,0
-0.42942409948328,48.136943739843,0
-0.42928453884751,48.136923092504,0
-0.42914281283519,48.136910640912,0
-0.42899999999996,48.136906479826,0
-0.42885718716476,48.136910640912,0
-0.42871546115245,48.136923092504,0
-0.42857590051667,48.136943739843,0
-0.42843956733532,48.1369724258,0
-0.42830749912951,48.137008932072,0
-0.42818070096901,48.137052980839,0

-0.42806013782472,48.137104236883,0
-0.4279467272256,48.137162310136,0
-0.42784133227694,48.137226758647,0
-0.42774475509183,48.137297091948,0
-0.42765773068707,48.137372774782,0
-0.42758092138878,48.137453231179,0
-0.42751491179138,48.137537848839,0
-0.42746020430754,48.13762598379,0
-0.42741721534321,48.137716965287,0
-0.42738627212711,48.13781010092,0
-0.42736761021854,48.13790468188,0
-0.42736137171287,48.137999988354,0
-0.42899999999997,48.138,0
</coordinates>
</LinearRing>
</outerBoundaryIs>
</Polygon>
</Placemark>
<Placemark>
<name>527</name>
<styleUrl>#stylehap1</styleUrl>
<Polygon>
<outerBoundaryIs>
<LinearRing>
<coordinates>
-0.41800000000001,48.194,0
-0.41635958161511,48.193999988331,0
-0.41636582086269,48.194095294983,0
-0.41638449726945,48.194189876468,0
-0.41641546876378,48.19428301296,0
-0.41645849969691,48.194373995623,0
-0.41651326263436,48.19446213201,0
-0.41657934084626,48.194546751335,0
-0.41665623147737,48.194627209574,0
-0.41674334937294,48.194702894371,0
-0.41684003153115,48.194773229696,0
-0.41694554214848,48.194837680231,0
-0.41705907821935,48.194895755447,0
-0.41717977564776,48.194947013333,0
-0.41730671582402,48.194991063764,0
-0.41743893261684,48.195027571473,0
-0.41757541972753,48.195056258595,0
-0.41771513834977,48.195076906793,0
-0.41785702507753,48.19508935891,0
-0.418,48.195093520174,0
-0.41814297492248,48.19508935891,0
-0.41828486165023,48.195076906793,0
-0.41842458027248,48.195056258595,0
-0.41856106738316,48.195027571473,0
-0.41869328417599,48.194991063764,0
-0.41882022435225,48.194947013333,0
-0.41894092178065,48.194895755447,0
-0.41905445785153,48.194837680231,0
-0.41915996846886,48.194773229696,0
-0.4192566506271,48.194702894371,0
-0.41934376852264,48.194627209574,0
-0.41942065915377,48.194546751335,0

-0.41948673736565,48.19446213201,0
-0.4195415003031,48.194373995623,0
-0.41958453123623,48.19428301296,0
-0.41961550273056,48.194189876468,0
-0.41963417913732,48.194095294983,0
-0.4196404183849,48.193999988331,0
-0.41963417305808,48.193904681857,0
-0.41961549075679,48.193810100898,0
-0.41958451373172,48.193716965266,0
-0.41954147779976,48.19362598377,0
-0.41948671054722,48.193537848821,0
-0.41942062883507,48.193453231162,0
-0.41934373562497,48.193372774766,0
-0.41925661614996,48.193297091934,0
-0.41915993345988,48.193226758636,0
-0.4190544233744,48.193162310127,0
-0.41894088888296,48.193104236876,0
-0.41882019403357,48.193052980833,0
-0.41869325735756,48.193008932067,0
-0.4185610448798,48.192972425797,0
-0.41842456276798,48.192943739841,0
-0.41828484967646,48.192923092503,0
-0.41814296884325,48.192910640912,0
-0.418,48.192906479826,0
-0.41785703115676,48.192910640912,0
-0.41771515032355,48.192923092503,0
-0.41757543723203,48.192943739841,0
-0.41743895512021,48.192972425797,0
-0.41730674264245,48.193008932067,0
-0.41717980596644,48.193052980833,0
-0.41705911111705,48.193104236876,0
-0.41694557662561,48.193162310127,0
-0.41684006654013,48.193226758636,0
-0.41674338385005,48.193297091934,0
-0.41665626437504,48.193372774766,0
-0.41657937116494,48.193453231162,0
-0.41651328945279,48.193537848821,0
-0.41645852220025,48.19362598377,0
-0.41641548626828,48.193716965266,0
-0.41638450924324,48.193810100898,0
-0.41636582694193,48.193904681857,0
-0.41635958161511,48.193999988331,0
-0.41800000000001,48.194,0
</coordinates>
</LinearRing>
</outerBoundaryIs>
</Polygon>
</Placemark>
<Placemark>
<name>528</name>
<styleUrl>#stylehap1</styleUrl>
<Polygon>
<outerBoundaryIs>
<LinearRing>
<coordinates>
-0.41700000000003,48.207,0
-0.41535916527291,48.206999988326,0

-0.41536540610265,48.207095294978,0
-0.41538408724818,48.207189876463,0
-0.4154150666019,48.207283012955,0
-0.41545810845524,48.207373995618,0
-0.41551288529067,48.207462132006,0
-0.41557898027257,48.207546751331,0
-0.41565589041813,48.207627209571,0
-0.41574303042405,48.207702894368,0
-0.41583973712032,48.207773229693,0
-0.41594527451665,48.207837680229,0
-0.41605883940363,48.207895755445,0
-0.41617956746592,48.207947013331,0
-0.41630653986068,48.207991063763,0
-0.41643879021147,48.208027571472,0
-0.41657531196404,48.208056258595,0
-0.41671506604848,48.208076906793,0
-0.41685698878879,48.20808935891,0
-0.41700000000002,48.208093520174,0
-0.41714301121128,48.20808935891,0
-0.41728493395159,48.208076906793,0
-0.41742468803603,48.208056258595,0
-0.4175612097886,48.208027571472,0
-0.41769346013938,48.207991063763,0
-0.41782043253414,48.207947013331,0
-0.41794116059643,48.207895755445,0
-0.41805472548342,48.207837680229,0
-0.41816026287974,48.207773229693,0
-0.41825696957599,48.207702894368,0
-0.41834410958193,48.207627209571,0
-0.41842101972749,48.207546751331,0
-0.4184871147094,48.207462132006,0
-0.41854189154483,48.207373995618,0
-0.41858493339817,48.207283012955,0
-0.41861591275189,48.207189876463,0
-0.41863459389741,48.207095294978,0
-0.41864083472713,48.206999988326,0
-0.41863458781385,48.206904681852,0
-0.41861590076959,48.206810100893,0
-0.41858491588123,48.206716965261,0
-0.41854186902548,48.206625983765,0
-0.41848708787188,48.206537848816,0
-0.41842098938727,48.206453231158,0
-0.41834407666087,48.206372774763,0
-0.41825693507437,48.206297091931,0
-0.41816022784585,48.206226758633,0
-0.4180546909818,48.206162310124,0
-0.41794112767538,48.206104236874,0
-0.41782040219392,48.206052980832,0
-0.41769343330186,48.206008932066,0
-0.41756118726925,48.205972425797,0
-0.41742467051908,48.205943739841,0
-0.41728492196929,48.205923092503,0
-0.41714300512772,48.205910640912,0
-0.41700000000002,48.205906479826,0
-0.41685699487235,48.205910640912,0
-0.41671507803075,48.205923092503,0
-0.41657532948098,48.205943739841,0

-0.41643881273079,48.205972425797,0
-0.4163065666982,48.206008932066,0
-0.41617959780615,48.206052980832,0
-0.41605887232469,48.206104236874,0
-0.41594530901826,48.206162310124,0
-0.41583977215421,48.206226758633,0
-0.41574306492569,48.206297091931,0
-0.41565592333919,48.206372774763,0
-0.4155790106128,48.206453231158,0
-0.41551291212816,48.206537848816,0
-0.41545813097458,48.206625983765,0
-0.41541508411884,48.206716965261,0
-0.41538409923047,48.206810100893,0
-0.41536541218622,48.206904681852,0
-0.41535916527291,48.206999988326,0
-0.41700000000003,48.207,0
</coordinates>
</LinearRing>
</outerBoundaryIs>
</Polygon>
</Placemark>
<Placemark>
<name>529</name>
<styleUrl>#stylehap1</styleUrl>
<Polygon>
<outerBoundaryIs>
<LinearRing>
<coordinates>
-0.41100000000006,48.456,0
-0.4093511332901,48.455999988223,0
-0.40935740464218,48.456095294876,0
-0.409376177207,48.456189876364,0
-0.40940730818203,48.456283012859,0
-0.40945056070567,48.456373995527,0
-0.40950560565789,48.456462131922,0
-0.40957202416317,48.456546751254,0
-0.40964931077702,48.456627209502,0
-0.40973687733145,48.456702894308,0
-0.40983405741034,48.456773229642,0
-0.4099401114208,48.456837680186,0
-0.41005423222157,48.456895755411,0
-0.41017555126626,48.456947013306,0
-0.41030314521387,48.456991063745,0
-0.410436042957,48.45702757146,0
-0.4105732330137,48.457056258588,0
-0.41071367122705,48.45707690679,0
-0.4108562887136,48.45708935891,0
-0.41100000000006,48.457093520174,0
-0.41114371128649,48.45708935891,0
-0.41128632877304,48.45707690679,0
-0.41142676698643,48.457056258588,0
-0.41156395704312,48.45702757146,0
-0.41169685478625,48.456991063745,0
-0.41182444873386,48.456947013306,0
-0.41194576777853,48.456895755411,0
-0.41205988857932,48.456837680186,0
-0.41216594258978,48.456773229642,0

-0.41226312266867,48.456702894308,0
-0.4123506892231,48.456627209502,0
-0.41242797583695,48.456546751254,0
-0.41249439434223,48.456462131922,0
-0.41254943929442,48.456373995527,0
-0.41259269181809,48.456283012859,0
-0.41262382279313,48.456189876364,0
-0.41264259535792,48.456095294876,0
-0.41264886671002,48.455999988223,0
-0.41264258919085,48.45590468175,0
-0.41262381064636,48.455810100793,0
-0.4125926740607,48.455716965165,0
-0.41254941646595,48.455625983675,0
-0.41249436713633,48.455537848732,0
-0.41242794508025,48.455453231081,0
-0.41235065585012,48.455372774694,0
-0.41226308769343,48.455297091871,0
-0.41216590707497,48.455226758582,0
-0.41205985360408,48.455162310082,0
-0.41194573440555,48.45510423684,0
-0.41182441797713,48.455052980806,0
-0.41169682758034,48.455008932048,0
-0.41156393421465,48.454972425785,0
-0.41142674922903,48.454943739834,0
-0.41128631662628,48.4549230925,0
-0.41114370511942,48.454910640911,0
-0.41100000000006,48.454906479826,0
-0.4108562948807,48.454910640911,0
-0.41071368337385,48.4549230925,0
-0.41057325077109,48.454943739834,0
-0.41043606578547,48.454972425785,0
-0.41030317241978,48.455008932048,0
-0.41017558202299,48.455052980806,0
-0.41005426559457,48.45510423684,0
-0.40994014639604,48.455162310082,0
-0.40983409292515,48.455226758582,0
-0.40973691230669,48.455297091871,0
-0.40964934415,48.455372774694,0
-0.40957205491987,48.455453231081,0
-0.4095056328638,48.455537848732,0
-0.40945058353414,48.455625983675,0
-0.40940732593942,48.455716965165,0
-0.40937618935376,48.455810100793,0
-0.40935741080927,48.45590468175,0
-0.4093511332901,48.455999988223,0
-0.41100000000006,48.456,0
</coordinates>
</LinearRing>
</outerBoundaryIs>
</Polygon>
</Placemark>
<Placemark>
<name>530</name>
<styleUrl>#stylehap1</styleUrl>
<Polygon>
<outerBoundaryIs>
<LinearRing>

<coordinates>
-0.40899999999999,48.374,0
-0.40735379046602,48.373999988257,0
-0.40736005172057,48.374095294909,0
-0.40737879404167,48.374189876397,0
-0.40740987485669,48.374283012891,0
-0.40745305768547,48.374373995557,0
-0.4075080139381,48.374462131949,0
-0.40757432541397,48.37454675128,0
-0.40765148748293,48.374627209525,0
-0.4077389129247,48.374702894327,0
-0.40783593639709,48.374773229659,0
-0.40794181949897,48.3748376802,0
-0.40805575638989,48.374895755422,0
-0.40817687992316,48.374947013314,0
-0.40830426824591,48.374991063751,0
-0.40843695181582,48.375027571464,0
-0.4085739207812,48.375056258591,0
-0.40871413266801,48.375076906791,0
-0.40885652031552,48.37508935891,0
-0.40899999999999,48.375093520174,0
-0.40914347968446,48.37508935891,0
-0.40928586733197,48.375076906791,0
-0.40942607921878,48.375056258591,0
-0.40956304818416,48.375027571464,0
-0.40969573175407,48.374991063751,0
-0.40982312007682,48.374947013314,0
-0.40994424361009,48.374895755422,0
-0.41005818050103,48.3748376802,0
-0.41016406360291,48.374773229659,0
-0.41026108707528,48.374702894327,0
-0.41034851251705,48.374627209525,0
-0.41042567458601,48.37454675128,0
-0.41049198606188,48.374462131949,0
-0.41054694231451,48.374373995557,0
-0.41059012514329,48.374283012891,0
-0.41062120595833,48.374189876397,0
-0.41063994827941,48.374095294909,0
-0.41064620953399,48.373999988257,0
-0.41063994214,48.373904681784,0
-0.41062119386604,48.373810100826,0
-0.41059010746557,48.373716965197,0
-0.41054691958845,48.373625983705,0
-0.41049195897798,48.3735378487 6,0
-0.41042564396726,48.373453231106,0
-0.41034847929377,48.373372774716,0
-0.41026105225691,48.373297091891,0
-0.41016402824741,48.373226758599,0
-0.41005814568266,48.373162310096,0
-0.4099442103868,48.373104236851,0
-0.40982308945805,48.373052980815,0
-0.40969570467017,48.373008932054,0
-0.40956302545808,48.372972425789,0
-0.40942606154103,48.372943739836,0
-0.40928585523968,48.372923092501,0
-0.40914347354505,48.372910640912,0
-0.40899999999999,48.372906479826,0

-0.40885652645493,48.372910640912,0
-0.4087141447603,48.372923092501,0
-0.40857393845895,48.372943739836,0
-0.4084369745419,48.372972425789,0
-0.40830429532981,48.373008932054,0
-0.40817691054193,48.373052980815,0
-0.40805578961318,48.373104236851,0
-0.40794185431732,48.373162310096,0
-0.40783597175257,48.373226758599,0
-0.40773894774307,48.373297091891,0
-0.40765152070621,48.373372774716,0
-0.40757435603272,48.373453231106,0
-0.407508041022,48.37353784876,0
-0.40745308041155,48.373625983705,0
-0.40740989253444,48.373716965197,0
-0.40737880613396,48.373810100826,0
-0.40736005785998,48.373904681784,0
-0.40735379046602,48.373999988257,0
-0.40899999999999,48.374,0
</coordinates>
</LinearRing>
</outerBoundaryIs>
</Polygon>
</Placemark>
<Placemark>
<name>531</name>
<styleUrl>#stylehap1</styleUrl>
<Polygon>
<outerBoundaryIs>
<LinearRing>
<coordinates>
-0.40700000000004,48.046,0
-0.40536430073467,48.045999988392,0
-0.40537052204903,48.046095295043,0
-0.40538914474316,48.046189876527,0
-0.40542002715367,48.046283013016,0
-0.40546293430928,48.046373995676,0
-0.40551753971706,48.04646213206,0
-0.40558342784566,48.04654675138,0
-0.40566009728616,48.046627209615,0
-0.40574696456694,48.046702894406,0
-0.40584336859344,48.046773229726,0
-0.40594857567878,48.046837680256,0
-0.4060617851275,48.046895755467,0
-0.40618213532954,48.046947013348,0
-0.40630871031804,48.046991063775,0
-0.40644054674136,48.04702757148,0
-0.40657664119598,48.0470562586,0
-0.40671595786449,48.047076906795,0
-0.40685743640039,48.047089358911,0
-0.40700000000005,48.047093520174,0
-0.40714256359971,48.047089358911,0
-0.40728404213561,48.047076906795,0
-0.40742335880409,48.0470562586,0
-0.40755945325873,48.04702757148,0
-0.40769128968205,48.046991063775,0
-0.40781786467055,48.046947013348,0

-0.40793821487256,48.046895755467,0
-0.40805142432131,48.046837680256,0
-0.40815663140665,48.046773229726,0
-0.40825303543312,48.046702894406,0
-0.40833990271393,48.046627209615,0
-0.40841657215443,48.04654675138,0
-0.40848246028301,48.04646213206,0
-0.40853706569081,48.046373995676,0
-0.40857997284639,48.046283013016,0
-0.40861085525693,48.046189876527,0
-0.40862947795103,48.046095295043,0
-0.4086356992654,48.045999988392,0
-0.4086294719207,48.045904681917,0
-0.40861084337947,48.045810100957,0
-0.40857995548273,48.045716965322,0
-0.40853704336851,48.045625983823,0
-0.40848243368034,48.04553784887,0
-0.40841654207967,48.045453231207,0
-0.40833987008091,48.045372774807,0
-0.40825300123338,48.04529709197,0
-0.40815659667932,48.045226758666,0
-0.40805139012155,48.045162310152,0
-0.40793818223956,48.045104236895,0
-0.40781783459579,48.045052980848,0
-0.40769126307936,48.045008932078,0
-0.40755943093642,48.044972425804,0
-0.40742334144042,48.044943739845,0
-0.40728403025817,48.044923092505,0
-0.40714255756935,48.044910640913,0
-0.40700000000005,48.044906479827,0
-0.40685744243072,48.044910640913,0
-0.40671596974192,48.044923092505,0
-0.40657665855965,48.044943739845,0
-0.40644056906367,48.044972425804,0
-0.40630873692071,48.045008932078,0
-0.40618216540428,48.045052980848,0
-0.40606181776053,48.045104236895,0
-0.40594860987852,48.045162310152,0
-0.40584340332077,48.045226758666,0
-0.40574699876669,48.04529709197,0
-0.40566012991916,48.045372774807,0
-0.4055834579204,48.045453231207,0
-0.40551756631973,48.04553784887,0
-0.40546295663158,48.045625983823,0
-0.40542004451734,48.045716965322,0
-0.4053891566206,48.045810100957,0
-0.40537052807936,48.045904681917,0
-0.40536430073467,48.045999988392,0
-0.40700000000004,48.046,0
</coordinates>
</LinearRing>
</outerBoundaryIs>
</Polygon>
</Placemark>
<Placemark>
<name>533</name>
<styleUrl>#stylehap1</styleUrl>

<Polygon>
<outerBoundaryIs>
<LinearRing>
<coordinates>
-0.40300000000002,47.974,0
-0.40136658278551,47.973999988421,0
-0.40137279542778,47.974095295072,0
-0.40139139214777,47.974189876556,0
-0.4014222314795,47.974283013044,0
-0.4014650787793,47.974373995702,0
-0.40151960800959,47.974462132084,0
-0.40158540421853,47.974546751402,0
-0.40166196669665,47.974627209635,0
-0.40174871278626,47.974702894424,0
-0.40184498231504,47.97477322974,0
-0.40195004261974,47.974837680268,0
-0.40206309412197,47.974895755476,0
-0.40218327641382,47.974947013355,0
-0.40230967480645,47.97499106378,0
-0.40244132729239,47.975027571483,0
-0.40257723186817,47.975056258601,0
-0.40271635416169,47.975076906796,0
-0.402857635306,47.975089358911,0
-0.40300000000003,47.975093520174,0
-0.40314236469404,47.975089358911,0
-0.40328364583835,47.975076906796,0
-0.40342276813186,47.975056258601,0
-0.40355867270764,47.975027571483,0
-0.40369032519359,47.97499106378,0
-0.40381672358622,47.974947013355,0
-0.40393690587807,47.974895755476,0
-0.40404995738032,47.974837680268,0
-0.404155017685,47.97477322974,0
-0.40425128721377,47.974702894424,0
-0.40433803330339,47.974627209635,0
-0.4044145957815,47.974546751402,0
-0.40448039199044,47.974462132084,0
-0.40453492122073,47.974373995702,0
-0.40457776852054,47.974283013044,0
-0.40460860785227,47.974189876556,0
-0.40462720457226,47.974095295072,0
-0.40463341721453,47.973999988421,0
-0.40462719856554,47.973904681946,0
-0.40460859602134,47.973810100985,0
-0.40457775122486,47.97371696535,0
-0.40453489898583,47.973625983849,0
-0.40448036549195,47.973537848894,0
-0.40441456582452,47.973453231229,0
-0.40433800079814,47.973372774826,0
-0.40425125314795,47.973297091987,0
-0.40415498309364,47.973226758681,0
-0.40404992331447,47.973162310164,0
-0.40393687337283,47.973104236905,0
-0.40381669362921,47.973052980856,0
-0.40369029869506,47.973008932083,0
-0.40355865047274,47.972972425808,0
-0.40342275083618,47.972943739847,0

-0.40328363400742,47.972923092506,0
-0.40314235868732,47.972910640913,0
-0.40300000000003,47.972906479826,0
-0.40285764131275,47.972910640913,0
-0.40271636599261,47.972923092506,0
-0.40257724916385,47.972943739847,0
-0.40244134952729,47.972972425808,0
-0.40230970130498,47.973008932083,0
-0.40218330637083,47.973052980856,0
-0.40206312662721,47.973104236905,0
-0.40195007668557,47.973162310164,0
-0.4018450169064,47.973226758681,0
-0.40174874685209,47.973297091987,0
-0.40166199920189,47.973372774826,0
-0.40158543417552,47.973453231229,0
-0.40151963450809,47.973537848894,0
-0.40146510101421,47.973625983849,0
-0.40142224877518,47.97371696535,0
-0.40139140397872,47.973810100985,0
-0.40137280143452,47.973904681946,0
-0.40136658278551,47.973999988421,0
-0.40300000000002,47.974,0
</coordinates>
</LinearRing>
</outerBoundaryIs>
</Polygon>
</Placemark>
<Placemark>
<name>534</name>
<styleUrl>#stylehap1</styleUrl>
<Polygon>
<outerBoundaryIs>
<LinearRing>
<coordinates>
-0.40300000000002,47.974,0
-0.40136658278551,47.973999988421,0
-0.40137279542778,47.974095295072,0
-0.40139139214777,47.974189876556,0
-0.4014222314795,47.974283013044,0
-0.4014650787793,47.974373995702,0
-0.40151960800959,47.974462132084,0
-0.40158540421853,47.974546751402,0
-0.40166196669665,47.974627209635,0
-0.40174871278626,47.974702894424,0
-0.40184498231504,47.97477322974,0
-0.40195004261974,47.974837680268,0
-0.40206309412197,47.974895755476,0
-0.40218327641382,47.974947013355,0
-0.40230967480645,47.97499106378,0
-0.40244132729239,47.975027571483,0
-0.40257723186817,47.975056258601,0
-0.40271635416169,47.975076906796,0
-0.402857635306,47.975089358911,0
-0.40300000000003,47.975093520174,0
-0.40314236469404,47.975089358911,0
-0.40328364583835,47.975076906796,0
-0.40342276813186,47.975056258601,0

-0.40355867270764,47.975027571483,0
-0.40369032519359,47.97499106378,0
-0.40381672358622,47.974947013355,0
-0.40393690587807,47.974895755476,0
-0.40404995738032,47.974837680268,0
-0.404155017685,47.97477322974,0
-0.40425128721377,47.974702894424,0
-0.40433803330339,47.974627209635,0
-0.4044145957815,47.974546751402,0
-0.40448039199044,47.974462132084,0
-0.40453492122073,47.974373995702,0
-0.40457776852054,47.974283013044,0
-0.40460860785227,47.974189876556,0
-0.40462720457226,47.974095295072,0
-0.40463341721453,47.973999988421,0
-0.40462719856554,47.973904681946,0
-0.40460859602134,47.973810100985,0
-0.40457775122486,47.97371696535,0
-0.40453489898583,47.973625983849,0
-0.40448036549195,47.973537848894,0
-0.40441456582452,47.973453231229,0
-0.40433800079814,47.973372774826,0
-0.40425125314795,47.973297091987,0
-0.40415498309364,47.973226758681,0
-0.40404992331447,47.973162310164,0
-0.40393687337283,47.973104236905,0
-0.40381669362921,47.973052980856,0
-0.40369029869506,47.973008932083,0
-0.40355865047274,47.972972425808,0
-0.40342275083618,47.972943739847,0
-0.40328363400742,47.972923092506,0
-0.40314235868732,47.972910640913,0
-0.40300000000003,47.972906479826,0
-0.40285764131275,47.972910640913,0
-0.40271636599261,47.972923092506,0
-0.40257724916385,47.972943739847,0
-0.40244134952729,47.972972425808,0
-0.40230970130498,47.973008932083,0
-0.40218330637083,47.973052980856,0
-0.40206312662721,47.973104236905,0
-0.40195007668557,47.973162310164,0
-0.4018450169064,47.973226758681,0
-0.40174874685209,47.973297091987,0
-0.40166199920189,47.973372774826,0
-0.40158543417552,47.973453231229,0
-0.40151963450809,47.973537848894,0
-0.40146510101421,47.973625983849,0
-0.40142224877518,47.97371696535,0
-0.40139140397872,47.973810100985,0
-0.40137280143452,47.973904681946,0
-0.40136658278551,47.973999988421,0
-0.40300000000002,47.974,0
</coordinates>
</LinearRing>
</outerBoundaryIs>
</Polygon>
</Placemark>

<Placemark>
<name>536</name>
<styleUrl>#stylehap1</styleUrl>
<Polygon>
<outerBoundaryIs>
<LinearRing>
<coordinates>
-0.39999999999998,48.105,0
-0.39836242403166,48.104999988368,0
-0.39836865247772,48.105095295019,0
-0.39838729653233,48.105189876504,0
-0.3984182143698,48.105283012994,0
-0.39846117074932,48.105373995655,0
-0.39851583880362,48.10546213204,0
-0.39858180252469,48.105546751362,0
-0.39865855992845,48.105627209599,0
-0.3987455268739,48.105702894392,0
-0.39884204150802,48.105773229714,0
-0.3989473693022,48.105837680246,0
-0.39906070864226,48.105895755459,0
-0.39918119692953,48.105947013342,0
-0.39930791714616,48.105991063771,0
-0.39943990483511,48.106027571477,0
-0.39957615544147,48.106056258598,0
-0.39971563195924,48.106076906794,0
-0.39985727282515,48.106089358911,0
-0.39999999999998,48.106093520174,0
-0.4001427271748,48.106089358911,0
-0.40028436804074,48.106076906794,0
-0.40042384455848,48.106056258598,0
-0.40056009516484,48.106027571477,0
-0.40069208285379,48.105991063771,0
-0.40081880307042,48.105947013342,0
-0.40093929135769,48.105895755459,0
-0.40105263069777,48.105837680246,0
-0.40115795849193,48.105773229714,0
-0.40125447312606,48.105702894392,0
-0.4013414400715,48.105627209599,0
-0.40141819747526,48.105546751362,0
-0.40148416119633,48.10546213204,0
-0.40153882925063,48.105373995655,0
-0.40158178563018,48.105283012994,0
-0.40161270346762,48.105189876504,0
-0.40163134752223,48.105095295019,0
-0.40163757596829,48.104999988368,0
-0.40163134147245,48.104904681893,0
-0.40161269155189,48.104810100933,0
-0.40158176821053,48.1047169653,0
-0.40153880685637,48.104625983802,0
-0.40148413450789,48.104537848851,0
-0.40141816730357,48.104453231189,0
-0.40134140733332,48.104372774791,0
-0.40125443881606,48.104297091956,0
-0.40115792365267,48.104226758654,0
-0.40105259638778,48.104162310142,0
-0.40093925861947,48.104104236888,0
-0.40081877289873,48.104052980842,0

-0.40069205616538,48.104008932074,0
-0.40056007277061,48.103972425802,0
-0.40042382713884,48.103943739844,0
-0.40028435612501,48.103923092504,0
-0.40014272112503,48.103910640913,0
-0.39999999999998,48.103906479826,0
-0.39985727887495,48.103910640913,0
-0.39971564387497,48.103923092504,0
-0.39957617286111,48.103943739844,0
-0.39943992722937,48.103972425802,0
-0.3993079438346,48.104008932074,0
-0.39918122710122,48.104052980842,0
-0.39906074138048,48.104104236888,0
-0.39894740361217,48.104162310142,0
-0.39884207634731,48.104226758654,0
-0.39874556118389,48.104297091956,0
-0.39865859266663,48.104372774791,0
-0.39858183269638,48.104453231189,0
-0.39851586549206,48.104537848851,0
-0.39846119314358,48.104625983802,0
-0.39841823178942,48.1047169653,0
-0.39838730844806,48.104810100933,0
-0.39836865852752,48.104904681893,0
-0.39836242403166,48.104999988368,0
-0.39999999999998,48.105,0
</coordinates>
</LinearRing>
</outerBoundaryIs>
</Polygon>
</Placemark>
<Placemark>
<name>537</name>
<styleUrl>#stylehap1</styleUrl>
<Polygon>
<outerBoundaryIs>
<LinearRing>
<coordinates>
-0.399,48.29,0
-0.39735650010101,48.289999988292,0
-0.39736275105868,48.290095294944,0
-0.39738146253896,48.29018987643,0
-0.39741249220368,48.290283012923,0
-0.39745560396162,48.290373995588,0
-0.39751046976355,48.290462131978,0
-0.39757667209699,48.290546751306,0
-0.39765370716222,48.290627209548,0
-0.39774098870544,48.290702894348,0
-0.39783785247949,48.290773229676,0
-0.39794356129865,48.290837680215,0
-0.39805731064893,48.290895755434,0
-0.39817823481093,48.290947013323,0
-0.3983054134493,48.290991063757,0
-0.39843787861773,48.291027571468,0
-0.39857462212691,48.291056258593,0
-0.39871460321894,48.291076906792,0
-0.39885675648987,48.29108935891,0
-0.39899999999999,48.291093520174,0

-0.39914324351013,48.29108935891,0
-0.39928539678106,48.291076906792,0
-0.39942537787309,48.291056258593,0
-0.39956212138226,48.291027571468,0
-0.39969458655071,48.290991063757,0
-0.39982176518907,48.290947013323,0
-0.39994268935108,48.290895755434,0
-0.40005643870133,48.290837680215,0
-0.40016214752052,48.290773229676,0
-0.40025901129456,48.290702894348,0
-0.40034629283779,48.290627209548,0
-0.40042332790302,48.290546751306,0
-0.40048953023643,48.290462131978,0
-0.40054439603836,48.290373995588,0
-0.40058750779632,48.290283012923,0
-0.40061853746105,48.29018987643,0
-0.40063724894133,48.290095294944,0
-0.400643499899,48.289999988292,0
-0.40063724283008,48.289904681818,0
-0.40061852542423,48.28981010086,0
-0.40058749019971,48.289716965229,0
-0.40054437341657,48.289625983735,0
-0.40048950327685,48.289537848788,0
-0.40042329742478,48.289453231132,0
-0.40034625976696,48.28937277474,0
-0.40025897663598,48.289297091911,0
-0.40016211232725,48.289226758616,0
-0.40005640404275,48.28916231011,0
-0.39994265628026,48.289104236863,0
-0.39982173471083,48.289052980823,0
-0.3996945595911,48.28900893206,0
-0.39956209876047,48.288972425793,0
-0.39942536027646,48.288943739839,0
-0.39928538474427,48.288923092502,0
-0.39914323739889,48.288910640912,0
-0.39899999999999,48.288906479827,0
-0.39885676260112,48.288910640912,0
-0.39871461525574,48.288923092502,0
-0.39857463972355,48.288943739839,0
-0.39843790123951,48.288972425793,0
-0.3983054404089,48.28900893206,0
-0.39817826528917,48.289052980823,0
-0.39805734371975,48.289104236863,0
-0.39794359595726,48.28916231011,0
-0.39783788767276,48.289226758616,0
-0.39774102336402,48.289297091911,0
-0.39765374023304,48.28937277474,0
-0.39757670257523,48.289453231132,0
-0.39751049672315,48.289537848788,0
-0.39745562658341,48.289625983735,0
-0.39741250980029,48.289716965229,0
-0.39738147457576,48.28981010086,0
-0.39736275716992,48.289904681818,0
-0.39735650010101,48.289999988292,0
-0.399,48.29,0
</coordinates>
</LinearRing>

</outerBoundaryIs>
</Polygon>
</Placemark>
<Placemark>
<name>538</name>
<styleUrl>#stylehap1</styleUrl>
<Polygon>
<outerBoundaryIs>
<LinearRing>
<coordinates>
-0.399,48.289,0
-0.39735653228359,48.288999988292,0
-0.39736278311895,48.289095294944,0
-0.39738149423293,48.289189876431,0
-0.39741252329012,48.289283012924,0
-0.39745563420397,48.289373995588,0
-0.3975104989316,48.289462131978,0
-0.39757669996872,48.289546751306,0
-0.39765373352553,48.289627209548,0
-0.39774101335968,48.289702894348,0
-0.39783787523697,48.289773229676,0
-0.39794358198617,48.289837680215,0
-0.39805732910899,48.289895755434,0
-0.39817825090304,48.289947013323,0
-0.39830542705098,48.289991063757,0
-0.39843788962545,48.290027571468,0
-0.39857463045686,48.290056258593,0
-0.39871460880771,48.290076906792,0
-0.39885675929494,48.29008935891,0
-0.39899999999999,48.290093520174,0
-0.39914324070506,48.29008935891,0
-0.3992853911923,48.290076906792,0
-0.39942536954314,48.290056258593,0
-0.39956211037456,48.290027571468,0
-0.39969457294903,48.289991063757,0
-0.39982174909694,48.289947013323,0
-0.39994267089101,48.289895755434,0
-0.40005641801383,48.289837680215,0
-0.40016212476304,48.289773229676,0
-0.40025898664033,48.289702894348,0
-0.40034626647447,48.289627209548,0
-0.40042330003126,48.289546751306,0
-0.4004895010684,48.289462131978,0
-0.40054436579602,48.289373995588,0
-0.40058747670986,48.289283012924,0
-0.40061850576707,48.289189876431,0
-0.40063721688106,48.289095294944,0
-0.40064346771642,48.288999988292,0
-0.40063721077014,48.288904681818,0
-0.40061849373091,48.28881010086,0
-0.40058745911422,48.28871696523,0
-0.40054434317548,48.288625983735,0
-0.40048947411027,48.288537848789,0
-0.4004232695547,48.288453231133,0
-0.40034623340543,48.28837277474,0
-0.40025895198366,48.288297091911,0
-0.4001620895717,48.288226758616,0

-0.40005638335713,48.28816231011,0
-0.399942637822,48.288104236863,0
-0.39982171862038,48.288052980823,0
-0.3996945459909,48.28800893206,0
-0.39956208775402,48.287972425793,0
-0.39942535194748,48.287943739839,0
-0.39928537915614,48.287923092502,0
-0.39914323459415,48.287910640912,0
-0.39899999999999,48.287906479826,0
-0.39885676540583,48.287910640912,0
-0.39871462084385,48.287923092502,0
-0.39857464805253,48.287943739839,0
-0.39843791224598,48.287972425793,0
-0.39830545400911,48.28800893206,0
-0.39817828137962,48.288052980823,0
-0.39805736217801,48.288104236863,0
-0.39794361664285,48.28816231011,0
-0.3978379104283,48.288226758616,0
-0.39774104801635,48.288297091911,0
-0.39765376659457,48.28837277474,0
-0.39757673044531,48.288453231133,0
-0.39751052588973,48.288537848789,0
-0.39745565682453,48.288625983735,0
-0.39741254088579,48.28871696523,0
-0.39738150626909,48.28881010086,0
-0.39736278922986,48.288904681818,0
-0.39735653228359,48.288999988292,0
-0.399,48.289,0
</coordinates>
</LinearRing>
</outerBoundaryIs>
</Polygon>
</Placemark>
<Placemark>
<name>539</name>
<styleUrl>#stylehap1</styleUrl>
<Polygon>
<outerBoundaryIs>
<LinearRing>
<coordinates>
-0.399,48.106,0
-0.3973623921711,48.105999988367,0
-0.39736862073822,48.106095295019,0
-0.39738726515546,48.106189876503,0
-0.39741818359436,48.106283012994,0
-0.39746114080955,48.106373995654,0
-0.39751580992741,48.10646213204,0
-0.3975817749318,48.106546751362,0
-0.39765853382888,48.106627209598,0
-0.39774550246636,48.106702894392,0
-0.39784201897823,48.106773229714,0
-0.39794734882169,48.106837680246,0
-0.39806069036689,48.106895755458,0
-0.39818118099843,48.106947013342,0
-0.39830790368057,48.106991063771,0
-0.39843989393755,48.107027571477,0
-0.39857614719489,48.107056258598,0

-0.39871562642638,48.107076906794,0
-0.39885727004819,48.107089358911,0
-0.39899999999999,48.107093520173,0
-0.39914272995182,48.107089358911,0
-0.39928437357362,48.107076906794,0
-0.39942385280511,48.107056258598,0
-0.39956010606246,48.107027571477,0
-0.39969209631941,48.106991063771,0
-0.39981881900158,48.106947013342,0
-0.39993930963309,48.106895755458,0
-0.40005265117832,48.106837680246,0
-0.40015798102175,48.106773229714,0
-0.40025449753365,48.106702894392,0
-0.40034146617113,48.106627209598,0
-0.40041822506821,48.106546751362,0
-0.4004841900726,48.10646213204,0
-0.40053885919043,48.106373995654,0
-0.40058181640565,48.106283012994,0
-0.40061273484454,48.106189876503,0
-0.40063137926178,48.106095295019,0
-0.40063760782891,48.105999988367,0
-0.40063137321168,48.105904681893,0
-0.40061272292815,48.105810100933,0
-0.40058179898506,48.1057169653,0
-0.40053883679497,48.105625983802,0
-0.40048416338271,48.10553784885,0
-0.40041819489487,48.105453231189,0
-0.40034143343113,48.1053727479,0
-0.4002544632218,48.105297091955,0
-0.40015794618058,48.105226758654,0
-0.40005261686647,48.105162310141,0
-0.3999392768931,48.105104236887,0
-0.39981878882824,48.105052980842,0
-0.39969206962955,48.105008932074,0
-0.39956008366698,48.104972425802,0
-0.39942383538453,48.104943739844,0
-0.39928436165723,48.104923092504,0
-0.39914272390172,48.104910640913,0
-0.39899999999999,48.104906479826,0
-0.39885727609829,48.104910640913,0
-0.39871563834278,48.104923092504,0
-0.39857616461548,48.104943739844,0
-0.39843991633303,48.104972425802,0
-0.39830793037046,48.105008932074,0
-0.39818121117177,48.105052980842,0
-0.39806072310688,48.105104236887,0
-0.39794738313354,48.105162310141,0
-0.39784205381943,48.105226758654,0
-0.39774553677821,48.105297091955,0
-0.39765856656887,48.1053727479,0
-0.39758180510513,48.105453231189,0
-0.3975158366173,48.10553784885,0
-0.39746116320504,48.105625983802,0
-0.39741820101494,48.1057169653,0
-0.39738727707186,48.105810100933,0
-0.39736862678832,48.105904681893,0
-0.3973623921711,48.105999988367,0

-0.399,48.106,0
</coordinates>
</LinearRing>
</outerBoundaryIs>
</Polygon>
</Placemark>
<Placemark>
<name>540</name>
<styleUrl>#stylehap1</styleUrl>
<Polygon>
<outerBoundaryIs>
<LinearRing>
<coordinates>
-0.39800000000002,48.42,0
-0.39635230132629,48.419999988238,0
-0.39635856823973,48.420095294891,0
-0.39637732751006,48.420189876378,0
-0.39640843643586,48.420283012873,0
-0.39645165832318,48.420373995541,0
-0.396506664285,48.420462131934,0
-0.3965730357425,48.420546751265,0
-0.39665026760916,48.420627209512,0
-0.39673777213356,48.420702894316,0
-0.39683488337169,48.420773229649,0
-0.39694086225447,48.420837680193,0
-0.39705490221251,48.420895755416,0
-0.39717613531467,48.420947013309,0
-0.39730363887412,48.420991063748,0
-0.39743644247138,48.421027571462,0
-0.39757353534119,48.421056258589,0
-0.39771387406636,48.42107690679,0
-0.39785639052074,48.42108935891,0
-0.39800000000003,48.421093520174,0
-0.39814360947932,48.42108935891,0
-0.39828612593367,48.42107690679,0
-0.39842646465885,48.421056258589,0
-0.39856355752866,48.421027571462,0
-0.39869636112594,48.420991063748,0
-0.39882386468537,48.420947013309,0
-0.39894509778753,48.420895755416,0
-0.39905913774559,48.420837680193,0
-0.39916511662838,48.420773229649,0
-0.39926222786647,48.420702894316,0
-0.3993497323909,48.420627209512,0
-0.39942696425754,48.420546751265,0
-0.39949333571503,48.420462131934,0
-0.39954834167686,48.420373995541,0
-0.39959156356418,48.420283012873,0
-0.39962267248998,48.420189876378,0
-0.3996414317603,48.420095294891,0
-0.39964769867375,48.419999988238,0
-0.3996414256054,48.419904681765,0
-0.39962266036718,48.419810100808,0
-0.39959154584182,48.419716965179,0
-0.39954831889342,48.419625983688,0
-0.39949330856279,48.419537848744,0
-0.39942693356149,48.419453231092,0

-0.39934969908373,48.419372774704,0
-0.39926219296022,48.41929709188,0
-0.39916508118361,48.419226758589,0
-0.39905910283931,48.419162310088,0
-0.39894506448038,48.419104236845,0
-0.39882383398933,48.41905298081,0
-0.3986963339737,48.419008932051,0
-0.39856353474523,48.418972425786,0
-0.39842644693649,48.418943739835,0
-0.39828611381085,48.4189230925,0
-0.39814360332442,48.418910640912,0
-0.39800000000003,48.418906479827,0
-0.39785639667565,48.418910640912,0
-0.39771388618919,48.4189230925,0
-0.39757355306357,48.418943739835,0
-0.39743646525484,48.418972425786,0
-0.39730366602637,48.419008932051,0
-0.39717616601074,48.41905298081,0
-0.39705493551968,48.419104236845,0
-0.39694089716073,48.419162310088,0
-0.39683491881642,48.419226758589,0
-0.39673780703982,48.41929709188,0
-0.39665030091634,48.419372774704,0
-0.39657306643857,48.419453231092,0
-0.39650669143727,48.419537848744,0
-0.39645168110664,48.419625983688,0
-0.39640845415824,48.419716965179,0
-0.39637733963289,48.419810100808,0
-0.39635857439467,48.419904681765,0
-0.39635230132629,48.419999988238,0
-0.39800000000002,48.42,0
</coordinates>
</LinearRing>
</outerBoundaryIs>
</Polygon>
</Placemark>
<Placemark>
<name>541</name>
<styleUrl>#stylehap1</styleUrl>
<Polygon>
<outerBoundaryIs>
<LinearRing>
<coordinates>
-0.39699999999993,48.296,0
-0.39535630696855,48.295999988289,0
-0.39536255866014,48.296095294941,0
-0.39538127233864,48.296189876428,0
-0.39541230564914,48.296283012921,0
-0.39545542247277,48.296373995586,0
-0.39551029472168,48.296462131976,0
-0.39557650483434,48.296546751304,0
-0.39565354895192,48.296627209546,0
-0.39574084075169,48.296702894346,0
-0.39583771590844,48.296773229675,0
-0.3959434371498,48.296837680214,0
-0.39605719986723,48.296895755433,0
-0.39617813823966,48.296947013322,0

-0.39630533182353,48.296991063757,0
-0.39643781255878,48.297027571468,0
-0.39657457213761,48.297056258593,0
-0.39671456967982,48.297076906792,0
-0.3968567396562,48.29708935891,0
-0.39699999999994,48.297093520174,0
-0.39714326034367,48.29708935891,0
-0.39728543032007,48.297076906792,0
-0.39742542786226,48.297056258593,0
-0.39756218744108,48.297027571468,0
-0.39769466817634,48.296991063757,0
-0.39782186176021,48.296947013322,0
-0.39794280013266,48.296895755433,0
-0.39805656285006,48.296837680214,0
-0.39816228409145,48.296773229675,0
-0.3982591592482,48.296702894346,0
-0.39834645104794,48.296627209546,0
-0.39842349516552,48.296546751304,0
-0.39848970527818,48.296462131976,0
-0.3985445775271,48.296373995586,0
-0.39858769435072,48.296283012921,0
-0.39861872766122,48.296189876428,0
-0.39863744133973,48.296095294941,0
-0.39864369303132,48.295999988289,0
-0.39863743522647,48.295904681815,0
-0.39861871562048,48.295810100857,0
-0.39858767674831,48.295716965227,0
-0.39854455489788,48.295625983733,0
-0.39848967830975,48.295537848786,0
-0.39842346467726,48.29545323113,0
-0.39834641796626,48.295372774738,0
-0.39825912457822,48.29529709191,0
-0.39816224888663,48.295226758615,0
-0.39805652818011,48.295162310109,0
-0.39794276705094,48.295104236862,0
-0.39782183127194,48.295052980823,0
-0.39769464120788,48.29500893206,0
-0.39756216481187,48.294972425792,0
-0.39742541025985,48.294943739838,0
-0.3972854182793,48.294923092502,0
-0.39714325423041,48.294910640912,0
-0.39699999999994,48.294906479827,0
-0.39685674576945,48.294910640912,0
-0.39671458172056,48.294923092502,0
-0.39657458974002,48.294943739838,0
-0.39643783518799,48.294972425792,0
-0.39630535879198,48.29500893206,0
-0.39617816872792,48.295052980823,0
-0.39605723294892,48.295104236862,0
-0.39594347181976,48.295162310109,0
-0.39583775111323,48.295226758615,0
-0.39574087542165,48.29529709191,0
-0.39565358203361,48.295372774738,0
-0.39557653532261,48.29545323113,0
-0.39551032169011,48.295537848786,0
-0.39545544510198,48.295625983733,0
-0.39541232325155,48.295716965227,0

-0.39538128437941,48.295810100857,0
-0.39536256477339,48.295904681815,0
-0.39535630696855,48.295999988289,0
-0.39699999999993,48.296,0
</coordinates>
</LinearRing>
</outerBoundaryIs>
</Polygon>
</Placemark>
<Placemark>
<name>542</name>
<styleUrl>#stylehap1</styleUrl>
<Polygon>
<outerBoundaryIs>
<LinearRing>
<coordinates>
-0.39300000000003,48.077,0
-0.39136331542342,48.076999988379,0
-0.39136954048208,48.07709529503,0
-0.39138817439093,48.077189876515,0
-0.39141907540137,48.077283013005,0
-0.39146200840067,48.077373995665,0
-0.39151664669927,48.07746213205,0
-0.39158257451562,48.077546751371,0
-0.39165929013879,48.077627209606,0
-0.39174620974578,48.077702894399,0
-0.39184267184376,48.07777322972,0
-0.39194794230397,48.077837680251,0
-0.39206121994857,48.077895755462,0
-0.39218164264845,48.077947013345,0
-0.39230829388498,48.077991063773,0
-0.39244020972616,48.078027571478,0
-0.39257638616398,48.078056258599,0
-0.39271578675692,48.078076906795,0
-0.39285735051975,48.078089358911,0
-0.39300000000003,48.078093520173,0
-0.39314264948032,48.078089358911,0
-0.39328421324315,48.078076906795,0
-0.39342361383609,48.078056258599,0
-0.3935597902739,48.078027571478,0
-0.39369170611506,48.077991063773,0
-0.39381835735161,48.077947013345,0
-0.39393878005149,48.077895755462,0
-0.39405205769609,48.077837680251,0
-0.39415732815627,48.07777322972,0
-0.39425379025428,48.077702894399,0
-0.39434070986127,48.077627209606,0
-0.39441742548444,48.077546751371,0
-0.39448335330079,48.07746213205,0
-0.3945379915994,48.077373995665,0
-0.39458092459866,48.077283013005,0
-0.39461182560914,48.077189876515,0
-0.39463045951798,48.07709529503,0
-0.39463668457665,48.076999988379,0
-0.39463045347744,48.076904681905,0
-0.39461181371157,48.076810100944,0
-0.39458090720564,48.076716965311,0

-0.39453796923934,48.076625983812,0
-0.39448332665308,48.07653784886,0
-0.39441739535881,48.076453231198,0
-0.39434067717303,48.076372774798,0
-0.39425375599665,48.076297091962,0
-0.39415729337019,48.07622675866,0
-0.39405202343849,48.076162310146,0
-0.39393874736325,48.076104236891,0
-0.39381832722596,48.076052980845,0
-0.39369167946738,48.076008932076,0
-0.39355976791384,48.075972425803,0
-0.39342359644306,48.075943739844,0
-0.39328420134561,48.075923092505,0
-0.39314264343978,48.075910640913,0
-0.39300000000003,48.075906479826,0
-0.39285735656028,48.075910640913,0
-0.39271579865445,48.075923092505,0
-0.39257640355701,48.075943739844,0
-0.39244023208623,48.075972425803,0
-0.39230832053268,48.076008932076,0
-0.39218167277411,48.076052980845,0
-0.39206125263681,48.076104236891,0
-0.39194797656157,48.076162310146,0
-0.39184270662987,48.07622675866,0
-0.39174624400341,48.076297091962,0
-0.39165932282703,48.076372774798,0
-0.39158260464125,48.076453231198,0
-0.39151667334698,48.07653784886,0
-0.39146203076073,48.076625983812,0
-0.39141909279443,48.076716965311,0
-0.39138818628847,48.076810100944,0
-0.39136954652262,48.076904681905,0
-0.39136331542342,48.076999988379,0
-0.39300000000003,48.077,0
</coordinates>
</LinearRing>
</outerBoundaryIs>
</Polygon>
</Placemark>
<Placemark>
<name>543</name>
<styleUrl>#stylehap1</styleUrl>
<Polygon>
<outerBoundaryIs>
<LinearRing>
<coordinates>
-0.39300000000003,48.354,0
-0.39135443674823,48.353999988266,0
-0.39136069554686,48.354095294918,0
-0.39137943051203,48.354189876405,0
-0.39141049912705,48.354283012899,0
-0.39145366500454,48.354373995565,0
-0.39150859968354,48.354462131956,0
-0.39157488512757,48.354546751286,0
-0.39165201690451,48.35462720953,0
-0.39173940802469,48.354702894332,0
-0.391836393340699,48.354773229663,0

-0.39194223494024,48.354837680204,0
-0.39205612710035,48.354895755425,0
-0.39217720308107,48.354947013316,0
-0.39230454139145,48.354991063753,0
-0.39243717286983,48.355027571465,0
-0.39257408806106,48.355056258591,0
-0.39271424490039,48.355076906791,0
-0.39285657664614,48.35508935891,0
-0.39300000000003,48.355093520174,0
-0.39314342335393,48.35508935891,0
-0.39328575509967,48.355076906791,0
-0.393425911939,48.355056258591,0
-0.39356282713023,48.355027571465,0
-0.39369545860861,48.354991063753,0
-0.39382279691899,48.354947013316,0
-0.39394387289972,48.354895755425,0
-0.39405776505982,48.354837680204,0
-0.39416360659307,48.354773229663,0
-0.39426059197537,48.354702894332,0
-0.39434798309552,48.35462720953,0
-0.39442511487249,48.354546751286,0
-0.39449140031649,48.354462131956,0
-0.39454633499552,48.354373995565,0
-0.39458950087302,48.354283012899,0
-0.39462056948804,48.354189876405,0
-0.3946393044532,48.354095294918,0
-0.39464556325183,48.353999988266,0
-0.39463929832051,48.353904681792,0
-0.39462055740898,48.353810100834,0
-0.39458948321463,48.353716965205,0
-0.39454631229433,48.353625983712,0
-0.39449137326229,48.353537848767,0
-0.39442508428726,48.353453231113,0
-0.39434794990862,48.353372774722,0
-0.39426055719514,48.353297091896,0
-0.3941635712763,48.353226758603,0
-0.39405773027959,48.353162310099,0
-0.39394383971279,48.353104236854,0
-0.39382276633376,48.353052980817,0
-0.39369543155441,48.353008932056,0
-0.39356280442903,48.35297242579,0
-0.39342589428061,48.352943739837,0
-0.39328574302061,48.352923092501,0
-0.39314341722123,48.352910640912,0
-0.39300000000003,48.352906479826,0
-0.39285658277883,48.352910640912,0
-0.39271425697945,48.352923092501,0
-0.39257410571945,48.352943739837,0
-0.39243719557103,48.35297242579,0
-0.39230456844566,48.353008932056,0
-0.39217723366628,48.353052980817,0
-0.39205616028727,48.353104236854,0
-0.39194226972047,48.353162310099,0
-0.39183642872377,48.353226758603,0
-0.39173944280492,48.353297091896,0
-0.39165205009144,48.353372774722,0
-0.39157491571278,48.353453231113,0

-0.39150862673778,48.353537848767,0
-0.39145368770574,48.353625983712,0
-0.39141051678543,48.353716965205,0
-0.39137944259106,48.353810100834,0
-0.39136070167956,48.353904681792,0
-0.39135443674823,48.353999988266,0
-0.39300000000003,48.354,0
</coordinates>
</LinearRing>
</outerBoundaryIs>
</Polygon>
</Placemark>
<Placemark>
<name>547</name>
<styleUrl>#stylehap1</styleUrl>
<Polygon>
<outerBoundaryIs>
<LinearRing>
<coordinates>
-0.38900000000001,48.333,0
-0.38735511458297,48.332999988274,0
-0.38736137080576,48.333095294926,0
-0.38738009805586,48.333189876413,0
-0.38741115387528,48.333283012907,0
-0.38745430197391,48.333373995572,0
-0.38750921402601,48.333462131963,0
-0.38757547216724,48.333546751292,0
-0.38765257217332,48.333627209536,0
-0.38773992729621,48.333702894337,0
-0.38783687272883,48.333773229667,0
-0.38794267066401,48.333837680207,0
-0.38805651590945,48.333895755428,0
-0.388177542016,48.333947013318,0
-0.38830482787232,48.333991063754,0
-0.38843740471599,48.334027571466,0
-0.38857426350772,48.334056258592,0
-0.38871436261209,48.334076906791,0
-0.38885663572683,48.33408935891,0
-0.38900000000002,48.334093520174,0
-0.3891433642732,48.33408935891,0
-0.38928563738794,48.334076906791,0
-0.38942573649229,48.334056258592,0
-0.38956259528401,48.334027571466,0
-0.38969517212772,48.333991063754,0
-0.38982245798403,48.333947013318,0
-0.38994348409058,48.333895755428,0
-0.39005732933603,48.333837680207,0
-0.3901631272712,48.333773229667,0
-0.39026007270383,48.333702894337,0
-0.39034742782671,48.333627209536,0
-0.39042452783279,48.333546751292,0
-0.39049078597402,48.333462131963,0
-0.39054569802613,48.333373995572,0
-0.39058884612475,48.333283012907,0
-0.39061990194415,48.333189876413,0
-0.39063862919428,48.333095294926,0
-0.39064488541706,48.332999988274,0

-0.39063862306863,48.3329046818,0
-0.39061988987898,48.332810100842,0
-0.39058882848664,48.332716965213,0
-0.39054567535104,48.332625983719,0
-0.39049075895088,48.332537848774,0
-0.3904244972827,48.332453231119,0
-0.39034739467793,48.332372774728,0
-0.39026003796354,48.332297091901,0
-0.39016309199501,48.332226758607,0
-0.39005729459577,48.332162310103,0
-0.3899434509418,48.332104236857,0
-0.38982242743394,48.332052980819,0
-0.38969514510458,48.332008932057,0
-0.38956257260892,48.331972425791,0
-0.3894257188542,48.331943739837,0
-0.38928562532277,48.331923092501,0
-0.38914335814755,48.331910640912,0
-0.38900000000002,48.331906479827,0
-0.38885664185248,48.331910640912,0
-0.38871437467726,48.331923092501,0
-0.38857428114583,48.331943739837,0
-0.38843742739111,48.331972425791,0
-0.38830485489543,48.332008932057,0
-0.38817757256607,48.332052980819,0
-0.38805654905821,48.332104236857,0
-0.38794270540426,48.332162310103,0
-0.387836908005,48.332226758607,0
-0.38773996203646,48.332297091901,0
-0.38765260532208,48.332372774728,0
-0.38757550271731,48.332453231119,0
-0.38750924104912,48.332537848774,0
-0.387454324649,48.332625983719,0
-0.38741117151336,48.332716965213,0
-0.38738011012103,48.332810100842,0
-0.3873613769314,48.3329046818,0
-0.38735511458297,48.332999988274,0
-0.38900000000001,48.333,0
</coordinates>
</LinearRing>
</outerBoundaryIs>
</Polygon>
</Placemark>
<Placemark>
<name>548</name>
<styleUrl>#stylehap1</styleUrl>
<Polygon>
<outerBoundaryIs>
<LinearRing>
<coordinates>
-0.38900000000001,47.967,0
-0.38736680417449,47.966999988424,0
-0.38737301597546,47.967095295075,0
-0.38739161017559,47.967189876558,0
-0.38742244532815,47.967283013046,0
-0.38746528682113,47.967373995704,0
-0.38751980866118,47.967462132086,0
-0.38758559595271,47.967546751405,0

-0.38766214805408,47.967627209636,0
-0.38774888238655,47.967702894425,0
-0.38784513886727,47.967773229742,0
-0.3879501849323,47.967837680269,0
-0.38806322111168,47.967895755477,0
-0.38818338711403,47.967947013356,0
-0.38830976837454,47.967991063781,0
-0.38844140301612,47.968027571484,0
-0.38857728917117,47.968056258602,0
-0.38871639260773,47.968076906796,0
-0.38885765460246,47.968089358911,0
-0.38900000000002,47.968093520174,0
-0.38914234539754,47.968089358911,0
-0.3892836073923,47.968076906796,0
-0.38942271082886,47.968056258602,0
-0.38955859698389,47.968027571484,0
-0.38969023162549,47.967991063781,0
-0.389816612886,47.967947013356,0
-0.38993677888835,47.967895755477,0
-0.39004981506771,47.967837680269,0
-0.39015486113274,47.967773229742,0
-0.39025111761348,47.967702894425,0
-0.39033785194596,47.967627209636,0
-0.3904144040473,47.967546751405,0
-0.39048019133883,47.967462132086,0
-0.39053471317888,47.967373995704,0
-0.39057755467188,47.967283013046,0
-0.39060838982442,47.967189876558,0
-0.39062698402457,47.967095295075,0
-0.39063319582554,47.966999988424,0
-0.39062697802014,47.966904681949,0
-0.390608377998,47.966810100988,0
-0.3905775373828,47.966716965352,0
-0.39053469095248,47.966625983852,0
-0.3904801648504,47.966537848897,0
-0.39041437410174,47.966453231231,0
-0.39033781945311,47.966372774828,0
-0.39025108356063,47.966297091989,0
-0.39015482655456,47.966226758682,0
-0.39004978101486,47.966162310165,0
-0.3899367463955,47.966104236906,0
-0.38981658294042,47.966052980856,0
-0.38969020513707,47.966008932084,0
-0.38955857475749,47.965972425808,0
-0.38942269353978,47.965943739847,0
-0.38928359556588,47.965923092506,0
-0.38914233939312,47.965910640913,0
-0.38900000000002,47.965906479826,0
-0.38885766060689,47.965910640913,0
-0.38871640443416,47.965923092506,0
-0.38857730646023,47.965943739847,0
-0.38844142524255,47.965972425808,0
-0.38830979486294,47.966008932084,0
-0.38818341705959,47.966052980856,0
-0.38806325360453,47.966104236906,0
-0.38795021898515,47.966162310165,0
-0.38784517344545,47.966226758682,0

-0.38774891643941,47.966297091989,0
-0.38766218054693,47.966372774828,0
-0.3875856258983,47.966453231231,0
-0.3875198351496,47.966537848897,0
-0.38746530904755,47.966625983852,0
-0.38742246261721,47.966716965352,0
-0.38739162200204,47.966810100988,0
-0.38737302197989,47.966904681949,0
-0.38736680417449,47.966999988424,0
-0.38900000000001,47.967,0
</coordinates>
</LinearRing>
</outerBoundaryIs>
</Polygon>
</Placemark>
<Placemark>
<name>549</name>
<styleUrl>#stylehap1</styleUrl>
<Polygon>
<outerBoundaryIs>
<LinearRing>
<coordinates>
-0.38200000000006,48.442,0
-0.38035158780046,48.441999988229,0
-0.38035785742537,48.442095294881,0
-0.38037662481698,48.442189876369,0
-0.38040774721215,48.442283012865,0
-0.38045098781448,48.442373995532,0
-0.38050601759462,48.442462131926,0
-0.3805724177925,48.442546751259,0
-0.38064968310292,48.442627209506,0
-0.38073722552002,48.442702894311,0
-0.38083437881136,48.442773229645,0
-0.38094040358786,48.442837680189,0
-0.38105449293083,48.442895755413,0
-0.38117577853327,48.442947013307,0
-0.3813033373087,48.442991063746,0
-0.38143619841746,48.443027571461,0
-0.38157335065645,48.443056258589,0
-0.38171375015663,48.44307690679,0
-0.38185632832918,48.44308935891,0
-0.38200000000007,48.443093520173,0
-0.38214367167094,48.44308935891,0
-0.38228624984349,48.44307690679,0
-0.38242664934367,48.443056258589,0
-0.38256380158266,48.443027571461,0
-0.38269666269142,48.442991063746,0
-0.38282422146685,48.442947013307,0
-0.38294550706929,48.442895755413,0
-0.38305959641228,48.442837680189,0
-0.38316562118879,48.442773229645,0
-0.3832627744801,48.442702894311,0
-0.3833503168972,48.442627209506,0
-0.38342758220762,48.442546751259,0
-0.3834939824055,48.442462131926,0
-0.38354901218564,48.442373995532,0
-0.38359225278797,48.442283012865,0

-0.38362337518314,48.442189876369,0
-0.38364214257475,48.442095294881,0
-0.38364841219966,48.441999988229,0
-0.38364213641239,48.441904681756,0
-0.38362336304569,48.441810100799,0
-0.38359223504422,48.441716965171,0
-0.3835489893747,48.4416259836,0
-0.38349395522049,48.441537848737,0
-0.38342755147453,48.441453231085,0
-0.38335028354983,48.441372774698,0
-0.38326273953171,48.441297091874,0
-0.38316558570125,48.441226758585,0
-0.38305956146386,48.441162310084,0
-0.38294547372193,48.441104236842,0
-0.38282419073377,48.441052980808,0
-0.3826966355064,48.441008932049,0
-0.38256377877172,48.440972425785,0
-0.38242663159989,48.440943739834,0
-0.38228623770604,48.4409230925,0
-0.3821436655086,48.440910640911,0
-0.38200000000007,48.440906479826,0
-0.38185633449152,48.440910640911,0
-0.38171376229408,48.4409230925,0
-0.38157336840023,48.440943739834,0
-0.3814362212284,48.440972425785,0
-0.38130336449372,48.441008932049,0
-0.38117580926635,48.441052980808,0
-0.38105452627821,48.441104236842,0
-0.38094043853626,48.441162310084,0
-0.38083441429887,48.441226758585,0
-0.38073726046841,48.441297091874,0
-0.3806497164503,48.441372774698,0
-0.38057244852561,48.441453231085,0
-0.38050604477966,48.441537848737,0
-0.38045101062542,48.44162598368,0
-0.38040776495593,48.441716965171,0
-0.38037663695443,48.441810100799,0
-0.38035786358773,48.441904681756,0
-0.38035158780046,48.441999988229,0
-0.38200000000006,48.442,0
</coordinates>
</LinearRing>
</outerBoundaryIs>
</Polygon>
</Placemark>
<Placemark>
<name>551</name>
<styleUrl>#stylehap1</styleUrl>
<Polygon>
<outerBoundaryIs>
<LinearRing>
<coordinates>
-0.37400000000002,48.047,0
-0.37236426897642,48.046999988391,0
-0.37237049041147,48.047095295042,0
-0.37238911346706,48.047189876527,0
-0.37241999647707,48.047283013016,0

-0.37246290446567,48.047373995676,0
-0.37251751093357,48.04746213206,0
-0.37258340034137,48.04754675138,0
-0.37266007127042,48.047627209615,0
-0.37274694023778,48.047702894406,0
-0.372843346136,48.047773229726,0
-0.37294855526401,48.047837680256,0
-0.37306176691081,48.047895755466,0
-0.37318211944959,48.047947013348,0
-0.37330869689567,48.047991063775,0
-0.37344053587877,48.04802757148,0
-0.37357663297585,48.0480562586,0
-0.37371595234938,48.048076906795,0
-0.37385743363228,48.048089358911,0
-0.37400000000002,48.048093520174,0
-0.37414256636775,48.048089358911,0
-0.37428404765068,48.048076906795,0
-0.37442336702419,48.0480562586,0
-0.37455946412129,48.04802757148,0
-0.37469130310437,48.047991063775,0
-0.37481788055047,48.047947013348,0
-0.37493823308923,48.047895755466,0
-0.37505144473602,48.047837680256,0
-0.37515665386404,48.047773229726,0
-0.37525305976228,48.047702894406,0
-0.37533992872965,48.047627209615,0
-0.37541659965867,48.04754675138,0
-0.37548248906646,48.04746213206,0
-0.37553709553439,48.047373995676,0
-0.37558000352297,48.047283013016,0
-0.375610886533,48.047189876527,0
-0.37562950958859,48.047095295042,0
-0.37563573102362,48.046999988391,0
-0.37562950355793,48.046904681917,0
-0.3756108746549,48.046810100956,0
-0.37557998615836,48.046716965322,0
-0.37553707321086,48.046625983823,0
-0.37548246246234,48.04653784887,0
-0.37541656958227,48.046453231207,0
-0.37533989609484,48.046372774806,0
-0.37525302556065,48.046297091969,0
-0.37515661913482,48.046226758666,0
-0.37505141053442,48.046162310151,0
-0.37493820045442,48.046104236895,0
-0.37481785047408,48.046052980848,0
-0.37469127650025,48.046008932078,0
-0.37455944179776,48.045972425804,0
-0.37442334965958,48.045943739845,0
-0.37428403577258,48.045923092505,0
-0.37414256033709,48.045910640913,0
-0.37400000000002,48.045906479826,0
-0.37385743966294,48.045910640913,0
-0.37371596422748,48.045923092505,0
-0.37357665034048,48.045943739845,0
-0.3734405582023,48.045972425804,0
-0.37330872349981,48.046008932078,0
-0.37318214952598,48.046052980848,0

-0.37306179954562,48.046104236895,0
-0.37294858946564,48.046162310151,0
-0.37284338086524,48.046226758666,0
-0.37274697443938,48.046297091969,0
-0.37266010390522,48.046372774806,0
-0.37258343041776,48.046453231207,0
-0.37251753753772,48.04653784887,0
-0.37246292678917,48.046625983823,0
-0.3724200138417,48.046716965322,0
-0.37238912534513,48.046810100956,0
-0.37237049644213,48.046904681917,0
-0.37236426897642,48.046999988391,0
-0.37400000000002,48.047,0
</coordinates>
</LinearRing>
</outerBoundaryIs>
</Polygon>
</Placemark>
<Placemark>
<name>552</name>
<styleUrl>#stylehap1</styleUrl>
<Polygon>
<outerBoundaryIs>
<LinearRing>
<coordinates>
-0.37200000000007,48.464,0
-0.37035087341313,48.46399998822,0
-0.37035714575277,48.464095294872,0
-0.37037592127546,48.464189876361,0
-0.37040705715626,48.464283012856,0
-0.37045031649621,48.464373995524,0
-0.3705053701234,48.464462131919,0
-0.37057179909634,48.464546751252,0
-0.37064909789093,48.4646272095,0
-0.37073667824645,48.464702894306,0
-0.3708338736418,48.46477322964,0
-0.37093994436742,48.464837680185,0
-0.37105408315496,48.46489575541,0
-0.37117542132105,48.464947013305,0
-0.37130303537916,48.464991063745,0
-0.37143595406883,48.46502757146,0
-0.37157316574869,48.465056258588,0
-0.37171362609726,48.46507690679,0
-0.3718562660625,48.46508935891,0
-0.37200000000007,48.465093520174,0
-0.37214373393765,48.46508935891,0
-0.37228637390289,48.46507690679,0
-0.37242683425145,48.465056258588,0
-0.37256404593132,48.46502757146,0
-0.37269696462099,48.464991063745,0
-0.3728245786791,48.464947013305,0
-0.37294591684518,48.46489575541,0
-0.37306005563273,48.464837680185,0
-0.37316612635835,48.46477322964,0
-0.37326332175367,48.464702894306,0
-0.37335090210919,48.4646272095,0
-0.37342820090378,48.464546751252,0

-0.37349462987675,48.464462131919,0
-0.37354968350394,48.464373995524,0
-0.37359294284389,48.464283012856,0
-0.37362407872466,48.464189876361,0
-0.37364285424737,48.464095294872,0
-0.373649126587,48.46399998822,0
-0.37364284807758,48.463904681747,0
-0.37362406657258,48.46381010079,0
-0.37359292507869,48.463716965162,0
-0.37354966066544,48.463625983672,0
-0.37349460265888,48.463537848729,0
-0.37342817013356,48.463453231078,0
-0.37335086872156,48.463372774691,0
-0.37326328676309,48.463297091869,0
-0.37316609082797,48.46322675858,0
-0.37306002064212,48.463162310081,0
-0.37294588345755,48.463104236839,0
-0.37282454790889,48.463052980805,0
-0.37269693740313,48.463008932048,0
-0.37256402309283,48.462972425784,0
-0.37242681648625,48.462943739834,0
-0.37228636175078,48.4629230925,0
-0.37214372776786,48.462910640911,0
-0.37200000000007,48.462906479827,0
-0.37185627223229,48.462910640911,0
-0.37171363824935,48.4629230925,0
-0.3715731835139,48.462943739834,0
-0.37143597690732,48.462972425784,0
-0.37130306259702,48.463008932048,0
-0.37117545209126,48.463052980805,0
-0.37105411654259,48.463104236839,0
-0.37093997935803,48.463162310081,0
-0.37083390917218,48.46322675858,0
-0.37073671323706,48.463297091869,0
-0.37064913127859,48.463372774691,0
-0.37057182986658,48.463453231078,0
-0.37050539734124,48.463537848729,0
-0.3704503393347,48.463625983672,0
-0.37040707492146,48.463716965162,0
-0.37037593342757,48.46381010079,0
-0.37035715192257,48.463904681747,0
-0.37035087341313,48.46399998822,0
-0.37200000000007,48.464,0
</coordinates>
</LinearRing>
</outerBoundaryIs>
</Polygon>
</Placemark>
<Placemark>
<name>553</name>
<styleUrl>#stylehap1</styleUrl>
<Polygon>
<outerBoundaryIs>
<LinearRing>
<coordinates>
-0.36799999999994,48.418,0
-0.36635236614953,48.417999988239,0

-0.36635863281662,48.418095294891,0
-0.36637739134914,48.418189876379,0
-0.36640849905127,48.418283012874,0
-0.36645171923834,48.418373995541,0
-0.36650672303629,48.418462131934,0
-0.36657309188275,48.418546751266,0
-0.36665032071104,48.418627209512,0
-0.36673782179294,48.418702894317,0
-0.36683492921053,48.418773229649,0
-0.36694090392391,48.418837680193,0
-0.36705493939538,48.418895755416,0
-0.36717616772793,48.41894701331,0
-0.36730366627104,48.418991063748,0
-0.36743646464343,48.419027571462,0
-0.36757355211958,48.419056258589,0
-0.36771388532339,48.41907690679,0
-0.3678563961707,48.41908935891,0
-0.36799999999993,48.419093520174,0
-0.36814360382918,48.41908935891,0
-0.36828611467647,48.41907690679,0
-0.36842644788028,48.419056258589,0
-0.36856353535645,48.419027571462,0
-0.36869633372885,48.418991063748,0
-0.36882383227193,48.41894701331,0
-0.36894506060448,48.418895755416,0
-0.36905909607595,48.418837680193,0
-0.36916507078933,48.418773229649,0
-0.36926217820695,48.418702894317,0
-0.36934967928882,48.418627209512,0
-0.36942690811714,48.418546751266,0
-0.36949327696357,48.418462131934,0
-0.36954828076153,48.418373995541,0
-0.36959150094862,48.418283012874,0
-0.36962260865072,48.418189876379,0
-0.36964136718324,48.418095294891,0
-0.36964763385036,48.417999988239,0
-0.36964136102899,48.417904681766,0
-0.36962259652925,48.417810100808,0
-0.36959148322817,48.41771696518,0
-0.36954825798059,48.417625983688,0
-0.36949324981431,48.417537848745,0
-0.36942687742445,48.417453231093,0
-0.36934964598531,48.417372774704,0
-0.36926214330451,48.41729709188,0
-0.36916503534849,48.41722675859,0
-0.36905906117354,48.417162310088,0
-0.36894502730097,48.417104236845,0
-0.36882380157925,48.41705298081,0
-0.36869630657958,48.417008932051,0
-0.36856351257549,48.416972425787,0
-0.36842643015986,48.416943739835,0
-0.36828610255499,48.4169230925,0
-0.36814359767494,48.416910640912,0
-0.36799999999993,48.416906479827,0
-0.36785640232495,48.416910640912,0
-0.36771389744489,48.4169230925,0
-0.36757356984001,48.416943739835,0

-0.36743648742437,48.416972425787,0
-0.36730369342031,48.417008932051,0
-0.36717619842062,48.41705298081,0
-0.36705497269889,48.417104236845,0
-0.36694093882635,48.417162310088,0
-0.3668349646514,48.41722675859,0
-0.36673785669536,48.41729709188,0
-0.36665035401455,48.417372774704,0
-0.36657312257544,48.417453231093,0
-0.36650675018555,48.417537848745,0
-0.3664517420193,48.417625983688,0
-0.36640851677169,48.41771696518,0
-0.36637740347064,48.417810100808,0
-0.36635863897087,48.417904681766,0
-0.36635236614953,48.417999988239,0
-0.36799999999994,48.418,0
</coordinates>
</LinearRing>
</outerBoundaryIs>
</Polygon>
</Placemark>
<Placemark>
<name>555</name>
<styleUrl>#stylehap1</styleUrl>
<Polygon>
<outerBoundaryIs>
<LinearRing>
<coordinates>
-0.36699999999996,48.233,0
-0.36535833170045,48.232999988315,0
-0.36536457569784,48.233095294967,0
-0.36538326633104,48.233189876453,0
-0.36541426142026,48.233283012945,0
-0.36545732513732,48.233373995609,0
-0.36551212979835,48.233462131997,0
-0.36557825835605,48.233546751323,0
-0.3656552075721,48.233627209564,0
-0.36574239184595,48.233702894361,0
-0.36583914767065,48.233773229688,0
-0.36594473868201,48.233837680224,0
-0.36605836126266,48.233895755441,0
-0.36617915065807,48.233947013329,0
-0.36630618755861,48.233991063762,0
-0.36643850509683,48.234027571471,0
-0.36657509620709,48.234056258594,0
-0.36671492129154,48.234076906793,0
-0.36685691613375,48.23408935891,0
-0.36699999999997,48.234093520174,0
-0.36714308386617,48.23408935891,0
-0.36728507870838,48.234076906793,0
-0.36742490379282,48.234056258594,0
-0.36756149490309,48.234027571471,0
-0.36769381244131,48.233991063762,0
-0.36782084934184,48.233947013329,0
-0.36794163873726,48.233895755441,0
-0.36805526131791,48.233837680224,0
-0.36816085232927,48.233773229688,0

-0.36825760815397,48.233702894361,0
-0.36834479242781,48.233627209564,0
-0.36842174164387,48.233546751323,0
-0.36848787020157,48.233462131997,0
-0.3685426748626,48.233373995609,0
-0.36858573857966,48.233283012945,0
-0.36861673366888,48.233189876453,0
-0.36863542430207,48.233095294967,0
-0.36864166829947,48.232999988315,0
-0.36863541820986,48.232904681841,0
-0.36861672166956,48.232810100882,0
-0.36858572103781,48.232716965251,0
-0.36854265231122,48.232625983756,0
-0.36848784332588,48.232537848807,0
-0.36842171126049,48.23245323115,0
-0.36834475945991,48.232372774755,0
-0.36825757360327,48.232297091925,0
-0.36816081724557,48.232226758628,0
-0.36805522676721,48.23216231012,0
-0.36794160576936,48.23210423687,0
-0.36782081895846,48.232052980829,0
-0.36769378556562,48.232008932065,0
-0.36756147235173,48.231972425795,0
-0.36742488625097,48.23194373984,0
-0.36728506670904,48.231923092503,0
-0.36714307777395,48.231910640912,0
-0.36699999999997,48.231906479826,0
-0.36685692222599,48.231910640912,0
-0.36671493329088,48.231923092503,0
-0.36657511374895,48.23194373984,0
-0.36643852764818,48.231972425795,0
-0.3663062144343,48.232008932065,0
-0.36617918104146,48.232052980829,0
-0.36605839423055,48.23210423687,0
-0.36594477323273,48.23216231012,0
-0.36583918275435,48.232226758628,0
-0.36574242639665,48.232297091925,0
-0.36565524054,48.232372774755,0
-0.36557828873943,48.23245323115,0
-0.36551215667404,48.232537848807,0
-0.3654573476887,48.232625983756,0
-0.36541427896211,48.232716965251,0
-0.36538327833036,48.232810100882,0
-0.36536458179008,48.232904681841,0
-0.36535833170045,48.232999988315,0
-0.36699999999996,48.233,0
</coordinates>
</LinearRing>
</outerBoundaryIs>
</Polygon>
</Placemark>
<Placemark>
<name>556</name>
<styleUrl>#stylehap1</styleUrl>
<Polygon>
<outerBoundaryIs>
<LinearRing>

<coordinates>
-0.36400000000003,48.443,0
-0.362351555347,48.442999988229,0
-0.36235782509523,48.443095294881,0
-0.36237659285622,48.443189876369,0
-0.36240771586401,48.443283012864,0
-0.36245095731757,48.443373995532,0
-0.36250598818102,48.443462131926,0
-0.36257238968611,48.443546751258,0
-0.36264965651767,48.443627209505,0
-0.36273720065823,48.443702894311,0
-0.36283435586228,48.443773229644,0
-0.3629403827262,48.443837680189,0
-0.36305447431535,48.443895755413,0
-0.36317576230566,48.443947013307,0
-0.36330332359252,48.443991063746,0
-0.36343618731706,48.444027571461,0
-0.36357334225637,48.444056258589,0
-0.36371374452078,48.44407690679,0
-0.36385632550047,48.44408935891,0
-0.36400000000004,48.444093520174,0
-0.36414367449959,48.44408935891,0
-0.36428625547928,48.44407690679,0
-0.36442665774369,48.444056258589,0
-0.364563812683,48.444027571461,0
-0.36469667640757,48.443991063746,0
-0.3648242376944,48.443947013307,0
-0.36494552568471,48.443895755413,0
-0.36505961727387,48.443837680189,0
-0.36516564413778,48.443773229644,0
-0.36526279934183,48.443702894311,0
-0.36535034348239,48.443627209505,0
-0.36542761031395,48.443546751258,0
-0.36549401181904,48.443462131926,0
-0.36554904268249,48.443373995532,0
-0.36559228413605,48.443283012864,0
-0.36562340714385,48.443189876369,0
-0.36564217490483,48.443095294881,0
-0.36564844465307,48.442999988229,0
-0.36564216874216,48.442904681755,0
-0.36562339500573,48.442810100798,0
-0.36559226639133,48.44271696517,0
-0.36554901987031,48.442625983679,0
-0.36549398463252,48.442537848736,0
-0.36542757957916,48.442453231085,0
-0.3653503101332,48.442372774697,0
-0.36526276439151,48.442297091874,0
-0.36516560864829,48.442226758584,0
-0.36505958232357,48.442162310084,0
-0.3649454923355,48.442104236842,0
-0.3648242069596,48.442052980807,0
-0.36469664922105,48.442008932049,0
-0.36456378987081,48.441972425785,0
-0.36442663999897,48.441943739834,0
-0.36428624334116,48.4419230925,0
-0.3641436683369,48.441910640911,0
-0.36400000000004,48.441906479826,0

-0.36385633166316,48.441910640911,0
-0.3637137566589,48.4419230925,0
-0.36357336000112,48.441943739834,0
-0.36343621012928,48.441972425785,0
-0.36330335077903,48.442008932049,0
-0.36317579304046,48.442052980807,0
-0.36305450766456,48.442104236842,0
-0.3629404176765,48.442162310084,0
-0.36283439135177,48.442226758584,0
-0.36273723560856,48.442297091874,0
-0.36264968986686,48.442372774697,0
-0.3625724204209,48.442453231085,0
-0.36250601536754,48.442537848736,0
-0.36245098012975,48.442625983679,0
-0.36240773360873,48.44271696517,0
-0.36237660499433,48.442810100798,0
-0.3623578312579,48.442904681755,0
-0.362351555347,48.442999988229,0
-0.36400000000003,48.443,0
</coordinates>
</LinearRing>
</outerBoundaryIs>
</Polygon>
</Placemark>
<Placemark>
<name>557</name>
<styleUrl>#stylehap1</styleUrl>
<Polygon>
<outerBoundaryIs>
<LinearRing>
<coordinates>
-0.36300000000006,48.1,0
-0.36136258330874,48.09999998837,0
-0.36136881114952,48.100095295021,0
-0.36138745339124,48.100189876506,0
-0.36141836822199,48.100283012996,0
-0.36146132042387,48.100373995657,0
-0.36151598316132,48.100462132042,0
-0.36158194046681,48.100546751364,0
-0.36165869040503,48.1006272096,0
-0.36174564889191,48.100702894393,0
-0.3618421541387,48.100773229715,0
-0.36194747168821,48.100837680247,0
-0.36206080000434,48.100895755459,0
-0.36218127657221,48.100947013342,0
-0.36230798446324,48.100991063771,0
-0.36243995931417,48.101027571477,0
-0.36257619666785,48.101056258598,0
-0.36271565961908,48.101076906794,0
-0.36285728670797,48.101089358911,0
-0.36300000000006,48.101093520174,0
-0.36314271329214,48.101089358911,0
-0.36328434038104,48.101076906794,0
-0.36342380333226,48.101056258598,0
-0.36356004068594,48.101027571477,0
-0.36369201553688,48.100991063771,0
-0.36381872342789,48.100947013342,0

-0.36393919999578,48.100895755459,0
-0.36405252831191,48.100837680247,0
-0.36415784586142,48.100773229715,0
-0.3642543511082,48.100702894393,0
-0.36434130959506,48.1006272096,0
-0.36441805953331,48.100546751364,0
-0.3644840168388,48.100462132042,0
-0.36453867957625,48.100373995657,0
-0.36458163177813,48.100283012996,0
-0.36461254660888,48.100189876506,0
-0.36463118885059,48.100095295021,0
-0.36463741669138,48.09999998837,0
-0.36463118280248,48.099904681895,0
-0.36461253469638,48.099810100935,0
-0.36458161436324,48.099716965302,0
-0.3645386571881,48.099625983804,0
-0.36448399015766,48.099537848852,0
-0.36441802936984,48.099453231191,0
-0.3643412768658,48.099372774792,0
-0.36425431680758,48.099297091957,0
-0.36415781103165,48.099226758655,0
-0.36405249401128,48.099162310142,0
-0.36393916726652,48.099104236888,0
-0.36381869326445,48.099052980843,0
-0.36369198885574,48.099008932074,0
-0.36356001829779,48.098972425802,0
-0.36342378591738,48.098943739844,0
-0.36328432846854,48.098923092504,0
-0.36314270724403,48.098910640913,0
-0.36300000000006,48.098906479827,0
-0.36285729275609,48.098910640913,0
-0.36271567153158,48.098923092504,0
-0.36257621408274,48.098943739844,0
-0.36243998170233,48.098972425802,0
-0.36230801114438,48.099008932074,0
-0.36218130673567,48.099052980843,0
-0.3620608327336,48.099104236888,0
-0.36194750598884,48.099162310142,0
-0.36184218896844,48.099226758655,0
-0.36174568319254,48.099297091957,0
-0.36165872313432,48.099372774792,0
-0.36158197063025,48.099453231191,0
-0.36151600984246,48.099537848852,0
-0.36146134281199,48.099625983804,0
-0.36141838563687,48.099716965302,0
-0.36138746530374,48.099810100935,0
-0.36136881719764,48.099904681895,0
-0.36136258330874,48.09999998837,0
-0.36300000000006,48.1,0
</coordinates>
</LinearRing>
</outerBoundaryIs>
</Polygon>
</Placemark>
<Placemark>
<name>558</name>
<styleUrl>#stylehap1</styleUrl>

<Polygon>
<outerBoundaryIs>
<LinearRing>
<coordinates>
-0.36300000000006,48.145,0
-0.36136114825002,48.144999988351,0
-0.36136738154419,48.145095295003,0
-0.36138604011966,48.145189876488,0
-0.36141698204031,48.145283012979,0
-0.36145997188224,48.14537399564,0
-0.36151468252361,48.145462132027,0
-0.3615806976324,48.14554675135,0
-0.36165751483353,48.145627209588,0
-0.36174454953097,48.145702894383,0
-0.36184113935611,48.145773229706,0
-0.36194654920807,48.145837680239,0
-0.36205997684821,48.145895755453,0
-0.36218055900576,48.145947013338,0
-0.36230737794841,48.145991063768,0
-0.36243946846767,48.146027571475,0
-0.36257582522591,48.146056258597,0
-0.36271541040911,48.146076906794,0
-0.36285716162694,48.146089358911,0
-0.36300000000006,48.146093520174,0
-0.36314283837318,48.146089358911,0
-0.36328458959101,48.146076906794,0
-0.36342417477421,48.146056258597,0
-0.36356053153245,48.146027571475,0
-0.36369262205171,48.145991063768,0
-0.36381944099435,48.145947013338,0
-0.3639400231519,48.145895755453,0
-0.36405345079205,48.145837680239,0
-0.36415886064401,48.145773229706,0
-0.36425545046914,48.145702894383,0
-0.36434248516659,48.145627209588,0
-0.36441930236772,48.14554675135,0
-0.36448531747651,48.145462132027,0
-0.36454002811787,48.14537399564,0
-0.36458301795981,48.145283012979,0
-0.36461395988046,48.145189876488,0
-0.36463261845593,48.145095295003,0
-0.3646388517501,48.144999988351,0
-0.36463261239293,48.144904681877,0
-0.3646139479387,48.144810100917,0
-0.3645830005021,48.144716965285,0
-0.36454000567466,48.144625983788,0
-0.36448529072972,48.144537848837,0
-0.36441927213006,48.144453231177,0
-0.36434245235683,48.14437277478,0
-0.36425541608417,48.144297091946,0
-0.36415882572859,48.144226758646,0
-0.36405341640707,48.144162310135,0
-0.36393999034214,48.144104236882,0
-0.36381941075672,48.144052980838,0
-0.36369259530495,48.144008932071,0
-0.36356050908926,48.1439724258,0
-0.3634241573165,48.143943739843,0

-0.36328457764922,48.143923092504,0
-0.36314283231018,48.143910640912,0
-0.36300000000006,48.143906479826,0
-0.36285716768991,48.143910640912,0
-0.36271542235089,48.143923092504,0
-0.36257584268362,48.143943739843,0
-0.36243949091086,48.1439724258,0
-0.36230740469517,48.144008932071,0
-0.3621805892434,48.144052980838,0
-0.36206000965798,48.144104236882,0
-0.36194658359305,48.144162310135,0
-0.36184117427153,48.144226758646,0
-0.36174458391595,48.144297091946,0
-0.36165754764329,48.14437277478,0
-0.36158072787003,48.144453231177,0
-0.36151470927037,48.144537848837,0
-0.36145999432543,48.144625983788,0
-0.36141699949801,48.144716965285,0
-0.36138605206142,48.144810100917,0
-0.36136738760719,48.144904681877,0
-0.36136114825002,48.144999988351,0
-0.36300000000006,48.145,0
</coordinates>
</LinearRing>
</outerBoundaryIs>
</Polygon>
</Placemark>
<Placemark>
<name>559</name>
<styleUrl>#stylehap1</styleUrl>
<Polygon>
<outerBoundaryIs>
<LinearRing>
<coordinates>
-0.36300000000006,48.401,0
-0.36135291686095,48.400999988246,0
-0.36135918143528,48.401095294898,0
-0.36137793369966,48.401189876386,0
-0.36140903100591,48.401283012881,0
-0.36145223674842,48.401373995547,0
-0.361507222163,48.40146213194,0
-0.36157356882713,48.401546751271,0
-0.36165077184298,48.401627209517,0
-0.36173824367863,48.401702894321,0
-0.36183531863887,48.401773229653,0
-0.36194125793069,48.401837680196,0
-0.36205525528604,48.401895755419,0
-0.36217644309799,48.401947013311,0
-0.36230389902444,48.401991063749,0
-0.36243665300853,48.402027571463,0
-0.36257369466253,48.40205625859,0
-0.36271398095919,48.40207690679,0
-0.36285644417133,48.40208935891,0
-0.36300000000006,48.402093520174,0
-0.36314355582876,48.40208935891,0
-0.36328601904093,48.40207690679,0
-0.36342630533759,48.40205625859,0

-0.36356334699159,48.402027571463,0
-0.36369610097565,48.401991063749,0
-0.3638235569021,48.401947013311,0
-0.36394474471408,48.401895755419,0
-0.36405874206943,48.401837680196,0
-0.36416468136125,48.401773229653,0
-0.36426175632148,48.401702894321,0
-0.36434922815714,48.401627209517,0
-0.36442643117299,48.401546751271,0
-0.36449277783712,48.40146213194,0
-0.36454776325169,48.401373995547,0
-0.36459096899421,48.401283012881,0
-0.36462206630045,48.401189876386,0
-0.36464081856481,48.401095294898,0
-0.36464708313917,48.400999988246,0
-0.36464081241631,48.400904681773,0
-0.36462205419025,48.400810100815,0
-0.36459095129028,48.400716965186,0
-0.36454774049198,48.400625983695,0
-0.36449275071314,48.400537848751,0
-0.36442640050888,48.400453231098,0
-0.36434919488464,48.400372774709,0
-0.36426172145156,48.400297091884,0
-0.36416464595341,48.400226758593,0
-0.36405870719951,48.400162310091,0
-0.36394471144159,48.400104236848,0
-0.36382352623802,48.400052980812,0
-0.36369607385168,48.400008932052,0
-0.36356332423187,48.399972425787,0
-0.36342628763365,48.399943739835,0
-0.36328600693073,48.399923092501,0
-0.36314354968027,48.399910640912,0
-0.36300000000006,48.399906479826,0
-0.36285645031985,48.399910640912,0
-0.36271399306939,48.399923092501,0
-0.36257371236646,48.399943739835,0
-0.36243667576825,48.399972425787,0
-0.36230392614844,48.400008932052,0
-0.3621764737621,48.400052980812,0
-0.36205528855853,48.400104236848,0
-0.36194129280061,48.400162310091,0
-0.36183535404671,48.400226758593,0
-0.36173827854856,48.400297091884,0
-0.36165080511545,48.400372774709,0
-0.36157359949121,48.400453231098,0
-0.36150724928697,48.400537848751,0
-0.36145225950814,48.400625983695,0
-0.36140904870982,48.400716965186,0
-0.36137794580987,48.400810100815,0
-0.36135918758381,48.400904681773,0
-0.36135291686095,48.400999988246,0
-0.36300000000006,48.401,0
</coordinates>
</LinearRing>
</outerBoundaryIs>
</Polygon>
</Placemark>

<Placemark>
<name>560</name>
<styleUrl>#stylehap1</styleUrl>
<Polygon>
<outerBoundaryIs>
<LinearRing>
<coordinates>
-0.36300000000006,48.1,0
-0.36136258330874,48.09999998837,0
-0.36136881114952,48.100095295021,0
-0.36138745339124,48.100189876506,0
-0.36141836822199,48.100283012996,0
-0.36146132042387,48.100373995657,0
-0.36151598316132,48.100462132042,0
-0.36158194046681,48.100546751364,0
-0.36165869040503,48.1006272096,0
-0.36174564889191,48.100702894393,0
-0.3618421541387,48.100773229715,0
-0.36194747168821,48.100837680247,0
-0.36206080000434,48.100895755459,0
-0.36218127657221,48.100947013342,0
-0.36230798446324,48.100991063771,0
-0.36243995931417,48.101027571477,0
-0.36257619666785,48.101056258598,0
-0.36271565961908,48.101076906794,0
-0.36285728670797,48.101089358911,0
-0.36300000000006,48.101093520174,0
-0.36314271329214,48.101089358911,0
-0.36328434038104,48.101076906794,0
-0.36342380333226,48.101056258598,0
-0.36356004068594,48.101027571477,0
-0.36369201553688,48.100991063771,0
-0.36381872342789,48.100947013342,0
-0.36393919999578,48.100895755459,0
-0.36405252831191,48.100837680247,0
-0.36415784586142,48.100773229715,0
-0.3642543511082,48.100702894393,0
-0.36434130959506,48.1006272096,0
-0.36441805953331,48.100546751364,0
-0.3644840168388,48.100462132042,0
-0.36453867957625,48.100373995657,0
-0.36458163177813,48.100283012996,0
-0.36461254660888,48.100189876506,0
-0.36463118885059,48.100095295021,0
-0.36463741669138,48.09999998837,0
-0.36463118280248,48.099904681895,0
-0.36461253469638,48.099810100935,0
-0.36458161436324,48.099716965302,0
-0.3645386571881,48.099625983804,0
-0.36448399015766,48.099537848852,0
-0.36441802936984,48.099453231191,0
-0.3643412768658,48.099372774792,0
-0.36425431680758,48.099297091957,0
-0.36415781103165,48.099226758655,0
-0.36405249401128,48.099162310142,0
-0.36393916726652,48.099104236888,0
-0.36381869326445,48.099052980843,0

-0.36369198885574,48.099008932074,0
-0.36356001829779,48.098972425802,0
-0.36342378591738,48.098943739844,0
-0.36328432846854,48.098923092504,0
-0.36314270724403,48.098910640913,0
-0.36300000000006,48.098906479827,0
-0.36285729275609,48.098910640913,0
-0.36271567153158,48.098923092504,0
-0.36257621408274,48.098943739844,0
-0.36243998170233,48.098972425802,0
-0.36230801114438,48.099008932074,0
-0.36218130673567,48.099052980843,0
-0.3620608327336,48.099104236888,0
-0.36194750598884,48.099162310142,0
-0.36184218896844,48.099226758655,0
-0.36174568319254,48.099297091957,0
-0.36165872313432,48.099372774792,0
-0.36158197063025,48.099453231191,0
-0.36151600984246,48.099537848852,0
-0.36146134281199,48.099625983804,0
-0.36141838563687,48.099716965302,0
-0.36138746530374,48.099810100935,0
-0.36136881719764,48.099904681895,0
-0.36136258330874,48.09999998837,0
-0.36300000000006,48.1,0
</coordinates>
</LinearRing>
</outerBoundaryIs>
</Polygon>
</Placemark>
<Placemark>
<name>562</name>
<styleUrl>#stylehap1</styleUrl>
<Polygon>
<outerBoundaryIs>
<LinearRing>
<coordinates>
-0.36099999999999,48.376,0
-0.35935372579883,48.375999988256,0
-0.35935998729915,48.376095294909,0
-0.35937873035625,48.376189876396,0
-0.35940981239202,48.37628301289,0
-0.35945299691695,48.376373995557,0
-0.35950795532826,48.376462131949,0
-0.35957426940888,48.376546751279,0
-0.35965143450885,48.376627209524,0
-0.35973886338486,48.376702894327,0
-0.35983589066855,48.376773229658,0
-0.35994177792978,48.3768376802,0
-0.3600557192965,48.376895755422,0
-0.36017684758791,48.376947013314,0
-0.36030424091491,48.376991063751,0
-0.3604369296971,48.377027571464,0
-0.36057390404313,48.37705625859,0
-0.36071412143801,48.377076906791,0
-0.36085651467907,48.37708935891,0
-0.36099999999999,48.377093520174,0

-0.36114348532091,48.37708935891,0
-0.36128587856197,48.377076906791,0
-0.36142609595684,48.37705625859,0
-0.36156307030288,48.377027571464,0
-0.36169575908509,48.376991063751,0
-0.36182315241209,48.376947013314,0
-0.36194428070347,48.376895755422,0
-0.36205822207019,48.3768376802,0
-0.36216410933142,48.376773229658,0
-0.36226113661512,48.376702894327,0
-0.36234856549112,48.376627209524,0
-0.36242573059109,48.376546751279,0
-0.36249204467171,48.376462131949,0
-0.36254700308302,48.376373995557,0
-0.36259018760796,48.37628301289,0
-0.36262126964372,48.376189876396,0
-0.36264001270085,48.376095294909,0
-0.36264627420115,48.375999988256,0
-0.36264000656076,48.375904681783,0
-0.36262125755011,48.375810100825,0
-0.36259016992827,48.375716965196,0
-0.36254698035445,48.375625983704,0
-0.36249201758486,48.375537848759,0
-0.36242569996899,48.375453231106,0
-0.3623485322642,48.375372774716,0
-0.36226110179293,48.37529709189,0
-0.36216407397208,48.375226758598,0
-0.36205818724801,48.375162310096,0
-0.36194424747655,48.375104236851,0
-0.36182312178996,48.375052980814,0
-0.36169573199824,48.375008932054,0
-0.3615630475743,48.374972425789,0
-0.36142607827716,48.374943739836,0
-0.36128586646835,48.374923092501,0
-0.36114347918081,48.374910640912,0
-0.36099999999999,48.374906479826,0
-0.36085652081917,48.374910640912,0
-0.36071413353162,48.374923092501,0
-0.36057392172282,48.374943739836,0
-0.36043695242567,48.374972425789,0
-0.36030426800174,48.375008932054,0
-0.36017687821001,48.375052980814,0
-0.36005575252343,48.375104236851,0
-0.35994181275197,48.375162310096,0
-0.35983592602792,48.375226758598,0
-0.35973889820705,48.37529709189,0
-0.3596514677358,48.375372774716,0
-0.35957430003098,48.375453231106,0
-0.35950798241511,48.375537848759,0
-0.35945301964553,48.375625983704,0
-0.3594098300717,48.375716965196,0
-0.35937874244987,48.375810100825,0
-0.35935999343922,48.375904681783,0
-0.35935372579883,48.375999988256,0
-0.36099999999999,48.376,0
</coordinates>
</LinearRing>

</outerBoundaryIs>
</Polygon>
</Placemark>
<Placemark>
<name>563</name>
<styleUrl>#stylehap1</styleUrl>
<Polygon>
<outerBoundaryIs>
<LinearRing>
<coordinates>
-0.35900000000004,48.432,0
-0.35735191223711,48.431999988233,0
-0.35735818062911,48.432095294886,0
-0.35737694432802,48.432189876373,0
-0.35740806059874,48.432283012869,0
-0.35745129269144,48.432373995536,0
-0.3575063116415,48.43246213193,0
-0.35757269877127,48.432546751262,0
-0.357649948875,48.432627209509,0
-0.35773747406248,48.432702894313,0
-0.3578346082324,48.432773229647,0
-0.35794061214126,48.432837680191,0
-0.35805467902916,48.432895755414,0
-0.35817594076,48.432947013308,0
-0.35830347442898,48.432991063747,0
-0.35843630938757,48.433027571461,0
-0.35857343463169,48.433056258589,0
-0.35871380649776,48.43307690679,0
-0.35885635660736,48.43308935891,0
-0.35900000000004,48.433093520174,0
-0.35914364339273,48.43308935891,0
-0.3592861935023,48.43307690679,0
-0.3594265653684,48.433056258589,0
-0.35956369061252,48.433027571461,0
-0.35969652557109,48.432991063747,0
-0.35982405924009,48.432947013308,0
-0.35994532097091,48.432895755414,0
-0.3600593878588,48.432837680191,0
-0.36016539176767,48.432773229647,0
-0.36026252593761,48.432702894313,0
-0.36035005112509,48.432627209509,0
-0.36042730122879,48.432546751262,0
-0.36049368835856,48.43246213193,0
-0.36054870730863,48.432373995536,0
-0.36059193940135,48.432283012869,0
-0.36062305567205,48.432189876373,0
-0.36064181937095,48.432095294886,0
-0.36064808776297,48.431999988233,0
-0.360641813212,48.43190468176,0
-0.36062304354126,48.431810100803,0
-0.36059192166732,48.431716965175,0
-0.36054868451018,48.431625983683,0
-0.36049366118843,48.43153784874,0
-0.36042727051255,48.431453231088,0
-0.36035001779601,48.4313727747,0
-0.36026249100838,48.431297091877,0
-0.3601653562996,48.431226758587,0

-0.36005935292957,48.431162310086,0
-0.35994528764182,48.431104236843,0
-0.35982402852384,48.431052980809,0
-0.35969649840096,48.43100893205,0
-0.35956366781407,48.430972425786,0
-0.35942654763437,48.430943739835,0
-0.35928618137152,48.4309230925,0
-0.35914363723375,48.430910640912,0
-0.35900000000004,48.430906479826,0
-0.35885636276632,48.430910640912,0
-0.35871381862857,48.4309230925,0
-0.35857345236572,48.430943739835,0
-0.35843633218599,48.430972425786,0
-0.35830350159911,48.43100893205,0
-0.35817597147624,48.431052980809,0
-0.35805471235826,48.431104236843,0
-0.35794064707052,48.431162310086,0
-0.35783464370049,48.431226758587,0
-0.35773750899171,48.431297091877,0
-0.35764998220408,48.4313727747,0
-0.35757272948754,48.431453231088,0
-0.35750633881163,48.43153784874,0
-0.35745131548988,48.431625983683,0
-0.35740807833277,48.431716965175,0
-0.3573769564588,48.431810100803,0
-0.35735818678809,48.43190468176,0
-0.35735191223711,48.431999988233,0
-0.35900000000004,48.432,0
</coordinates>
</LinearRing>
</outerBoundaryIs>
</Polygon>
</Placemark>
<Placemark>
<name>564</name>
<styleUrl>#stylehap1</styleUrl>
<Polygon>
<outerBoundaryIs>
<LinearRing>
<coordinates>
-0.35500000000002,48.316,0
-0.35335566273575,48.315999988281,0
-0.35336191687551,48.316095294933,0
-0.35338063788659,48.31618987642,0
-0.35341168335841,48.316283012913,0
-0.35345481707958,48.316373995578,0
-0.3535097108337,48.316462131969,0
-0.3535759468957,48.316546751298,0
-0.35365302120921,48.316627209541,0
-0.35374034722177,48.316702894341,0
-0.3538372603478,48.316773229671,0
-0.35394302302597,48.31683768021,0
-0.35405683033238,48.31689575543,0
-0.3541778161066,48.31694701332,0
-0.35430505954426,48.316991063755,0
-0.35443759220586,48.317027571467,0
-0.35457440538833,48.317056258592,0

-0.35471445780344,48.317076906792,0
-0.35485668350435,48.31708935891,0
-0.35500000000003,48.317093520174,0
-0.35514331649568,48.31708935891,0
-0.3552855421966,48.317076906792,0
-0.35542559461172,48.317056258592,0
-0.35556240779419,48.317027571467,0
-0.35569494045577,48.316991063755,0
-0.35582218389343,48.31694701332,0
-0.35594316966765,48.31689575543,0
-0.35605697697407,48.31683768021,0
-0.35616273965226,48.316773229671,0
-0.35625965277828,48.316702894341,0
-0.35634697879083,48.316627209541,0
-0.35642405310436,48.316546751298,0
-0.35649028916633,48.316462131969,0
-0.35654518292047,48.316373995578,0
-0.35658831664162,48.316283012913,0
-0.35661936211345,48.31618987642,0
-0.35663808312453,48.316095294933,0
-0.35664433726428,48.315999988281,0
-0.35663807700458,48.315904681807,0
-0.3566193500595,48.315810100849,0
-0.35658829901995,48.315716965219,0
-0.35654516026645,48.315625983726,0
-0.35649026216833,48.315537848779,0
-0.35642402258269,48.315453231124,0
-0.35634694567291,48.315372774733,0
-0.35625961807034,48.315297091905,0
-0.35616270440889,48.315226758611,0
-0.35605694226612,48.315162310106,0
-0.3559431365497,48.315104236859,0
-0.35582215337179,48.315052980821,0
-0.35569491345777,48.315008932058,0
-0.35556238514017,48.314972425791,0
-0.35542557699003,48.314943739838,0
-0.35528553014265,48.314923092502,0
-0.35514331037574,48.314910640912,0
-0.35500000000003,48.314906479826,0
-0.3548566896243,48.314910640912,0
-0.35471446985738,48.314923092502,0
-0.35457442301001,48.314943739838,0
-0.35443761485986,48.314972425791,0
-0.35430508654226,48.315008932058,0
-0.35417784662827,48.315052980821,0
-0.35405686345033,48.315104236859,0
-0.35394305773391,48.315162310106,0
-0.35383729559114,48.315226758611,0
-0.35374038192969,48.315297091905,0
-0.35365305432713,48.315372774733,0
-0.35357597741735,48.315453231124,0
-0.3535097378317,48.315537848779,0
-0.35345483973358,48.315625983726,0
-0.35341170098011,48.315716965219,0
-0.35338064994053,48.315810100849,0
-0.35336192299545,48.315904681807,0
-0.35335566273575,48.315999988281,0

-0.35500000000002,48.316,0
</coordinates>
</LinearRing>
</outerBoundaryIs>
</Polygon>
</Placemark>
<Placemark>
<name>565</name>
<styleUrl>#stylehap1</styleUrl>
<Polygon>
<outerBoundaryIs>
<LinearRing>
<coordinates>
-0.35500000000002,48.373,0
-0.35335382279696,48.372999988258,0
-0.35336008392866,48.37309529491,0
-0.35337882588176,48.373189876397,0
-0.35340990608647,48.373283012891,0
-0.35345308806726,48.373373995558,0
-0.35350804324067,48.37346213195,0
-0.35357435341424,48.37354675128,0
-0.3536515139678,48.373627209525,0
-0.35373893769261,48.373702894328,0
-0.35383595925949,48.373773229659,0
-0.35394184028185,48.373837680201,0
-0.35405577493508,48.373895755422,0
-0.3541768960895,48.373947013314,0
-0.35430428191032,48.373991063751,0
-0.35443696287432,48.374027571464,0
-0.35457392914958,48.374056258591,0
-0.35471413828257,48.374076906791,0
-0.35485652313356,48.37408935891,0
-0.35500000000003,48.374093520174,0
-0.3551434768665,48.37408935891,0
-0.35528586171746,48.374076906791,0
-0.35542607085045,48.374056258591,0
-0.35556303712571,48.374027571464,0
-0.35569571808971,48.373991063751,0
-0.35582310391053,48.373947013314,0
-0.35594422506495,48.373895755422,0
-0.35605815971818,48.373837680201,0
-0.35616404074054,48.373773229659,0
-0.35626106230742,48.373702894328,0
-0.35634848603223,48.373627209525,0
-0.35642564658579,48.37354675128,0
-0.35649195675939,48.37346213195,0
-0.35654691193277,48.373373995558,0
-0.35659009391357,48.373283012891,0
-0.35662117411827,48.373189876397,0
-0.35663991607137,48.37309529491,0
-0.35664617720308,48.372999988258,0
-0.35663990993229,48.372904681784,0
-0.35662116202664,48.372810100826,0
-0.35659007623678,48.372716965197,0
-0.35654688920796,48.372625983705,0
-0.35649192967699,48.37253784876,0
-0.35642561596873,48.372453231107,0

-0.35634845281075,48.372372774717,0
-0.35626102749096,48.372297091891,0
-0.35616400538699,48.372226758599,0
-0.35605812490172,48.372162310096,0
-0.35594419184347,48.372104236851,0
-0.35582307329347,48.372052980815,0
-0.35569569100731,48.372008932054,0
-0.35556301440091,48.371972425789,0
-0.35542605317369,48.371943739836,0
-0.35528584962583,48.371923092501,0
-0.35514347072742,48.371910640912,0
-0.35500000000003,48.371906479826,0
-0.35485652927264,48.371910640912,0
-0.3547141503742,48.371923092501,0
-0.35457394682634,48.371943739836,0
-0.35443698559913,48.371972425789,0
-0.35430430899272,48.372008932054,0
-0.35417692670657,48.372052980815,0
-0.35405580815656,48.372104236851,0
-0.35394187509831,48.372162310096,0
-0.35383599461306,48.372226758599,0
-0.35373897250907,48.372297091891,0
-0.35365154718928,48.372372774717,0
-0.35357438403133,48.372453231107,0
-0.35350807032304,48.37253784876,0
-0.35345311079207,48.372625983705,0
-0.35340992376325,48.372716965197,0
-0.35337883797339,48.372810100826,0
-0.35336009006774,48.372904681784,0
-0.35335382279696,48.372999988258,0
-0.35500000000002,48.373,0
</coordinates>
</LinearRing>
</outerBoundaryIs>
</Polygon>
</Placemark>
<Placemark>
<name>566</name>
<styleUrl>#stylehap1</styleUrl>
<Polygon>
<outerBoundaryIs>
<LinearRing>
<coordinates>
-0.34899999999993,48.38,0
-0.34735359644316,48.379999988255,0
-0.34735985843504,48.380095294907,0
-0.34737860296446,48.380189876394,0
-0.3474096874421,48.380283012889,0
-0.3474528753599,48.380373995555,0
-0.34750783808923,48.380462131947,0
-0.34757415738021,48.380546751278,0
-0.34765132854324,48.380627209523,0
-0.34773876428886,48.380702894326,0
-0.34783579919639,48.380773229657,0
-0.34794169477772,48.380837680199,0
-0.34805564509748,48.380895755421,0
-0.34817678290668,48.380947013313,0

-0.34830418624383,48.380991063751,0
-0.34843688545233,48.381027571464,0
-0.34857387056144,48.38105625859,0
-0.34871409897426,48.381076906791,0
-0.34885650340426,48.38108935891,0
-0.34899999999994,48.381093520174,0
-0.3491434965956,48.38108935891,0
-0.3492859010256,48.381076906791,0
-0.34942612943842,48.38105625859,0
-0.34956311454753,48.381027571464,0
-0.34969581375603,48.380991063751,0
-0.34982321709318,48.380947013313,0
-0.34994435490238,48.380895755421,0
-0.35005830522214,48.380837680199,0
-0.35016420080347,48.380773229657,0
-0.35026123571103,48.380702894326,0
-0.35034867145662,48.380627209523,0
-0.35042584261965,48.380546751278,0
-0.35049216191063,48.380462131947,0
-0.35054712463996,48.380373995555,0
-0.35059031255776,48.380283012889,0
-0.3506213970354,48.380189876394,0
-0.35064014156482,48.380095294907,0
-0.3506464035567,48.379999988255,0
-0.3506401354234,48.379904681781,0
-0.35062138493914,48.379810100824,0
-0.35059029487421,48.379716965195,0
-0.35054710190643,48.379625983702,0
-0.35049213481785,48.379537848758,0
-0.35042581199083,48.379453231104,0
-0.3503486382224,48.379372774715,0
-0.35026120088121,48.379297091889,0
-0.35016416543636,48.379226758597,0
-0.35005827039232,48.379162310095,0
-0.34994432166818,48.37910423685,0
-0.34982318646436,48.379052980814,0
-0.34969578666325,48.379008932054,0
-0.34956309181399,48.378972425788,0
-0.34942611175487,48.378943739836,0
-0.34928588892934,48.378923092501,0
-0.34914349045418,48.378910640912,0
-0.34899999999994,48.378906479826,0
-0.34885650954568,48.378910640912,0
-0.34871411107052,48.378923092501,0
-0.34857388824499,48.378943739836,0
-0.34843690818589,48.378972425788,0
-0.34830421333661,48.379008932054,0
-0.3481768135355,48.379052980814,0
-0.34805567833168,48.37910423685,0
-0.34794172960754,48.379162310095,0
-0.3478358345635,48.379226758597,0
-0.34773879911865,48.379297091889,0
-0.34765136177746,48.379372774715,0
-0.34757418800903,48.379453231104,0
-0.34750786518203,48.379537848758,0
-0.34745289809343,48.379625983702,0
-0.34740970512565,48.379716965195,0

-0.34737861506075,48.379810100824,0
-0.34735986457646,48.379904681781,0
-0.34735359644316,48.379999988255,0
-0.34899999999993,48.38,0
</coordinates>
</LinearRing>
</outerBoundaryIs>
</Polygon>
</Placemark>
<Placemark>
<name>567</name>
<styleUrl>#stylehap1</styleUrl>
<Polygon>
<outerBoundaryIs>
<LinearRing>
<coordinates>
-0.34799999999996,48.14,0
-0.34636130787501,48.139999988353,0
-0.34636754056261,48.140095295005,0
-0.34638619732122,48.14018987649,0
-0.34641713622859,48.140283012981,0
-0.34646012188374,48.140373995642,0
-0.34651482719664,48.140462132028,0
-0.34658083587582,48.140546751352,0
-0.34665764559511,48.140627209589,0
-0.34674467181549,48.140702894384,0
-0.34684125223278,48.140773229707,0
-0.3469466518177,48.14083768024,0
-0.34706006840979,48.140895755454,0
-0.34718063882234,48.140947013338,0
-0.34730744541245,48.140991063768,0
-0.34743952306563,48.141027571475,0
-0.34757586654221,48.141056258597,0
-0.34771543812925,48.141076906794,0
-0.3478571755399,48.141089358911,0
-0.34799999999996,48.141093520173,0
-0.34814282446001,48.141089358911,0
-0.34828456187067,48.141076906794,0
-0.34842413345771,48.141056258597,0
-0.34856047693429,48.141027571475,0
-0.34869255458746,48.140991063768,0
-0.34881936117757,48.140947013338,0
-0.34893993159012,48.140895755454,0
-0.34905334818222,48.14083768024,0
-0.34915874776714,48.140773229707,0
-0.34925532818442,48.140702894384,0
-0.34934235440478,48.140627209589,0
-0.3494191641241,48.140546751352,0
-0.34948517280328,48.140462132028,0
-0.34953987811617,48.140373995642,0
-0.34958286377133,48.140283012981,0
-0.3496138026787,48.14018987649,0
-0.34963245943731,48.140095295005,0
-0.3496386921249,48.139999988353,0
-0.34963245337596,48.139904681879,0
-0.34961379074017,48.139810100919,0
-0.34958284631838,48.139716965287,0

-0.34953985567909,48.139625983789,0
-0.34948514606382,48.139537848839,0
-0.34941913389471,48.139453231178,0
-0.34934232160397,48.139372774781,0
-0.34925529380884,48.139297091947,0
-0.34915871286127,48.139226758647,0
-0.3490533138066,48.139162310136,0
-0.34893989878932,48.139104236883,0
-0.34881933094818,48.139052980839,0
-0.34869252784801,48.139008932071,0
-0.34856045449723,48.1389724258,0
-0.34842411600476,48.138943739843,0
-0.34828454993214,48.138923092504,0
-0.34814281839866,48.138910640912,0
-0.34799999999996,48.138906479826,0
-0.34785718160125,48.138910640912,0
-0.34771545006775,48.138923092504,0
-0.34757588399516,48.138943739843,0
-0.34743954550269,48.1389724258,0
-0.34730747215191,48.139008932071,0
-0.34718066905174,48.139052980839,0
-0.3470601012106,48.139104236883,0
-0.34694668619329,48.139162310136,0
-0.34684128713865,48.139226758647,0
-0.34674470619108,48.139297091947,0
-0.34665767839592,48.139372774781,0
-0.34658086610521,48.139453231178,0
-0.34651485393609,48.139537848839,0
-0.34646014432082,48.139625983789,0
-0.34641715368154,48.139716965287,0
-0.34638620925972,48.139810100919,0
-0.34636754662393,48.139904681879,0
-0.34636130787501,48.139999988353,0
-0.34799999999996,48.14,0
</coordinates>
</LinearRing>
</outerBoundaryIs>
</Polygon>
</Placemark>
<Placemark>
<name>568</name>
<styleUrl>#stylehap1</styleUrl>
<Polygon>
<outerBoundaryIs>
<LinearRing>
<coordinates>
-0.346,48.39,0
-0.34435327293013,48.389999988251,0
-0.34435953615138,48.390095294903,0
-0.34437828436303,48.39018987639,0
-0.34440937494766,48.390283012885,0
-0.34445257135087,48.390373995551,0
-0.34450754487944,48.390462131944,0
-0.34457387720133,48.390546751275,0
-0.34465106352779,48.39062720952,0
-0.34473851645395,48.390702894324,0
-0.34483557042845,48.390773229655,0

-0.34494148681804,48.390837680198,0
-0.34505545952895,48.39089575542,0
-0.34517662114181,48.390947013312,0
-0.34530404951394,48.39099106375,0
-0.34543677479818,48.391027571463,0
-0.34557378682532,48.39105625859,0
-0.34571404279355,48.391076906791,0
-0.34585647520663,48.39108935891,0
-0.34600000000001,48.391093520174,0
-0.34614352479337,48.39108935891,0
-0.34628595720645,48.391076906791,0
-0.34642621317469,48.39105625859,0
-0.34656322520182,48.391027571463,0
-0.34669595048606,48.39099106375,0
-0.34682337885819,48.390947013312,0
-0.34694454047105,48.39089575542,0
-0.34705851318196,48.390837680198,0
-0.34716442957155,48.390773229655,0
-0.34726148354605,48.390702894324,0
-0.34734893647222,48.39062720952,0
-0.34742612279868,48.390546751275,0
-0.34749245512057,48.390462131944,0
-0.34754742864913,48.390373995551,0
-0.34759062505234,48.390283012885,0
-0.34762171563698,48.39018987639,0
-0.34764046384862,48.390095294903,0
-0.34764672706987,48.389999988251,0
-0.34764045770381,48.389904681777,0
-0.3476217035341,48.38981010082,0
-0.34759060735909,48.389716965191,0
-0.34754740590313,48.389625983699,0
-0.34749242801293,48.389537848754,0
-0.34742609215306,48.389453231101,0
-0.34734890321977,48.389372774712,0
-0.34726144869715,48.389297091887,0
-0.34716439418506,48.389226758595,0
-0.34705847833306,48.389162310093,0
-0.34694450721863,48.389104236849,0
-0.34682334821258,48.389052980813,0
-0.34669592337845,48.389008932053,0
-0.34656320245582,48.388972425788,0
-0.34642619548144,48.388943739836,0
-0.34628594510355,48.388923092501,0
-0.34614351864859,48.388910640912,0
-0.34600000000001,48.388906479827,0
-0.34585648135143,48.388910640912,0
-0.34571405489645,48.388923092501,0
-0.34557380451856,48.388943739836,0
-0.34543679754419,48.388972425788,0
-0.34530407662158,48.389008932053,0
-0.34517665178742,48.389052980813,0
-0.34505549278137,48.389104236849,0
-0.34494152166695,48.389162310093,0
-0.34483560581497,48.389226758595,0
-0.34473855130286,48.389297091887,0
-0.34465109678023,48.389372774712,0
-0.34457390784694,48.389453231101,0

-0.34450757198707,48.389537848754,0
-0.34445259409688,48.389625983699,0
-0.34440939264091,48.389716965191,0
-0.34437829646593,48.38981010082,0
-0.34435954229619,48.389904681777,0
-0.34435327293013,48.389999988251,0
-0.346,48.39,0
</coordinates>
</LinearRing>
</outerBoundaryIs>
</Polygon>
</Placemark>
<Placemark>
<name>571</name>
<styleUrl>#stylehap1</styleUrl>
<Polygon>
<outerBoundaryIs>
<LinearRing>
<coordinates>
-0.34299999999996,48.302,0
-0.34135611377276,48.301999988287,0
-0.3413623661985,48.302095294939,0
-0.34138108207594,48.302189876425,0
-0.34141211903344,48.302283012919,0
-0.34145524092438,48.302373995584,0
-0.34151011962239,48.302462131974,0
-0.34157633751688,48.302546751302,0
-0.34165339068977,48.302627209545,0
-0.34174069274943,48.302702894345,0
-0.34183757929258,48.302773229673,0
-0.34194331296025,48.302837680213,0
-0.34205708904924,48.302895755432,0
-0.34217804163676,48.302947013321,0
-0.34230525017104,48.302991063756,0
-0.34243774647824,48.303027571468,0
-0.342574522132,48.303056258593,0
-0.34271453612977,48.303076906792,0
-0.34285672281711,48.30308935891,0
-0.34299999999996,48.303093520174,0
-0.34314327718281,48.30308935891,0
-0.34328546387015,48.303076906792,0
-0.34342547786792,48.303056258593,0
-0.3435622535217,48.303027571468,0
-0.34369474982888,48.302991063756,0
-0.34382195836316,48.302947013321,0
-0.34394291095067,48.302895755432,0
-0.34405668703967,48.302837680213,0
-0.34416242070733,48.302773229673,0
-0.34425930725049,48.302702894345,0
-0.34434660931017,48.302627209545,0
-0.34442366248304,48.302546751302,0
-0.34448988037753,48.302462131974,0
-0.34454475907554,48.302373995584,0
-0.34458788096647,48.302283012919,0
-0.34461891792398,48.302189876425,0
-0.34463763380141,48.302095294939,0
-0.34464388622718,48.301999988287,0

-0.34463762768615,48.301904681813,0
-0.34461890587927,48.301810100855,0
-0.34458786335829,48.301716965225,0
-0.34454473643889,48.301625983731,0
-0.34448985340022,48.301537848784,0
-0.34442363198478,48.301453231129,0
-0.34434657621759,48.301372774736,0
-0.34425927256913,48.301297091908,0
-0.34416238549096,48.301226758614,0
-0.34405665235832,48.301162310108,0
-0.34394287785812,48.301104236861,0
-0.34382192786487,48.301052980822,0
-0.34369472285156,48.301008932059,0
-0.34356223088503,48.300972425792,0
-0.34342546025973,48.300943739838,0
-0.34328545182547,48.300923092502,0
-0.34314327106757,48.300910640912,0
-0.34299999999996,48.300906479826,0
-0.34285672893237,48.300910640912,0
-0.34271454817447,48.300923092502,0
-0.34257453974018,48.300943739838,0
-0.34243776911489,48.300972425792,0
-0.34230527714835,48.301008932059,0
-0.34217807213505,48.301052980822,0
-0.34205712214182,48.301104236861,0
-0.3419433476416,48.301162310108,0
-0.34183761450896,48.301226758614,0
-0.34174072743078,48.301297091908,0
-0.34165342378232,48.301372774736,0
-0.34157636801514,48.301453231129,0
-0.3415101465997,48.301537848784,0
-0.34145526356102,48.301625983731,0
-0.34141213664163,48.301716965225,0
-0.34138109412065,48.301810100855,0
-0.34136237231377,48.301904681813,0
-0.34135611377276,48.301999988287,0
-0.34299999999996,48.302,0
</coordinates>
</LinearRing>
</outerBoundaryIs>
</Polygon>
</Placemark>
<Placemark>
<name>572</name>
<styleUrl>#stylehap1</styleUrl>
<Polygon>
<outerBoundaryIs>
<LinearRing>
<coordinates>
-0.34199999999998,48.137,0
-0.34036140362919,48.136999988355,0
-0.34036763595291,48.137095295006,0
-0.34038629162165,48.137189876491,0
-0.34041722872145,48.137283012982,0
-0.34046021186506,48.137373995643,0
-0.3405149139816,48.137462132029,0
-0.34058091880386,48.137546751353,0

-0.34065772403504,48.13762720959,0
-0.34074474517027,48.137702894385,0
-0.34084131994405,48.137773229707,0
-0.34094671337013,48.137837680241,0
-0.34106012333483,48.137895755454,0
-0.34118068670192,48.137947013338,0
-0.3413074858821,48.137991063769,0
-0.34143955581734,48.138027571475,0
-0.34157589132666,48.138056258597,0
-0.34171545475779,48.138076906794,0
-0.34185718388595,48.138089358911,0
-0.34199999999997,48.138093520174,0
-0.34214281611402,48.138089358911,0
-0.34228454524219,48.138076906794,0
-0.34242410867332,48.138056258597,0
-0.34256044418263,48.138027571475,0
-0.34269251411785,48.137991063769,0
-0.34281931329805,48.137947013338,0
-0.34293987666514,48.137895755454,0
-0.34305328662985,48.137837680241,0
-0.34315868005592,48.137773229707,0
-0.3432552548297,48.137702894385,0
-0.34334227596494,48.13762720959,0
-0.34341908119611,48.137546751353,0
-0.34348508601837,48.137462132029,0
-0.34353978813489,48.137373995643,0
-0.34358277127853,48.137283012982,0
-0.34361370837832,48.137189876491,0
-0.34363236404707,48.137095295006,0
-0.34363859637078,48.136999988355,0
-0.34363235798671,48.13690468188,0
-0.34361369644175,48.13681010092,0
-0.34358275382843,48.136716965288,0
-0.34353976570149,48.136625983791,0
-0.34348505928329,48.13653784884,0
-0.34341905097168,48.136453231179,0
-0.3433422431695,48.136372774782,0
-0.34325522045974,48.136297091948,0
-0.34315864515574,48.136226758647,0
-0.34305325225988,48.136162310136,0
-0.3429398438697,48.136104236883,0
-0.34281928307362,48.136052980839,0
-0.34269248738277,48.136008932072,0
-0.34256042174923,48.1359724258,0
-0.34242409122322,48.135943739843,0
-0.34228453330562,48.135923092504,0
-0.34214281005367,48.135910640912,0
-0.34199999999997,48.135906479826,0
-0.34185718994631,48.135910640912,0
-0.34171546669436,48.135923092504,0
-0.34157590877673,48.135943739843,0
-0.34143957825074,48.1359724258,0
-0.34130751261718,48.136008932072,0
-0.34118071692635,48.136052980839,0
-0.34106015613027,48.136104236883,0
-0.34094674774009,48.136162310136,0
-0.34084135484423,48.136226758647,0

-0.34074477954023,48.136297091948,0
-0.34065775683047,48.136372774782,0
-0.34058094902829,48.136453231179,0
-0.34051494071668,48.13653784884,0
-0.34046023429848,48.136625983791,0
-0.34041724617155,48.136716965288,0
-0.34038630355822,48.13681010092,0
-0.34036764201326,48.13690468188,0
-0.34036140362919,48.136999988355,0
-0.34199999999998,48.137,0
</coordinates>
</LinearRing>
</outerBoundaryIs>
</Polygon>
</Placemark>
<Placemark>
<name>574</name>
<styleUrl>#stylehap1</styleUrl>
<Polygon>
<outerBoundaryIs>
<LinearRing>
<coordinates>
-0.33100000000002,48.133,0
-0.32936153127703,48.132999988356,0
-0.32936776311567,48.133095295008,0
-0.32938641733154,48.133189876493,0
-0.3294173520217,48.133283012983,0
-0.32946033181725,48.133373995645,0
-0.32951502967274,48.133462132031,0
-0.32958102935342,48.133546751354,0
-0.32965782860161,48.133627209591,0
-0.32974484295794,48.133702894386,0
-0.32984141020854,48.133773229708,0
-0.32994679542433,48.133837680241,0
-0.33006019655422,48.133895755455,0
-0.33018075052916,48.133947013339,0
-0.33030753983135,48.133991063769,0
-0.33043959947793,48.134027571476,0
-0.33057592436627,48.134056258597,0
-0.33071547692492,48.134076906794,0
-0.33085719501188,48.134089358911,0
-0.33100000000002,48.134093520174,0
-0.33114280498815,48.134089358911,0
-0.33128452307511,48.134076906794,0
-0.33142407563376,48.134056258597,0
-0.3315604005221,48.134027571476,0
-0.33169246016868,48.133991063769,0
-0.33181924947087,48.133947013339,0
-0.33193980344581,48.133895755455,0
-0.3320532045757,48.133837680241,0
-0.33215858979151,48.133773229708,0
-0.33225515704209,48.133702894386,0
-0.33234217139842,48.133627209591,0
-0.33241897064661,48.133546751354,0
-0.33248497032729,48.133462132031,0
-0.33253966818278,48.133373995645,0
-0.33258264797833,48.133283012983,0

-0.33261358266849,48.133189876493,0
-0.33263223688438,48.133095295008,0
-0.332638468723,48.132999988356,0
-0.33263223082535,48.132904681882,0
-0.33261357073454,48.132810100922,0
-0.33258263053205,48.132716965289,0
-0.33253964575427,48.132625983792,0
-0.33248494359806,48.132537848841,0
-0.33241894042877,48.132453231181,0
-0.33234213861015,48.132372774783,0
-0.33225512267963,48.132297091949,0
-0.33215855489895,48.132226758648,0
-0.33205317021324,48.132162310137,0
-0.33193977065755,48.132104236884,0
-0.33181921925303,48.132052980839,0
-0.33169243343943,48.132008932072,0
-0.33156037809359,48.1319724258,0
-0.33142405818747,48.131943739843,0
-0.33128451114116,48.131923092504,0
-0.33114279892912,48.131910640912,0
-0.33100000000002,48.131906479826,0
-0.33085720107091,48.131910640912,0
-0.3307154888589,48.131923092504,0
-0.33057594181256,48.131943739843,0
-0.33043962190644,48.1319724258,0
-0.3303075665606,48.132008932072,0
-0.330180780747,48.132052980839,0
-0.33006022934248,48.132104236884,0
-0.32994682978679,48.132162310137,0
-0.32984144510108,48.132226758648,0
-0.3297448773204,48.132297091949,0
-0.3296578613899,48.132372774783,0
-0.32958105957126,48.132453231181,0
-0.32951505640199,48.132537848841,0
-0.32946035424576,48.132625983792,0
-0.32941736946798,48.132716965289,0
-0.32938642926549,48.132810100922,0
-0.32936776917468,48.132904681882,0
-0.32936153127703,48.132999988356,0
-0.33100000000002,48.133,0
</coordinates>
</LinearRing>
</outerBoundaryIs>
</Polygon>
</Placemark>
<Placemark>
<name>577</name>
<styleUrl>#stylehap1</styleUrl>
<Polygon>
<outerBoundaryIs>
<LinearRing>
<coordinates>
-0.32500000000005,48.265,0
-0.32335730413726,48.264999988302,0
-0.32336355203951,48.265095294954,0
-0.32338225436832,48.26518987644,0
-0.32341326885508,48.265283012933,0

-0.32345635952403,48.265373995597,0
-0.3235111984863,48.265462131986,0
-0.32357736843362,48.265546751313,0
-0.32365436581274,48.265627209555,0
-0.32374160465664,48.265702894354,0
-0.32383842104304,48.265773229681,0
-0.32394407814689,48.265837680219,0
-0.32405777184777,48.265895755437,0
-0.32417863684991,48.265947013325,0
-0.32430575326815,48.265991063759,0
-0.32443815362982,48.266027571469,0
-0.32457483023886,48.266056258594,0
-0.32471474284663,48.266076906792,0
-0.32485682657046,48.26608935891,0
-0.32500000000006,48.266093520174,0
-0.32514317342963,48.26608935891,0
-0.32528525715346,48.266076906792,0
-0.32542516976122,48.266056258594,0
-0.32556184637027,48.266027571469,0
-0.32569424673194,48.265991063759,0
-0.32582136315018,48.265947013325,0
-0.32594222815232,48.265895755437,0
-0.32605592185319,48.265837680219,0
-0.32616157895705,48.265773229681,0
-0.32625839534345,48.265702894354,0
-0.32634563418734,48.265627209555,0
-0.32642263156646,48.265546751313,0
-0.32648880151379,48.265462131986,0
-0.32654364047606,48.265373995597,0
-0.32658673114501,48.265283012933,0
-0.32661774563177,48.26518987644,0
-0.32663644796058,48.265095294954,0
-0.32664269586283,48.264999988302,0
-0.32663644185771,48.264904681828,0
-0.32661773361141,48.26481010087,0
-0.32658671357245,48.264716965239,0
-0.32654361788521,48.264625983744,0
-0.32648877459105,48.264537848797,0
-0.32642260112988,48.26445323114,0
-0.32634560116171,48.264372774747,0
-0.32625836073224,48.264297091917,0
-0.32616154381189,48.264226758621,0
-0.32605588724197,48.264162310114,0
-0.32594219512669,48.264104236866,0
-0.3258213327136,48.264052980826,0
-0.32569421980918,48.264008932062,0
-0.3255618237794,48.263972425794,0
-0.32542515218866,48.263943739839,0
-0.3252852451331,48.263923092502,0
-0.32514316732673,48.263910640912,0
-0.32500000000006,48.263906479826,0
-0.32485683267336,48.263910640912,0
-0.32471475486699,48.263923092502,0
-0.32457484781143,48.263943739839,0
-0.32443817622069,48.263972425794,0
-0.32430578019091,48.264008932062,0
-0.32417866728649,48.264052980826,0

-0.32405780487342,48.264104236866,0
-0.32394411275812,48.264162310114,0
-0.3238384561882,48.264226758621,0
-0.32374163926784,48.264297091917,0
-0.3236543988384,48.264372774747,0
-0.32357739887021,48.26445323114,0
-0.32351122540904,48.264537848797,0
-0.3234563821149,48.264625983744,0
-0.32341328642764,48.264716965239,0
-0.32338226638867,48.26481010087,0
-0.32336355814241,48.264904681828,0
-0.32335730413726,48.264999988302,0
-0.32500000000005,48.265,0
</coordinates>
</LinearRing>
</outerBoundaryIs>
</Polygon>
</Placemark>
<Placemark>
<name>578</name>
<styleUrl>#stylehap1</styleUrl>
<Polygon>
<outerBoundaryIs>
<LinearRing>
<coordinates>
-0.322,48.446,0
-0.32035145797592,48.445999988228,0
-0.32035772809417,48.44609529488,0
-0.32037649696344,48.446189876368,0
-0.32040762180932,48.446283012863,0
-0.32045086581681,48.446373995531,0
-0.32050589993064,48.446462131925,0
-0.32057230535776,48.446546751257,0
-0.32064957675321,48.446627209505,0
-0.32073712606479,48.44670289431,0
-0.32083428700763,48.446773229644,0
-0.32094032013439,48.446837680188,0
-0.32105441846283,48.446895755412,0
-0.32117571361759,48.446947013307,0
-0.32130328243946,48.446991063746,0
-0.32143615401231,48.447027571461,0
-0.32157331705341,48.447056258589,0
-0.32171372761146,48.44707690679,0
-0.32185631701348,48.44708935891,0
-0.322,48.447093520173,0
-0.32214368298652,48.44708935891,0
-0.32228627238854,48.44707690679,0
-0.32242668294662,48.447056258589,0
-0.32256384598771,48.447027571461,0
-0.32269671756054,48.446991063746,0
-0.32282428638241,48.446947013307,0
-0.32294558153717,48.446895755412,0
-0.32305967986561,48.446837680188,0
-0.3231657129924,48.446773229644,0
-0.32326287393521,48.44670289431,0
-0.32335042324679,48.446627209505,0
-0.32342769464225,48.446546751257,0

-0.32349410006936,48.446462131925,0
-0.32354913418319,48.446373995531,0
-0.32359237819068,48.446283012863,0
-0.32362350303658,48.446189876368,0
-0.32364227190583,48.44609529488,0
-0.32364854202408,48.445999988228,0
-0.32364226574214,48.445904681754,0
-0.32362349089646,48.445810100797,0
-0.32359236044304,48.445716965169,0
-0.32354911136724,48.445625983678,0
-0.32349407287839,48.445537848735,0
-0.32342766390241,48.445453231084,0
-0.32335038989211,48.445372774696,0
-0.32326283897916,48.445297091873,0
-0.32316567749708,48.445226758584,0
-0.32305964490956,48.445162310084,0
-0.32294554818246,48.445104236841,0
-0.32282425564258,48.445052980807,0
-0.32269669036955,48.445008932049,0
-0.32256382317176,48.444972425785,0
-0.32242666519895,48.444943739834,0
-0.32228626024844,48.4449230925,0
-0.32214367682283,48.444910640911,0
-0.322,48.444906479826,0
-0.3218563231772,48.444910640911,0
-0.32171373975156,48.4449230925,0
-0.32157333480105,48.444943739834,0
-0.32143617682826,48.444972425785,0
-0.32130330963045,48.445008932049,0
-0.32117574435742,48.445052980807,0
-0.32105445181754,48.445104236841,0
-0.32094035509044,48.445162310084,0
-0.32083432250292,48.445226758584,0
-0.32073716102087,48.445297091873,0
-0.32064961010789,48.445372774696,0
-0.32057233609761,48.445453231084,0
-0.32050592712163,48.445537848735,0
-0.32045088863276,48.445625983678,0
-0.32040763955696,48.445716965169,0
-0.32037650910354,48.445810100797,0
-0.32035773425788,48.445904681754,0
-0.32035145797592,48.445999988228,0
-0.322,48.446,0
</coordinates>
</LinearRing>
</outerBoundaryIs>
</Polygon>
</Placemark>
<Placemark>
<name>579</name>
<styleUrl>#stylehap1</styleUrl>
<Polygon>
<outerBoundaryIs>
<LinearRing>
<coordinates>
-0.32100000000003,48.397,0
-0.31935304636548,48.396999988248,0

-0.3193593104477,48.3970952949,0
-0.31937806123806,48.397189876387,0
-0.31940915609962,48.397283012882,0
-0.31945235844536,48.397373995549,0
-0.31950733953692,48.397462131942,0
-0.31957368098472,48.397546751272,0
-0.31965087793051,48.397627209518,0
-0.31973834288869,48.397702894322,0
-0.31983541021628,48.397773229654,0
-0.31994134117841,48.397837680197,0
-0.32005532957042,48.397895755419,0
-0.32017650785361,48.397947013312,0
-0.3203039537583 9,48.397991063749,0
-0.32043669730415,48.398027571463,0
-0.32057372818268,48.39805625859,0
-0.32071400344872,48.398076906791,0
-0.32085645545905,48.39808935891,0
-0.32100000000002,48.398093520174,0
-0.32114354454101,48.39808935891,0
-0.32128599655134,48.398076906791,0
-0.32142627181737,48.39805625859,0
-0.32156330269591,48.398027571463,0
-0.32169604624167,48.397991063749,0
-0.32182349214645,48.397947013312,0
-0.32194467042964,48.397895755419,0
-0.32205865882165,48.397837680197,0
-0.32216458978378,48.397773229654,0
-0.32226165711136,48.397702894322,0
-0.32234912206954,48.397627209518,0
-0.32242631901534,48.397546751272,0
-0.32249266046311,48.397462131942,0
-0.3225476415547,48.397373995549,0
-0.32259084390043,48.397283012882,0
-0.32262193876197,48.397189876387,0
-0.32264068955236,48.3970952949,0
-0.32264695363457,48.396999988248,0
-0.32264068340519,48.396904681774,0
-0.32262192665444,48.396810100817,0
-0.32259082620039,48.396716965188,0
-0.32254761879994,48.396625983696,0
-0.32249263334509,48.396537848752,0
-0.32242628835797,48.396453231099,0
-0.32234908880435,48.39637277471,0
-0.3222616222491,48.396297091885,0
-0.3221645543837,48.396226758594,0
-0.32205862395936,48.396162310092,0
-0.32194463716445,48.396104236848,0
-0.32182346148906,48.396052980812,0
-0.32169601912363,48.396008932053,0
-0.32156327994115,48.395972425788,0
-0.32142625411733,48.395943739836,0
-0.32128598444381,48.395923092501,0
-0.32114353839383,48.395910640912,0
-0.32100000000002,48.395906479826,0
-0.32085646160622,48.395910640912,0
-0.32071401555625,48.395923092501,0
-0.32057374588272,48.395943739836,0

-0.32043672005888,48.395972425788,0
-0.32030398087643,48.396008932053,0
-0.32017653851097,48.396052980812,0
-0.32005536283561,48.396104236848,0
-0.3199413760407,48.396162310092,0
-0.31983544561636,48.396226758594,0
-0.31973837775096,48.396297091885,0
-0.31965091119571,48.39637277471,0
-0.31957371164208,48.396453231099,0
-0.31950736665497,48.396537848752,0
-0.31945238120009,48.396625983696,0
-0.31940917379966,48.396716965188,0
-0.31937807334562,48.396810100817,0
-0.31935931659487,48.396904681774,0
-0.31935304636548,48.396999988248,0
-0.32100000000003,48.397,0
</coordinates>
</LinearRing>
</outerBoundaryIs>
</Polygon>
</Placemark>
<Placemark>
<name>580</name>
<styleUrl>#stylehap1</styleUrl>
<Polygon>
<outerBoundaryIs>
<LinearRing>
<coordinates>
-0.31799999999998,48.459,0
-0.31635103584954,48.458999988222,0
-0.3163573075719,48.459095294875,0
-0.31637608124574,48.459189876363,0
-0.31640721406018,48.459283012858,0
-0.31645046913962,48.459373995526,0
-0.3165055173445,48.459462131921,0
-0.31657193977461,48.459546751253,0
-0.31664923095561,48.459627209501,0
-0.31673680268473,48.459702894307,0
-0.3168339885065,48.459773229641,0
-0.31694004878428,48.459837680186,0
-0.31705417632917,48.459895755411,0
-0.31717550254342,48.459947013305,0
-0.31730310403142,48.459991063745,0
-0.31743600962842,48.46002757146,0
-0.31757320779269,48.460056258588,0
-0.31771365430561,48.46007690679,0
-0.31785628022053,48.46008935891,0
-0.31799999999999,48.460093520174,0
-0.31814371977944,48.46008935891,0
-0.31828634569433,48.46007690679,0
-0.31842679220726,48.460056258588,0
-0.31856399037155,48.46002757146,0
-0.31869689596855,48.459991063745,0
-0.31882449745655,48.459947013305,0
-0.3189458236708,48.459895755411,0
-0.31905995121569,48.459837680186,0
-0.31916601149345,48.459773229641,0

-0.31926319731524,48.459702894307,0
-0.31935076904436,48.459627209501,0
-0.31942806022536,48.459546751253,0
-0.31949448265547,48.459462131921,0
-0.31954953086035,48.459373995526,0
-0.31959278593976,48.459283012858,0
-0.31962391875423,48.459189876363,0
-0.31964269242807,48.459095294875,0
-0.31964896415043,48.458999988222,0
-0.31964268625998,48.458904681749,0
-0.31962390660545,48.458810100792,0
-0.31959276817944,48.458716965164,0
-0.31954950802812,48.458625983674,0
-0.31949445544509,48.458537848731,0
-0.3194280294636,48.45845323108,0
-0.31935073566587,48.458372774693,0
-0.31926316233423,48.45829709187,0
-0.31916597597281,48.458226758581,0
-0.31905991623468,48.458162310081,0
-0.3189457902923,48.45810423684,0
-0.31882446669478,48.458052980806,0
-0.31869686875814,48.458008932048,0
-0.31856396753931,48.457972425785,0
-0.31842677444694,48.457943739834,0
-0.31828633354555,48.4579230925,0
-0.31814371361135,48.457910640911,0
-0.31799999999999,48.457906479826,0
-0.31785628638862,48.457910640911,0
-0.31771366645439,48.4579230925,0
-0.31757322555303,48.457943739834,0
-0.31743603246066,48.457972425785,0
-0.31730313124183,48.458008932048,0
-0.31717553330519,48.458052980806,0
-0.31705420970767,48.45810423684,0
-0.31694008376529,48.458162310081,0
-0.31683402402716,48.458226758581,0
-0.31673683766574,48.45829709187,0
-0.31664926433408,48.458372774693,0
-0.31657197053637,48.45845323108,0
-0.31650554455488,48.458537848731,0
-0.31645049197183,48.458625983674,0
-0.3164072318205,48.458716965164,0
-0.31637609339452,48.458810100792,0
-0.31635731373999,48.458904681749,0
-0.31635103584954,48.458999988222,0
-0.31799999999998,48.459,0
</coordinates>
</LinearRing>
</outerBoundaryIs>
</Polygon>
</Placemark>
<Placemark>
<name>581</name>
<styleUrl>#stylehap1</styleUrl>
<Polygon>
<outerBoundaryIs>
<LinearRing>

<coordinates>
-0.31799999999998,48.378,0
-0.31635366112456,48.377999988256,0
-0.31635992287065,48.378095294908,0
-0.31637866666389,48.378189876395,0
-0.3164097499205,48.378283012889,0
-0.31645293614179,48.378373995556,0
-0.31650789671198,48.378462131948,0
-0.31657421339763,48.378546751278,0
-0.31665138152898,48.378627209523,0
-0.3167388138396,48.378702894326,0
-0.316835844935,48.378773229658,0
-0.31694173635606,48.3788376802,0
-0.31705568219905,48.378895755422,0
-0.3171768152491,48.378947013314,0
-0.31730421358089,48.378991063751,0
-0.31743690757595,48.379027571464,0
-0.31757388730323,48.37905625859,0
-0.31771411020678,48.379076906791,0
-0.317856509042,48.37908935891,0
-0.31799999999999,48.379093520174,0
-0.31814349095797,48.37908935891,0
-0.31828588979319,48.379076906791,0
-0.31842611269674,48.37905625859,0
-0.31856309242402,48.379027571464,0
-0.31869578641908,48.378991063751,0
-0.31882318475087,48.378947013314,0
-0.31894431780092,48.378895755422,0
-0.31905826364391,48.3788376802,0
-0.31916415506497,48.378773229658,0
-0.31926118616037,48.378702894326,0
-0.31934861847099,48.378627209523,0
-0.31942578660234,48.378546751278,0
-0.31949210328799,48.378462131948,0
-0.31954706385818,48.378373995556,0
-0.31959025007947,48.378283012889,0
-0.31962133333608,48.378189876395,0
-0.31964007712932,48.378095294908,0
-0.31964633887541,48.377999988256,0
-0.31964007098856,48.377904681782,0
-0.31962132124115,48.377810100824,0
-0.31959023239785,48.377716965195,0
-0.31954704112714,48.377625983703,0
-0.31949207619816,48.377537848758,0
-0.31942575597685,48.377453231105,0
-0.3193485852404,48.377372774715,0
-0.31926115133436,48.37729709189,0
-0.31916411970174,48.377226758598,0
-0.3190582288179,48.377162310095,0
-0.31894428457036,48.377104236851,0
-0.31882315412541,48.377052980814,0
-0.31869575932927,48.377008932054,0
-0.31856306969295,48.376972425788,0
-0.31842609501512,48.376943739836,0
-0.31828587769825,48.376923092501,0
-0.31814348481721,48.376910640912,0
-0.31799999999999,48.376906479826,0

-0.31785651518276,48.376910640912,0
-0.31771412230172,48.376923092501,0
-0.31757390498485,48.376943739836,0
-0.31743693030702,48.376972425788,0
-0.3173042406707,48.377008932054,0
-0.31717684587456,48.377052980814,0
-0.31705571542961,48.377104236851,0
-0.31694177118207,48.377162310095,0
-0.31683588029824,48.377226758598,0
-0.31673884866558,48.37729709189,0
-0.31665141475954,48.377372774715,0
-0.31657424402309,48.377453231105,0
-0.3165079238018,48.377537848758,0
-0.31645295887283,48.377625983703,0
-0.31640976760212,48.377716965195,0
-0.31637867875883,48.377810100824,0
-0.31635992901141,48.377904681782,0
-0.31635366112456,48.377999988256,0
-0.31799999999998,48.378,0
</coordinates>
</LinearRing>
</outerBoundaryIs>
</Polygon>
</Placemark>
<Placemark>
<name>582</name>
<styleUrl>#stylehap1</styleUrl>
<Polygon>
<outerBoundaryIs>
<LinearRing>
<coordinates>
-0.31700000000001,48.184,0
-0.31535990167715,48.183999988335,0
-0.31536613970846,48.184095294987,0
-0.31538481247231,48.184189876472,0
-0.31541577792476,48.184283012964,0
-0.31545880046301,48.184373995626,0
-0.31551355271641,48.184462132014,0
-0.31557961803641,48.184546751338,0
-0.31565649366583,48.184627209577,0
-0.31574359456411,48.184702894373,0
-0.31584025785876,48.184773229698,0
-0.3159457478898,48.184837680233,0
-0.3160592618084,48.184895755448,0
-0.31617993568709,48.184947013334,0
-0.31630685109542,48.184991063765,0
-0.31643904209067,48.185027571473,0
-0.31657550257046,48.185056258596,0
-0.31671519393124,48.185076906793,0
-0.31685705297442,48.18508935891,0
-0.317,48.185093520174,0
-0.31714294702558,48.18508935891,0
-0.31728480606879,48.185076906793,0
-0.31742449742956,48.185056258596,0
-0.31756095790933,48.185027571473,0
-0.31769314890461,48.184991063765,0
-0.31782006431294,48.184947013334,0

-0.3179407381916,48.184895755448,0
-0.3180542521102,48.184837680233,0
-0.31815974214127,48.184773229698,0
-0.31825640543592,48.184702894373,0
-0.3183435063342,48.184627209577,0
-0.31842038196362,48.184546751338,0
-0.31848644728359,48.184462132014,0
-0.31854119953702,48.184373995626,0
-0.31858422207524,48.184283012964,0
-0.31861518752772,48.184189876472,0
-0.31863386029154,48.184095294987,0
-0.31864009832285,48.183999988335,0
-0.31863385421561,48.183904681861,0
-0.31861517556046,48.183810100902,0
-0.3185842045803,48.18371696527,0
-0.31854117704595,48.183625983774,0
-0.31848642047981,48.183537848824,0
-0.31842035166151,48.183453231165,0
-0.31834347345446,48.183372774769,0
-0.31825637097761,48.183297091937,0
-0.3181597071514,48.183226758638,0
-0.31805421765189,48.183162310128,0
-0.3179407053119,48.183104236877,0
-0.31782003401083,48.183052980834,0
-0.3176931221008,48.183008932068,0
-0.31756093541828,48.182972425798,0
-0.31742447993463,48.182943739841,0
-0.31728479410153,48.182923092503,0
-0.31714294094965,48.182910640912,0
-0.317,48.182906479826,0
-0.31685705905035,48.182910640912,0
-0.31671520589847,48.182923092503,0
-0.3165755200654,48.182943739841,0
-0.31643906458172,48.182972425798,0
-0.3163068778992,48.183008932068,0
-0.3161799659892,48.183052980834,0
-0.31605929468813,48.183104236877,0
-0.31594578234811,48.183162310128,0
-0.31584029284862,48.183226758638,0
-0.31574362902239,48.183297091937,0
-0.31565652654554,48.183372774769,0
-0.31557964833852,48.183453231165,0
-0.31551357952022,48.183537848824,0
-0.31545882295406,48.183625983774,0
-0.3154157954197,48.18371696527,0
-0.31538482443954,48.183810100902,0
-0.31536614578439,48.183904681861,0
-0.31535990167715,48.183999988335,0
-0.31700000000001,48.184,0
</coordinates>
</LinearRing>
</outerBoundaryIs>
</Polygon>
</Placemark>
<Placemark>
<name>584</name>
<styleUrl>#stylehap1</styleUrl>

<Polygon>
<outerBoundaryIs>
<LinearRing>
<coordinates>
-0.31700000000001,48.463,0
-0.31535090590392,48.46299998822,0
-0.31535717812011,48.463095294873,0
-0.31537595327299,48.463189876361,0
-0.31540708854045,48.463283012857,0
-0.31545034702819,48.463373995525,0
-0.31550539957081,48.463462131919,0
-0.31557182723505,48.463546751252,0
-0.31564912450676,48.4636272095,0
-0.31573670313679,48.463702894306,0
-0.31583389661721,48.46377322964,0
-0.31593996525303,48.463837680185,0
-0.3160541017918,48.46389575541,0
-0.31617543756725,48.463947013305,0
-0.31630304911106,48.463991063745,0
-0.31643596518192,48.46402757146,0
-0.31657317415836,48.464056258588,0
-0.31671363173949,48.46407690679,0
-0.31685626889436,48.46408935891,0
-0.317,48.464093520174,0
-0.31714373110564,48.46408935891,0
-0.31728636826051,48.46407690679,0
-0.31742682584164,48.464056258588,0
-0.31756403481808,48.46402757146,0
-0.31769695088894,48.463991063745,0
-0.31782456243278,48.463947013305,0
-0.31794589820821,48.46389575541,0
-0.31806003474697,48.463837680185,0
-0.31816610338282,48.46377322964,0
-0.31826329686321,48.463702894306,0
-0.31835087549327,48.4636272095,0
-0.31842817276496,48.463546751252,0
-0.31849460042921,48.463462131919,0
-0.31854965297181,48.463373995525,0
-0.31859291145958,48.463283012857,0
-0.31862404672701,48.463189876361,0
-0.31864282187991,48.463095294873,0
-0.31864909409608,48.46299998822,0
-0.31864281571045,48.462904681747,0
-0.31862403457559,48.46281010079,0
-0.31859289369537,48.462716965163,0
-0.31854963013459,48.462625983672,0
-0.31849457321285,48.46253784873,0
-0.31842814199645,48.462453231079,0
-0.31835084210747,48.462372774692,0
-0.31826326187454,48.462297091869,0
-0.31816606785437,48.46222675858,0
-0.3180599997583,48.462162310081,0
-0.31794586482241,48.462104236839,0
-0.31782453166424,48.462052980805,0
-0.31769692367257,48.462008932048,0
-0.31756401198086,48.461972425784,0
-0.31742680807743,48.461943739834,0

-0.31728635610909,48.4619230925,0
-0.3171437249362,48.461910640911,0
-0.317,48.461906479827,0
-0.31685627506383,48.461910640911,0
-0.31671364389094,48.4619230925,0
-0.3165731919226,48.461943739834,0
-0.31643598801917,48.461972425784,0
-0.31630307632743,48.462008932048,0
-0.31617546833578,48.462052980805,0
-0.31605413517762,48.462104236839,0
-0.31594000024173,48.462162310081,0
-0.31583393214564,48.46222675858,0
-0.31573673812549,48.462297091869,0
-0.31564915789256,48.462372774692,0
-0.31557185800358,48.462453231079,0
-0.31550542678715,48.46253784873,0
-0.31545036986544,48.462625983672,0
-0.31540710630466,48.462716965163,0
-0.31537596542444,48.46281010079,0
-0.31535718428955,48.462904681747,0
-0.31535090590392,48.46299998822,0
-0.31700000000001,48.463,0
</coordinates>
</LinearRing>
</outerBoundaryIs>
</Polygon>
</Placemark>
<Placemark>
<name>587</name>
<styleUrl>#stylehap1</styleUrl>
<Polygon>
<outerBoundaryIs>
<LinearRing>
<coordinates>
-0.312999999999999,48.447,0
-0.31135142551535,48.446999988227,0
-0.31135769575695,48.447095294879,0
-0.31137646499565,48.447189876367,0
-0.3114075904543,48.447283012863,0
-0.31145083531321,48.447373995531,0
-0.31150587051059,48.447462131925,0
-0.31157227724522,48.447546751257,0
-0.31164955016213,48.447627209504,0
-0.31173710119757,48.44770289431,0
-0.31183426405352,48.447773229643,0
-0.31194029926815,48.447837680188,0
-0.31205439984329,48.447895755412,0
-0.31217569738644,48.447947013307,0
-0.31230326872026,48.447991063746,0
-0.31243614290949,48.448027571461,0
-0.31257330865149,48.448056258589,0
-0.31271372197438,48.44807690679,0
-0.31285631418417,48.44808935891,0
-0.312999999999999,48.448093520174,0
-0.3131436858158,48.44808935891,0
-0.31328627802559,48.44807690679,0
-0.31342669134849,48.448056258589,0

-0.31356385709048,48.448027571461,0
-0.31369673127971,48.447991063746,0
-0.31382430261353,48.447947013307,0
-0.31394560015668,48.447895755412,0
-0.31405970073182,48.447837680188,0
-0.31416573594645,48.447773229643,0
-0.3142628988024,48.44770289431,0
-0.31435044983784,48.447627209504,0
-0.31442772275477,48.447546751257,0
-0.31449412948938,48.447462131925,0
-0.31454916468676,48.447373995531,0
-0.31459240954567,48.447283012863,0
-0.31462353500433,48.447189876367,0
-0.31464230424305,48.447095294879,0
-0.31464857448465,48.446999988227,0
-0.314642298079,48.446904681754,0
-0.31462352286354,48.446810100797,0
-0.31459239179703,48.446716965169,0
-0.31454914186956,48.446625983678,0
-0.31449410229688,48.446537848735,0
-0.31442769201324,48.446453231084,0
-0.31435041648133,48.446372774696,0
-0.31426286384442,48.446297091873,0
-0.31416570044917,48.446226758584,0
-0.31405966577383,48.446162310083,0
-0.3139455668017,48.446104236841,0
-0.31382427187199,48.446052980807,0
-0.31369670408722,48.446008932049,0
-0.31356383427328,48.445972425785,0
-0.31342667359987,48.445943739834,0
-0.3132862658848,48.4459230925,0
-0.31314367965175,48.445910640911,0
-0.31299999999999,48.445906479826,0
-0.31285632034822,48.445910640911,0
-0.31271373411517,48.4459230925,0
-0.3125733264001,48.445943739834,0
-0.31243616572669,48.445972425785,0
-0.31230329591275,48.446008932049,0
-0.31217572812798,48.446052980807,0
-0.3120544331998,48.446104236841,0
-0.31194033422614,48.446162310083,0
-0.3118342995508,48.446226758584,0
-0.31173713615556,48.446297091873,0
-0.31164958351865,48.446372774696,0
-0.31157230798676,48.446453231084,0
-0.31150589770309,48.446537848735,0
-0.31145085813041,48.446625983678,0
-0.31140760820294,48.446716965169,0
-0.31137647713643,48.446810100797,0
-0.31135770192097,48.446904681754,0
-0.31135142551535,48.446999988227,0
-0.31299999999999,48.447,0
</coordinates>
</LinearRing>
</outerBoundaryIs>
</Polygon>
</Placemark>

<Placemark>
<name>588</name>
<styleUrl>#stylehap1</styleUrl>
<Polygon>
<outerBoundaryIs>
<LinearRing>
<coordinates>
-0.30600000000004,48.471,0
-0.30435064592715,48.470999988217,0
-0.30435691913128,48.47109529487,0
-0.30437569724319,48.471189876358,0
-0.30440683741827,48.471283012853,0
-0.30445010272491,48.471373995522,0
-0.30450516394585,48.471462131916,0
-0.30457160208182,48.47154675125,0
-0.30464891153892,48.471627209498,0
-0.30473650397536,48.471702894304,0
-0.30483371277809,48.471773229639,0
-0.30493979813554,48.471837680184,0
-0.30505395266794,48.471895755409,0
-0.30517530757212,48.471947013304,0
-0.30530293923419,48.471991063744,0
-0.30543587625963,48.472027571459,0
-0.30557310686756,48.472056258588,0
-0.30571358659237,48.47207690679,0
-0.30585624623458,48.47208935891,0
-0.30600000000004,48.472093520174,0
-0.3061437537655,48.47208935891,0
-0.30628641340771,48.47207690679,0
-0.30642689313252,48.472056258588,0
-0.30656412374045,48.472027571459,0
-0.3066970607659,48.471991063744,0
-0.30682469242797,48.471947013304,0
-0.30694604733214,48.471895755409,0
-0.30706020186455,48.471837680184,0
-0.307166287222,48.471773229639,0
-0.3072634960247,48.471702894304,0
-0.30735108846116,48.471627209498,0
-0.30742839791827,48.47154675125,0
-0.30749483605421,48.471462131916,0
-0.30754989727515,48.471373995522,0
-0.30759316258182,48.471283012853,0
-0.3076243027569,48.471189876358,0
-0.30764308086881,48.47109529487,0
-0.30764935407293,48.470999988217,0
-0.30764307469665,48.470904681744,0
-0.30762429060013,48.470810100787,0
-0.30759314480979,48.470716965159,0
-0.3075498744279,48.470625983669,0
-0.30749480882592,48.470537848727,0
-0.30742836713622,48.470453231076,0
-0.30735105506068,48.47037277469,0
-0.30726346102066,48.470297091867,0
-0.30716625167798,48.470226758579,0
-0.30706016686051,48.470162310079,0
-0.30694601393169,48.470104236838,0
-0.30682466164592,48.470052980805,0

-0.30669703353757,48.470008932047,0
-0.30656410089318,48.469972425784,0
-0.3064268753605,48.469943739834,0
-0.30628640125094,48.4699230925,0
-0.30614374759334,48.469910640911,0
-0.30600000000004,48.469906479826,0
-0.30585625240674,48.469910640911,0
-0.30571359874914,48.4699230925,0
-0.30557312463958,48.469943739834,0
-0.3054358991069,48.469972425784,0
-0.30530296646248,48.470008932047,0
-0.30517533835416,48.470052980805,0
-0.3050539860684,48.470104236838,0
-0.30493983313958,48.470162310079,0
-0.30483374832211,48.470226758579,0
-0.3047365389794,48.470297091867,0
-0.3046489449394,48.47037277469,0
-0.30457163286383,48.470453231076,0
-0.30450519117417,48.470537848727,0
-0.30445012557219,48.470625983669,0
-0.30440685519029,48.470716965159,0
-0.30437570939995,48.470810100787,0
-0.30435692530344,48.470904681744,0
-0.30435064592715,48.470999988217,0
-0.30600000000004,48.471,0
</coordinates>
</LinearRing>
</outerBoundaryIs>
</Polygon>
</Placemark>
<Placemark>
<name>589</name>
<styleUrl>#stylehap1</styleUrl>
<Polygon>
<outerBoundaryIs>
<LinearRing>
<coordinates>
-0.28999999999996,48.433,0
-0.28835187980137,48.432999988233,0
-0.28835814831665,48.433095294885,0
-0.28837691238473,48.433189876373,0
-0.28840802926774,48.433283012868,0
-0.2884512622112,48.433373995536,0
-0.288506282244,48.433462131929,0
-0.28857267068027,48.433546751261,0
-0.28864992230428,48.433627209508,0
-0.28873744921427,48.433702894313,0
-0.28883458529588,48.433773229646,0
-0.28894059129101,48.43383768019,0
-0.28905466042384,48.433895755414,0
-0.28917592454124,48.433947013308,0
-0.28930346072027,48.433991063747,0
-0.28943629829322,48.434027571461,0
-0.28957342623616,48.434056258589,0
-0.28971380086496,48.43407690679,0
-0.28985635378017,48.43408935891,0
-0.28999999999996,48.434093520174,0

-0.29014364621977,48.43408935891,0
-0.29028619913495,48.43407690679,0
-0.29042657376378,48.434056258589,0
-0.29056370170672,48.434027571461,0
-0.29069653927967,48.433991063747,0
-0.29082407545869,48.433947013308,0
-0.29094533957607,48.433895755414,0
-0.29105940870893,48.43383768019,0
-0.29116541470403,48.433773229646,0
-0.29126255078565,48.433702894313,0
-0.29135007769566,48.433627209508,0
-0.29142732931967,48.433546751261,0
-0.29149371775594,48.433462131929,0
-0.29154873778874,48.433373995536,0
-0.2915919707322,48.433283012868,0
-0.29162308761521,48.433189876373,0
-0.29164185168329,48.433095294885,0
-0.29164812019857,48.432999988233,0
-0.29164184552398,48.432904681759,0
-0.29162307548374,48.432810100802,0
-0.2915919529972,48.432716965174,0
-0.29154871498904,48.432625983683,0
-0.2914936905843,48.43253784874,0
-0.29142729860172,48.432453231088,0
-0.29135004436475,48.4323727747,0
-0.2912625158545,48.432297091876,0
-0.29116537923403,48.432226758587,0
-0.29105937377779,48.432162310086,0
-0.29094530624516,48.432104236843,0
-0.29082404474074,48.432052980808,0
-0.29069651210804,48.43200893205,0
-0.29056367890702,48.431972425786,0
-0.29042655602875,48.431943739835,0
-0.2902861870035,48.4319230925,0
-0.29014364006046,48.431910640912,0
-0.28999999999996,48.431906479826,0
-0.28985635993945,48.431910640912,0
-0.28971381299643,48.4319230925,0
-0.28957344397116,48.431943739835,0
-0.28943632109289,48.431972425786,0
-0.28930348789187,48.43200893205,0
-0.28917595525917,48.432052980808,0
-0.28905469375476,48.432104236843,0
-0.28894062622215,48.432162310086,0
-0.28883462076591,48.432226758587,0
-0.28873748414543,48.432297091876,0
-0.28864995563519,48.4323727747,0
-0.28857270139819,48.432453231088,0
-0.28850630941561,48.43253784874,0
-0.28845128501089,48.432625983683,0
-0.28840804700274,48.432716965174,0
-0.28837692451618,48.432810100802,0
-0.28835815447596,48.432904681759,0
-0.28835187980137,48.432999988233,0
-0.28999999999996,48.433,0
</coordinates>
</LinearRing>

</outerBoundaryIs>
</Polygon>
</Placemark>
<Placemark>
<name>590</name>
<styleUrl>#stylehap1</styleUrl>
<Polygon>
<outerBoundaryIs>
<LinearRing>
<coordinates>
-0.28899999999999,48.427,0
-0.28735207438867,48.426999988235,0
-0.2873583421645,48.427095294888,0
-0.28737710401779,48.427189876375,0
-0.28740821722755,48.42728301287,0
-0.28745144506721,48.427373995538,0
-0.28750645860445,48.427462131931,0
-0.28757283920286,48.427546751263,0
-0.28765008170634,48.42762720951,0
-0.28773759828255,48.427702894315,0
-0.28783472289572,48.427773229648,0
-0.28794071637506,48.427837680191,0
-0.28805477204003,48.427895755415,0
-0.28817602183995,48.427947013309,0
-0.2883035429609,48.427991063747,0
-0.28843636484975,48.428027571461,0
-0.28857347660199,48.428056258589,0
-0.28871383465671,48.42807690679,0
-0.28885637074061,48.42808935891,0
-0.289,48.428093520173,0
-0.28914362925939,48.42808935891,0
-0.28928616534326,48.42807690679,0
-0.28942652339798,48.428056258589,0
-0.28956363515021,48.428027571461,0
-0.28969645703907,48.427991063747,0
-0.28982397816002,48.427947013309,0
-0.28994522795994,48.427895755415,0
-0.29005928362491,48.427837680191,0
-0.29016527710425,48.427773229648,0
-0.29026240171741,48.427702894315,0
-0.29034991829363,48.42762720951,0
-0.29042716079711,48.427546751263,0
-0.29049354139552,48.427462131931,0
-0.29054855493279,48.427373995538,0
-0.29059178277242,48.42728301287,0
-0.29062289598218,48.427189876375,0
-0.29064165783547,48.427095294888,0
-0.2906479256113,48.426999988235,0
-0.2906416516782,48.426904681762,0
-0.29062288385469,48.426810100805,0
-0.29059176504325,48.426716965176,0
-0.29054853214057,48.426625983685,0
-0.29049351423284,48.426537848742,0
-0.29042713008928,48.42645323109,0
-0.29034988497368,48.426372774702,0
-0.29026236679777,48.426297091878,0
-0.2901652416459,48.426226758588,0

-0.29005924870527,48.426162310087,0
-0.28994519463999,48.426104236844,0
-0.2898239474522,48.426052980809,0
-0.28969642987642,48.42600893205,0
-0.28956361235803,48.425972425786,0
-0.28942650566881,48.425943739835,0
-0.2892861532158,48.4259230925,0
-0.28914362310209,48.425910640912,0
-0.289,48.425906479826,0
-0.28885637689788,48.425910640912,0
-0.28871384678419,48.4259230925,0
-0.28857349433116,48.425943739835,0
-0.28843638764194,48.425972425786,0
-0.28830357012358,48.42600893205,0
-0.2881760525478,48.426052980809,0
-0.28805480535998,48.426104236844,0
-0.28794075129473,48.426162310087,0
-0.28783475835407,48.426226758588,0
-0.28773763320222,48.426297091878,0
-0.28765011502629,48.426372774702,0
-0.28757286991069,48.42645323109,0
-0.28750648576713,48.426537848742,0
-0.28745146785939,48.426625983685,0
-0.28740823495672,48.426716965176,0
-0.28737711614527,48.426810100805,0
-0.28735834832177,48.426904681762,0
-0.28735207438867,48.426999988235,0
-0.28899999999999,48.427,0
</coordinates>
</LinearRing>
</outerBoundaryIs>
</Polygon>
</Placemark>
<Placemark>
<name>591</name>
<styleUrl>#stylehap1</styleUrl>
<Polygon>
<outerBoundaryIs>
<LinearRing>
<coordinates>
-0.28800000000001,48.425,0
-0.28635213923688,48.424999988236,0
-0.28635840676629,48.425095294888,0
-0.28637716788152,48.425189876376,0
-0.28640827986709,48.425283012871,0
-0.28645150600584,48.425373995539,0
-0.2865065173784,48.425462131932,0
-0.28657289536476,48.425546751264,0
-0.28665013482872,48.42562720951,0
-0.2867376479611,48.425702894315,0
-0.28683476875229,48.425773229648,0
-0.28694075806062,48.425837680192,0
-0.28705480923729,48.425895755415,0
-0.28717605426577,48.425947013309,0
-0.28730357036844,48.425991063747,0
-0.28743638703042,48.426027571462,0
-0.28757349338693,48.426056258589,0

-0.28771384591818,48.42607690679,0
-0.28785637639285,48.42608935891,0
-0.28800000000001,48.426093520174,0
-0.28814362360718,48.42608935891,0
-0.28828615408184,48.42607690679,0
-0.28842650661309,48.426056258589,0
-0.2885636129696,48.426027571462,0
-0.28869642963158,48.425991063747,0
-0.28882394573425,48.425947013309,0
-0.28894519076273,48.425895755415,0
-0.2890592419394,48.425837680192,0
-0.28916523124771,48.425773229648,0
-0.28926235203892,48.425702894315,0
-0.28934986517131,48.42562720951,0
-0.28942710463527,48.425546751264,0
-0.28949348262163,48.425462131932,0
-0.28954849399416,48.425373995539,0
-0.28959172013294,48.425283012871,0
-0.28962283211851,48.425189876376,0
-0.28964159323374,48.425095294888,0
-0.28964786076312,48.424999988236,0
-0.28964158707713,48.424904681763,0
-0.28962281999238,48.424810100806,0
-0.2895917024057,48.424716965177,0
-0.2895484712045,48.424625983686,0
-0.28949345546193,48.424537848743,0
-0.2894270739308,48.424453231091,0
-0.289349831855,48.424372774702,0
-0.2892623171231,48.424297091878,0
-0.28916519579326,48.424226758588,0
-0.28905920702358,48.424162310087,0
-0.28894515744645,48.424104236844,0
-0.28882391502979,48.424052980809,0
-0.28869640247188,48.42400893205,0
-0.28856359017991,48.423972425786,0
-0.28842648888585,48.423943739835,0
-0.28828614195571,48.4239230925,0
-0.28814361745056,48.423910640912,0
-0.28800000000001,48.423906479826,0
-0.28785638254946,48.423910640912,0
-0.28771385804431,48.4239230925,0
-0.28757351111415,48.423943739835,0
-0.28743640982012,48.423972425786,0
-0.28730359752814,48.42400893205,0
-0.28717608497023,48.424052980809,0
-0.28705484255358,48.424104236844,0
-0.28694079297645,48.424162310087,0
-0.28683480420677,48.424226758588,0
-0.28673768287693,48.424297091878,0
-0.28665016814503,48.424372774702,0
-0.28657292606922,48.424453231091,0
-0.28650654453809,48.424537848743,0
-0.28645152879553,48.424625983686,0
-0.28640829759433,48.424716965177,0
-0.28637718000765,48.424810100806,0
-0.2863584129229,48.424904681763,0
-0.28635213923688,48.424999988236,0

-0.28800000000001,48.425,0
</coordinates>
</LinearRing>
</outerBoundaryIs>
</Polygon>
</Placemark>
<Placemark>
<name>592</name>
<styleUrl>#stylehap1</styleUrl>
<Polygon>
<outerBoundaryIs>
<LinearRing>
<coordinates>
-0.28800000000001,48.425,0
-0.28635213923688,48.424999988236,0
-0.28635840676629,48.425095294888,0
-0.28637716788152,48.425189876376,0
-0.28640827986709,48.425283012871,0
-0.28645150600584,48.425373995539,0
-0.2865065173784,48.425462131932,0
-0.28657289536476,48.425546751264,0
-0.28665013482872,48.42562720951,0
-0.2867376479611,48.425702894315,0
-0.28683476875229,48.425773229648,0
-0.28694075806062,48.425837680192,0
-0.28705480923729,48.425895755415,0
-0.28717605426577,48.425947013309,0
-0.28730357036844,48.425991063747,0
-0.28743638703042,48.426027571462,0
-0.28757349338693,48.426056258589,0
-0.28771384591818,48.42607690679,0
-0.28785637639285,48.42608935891,0
-0.28800000000001,48.426093520174,0
-0.28814362360718,48.42608935891,0
-0.28828615408184,48.42607690679,0
-0.28842650661309,48.426056258589,0
-0.2885636129696,48.426027571462,0
-0.28869642963158,48.425991063747,0
-0.28882394573425,48.425947013309,0
-0.28894519076273,48.425895755415,0
-0.2890592419394,48.425837680192,0
-0.28916523124771,48.425773229648,0
-0.28926235203892,48.425702894315,0
-0.28934986517131,48.42562720951,0
-0.28942710463527,48.425546751264,0
-0.28949348262163,48.425462131932,0
-0.28954849399416,48.425373995539,0
-0.28959172013294,48.425283012871,0
-0.28962283211851,48.425189876376,0
-0.28964159323374,48.425095294888,0
-0.28964786076312,48.424999988236,0
-0.28964158707713,48.424904681763,0
-0.28962281999238,48.424810100806,0
-0.2895917024057,48.424716965177,0
-0.2895484712045,48.424625983686,0
-0.28949345546193,48.424537848743,0
-0.2894270739308,48.424453231091,0

-0.289349831855,48.424372774702,0
-0.2892623171231,48.424297091878,0
-0.28916519579326,48.424226758588,0
-0.28905920702358,48.424162310087,0
-0.28894515744645,48.424104236844,0
-0.28882391502979,48.424052980809,0
-0.28869640247188,48.42400893205,0
-0.28856359017991,48.423972425786,0
-0.28842648888585,48.423943739835,0
-0.28828614195571,48.4239230925,0
-0.28814361745056,48.423910640912,0
-0.28800000000001,48.423906479826,0
-0.28785638254946,48.423910640912,0
-0.28771385804431,48.4239230925,0
-0.28757351111415,48.423943739835,0
-0.28743640982012,48.423972425786,0
-0.28730359752814,48.42400893205,0
-0.28717608497023,48.424052980809,0
-0.28705484255358,48.424104236844,0
-0.28694079297645,48.424162310087,0
-0.28683480420677,48.424226758588,0
-0.28673768287693,48.424297091878,0
-0.28665016814503,48.424372774702,0
-0.28657292606922,48.424453231091,0
-0.28650654453809,48.424537848743,0
-0.28645152879553,48.424625983686,0
-0.28640829759433,48.424716965177,0
-0.28637718000765,48.424810100806,0
-0.2863584129229,48.424904681763,0
-0.28635213923688,48.424999988236,0
-0.28800000000001,48.425,0
</coordinates>
</LinearRing>
</outerBoundaryIs>
</Polygon>
</Placemark>
<Placemark>
<name>593</name>
<styleUrl>#stylehap1</styleUrl>
<Polygon>
<outerBoundaryIs>
<LinearRing>
<coordinates>
-0.28800000000001,48.468,0
-0.28635074343181,48.467999988218,0
-0.28635701626539,48.468095294871,0
-0.28637579326751,48.468189876359,0
-0.28640693160199,48.468283012855,0
-0.28645019435119,48.468373995523,0
-0.28650525231733,48.468462131917,0
-0.28657168652584,48.46854675125,0
-0.2866489914128,48.468627209499,0
-0.28673657867114,48.468702894305,0
-0.28683378172719,48.468773229639,0
-0.28693986081317,48.468837680184,0
-0.28705400859703,48.468895755409,0
-0.28717535632696,48.468947013304,0

-0.28730298044366,48.468991063744,0
-0.28743590961006,48.46902757146,0
-0.28757313210505,48.469056258588,0
-0.28771360352485,48.46907690679,0
-0.28785625473315,48.46908935891,0
-0.28800000000001,48.469093520174,0
-0.28814374526688,48.46908935891,0
-0.28828639647517,48.46907690679,0
-0.28842686789497,48.469056258588,0
-0.28856409038997,48.46902757146,0
-0.28869701955636,48.468991063744,0
-0.28882464367306,48.468947013304,0
-0.28894599140297,48.468895755409,0
-0.28906013918685,48.468837680184,0
-0.28916621827283,48.468773229639,0
-0.28926342132889,48.468702894305,0
-0.28935100858723,48.468627209499,0
-0.28942831347418,48.46854675125,0
-0.2894947476827,48.468462131917,0
-0.28954980564883,48.468373995523,0
-0.28959306839803,48.468283012855,0
-0.28962420673251,48.468189876359,0
-0.28964298373464,48.468095294871,0
-0.28964925656822,48.467999988218,0
-0.2896429775635,48.467904681745,0
-0.28962419457773,48.467810100788,0
-0.28959305062896,48.467716965161,0
-0.28954978280532,48.46762598367,0
-0.28949472045888,48.467537848728,0
-0.2894282826972,48.467453231077,0
-0.28935097519224,48.46737277469,0
-0.2892633863306,48.467297091868,0
-0.28916618273464,48.467226758579,0
-0.28906010418856,48.46716231008,0
-0.28894595800801,48.467104236838,0
-0.28882461289608,48.467052980805,0
-0.28869699233254,48.467008932047,0
-0.28856406754646,48.466972425784,0
-0.28842685012587,48.466943739834,0
-0.28828638432042,48.4669230925,0
-0.28814373909574,48.466910640911,0
-0.28800000000001,48.466906479826,0
-0.28785626090429,48.466910640911,0
-0.28771361567961,48.4669230925,0
-0.28757314987415,48.466943739834,0
-0.28743593245356,48.466972425784,0
-0.28730300766748,48.467008932047,0
-0.28717538710394,48.467052980805,0
-0.28705404199201,48.467104236838,0
-0.28693989581146,48.46716231008,0
-0.28683381726539,48.467226758579,0
-0.2867366136694,48.467297091868,0
-0.28664902480776,48.46737277469,0
-0.2865717173028,48.467453231077,0
-0.28650527954114,48.467537848728,0
-0.2864502171947,48.46762598367,0
-0.28640694937106,48.467716965161,0

```
-0.28637580542229,48.467810100788,0
-0.28635702243653,48.467904681745,0
-0.28635074343181,48.467999988218,0
-0.28800000000001,48.468,0
</coordinates>
</LinearRing>
</outerBoundaryIs>
</Polygon>
</Placemark>
<Placemark>
<name>595</name>
<styleUrl>#stylehap1</styleUrl>
<Polygon>
<outerBoundaryIs>
<LinearRing>
<coordinates>
-0.28300000000002,48.501,0
-0.28134966999697,48.500999988205,0
-0.2813559469097,48.501095294857,0
-0.28137473612956,48.501189876346,0
-0.28140589472743,48.501283012842,0
-0.28144918563169,48.501373995511,0
-0.28150427943028,48.501462131906,0
-0.2815707568761,48.50154675124,0
-0.28164811207619,48.501627209489,0
-0.28173575634061,48.501702894297,0
-0.28183302266191,48.501773229632,0
-0.28193917079084,48.501837680179,0
-0.28205339286983,48.501895755405,0
-0.28217481958164,48.501947013301,0
-0.28230252676581,48.501991063742,0
-0.28243554245301,48.502027571458,0
-0.28257285426358,48.502056258587,0
-0.28271341711382,48.502076906789,0
-0.28285616117156,48.502089358909,0
-0.28300000000001,48.502093520174,0
-0.28314383882847,48.502089358909,0
-0.28328658288621,48.502076906789,0
-0.28342714573645,48.502056258587,0
-0.28356445754702,48.502027571458,0
-0.28369747323422,48.501991063742,0
-0.28382518041838,48.501947013301,0
-0.28394660713019,48.501895755405,0
-0.28406082920919,48.501837680179,0
-0.28416697733811,48.501773229632,0
-0.28426424365942,48.501702894297,0
-0.28435188792386,48.501627209489,0
-0.28442924312393,48.50154675124,0
-0.28449572056975,48.501462131906,0
-0.28455081436834,48.501373995511,0
-0.2845941052726,48.501283012842,0
-0.2846252638705,48.501189876346,0
-0.28464405309033,48.501095294857,0
-0.28465033000305,48.500999988205,0
-0.28464404690799,48.500904681731,0
-0.28462525169368,48.500810100775,0
-0.28459408747129,48.500716965148,0
```

-0.28455079148341,48.500625983658,0
-0.28449569329657,48.500537848717,0
-0.28442921229117,48.500453231067,0
-0.28435185446834,48.500372774681,0
-0.28426420859769,48.50029709186,0
-0.28416694173549,48.500226758572,0
-0.28406079414746,48.500162310074,0
-0.28394657367468,48.500104236834,0
-0.28382514958563,48.500052980801,0
-0.28369744596104,48.500008932045,0
-0.28356443466211,48.499972425783,0
-0.28342712793516,48.499943739833,0
-0.28328657070939,48.499923092499,0
-0.28314383264616,48.499910640911,0
-0.28300000000001,48.499906479826,0
-0.28285616735387,48.499910640911,0
-0.28271342929063,48.499923092499,0
-0.28257287206489,48.499943739833,0
-0.28243556533791,48.499972425783,0
-0.28230255403899,48.500008932045,0
-0.28217485041442,48.500052980801,0
-0.28205342632535,48.500104236834,0
-0.28193920585256,48.500162310074,0
-0.28183305826453,48.500226758572,0
-0.28173579140233,48.50029709186,0
-0.28164814553168,48.500372774681,0
-0.28157078770885,48.500453231067,0
-0.28150430670346,48.500537848717,0
-0.28144920851662,48.500625983658,0
-0.28140591252874,48.500716965148,0
-0.28137474830635,48.500810100775,0
-0.28135595309204,48.500904681731,0
-0.28134966999697,48.500999988205,0
-0.28300000000002,48.501,0
</coordinates>
</LinearRing>
</outerBoundaryIs>
</Polygon>
</Placemark>
<Placemark>
<name>599</name>
<styleUrl>#stylehap1</styleUrl>
<Polygon>
<outerBoundaryIs>
<LinearRing>
<coordinates>
-0.27700000000004,48.39,0
-0.27535327293017,48.389999988251,0
-0.27535953615142,48.390095294903,0
-0.27537828436307,48.39018987639,0
-0.2754093749477,48.390283012885,0
-0.27545257135091,48.390373995551,0
-0.27550754487948,48.390462131944,0
-0.27557387720137,48.390546751275,0
-0.27565106352783,48.39062720952,0
-0.27573851645399,48.390702894324,0
-0.27583557042849,48.390773229655,0

-0.27594148681808,48.390837680198,0
-0.27605545952899,48.39089575542,0
-0.27617662114185,48.390947013312,0
-0.27630404951398,48.39099106375,0
-0.27643677479822,48.391027571463,0
-0.27657378682536,48.39105625859,0
-0.27671404279359,48.391076906791,0
-0.27685647520667,48.39108935891,0
-0.27700000000005,48.391093520174,0
-0.27714352479341,48.39108935891,0
-0.27728595720649,48.391076906791,0
-0.27742621317473,48.39105625859,0
-0.27756322520186,48.391027571463,0
-0.2776959504861,48.39099106375,0
-0.27782337885823,48.390947013312,0
-0.27794454047109,48.39089575542,0
-0.278058513182,48.390837680198,0
-0.27816442957159,48.390773229655,0
-0.27826148354609,48.390702894324,0
-0.27834893647226,48.39062720952,0
-0.27842612279872,48.390546751275,0
-0.27849245512061,48.390462131944,0
-0.27854742864917,48.390373995551,0
-0.27859062505238,48.390283012885,0
-0.27862171563702,48.39018987639,0
-0.27864046384866,48.390095294903,0
-0.27864672706991,48.389999988251,0
-0.27864045770385,48.389904681777,0
-0.27862170353414,48.38981010082,0
-0.27859060735913,48.389716965191,0
-0.27854740590317,48.389625983699,0
-0.27849242801297,48.389537848754,0
-0.2784260921531,48.389453231101,0
-0.27834890321981,48.389372774712,0
-0.27826144869719,48.389297091887,0
-0.2781643941851,48.389226758595,0
-0.2780584783331,48.389162310093,0
-0.27794450721867,48.389104236849,0
-0.27782334821262,48.389052980813,0
-0.27769592337849,48.389008932053,0
-0.27756320245586,48.388972425788,0
-0.27742619548148,48.388943739836,0
-0.27728594510359,48.388923092501,0
-0.27714351864863,48.388910640912,0
-0.27700000000005,48.388906479827,0
-0.27685648135147,48.388910640912,0
-0.27671405489649,48.388923092501,0
-0.2765738045186,48.388943739836,0
-0.27643679754423,48.388972425788,0
-0.27630407662162,48.389008932053,0
-0.27617665178746,48.389052980813,0
-0.27605549278141,48.389104236849,0
-0.27594152166699,48.389162310093,0
-0.27583560581501,48.389226758595,0
-0.2757385513029,48.389297091887,0
-0.27565109678027,48.389372774712,0
-0.27557390784698,48.389453231101,0

-0.27550757198711,48.389537848754,0
-0.27545259409692,48.389625983699,0
-0.27540939264095,48.389716965191,0
-0.27537829646597,48.38981010082,0
-0.27535954229623,48.389904681777,0
-0.27535327293017,48.389999988251,0
-0.27700000000004,48.39,0
</coordinates>
</LinearRing>
</outerBoundaryIs>
</Polygon>
</Placemark>
<Placemark>
<name>603</name>
<styleUrl>#stylehap1</styleUrl>
<Polygon>
<outerBoundaryIs>
<LinearRing>
<coordinates>
-0.26900000000001,48.431,0
-0.26735194467094,48.430999988234,0
-0.26735821293971,48.431095294886,0
-0.26737697626947,48.431189876374,0
-0.26740809192792,48.431283012869,0
-0.26745132316991,48.431373995536,0
-0.26750634103731,48.43146213193,0
-0.26757272686066,48.431546751262,0
-0.26764997544416,48.431627209509,0
-0.26773749890918,48.431702894314,0
-0.26783463116756,48.431773229647,0
-0.26794063299028,48.431837680191,0
-0.26805469763337,48.431895755415,0
-0.26817595697773,48.431947013308,0
-0.26830348813684,48.431991063747,0
-0.2684363204812,48.432027571461,0
-0.26857344302663,48.432056258589,0
-0.26871381213014,48.43207690679,0
-0.2688563594343,48.43208935891,0
-0.269,48.432093520173,0
-0.26914364056573,48.43208935891,0
-0.26928618786985,48.43207690679,0
-0.26942655697337,48.432056258589,0
-0.26956367951883,48.432027571461,0
-0.26969651186316,48.431991063747,0
-0.26982404302227,48.431947013308,0
-0.26994530236665,48.431895755415,0
-0.27005936700971,48.431837680191,0
-0.27016536883244,48.431773229647,0
-0.27026250109082,48.431702894314,0
-0.27035002455586,48.431627209509,0
-0.27042727313936,48.431546751262,0
-0.27049365896271,48.43146213193,0
-0.27054867683009,48.431373995536,0
-0.27059190807211,48.431283012869,0
-0.27062302373055,48.431189876374,0
-0.27064178706029,48.431095294886,0
-0.27064805532906,48.430999988234,0

-0.27064178090167,48.43090468176,0
-0.27062301160043,48.430810100803,0
-0.27059189033904,48.430716965175,0
-0.27054865403289,48.430625983684,0
-0.27049363179408,48.43053784874,0
-0.2704272424248,48.430453231089,0
-0.27034999122861,48.430372774701,0
-0.27026246616349,48.430297091877,0
-0.27016533336633,48.430226758587,0
-0.27005933208239,48.430162310086,0
-0.2699452690394,48.430104236843,0
-0.2698240123077,48.430052980809,0
-0.26969648469453,48.43000893205,0
-0.26956365672163,48.429972425786,0
-0.2694265392403,48.429943739835,0
-0.26928617573973,48.4299230925,0
-0.26914363440708,48.429910640912,0
-0.269,48.429906479826,0
-0.26885636559292,48.429910640912,0
-0.26871382426027,48.4299230925,0
-0.26857346075969,48.429943739835,0
-0.26843634327837,48.429972425786,0
-0.26830351530547,48.43000893205,0
-0.26817598769232,48.430052980809,0
-0.26805473096062,48.430104236843,0
-0.26794066791761,48.430162310086,0
-0.2678346666337,48.430226758587,0
-0.2677375338365,48.430297091877,0
-0.26765000877141,48.430372774701,0
-0.26757275757523,48.430453231089,0
-0.26750636820594,48.43053784874,0
-0.26745134596711,48.430625983684,0
-0.26740810966098,48.430716965175,0
-0.26737698839959,48.430810100803,0
-0.26735821909836,48.43090468176,0
-0.26735194467094,48.430999988234,0
-0.26900000000001,48.431,0
</coordinates>
</LinearRing>
</outerBoundaryIs>
</Polygon>
</Placemark>
<Placemark>
<name>604</name>
<styleUrl>#stylehap1</styleUrl>
<Polygon>
<outerBoundaryIs>
<LinearRing>
<coordinates>
-0.26700000000005,48.205,0
-0.26535922934484,48.204999988327,0
-0.2653654699311,48.205095294978,0
-0.26538415034736,48.205189876464,0
-0.26541512849158,48.205283012956,0
-0.2654581686644,48.205373995619,0
-0.26551294336101,48.205462132006,0
-0.26557903576214,48.205546751332,0

-0.26565594290457,48.205627209571,0
-0.26574307950788,48.205702894368,0
-0.26583978242795,48.205773229693,0
-0.26594531570316,48.205837680229,0
-0.26605887615559,48.205895755445,0
-0.26617959950356,48.205947013331,0
-0.26630656694014,48.205991063764,0
-0.26643881212659,48.206027571472,0
-0.26657532854804,48.206056258595,0
-0.26671507717511,48.206076906793,0
-0.26685699437337,48.20608935891,0
-0.26700000000005,48.206093520173,0
-0.26714300562674,48.20608935891,0
-0.267284922825,48.206076906793,0
-0.26742467145206,48.206056258595,0
-0.26756118787352,48.206027571472,0
-0.26769343305997,48.205991063764,0
-0.26782040049655,48.205947013331,0
-0.26794112384452,48.205895755445,0
-0.26805468429692,48.205837680229,0
-0.26816021757216,48.205773229693,0
-0.2682569204922,48.205702894368,0
-0.26834405709554,48.205627209571,0
-0.26842096423794,48.205546751332,0
-0.26848705663909,48.205462132006,0
-0.26854183133571,48.205373995619,0
-0.26858487150853,48.205283012956,0
-0.26861584965274,48.205189876464,0
-0.26863453006901,48.205095294978,0
-0.26864077065527,48.204999988327,0
-0.26863452398611,48.204904681853,0
-0.26861583767175,48.204810100893,0
-0.26858485399349,48.204716965262,0
-0.26854180881884,48.204625983766,0
-0.26848702980453,48.204537848817,0
-0.26842093390105,48.204453231158,0
-0.26834402417807,48.204372774763,0
-0.26825688599435,48.204297091932,0
-0.26816018254212,48.204226758633,0
-0.26805464979908,48.204162310125,0
-0.26794109092706,48.204104236874,0
-0.26782037015966,48.204052980832,0
-0.2676934062254,48.204008932067,0
-0.26756116535664,48.203972425797,0
-0.26742465393706,48.203943739841,0
-0.267284910844,48.203923092503,0
-0.26714299954384,48.203910640912,0
-0.26700000000005,48.203906479826,0
-0.26685700045627,48.203910640912,0
-0.26671508915608,48.203923092503,0
-0.26657534606305,48.203943739841,0
-0.26643883464347,48.203972425797,0
-0.26630659377471,48.204008932067,0
-0.26617962984045,48.204052980832,0
-0.26605890907305,48.204104236874,0
-0.26594535020103,48.204162310125,0
-0.26583981745799,48.204226758633,0

-0.26574311400576,48.204297091932,0
-0.26565597582204,48.204372774763,0
-0.26557906609906,48.204453231158,0
-0.26551297019558,48.204537848817,0
-0.26545819118127,48.204625983766,0
-0.26541514600662,48.204716965262,0
-0.26538416232834,48.204810100893,0
-0.265365476014,48.204904681853,0
-0.26535922934484,48.204999988327,0
-0.26700000000005,48.205,0
</coordinates>
</LinearRing>
</outerBoundaryIs>
</Polygon>
</Placemark>
<Placemark>
<name>605</name>
<styleUrl>#stylehap1</styleUrl>
<Polygon>
<outerBoundaryIs>
<LinearRing>
<coordinates>
-0.26499999999999,48.443,0
-0.26335155534694,48.442999988229,0
-0.26335782509517,48.443095294881,0
-0.26337659285616,48.443189876369,0
-0.26340771586395,48.443283012864,0
-0.26345095731751,48.443373995532,0
-0.26350598818099,48.443462131926,0
-0.26357238968607,48.443546751258,0
-0.26364965651761,48.443627209505,0
-0.26373720065819,48.443702894311,0
-0.26383435586224,48.443773229644,0
-0.26394038272614,48.443837680189,0
-0.26405447431531,48.443895755413,0
-0.2641757623056,48.443947013307,0
-0.26430332359246,48.443991063746,0
-0.26443618731703,48.444027571461,0
-0.26457334225631,48.444056258589,0
-0.26471374452075,48.44407690679,0
-0.26485632550043,48.44408935891,0
-0.26499999999998,48.444093520174,0
-0.26514367449956,48.44408935891,0
-0.26528625547925,48.44407690679,0
-0.26542665774366,48.444056258589,0
-0.26556381268296,48.444027571461,0
-0.26569667640751,48.443991063746,0
-0.26582423769436,48.443947013307,0
-0.26594552568468,48.443895755413,0
-0.26605961727383,48.443837680189,0
-0.26616564413772,48.443773229644,0
-0.26626279934177,48.443702894311,0
-0.26635034348235,48.443627209505,0
-0.26642761031392,48.443546751258,0
-0.26649401181898,48.443462131926,0
-0.26654904268246,48.443373995532,0
-0.26659228413602,48.443283012864,0

-0.26662340714381,48.443189876369,0
-0.2666421749048,48.443095294881,0
-0.26664844465303,48.442999988229,0
-0.2666421687421,48.442904681755,0
-0.2666233950057,48.442810100798,0
-0.26659226639127,48.44271696517,0
-0.26654901987025,48.442625983679,0
-0.26649398463246,48.442537848736,0
-0.26642757957912,48.442453231085,0
-0.26635031013314,48.442372774697,0
-0.26626276439147,48.442297091874,0
-0.26616560864826,48.442226758584,0
-0.26605958232351,48.442162310084,0
-0.26594549233547,48.442104236842,0
-0.26582420695957,48.442052980807,0
-0.26569664922099,48.442008932049,0
-0.26556378987075,48.441972425785,0
-0.26542663999891,48.441943739834,0
-0.26528624334113,48.4419230925,0
-0.26514366833687,48.441910640911,0
-0.26499999999998,48.441906479826,0
-0.2648563316631,48.441910640911,0
-0.26471375665886,48.4419230925,0
-0.26457336000106,48.441943739834,0
-0.26443621012922,48.441972425785,0
-0.26430335077897,48.442008932049,0
-0.2641757930404,48.442052980807,0
-0.2640545076645,48.442104236842,0
-0.26394041767646,48.442162310084,0
-0.26383439135171,48.442226758584,0
-0.2637372356085,48.442297091874,0
-0.26364968986682,48.442372774697,0
-0.26357242042084,48.442453231085,0
-0.2635060153675,48.442537848736,0
-0.26345098012972,48.442625983679,0
-0.2634077336087,48.44271696517,0
-0.26337660499427,48.442810100798,0
-0.26335783125786,48.442904681755,0
-0.26335155534694,48.442999988229,0
-0.26499999999999,48.443,0
</coordinates>
</LinearRing>
</outerBoundaryIs>
</Polygon>
</Placemark>
<Placemark>
<name>606</name>
<styleUrl>#stylehap1</styleUrl>
<Polygon>
<outerBoundaryIs>
<LinearRing>
<coordinates>
-0.26099999999997,48.443,0
-0.25935155534692,48.442999988229,0
-0.25935782509516,48.443095294881,0
-0.25937659285614,48.443189876369,0
-0.25940771586394,48.443283012864,0

-0.25945095731749,48.443373995532,0
-0.25950598818097,48.443462131926,0
-0.25957238968603,48.443546751258,0
-0.2596496565176,48.443627209505,0
-0.25973720065818,48.443702894311,0
-0.25983435586223,48.443773229644,0
-0.25994038272612,48.443837680189,0
-0.26005447431527,48.443895755413,0
-0.26017576230559,48.443947013307,0
-0.26030332359244,48.443991063746,0
-0.26043618731702,48.444027571461,0
-0.26057334225629,48.444056258589,0
-0.26071374452071,48.44407690679,0
-0.26085632550042,48.44408935891,0
-0.26099999999997,48.444093520174,0
-0.26114367449952,48.44408935891,0
-0.26128625547921,48.44407690679,0
-0.26142665774364,48.444056258589,0
-0.26156381268292,48.444027571461,0
-0.26169667640749,48.443991063746,0
-0.26182423769435,48.443947013307,0
-0.26194552568464,48.443895755413,0
-0.26205961727382,48.443837680189,0
-0.26216564413771,48.443773229644,0
-0.26226279934176,48.443702894311,0
-0.26235034348234,48.443627209505,0
-0.2624276103139,48.443546751258,0
-0.26249401181896,48.443462131926,0
-0.26254904268245,48.443373995532,0
-0.262592284136,48.443283012864,0
-0.2626234071438,48.443189876369,0
-0.26264217490478,48.443095294881,0
-0.26264844465302,48.442999988229,0
-0.26264216874209,48.442904681755,0
-0.26262339500568,48.442810100798,0
-0.26259226639125,48.44271696517,0
-0.26254901987023,48.442625983679,0
-0.26249398463245,48.442537848736,0
-0.26242757957911,48.442453231085,0
-0.26235031013313,48.442372774697,0
-0.26226276439146,48.442297091874,0
-0.26216560864824,48.442226758584,0
-0.26205958232349,48.442162310084,0
-0.26194549233545,48.442104236842,0
-0.26182420695955,48.442052980807,0
-0.26169664922098,48.442008932049,0
-0.26156378987074,48.441972425785,0
-0.2614266399989,48.441943739834,0
-0.26128624334109,48.4419230925,0
-0.26114366833685,48.441910640911,0
-0.26099999999997,48.441906479826,0
-0.26085633166309,48.441910640911,0
-0.26071375665882,48.4419230925,0
-0.26057336000104,48.441943739834,0
-0.2604362101292,48.441972425785,0
-0.26030335077896,48.442008932049,0
-0.26017579304038,48.442052980807,0

-0.26005450766448,48.442104236842,0
-0.25994041767645,48.442162310084,0
-0.2598343913517,48.442226758584,0
-0.25973723560848,48.442297091874,0
-0.25964968986681,48.442372774697,0
-0.25957242042083,48.442453231085,0
-0.25950601536749,48.442537848736,0
-0.2594509801297,48.442625983679,0
-0.25940773360868,48.44271696517,0
-0.25937660499426,48.442810100798,0
-0.25935783125785,48.442904681755,0
-0.25935155534692,48.442999988229,0
-0.26099999999997,48.443,0
</coordinates>
</LinearRing>
</outerBoundaryIs>
</Polygon>
</Placemark>
<Placemark>
<name>608</name>
<styleUrl>#stylehap1</styleUrl>
<Polygon>
<outerBoundaryIs>
<LinearRing>
<coordinates>
-0.25999999999999,48.443,0
-0.25835155534696,48.442999988229,0
-0.2583578250952,48.443095294881,0
-0.25837659285618,48.443189876369,0
-0.25840771586395,48.443283012864,0
-0.25845095731753,48.443373995532,0
-0.25850598818099,48.443462131926,0
-0.25857238968607,48.443546751258,0
-0.25864965651761,48.443627209505,0
-0.25873720065819,48.443702894311,0
-0.25883435586224,48.443773229644,0
-0.25894038272614,48.443837680189,0
-0.25905447431531,48.443895755413,0
-0.25917576230563,48.443947013307,0
-0.25930332359246,48.443991063746,0
-0.25943618731703,48.444027571461,0
-0.25957334225633,48.444056258589,0
-0.25971374452075,48.44407690679,0
-0.25985632550043,48.44408935891,0
-0.25999999999998,48.444093520174,0
-0.26014367449956,48.44408935891,0
-0.26028625547925,48.44407690679,0
-0.26042665774366,48.444056258589,0
-0.26056381268296,48.444027571461,0
-0.26069667640751,48.443991063746,0
-0.26082423769436,48.443947013307,0
-0.26094552568468,48.443895755413,0
-0.26105961727383,48.443837680189,0
-0.26116564413773,48.443773229644,0
-0.26126279934177,48.443702894311,0
-0.26135034348235,48.443627209505,0
-0.26142761031392,48.443546751258,0

-0.26149401181901,48.443462131926,0
-0.26154904268246,48.443373995532,0
-0.26159228413602,48.443283012864,0
-0.26162340714381,48.443189876369,0
-0.2616421749048,48.443095294881,0
-0.26164844465303,48.442999988229,0
-0.2616421687421,48.442904681755,0
-0.2616233950057,48.442810100798,0
-0.26159226639127,48.44271696517,0
-0.26154901987025,48.442625983679,0
-0.26149398463249,48.442537848736,0
-0.26142757957912,48.442453231085,0
-0.26135031013314,48.442372774697,0
-0.26126276439147,48.442297091874,0
-0.26116560864826,48.442226758584,0
-0.26105958232353,48.442162310084,0
-0.26094549233547,48.442104236842,0
-0.26082420695957,48.442052980807,0
-0.26069664922099,48.442008932049,0
-0.26056378987075,48.441972425785,0
-0.26042663999891,48.441943739834,0
-0.26028624334113,48.4419230925,0
-0.26014366833687,48.441910640911,0
-0.25999999999998,48.441906479826,0
-0.2598563316631,48.441910640911,0
-0.25971375665886,48.4419230925,0
-0.25957336000106,48.441943739834,0
-0.25943621012922,48.441972425785,0
-0.25930335077898,48.442008932049,0
-0.2591757930404,48.442052980807,0
-0.2590545076645,48.442104236842,0
-0.25894041767646,48.442162310084,0
-0.25883439135171,48.442226758584,0
-0.25873723560852,48.442297091874,0
-0.25864968986682,48.442372774697,0
-0.25857242042087,48.442453231085,0
-0.2585060153675,48.442537848736,0
-0.25845098012972,48.442625983679,0
-0.2584077336087,48.44271696517,0
-0.2583766049943,48.442810100798,0
-0.25835783125786,48.442904681755,0
-0.25835155534696,48.442999988229,0
-0.25999999999999,48.443,0
</coordinates>
</LinearRing>
</outerBoundaryIs>
</Polygon>
</Placemark>
<Placemark>
<name>609</name>
<styleUrl>#stylehap1</styleUrl>
<Polygon>
<outerBoundaryIs>
<LinearRing>
<coordinates>
-0.25700000000006,48.44,0
-0.25535165270203,48.43999998823,0

-0.2553579220803,48.440095294882,0
-0.25537668873321,48.44018987637,0
-0.25540780990323,48.440283012865,0
-0.25545104880326,48.440373995533,0
-0.2555060764169,48.440462131927,0
-0.25557247400059,48.440546751259,0
-0.255649736269,48.440627209506,0
-0.25573727523942,48.440702894311,0
-0.25583442470562,48.440773229645,0
-0.25594044530769,48.440837680189,0
-0.25605453015867,48.440895755413,0
-0.25617581098572,48.440947013307,0
-0.25630336473878,48.440991063746,0
-0.25643622061636,48.441027571461,0
-0.25657336745518,48.441056258589,0
-0.25671376142732,48.44107690679,0
-0.25685633398605,48.44108935891,0
-0.25700000000006,48.441093520174,0
-0.25714366601406,48.44108935891,0
-0.25728623857279,48.44107690679,0
-0.25742663254493,48.441056258589,0
-0.25756377938377,48.441027571461,0
-0.25769663526133,48.440991063746,0
-0.25782418901439,48.440947013307,0
-0.25794546984146,48.440895755413,0
-0.25805955469242,48.440837680189,0
-0.25816557529449,48.440773229645,0
-0.25826272476071,48.440702894311,0
-0.25835026373111,48.440627209506,0
-0.25842752599955,48.440546751259,0
-0.25849392358321,48.440462131927,0
-0.25854895119685,48.440373995533,0
-0.25859219009691,48.440283012865,0
-0.2586233112669,48.44018987637,0
-0.25864207791981,48.440095294882,0
-0.25864834729808,48.43999998823,0
-0.25864207175813,48.439904681757,0
-0.2586232991308,48.4398101008,0
-0.25859217235509,48.439716965171,0
-0.25854892838841,48.43962598368,0
-0.25849389640117,48.439537848737,0
-0.25842749526982,48.439453231086,0
-0.2583502303874,48.439372774698,0
-0.25826268981614,48.439297091875,0
-0.25816553981084,48.439226758585,0
-0.25805951974787,48.439162310085,0
-0.25794543649775,48.439104236842,0
-0.25782415828466,48.439052980808,0
-0.25769660807929,48.439008932049,0
-0.25756375657533,48.438972425785,0
-0.25742661480311,48.438943739834,0
-0.25728622643668,48.4389230925,0
-0.25714365985239,48.438910640911,0
-0.25700000000006,48.438906479826,0
-0.25685634014772,48.438910640911,0
-0.25671377356346,48.4389230925,0
-0.256573385197,48.438943739834,0

-0.25643624342478,48.438972425785,0
-0.25630339192082,48.439008932049,0
-0.25617584171545,48.439052980808,0
-0.25605456350239,48.439104236842,0
-0.25594048025227,48.439162310085,0
-0.25583446018927,48.439226758585,0
-0.25573731018397,48.439297091875,0
-0.25564976961271,48.439372774698,0
-0.25557250473032,48.439453231086,0
-0.25550610359896,48.439537848737,0
-0.2554510716117,48.43962598368,0
-0.25540782764505,48.439716965171,0
-0.25537670086934,48.4398101008,0
-0.25535792824198,48.439904681757,0
-0.25535165270203,48.43999998823,0
-0.25700000000006,48.44,0
</coordinates>
</LinearRing>
</outerBoundaryIs>
</Polygon>
</Placemark>
<Placemark>
<name>610</name>
<styleUrl>#stylehap1</styleUrl>
<Polygon>
<outerBoundaryIs>
<LinearRing>
<coordinates>
-0.25599999999997,48.43,0
-0.25435197710303,48.429999988234,0
-0.25435824524854,48.430095294886,0
-0.25437700820915,48.430189876374,0
-0.25440812325536,48.430283012869,0
-0.25445135364672,48.430373995537,0
-0.25450637043148,48.43046213193,0
-0.25457275494849,48.430546751262,0
-0.25465000201187,48.430627209509,0
-0.25473752375455,48.430702894314,0
-0.25483465410147,48.430773229647,0
-0.25494065383818,48.430837680191,0
-0.25505471623653,48.430895755415,0
-0.25517597319461,48.430947013308,0
-0.25530350184396,48.430991063747,0
-0.2554363315742,48.431027571461,0
-0.25557345142112,48.431056258589,0
-0.25571381776221,48.43107690679,0
-0.25585636226107,48.43108935891,0
-0.25599999999997,48.431093520173,0
-0.25614363773887,48.43108935891,0
-0.25628618223772,48.43107690679,0
-0.25642654857881,48.431056258589,0
-0.25656366842573,48.431027571461,0
-0.25669649815598,48.430991063747,0
-0.25682402680535,48.430947013308,0
-0.25694528376341,48.430895755415,0
-0.25705934616178,48.430837680191,0
-0.25716534589847,48.430773229647,0

-0.25726247624539,48.430702894314,0
-0.2573499979881,48.430627209509,0
-0.25742724505145,48.430546751262,0
-0.25749362956845,48.43046213193,0
-0.25754864635322,48.430373995537,0
-0.25759187674458,48.430283012869,0
-0.25762299179081,48.430189876374,0
-0.2576417547514,48.430095294886,0
-0.25764802289691,48.429999988234,0
-0.2576417485931,48.429904681761,0
-0.25762297966134,48.429810100804,0
-0.25759185901248,48.429716965175,0
-0.2575486235573,48.429625983684,0
-0.25749360240133,48.429537848741,0
-0.25742721433856,48.429453231089,0
-0.25734996466265,48.429372774701,0
-0.25726244132,48.429297091877,0
-0.25716531043429,48.429226758587,0
-0.25705931123637,48.429162310086,0
-0.25694525043799,48.429104236844,0
-0.25682399609247,48.429052980809,0
-0.25669647098885,48.42900893205,0
-0.25656364562978,48.428972425786,0
-0.25642653084671,48.428943739835,0
-0.25628617010826,48.4289230925,0
-0.25614363158058,48.428910640912,0
-0.25599999999997,48.428906479826,0
-0.25585636841936,48.428910640912,0
-0.2557138298916,48.4289230925,0
-0.25557346915322,48.428943739835,0
-0.25543635437015,48.428972425786,0
-0.25530352901111,48.42900893205,0
-0.25517600390747,48.429052980809,0
-0.25505474956195,48.429104236844,0
-0.25494068876357,48.429162310086,0
-0.25483468956564,48.429226758587,0
-0.25473755867994,48.429297091877,0
-0.25465003533729,48.429372774701,0
-0.25457278566138,48.429453231089,0
-0.25450639759861,48.429537848741,0
-0.25445137644267,48.429625983684,0
-0.25440814098746,48.429716965175,0
-0.25437702033862,48.429810100804,0
-0.25435825140683,48.429904681761,0
-0.25435197710303,48.429999988234,0
-0.25599999999997,48.43,0
</coordinates>
</LinearRing>
</outerBoundaryIs>
</Polygon>
</Placemark>
<Placemark>
<name>611</name>
<styleUrl>#stylehap1</styleUrl>
<Polygon>
<outerBoundaryIs>
<LinearRing>

<coordinates>
-0.25599999999997,48.431,0
-0.25435194467091,48.430999988234,0
-0.25435821293968,48.431095294886,0
-0.25437697626941,48.431189876374,0
-0.25440809192786,48.431283012869,0
-0.25445132316988,48.431373995536,0
-0.25450634103728,48.43146213193,0
-0.25457272686063,48.431546751262,0
-0.25464997544411,48.431627209509,0
-0.25473749890915,48.431702894314,0
-0.25483463116753,48.431773229647,0
-0.25494063299025,48.431837680191,0
-0.25505469763332,48.431895755415,0
-0.2551759569777,48.431947013308,0
-0.25530348813681,48.431991063747,0
-0.25543632048117,48.432027571461,0
-0.2555734430266,48.432056258589,0
-0.25571381213011,48.43207690679,0
-0.25585635943427,48.43208935891,0
-0.25599999999997,48.432093520173,0
-0.25614364056567,48.43208935891,0
-0.25628618786982,48.43207690679,0
-0.25642655697334,48.432056258589,0
-0.2565636795188,48.432027571461,0
-0.25669651186313,48.431991063747,0
-0.25682404302224,48.431947013308,0
-0.25694530236662,48.431895755415,0
-0.25705936700968,48.431837680191,0
-0.25716536883241,48.431773229647,0
-0.25726250109079,48.431702894314,0
-0.25735002455583,48.431627209509,0
-0.25742727313933,48.431546751262,0
-0.25749365896268,48.43146213193,0
-0.25754867683006,48.431373995536,0
-0.25759190807208,48.431283012869,0
-0.25762302373053,48.431189876374,0
-0.25764178706026,48.431095294886,0
-0.25764805532903,48.430999988234,0
-0.25764178090164,48.43090468176,0
-0.2576230116004,48.430810100803,0
-0.25759189033901,48.430716965175,0
-0.25754865403286,48.430625983684,0
-0.25749363179405,48.43053784874,0
-0.25742724242477,48.430453231089,0
-0.25734999122855,48.430372774701,0
-0.25726246616346,48.430297091877,0
-0.25716533336627,48.430226758587,0
-0.25705933208236,48.430162310086,0
-0.25694526903937,48.430104236843,0
-0.25682401230767,48.430052980809,0
-0.2566964846945,48.43000893205,0
-0.2565636567216,48.429972425786,0
-0.25642653924027,48.429943739835,0
-0.2562861757397,48.4299230925,0
-0.25614363440705,48.429910640912,0
-0.25599999999997,48.429906479826,0

-0.25585636559289,48.429910640912,0
-0.25571382426024,48.4299230925,0
-0.25557346075966,48.429943739835,0
-0.25543634327834,48.429972425786,0
-0.25530351530544,48.43000893205,0
-0.25517598769226,48.430052980809,0
-0.25505473096059,48.430104236843,0
-0.25494066791758,48.430162310086,0
-0.25483466663367,48.430226758587,0
-0.25473753383647,48.430297091877,0
-0.25465000877138,48.430372774701,0
-0.2545727575752,48.430453231089,0
-0.25450636820591,48.43053784874,0
-0.25445134596708,48.430625983684,0
-0.25440810966093,48.430716965175,0
-0.25437698839956,48.430810100803,0
-0.2543582190983,48.43090468176,0
-0.25435194467091,48.430999988234,0
-0.25599999999997,48.431,0
</coordinates>
</LinearRing>
</outerBoundaryIs>
</Polygon>
</Placemark>
<Placemark>
<name>612</name>
<styleUrl>#stylehap1</styleUrl>
<Polygon>
<outerBoundaryIs>
<LinearRing>
<coordinates>
-0.255,48.436,0
-0.25335178248376,48.435999988232,0
-0.25335805136884,48.436095294884,0
-0.25337681654459,48.436189876372,0
-0.25340793526467,48.436283012867,0
-0.25345117076066,48.436373995535,0
-0.25350619404206,48.436462131928,0
-0.25357258639822,48.43654675126,0
-0.2536498425836,48.436627209507,0
-0.25373737466179,48.436702894312,0
-0.25383451647903,48.436773229646,0
-0.25394052873357,48.43683768019,0
-0.25405460460203,48.436895755414,0
-0.25417587587993,48.436947013308,0
-0.25430341958984,48.436991063747,0
-0.25443626500676,48.437027571461,0
-0.25457340104706,48.437056258589,0
-0.25471378396497,48.43707690679,0
-0.25485634529789,48.43708935891,0
-0.25499999999998,48.437093520174,0
-0.25514365470211,48.43708935891,0
-0.25528621603502,48.43707690679,0
-0.25542659895294,48.437056258589,0
-0.25556373499323,48.437027571461,0
-0.25569658041015,48.436991063747,0
-0.25582412412006,48.436947013308,0

-0.25594539539796,48.436895755414,0
-0.25605947126642,48.43683768019,0
-0.25616548352096,48.436773229646,0
-0.25626262533821,48.436702894312,0
-0.25635015741639,48.436627209507,0
-0.25642741360178,48.43654675126,0
-0.25649380595793,48.436462131928,0
-0.2565488292393,48.436373995535,0
-0.25659206473532,48.436283012867,0
-0.2566231834554,48.436189876372,0
-0.25664194863115,48.436095294884,0
-0.25664821751624,48.435999988232,0
-0.25664194247083,48.435904681758,0
-0.25662317132194,48.435810100801,0
-0.2565920469974,48.435716965173,0
-0.25654880643587,48.435625983682,0
-0.25649377878185,48.435537848739,0
-0.25642738287879,48.435453231087,0
-0.25635012407998,48.435372774699,0
-0.25626259040131,48.435297091876,0
-0.2561654480451,48.435226758586,0
-0.25605943632951,48.435162310085,0
-0.25594536206155,48.435104236843,0
-0.25582409339708,48.435052980808,0
-0.25569655323407,48.43500893205,0
-0.25556371218978,48.434972425786,0
-0.25542658121501,48.434943739835,0
-0.25528620390156,48.4349230925,0
-0.25514364854178,48.434910640912,0
-0.25499999999998,48.434906479826,0
-0.25485635145821,48.434910640912,0
-0.25471379609843,48.4349230925,0
-0.25457341878498,48.434943739835,0
-0.25443628781022,48.434972425786,0
-0.25430344676593,48.43500893205,0
-0.25417590660292,48.435052980808,0
-0.25405463793842,48.435104236843,0
-0.25394056367049,48.435162310085,0
-0.25383455195489,48.435226758586,0
-0.25373740959868,48.435297091876,0
-0.25364987592001,48.435372774699,0
-0.2535726171212,48.435453231087,0
-0.25350622121814,48.435537848739,0
-0.25345119356412,48.435625983682,0
-0.2534079530026,48.435716965173,0
-0.25337682867805,48.435810100801,0
-0.25335805752917,48.435904681758,0
-0.25335178248376,48.435999988232,0
-0.255,48.436,0
</coordinates>
</LinearRing>
</outerBoundaryIs>
</Polygon>
</Placemark>
<Placemark>
<name>613</name>
<styleUrl>#stylehap1</styleUrl>

<Polygon>
<outerBoundaryIs>
<LinearRing>
<coordinates>
-0.25400000000002,48.102,0
-0.25236251960311,48.101999988369,0
-0.252368747686,48.10209529502,0
-0.25238739065281,48.102189876505,0
-0.25241830668613,48.102283012995,0
-0.25246126055895,48.102373995656,0
-0.25251592542297,48.102462132041,0
-0.25258188529446,48.102546751363,0
-0.25265863821866,48.1026272096,0
-0.25274560008869,48.102702894393,0
-0.2528421090901,48.102773229714,0
-0.25294743073716,48.102837680247,0
-0.25306076346248,48.102895755459,0
-0.25318124471775,48.102947013342,0
-0.25330795753861,48.102991063771,0
-0.25343993752434,48.103027571477,0
-0.25357618017863,48.103056258598,0
-0.25371564855603,48.103076906794,0
-0.2538572811553,48.103089358911,0
-0.25400000000002,48.103093520174,0
-0.25414271884475,48.103089358911,0
-0.25428435144399,48.103076906794,0
-0.2544238198214,48.103056258598,0
-0.25456006247571,48.103027571477,0
-0.25469204246144,48.102991063771,0
-0.2548187552823,48.102947013342,0
-0.25493923653754,48.102895755459,0
-0.25505256926289,48.102837680247,0
-0.25515789090992,48.102773229714,0
-0.25525439991134,48.102702894393,0
-0.25534136178136,48.1026272096,0
-0.25541811470557,48.102546751363,0
-0.25548407457708,48.102462132041,0
-0.2555387394411,48.102373995656,0
-0.25558169331389,48.102283012995,0
-0.25561260934722,48.102189876505,0
-0.25563125231405,48.10209529502,0
-0.25563748039692,48.101999988369,0
-0.25563124626527,48.101904681894,0
-0.25561259743344,48.101810100934,0
-0.25558167589712,48.101716965301,0
-0.25553871705051,48.101625983803,0
-0.25548404789301,48.101537848852,0
-0.25541808453882,48.10145323119,0
-0.25534132904852,48.101372774791,0
-0.25525436560697,48.101297091956,0
-0.25515785607636,48.101226758655,0
-0.25505253495852,48.101162310142,0
-0.2549392038047,48.101104236888,0
-0.25481872511553,48.101052980843,0
-0.25469201577737,48.101008932074,0
-0.25456004008512,48.100972425802,0
-0.25442380240463,48.100943739844,0

-0.25428433953022,48.100923092504,0
-0.25414271279597,48.100910640913,0
-0.25400000000002,48.100906479826,0
-0.25385728720408,48.100910640913,0
-0.25371566046983,48.100923092504,0
-0.25357619759542,48.100943739844,0
-0.25343995991491,48.100972425802,0
-0.25330798422265,48.101008932074,0
-0.25318127488449,48.101052980843,0
-0.25306079619532,48.101104236888,0
-0.25294746504153,48.101162310142,0
-0.25284214392366,48.101226758655,0
-0.25274563439305,48.101297091956,0
-0.25265867095151,48.101372774791,0
-0.25258191546123,48.10145323119,0
-0.25251595210701,48.101537848852,0
-0.25246128294952,48.101625983803,0
-0.25241832410293,48.101716965301,0
-0.25238740256661,48.101810100934,0
-0.25236875373478,48.101904681894,0
-0.25236251960311,48.101999988369,0
-0.25400000000002,48.102,0
</coordinates>
</LinearRing>
</outerBoundaryIs>
</Polygon>
</Placemark>
<Placemark>
<name>614</name>
<styleUrl>#stylehap1</styleUrl>
<Polygon>
<outerBoundaryIs>
<LinearRing>
<coordinates>
-0.25400000000002,48.471,0
-0.25235064592713,48.470999988217,0
-0.25235691913126,48.47109529487,0
-0.25237569724317,48.471189876358,0
-0.25240683741825,48.471283012853,0
-0.2524501027249,48.471373995522,0
-0.25250516394583,48.471462131916,0
-0.2525716020818,48.47154675125,0
-0.2526489115389,48.471627209498,0
-0.25273650397534,48.471702894304,0
-0.25283371277807,48.471773229639,0
-0.25293979813549,48.471837680184,0
-0.2530539526679,48.471895755409,0
-0.2531753075721,48.471947013304,0
-0.25330293923417,48.471991063744,0
-0.25343587625961,48.472027571459,0
-0.25357310686754,48.472056258588,0
-0.25371358659233,48.47207690679,0
-0.25385624623456,48.47208935891,0
-0.25400000000002,48.472093520174,0
-0.25414375376549,48.47208935891,0
-0.25428641340769,48.47207690679,0
-0.25442689313251,48.472056258588,0

-0.25456412374041,48.472027571459,0
-0.25469706076588,48.471991063744,0
-0.25482469242792,48.471947013304,0
-0.25494604733212,48.471895755409,0
-0.25506020186453,48.471837680184,0
-0.25516628722198,48.471773229639,0
-0.25526349602468,48.471702894304,0
-0.25535108846115,48.471627209498,0
-0.25542839791825,48.47154675125,0
-0.25549483605419,48.471462131916,0
-0.25554989727513,48.471373995522,0
-0.2555931625818,48.471283012853,0
-0.25562430275688,48.471189876358,0
-0.25564308086879,48.47109529487,0
-0.25564935407289,48.470999988217,0
-0.25564307469663,48.470904681744,0
-0.25562429060009,48.470810100787,0
-0.25559314480978,48.470716965159,0
-0.25554987442788,48.470625983669,0
-0.2554948088259,48.470537848727,0
-0.25542836713621,48.470453231076,0
-0.25535105506067,48.47037277469,0
-0.25526346102064,48.470297091867,0
-0.25516625167793,48.470226758579,0
-0.25506016686049,48.470162310079,0
-0.25494601393167,48.470104236838,0
-0.25482466164591,48.470052980805,0
-0.25469703353756,48.470008932047,0
-0.25456410089316,48.469972425784,0
-0.25442687536048,48.469943739834,0
-0.25428640125093,48.4699230925,0
-0.25414374759333,48.469910640911,0
-0.25400000000002,48.469906479826,0
-0.25385625240672,48.469910640911,0
-0.25371359874912,48.4699230925,0
-0.25357312463954,48.469943739834,0
-0.25343589910689,48.469972425784,0
-0.25330296646247,48.470008932047,0
-0.25317533835414,48.470052980805,0
-0.25305398606838,48.470104236838,0
-0.25293983313956,48.470162310079,0
-0.25283374832209,48.470226758579,0
-0.25273653897938,48.470297091867,0
-0.25264894493936,48.47037277469,0
-0.25257163286382,48.470453231076,0
-0.25250519117415,48.470537848727,0
-0.25245012557217,48.470625983669,0
-0.25240685519025,48.470716965159,0
-0.25237570939994,48.470810100787,0
-0.25235692530342,48.470904681744,0
-0.25235064592713,48.470999988217,0
-0.25400000000002,48.471,0
</coordinates>
</LinearRing>
</outerBoundaryIs>
</Polygon>
</Placemark>

<Placemark>
<name>615</name>
<styleUrl>#stylehap1</styleUrl>
<Polygon>
<outerBoundaryIs>
<LinearRing>
<coordinates>
-0.25199999999995,48.471,0
-0.25035064592706,48.470999988217,0
-0.25035691913119,48.47109529487,0
-0.2503756972431,48.471189876358,0
-0.25040683741818,48.471283012853,0
-0.25045010272483,48.471373995522,0
-0.25050516394576,48.471462131916,0
-0.25057160208173,48.47154675125,0
-0.25064891153883,48.471627209498,0
-0.25073650397527,48.471702894304,0
-0.250833712778,48.471773229639,0
-0.25093979813545,48.471837680184,0
-0.25105395266785,48.471895755409,0
-0.25117530757203,48.471947013304,0
-0.2513029392341,48.471991063744,0
-0.25143587625954,48.472027571459,0
-0.25157310686747,48.472056258588,0
-0.25171358659228,48.47207690679,0
-0.25185624623449,48.47208935891,0
-0.25199999999995,48.472093520174,0
-0.25214375376541,48.47208935891,0
-0.25228641340762,48.47207690679,0
-0.25242689313243,48.472056258588,0
-0.25256412374037,48.472027571459,0
-0.25269706076581,48.471991063744,0
-0.25282469242788,48.471947013304,0
-0.25294604733205,48.471895755409,0
-0.25306020186446,48.471837680184,0
-0.25316628722191,48.471773229639,0
-0.25326349602461,48.471702894304,0
-0.25335108846108,48.471627209498,0
-0.25342839791818,48.47154675125,0
-0.25349483605415,48.471462131916,0
-0.25354989727506,48.471373995522,0
-0.25359316258173,48.471283012853,0
-0.25362430275681,48.471189876358,0
-0.25364308086872,48.47109529487,0
-0.25364935407284,48.470999988217,0
-0.25364307469656,48.470904681744,0
-0.25362429060004,48.470810100787,0
-0.25359314480971,48.470716965159,0
-0.25354987442781,48.470625983669,0
-0.25349480882583,48.470537848727,0
-0.25342836713614,48.470453231076,0
-0.2533510550606,48.47037277469,0
-0.25326346102057,48.470297091867,0
-0.25316625167789,48.470226758579,0
-0.25306016686042,48.470162310079,0
-0.2529460139316,48.470104236838,0
-0.25282466164583,48.470052980805,0

-0.25269703353749,48.470008932047,0
-0.25256410089309,48.469972425784,0
-0.25242687536041,48.469943739834,0
-0.25228640125086,48.4699230925,0
-0.25214374759326,48.469910640911,0
-0.25199999999995,48.469906479826,0
-0.25185625240665,48.469910640911,0
-0.25171359874905,48.4699230925,0
-0.2515731246395,48.469943739834,0
-0.25143589910681,48.469972425784,0
-0.2513029664624,48.470008932047,0
-0.25117533835407,48.470052980805,0
-0.25105398606831,48.470104236838,0
-0.25093983313949,48.470162310079,0
-0.25083374832202,48.470226758579,0
-0.25073653897931,48.470297091867,0
-0.25064894493931,48.47037277469,0
-0.25057163286375,48.470453231076,0
-0.25050519117408,48.470537848727,0
-0.2504501255721,48.470625983669,0
-0.2504068551902,48.470716965159,0
-0.25037570939986,48.470810100787,0
-0.25035692530335,48.470904681744,0
-0.25035064592706,48.470999988217,0
-0.25199999999995,48.471,0
</coordinates>
</LinearRing>
</outerBoundaryIs>
</Polygon>
</Placemark>
<Placemark>
<name>617</name>
<styleUrl>#stylehap1</styleUrl>
<Polygon>
<outerBoundaryIs>
<LinearRing>
<coordinates>
-0.25099999999998,48.447,0
-0.24935142551533,48.446999988227,0
-0.24935769575693,48.447095294879,0
-0.24937646499565,48.447189876367,0
-0.24940759045428,48.447283012863,0
-0.2494508353132,48.447373995531,0
-0.24950587051058,48.447462131925,0
-0.24957227724521,48.447546751257,0
-0.24964955016212,48.447627209504,0
-0.24973710119755,48.44770289431,0
-0.24983426405353,48.447773229643,0
-0.24994029926814,48.447837680188,0
-0.25005439984327,48.447895755412,0
-0.25017569738642,48.447947013307,0
-0.25030326872027,48.447991063746,0
-0.25043614290948,48.448027571461,0
-0.25057330865147,48.448056258589,0
-0.25071372197437,48.44807690679,0
-0.25085631418416,48.44808935891,0
-0.25099999999997,48.448093520174,0

-0.25114368581578,48.44808935891,0
-0.25128627802557,48.44807690679,0
-0.25142669134849,48.448056258589,0
-0.25156385709046,48.448027571461,0
-0.2516967312797,48.447991063746,0
-0.25182430261351,48.447947013307,0
-0.25194560015666,48.447895755412,0
-0.2520597007318,48.447837680188,0
-0.25216573594643,48.447773229643,0
-0.25226289880238,48.44770289431,0
-0.25235044983782,48.447627209504,0
-0.25242772275476,48.447546751257,0
-0.25249412948936,48.447462131925,0
-0.25254916468677,48.447373995531,0
-0.25259240954565,48.447283012863,0
-0.25262353500431,48.447189876367,0
-0.25264230424303,48.447095294879,0
-0.25264857448463,48.446999988227,0
-0.25264229807899,48.446904681754,0
-0.25262352286352,48.446810100797,0
-0.25259239179701,48.446716965169,0
-0.25254914186954,48.446625983678,0
-0.25249410229689,48.446537848735,0
-0.25242769201322,48.446453231084,0
-0.25235041648131,48.446372774696,0
-0.2522628638444,48.446297091873,0
-0.25216570044915,48.446226758584,0
-0.25205966577382,48.446162310083,0
-0.25194556680015,48.446104236841,0
-0.251824271872,48.446052980807,0
-0.2516967040872,48.446008932049,0
-0.25156383427327,48.445972425785,0
-0.25142667359985,48.445943739834,0
-0.25128626588481,48.4459230925,0
-0.25114367965174,48.445910640911,0
-0.25099999999997,48.445906479826,0
-0.2508563203482,48.445910640911,0
-0.25071373411515,48.4459230925,0
-0.25057332640011,48.445943739834,0
-0.25043616572667,48.445972425785,0
-0.25030329591274,48.446008932049,0
-0.25017572812796,48.446052980807,0
-0.25005443319981,48.446104236841,0
-0.24994033422612,48.446162310083,0
-0.24983429955079,48.446226758584,0
-0.24973713615554,48.446297091873,0
-0.24964958351863,48.446372774696,0
-0.24957230798674,48.446453231084,0
-0.24950589770307,48.446537848735,0
-0.24945085813039,48.446625983678,0
-0.24940760820292,48.446716965169,0
-0.24937647713642,48.446810100797,0
-0.24935770192098,48.446904681754,0
-0.24935142551533,48.446999988227,0
-0.25099999999998,48.447,0
</coordinates>
</LinearRing>

</outerBoundaryIs>
</Polygon>
</Placemark>
<Placemark>
<name>619</name>
<styleUrl>#stylehap1</styleUrl>
<Polygon>
<outerBoundaryIs>
<LinearRing>
<coordinates>
-0.24800000000005,48.124,0
-0.24636181838272,48.12399998836,0
-0.24636804913033,48.124095295011,0
-0.24638670007837,48.124189876496,0
-0.24641762934879,48.124283012987,0
-0.24646060161386,48.124373995648,0
-0.24651528988544,48.124462132034,0
-0.24658127800168,48.124546751357,0
-0.2466580637929,48.124627209594,0
-0.24674506290212,48.124702894388,0
-0.24684161323149,48.12477322971,0
-0.24694697998077,48.124837680243,0
-0.24706036123935,48.124895755456,0
-0.24718089408945,48.12494701334,0
-0.24730766117404,48.12499106377,0
-0.24743969767939,48.125027571476,0
-0.24757599867902,48.125056258597,0
-0.24771552678327,48.125076906794,0
-0.2478572200363,48.125089358911,0
-0.24800000000004,48.125093520174,0
-0.24814277996378,48.125089358911,0
-0.24828447321684,48.125076906794,0
-0.24842400132109,48.125056258597,0
-0.24856030232069,48.125027571476,0
-0.24869233882605,48.12499106377,0
-0.24881910591066,48.12494701334,0
-0.24893963876076,48.124895755456,0
-0.24905302001931,48.124837680243,0
-0.24915838676859,48.12477322971,0
-0.24925493709798,48.124702894388,0
-0.24934193620719,48.124627209594,0
-0.2494187219984,48.124546751357,0
-0.24948471011466,48.124462132034,0
-0.24953939838622,48.124373995648,0
-0.24958237065129,48.124283012987,0
-0.24961329992171,48.124189876496,0
-0.24963195086977,48.124095295011,0
-0.24963818161736,48.12399998836,0
-0.24963194481372,48.123904681885,0
-0.24961328799361,48.123810100926,0
-0.24958235321359,48.123716965293,0
-0.24953937596873,48.123625983795,0
-0.24948468339854,48.123537848844,0
-0.24941869179542,48.123453231183,0
-0.24934190343503,48.123372774785,0
-0.24925490275239,48.123297091951,0
-0.24915835189317,48.12322675865,0

-0.24905298567374,48.123162310138,0
-0.24893960598858,48.123104236885,0
-0.24881907570766,48.12305298084,0
-0.24869231210992,48.123008932073,0
-0.2485602799032,48.122972425801,0
-0.24842398388338,48.122943739843,0
-0.24828446128874,48.122923092504,0
-0.24814277390775,48.122910640912,0
-0.24800000000004,48.122906479826,0
-0.24785722609236,48.122910640912,0
-0.24771553871137,48.122923092504,0
-0.2475760161167,48.122943739843,0
-0.24743972009688,48.122972425801,0
-0.24730768789016,48.123008932073,0
-0.24718092429243,48.12305298084,0
-0.2470603940115,48.123104236885,0
-0.24694701432636,48.123162310138,0
-0.24684164810691,48.12322675865,0
-0.24674509724769,48.123297091951,0
-0.24665809656508,48.123372774785,0
-0.24658130820468,48.123453231183,0
-0.24651531660154,48.123537848844,0
-0.24646062403135,48.123625983795,0
-0.2464176467865,48.123716965293,0
-0.24638671200647,48.123810100926,0
-0.24636805518639,48.123904681885,0
-0.24636181838272,48.12399998836,0
-0.24800000000005,48.124,0
</coordinates>
</LinearRing>
</outerBoundaryIs>
</Polygon>
</Placemark>
<Placemark>
<name>620</name>
<styleUrl>#stylehap1</styleUrl>
<Polygon>
<outerBoundaryIs>
<LinearRing>
<coordinates>
-0.24700000000007,48.406,0
-0.24535275494033,48.405999988244,0
-0.24535902012999,48.406095294896,0
-0.24537777423731,48.406189876384,0
-0.24540887460017,48.406283012879,0
-0.24545208458969,48.406373995546,0
-0.24550707540935,48.406462131939,0
-0.24557342859556,48.40654675127,0
-0.24565063920079,48.406627209516,0
-0.24573811963547,48.40670289432,0
-0.24583520413884,48.406773229652,0
-0.24594115384535,48.406837680195,0
-0.24605516240763,48.406895755418,0
-0.24617636213348,48.406947013311,0
-0.24630383059012,48.406991063749,0
-0.2464365976253,48.407027571462,0
-0.24657365275198,48.40705625859,0

-0.24671395284031,48.40707690679,0
-0.24685643005819,48.40708935891,0
-0.24700000000008,48.407093520174,0
-0.24714356994195,48.40708935891,0
-0.24728604715983,48.40707690679,0
-0.24742634724816,48.40705625859,0
-0.24756340237484,48.407027571462,0
-0.24769616941001,48.406991063749,0
-0.24782363786666,48.406947013311,0
-0.24794483759251,48.406895755418,0
-0.24805884615478,48.406837680195,0
-0.24816479586129,48.406773229652,0
-0.24826188036466,48.40670289432,0
-0.24834936079935,48.406627209516,0
-0.24842657140458,48.40654675127,0
-0.24849292459078,48.406462131939,0
-0.24854791541047,48.406373995546,0
-0.24859112539997,48.406283012879,0
-0.24862222576283,48.406189876384,0
-0.24864097987015,48.406095294896,0
-0.24864724505983,48.405999988244,0
-0.24864097371997,48.40590468177,0
-0.24862221364931,48.405810100813,0
-0.2485911076912,48.405716965185,0
-0.24854789264449,48.405625983693,0
-0.24849289745938,48.405537848749,0
-0.24842654073208,48.405453231096,0
-0.24834932751774,48.405372774708,0
-0.2482618454852,48.405297091883,0
-0.24816476044376,48.405226758592,0
-0.24805881127532,48.40516231009,0
-0.2479448043109,48.405104236847,0
-0.24782360719415,48.405052980811,0
-0.24769614227859,48.405008932052,0
-0.24756337960889,48.404972425787,0
-0.24742632953939,48.404943739835,0
-0.24728603504632,48.404923092501,0
-0.24714356379175,48.404910640912,0
-0.24700000000008,48.404906479826,0
-0.24685643620839,48.404910640912,0
-0.24671396495382,48.404923092501,0
-0.24657367046074,48.404943739835,0
-0.24643662039125,48.404972425787,0
-0.24630385772155,48.405008932052,0
-0.24617639280599,48.405052980811,0
-0.24605519568923,48.405104236847,0
-0.24594118872482,48.40516231009,0
-0.24583523955638,48.405226758592,0
-0.24573815451494,48.405297091883,0
-0.24565067248242,48.405372774708,0
-0.24557345926806,48.405453231096,0
-0.24550710254078,48.405537848749,0
-0.24545210735564,48.405625983693,0
-0.24540889230894,48.405716965185,0
-0.24537778635085,48.405810100813,0
-0.24535902628016,48.40590468177,0
-0.24535275494033,48.405999988244,0

-0.24700000000007,48.406,0
</coordinates>
</LinearRing>
</outerBoundaryIs>
</Polygon>
</Placemark>
<Placemark>
<name>624</name>
<styleUrl>#stylehap1</styleUrl>
<Polygon>
<outerBoundaryIs>
<LinearRing>
<coordinates>
-0.24099999999999,48.431,0
-0.23935194467094,48.430999988234,0
-0.2393582129397,48.431095294886,0
-0.23937697626944,48.431189876374,0
-0.23940809192789,48.431283012869,0
-0.2394513231699,48.431373995536,0
-0.23950634103728,48.43146213193,0
-0.23957272686063,48.431546751262,0
-0.23964997544413,48.431627209509,0
-0.23973749890917,48.431702894314,0
-0.23983463116756,48.431773229647,0
-0.23994063299028,48.431837680191,0
-0.24005469763334,48.431895755415,0
-0.24017595697772,48.431947013308,0
-0.24030348813684,48.431991063747,0
-0.24043632048117,48.432027571461,0
-0.24057344302662,48.432056258589,0
-0.24071381213014,48.43207690679,0
-0.24085635943427,48.43208935891,0
-0.241,48.432093520173,0
-0.2411436405657,48.43208935891,0
-0.24128618786985,48.43207690679,0
-0.24142655697334,48.432056258589,0
-0.2415636795188,48.432027571461,0
-0.24169651186315,48.431991063747,0
-0.24182404302227,48.431947013308,0
-0.24194530236662,48.431895755415,0
-0.24205936700971,48.431837680191,0
-0.24216536883243,48.431773229647,0
-0.24226250109082,48.431702894314,0
-0.24235002455583,48.431627209509,0
-0.24242727313933,48.431546751262,0
-0.24249365896268,48.43146213193,0
-0.24254867683006,48.431373995536,0
-0.24259190807208,48.431283012869,0
-0.24262302373053,48.431189876374,0
-0.24264178706029,48.431095294886,0
-0.24264805532903,48.430999988234,0
-0.24264178090164,48.43090468176,0
-0.2426230116004,48.430810100803,0
-0.24259189033901,48.430716965175,0
-0.24254865403289,48.430625983684,0
-0.24249363179405,48.43053784874,0
-0.24242724242477,48.430453231089,0

-0.24234999122858,48.430372774701,0
-0.24226246616349,48.430297091877,0
-0.2421653333663,48.430226758587,0
-0.24205933208239,48.430162310086,0
-0.24194526903937,48.430104236843,0
-0.24182401230767,48.430052980809,0
-0.2416964846945,48.43000893205,0
-0.2415636567216,48.429972425786,0
-0.2414265392403,48.429943739835,0
-0.2412861757397,48.4299230925,0
-0.24114363440708,48.429910640912,0
-0.241,48.429906479826,0
-0.24085636559289,48.429910640912,0
-0.24071382426027,48.4299230925,0
-0.24057346075969,48.429943739835,0
-0.24043634327837,48.429972425786,0
-0.24030351530547,48.43000893205,0
-0.24017598769229,48.430052980809,0
-0.24005473096059,48.430104236843,0
-0.2399406679176,48.430162310086,0
-0.23983466663367,48.430226758587,0
-0.23973753383647,48.430297091877,0
-0.23965000877139,48.430372774701,0
-0.2395727575752,48.430453231089,0
-0.23950636820591,48.43053784874,0
-0.23945134596708,48.430625983684,0
-0.23940810966096,48.430716965175,0
-0.23937698839957,48.430810100803,0
-0.23935821909833,48.43090468176,0
-0.23935194467094,48.430999988234,0
-0.24099999999999,48.431,0
</coordinates>
</LinearRing>
</outerBoundaryIs>
</Polygon>
</Placemark>
<Placemark>
<name>627</name>
<styleUrl>#stylehap1</styleUrl>
<Polygon>
<outerBoundaryIs>
<LinearRing>
<coordinates>
-0.23000000000002,48.44,0
-0.22835165270199,48.43999998823,0
-0.22835792208026,48.440095294882,0
-0.22837668873316,48.44018987637,0
-0.22840780990318,48.440283012865,0
-0.22845104880321,48.440373995533,0
-0.22850607641685,48.440462131927,0
-0.22857247400055,48.440546751259,0
-0.22864973626896,48.440627209506,0
-0.22873727523938,48.440702894311,0
-0.22883442470558,48.440773229645,0
-0.22894044530765,48.440837680189,0
-0.22905453015863,48.440895755413,0
-0.22917581098568,48.440947013307,0

-0.22930336473874,48.440991063746,0
-0.22943622061632,48.441027571461,0
-0.22957336745513,48.441056258589,0
-0.22971376142728,48.44107690679,0
-0.229856333986,48.44108935891,0
-0.23000000000001,48.441093520174,0
-0.23014366601402,48.44108935891,0
-0.23028623857274,48.44107690679,0
-0.23042663254489,48.441056258589,0
-0.23056377938373,48.441027571461,0
-0.23069663526131,48.440991063746,0
-0.23082418901435,48.440947013307,0
-0.23094546984142,48.440895755413,0
-0.2310595546924,48.440837680189,0
-0.23116557529444,48.440773229645,0
-0.23126272476067,48.440702894311,0
-0.23135026373107,48.440627209506,0
-0.2314275259995,48.440546751259,0
-0.23149392358317,48.440462131927,0
-0.23154895119681,48.440373995533,0
-0.23159219009686,48.440283012865,0
-0.23162331126686,48.44018987637,0
-0.23164207791976,48.440095294882,0
-0.23164834729804,48.43999998823,0
-0.23164207175809,48.439904681757,0
-0.23162329913075,48.4398101008,0
-0.23159217235504,48.439716965171,0
-0.23154892838836,48.43962598368,0
-0.23149389640113,48.439537848737,0
-0.23142749526977,48.439453231086,0
-0.23135023038735,48.439372774698,0
-0.23126268981609,48.439297091875,0
-0.2311655398108,48.439226758585,0
-0.23105951974782,48.439162310085,0
-0.2309454364977,48.439104236842,0
-0.23082415828462,48.439052980808,0
-0.23069660807925,48.439008932049,0
-0.23056375657528,48.438972425785,0
-0.23042661480307,48.438943739834,0
-0.23028622643664,48.4389230925,0
-0.23014365985234,48.438910640911,0
-0.23000000000001,48.438906479826,0
-0.22985634014768,48.438910640911,0
-0.22971377356341,48.4389230925,0
-0.22957338519695,48.438943739834,0
-0.22943624342477,48.438972425785,0
-0.22930339192078,48.439008932049,0
-0.22917584171541,48.439052980808,0
-0.22905456350234,48.439104236842,0
-0.22894048025222,48.439162310085,0
-0.22883446018922,48.439226758585,0
-0.22873731018393,48.439297091875,0
-0.22864976961267,48.439372774698,0
-0.22857250473028,48.439453231086,0
-0.22850610359892,48.439537848737,0
-0.22845107161166,48.43962598368,0
-0.22840782764501,48.439716965171,0

-0.2283767008693,48.4398101008,0
-0.22835792824194,48.439904681757,0
-0.22835165270199,48.43999998823,0
-0.23000000000002,48.44,0
</coordinates>
</LinearRing>
</outerBoundaryIs>
</Polygon>
</Placemark>
<Placemark>
<name>629</name>
<styleUrl>#stylehap1</styleUrl>
<Polygon>
<outerBoundaryIs>
<LinearRing>
<coordinates>
-0.22000000000003,48.401,0
-0.21835291686092,48.400999988246,0
-0.21835918143526,48.401095294898,0
-0.21837793369964,48.401189876386,0
-0.21840903100588,48.401283012881,0
-0.21845223674838,48.401373995547,0
-0.21850722216298,48.40146213194,0
-0.21857356882711,48.401546751271,0
-0.21865077184293,48.401627209517,0
-0.21873824367861,48.401702894321,0
-0.21883531863884,48.401773229653,0
-0.21894125793067,48.401837680196,0
-0.21905525528602,48.401895755419,0
-0.21917644309797,48.401947013311,0
-0.21930389902442,48.401991063749,0
-0.21943665300848,48.402027571463,0
-0.21957369466251,48.40205625859,0
-0.21971398095916,48.40207690679,0
-0.21985644417131,48.40208935891,0
-0.22000000000004,48.402093520174,0
-0.22014355582874,48.40208935891,0
-0.22028601904091,48.40207690679,0
-0.22042630533754,48.40205625859,0
-0.22056334699157,48.402027571463,0
-0.22069610097563,48.401991063749,0
-0.22082355690208,48.401947013311,0
-0.22094474471403,48.401895755419,0
-0.22105874206938,48.401837680196,0
-0.22116468136123,48.401773229653,0
-0.22126175632144,48.401702894321,0
-0.22134922815712,48.401627209517,0
-0.22142643117294,48.401546751271,0
-0.2214927778371,48.40146213194,0
-0.22154776325167,48.401373995547,0
-0.22159096899419,48.401283012881,0
-0.22162206630041,48.401189876386,0
-0.22164081856479,48.401095294898,0
-0.22164708313915,48.400999988246,0
-0.22164081241629,48.400904681773,0
-0.22162205419023,48.400810100815,0
-0.22159095129026,48.400716965186,0

-0.22154774049195,48.400625983695,0
-0.2214927507131,48.400537848751,0
-0.22142640050886,48.400453231098,0
-0.22134919488462,48.400372774709,0
-0.22126172145151,48.400297091884,0
-0.22116464595339,48.400226758593,0
-0.22105870719946,48.400162310091,0
-0.22094471144156,48.400104236848,0
-0.220823526238,48.400052980812,0
-0.22069607385166,48.400008932052,0
-0.22056332423185,48.399972425787,0
-0.22042628763363,48.399943739835,0
-0.22028600693071,48.399923092501,0
-0.22014354968025,48.399910640912,0
-0.22000000000004,48.399906479826,0
-0.21985645031983,48.399910640912,0
-0.21971399306934,48.399923092501,0
-0.21957371236642,48.399943739835,0
-0.21943667576822,48.399972425787,0
-0.21930392614839,48.400008932052,0
-0.21917647376205,48.400052980812,0
-0.21905528855851,48.400104236848,0
-0.21894129280059,48.400162310091,0
-0.21883535404669,48.400226758593,0
-0.21873827854854,48.400297091884,0
-0.21865080511543,48.400372774709,0
-0.21857359949119,48.400453231098,0
-0.21850724928695,48.400537848751,0
-0.21845225950812,48.400625983695,0
-0.21840904870979,48.400716965186,0
-0.21837794580985,48.400810100815,0
-0.21835918758378,48.400904681773,0
-0.21835291686092,48.400999988246,0
-0.22000000000003,48.401,0
</coordinates>
</LinearRing>
</outerBoundaryIs>
</Polygon>
</Placemark>
<Placemark>
<name>630</name>
<styleUrl>#stylehap1</styleUrl>
<Polygon>
<outerBoundaryIs>
<LinearRing>
<coordinates>
-0.21199999999999,48.438,0
-0.21035171759642,48.437999988231,0
-0.21035798672808,48.438095294883,0
-0.21037675264236,48.438189876371,0
-0.21040787258733,48.438283012866,0
-0.21045110978528,48.438373995534,0
-0.21050613523266,48.438462131928,0
-0.21057253020244,48.43854675126,0
-0.21064978942918,48.438627209507,0
-0.21073732495328,48.438702894312,0
-0.2108344705948,48.438773229645,0

-0.21094048702288,48.43883768019,0
-0.21105456738234,48.438895755414,0
-0.21117584343456,48.438947013307,0
-0.21130339216578,48.438991063746,0
-0.21143624281275,48.439027571461,0
-0.21157338425199,48.439056258589,0
-0.21171377269672,48.43907690679,0
-0.21185633964225,48.43908935891,0
-0.21199999999998,48.439093520174,0
-0.21214366035774,48.43908935891,0
-0.21228622730324,48.43907690679,0
-0.21242661574797,48.439056258589,0
-0.21256375718724,48.439027571461,0
-0.21269660783419,48.438991063746,0
-0.21282415656541,48.438947013307,0
-0.21294543261763,48.438895755414,0
-0.21305951297711,48.43883768019,0
-0.21316552940517,48.438773229645,0
-0.21326267504669,48.438702894312,0
-0.21335021057079,48.438627209507,0
-0.21342746979754,48.43854675126,0
-0.2134938647673,48.438462131928,0
-0.21354889021471,48.438373995534,0
-0.21359212741263,48.438283012866,0
-0.2136232473576,48.438189876371,0
-0.21364201327188,48.438095294883,0
-0.21364828240357,48.437999988231,0
-0.21364200711089,48.437904681757,0
-0.21362323522282,48.4378101008,0
-0.21359210967277,48.437716965172,0
-0.21354886740876,48.437625983681,0
-0.21349383758824,48.437537848738,0
-0.21342743907117,48.437453231086,0
-0.21335017723074,48.437372774699,0
-0.21326264010595,48.437297091875,0
-0.21316549392542,48.437226758585,0
-0.21305947803637,48.437162310085,0
-0.21294539927758,48.437104236843,0
-0.21282412583906,48.437052980808,0
-0.21269658065513,48.437008932049,0
-0.21256373438129,48.436972425786,0
-0.21242659800811,48.436943739834,0
-0.21228621516846,48.4369230925,0
-0.21214365419673,48.436910640911,0
-0.21199999999998,48.436906479826,0
-0.21185634580324,48.436910640911,0
-0.21171378483153,48.4369230925,0
-0.21157340199188,48.436943739834,0
-0.21143626561867,48.436972425786,0
-0.21130341934484,48.437008932049,0
-0.21117587416093,48.437052980808,0
-0.21105460072241,48.437104236843,0
-0.21094052196362,48.437162310085,0
-0.21083450607455,48.437226758585,0
-0.21073735989401,48.437297091875,0
-0.21064982276923,48.437372774699,0
-0.21057256092879,48.437453231086,0

-0.21050616241172,48.437537848738,0
-0.21045113259121,48.437625983681,0
-0.21040789032722,48.437716965172,0
-0.21037676477714,48.4378101008,0
-0.21035799288907,48.437904681757,0
-0.21035171759642,48.437999988231,0
-0.21199999999999,48.438,0
</coordinates>
</LinearRing>
</outerBoundaryIs>
</Polygon>
</Placemark>
<Placemark>
<name>631</name>
<styleUrl>#stylehap1</styleUrl>
<Polygon>
<outerBoundaryIs>
<LinearRing>
<coordinates>
-0.20500000000004,48.48,0
-0.2033503533168,48.479999988213,0
-0.20335662763288,48.480095294866,0
-0.20337540907523,48.480189876354,0
-0.20340655477397,48.48028301285,0
-0.2034498277555,48.480373995519,0
-0.20350489874409,48.480462131913,0
-0.20357134866621,48.480546751247,0
-0.2036486718383,48.480627209495,0
-0.20373627981419,48.480702894302,0
-0.20383350586254,48.480773229637,0
-0.20393961004058,48.480837680182,0
-0.20405378482529,48.480895755408,0
-0.20417516125935,48.480947013303,0
-0.20430281556498,48.480991063743,0
-0.20443577617536,48.481027571459,0
-0.20457303113003,48.481056258588,0
-0.2047135357781,48.48107690679,0
-0.2048562207304,48.48108935891,0
-0.20500000000004,48.481093520173,0
-0.20514377926971,48.48108935891,0
-0.20528646422198,48.48107690679,0
-0.20542696887005,48.481056258588,0
-0.20556422382472,48.481027571459,0
-0.20569718443509,48.480991063743,0
-0.20582483874073,48.480947013303,0
-0.20594621517479,48.480895755408,0
-0.20606038995952,48.480837680182,0
-0.20616649413753,48.480773229637,0
-0.20626372018589,48.480702894302,0
-0.20635132816178,48.480627209495,0
-0.20642865133387,48.480546751247,0
-0.20649510125598,48.480462131913,0
-0.20655017224458,48.480373995519,0
-0.20659344522611,48.48028301285,0
-0.20662459092484,48.480189876354,0
-0.2066433723672,48.480095294866,0
-0.20664964668327,48.479999988213,0

-0.20664336619201,48.47990468174,0
-0.20662457876207,48.479810100784,0
-0.2065934274453,48.479716965156,0
-0.20655014938603,48.479625983666,0
-0.20649507401423,48.479537848724,0
-0.20642862053664,48.479453231073,0
-0.20635129474481,48.479372774687,0
-0.20626368516455,48.479297091865,0
-0.20616645857595,48.479226758577,0
-0.20606035493818,48.479162310078,0
-0.20594618175782,48.479104236837,0
-0.2058248079435,48.479052980804,0
-0.20569715719334,48.479008932046,0
-0.20556420096618,48.478972425784,0
-0.20542695108925,48.478943739833,0
-0.20528645205918,48.4789230925,0
-0.20514377309449,48.478910640911,0
-0.20500000000004,48.478906479826,0
-0.20485622690558,48.478910640911,0
-0.20471354794089,48.4789230925,0
-0.20457304891083,48.478943739833,0
-0.2044357990339,48.478972425784,0
-0.20430284280674,48.479008932046,0
-0.20417519205658,48.479052980804,0
-0.20405381824225,48.479104236837,0
-0.2039396450619,48.479162310078,0
-0.20383354142412,48.479226758577,0
-0.20373631483553,48.479297091865,0
-0.20364870525526,48.479372774687,0
-0.20357137946344,48.479453231073,0
-0.20350492598585,48.479537848724,0
-0.20344985061404,48.479625983666,0
-0.20340657255477,48.479716965156,0
-0.20337542123803,48.479810100784,0
-0.20335663380809,48.47990468174,0
-0.2033503533168,48.479999988213,0
-0.20500000000004,48.48,0
</coordinates>
</LinearRing>
</outerBoundaryIs>
</Polygon>
</Placemark>
<Placemark>
<name>632</name>
<styleUrl>#stylehap1</styleUrl>
<Polygon>
<outerBoundaryIs>
<LinearRing>
<coordinates>
-0.197,48.152,0
-0.19536092470158,48.151999988349,0
-0.19536715884529,48.152095295,0
-0.19538581996515,48.152189876485,0
-0.19541676610576,48.152283012976,0
-0.19545976181114,48.152373995638,0
-0.19551447991482,48.152462132024,0
-0.19558050402799,48.152546751348,0

-0.19565733170709,48.152627209586,0
-0.19574437827635,48.152702894381,0
-0.19584098127682,48.152773229704,0
-0.19594640550733,48.152837680238,0
-0.19605984861983,48.152895755452,0
-0.19618044722576,48.152947013337,0
-0.19630728346761,48.152991063767,0
-0.19643939200528,48.153027571475,0
-0.19657576736394,48.153056258597,0
-0.196715371588,48.153076906794,0
-0.1968571421422,48.153089358911,0
-0.19700000000001,48.153093520174,0
-0.19714285785782,48.153089358911,0
-0.197284628412,48.153076906794,0
-0.19742423263608,48.153056258597,0
-0.19756060799474,48.153027571475,0
-0.19769271653238,48.152991063767,0
-0.19781955277426,48.152947013337,0
-0.19794015138019,48.152895755452,0
-0.19805359449269,48.152837680238,0
-0.1981590187232,48.152773229704,0
-0.19825562172364,48.152702894381,0
-0.19834266829293,48.152627209586,0
-0.198419495972,48.152546751348,0
-0.19848552008519,48.152462132024,0
-0.19854023818886,48.152373995638,0
-0.19858323389423,48.152283012976,0
-0.19861418003487,48.152189876485,0
-0.19863284115473,48.152095295,0
-0.19863907529841,48.151999988349,0
-0.19863283508941,48.151904681874,0
-0.19861416808853,48.151810100915,0
-0.19858321642986,48.151716965282,0
-0.19854021573709,48.151625983785,0
-0.19848549332821,48.151537848835,0
-0.19841946572282,48.151453231175,0
-0.19834263547062,48.151372774778,0
-0.19825558732554,48.151297091944,0
-0.19815898379442,48.151226758644,0
-0.19805356009456,48.151162310134,0
-0.19794011855788,48.151104236881,0
-0.19781952252505,48.151052980837,0
-0.19769268977541,48.151008932071,0
-0.19756058554295,48.150972425799,0
-0.19742421517168,48.150943739842,0
-0.19728461646565,48.150923092504,0
-0.1971428517925,48.150910640912,0
-0.19700000000001,48.150906479827,0
-0.19685714820751,48.150910640912,0
-0.19671538353434,48.150923092504,0
-0.19657578482831,48.150943739842,0
-0.19643941445704,48.150972425799,0
-0.1963073102246,48.151008932071,0
-0.19618047747494,48.151052980837,0
-0.19605988144211,48.151104236881,0
-0.19594643990546,48.151162310134,0
-0.19584101620557,48.151226758644,0

-0.19574441267448,48.151297091944,0
-0.19565736452939,48.151372774778,0
-0.1955805342772,48.151453231175,0
-0.19551450667181,48.151537848835,0
-0.19545978426293,48.151625983785,0
-0.19541678357013,48.151716965282,0
-0.19538583191149,48.151810100915,0
-0.1953671649106,48.151904681874,0
-0.19536092470158,48.151999988349,0
-0.197,48.152,0
</coordinates>
</LinearRing>
</outerBoundaryIs>
</Polygon>
</Placemark>
<Placemark>
<name>635</name>
<styleUrl>#stylehap1</styleUrl>
<Polygon>
<outerBoundaryIs>
<LinearRing>
<coordinates>
-0.18600000000004,48.409,0
-0.18435265776659,48.408999988243,0
-0.18435892332553,48.409095294895,0
-0.18437767853889,48.409189876383,0
-0.18440878073609,48.409283012877,0
-0.18445199327438,48.409373995545,0
-0.18450698733783,48.409462131938,0
-0.18457334443812,48.409546751269,0
-0.18465055959799,48.409627209515,0
-0.18473804519321,48.409702894319,0
-0.18483513542372,48.409773229651,0
-0.1849410913804,48.409837680194,0
-0.18505510666833,48.409895755418,0
-0.18517631354409,48.40994701331,0
-0.18530378952048,48.409991063749,0
-0.18543656438804,48.410027571462,0
-0.18557362760009,48.41005625859,0
-0.18571393596524,48.41007690679,0
-0.18585642158842,48.41008935891,0
-0.18600000000002,48.410093520174,0
-0.18614357841166,48.41008935891,0
-0.18628606403483,48.41007690679,0
-0.18642637239998,48.41005625859,0
-0.18656343561204,48.410027571462,0
-0.1866962104796,48.409991063749,0
-0.18682368645598,48.40994701331,0
-0.18694489333175,48.409895755418,0
-0.18705890861967,48.409837680194,0
-0.18716486457636,48.409773229651,0
-0.18726195480686,48.409702894319,0
-0.18734944040208,48.409627209515,0
-0.18742665556195,48.409546751269,0
-0.18749301266224,48.409462131938,0
-0.18754800672569,48.409373995545,0
-0.18759121926398,48.409283012877,0

-0.18762232146118,48.409189876383,0
-0.18764107667455,48.409095294895,0
-0.18764734223348,48.408999988243,0
-0.18764107052333,48.408904681769,0
-0.18762230934568,48.408810100812,0
-0.18759120155229,48.408716965183,0
-0.187547983956,48.408625983692,0
-0.18749298552636,48.408537848748,0
-0.18742662488441,48.408453231095,0
-0.18734940711498,48.408372774707,0
-0.18726191992165,48.408297091882,0
-0.18716482915297,48.408226758591,0
-0.18705887373446,48.40816231009,0
-0.18694486004467,48.408104236846,0
-0.18682365577844,48.408052980811,0
-0.18669618334371,48.408008932052,0
-0.18656341284234,48.407972425787,0
-0.18642635468831,48.407943739835,0
-0.18628605191931,48.407923092501,0
-0.18614357226047,48.407910640912,0
-0.18600000000002,48.407906479827,0
-0.18585642773961,48.407910640912,0
-0.18571394808077,48.407923092501,0
-0.18557364531176,48.407943739835,0
-0.18543658715773,48.407972425787,0
-0.18530381665636,48.408008932052,0
-0.18517634422163,48.408052980811,0
-0.1850551399554,48.408104236846,0
-0.18494112626561,48.40816231009,0
-0.18483517084711,48.408226758591,0
-0.18473808007842,48.408297091882,0
-0.18465059288509,48.408372774707,0
-0.18457337511567,48.408453231095,0
-0.18450701447371,48.408537848748,0
-0.18445201604408,48.408625983692,0
-0.18440879844778,48.408716965183,0
-0.18437769065439,48.408810100812,0
-0.18435892947675,48.408904681769,0
-0.18435265776659,48.408999988243,0
-0.18600000000004,48.409,0
</coordinates>
</LinearRing>
</outerBoundaryIs>
</Polygon>
</Placemark>
<Placemark>
<name>636</name>
<styleUrl>#stylehap1</styleUrl>
<Polygon>
<outerBoundaryIs>
<LinearRing>
<coordinates>
-0.18499999999995,48.388,0
-0.18335333764689,48.387999988252,0
-0.18335960062219,48.388095294904,0
-0.18337834809724,48.388189876391,0
-0.18340943746022,48.388283012886,0

-0.18345263216595,48.388373995552,0
-0.18350760353421,48.388462131945,0
-0.18357393324934,48.388546751275,0
-0.18365111654247,48.388627209521,0
-0.18373856603175,48.388702894324,0
-0.18383561619203,48.388773229656,0
-0.18394152841906,48.388837680198,0
-0.18405549665073,48.38889575542,0
-0.18417665350184,48.388947013313,0
-0.18430407686587,48.38899106375,0
-0.18443679693382,48.389027571463,0
-0.18457380357618,48.38905625859,0
-0.18471405403211,48.389076906791,0
-0.18485648084733,48.38908935891,0
-0.18499999999994,48.389093520174,0
-0.18514351915255,48.38908935891,0
-0.18528594596777,48.389076906791,0
-0.18542619642372,48.38905625859,0
-0.18556320306608,48.389027571463,0
-0.18569592313403,48.38899106375,0
-0.18582334649806,48.388947013313,0
-0.18594450334914,48.38889575542,0
-0.18605847158084,48.388837680198,0
-0.18616438380787,48.388773229656,0
-0.18626143396812,48.388702894324,0
-0.18634888345743,48.388627209521,0
-0.18642606675053,48.388546751275,0
-0.18649239646567,48.388462131945,0
-0.18654736783395,48.388373995552,0
-0.18659056253968,48.388283012886,0
-0.18662165190266,48.388189876391,0
-0.18664039937768,48.388095294904,0
-0.18664666235301,48.387999988252,0
-0.18664039323356,48.387904681778,0
-0.18662163980108,48.387810100082,0
-0.18659054484837,48.387716965191,0
-0.18654734509041,48.387625983699,0
-0.18649236936101,48.387537848755,0
-0.18642603610828,48.387453231102,0
-0.18634885020865,48.387372774713,0
-0.18626139912304,48.387297091887,0
-0.18616434842525,48.387226758596,0
-0.18605843673575,48.387162310094,0
-0.18594447010036,48.387104236849,0
-0.18582331585581,48.387052980813,0
-0.18569589602936,48.387008932053,0
-0.18556318032257,48.386972425788,0
-0.18542617873241,48.386943739836,0
-0.18528593386622,48.386923092501,0
-0.18514351300843,48.386910640912,0
-0.18499999999994,48.386906479826,0
-0.18485648699147,48.386910640912,0
-0.18471406613368,48.386923092501,0
-0.18457382126749,48.386943739836,0
-0.18443681967733,48.386972425788,0
-0.18430410397054,48.387008932053,0
-0.18417668414409,48.387052980813,0

-0.18405552989954,48.387104236849,0
-0.18394156326415,48.387162310094,0
-0.18383565157465,48.387226758596,0
-0.18373860087684,48.387297091887,0
-0.18365114979125,48.387372774713,0
-0.18357396389159,48.387453231102,0
-0.18350763063887,48.387537848755,0
-0.18345265490946,48.387625983699,0
-0.18340945515153,48.387716965191,0
-0.1833783601988,48.38781010082,0
-0.18335960676634,48.387904681778,0
-0.18335333764689,48.387999988252,0
-0.18499999999995,48.388,0
</coordinates>
</LinearRing>
</outerBoundaryIs>
</Polygon>
</Placemark>
<Placemark>
<name>637</name>
<styleUrl>#stylehap1</styleUrl>
<Polygon>
<outerBoundaryIs>
<LinearRing>
<coordinates>
-0.18299999999999,48.372,0
-0.18135385512609,48.371999988258,0
-0.18136011613495,48.37209529491,0
-0.18137885772007,48.372189876398,0
-0.18140993731449,48.372283012892,0
-0.18145311844733,48.372373995558,0
-0.18150807254153,48.37246213195,0
-0.18157438141294,48.37254675128,0
-0.1816515404512,48.372627209525,0
-0.18173896245912,48.372702894328,0
-0.1818359821206,48.372773229659,0
-0.18194186106358,48.372837680201,0
-0.18205579347921,48.372895755423,0
-0.18217691225488,48.372947013314,0
-0.18230429557395,48.372991063751,0
-0.18243697393213,48.373027571464,0
-0.18257393751743,48.373056258591,0
-0.18271414389677,48.373076906791,0
-0.18285652595137,48.37308935891,0
-0.18299999999999,48.373093520174,0
-0.18314347404862,48.37308935891,0
-0.18328585610321,48.373076906791,0
-0.18342606248256,48.373056258591,0
-0.18356302606786,48.373027571464,0
-0.18369570442604,48.372991063751,0
-0.18382308774508,48.372947013314,0
-0.18394420652078,48.372895755423,0
-0.1840581389364,48.372837680201,0
-0.18416401787939,48.372773229659,0
-0.18426103754087,48.372702894328,0
-0.18434845954878,48.372627209525,0
-0.18442561858705,48.37254675128,0

-0.18449192745846,48.37246213195,0
-0.18454688155266,48.372373995558,0
-0.1845900626855,48.372283012892,0
-0.18462114227992,48.372189876398,0
-0.18463988386504,48.37209529491,0
-0.1846461448739,48.371999988258,0
-0.18463987772629,48.371904681784,0
-0.18462113018895,48.371810100827,0
-0.18459004500968,48.371716965198,0
-0.18454685882907,48.371625983705,0
-0.18449190037754,48.371537848761,0
-0.18442558797167,48.371453231107,0
-0.18434842632914,48.371372774717,0
-0.18426100272631,48.371297091891,0
-0.18416398252775,48.371226758599,0
-0.18405810412187,48.371162310096,0
-0.18394417330111,48.371104236852,0
-0.1838230571297,48.371052980815,0
-0.18369567734514,48.371008932054,0
-0.18356300334427,48.370972425789,0
-0.18342604480674,48.370943739836,0
-0.18328584401225,48.370923092501,0
-0.18314346790987,48.370910640912,0
-0.18299999999999,48.370906479827,0
-0.18285653209012,48.370910640912,0
-0.18271415598774,48.370923092501,0
-0.18257395519325,48.370943739836,0
-0.18243699665572,48.370972425789,0
-0.18230432265485,48.371008932054,0
-0.18217694287029,48.371052980815,0
-0.18205582669888,48.371104236852,0
-0.18194189587811,48.371162310096,0
-0.18183601747223,48.371226758599,0
-0.18173899727367,48.371297091891,0
-0.18165157367085,48.371372774717,0
-0.18157441202832,48.371453231107,0
-0.18150809962245,48.371537848761,0
-0.18145314117091,48.371625983705,0
-0.18140995499031,48.371716965198,0
-0.18137886981104,48.371810100827,0
-0.1813601222737,48.371904681784,0
-0.18135385512609,48.371999988258,0
-0.18299999999999,48.372,0
</coordinates>
</LinearRing>
</outerBoundaryIs>
</Polygon>
</Placemark>
<Placemark>
<name>638</name>
<styleUrl>#stylehap1</styleUrl>
<Polygon>
<outerBoundaryIs>
<LinearRing>
<coordinates>
-0.16499999999996,48.448,0
-0.16335139305296,48.447999988227,0

-0.16335766341791,48.448095294879,0
-0.16337643302611,48.448189876367,0
-0.16340755909757,48.448283012862,0
-0.16345080480793,48.44837399553,0
-0.16350584108894,48.448462131924,0
-0.16357224913114,48.448546751257,0
-0.1636495235696,48.448627209504,0
-0.163737076329,48.448702894309,0
-0.16383424109819,48.448773229643,0
-0.16394027840077,48.448837680188,0
-0.16405438122272,48.448895755412,0
-0.16417568115439,48.448947013306,0
-0.16430325500032,48.448991063746,0
-0.16443613180605,48.44902757146,0
-0.1645733002491,48.449056258588,0
-0.16471371633699,48.44907690679,0
-0.16485631135471,48.44908935891,0
-0.16499999999996,48.449093520173,0
-0.16514368864522,48.44908935891,0
-0.16528628366294,48.44907690679,0
-0.16542669975083,48.449056258588,0
-0.16556386819385,48.44902757146,0
-0.16569674499961,48.448991063746,0
-0.16582431884553,48.448947013306,0
-0.1659456187772,48.448895755412,0
-0.16605972159916,48.448837680188,0
-0.16616575890174,48.448773229643,0
-0.16626292367093,48.448702894309,0
-0.16635047643033,48.448627209504,0
-0.16642775086879,48.448546751257,0
-0.16649415891099,48.448462131924,0
-0.166549195192,48.44837399553,0
-0.16659244090236,48.448283012862,0
-0.16662356697381,48.448189876367,0
-0.16664233658201,48.448095294879,0
-0.16664860694697,48.447999988227,0
-0.16664233041764,48.447904681753,0
-0.16662355483237,48.447810100796,0
-0.16659242315275,48.447716965168,0
-0.16654917237355,48.447625983678,0
-0.16649413171702,48.447537848735,0
-0.16642772012558,48.447453231083,0
-0.16635044307199,48.447372774696,0
-0.16626288871104,48.447297091873,0
-0.16616572340252,48.447226758583,0
-0.16605968663924,48.447162310083,0
-0.16594558541886,48.447104236841,0
-0.16582428810232,48.447052980807,0
-0.16569671780564,48.447008932049,0
-0.16556384537541,48.446972425785,0
-0.16542668200122,48.446943739834,0
-0.16528627152149,48.4469230925,0
-0.16514368248085,48.446910640911,0
-0.16499999999996,48.446906479826,0
-0.16485631751908,48.446910640911,0
-0.16471372847844,48.4469230925,0
-0.16457331799871,48.446943739834,0

-0.16443615462452,48.446972425785,0
-0.16430328219429,48.447008932049,0
-0.16417571189761,48.447052980807,0
-0.16405441458107,48.447104236841,0
-0.16394031336069,48.447162310083,0
-0.16383427659741,48.447226758583,0
-0.16373711128889,48.447297091873,0
-0.16364955692794,48.447372774696,0
-0.16357227987435,48.447453231083,0
-0.16350586828291,48.447537848735,0
-0.16345082762638,48.447625983678,0
-0.16340757684718,48.447716965168,0
-0.16337644516756,48.447810100796,0
-0.16335766958229,48.447904681753,0
-0.16335139305296,48.447999988227,0
-0.16499999999996,48.448,0
</coordinates>
</LinearRing>
</outerBoundaryIs>
</Polygon>
</Placemark>
<Placemark>
<name>640</name>
<styleUrl>#stylehap1</styleUrl>
<Polygon>
<outerBoundaryIs>
<LinearRing>
<coordinates>
-0.13,48.389,0
-0.12835330528943,48.388999988251,0
-0.12835956838771,48.389095294903,0
-0.12837831623102,48.389189876391,0
-0.12840940620481,48.389283012885,0
-0.12845260175927,48.389373995552,0
-0.12850757420767,48.389462131944,0
-0.12857390522614,48.389546751275,0
-0.12865109003588,48.38962720952,0
-0.12873854124355,48.389702894324,0
-0.12883559331089,48.389773229655,0
-0.12894150761915,48.389837680198,0
-0.12905547809037,48.38989575542,0
-0.12917663732229,48.389947013313,0
-0.12930406319031,48.38999106375,0
-0.12943678586631,48.390027571463,0
-0.12957379520099,48.39005625859,0
-0.12971404841301,48.390076906791,0
-0.12985647802707,48.39008935891,0
-0.12999999999999,48.390093520174,0
-0.13014352197291,48.39008935891,0
-0.13028595158697,48.390076906791,0
-0.13042620479899,48.39005625859,0
-0.13056321413367,48.390027571463,0
-0.1306959368097,48.38999106375,0
-0.1308233626777,48.389947013313,0
-0.13094452190962,48.38989575542,0
-0.13105849238083,48.389837680198,0
-0.13116440668909,48.389773229655,0

-0.13126145875643,48.389702894324,0
-0.13134890996413,48.38962720952,0
-0.13142609477387,48.389546751275,0
-0.13149242579234,48.389462131944,0
-0.13154739824071,48.389373995552,0
-0.13159059379518,48.389283012885,0
-0.13162168376897,48.389189876391,0
-0.1316404316123,48.389095294903,0
-0.13164669471058,48.388999988251,0
-0.13164042546783,48.388904681777,0
-0.13162167166673,48.38881010082,0
-0.1315905761029,48.388716965191,0
-0.13154737549596,48.388625983699,0
-0.13149239868618,48.388537848755,0
-0.13142606412994,48.388453231102,0
-0.13134887671352,48.388372774712,0
-0.13126142390943,48.388297091887,0
-0.13116437130453,48.388226758596,0
-0.13105845753386,48.388162310093,0
-0.13094448865901,48.388104236849,0
-0.13082333203377,48.388052980813,0
-0.13069590970354,48.388008932053,0
-0.13056319138892,48.387972425788,0
-0.13042618710671,48.387943739836,0
-0.13028593948476,48.387923092501,0
-0.13014351582844,48.387910640912,0
-0.12999999999999,48.387906479826,0
-0.12985648417155,48.387910640912,0
-0.12971406051525,48.387923092501,0
-0.12957381289327,48.387943739836,0
-0.12943680861109,48.387972425788,0
-0.12930409029644,48.388008932053,0
-0.12917666796622,48.388052980813,0
-0.12905551134098,48.388104236849,0
-0.12894154246615,48.388162310093,0
-0.12883562869545,48.388226758596,0
-0.12873857609055,48.388297091887,0
-0.12865112328649,48.388372774712,0
-0.12857393587007,48.388453231102,0
-0.1285076013138,48.388537848755,0
-0.12845262450403,48.388625983699,0
-0.12840942389709,48.388716965191,0
-0.12837832833326,48.38881010082,0
-0.12835957453216,48.388904681777,0
-0.12835330528943,48.388999988251,0
-0.13,48.389,0
</coordinates>
</LinearRing>
</outerBoundaryIs>
</Polygon>
</Placemark>
</Folder>
</Folder>
<Folder>
<name>hapgroup1</name>
<Folder>
<name>hap1</name>

<Placemark>
<name>1</name>
<styleUrl>#stylehap2</styleUrl>
<Polygon>
<outerBoundaryIs>
<LinearRing>
<coordinates>
-0.69100000000003,48.276,0
-0.68935695049679,48.275999988298,0
-0.68936319974291,48.276095294949,0
-0.68938190609683,48.276189876436,0
-0.68941292725934,48.276283012929,0
-0.68945602720389,48.276373995593,0
-0.68951087797108,48.276462131983,0
-0.68957706216284,48.27654675131,0
-0.68965407611751,48.276627209552,0
-0.68974133374191,48.276702894351,0
-0.6898381709709,48.276773229679,0
-0.68994385082078,48.276837680217,0
-0.69005756899801,48.276895755436,0
-0.69017846002052,48.276947013324,0
-0.69030560380512,48.276991063758,0
-0.69043803267085,48.277027571469,0
-0.69057473870468,48.277056258593,0
-0.69071468143397,48.277076906792,0
-0.69085679574683,48.27708935891,0
-0.69100000000004,48.277093520174,0
-0.69114320425324,48.27708935891,0
-0.69128531856609,48.277076906792,0
-0.69142526129538,48.277056258593,0
-0.69156196732922,48.277027571469,0
-0.69169439619494,48.276991063758,0
-0.69182153997954,48.276947013324,0
-0.69194243100206,48.276895755436,0
-0.69205614917928,48.276837680217,0
-0.69216182902916,48.276773229679,0
-0.69225866625815,48.276702894351,0
-0.69234592388256,48.276627209552,0
-0.69242293783723,48.27654675131,0
-0.69248912202898,48.276462131983,0
-0.69254397279617,48.276373995593,0
-0.69258707274072,48.276283012929,0
-0.69261809390323,48.276189876436,0
-0.69263680025715,48.276095294949,0
-0.69264304950327,48.275999988298,0
-0.69263679415059,48.275904681824,0
-0.69261808187565,48.275810100865,0
-0.69258705515757,48.275716965235,0
-0.69254395019172,48.27562598374,0
-0.69248909509001,48.275537848793,0
-0.69242290738232,48.275453231137,0
-0.69234589083705,48.275372774744,0
-0.6922586316261,48.275297091914,0
-0.69216179386286,48.275226758619,0
-0.69205611454724,48.275162310113,0
-0.69194239795655,48.275104236865,0
-0.69182150952464,48.275052980825,0

-0.69169436925598,48.275008932061,0
-0.69156194472476,48.274972425793,0
-0.69142524371224,48.274943739839,0
-0.6912853065385,48.274923092502,0
-0.69114319814667,48.274910640912,0
-0.69100000000004,48.274906479826,0
-0.69085680185339,48.274910640912,0
-0.69071469346156,48.274923092502,0
-0.69057475628783,48.274943739839,0
-0.6904380552753,48.274972425793,0
-0.69030563074409,48.275008932061,0
-0.69017849047542,48.275052980825,0
-0.69005760204351,48.275104236865,0
-0.68994388545282,48.275162310113,0
-0.6898382061372,48.275226758619,0
-0.68974136837396,48.275297091914,0
-0.68965410916301,48.275372774744,0
-0.68957709261774,48.275453231137,0
-0.68951090491005,48.275537848793,0
-0.68945604980835,48.27562598374,0
-0.68941294484249,48.275716965235,0
-0.68938191812441,48.275810100865,0
-0.68936320584948,48.275904681824,0
-0.68935695049679,48.275999988298,0
-0.69100000000003,48.276,0
</coordinates>
</LinearRing>
</outerBoundaryIs>
</Polygon>
</Placemark>
<Placemark>
<name>2</name>
<styleUrl>#stylehap2</styleUrl>
<Polygon>
<outerBoundaryIs>
<LinearRing>
<coordinates>
-0.598999999999993,48.197,0
-0.59735948556231,48.19699998833,0
-0.59736572517489,48.197095294982,0
-0.59738440267494,48.197189876467,0
-0.59741537598247,48.197283012959,0
-0.59745840943495,48.197373995622,0
-0.59751317557876,48.197462132009,0
-0.59757925765959,48.197546751334,0
-0.59765615279281,48.197627209573,0
-0.59774327578938,48.19770289437,0
-0.59783996360868,48.197773229695,0
-0.59794548040409,48.197837680231,0
-0.59805902312301,48.197895755446,0
-0.59817972761884,48.197947013332,0
-0.5983066752281,48.197991063764,0
-0.59843889976296,48.198027571473,0
-0.59857539486574,48.198056258595,0
-0.59871512166934,48.198076906793,0
-0.59885701670542,48.19808935891,0
-0.598999999999993,48.198093520174,0

-0.59914298329446,48.19808935891,0
-0.59928487833054,48.198076906793,0
-0.59942460513412,48.198056258595,0
-0.59956110023689,48.198027571473,0
-0.59969332477175,48.197991063764,0
-0.59982027238101,48.197947013332,0
-0.59994097687684,48.197895755446,0
-0.60005451959579,48.197837680231,0
-0.6001600363912,48.197773229695,0
-0.6002567242105,48.19770289437,0
-0.60034384720707,48.197627209573,0
-0.60042074234026,48.197546751334,0
-0.6004868244211,48.197462132009,0
-0.6005415905649,48.197373995622,0
-0.60058462401738,48.197283012959,0
-0.60061559732494,48.197189876467,0
-0.60063427482496,48.197095294982,0
-0.60064051443754,48.19699998833,0
-0.60063426874471,48.196904681856,0
-0.60061558534918,48.196810100897,0
-0.60058460651003,48.196716965265,0
-0.60054156805787,48.196625983769,0
-0.60048679759826,48.19653784882,0
-0.60042071201662,48.196453231161,0
-0.60034381430398,48.196372774765,0
-0.60025668972772,48.196297091933,0
-0.60016000137645,48.196226758635,0
-0.60005448511301,48.196162310126,0
-0.59994094397375,48.196104236875,0
-0.59982024205738,48.196052980833,0
-0.59969329794891,48.196008932067,0
-0.59956107772986,48.195972425797,0
-0.59942458762676,48.195943739841,0
-0.59928486635478,48.195923092503,0
-0.59914297721421,48.195910640912,0
-0.59899999999993,48.195906479826,0
-0.59885702278564,48.195910640912,0
-0.59871513364507,48.195923092503,0
-0.59857541237311,48.195943739841,0
-0.59843892227002,48.195972425797,0
-0.59830670205094,48.196008932067,0
-0.5981797579425,48.196052980833,0
-0.5980590560261,48.196104236875,0
-0.59794551488685,48.196162310126,0
-0.5978399986234,48.196226758635,0
-0.59774331027216,48.196297091933,0
-0.5976561856959,48.196372774765,0
-0.59757928798326,48.196453231161,0
-0.5975132024016,48.19653784882,0
-0.59745843194198,48.196625983769,0
-0.59741539348985,48.196716965265,0
-0.59738441465067,48.196810100897,0
-0.59736573125514,48.196904681856,0
-0.59735948556231,48.19699998833,0
-0.59899999999993,48.197,0
</coordinates>
</LinearRing>

</outerBoundaryIs>
</Polygon>
</Placemark>
<Placemark>
<name>3</name>
<styleUrl>#stylehap2</styleUrl>
<Polygon>
<outerBoundaryIs>
<LinearRing>
<coordinates>
-0.59199999999998,48.206,0
-0.59035919730971,48.205999988326,0
-0.59036543801768,48.206095294978,0
-0.59038411879858,48.206189876464,0
-0.59041509754754,48.206283012955,0
-0.59045813856058,48.206373995618,0
-0.59051291432658,48.206462132006,0
-0.59057900801806,48.206546751331,0
-0.590655916662,48.206627209571,0
-0.5907430549666,48.206702894368,0
-0.59083975977469,48.206773229693,0
-0.5909452951104,48.206837680229,0
-0.59105885778005,48.206895755445,0
-0.59117958348512,48.206947013331,0
-0.59130655340073,48.206991063764,0
-0.59143880116928,48.207027571472,0
-0.59157532025622,48.207056258595,0
-0.5917150716119,48.207076906793,0
-0.5918569915811,48.20708935891,0
-0.59199999999998,48.207093520174,0
-0.59214300841889,48.20708935891,0
-0.59228492838807,48.207076906793,0
-0.59242467974375,48.207056258595,0
-0.59256119883071,48.207027571472,0
-0.59269344659924,48.206991063764,0
-0.59282041651485,48.206947013331,0
-0.59294114221992,48.206895755445,0
-0.59305470488957,48.206837680229,0
-0.59316024022528,48.206773229693,0
-0.59325694503337,48.206702894368,0
-0.59334408333797,48.206627209571,0
-0.5934209919819,48.206546751331,0
-0.59348708567339,48.206462132006,0
-0.59354186143939,48.206373995618,0
-0.59358490245243,48.206283012955,0
-0.59361588120138,48.206189876464,0
-0.59363456198229,48.206095294978,0
-0.59364080269025,48.205999988326,0
-0.59363455589906,48.205904681852,0
-0.59361586921975,48.205810100893,0
-0.59358488493645,48.205716965261,0
-0.59354183892129,48.205625983766,0
-0.59348705883735,48.205537848817,0
-0.59342096164333,48.205453231158,0
-0.59334405041869,48.205372774763,0
-0.59325691053364,48.205297091931,0
-0.59316020519332,48.205226758633,0

-0.59305467038981,48.205162310124,0
-0.59294110930067,48.205104236874,0
-0.5928203861763,48.205052980832,0
-0.5926934197632,48.205008932067,0
-0.59256117631259,48.204972425797,0
-0.59242466222777,48.204943739841,0
-0.59228491640644,48.204923092503,0
-0.59214300233564,48.204910640912,0
-0.59199999999998,48.204906479826,0
-0.59185699766433,48.204910640912,0
-0.59171508359353,48.204923092503,0
-0.59157533777219,48.204943739841,0
-0.59143882368738,48.204972425797,0
-0.59130658023677,48.205008932067,0
-0.59117961382369,48.205052980832,0
-0.59105889069933,48.205104236874,0
-0.59094532961015,48.205162310124,0
-0.59083979480664,48.205226758633,0
-0.59074308946633,48.205297091931,0
-0.59065594958127,48.205372774763,0
-0.59057903835664,48.205453231158,0
-0.59051294116262,48.205537848817,0
-0.59045816107868,48.205625983766,0
-0.59041511506351,48.205716965261,0
-0.59038413078022,48.205810100893,0
-0.59036544410093,48.205904681852,0
-0.59035919730971,48.205999988326,0
-0.59199999999998,48.206,0
</coordinates>
</LinearRing>
</outerBoundaryIs>
</Polygon>
</Placemark>
<Placemark>
<name>4</name>
<styleUrl>#stylehap2</styleUrl>
<Polygon>
<outerBoundaryIs>
<LinearRing>
<coordinates>
-0.58799999999997,48.219,0
-0.58635878069434,48.218999988321,0
-0.5863650229855,48.219095294973,0
-0.58638370850829,48.219189876459,0
-0.58641469512176,48.21928301295,0
-0.58645774706219,48.219373995614,0
-0.58651253673531,48.219462132002,0
-0.58657864720778,48.219546751327,0
-0.58665557537898,48.219627209567,0
-0.58674273580843,48.219702894365,0
-0.58683946517069,48.21977322969,0
-0.58694502730295,48.219837680227,0
-0.58705861880758,48.219895755443,0
-0.58717937516663,48.21994701333,0
-0.58730637732192,48.219991063763,0
-0.58743865867042,48.220027571472,0
-0.58757521242199,48.220056258595,0

-0.58771499926312,48.220076906793,0
-0.58785695526849,48.22008935891,0
-0.58799999999997,48.220093520174,0
-0.58814304473145,48.22008935891,0
-0.58828500073682,48.220076906793,0
-0.58842478757795,48.220056258595,0
-0.58856134132952,48.220027571472,0
-0.58869362267802,48.219991063763,0
-0.58882062483328,48.21994701333,0
-0.58894138119235,48.219895755443,0
-0.58905497269699,48.219837680227,0
-0.58916053482923,48.21977322969,0
-0.58925726419148,48.219702894365,0
-0.58934442462096,48.219627209567,0
-0.58942135279213,48.219546751327,0
-0.58948746326463,48.219462132002,0
-0.58954225293774,48.219373995614,0
-0.58958530487815,48.21928301295,0
-0.58961629149165,48.219189876459,0
-0.58963497701441,48.219095294973,0
-0.58964121930558,48.218999988321,0
-0.58963497092685,48.218904681847,0
-0.58961627950149,48.218810100888,0
-0.58958528734973,48.218716965256,0
-0.58954223040361,48.218625983761,0
-0.58948743640953,48.218537848812,0
-0.589421322432,48.218453231154,0
-0.58934439167828,48.218372774759,0
-0.58925722966722,48.218297091928,0
-0.58916049977239,48.218226758631,0
-0.58905493817271,48.218162310122,0
-0.58894134824967,48.218104236872,0
-0.58882059447315,48.218052980831,0
-0.58869359582291,48.218008932066,0
-0.58856131879542,48.217972425796,0
-0.58842477004953,48.217943739841,0
-0.58828498874666,48.217923092503,0
-0.58814303864389,48.217910640912,0
-0.58799999999997,48.217906479827,0
-0.58785696135605,48.217910640912,0
-0.58771501125327,48.217923092503,0
-0.58757522995041,48.217943739841,0
-0.58743868120452,48.217972425796,0
-0.58730640417702,48.218008932066,0
-0.58717940552678,48.218052980831,0
-0.58705865175024,48.218104236872,0
-0.5869450618272,48.218162310122,0
-0.58683950022755,48.218226758631,0
-0.58674277033272,48.218297091928,0
-0.58665560832166,48.218372774759,0
-0.58657867756793,48.218453231154,0
-0.58651256359041,48.218537848812,0
-0.5864577695963,48.218625983761,0
-0.5864147126502,48.218716965256,0
-0.58638372049844,48.218810100888,0
-0.58636502907306,48.218904681847,0
-0.58635878069434,48.218999988321,0

-0.58799999999997,48.219,0
</coordinates>
</LinearRing>
</outerBoundaryIs>
</Polygon>
</Placemark>
<Placemark>
<name>5</name>
<styleUrl>#stylehap2</styleUrl>
<Polygon>
<outerBoundaryIs>
<LinearRing>
<coordinates>
-0.58299999999997,48.211,0
-0.58135903710806,48.210999988324,0
-0.58136527842481,48.211095294976,0
-0.58138396102911,48.211189876462,0
-0.58141494280221,48.211283012954,0
-0.58145798801718,48.211373995617,0
-0.58151276913091,48.211462132004,0
-0.58157886927521,48.21154675133,0
-0.581655785428,48.21162720957,0
-0.58174293224028,48.211702894367,0
-0.58183964649024,48.211773229692,0
-0.58194519213005,48.211837680228,0
-0.58205876588765,48.211895755444,0
-0.58217950338014,48.211947013331,0
-0.58230648569291,48.211991063763,0
-0.58243874637398,48.212027571472,0
-0.58257527879057,48.212056258595,0
-0.58271504379152,48.212076906793,0
-0.58285697761774,48.21208935891,0
-0.58299999999997,48.212093520174,0
-0.5831430223822,48.21208935891,0
-0.58328495620842,48.212076906793,0
-0.58342472120937,48.212056258595,0
-0.58356125362595,48.212027571472,0
-0.58369351430703,48.211991063763,0
-0.5838204966198,48.211947013331,0
-0.58394123411228,48.211895755444,0
-0.58405480786989,48.211837680228,0
-0.58416035350972,48.211773229692,0
-0.58425706775966,48.211702894367,0
-0.58434421457194,48.21162720957,0
-0.58442113072473,48.21154675133,0
-0.58448723086903,48.211462132004,0
-0.58454201198276,48.211373995617,0
-0.58458505719772,48.211283012954,0
-0.58461603897083,48.211189876462,0
-0.58463472157513,48.211095294976,0
-0.58464096289188,48.210999988324,0
-0.58463471549022,48.21090468185,0
-0.58461602698591,48.210810100891,0
-0.58458503967694,48.210716965259,0
-0.58454198945848,48.210625983764,0
-0.58448720402566,48.210537848815,0
-0.58442110037786,48.210453231157,0

-0.58434418164368,48.210372774761,0
-0.58425703325049,48.21029709193,0
-0.58416031846818,48.210226758632,0
-0.58405477336071,48.210162310124,0
-0.58394120118403,48.210104236873,0
-0.58382046627296,48.210052980831,0
-0.58369348746365,48.210008932066,0
-0.5835612311017,48.209972425797,0
-0.58342470368861,48.209943739841,0
-0.5832849442235,48.209923092503,0
-0.58314301629729,48.209910640912,0
-0.58299999999997,48.209906479827,0
-0.58285698370265,48.209910640912,0
-0.58271505577643,48.209923092503,0
-0.58257529631133,48.209943739841,0
-0.58243876889827,48.209972425797,0
-0.58230651253628,48.210008932066,0
-0.582179533727,48.210052980831,0
-0.58205879881591,48.210104236873,0
-0.58194522663922,48.210162310124,0
-0.58183968153176,48.210226758632,0
-0.58174296674945,48.21029709193,0
-0.58165581835626,48.210372774761,0
-0.58157889962207,48.210453231157,0
-0.58151279597428,48.210537848815,0
-0.58145801054146,48.210625983764,0
-0.581414960323,48.210716965259,0
-0.58138397301403,48.210810100891,0
-0.58136528450972,48.21090468185,0
-0.58135903710806,48.210999988324,0
-0.58299999999997,48.211,0
</coordinates>
</LinearRing>
</outerBoundaryIs>
</Polygon>
</Placemark>
<Placemark>
<name>6</name>
<styleUrl>#stylehap2</styleUrl>
<Polygon>
<outerBoundaryIs>
<LinearRing>
<coordinates>
-0.58100000000002,48.209,0
-0.579359101194,48.208999988325,0
-0.57936534226725,48.209095294977,0
-0.57938402414211,48.209189876463,0
-0.57941500470546,48.209283012954,0
-0.57945804823953,48.209373995617,0
-0.57951282721397,48.209462132005,0
-0.57957892477693,48.209546751331,0
-0.57965583792593,48.20962720957,0
-0.57974298133487,48.209702894367,0
-0.57983969180778,48.209773229693,0
-0.57994523332562,48.209837680228,0
-0.58005880264767,48.209895755445,0
-0.58017953542479,48.209947013331,0

-0.58030651277829,48.209991063763,0
-0.58043876829394,48.210027571472,0
-0.58057529537821,48.210056258595,0
-0.58071505492061,48.210076906793,0
-0.58085698320359,48.21008935891,0
-0.58100000000002,48.210093520174,0
-0.58114301679644,48.21008935891,0
-0.58128494507941,48.210076906793,0
-0.58142470462181,48.210056258595,0
-0.58156123170608,48.210027571472,0
-0.58169348722173,48.209991063763,0
-0.58182046457524,48.209947013331,0
-0.58194119735238,48.209895755445,0
-0.58205476667443,48.209837680228,0
-0.58216030819227,48.209773229693,0
-0.58225701866516,48.209702894367,0
-0.58234416207409,48.20962720957,0
-0.58242107522309,48.209546751331,0
-0.58248717278605,48.209462132005,0
-0.58254195176052,48.209373995617,0
-0.58258499529457,48.209283012954,0
-0.58261597585791,48.209189876463,0
-0.5826346577328,48.209095294977,0
-0.58264089880602,48.208999988325,0
-0.58263465164855,48.208904681851,0
-0.58261596387432,48.208810100892,0
-0.58258497777572,48.20871696526,0
-0.5825419292387,48.208625983764,0
-0.58248714594563,48.208537848816,0
-0.58242104487956,48.208453231157,0
-0.58234412914942,48.208372774762,0
-0.58225698415975,48.208297091931,0
-0.58216027315457,48.208226758633,0
-0.58205473216903,48.208162310124,0
-0.58194116442771,48.208104236874,0
-0.5818204342317,48.208052980832,0
-0.58169346038131,48.208008932066,0
-0.5815612091843,48.207972425797,0
-0.58142468710296,48.207943739841,0
-0.58128493309582,48.207923092503,0
-0.58114301071222,48.207910640912,0
-0.58100000000002,48.207906479826,0
-0.58085698928781,48.207910640912,0
-0.58071506690421,48.207923092503,0
-0.58057531289706,48.207943739841,0
-0.58043879081575,48.207972425797,0
-0.58030653961874,48.208008932066,0
-0.58017956576832,48.208052980832,0
-0.58005883557234,48.208104236874,0
-0.57994526783102,48.208162310124,0
-0.57983972684548,48.208226758633,0
-0.57974301584027,48.208297091931,0
-0.5796558708506,48.208372774762,0
-0.57957895512049,48.208453231157,0
-0.57951285405442,48.208537848816,0
-0.57945807076132,48.208625983764,0
-0.57941502222431,48.20871696526,0

-0.5793840361257,48.208810100892,0
-0.57936534835147,48.208904681851,0
-0.579359101194,48.208999988325,0
-0.58100000000002,48.209,0
</coordinates>
</LinearRing>
</outerBoundaryIs>
</Polygon>
</Placemark>
<Placemark>
<name>7</name>
<styleUrl>#stylehap2</styleUrl>
<Polygon>
<outerBoundaryIs>
<LinearRing>
<coordinates>
-0.57899999999995,48.235,0
-0.5773582675304,48.234999988315,0
-0.57736451177165,48.235095294966,0
-0.57738320313521,48.235189876452,0
-0.57741419943581,48.235283012944,0
-0.57745726483597,48.235373995608,0
-0.57751207163907,48.235462131996,0
-0.57757820278151,48.235546751323,0
-0.57765515500528,48.235627209563,0
-0.57774234268695,48.235702894361,0
-0.57783910229366,48.235773229687,0
-0.57794469743241,48.235837680224,0
-0.57805832445441,48.235895755441,0
-0.57817911857137,48.235947013328,0
-0.5783061604377,48.235991063761,0
-0.57843848314813,48.236027571471,0
-0.57857507959768,48.236056258594,0
-0.57871491014788,48.236076906793,0
-0.57885691054064,48.23608935891,0
-0.57899999999995,48.236093520174,0
-0.57914308945927,48.23608935891,0
-0.57928508985203,48.236076906793,0
-0.5794249204022,48.236056258594,0
-0.57956151685177,48.236027571471,0
-0.57969383956219,48.235991063761,0
-0.57982088142853,48.235947013328,0
-0.57994167554547,48.235895755441,0
-0.58005530256749,48.235837680224,0
-0.58016089770625,48.235773229687,0
-0.58025765731296,48.235702894361,0
-0.58034484499463,48.235627209563,0
-0.5804217972184,48.235546751323,0
-0.58048792836084,48.235462131996,0
-0.58054273516394,48.235373995608,0
-0.5805858005641,48.235283012944,0
-0.58061679686468,48.235189876452,0
-0.58063548822825,48.235095294966,0
-0.58064173246948,48.234999988315,0
-0.58063548213535,48.23490468184,0
-0.58061678486404,48.234810100882,0
-0.58058578302034,48.23471696525,0

-0.58054271261009,48.234625983755,0
-0.58048790148223,48.234537848807,0
-0.58042176683171,48.234453231149,0
-0.58034481202312,48.234372774755,0
-0.58025762275847,48.234297091924,0
-0.5801608626187,48.234226758627,0
-0.58005526801301,48.23416231012,0
-0.57994164257399,48.23410423687,0
-0.57982085104182,48.234052980829,0
-0.57969381268357,48.234008932064,0
-0.57956149429793,48.233972425795,0
-0.57942490285844,48.23394373984,0
-0.57928507785139,48.233923092503,0
-0.57914308336639,48.233910640912,0
-0.57899999999995,48.233906479827,0
-0.57885691663351,48.233910640912,0
-0.57871492214852,48.233923092503,0
-0.57857509714147,48.23394373984,0
-0.57843850570198,48.233972425795,0
-0.57830618731631,48.234008932064,0
-0.57817914895809,48.234052980829,0
-0.57805835742592,48.23410423687,0
-0.5779447319869,48.23416231012,0
-0.57783913738118,48.234226758627,0
-0.57774237724144,48.234297091924,0
-0.57765518797677,48.234372774755,0
-0.5775782331682,48.234453231149,0
-0.57751209851768,48.234537848807,0
-0.57745728738979,48.234625983755,0
-0.57741421697957,48.23471696525,0
-0.57738321513585,48.234810100882,0
-0.57736451786453,48.23490468184,0
-0.5773582675304,48.234999988315,0
-0.57899999999995,48.235,0
</coordinates>
</LinearRing>
</outerBoundaryIs>
</Polygon>
</Placemark>
<Placemark>
<name>8</name>
<styleUrl>#stylehap2</styleUrl>
<Polygon>
<outerBoundaryIs>
<LinearRing>
<coordinates>
-0.577,48.224,0
-0.57535862037888,48.223999988319,0
-0.57536486327926,48.224095294971,0
-0.57538355062671,48.224189876457,0
-0.57541454026652,48.224283012949,0
-0.57545759641185,48.224373995612,0
-0.57551239143647,48.224462132,0
-0.57557850836636,48.224546751326,0
-0.57565544405173,48.224627209566,0
-0.57574261299494,48.224702894364,0
-0.57583935180576,48.224773229689,0

-0.57594492424943,48.224837680226,0
-0.5760585268499,48.224895755443,0
-0.57617929500476,48.224947013329,0
-0.57630630956602,48.224991063762,0
-0.57643860383622,48.225027571471,0
-0.57657517092691,48.225056258595,0
-0.57671497142302,48.225076906793,0
-0.57685694129526,48.22508935891,0
-0.57700000000001,48.225093520174,0
-0.57714305870473,48.22508935891,0
-0.577285028577,48.225076906793,0
-0.57742482907308,48.225056258595,0
-0.57756139616377,48.225027571471,0
-0.57769369043397,48.224991063762,0
-0.57782070499523,48.224947013329,0
-0.57794147315009,48.224895755443,0
-0.57805507575057,48.224837680226,0
-0.57816064819424,48.224773229689,0
-0.57825738700505,48.224702894364,0
-0.57834455594827,48.224627209566,0
-0.57842149163363,48.224546751326,0
-0.57848760856353,48.224462132,0
-0.57854240358817,48.224373995612,0
-0.57858545973347,48.224283012949,0
-0.57861644937328,48.224189876457,0
-0.57863513672073,48.224095294971,0
-0.57864137962112,48.223999988319,0
-0.57863513063152,48.223904681845,0
-0.57861643737984,48.223810100886,0
-0.57858544220027,48.223716965254,0
-0.57854238104788,48.223625983759,0
-0.57848758170107,48.223537848811,0
-0.57842146126519,48.223453231153,0
-0.57834452299658,48.223372774758,0
-0.57825735247135,48.223297091927,0
-0.57816061312781,48.22322675863,0
-0.57805504121684,48.223162310121,0
-0.5779414401984,48.223104236872,0
-0.57782067462679,48.22305298083,0
-0.57769366357152,48.223008932065,0
-0.57756137362349,48.222972425796,0
-0.57742481153988,48.22294373984,0
-0.57728501658356,48.222923092503,0
-0.5771430526155,48.222910640912,0
-0.57700000000001,48.222906479826,0
-0.5768569473845,48.222910640912,0
-0.57671498341644,48.222923092503,0
-0.57657518846014,48.22294373984,0
-0.57643862637651,48.222972425796,0
-0.57630633642848,48.223008932065,0
-0.57617932537321,48.22305298083,0
-0.57605855980159,48.223104236872,0
-0.57594495878315,48.223162310121,0
-0.57583938687219,48.22322675863,0
-0.57574264752864,48.223297091927,0
-0.57565547700342,48.223372774758,0
-0.5755785387348,48.223453231153,0

-0.57551241829892,48.223537848811,0
-0.57545761895211,48.223625983759,0
-0.57541455779972,48.223716965254,0
-0.57538356262015,48.223810100886,0
-0.57536486936847,48.223904681845,0
-0.57535862037888,48.223999988319,0
-0.577,48.224,0
</coordinates>
</LinearRing>
</outerBoundaryIs>
</Polygon>
</Placemark>
<Placemark>
<name>9</name>
<styleUrl>#stylehap2</styleUrl>
<Polygon>
<outerBoundaryIs>
<LinearRing>
<coordinates>
-0.57100000000003,48.23,0
-0.56935842794243,48.229999988317,0
-0.56936467157409,48.230095294968,0
-0.56938336111185,48.230189876454,0
-0.56941435438431,48.230283012946,0
-0.56945741557706,48.23037399561,0
-0.56951221702542,48.230462131998,0
-0.56957834170656,48.230546751324,0
-0.56965528641162,48.230627209564,0
-0.56974246557442,48.230702894362,0
-0.56983921572689,48.230773229688,0
-0.56994480054803,48.230837680225,0
-0.57005841646752,48.230895755442,0
-0.57017919878157,48.230947013329,0
-0.57030622823447,48.230991063762,0
-0.57043853801542,48.231027571471,0
-0.57057512111784,48.231056258594,0
-0.57071493800482,48.231076906793,0
-0.57085692452235,48.23108935891,0
-0.57100000000003,48.231093520174,0
-0.5711430754777,48.23108935891,0
-0.57128506199523,48.231076906793,0
-0.57142487888221,48.231056258594,0
-0.57156146198463,48.231027571471,0
-0.57169377176558,48.230991063762,0
-0.57182080121848,48.230947013329,0
-0.57194158353253,48.230895755442,0
-0.57205519945202,48.230837680225,0
-0.57216078427316,48.230773229688,0
-0.57225753442563,48.230702894362,0
-0.57234471358843,48.230627209564,0
-0.57242165829349,48.230546751324,0
-0.57248778297463,48.230462131998,0
-0.57254258442299,48.23037399561,0
-0.57258564561574,48.230283012946,0
-0.5726166388882,48.230189876454,0
-0.57263532842596,48.230095294968,0
-0.57264157205762,48.229999988317,0

-0.57263532233474,48.229904681842,0
-0.57261662689084,48.229810100884,0
-0.57258562807676,48.229716965252,0
-0.57254256187533,48.229625983757,0
-0.57248775610337,48.229537848808,0
-0.5724216279151,48.229453231151,0
-0.57234468062593,48.229372774756,0
-0.57225749988059,48.229297091926,0
-0.57216074919521,48.229226758628,0
-0.572055164907,48.22916231012,0
-0.57194155057005,48.229104236871,0
-0.57182077084008,48.229052980829,0
-0.57169374489432,48.229008932065,0
-0.57156143943697,48.228972425796,0
-0.57142486134324,48.22894373984,0
-0.57128504999787,48.228923092503,0
-0.57114306938648,48.228910640912,0
-0.57100000000003,48.228906479826,0
-0.57085693061357,48.228910640912,0
-0.57071495000221,48.228923092503,0
-0.57057513865681,48.22894373984,0
-0.57043856056309,48.228972425796,0
-0.57030625510573,48.229008932065,0
-0.57017922915997,48.229052980829,0
-0.57005844943,48.229104236871,0
-0.56994483509305,48.22916231012,0
-0.56983925080484,48.229226758628,0
-0.56974250011946,48.229297091926,0
-0.56965531937413,48.229372774756,0
-0.56957837208495,48.229453231151,0
-0.56951224389668,48.229537848808,0
-0.56945743812472,48.229625983757,0
-0.56941437192329,48.229716965252,0
-0.56938337310923,48.229810100884,0
-0.56936467766531,48.229904681842,0
-0.56935842794243,48.229999988317,0
-0.57100000000003,48.23,0
</coordinates>
</LinearRing>
</outerBoundaryIs>
</Polygon>
</Placemark>
<Placemark>
<name>10</name>
<styleUrl>#stylehap2</styleUrl>
<Polygon>
<outerBoundaryIs>
<LinearRing>
<coordinates>
-0.56999999999994,48.242,0
-0.56835804287997,48.241999988312,0
-0.56836428797494,48.242095294963,0
-0.56838298189543,48.242189876449,0
-0.5684139824368,48.242283012942,0
-0.56845705372931,48.242373995605,0
-0.56851186803149,48.242462131994,0
-0.56857800822272,48.24254675132,0

-0.56865497097611,48.242627209561,0
-0.56874217058811,48.242702894359,0
-0.56883894343509,48.242773229686,0
-0.56894455302328,48.242837680223,0
-0.56905819559392,48.24289575544,0
-0.56917900624035,48.242947013328,0
-0.56930606549118,48.242991063761,0
-0.56943840630884,48.24302757147,0
-0.56957502145051,48.243056258594,0
-0.56971487113543,48.243076906792,0
-0.56985689095988,48.24308935891,0
-0.56999999999994,48.243093520174,0
-0.57014310903999,48.24308935891,0
-0.57028512886444,48.243076906792,0
-0.57042497854936,48.243056258594,0
-0.57056159369103,48.24302757147,0
-0.5706939345087,48.242991063761,0
-0.57082099375953,48.242947013328,0
-0.57094180440596,48.24289575544,0
-0.57105544697659,48.242837680223,0
-0.57116105656479,48.242773229686,0
-0.57125782941174,48.242702894359,0
-0.57134502902377,48.242627209561,0
-0.57142199177716,48.24254675132,0
-0.57148813196838,48.242462131994,0
-0.57154294627057,48.242373995605,0
-0.57158601756308,48.242283012942,0
-0.57161701810443,48.242189876449,0
-0.57163571202494,48.242095294963,0
-0.57164195711988,48.241999988312,0
-0.57163570592973,48.241904681838,0
-0.57161700609921,48.241810100879,0
-0.5715860000126,48.241716965248,0
-0.5715429237081,48.241625983753,0
-0.57148810507947,48.241537848804,0
-0.57142196137884,48.241453231147,0
-0.57134499603964,48.241372774753,0
-0.57125779484405,48.241297091923,0
-0.57116102146381,48.241226758626,0
-0.57105541240888,48.241162310118,0
-0.57094177142186,48.241104236869,0
-0.57082096336121,48.241052980828,0
-0.57069390761978,48.241008932064,0
-0.57056157112856,48.240972425795,0
-0.57042496099886,48.24094373984,0
-0.5702851168592,48.240923092503,0
-0.57014310294478,48.240910640912,0
-0.56999999999994,48.240906479826,0
-0.5698568970551,48.240910640912,0
-0.56971488314065,48.240923092503,0
-0.56957503900099,48.24094373984,0
-0.56943842887132,48.240972425795,0
-0.56930609238007,48.241008932064,0
-0.56917903663867,48.241052980828,0
-0.56905822857802,48.241104236869,0
-0.568944587591,48.241162310118,0
-0.56883897853607,48.241226758626,0

-0.56874220515583,48.241297091923,0
-0.56865500396024,48.241372774753,0
-0.56857803862103,48.241453231147,0
-0.56851189492039,48.241537848804,0
-0.56845707629176,48.241625983753,0
-0.56841399998728,48.241716965248,0
-0.56838299390067,48.241810100879,0
-0.56836429407015,48.241904681838,0
-0.56835804287997,48.241999988312,0
-0.56999999999994,48.242,0
</coordinates>
</LinearRing>
</outerBoundaryIs>
</Polygon>
</Placemark>
<Placemark>
<name>11</name>
<styleUrl>#stylehap2</styleUrl>
<Polygon>
<outerBoundaryIs>
<LinearRing>
<coordinates>
-0.56799999999998,48.186,0
-0.56635983767871,48.185999988335,0
-0.56636607595322,48.186095294986,0
-0.56638474944549,48.186189876472,0
-0.56641571610607,48.186283012963,0
-0.56645874032291,48.186373995626,0
-0.56651349471265,48.186462132013,0
-0.56657956261045,48.186546751338,0
-0.56665644123957,48.186627209576,0
-0.56674354553656,48.186702894373,0
-0.5668402126031,48.186773229697,0
-0.56694570675052,48.186837680232,0
-0.56705922509861,48.186895755448,0
-0.5671799036862,48.186947013333,0
-0.56730682404701,48.186991063765,0
-0.56743902020066,48.187027571473,0
-0.56757548600547,48.187056258596,0
-0.56771518281733,48.187076906793,0
-0.56785704739626,48.18708935891,0
-0.568,48.187093520173,0
-0.56814295260373,48.18708935891,0
-0.56828481718264,48.187076906793,0
-0.5684245139945,48.187056258596,0
-0.5685609797993,48.187027571473,0
-0.56869317595295,48.186991063765,0
-0.56882009631379,48.186947013333,0
-0.56894077490138,48.186895755448,0
-0.56905429324945,48.186837680232,0
-0.56915978739687,48.186773229697,0
-0.5692564544634,48.186702894373,0
-0.56934355876039,48.186627209576,0
-0.56942043738951,48.186546751338,0
-0.56948650528731,48.186462132013,0
-0.56954125967706,48.186373995626,0
-0.56958428389389,48.186283012963,0

-0.56961525055447,48.186189876472,0
-0.56963392404674,48.186095294986,0
-0.56964016232125,48.185999988335,0
-0.56963391797015,48.18590468186,0
-0.56961523858592,48.185810100901,0
-0.56958426639705,48.185716965269,0
-0.56954123718354,48.185625983773,0
-0.56948647848058,48.185537848823,0
-0.56942040708407,48.185453231164,0
-0.56934352587707,48.185372774768,0
-0.56925642000135,48.185297091936,0
-0.56915975240318,48.185226758637,0
-0.5690542587874,48.185162310128,0
-0.56894074201806,48.185104236877,0
-0.56882006600835,48.185052980834,0
-0.56869314914622,48.185008932068,0
-0.56856095730579,48.184972425798,0
-0.56842449649766,48.184943739841,0
-0.56828480521408,48.184923092503,0
-0.56814294652712,48.184910640912,0
-0.568,48.184906479826,0
-0.56785705347285,48.184910640912,0
-0.56771519478588,48.184923092503,0
-0.56757550350231,48.184943739841,0
-0.56743904269418,48.184972425798,0
-0.56730685085374,48.185008932068,0
-0.56717993399162,48.185052980834,0
-0.5670592579819,48.185104236877,0
-0.56694574121257,48.185162310128,0
-0.56684024759678,48.185226758637,0
-0.56674357999861,48.185297091936,0
-0.56665647412289,48.185372774768,0
-0.5665795929159,48.185453231164,0
-0.56651352151939,48.185537848823,0
-0.56645876281643,48.185625983773,0
-0.56641573360291,48.185716965269,0
-0.56638476141405,48.185810100901,0
-0.56636608202981,48.18590468186,0
-0.56635983767871,48.185999988335,0
-0.56799999999998,48.186,0
</coordinates>
</LinearRing>
</outerBoundaryIs>
</Polygon>
</Placemark>
<Placemark>
<name>12</name>
<styleUrl>#stylehap2</styleUrl>
<Polygon>
<outerBoundaryIs>
<LinearRing>
<coordinates>
-0.56700000000001,48.233,0
-0.56535833170051,48.232999988315,0
-0.56536457569791,48.233095294967,0
-0.56538326633108,48.233189876453,0
-0.56541426142032,48.233283012945,0

-0.56545732513736,48.233373995609,0
-0.56551212979842,48.233462131997,0
-0.56557825835611,48.233546751323,0
-0.56565520757214,48.233627209564,0
-0.56574239184599,48.233702894361,0
-0.56583914767069,48.233773229688,0
-0.56594473868208,48.233837680224,0
-0.56605836126269,48.233895755441,0
-0.56617915065811,48.233947013329,0
-0.56630618755867,48.233991063762,0
-0.56643850509687,48.234027571471,0
-0.56657509620713,48.234056258594,0
-0.56671492129161,48.234076906793,0
-0.56685691613382,48.23408935891,0
-0.56700000000001,48.234093520174,0
-0.56714308386621,48.23408935891,0
-0.56728507870842,48.234076906793,0
-0.56742490379286,48.234056258594,0
-0.56756149490315,48.234027571471,0
-0.56769381244135,48.233991063762,0
-0.56782084934191,48.233947013329,0
-0.5679416387373,48.233895755441,0
-0.56805526131794,48.233837680224,0
-0.56816085232931,48.233773229688,0
-0.56825760815401,48.233702894361,0
-0.56834479242785,48.233627209564,0
-0.56842174164391,48.233546751323,0
-0.56848787020161,48.233462131997,0
-0.56854267486264,48.233373995609,0
-0.5685857385797,48.233283012945,0
-0.56861673366894,48.233189876453,0
-0.56863542430211,48.233095294967,0
-0.56864166829951,48.232999988315,0
-0.5686354182099,48.232904681841,0
-0.5686167216696,48.232810100882,0
-0.56858572103785,48.232716965251,0
-0.56854265231128,48.232625983756,0
-0.56848784332594,48.232537848807,0
-0.56842171126053,48.23245323115,0
-0.56834475945998,48.232372774755,0
-0.56825757360331,48.232297091925,0
-0.56816081724563,48.232226758628,0
-0.56805522676725,48.23216231012,0
-0.56794160576943,48.23210423687,0
-0.56782081895853,48.232052980829,0
-0.56769378556569,48.232008932065,0
-0.56756147235177,48.231972425795,0
-0.56742488625104,48.23194373984,0
-0.56728506670907,48.231923092503,0
-0.56714307777399,48.231910640912,0
-0.56700000000001,48.231906479826,0
-0.56685692222603,48.231910640912,0
-0.56671493329092,48.231923092503,0
-0.56657511374899,48.23194373984,0
-0.56643852764825,48.231972425795,0
-0.56630621443433,48.232008932065,0
-0.56617918104149,48.232052980829,0

-0.56605839423059,48.23210423687,0
-0.56594477323277,48.23216231012,0
-0.56583918275439,48.232226758628,0
-0.56574242639671,48.232297091925,0
-0.56565524054004,48.232372774755,0
-0.56557828873947,48.23245323115,0
-0.56551215667408,48.232537848807,0
-0.56545734768874,48.232625983756,0
-0.56541427896218,48.232716965251,0
-0.56538327833042,48.232810100882,0
-0.56536458179012,48.232904681841,0
-0.56535833170051,48.232999988315,0
-0.56700000000001,48.233,0
</coordinates>
</LinearRing>
</outerBoundaryIs>
</Polygon>
</Placemark>
<Placemark>
<name>13</name>
<styleUrl>#stylehap2</styleUrl>
<Polygon>
<outerBoundaryIs>
<LinearRing>
<coordinates>
-0.56399999999996,48.22,0
-0.56235874863474,48.219999988321,0
-0.56236499104773,48.220095294972,0
-0.56238367693542,48.220189876458,0
-0.56241466415411,48.22028301295,0
-0.56245771693541,48.220373995613,0
-0.56251250767869,48.220462132001,0
-0.56257861944255,48.220546751327,0
-0.56265554911638,48.220627209567,0
-0.56274271124843,48.220702894365,0
-0.56283944250019,48.22077322969,0
-0.56294500669449,48.220837680227,0
-0.56305860041806,48.220895755443,0
-0.56317935913601,48.22094701333,0
-0.56330636377221,48.220991063762,0
-0.56343864770477,48.221027571471,0
-0.56357520412386,48.221056258595,0
-0.56371499369571,48.221076906793,0
-0.56385695247414,48.22108935891,0
-0.56399999999995,48.221093520174,0
-0.56414304752579,48.22108935891,0
-0.56428500630423,48.221076906793,0
-0.56442479587608,48.221056258595,0
-0.56456135229517,48.221027571471,0
-0.56469363622772,48.220991063762,0
-0.56482064086392,48.22094701333,0
-0.56494139958187,48.220895755443,0
-0.56505499330545,48.220837680227,0
-0.56516055749975,48.22077322969,0
-0.56525728875151,48.220702894365,0
-0.56534445088352,48.220627209567,0
-0.56542138055739,48.220546751327,0

-0.56548749232122,48.220462132001,0
-0.56554228306453,48.220373995613,0
-0.56558533584583,48.22028301295,0
-0.56561632306452,48.220189876458,0
-0.56563500895217,48.220095294972,0
-0.56564125136517,48.219999988321,0
-0.56563500286429,48.219904681846,0
-0.5656163110737,48.219810100888,0
-0.56558531831644,48.219716965256,0
-0.56554226052918,48.219625983761,0
-0.56548746546464,48.219537848812,0
-0.56542135019561,48.219453231154,0
-0.56534441793906,48.219372774759,0
-0.56525725422536,48.219297091928,0
-0.56516052244098,48.21922675863,0
-0.56505495877928,48.219162310122,0
-0.56494136663741,48.219104236872,0
-0.56482061050212,48.21905298083,0
-0.56469360937115,48.219008932066,0
-0.56456132975982,48.218972425796,0
-0.56442477834667,48.218943739841,0
-0.56428499431344,48.218923092503,0
-0.56414304143788,48.218910640912,0
-0.56399999999995,48.218906479827,0
-0.56385695856203,48.218910640912,0
-0.5637150056865,48.218923092503,0
-0.56357522165324,48.218943739841,0
-0.56343867024012,48.218972425796,0
-0.56330639062879,48.219008932066,0
-0.56317938949779,48.21905298083,0
-0.56305863336253,48.219104236872,0
-0.56294504122066,48.219162310122,0
-0.56283947755896,48.21922675863,0
-0.56274274577457,48.219297091928,0
-0.56265558206088,48.219372774759,0
-0.56257864980432,48.219453231154,0
-0.56251253453529,48.219537848812,0
-0.56245773947076,48.219625983761,0
-0.5624146816835,48.219716965256,0
-0.56238368892623,48.219810100888,0
-0.56236499713565,48.219904681846,0
-0.56235874863474,48.219999988321,0
-0.56399999999996,48.22,0
</coordinates>
</LinearRing>
</outerBoundaryIs>
</Polygon>
</Placemark>
<Placemark>
<name>14</name>
<styleUrl>#stylehap2</styleUrl>
<Polygon>
<outerBoundaryIs>
<LinearRing>
<coordinates>
-0.56100000000004,48.236,0
-0.55935823544285,48.235999988314,0

-0.55936447980603,48.236095294966,0
-0.55938317153481,48.236189876452,0
-0.55941416844112,48.236283012944,0
-0.55945723468291,48.236373995607,0
-0.55951204255714,48.236462131996,0
-0.55957817499203,48.236546751322,0
-0.5596551287198,48.236627209563,0
-0.55974231810553,48.236702894361,0
-0.55983907960337,48.236773229687,0
-0.559944676806,48.236837680224,0
-0.56005830604889,48.236895755441,0
-0.56017910252682,48.236947013328,0
-0.56030614687624,48.236991063761,0
-0.56043847217298,48.237027571471,0
-0.56057507129241,48.237056258594,0
-0.56071490457567,48.237076906793,0
-0.56085690774394,48.23708935891,0
-0.56100000000003,48.237093520174,0
-0.56114309225614,48.23708935891,0
-0.56128509542441,48.237076906793,0
-0.56142492870767,48.237056258594,0
-0.5615615278271,48.237027571471,0
-0.56169385312384,48.236991063761,0
-0.56182089747326,48.236947013328,0
-0.56194169395119,48.236895755441,0
-0.56205532319405,48.236837680224,0
-0.56216092039668,48.236773229687,0
-0.56225768189455,48.236702894361,0
-0.56234487128028,48.236627209563,0
-0.56242182500804,48.236546751322,0
-0.56248795744294,48.236462131996,0
-0.56254276531717,48.236373995607,0
-0.56258583155896,48.236283012944,0
-0.56261682846526,48.236189876452,0
-0.56263552019405,48.236095294966,0
-0.56264176455723,48.235999988314,0
-0.56263551410082,48.23590468184,0
-0.56261681646396,48.235810100881,0
-0.56258581401423,48.23571696525,0
-0.5625427427621,48.235625983755,0
-0.56248793056285,48.235537848806,0
-0.56242179461968,48.235453231149,0
-0.56234483830699,48.235372774755,0
-0.56225764733818,48.235297091924,0
-0.56216088530725,48.235226758627,0
-0.5620552886377,48.235162310119,0
-0.56194166097789,48.23510423687,0
-0.56182086708492,48.235052980829,0
-0.56169382624375,48.235008932064,0
-0.56156150527203,48.234972425795,0
-0.56142491116294,48.23494373984,0
-0.56128508342311,48.234923092503,0
-0.56114308616294,48.234910640912,0
-0.56100000000003,48.234906479826,0
-0.56085691383714,48.234910640912,0
-0.56071491657697,48.234923092503,0
-0.56057508883714,48.23494373984,0

-0.56043849472805,48.234972425795,0
-0.5603061737563,48.235008932064,0
-0.56017913291516,48.235052980829,0
-0.56005833902218,48.23510423687,0
-0.55994471136237,48.235162310119,0
-0.55983911469283,48.235226758627,0
-0.5597423526619,48.235297091924,0
-0.55965516169309,48.235372774755,0
-0.5595782053804,48.235453231149,0
-0.5595120694372,48.235537848806,0
-0.55945725723798,48.235625983755,0
-0.55941418598585,48.23571696525,0
-0.55938318353612,48.235810100881,0
-0.55936448589924,48.23590468184,0
-0.55935823544285,48.235999988314,0
-0.56100000000004,48.236,0
</coordinates>
</LinearRing>
</outerBoundaryIs>
</Polygon>
</Placemark>
<Placemark>
<name>15</name>
<styleUrl>#stylehap2</styleUrl>
<Polygon>
<outerBoundaryIs>
<LinearRing>
<coordinates>
-0.56100000000004,48.189,0
-0.55935974166803,48.188999988333,0
-0.55936598030738,48.189095294985,0
-0.55938465489244,48.18918987647,0
-0.55941562336543,48.189283012962,0
-0.55945865010053,48.189373995624,0
-0.55951340769523,48.189462132012,0
-0.55957947946028,48.189546751337,0
-0.55965636258952,48.189627209576,0
-0.55974347198527,48.189702894372,0
-0.55984014471042,48.189773229697,0
-0.55994564503323,48.189837680232,0
-0.56005917002644,48.189895755447,0
-0.56017985567838,48.189947013333,0
-0.56030678346899,48.189991063765,0
-0.56043898736126,48.190027571473,0
-0.56057546115468,48.190056258596,0
-0.56071516614433,48.190076906793,0
-0.56085703902792,48.19008935891,0
-0.56100000000003,48.190093520174,0
-0.56114296097216,48.19008935891,0
-0.56128483385574,48.190076906793,0
-0.5614245388454,48.190056258596,0
-0.56156101263879,48.190027571473,0
-0.56169321653109,48.189991063765,0
-0.5618201443217,48.189947013333,0
-0.56194082997361,48.189895755447,0
-0.56205435496684,48.189837680232,0
-0.56215985528963,48.189773229697,0

-0.56225652801478,48.189702894372,0
-0.56234363741053,48.189627209576,0
-0.56242052053977,48.189546751337,0
-0.56248659230485,48.189462132012,0
-0.56254134989955,48.189373995624,0
-0.56258437663465,48.189283012962,0
-0.56261534510764,48.18918987647,0
-0.5626340196927,48.189095294985,0
-0.56264025833205,48.188999988333,0
-0.56263401361511,48.188904681859,0
-0.56261533313712,48.1888101009,0
-0.56258435913493,48.188716965268,0
-0.56254132740234,48.188625983772,0
-0.56248656549372,48.188537848822,0
-0.56242049022939,48.188453231163,0
-0.56234360452184,48.188372774768,0
-0.56225649354708,48.188297091935,0
-0.56215982029019,48.188226758637,0
-0.56205432049912,48.188162310127,0
-0.56194079708492,48.188104236876,0
-0.56182011401129,48.188052980834,0
-0.56169318971998,48.188008932068,0
-0.56156099014161,48.187972425798,0
-0.56142452134568,48.187943739841,0
-0.56128482188523,48.187923092503,0
-0.56114295489458,48.187910640912,0
-0.56100000000003,48.187906479827,0
-0.5608570451055,48.187910640912,0
-0.56071517811482,48.187923092503,0
-0.5605754786544,48.187943739841,0
-0.56043900985847,48.187972425798,0
-0.5603068102801,48.188008932068,0
-0.56017988598878,48.188052980834,0
-0.56005920291516,48.188104236876,0
-0.55994567950093,48.188162310127,0
-0.55984017970986,48.188226758637,0
-0.559743506453,48.188297091935,0
-0.55965639547824,48.188372774768,0
-0.55957950977068,48.188453231163,0
-0.55951343450634,48.188537848822,0
-0.55945867259774,48.188625983772,0
-0.55941564086515,48.188716965268,0
-0.55938466686295,48.1888101009,0
-0.55936598638496,48.188904681859,0
-0.55935974166803,48.188999988333,0
-0.56100000000004,48.189,0
</coordinates>
</LinearRing>
</outerBoundaryIs>
</Polygon>
</Placemark>
<Placemark>
<name>16</name>
<styleUrl>#stylehap2</styleUrl>
<Polygon>
<outerBoundaryIs>
<LinearRing>

<coordinates>
-0.55600000000004,48.165,0
-0.55436050931357,48.164999988343,0
-0.5543667450358,48.165095294995,0
-0.55438541088357,48.16518987648,0
-0.55441636486557,48.165283012971,0
-0.55445937146613,48.165373995633,0
-0.55451410343594,48.16546213202,0
-0.55458014428071,48.165546751344,0
-0.55465699142951,48.165627209582,0
-0.55474406005849,48.165702894378,0
-0.55484068754075,48.165773229702,0
-0.55494613848886,48.165837680236,0
-0.55505961035142,48.165895755451,0
-0.55518023952102,48.165947013336,0
-0.55530710790758,48.165991063767,0
-0.55543924992625,48.166027571474,0
-0.55557565984744,48.166056258596,0
-0.55571529945242,48.166076906793,0
-0.55585710593661,48.166089358911,0
-0.55600000000005,48.166093520174,0
-0.55614289406347,48.166089358911,0
-0.55628470054766,48.166076906793,0
-0.55642434015264,48.166056258596,0
-0.55656075007383,48.166027571474,0
-0.5566928920925,48.165991063767,0
-0.55681976047905,48.165947013336,0
-0.55694038964866,48.165895755451,0
-0.55705386151122,48.165837680236,0
-0.55715931245932,48.165773229702,0
-0.55725593994159,48.165702894378,0
-0.55734300857057,48.165627209582,0
-0.55741985571937,48.165546751344,0
-0.55748589656414,48.16546213202,0
-0.55754062853395,48.165373995633,0
-0.55758363513451,48.165283012971,0
-0.55761458911651,48.16518987648,0
-0.55763325496428,48.165095294995,0
-0.5576394906865,48.164999988343,0
-0.55763324889466,48.164904681869,0
-0.55761457716167,48.164810100909,0
-0.55758361765774,48.164716965277,0
-0.55754060606623,48.16462598378,0
-0.55748586978814,48.16453784883,0
-0.55741982544868,48.164453231171,0
-0.55734297572496,48.164372774774,0
-0.55725590551906,48.164297091941,0
-0.55715927750577,48.164226758642,0
-0.55705382708866,48.164162310131,0
-0.55694035680305,48.16410423688,0
-0.55681973020837,48.164052980836,0
-0.5566928653165,48.16400893207,0
-0.55656072760611,48.163972425799,0
-0.55642432267585,48.163943739842,0
-0.55628468859284,48.163923092504,0
-0.55614288799385,48.163910640912,0
-0.55600000000005,48.163906479827,0

-0.55585711200623,48.163910640912,0
-0.55571531140724,48.163923092504,0
-0.55557567732423,48.163943739842,0
-0.55543927239397,48.163972425799,0
-0.55530713468357,48.16400893207,0
-0.55518026979171,48.164052980836,0
-0.55505964319703,48.16410423688,0
-0.55494617291142,48.164162310131,0
-0.55484072249431,48.164226758642,0
-0.55474409448105,48.164297091941,0
-0.55465702427512,48.164372774774,0
-0.55458017455139,48.164453231171,0
-0.55451413021194,48.16453784883,0
-0.55445939393385,48.16462598378,0
-0.55441638234234,48.164716965277,0
-0.55438542283841,48.164810100909,0
-0.55436675110542,48.164904681869,0
-0.55436050931357,48.164999988343,0
-0.55600000000004,48.165,0
</coordinates>
</LinearRing>
</outerBoundaryIs>
</Polygon>
</Placemark>
<Placemark>
<name>17</name>
<styleUrl>#stylehap2</styleUrl>
<Polygon>
<outerBoundaryIs>
<LinearRing>
<coordinates>
-0.55500000000006,48.243,0
-0.55335801078018,48.242999988311,0
-0.55336425599711,48.243095294963,0
-0.55338295028298,48.243189876449,0
-0.55341395143029,48.243283012941,0
-0.55345702356475,48.243373995605,0
-0.55351183893848,48.243462131994,0
-0.55357798042263,48.24354675132,0
-0.5536549446806,48.243627209561,0
-0.55374214599734,48.243702894359,0
-0.55383892073618,48.243773229686,0
-0.55394453238902,48.243837680223,0
-0.55405817718138,48.24389575544,0
-0.55417899018967,48.243947013328,0
-0.55430605192456,48.243991063761,0
-0.55443839532952,48.24402757147,0
-0.55457501314209,48.244056258594,0
-0.55471486556114,48.244076906792,0
-0.55485688816214,48.24408935891,0
-0.55500000000007,48.244093520174,0
-0.55514311183799,48.24408935891,0
-0.55528513443899,48.244076906792,0
-0.55542498685804,48.244056258594,0
-0.55556160467061,48.24402757147,0
-0.55569394807557,48.243991063761,0
-0.55582100981046,48.243947013328,0

-0.55594182281875,48.24389575544,0
-0.55605546761109,48.243837680223,0
-0.55616107926395,48.243773229686,0
-0.5562578540028,48.243702894359,0
-0.55634505531953,48.243627209561,0
-0.55642201957747,48.24354675132,0
-0.55648816106166,48.243462131994,0
-0.55654297643538,48.243373995605,0
-0.55658604856985,48.243283012941,0
-0.55661704971716,48.243189876449,0
-0.55663574400303,48.243095294963,0
-0.55664198921995,48.242999988311,0
-0.55663573790748,48.242904681837,0
-0.55661703771128,48.242810100878,0
-0.5565860310184,48.242716965247,0
-0.55654295387169,48.242625983752,0
-0.55648813417129,48.242537848804,0
-0.5564219891775,48.242453231147,0
-0.55634502233359,48.242372774753,0
-0.5562578194332,48.242297091922,0
-0.55616104416106,48.242226758626,0
-0.55605543304149,48.242162310118,0
-0.55594178983284,48.242104236869,0
-0.55582097941047,48.242052980828,0
-0.55569392118521,48.242008932064,0
-0.55556158210689,48.241972425795,0
-0.5554249693066,48.24194373984,0
-0.55528512243311,48.241923092503,0
-0.55514310574242,48.241910640912,0
-0.55500000000007,48.241906479826,0
-0.55485689425769,48.241910640912,0
-0.55471487756702,48.241923092503,0
-0.55457503069354,48.24194373984,0
-0.55443841789321,48.241972425795,0
-0.55430607881493,48.242008932064,0
-0.55417902058967,48.242052980828,0
-0.55405821016729,48.242104236869,0
-0.55394456695862,48.242162310118,0
-0.55383895583907,48.242226758626,0
-0.55374218056691,48.242297091922,0
-0.55365497766652,48.242372774753,0
-0.55357801082263,48.242453231147,0
-0.55351186582885,48.242537848804,0
-0.55345704612845,48.242625983752,0
-0.55341396898173,48.242716965247,0
-0.55338296228886,48.242810100878,0
-0.55336426209265,48.242904681837,0
-0.55335801078018,48.242999988311,0
-0.55500000000006,48.243,0
</coordinates>
</LinearRing>
</outerBoundaryIs>
</Polygon>
</Placemark>
<Placemark>
<name>18</name>
<styleUrl>#stylehap2</styleUrl>

<Polygon>
<outerBoundaryIs>
<LinearRing>
<coordinates>
-0.55500000000006,48.243,0
-0.55335801078018,48.242999988311,0
-0.55336425599711,48.243095294963,0
-0.55338295028298,48.243189876449,0
-0.55341395143029,48.243283012941,0
-0.55345702356475,48.243373995605,0
-0.55351183893848,48.243462131994,0
-0.55357798042263,48.24354675132,0
-0.5536549446806,48.243627209561,0
-0.55374214599734,48.243702894359,0
-0.55383892073618,48.243773229686,0
-0.55394453238902,48.243837680223,0
-0.55405817718138,48.24389575544,0
-0.55417899018967,48.243947013328,0
-0.55430605192456,48.243991063761,0
-0.55443839532952,48.24402757147,0
-0.55457501314209,48.244056258594,0
-0.55471486556114,48.244076906792,0
-0.55485688816214,48.24408935891,0
-0.55500000000007,48.244093520174,0
-0.55514311183799,48.24408935891,0
-0.55528513443899,48.244076906792,0
-0.55542498685804,48.244056258594,0
-0.55556160467061,48.24402757147,0
-0.55569394807557,48.243991063761,0
-0.55582100981046,48.243947013328,0
-0.55594182281875,48.24389575544,0
-0.55605546761109,48.243837680223,0
-0.55616107926395,48.243773229686,0
-0.5562578540028,48.243702894359,0
-0.55634505531953,48.243627209561,0
-0.55642201957747,48.24354675132,0
-0.55648816106166,48.243462131994,0
-0.55654297643538,48.243373995605,0
-0.55658604856985,48.243283012941,0
-0.55661704971716,48.243189876449,0
-0.55663574400303,48.243095294963,0
-0.55664198921995,48.242999988311,0
-0.55663573790748,48.242904681837,0
-0.55661703771128,48.242810100878,0
-0.5565860310184,48.242716965247,0
-0.55654295387169,48.242625983752,0
-0.55648813417129,48.242537848804,0
-0.5564219891775,48.242453231147,0
-0.55634502233359,48.242372774753,0
-0.5562578194332,48.242297091922,0
-0.55616104416106,48.242226758626,0
-0.55605543304149,48.242162310118,0
-0.55594178983284,48.242104236869,0
-0.55582097941047,48.242052980828,0
-0.55569392118521,48.242008932064,0
-0.55556158210689,48.241972425795,0
-0.5554249693066,48.24194373984,0

-0.55528512243311,48.241923092503,0
-0.55514310574242,48.241910640912,0
-0.55500000000007,48.241906479826,0
-0.55485689425769,48.241910640912,0
-0.55471487756702,48.241923092503,0
-0.55457503069354,48.24194373984,0
-0.55443841789321,48.241972425795,0
-0.55430607881493,48.242008932064,0
-0.55417902058967,48.242052980828,0
-0.55405821016729,48.242104236869,0
-0.55394456695862,48.242162310118,0
-0.55383895583907,48.242226758626,0
-0.55374218056691,48.242297091922,0
-0.55365497766652,48.242372774753,0
-0.55357801082263,48.242453231147,0
-0.55351186582885,48.242537848804,0
-0.55345704612845,48.242625983752,0
-0.55341396898173,48.242716965247,0
-0.55338296228886,48.242810100878,0
-0.55336426209265,48.242904681837,0
-0.55335801078018,48.242999988311,0
-0.55500000000006,48.243,0
</coordinates>
</LinearRing>
</outerBoundaryIs>
</Polygon>
</Placemark>
<Placemark>
<name>19</name>
<styleUrl>#stylehap2</styleUrl>
<Polygon>
<outerBoundaryIs>
<LinearRing>
<coordinates>
-0.55500000000006,48.249,0
-0.55335781814368,48.248999988309,0
-0.55336406409267,48.24909529496,0
-0.55338276057111,48.249189876447,0
-0.55341376535486,48.249283012939,0
-0.55345684254196,48.249373995603,0
-0.5535116643461,48.249462131992,0
-0.55357781358956,48.249546751318,0
-0.55365478687662,48.249627209559,0
-0.55374199842354,48.249702894358,0
-0.55383878451585,48.249773229684,0
-0.55394440855901,48.249837680222,0
-0.5540580666842,48.249895755439,0
-0.55417889386644,48.249947013327,0
-0.55430597050844,48.24999106376,0
-0.55443832944026,48.25002757147,0
-0.55457496328122,48.250056258594,0
-0.5547148321082,48.250076906792,0
-0.55485687137176,48.25008935891,0
-0.55500000000007,48.250093520174,0
-0.55514312862837,48.25008935891,0
-0.55528516789194,48.250076906792,0
-0.55542503671891,48.250056258594,0

-0.55556167055985,48.25002757147,0
-0.55569402949167,48.24999106376,0
-0.55582110613369,48.249947013327,0
-0.55594193331591,48.249895755439,0
-0.5560555914411,48.249837680222,0
-0.55616121548426,48.249773229684,0
-0.55625800157657,48.249702894358,0
-0.55634521312349,48.249627209559,0
-0.55642218641057,48.249546751318,0
-0.55648833565401,48.249462131992,0
-0.55654315745818,48.249373995603,0
-0.55658623464527,48.249283012939,0
-0.55661723942902,48.249189876447,0
-0.55663593590747,48.24909529496,0
-0.55664218185643,48.248999988309,0
-0.55663592980991,48.248904681835,0
-0.5566172274192,48.248810100876,0
-0.55658621708808,48.248716965245,0
-0.55654313488705,48.24862598375,0
-0.55648830875481,48.248537848802,0
-0.5564221560006,48.248453231145,0
-0.55634518012676,48.248372774751,0
-0.55625796699564,48.248297091921,0
-0.55616118036987,48.248226758624,0
-0.55605555686018,48.248162310117,0
-0.55594190031918,48.248104236868,0
-0.55582107572372,48.248052980827,0
-0.5556940025925,48.248008932063,0
-0.55556164798875,48.247972425795,0
-0.55542501916171,48.24794373984,0
-0.55528515588211,48.247923092503,0
-0.55514312253081,48.247910640912,0
-0.55500000000007,48.247906479826,0
-0.55485687746932,48.247910640912,0
-0.55471484411802,48.247923092503,0
-0.55457498083842,48.24794373984,0
-0.55443835201138,48.247972425795,0
-0.55430599740764,48.248008932063,0
-0.55417892427641,48.248052980827,0
-0.55405809968096,48.248104236868,0
-0.55394444313995,48.248162310117,0
-0.55383881963027,48.248226758624,0
-0.55374203300449,48.248297091921,0
-0.55365481987337,48.248372774751,0
-0.55357784399953,48.248453231145,0
-0.5535116912453,48.248537848802,0
-0.55345686511305,48.24862598375,0
-0.55341378291206,48.248716965245,0
-0.55338277258094,48.248810100876,0
-0.55336407019022,48.248904681835,0
-0.55335781814368,48.248999988309,0
-0.55500000000006,48.249,0
</coordinates>
</LinearRing>
</outerBoundaryIs>
</Polygon>
</Placemark>

<Placemark>
<name>20</name>
<styleUrl>#stylehap2</styleUrl>
<Polygon>
<outerBoundaryIs>
<LinearRing>
<coordinates>
-0.53499999999997,48.233,0
-0.53335833170047,48.232999988315,0
-0.53336457569786,48.233095294967,0
-0.53338326633103,48.233189876453,0
-0.53341426142028,48.233283012945,0
-0.53345732513734,48.233373995609,0
-0.53351212979837,48.233462131997,0
-0.53357825835607,48.233546751323,0
-0.53365520757213,48.233627209564,0
-0.53374239184597,48.233702894361,0
-0.53383914767067,48.233773229688,0
-0.53394473868203,48.233837680224,0
-0.53405836126265,48.233895755441,0
-0.53417915065807,48.233947013329,0
-0.53430618755863,48.233991063762,0
-0.53443850509682,48.234027571471,0
-0.53457509620711,48.234056258594,0
-0.53471492129156,48.234076906793,0
-0.53485691613377,48.23408935891,0
-0.53499999999997,48.234093520174,0
-0.53514308386616,48.23408935891,0
-0.53528507870837,48.234076906793,0
-0.53542490379285,48.234056258594,0
-0.53556149490311,48.234027571471,0
-0.5356938124413,48.233991063762,0
-0.53582084934186,48.233947013329,0
-0.53594163873728,48.233895755441,0
-0.5360552613179,48.233837680224,0
-0.53616085232929,48.233773229688,0
-0.53625760815399,48.233702894361,0
-0.53634479242781,48.233627209564,0
-0.53642174164387,48.233546751323,0
-0.53648787020156,48.233462131997,0
-0.53654267486259,48.233373995609,0
-0.53658573857966,48.233283012945,0
-0.5366167336689,48.233189876453,0
-0.53663542430207,48.233095294967,0
-0.53664166829947,48.232999988315,0
-0.53663541820986,48.232904681841,0
-0.53661672166956,48.232810100882,0
-0.5365857210378,48.232716965251,0
-0.53654265231124,48.232625983756,0
-0.5364878433259,48.232537848807,0
-0.53642171126051,48.23245323115,0
-0.53634475945993,48.232372774755,0
-0.53625757360327,48.232297091925,0
-0.53616081724559,48.232226758628,0
-0.5360552267672,48.23216231012,0
-0.53594160576938,48.23210423687,0
-0.53582081895848,48.232052980829,0

-0.53569378556564,48.232008932065,0
-0.53556147235173,48.231972425795,0
-0.53542488625099,48.23194373984,0
-0.53528506670906,48.231923092503,0
-0.53514307777395,48.231910640912,0
-0.53499999999997,48.231906479826,0
-0.53485692222598,48.231910640912,0
-0.53471493329088,48.231923092503,0
-0.53457511374894,48.23194373984,0
-0.5344385276482,48.231972425795,0
-0.53430621443429,48.232008932065,0
-0.53417918104145,48.232052980829,0
-0.53405839423055,48.23210423687,0
-0.53394477323273,48.23216231012,0
-0.53383918275435,48.232226758628,0
-0.53374242639667,48.232297091925,0
-0.53365524054,48.232372774755,0
-0.53357828873945,48.23245323115,0
-0.53351215667403,48.232537848807,0
-0.53345734768869,48.232625983756,0
-0.53341427896213,48.232716965251,0
-0.53338327833038,48.232810100882,0
-0.53336458179008,48.232904681841,0
-0.53335833170047,48.232999988315,0
-0.53499999999997,48.233,0
</coordinates>
</LinearRing>
</outerBoundaryIs>
</Polygon>
</Placemark>
<Placemark>
<name>21</name>
<styleUrl>#stylehap2</styleUrl>
<Polygon>
<outerBoundaryIs>
<LinearRing>
<coordinates>
-0.53200000000004,48.245,0
-0.53035794657501,48.24499998831,0
-0.53036419203592,48.245095294962,0
-0.53038288705258,48.245189876448,0
-0.53041388941189,48.24528301294,0
-0.53045696323039,48.245373995604,0
-0.53051178074735,48.245462131993,0
-0.53057792481768,48.245546751319,0
-0.53065489208501,48.24562720956,0
-0.53074209681143,48.245702894359,0
-0.53083887533434,48.245773229685,0
-0.53094449111684,48.245837680222,0
-0.531058140353,48.24589575544,0
-0.53117895808543,48.245947013327,0
-0.5313060247888,48.245991063761,0
-0.5314383733688,48.24602757147,0
-0.53157499652359,48.246056258594,0
-0.53171485441134,48.246076906792,0
-0.53185688256594,48.24608935891,0
-0.53200000000004,48.246093520174,0

-0.53214311743414,48.24608935891,0
-0.53228514558874,48.246076906792,0
-0.53242500347649,48.246056258594,0
-0.53256162663127,48.24602757147,0
-0.53269397521127,48.245991063761,0
-0.53282104191465,48.245947013327,0
-0.53294185964708,48.24589575544,0
-0.53305550888324,48.245837680222,0
-0.53316112466573,48.245773229685,0
-0.53325790318865,48.245702894359,0
-0.53334510791506,48.24562720956,0
-0.5334220751824,48.245546751319,0
-0.53348821925273,48.245462131993,0
-0.53354303676969,48.245373995604,0
-0.53358611058819,48.24528301294,0
-0.5336171129475,48.245189876448,0
-0.53363580796416,48.245095294962,0
-0.53364205342507,48.24499998831,0
-0.53363580186792,48.244904681836,0
-0.53361710094029,48.244810100878,0
-0.53358609303481,48.244716965246,0
-0.53354301420352,48.244625983751,0
-0.53348819235941,48.244537848803,0
-0.53342204477909,48.244453231146,0
-0.53334507492554,48.244372774752,0
-0.53325786861528,48.244297091922,0
-0.533161089559,48.244226758625,0
-0.53305547430985,48.244162310118,0
-0.53294182665755,48.244104236869,0
-0.53282101151135,48.244052980828,0
-0.53269394831798,48.244008932064,0
-0.53256160406508,48.243972425795,0
-0.53242498592313,48.24394373984,0
-0.53228513358154,48.243923092503,0
-0.53214311133793,48.243910640912,0
-0.53200000000004,48.243906479826,0
-0.53185688866215,48.243910640912,0
-0.53171486641854,48.243923092503,0
-0.53157501407697,48.24394373984,0
-0.53143839593499,48.243972425795,0
-0.5313060516821,48.244008932064,0
-0.53117898848873,48.244052980828,0
-0.53105817334252,48.244104236869,0
-0.53094452569023,48.244162310118,0
-0.53083891044108,48.244226758625,0
-0.5307421313848,48.244297091922,0
-0.53065492507454,48.244372774752,0
-0.53057795522098,48.244453231146,0
-0.53051180764067,48.244537848803,0
-0.53045698579655,48.244625983751,0
-0.53041390696527,48.244716965246,0
-0.53038289905978,48.244810100878,0
-0.53036419813215,48.244904681836,0
-0.53035794657501,48.24499998831,0
-0.53200000000004,48.245,0
</coordinates>
</LinearRing>

</outerBoundaryIs>
</Polygon>
</Placemark>
<Placemark>
<name>22</name>
<styleUrl>#stylehap2</styleUrl>
<Polygon>
<outerBoundaryIs>
<LinearRing>
<coordinates>
-0.52999999999997,48.266,0
-0.52835727199685,48.265999988302,0
-0.52836352002123,48.266095294954,0
-0.52838222271585,48.26618987644,0
-0.52841323780934,48.266283012932,0
-0.52845632932129,48.266373995597,0
-0.52851116935645,48.266462131986,0
-0.52857734059836,48.266546751313,0
-0.52865433948395,48.266627209554,0
-0.52874158003468,48.266702894354,0
-0.52883839831534,48.266773229681,0
-0.52894405748648,48.266837680219,0
-0.52905775341186,48.266895755437,0
-0.52917862077883,48.266947013325,0
-0.52930573968424,48.266991063759,0
-0.52943814263649,48.267027571469,0
-0.52957482191979,48.267056258593,0
-0.52971473726513,48.267076906792,0
-0.52985682376901,48.26708935891,0
-0.52999999999997,48.267093520173,0
-0.53014317623095,48.26708935891,0
-0.53028526273483,48.267076906792,0
-0.53042517808017,48.267056258593,0
-0.53056185736347,48.267027571469,0
-0.53069426031569,48.266991063759,0
-0.5308213792211,48.266947013325,0
-0.53094224658808,48.266895755437,0
-0.53105594251348,48.266837680219,0
-0.53116160168459,48.266773229681,0
-0.53125841996525,48.266702894354,0
-0.53134566051599,48.266627209554,0
-0.53142265940157,48.266546751313,0
-0.53148883064351,48.266462131986,0
-0.53154367067864,48.266373995597,0
-0.5315867621906,48.266283012932,0
-0.53161777728408,48.26618987644,0
-0.53163647997873,48.266095294954,0
-0.53164272800311,48.265999988302,0
-0.5316364738755,48.265904681828,0
-0.53161776526308,48.265810100869,0
-0.53158674461706,48.265716965238,0
-0.53154364808655,48.265625983744,0
-0.53148880371927,48.265537848796,0
-0.53142262896333,48.26545323114,0
-0.53134562748855,48.265372774746,0
-0.53125838535214,48.265297091917,0
-0.53116156653753,48.265226758621,0

-0.53105590790037,48.265162310114,0
-0.53094221356064,48.265104236866,0
-0.53082134878287,48.265052980826,0
-0.53069423339148,48.265008932062,0
-0.53056183477136,48.264972425794,0
-0.53042516050663,48.264943739839,0
-0.53028525071381,48.264923092502,0
-0.53014317012772,48.264910640912,0
-0.52999999999997,48.264906479826,0
-0.52985682987224,48.264910640912,0
-0.52971474928612,48.264923092502,0
-0.52957483949332,48.264943739839,0
-0.52943816522858,48.264972425794,0
-0.52930576660848,48.265008932062,0
-0.52917865121709,48.265052980826,0
-0.5290577864393,48.265104236866,0
-0.52894409209957,48.265162310114,0
-0.52883843346243,48.265226758621,0
-0.52874161464779,48.265297091917,0
-0.52865437251139,48.265372774746,0
-0.52857737103663,48.26545323114,0
-0.52851119628066,48.265537848796,0
-0.52845635191338,48.265625983744,0
-0.52841325538287,48.265716965238,0
-0.52838223473688,48.265810100869,0
-0.52836352612446,48.265904681828,0
-0.52835727199685,48.265999988302,0
-0.52999999999997,48.266,0
</coordinates>
</LinearRing>
</outerBoundaryIs>
</Polygon>
</Placemark>
<Placemark>
<name>23</name>
<styleUrl>#stylehap2</styleUrl>
<Polygon>
<outerBoundaryIs>
<LinearRing>
<coordinates>
-0.52800000000002,48.328,0
-0.52635527585735,48.327999988276,0
-0.52636153146728,48.328095294928,0
-0.52638025688178,48.328189876415,0
-0.52641130965679,48.328283012909,0
-0.52645445352533,48.328373995574,0
-0.52650936019393,48.328462131965,0
-0.52657561183914,48.328546751294,0
-0.52665270428609,48.328627209537,0
-0.5267400508443,48.328702894339,0
-0.52683698677187,48.328773229668,0
-0.52694277433394,48.328837680208,0
-0.52705660841717,48.328895755429,0
-0.52717762265737,48.328947013319,0
-0.52730489603352,48.328991063755,0
-0.52743745987819,48.329027571466,0
-0.52757430525103,48.329056258592,0

-0.52771439061874,48.329076906791,0
-0.52785664978367,48.32908935891,0
-0.52800000000002,48.329093520174,0
-0.52814335021638,48.32908935891,0
-0.52828560938131,48.329076906791,0
-0.52842569474902,48.329056258592,0
-0.52856254012183,48.329027571466,0
-0.52869510396653,48.328991063755,0
-0.52882237734265,48.328947013319,0
-0.52894339158286,48.328895755429,0
-0.52905722566611,48.328837680208,0
-0.52916301322817,48.328773229668,0
-0.52925994915575,48.328702894339,0
-0.52934729571396,48.328627209537,0
-0.5294243881609,48.328546751294,0
-0.52949063980612,48.328462131965,0
-0.52954554647469,48.328373995574,0
-0.52958869034325,48.328283012909,0
-0.52961974311827,48.328189876415,0
-0.52963846853276,48.328095294928,0
-0.52964472414269,48.327999988276,0
-0.5296384624088,48.327904681802,0
-0.52961973105638,48.327810100844,0
-0.52958867271,48.327716965215,0
-0.5295455238058,48.327625983721,0
-0.52949061279038,48.327537848775,0
-0.52942435761921,48.327453231121,0
-0.52934726257426,48.327372774729,0
-0.52925991442498,48.327297091902,0
-0.52916297796165,48.327226758608,0
-0.52905719093537,48.327162310104,0
-0.52894335844318,48.327104236857,0
-0.52882234680096,48.327052980819,0
-0.5286950769508,48.327008932058,0
-0.52856251745295,48.326972425791,0
-0.52842567711574,48.326943739838,0
-0.52828559731942,48.326923092502,0
-0.52814334409241,48.326910640912,0
-0.52800000000002,48.326906479826,0
-0.52785665590764,48.326910640912,0
-0.5277144026806,48.326923092502,0
-0.52757432288431,48.326943739838,0
-0.5274374825471,48.326972425791,0
-0.52730492304925,48.327008932058,0
-0.52717765319909,48.327052980819,0
-0.52705664155687,48.327104236857,0
-0.52694280906468,48.327162310104,0
-0.5268370220384,48.327226758608,0
-0.52674008557507,48.327297091902,0
-0.52665273742579,48.327372774729,0
-0.52657564238084,48.327453231121,0
-0.52650938720966,48.327537848775,0
-0.52645447619424,48.327625983721,0
-0.52641132729004,48.327716965215,0
-0.52638026894364,48.327810100844,0
-0.52636153759125,48.327904681802,0
-0.52635527585735,48.327999988276,0

-0.52800000000002,48.328,0
</coordinates>
</LinearRing>
</outerBoundaryIs>
</Polygon>
</Placemark>
<Placemark>
<name>24</name>
<styleUrl>#stylehap2</styleUrl>
<Polygon>
<outerBoundaryIs>
<LinearRing>
<coordinates>
-0.52600000000007,48.234,0
-0.52435829961642,48.233999988315,0
-0.52436454373574,48.234095294967,0
-0.52438323473409,48.234189876453,0
-0.52441423042899,48.234283012945,0
-0.52445729498758,48.234373995608,0
-0.52451210071961,48.234462131997,0
-0.52457823056966,48.234546751323,0
-0.52465518128953,48.234627209563,0
-0.52474236726724,48.234702894361,0
-0.52483912498289,48.234773229687,0
-0.5249447180579,48.234837680224,0
-0.52505834285916,48.234895755441,0
-0.52517913461529,48.234947013328,0
-0.52530617399864,48.234991063761,0
-0.5254384941229,48.235027571471,0
-0.52557508790272,48.235056258594,0
-0.52571491571998,48.235076906793,0
-0.52585691333739,48.23508935891,0
-0.52600000000008,48.235093520174,0
-0.52614308666274,48.23508935891,0
-0.52628508428015,48.235076906793,0
-0.52642491209741,48.235056258594,0
-0.52656150587725,48.235027571471,0
-0.5266938260015,48.234991063761,0
-0.52682086538485,48.234947013328,0
-0.52694165714098,48.234895755441,0
-0.52705528194223,48.234837680224,0
-0.52716087501724,48.234773229687,0
-0.52725763273289,48.234702894361,0
-0.5273448187106,48.234627209563,0
-0.52742176943047,48.234546751323,0
-0.52748789928052,48.234462131997,0
-0.52754270501256,48.234373995608,0
-0.52758576957114,48.234283012945,0
-0.52761676526604,48.234189876453,0
-0.52763545626439,48.234095294967,0
-0.52764170038372,48.233999988315,0
-0.52763545017185,48.233904681841,0
-0.52761675326604,48.233810100882,0
-0.52758575202832,48.233716965251,0
-0.52754268245996,48.233625983755,0
-0.52748787240338,48.233537848807,0
-0.52742173904544,48.23345323115,0

-0.52734478574092,48.233372774755,0
-0.52725759818031,48.233297091925,0
-0.52716083993163,48.233226758628,0
-0.52705524738965,48.23316231012,0
-0.5269416241713,48.23310423687,0
-0.52682083499981,48.233052980829,0
-0.52669379912436,48.233008932064,0
-0.52656148332465,48.232972425795,0
-0.52642489455459,48.23294373984,0
-0.52628507228018,48.232923092503,0
-0.5261430805702,48.232910640912,0
-0.52600000000008,48.232906479827,0
-0.52585691942993,48.232910640912,0
-0.52571492771996,48.232923092503,0
-0.52557510544554,48.23294373984,0
-0.5254385166755,48.232972425795,0
-0.52530620087577,48.233008932064,0
-0.52517916500032,48.233052980829,0
-0.52505837582884,48.23310423687,0
-0.52494475261048,48.23316231012,0
-0.52483916006851,48.233226758628,0
-0.52474240181982,48.233297091925,0
-0.52465521425921,48.233372774755,0
-0.52457826095469,48.23345323115,0
-0.52451212759675,48.233537848807,0
-0.52445731754018,48.233625983755,0
-0.52441424797181,48.233716965251,0
-0.5243832467341,48.233810100882,0
-0.52436454982829,48.233904681841,0
-0.52435829961642,48.233999988315,0
-0.52600000000007,48.234,0
</coordinates>
</LinearRing>
</outerBoundaryIs>
</Polygon>
</Placemark>
<Placemark>
<name>25</name>
<styleUrl>#stylehap2</styleUrl>
<Polygon>
<outerBoundaryIs>
<LinearRing>
<coordinates>
-0.52499999999998,48.235,0
-0.52335826753044,48.234999988315,0
-0.52336451177169,48.235095294966,0
-0.52338320313525,48.235189876452,0
-0.52341419943582,48.235283012944,0
-0.52345726483598,48.235373995608,0
-0.52351207163911,48.235462131996,0
-0.52357820278152,48.235546751323,0
-0.52365515500529,48.235627209563,0
-0.52374234268699,48.235702894361,0
-0.52383910229367,48.235773229687,0
-0.52394469743245,48.235837680224,0
-0.52405832445445,48.235895755441,0
-0.52417911857141,48.235947013328,0

-0.52430616043773,48.235991063761,0
-0.52443848314817,48.236027571471,0
-0.52457507959772,48.236056258594,0
-0.52471491014789,48.236076906793,0
-0.52485691054065,48.23608935891,0
-0.52499999999997,48.236093520174,0
-0.52514308945931,48.23608935891,0
-0.52528508985204,48.236076906793,0
-0.52542492040224,48.236056258594,0
-0.52556151685179,48.236027571471,0
-0.52569383956223,48.235991063761,0
-0.52582088142855,48.235947013328,0
-0.52594167554551,48.235895755441,0
-0.52605530256751,48.235837680224,0
-0.52616089770627,48.235773229687,0
-0.52625765731297,48.235702894361,0
-0.52634484499464,48.235627209563,0
-0.52642179721844,48.235546751323,0
-0.52648792836085,48.235462131996,0
-0.52654273516395,48.235373995608,0
-0.52658580056414,48.235283012944,0
-0.52661679686472,48.235189876452,0
-0.52663548822827,48.235095294966,0
-0.52664173246952,48.234999988315,0
-0.52663548213539,48.23490468184,0
-0.52661678486408,48.234810100882,0
-0.52658578302035,48.23471696525,0
-0.52654271261013,48.234625983755,0
-0.52648790148224,48.234537848807,0
-0.52642176683172,48.234453231149,0
-0.52634481202316,48.234372774755,0
-0.52625762275851,48.234297091924,0
-0.52616086261874,48.234226758627,0
-0.52605526801304,48.23416231012,0
-0.525941642574,48.23410423687,0
-0.52582085104186,48.234052980829,0
-0.52569381268361,48.234008932064,0
-0.52556149429796,48.233972425795,0
-0.52542490285847,48.23394373984,0
-0.5252850778514,48.233923092503,0
-0.52514308336641,48.233910640912,0
-0.52499999999997,48.233906479827,0
-0.52485691663355,48.233910640912,0
-0.52471492214856,48.233923092503,0
-0.52457509714149,48.23394373984,0
-0.524438505702,48.233972425795,0
-0.52430618731635,48.234008932064,0
-0.5241791489581,48.234052980829,0
-0.52405835742593,48.23410423687,0
-0.52394473198692,48.23416231012,0
-0.52383913738122,48.234226758627,0
-0.52374237724145,48.234297091924,0
-0.5236551879768,48.234372774755,0
-0.52357823316821,48.234453231149,0
-0.52351209851772,48.234537848807,0
-0.52345728738983,48.234625983755,0
-0.52341421697958,48.23471696525,0

-0.52338321513588,48.234810100882,0
-0.52336451786457,48.23490468184,0
-0.52335826753044,48.234999988315,0
-0.52499999999998,48.235,0
</coordinates>
</LinearRing>
</outerBoundaryIs>
</Polygon>
</Placemark>
<Placemark>
<name>26</name>
<styleUrl>#stylehap2</styleUrl>
<Polygon>
<outerBoundaryIs>
<LinearRing>
<coordinates>
-0.52099999999996,48.33,0
-0.51935521135283,48.329999988275,0
-0.5193614672079,48.330095294927,0
-0.51938019335659,48.330189876414,0
-0.51941124734925,48.330283012908,0
-0.51945439290969,48.330373995573,0
-0.5195093017315,48.330462131964,0
-0.51957555597489,48.330546751293,0
-0.51965265144525,48.330627209537,0
-0.51974000142907,48.330702894338,0
-0.51983694115835,48.330773229668,0
-0.51994273286931,48.330837680208,0
-0.52005657141707,48.330895755428,0
-0.52017759040342,48.330947013319,0
-0.52030486877121,48.330991063754,0
-0.52043743781507,48.331027571466,0
-0.52057428855503,48.331056258592,0
-0.52071437941693,48.331076906791,0
-0.52085664416132,48.33108935891,0
-0.52099999999995,48.331093520174,0
-0.52114335583858,48.33108935891,0
-0.52128562058297,48.331076906791,0
-0.5214257114449,48.331056258592,0
-0.52156256218483,48.331027571466,0
-0.5216951312287,48.330991063754,0
-0.52182240959651,48.330947013319,0
-0.52194342858286,48.330895755428,0
-0.52205726713062,48.330837680208,0
-0.52216305884158,48.330773229668,0
-0.52225999857086,48.330702894338,0
-0.52234734855466,48.330627209537,0
-0.52242444402501,48.330546751293,0
-0.52249069826841,48.330462131964,0
-0.52254560709024,48.330373995573,0
-0.52258875265068,48.330283012908,0
-0.52261980664334,48.330189876414,0
-0.52263853279203,48.330095294927,0
-0.52264478864707,48.329999988275,0
-0.52263852666741,48.329904681802,0
-0.52261979458016,48.329810100844,0
-0.52258873501547,48.329716965214,0

-0.52254558441886,48.329625983721,0
-0.52249067124972,48.329537848775,0
-0.52242441347996,48.32945323112,0
-0.52234731541134,48.329372774729,0
-0.5222599638363,48.329297091901,0
-0.52216302357119,48.329226758608,0
-0.52205723239607,48.329162310103,0
-0.52194339543953,48.329104236857,0
-0.52182237905146,48.329052980819,0
-0.52169510421001,48.329008932057,0
-0.52156253951346,48.328972425791,0
-0.52142569380969,48.328943739837,0
-0.52128560851979,48.328923092502,0
-0.52114334971395,48.328910640912,0
-0.52099999999995,48.328906479827,0
-0.52085665028598,48.328910640912,0
-0.52071439148012,48.328923092502,0
-0.52057430619022,48.328943739837,0
-0.52043746048645,48.328972425791,0
-0.52030489578989,48.329008932057,0
-0.52017762094847,48.329052980819,0
-0.52005660456038,48.329104236857,0
-0.51994276760386,48.329162310103,0
-0.51983697642872,48.329226758608,0
-0.51974003616363,48.329297091901,0
-0.51965268458859,48.329372774729,0
-0.51957558651995,48.32945323112,0
-0.51950932875018,48.329537848775,0
-0.51945441558107,48.329625983721,0
-0.51941126498443,48.329716965214,0
-0.51938020541977,48.329810100844,0
-0.51936147333253,48.329904681802,0
-0.51935521135283,48.329999988275,0
-0.52099999999996,48.33,0
</coordinates>
</LinearRing>
</outerBoundaryIs>
</Polygon>
</Placemark>
<Placemark>
<name>27</name>
<styleUrl>#stylehap2</styleUrl>
<Polygon>
<outerBoundaryIs>
<LinearRing>
<coordinates>
-0.50900000000001,48.212,0
-0.50735900506251,48.211999988324,0
-0.50736524650106,48.212095294976,0
-0.50738392947009,48.212189876461,0
-0.50741491184813,48.212283012953,0
-0.50745795790362,48.212373995616,0
-0.50751274008706,48.212462132004,0
-0.50757884152213,48.21254675133,0
-0.50765575917694,48.212627209569,0
-0.50774290769104,48.212702894367,0
-0.50783962382967,48.212773229692,0

-0.50794517153066,48.212837680228,0
-0.5080587475062,48.212895755444,0
-0.50817948735657,48.212947013331,0
-0.50830647214916,48.212991063763,0
-0.5084387354132,48.213027571472,0
-0.50857527049613,48.213056258595,0
-0.50871503822658,48.213076906793,0
-0.50885697482467,48.21308935891,0
-0.50900000000001,48.213093520174,0
-0.50914302517535,48.21308935891,0
-0.50928496177346,48.213076906793,0
-0.50942472950391,48.213056258595,0
-0.50956126458685,48.213027571472,0
-0.50969352785086,48.212991063763,0
-0.50982051264348,48.212947013331,0
-0.50994125249382,48.212895755444,0
-0.51005482846939,48.212837680228,0
-0.51016037617035,48.212773229692,0
-0.51025709230898,48.212702894367,0
-0.51034424082308,48.212627209569,0
-0.51042115847788,48.21254675133,0
-0.51048725991298,48.212462132004,0
-0.5105420420964,48.212373995616,0
-0.51058508815189,48.212283012953,0
-0.51061607052993,48.212189876461,0
-0.51063475349898,48.212095294976,0
-0.51064099493751,48.211999988324,0
-0.51063474741374,48.21190468185,0
-0.51061605854438,48.211810100891,0
-0.51058507063016,48.211716965259,0
-0.51054201957092,48.211625983763,0
-0.51048723306813,48.211537848815,0
-0.51042112812936,48.211453231156,0
-0.51034420789302,48.211372774761,0
-0.51025705779792,48.21129709193,0
-0.51016034112692,48.211226758632,0
-0.51005479395833,48.211162310123,0
-0.50994121956376,48.211104236873,0
-0.50982048229496,48.211052980831,0
-0.50969350100601,48.211008932066,0
-0.50956124206134,48.210972425796,0
-0.50942471198218,48.210943739841,0
-0.50928494978789,48.210923092503,0
-0.50914301909014,48.210910640912,0
-0.50900000000001,48.210906479826,0
-0.50885698090991,48.210910640912,0
-0.50871505021213,48.210923092503,0
-0.50857528801783,48.210943739841,0
-0.50843875793867,48.210972425796,0
-0.50830649899401,48.211008932066,0
-0.50817951770506,48.211052980831,0
-0.50805878043626,48.211104236873,0
-0.50794520604171,48.211162310123,0
-0.50783965887312,48.211226758632,0
-0.5077429422021,48.21129709193,0
-0.507655792107,48.211372774761,0
-0.50757887187066,48.211453231156,0

-0.50751276693189,48.211537848815,0
-0.50745798042912,48.211625983763,0
-0.50741492936986,48.211716965259,0
-0.50738394145566,48.211810100891,0
-0.50736525258628,48.21190468185,0
-0.50735900506251,48.211999988324,0
-0.50900000000001,48.212,0
</coordinates>
</LinearRing>
</outerBoundaryIs>
</Polygon>
</Placemark>
<Placemark>
<name>28</name>
<styleUrl>#stylehap2</styleUrl>
<Polygon>
<outerBoundaryIs>
<LinearRing>
<coordinates>
-0.505,48.229,0
-0.50335846001952,48.228999988317,0
-0.50336470352929,48.229095294969,0
-0.50338339270195,48.229189876455,0
-0.50341438536889,48.229283012947,0
-0.50345744572026,48.22937399561,0
-0.50351224609787,48.229462131998,0
-0.50357836948697,48.229546751324,0
-0.50365531268854,48.229627209565,0
-0.50374249014786,48.229702894362,0
-0.50383923840978,48.229773229688,0
-0.50394482116771,48.229837680225,0
-0.50405843486707,48.229895755442,0
-0.50417921482095,48.229947013329,0
-0.50430624179157,48.229991063762,0
-0.50443854898703,48.230027571471,0
-0.50457512942045,48.230056258595,0
-0.50471494357527,48.230076906793,0
-0.50485692731821,48.23008935891,0
-0.50499999999999,48.230093520174,0
-0.50514307268178,48.23008935891,0
-0.50528505642472,48.230076906793,0
-0.50542487057954,48.230056258595,0
-0.50556145101298,48.230027571471,0
-0.50569375820842,48.229991063762,0
-0.50582078517904,48.229947013329,0
-0.50594156513291,48.229895755442,0
-0.50605517883228,48.229837680225,0
-0.50616076159021,48.229773229688,0
-0.50625750985215,48.229702894362,0
-0.50634468731144,48.229627209565,0
-0.50642163051302,48.229546751324,0
-0.50648775390211,48.229462131998,0
-0.50654255427973,48.22937399561,0
-0.5065856146311,48.229283012947,0
-0.50661660729803,48.229189876455,0
-0.50663529647069,48.229095294969,0
-0.50664153998047,48.228999988317,0

-0.5066352903798,48.228904681843,0
-0.50661659530131,48.228810100884,0
-0.50658559709309,48.228716965253,0
-0.50654253173328,48.228625983757,0
-0.50648772703233,48.228537848809,0
-0.50642160013631,48.228453231151,0
-0.50634465435077,48.228372774756,0
-0.50625747530899,48.228297091926,0
-0.50616072651418,48.228226758629,0
-0.50605514428914,48.228162310121,0
-0.50594153217224,48.228104236871,0
-0.50582075480232,48.228052980829,0
-0.50569373133864,48.228008932065,0
-0.50556142846654,48.227972425796,0
-0.50542485304152,48.22794373984,0
-0.50528504442802,48.227923092503,0
-0.50514306659089,48.227910640912,0
-0.50499999999999,48.227906479826,0
-0.5048569334091,48.227910640912,0
-0.50471495557196,48.227923092503,0
-0.50457514695846,48.22794373984,0
-0.50443857153345,48.227972425796,0
-0.50430626866135,48.228008932065,0
-0.50417924519767,48.228052980829,0
-0.50405846782775,48.228104236871,0
-0.50394485571088,48.228162310121,0
-0.5038392734858,48.228226758629,0
-0.503742524691,48.228297091926,0
-0.50365534564924,48.228372774756,0
-0.50357839986368,48.228453231151,0
-0.50351227296766,48.228537848809,0
-0.5034574682667,48.228625983757,0
-0.5034144029069,48.228716965253,0
-0.50338340469868,48.228810100884,0
-0.50336470962018,48.228904681843,0
-0.50335846001952,48.228999988317,0
-0.505,48.229,0
</coordinates>
</LinearRing>
</outerBoundaryIs>
</Polygon>
</Placemark>
<Placemark>
<name>29</name>
<styleUrl>#stylehap2</styleUrl>
<Polygon>
<outerBoundaryIs>
<LinearRing>
<coordinates>
-0.5,48.198,0
-0.49835945354132,48.19799998833,0
-0.49836569327557,48.198095294981,0
-0.49838437114006,48.198189876467,0
-0.49841534505208,48.198283012958,0
-0.49845837934446,48.198373995621,0
-0.49851314655715,48.198462132009,0
-0.49857922992777,48.198546751334,0

-0.49865612656185,48.198627209573,0
-0.49874325125892,48.19870289437,0
-0.49883994096546,48.198773229695,0
-0.49894545982044,48.19883768023,0
-0.49905900475566,48.198895755446,0
-0.49917971160756,48.198947013332,0
-0.49930666169476,48.198991063764,0
-0.49943888881057,48.199027571473,0
-0.49957538657766,48.199056258595,0
-0.49971511610868,48.199076906793,0
-0.49985701391448,48.19908935891,0
-0.49999999999999,48.199093520174,0
-0.50014298608551,48.19908935891,0
-0.50028488389133,48.199076906793,0
-0.50042461342233,48.199056258595,0
-0.50056111118942,48.199027571473,0
-0.50069333830526,48.198991063764,0
-0.50082028839245,48.198947013332,0
-0.50094099524436,48.198895755446,0
-0.50105454017954,48.19883768023,0
-0.50116005903453,48.198773229695,0
-0.50125674874107,48.19870289437,0
-0.50134387343816,48.198627209573,0
-0.50142077007222,48.198546751334,0
-0.50148685344286,48.198462132009,0
-0.50154162065556,48.198373995621,0
-0.50158465494791,48.198283012958,0
-0.50161562885993,48.198189876467,0
-0.50163430672442,48.198095294981,0
-0.5016405464587,48.19799998833,0
-0.50163430064383,48.197904681855,0
-0.50161561688354,48.197810100896,0
-0.50158463743959,48.197716965264,0
-0.50154159814728,48.197625983768,0
-0.50148682661855,48.197537848819,0
-0.50142073974693,48.197453231161,0
-0.50134384053326,48.197372774765,0
-0.5012567142564,48.197297091933,0
-0.5011600240179,48.197226758635,0
-0.50105450569488,48.197162310126,0
-0.50094096233946,48.197104236875,0
-0.50082025806713,48.197052980833,0
-0.50069331148094,48.197008932067,0
-0.50056108868117,48.196972425797,0
-0.50042459591401,48.196943739841,0
-0.50028487191494,48.196923092503,0
-0.50014298000495,48.196910640912,0
-0.49999999999999,48.196906479826,0
-0.49985701999507,48.196910640912,0
-0.49971512808507,48.196923092503,0
-0.49957540408598,48.196943739841,0
-0.49943891131885,48.196972425797,0
-0.49930668851907,48.197008932067,0
-0.49917974193286,48.197052980833,0
-0.49905903766053,48.197104236875,0
-0.49894549430511,48.197162310126,0
-0.49883997598212,48.197226758635,0

-0.49874328574359,48.197297091933,0
-0.49865615946673,48.197372774765,0
-0.49857926025308,48.197453231161,0
-0.49851317338144,48.197537848819,0
-0.49845840185271,48.197625983768,0
-0.4984153625604,48.197716965264,0
-0.49838438311648,48.197810100896,0
-0.49836569935616,48.197904681855,0
-0.49835945354132,48.19799998833,0
-0.5,48.198,0
</coordinates>
</LinearRing>
</outerBoundaryIs>
</Polygon>
</Placemark>
<Placemark>
<name>30</name>
<styleUrl>#stylehap2</styleUrl>
<Polygon>
<outerBoundaryIs>
<LinearRing>
<coordinates>
-0.495,48.215,0
-0.49335890891524,48.214999988323,0
-0.49336515071915,48.215095294974,0
-0.49338383478254,48.21518987646,0
-0.49341481897556,48.215283012952,0
-0.4934578675529,48.215373995615,0
-0.49351265294583,48.215462132003,0
-0.49357875825367,48.215546751329,0
-0.49365568041501,48.215627209569,0
-0.49374283403513,48.215702894366,0
-0.49383955584041,48.215773229691,0
-0.49394510972554,48.215837680227,0
-0.4940586923557,48.215895755444,0
-0.49417943928043,48.21594701333,0
-0.49430643151338,48.215991063763,0
-0.49443870252704,48.216027571472,0
-0.49457524560993,48.216056258595,0
-0.49471502152977,48.216076906793,0
-0.49485696644437,48.21608935891,0
-0.49499999999999,48.216093520174,0
-0.49514303355564,48.21608935891,0
-0.49528497847022,48.216076906793,0
-0.49542475439008,48.216056258595,0
-0.49556129747298,48.216027571472,0
-0.49569356848663,48.215991063763,0
-0.49582056071958,48.21594701333,0
-0.49594130764432,48.215895755444,0
-0.49605489027447,48.215837680227,0
-0.4961604441596,48.215773229691,0
-0.49625716596488,48.215702894366,0
-0.496344319585,48.215627209569,0
-0.49642124174634,48.215546751329,0
-0.49648734705416,48.215462132003,0
-0.49654213244711,48.215373995615,0
-0.49658518102443,48.215283012952,0

-0.49661616521747,48.21518987646,0
-0.49663484928084,48.215095294974,0
-0.49664109108477,48.214999988323,0
-0.49663484319461,48.214904681849,0
-0.49661615322994,48.21481010089,0
-0.49658516349985,48.214716965258,0
-0.49654210991792,48.214625983762,0
-0.49648732020493,48.214537848814,0
-0.49642121139283,48.214453231155,0
-0.49634428664954,48.21437277476,0
-0.49625713144816,48.214297091929,0
-0.4961604091104,48.214226758631,0
-0.49605485575774,48.214162310123,0
-0.49594127470886,48.214104236873,0
-0.49582053036607,48.214052980831,0
-0.49569354163741,48.214008932066,0
-0.49556127494379,48.213972425796,0
-0.49542473686548,48.213943739841,0
-0.49528496648271,48.213923092503,0
-0.49514302746941,48.213910640912,0
-0.49499999999999,48.213906479827,0
-0.49485697253061,48.213910640912,0
-0.49471503351731,48.213923092503,0
-0.49457526313453,48.213943739841,0
-0.49443872505623,48.213972425796,0
-0.49430645836261,48.214008932066,0
-0.49417946963392,48.214052980831,0
-0.49405872529116,48.214104236873,0
-0.49394514424227,48.214162310123,0
-0.49383959088962,48.214226758631,0
-0.49374286855186,48.214297091929,0
-0.49365571335047,48.21437277476,0
-0.49357878860718,48.214453231155,0
-0.49351267979508,48.214537848814,0
-0.4934578900821,48.214625983762,0
-0.49341483650016,48.214716965258,0
-0.49338384677008,48.21481010089,0
-0.49336515680538,48.214904681849,0
-0.49335890891524,48.214999988323,0
-0.495,48.215,0
</coordinates>
</LinearRing>
</outerBoundaryIs>
</Polygon>
</Placemark>
<Placemark>
<name>31</name>
<styleUrl>#stylehap2</styleUrl>
<Polygon>
<outerBoundaryIs>
<LinearRing>
<coordinates>
-0.48800000000006,48.219,0
-0.48635878069444,48.218999988321,0
-0.48636502298561,48.219095294973,0
-0.48638370850837,48.219189876459,0
-0.48641469512187,48.21928301295,0

-0.48645774706228,48.219373995614,0
-0.48651253673539,48.219462132002,0
-0.48657864720789,48.219546751327,0
-0.48665557537908,48.219627209567,0
-0.48674273580854,48.219702894365,0
-0.48683946517079,48.21977322969,0
-0.48694502730303,48.219837680227,0
-0.48705861880767,48.219895755443,0
-0.48717937516674,48.21994701333,0
-0.487306377322,48.219991063763,0
-0.4874386586705,48.220027571472,0
-0.48757521242207,48.220056258595,0
-0.4877149992632,48.220076906793,0
-0.48785695526857,48.22008935891,0
-0.48800000000005,48.220093520174,0
-0.48814304473153,48.22008935891,0
-0.4882850007369,48.220076906793,0
-0.48842478757806,48.220056258595,0
-0.4885613413296,48.220027571472,0
-0.4886936226781,48.219991063763,0
-0.48882062483339,48.21994701333,0
-0.48894138119244,48.219895755443,0
-0.48905497269707,48.219837680227,0
-0.48916053482933,48.21977322969,0
-0.48925726419159,48.219702894365,0
-0.48934442462104,48.219627209567,0
-0.48942135279224,48.219546751327,0
-0.48948746326471,48.219462132002,0
-0.48954225293783,48.219373995614,0
-0.48958530487826,48.21928301295,0
-0.48961629149173,48.219189876459,0
-0.48963497701452,48.219095294973,0
-0.48964121930568,48.218999988321,0
-0.48963497092696,48.218904681847,0
-0.48961627950158,48.218810100888,0
-0.48958528734982,48.218716965256,0
-0.48954223040372,48.218625983761,0
-0.48948743640961,48.218537848812,0
-0.48942132243211,48.218453231154,0
-0.48934439167836,48.218372774759,0
-0.4892572296673,48.218297091928,0
-0.48916049977247,48.218226758631,0
-0.48905493817281,48.218162310122,0
-0.48894134824978,48.218104236872,0
-0.48882059447326,48.218052980831,0
-0.488693595823,48.218008932066,0
-0.4885613187955,48.217972425796,0
-0.48842477004961,48.217943739841,0
-0.48828498874675,48.217923092503,0
-0.48814303864397,48.217910640912,0
-0.48800000000005,48.217906479827,0
-0.48785696135613,48.217910640912,0
-0.48771501125336,48.217923092503,0
-0.48757522995049,48.217943739841,0
-0.4874386812046,48.217972425796,0
-0.48730640417711,48.218008932066,0
-0.48717940552686,48.218052980831,0

-0.48705865175035,48.218104236872,0
-0.48694506182731,48.218162310122,0
-0.48683950022766,48.218226758631,0
-0.4867427703328,48.218297091928,0
-0.48665560832174,48.218372774759,0
-0.48657867756802,48.218453231154,0
-0.48651256359049,48.218537848812,0
-0.4864577695964,48.218625983761,0
-0.48641471265029,48.218716965256,0
-0.48638372049853,48.218810100888,0
-0.48636502907317,48.218904681847,0
-0.48635878069444,48.218999988321,0
-0.48800000000006,48.219,0
</coordinates>
</LinearRing>
</outerBoundaryIs>
</Polygon>
</Placemark>
<Placemark>
<name>32</name>
<styleUrl>#stylehap2</styleUrl>
<Polygon>
<outerBoundaryIs>
<LinearRing>
<coordinates>
-0.48299999999995,48.205,0
-0.48135922934473,48.204999988327,0
-0.481365469931,48.205095294978,0
-0.48138415034726,48.205189876464,0
-0.48141512849148,48.205283012956,0
-0.48145816866429,48.205373995619,0
-0.48151294336091,48.205462132006,0
-0.48157903576203,48.205546751332,0
-0.48165594290447,48.205627209571,0
-0.48174307950778,48.205702894368,0
-0.48183978242784,48.205773229693,0
-0.48194531570306,48.205837680229,0
-0.48205887615548,48.205895755445,0
-0.48217959950345,48.205947013331,0
-0.48230656694003,48.205991063764,0
-0.48243881212649,48.206027571472,0
-0.48257532854794,48.206056258595,0
-0.48271507717501,48.206076906793,0
-0.48285699437326,48.20608935891,0
-0.48299999999995,48.206093520173,0
-0.48314300562664,48.20608935891,0
-0.48328492282489,48.206076906793,0
-0.48342467145196,48.206056258595,0
-0.48356118787341,48.206027571472,0
-0.48369343305987,48.205991063764,0
-0.48382040049645,48.205947013331,0
-0.48394112384442,48.205895755445,0
-0.48405468429682,48.205837680229,0
-0.48416021757206,48.205773229693,0
-0.4842569204921,48.205702894368,0
-0.48434405709543,48.205627209571,0
-0.48442096423784,48.205546751332,0

-0.48448705663899,48.205462132006,0
-0.48454183133561,48.205373995619,0
-0.48458487150842,48.205283012956,0
-0.48461584965264,48.205189876464,0
-0.4846345300689,48.205095294978,0
-0.48464077065516,48.204999988327,0
-0.484634523986,48.204904681853,0
-0.48461583767164,48.204810100893,0
-0.48458485399339,48.204716965262,0
-0.48454180881873,48.204625983766,0
-0.48448702980442,48.204537848817,0
-0.48442093390095,48.204453231158,0
-0.48434402417796,48.204372774763,0
-0.48425688599425,48.204297091932,0
-0.48416018254201,48.204226758633,0
-0.48405464979897,48.204162310125,0
-0.48394109092695,48.204104236874,0
-0.48382037015956,48.204052980832,0
-0.4836934062253,48.204008932067,0
-0.48356116535653,48.203972425797,0
-0.48342465393693,48.203943739841,0
-0.48328491084389,48.203923092503,0
-0.48314299954374,48.203910640912,0
-0.48299999999995,48.203906479826,0
-0.48285700045616,48.203910640912,0
-0.48271508915598,48.203923092503,0
-0.48257534606295,48.203943739841,0
-0.48243883464337,48.203972425797,0
-0.4823065937746,48.204008932067,0
-0.48217962984034,48.204052980832,0
-0.48205890907295,48.204104236874,0
-0.48194535020093,48.204162310125,0
-0.48183981745788,48.204226758633,0
-0.48174311400565,48.204297091932,0
-0.48165597582194,48.204372774763,0
-0.48157906609895,48.204453231158,0
-0.48151297019548,48.204537848817,0
-0.48145819118117,48.204625983766,0
-0.48141514600651,48.204716965262,0
-0.48138416232823,48.204810100893,0
-0.4813654760139,48.204904681853,0
-0.48135922934473,48.204999988327,0
-0.48299999999995,48.205,0
</coordinates>
</LinearRing>
</outerBoundaryIs>
</Polygon>
</Placemark>
<Placemark>
<name>33</name>
<styleUrl>#stylehap2</styleUrl>
<Polygon>
<outerBoundaryIs>
<LinearRing>
<coordinates>
-0.47800000000007,48.275,0
-0.47635698265475,48.274999988298,0

-0.47636323177866,48.27509529495,0
-0.47638193776659,48.275189876436,0
-0.47641295832202,48.275283012929,0
-0.47645605742311,48.275373995593,0
-0.47651090711685,48.275462131983,0
-0.47657709001328,48.27554675131,0
-0.47665410246068,48.275627209552,0
-0.47674135837728,48.275702894351,0
-0.47683819371098,48.275773229679,0
-0.47694387149246,48.275837680217,0
-0.47705758744396,48.275895755436,0
-0.47717847610034,48.275947013324,0
-0.47730561739643,48.275991063758,0
-0.47743804367015,48.276027571469,0
-0.47757474702827,48.276056258593,0
-0.4777146870185,48.276076906792,0
-0.47785679854977,48.27608935891,0
-0.47800000000008,48.276093520174,0
-0.47814320145036,48.27608935891,0
-0.47828531298163,48.276076906792,0
-0.47842525297186,48.276056258593,0
-0.47856195632998,48.276027571469,0
-0.4786943826037,48.275991063758,0
-0.47882152389979,48.275947013324,0
-0.47894241255617,48.275895755436,0
-0.47905612850767,48.275837680217,0
-0.47916180628915,48.275773229679,0
-0.47925864162285,48.275702894351,0
-0.47934589753945,48.275627209552,0
-0.47942290998685,48.27554675131,0
-0.4794890928833,48.275462131983,0
-0.47954394257702,48.275373995593,0
-0.47958704167811,48.275283012929,0
-0.47961806223357,48.275189876436,0
-0.47963676822146,48.27509529495,0
-0.47964301734538,48.274999988298,0
-0.47963676211523,48.274904681824,0
-0.47961805020664,48.274810100866,0
-0.47958702409592,48.274716965235,0
-0.47954391997378,48.274625983741,0
-0.47948906594581,48.274537848793,0
-0.47942287953359,48.274453231137,0
-0.47934586449576,48.274372774744,0
-0.47925860699269,48.274297091915,0
-0.47916177112478,48.274226758619,0
-0.47905609387751,48.274162310113,0
-0.47894237951247,48.274104236865,0
-0.47882149344656,48.274052980825,0
-0.47869435566624,48.274008932061,0
-0.47856193372674,48.273972425793,0
-0.47842523538967,48.273943739839,0
-0.4782853009547,48.273923092502,0
-0.47814319534413,48.273910640912,0
-0.47800000000008,48.273906479826,0
-0.477856804656,48.273910640912,0
-0.47771469904543,48.273923092502,0
-0.47757476461048,48.273943739839,0

-0.47743806627339,48.273972425793,0
-0.47730564433389,48.274008932061,0
-0.47717850655359,48.274052980825,0
-0.47705762048768,48.274104236865,0
-0.47694390612262,48.274162310113,0
-0.47683822887535,48.274226758619,0
-0.47674139300744,48.274297091915,0
-0.47665413550437,48.274372774744,0
-0.47657712046653,48.274453231137,0
-0.47651093405432,48.274537848793,0
-0.47645608002635,48.274625983741,0
-0.4764129759042,48.274716965235,0
-0.47638194979351,48.274810100866,0
-0.4763632378849,48.274904681824,0
-0.47635698265475,48.274999988298,0
-0.47800000000007,48.275,0
</coordinates>
</LinearRing>
</outerBoundaryIs>
</Polygon>
</Placemark>
<Placemark>
<name>34</name>
<styleUrl>#stylehap2</styleUrl>
<Polygon>
<outerBoundaryIs>
<LinearRing>
<coordinates>
-0.47500000000002,48.27,0
-0.47335714341797,48.2699999883,0
-0.47336339193096,48.270095294952,0
-0.47338209608907,48.270189876438,0
-0.47341311360975,48.270283012931,0
-0.47345620849419,48.270373995595,0
-0.47351105282147,48.270462131985,0
-0.47357722924247,48.270546751312,0
-0.47365423415469,48.270627209553,0
-0.47374148153377,48.270702894353,0
-0.47383830739253,48.27077322968,0
-0.47394397483379,48.270837680218,0
-0.47405767965844,48.270895755436,0
-0.47417855648608,48.270947013325,0
-0.47430568534154,48.270991063759,0
-0.47443809865749,48.271027571469,0
-0.47457478863924,48.271056258593,0
-0.47471471493636,48.271076906792,0
-0.47485681256199,48.27108935891,0
-0.47500000000002,48.271093520174,0
-0.47514318743805,48.27108935891,0
-0.47528528506368,48.271076906792,0
-0.47542521136081,48.271056258593,0
-0.47556190134258,48.271027571469,0
-0.4756943146585,48.270991063759,0
-0.47582144351396,48.270947013325,0
-0.47594232034161,48.270895755436,0
-0.47605602516625,48.270837680218,0
-0.47616169260752,48.27077322968,0

-0.47625851846627,48.270702894353,0
-0.47634576584535,48.270627209553,0
-0.47642277075758,48.270546751312,0
-0.47648894717857,48.270462131985,0
-0.47654379150586,48.270373995595,0
-0.47658688639029,48.270283012931,0
-0.47661790391097,48.270189876438,0
-0.47663660806908,48.270095294952,0
-0.4766428565821,48.2699999883,0
-0.47663660196453,48.269904681826,0
-0.47661789188736,48.269810100868,0
-0.47658686881292,48.269716965237,0
-0.4765437689088,48.269625983742,0
-0.47648892024846,48.269537848795,0
-0.47642274031267,48.269453231138,0
-0.47634573281069,48.269372774745,0
-0.4762584838456,48.269297091916,0
-0.47616165745277,48.26922675862,0
-0.47605599054558,48.269162310114,0
-0.47594228730694,48.269104236865,0
-0.47582141306905,48.269052980825,0
-0.47569428772839,48.269008932062,0
-0.47556187874553,48.268972425794,0
-0.47542519378343,48.268943739839,0
-0.47528527304006,48.268923092502,0
-0.47514318133347,48.268910640912,0
-0.47500000000002,48.268906479826,0
-0.47485681866657,48.268910640912,0
-0.47471472696,48.268923092502,0
-0.47457480621661,48.268943739839,0
-0.47443812125451,48.268972425794,0
-0.47430571227165,48.269008932062,0
-0.47417858693099,48.269052980825,0
-0.47405771269313,48.269104236865,0
-0.47394400945446,48.269162310114,0
-0.47383834254727,48.26922675862,0
-0.47374151615444,48.269297091916,0
-0.47365426718936,48.269372774745,0
-0.47357725968737,48.269453231138,0
-0.47351107975158,48.269537848795,0
-0.47345623109124,48.269625983742,0
-0.47341313118712,48.269716965237,0
-0.47338210811269,48.269810100868,0
-0.47336339803551,48.269904681826,0
-0.47335714341797,48.2699999883,0
-0.47500000000002,48.27,0
</coordinates>
</LinearRing>
</outerBoundaryIs>
</Polygon>
</Placemark>
<Placemark>
<name>35</name>
<styleUrl>#stylehap2</styleUrl>
<Polygon>
<outerBoundaryIs>
<LinearRing>

<coordinates>
-0.47399999999993,48.269,0
-0.47235717556525,48.268999988301,0
-0.47236342395608,48.269095294952,0
-0.47238212774828,48.269189876439,0
-0.47241314466211,48.269283012931,0
-0.47245623870336,48.269373995595,0
-0.47251108195753,48.269462131985,0
-0.47257725708363,48.269546751312,0
-0.47265426048909,48.269627209554,0
-0.47274150616095,48.269702894353,0
-0.47283833012501,48.26977322968,0
-0.47294399549858,48.269837680218,0
-0.47305769809824,48.269895755437,0
-0.47317857256052,48.269947013325,0
-0.47330569892825,48.269991063759,0
-0.47343810965306,48.270027571469,0
-0.47357479695996,48.270056258593,0
-0.47371472051894,48.270076906792,0
-0.4738568153639,48.27008935891,0
-0.47399999999993,48.270093520174,0
-0.47414318463595,48.27008935891,0
-0.47428527948093,48.270076906792,0
-0.47442520303988,48.270056258593,0
-0.47456189034681,48.270027571469,0
-0.47469430107159,48.269991063759,0
-0.47482142743935,48.269947013325,0
-0.47494230190163,48.269895755437,0
-0.47505600450129,48.269837680218,0
-0.47516166987484,48.26977322968,0
-0.47525849383892,48.269702894353,0
-0.47534573951078,48.269627209554,0
-0.47542274291621,48.269546751312,0
-0.47548891804234,48.269462131985,0
-0.47554376129651,48.269373995595,0
-0.47558685533774,48.269283012931,0
-0.47561787225159,48.269189876439,0
-0.47563657604379,48.269095294952,0
-0.47564282443462,48.268999988301,0
-0.47563656993956,48.268904681827,0
-0.47561786022861,48.268810100868,0
-0.47558683776133,48.268716965237,0
-0.4755437387007,48.268625983743,0
-0.47548889111371,48.268537848795,0
-0.47542271247296,48.268453231139,0
-0.47534570647792,48.268372774745,0
-0.47525845922014,48.268297091916,0
-0.475161634722,48.26822675862,0
-0.4750559698825,48.268162310114,0
-0.47494226886877,48.268104236865,0
-0.4748213969961,48.268052980825,0
-0.47469427414296,48.268008932062,0
-0.474561867751,48.267972425794,0
-0.47442518546347,48.267943739839,0
-0.47428526745795,48.267923092502,0
-0.47414317853173,48.267910640912,0
-0.47399999999993,48.267906479827,0

-0.47385682146814,48.267910640912,0
-0.47371473254192,48.267923092502,0
-0.4735748145364,48.267943739839,0
-0.47343813224887,48.267972425794,0
-0.47330572585689,48.268008932062,0
-0.47317860300377,48.268052980825,0
-0.4730577311311,48.268104236865,0
-0.47294403011737,48.268162310114,0
-0.47283836527785,48.26822675862,0
-0.47274154077973,48.268297091916,0
-0.47265429352195,48.268372774745,0
-0.47257728752688,48.268453231139,0
-0.47251110888616,48.268537848795,0
-0.47245626129917,48.268625983743,0
-0.47241316223854,48.268716965237,0
-0.47238213977126,48.268810100868,0
-0.47236343006031,48.268904681827,0
-0.47235717556525,48.268999988301,0
-0.47399999999993,48.269,0
</coordinates>
</LinearRing>
</outerBoundaryIs>
</Polygon>
</Placemark>
<Placemark>
<name>36</name>
<styleUrl>#stylehap2</styleUrl>
<Polygon>
<outerBoundaryIs>
<LinearRing>
<coordinates>
-0.46199999999999,48.226,0
-0.46035855624038,48.225999988318,0
-0.46036479938449,48.22609529497,0
-0.460383487462,48.226189876456,0
-0.46041447831253,48.226283012948,0
-0.46045753614015,48.226373995611,0
-0.46051233330578,48.226462131999,0
-0.46057845281915,48.226546751325,0
-0.46065539151075,48.226627209565,0
-0.46074256386014,48.226702894363,0
-0.46083930645108,48.226773229689,0
-0.46094488302013,48.226837680226,0
-0.46105849005977,48.226895755442,0
-0.46117926293386,48.226947013329,0
-0.46130628245844,48.226991063762,0
-0.46143858189834,48.227027571471,0
-0.46157515432567,48.227056258595,0
-0.46171496028483,48.227076906793,0
-0.46185693570489,48.22708935891,0
-0.46199999999999,48.227093520174,0
-0.46214306429509,48.22708935891,0
-0.46228503971515,48.227076906793,0
-0.46242484567429,48.227056258595,0
-0.46256141810164,48.227027571471,0
-0.46269371754154,48.226991063762,0
-0.46282073706612,48.226947013329,0

-0.46294150994019,48.226895755442,0
-0.46305511697985,48.226837680226,0
-0.4631606935489,48.226773229689,0
-0.46325743613985,48.226702894363,0
-0.46334460848923,48.226627209565,0
-0.46342154718083,48.226546751325,0
-0.4634876666942,48.226462131999,0
-0.46354246385984,48.226373995611,0
-0.46358552168745,48.226283012948,0
-0.46361651253798,48.226189876456,0
-0.46363520061549,48.22609529497,0
-0.46364144375961,48.225999988318,0
-0.46363519452559,48.225904681844,0
-0.46361650054325,48.225810100885,0
-0.46358550415229,48.225716965254,0
-0.46354244131708,48.225625983758,0
-0.46348763982879,48.22553784881,0
-0.46342151680908,48.225453231152,0
-0.46334457553395,48.225372774757,0
-0.46325740160238,48.225297091926,0
-0.46316065847863,48.225226758629,0
-0.46305508244238,48.225162310121,0
-0.46294147698491,48.225104236871,0
-0.46282070669437,48.22505298083,0
-0.46269369067613,48.225008932065,0
-0.46256139555892,48.224972425796,0
-0.46242482813915,48.22494373984,0
-0.46228502772042,48.224923092503,0
-0.46214305820519,48.224910640912,0
-0.46199999999999,48.224906479827,0
-0.46185694179479,48.224910640912,0
-0.46171497227957,48.224923092503,0
-0.4615751718608,48.22494373984,0
-0.46143860444107,48.224972425796,0
-0.46130630932385,48.225008932065,0
-0.46117929330562,48.22505298083,0
-0.46105852301505,48.225104236871,0
-0.4609449175576,48.225162310121,0
-0.46083934152135,48.225226758629,0
-0.4607425983976,48.225297091926,0
-0.46065542446603,48.225372774757,0
-0.4605784831909,48.225453231152,0
-0.46051236017116,48.22553784881,0
-0.4604575586829,48.225625983758,0
-0.46041449584767,48.225716965254,0
-0.46038349945674,48.225810100885,0
-0.46036480547439,48.225904681844,0
-0.46035855624038,48.225999988318,0
-0.46199999999999,48.226,0
</coordinates>
</LinearRing>
</outerBoundaryIs>
</Polygon>
</Placemark>
<Placemark>
<name>37</name>
<styleUrl>#stylehap2</styleUrl>

<Polygon>
<outerBoundaryIs>
<LinearRing>
<coordinates>
-0.46100000000001,48.221,0
-0.45935871657345,48.22099998832,0
-0.45936495910828,48.221095294972,0
-0.45938364536088,48.221189876458,0
-0.45941463318477,48.22128301295,0
-0.45945768680703,48.221373995613,0
-0.45951247862056,48.221462132001,0
-0.45957859167582,48.221546751327,0
-0.45965552285243,48.221627209567,0
-0.45974268668712,48.221702894364,0
-0.45983941982846,48.22177322969,0
-0.45994498608495,48.221837680226,0
-0.46005858202756,48.221895755443,0
-0.46017934310457,48.22194701333,0
-0.4603063502218,48.221991063762,0
-0.46043863673857,48.222027571471,0
-0.46057519582533,48.222056258595,0
-0.46071498812803,48.222076906793,0
-0.46085694967968,48.22208935891,0
-0.46100000000001,48.222093520174,0
-0.46114305032033,48.22208935891,0
-0.46128501187201,48.222076906793,0
-0.46142480417468,48.222056258595,0
-0.46156136326147,48.222027571471,0
-0.46169364977821,48.221991063762,0
-0.46182065689547,48.22194701333,0
-0.46194141797245,48.221895755443,0
-0.46205501391506,48.221837680226,0
-0.46216058017156,48.22177322969,0
-0.46225731331292,48.221702894364,0
-0.46234447714761,48.221627209567,0
-0.46242140832422,48.221546751327,0
-0.46248752137946,48.221462132001,0
-0.46254231319301,48.221373995613,0
-0.46258536681524,48.22128301295,0
-0.46261635463916,48.221189876458,0
-0.46263504089174,48.221095294972,0
-0.46264128342659,48.22099998832,0
-0.46263503480352,48.220904681846,0
-0.46261634264768,48.220810100887,0
-0.46258534928489,48.220716965256,0
-0.46254229065641,48.22062598376,0
-0.4624874945214,48.220537848812,0
-0.46242137796076,48.220453231154,0
-0.46234444420133,48.220372774759,0
-0.46225727878487,48.220297091928,0
-0.46216054511085,48.22022675863,0
-0.46205497938703,48.220162310122,0
-0.46194138502618,48.220104236872,0
-0.46182062653201,48.22005298083,0
-0.46169362292016,48.220008932065,0
-0.46156134072487,48.219972425796,0
-0.46142478664433,48.21994373984,0

-0.46128499988055,48.219923092503,0
-0.46114304423211,48.219910640912,0
-0.46100000000001,48.219906479826,0
-0.46085695576793,48.219910640912,0
-0.46071500011949,48.219923092503,0
-0.46057521335568,48.21994373984,0
-0.46043865927514,48.219972425796,0
-0.46030637707986,48.220008932065,0
-0.460179373468,48.22005298083,0
-0.46005861497383,48.220104236872,0
-0.45994502061301,48.220162310122,0
-0.45983945488916,48.22022675863,0
-0.45974272121514,48.220297091928,0
-0.45965555579871,48.220372774759,0
-0.45957862203925,48.220453231154,0
-0.45951250547861,48.220537848812,0
-0.4594577093436,48.22062598376,0
-0.45941465071512,48.220716965256,0
-0.45938365735233,48.220810100887,0
-0.45936496519649,48.220904681846,0
-0.45935871657345,48.22099998832,0
-0.46100000000001,48.221,0
</coordinates>
</LinearRing>
</outerBoundaryIs>
</Polygon>
</Placemark>
<Placemark>
<name>38</name>
<styleUrl>#stylehap2</styleUrl>
<Polygon>
<outerBoundaryIs>
<LinearRing>
<coordinates>
-0.452,48.221,0
-0.45035871657344,48.22099998832,0
-0.45036495910826,48.221095294972,0
-0.45038364536087,48.221189876458,0
-0.45041463318476,48.22128301295,0
-0.45045768680702,48.221373995613,0
-0.45051247862054,48.221462132001,0
-0.45057859167581,48.221546751327,0
-0.45065552285242,48.221627209567,0
-0.4507426866871,48.221702894364,0
-0.45083941982847,48.22177322969,0
-0.45094498608494,48.221837680226,0
-0.45105858202755,48.221895755443,0
-0.45117934310455,48.22194701333,0
-0.45130635022179,48.221991063762,0
-0.45143863673856,48.222027571471,0
-0.45157519582532,48.222056258595,0
-0.45171498812802,48.222076906793,0
-0.45185694967967,48.22208935891,0
-0.45199999999999,48.222093520174,0
-0.45214305032032,48.22208935891,0
-0.45228501187199,48.222076906793,0
-0.45242480417467,48.222056258595,0

-0.45256136326145,48.222027571471,0
-0.4526936497782,48.221991063762,0
-0.45282065689546,48.22194701333,0
-0.45294141797244,48.221895755443,0
-0.45305501391504,48.221837680226,0
-0.45316058017154,48.22177322969,0
-0.45325731331291,48.221702894364,0
-0.45334447714759,48.221627209567,0
-0.4534214083242,48.221546751327,0
-0.45348752137944,48.221462132001,0
-0.45354231319299,48.221373995613,0
-0.45358536681523,48.22128301295,0
-0.45361635463914,48.221189876458,0
-0.45363504089172,48.221095294972,0
-0.45364128342657,48.22099998832,0
-0.45363503480351,48.220904681846,0
-0.45361634264766,48.220810100887,0
-0.45358534928488,48.220716965256,0
-0.4535422906564,48.22062598376,0
-0.45348749452139,48.220537848812,0
-0.45342137796075,48.220453231154,0
-0.45334444420132,48.220372774759,0
-0.45325727878485,48.220297091928,0
-0.45316054511084,48.22022675863,0
-0.45305497938702,48.220162310122,0
-0.45294138502617,48.220104236872,0
-0.452820626532,48.22005298083,0
-0.45269362292014,48.220008932065,0
-0.45256134072486,48.219972425796,0
-0.45242478664431,48.21994373984,0
-0.45228499988054,48.219923092503,0
-0.4521430442321,48.219910640912,0
-0.45199999999999,48.219906479826,0
-0.45185695576791,48.219910640912,0
-0.45171500011947,48.219923092503,0
-0.45157521335567,48.21994373984,0
-0.45143865927513,48.219972425796,0
-0.45130637707984,48.220008932065,0
-0.45117937346799,48.22005298083,0
-0.45105861497382,48.220104236872,0
-0.45094502061299,48.220162310122,0
-0.45083945488915,48.22022675863,0
-0.45074272121513,48.220297091928,0
-0.45065555579869,48.220372774759,0
-0.45057862203924,48.220453231154,0
-0.4505125054786,48.220537848812,0
-0.45045770934359,48.22062598376,0
-0.45041465071511,48.220716965256,0
-0.45038365735232,48.220810100887,0
-0.45036496519648,48.220904681846,0
-0.45035871657344,48.22099998832,0
-0.452,48.221,0
</coordinates>
</LinearRing>
</outerBoundaryIs>
</Polygon>
</Placemark>

<Placemark>
<name>39</name>
<styleUrl>#stylehap2</styleUrl>
<Polygon>
<outerBoundaryIs>
<LinearRing>
<coordinates>
-0.43000000000006,48.284,0
-0.42835669317007,48.283999988294,0
-0.42836294339405,48.284095294946,0
-0.42838165267685,48.284189876433,0
-0.42841267869696,48.284283012926,0
-0.42845578539093,48.28437399559,0
-0.42851064474798,48.28446213198,0
-0.42857683930471,48.284546751307,0
-0.42865386532058,48.284627209549,0
-0.42874113661063,48.284702894349,0
-0.42883798900572,48.284773229677,0
-0.42894368540678,48.284837680216,0
-0.42905742139424,48.284895755434,0
-0.4291783313505,48.284947013323,0
-0.42930549504827,48.284991063758,0
-0.42943794465501,48.285027571468,0
-0.4295746720998,48.285056258593,0
-0.42971463674706,48.285076906792,0
-0.42985677331802,48.28508935891,0
-0.43000000000008,48.285093520174,0
-0.43014322668211,48.28508935891,0
-0.43028536325306,48.285076906792,0
-0.43042532790033,48.285056258593,0
-0.43056205534512,48.285027571468,0
-0.43069450495185,48.284991063758,0
-0.43082166864963,48.284947013323,0
-0.43094257860591,48.284895755434,0
-0.43105631459335,48.284837680216,0
-0.4311620109944,48.284773229677,0
-0.43125886338949,48.284702894349,0
-0.43134613467955,48.284627209549,0
-0.43142316069544,48.284546751307,0
-0.43148935525214,48.28446213198,0
-0.4315442146092,48.28437399559,0
-0.43158732130316,48.284283012926,0
-0.43161834732327,48.284189876433,0
-0.43163705660607,48.284095294946,0
-0.43164330683005,48.283999988294,0
-0.43163705049684,48.28390468182,0
-0.43161833529042,48.283810100862,0
-0.43158730371233,48.283716965231,0
-0.43154419199484,48.283625983737,0
-0.43148932830139,48.28353784879,0
-0.43142313022718,48.283453231134,0
-0.43134610161959,48.283372774741,0
-0.43125882874228,48.283297091912,0
-0.43116197581271,48.283226758617,0
-0.43105627994614,48.283162310111,0
-0.43094254554592,48.283104236863,0
-0.43082163818139,48.283052980824,0

-0.43069447800111,48.283008932061,0
-0.43056203273076,48.282972425793,0
-0.43042531030947,48.282943739839,0
-0.43028535122021,48.282923092502,0
-0.43014322057287,48.282910640912,0
-0.43000000000008,48.282906479827,0
-0.42985677942725,48.282910640912,0
-0.42971464877991,48.282923092502,0
-0.42957468969066,48.282943739839,0
-0.42943796726936,48.282972425793,0
-0.42930552199902,48.283008932061,0
-0.42917836181874,48.283052980824,0
-0.4290574544542,48.283104236863,0
-0.42894372005399,48.283162310111,0
-0.42883802418744,48.283226758617,0
-0.42874117125784,48.283297091912,0
-0.42865389838056,48.283372774741,0
-0.42857686977295,48.283453231134,0
-0.42851067169873,48.28353784879,0
-0.42845580800528,48.283625983737,0
-0.4284126962878,48.283716965231,0
-0.4283816647097,48.283810100862,0
-0.42836294950329,48.28390468182,0
-0.42835669317007,48.283999988294,0
-0.43000000000006,48.284,0
</coordinates>
</LinearRing>
</outerBoundaryIs>
</Polygon>
</Placemark>
<Placemark>
<name>40</name>
<styleUrl>#stylehap2</styleUrl>
<Polygon>
<outerBoundaryIs>
<LinearRing>
<coordinates>
-0.428,48.264,0
-0.42635733627579,48.263999988303,0
-0.42636358405591,48.264095294954,0
-0.42638228601893,48.264189876441,0
-0.42641329989899,48.264283012933,0
-0.42645638972497,48.264373995597,0
-0.42651122761443,48.264462131987,0
-0.42657739626724,48.264546751314,0
-0.42665439214,48.264627209555,0
-0.42674162927711,48.264702894354,0
-0.42683844376937,48.264773229681,0
-0.42694409880607,48.264837680219,0
-0.42705779028256,48.264895755437,0
-0.42717865291997,48.264947013325,0
-0.4273057668512,48.264991063759,0
-0.42743816462243,48.265027571469,0
-0.42757483855739,48.265056258594,0
-0.42771474842771,48.265076906792,0
-0.42785682937164,48.26508935891,0
-0.428,48.265093520174,0

-0.42814317062835,48.26508935891,0
-0.42828525157227,48.265076906792,0
-0.42842516144262,48.265056258594,0
-0.42856183537756,48.265027571469,0
-0.42869423314881,48.264991063759,0
-0.42882134708001,48.264947013325,0
-0.42894220971742,48.264895755437,0
-0.42905590119391,48.264837680219,0
-0.42916155623063,48.264773229681,0
-0.42925837072287,48.264702894354,0
-0.42934560786001,48.264627209555,0
-0.42942260373277,48.264546751314,0
-0.42948877238555,48.264462131987,0
-0.42954361027501,48.264373995597,0
-0.42958670010099,48.264283012933,0
-0.42961771398108,48.264189876441,0
-0.4296364159441,48.264095294954,0
-0.42964266372421,48.263999988303,0
-0.42963640984153,48.263904681829,0
-0.42961770196138,48.26381010087,0
-0.42958668252939,48.263716965239,0
-0.42954358768539,48.263625983745,0
-0.42948874546429,48.263537848797,0
-0.42942257329783,48.26345323114,0
-0.42934557483618,48.263372774747,0
-0.42925833611355,48.263297091917,0
-0.42916152108741,48.263226758621,0
-0.42905586658459,48.263162310115,0
-0.42894217669359,48.263104236866,0
-0.42882131664508,48.263052980826,0
-0.42869420622752,48.263008932062,0
-0.42856181278793,48.262972425794,0
-0.428425143871,48.262943739839,0
-0.42828523955257,48.262923092502,0
-0.4281431645258,48.262910640912,0
-0.428,48.262906479826,0
-0.4278568354742,48.262910640912,0
-0.42771476044741,48.262923092502,0
-0.42757485612899,48.262943739839,0
-0.42743818721205,48.262972425794,0
-0.42730579377246,48.263008932062,0
-0.42717868335491,48.263052980826,0
-0.42705782330639,48.263104236866,0
-0.42694413341539,48.263162310115,0
-0.4268384789126,48.263226758621,0
-0.42674166388643,48.263297091917,0
-0.42665442516383,48.263372774747,0
-0.42657742670217,48.26345323114,0
-0.42651125453571,48.263537848797,0
-0.42645641231462,48.263625983745,0
-0.42641331747062,48.263716965239,0
-0.42638229803863,48.26381010087,0
-0.42636359015845,48.263904681829,0
-0.42635733627579,48.263999988303,0
-0.428,48.264,0
</coordinates>
</LinearRing>

</outerBoundaryIs>
</Polygon>
</Placemark>
<Placemark>
<name>41</name>
<styleUrl>#stylehap2</styleUrl>
<Polygon>
<outerBoundaryIs>
<LinearRing>
<coordinates>
-0.40499999999997,48.237,0
-0.40335820335337,48.236999988314,0
-0.40336444783851,48.237095294965,0
-0.40338313993252,48.237189876451,0
-0.40341413744458,48.237283012944,0
-0.40345720452804,48.237373995607,0
-0.40351201347346,48.237462131996,0
-0.40357814720091,48.237546751322,0
-0.40365510243272,48.237627209562,0
-0.40374229352262,48.237702894361,0
-0.40383905691172,48.237773229687,0
-0.40394465617833,48.237837680224,0
-0.40405828764218,48.237895755441,0
-0.4041790864812,48.237947013328,0
-0.40430613331387,48.237991063761,0
-0.40443846119709,48.238027571471,0
-0.40457506298651,48.238056258594,0
-0.40471489900301,48.238076906793,0
-0.40485690494693,48.23808935891,0
-0.40499999999997,48.238093520174,0
-0.40514309505302,48.23808935891,0
-0.40528510099694,48.238076906793,0
-0.40542493701344,48.238056258594,0
-0.40556153880286,48.238027571471,0
-0.40569386668608,48.237991063761,0
-0.40582091351872,48.237947013328,0
-0.40594171235777,48.237895755441,0
-0.40605534382162,48.237837680224,0
-0.40616094308823,48.237773229687,0
-0.40625770647733,48.237702894361,0
-0.40634489756721,48.237627209562,0
-0.40642185279904,48.237546751322,0
-0.40648798652649,48.237462131996,0
-0.40654279547191,48.237373995607,0
-0.40658586255537,48.237283012944,0
-0.40661686006743,48.237189876451,0
-0.40663555216144,48.237095294965,0
-0.40664179664658,48.236999988314,0
-0.40663554606788,48.23690468184,0
-0.40661684806547,48.236810100881,0
-0.40658584500967,48.236716965249,0
-0.40654277291559,48.236625983754,0
-0.40648795964495,48.236537848806,0
-0.40642182240902,48.236453231149,0
-0.40634486459211,48.236372774754,0
-0.40625767191908,48.236297091924,0
-0.40616090799684,48.236226758627,0

-0.40605530926337,48.236162310119,0
-0.40594167938264,48.23610423687,0
-0.4058208831287,48.236052980829,0
-0.40569383980454,48.236008932064,0
-0.40556151624657,48.235972425795,0
-0.40542491946775,48.23594373984,0
-0.40528508899498,48.235923092503,0
-0.40514308895948,48.235910640912,0
-0.40499999999997,48.235906479827,0
-0.40485691104047,48.235910640912,0
-0.40471491100497,48.235923092503,0
-0.4045750805322,48.23594373984,0
-0.40443848375338,48.235972425795,0
-0.40430616019541,48.236008932064,0
-0.40417911687122,48.236052980829,0
-0.40405832061728,48.23610423687,0
-0.40394469073658,48.236162310119,0
-0.40383909200308,48.236226758627,0
-0.40374232808087,48.236297091924,0
-0.40365513540784,48.236372774754,0
-0.40357817759093,48.236453231149,0
-0.403512040355,48.236537848806,0
-0.40345722708436,48.236625983754,0
-0.40341415499028,48.236716965249,0
-0.40338315193448,48.236810100881,0
-0.40336445393204,48.23690468184,0
-0.40335820335337,48.236999988314,0
-0.40499999999997,48.237,0
</coordinates>
</LinearRing>
</outerBoundaryIs>
</Polygon>
</Placemark>
<Placemark>
<name>49</name>
<styleUrl>#stylehap2</styleUrl>
<Polygon>
<outerBoundaryIs>
<LinearRing>
<coordinates>
-1.056,48.145,0
-1.05436114825,48.144999988351,0
-1.0543673815442,48.145095295003,0
-1.0543860401196,48.145189876488,0
-1.0544169820403,48.145283012979,0
-1.0544599718822,48.14537399564,0
-1.0545146825236,48.145462132027,0
-1.0545806976324,48.14554675135,0
-1.0546575148335,48.145627209588,0
-1.054744549531,48.145702894383,0
-1.0548411393561,48.145773229706,0
-1.0549465492081,48.145837680239,0
-1.0550599768482,48.145895755453,0
-1.0551805590058,48.145947013338,0
-1.0553073779484,48.145991063768,0
-1.0554394684676,48.146027571475,0
-1.0555758252259,48.146056258597,0

-1.0557154104091,48.146076906794,0
-1.0558571616269,48.146089358911,0
-1.056,48.146093520174,0
-1.0561428383732,48.146089358911,0
-1.056284589591,48.146076906794,0
-1.0564241747742,48.146056258597,0
-1.0565605315324,48.146027571475,0
-1.0566926220517,48.145991063768,0
-1.0568194409943,48.145947013338,0
-1.0569400231519,48.145895755453,0
-1.057053450792,48.145837680239,0
-1.057158860644,48.145773229706,0
-1.0572554504691,48.145702894383,0
-1.0573424851666,48.145627209588,0
-1.0574193023677,48.14554675135,0
-1.0574853174765,48.145462132027,0
-1.0575400281179,48.14537399564,0
-1.0575830179598,48.145283012979,0
-1.0576139598804,48.145189876488,0
-1.0576326184559,48.145095295003,0
-1.0576388517501,48.144999988351,0
-1.0576326123929,48.144904681877,0
-1.0576139479387,48.144810100917,0
-1.0575830005021,48.144716965285,0
-1.0575400056746,48.144625983788,0
-1.0574852907297,48.144537848837,0
-1.05741927213,48.144453231177,0
-1.0573424523568,48.14437277478,0
-1.0572554160842,48.144297091946,0
-1.0571588257286,48.144226758646,0
-1.057053416407,48.144162310135,0
-1.0569399903421,48.144104236882,0
-1.0568194107567,48.144052980838,0
-1.0566925953049,48.144008932071,0
-1.0565605090892,48.1439724258,0
-1.0564241573165,48.143943739843,0
-1.0562845776492,48.143923092504,0
-1.0561428323102,48.143910640912,0
-1.056,48.143906479826,0
-1.0558571676899,48.143910640912,0
-1.0557154223509,48.143923092504,0
-1.0555758426836,48.143943739843,0
-1.0554394909108,48.1439724258,0
-1.0553074046952,48.144008932071,0
-1.0551805892434,48.144052980838,0
-1.055060009658,48.144104236882,0
-1.054946583593,48.144162310135,0
-1.0548411742715,48.144226758646,0
-1.0547445839159,48.144297091946,0
-1.0546575476433,48.14437277478,0
-1.05458072787,48.144453231177,0
-1.0545147092704,48.144537848837,0
-1.0544599943254,48.144625983788,0
-1.054416999498,48.144716965285,0
-1.0543860520614,48.144810100917,0
-1.0543673876072,48.144904681877,0
-1.05436114825,48.144999988351,0

-1.056,48.145,0
</coordinates>
</LinearRing>
</outerBoundaryIs>
</Polygon>
</Placemark>
<Placemark>
<name>50</name>
<styleUrl>#stylehap2</styleUrl>
<Polygon>
<outerBoundaryIs>
<LinearRing>
<coordinates>
-1.0509999999999,48.236,0
-1.0493582354427,48.235999988314,0
-1.0493644798059,48.236095294966,0
-1.0493831715347,48.236189876452,0
-1.049414168441,48.236283012944,0
-1.0494572346828,48.236373995607,0
-1.049512042557,48.236462131996,0
-1.0495781749919,48.236546751322,0
-1.0496551287197,48.236627209563,0
-1.0497423181054,48.236702894361,0
-1.0498390796033,48.236773229687,0
-1.0499446768059,48.236837680224,0
-1.0500583060488,48.236895755441,0
-1.0501791025267,48.236947013328,0
-1.0503061468761,48.236991063761,0
-1.0504384721729,48.237027571471,0
-1.0505750712923,48.237056258594,0
-1.0507149045756,48.237076906793,0
-1.0508569077438,48.23708935891,0
-1.0509999999999,48.237093520174,0
-1.051143092256,48.23708935891,0
-1.0512850954243,48.237076906793,0
-1.0514249287076,48.237056258594,0
-1.051561527827,48.237027571471,0
-1.0516938531237,48.236991063761,0
-1.0518208974732,48.236947013328,0
-1.0519416939511,48.236895755441,0
-1.052055323194,48.236837680224,0
-1.0521609203966,48.236773229687,0
-1.0522576818944,48.236702894361,0
-1.0523448712802,48.236627209563,0
-1.0524218250079,48.236546751322,0
-1.0524879574428,48.236462131996,0
-1.0525427653171,48.236373995607,0
-1.0525858315588,48.236283012944,0
-1.0526168284652,48.236189876452,0
-1.0526355201939,48.236095294966,0
-1.0526417645571,48.235999988314,0
-1.0526355141007,48.23590468184,0
-1.0526168164639,48.235810100881,0
-1.0525858140141,48.23571696525,0
-1.052542742762,48.235625983755,0
-1.0524879305627,48.235537848806,0
-1.0524217946196,48.235453231149,0

-1.0523448383069,48.235372774755,0
-1.0522576473381,48.235297091924,0
-1.0521608853071,48.235226758627,0
-1.0520552886376,48.235162310119,0
-1.0519416609778,48.23510423687,0
-1.0518208670848,48.235052980829,0
-1.0516938262437,48.235008932064,0
-1.0515615052719,48.234972425795,0
-1.0514249111628,48.23494373984,0
-1.051285083423,48.234923092503,0
-1.0511430861628,48.234910640912,0
-1.0509999999999,48.234906479826,0
-1.050856913837,48.234910640912,0
-1.0507149165769,48.234923092503,0
-1.050575088837,48.23494373984,0
-1.0504384947279,48.234972425795,0
-1.0503061737562,48.235008932064,0
-1.0501791329151,48.235052980829,0
-1.0500583390221,48.23510423687,0
-1.0499447113623,48.235162310119,0
-1.0498391146927,48.235226758627,0
-1.0497423526618,48.235297091924,0
-1.049655161693,48.235372774755,0
-1.0495782053803,48.235453231149,0
-1.0495120694371,48.235537848806,0
-1.0494572572379,48.235625983755,0
-1.0494141859857,48.23571696525,0
-1.049383183536,48.235810100881,0
-1.0493644858991,48.23590468184,0
-1.0493582354427,48.235999988314,0
-1.0509999999999,48.236,0
</coordinates>
</LinearRing>
</outerBoundaryIs>
</Polygon>
</Placemark>
<Placemark>
<name>51</name>
<styleUrl>#stylehap2</styleUrl>
<Polygon>
<outerBoundaryIs>
<LinearRing>
<coordinates>
-1.049,47.961,0
-1.0473669938693,47.960999988426,0
-1.0473732049494,47.961095295077,0
-1.0473917969904,47.961189876561,0
-1.0474226285621,47.961283013049,0
-1.0474654650796,47.961373995707,0
-1.0475199805874,47.961462132088,0
-1.0475857602381,47.961546751406,0
-1.0476623034483,47.961627209638,0
-1.0477490277067,47.961702894427,0
-1.0478452730074,47.961773229743,0
-1.0479503068713,47.96183768027,0
-1.0480633299214,47.961895755478,0
-1.0481834819663,47.961947013356,0

-1.0483098485473,47.961991063781,0
-1.0484414678992,47.962027571484,0
-1.0485773382706,47.962056258602,0
-1.0487164255498,47.962076906796,0
-1.0488576711364,47.962089358911,0
-1.049,47.962093520174,0
-1.0491423288636,47.962089358911,0
-1.0492835744502,47.962076906796,0
-1.0494226617294,47.962056258602,0
-1.0495585321008,47.962027571484,0
-1.0496901514526,47.961991063781,0
-1.0498165180337,47.961947013356,0
-1.0499366700785,47.961895755478,0
-1.0500496931286,47.96183768027,0
-1.0501547269925,47.961773229743,0
-1.0502509722932,47.961702894427,0
-1.0503376965517,47.961627209638,0
-1.0504142397618,47.961546751406,0
-1.0504800194126,47.961462132088,0
-1.0505345349204,47.961373995707,0
-1.0505773714379,47.961283013049,0
-1.0506082030095,47.961189876561,0
-1.0506267950506,47.961095295077,0
-1.0506330061307,47.960999988426,0
-1.0506267890481,47.960904681951,0
-1.050608191187,47.96081010099,0
-1.0505773541545,47.960716965355,0
-1.0505345127012,47.960625983854,0
-1.0504799929328,47.960537848899,0
-1.050414209826,47.960453231233,0
-1.0503376640695,47.96037277483,0
-1.0502509382515,47.96029709199,0
-1.0501546924257,47.960226758683,0
-1.0500496590869,47.960162310166,0
-1.0499366375963,47.960104236907,0
-1.0498164880979,47.960052980857,0
-1.0496901249729,47.960008932084,0
-1.0495585098816,47.959972425808,0
-1.0494226444459,47.959943739848,0
-1.0492835626276,47.959923092506,0
-1.0491423228611,47.959910640913,0
-1.049,47.959906479826,0
-1.0488576771389,47.959910640913,0
-1.0487164373723,47.959923092506,0
-1.048577355554,47.959943739848,0
-1.0484414901183,47.959972425808,0
-1.0483098750271,47.960008932084,0
-1.0481835119021,47.960052980857,0
-1.0480633624037,47.960104236907,0
-1.047950340913,47.960162310166,0
-1.0478453075743,47.960226758683,0
-1.0477490617485,47.96029709199,0
-1.0476623359305,47.96037277483,0
-1.0475857901739,47.960453231233,0
-1.0475200070671,47.960537848899,0
-1.0474654872987,47.960625983854,0
-1.0474226458455,47.960716965355,0

-1.047391808813,47.96081010099,0
-1.0473732109519,47.960904681951,0
-1.0473669938693,47.960999988426,0
-1.049,47.961,0
</coordinates>
</LinearRing>
</outerBoundaryIs>
</Polygon>
</Placemark>
<Placemark>
<name>54</name>
<styleUrl>#stylehap2</styleUrl>
<Polygon>
<outerBoundaryIs>
<LinearRing>
<coordinates>
-1.048,47.962,0
-1.0463669622578,47.961999988426,0
-1.046373173458,47.962095295077,0
-1.0463917658589,47.96218987656,0
-1.0464225980273,47.962283013048,0
-1.0464654353739,47.962373995706,0
-1.0465199519369,47.962462132088,0
-1.046585732861,47.962546751406,0
-1.0466622775528,47.962627209638,0
-1.04674900349,47.962702894426,0
-1.0468452506538,47.962773229743,0
-1.0469502865509,47.96283768027,0
-1.047063311789,47.962895755478,0
-1.0471834661598,47.962947013356,0
-1.047309835187,47.962991063781,0
-1.0474414570868,47.963027571484,0
-1.0475773300885,47.963056258602,0
-1.0477164200602,47.963076906796,0
-1.0478576683811,47.963089358911,0
-1.048,47.963093520174,0
-1.0481423316188,47.963089358911,0
-1.0482835799398,47.963076906796,0
-1.0484226699115,47.963056258602,0
-1.0485585429132,47.963027571484,0
-1.048690164813,47.962991063781,0
-1.0488165338402,47.962947013356,0
-1.048936688211,47.962895755478,0
-1.0490497134491,47.96283768027,0
-1.0491547493462,47.962773229743,0
-1.04925099651,47.962702894426,0
-1.0493377224472,47.962627209638,0
-1.049414267139,47.962546751406,0
-1.0494800480631,47.962462132088,0
-1.0495345646261,47.962373995706,0
-1.0495774019727,47.962283013048,0
-1.0496082341411,47.96218987656,0
-1.049626826542,47.962095295077,0
-1.0496330377422,47.961999988426,0
-1.0496268205392,47.961904681951,0
-1.0496082223179,47.96181010099,0
-1.0495773846884,47.961716965354,0

-1.0495345424057,47.961625983853,0
-1.0494800215819,47.961537848898,0
-1.0494142372016,47.961453231233,0
-1.0493376899632,47.96137277483,0
-1.0492509624664,47.96129709199,0
-1.0491547147775,47.961226758683,0
-1.0490496794055,47.961162310166,0
-1.048936655727,47.961104236907,0
-1.0488165039028,47.961052980857,0
-1.0486901383318,47.961008932084,0
-1.0485585206928,47.960972425808,0
-1.0484226526272,47.960943739848,0
-1.0482835681166,47.960923092506,0
-1.048142325616,47.960910640913,0
-1.048,47.960906479826,0
-1.0478576743839,47.960910640913,0
-1.0477164318834,47.960923092506,0
-1.0475773473729,47.960943739848,0
-1.0474414793072,47.960972425808,0
-1.0473098616682,47.961008932084,0
-1.0471834960972,47.961052980857,0
-1.047063344273,47.961104236907,0
-1.0469503205945,47.961162310166,0
-1.0468452852225,47.961226758683,0
-1.0467490375336,47.96129709199,0
-1.0466623100368,47.96137277483,0
-1.0465857627984,47.961453231233,0
-1.0465199784181,47.961537848898,0
-1.0464654575943,47.961625983853,0
-1.0464226153116,47.961716965354,0
-1.0463917776821,47.96181010099,0
-1.0463731794608,47.961904681951,0
-1.0463669622578,47.961999988426,0
-1.048,47.962,0
</coordinates>
</LinearRing>
</outerBoundaryIs>
</Polygon>
</Placemark>
<Placemark>
<name>58</name>
<styleUrl>#stylehap2</styleUrl>
<Polygon>
<outerBoundaryIs>
<LinearRing>
<coordinates>
-1.04,47.966,0
-1.0383668357945,47.965999988424,0
-1.0383730474754,47.966095295075,0
-1.0383916413156,47.966189876559,0
-1.0384224758712,47.966283013047,0
-1.0384653165349,47.966373995705,0
-1.0385198373194,47.966462132087,0
-1.0385856233373,47.966546751405,0
-1.0386621739566,47.966627209637,0
-1.0387489066098,47.966702894425,0
-1.038845161227,47.966773229742,0

-1.0389502052582,47.966837680269,0
-1.0390632392491,47.966895755477,0
-1.0391834029248,47.966947013356,0
-1.0393097817384,47.966991063781,0
-1.0394414138314,47.967027571484,0
-1.0395772973555,47.967056258602,0
-1.0397163980988,47.967076906796,0
-1.0398576573584,47.967089358911,0
-1.04,47.967093520174,0
-1.0401423426415,47.967089358911,0
-1.0402836019012,47.967076906796,0
-1.0404227026445,47.967056258602,0
-1.0405585861685,47.967027571484,0
-1.0406902182615,47.966991063781,0
-1.0408165970751,47.966947013356,0
-1.0409367607509,47.966895755477,0
-1.0410497947417,47.966837680269,0
-1.0411548387729,47.966773229742,0
-1.0412510933901,47.966702894425,0
-1.0413378260433,47.966627209637,0
-1.0414143766626,47.966546751405,0
-1.0414801626805,47.966462132087,0
-1.041534683465,47.966373995705,0
-1.0415775241287,47.966283013047,0
-1.0416083586843,47.966189876559,0
-1.0416269525246,47.966095295075,0
-1.0416331642054,47.965999988424,0
-1.0416269465205,47.965904681949,0
-1.0416083468585,47.965810100988,0
-1.0415775068405,47.965716965353,0
-1.0415346612398,47.965625983852,0
-1.0414801361936,47.965537848897,0
-1.0414143467187,47.965453231232,0
-1.0413377935523,47.965372774829,0
-1.0412510593391,47.965297091989,0
-1.0411548041967,47.965226758682,0
-1.0410497606907,47.965162310165,0
-1.0409367282598,47.965104236906,0
-1.0408165671311,47.965052980856,0
-1.0406901917745,47.965008932084,0
-1.0405585639433,47.964972425808,0
-1.0404226853563,47.964943739847,0
-1.0402835900754,47.964923092506,0
-1.0401423366374,47.964910640913,0
-1.04,47.964906479826,0
-1.0398576633626,47.964910640913,0
-1.0397164099246,47.964923092506,0
-1.0395773146436,47.964943739847,0
-1.0394414360566,47.964972425808,0
-1.0393098082254,47.965008932084,0
-1.0391834328688,47.965052980856,0
-1.0390632717401,47.965104236906,0
-1.0389502393092,47.965162310165,0
-1.0388451958033,47.965226758682,0
-1.0387489406608,47.965297091989,0
-1.0386622064477,47.965372774829,0
-1.0385856532812,47.965453231232,0

-1.0385198638064,47.965537848897,0
-1.0384653387601,47.965625983852,0
-1.0384224931594,47.965716965353,0
-1.0383916531414,47.965810100988,0
-1.0383730534795,47.965904681949,0
-1.0383668357945,47.965999988424,0
-1.04,47.966,0
</coordinates>
</LinearRing>
</outerBoundaryIs>
</Polygon>
</Placemark>
<Placemark>
<name>59</name>
<styleUrl>#stylehap2</styleUrl>
<Polygon>
<outerBoundaryIs>
<LinearRing>
<coordinates>
-1.037,48.408,0
-1.0353526901596,48.407999988243,0
-1.0353589555954,48.408095294895,0
-1.0353777104401,48.408189876383,0
-1.0354088120258,48.408283012878,0
-1.0354520237145,48.408373995545,0
-1.0355070166966,48.408462131938,0
-1.0355733724921,48.408546751269,0
-1.0356505861337,48.408627209515,0
-1.0357380700086,48.408702894319,0
-1.03583515833,48.408773229652,0
-1.0359411122032,48.408837680195,0
-1.0360551252491,48.408895755418,0
-1.0361763297414,48.408947013311,0
-1.0363038032111,48.408991063749,0
-1.0364365754677,48.409027571462,0
-1.0365736359845,48.40905625859,0
-1.0367139415906,48.40907690679,0
-1.0368564244118,48.40908935891,0
-1.037,48.409093520174,0
-1.0371435755882,48.40908935891,0
-1.0372860584095,48.40907690679,0
-1.0374263640156,48.40905625859,0
-1.0375634245324,48.409027571462,0
-1.037696196789,48.408991063749,0
-1.0378236702586,48.408947013311,0
-1.037944874751,48.408895755418,0
-1.0380588877969,48.408837680195,0
-1.0381648416701,48.408773229652,0
-1.0382619299914,48.408702894319,0
-1.0383494138664,48.408627209515,0
-1.0384266275079,48.408546751269,0
-1.0384929833035,48.408462131938,0
-1.0385479762856,48.408373995545,0
-1.0385911879743,48.408283012878,0
-1.03862228956,48.408189876383,0
-1.0386410444046,48.408095294895,0
-1.0386473098405,48.407999988243,0

-1.0386410382538,48.40790468177,0
-1.0386222774451,48.407810100812,0
-1.0385911702635,48.407716965184,0
-1.0385479535171,48.407625983692,0
-1.0384929561691,48.407537848748,0
-1.0384265968321,48.407453231096,0
-1.0383493805811,48.407372774707,0
-1.0382618951081,48.407297091883,0
-1.0381648062486,48.407226758592,0
-1.0380588529136,48.40716231009,0
-1.0379448414657,48.407104236847,0
-1.0378236395828,48.407052980811,0
-1.0376961696546,48.407008932052,0
-1.0375634017639,48.406972425787,0
-1.0374263463049,48.406943739835,0
-1.0372860462947,48.406923092501,0
-1.0371435694374,48.406910640912,0
-1.037,48.406906479827,0
-1.0368564305627,48.406910640912,0
-1.0367139537054,48.406923092501,0
-1.0365736536952,48.406943739835,0
-1.0364365982362,48.406972425787,0
-1.0363038303455,48.407008932052,0
-1.0361763604173,48.407052980811,0
-1.0360551585344,48.407104236847,0
-1.0359411470865,48.40716231009,0
-1.0358351937514,48.407226758592,0
-1.0357381048919,48.407297091883,0
-1.035650619419,48.407372774707,0
-1.035573403168,48.407453231096,0
-1.035507043831,48.407537848748,0
-1.0354520464829,48.407625983692,0
-1.0354088297365,48.407716965184,0
-1.035377722555,48.407810100812,0
-1.0353589617463,48.40790468177,0
-1.0353526901596,48.407999988243,0
-1.037,48.408,0
</coordinates>
</LinearRing>
</outerBoundaryIs>
</Polygon>
</Placemark>
<Placemark>
<name>69</name>
<styleUrl>#stylehap2</styleUrl>
<Polygon>
<outerBoundaryIs>
<LinearRing>
<coordinates>
-0.98299999999995,48.238,0
-0.98135817126217,48.237999988313,0
-0.98136441586927,48.238095294965,0
-0.98138310832854,48.238189876451,0
-0.98141410644641,48.238283012943,0
-0.98145717437157,48.238373995607,0
-0.98151198438822,48.238462131995,0
-0.98157811940827,48.238546751322,0

-0.98165507614425,48.238627209562,0
-0.98174226893837,48.23870289436,0
-0.98183903421886,48.238773229687,0
-0.98194463554955,48.238837680224,0
-0.98205826923452,48.238895755441,0
-0.98217907043476,48.238947013328,0
-0.98230611975078,48.238991063761,0
-0.98243845022061,48.239027571471,0
-0.98257505468022,48.239056258594,0
-0.98271489343008,48.239076906793,0
-0.98285690214979,48.23908935891,0
-0.98299999999994,48.239093520174,0
-0.9831430978501,48.23908935891,0
-0.9832851065698,48.239076906793,0
-0.9834249453197,48.239056258594,0
-0.98356154977928,48.239027571471,0
-0.9836938802491,48.238991063761,0
-0.98382092956513,48.238947013328,0
-0.98394173076536,48.238895755441,0
-0.98405536445034,48.238837680224,0
-0.98416096578103,48.238773229687,0
-0.98425773106151,48.23870289436,0
-0.98434492385563,48.238627209562,0
-0.98442188059162,48.238546751322,0
-0.98448801561166,48.238462131995,0
-0.98454282562832,48.238373995607,0
-0.98458589355351,48.238283012943,0
-0.98461689167135,48.238189876451,0
-0.98463558413064,48.238095294965,0
-0.98464182873771,48.237999988313,0
-0.98463557803675,48.237904681839,0
-0.98461687966875,48.23781010088,0
-0.98458587600685,48.237716965249,0
-0.98454280307078,48.237625983754,0
-0.98448798872865,48.237537848806,0
-0.98442185019994,48.237453231148,0
-0.98434489087873,48.237372774754,0
-0.98425769650138,48.237297091924,0
-0.98416093068775,48.237226758627,0
-0.98405532989018,48.237162310119,0
-0.98394169778846,48.23710423687,0
-0.98382089917345,48.237052980829,0
-0.98369385336609,48.237008932064,0
-0.98356152722175,48.236972425795,0
-0.98342492777303,48.23694373984,0
-0.98328509456721,48.236923092503,0
-0.9831430917562,48.236910640912,0
-0.98299999999994,48.236906479827,0
-0.98285690824368,48.236910640912,0
-0.98271490543271,48.236923092503,0
-0.98257507222685,48.23694373984,0
-0.98243847277814,48.236972425795,0
-0.98230614663382,48.237008932064,0
-0.98217910082646,48.237052980829,0
-0.98205830221143,48.23710423687,0
-0.98194467010971,48.237162310119,0
-0.98183906931216,48.237226758627,0

-0.98174230349854,48.237297091924,0
-0.98165510912116,48.23737274754,0
-0.98157814979997,48.237453231148,0
-0.98151201127124,48.237537848806,0
-0.98145719692911,48.237625983754,0
-0.98141412399304,48.237716965249,0
-0.98138312033116,48.23781010088,0
-0.98136442196314,48.237904681839,0
-0.98135817126217,48.237999988313,0
-0.98299999999995,48.238,0
</coordinates>
</LinearRing>
</outerBoundaryIs>
</Polygon>
</Placemark>
<Placemark>
<name>70</name>
<styleUrl>#stylehap2</styleUrl>
<Polygon>
<outerBoundaryIs>
<LinearRing>
<coordinates>
-0.97800000000007,48.026,0
-0.97636493553613,48.0259999884,0
-0.97637115443816,48.026095295051,0
-0.97638976990704,48.026189876535,0
-0.97642064033426,48.026283013024,0
-0.97646353083969,48.026373995683,0
-0.97651811505706,48.026462132067,0
-0.97658397761624,48.026546751387,0
-0.97666061730286,48.02662720962,0
-0.97674745087172,48.026702894411,0
-0.97684381748479,48.02677322973,0
-0.97694898373992,48.026837680259,0
-0.97706214925247,48.026895755469,0
-0.97718245274666,48.02694701335,0
-0.97730897861124,48.026991063777,0
-0.97744076386842,48.027027571481,0
-0.97757680550417,48.0270562586,0
-0.9777160681031,48.027076906795,0
-0.97785749173028,48.027089358911,0
-0.97800000000007,48.027093520174,0
-0.97814250826984,48.027089358911,0
-0.97828393189705,48.027076906795,0
-0.97842319449597,48.0270562586,0
-0.9785592361317,48.027027571481,0
-0.9786910213889,48.026991063777,0
-0.97881754725346,48.02694701335,0
-0.97893785074768,48.026895755469,0
-0.97905101626019,48.026837680259,0
-0.97915618251535,48.02677322973,0
-0.97925254912842,48.026702894411,0
-0.97933938269729,48.02662720962,0
-0.9794160223839,48.026546751387,0
-0.97948188494306,48.026462132067,0
-0.97953646916045,48.026373995683,0
-0.97957935966588,48.026283013024,0

-0.97961023009311,48.026189876535,0
-0.97962884556196,48.026095295051,0
-0.97963506446399,48.0259999884,0
-0.97962883953819,48.025904681925,0
-0.97961021822859,48.025810100964,0
-0.97957934232112,48.02571696533,0
-0.97953644686247,48.025625983831,0
-0.97948185836937,48.025537848877,0
-0.97941599234191,48.025453231213,0
-0.97933935009983,48.025372774812,0
-0.97925251496593,48.025297091974,0
-0.97915614782586,48.02522675867,0
-0.97905098209773,48.025162310155,0
-0.97893781815022,48.025104236898,0
-0.97881751721149,48.02505298085,0
-0.97869099481522,48.02500893208,0
-0.97855921383372,48.024972425805,0
-0.97842317715121,48.024943739846,0
-0.97828392003253,48.024923092505,0
-0.97814250224607,48.024910640913,0
-0.97800000000007,48.024906479826,0
-0.97785749775405,48.024910640913,0
-0.97771607996758,48.024923092505,0
-0.97757682284891,48.024943739846,0
-0.9774407861664,48.024972425805,0
-0.97730900518493,48.02500893208,0
-0.97718248278865,48.02505298085,0
-0.97706218184992,48.025104236898,0
-0.97694901790241,48.025162310155,0
-0.97684385217429,48.02522675867,0
-0.97674748503421,48.025297091974,0
-0.97666064990031,48.025372774812,0
-0.97658400765821,48.025453231213,0
-0.97651814163075,48.025537848877,0
-0.97646355313767,48.025625983831,0
-0.976420657679,48.02571696533,0
-0.97638978177152,48.025810100964,0
-0.97637116046193,48.025904681925,0
-0.97636493553613,48.0259999884,0
-0.97800000000007,48.026,0
</coordinates>
</LinearRing>
</outerBoundaryIs>
</Polygon>
</Placemark>
<Placemark>
<name>71</name>
<styleUrl>#stylehap2</styleUrl>
<Polygon>
<outerBoundaryIs>
<LinearRing>
<coordinates>
-0.97299999999996,48.443,0
-0.97135155534692,48.442999988229,0
-0.97135782509516,48.443095294881,0
-0.97137659285614,48.443189876369,0
-0.97140771586394,48.443283012864,0

-0.97145095731749,48.443373995532,0
-0.97150598818095,48.443462131926,0
-0.97157238968604,48.443546751258,0
-0.9716496565176,48.443627209505,0
-0.97173720065816,48.443702894311,0
-0.9718343558622,48.443773229644,0
-0.97194038272612,48.443837680189,0
-0.97205447431528,48.443895755413,0
-0.97217576230559,48.443947013307,0
-0.97230332359242,48.443991063746,0
-0.97243618731699,48.444027571461,0
-0.9725733422563,48.444056258589,0
-0.97271374452071,48.44407690679,0
-0.97285632550039,48.44408935891,0
-0.97299999999994,48.444093520174,0
-0.97314367449952,48.44408935891,0
-0.97328625547921,48.44407690679,0
-0.97342665774362,48.444056258589,0
-0.97356381268292,48.444027571461,0
-0.97369667640747,48.443991063746,0
-0.97382423769432,48.443947013307,0
-0.97394552568464,48.443895755413,0
-0.97405961727379,48.443837680189,0
-0.97416564413771,48.443773229644,0
-0.97426279934176,48.443702894311,0
-0.97435034348231,48.443627209505,0
-0.97442761031388,48.443546751258,0
-0.97449401181897,48.443462131926,0
-0.97454904268242,48.443373995532,0
-0.97459228413598,48.443283012864,0
-0.97462340714377,48.443189876369,0
-0.97464217490476,48.443095294881,0
-0.97464844465299,48.442999988229,0
-0.97464216874209,48.442904681755,0
-0.97462339500566,48.442810100798,0
-0.97459226639126,48.44271696517,0
-0.97454901987024,48.442625983679,0
-0.97449398463245,48.442537848736,0
-0.97442757957908,48.442453231085,0
-0.97435031013313,48.442372774697,0
-0.97426276439143,48.442297091874,0
-0.97416560864822,48.442226758584,0
-0.97405958232349,48.442162310084,0
-0.97394549233543,48.442104236842,0
-0.97382420695953,48.442052980807,0
-0.97369664922095,48.442008932049,0
-0.97356378987071,48.441972425785,0
-0.9734266399989,48.441943739834,0
-0.97328624334109,48.4419230925,0
-0.97314366833683,48.441910640911,0
-0.97299999999994,48.441906479826,0
-0.97285633166309,48.441910640911,0
-0.97271375665882,48.4419230925,0
-0.97257336000102,48.441943739834,0
-0.9724362101292,48.441972425785,0
-0.97230335077896,48.442008932049,0
-0.97217579304039,48.442052980807,0

-0.97205450766449,48.442104236842,0
-0.97194041767642,48.442162310084,0
-0.9718343913517,48.442226758584,0
-0.97173723560848,48.442297091874,0
-0.97164968986679,48.442372774697,0
-0.97157242042083,48.442453231085,0
-0.97150601536746,48.442537848736,0
-0.97145098012968,48.442625983679,0
-0.97140773360866,48.44271696517,0
-0.97137660499426,48.442810100798,0
-0.97135783125783,48.442904681755,0
-0.97135155534692,48.442999988229,0
-0.97299999999996,48.443,0
</coordinates>
</LinearRing>
</outerBoundaryIs>
</Polygon>
</Placemark>
<Placemark>
<name>73</name>
<styleUrl>#stylehap2</styleUrl>
<Polygon>
<outerBoundaryIs>
<LinearRing>
<coordinates>
-0.96500000000003,48.228,0
-0.96335849209495,48.227999988317,0
-0.96336473548282,48.228095294969,0
-0.96338342429041,48.228189876455,0
-0.96341441635184,48.228283012947,0
-0.96345747586192,48.22837399561,0
-0.9635122751688,48.228462131999,0
-0.9635783972659,48.228546751325,0
-0.96365533896409,48.228627209565,0
-0.96374251471998,48.228702894363,0
-0.96383926109149,48.228773229689,0
-0.96394484178635,48.228837680225,0
-0.96405845326569,48.228895755442,0
-0.96417923085949,48.228947013329,0
-0.96430625534798,48.228991063762,0
-0.96443855995811,48.229027571471,0
-0.96457513772269,48.229056258595,0
-0.96471494914546,48.229076906793,0
-0.96485693011396,48.22908935891,0
-0.96500000000004,48.229093520174,0
-0.9651430698861,48.22908935891,0
-0.9652850508546,48.229076906793,0
-0.96542486227737,48.229056258595,0
-0.96556144004197,48.229027571471,0
-0.96569374465211,48.228991063762,0
-0.96582076914056,48.228947013329,0
-0.96594154673437,48.228895755442,0
-0.96605515821371,48.228837680225,0
-0.96616073890857,48.228773229689,0
-0.96625748528008,48.228702894363,0
-0.96634466103596,48.228627209565,0
-0.96642160273416,48.228546751325,0

-0.96648772483126,48.228462131999,0
-0.96654252413814,48.22837399561,0
-0.96658558364822,48.228283012947,0
-0.96661657570965,48.228189876455,0
-0.96663526451726,48.228095294969,0
-0.96664150790513,48.227999988317,0
-0.9666352584267,48.227904681843,0
-0.96661656371362,48.227810100884,0
-0.96658556611118,48.227716965253,0
-0.96654250159294,48.227625983758,0
-0.96648769796292,48.227537848809,0
-0.96642157235907,48.227453231151,0
-0.9663446280771,48.227372774757,0
-0.96625745073882,48.227297091926,0
-0.96616070383446,48.227226758629,0
-0.96605512367245,48.227162310121,0
-0.9659415137755,48.227104236871,0
-0.9658207387655,48.22705298083,0
-0.96569371778377,48.227008932065,0
-0.96556141749675,48.226972425796,0
-0.96542484474032,48.22694373984,0
-0.96528503885856,48.226923092503,0
-0.96514306379556,48.226910640912,0
-0.96500000000004,48.226906479827,0
-0.96485693620452,48.226910640912,0
-0.96471496114152,48.226923092503,0
-0.96457515525973,48.22694373984,0
-0.96443858250331,48.226972425796,0
-0.96430628221629,48.227008932065,0
-0.96417926123458,48.22705298083,0
-0.96405848622456,48.227104236871,0
-0.96394487632761,48.227162310121,0
-0.9638392961656,48.227226758629,0
-0.96374254926124,48.227297091926,0
-0.96365537192299,48.227372774757,0
-0.96357842764099,48.227453231151,0
-0.96351230203714,48.227537848809,0
-0.96345749840711,48.227625983758,0
-0.96341443388888,48.227716965253,0
-0.96338343628647,48.227810100884,0
-0.96336474157336,48.227904681843,0
-0.96335849209495,48.227999988317,0
-0.96500000000003,48.228,0
</coordinates>
</LinearRing>
</outerBoundaryIs>
</Polygon>
</Placemark>
<Placemark>
<name>82</name>
<styleUrl>#stylehap2</styleUrl>
<Polygon>
<outerBoundaryIs>
<LinearRing>
<coordinates>
-0.92399999999998,48.016,0
-0.92236525267737,48.015999988404,0

-0.92237147037422,48.016095295055,0
-0.9223900822334,48.016189876539,0
-0.92242094667388,48.016283013028,0
-0.92246382886101,48.016373995687,0
-0.92251840249184,48.01646213207,0
-0.92258425227677,48.01654675139,0
-0.92266087709859,48.016627209623,0
-0.92274769382527,48.016702894414,0
-0.92284404174691,48.016773229732,0
-0.92294918760364,48.016837680261,0
-0.92306233116603,48.016895755471,0
-0.92318261132541,48.016947013351,0
-0.9233091126481,48.016991063777,0
-0.92344087234313,48.017027571481,0
-0.92357688759102,48.0170562586,0
-0.92371612317724,48.017076906795,0
-0.92385751937252,48.017089358911,0
-0.92399999999998,48.017093520173,0
-0.92414248062742,48.017089358911,0
-0.92428387682273,48.017076906795,0
-0.92442311240895,48.0170562586,0
-0.92455912765683,48.017027571481,0
-0.92469088735187,48.016991063777,0
-0.92481738867453,48.016947013351,0
-0.92493766883393,48.016895755471,0
-0.9250508123963,48.016837680261,0
-0.92515595825303,48.016773229732,0
-0.92525230617467,48.016702894414,0
-0.92533912290138,48.016627209623,0
-0.9254157477232,48.01654675139,0
-0.9254815975081,48.01646213207,0
-0.92553617113893,48.016373995687,0
-0.92557905332609,48.016283013028,0
-0.92560991776657,48.016189876539,0
-0.92562852962574,48.016095295055,0
-0.9256347473226,48.015999988404,0
-0.92562852360526,48.015904681929,0
-0.92560990590852,48.015810100968,0
-0.92557903599079,48.015716965334,0
-0.92553614885311,48.015625983834,0
-0.92548157094889,48.01553784888,0
-0.92541571769759,48.015453231216,0
-0.92533909032166,48.015372774815,0
-0.9252522720308,48.015297091977,0
-0.92515592358244,48.015226758672,0
-0.92505077825243,48.015162310157,0
-0.92493763625421,48.0151042369,0
-0.92481735864892,48.015052980851,0
-0.92469086079266,48.01500893208,0
-0.92455910537099,48.014972425806,0
-0.92442309507365,48.014943739846,0
-0.92428386496468,48.014923092505,0
-0.92414247460693,48.014910640913,0
-0.92399999999998,48.014906479826,0
-0.92385752539301,48.014910640913,0
-0.92371613503529,48.014923092505,0
-0.92357690492632,48.014943739846,0

-0.92344089462895,48.014972425806,0
-0.92330913920731,48.01500893208,0
-0.92318264135105,48.015052980851,0
-0.92306236374573,48.0151042369,0
-0.92294922174751,48.015162310157,0
-0.9228440764175,48.015226758672,0
-0.92274772796914,48.015297091977,0
-0.92266090967828,48.015372774815,0
-0.92258428230238,48.015453231216,0
-0.92251842905108,48.01553784888,0
-0.92246385114685,48.015625983834,0
-0.92242096400917,48.015716965334,0
-0.92239009409142,48.015810100968,0
-0.92237147639471,48.015904681929,0
-0.92236525267737,48.015999988404,0
-0.92399999999998,48.016,0
</coordinates>
</LinearRing>
</outerBoundaryIs>
</Polygon>
</Placemark>
<Placemark>
<name>84</name>
<styleUrl>#stylehap2</styleUrl>
<Polygon>
<outerBoundaryIs>
<LinearRing>
<coordinates>
-0.91800000000001,48.097,0
-0.91636267885401,48.096999988371,0
-0.91636890633171,48.097095295022,0
-0.91638754748596,48.097189876507,0
-0.91641846051308,48.097283012997,0
-0.91646141020888,48.097373995658,0
-0.91651606975693,48.097462132043,0
-0.91658202321389,48.097546751365,0
-0.91665876867382,48.097627209601,0
-0.91674572208662,48.097702894394,0
-0.91684222170224,48.097773229715,0
-0.91694753310634,48.097837680247,0
-0.91706085480951,48.09789575546,0
-0.91718132434731,48.097947013342,0
-0.91730802484457,48.097991063771,0
-0.91743999199439,48.098027571477,0
-0.91757622139816,48.098056258598,0
-0.91771567621129,48.098076906794,0
-0.91785729503575,48.098089358911,0
-0.918,48.098093520174,0
-0.91814270496427,48.098089358911,0
-0.91828432378874,48.098076906794,0
-0.91842377860184,48.098056258598,0
-0.91856000800563,48.098027571477,0
-0.91869197515545,48.097991063771,0
-0.91881867565271,48.097947013342,0
-0.91893914519051,48.09789575546,0
-0.91905246689368,48.097837680247,0
-0.91915777829778,48.097773229715,0

-0.91925427791338,48.097702894394,0
-0.91934123132618,48.097627209601,0
-0.91941797678611,48.097546751365,0
-0.9194839302431,48.097462132043,0
-0.91953858979112,48.097373995658,0
-0.91958153948695,48.097283012997,0
-0.91961245251406,48.097189876507,0
-0.91963109366831,48.097095295022,0
-0.91963732114601,48.096999988371,0
-0.91963108762116,48.096904681896,0
-0.91961244060352,48.096810100936,0
-0.91958152207491,48.096716965303,0
-0.91953856740665,48.096625983805,0
-0.91948390356631,48.096537848853,0
-0.91941794662758,48.096453231192,0
-0.91934119860227,48.096372774793,0
-0.91925424361837,48.096297091957,0
-0.91915774347371,48.096226758656,0
-0.91905243259868,48.096162310143,0
-0.9189391124666,48.096104236889,0
-0.91881864549419,48.096052980843,0
-0.91869194847867,48.096008932075,0
-0.91855998562114,48.095972425802,0
-0.9184237611898,48.095943739844,0
-0.9182843118782,48.095923092504,0
-0.91814269891715,48.095910640913,0
-0.918,48.095906479826,0
-0.91785730108288,48.095910640913,0
-0.91771568812183,48.095923092504,0
-0.91757623881019,48.095943739844,0
-0.91744001437885,48.095972425802,0
-0.91730805152136,48.096008932075,0
-0.91718135450581,48.096052980843,0
-0.91706088753342,48.096104236889,0
-0.91694756740135,48.096162310143,0
-0.91684225652631,48.096226758656,0
-0.91674575638165,48.096297091957,0
-0.91665880139773,48.096372774793,0
-0.91658205337242,48.096453231192,0
-0.91651609643371,48.096537848853,0
-0.91646143259337,48.096625983805,0
-0.91641847792509,48.096716965303,0
-0.91638755939647,48.096810100936,0
-0.91636891237884,48.096904681896,0
-0.91636267885401,48.096999988371,0
-0.91800000000001,48.097,0
</coordinates>
</LinearRing>
</outerBoundaryIs>
</Polygon>
</Placemark>
<Placemark>
<name>86</name>
<styleUrl>#stylehap2</styleUrl>
<Polygon>
<outerBoundaryIs>
<LinearRing>

<coordinates>
-0.90100000000007,48.034,0
-0.89936468169861,48.033999988397,0
-0.89937090156527,48.034095295048,0
-0.89938951992329,48.034189876532,0
-0.89942039514226,48.034283013021,0
-0.89946329230558,48.03437399568,0
-0.89951788499633,48.034462132064,0
-0.89958375777993,48.034546751384,0
-0.8996604093642,48.034627209618,0
-0.89974725641346,48.034702894409,0
-0.89984363798699,48.034773229728,0
-0.89994882056885,48.034837680258,0
-0.90006200365012,48.034895755468,0
-0.90018232582137,48.034947013349,0
-0.90030887132905,48.034991063776,0
-0.900440677046,48.03502757148,0
-0.9005767398024,48.0350562586,0
-0.90071602402207,48.035076906795,0
-0.90085746960556,48.035089358911,0
-0.90100000000006,48.035093520174,0
-0.90114253039455,48.035089358911,0
-0.90128397597807,48.035076906795,0
-0.90142326019772,48.0350562586,0
-0.90155932295411,48.03502757148,0
-0.90169112867108,48.034991063776,0
-0.90181767417877,48.034947013349,0
-0.90193799635002,48.034895755468,0
-0.90205117943126,48.034837680258,0
-0.90215636201315,48.034773229728,0
-0.90225274358668,48.034702894409,0
-0.90233959063593,48.034627209618,0
-0.90241624222019,48.034546751384,0
-0.90248211500378,48.034462132064,0
-0.90253670769456,48.03437399568,0
-0.90257960485788,48.034283013021,0
-0.90261048007685,48.034189876532,0
-0.90262909843487,48.034095295048,0
-0.90263531830152,48.033999988397,0
-0.90262909240848,48.033904681922,0
-0.90261046820717,48.033810100961,0
-0.90257958750556,48.033716965327,0
-0.90253668538686,48.033625983828,0
-0.90248208841852,48.033537848874,0
-0.90241621216511,48.033453231211,0
-0.90233955802425,48.03337277481,0
-0.90225270940931,48.033297091973,0
-0.90215632730854,48.033226758668,0
-0.90205114525388,48.033162310154,0
-0.90193796373834,48.033104236897,0
-0.9018176441237,48.033052980849,0
-0.90169110208579,48.033008932079,0
-0.90155930064641,48.032972425805,0
-0.90142324284542,48.032943739846,0
-0.90128396410839,48.032923092505,0
-0.90114252436816,48.032910640913,0
-0.90100000000006,48.032906479827,0

-0.90085747563198,48.032910640913,0
-0.90071603589175,48.032923092505,0
-0.90057675715471,48.032943739846,0
-0.90044069935373,48.032972425805,0
-0.90030889791434,48.033008932079,0
-0.90018235587644,48.033052980849,0
-0.90006203626179,48.033104236897,0
-0.89994885474625,48.033162310154,0
-0.8998436726916,48.033226758668,0
-0.89974729059083,48.033297091973,0
-0.89966044197588,48.03337277481,0
-0.89958378783502,48.033453231211,0
-0.89951791158162,48.033537848874,0
-0.89946331461328,48.033625983828,0
-0.89942041249458,48.033716965327,0
-0.89938953179296,48.033810100961,0
-0.89937090759166,48.033904681922,0
-0.89936468169861,48.033999988397,0
-0.90100000000007,48.034,0
</coordinates>
</LinearRing>
</outerBoundaryIs>
</Polygon>
</Placemark>
<Placemark>
<name>88</name>
<styleUrl>#stylehap2</styleUrl>
<Polygon>
<outerBoundaryIs>
<LinearRing>
<coordinates>
-0.89999999999998,48.396,0
-0.89835307873715,48.395999988248,0
-0.89835934269634,48.3960952949,0
-0.89837809311826,48.396189876388,0
-0.89840918736873,48.396283012882,0
-0.89845238886538,48.396373995549,0
-0.89850736887635,48.396462131942,0
-0.89857370902022,48.396546751273,0
-0.89865090444871,48.396627209518,0
-0.89873836768776,48.396702894322,0
-0.89883543310745,48.396773229654,0
-0.89894136198746,48.396837680197,0
-0.89905534813893,48.396895755419,0
-0.89917652404024,48.396947013312,0
-0.89930396743997,48.39699106375,0
-0.8994367083765,48.397027571463,0
-0.89957373656154,48.39705625859,0
-0.89971400907029,48.397076906791,0
-0.89985645828056,48.39708935891,0
-0.89999999999997,48.397093520174,0
-0.9001435417194,48.39708935891,0
-0.90028599092967,48.397076906791,0
-0.90042626343842,48.39705625859,0
-0.90056329162346,48.397027571463,0
-0.90069603256,48.39699106375,0
-0.9008234759597,48.396947013312,0

-0.90094465186103,48.396895755419,0
-0.90105863801251,48.396837680197,0
-0.90116456689252,48.396773229654,0
-0.9012616323122,48.396702894322,0
-0.90134909555123,48.396627209518,0
-0.90142629097975,48.396546751273,0
-0.90149263112359,48.396462131942,0
-0.90154761113455,48.396373995549,0
-0.90159081263123,48.396283012882,0
-0.9016219068817,48.396189876388,0
-0.90164065730362,48.3960952949,0
-0.90164692126282,48.395999988248,0
-0.90164065115678,48.395904681775,0
-0.90162189477483,48.395810100817,0
-0.90159079493216,48.395716965188,0
-0.90154758838107,48.395625983697,0
-0.90149260400704,48.395537848752,0
-0.90142626032406,48.3954532311,0
-0.90134906228787,48.39537277471,0
-0.90126159745182,48.395297091885,0
-0.90116453149437,48.395226758594,0
-0.90105860315213,48.395162310092,0
-0.90094461859764,48.395104236848,0
-0.90082344530401,48.395052980812,0
-0.90069600544345,48.395008932053,0
-0.90056326886995,48.394972425788,0
-0.90042624573935,48.394943739836,0
-0.9002859788228,48.394923092501,0
-0.90014353557259,48.394910640912,0
-0.89999999999997,48.394906479826,0
-0.89985646442738,48.394910640912,0
-0.89971402117716,48.394923092501,0
-0.89957375426062,48.394943739836,0
-0.89943673112999,48.394972425788,0
-0.89930399455651,48.395008932053,0
-0.89917655469596,48.395052980812,0
-0.89905538140229,48.395104236848,0
-0.89894139684781,48.395162310092,0
-0.89883546850557,48.395226758594,0
-0.89873840254812,48.395297091885,0
-0.8986509377121,48.39537277471,0
-0.8985737396759,48.3954532311,0
-0.89850739599289,48.395537848752,0
-0.89845241161887,48.395625983697,0
-0.89840920506778,48.395716965188,0
-0.89837810522513,48.395810100817,0
-0.89835934884316,48.395904681775,0
-0.89835307873715,48.395999988248,0
-0.89999999999998,48.396,0
</coordinates>
</LinearRing>
</outerBoundaryIs>
</Polygon>
</Placemark>
<Placemark>
<name>90</name>
<styleUrl>#stylehap2</styleUrl>

<Polygon>
<outerBoundaryIs>
<LinearRing>
<coordinates>
-0.88099999999997,48.193,0
-0.87935961362915,48.192999988332,0
-0.87936585275506,48.193095294983,0
-0.87938452879745,48.193189876469,0
-0.87941549968745,48.19328301296,0
-0.87945852978089,48.193373995623,0
-0.87951329164967,48.193462132011,0
-0.87957936857205,48.193546751335,0
-0.87965625770262,48.193627209575,0
-0.87974337389804,48.193702894371,0
-0.87984005416943,48.193773229696,0
-0.87994556272763,48.193837680231,0
-0.88005909658276,48.193895755447,0
-0.8801797916556,48.193947013333,0
-0.88030672935445,48.193991063764,0
-0.88043894356689,48.194027571473,0
-0.88057542801381,48.194056258596,0
-0.88071514390923,48.194076906793,0
-0.88085702786787,48.19408935891,0
-0.88099999999998,48.194093520173,0
-0.88114297213207,48.19408935891,0
-0.8812848560907,48.194076906793,0
-0.88142457198612,48.194056258596,0
-0.88156105643305,48.194027571473,0
-0.88169327064551,48.193991063764,0
-0.88182020834434,48.193947013333,0
-0.8819409034172,48.193895755447,0
-0.8820544372723,48.193837680231,0
-0.88215994583051,48.193773229696,0
-0.88225662610192,48.193702894371,0
-0.88234374229732,48.193627209575,0
-0.88242063142789,48.193546751335,0
-0.88248670835027,48.193462132011,0
-0.88254147021905,48.193373995623,0
-0.88258450031248,48.19328301296,0
-0.88261547120249,48.193189876469,0
-0.88263414724488,48.193095294983,0
-0.88264038637078,48.192999988332,0
-0.88263414116595,48.192904681857,0
-0.88261545922935,48.192810100898,0
-0.88258448280895,48.192716965266,0
-0.88254144771696,48.19262598377,0
-0.88248668153331,48.192537848821,0
-0.88242060111086,48.192453231162,0
-0.88234370940142,48.192372774766,0
-0.88225659162667,48.192297091934,0
-0.88215991082342,48.192226758636,0
-0.88205440279707,48.192162310127,0
-0.88194087052131,48.192104236876,0
-0.88182017802732,48.192052980833,0
-0.88169324382852,48.192008932067,0
-0.88156103393093,48.191972425797,0
-0.88142455448259,48.191943739841,0

-0.88128484411757,48.191923092503,0
-0.88114296605316,48.191910640912,0
-0.88099999999998,48.191906479826,0
-0.88085703394677,48.191910640912,0
-0.88071515588237,48.191923092503,0
-0.88057544551735,48.191943739841,0
-0.880438966069,48.191972425797,0
-0.88030675617142,48.192008932067,0
-0.88017982197262,48.192052980833,0
-0.88005912947865,48.192104236876,0
-0.87994559720289,48.192162310127,0
-0.87984008917652,48.192226758636,0
-0.87974340837327,48.192297091934,0
-0.87965629059851,48.192372774766,0
-0.87957939888907,48.192453231162,0
-0.87951331846665,48.192537848821,0
-0.87945855228301,48.19262598377,0
-0.87941551719099,48.192716965266,0
-0.87938454077058,48.192810100898,0
-0.87936585883399,48.192904681857,0
-0.87935961362915,48.192999988332,0
-0.88099999999997,48.193,0
</coordinates>
</LinearRing>
</outerBoundaryIs>
</Polygon>
</Placemark>
<Placemark>
<name>92</name>
<styleUrl>#stylehap2</styleUrl>
<Polygon>
<outerBoundaryIs>
<LinearRing>
<coordinates>
-0.87900000000002,48.189,0
-0.87735974166801,48.188999988333,0
-0.87736598030737,48.189095294985,0
-0.87738465489243,48.18918987647,0
-0.87741562336541,48.189283012962,0
-0.87745865010051,48.189373995624,0
-0.87751340769521,48.189462132012,0
-0.87757947946026,48.189546751337,0
-0.87765636258951,48.189627209576,0
-0.87774347198526,48.189702894372,0
-0.87784014471041,48.189773229697,0
-0.87794564503322,48.189837680232,0
-0.87805917002643,48.189895755447,0
-0.87817985567836,48.189947013333,0
-0.87830678346898,48.189991063765,0
-0.87843898736125,48.190027571473,0
-0.87857546115466,48.190056258596,0
-0.87871516614432,48.190076906793,0
-0.87885703902791,48.19008935891,0
-0.87900000000001,48.190093520174,0
-0.87914296097214,48.19008935891,0
-0.87928483385573,48.190076906793,0
-0.87942453884538,48.190056258596,0

-0.87956101263878,48.190027571473,0
-0.87969321653107,48.189991063765,0
-0.87982014432166,48.189947013333,0
-0.87994082997359,48.189895755447,0
-0.88005435496683,48.189837680232,0
-0.88015985528961,48.189773229697,0
-0.88025652801476,48.189702894372,0
-0.88034363741051,48.189627209576,0
-0.88042052053976,48.189546751337,0
-0.88048659230484,48.189462132012,0
-0.88054134989954,48.189373995624,0
-0.88058437663463,48.189283012962,0
-0.88061534510762,48.18918987647,0
-0.88063401969268,48.189095294985,0
-0.88064025833204,48.188999988333,0
-0.8806340136151,48.188904681859,0
-0.88061533313711,48.1888101009,0
-0.88058435913492,48.188716965268,0
-0.88054132740233,48.188625983772,0
-0.8804865654937,48.188537848822,0
-0.88042049022938,48.188453231163,0
-0.88034360452182,48.188372774768,0
-0.88025649354707,48.188297091935,0
-0.88015982029018,48.188226758637,0
-0.88005432049911,48.188162310127,0
-0.8799407970849,48.188104236876,0
-0.87982011401128,48.188052980834,0
-0.87969318971996,48.188008932068,0
-0.87956099014159,48.187972425798,0
-0.87942452134567,48.187943739841,0
-0.87928482188522,48.187923092503,0
-0.87914295489456,48.187910640912,0
-0.87900000000001,48.187906479827,0
-0.87885704510549,48.187910640912,0
-0.87871517811481,48.187923092503,0
-0.87857547865438,48.187943739841,0
-0.87843900985845,48.187972425798,0
-0.87830681028009,48.188008932068,0
-0.87817988598877,48.188052980834,0
-0.87805920291515,48.188104236876,0
-0.87794567950092,48.188162310127,0
-0.87784017970984,48.188226758637,0
-0.87774350645298,48.188297091935,0
-0.87765639547822,48.188372774768,0
-0.87757950977067,48.188453231163,0
-0.87751343450632,48.188537848822,0
-0.87745867259772,48.188625983772,0
-0.87741564086513,48.188716965268,0
-0.87738466686294,48.1888101009,0
-0.87736598638495,48.188904681859,0
-0.87735974166801,48.188999988333,0
-0.87900000000002,48.189,0
</coordinates>
</LinearRing>
</outerBoundaryIs>
</Polygon>
</Placemark>

<Placemark>
<name>94</name>
<styleUrl>#stylehap2</styleUrl>
<Polygon>
<outerBoundaryIs>
<LinearRing>
<coordinates>
-0.87699999999995,48.188,0
-0.87535977367328,48.187999988334,0
-0.87536601219101,48.188095294985,0
-0.87538468641178,48.188189876471,0
-0.87541565428059,48.188283012962,0
-0.87545868017622,48.188373995625,0
-0.87551343670256,48.188462132012,0
-0.87557950717847,48.188546751337,0
-0.87565638880759,48.188627209576,0
-0.87574349650365,48.188702894372,0
-0.8758401673425,48.188773229697,0
-0.87594566560671,48.188837680232,0
-0.87605918838477,48.188895755447,0
-0.8761798716818,48.188947013333,0
-0.87630679699568,48.188991063765,0
-0.87643899830826,48.189027571473,0
-0.87657546943867,48.189056258596,0
-0.87671517170223,48.189076906793,0
-0.87685704181744,48.18908935891,0
-0.87699999999994,48.189093520174,0
-0.87714295818247,48.18908935891,0
-0.87728482829768,48.189076906793,0
-0.87742453056124,48.189056258596,0
-0.87756100169164,48.189027571473,0
-0.87769320300423,48.188991063765,0
-0.87782012831811,48.188947013333,0
-0.87794081161513,48.188895755447,0
-0.8780543343932,48.188837680232,0
-0.8781598326574,48.188773229697,0
-0.87825650349625,48.188702894372,0
-0.87834361119232,48.188627209576,0
-0.87842049282143,48.188546751337,0
-0.87848656329734,48.188462132012,0
-0.87854131982368,48.188373995625,0
-0.87858434571932,48.188283012962,0
-0.87861531358813,48.188189876471,0
-0.8786339878089,48.188095294985,0
-0.87864022632663,48.187999988334,0
-0.87863398173165,48.18790468186,0
-0.87861530161828,48.1878101009,0
-0.87858432822054,48.187716965268,0
-0.87854129732769,48.187625983772,0
-0.87848653648768,48.187537848823,0
-0.87842046251271,48.187453231164,0
-0.87834357830541,48.187372774768,0
-0.87825646903044,48.187297091936,0
-0.8781597976599,48.187226758637,0
-0.87805429992736,48.187162310128,0
-0.87794077872822,48.187104236876,0
-0.87782009800938,48.187052980834,0

-0.8776931761946,48.187008932068,0
-0.87756097919566,48.186972425798,0
-0.87742451306249,48.186943739841,0
-0.87728481632783,48.186923092503,0
-0.87714295210519,48.186910640912,0
-0.87699999999994,48.186906479826,0
-0.87685704789471,48.186910640912,0
-0.87671518367208,48.186923092503,0
-0.87657548693742,48.186943739841,0
-0.87643902080425,48.186972425798,0
-0.87630682380531,48.187008932068,0
-0.87617990199053,48.187052980834,0
-0.8760592212716,48.187104236876,0
-0.87594570007255,48.187162310128,0
-0.87584020234,48.187226758637,0
-0.87574353096947,48.187297091936,0
-0.8756564216945,48.187372774768,0
-0.8755795374872,48.187453231164,0
-0.87551346351222,48.187537848823,0
-0.87545870267221,48.187625983772,0
-0.87541567177936,48.187716965268,0
-0.87538469838163,48.1878101009,0
-0.87536601826826,48.18790468186,0
-0.87535977367328,48.187999988334,0
-0.87699999999995,48.188,0
</coordinates>
</LinearRing>
</outerBoundaryIs>
</Polygon>
</Placemark>
<Placemark>
<name>95</name>
<styleUrl>#stylehap2</styleUrl>
<Polygon>
<outerBoundaryIs>
<LinearRing>
<coordinates>
-0.87599999999998,47.989,0
-0.87436610809617,47.988999988415,0
-0.87437232254235,47.989095295066,0
-0.87439092466518,47.98918987655,0
-0.87442177295776,47.989283013038,0
-0.87446463270816,47.989373995697,0
-0.8745191777841,47.989462132079,0
-0.87458499311327,47.989546751398,0
-0.87466157784061,47.98962720963,0
-0.8747483491392,47.98970289442,0
-0.87484464664485,47.989773229737,0
-0.87494973748137,47.989837680266,0
-0.87506282183807,47.989895755474,0
-0.87518303905689,47.989947013353,0
-0.87530947418322,47.989991063779,0
-0.87544116492996,47.990027571482,0
-0.87557710900246,47.990056258601,0
-0.87571627172787,47.990076906796,0
-0.87585759393162,47.990089358911,0
-0.87599999999998,47.990093520174,0

-0.87614240606835,47.990089358911,0
-0.87628372827207,47.990076906796,0
-0.87642289099751,47.990056258601,0
-0.87655883506997,47.990027571482,0
-0.87669052581672,47.989991063779,0
-0.87681696094305,47.989947013353,0
-0.8769371781619,47.989895755474,0
-0.87705026251859,47.989837680266,0
-0.87715535335511,47.989773229737,0
-0.87725165086074,47.98970289442,0
-0.87733842215933,47.98962720963,0
-0.87741500688669,47.989546751398,0
-0.87748082221583,47.989462132079,0
-0.8775353672918,47.989373995697,0
-0.8775782270422,47.989283013038,0
-0.87760907533475,47.98918987655,0
-0.87762767745762,47.989095295066,0
-0.87763389190376,47.988999988415,0
-0.87762767144599,47.98890468194,0
-0.87760906349416,47.988810100979,0
-0.87757820973238,47.988716965344,0
-0.87753534503873,47.988625983844,0
-0.87748079569566,47.988537848889,0
-0.87741497690521,47.988453231225,0
-0.87733838962752,47.988372774822,0
-0.87725161676707,47.988297091983,0
-0.87715531873549,47.988226758678,0
-0.8770502284249,47.988162310161,0
-0.87693714563009,47.988104236903,0
-0.87681693096159,47.988052980854,0
-0.87669049929656,47.988008932082,0
-0.8765588128169,47.987972425807,0
-0.87642287368771,47.987943739847,0
-0.87628371643147,47.987923092506,0
-0.87614240005672,47.987910640913,0
-0.87599999999998,47.987906479826,0
-0.87585759994325,47.987910640913,0
-0.87571628356849,47.987923092506,0
-0.87557712631226,47.987943739847,0
-0.87544118718303,47.987972425807,0
-0.8753095007034,47.988008932082,0
-0.87518306903837,47.988052980854,0
-0.87506285436987,47.988104236903,0
-0.87494977157503,47.988162310161,0
-0.87484468126447,47.988226758678,0
-0.87474838323289,47.988297091983,0
-0.87466161037242,47.988372774822,0
-0.87458502309473,47.988453231225,0
-0.87451920430428,47.988537848889,0
-0.87446465496123,47.988625983844,0
-0.87442179026756,47.988716965344,0
-0.8743909365058,47.988810100979,0
-0.87437232855398,47.98890468194,0
-0.87436610809617,47.988999988415,0
-0.87599999999998,47.989,0
</coordinates>
</LinearRing>

</outerBoundaryIs>
</Polygon>
</Placemark>
<Placemark>
<name>101</name>
<styleUrl>#stylehap2</styleUrl>
<Polygon>
<outerBoundaryIs>
<LinearRing>
<coordinates>
-0.86900000000003,48.148,0
-0.86736105245401,48.14799998835,0
-0.86736728611221,48.148095295002,0
-0.86738594577802,48.148189876487,0
-0.86741688950703,48.148283012978,0
-0.86745988186161,48.148373995639,0
-0.86751459570076,48.148462132026,0
-0.86758061466814,48.148546751349,0
-0.86765743635932,48.148627209587,0
-0.86774447614416,48.148702894382,0
-0.86784107161524,48.148773229705,0
-0.86794648762877,48.148837680239,0
-0.86805992189917,48.148895755453,0
-0.86818051110527,48.148947013337,0
-0.86830733746104,48.148991063768,0
-0.86843943570162,48.149027571475,0
-0.86857580043062,48.149056258597,0
-0.86871539377331,48.149076906794,0
-0.86885715327722,48.149089358911,0
-0.86900000000004,48.149093520174,0
-0.86914284672283,48.149089358911,0
-0.86928460622677,48.149076906794,0
-0.86942419956943,48.149056258597,0
-0.86956056429843,48.149027571475,0
-0.86969266253901,48.148991063768,0
-0.86981948889478,48.148947013337,0
-0.86994007810088,48.148895755453,0
-0.87005351237131,48.148837680239,0
-0.87015892838481,48.148773229705,0
-0.87025552385592,48.148702894382,0
-0.87034256364073,48.148627209587,0
-0.87041938533191,48.148546751349,0
-0.87048540429929,48.148462132026,0
-0.87054011813847,48.148373995639,0
-0.87058311049304,48.148283012978,0
-0.87061405422203,48.148189876487,0
-0.87063271388784,48.148095295002,0
-0.87063894754607,48.14799998835,0
-0.87063270782385,48.147904681876,0
-0.87061404227831,48.147810100916,0
-0.87058309303246,48.147716965284,0
-0.87054009569159,48.147625983787,0
-0.87048537754816,48.147537848836,0
-0.87041935508931,48.147453231176,0
-0.87034253082559,48.147372774779,0
-0.87025548946529,48.147297091945,0
-0.8701588934637,48.147226758645,0

-0.87005347798068,48.147162310134,0
-0.86994004528574,48.147104236882,0
-0.86981945865219,48.147052980838,0
-0.86969263578787,48.147008932071,0
-0.86956054185155,48.1469724258,0
-0.86942418210888,48.146943739842,0
-0.86928459428302,48.146923092504,0
-0.86914284065883,48.146910640912,0
-0.86900000000004,48.146906479826,0
-0.86885715934122,48.146910640912,0
-0.86871540571703,48.146923092504,0
-0.8685758178912,48.146943739842,0
-0.8684394581485,48.1469724258,0
-0.86830736421221,48.147008932071,0
-0.86818054134786,48.147052980838,0
-0.86805995471433,48.147104236882,0
-0.86794652201937,48.147162310134,0
-0.86784110653638,48.147226758645,0
-0.86774451053476,48.147297091945,0
-0.86765746917445,48.147372774779,0
-0.86758064491074,48.147453231176,0
-0.86751462245189,48.147537848836,0
-0.86745990430849,48.147625983787,0
-0.86741690696759,48.147716965284,0
-0.86738595772174,48.147810100916,0
-0.8673672921762,48.147904681876,0
-0.86736105245401,48.14799998835,0
-0.86900000000003,48.148,0
</coordinates>
</LinearRing>
</outerBoundaryIs>
</Polygon>
</Placemark>
<Placemark>
<name>105</name>
<styleUrl>#stylehap2</styleUrl>
<Polygon>
<outerBoundaryIs>
<LinearRing>
<coordinates>
-0.86400000000003,48.279,0
-0.86235685401247,48.278999988296,0
-0.86236310362522,48.279095294948,0
-0.86238181107732,48.279189876435,0
-0.86241283406117,48.279283012927,0
-0.86245593653642,48.279373995592,0
-0.86251079052437,48.279462131982,0
-0.86257697860246,48.279546751309,0
-0.86265399707944,48.279627209551,0
-0.86274125982778,48.27970289435,0
-0.86283810274329,48.279773229678,0
-0.86294378879899,48.279837680217,0
-0.86305751365413,48.279895755435,0
-0.86317841177585,48.279947013324,0
-0.86330556302687,48.279991063758,0
-0.8634379996694,48.280027571469,0
-0.86357471373126,48.280056258593,0

-0.86371466467868,48.280076906792,0
-0.86385678733715,48.28008935891,0
-0.86400000000004,48.280093520173,0
-0.86414321266292,48.28008935891,0
-0.8642853353214,48.280076906792,0
-0.86442528626881,48.280056258593,0
-0.86456200033065,48.280027571469,0
-0.86469443697318,48.279991063758,0
-0.86482158822423,48.279947013324,0
-0.86494248634592,48.279895755435,0
-0.86505621120106,48.279837680217,0
-0.86516189725679,48.279773229678,0
-0.8652587401723,48.27970289435,0
-0.86534600292061,48.279627209551,0
-0.86542302139762,48.279546751309,0
-0.86548920947568,48.279462131982,0
-0.86554406346365,48.279373995592,0
-0.86558716593888,48.279283012927,0
-0.86561818892273,48.279189876435,0
-0.86563689637483,48.279095294948,0
-0.86564314598761,48.278999988296,0
-0.86563689026727,48.278904681822,0
-0.86561817689319,48.278810100864,0
-0.86558714835285,48.278716965233,0
-0.86554404085548,48.278625983739,0
-0.86548918253231,48.278537848792,0
-0.8654229909377,48.278453231136,0
-0.86534596986968,48.278372774743,0
-0.86525870553456,48.278297091914,0
-0.86516186208471,48.278226758618,0
-0.86505617656334,48.278162310112,0
-0.86494245329499,48.278104236864,0
-0.86482155776431,48.278052980824,0
-0.86469441002982,48.278008932061,0
-0.86456197772247,48.277972425793,0
-0.86442526868276,48.277943739839,0
-0.86428532329183,48.277923092502,0
-0.86414320655534,48.277910640912,0
-0.86400000000004,48.277906479826,0
-0.86385679344471,48.277910640912,0
-0.86371467670822,48.277923092502,0
-0.86357473131729,48.277943739839,0
-0.86343802227758,48.277972425793,0
-0.86330558997026,48.278008932061,0
-0.86317844223574,48.278052980824,0
-0.86305754670508,48.278104236864,0
-0.86294382343673,48.278162310112,0
-0.86283813791536,48.278226758618,0
-0.86274129446552,48.278297091914,0
-0.86265403013039,48.278372774743,0
-0.86257700906238,48.278453231136,0
-0.86251081746774,48.278537848792,0
-0.86245595914459,48.278625983739,0
-0.86241285164722,48.278716965233,0
-0.86238182310689,48.278810100864,0
-0.8623631097328,48.278904681822,0
-0.86235685401247,48.278999988296,0

-0.86400000000003,48.279,0
</coordinates>
</LinearRing>
</outerBoundaryIs>
</Polygon>
</Placemark>
<Placemark>
<name>108</name>
<styleUrl>#stylehap2</styleUrl>
<Polygon>
<outerBoundaryIs>
<LinearRing>
<coordinates>
-0.85300000000007,48.151,0
-0.85136095664236,48.150999988349,0
-0.85136719066467,48.151095295,0
-0.85138585142101,48.151189876486,0
-0.85141679695866,48.151283012976,0
-0.85145979182628,48.151373995638,0
-0.85151450886374,48.151462132025,0
-0.85158053169036,48.151546751348,0
-0.85165735787233,48.151627209586,0
-0.85174440274537,48.151702894381,0
-0.85184100386333,48.151773229704,0
-0.85194642603943,48.151837680238,0
-0.85205986694122,48.151895755452,0
-0.85218046319699,48.151947013337,0
-0.85230729696713,48.151991063768,0
-0.8524394029303,48.152027571475,0
-0.85257577563134,48.152056258597,0
-0.85271537713483,48.152076906794,0
-0.85285714492622,48.152089358911,0
-0.85300000000005,48.152093520174,0
-0.85314285507389,48.152089358911,0
-0.85328462286528,48.152076906794,0
-0.8534242243688,48.152056258597,0
-0.85356059706981,48.152027571475,0
-0.853692703033,48.151991063768,0
-0.85381953680314,48.151947013337,0
-0.85394013305892,48.151895755452,0
-0.8540535739607,48.151837680238,0
-0.8541589961368,48.151773229704,0
-0.85425559725476,48.151702894381,0
-0.85434264212777,48.151627209586,0
-0.85441946830977,48.151546751348,0
-0.85448549113639,48.151462132025,0
-0.85454020817385,48.151373995638,0
-0.85458320304147,48.151283012976,0
-0.85461414857913,48.151189876486,0
-0.85463280933546,48.151095295,0
-0.85463904335777,48.150999988349,0
-0.85463280327048,48.150904681875,0
-0.85461413663345,48.150810100915,0
-0.85458318557804,48.150716965282,0
-0.85454018572331,48.150625983785,0
-0.85448546438088,48.150537848835,0
-0.85441943806221,48.150453231175,0

-0.85434260930727,48.150372774778,0
-0.85425556285852,48.150297091945,0
-0.85415896120994,48.150226758645,0
-0.85405353956445,48.150162310134,0
-0.85394010023842,48.150104236881,0
-0.85381950655558,48.150052980838,0
-0.85369267627747,48.150008932071,0
-0.85356057461927,48.149972425799,0
-0.85342420690536,48.149943739842,0
-0.8532846109196,48.149923092504,0
-0.8531428490089,48.149910640912,0
-0.85300000000005,48.149906479826,0
-0.85285715099121,48.149910640912,0
-0.85271538908053,48.149923092504,0
-0.85257579309477,48.149943739842,0
-0.85243942538087,48.149972425799,0
-0.85230732372267,48.150008932071,0
-0.85218049344455,48.150052980838,0
-0.85205989976172,48.150104236881,0
-0.85194646043568,48.150162310134,0
-0.85184103879017,48.150226758645,0
-0.85174443714162,48.150297091945,0
-0.85165739069286,48.150372774778,0
-0.85158056193789,48.150453231175,0
-0.85151453561926,48.150537848835,0
-0.85145981427682,48.150625983785,0
-0.85141681442209,48.150716965282,0
-0.85138586336669,48.150810100915,0
-0.85136719672966,48.150904681875,0
-0.85136095664236,48.150999988349,0
-0.85300000000007,48.151,0
</coordinates>
</LinearRing>
</outerBoundaryIs>
</Polygon>
</Placemark>
<Placemark>
<name>109</name>
<styleUrl>#stylehap2</styleUrl>
<Polygon>
<outerBoundaryIs>
<LinearRing>
<coordinates>
-0.85000000000002,48.15,0
-0.84836098858128,48.149999988349,0
-0.84836722248222,48.150095295001,0
-0.84838588287503,48.150189876486,0
-0.84841682780978,48.150283012977,0
-0.84845982183967,48.150373995639,0
-0.84851453781096,48.150462132025,0
-0.84858055935111,48.150546751349,0
-0.84865738403608,48.150627209586,0
-0.84874442721294,48.150702894381,0
-0.8488410264485,48.150773229705,0
-0.84894644657029,48.150837680238,0
-0.84905988526151,48.150895755453,0
-0.84918047916727,48.150947013337,0

-0.84930731046581,48.150991063768,0
-0.84943941385466,48.151027571475,0
-0.84957578389818,48.151056258597,0
-0.84971538268128,48.151076906794,0
-0.84985714771002,48.151089358911,0
-0.85000000000002,48.151093520174,0
-0.85014285229003,48.151089358911,0
-0.85028461731877,48.151076906794,0
-0.85042421610187,48.151056258597,0
-0.85056058614539,48.151027571475,0
-0.85069268953422,48.150991063768,0
-0.85081952083278,48.150947013337,0
-0.85094011473854,48.150895755453,0
-0.85105355342976,48.150837680238,0
-0.85115897355155,48.150773229705,0
-0.85125557278711,48.150702894381,0
-0.85134261596397,48.150627209586,0
-0.85141944064894,48.150546751349,0
-0.85148546218909,48.150462132025,0
-0.85154017816038,48.150373995639,0
-0.85158317219027,48.150283012977,0
-0.85161411712501,48.150189876486,0
-0.85163277751783,48.150095295001,0
-0.85163901141877,48.149999988349,0
-0.85163277145317,48.149904681875,0
-0.85161410518,48.149810100915,0
-0.8515831547278,48.149716965283,0
-0.85154015571106,48.149625983786,0
-0.85148543543503,48.149537848835,0
-0.85141941040306,48.149453231175,0
-0.85134258314525,48.149372774778,0
-0.85125553839274,48.149297091945,0
-0.85115893862659,48.149226758645,0
-0.85105351903539,48.149162310134,0
-0.85094008191982,48.149104236882,0
-0.8508194905869,48.149052980838,0
-0.85069266278015,48.149008932071,0
-0.85056056369607,48.148972425799,0
-0.85042419863938,48.148943739842,0
-0.85028460537375,48.148923092504,0
-0.85014284622538,48.148910640912,0
-0.85000000000002,48.148906479826,0
-0.84985715377467,48.148910640912,0
-0.8497153946263,48.148923092504,0
-0.84957580136067,48.148943739842,0
-0.84943943630398,48.148972425799,0
-0.84930733721989,48.149008932071,0
-0.84918050941315,48.149052980838,0
-0.84905991808023,48.149104236882,0
-0.84894648096465,48.149162310134,0
-0.84884106137345,48.149226758645,0
-0.8487444616073,48.149297091945,0
-0.8486574168548,48.149372774778,0
-0.84858058959698,48.149453231175,0
-0.84851456456502,48.149537848835,0
-0.84845984428899,48.149625983786,0
-0.84841684527225,48.149716965283,0

-0.84838589482005,48.149810100915,0
-0.84836722854688,48.149904681875,0
-0.84836098858128,48.149999988349,0
-0.85000000000002,48.15,0
</coordinates>
</LinearRing>
</outerBoundaryIs>
</Polygon>
</Placemark>
<Placemark>
<name>110</name>
<styleUrl>#stylehap2</styleUrl>
<Polygon>
<outerBoundaryIs>
<LinearRing>
<coordinates>
-0.84899999999993,48.15,0
-0.84736098858119,48.149999988349,0
-0.84736722248213,48.150095295001,0
-0.84738588287492,48.150189876486,0
-0.84741682780967,48.150283012977,0
-0.84745982183958,48.150373995639,0
-0.84751453781087,48.150462132025,0
-0.84758055935102,48.150546751349,0
-0.84765738403597,48.150627209586,0
-0.84774442721285,48.150702894381,0
-0.84784102644841,48.150773229705,0
-0.8479464465702,48.150837680238,0
-0.84805988526142,48.150895755453,0
-0.84818047916718,48.150947013337,0
-0.84830731046572,48.150991063768,0
-0.84843941385457,48.151027571475,0
-0.84857578389809,48.151056258597,0
-0.84871538268116,48.151076906794,0
-0.84885714770993,48.151089358911,0
-0.84899999999994,48.151093520174,0
-0.84914285228995,48.151089358911,0
-0.84928461731868,48.151076906794,0
-0.84942421610178,48.151056258597,0
-0.8495605861453,48.151027571475,0
-0.84969268953413,48.150991063768,0
-0.84981952083269,48.150947013337,0
-0.84994011473845,48.150895755453,0
-0.85005355342967,48.150837680238,0
-0.85015897355146,48.150773229705,0
-0.85025557278702,48.150702894381,0
-0.85034261596388,48.150627209586,0
-0.85041944064886,48.150546751349,0
-0.850485462189,48.150462132025,0
-0.85054017816029,48.150373995639,0
-0.85058317219018,48.150283012977,0
-0.85061411712493,48.150189876486,0
-0.85063277751771,48.150095295001,0
-0.85063901141868,48.149999988349,0
-0.85063277145308,48.149904681875,0
-0.85061410517988,48.149810100915,0
-0.85058315472771,48.149716965283,0

-0.85054015571097,48.149625983786,0
-0.85048543543494,48.149537848835,0
-0.85041941040295,48.149453231175,0
-0.85034258314516,48.149372774778,0
-0.85025553839266,48.149297091945,0
-0.85015893862651,48.149226758645,0
-0.85005351903531,48.149162310134,0
-0.84994008191973,48.149104236882,0
-0.84981949058679,48.149052980838,0
-0.84969266278007,48.149008932071,0
-0.84956056369598,48.148972425799,0
-0.84942419863929,48.148943739842,0
-0.84928460537367,48.148923092504,0
-0.84914284622529,48.148910640912,0
-0.84899999999994,48.148906479826,0
-0.84885715377459,48.148910640912,0
-0.84871539462621,48.148923092504,0
-0.84857580136059,48.148943739842,0
-0.84843943630389,48.148972425799,0
-0.84830733721978,48.149008932071,0
-0.84818050941306,48.149052980838,0
-0.84805991808014,48.149104236882,0
-0.84794648096457,48.149162310134,0
-0.84784106137337,48.149226758645,0
-0.84774446160722,48.149297091945,0
-0.84765741685471,48.149372774778,0
-0.8475805895969,48.149453231175,0
-0.84751456456493,48.149537848835,0
-0.8474598442889,48.149625983786,0
-0.84741684527216,48.149716965283,0
-0.84738589481997,48.149810100915,0
-0.84736722854679,48.149904681875,0
-0.84736098858119,48.149999988349,0
-0.84899999999993,48.15,0
</coordinates>
</LinearRing>
</outerBoundaryIs>
</Polygon>
</Placemark>
<Placemark>
<name>113</name>
<styleUrl>#stylehap2</styleUrl>
<Polygon>
<outerBoundaryIs>
<LinearRing>
<coordinates>
-0.84799999999996,48.151,0
-0.84636095664226,48.150999988349,0
-0.84636719066457,48.151095295,0
-0.84638585142088,48.151189876486,0
-0.84641679695856,48.151283012976,0
-0.84645979182618,48.151373995638,0
-0.84651450886362,48.151462132025,0
-0.84658053169026,48.151546751348,0
-0.84665735787223,48.151627209586,0
-0.84674440274527,48.151702894381,0
-0.8468410038632,48.151773229704,0

-0.8469464260393,48.151837680238,0
-0.84705986694111,48.151895755452,0
-0.84718046319689,48.151947013337,0
-0.84730729696703,48.151991063768,0
-0.8474394029302,48.152027571475,0
-0.84757577563124,48.152056258597,0
-0.84771537713473,48.152076906794,0
-0.84785714492612,48.152089358911,0
-0.84799999999995,48.152093520174,0
-0.84814285507379,48.152089358911,0
-0.84828462286518,48.152076906794,0
-0.84842422436869,48.152056258597,0
-0.8485605970697l,48.152027571475,0
-0.84869270303288,48.151991063768,0
-0.84881953680304,48.151947013337,0
-0.84894013305882,48.151895755452,0
-0.8490535739606,48.151837680238,0
-0.8491589961367,48.151773229704,0
-0.84925559725466,48.151702894381,0
-0.84934264212767,48.151627209586,0
-0.84941946830967,48.151546751348,0
-0.84948549113629,48.151462132025,0
-0.84954020817375,48.151373995638,0
-0.84958320304137,48.151283012976,0
-0.84961414857903,48.151189876486,0
-0.84963280933534,48.151095295,0
-0.84963904335767,48.150999988349,0
-0.84963280327035,48.150904681875,0
-0.84961413663335,48.150810100915,0
-0.84958318557794,48.150716965282,0
-0.84954018572318,48.150625983785,0
-0.84948546438075,48.150537848835,0
-0.84941943806211,48.150453231175,0
-0.84934260930717,48.150372774778,0
-0.84925556285841,48.150297091945,0
-0.84915896120983,48.150226758645,0
-0.84905353956435,48.150162310134,0
-0.84894010023829,48.150104236881,0
-0.84881950655548,48.150052980838,0
-0.84869267627736,48.150008932071,0
-0.84856057461916,48.149972425799,0
-0.84842420690526,48.149943739842,0
-0.8482846109195,48.149923092504,0
-0.8481428490088,48.149910640912,0
-0.84799999999995,48.149906479826,0
-0.84785715099111,48.149910640912,0
-0.84771538908043,48.149923092504,0
-0.84757579309467,48.149943739842,0
-0.84743942538077,48.149972425799,0
-0.84730732372257,48.150008932071,0
-0.84718049344443,48.150052980838,0
-0.84705989976161,48.150104236881,0
-0.84694646043555,48.150162310134,0
-0.84684103879007,48.150226758645,0
-0.84674443714149,48.150297091945,0
-0.84665739069276,48.150372774778,0
-0.84658056193779,48.150453231175,0

-0.84651453561915,48.150537848835,0
-0.84645981427672,48.150625983785,0
-0.84641681442199,48.150716965282,0
-0.84638586336658,48.150810100915,0
-0.84636719672956,48.150904681875,0
-0.84636095664226,48.150999988349,0
-0.84799999999996,48.151,0
</coordinates>
</LinearRing>
</outerBoundaryIs>
</Polygon>
</Placemark>
<Placemark>
<name>114</name>
<styleUrl>#stylehap2</styleUrl>
<Polygon>
<outerBoundaryIs>
<LinearRing>
<coordinates>
-0.84699999999998,48.191,0
-0.84535967765205,48.190999988333,0
-0.84536591653468,48.191095294984,0
-0.84538459184837,48.19118987647,0
-0.84541556152979,48.191283012961,0
-0.84545858994397,48.191373995624,0
-0.84551334967559,48.191462132011,0
-0.84557942401918,48.191546751336,0
-0.84565631014891,48.191627209575,0
-0.84574342294432,48.191702894372,0
-0.8458400994424,48.191773229696,0
-0.84594560388265,48.191837680232,0
-0.84605913330658,48.191895755447,0
-0.84617982366871,48.191947013333,0
-0.84630675641317,48.191991063765,0
-0.84643896546525,48.192027571473,0
-0.84657544458513,48.192056258596,0
-0.84671515502737,48.192076906793,0
-0.84685703344818,48.19208935891,0
-0.84699999999997,48.192093520174,0
-0.84714296655178,48.19208935891,0
-0.84728484497259,48.192076906793,0
-0.84742455541483,48.192056258596,0
-0.84756103453471,48.192027571473,0
-0.84769324358679,48.191991063765,0
-0.84782017633125,48.191947013333,0
-0.84794086669339,48.191895755447,0
-0.84805439611731,48.191837680232,0
-0.84815990055757,48.191773229696,0
-0.84825657705564,48.191702894372,0
-0.84834368985105,48.191627209575,0
-0.84842057598078,48.191546751336,0
-0.84848665032437,48.191462132011,0
-0.84854141005599,48.191373995624,0
-0.84858443847017,48.191283012961,0
-0.84861540815159,48.19118987647,0
-0.84863408346528,48.191095294984,0
-0.84864032234791,48.190999988333,0

-0.84863407738703,48.190904681858,0
-0.84861539617978,48.190810100899,0
-0.84858442096855,48.190716965267,0
-0.84854138755634,48.190625983771,0
-0.84848662351031,48.190537848822,0
-0.84842054566709,48.190453231163,0
-0.84834365695875,48.190372774767,0
-0.84825654258418,48.190297091935,0
-0.84815986555431,48.190226758636,0
-0.84805436164582,48.190162310127,0
-0.84794083380108,48.190104236876,0
-0.84782014601754,48.190052980833,0
-0.84769321677275,48.190008932068,0
-0.84756101203504,48.189972425797,0
-0.84742453791321,48.189943739841,0
-0.84728483300078,48.189923092503,0
-0.84714296047354,48.189910640912,0
-0.84699999999997,48.189906479827,0
-0.84685703952642,48.189910640912,0
-0.84671516699918,48.189923092503,0
-0.84657546208675,48.189943739841,0
-0.84643898796492,48.189972425797,0
-0.84630678322721,48.190008932068,0
-0.84617985398242,48.190052980833,0
-0.84605916619888,48.190104236876,0
-0.84594563835414,48.190162310127,0
-0.84584013444565,48.190226758636,0
-0.84574345741578,48.190297091935,0
-0.84565634304122,48.190372774767,0
-0.84557945433287,48.190453231163,0
-0.84551337648965,48.190537848822,0
-0.84545861244362,48.190625983771,0
-0.84541557903142,48.190716965267,0
-0.84538460382018,48.190810100899,0
-0.84536592261293,48.190904681858,0
-0.84535967765205,48.190999988333,0
-0.84699999999998,48.191,0
</coordinates>
</LinearRing>
</outerBoundaryIs>
</Polygon>
</Placemark>
<Placemark>
<name>115</name>
<styleUrl>#stylehap2</styleUrl>
<Polygon>
<outerBoundaryIs>
<LinearRing>
<coordinates>
-0.84500000000003,48.114,0
-0.84336213722364,48.113999988364,0
-0.84336836675959,48.114095295015,0
-0.84338701407864,48.1141898765,0
-0.84341793733023,48.114283012991,0
-0.84346090123242,48.114373995652,0
-0.84351557886073,48.114462132037,0
-0.84358155413424,48.11454675136,0

-0.84365832498102,48.114627209596,0
-0.84374530715779,48.11470289439,0
-0.84384183869557,48.114773229712,0
-0.84394718493713,48.114837680245,0
-0.84406054412792,48.114895755457,0
-0.84418105351812,48.114947013341,0
-0.84430779592931,48.11499106377,0
-0.8444398067355,48.115027571477,0
-0.84457608120585,48.115056258598,0
-0.84471558215264,48.115076906794,0
-0.84485724782675,48.115089358911,0
-0.84500000000002,48.115093520173,0
-0.8451427521733,48.115089358911,0
-0.84528441784741,48.115076906794,0
-0.8454239187942,48.115056258598,0
-0.84556019326455,48.115027571477,0
-0.84569220407074,48.11499106377,0
-0.84581894648193,48.114947013341,0
-0.84593945587216,48.114895755457,0
-0.84605281506292,48.114837680245,0
-0.84615816130447,48.114773229712,0
-0.84625469284228,48.11470289439,0
-0.84634167501905,48.114627209596,0
-0.84641844586581,48.11454675136,0
-0.84648442113935,48.114462132037,0
-0.84653909876762,48.114373995652,0
-0.84658206266985,48.114283012991,0
-0.84661298592143,48.1141898765,0
-0.84663163324046,48.114095295015,0
-0.84663786277643,48.113999988364,0
-0.84663162718771,48.11390468189,0
-0.84661297399985,48.11381010093,0
-0.84658204524165,48.113716965296,0
-0.84653907636237,48.113625983799,0
-0.8464843944378,48.113537848847,0
-0.84641841567929,48.113453231186,0
-0.84634164226476,48.113372774788,0
-0.84625465851545,48.113297091953,0
-0.84615812644809,48.113226758652,0
-0.84605278073609,48.11316231014,0
-0.84593942311786,48.113104236886,0
-0.84581891629541,48.113052980841,0
-0.84569217736919,48.113008932073,0
-0.84556017085929,48.112972425801,0
-0.84542390136601,48.112943739843,0
-0.84528440592582,48.112923092504,0
-0.84514274612055,48.112910640913,0
-0.84500000000002,48.112906479826,0
-0.8448572538795,48.112910640913,0
-0.84471559407422,48.112923092504,0
-0.84457609863404,48.112943739843,0
-0.84443982914075,48.112972425801,0
-0.84430782263086,48.113008932073,0
-0.84418108370463,48.113052980841,0
-0.84406057688219,48.113104236886,0
-0.84394721926396,48.11316231014,0
-0.84384187355196,48.113226758652,0

-0.84374534148463,48.113297091953,0
-0.84365835773529,48.113372774788,0
-0.84358158432075,48.113453231186,0
-0.84351560556225,48.113537848847,0
-0.84346092363768,48.113625983799,0
-0.84341795475842,48.113716965296,0
-0.84338702600023,48.11381010093,0
-0.84336837281234,48.11390468189,0
-0.84336213722364,48.113999988364,0
-0.84500000000003,48.114,0
</coordinates>
</LinearRing>
</outerBoundaryIs>
</Polygon>
</Placemark>
<Placemark>
<name>117</name>
<styleUrl>#stylehap2</styleUrl>
<Polygon>
<outerBoundaryIs>
<LinearRing>
<coordinates>
-0.84299999999996,48.26,0
-0.84135746481249,48.259999988304,0
-0.84136371210415,48.260095294956,0
-0.84138241260419,48.260189876442,0
-0.84141342405785,48.260283012935,0
-0.84145651051244,48.260373995599,0
-0.8415113441112,48.260462131988,0
-0.8415775075866,48.260546751315,0
-0.84165449743471,48.260627209556,0
-0.84174172774573,48.260702894355,0
-0.84183853466238,48.260773229682,0
-0.84194418143166,48.26083768022,0
-0.84205786401179,48.260895755438,0
-0.84217871719166,48.260947013326,0
-0.84230582117607,48.26099106376,0
-0.84243820858703,48.26102757147,0
-0.84257487182703,48.261056258594,0
-0.84271477074918,48.261076906792,0
-0.842856840575,48.26108935891,0
-0.84299999999995,48.261093520173,0
-0.84314315942493,48.26108935891,0
-0.84328522925075,48.261076906792,0
-0.8434251281729,48.261056258594,0
-0.8435617914129,48.26102757147,0
-0.84369417882384,48.26099106376,0
-0.84382128280827,48.260947013326,0
-0.84394213598814,48.260895755438,0
-0.84405581856827,48.26083768022,0
-0.84416146533756,48.260773229682,0
-0.8442582722542,48.260702894355,0
-0.84434550256522,48.260627209556,0
-0.8444224924133,48.260546751315,0
-0.84448865588873,48.260462131988,0
-0.84454348948747,48.260373995599,0
-0.84458657594208,48.260283012935,0

-0.84461758739574,48.260189876442,0
-0.84463628789575,48.260095294956,0
-0.84464253518741,48.259999988304,0
-0.84463628179453,48.25990468183,0
-0.84461757537866,48.259810100872,0
-0.8445865583743,48.259716965241,0
-0.84454346690278,48.259625983746,0
-0.84448862897335,48.259537848798,0
-0.84442246198504,48.259453231142,0
-0.8443454695486,48.259372774748,0
-0.84425823765246,48.259297091918,0
-0.84416143020202,48.259226758622,0
-0.8440557839665,48.259162310115,0
-0.84394210297151,48.259104236867,0
-0.84382125238001,48.259052980826,0
-0.84369415190846,48.259008932063,0
-0.84356176882821,48.258972425794,0
-0.84342511060514,48.258943739839,0
-0.84328521723368,48.258923092502,0
-0.84314315332371,48.258910640912,0
-0.84299999999995,48.258906479826,0
-0.84285684667622,48.258910640912,0
-0.84271478276623,48.258923092502,0
-0.84257488939479,48.258943739839,0
-0.84243823117172,48.258972425794,0
-0.84230584809147,48.259008932063,0
-0.84217874761992,48.259052980826,0
-0.84205789702839,48.259104236867,0
-0.8419442160334,48.259162310115,0
-0.84183856979791,48.259226758622,0
-0.84174176234747,48.259297091918,0
-0.84165453045131,48.259372774748,0
-0.8415775380149,48.259453231142,0
-0.84151137102658,48.259537848798,0
-0.84145653309713,48.259625983746,0
-0.8414134416256,48.259716965241,0
-0.84138242462124,48.259810100872,0
-0.8413637182054,48.25990468183,0
-0.84135746481249,48.259999988304,0
-0.84299999999996,48.26,0
</coordinates>
</LinearRing>
</outerBoundaryIs>
</Polygon>
</Placemark>
<Placemark>
<name>118</name>
<styleUrl>#stylehap2</styleUrl>
<Polygon>
<outerBoundaryIs>
<LinearRing>
<coordinates>
-0.83799999999997,48.222,0
-0.8363586845103,48.22199998832,0
-0.83636492716698,48.222095294971,0
-0.83638361378451,48.222189876457,0
-0.83641460221367,48.222283012949,0

-0.8364576566769,48.222373995613,0
-0.83651244956074,48.222462132001,0
-0.83657856390746,48.222546751327,0
-0.83665549658693,48.222627209567,0
-0.83674266212435,48.222702894364,0
-0.83683939715542,48.22277322969,0
-0.83694496547419,48.222837680226,0
-0.83705856363599,48.222895755443,0
-0.83717932707213,48.22294701333,0
-0.83730633667058,48.222991063762,0
-0.83743862577169,48.223027571471,0
-0.83757518752627,48.223056258595,0
-0.83771498255995,48.223076906793,0
-0.83785694688499,48.22308935891,0
-0.83799999999995,48.223093520174,0
-0.83814305311494,48.22308935891,0
-0.83828501743998,48.223076906793,0
-0.83842481247366,48.223056258595,0
-0.83856137422825,48.223027571471,0
-0.83869366332936,48.222991063762,0
-0.83882067292781,48.22294701333,0
-0.83894143636394,48.222895755443,0
-0.83905503452574,48.222837680226,0
-0.83916060284451,48.22277322969,0
-0.83925733787558,48.222702894364,0
-0.839344503413,48.222627209567,0
-0.83942143609247,48.222546751327,0
-0.83948755043919,48.222462132001,0
-0.83954234332304,48.222373995613,0
-0.83958539778626,48.222283012949,0
-0.83961638621542,48.222189876457,0
-0.83963507283295,48.222095294971,0
-0.83964131548963,48.22199998832,0
-0.8396350667444,48.221904681846,0
-0.83961637422331,48.221810100887,0
-0.83958538025494,48.221716965255,0
-0.83954232078522,48.22162598376,0
-0.83948752357966,48.221537848811,0
-0.83942140572736,48.221453231153,0
-0.83934447046493,48.221372774758,0
-0.83925730334564,48.221297091927,0
-0.83916056778189,48.22122675863,0
-0.8390549999958,48.221162310122,0
-0.83894140341589,48.221104236872,0
-0.83882064256269,48.22105298083,0
-0.83869363646983,48.221008932065,0
-0.83856135169045,48.220972425796,0
-0.83842479494234,48.22094373984,0
-0.83828500544787,48.220923092503,0
-0.83814304702637,48.220910640912,0
-0.83799999999995,48.220906479827,0
-0.83785695297356,48.220910640912,0
-0.83771499455206,48.220923092503,0
-0.83757520505759,48.22094373984,0
-0.83743864830948,48.220972425796,0
-0.83730636353011,48.221008932065,0
-0.83717935743724,48.22105298083,0

-0.83705859658404,48.221104236872,0
-0.83694500000413,48.221162310122,0
-0.83683943221804,48.22122675863,0
-0.83674269665429,48.221297091927,0
-0.83665552953501,48.221372774758,0
-0.83657859427258,48.221453231153,0
-0.83651247642025,48.221537848811,0
-0.83645767921471,48.22162598376,0
-0.83641461974499,48.221716965255,0
-0.83638362577662,48.221810100887,0
-0.83636493325553,48.221904681846,0
-0.8363586845103,48.22199998832,0
-0.83799999999997,48.222,0
</coordinates>
</LinearRing>
</outerBoundaryIs>
</Polygon>
</Placemark>
<Placemark>
<name>120</name>
<styleUrl>#stylehap2</styleUrl>
<Polygon>
<outerBoundaryIs>
<LinearRing>
<coordinates>
-0.83600000000001,48.156,0
-0.8343607969213,48.155999988347,0
-0.83436703155058,48.156095294998,0
-0.83438569412482,48.156189876484,0
-0.83441664267757,48.156283012975,0
-0.83445964173451,48.156373995636,0
-0.83451436410363,48.156462132023,0
-0.83458039336372,48.156546751347,0
-0.83465722703203,48.156627209585,0
-0.83474428038722,48.15670289438,0
-0.83484089091869,48.156773229703,0
-0.83494632336798,48.156837680237,0
-0.83505977532448,48.156895755452,0
-0.83518038333231,48.156947013336,0
-0.83530722946241,48.156991063767,0
-0.83543934829939,48.157027571475,0
-0.83557573429006,48.157056258597,0
-0.8357153493979,48.157076906794,0
-0.83585713100476,48.157089358911,0
-0.83600000000001,48.157093520174,0
-0.83614286899528,48.157089358911,0
-0.83628465060214,48.157076906794,0
-0.83642426570996,48.157056258597,0
-0.83656065170063,48.157027571475,0
-0.83669277053761,48.156991063767,0
-0.83681961666771,48.156947013336,0
-0.83694022467556,48.156895755452,0
-0.83705367663204,48.156837680237,0
-0.83715910908133,48.156773229703,0
-0.8372557196128,48.15670289438,0
-0.83734277296801,48.156627209585,0
-0.8374196066363,48.156546751347,0

-0.83748563589639,48.156462132023,0
-0.83754035826553,48.156373995636,0
-0.83758335732245,48.156283012975,0
-0.83761430587519,48.156189876484,0
-0.83763296844946,48.156095294998,0
-0.83763920307872,48.155999988347,0
-0.83763296238282,48.155904681873,0
-0.83761429392626,48.155810100913,0
-0.83758333985426,48.15571696528,0
-0.83754033580883,48.155625983784,0
-0.83748560913355,48.155537848833,0
-0.8374195763805,48.155453231174,0
-0.83734274013853,48.155372774777,0
-0.83725568520716,48.155297091943,0
-0.83715907414492,48.155226758644,0
-0.8370536422264,48.155162310133,0
-0.83694019184608,48.155104236881,0
-0.83681958641191,48.155052980837,0
-0.83669274377477,48.15500893207,0
-0.83656062924395,48.154972425799,0
-0.83642424824176,48.154943739842,0
-0.83628463865318,48.154923092504,0
-0.83614286292864,48.154910640912,0
-0.83600000000001,48.154906479827,0
-0.8358571370714,48.154910640912,0
-0.83571536134684,48.154923092504,0
-0.83557575175825,48.154943739842,0
-0.83543937075607,48.154972425799,0
-0.83530725622525,48.15500893207,0
-0.83518041358811,48.155052980837,0
-0.83505980815394,48.155104236881,0
-0.83494635777361,48.155162310133,0
-0.83484092585509,48.155226758644,0
-0.83474431479286,48.155297091943,0
-0.83465725986149,48.155372774777,0
-0.83458042361952,48.155453231174,0
-0.83451439086647,48.155537848833,0
-0.83445966419119,48.155625983784,0
-0.83441666014578,48.15571696528,0
-0.83438570607379,48.155810100913,0
-0.83436703761722,48.155904681873,0
-0.8343607969213,48.155999988347,0
-0.83600000000001,48.156,0
</coordinates>
</LinearRing>
</outerBoundaryIs>
</Polygon>
</Placemark>
<Placemark>
<name>122</name>
<styleUrl>#stylehap2</styleUrl>
<Polygon>
<outerBoundaryIs>
<LinearRing>
<coordinates>
-0.83400000000006,48.155,0
-0.83236082886903,48.154999988347,0

-0.8323670633769,48.155095294999,0
-0.83238572558754,48.155189876484,0
-0.8324166735372,48.155283012975,0
-0.83245967175618,48.155373995637,0
-0.83251439305884,48.155462132023,0
-0.83258042103211,48.155546751347,0
-0.83265725320299,48.155627209585,0
-0.83274430486157,48.15570289438,0
-0.83284091351012,48.155773229704,0
-0.83294634390454,48.155837680238,0
-0.83305979364985,48.155895755452,0
-0.83318039930702,48.155947013337,0
-0.83330724296488,48.155991063767,0
-0.83343935922679,48.156027571475,0
-0.83357574255925,48.156056258597,0
-0.83371535494591,48.156076906794,0
-0.83385713378938,48.156089358911,0
-0.83400000000007,48.156093520174,0
-0.83414286621073,48.156089358911,0
-0.83428464505419,48.156076906794,0
-0.83442425744085,48.156056258597,0
-0.83456064077331,48.156027571475,0
-0.83469275703525,48.155991063767,0
-0.83481960069309,48.155947013337,0
-0.83494020635026,48.155895755452,0
-0.83505365609556,48.155837680238,0
-0.83515908648999,48.155773229704,0
-0.83525569513856,48.15570289438,0
-0.83534274679714,48.155627209585,0
-0.83541957896802,48.155546751347,0
-0.83548560694126,48.155462132023,0
-0.83554032824395,48.155373995637,0
-0.83558332646291,48.155283012975,0
-0.83561427441259,48.155189876484,0
-0.83563293662321,48.155095294999,0
-0.83563917113107,48.154999988347,0
-0.8356329305569,48.154904681873,0
-0.83561426246429,48.154810100913,0
-0.83558330899569,48.154716965281,0
-0.8355403057885,48.154625983784,0
-0.8354855801799,48.154537848834,0
-0.83541954871385,48.154453231174,0
-0.83534271396947,48.154372774777,0
-0.83525566073481,48.154297091944,0
-0.83515905155551,48.154226758644,0
-0.83505362169181,48.154162310133,0
-0.83494017352258,48.154104236881,0
-0.83481957043894,48.154052980837,0
-0.83469273027388,48.15400893207,0
-0.83456061831786,48.153972425799,0
-0.83442423997363,48.153943739842,0
-0.83428463310589,48.153923092504,0
-0.83414286014441,48.153910640912,0
-0.83400000000007,48.153906479827,0
-0.83385713985569,48.153910640912,0
-0.83371536689422,48.153923092504,0
-0.8335757600265,48.153943739842,0

-0.83343938168227,48.153972425799,0
-0.83330726972625,48.15400893207,0
-0.83318042956119,48.154052980837,0
-0.83305982647755,48.154104236881,0
-0.83294637830832,48.154162310133,0
-0.83284094844462,48.154226758644,0
-0.83274433926532,48.154297091944,0
-0.83265728603066,48.154372774777,0
-0.83258045128625,48.154453231174,0
-0.83251441982023,48.154537848834,0
-0.83245969421163,48.154625983784,0
-0.83241669100445,48.154716965281,0
-0.83238573753584,48.154810100913,0
-0.83236706944321,48.154904681873,0
-0.83236082886903,48.154999988347,0
-0.83400000000006,48.155,0
</coordinates>
</LinearRing>
</outerBoundaryIs>
</Polygon>
</Placemark>
<Placemark>
<name>125</name>
<styleUrl>#stylehap2</styleUrl>
<Polygon>
<outerBoundaryIs>
<LinearRing>
<coordinates>
-0.82899999999995,48.203,0
-0.82735929340964,48.202999988328,0
-0.82736553375243,48.203095294979,0
-0.82738421343953,48.203189876465,0
-0.82741519037437,48.203283012957,0
-0.82745822886684,48.203373995619,0
-0.8275130014249,48.203462132007,0
-0.82757909124553,48.203546751332,0
-0.82765599538516,48.203627209572,0
-0.82774312858624,48.203702894369,0
-0.82783982773048,48.203773229694,0
-0.82794535688507,48.20383768023,0
-0.82805891290339,48.203895755445,0
-0.82817963153755,48.203947013332,0
-0.82830659401649,48.203991063764,0
-0.8284388340392,48.204027571472,0
-0.82857534513012,48.204056258595,0
-0.82871508830042,48.204076906793,0
-0.82885699995721,48.20408935891,0
-0.82899999999996,48.204093520174,0
-0.82914300004269,48.20408935891,0
-0.82928491169948,48.204076906793,0
-0.82942465486978,48.204056258595,0
-0.8295611659607,48.204027571472,0
-0.82969340598341,48.203991063764,0
-0.82982036846235,48.203947013332,0
-0.82994108709651,48.203895755445,0
-0.83005464311483,48.20383768023,0
-0.83016017226942,48.203773229694,0

-0.83025687141366,48.203702894369,0
-0.83034400461474,48.203627209572,0
-0.83042090875437,48.203546751332,0
-0.830486998575,48.203462132007,0
-0.83054177113307,48.203373995619,0
-0.83058480962553,48.203283012957,0
-0.83061578656037,48.203189876465,0
-0.83063446624747,48.203095294979,0
-0.83064070659026,48.202999988328,0
-0.83063446016523,48.202904681853,0
-0.8306157745807,48.202810100894,0
-0.8305847921124,48.202716965263,0
-0.83054174861866,48.202625983767,0
-0.83048697174336,48.202537848818,0
-0.83042087842079,48.202453231159,0
-0.83034397170086,48.202372774764,0
-0.83025683691958,48.202297091932,0
-0.83016013724322,48.202226758634,0
-0.83005460862075,48.202162310125,0
-0.82994105418263,48.202104236874,0
-0.82982033812876,48.202052980832,0
-0.82969337915177,48.202008932067,0
-0.82956114344627,48.201972425797,0
-0.82942463735668,48.201943739841,0
-0.8292848997198,48.201923092503,0
-0.82914299396045,48.201910640912,0
-0.82899999999996,48.201906479826,0
-0.82885700603945,48.201910640912,0
-0.8287151002801,48.201923092503,0
-0.82857536264322,48.201943739841,0
-0.82843885655364,48.201972425797,0
-0.82830662084813,48.202008932067,0
-0.82817966187114,48.202052980832,0
-0.82805894581728,48.202104236874,0
-0.82794539137915,48.202162310125,0
-0.82783986275668,48.202226758634,0
-0.82774316308033,48.202297091932,0
-0.82765602829904,48.202372774764,0
-0.82757912157911,48.202453231159,0
-0.82751302825654,48.202537848818,0
-0.82745825138124,48.202625983767,0
-0.8274152078875,48.202716965263,0
-0.8273842254192,48.202810100894,0
-0.82736553983467,48.202904681853,0
-0.82735929340964,48.202999988328,0
-0.82899999999995,48.203,0
</coordinates>
</LinearRing>
</outerBoundaryIs>
</Polygon>
</Placemark>
<Placemark>
<name>131</name>
<styleUrl>#stylehap2</styleUrl>
<Polygon>
<outerBoundaryIs>
<LinearRing>

<coordinates>
-0.79600000000005,47.877,0
-0.79436964310243,47.87699998846,0
-0.79437584411509,47.877095295111,0
-0.79439440600274,47.877189876594,0
-0.7944251875642,47.87728301308,0
-0.79446795459507,47.877373995737,0
-0.79452238166839,47.877462132116,0
-0.79458805460959,47.877546751432,0
-0.79466447364708,47.877627209661,0
-0.79475105721473,47.877702894447,0
-0.79484714637697,47.87777322976,0
-0.79495200984322,47.877837680284,0
-0.79506484953323,47.877895755489,0
-0.79518480665118,47.877947013365,0
-0.79531096822204,47.877991063787,0
-0.79544237404098,47.878027571488,0
-0.79557802398204,47.878056258604,0
-0.79571688561131,47.878076906797,0
-0.79585790204605,47.878089358911,0
-0.79600000000004,47.878093520174,0
-0.79614209795405,47.878089358911,0
-0.79628311438879,47.878076906797,0
-0.79642197601806,47.878056258604,0
-0.79655762595911,47.878027571488,0
-0.79668903177805,47.877991063787,0
-0.79681519334892,47.877947013365,0
-0.79693515046687,47.877895755489,0
-0.79704799015688,47.877837680284,0
-0.79715285362313,47.87777322976,0
-0.79724894278537,47.877702894447,0
-0.79733552635302,47.877627209661,0
-0.79741194539051,47.877546751432,0
-0.79747761833171,47.877462132116,0
-0.79753204540503,47.877373995737,0
-0.7975748124359,47.87728301308,0
-0.79760559399736,47.877189876594,0
-0.79762415588501,47.877095295111,0
-0.79763035689767,47.87699998846,0
-0.79762414990992,47.876904681985,0
-0.79760558222872,47.876810101023,0
-0.79757479523129,47.876716965386,0
-0.79753202328722,47.876625983884,0
-0.7974775919727,47.876537848927,0
-0.79741191559126,47.876453231259,0
-0.79733549401892,47.876372774853,0
-0.7972489088989,47.87629709201,0
-0.7971528192139,47.8762267587,0
-0.79704795627041,47.87616231018,0
-0.79693511813277,47.876104236918,0
-0.79681516354967,47.876052980865,0
-0.79668900541904,47.87600893209,0
-0.79655760384128,47.875972425812,0
-0.79642195881345,47.87594373985,0
-0.79628310262015,47.875923092507,0
-0.79614209197896,47.875910640913,0
-0.79600000000004,47.875906479826,0

-0.79585790802114,47.875910640913,0
-0.79571689737995,47.875923092507,0
-0.79557804118665,47.87594373985,0
-0.79544239615882,47.875972425812,0
-0.79531099458106,47.87600893209,0
-0.79518483645043,47.876052980865,0
-0.79506488186733,47.876104236918,0
-0.79495204372969,47.87616231018,0
-0.7948471807862,47.8762267587,0
-0.7947510911012,47.87629709201,0
-0.79466450598118,47.876372774853,0
-0.79458808440884,47.876453231259,0
-0.7945224080274,47.876537848927,0
-0.79446797671288,47.876625983884,0
-0.79442520476881,47.876716965386,0
-0.79439441777138,47.876810101023,0
-0.79437585009018,47.876904681985,0
-0.79436964310243,47.87699998846,0
-0.79600000000005,47.877,0
</coordinates>
</LinearRing>
</outerBoundaryIs>
</Polygon>
</Placemark>
<Placemark>
<name>135</name>
<styleUrl>#stylehap2</styleUrl>
<Polygon>
<outerBoundaryIs>
<LinearRing>
<coordinates>
-0.78800000000001,48.034,0
-0.78636468169857,48.033999988397,0
-0.78637090156519,48.034095295048,0
-0.78638951992321,48.034189876532,0
-0.78642039514221,48.034283013021,0
-0.78646329230553,48.03437399568,0
-0.78651788499628,48.034462132064,0
-0.78658375777988,48.034546751384,0
-0.78666040936416,48.034627209618,0
-0.78674725641338,48.034702894409,0
-0.78684363798692,48.034773229728,0
-0.7869488205688,48.034837680258,0
-0.78706200365007,48.034895755468,0
-0.78718232582129,48.034947013349,0
-0.787308871329,48.034991063776,0
-0.78744067704595,48.03502757148,0
-0.78757673980235,48.0350562586,0
-0.78771602402202,48.035076906795,0
-0.78785746960552,48.035089358911,0
-0.78800000000001,48.035093520174,0
-0.7881425303945,48.035089358911,0
-0.78828397597799,48.035076906795,0
-0.78842326019767,48.0350562586,0
-0.78855932295406,48.03502757148,0
-0.78869112867101,48.034991063776,0
-0.78881767417872,48.034947013349,0

-0.78893799634995,48.034895755468,0
-0.78905117943121,48.034837680258,0
-0.7891563620131,48.034773229728,0
-0.78925274358663,48.034702894409,0
-0.78933959063586,48.034627209618,0
-0.78941624222014,48.034546751384,0
-0.78948211500373,48.034462132064,0
-0.78953670769451,48.03437399568,0
-0.78957960485783,48.034283013021,0
-0.7896104800768,48.034189876532,0
-0.78962909843482,48.034095295048,0
-0.78963531830147,48.033999988397,0
-0.78962909240843,48.033904681922,0
-0.78961046820713,48.033810100961,0
-0.78957958750551,48.033716965327,0
-0.78953668538681,48.033625983828,0
-0.78948208841844,48.033537848874,0
-0.78941621216507,48.033453231211,0
-0.78933955802418,48.03337277481,0
-0.78925270940926,48.033297091973,0
-0.78915632730846,48.033226758668,0
-0.78905114525384,48.033162310154,0
-0.78893796373827,48.033104236897,0
-0.78881764412365,48.033052980849,0
-0.78869110208572,48.033008932079,0
-0.78855930064636,48.032972425805,0
-0.78842324284535,48.032943739846,0
-0.78828396410832,48.032923092505,0
-0.78814252436811,48.032910640913,0
-0.78800000000001,48.032906479827,0
-0.7878574756319,48.032910640913,0
-0.7877160358917,48.032923092505,0
-0.78757675715466,48.032943739846,0
-0.78744069935365,48.032972425805,0
-0.78730889791429,48.033008932079,0
-0.78718235587639,48.033052980849,0
-0.78706203626175,48.033104236897,0
-0.78694885474618,48.033162310154,0
-0.78684367269155,48.033226758668,0
-0.78674729059076,48.033297091973,0
-0.78666044197583,48.03337277481,0
-0.78658378783497,48.033453231211,0
-0.78651791158157,48.033537848874,0
-0.78646331461323,48.033625983828,0
-0.7864204124945,48.033716965327,0
-0.78638953179291,48.033810100961,0
-0.78637090759161,48.033904681922,0
-0.78636468169857,48.033999988397,0
-0.78800000000001,48.034,0
</coordinates>
</LinearRing>
</outerBoundaryIs>
</Polygon>
</Placemark>
<Placemark>
<name>136</name>
<styleUrl>#stylehap2</styleUrl>

<Polygon>
<outerBoundaryIs>
<LinearRing>
<coordinates>
-0.78800000000001,48.037,0
-0.78636458648095,48.036999988395,0
-0.78637080670943,48.037095295047,0
-0.7863894261512,48.037189876531,0
-0.78642030316764,48.03728301302,0
-0.78646320282841,48.037373995679,0
-0.78651779869766,48.037462132063,0
-0.78658367531654,48.037546751383,0
-0.78666033136378,48.037627209617,0
-0.78674718346967,48.037702894409,0
-0.78684357065506,48.037773229728,0
-0.78694875936132,48.037837680258,0
-0.78706194903282,48.037895755468,0
-0.78718227821004,48.037947013349,0
-0.78730883108613,48.037991063776,0
-0.78744064447779,48.03802757148,0
-0.78757671515679,48.0380562586,0
-0.78771600748667,48.038076906795,0
-0.78785746130625,48.038089358911,0
-0.78800000000001,48.038093520174,0
-0.78814253869376,48.038089358911,0
-0.78828399251334,48.038076906795,0
-0.78842328484323,48.0380562586,0
-0.78855935552223,48.03802757148,0
-0.78869116891388,48.037991063776,0
-0.78881772179,48.037947013349,0
-0.78893805096719,48.037895755468,0
-0.78905124063872,48.037837680258,0
-0.78915642934496,48.037773229728,0
-0.78925281653036,48.037702894409,0
-0.78933966863624,48.037627209617,0
-0.78941632468347,48.037546751383,0
-0.78948220130238,48.037462132063,0
-0.78953679717163,48.037373995679,0
-0.7895796968324,48.03728301302,0
-0.78961057384882,48.037189876531,0
-0.78962919329059,48.037095295047,0
-0.78963541351909,48.036999988395,0
-0.78962918726321,48.036904681921,0
-0.78961056197721,48.03681010096,0
-0.78957967947723,48.036716965326,0
-0.78953677486027,48.036625983827,0
-0.78948217471274,48.036537848873,0
-0.78941629462346,48.03645323121,0
-0.78933963601922,48.036372774809,0
-0.78925278234739,48.036297091972,0
-0.78915639463465,48.036226758668,0
-0.78905120645575,48.036162310153,0
-0.7889380183502,48.036104236897,0
-0.78881769172999,48.036052980849,0
-0.78869114232427,48.036008932079,0
-0.78855933321089,48.035972425805,0
-0.78842326748808,48.035943739846,0

-0.78828398064173,48.035923092505,0
-0.78814253266638,48.035910640913,0
-0.78800000000001,48.035906479827,0
-0.78785746733363,48.035910640913,0
-0.78771601935831,48.035923092505,0
-0.78757673251196,48.035943739846,0
-0.78744066678915,48.035972425805,0
-0.78730885767577,48.036008932079,0
-0.78718230827002,48.036052980849,0
-0.78706198164984,48.036104236897,0
-0.78694879354429,48.036162310153,0
-0.78684360536537,48.036226758668,0
-0.78674721765265,48.036297091972,0
-0.7866603639808,48.036372774809,0
-0.78658370537655,48.03645323121,0
-0.78651782528727,48.036537848873,0
-0.78646322513975,48.036625983827,0
-0.78642032052278,48.036716965326,0
-0.78638943802283,48.03681010096,0
-0.78637081273681,48.036904681921,0
-0.78636458648095,48.036999988395,0
-0.78800000000001,48.037,0
</coordinates>
</LinearRing>
</outerBoundaryIs>
</Polygon>
</Placemark>
<Placemark>
<name>139</name>
<styleUrl>#stylehap2</styleUrl>
<Polygon>
<outerBoundaryIs>
<LinearRing>
<coordinates>
-0.78700000000003,48.289,0
-0.78535653228362,48.288999988292,0
-0.78536278311898,48.289095294944,0
-0.78538149423296,48.289189876431,0
-0.78541252329018,48.289283012924,0
-0.785455634204,48.289373995588,0
-0.78551049893164,48.289462131978,0
-0.78557669996875,48.289546751306,0
-0.78565373352556,48.289627209548,0
-0.78574101335971,48.289702894348,0
-0.785837875237,48.289773229676,0
-0.78594358198621,48.289837680215,0
-0.78605732910902,48.289895755434,0
-0.78617825090307,48.289947013323,0
-0.78630542705101,48.289991063757,0
-0.78643788962548,48.290027571468,0
-0.78657463045689,48.290056258593,0
-0.78671460880774,48.290076906792,0
-0.78685675929498,48.29008935891,0
-0.78700000000002,48.290093520174,0
-0.78714324070509,48.29008935891,0
-0.78728539119233,48.290076906792,0
-0.78742536954318,48.290056258593,0

-0.78756211037459,48.290027571468,0
-0.78769457294906,48.289991063757,0
-0.787821749097,48.289947013323,0
-0.78794267089105,48.289895755434,0
-0.78805641801386,48.289837680215,0
-0.78816212476307,48.289773229676,0
-0.78825898664036,48.289702894348,0
-0.78834626647451,48.289627209548,0
-0.78842330003132,48.289546751306,0
-0.78848950106843,48.289462131978,0
-0.78854436579607,48.289373995588,0
-0.78858747670989,48.289283012924,0
-0.78861850576711,48.289189876431,0
-0.78863721688109,48.289095294944,0
-0.78864346771645,48.288999988292,0
-0.78863721077017,48.288904681818,0
-0.78861849373095,48.28881010086,0
-0.78858745911425,48.28871696523,0
-0.78854434317551,48.288625983735,0
-0.78848947411031,48.288537848789,0
-0.78842326955473,48.288453231133,0
-0.78834623340546,48.28837277474,0
-0.78825895198369,48.288297091911,0
-0.78816208957173,48.288226758616,0
-0.78805638335717,48.28816231011,0
-0.78794263782203,48.288104236863,0
-0.78782171862041,48.288052980823,0
-0.78769454599093,48.28800893206,0
-0.78756208775405,48.287972425793,0
-0.78742535194751,48.287943739839,0
-0.78728537915617,48.287923092502,0
-0.78714323459418,48.287910640912,0
-0.78700000000002,48.287906479826,0
-0.78685676540589,48.287910640912,0
-0.7867146208439,48.287923092502,0
-0.78657464805256,48.287943739839,0
-0.78643791224602,48.287972425793,0
-0.78630545400914,48.28800893206,0
-0.78617828137966,48.288052980823,0
-0.78605736217804,48.288104236863,0
-0.78594361664288,48.28816231011,0
-0.78583791042834,48.288226758616,0
-0.78574104801638,48.288297091911,0
-0.78565376659461,48.28837277474,0
-0.78557673044534,48.288453231133,0
-0.78551052588976,48.288537848789,0
-0.78545565682456,48.288625983735,0
-0.78541254088582,48.28871696523,0
-0.78538150626912,48.28881010086,0
-0.7853627892299,48.288904681818,0
-0.78535653228362,48.288999988292,0
-0.78700000000003,48.289,0
</coordinates>
</LinearRing>
</outerBoundaryIs>
</Polygon>
</Placemark>

<Placemark>
<name>140</name>
<styleUrl>#stylehap2</styleUrl>
<Polygon>
<outerBoundaryIs>
<LinearRing>
<coordinates>
-0.78600000000006,48.289,0
-0.78435653228364,48.288999988292,0
-0.78436278311902,48.289095294944,0
-0.784381494233,48.289189876431,0
-0.78441252329019,48.289283012924,0
-0.78445563420404,48.289373995588,0
-0.78451049893168,48.289462131978,0
-0.78457669996879,48.289546751306,0
-0.78465373352558,48.289627209548,0
-0.78474101335972,48.289702894348,0
-0.78483787523704,48.289773229676,0
-0.78494358198622,48.289837680215,0
-0.78505732910904,48.289895755434,0
-0.78517825090311,48.289947013323,0
-0.78530542705103,48.289991063757,0
-0.78543788962549,48.290027571468,0
-0.78557463045691,48.290056258593,0
-0.78571460880778,48.290076906792,0
-0.78585675929499,48.29008935891,0
-0.78600000000006,48.290093520174,0
-0.78614324070513,48.29008935891,0
-0.78628539119234,48.290076906792,0
-0.78642536954319,48.290056258593,0
-0.78656211037461,48.290027571468,0
-0.78669457294907,48.289991063757,0
-0.78682174909701,48.289947013323,0
-0.78694267089109,48.289895755434,0
-0.78705641801388,48.289837680215,0
-0.78716212476309,48.289773229676,0
-0.7872589866404,48.289702894348,0
-0.78734626647452,48.289627209548,0
-0.78742330003133,48.289546751306,0
-0.78748950106845,48.289462131978,0
-0.78754436579609,48.289373995588,0
-0.78758747670993,48.289283012924,0
-0.78761850576712,48.289189876431,0
-0.7876372168811,48.289095294944,0
-0.78764346771649,48.288999988292,0
-0.78763721077019,48.288904681818,0
-0.78761849373099,48.28881010086,0
-0.78758745911427,48.28871696523,0
-0.78754434317553,48.288625983735,0
-0.78748947411032,48.288537848789,0
-0.78742326955475,48.288453231133,0
-0.78734623340551,48.28837277474,0
-0.7872589519837,48.288297091911,0
-0.78716208957177,48.288226758616,0
-0.78705638335721,48.28816231011,0
-0.78694263782205,48.288104236863,0
-0.78682171862043,48.288052980823,0

-0.78669454599095,48.28800893206,0
-0.78656208775407,48.287972425793,0
-0.78642535194755,48.287943739839,0
-0.78628537915621,48.287923092502,0
-0.78614323459422,48.287910640912,0
-0.78600000000006,48.287906479826,0
-0.78585676540591,48.287910640912,0
-0.78571462084392,48.287923092502,0
-0.78557464805258,48.287943739839,0
-0.78543791224606,48.287972425793,0
-0.78530545400916,48.28800893206,0
-0.7851782813797,48.288052980823,0
-0.78505736217805,48.288104236863,0
-0.78494361664292,48.28816231011,0
-0.78483791042835,48.288226758616,0
-0.78474104801642,48.288297091911,0
-0.78465376659462,48.28837277474,0
-0.78457673044538,48.288453231133,0
-0.78451052588978,48.288537848789,0
-0.78445565682458,48.288625983735,0
-0.78441254088586,48.28871696523,0
-0.78438150626914,48.28881010086,0
-0.78436278922991,48.288904681818,0
-0.78435653228364,48.288999988292,0
-0.78600000000006,48.289,0
</coordinates>
</LinearRing>
</outerBoundaryIs>
</Polygon>
</Placemark>
<Placemark>
<name>145</name>
<styleUrl>#stylehap2</styleUrl>
<Polygon>
<outerBoundaryIs>
<LinearRing>
<coordinates>
-0.78200000000004,48.393,0
-0.78035317584167,48.392999988249,0
-0.78035943943187,48.393095294902,0
-0.78037818874854,48.393189876389,0
-0.78040928116596,48.393283012884,0
-0.78045248011568,48.39337399555,0
-0.78050745688518,48.393462131943,0
-0.78057379311772,48.393546751274,0
-0.78065098399484,48.393627209519,0
-0.78073844207702,48.393702894323,0
-0.78083550177364,48.393773229655,0
-0.78094142440793,48.393837680197,0
-0.78105540383857,48.39389575542,0
-0.78117657259508,48.393947013312,0
-0.78130400848037,48.39399106375,0
-0.78143674159012,48.394027571463,0
-0.78157376169553,48.39405625859,0
-0.78171402593336,48.394076906791,0
-0.78185646674433,48.39408935891,0
-0.78200000000005,48.394093520173,0

-0.78214353325574,48.39408935891,0
-0.78228597406673,48.394076906791,0
-0.78242623830454,48.39405625859,0
-0.78256325840995,48.394027571463,0
-0.7826959915197,48.39399106375,0
-0.78282342740499,48.393947013312,0
-0.78294459616151,48.39389575542,0
-0.78305857559214,48.393837680197,0
-0.78316449822643,48.393773229655,0
-0.78326155792305,48.393702894323,0
-0.78334901600523,48.393627209519,0
-0.78342620688235,48.393546751274,0
-0.78349254311489,48.393462131943,0
-0.78354751988439,48.39337399555,0
-0.78359071883413,48.393283012884,0
-0.78362181125153,48.393189876389,0
-0.7836405605682,48.393095294902,0
-0.7836468241584,48.392999988249,0
-0.7836405544224,48.392904681776,0
-0.78362179914665,48.392810100818,0
-0.78359070113796,48.39271696519,0
-0.78354749713465,48.392625983698,0
-0.7834925160028,48.392537848753,0
-0.7834261762317,48.3924532311,0
-0.78334898274734,48.392372774711,0
-0.78326152306842,48.392297091886,0
-0.78316446283411,48.392226758595,0
-0.78305854073751,48.392162310093,0
-0.78294456290362,48.392104236849,0
-0.78282339675434,48.392052980813,0
-0.78269596440761,48.392008932053,0
-0.78256323566021,48.391972425788,0
-0.78242622060836,48.391943739836,0
-0.78228596196185,48.391923092501,0
-0.78214352710995,48.391910640912,0
-0.78200000000005,48.391906479826,0
-0.78185647289015,48.391910640912,0
-0.78171403803825,48.391923092501,0
-0.78157377939171,48.391943739836,0
-0.78143676433986,48.391972425788,0
-0.78130403559246,48.392008932053,0
-0.78117660324573,48.392052980813,0
-0.78105543709648,48.392104236849,0
-0.78094145926256,48.392162310093,0
-0.78083553716596,48.392226758595,0
-0.78073847693165,48.392297091886,0
-0.78065101725276,48.392372774711,0
-0.7805738237684,48.3924532311,0
-0.78050748399727,48.392537848753,0
-0.78045250286542,48.392625983698,0
-0.78040929886211,48.39271696519,0
-0.78037820085342,48.392810100818,0
-0.78035944557767,48.392904681776,0
-0.78035317584167,48.392999988249,0
-0.78200000000004,48.393,0
</coordinates>
</LinearRing>

</outerBoundaryIs>
</Polygon>
</Placemark>
<Placemark>
<name>161</name>
<styleUrl>#stylehap2</styleUrl>
<Polygon>
<outerBoundaryIs>
<LinearRing>
<coordinates>
-0.77099999999996,47.792,0
-0.76937231158298,47.791999988495,0
-0.76937850245503,47.792095295145,0
-0.76939703397019,47.792189876627,0
-0.7694277651581,47.792283013113,0
-0.76947046219751,47.792373995767,0
-0.76952480019382,47.792462132145,0
-0.76959036565019,47.792546751458,0
-0.76966665961287,47.792627209684,0
-0.76975310146747,47.792702894467,0
-0.76984903335691,47.792773229777,0
-0.76995372518751,47.792837680299,0
-0.77006638018527,47.7928957555,0
-0.77018614096007,47.792947013373,0
-0.77031209603137,47.792991063794,0
-0.77044328676601,47.793027571492,0
-0.77057871467522,47.793056258606,0
-0.77071734901513,47.793076906798,0
-0.77085813463315,47.793089358912,0
-0.77099999999996,47.793093520174,0
-0.77114186536678,47.793089358912,0
-0.77128265098477,47.793076906798,0
-0.77142128532471,47.793056258606,0
-0.77155671323391,47.793027571492,0
-0.77168790396856,47.792991063794,0
-0.77181385903985,47.792947013373,0
-0.77193361981465,47.7928957555,0
-0.77204627481242,47.792837680299,0
-0.77215096664299,47.792773229777,0
-0.77224689853243,47.792702894467,0
-0.77233334038705,47.792627209684,0
-0.77240963434974,47.792546751458,0
-0.77247519980608,47.792462132145,0
-0.77252953780241,47.792373995767,0
-0.77257223484182,47.792283013113,0
-0.77260296602973,47.792189876627,0
-0.77262149754489,47.792095295145,0
-0.77262768841695,47.791999988495,0
-0.77262149159733,47.791904682019,0
-0.77260295431532,47.791810101056,0
-0.77257221771649,47.791716965418,0
-0.77252951578654,47.791625983914,0
-0.77247517356859,47.791537848955,0
-0.77240960468781,47.791453231284,0
-0.77233330820197,47.791372774876,0
-0.77224686480213,47.79129709203,0
-0.77215093239236,47.791226758717,0

-0.77204624108213,47.791162310194,0
-0.77193358762958,47.791104236929,0
-0.77181382937792,47.791052980874,0
-0.77168787773104,47.791008932097,0
-0.77155669121804,47.790972425816,0
-0.77142126819938,47.790943739852,0
-0.77128263927038,47.790923092508,0
-0.77114185941922,47.790910640914,0
-0.77099999999996,47.790906479827,0
-0.77085814058068,47.790910640914,0
-0.77071736072954,47.790923092508,0
-0.77057873180052,47.790943739852,0
-0.77044330878189,47.790972425816,0
-0.77031212226886,47.791008932097,0
-0.770186170622,47.791052980874,0
-0.77006641237035,47.791104236929,0
-0.7699537589178,47.791162310194,0
-0.76984906760757,47.791226758717,0
-0.76975313519776,47.79129709203,0
-0.76966669179795,47.791372774876,0
-0.76959039531212,47.791453231284,0
-0.76952482643134,47.791537848955,0
-0.76947048421339,47.791625983914,0
-0.76942778228343,47.791716965418,0
-0.76939704568458,47.791810101056,0
-0.76937850840259,47.791904682019,0
-0.76937231158298,47.791999988495,0
-0.77099999999996,47.792,0
</coordinates>
</LinearRing>
</outerBoundaryIs>
</Polygon>
</Placemark>
<Placemark>
<name>164</name>
<styleUrl>#stylehap2</styleUrl>
<Polygon>
<outerBoundaryIs>
<LinearRing>
<coordinates>
-0.76800000000003,48.306,0
-0.76635598494035,48.305999988285,0
-0.76636223785569,48.306095294937,0
-0.76638095519949,48.306189876424,0
-0.76641199458897,48.306283012917,0
-0.76645511985906,48.306373995582,0
-0.76651000285763,48.306462131972,0
-0.76657622594141,48.306546751301,0
-0.76665328515283,48.306627209543,0
-0.7667405940543,48.306702894344,0
-0.7668374881905,48.306773229673,0
-0.76694323014461,48.306837680212,0
-0.76705701515043,48.306895755431,0
-0.76717797721727,48.306947013321,0
-0.76730519572122,48.306991063756,0
-0.76743770241252,48.307027571467,0
-0.76757448878584,48.307056258592,0

-0.76771451375698,48.307076906792,0
-0.76785671158802,48.30708935891,0
-0.76800000000003,48.307093520173,0
-0.76814328841205,48.30708935891,0
-0.76828548624306,48.307076906792,0
-0.76842551121423,48.307056258592,0
-0.76856229758752,48.307027571467,0
-0.76869480427885,48.306991063756,0
-0.7688220227828,48.306947013321,0
-0.76894298484964,48.306895755431,0
-0.76905676985546,48.306837680212,0
-0.76916251180956,48.306773229673,0
-0.76925940594574,48.306702894344,0
-0.76934671484723,48.306627209543,0
-0.76942377405865,48.306546751301,0
-0.76948999714241,48.306462131972,0
-0.76954488014099,48.306373995582,0
-0.76958800541107,48.306283012917,0
-0.76961904480058,48.306189876424,0
-0.76963776214435,48.306095294937,0
-0.76964401505969,48.305999988285,0
-0.76963775602776,48.305904681811,0
-0.76961903275322,48.305810100853,0
-0.76958798779904,48.305716965223,0
-0.76954485749941,48.305625983729,0
-0.76948997015919,48.305537848783,0
-0.7694237435537,48.305453231127,0
-0.76934668174743,48.305372774735,0
-0.76925937125681,48.305297091907,0
-0.76916247658548,48.305226758613,0
-0.7690567351665,48.305162310108,0
-0.76894295174981,48.30510423686,0
-0.76882199227784,48.305052980822,0
-0.76869477729564,48.305008932059,0
-0.76856227494594,48.304972425792,0
-0.76842549360217,48.304943739838,0
-0.76828547419573,48.304923092502,0
-0.76814328229544,48.304910640912,0
-0.76800000000003,48.304906479826,0
-0.7678567177046,48.304910640912,0
-0.76771452580434,48.304923092502,0
-0.76757450639787,48.304943739838,0
-0.76743772505413,48.304972425792,0
-0.76730522270443,48.305008932059,0
-0.76717800772222,48.305052980822,0
-0.76705704825023,48.30510423686,0
-0.76694326483354,48.305162310108,0
-0.76683752341459,48.305226758613,0
-0.76674062874326,48.305297091907,0
-0.76665331825264,48.305372774735,0
-0.76657625644637,48.305453231127,0
-0.76651002984085,48.305537848783,0
-0.76645514250066,48.305625983729,0
-0.76641201220103,48.305716965223,0
-0.76638096724684,48.305810100853,0
-0.7663622439723,48.305904681811,0
-0.76635598494035,48.305999988285,0

-0.76800000000003,48.306,0
</coordinates>
</LinearRing>
</outerBoundaryIs>
</Polygon>
</Placemark>
<Placemark>
<name>166</name>
<styleUrl>#stylehap2</styleUrl>
<Polygon>
<outerBoundaryIs>
<LinearRing>
<coordinates>
-0.76599999999996,48.181,0
-0.76435999766165,48.180999988337,0
-0.76436623532819,48.181095294988,0
-0.76438490699955,48.181189876474,0
-0.76441587064008,48.181283012965,0
-0.76445889066075,48.181373995627,0
-0.76451363971009,48.181462132015,0
-0.76457970116388,48.181546751339,0
-0.7646565722944,48.181627209578,0
-0.76474366809529,48.181702894374,0
-0.76484032573288,48.181773229698,0
-0.76494580959026,48.181837680233,0
-0.76505931686552,48.181895755448,0
-0.76517998368177,48.181947013334,0
-0.76530689166236,48.181991063765,0
-0.7654390749211,48.182027571473,0
-0.76557552741447,48.182056258596,0
-0.7657152105997,48.182076906793,0
-0.76585706134049,48.18208935891,0
-0.76599999999996,48.182093520173,0
-0.76614293865943,48.18208935891,0
-0.76628478940023,48.182076906793,0
-0.76642447258545,48.182056258596,0
-0.76656092507882,48.182027571473,0
-0.76669310833756,48.181991063765,0
-0.76682001631815,48.181947013334,0
-0.76694068313441,48.181895755448,0
-0.76705419040966,48.181837680233,0
-0.76715967426705,48.181773229698,0
-0.76725633190464,48.181702894374,0
-0.76734342770553,48.181627209578,0
-0.76742029883604,48.181546751339,0
-0.76748636028984,48.181462132015,0
-0.76754110933917,48.181373995627,0
-0.76758412935984,48.181283012965,0
-0.76761509300037,48.181189876474,0
-0.76763376467174,48.181095294988,0
-0.76764000233828,48.180999988337,0
-0.7676337585968,48.180904681862,0
-0.7676150810351,48.180810100903,0
-0.76758411186776,48.180716965271,0
-0.76754108685181,48.180625983775,0
-0.76748633349043,48.180537848825,0
-0.76742026853889,48.180453231166,0

-0.76734339483119,48.18037277477,0
-0.76725629745201,48.180297091937,0
-0.76715963928291,48.180226758638,0
-0.76705415595703,48.180162310129,0
-0.76694065026007,48.180104236877,0
-0.766819986021,48.180052980834,0
-0.76669308153816,48.180008932068,0
-0.76656090259146,48.179972425798,0
-0.76642445509339,48.179943739842,0
-0.76628477743496,48.179923092503,0
-0.7661429325845,48.179910640912,0
-0.76599999999996,48.179906479826,0
-0.76585706741543,48.179910640912,0
-0.76571522256499,48.179923092503,0
-0.76557554490653,48.179943739842,0
-0.76543909740849,48.179972425798,0
-0.76530691846177,48.180008932068,0
-0.76518001397892,48.180052980834,0
-0.76505934973986,48.180104236877,0
-0.76494584404289,48.180162310129,0
-0.76484036071702,48.180226758638,0
-0.76474370254794,48.180297091937,0
-0.76465660516874,48.18037277477,0
-0.76457973146103,48.180453231166,0
-0.76451366650949,48.180537848825,0
-0.76445891314811,48.180625983775,0
-0.76441588813217,48.180716965271,0
-0.76438491896482,48.180810100903,0
-0.76436624140312,48.180904681862,0
-0.76435999766165,48.180999988337,0
-0.76599999999996,48.181,0
</coordinates>
</LinearRing>
</outerBoundaryIs>
</Polygon>
</Placemark>
<Placemark>
<name>169</name>
<styleUrl>#stylehap2</styleUrl>
<Polygon>
<outerBoundaryIs>
<LinearRing>
<coordinates>
-0.76400000000001,48.308,0
-0.76235592051352,48.307999988284,0
-0.76236217367368,48.308095294936,0
-0.76238089175079,48.308189876423,0
-0.7624119323565,48.308283012916,0
-0.76245505931639,48.308373995581,0
-0.76250994446564,48.308462131972,0
-0.76257617014447,48.3085467513,0
-0.76265323237564,48.308627209543,0
-0.76274054469859,48.308702894343,0
-0.76283744263192,48.308773229672,0
-0.76294318872993,48.308837680212,0
-0.76305697819489,48.308895755431,0
-0.76317794500218,48.308947013321,0

-0.76330516849176,48.308991063756,0
-0.76343768037599,48.309027571467,0
-0.76357447210997,48.309056258592,0
-0.76371450256871,48.309076906792,0
-0.76385670597249,48.30908935891,0
-0.76400000000002,48.309093520174,0
-0.76414329402752,48.30908935891,0
-0.76428549743132,48.309076906792,0
-0.76442552789004,48.309056258592,0
-0.76456231962402,48.309027571467,0
-0.76469483150825,48.308991063756,0
-0.76482205499786,48.308947013321,0
-0.76494302180513,48.308895755431,0
-0.76505681127008,48.308837680212,0
-0.76516255736809,48.308773229672,0
-0.76525945530142,48.308702894343,0
-0.76534676762438,48.308627209543,0
-0.76542382985554,48.3085467513,0
-0.7654900555344,48.308462131972,0
-0.76554494068362,48.308373995581,0
-0.76558806764354,48.308283012916,0
-0.76561910824925,48.308189876423,0
-0.76563782632633,48.308095294936,0
-0.76564407948649,48.307999988284,0
-0.76563782020905,48.307904681811,0
-0.76561909620057,48.307810100852,0
-0.76558805002958,48.307716965222,0
-0.76554491803954,48.307625983729,0
-0.76549002854823,48.307537848782,0
-0.76542379934725,48.307453231127,0
-0.76534673452093,48.307372774735,0
-0.76525942060867,48.307297091907,0
-0.76516252214017,48.307226758612,0
-0.76505677657733,48.307162310107,0
-0.76494298870168,48.30710423686,0
-0.76482202448957,48.307052980821,0
-0.76469480452208,48.307008932059,0
-0.76456229697994,48.306972425792,0
-0.76442551027608,48.306943739838,0
-0.76428548538264,48.306923092502,0
-0.76414328791027,48.306910640912,0
-0.76400000000002,48.306906479827,0
-0.76385671208977,48.306910640912,0
-0.76371451461737,48.306923092502,0
-0.76357448972394,48.306943739838,0
-0.76343770302009,48.306972425792,0
-0.76330519547793,48.307008932059,0
-0.76317797551047,48.307052980821,0
-0.76305701129833,48.30710423686,0
-0.76294322342268,48.307162310107,0
-0.76283747785984,48.307226758612,0
-0.76274057939134,48.307297091907,0
-0.76265326547908,48.307372774735,0
-0.76257620065276,48.307453231127,0
-0.7625099714518,48.307537848782,0
-0.76245508196049,48.307625983729,0
-0.76241194997046,48.307716965222,0

-0.76238090379945,48.307810100852,0
-0.76236217979096,48.307904681811,0
-0.76235592051352,48.307999988284,0
-0.76400000000001,48.308,0
</coordinates>
</LinearRing>
</outerBoundaryIs>
</Polygon>
</Placemark>
<Placemark>
<name>173</name>
<styleUrl>#stylehap2</styleUrl>
<Polygon>
<outerBoundaryIs>
<LinearRing>
<coordinates>
-0.75999999999999,48.224,0
-0.75835862037887,48.223999988319,0
-0.75836486327925,48.224095294971,0
-0.75838355062671,48.224189876457,0
-0.75841454026652,48.224283012949,0
-0.75845759641185,48.224373995612,0
-0.75851239143646,48.224462132,0
-0.75857850836635,48.224546751326,0
-0.75865544405172,48.224627209566,0
-0.75874261299494,48.224702894364,0
-0.75883935180575,48.224773229689,0
-0.75894492424942,48.224837680226,0
-0.75905852684989,48.224895755443,0
-0.75917929500476,48.224947013329,0
-0.75930630956602,48.224991063762,0
-0.75943860383621,48.225027571471,0
-0.7595751709269,48.225056258595,0
-0.75971497142302,48.225076906793,0
-0.75985694129525,48.22508935891,0
-0.76,48.225093520174,0
-0.76014305870473,48.22508935891,0
-0.76028502857696,48.225076906793,0
-0.76042482907308,48.225056258595,0
-0.76056139616377,48.225027571471,0
-0.76069369043397,48.224991063762,0
-0.76082070499523,48.224947013329,0
-0.76094147315009,48.224895755443,0
-0.76105507575056,48.224837680226,0
-0.76116064819423,48.224773229689,0
-0.76125738700505,48.224702894364,0
-0.76134455594826,48.224627209566,0
-0.76142149163363,48.224546751326,0
-0.76148760856352,48.224462132,0
-0.76154240358813,48.224373995612,0
-0.76158545973347,48.224283012949,0
-0.76161644937328,48.224189876457,0
-0.76163513672073,48.224095294971,0
-0.76164137962111,48.223999988319,0
-0.76163513063152,48.223904681845,0
-0.76161643737984,48.223810100886,0
-0.76158544220026,48.223716965254,0

-0.76154238104787,48.223625983759,0
-0.76148758170107,48.223537848811,0
-0.76142146126518,48.223453231153,0
-0.76134452299657,48.223372774758,0
-0.76125735247135,48.223297091927,0
-0.7611606131278,48.22322675863,0
-0.76105504121684,48.223162310121,0
-0.7609414401984,48.223104236872,0
-0.76082067462678,48.22305298083,0
-0.76069366357151,48.223008932065,0
-0.76056137362348,48.222972425796,0
-0.76042481153985,48.22294373984,0
-0.76028501658355,48.222923092503,0
-0.76014305261549,48.222910640912,0
-0.76,48.222906479826,0
-0.75985694738449,48.222910640912,0
-0.75971498341643,48.222923092503,0
-0.75957518846013,48.22294373984,0
-0.7594386263765,48.222972425796,0
-0.75930633642847,48.223008932065,0
-0.7591793253732,48.22305298083,0
-0.75905855980159,48.223104236872,0
-0.75894495878315,48.223162310121,0
-0.75883938687218,48.22322675863,0
-0.75874264752864,48.223297091927,0
-0.75865547700341,48.223372774758,0
-0.7585785387348,48.223453231153,0
-0.75851241829892,48.223537848811,0
-0.75845761895211,48.223625983759,0
-0.75841455779972,48.223716965254,0
-0.75838356262015,48.223810100886,0
-0.75836486936846,48.223904681845,0
-0.75835862037887,48.223999988319,0
-0.75999999999999,48.224,0
</coordinates>
</LinearRing>
</outerBoundaryIs>
</Polygon>
</Placemark>
<Placemark>
<name>174</name>
<styleUrl>#stylehap2</styleUrl>
<Polygon>
<outerBoundaryIs>
<LinearRing>
<coordinates>
-0.75900000000001,48.255,0
-0.75735762544395,48.254999988306,0
-0.7573638721252,48.255095294958,0
-0.75738257079693,48.255189876444,0
-0.7574135792183,48.255283012937,0
-0.75745666145972,48.255373995601,0
-0.7575114896964,48.25546213199,0
-0.75757764670169,48.255546751316,0
-0.75765462902081,48.255627209557,0
-0.75774185080131,48.255702894356,0
-0.75783864825079,48.255773229683,0

-0.75794428468831,48.255837680221,0
-0.75805795615075,48.255895755438,0
-0.75817879751157,48.255947013326,0
-0.75830588906558,48.25599106376,0
-0.75843826352935,48.25602757147,0
-0.75857491340395,48.256056258594,0
-0.75871479864421,48.256076906792,0
-0.75885685457584,48.25608935891,0
-0.75900000000002,48.256093520174,0
-0.7591431454242,48.25608935891,0
-0.7592852013558,48.256076906792,0
-0.75942508659609,48.256056258594,0
-0.75956173647069,48.25602757147,0
-0.75969411093445,48.25599106376,0
-0.75982120248847,48.255947013326,0
-0.75994204384929,48.255895755438,0
-0.76005571531173,48.255837680221,0
-0.76016135174925,48.255773229683,0
-0.76025814919873,48.255702894356,0
-0.76034537097923,48.255627209557,0
-0.76042235329832,48.255546751316,0
-0.76048851030361,48.25546213199,0
-0.76054333854031,48.255373995601,0
-0.76058642078172,48.255283012937,0
-0.76061742920311,48.255189876444,0
-0.76063612787484,48.255095294958,0
-0.76064237455609,48.254999988306,0
-0.7606361217753,48.254904681832,0
-0.76061741718934,48.254810100874,0
-0.76058640321877,48.254716965243,0
-0.76054331596179,48.254625983748,0
-0.76048848339558,48.2545378488,0
-0.76042232287838,48.254453231143,0
-0.76034533797164,48.254372774749,0
-0.76025811460646,48.254297091919,0
-0.76016131662331,48.254226758623,0
-0.76005568071943,48.254162310116,0
-0.7599420108417,48.254104236867,0
-0.75982117206853,48.254052980827,0
-0.75969408402643,48.254008932063,0
-0.75956171389219,48.253972425794,0
-0.75942506903312,48.25394373984,0
-0.75928518934203,48.253923092502,0
-0.75914313932465,48.253910640912,0
-0.75900000000002,48.253906479826,0
-0.75885686067538,48.253910640912,0
-0.758714810658,48.253923092502,0
-0.75857493096689,48.25394373984,0
-0.75843828610785,48.253972425794,0
-0.75830591597358,48.254008932063,0
-0.75817882793151,48.254052980827,0
-0.75805798915831,48.254104236867,0
-0.75794431928061,48.254162310116,0
-0.75783868337671,48.254226758623,0
-0.75774188539358,48.254297091919,0
-0.75765466202838,48.254372774749,0
-0.75757767712163,48.254453231143,0

-0.75751151660443,48.2545378488,0
-0.75745668403823,48.254625983748,0
-0.75741359678127,48.254716965243,0
-0.7573825828107,48.254810100874,0
-0.75736387822474,48.254904681832,0
-0.75735762544395,48.254999988306,0
-0.75900000000001,48.255,0
</coordinates>
</LinearRing>
</outerBoundaryIs>
</Polygon>
</Placemark>
<Placemark>
<name>175</name>
<styleUrl>#stylehap2</styleUrl>
<Polygon>
<outerBoundaryIs>
<LinearRing>
<coordinates>
-0.75700000000006,48.185,0
-0.75535986967889,48.184999988335,0
-0.75536610783177,48.185095294987,0
-0.75538478095983,48.185189876472,0
-0.75541574701633,48.185283012963,0
-0.75545877039384,48.185373995626,0
-0.7555135237154,48.185462132013,0
-0.75557959032426,48.185546751338,0
-0.75565646745348,48.185627209577,0
-0.75574357005106,48.185702894373,0
-0.7558402352316,48.185773229697,0
-0.7559457273208,48.185837680233,0
-0.75605924345408,48.185895755448,0
-0.75617991968714,48.185947013333,0
-0.75630683757165,48.185991063765,0
-0.75643903114605,48.186027571473,0
-0.75657549428824,48.186056258596,0
-0.7567151883745,48.186076906793,0
-0.75685705018548,48.186089358911,0
-0.75700000000005,48.186093520174,0
-0.75714294981464,48.186089358911,0
-0.75728481162562,48.186076906793,0
-0.75742450571188,48.186056258596,0
-0.75756096885408,48.186027571473,0
-0.75769316242848,48.185991063765,0
-0.75782008031298,48.185947013333,0
-0.75794075654605,48.185895755448,0
-0.75805427267933,48.185837680233,0
-0.75815976476852,48.185773229697,0
-0.75825642994906,48.185702894373,0
-0.75834353254665,48.185627209577,0
-0.75842040967587,48.185546751338,0
-0.75848647628473,48.185462132013,0
-0.75854122960629,48.185373995626,0
-0.75858425298379,48.185283012963,0
-0.7586152190403,48.185189876472,0
-0.75863389216835,48.185095294987,0
-0.75864013032124,48.184999988335,0

-0.75863388609209,48.184904681861,0
-0.7586152070724,48.184810100901,0
-0.75858423548791,48.184716965269,0
-0.75854120711399,48.184625983773,0
-0.75848644947947,48.184537848824,0
-0.7584203793721,48.184453231165,0
-0.75834349966513,48.184372774769,0
-0.75825639548887,48.184297091936,0
-0.75815972977672,48.184226758638,0
-0.75805423821916,48.184162310128,0
-0.75794072366453,48.184104236877,0
-0.75782005000922,48.184052980834,0
-0.75769313562322,48.184008932068,0
-0.75756094636181,48.183972425798,0
-0.75742448821598,48.183943739841,0
-0.75728479965773,48.183923092503,0
-0.75714294373838,48.183910640912,0
-0.75700000000005,48.183906479826,0
-0.75685705626174,48.183910640912,0
-0.75671520034239,48.183923092503,0
-0.75657551178415,48.183943739841,0
-0.75643905363832,48.183972425798,0
-0.75630686437691,48.184008932068,0
-0.75617994999091,48.184052980834,0
-0.75605927633559,48.184104236877,0
-0.75594576178096,48.184162310128,0
-0.7558402702234,48.184226758638,0
-0.75574360451125,48.184297091936,0
-0.75565650033499,48.184372774769,0
-0.75557962062802,48.184453231165,0
-0.75551355052065,48.184537848824,0
-0.75545879288613,48.184625983773,0
-0.75541576451221,48.184716965269,0
-0.75538479292772,48.184810100901,0
-0.75536611390803,48.184904681861,0
-0.75535986967889,48.184999988335,0
-0.75700000000006,48.185,0
</coordinates>
</LinearRing>
</outerBoundaryIs>
</Polygon>
</Placemark>
<Placemark>
<name>183</name>
<styleUrl>#stylehap2</styleUrl>
<Polygon>
<outerBoundaryIs>
<LinearRing>
<coordinates>
-0.74599999999998,47.996,0
-0.74436588644175,47.995999988412,0
-0.74437210173021,47.996095295063,0
-0.74439070637594,47.996189876547,0
-0.74442155885272,47.996283013035,0
-0.74446442441688,47.996373995694,0
-0.74451897689193,47.996462132077,0
-0.74458480114917,47.996546751396,0

-0.74466139626573,47.996627209629,0
-0.74474817933555,47.996702894418,0
-0.74484448990491,47.996773229736,0
-0.74494959499815,47.996837680264,0
-0.74506269469609,47.996895755473,0
-0.74518292822398,47.996947013353,0
-0.74530938050297,47.996991063779,0
-0.74544108911545,47.997027571482,0
-0.74557705163075,47.997056258601,0
-0.74571623323573,47.997076906796,0
-0.74585757461202,47.997089358911,0
-0.74599999999999,47.997093520174,0
-0.74614242538796,47.997089358911,0
-0.74628376676422,47.997076906796,0
-0.7464229483692,47.997056258601,0
-0.74655891088451,47.997027571482,0
-0.74669061949701,47.996991063779,0
-0.746817071776,47.996947013353,0
-0.74693730530387,47.996895755473,0
-0.74705040500183,47.996837680264,0
-0.74715551009507,47.996773229736,0
-0.7472518206644,47.996702894418,0
-0.74733860373423,47.996627209629,0
-0.74741519885078,47.996546751396,0
-0.74748102310805,47.996462132077,0
-0.74753557558308,47.996373995694,0
-0.74757844114725,47.996283013035,0
-0.74760929362401,47.996189876547,0
-0.74762789826974,47.996095295063,0
-0.74763411355821,47.995999988412,0
-0.74762789225582,47.995904681937,0
-0.74760928177889,47.995810100976,0
-0.74757842383084,47.995716965341,0
-0.74753555332153,47.995625983841,0
-0.74748099657774,47.995537848887,0
-0.74741516885787,47.995453231222,0
-0.74733857119003,47.99537277482,0
-0.74725178655771,47.995297091982,0
-0.74715547546224,47.995226758676,0
-0.74705037089514,47.99516231016,0
-0.74693727275964,47.995104236902,0
-0.74681704178309,47.995052980853,0
-0.74669059296671,47.995008932082,0
-0.74655888862297,47.994972425807,0
-0.74642293105278,47.994943739847,0
-0.74628375491909,47.994923092506,0
-0.74614241937401,47.994910640913,0
-0.74599999999999,47.994906479827,0
-0.74585758062594,47.994910640913,0
-0.74571624508086,47.994923092506,0
-0.74557706894717,47.994943739847,0
-0.74544111137701,47.994972425807,0
-0.74530940703325,47.995008932082,0
-0.74518295821689,47.995052980853,0
-0.74506272724031,47.995104236902,0
-0.74494962910482,47.99516231016,0
-0.74484452453774,47.995226758676,0

-0.74474821344224,47.995297091982,0
-0.74466142880995,47.99537277482,0
-0.74458483114208,47.995453231222,0
-0.74451900342221,47.995537848887,0
-0.74446444667845,47.995625983841,0
-0.74442157616914,47.995716965341,0
-0.74439071822106,47.995810100976,0
-0.74437210774415,47.995904681937,0
-0.74436588644175,47.995999988412,0
-0.74599999999998,47.996,0
</coordinates>
</LinearRing>
</outerBoundaryIs>
</Polygon>
</Placemark>
<Placemark>
<name>186</name>
<styleUrl>#stylehap2</styleUrl>
<Polygon>
<outerBoundaryIs>
<LinearRing>
<coordinates>
-0.74400000000003,48.309,0
-0.7423558882975,48.308999988284,0
-0.74236214158008,48.309095294936,0
-0.74238086002387,48.309189876423,0
-0.7424119012377,48.309283012916,0
-0.74245502904263,48.309373995581,0
-0.74250991526726,48.309462131971,0
-0.74257614224373,48.3095467513,0
-0.74265320598492,48.309627209543,0
-0.74274052001873,48.309702894343,0
-0.74283741985078,48.309773229672,0
-0.74294316802093,48.309837680212,0
-0.74305695971563,48.309895755431,0
-0.74317792889333,48.309947013321,0
-0.74330515487596,48.309991063756,0
-0.74343766935685,48.310027571467,0
-0.74357446377137,48.310056258592,0
-0.74371449697415,48.310076906792,0
-0.74385670316452,48.31008935891,0
-0.74400000000002,48.310093520174,0
-0.74414329683552,48.31008935891,0
-0.74428550302592,48.310076906792,0
-0.74442553622867,48.310056258592,0
-0.74456233064319,48.310027571467,0
-0.74469484512411,48.309991063756,0
-0.74482207110673,48.309947013321,0
-0.7449430402844,48.309895755431,0
-0.74505683197913,48.309837680212,0
-0.74516258014926,48.309773229672,0
-0.74525947998133,48.309702894343,0
-0.74534679401514,48.309627209543,0
-0.74542385775631,48.3095467513,0
-0.74549008473281,48.309462131971,0
-0.74554497095744,48.309373995581,0
-0.74558809876234,48.309283012916,0

-0.7456191399762,48.309189876423,0
-0.74563785841998,48.309095294936,0
-0.74564411170256,48.308999988284,0
-0.74563785230238,48.30890468181,0
-0.74561912792688,48.308810100852,0
-0.74558808114741,48.308716965222,0
-0.74554494831212,48.308625983728,0
-0.74549005774517,48.308537848782,0
-0.74542382724637,48.308453231126,0
-0.7453467609099,48.308372774734,0
-0.74525944528667,48.308297091906,0
-0.7451625449194,48.308226758612,0
-0.7450567972845,48.308162310107,0
-0.74494300717918,48.30810423686,0
-0.74482204059677,48.308052980821,0
-0.74469481813646,48.308008932059,0
-0.74456230799787,48.307972425792,0
-0.74442551861374,48.307943739838,0
-0.7442854909766,48.307923092502,0
-0.74414329071791,48.307910640912,0
-0.74400000000002,48.307906479826,0
-0.74385670928213,48.307910640912,0
-0.74371450902346,48.307923092502,0
-0.7435744813863,48.307943739838,0
-0.74343769200217,48.307972425792,0
-0.7433051818636,48.308008932059,0
-0.7431779594033,48.308052980821,0
-0.74305699282088,48.30810423686,0
-0.74294320271557,48.308162310107,0
-0.74283745508064,48.308226758612,0
-0.74274055471337,48.308297091906,0
-0.74265323909017,48.308372774734,0
-0.7425761727537,48.308453231126,0
-0.7425099422549,48.308537848782,0
-0.74245505168795,48.308625983728,0
-0.74241191885263,48.308716965222,0
-0.74238087207318,48.308810100852,0
-0.74236214769769,48.30890468181,0
-0.7423558882975,48.308999988284,0
-0.74400000000003,48.309,0
</coordinates>
</LinearRing>
</outerBoundaryIs>
</Polygon>
</Placemark>
<Placemark>
<name>191</name>
<styleUrl>#stylehap2</styleUrl>
<Polygon>
<outerBoundaryIs>
<LinearRing>
<coordinates>
-0.74000000000001,48.199,0
-0.73835942151848,48.198999988329,0
-0.73836566137447,48.199095294981,0
-0.73838433960342,48.199189876467,0
-0.73841531411995,48.199283012958,0

-0.73845834925222,48.199373995621,0
-0.73851311753389,48.199462132009,0
-0.73857920219438,48.199546751334,0
-0.73865610032938,48.199627209573,0
-0.73874322672708,48.19970289437,0
-0.73883991832096,48.199773229695,0
-0.73894543923565,48.19983768023,0
-0.73905898638723,48.199895755446,0
-0.73917969559531,48.199947013332,0
-0.73930664816059,48.199991063764,0
-0.73943887785752,48.200027571472,0
-0.73957537828907,48.200056258595,0
-0.73971511054766,48.200076906793,0
-0.73985701112337,48.20008935891,0
-0.74,48.200093520173,0
-0.74014298887667,48.20008935891,0
-0.74028488945238,48.200076906793,0
-0.74042462171094,48.200056258595,0
-0.74056112214249,48.200027571472,0
-0.74069335183942,48.199991063764,0
-0.7408203044047,48.199947013332,0
-0.74094101361278,48.199895755446,0
-0.74105456076436,48.19983768023,0
-0.74116008167905,48.199773229695,0
-0.74125677327293,48.19970289437,0
-0.74134389967063,48.199627209573,0
-0.74142079780565,48.199546751334,0
-0.74148688246612,48.199462132009,0
-0.74154165074779,48.199373995621,0
-0.74158468588009,48.199283012958,0
-0.74161566039659,48.199189876467,0
-0.74163433862557,48.199095294981,0
-0.74164057848153,48.198999988329,0
-0.74163433254465,48.198904681855,0
-0.74161564841953,48.198810100896,0
-0.7415846683708,48.198716965264,0
-0.74154162823829,48.198625983768,0
-0.74148685564035,48.198537848819,0
-0.74142076747868,48.19845323116,0
-0.74134386676394,48.198372774765,0
-0.74125673878638,48.198297091933,0
-0.7411600466605,48.198226758635,0
-0.74105452627783,48.198162310126,0
-0.7409409807061,48.198104236875,0
-0.74082027407773,48.198052980833,0
-0.74069332501365,48.198008932067,0
-0.740561099633,48.197972425797,0
-0.74042460420165,48.197943739841,0
-0.74028487747533,48.197923092503,0
-0.74014298279575,48.197910640912,0
-0.74,48.197906479826,0
-0.73985701720426,48.197910640912,0
-0.73971512252471,48.197923092503,0
-0.73957539579836,48.197943739841,0
-0.73943890036701,48.197972425797,0
-0.73930667498638,48.198008932067,0
-0.73917972592228,48.198052980833,0

-0.73905901929391,48.198104236875,0
-0.7389454737222,48.198162310126,0
-0.73883995333953,48.198226758635,0
-0.73874326121363,48.198297091933,0
-0.73865613323609,48.198372774765,0
-0.73857923252133,48.19845323116,0
-0.73851314435965,48.198537848819,0
-0.73845837176172,48.198625983768,0
-0.73841533162923,48.198716965264,0
-0.73838435158048,48.198810100896,0
-0.73836566745536,48.198904681855,0
-0.73835942151848,48.198999988329,0
-0.74000000000001,48.199,0
</coordinates>
</LinearRing>
</outerBoundaryIs>
</Polygon>
</Placemark>
<Placemark>
<name>201</name>
<styleUrl>#stylehap2</styleUrl>
<Polygon>
<outerBoundaryIs>
<LinearRing>
<coordinates>
-0.72500000000002,48,0
-0.72336575974416,47.999999988411,0
-0.72337197551409,48.000095295061,0
-0.72339058160188,48.000189876545,0
-0.72342143647034,48.000283013034,0
-0.72346430535766,48.000373995693,0
-0.72351886206202,48.000462132075,0
-0.72358469142257,48.000546751395,0
-0.7236612924776,48.000627209627,0
-0.72374808227588,48.000702894417,0
-0.72384440031241,48.000773229735,0
-0.7239495135548,48.000837680264,0
-0.7240626220218,48.000895755473,0
-0.72418286487193,48.000947013352,0
-0.72430932695537,48.000991063779,0
-0.72444104577992,48.001027571482,0
-0.72457701883717,48.001056258601,0
-0.72471621123367,48.001076906795,0
-0.72485756356897,48.001089358911,0
-0.72500000000003,48.001093520173,0
-0.72514243643106,48.001089358911,0
-0.72528378876637,48.001076906795,0
-0.7254229811629,48.001056258601,0
-0.72555895422012,48.001027571482,0
-0.72569067304467,48.000991063779,0
-0.72581713512811,48.000947013352,0
-0.72593737797823,48.000895755473,0
-0.72605048644524,48.000837680264,0
-0.72615559968765,48.000773229735,0
-0.72625191772418,48.000702894417,0
-0.72633870752243,48.000627209627,0
-0.72641530857747,48.000546751395,0

-0.72648113793802,48.000462132075,0
-0.72653569464237,48.000373995693,0
-0.7265785635297,48.000283013034,0
-0.72660941839816,48.000189876545,0
-0.72662802448595,48.000095295061,0
-0.7266342402559,47.999999988411,0
-0.72662801847071,47.999904681936,0
-0.72660940655046,47.999810100975,0
-0.72657854620952,47.99971696534,0
-0.72653567237597,47.99962598384,0
-0.72648111140197,47.999537848886,0
-0.72641527857802,47.999453231221,0
-0.72633867497114,47.999372774819,0
-0.72625188361006,47.999297091981,0
-0.72615556504726,47.999226758675,0
-0.72605045233112,47.999162310159,0
-0.72593734542691,47.999104236902,0
-0.72581710512866,47.999052980853,0
-0.72569064650859,47.999008932082,0
-0.72555893195371,47.998972425807,0
-0.72542296384272,47.998943739847,0
-0.72528377691868,47.998923092506,0
-0.72514243041582,47.998910640913,0
-0.72500000000003,47.998906479826,0
-0.72485756958422,47.998910640913,0
-0.72471622308139,47.998923092506,0
-0.72457703615735,47.998943739847,0
-0.72444106804632,47.998972425807,0
-0.72430935349145,47.999008932082,0
-0.72418289487138,47.999052980853,0
-0.72406265457313,47.999104236902,0
-0.72394954766892,47.999162310159,0
-0.72384443495278,47.999226758675,0
-0.72374811638997,47.999297091981,0
-0.7236613250289,47.999372774819,0
-0.72358472142202,47.999453231221,0
-0.7235188885981,47.999537848886,0
-0.72346432762407,47.99962598384,0
-0.72342145379055,47.99971696534,0
-0.72339059344957,47.999810100975,0
-0.72337198152933,47.999904681936,0
-0.72336575974416,47.999999988411,0
-0.72500000000002,48,0
</coordinates>
</LinearRing>
</outerBoundaryIs>
</Polygon>
</Placemark>
<Placemark>
<name>202</name>
<styleUrl>#stylehap2</styleUrl>
<Polygon>
<outerBoundaryIs>
<LinearRing>
<coordinates>
-0.72299999999996,48.227,0
-0.72135852416847,48.226999988318,0

-0.72136476743447,48.227095294969,0
-0.721383455877,48.227189876455,0
-0.72141444733298,48.227283012947,0
-0.72145750600181,48.227373995611,0
-0.72151230423802,48.227462131999,0
-0.72157842504323,48.227546751325,0
-0.72165536523809,48.227627209565,0
-0.72174253929068,48.227702894363,0
-0.72183928377184,48.227773229689,0
-0.72194486240375,48.227837680225,0
-0.72205847166318,48.227895755442,0
-0.72217924689707,48.227947013329,0
-0.72230626890352,48.227991063762,0
-0.72243857092847,48.228027571471,0
-0.72257514602436,48.228056258595,0
-0.72271495471524,48.228076906793,0
-0.72285693290944,48.22808935891,0
-0.72299999999996,48.228093520174,0
-0.72314306709046,48.22808935891,0
-0.72328504528468,48.228076906793,0
-0.72342485397556,48.228056258595,0
-0.72356142907145,48.228027571471,0
-0.7236937310964,48.227991063762,0
-0.72382075310285,48.227947013329,0
-0.72394152833674,48.227895755442,0
-0.72405513759617,48.227837680225,0
-0.72416071622806,48.227773229689,0
-0.72425746070924,48.227702894363,0
-0.72434463476183,48.227627209565,0
-0.72442157495669,48.227546751325,0
-0.72448769576187,48.227462131999,0
-0.72454249399811,48.227373995611,0
-0.72458555266694,48.227283012947,0
-0.7246165441229,48.227189876455,0
-0.72463523256543,48.227095294969,0
-0.72464147583142,48.226999988318,0
-0.72463522647523,48.226904681844,0
-0.72461653212752,48.226810100885,0
-0.72458553513084,48.226716965253,0
-0.72454247145413,48.226625983758,0
-0.72448766889501,48.226537848809,0
-0.72442154458326,48.226453231152,0
-0.72434460180474,48.226372774757,0
-0.72425742616987,48.226297091926,0
-0.72416068115588,48.226226758629,0
-0.7240551030568,48.226162310121,0
-0.72394149537965,48.226104236871,0
-0.72382072272945,48.22605298083,0
-0.72369370422954,48.226008932065,0
-0.72356140652747,48.225972425796,0
-0.72342483643945,48.22594373984,0
-0.72328503328928,48.225923092503,0
-0.72314306100023,48.225910640912,0
-0.72299999999996,48.225906479826,0
-0.72285693899966,48.225910640912,0
-0.72271496671064,48.225923092503,0
-0.72257516356044,48.22594373984,0

-0.72243859347242,48.225972425796,0
-0.72230629577038,48.226008932065,0
-0.72217927727047,48.22605298083,0
-0.72205850462027,48.226104236871,0
-0.72194489694312,48.226162310121,0
-0.72183931884404,48.226226758629,0
-0.72174257383005,48.226297091926,0
-0.72165539819518,48.226372774757,0
-0.72157845541664,48.226453231152,0
-0.72151233110488,48.226537848809,0
-0.72145752854576,48.226625983758,0
-0.72141446486908,48.226716965253,0
-0.7213834678724,48.226810100885,0
-0.72136477352469,48.226904681844,0
-0.72135852416847,48.226999988318,0
-0.72299999999996,48.227,0
</coordinates>
</LinearRing>
</outerBoundaryIs>
</Polygon>
</Placemark>
<Placemark>
<name>203</name>
<styleUrl>#stylehap2</styleUrl>
<Polygon>
<outerBoundaryIs>
<LinearRing>
<coordinates>
-0.71900000000005,48.359,0
-0.71735427524408,48.358999988263,0
-0.71736053465643,48.359095294916,0
-0.71737927145983,48.359189876403,0
-0.7174103431236,48.359283012897,0
-0.71745351323719,48.359373995563,0
-0.71750845330739,48.359462131954,0
-0.71757474525669,48.359546751284,0
-0.71765188460353,48.359627209529,0
-0.71773928430057,48.359702894331,0
-0.71783627920148,48.359773229662,0
-0.71794213112263,48.359837680203,0
-0.71805603446083,48.359895755424,0
-0.71817712232482,48.359947013316,0
-0.71830447313315,48.359991063752,0
-0.71843711762906,48.360027571465,0
-0.71857404625831,48.360056258591,0
-0.71871421685384,48.360076906791,0
-0.7188565625693,48.36008935891,0
-0.71900000000005,48.360093520174,0
-0.7191434374308,48.36008935891,0
-0.71928578314625,48.360076906791,0
-0.71942595374181,48.360056258591,0
-0.71956288237104,48.360027571465,0
-0.71969552686697,48.359991063752,0
-0.7198228776753,48.359947013316,0
-0.71994396553926,48.359895755424,0
-0.72005786887749,48.359837680203,0
-0.72016372079861,48.359773229662,0

-0.72026071569952,48.359702894331,0
-0.72034811539656,48.359627209529,0
-0.72042525474343,48.359546751284,0
-0.72049154669271,48.359462131954,0
-0.72054648676293,48.359373995563,0
-0.72058965687649,48.359283012897,0
-0.72062072854026,48.359189876403,0
-0.72063946534366,48.359095294916,0
-0.72064572475604,48.358999988263,0
-0.72063945920929,48.35890468179,0
-0.72062071645792,48.358810100832,0
-0.72058963921327,48.358716965203,0
-0.72054646405553,48.35862598371,0
-0.7204915196311,48.358537848765,0
-0.72042522414983,48.358453231111,0
-0.72034808220055,48.358372774721,0
-0.72026068090978,48.358297091894,0
-0.72016368547217,48.358226758602,0
-0.72005783408772,48.358162310098,0
-0.71994393234325,48.358104236853,0
-0.7198228470817,48.358052980816,0
-0.71969549980534,48.358008932055,0
-0.71956285966363,48.357972425789,0
-0.71942593607856,48.357943739837,0
-0.71928577106388,48.357923092501,0
-0.71914343129642,48.357910640912,0
-0.71900000000005,48.357906479826,0
-0.71885656870367,48.357910640912,0
-0.71871422893621,48.357923092501,0
-0.71857406392153,48.357943739837,0
-0.71843714033646,48.357972425789,0
-0.71830450019478,48.358008932055,0
-0.71817715291842,48.358052980816,0
-0.71805606765684,48.358104236853,0
-0.71794216591237,48.358162310098,0
-0.71783631452793,48.358226758602,0
-0.71773931909034,48.358297091894,0
-0.71765191779954,48.358372774721,0
-0.71757477585029,48.358453231111,0
-0.71750848036902,48.358537848765,0
-0.71745353594459,48.35862598371,0
-0.71741036078682,48.358716965203,0
-0.7173792835422,48.358810100832,0
-0.71736054079081,48.35890468179,0
-0.71735427524408,48.358999988263,0
-0.71900000000005,48.359,0
</coordinates>
</LinearRing>
</outerBoundaryIs>
</Polygon>
</Placemark>
<Placemark>
<name>213</name>
<styleUrl>#stylehap2</styleUrl>
<Polygon>
<outerBoundaryIs>
<LinearRing>

<coordinates>
-0.70699999999999,47.807,0
-0.70537184156988,47.806999988489,0
-0.70537803422804,47.807095295139,0
-0.70539657109286,47.807189876621,0
-0.70542731115335,47.807283013107,0
-0.70547002052069,47.807373995762,0
-0.7055243742066,47.80746213214,0
-0.70558995859483,47.807546751453,0
-0.70566627458756,47.80762720968,0
-0.70575274140282,47.807702894463,0
-0.70584870099352,47.807773229774,0
-0.70595342305518,47.807837680296,0
-0.70606611058374,47.807895755498,0
-0.70618590594145,47.807947013372,0
-0.70631189738457,47.807991063792,0
-0.70644312600311,47.808027571491,0
-0.70657859301993,47.808056258606,0
-0.70671726739348,47.808076906798,0
-0.70685809366639,47.808089358912,0
-0.707,47.808093520174,0
-0.70714190633358,47.808089358912,0
-0.70728273260652,47.808076906798,0
-0.70742140698007,47.808056258606,0
-0.7075568739969,47.808027571491,0
-0.70768810261543,47.807991063792,0
-0.70781409405853,47.807947013372,0
-0.70793388941627,47.807895755498,0
-0.7080465769448,47.807837680296,0
-0.70815129900646,47.807773229774,0
-0.70824725859716,47.807702894463,0
-0.70833372541242,47.80762720968,0
-0.70841004140515,47.807546751453,0
-0.70847562579338,47.80746213214,0
-0.70852997947929,47.807373995762,0
-0.70857268884665,47.807283013107,0
-0.70860342890712,47.807189876621,0
-0.70862196577194,47.807095295139,0
-0.70862815843012,47.806999988489,0
-0.70862195981955,47.806904682013,0
-0.70860341718316,47.806810101051,0
-0.70857267170738,47.806716965413,0
-0.70852995744547,47.806625983909,0
-0.70847559953449,47.80653784895,0
-0.70841001171908,47.80645323128,0
-0.70833369320111,47.806372774872,0
-0.70824722483939,47.806297092027,0
-0.70815126472792,47.806226758714,0
-0.70804654318703,47.806162310192,0
-0.70793385720496,47.806104236927,0
-0.70781406437243,47.806052980872,0
-0.70768807635652,47.806008932096,0
-0.70755685196306,47.805972425816,0
-0.7074213898408,47.805943739852,0
-0.70728272088256,47.805923092508,0
-0.70714190038119,47.805910640913,0
-0.707,47.805906479826,0

-0.70685809961881,47.805910640913,0
-0.70671727911744,47.805923092508,0
-0.7065786101592,47.805943739852,0
-0.70644314803692,47.805972425816,0
-0.70631192364346,47.806008932096,0
-0.70618593562755,47.806052980872,0
-0.70606614279505,47.806104236927,0
-0.70595345681295,47.806162310192,0
-0.70584873527208,47.806226758714,0
-0.70575277516062,47.806297092027,0
-0.70566630679887,47.806372774872,0
-0.70558998828093,47.80645323128,0
-0.70552440046552,47.80653784895,0
-0.70547004255453,47.806625983909,0
-0.70542732829262,47.806716965413,0
-0.70539658281682,47.806810101051,0
-0.70537804018046,47.806904682013,0
-0.70537184156988,47.806999988489,0
-0.70699999999999,47.807,0
</coordinates>
</LinearRing>
</outerBoundaryIs>
</Polygon>
</Placemark>
<Placemark>
<name>216</name>
<styleUrl>#stylehap2</styleUrl>
<Polygon>
<outerBoundaryIs>
<LinearRing>
<coordinates>
-0.70600000000002,48.258,0
-0.7043575290704,48.257999988305,0
-0.70436377611787,48.258095294957,0
-0.70438247588651,48.258189876443,0
-0.70441348612715,48.258283012936,0
-0.70445657089635,48.258373995599,0
-0.7045114023501,48.258462131989,0
-0.70457756323724,48.258546751315,0
-0.70465455007351,48.258627209557,0
-0.70474177697203,48.258702894355,0
-0.7048385801015,48.258773229682,0
-0.70494422273774,48.25883768022,0
-0.70505790087044,48.258895755438,0
-0.70517874932227,48.258947013326,0
-0.70530584833415,48.25899106376,0
-0.7054382305658,48.25902757147,0
-0.70557488845918,48.259056258594,0
-0.70571478190813,48.259076906792,0
-0.70585684617582,48.25908935891,0
-0.70600000000002,48.259093520174,0
-0.70614315382422,48.25908935891,0
-0.70628521809191,48.259076906792,0
-0.70642511154085,48.259056258594,0
-0.70656176943424,48.25902757147,0
-0.70669415166588,48.25899106376,0
-0.70682125067776,48.258947013326,0

-0.7069420991296,48.258895755438,0
-0.70705577726229,48.25883768022,0
-0.70716141989854,48.258773229682,0
-0.707258223028,48.258702894355,0
-0.70734544992652,48.258627209557,0
-0.70742243676279,48.258546751315,0
-0.70748859764994,48.258462131989,0
-0.70754342910368,48.258373995599,0
-0.70758651387289,48.258283012936,0
-0.70761752411353,48.258189876443,0
-0.70763622388217,48.258095294957,0
-0.70764247092964,48.257999988305,0
-0.70763621778161,48.257904681831,0
-0.70761751209777,48.257810100872,0
-0.70758649630704,48.257716965241,0
-0.70754340652146,48.257625983747,0
-0.70748857073751,48.257537848799,0
-0.70742240633784,48.257453231142,0
-0.70734541691354,48.257372774748,0
-0.70725818843003,48.257297091919,0
-0.70716138476684,48.257226758623,0
-0.70705574266432,48.257162310116,0
-0.70694206611661,48.257104236867,0
-0.70682122025281,48.257052980827,0
-0.70669412475345,48.257008932063,0
-0.70656174685205,48.256972425794,0
-0.706425093975,48.256943739839,0
-0.70628520607615,48.256923092502,0
-0.70614314772366,48.256910640912,0
-0.70600000000002,48.256906479826,0
-0.70585685227638,48.256910640912,0
-0.70571479392388,48.256923092502,0
-0.70557490602503,48.256943739839,0
-0.70543825314801,48.256972425794,0
-0.70530587524658,48.257008932063,0
-0.70517877974723,48.257052980827,0
-0.70505793388343,48.257104236867,0
-0.70494425733572,48.257162310116,0
-0.70483861523319,48.257226758623,0
-0.70474181157,48.257297091919,0
-0.7046545830865,48.257372774748,0
-0.7045775936622,48.257453231142,0
-0.70451142926253,48.257537848799,0
-0.70445659347857,48.257625983747,0
-0.704413503693,48.257716965241,0
-0.70438248790226,48.257810100872,0
-0.70436378221842,48.257904681831,0
-0.7043575290704,48.257999988305,0
-0.70600000000002,48.258,0
</coordinates>
</LinearRing>
</outerBoundaryIs>
</Polygon>
</Placemark>
<Placemark>
<name>217</name>
<styleUrl>#stylehap2</styleUrl>

<Polygon>
<outerBoundaryIs>
<LinearRing>
<coordinates>
-0.70500000000004,48.234,0
-0.7033582996164,48.233999988315,0
-0.70336454373572,48.234095294967,0
-0.70338323473407,48.234189876453,0
-0.70341423042897,48.234283012945,0
-0.70345729498755,48.234373995608,0
-0.70351210071959,48.234462131997,0
-0.70357823056961,48.234546751323,0
-0.70365518128949,48.234627209563,0
-0.70374236726722,48.234702894361,0
-0.70383912498284,48.234773229687,0
-0.70394471805788,48.234837680224,0
-0.70405834285914,48.234895755441,0
-0.70417913461524,48.234947013328,0
-0.70430617399862,48.234991063761,0
-0.70443849412286,48.235027571471,0
-0.7045750879027,48.235056258594,0
-0.70471491571996,48.235076906793,0
-0.70485691333737,48.23508935891,0
-0.70500000000003,48.235093520174,0
-0.70514308666272,48.23508935891,0
-0.70528508428013,48.235076906793,0
-0.70542491209736,48.235056258594,0
-0.70556150587721,48.235027571471,0
-0.70569382600147,48.234991063761,0
-0.70582086538483,48.234947013328,0
-0.70594165714095,48.234895755441,0
-0.70605528194221,48.234837680224,0
-0.70616087501722,48.234773229687,0
-0.70625763273287,48.234702894361,0
-0.70634481871058,48.234627209563,0
-0.70642176943045,48.234546751323,0
-0.7064878992805,48.234462131997,0
-0.70654270501254,48.234373995608,0
-0.70658576957112,48.234283012945,0
-0.70661676526602,48.234189876453,0
-0.70663545626437,48.234095294967,0
-0.70664170038369,48.233999988315,0
-0.70663545017183,48.233904681841,0
-0.70661675326602,48.233810100882,0
-0.7065857520283,48.233716965251,0
-0.70654268245994,48.233625983755,0
-0.70648787240333,48.233537848807,0
-0.70642173904542,48.23345323115,0
-0.7063447857409,48.233372774755,0
-0.70625759818029,48.233297091925,0
-0.70616083993161,48.233226758628,0
-0.70605524738963,48.23316231012,0
-0.70594162417127,48.23310423687,0
-0.70582083499979,48.233052980829,0
-0.70569379912431,48.233008932064,0
-0.70556148332461,48.232972425795,0
-0.70542489455457,48.23294373984,0

-0.70528507228016,48.232923092503,0
-0.70514308057018,48.232910640912,0
-0.70500000000003,48.232906479827,0
-0.70485691942991,48.232910640912,0
-0.70471492771994,48.232923092503,0
-0.70457510544552,48.23294373984,0
-0.70443851667546,48.232972425795,0
-0.70430620087575,48.233008932064,0
-0.70417916500027,48.233052980829,0
-0.70405837582882,48.23310423687,0
-0.70394475261046,48.23316231012,0
-0.70383916006848,48.233226758628,0
-0.7037424018198,48.233297091925,0
-0.70365521425919,48.233372774755,0
-0.70357826095465,48.23345323115,0
-0.70351212759673,48.233537848807,0
-0.70345731754015,48.233625983755,0
-0.70341424797176,48.233716965251,0
-0.70338324673405,48.233810100882,0
-0.70336454982826,48.233904681841,0
-0.7033582996164,48.233999988315,0
-0.70500000000004,48.234,0
</coordinates>
</LinearRing>
</outerBoundaryIs>
</Polygon>
</Placemark>
<Placemark>
<name>219</name>
<styleUrl>#stylehap2</styleUrl>
<Polygon>
<outerBoundaryIs>
<LinearRing>
<coordinates>
-0.702,48.154,0
-0.70036086081492,48.153999988348,0
-0.70036709520139,48.154095294999,0
-0.7003857570484,48.154189876484,0
-0.70041670439503,48.154283012975,0
-0.7004597017761,48.154373995637,0
-0.70051442201239,48.154462132024,0
-0.70058044869887,48.154546751347,0
-0.7006572793724,48.154627209585,0
-0.70074432933445,48.154702894381,0
-0.70084093610021,48.154773229704,0
-0.7009463644399,48.154837680238,0
-0.70105981197413,48.154895755452,0
-0.70118041528077,48.154947013337,0
-0.70130725646648,48.154991063767,0
-0.70143937015351,48.155027571475,0
-0.7015757508279,48.155056258597,0
-0.70171536049355,48.155076906794,0
-0.70185713657375,48.155089358911,0
-0.702,48.155093520174,0
-0.70214286342623,48.155089358911,0
-0.70228463950645,48.155076906794,0
-0.70242424917211,48.155056258597,0

-0.70256062984649,48.155027571475,0
-0.70269274353352,48.154991063767,0
-0.70281958471924,48.154947013337,0
-0.70294018802587,48.154895755452,0
-0.70305363556011,48.154837680238,0
-0.70315906389979,48.154773229704,0
-0.70325567066553,48.154702894381,0
-0.7033427206276,48.154627209585,0
-0.70341955130113,48.154546751347,0
-0.70348557798762,48.154462132024,0
-0.70354029822391,48.154373995637,0
-0.70358329560498,48.154283012975,0
-0.70361424295158,48.154189876484,0
-0.70363290479862,48.154095294999,0
-0.70363913918506,48.153999988348,0
-0.70363289873264,48.153904681873,0
-0.70361423100394,48.153810100914,0
-0.70358327813867,48.153716965281,0
-0.70354027576967,48.153625983784,0
-0.7034855512277,48.153537848834,0
-0.70341952104861,48.153453231174,0
-0.70334268780171,48.153372774777,0
-0.70325563626366,48.153297091944,0
-0.7031590289672,48.153226758644,0
-0.70305360115824,48.153162310133,0
-0.70294015519998,48.153104236881,0
-0.70281955446674,48.153052980837,0
-0.70269271677361,48.15300893207,0
-0.70256060739226,48.152972425799,0
-0.7024242317058,48.152943739842,0
-0.70228462755881,48.152923092504,0
-0.70214285736025,48.152910640912,0
-0.702,48.152906479827,0
-0.70185714263973,48.152910640912,0
-0.70171537244119,48.152923092504,0
-0.70157576829418,48.152943739842,0
-0.70143939260774,48.152972425799,0
-0.70130728322639,48.15300893207,0
-0.70118044553326,48.153052980837,0
-0.70105984480003,48.153104236881,0
-0.70094639884177,48.153162310133,0
-0.70084097103278,48.153226758644,0
-0.70074436373632,48.153297091944,0
-0.7006573121983,48.153372774777,0
-0.70058047895137,48.153453231174,0
-0.7005144487723,48.153537848834,0
-0.70045972423033,48.153625983784,0
-0.70041672186131,48.153716965281,0
-0.70038576899607,48.153810100914,0
-0.70036710126737,48.153904681873,0
-0.70036086081492,48.153999988348,0
-0.702,48.154,0
</coordinates>
</LinearRing>
</outerBoundaryIs>
</Polygon>
</Placemark>

<Placemark>
<name>221</name>
<styleUrl>#stylehap2</styleUrl>
<Polygon>
<outerBoundaryIs>
<LinearRing>
<coordinates>
-0.70000000000005,48.373,0
-0.69835382279699,48.372999988258,0
-0.69836008392869,48.37309529491,0
-0.69837882588179,48.373189876397,0
-0.6984099060865,48.373283012891,0
-0.69845308806729,48.373373995558,0
-0.69850804324067,48.37346213195,0
-0.69857435341427,48.37354675128,0
-0.69865151396783,48.373627209525,0
-0.69873893769264,48.373702894328,0
-0.69883595925952,48.373773229659,0
-0.69894184028188,48.373837680201,0
-0.69905577493511,48.373895755422,0
-0.69917689608953,48.373947013314,0
-0.69930428191035,48.373991063751,0
-0.69943696287435,48.374027571464,0
-0.69957392914961,48.374056258591,0
-0.6997141382826,48.374076906791,0
-0.69985652313359,48.37408935891,0
-0.70000000000006,48.374093520174,0
-0.7001434768665,48.37408935891,0
-0.70028586171749,48.374076906791,0
-0.70042607085048,48.374056258591,0
-0.70056303712574,48.374027571464,0
-0.70069571808974,48.373991063751,0
-0.70082310391056,48.373947013314,0
-0.70094422506498,48.373895755422,0
-0.70105815971821,48.373837680201,0
-0.70116404074057,48.373773229659,0
-0.70126106230745,48.373702894328,0
-0.70134848603226,48.373627209525,0
-0.70142564658582,48.37354675128,0
-0.70149195675942,48.37346213195,0
-0.7015469119328,48.373373995558,0
-0.7015900939136,48.373283012891,0
-0.7016211741183,48.373189876397,0
-0.7016399160714,48.37309529491,0
-0.7016461772031,48.372999988258,0
-0.70163990993232,48.372904681784,0
-0.70162116202667,48.372810100826,0
-0.70159007623681,48.372716965197,0
-0.70154688920799,48.372625983705,0
-0.70149192967702,48.37253784876,0
-0.70142561596876,48.372453231107,0
-0.70134845281078,48.372372774717,0
-0.70126102749099,48.372297091891,0
-0.701164005387,48.372226758599,0
-0.70105812490175,48.372162310096,0
-0.7009441918435,48.372104236851,0
-0.7008230732935,48.372052980815,0

-0.70069569100734,48.372008932054,0
-0.70056301440094,48.371972425789,0
-0.70042605317372,48.371943739836,0
-0.70028584962586,48.371923092501,0
-0.70014347072745,48.371910640912,0
-0.70000000000006,48.371906479826,0
-0.69985652927264,48.371910640912,0
-0.69971415037423,48.371923092501,0
-0.69957394682637,48.371943739836,0
-0.69943698559916,48.371972425789,0
-0.69930430899275,48.372008932054,0
-0.6991769267066,48.372052980815,0
-0.69905580815659,48.372104236851,0
-0.69894187509834,48.372162310096,0
-0.69883599461309,48.372226758599,0
-0.6987389725091,48.372297091891,0
-0.69865154718931,48.372372774717,0
-0.69857438403133,48.372453231107,0
-0.69850807032307,48.37253784876,0
-0.6984531107921,48.372625983705,0
-0.69840992376328,48.372716965197,0
-0.69837883797342,48.372810100826,0
-0.69836009006777,48.372904681784,0
-0.69835382279699,48.372999988258,0
-0.70000000000005,48.373,0
</coordinates>
</LinearRing>
</outerBoundaryIs>
</Polygon>
</Placemark>
<Placemark>
<name>222</name>
<styleUrl>#stylehap2</styleUrl>
<Polygon>
<outerBoundaryIs>
<LinearRing>
<coordinates>
-0.70000000000005,48.261,0
-0.69835743268103,48.260999988304,0
-0.6983636800948,48.261095294956,0
-0.69838238096055,48.261189876442,0
-0.69841339302075,48.261283012934,0
-0.69845648031814,48.261373995598,0
-0.69851131498946,48.261462131988,0
-0.69857747975912,48.261546751315,0
-0.69865447111325,48.261627209556,0
-0.69874170313066,48.261702894355,0
-0.69883851194106,48.261773229682,0
-0.69894416077704,48.26183768022,0
-0.69905784558107,48.261895755438,0
-0.69917870112513,48.261947013326,0
-0.69930580759605,48.261991063759,0
-0.69943819759684,48.262027571469,0
-0.69957486351037,48.262056258594,0
-0.69971476516934,48.262076906792,0
-0.69985683777446,48.26208935891,0
-0.70000000000006,48.262093520174,0

-0.70014316222563,48.26208935891,0
-0.70028523483075,48.262076906792,0
-0.70042513648972,48.262056258594,0
-0.70056180240325,48.262027571469,0
-0.70069419240405,48.261991063759,0
-0.70082129887496,48.261947013326,0
-0.70094215441903,48.261895755438,0
-0.70105583922305,48.26183768022,0
-0.70116148805903,48.261773229682,0
-0.70125829686943,48.261702894355,0
-0.70134552888684,48.261627209556,0
-0.70142252024097,48.261546751315,0
-0.70148868501063,48.261462131988,0
-0.70154351968195,48.261373995598,0
-0.70158660697934,48.261283012934,0
-0.70161761903954,48.261189876442,0
-0.70163631990529,48.261095294956,0
-0.70164256731906,48.260999988304,0
-0.70163631380374,48.26090468183,0
-0.70161760702183,48.260810100871,0
-0.70158658941062,48.26071696524,0
-0.70154349709604,48.260625983746,0
-0.70148865809377,48.260537848798,0
-0.70142248981103,48.260453231141,0
-0.70134549586841,48.260372774748,0
-0.70125826226579,48.260297091918,0
-0.70116145292156,48.260226758622,0
-0.70105580461943,48.260162310115,0
-0.70094212140062,48.260104236867,0
-0.70082126844505,48.260052980826,0
-0.70069416548719,48.260008932063,0
-0.70056177981731,48.259972425794,0
-0.700425118921,48.259943739839,0
-0.70028522281303,48.259923092502,0
-0.70014315612408,48.259910640912,0
-0.70000000000006,48.259906479826,0
-0.69985684387601,48.259910640912,0
-0.69971477718706,48.259923092502,0
-0.69957488107909,48.259943739839,0
-0.69943822018278,48.259972425794,0
-0.6993058345129,48.260008932063,0
-0.69917873155505,48.260052980826,0
-0.69905787859947,48.260104236867,0
-0.69894419538066,48.260162310115,0
-0.69883854707853,48.260226758622,0
-0.69874173773431,48.260297091918,0
-0.69865450413169,48.260372774748,0
-0.69857751018907,48.260453231141,0
-0.69851134190632,48.260537848798,0
-0.69845650290405,48.260625983746,0
-0.69841341058947,48.26071696524,0
-0.69838239297826,48.260810100871,0
-0.69836368619635,48.26090468183,0
-0.69835743268103,48.260999988304,0
-0.70000000000005,48.261,0
</coordinates>
</LinearRing>

</outerBoundaryIs>
</Polygon>
</Placemark>
<Placemark>
<name>223</name>
<styleUrl>#stylehap2</styleUrl>
<Polygon>
<outerBoundaryIs>
<LinearRing>
<coordinates>
-0.69799999999998,48.383,0
-0.69635349940789,48.382999988254,0
-0.69635976176851,48.383095294906,0
-0.6963785074024,48.383189876393,0
-0.69640959371178,48.383283012887,0
-0.69645278417471,48.383373995554,0
-0.6965077501432,48.383462131946,0
-0.6965740733427,48.383546751277,0
-0.69665124905389,48.383627209522,0
-0.69673868995267,48.383702894325,0
-0.6968357305792,48.383773229657,0
-0.69694163240182,48.383837680199,0
-0.69705558943761,48.383895755421,0
-0.69717673438655,48.383947013313,0
-0.69730414523276,48.383991063751,0
-0.69743685226247,48.384027571464,0
-0.69757384544546,48.38405625859,0
-0.6977140821233,48.384076906791,0
-0.69785649494663,48.38408935891,0
-0.69799999999999,48.384093520174,0
-0.69814350505335,48.38408935891,0
-0.69828591787665,48.384076906791,0
-0.69842615455452,48.38405625859,0
-0.69856314773748,48.384027571464,0
-0.69869585476721,48.383991063751,0
-0.6988232656134,48.383947013313,0
-0.69894441056234,48.383895755421,0
-0.69905836759813,48.383837680199,0
-0.69916426942078,48.383773229657,0
-0.6992613100473,48.383702894325,0
-0.69934875094608,48.383627209522,0
-0.69942592665725,48.383546751277,0
-0.69949224985675,48.383462131946,0
-0.69954721582526,48.383373995554,0
-0.69959040628819,48.383283012887,0
-0.69962149259758,48.383189876393,0
-0.69964023823144,48.383095294906,0
-0.69964650059206,48.382999988254,0
-0.699640232089,48.38290468178,0
-0.69962148049931,48.382810100822,0
-0.69959038860171,48.382716965193,0
-0.69954719308796,48.382625983701,0
-0.69949222275952,48.382537848757,0
-0.6994258960234,48.382453231104,0
-0.69934871770639,48.382372774714,0
-0.69926127521175,48.382297091889,0
-0.69916423404784,48.382226758597,0

-0.6990583327626,48.382162310094,0
-0.69894437732267,48.38210423685,0
-0.69882323497954,48.382052980814,0
-0.69869582766996,48.382008932054,0
-0.6985631250002,48.381972425788,0
-0.69842613686804,48.381943739836,0
-0.69828590577841,48.381923092501,0
-0.69814349891091,48.381910640912,0
-0.69799999999999,48.381906479826,0
-0.69785650108906,48.381910640912,0
-0.69771409422157,48.381923092501,0
-0.69757386313191,48.381943739836,0
-0.69743687499977,48.381972425788,0
-0.69730417232999,48.382008932054,0
-0.69717676502041,48.382052980814,0
-0.69705562267731,48.38210423685,0
-0.69694166723737,48.382162310094,0
-0.69683576595213,48.382226758597,0
-0.69673872478819,48.382297091889,0
-0.69665128229356,48.382372774714,0
-0.69657410397655,48.382453231104,0
-0.69650777724043,48.382537848757,0
-0.69645280691199,48.382625983701,0
-0.69640961139824,48.382716965193,0
-0.69637851950064,48.382810100822,0
-0.69635976791095,48.38290468178,0
-0.69635349940789,48.382999988254,0
-0.69799999999998,48.383,0
</coordinates>
</LinearRing>
</outerBoundaryIs>
</Polygon>
</Placemark>
<Placemark>
<name>225</name>
<styleUrl>#stylehap2</styleUrl>
<Polygon>
<outerBoundaryIs>
<LinearRing>
<coordinates>
-0.69499999999994,48.261,0
-0.69335743268093,48.260999988304,0
-0.6933636800947,48.261095294956,0
-0.69338238096042,48.261189876442,0
-0.69341339302065,48.261283012934,0
-0.69345648031801,48.261373995598,0
-0.69351131498936,48.261462131988,0
-0.69357747975902,48.261546751315,0
-0.69365447111315,48.261627209556,0
-0.69374170313056,48.261702894355,0
-0.69383851194096,48.261773229682,0
-0.69394416077692,48.26183768022,0
-0.69405784558096,48.261895755438,0
-0.69417870112503,48.261947013326,0
-0.69430580759594,48.261991063759,0
-0.69443819759674,48.262027571469,0
-0.69457486351027,48.262056258594,0

-0.69471476516924,48.262076906792,0
-0.69485683777436,48.26208935891,0
-0.69499999999993,48.262093520174,0
-0.69514316222553,48.26208935891,0
-0.69528523483065,48.262076906792,0
-0.69542513648962,48.262056258594,0
-0.69556180240312,48.262027571469,0
-0.69569419240394,48.261991063759,0
-0.69582129887486,48.261947013326,0
-0.6959421544189,48.261895755438,0
-0.69605583922295,48.26183768022,0
-0.69616148805891,48.261773229682,0
-0.6962582968693,48.261702894355,0
-0.69634552888671,48.261627209556,0
-0.69642252024084,48.261546751315,0
-0.6964886850105,48.261462131988,0
-0.69654351968185,48.261373995598,0
-0.69658660697924,48.261283012934,0
-0.69661761903944,48.261189876442,0
-0.69663631990519,48.261095294956,0
-0.69664256731896,48.260999988304,0
-0.69663631380361,48.26090468183,0
-0.69661760702173,48.260810100871,0
-0.69658658941049,48.26071696524,0
-0.69654349709592,48.260625983746,0
-0.69648865809365,48.260537848798,0
-0.69642248981092,48.260453231141,0
-0.6963454958683,48.260372774748,0
-0.69625826226568,48.260297091918,0
-0.69616145292146,48.260226758622,0
-0.6960558046193,48.260162310115,0
-0.69594212140049,48.260104236867,0
-0.69582126844492,48.260052980826,0
-0.69569416548709,48.260008932063,0
-0.69556177981721,48.259972425794,0
-0.6954251189209,48.259943739839,0
-0.69528522281293,48.259923092502,0
-0.69514315612395,48.259910640912,0
-0.69499999999993,48.259906479826,0
-0.69485684387591,48.259910640912,0
-0.69471477718695,48.259923092502,0
-0.69457488107899,48.259943739839,0
-0.69443822018268,48.259972425794,0
-0.6943058345128,48.260008932063,0
-0.69417873155494,48.260052980826,0
-0.69405787859937,48.260104236867,0
-0.69394419538056,48.260162310115,0
-0.6938385470784,48.260226758622,0
-0.6937417377342,48.260297091918,0
-0.69365450413156,48.260372774748,0
-0.69357751018896,48.260453231141,0
-0.69351134190622,48.260537848798,0
-0.69345650290395,48.260625983746,0
-0.69341341058937,48.26071696524,0
-0.69338239297816,48.260810100871,0
-0.69336368619625,48.26090468183,0
-0.69335743268093,48.260999988304,0

-0.69499999999994,48.261,0
</coordinates>
</LinearRing>
</outerBoundaryIs>
</Polygon>
</Placemark>
<Placemark>
<name>226</name>
<styleUrl>#stylehap2</styleUrl>
<Polygon>
<outerBoundaryIs>
<LinearRing>
<coordinates>
-0.69399999999996,48.373,0
-0.6923538227969,48.372999988258,0
-0.69236008392861,48.37309529491,0
-0.6923788258817,48.373189876397,0
-0.69240990608641,48.373283012891,0
-0.69245308806721,48.373373995558,0
-0.69250804324058,48.37346213195,0
-0.69257435341419,48.37354675128,0
-0.69265151396775,48.373627209525,0
-0.69273893769255,48.373702894328,0
-0.69283595925944,48.373773229659,0
-0.69294184028179,48.373837680201,0
-0.69305577493503,48.373895755422,0
-0.69317689608945,48.373947013314,0
-0.69330428191026,48.373991063751,0
-0.69343696287426,48.374027571464,0
-0.69357392914952,48.374056258591,0
-0.69371413828251,48.374076906791,0
-0.6938565231335,48.37408935891,0
-0.69399999999997,48.374093520174,0
-0.69414347686644,48.37408935891,0
-0.6942858617174,48.374076906791,0
-0.69442607085039,48.374056258591,0
-0.69456303712566,48.374027571464,0
-0.69469571808965,48.373991063751,0
-0.69482310391047,48.373947013314,0
-0.69494422506489,48.373895755422,0
-0.69505815971813,48.373837680201,0
-0.69516404074048,48.373773229659,0
-0.69526106230737,48.373702894328,0
-0.69534848603217,48.373627209525,0
-0.69542564658573,48.37354675128,0
-0.69549195675934,48.37346213195,0
-0.69554691193271,48.373373995558,0
-0.69559009391351,48.373283012891,0
-0.69562117411821,48.373189876397,0
-0.69563991607131,48.37309529491,0
-0.69564617720302,48.372999988258,0
-0.69563990993223,48.372904681784,0
-0.69562116202659,48.372810100826,0
-0.69559007623672,48.372716965197,0
-0.6955468892079,48.372625983705,0
-0.69549192967694,48.37253784876,0
-0.69542561596867,48.372453231107,0

-0.69534845281069,48.372372774717,0
-0.6952610274909,48.372297091891,0
-0.69516400538691,48.372226758599,0
-0.69505812490166,48.372162310096,0
-0.69494419184341,48.372104236851,0
-0.69482307329341,48.372052980815,0
-0.69469569100726,48.372008932054,0
-0.69456301440085,48.371972425789,0
-0.69442605317364,48.371943739836,0
-0.69428584962578,48.371923092501,0
-0.69414347072736,48.371910640912,0
-0.69399999999997,48.371906479826,0
-0.69385652927258,48.371910640912,0
-0.69371415037414,48.371923092501,0
-0.69357394682628,48.371943739836,0
-0.69343698559907,48.371972425789,0
-0.69330430899266,48.372008932054,0
-0.69317692670651,48.372052980815,0
-0.6930558081565,48.372104236851,0
-0.69294187509826,48.372162310096,0
-0.69283599461301,48.372226758599,0
-0.69273897250901,48.372297091891,0
-0.69265154718922,48.372372774717,0
-0.69257438403125,48.372453231107,0
-0.69250807032298,48.37253784876,0
-0.69245311079201,48.372625983705,0
-0.69240992376319,48.372716965197,0
-0.69237883797333,48.372810100826,0
-0.69236009006769,48.372904681784,0
-0.6923538227969,48.372999988258,0
-0.69399999999996,48.373,0
</coordinates>
</LinearRing>
</outerBoundaryIs>
</Polygon>
</Placemark>
<Placemark>
<name>229</name>
<styleUrl>#stylehap2</styleUrl>
<Polygon>
<outerBoundaryIs>
<LinearRing>
<coordinates>
-0.68799999999999,48.356,0
-0.68635437215184,48.355999988265,0
-0.68636063119593,48.356095294917,0
-0.68637936689631,48.356189876404,0
-0.68641043673075,48.356283012898,0
-0.68645360430254,48.356373995564,0
-0.68650854113783,48.356462131955,0
-0.68657482918376,48.356546751285,0
-0.68665196398844,48.35662720953,0
-0.68673935853906,48.356702894332,0
-0.68683634772848,48.356773229662,0
-0.68694219341656,48.356837680204,0
-0.68705609004753,48.356895755425,0
-0.68717717078117,48.356947013316,0

-0.68730451409031,48.356991063752,0
-0.68743715077529,48.357027571465,0
-0.68757407134127,48.357056258591,0
-0.68771423368266,48.357076906791,0
-0.6878565710158,48.35708935891,0
-0.68799999999999,48.357093520174,0
-0.68814342898418,48.35708935891,0
-0.68828576631732,48.357076906791,0
-0.6884259286587,48.357056258591,0
-0.68856284922469,48.357027571465,0
-0.68869548590966,48.356991063752,0
-0.6888228292188,48.356947013316,0
-0.68894390995245,48.356895755425,0
-0.68905780658342,48.356837680204,0
-0.68916365227149,48.356773229662,0
-0.68926064146092,48.356702894332,0
-0.68934803601156,48.35662720953,0
-0.68942517081622,48.356546751285,0
-0.68949145886214,48.356462131955,0
-0.68954639569743,48.356373995564,0
-0.68958956326922,48.356283012898,0
-0.68962063310366,48.356189876404,0
-0.68963936880405,48.356095294917,0
-0.68964562784814,48.355999988265,0
-0.68963936267069,48.355904681791,0
-0.68962062102328,48.355810100833,0
-0.6895895456089,48.355716965204,0
-0.68954637299377,48.355625983711,0
-0.68949143180493,48.355537848766,0
-0.68942514022765,48.355453231112,0
-0.689348002821,48.355372774721,0
-0.68926060667687,48.355297091895,0
-0.68916361695085,48.355226758602,0
-0.68905777179937,48.355162310099,0
-0.68894387676188,48.355104236854,0
-0.68882279863024,48.355052980816,0
-0.68869545885248,48.355008932056,0
-0.688562826521,48.35497242579,0
-0.68842591099838,48.354943739837,0
-0.68828575423696,48.354923092501,0
-0.68814342285079,48.354910640912,0
-0.68799999999999,48.354906479826,0
-0.68785657714918,48.354910640912,0
-0.68771424576301,48.354923092501,0
-0.6875740890016,48.354943739837,0
-0.68743717347898,48.35497242579,0
-0.6873045411475,48.355008932056,0
-0.68717720136974,48.355052980816,0
-0.68705612323809,48.355104236854,0
-0.6869422282006,48.355162310099,0
-0.68683638304913,48.355226758602,0
-0.68673939332311,48.355297091895,0
-0.68665199717898,48.355372774721,0
-0.68657485977232,48.355453231112,0
-0.68650856819504,48.355537848766,0
-0.68645362700621,48.355625983711,0
-0.68641045439108,48.355716965204,0

-0.68637937897669,48.355810100833,0
-0.68636063732928,48.355904681791,0
-0.68635437215184,48.355999988265,0
-0.68799999999999,48.356,0
</coordinates>
</LinearRing>
</outerBoundaryIs>
</Polygon>
</Placemark>
<Placemark>
<name>230</name>
<styleUrl>#stylehap2</styleUrl>
<Polygon>
<outerBoundaryIs>
<LinearRing>
<coordinates>
-0.68700000000001,48.177,0
-0.68536012561657,48.176999988338,0
-0.6853663627969,48.17709529499,0
-0.68538503301187,48.177189876475,0
-0.68541599423699,48.177283012967,0
-0.68545901090153,48.177373995629,0
-0.68551375567959,48.177462132016,0
-0.68557981197941,48.17754675134,0
-0.68565667711254,48.177627209579,0
-0.68574376611824,48.177702894375,0
-0.68584041621453,48.177773229699,0
-0.68594589184189,48.177837680234,0
-0.68605939026105,48.177895755449,0
-0.68618004766259,48.177947013334,0
-0.68630694574143,48.177991063766,0
-0.68643911868677,48.178027571474,0
-0.68657556053358,48.178056258596,0
-0.68671523282019,48.178076906793,0
-0.68685707249321,48.178089358911,0
-0.687,48.178093520174,0
-0.68714292750682,48.178089358911,0
-0.68728476717982,48.178076906793,0
-0.68742443946643,48.178056258596,0
-0.68756088131326,48.178027571474,0
-0.6876930542586,48.177991063766,0
-0.68781995233744,48.177947013334,0
-0.68794060973895,48.177895755449,0
-0.68805410815814,48.177837680234,0
-0.6881595837855,48.177773229699,0
-0.68825623388179,48.177702894375,0
-0.68834332288746,48.177627209579,0
-0.6884201880206,48.17754675134,0
-0.68848624432044,48.177462132016,0
-0.6885409890985,48.177373995629,0
-0.68858400576304,48.177283012967,0
-0.68861496698816,48.177189876475,0
-0.68863363720314,48.17709529499,0
-0.68863987438343,48.176999988338,0
-0.68863363112952,48.176904681864,0
-0.68861495502548,48.176810100905,0
-0.6885839882748,48.176716965272,0

-0.68854096661605,48.176625983776,0
-0.68848621752689,48.176537848826,0
-0.68842015773006,48.176453231167,0
-0.68834329002032,48.176372774771,0
-0.68825619943666,48.176297091938,0
-0.68815954880902,48.176226758639,0
-0.68805407371302,48.176162310129,0
-0.68794057687181,48.176104236878,0
-0.68781992204691,48.176052980835,0
-0.68769302746505,48.176008932069,0
-0.68756085883081,48.175972425798,0
-0.68742442197818,48.175943739842,0
-0.68728475521717,48.175923092503,0
-0.68714292143321,48.175910640912,0
-0.687,48.175906479826,0
-0.6868570785668,48.175910640912,0
-0.68671524478286,48.175923092503,0
-0.68657557802185,48.175943739842,0
-0.68643914116922,48.175972425798,0
-0.68630697253498,48.176008932069,0
-0.68618007795312,48.176052980835,0
-0.68605942312822,48.176104236878,0
-0.68594592628702,48.176162310129,0
-0.68584045119102,48.176226758639,0
-0.68574380056334,48.176297091938,0
-0.68565670997971,48.176372774771,0
-0.68557984226994,48.176453231167,0
-0.68551378247312,48.176537848826,0
-0.68545903338398,48.176625983776,0
-0.68541601172523,48.176716965272,0
-0.68538504497455,48.176810100905,0
-0.68536636887048,48.176904681864,0
-0.68536012561657,48.176999988338,0
-0.68700000000001,48.177,0
</coordinates>
</LinearRing>
</outerBoundaryIs>
</Polygon>
</Placemark>
<Placemark>
<name>234</name>
<styleUrl>#stylehap2</styleUrl>
<Polygon>
<outerBoundaryIs>
<LinearRing>
<coordinates>
-0.68200000000002,48.35,0
-0.68035456591972,48.349999988267,0
-0.68036082422747,48.350095294919,0
-0.68037955772243,48.350189876406,0
-0.68041062389906,48.3502830129,0
-0.68045378638853,48.350373995566,0
-0.68050871675564,48.350462131958,0
-0.68057499699668,48.350546751287,0
-0.68065212271925,48.350627209531,0
-0.68073950697958,48.350702894333,0
-0.68083648474888,48.350773229664,0

-0.68094231797386,48.350837680205,0
-0.68105620119368,48.350895755426,0
-0.68117726767014,48.350947013317,0
-0.68130459598463,48.350991063753,0
-0.68143721705158,48.351027571465,0
-0.68157412149502,48.351056258591,0
-0.6817142673321,48.351076906791,0
-0.68185658790481,48.35108935891,0
-0.68200000000003,48.351093520174,0
-0.68214341209522,48.35108935891,0
-0.68228573266793,48.351076906791,0
-0.68242587850501,48.351056258591,0
-0.68256278294848,48.351027571465,0
-0.68269540401541,48.350991063753,0
-0.68282273232989,48.350947013317,0
-0.68294379880635,48.350895755426,0
-0.68305768202617,48.350837680205,0
-0.68316351525115,48.350773229664,0
-0.68326049302045,48.350702894333,0
-0.68334787728078,48.350627209531,0
-0.68342500300335,48.350546751287,0
-0.68349128324439,48.350462131958,0
-0.68354621361151,48.350373995566,0
-0.68358937610097,48.3502830129,0
-0.6836204422776,48.350189876406,0
-0.68363917577256,48.350095294919,0
-0.68364543408032,48.349999988267,0
-0.68363916964122,48.349904681793,0
-0.68362043020121,48.349810100836,0
-0.68358935844645,48.349716965206,0
-0.6835461909153,48.349625983713,0
-0.68349125619609,48.349537848768,0
-0.68342497242481,48.349453231114,0
-0.68334784410116,48.349372774723,0
-0.68326045824783,48.349297091897,0
-0.68316347994211,48.349226758604,0
-0.68305764725355,48.3491623101,0
-0.6829437656267,48.349104236855,0
-0.68282270175137,48.349052980817,0
-0.68269537696713,48.349008932056,0
-0.68256276025225,48.34897242579,0
-0.68242586085049,48.348943739837,0
-0.68228572059154,48.348923092501,0
-0.68214340596385,48.348910640912,0
-0.68200000000003,48.348906479826,0
-0.68185659403619,48.348910640912,0
-0.68171427940849,48.348923092501,0
-0.68157413914955,48.348943739837,0
-0.68143723974779,48.34897242579,0
-0.6813046230329,48.349008932056,0
-0.68117729824866,48.349052980817,0
-0.68105623437333,48.349104236855,0
-0.68094235274648,48.3491623101,0
-0.68083652005792,48.349226758604,0
-0.6807395417522,48.349297091897,0
-0.68065215589887,48.349372774723,0
-0.68057502757522,48.349453231114,0

-0.68050874380394,48.349537848768,0
-0.68045380908473,48.349625983713,0
-0.68041064155358,48.349716965206,0
-0.68037956979882,48.349810100836,0
-0.68036083035882,48.349904681793,0
-0.68035456591972,48.349999988267,0
-0.68200000000002,48.35,0
</coordinates>
</LinearRing>
</outerBoundaryIs>
</Polygon>
</Placemark>
<Placemark>
<name>241</name>
<styleUrl>#stylehap2</styleUrl>
<Polygon>
<outerBoundaryIs>
<LinearRing>
<coordinates>
-0.67200000000003,48.139,0
-0.67036133979488,48.138999988354,0
-0.67036757236116,48.139095295005,0
-0.67038622875647,48.13918987649,0
-0.67041716706128,48.139283012981,0
-0.67046015187922,48.139373995643,0
-0.6705148561266,48.139462132029,0
-0.67058086352007,48.139546751352,0
-0.67065767174326,48.139627209589,0
-0.67074469626848,48.139702894384,0
-0.67084127480448,48.139773229707,0
-0.67094667233637,48.13983768024,0
-0.6710600867192,48.139895755454,0
-0.67118065478313,48.139947013338,0
-0.67130745890314,48.139991063768,0
-0.6714395339835,48.140027571475,0
-0.67157587480419,48.140056258597,0
-0.67171544367246,48.140076906794,0
-0.67185717832212,48.140089358911,0
-0.67200000000003,48.140093520174,0
-0.67214282167794,48.140089358911,0
-0.67228455632759,48.140076906794,0
-0.67242412519584,48.140056258597,0
-0.67256046601653,48.140027571475,0
-0.67269254109692,48.139991063768,0
-0.67281934521691,48.139947013338,0
-0.67293991328086,48.139895755454,0
-0.67305332766369,48.13983768024,0
-0.67315872519555,48.139773229707,0
-0.67325530373158,48.139702894384,0
-0.6733423282568,48.139627209589,0
-0.67341913647998,48.139546751352,0
-0.67348514387346,48.139462132029,0
-0.67353984812084,48.139373995643,0
-0.67358283293877,48.139283012981,0
-0.67361377124359,48.13918987649,0
-0.67363242763888,48.139095295005,0
-0.67363866020518,48.138999988354,0

-0.67363242157789,48.138904681879,0
-0.67361375930572,48.13881010092,0
-0.67358281548677,48.138716965287,0
-0.67353982568498,48.13862598379,0
-0.67348511713545,48.138537848839,0
-0.67341910625225,48.138453231179,0
-0.67334229545777,48.138372774781,0
-0.67325526935787,48.138297091947,0
-0.67315869029159,48.138226758647,0
-0.67305329328998,48.138162310136,0
-0.67293988048183,48.138104236883,0
-0.67281931498917,48.138052980839,0
-0.67269251435891,48.138008932071,0
-0.67256044358069,48.1379724258,0
-0.67242410774386,48.137943739843,0
-0.67228454438973,48.137923092504,0
-0.67214281561692,48.137910640912,0
-0.67200000000003,48.137906479826,0
-0.67185718438314,48.137910640912,0
-0.67171545561031,48.137923092504,0
-0.6715758922562,48.137943739843,0
-0.67143955641936,48.1379724258,0
-0.67130748564112,48.138008932071,0
-0.67118068501086,48.138052980839,0
-0.67106011951822,48.138104236883,0
-0.67094670671007,48.138162310136,0
-0.67084130970847,48.138226758647,0
-0.67074473064219,48.138297091947,0
-0.67065770454226,48.138372774781,0
-0.67058089374781,48.138453231179,0
-0.67051488286461,48.138537848839,0
-0.67046017431505,48.13862598379,0
-0.67041718451327,48.138716965287,0
-0.67038624069434,48.13881010092,0
-0.67036757842217,48.138904681879,0
-0.67036133979488,48.138999988354,0
-0.67200000000003,48.139,0
</coordinates>
</LinearRing>
</outerBoundaryIs>
</Polygon>
</Placemark>
<Placemark>
<name>248</name>
<styleUrl>#stylehap2</styleUrl>
<Polygon>
<outerBoundaryIs>
<LinearRing>
<coordinates>
-0.65599999999995,48.155,0
-0.65436082886893,48.154999988347,0
-0.6543670633768,48.155095294999,0
-0.65438572558742,48.155189876484,0
-0.6544166735371,48.155283012975,0
-0.65445967175606,48.155373995637,0
-0.65451439305875,48.155462132023,0
-0.65458042103199,48.155546751347,0

-0.65465725320287,48.155627209585,0
-0.65474430486145,48.15570289438,0
-0.65484091351002,48.155773229704,0
-0.65494634390445,48.155837680238,0
-0.65505979364975,48.155895755452,0
-0.65518039930692,48.155947013337,0
-0.65530724296476,48.155991063767,0
-0.6554393592267,48.156027571475,0
-0.65557574255916,48.156056258597,0
-0.65571535494582,48.156076906794,0
-0.65585713378928,48.156089358911,0
-0.65599999999994,48.156093520174,0
-0.65614286621063,48.156089358911,0
-0.65628464505409,48.156076906794,0
-0.65642425744075,48.156056258597,0
-0.65656064077322,48.156027571475,0
-0.65669275703512,48.155991063767,0
-0.65681960069299,48.155947013337,0
-0.65694020635016,48.155895755452,0
-0.65705365609547,48.155837680238,0
-0.65715908648989,48.155773229704,0
-0.65725569513844,48.15570289438,0
-0.65734274679702,48.155627209585,0
-0.6574195789679,48.155546751347,0
-0.65748560694117,48.155462132023,0
-0.65754032824383,48.155373995637,0
-0.65758332646281,48.155283012975,0
-0.65761427441247,48.155189876484,0
-0.65763293662311,48.155095294999,0
-0.65763917113098,48.154999988347,0
-0.6576329305568,48.154904681873,0
-0.65761426246417,48.154810100913,0
-0.65758330899556,48.154716965281,0
-0.65754030578838,48.154625983784,0
-0.65748558017978,48.154537848834,0
-0.65741954871375,48.154453231174,0
-0.65734271396935,48.154372774777,0
-0.65725566073468,48.154297091944,0
-0.65715905155539,48.154226758644,0
-0.65705362169169,48.154162310133,0
-0.65694017352246,48.154104236881,0
-0.65681957043882,48.154052980837,0
-0.65669273027376,48.15400893207,0
-0.65656061831774,48.153972425799,0
-0.6564242399735,48.153943739842,0
-0.65628463310579,48.153923092504,0
-0.65614286014432,48.153910640912,0
-0.65599999999994,48.153906479827,0
-0.65585713985559,48.153910640912,0
-0.65571536689412,48.153923092504,0
-0.65557576002638,48.153943739842,0
-0.65543938168215,48.153972425799,0
-0.65530726972613,48.15400893207,0
-0.65518042956107,48.154052980837,0
-0.65505982647743,48.154104236881,0
-0.6549463783082,48.154162310133,0
-0.6548409484445,48.154226758644,0

-0.6547443392652,48.154297091944,0
-0.65465728603054,48.154372774777,0
-0.65458045128616,48.154453231174,0
-0.65451441982011,48.154537848834,0
-0.65445969421151,48.154625983784,0
-0.65441669100432,48.154716965281,0
-0.65438573753572,48.154810100913,0
-0.65436706944311,48.154904681873,0
-0.65436082886893,48.154999988347,0
-0.65599999999995,48.155,0
</coordinates>
</LinearRing>
</outerBoundaryIs>
</Polygon>
</Placemark>
<Placemark>
<name>250</name>
<styleUrl>#stylehap2</styleUrl>
<Polygon>
<outerBoundaryIs>
<LinearRing>
<coordinates>
-0.65200000000004,48.334,0
-0.65035508232282,48.333999988274,0
-0.6503613386682,48.334095294926,0
-0.65038006628549,48.334189876413,0
-0.6504111227139,48.334283012906,0
-0.65045427165865,48.334373995572,0
-0.65050918478764,48.334462131963,0
-0.6505754442283,48.334546751292,0
-0.65065254574646,48.334627209536,0
-0.65073990258257,48.334702894337,0
-0.65083684991651,48.334773229667,0
-0.65094264992665,48.334837680207,0
-0.6510564974049,48.334895755428,0
-0.65117752588511,48.334947013318,0
-0.65130481423788,48.334991063754,0
-0.6514373936818,48.335027571466,0
-0.65157425515773,48.335056258592,0
-0.65171435700988,48.335076906791,0
-0.65185663291503,48.33508935891,0
-0.65200000000006,48.335093520174,0
-0.65214336708505,48.33508935891,0
-0.65228564299021,48.335076906791,0
-0.65242574484235,48.335056258592,0
-0.65256260631829,48.335027571466,0
-0.65269518576221,48.334991063754,0
-0.65282247411498,48.334947013318,0
-0.65294350259519,48.334895755428,0
-0.65305735007344,48.334837680207,0
-0.65316315008358,48.334773229667,0
-0.65326009741751,48.334702894337,0
-0.65334745425365,48.334627209536,0
-0.65342455577179,48.334546751292,0
-0.65349081521244,48.334462131963,0
-0.65354572834143,48.334373995572,0
-0.65358887728619,48.334283012906,0

-0.65361993371459,48.334189876413,0
-0.65363866133189,48.334095294926,0
-0.65364491767727,48.333999988274,0
-0.65363865520591,48.3339046818,0
-0.65361992164876,48.333810100842,0
-0.65358885964714,48.333716965212,0
-0.65354570566509,48.333625983719,0
-0.65349078818783,48.333537848773,0
-0.65342452522004,48.333453231119,0
-0.65334742110306,48.333372774728,0
-0.65326006267537,48.3332970919,0
-0.65316311480548,48.333226758607,0
-0.6530573153313,48.333162310103,0
-0.6529434694446,48.333104236857,0
-0.65282244356323,48.333052980819,0
-0.65269515873762,48.333008932057,0
-0.65256258364195,48.332972425791,0
-0.6524257272033,48.332943739837,0
-0.65228563092441,48.332923092501,0
-0.65214336095908,48.332910640912,0
-0.65200000000006,48.332906479826,0
-0.65185663904101,48.332910640912,0
-0.65171436907568,48.332923092501,0
-0.65157427279679,48.332943739837,0
-0.65143741635814,48.332972425791,0
-0.65130484126246,48.333008932057,0
-0.65117755643685,48.333052980819,0
-0.65105653055549,48.333104236857,0
-0.65094268466879,48.333162310103,0
-0.65083688519463,48.333226758607,0
-0.65073993732471,48.3332970919,0
-0.65065257889703,48.333372774728,0
-0.65057547478004,48.333453231119,0
-0.65050921181226,48.333537848773,0
-0.65045429433499,48.333625983719,0
-0.65041114035295,48.333716965212,0
-0.65038007835132,48.333810100842,0
-0.65036134479418,48.3339046818,0
-0.65035508232282,48.333999988274,0
-0.65200000000004,48.334,0
</coordinates>
</LinearRing>
</outerBoundaryIs>
</Polygon>
</Placemark>
<Placemark>
<name>251</name>
<styleUrl>#stylehap2</styleUrl>
<Polygon>
<outerBoundaryIs>
<LinearRing>
<coordinates>
-0.65200000000004,48.269,0
-0.65035717556534,48.268999988301,0
-0.65036342395618,48.269095294952,0
-0.6503821277484,48.269189876439,0
-0.65041314466223,48.269283012931,0

-0.65045623870349,48.269373995595,0
-0.65051108195762,48.269462131985,0
-0.65057725708375,48.269546751312,0
-0.65065426048921,48.269627209554,0
-0.65074150616104,48.269702894353,0
-0.65083833012513,48.26977322968,0
-0.65094399549868,48.269837680218,0
-0.65105769809834,48.269895755437,0
-0.65117857256062,48.269947013325,0
-0.65130569892837,48.269991063759,0
-0.65143810965316,48.270027571469,0
-0.65157479696009,48.270056258593,0
-0.65171472051903,48.270076906792,0
-0.65185681536402,48.27008935891,0
-0.65200000000006,48.270093520174,0
-0.65214318463607,48.27008935891,0
-0.65228527948105,48.270076906792,0
-0.65242520304,48.270056258593,0
-0.65256189034693,48.270027571469,0
-0.65269430107171,48.269991063759,0
-0.65282142743947,48.269947013325,0
-0.65294230190175,48.269895755437,0
-0.65305600450141,48.269837680218,0
-0.65316166987496,48.26977322968,0
-0.65325849383904,48.269702894353,0
-0.65334573951088,48.269627209554,0
-0.65342274291633,48.269546751312,0
-0.65348891804246,48.269462131985,0
-0.6535437612966,48.269373995595,0
-0.65358685533786,48.269283012931,0
-0.65361787225169,48.269189876439,0
-0.65363657604391,48.269095294952,0
-0.65364282443474,48.268999988301,0
-0.65363656993969,48.268904681827,0
-0.65361786022871,48.268810100868,0
-0.65358683776145,48.268716965237,0
-0.6535437387008,48.268625983743,0
-0.65348889111383,48.268537848795,0
-0.65342271247308,48.268453231139,0
-0.65334570647802,48.268372774745,0
-0.65325845922026,48.268297091916,0
-0.65316163472212,48.26822675862,0
-0.65305596988262,48.268162310114,0
-0.65294226886887,48.268104236865,0
-0.65282139699622,48.268052980825,0
-0.65269427414308,48.268008932062,0
-0.65256186775113,48.267972425794,0
-0.65242518546359,48.267943739839,0
-0.65228526745807,48.267923092502,0
-0.65214317853185,48.267910640912,0
-0.65200000000006,48.267906479827,0
-0.65185682146824,48.267910640912,0
-0.65171473254201,48.267923092502,0
-0.65157481453649,48.267943739839,0
-0.65143813224896,48.267972425794,0
-0.65130572585701,48.268008932062,0
-0.65117860300387,48.268052980825,0

-0.65105773113122,48.268104236865,0
-0.65094403011746,48.268162310114,0
-0.65083836527797,48.26822675862,0
-0.65074154077983,48.268297091916,0
-0.65065429352207,48.268372774745,0
-0.650577287527,48.268453231139,0
-0.65051110888626,48.268537848795,0
-0.65045626129929,48.268625983743,0
-0.65041316223863,48.268716965237,0
-0.65038213977138,48.268810100868,0
-0.6503634300604,48.268904681827,0
-0.65035717556534,48.268999988301,0
-0.65200000000004,48.269,0
</coordinates>
</LinearRing>
</outerBoundaryIs>
</Polygon>
</Placemark>
<Placemark>
<name>256</name>
<styleUrl>#stylehap2</styleUrl>
<Polygon>
<outerBoundaryIs>
<LinearRing>
<coordinates>
-0.64099999999996,48.192,0
-0.63935964564148,48.191999988332,0
-0.63936588464573,48.192095294984,0
-0.63938456032376,48.192189876469,0
-0.63941553060946,48.192283012961,0
-0.63945855986322,48.192373995623,0
-0.63951332066342,48.192462132011,0
-0.63957939629635,48.192546751336,0
-0.63965628392646,48.192627209575,0
-0.63974339842184,48.192702894371,0
-0.63984007680652,48.192773229696,0
-0.63994558330569,48.192837680231,0
-0.64005911494516,48.192895755447,0
-0.64017980766258,48.192947013333,0
-0.64030674288417,48.192991063765,0
-0.64043895451635,48.193027571473,0
-0.64057543629968,48.193056258596,0
-0.64071514946845,48.193076906793,0
-0.64085703065809,48.19308935891,0
-0.64099999999997,48.193093520174,0
-0.64114296934185,48.19308935891,0
-0.64128485053149,48.193076906793,0
-0.64142456370023,48.193056258596,0
-0.64156104548356,48.193027571473,0
-0.64169325711577,48.192991063765,0
-0.64182019233733,48.192947013333,0
-0.64194088505478,48.192895755447,0
-0.64205441669423,48.192837680231,0
-0.64215992319342,48.192773229696,0
-0.6422566015781,48.192702894371,0
-0.64234371607345,48.192627209575,0
-0.64242060370356,48.192546751336,0

-0.64248667933653,48.192462132011,0
-0.64254144013669,48.192373995623,0
-0.64258446939046,48.192283012961,0
-0.64261543967616,48.192189876469,0
-0.64263411535418,48.192095294984,0
-0.64264035435846,48.191999988332,0
-0.64263410927561,48.191904681858,0
-0.64261542770369,48.191810100899,0
-0.64258445188789,48.191716965267,0
-0.64254141763579,48.191625983771,0
-0.64248665252101,48.191537848821,0
-0.6424205733882,48.191453231162,0
-0.64234368317937,48.191372774767,0
-0.64225656710473,48.191297091935,0
-0.64215988818824,48.191226758636,0
-0.64205438222086,48.191162310127,0
-0.64194085216067,48.191104236876,0
-0.64182016202197,48.191052980833,0
-0.64169323030024,48.191008932068,0
-0.64156102298269,48.190972425797,0
-0.64142454619767,48.190943739841,0
-0.64128483855902,48.190923092503,0
-0.64114296326325,48.190910640912,0
-0.64099999999997,48.190906479826,0
-0.64085703673667,48.190910640912,0
-0.64071516144092,48.190923092503,0
-0.64057545380227,48.190943739841,0
-0.64043897701725,48.190972425797,0
-0.64030676969968,48.191008932068,0
-0.64017983797795,48.191052980833,0
-0.64005914783925,48.191104236876,0
-0.63994561777906,48.191162310127,0
-0.63984011181168,48.191226758636,0
-0.63974343289518,48.191297091935,0
-0.63965631682057,48.191372774767,0
-0.63957942661172,48.191453231162,0
-0.63951334747893,48.191537848821,0
-0.63945858236412,48.191625983771,0
-0.63941554811205,48.191716965267,0
-0.63938457229623,48.191810100899,0
-0.63936589072433,48.191904681858,0
-0.63935964564148,48.191999988332,0
-0.64099999999996,48.192,0
</coordinates>
</LinearRing>
</outerBoundaryIs>
</Polygon>
</Placemark>
<Placemark>
<name>272</name>
<styleUrl>#stylehap2</styleUrl>
<Polygon>
<outerBoundaryIs>
<LinearRing>
<coordinates>
-0.61300000000006,48.225,0
-0.61135858831057,48.224999988319,0

-0.61136483133281,48.22509529497,0
-0.61138351904529,48.225189876456,0
-0.61141450929045,48.225283012948,0
-0.61145756627689,48.225373995611,0
-0.61151236237198,48.225462132,0
-0.61157848059359,48.225546751326,0
-0.61165541778203,48.225627209566,0
-0.61174258842827,48.225702894363,0
-0.6118393291291,48.225773229689,0
-0.6119449036354,48.225837680226,0
-0.61205850845541,48.225895755442,0
-0.61217927896981,48.225947013329,0
-0.61230629601267,48.225991063762,0
-0.61243859286764,48.226027571471,0
-0.61257516262659,48.226056258595,0
-0.61271496585414,48.226076906793,0
-0.61285693850022,48.22608935891,0
-0.61300000000007,48.226093520174,0
-0.61314306149989,48.22608935891,0
-0.61328503414598,48.226076906793,0
-0.61342483737353,48.226056258595,0
-0.61356140713247,48.226027571471,0
-0.61369370398744,48.225991063762,0
-0.6138207210303,48.225947013329,0
-0.6139414915447,48.225895755442,0
-0.61405509636472,48.225837680226,0
-0.61416067087101,48.225773229689,0
-0.61425741157184,48.225702894363,0
-0.61434458221808,48.225627209566,0
-0.61442151940653,48.225546751326,0
-0.61448763762813,48.225462132,0
-0.61454243372322,48.225373995611,0
-0.61458549070967,48.225283012948,0
-0.61461648095482,48.225189876456,0
-0.6146351686673,48.22509529497,0
-0.61464141168954,48.224999988319,0
-0.61463516257774,48.224904681844,0
-0.61461646896075,48.224810100886,0
-0.6145854731755,48.224716965254,0
-0.61454241118174,48.224625983759,0
-0.6144876107642,48.22453784881,0
-0.61442148903645,48.224453231152,0
-0.61434454926461,48.224372774758,0
-0.61425737703623,48.224297091927,0
-0.61416063580265,48.224226758629,0
-0.61405506182911,48.224162310121,0
-0.61394145859123,48.224104236871,0
-0.6138206906602,48.22405298083,0
-0.61369367712354,48.224008932065,0
-0.61356138459096,48.223972425796,0
-0.61342481983936,48.22394373984,0
-0.6132850221519,48.223923092503,0
-0.61314305541032,48.223910640912,0
-0.61300000000007,48.223906479826,0
-0.61285694458979,48.223910640912,0
-0.61271497784821,48.223923092503,0
-0.61257518016076,48.22394373984,0

-0.61243861540915,48.223972425796,0
-0.61230632287657,48.224008932065,0
-0.61217930933991,48.22405298083,0
-0.61205854140889,48.224104236871,0
-0.611944938171,48.224162310121,0
-0.61183936419746,48.224226758629,0
-0.61174262296388,48.224297091927,0
-0.6116554507355,48.224372774758,0
-0.61157851096366,48.224453231152,0
-0.61151238923591,48.22453784881,0
-0.61145758881838,48.224625983759,0
-0.61141452682462,48.224716965254,0
-0.61138353103936,48.224810100886,0
-0.61136483742238,48.224904681844,0
-0.61135858831057,48.224999988319,0
-0.61300000000006,48.225,0
</coordinates>
</LinearRing>
</outerBoundaryIs>
</Polygon>
</Placemark>
<Placemark>
<name>275</name>
<styleUrl>#stylehap2</styleUrl>
<Polygon>
<outerBoundaryIs>
<LinearRing>
<coordinates>
-0.60500000000002,48.313,0
-0.60335575941564,48.312999988282,0
-0.603362013188,48.313095294934,0
-0.60338073309869,48.313189876421,0
-0.60341177674546,48.313283012914,0
-0.60345490793082,48.31337399558,0
-0.60350979845766,48.31346213197,0
-0.60357603062544,48.313546751298,0
-0.60365310040747,48.313627209541,0
-0.60374042128571,48.313702894342,0
-0.60383732871371,48.313773229671,0
-0.60394308517348,48.313837680211,0
-0.60405688578842,48.313895755431,0
-0.60417786444905,48.31394701332,0
-0.60430510040516,48.313991063756,0
-0.60443762527418,48.314027571467,0
-0.60457443041236,48.314056258592,0
-0.6047144745927,48.314076906792,0
-0.60485669193105,48.31408935891,0
-0.60500000000001,48.314093520174,0
-0.60514330806898,48.31408935891,0
-0.60528552540732,48.314076906792,0
-0.60542556958766,48.314056258592,0
-0.60556237472585,48.314027571467,0
-0.60569489959486,48.313991063756,0
-0.60582213555097,48.31394701332,0
-0.60594311421161,48.313895755431,0
-0.60605691482657,48.313837680211,0
-0.60616267128635,48.313773229671,0

-0.60625957871432,48.313702894342,0
-0.60634689959258,48.313627209541,0
-0.60642396937459,48.313546751298,0
-0.60649020154236,48.31346213197,0
-0.60654509206921,48.31337399558,0
-0.60658822325457,48.313283012914,0
-0.60661926690134,48.313189876421,0
-0.60663798681202,48.313095294934,0
-0.60664424058438,48.312999988282,0
-0.60663798069309,48.312904681809,0
-0.60661925484938,48.31281010085,0
-0.60658820563577,48.31271696522,0
-0.60654506941893,48.312625983727,0
-0.60649017454882,48.31253784878,0
-0.60642393885795,48.312453231125,0
-0.60634686648008,48.312372774733,0
-0.60625954401208,48.312297091905,0
-0.60616263604875,48.312226758611,0
-0.60605688012433,48.312162310106,0
-0.60594308109913,48.31210423686,0
-0.60582210503434,48.312052980821,0
-0.60569487260132,48.312008932059,0
-0.60556235207557,48.311972425792,0
-0.60542555196887,48.311943739838,0
-0.60528551335536,48.311923092502,0
-0.60514330195005,48.311910640912,0
-0.60500000000001,48.311906479826,0
-0.60485669805,48.311910640912,0
-0.60471448664466,48.311923092502,0
-0.60457444803116,48.311943739838,0
-0.60443764792446,48.311972425792,0
-0.60430512739873,48.312008932059,0
-0.60417789496571,48.312052980821,0
-0.60405691890092,48.31210423686,0
-0.6039431198757,48.312162310106,0
-0.60383736395127,48.312226758611,0
-0.60374045598795,48.312297091905,0
-0.60365313351995,48.312372774733,0
-0.60357606114207,48.312453231125,0
-0.60350982545123,48.31253784878,0
-0.6034549305811,48.312625983727,0
-0.60341179436426,48.31271696522,0
-0.60338074515065,48.31281010085,0
-0.60336201930696,48.312904681809,0
-0.60335575941564,48.312999988282,0
-0.60500000000002,48.313,0
</coordinates>
</LinearRing>
</outerBoundaryIs>
</Polygon>
</Placemark>
<Placemark>
<name>277</name>
<styleUrl>#stylehap2</styleUrl>
<Polygon>
<outerBoundaryIs>
<LinearRing>

<coordinates>
-0.60300000000007,48.243,0
-0.60135801078019,48.242999988311,0
-0.60136425599711,48.243095294963,0
-0.60138295028298,48.243189876449,0
-0.60141395143029,48.243283012941,0
-0.60145702356475,48.243373995605,0
-0.60151183893848,48.243462131994,0
-0.60157798042264,48.24354675132,0
-0.60165494468061,48.243627209561,0
-0.60174214599734,48.243702894359,0
-0.60183892073618,48.243773229686,0
-0.60194453238902,48.243837680223,0
-0.60205817718138,48.24389575544,0
-0.60217899018967,48.243947013328,0
-0.60230605192456,48.243991063761,0
-0.60243839532952,48.24402757147,0
-0.60257501314209,48.244056258594,0
-0.60271486556115,48.244076906792,0
-0.60285688816214,48.24408935891,0
-0.60300000000007,48.244093520174,0
-0.603143111838,48.24408935891,0
-0.60328513443899,48.244076906792,0
-0.60342498685804,48.244056258594,0
-0.60356160467062,48.24402757147,0
-0.60369394807558,48.243991063761,0
-0.60382100981047,48.243947013328,0
-0.60394182281876,48.24389575544,0
-0.60405546761109,48.243837680223,0
-0.60416107926396,48.243773229686,0
-0.6042578540028,48.243702894359,0
-0.60434505531953,48.243627209561,0
-0.60442201957748,48.24354675132,0
-0.60448816106166,48.243462131994,0
-0.60454297643539,48.243373995605,0
-0.60458604856985,48.243283012941,0
-0.60461704971716,48.243189876449,0
-0.60463574400303,48.243095294963,0
-0.60464198921995,48.242999988311,0
-0.60463573790748,48.242904681837,0
-0.60461703771128,48.242810100878,0
-0.6045860310184,48.242716965247,0
-0.60454295387169,48.242625983752,0
-0.60448813417129,48.242537848804,0
-0.60442198917751,48.242453231147,0
-0.60434502233359,48.242372774753,0
-0.6042578194332,48.242297091922,0
-0.60416104416106,48.242226758626,0
-0.60405543304149,48.242162310118,0
-0.60394178983284,48.242104236869,0
-0.60382097941047,48.242052980828,0
-0.60369392118521,48.242008932064,0
-0.6035615821069,48.241972425795,0
-0.6034249693066,48.24194373984,0
-0.60328512243311,48.241923092503,0
-0.60314310574242,48.241910640912,0
-0.60300000000007,48.241906479826,0

-0.60285689425769,48.241910640912,0
-0.60271487756703,48.241923092503,0
-0.60257503069354,48.24194373984,0
-0.60243841789322,48.241972425795,0
-0.60230607881493,48.242008932064,0
-0.60217902058967,48.242052980828,0
-0.6020582101673,48.242104236869,0
-0.60194456695862,48.242162310118,0
-0.60183895583907,48.242226758626,0
-0.60174218056691,48.242297091922,0
-0.60165497766652,48.242372774753,0
-0.60157801082263,48.242453231147,0
-0.60151186582885,48.242537848804,0
-0.60145704612845,48.242625983752,0
-0.60141396898174,48.242716965247,0
-0.60138296228886,48.242810100878,0
-0.60136426209265,48.242904681837,0
-0.60135801078019,48.242999988311,0
-0.60300000000007,48.243,0
</coordinates>
</LinearRing>
</outerBoundaryIs>
</Polygon>
</Placemark>
<Placemark>
<name>278</name>
<styleUrl>#stylehap2</styleUrl>
<Polygon>
<outerBoundaryIs>
<LinearRing>
<coordinates>
-0.60300000000007,48.243,0
-0.60135801078019,48.242999988311,0
-0.60136425599711,48.243095294963,0
-0.60138295028298,48.243189876449,0
-0.60141395143029,48.243283012941,0
-0.60145702356475,48.243373995605,0
-0.60151183893848,48.243462131994,0
-0.60157798042264,48.24354675132,0
-0.60165494468061,48.243627209561,0
-0.60174214599734,48.243702894359,0
-0.60183892073618,48.243773229686,0
-0.60194453238902,48.243837680223,0
-0.60205817718138,48.24389575544,0
-0.60217899018967,48.243947013328,0
-0.60230605192456,48.243991063761,0
-0.60243839532952,48.24402757147,0
-0.60257501314209,48.244056258594,0
-0.60271486556115,48.244076906792,0
-0.60285688816214,48.24408935891,0
-0.60300000000007,48.244093520174,0
-0.603143111838,48.24408935891,0
-0.60328513443899,48.244076906792,0
-0.60342498685804,48.244056258594,0
-0.60356160467062,48.24402757147,0
-0.60369394807558,48.243991063761,0
-0.60382100981047,48.243947013328,0

-0.60394182281876,48.24389575544,0
-0.60405546761109,48.243837680223,0
-0.60416107926396,48.243773229686,0
-0.6042578540028,48.243702894359,0
-0.60434505531953,48.243627209561,0
-0.60442201957748,48.24354675132,0
-0.60448816106166,48.243462131994,0
-0.60454297643539,48.243373995605,0
-0.60458604856985,48.243283012941,0
-0.60461704971716,48.243189876449,0
-0.60463574400303,48.243095294963,0
-0.60464198921995,48.242999988311,0
-0.60463573790748,48.242904681837,0
-0.60461703771128,48.242810100878,0
-0.6045860310184,48.242716965247,0
-0.60454295387169,48.242625983752,0
-0.60448813417129,48.242537848804,0
-0.60442198917751,48.242453231147,0
-0.60434502233359,48.242372774753,0
-0.6042578194332,48.242297091922,0
-0.60416104416106,48.242226758626,0
-0.60405543304149,48.242162310118,0
-0.60394178983284,48.242104236869,0
-0.60382097941047,48.242052980828,0
-0.60369392118521,48.242008932064,0
-0.6035615821069,48.241972425795,0
-0.6034249693066,48.24194373984,0
-0.60328512243311,48.241923092503,0
-0.60314310574242,48.241910640912,0
-0.60300000000007,48.241906479826,0
-0.60285689425769,48.241910640912,0
-0.60271487756703,48.241923092503,0
-0.60257503069354,48.24194373984,0
-0.60243841789322,48.241972425795,0
-0.60230607881493,48.242008932064,0
-0.60217902058967,48.242052980828,0
-0.6020582101673,48.242104236869,0
-0.60194456695862,48.242162310118,0
-0.60183895583907,48.242226758626,0
-0.60174218056691,48.242297091922,0
-0.60165497766652,48.242372774753,0
-0.60157801082263,48.242453231147,0
-0.60151186582885,48.242537848804,0
-0.60145704612845,48.242625983752,0
-0.60141396898174,48.242716965247,0
-0.60138296228886,48.242810100878,0
-0.60136426209265,48.242904681837,0
-0.60135801078019,48.242999988311,0
-0.60300000000007,48.243,0
</coordinates>
</LinearRing>
</outerBoundaryIs>
</Polygon>
</Placemark>
<Placemark>
<name>280</name>
<styleUrl>#stylehap2</styleUrl>

<Polygon>
<outerBoundaryIs>
<LinearRing>
<coordinates>
-0.60199999999998,48.106,0
-0.60036239217106,48.105999988367,0
-0.60036862073819,48.106095295019,0
-0.60038726515543,48.106189876503,0
-0.60041818359433,48.106283012994,0
-0.60046114080955,48.106373995654,0
-0.60051580992737,48.10646213204,0
-0.60058177493176,48.106546751362,0
-0.60065853382887,48.106627209598,0
-0.60074550246633,48.106702894392,0
-0.60084201897823,48.106773229714,0
-0.60094734882165,48.106837680246,0
-0.60106069036688,48.106895755458,0
-0.6011811809984,48.106947013342,0
-0.60130790368056,48.106991063771,0
-0.60143989393752,48.107027571477,0
-0.60157614719486,48.107056258598,0
-0.60171562642635,48.107076906794,0
-0.60185727004815,48.107089358911,0
-0.60199999999998,48.107093520173,0
-0.60214272995179,48.107089358911,0
-0.60228437357359,48.107076906794,0
-0.60242385280508,48.107056258598,0
-0.60256010606242,48.107027571477,0
-0.60269209631941,48.106991063771,0
-0.60281881900154,48.106947013342,0
-0.60293930963309,48.106895755458,0
-0.60305265117831,48.106837680246,0
-0.60315798102174,48.106773229714,0
-0.60325449753361,48.106702894392,0
-0.60334146617109,48.106627209598,0
-0.60341822506818,48.106546751362,0
-0.60348419007257,48.10646213204,0
-0.60353885919042,48.106373995654,0
-0.60358181640561,48.106283012994,0
-0.60361273484451,48.106189876503,0
-0.60363137926175,48.106095295019,0
-0.60363760782888,48.105999988367,0
-0.60363137321165,48.105904681893,0
-0.60361272292812,48.105810100933,0
-0.60358179898503,48.1057169653,0
-0.60353883679494,48.105625983802,0
-0.60348416338268,48.10553784885,0
-0.60341819489484,48.105453231189,0
-0.6033414334311,48.10537277479,0
-0.60325446322176,48.105297091955,0
-0.60315794618057,48.105226758654,0
-0.60305261686644,48.105162310141,0
-0.60293927689309,48.105104236887,0
-0.60281878882821,48.105052980842,0
-0.60269206962951,48.105008932074,0
-0.60256008366694,48.104972425802,0
-0.60242383538449,48.104943739844,0

-0.60228436165722,48.104923092504,0
-0.60214272390169,48.104910640913,0
-0.60199999999998,48.104906479826,0
-0.60185727609826,48.104910640913,0
-0.60171563834275,48.104923092504,0
-0.60157616461545,48.104943739844,0
-0.601439916333,48.104972425802,0
-0.60130793037043,48.105008932074,0
-0.60118121117173,48.105052980842,0
-0.60106072310687,48.105104236887,0
-0.6009473831335,48.105162310141,0
-0.6008420538194,48.105226758654,0
-0.60074553677818,48.105297091955,0
-0.60065856656884,48.10537277479,0
-0.60058180510512,48.105453231189,0
-0.60051583661727,48.10553784885,0
-0.600461163205,48.105625983802,0
-0.60041820101491,48.1057169653,0
-0.60038727707182,48.105810100933,0
-0.60036862678829,48.105904681893,0
-0.60036239217106,48.105999988367,0
-0.60199999999998,48.106,0
</coordinates>
</LinearRing>
</outerBoundaryIs>
</Polygon>
</Placemark>
<Placemark>
<name>282</name>
<styleUrl>#stylehap2</styleUrl>
<Polygon>
<outerBoundaryIs>
<LinearRing>
<coordinates>
-0.601,48.429,0
-0.59935200953336,48.428999988235,0
-0.59935827755565,48.429095294887,0
-0.59937704014714,48.429189876375,0
-0.59940815458115,48.42928301287,0
-0.5994513841219,48.429373995537,0
-0.59950639982411,48.429462131931,0
-0.59957278303483,48.429546751263,0
-0.59965002857815,48.429627209509,0
-0.5997375485986,48.429702894314,0
-0.59983467703416,48.429773229647,0
-0.59994067468496,48.429837680191,0
-0.60005473483874,48.429895755415,0
-0.60017598941063,48.429947013308,0
-0.60030351555037,48.429991063747,0
-0.60043634266668,48.430027571461,0
-0.60057345981522,48.430056258589,0
-0.60071382339404,48.43007690679,0
-0.60085636508777,48.43008935891,0
-0.601,48.430093520174,0
-0.60114363491225,48.43008935891,0
-0.60128617660596,48.43007690679,0
-0.60142654018477,48.430056258589,0

-0.60156365733331,48.430027571461,0
-0.60169648444962,48.429991063747,0
-0.60182401058936,48.429947013308,0
-0.60194526516126,48.429895755415,0
-0.60205932531503,48.429837680191,0
-0.60216532296584,48.429773229647,0
-0.60226245140139,48.429702894314,0
-0.60234997142184,48.429627209509,0
-0.60242721696517,48.429546751263,0
-0.60249360017588,48.429462131931,0
-0.60254861587809,48.429373995537,0
-0.60259184541884,48.42928301287,0
-0.60262295985285,48.429189876375,0
-0.60264172244434,48.429095294887,0
-0.60264799046663,48.428999988235,0
-0.60264171628638,48.428904681761,0
-0.60262294772405,48.428810100804,0
-0.60259182768771,48.428716965176,0
-0.60254859308341,48.428625983684,0
-0.60249357301023,48.428537848741,0
-0.60242718625396,48.428453231089,0
-0.60234993809823,48.428372774701,0
-0.60226241647791,48.428297091877,0
-0.60216528750359,48.428226758587,0
-0.60205929039155,48.428162310087,0
-0.60194523183765,48.428104236844,0
-0.60182397987818,48.428052980809,0
-0.60169645728397,48.42800893205,0
-0.60156363453861,48.427972425786,0
-0.60142652245364,48.427943739835,0
-0.60128616447715,48.4279230925,0
-0.60114362875429,48.427910640912,0
-0.601,48.427906479826,0
-0.6008563712457,48.427910640912,0
-0.60071383552284,48.4279230925,0
-0.60057347754636,48.427943739835,0
-0.60043636546139,48.427972425786,0
-0.60030354271602,48.42800893205,0
-0.60017602012184,48.428052980809,0
-0.60005476816235,48.428104236844,0
-0.59994070960845,48.428162310087,0
-0.5998347124964,48.428226758587,0
-0.59973758352208,48.428297091877,0
-0.59965006190177,48.428372774701,0
-0.59957281374604,48.428453231089,0
-0.59950642698977,48.428537848741,0
-0.59945140691658,48.428625983684,0
-0.59940817231229,48.428716965176,0
-0.59937705227595,48.428810100804,0
-0.59935828371361,48.428904681761,0
-0.59935200953336,48.428999988235,0
-0.601,48.429,0
</coordinates>
</LinearRing>
</outerBoundaryIs>
</Polygon>
</Placemark>

<Placemark>
<name>284</name>
<styleUrl>#stylehap2</styleUrl>
<Polygon>
<outerBoundaryIs>
<LinearRing>
<coordinates>
-0.59899999999993,48.255,0
-0.59735762544386,48.254999988306,0
-0.59736387212511,48.255095294958,0
-0.59738257079684,48.255189876444,0
-0.59741357921823,48.255283012937,0
-0.59745666145963,48.255373995601,0
-0.59751148969633,48.25546213199,0
-0.59757764670162,48.255546751316,0
-0.59765462902072,48.255627209557,0
-0.59774185080121,48.255702894356,0
-0.5978386482507,48.255773229683,0
-0.59794428468822,48.255837680221,0
-0.59805795615065,48.255895755438,0
-0.59817879751148,48.255947013326,0
-0.59830588906549,48.25599106376,0
-0.59843826352925,48.25602757147,0
-0.59857491340385,48.256056258594,0
-0.59871479864414,48.256076906792,0
-0.59885685457575,48.25608935891,0
-0.59899999999993,48.256093520174,0
-0.59914314542413,48.25608935891,0
-0.59928520135574,48.256076906792,0
-0.599425086596,48.256056258594,0
-0.59956173647062,48.25602757147,0
-0.59969411093436,48.25599106376,0
-0.5998212024884,48.255947013326,0
-0.5999420438492,48.255895755438,0
-0.60005571531164,48.255837680221,0
-0.60016135174916,48.255773229683,0
-0.60025814919867,48.255702894356,0
-0.60034537097914,48.255627209557,0
-0.60042235329826,48.255546751316,0
-0.60048851030355,48.25546213199,0
-0.60054333854022,48.255373995601,0
-0.60058642078165,48.255283012937,0
-0.60061742920302,48.255189876444,0
-0.60063612787474,48.255095294958,0
-0.60064237455599,48.254999988306,0
-0.6006361217752,48.254904681832,0
-0.60061741718925,48.254810100874,0
-0.60058640321868,48.254716965243,0
-0.60054331596172,48.254625983748,0
-0.60048848339552,48.2545378488,0
-0.60042232287831,48.254453231143,0
-0.60034533797157,48.254372774749,0
-0.60025811460637,48.254297091919,0
-0.60016131662324,48.254226758623,0
-0.60005568071934,48.254162310116,0
-0.59994201084163,48.254104236867,0
-0.59982117206843,48.254052980827,0

-0.59969408402636,48.254008932063,0
-0.5995617138921,48.253972425794,0
-0.59942506903305,48.25394373984,0
-0.59928518934194,48.253923092502,0
-0.59914313932456,48.253910640912,0
-0.59899999999993,48.253906479826,0
-0.59885686067529,48.253910640912,0
-0.59871481065791,48.253923092502,0
-0.59857493096683,48.25394373984,0
-0.59843828610776,48.253972425794,0
-0.59830591597352,48.254008932063,0
-0.59817882793142,48.254052980827,0
-0.59805798915825,48.254104236867,0
-0.59794431928052,48.254162310116,0
-0.59783868337664,48.254226758623,0
-0.59774188539351,48.254297091919,0
-0.59765466202831,48.254372774749,0
-0.59757767712157,48.254453231143,0
-0.59751151660436,48.2545378488,0
-0.59745668403816,48.254625983748,0
-0.5974135967812,48.254716965243,0
-0.59738258281061,48.254810100874,0
-0.59736387822468,48.254904681832,0
-0.59735762544386,48.254999988306,0
-0.59899999999993,48.255,0
</coordinates>
</LinearRing>
</outerBoundaryIs>
</Polygon>
</Placemark>
<Placemark>
<name>287</name>
<styleUrl>#stylehap2</styleUrl>
<Polygon>
<outerBoundaryIs>
<LinearRing>
<coordinates>
-0.59699999999998,48.205,0
-0.59535922934477,48.204999988327,0
-0.59536546993103,48.205095294978,0
-0.59538415034729,48.205189876464,0
-0.59541512849151,48.205283012956,0
-0.59545816866432,48.205373995619,0
-0.59551294336094,48.205462132006,0
-0.59557903576207,48.205546751332,0
-0.5956559429045,48.205627209571,0
-0.59574307950781,48.205702894368,0
-0.59583978242787,48.205773229693,0
-0.59594531570309,48.205837680229,0
-0.59605887615551,48.205895755445,0
-0.59617959950348,48.205947013331,0
-0.59630656694007,48.205991063764,0
-0.59643881212652,48.206027571472,0
-0.59657532854797,48.206056258595,0
-0.59671507717504,48.206076906793,0
-0.5968569943733,48.20608935891,0
-0.59699999999998,48.206093520173,0

-0.59714300562667,48.20608935891,0
-0.59728492282493,48.206076906793,0
-0.59742467145199,48.206056258595,0
-0.59756118787344,48.206027571472,0
-0.5976934330599,48.205991063764,0
-0.59782040049648,48.205947013331,0
-0.59794112384445,48.205895755445,0
-0.59805468429685,48.205837680229,0
-0.59816021757209,48.205773229693,0
-0.59825692049213,48.205702894368,0
-0.59834405709547,48.205627209571,0
-0.59842096423787,48.205546751332,0
-0.59848705663902,48.205462132006,0
-0.59854183133564,48.205373995619,0
-0.59858487150846,48.205283012956,0
-0.59861584965267,48.205189876464,0
-0.59863453006893,48.205095294978,0
-0.59864077065517,48.204999988327,0
-0.59863452398603,48.204904681853,0
-0.59861583767168,48.204810100893,0
-0.59858485399342,48.204716965262,0
-0.59854180881877,48.204625983766,0
-0.59848702980445,48.204537848817,0
-0.59842093390098,48.204453231158,0
-0.598344024178,48.204372774763,0
-0.59825688599428,48.204297091932,0
-0.59816018254205,48.204226758633,0
-0.598054649799,48.204162310125,0
-0.59794109092698,48.204104236874,0
-0.59782037015959,48.204052980832,0
-0.59769340622533,48.204008932067,0
-0.59756116535657,48.203972425797,0
-0.59742465393696,48.203943739841,0
-0.59728491084393,48.203923092503,0
-0.59714299954377,48.203910640912,0
-0.59699999999998,48.203906479826,0
-0.5968570004562,48.203910640912,0
-0.59671508915601,48.203923092503,0
-0.59657534606298,48.203943739841,0
-0.5964388346434,48.203972425797,0
-0.59630659377464,48.204008932067,0
-0.59617962984038,48.204052980832,0
-0.59605890907298,48.204104236874,0
-0.59594535020096,48.204162310125,0
-0.59583981745792,48.204226758633,0
-0.59574311400569,48.204297091932,0
-0.59565597582197,48.204372774763,0
-0.59557906609899,48.204453231158,0
-0.59551297019551,48.204537848817,0
-0.5954581911812,48.204625983766,0
-0.59541514600654,48.204716965262,0
-0.59538416232826,48.204810100893,0
-0.59536547601393,48.204904681853,0
-0.59535922934477,48.204999988327,0
-0.59699999999998,48.205,0
</coordinates>
</LinearRing>

</outerBoundaryIs>
</Polygon>
</Placemark>
<Placemark>
<name>294</name>
<styleUrl>#stylehap2</styleUrl>
<Polygon>
<outerBoundaryIs>
<LinearRing>
<coordinates>
-0.58899999999994,48.311,0
-0.58735582386001,48.310999988283,0
-0.58736207738747,48.311095294935,0
-0.58738079656466,48.311189876422,0
-0.58741183899491,48.311283012915,0
-0.58745496848994,48.31137399558,0
-0.58750985686559,48.311462131971,0
-0.58757608643758,48.311546751299,0
-0.58765315319899,48.311627209542,0
-0.58774047065485,48.311702894343,0
-0.58783737428466,48.311773229672,0
-0.58794312659938,48.311837680211,0
-0.58805692275399,48.311895755431,0
-0.58817789667287,48.311947013321,0
-0.58830512764197,48.311991063756,0
-0.58843764731666,48.312027571467,0
-0.58857444709271,48.312056258592,0
-0.58871448578395,48.312076906792,0
-0.58885669754803,48.31208935891,0
-0.58899999999995,48.312093520174,0
-0.58914330245185,48.31208935891,0
-0.58928551421594,48.312076906792,0
-0.58942555290717,48.312056258592,0
-0.58956235268322,48.312027571467,0
-0.58969487235791,48.311991063756,0
-0.58982210332701,48.311947013321,0
-0.58994307724592,48.311895755431,0
-0.5900568734005,48.311837680211,0
-0.59016262571522,48.311773229672,0
-0.59025952934503,48.311702894343,0
-0.59034684680089,48.311627209542,0
-0.59042391356233,48.311546751299,0
-0.59049014313432,48.311462131971,0
-0.59054503150994,48.31137399558,0
-0.59058816100497,48.311283012915,0
-0.59061920343522,48.311189876422,0
-0.59063792261242,48.311095294935,0
-0.59064417613987,48.310999988283,0
-0.59063791649412,48.310904681809,0
-0.59061919138458,48.310810100851,0
-0.59058814338811,48.310716965221,0
-0.59054500886213,48.310625983727,0
-0.5904901161437,48.310537848781,0
-0.59042388304903,48.310453231126,0
-0.59034681369202,48.310372774734,0
-0.5902594946466,48.310297091906,0
-0.59016259048152,48.310226758612,0

-0.59005683870205,48.310162310107,0
-0.58994304413703,48.31010423686,0
-0.58982207281371,48.310052980821,0
-0.58969484536731,48.310008932059,0
-0.58956233003543,48.309972425792,0
-0.58942553529031,48.309943739838,0
-0.5892855021653,48.309923092502,0
-0.58914329633359,48.309910640912,0
-0.58899999999995,48.309906479826,0
-0.58885670366629,48.309910640912,0
-0.58871449783461,48.309923092502,0
-0.58857446470957,48.309943739838,0
-0.58843766996445,48.309972425792,0
-0.58830515463257,48.310008932059,0
-0.58817792718617,48.310052980821,0
-0.58805695586285,48.31010423686,0
-0.58794316129783,48.310162310107,0
-0.58783740951838,48.310226758612,0
-0.58774050535328,48.310297091906,0
-0.58765318630786,48.310372774734,0
-0.58757611695085,48.310453231126,0
-0.58750988385618,48.310537848781,0
-0.58745499113776,48.310625983727,0
-0.58741185661178,48.310716965221,0
-0.58738080861533,48.310810100851,0
-0.58736208350576,48.310904681809,0
-0.58735582386001,48.310999988283,0
-0.58899999999994,48.311,0
</coordinates>
</LinearRing>
</outerBoundaryIs>
</Polygon>
</Placemark>
<Placemark>
<name>297</name>
<styleUrl>#stylehap2</styleUrl>
<Polygon>
<outerBoundaryIs>
<LinearRing>
<coordinates>
-0.58600000000001,48.23,0
-0.58435842794241,48.229999988317,0
-0.58436467157409,48.230095294968,0
-0.58438336111185,48.230189876454,0
-0.58441435438431,48.230283012946,0
-0.58445741557703,48.23037399561,0
-0.5845122170254,48.230462131998,0
-0.58457834170653,48.230546751324,0
-0.58465528641162,48.230627209564,0
-0.58474246557442,48.230702894362,0
-0.58483921572689,48.230773229688,0
-0.584944800548,48.230837680225,0
-0.58505841646749,48.230895755442,0
-0.58517919878157,48.230947013329,0
-0.58530622823447,48.230991063762,0
-0.58543853801539,48.231027571471,0
-0.58557512111781,48.231056258594,0

-0.58571493800482,48.231076906793,0
-0.58585692452235,48.23108935891,0
-0.58600000000002,48.231093520174,0
-0.58614307547767,48.23108935891,0
-0.5862850619952,48.231076906793,0
-0.58642487888221,48.231056258594,0
-0.58656146198463,48.231027571471,0
-0.58669377176555,48.230991063762,0
-0.58682080121845,48.230947013329,0
-0.58694158353253,48.230895755442,0
-0.58705519945202,48.230837680225,0
-0.58716078427314,48.230773229688,0
-0.58725753442561,48.230702894362,0
-0.5873447135884,48.230627209564,0
-0.58742165829349,48.230546751324,0
-0.58748778297463,48.230462131998,0
-0.58754258442299,48.23037399561,0
-0.58758564561571,48.230283012946,0
-0.58761663888817,48.230189876454,0
-0.58763532842593,48.230095294968,0
-0.58764157205762,48.229999988317,0
-0.58763532233471,48.229904681842,0
-0.58761662689082,48.229810100884,0
-0.58758562807676,48.229716965252,0
-0.58754256187533,48.229625983757,0
-0.58748775610337,48.229537848808,0
-0.5874216279151,48.229453231151,0
-0.58734468062592,48.229372774756,0
-0.58725749988059,48.229297091926,0
-0.58716074919521,48.229226758628,0
-0.58705516490697,48.22916231012,0
-0.58694155057005,48.229104236871,0
-0.58682077084006,48.229052980829,0
-0.58669374489429,48.229008932065,0
-0.58656143943696,48.228972425796,0
-0.58642486134324,48.22894373984,0
-0.58628504999784,48.228923092503,0
-0.58614306938645,48.228910640912,0
-0.58600000000002,48.228906479826,0
-0.58585693061357,48.228910640912,0
-0.58571495000218,48.228923092503,0
-0.58557513865679,48.22894373984,0
-0.58543856056306,48.228972425796,0
-0.58530625510573,48.229008932065,0
-0.58517922915997,48.229052980829,0
-0.58505844942997,48.229104236871,0
-0.58494483509305,48.22916231012,0
-0.58483925080482,48.229226758628,0
-0.58474250011944,48.229297091926,0
-0.5846553193741,48.229372774756,0
-0.58457837208492,48.229453231151,0
-0.58451224389668,48.229537848808,0
-0.58445743812469,48.229625983757,0
-0.58441437192326,48.229716965252,0
-0.58438337310921,48.229810100884,0
-0.58436467766531,48.229904681842,0
-0.58435842794241,48.229999988317,0

-0.58600000000001,48.23,0
</coordinates>
</LinearRing>
</outerBoundaryIs>
</Polygon>
</Placemark>
<Placemark>
<name>298</name>
<styleUrl>#stylehap2</styleUrl>
<Polygon>
<outerBoundaryIs>
<LinearRing>
<coordinates>
-0.58600000000001,48.236,0
-0.58435823544282,48.235999988314,0
-0.584364479806,48.236095294966,0
-0.58438317153479,48.236189876452,0
-0.58441416844109,48.236283012944,0
-0.58445723468288,48.236373995607,0
-0.5845120425571,48.236462131996,0
-0.58457817499201,48.236546751322,0
-0.58465512871977,48.236627209563,0
-0.5847423181055,48.236702894361,0
-0.58483907960337,48.236773229687,0
-0.58494467680598,48.236837680224,0
-0.58505830604886,48.236895755441,0
-0.58517910252679,48.236947013328,0
-0.58530614687621,48.236991063761,0
-0.58543847217295,48.237027571471,0
-0.58557507129239,48.237056258594,0
-0.58571490457564,48.237076906793,0
-0.58585690774391,48.23708935891,0
-0.58600000000002,48.237093520174,0
-0.58614309225612,48.23708935891,0
-0.58628509542438,48.237076906793,0
-0.58642492870764,48.237056258594,0
-0.58656152782707,48.237027571471,0
-0.58669385312381,48.236991063761,0
-0.58682089747323,48.236947013328,0
-0.58694169395116,48.236895755441,0
-0.58705532319405,48.236837680224,0
-0.58716092039668,48.236773229687,0
-0.58725768189452,48.236702894361,0
-0.58734487128025,48.236627209563,0
-0.58742182500802,48.236546751322,0
-0.58748795744291,48.236462131996,0
-0.58754276531714,48.236373995607,0
-0.58758583155893,48.236283012944,0
-0.58761682846524,48.236189876452,0
-0.58763552019402,48.236095294966,0
-0.5876417645572,48.235999988314,0
-0.58763551410081,48.23590468184,0
-0.58761681646394,48.235810100881,0
-0.5875858140142,48.23571696525,0
-0.58754274276207,48.235625983755,0
-0.58748793056282,48.235537848806,0
-0.58742179461965,48.235453231149,0

-0.58734483830696,48.235372774755,0
-0.58725764733815,48.235297091924,0
-0.58716088530722,48.235226758627,0
-0.58705528863768,48.235162310119,0
-0.58694166097787,48.23510423687,0
-0.58682086708489,48.235052980829,0
-0.58669382624375,48.235008932064,0
-0.586561505272,48.234972425795,0
-0.58642491116291,48.23494373984,0
-0.58628508342308,48.234923092503,0
-0.58614308616291,48.234910640912,0
-0.58600000000002,48.234906479826,0
-0.58585691383711,48.234910640912,0
-0.58571491657694,48.234923092503,0
-0.58557508883711,48.23494373984,0
-0.58543849472802,48.234972425795,0
-0.58530617375628,48.235008932064,0
-0.58517913291514,48.235052980829,0
-0.58505833902216,48.23510423687,0
-0.58494471136235,48.235162310119,0
-0.5848391146928,48.235226758627,0
-0.58474235266187,48.235297091924,0
-0.58465516169306,48.235372774755,0
-0.58457820538037,48.235453231149,0
-0.5845120694372,48.235537848806,0
-0.58445725723795,48.235625983755,0
-0.58441418598582,48.23571696525,0
-0.58438318353609,48.235810100881,0
-0.58436448589921,48.23590468184,0
-0.58435823544282,48.235999988314,0
-0.58600000000001,48.236,0
</coordinates>
</LinearRing>
</outerBoundaryIs>
</Polygon>
</Placemark>
<Placemark>
<name>300</name>
<styleUrl>#stylehap2</styleUrl>
<Polygon>
<outerBoundaryIs>
<LinearRing>
<coordinates>
-0.58500000000004,47.886,0
-0.5833693598348,47.885999988457,0
-0.58337556192391,47.886095295107,0
-0.58339412703571,47.88618987659,0
-0.58342491394447,47.886283013077,0
-0.58346768840517,47.886373995733,0
-0.5835221249343,47.886462132113,0
-0.58358780928534,47.886546751429,0
-0.58366424159992,47.886627209658,0
-0.58375084021083,47.886702894445,0
-0.58384694606808,47.886773229758,0
-0.583951827754,47.886837680283,0
-0.58406468704969,47.886895755488,0
-0.58418466501006,47.886947013364,0

-0.58431084850152,47.886991063787,0
-0.58444227715234,47.887027571487,0
-0.58457795066278,47.887056258604,0
-0.58471683641951,47.887076906797,0
-0.58485787735617,47.887089358911,0
-0.58500000000004,47.887093520174,0
-0.58514212264391,47.887089358911,0
-0.58528316358057,47.887076906797,0
-0.5854220493373,47.887056258604,0
-0.58555772284774,47.887027571487,0
-0.58568915149854,47.886991063787,0
-0.58581533499002,47.886947013364,0
-0.58593531295039,47.886895755488,0
-0.58604817224608,47.886837680283,0
-0.586153053932,47.886773229758,0
-0.58624915978925,47.886702894445,0
-0.58633575840016,47.886627209658,0
-0.58641219071474,47.886546751429,0
-0.58647787506578,47.886462132113,0
-0.58653231159491,47.886373995733,0
-0.58657508605561,47.886283013077,0
-0.58660587296437,47.88618987659,0
-0.58662443807617,47.886095295107,0
-0.58663064016528,47.885999988457,0
-0.58662443209812,47.885904681982,0
-0.58660586118996,47.88581010102,0
-0.58657506884258,47.885716965383,0
-0.58653228946627,47.885625983881,0
-0.58647784869387,47.885537848924,0
-0.58641216090088,47.885453231256,0
-0.58633572605023,47.88537277485,0
-0.58624912588617,47.885297092008,0
-0.58615301950593,47.885226758698,0
-0.58604813834302,47.885162310178,0
-0.58593528060046,47.885104236917,0
-0.58581530517616,47.885052980864,0
-0.58568912512665,47.88500893209,0
-0.5855577007191,47.884972425812,0
-0.58542203212427,47.88494373985,0
-0.58528315180616,47.884923092507,0
-0.58514211666589,47.884910640913,0
-0.58500000000004,47.884906479826,0
-0.58485788333419,47.884910640913,0
-0.58471684819392,47.884923092507,0
-0.5845779678758,47.88494373985,0
-0.58444229928098,47.884972425812,0
-0.58431087487343,47.88500893209,0
-0.58418469482392,47.885052980864,0
-0.58406471939962,47.885104236917,0
-0.58395186165706,47.885162310178,0
-0.58384698049415,47.885226758698,0
-0.58375087411389,47.885297092008,0
-0.58366427394982,47.88537277485,0
-0.58358783909918,47.885453231256,0
-0.58352215130618,47.885537848924,0
-0.58346771053381,47.885625983881,0
-0.5834249311575,47.885716965383,0

-0.58339413881012,47.88581010102,0
-0.58337556790193,47.885904681982,0
-0.5833693598348,47.885999988457,0
-0.58500000000004,47.886,0
</coordinates>
</LinearRing>
</outerBoundaryIs>
</Polygon>
</Placemark>
<Placemark>
<name>302</name>
<styleUrl>#stylehap2</styleUrl>
<Polygon>
<outerBoundaryIs>
<LinearRing>
<coordinates>
-0.58500000000004,48.215,0
-0.58335890891529,48.214999988323,0
-0.5833651507192,48.215095294974,0
-0.58338383478256,48.21518987646,0
-0.58341481897561,48.215283012952,0
-0.58345786755292,48.215373995615,0
-0.58351265294588,48.215462132003,0
-0.58357875825372,48.215546751329,0
-0.58365568041503,48.215627209569,0
-0.58374283403518,48.215702894366,0
-0.58383955584043,48.215773229691,0
-0.58394510972559,48.215837680227,0
-0.58405869235572,48.215895755444,0
-0.58417943928045,48.21594701333,0
-0.5843064315134,48.215991063763,0
-0.58443870252706,48.216027571472,0
-0.58457524560998,48.216056258595,0
-0.58471502152982,48.216076906793,0
-0.58485696644442,48.21608935891,0
-0.58500000000004,48.216093520174,0
-0.58514303355566,48.21608935891,0
-0.58528497847026,48.216076906793,0
-0.5854247543901,48.216056258595,0
-0.585561297473,48.216027571472,0
-0.58569356848668,48.215991063763,0
-0.5858205607196,48.21594701333,0
-0.58594130764436,48.215895755444,0
-0.58605489027449,48.215837680227,0
-0.58616044415962,48.215773229691,0
-0.5862571659649,48.215702894366,0
-0.58634431958505,48.215627209569,0
-0.58642124174636,48.215546751329,0
-0.5864873470542,48.215462132003,0
-0.58654213244713,48.215373995615,0
-0.58658518102447,48.215283012952,0
-0.58661616521749,48.21518987646,0
-0.58663484928088,48.215095294974,0
-0.58664109108479,48.214999988323,0
-0.58663484319465,48.214904681849,0
-0.58661615322998,48.21481010089,0
-0.58658516349987,48.214716965258,0

-0.58654210991796,48.214625983762,0
-0.58648732020495,48.214537848814,0
-0.58642121139288,48.214453231155,0
-0.58634428664956,48.21437277476,0
-0.58625713144818,48.214297091929,0
-0.58616040911044,48.214226758631,0
-0.58605485575779,48.214162310123,0
-0.58594127470888,48.214104236873,0
-0.58582053036612,48.214052980831,0
-0.58569354163743,48.214008932066,0
-0.58556127494383,48.213972425796,0
-0.5854247368655,48.213943739841,0
-0.58528496648273,48.213923092503,0
-0.58514302746943,48.213910640912,0
-0.58500000000004,48.213906479827,0
-0.58485697253063,48.213910640912,0
-0.58471503351735,48.213923092503,0
-0.58457526313455,48.213943739841,0
-0.58443872505625,48.213972425796,0
-0.58430645836265,48.214008932066,0
-0.58417946963396,48.214052980831,0
-0.5840587252912,48.214104236873,0
-0.58394514424229,48.214162310123,0
-0.58383959088964,48.214226758631,0
-0.58374286855188,48.214297091929,0
-0.58365571335052,48.21437277476,0
-0.5835787886072,48.214453231155,0
-0.58351267979513,48.214537848814,0
-0.58345789008212,48.214625983762,0
-0.58341483650021,48.214716965258,0
-0.5833838467701,48.21481010089,0
-0.58336515680543,48.214904681849,0
-0.58335890891529,48.214999988323,0
-0.58500000000004,48.215,0
</coordinates>
</LinearRing>
</outerBoundaryIs>
</Polygon>
</Placemark>
<Placemark>
<name>304</name>
<styleUrl>#stylehap2</styleUrl>
<Polygon>
<outerBoundaryIs>
<LinearRing>
<coordinates>
-0.58400000000006,48.214,0
-0.58235894096614,48.213999988323,0
-0.58236518264827,48.214095294975,0
-0.58238386634683,48.214189876461,0
-0.58241484993484,48.214283012952,0
-0.58245789767149,48.214373995615,0
-0.58251268199456,48.214462132003,0
-0.5825787860114,48.214546751329,0
-0.58265570667048,48.214627209569,0
-0.58274285858851,48.214702894366,0
-0.58283957850477,48.214773229691,0

-0.58294513032842,48.214837680228,0
-0.58305871074025,48.214895755444,0
-0.58317945530672,48.214947013331,0
-0.58330644505943,48.214991063763,0
-0.58343871348973,48.215027571472,0
-0.58357525390585,48.215056258595,0
-0.58371502709573,48.215076906793,0
-0.583856969238,48.21508935891,0
-0.58400000000006,48.215093520174,0
-0.58414303076211,48.21508935891,0
-0.58428497290438,48.215076906793,0
-0.58442474609429,48.215056258595,0
-0.58456128651038,48.215027571472,0
-0.58469355494068,48.214991063763,0
-0.58482054469339,48.214947013331,0
-0.58494128925988,48.214895755444,0
-0.58505486967169,48.214837680228,0
-0.58516042149534,48.214773229691,0
-0.58525714141162,48.214702894366,0
-0.58534429332966,48.214627209569,0
-0.58542121398871,48.214546751329,0
-0.58548731800558,48.214462132003,0
-0.58554210232862,48.214373995615,0
-0.58558515006527,48.214283012952,0
-0.58561613365328,48.214189876461,0
-0.58563481735187,48.214095294975,0
-0.58564105903397,48.213999988323,0
-0.58563481126597,48.213904681849,0
-0.5856161216664,48.21381010089,0
-0.58558513254164,48.213716965258,0
-0.58554207980067,48.213625983763,0
-0.5854872911578,48.213537848814,0
-0.58542118363688,48.213453231156,0
-0.58534426039598,48.213372774761,0
-0.58525710689678,48.213297091929,0
-0.58516038644807,48.213226758632,0
-0.58505483515687,48.213162310123,0
-0.5849412563262,48.213104236873,0
-0.58482051434156,48.213052980831,0
-0.58469352809291,48.213008932066,0
-0.58456126398243,48.212972425796,0
-0.58442472857065,48.212943739841,0
-0.58428496091751,48.212923092503,0
-0.5841430246762,48.212910640912,0
-0.58400000000006,48.212906479827,0
-0.58385697532391,48.212910640912,0
-0.5837150390826,48.212923092503,0
-0.58357527142948,48.212943739841,0
-0.5834387360177,48.212972425796,0
-0.5833064719072,48.213008932066,0
-0.58317948565858,48.213052980831,0
-0.58305874367391,48.213104236873,0
-0.58294516484327,48.213162310123,0
-0.58283961355207,48.213226758632,0
-0.58274289310333,48.213297091929,0
-0.58265573960413,48.213372774761,0
-0.58257881636323,48.213453231156,0

-0.58251270884233,48.213537848814,0
-0.58245792019946,48.213625983763,0
-0.58241486745847,48.213716965258,0
-0.58238387833371,48.21381010089,0
-0.58236518873414,48.213904681849,0
-0.58235894096614,48.213999988323,0
-0.58400000000006,48.214,0
</coordinates>
</LinearRing>
</outerBoundaryIs>
</Polygon>
</Placemark>
<Placemark>
<name>306</name>
<styleUrl>#stylehap2</styleUrl>
<Polygon>
<outerBoundaryIs>
<LinearRing>
<coordinates>
-0.58299999999997,48.208,0
-0.58135913323429,48.207999988326,0
-0.58136537418576,48.208095294977,0
-0.58138405569596,48.208189876463,0
-0.58141503565446,48.208283012955,0
-0.58145807834816,48.208373995618,0
-0.58151285625307,48.208462132006,0
-0.58157895252546,48.208546751331,0
-0.58165586417271,48.20862720957,0
-0.58174300588009,48.208702894368,0
-0.58183971446462,48.208773229693,0
-0.58194525392164,48.208837680229,0
-0.58205882102608,48.208895755445,0
-0.58217955144575,48.208947013331,0
-0.58230652631982,48.208991063763,0
-0.58243877925296,48.209027571472,0
-0.58257530367129,48.209056258595,0
-0.58271506048465,48.209076906793,0
-0.58285698599621,48.20908935891,0
-0.58299999999997,48.209093520174,0
-0.58314301400373,48.20908935891,0
-0.58328493951529,48.209076906793,0
-0.58342469632865,48.209056258595,0
-0.58356122074698,48.209027571472,0
-0.58369347368012,48.208991063763,0
-0.58382044855419,48.208947013331,0
-0.58394117897386,48.208895755445,0
-0.5840547460783,48.208837680229,0
-0.58416028553532,48.208773229693,0
-0.58425699411984,48.208702894368,0
-0.58434413582723,48.20862720957,0
-0.58442104747448,48.208546751331,0
-0.58448714374687,48.208462132006,0
-0.58454192165178,48.208373995618,0
-0.58458496434548,48.208283012955,0
-0.58461594430398,48.208189876463,0
-0.58463462581418,48.208095294977,0
-0.58464086676564,48.207999988326,0

-0.58463461973028,48.207904681851,0
-0.58461593232103,48.207810100892,0
-0.58458494682757,48.207716965261,0
-0.58454189913121,48.207625983765,0
-0.5844871169079,48.207537848816,0
-0.58442101713259,48.207453231158,0
-0.58434410290437,48.207372774762,0
-0.58425695961635,48.207297091931,0
-0.58416025049955,48.207226758633,0
-0.5840547115748,48.207162310124,0
-0.58394114605097,48.207104236874,0
-0.58382041821231,48.207052980832,0
-0.58369344684115,48.207008932066,0
-0.58356119822642,48.206972425797,0
-0.58342467881074,48.206943739841,0
-0.58328492753236,48.206923092503,0
-0.58314300791984,48.206910640912,0
-0.58299999999997,48.206906479827,0
-0.5828569920801,48.206910640912,0
-0.58271507246758,48.206923092503,0
-0.5825753211892,48.206943739841,0
-0.58243880177352,48.206972425797,0
-0.58230655315879,48.207008932066,0
-0.58217958178763,48.207052980832,0
-0.58205885394897,48.207104236874,0
-0.58194528842516,48.207162310124,0
-0.58183974950039,48.207226758633,0
-0.58174304038359,48.207297091931,0
-0.58165589709557,48.207372774762,0
-0.58157898286734,48.207453231158,0
-0.58151288309204,48.207537848816,0
-0.58145810086873,48.207625983765,0
-0.58141505317237,48.207716965261,0
-0.58138406767891,48.207810100892,0
-0.58136538026965,48.207904681851,0
-0.58135913323429,48.207999988326,0
-0.58299999999997,48.208,0
</coordinates>
</LinearRing>
</outerBoundaryIs>
</Polygon>
</Placemark>
<Placemark>
<name>308</name>
<styleUrl>#stylehap2</styleUrl>
<Polygon>
<outerBoundaryIs>
<LinearRing>
<coordinates>
-0.58199999999999,48.209,0
-0.58035910119399,48.208999988325,0
-0.58036534226721,48.209095294977,0
-0.5803840241421,48.209189876463,0
-0.58041500470544,48.209283012954,0
-0.58045804823949,48.209373995617,0
-0.58051282721396,48.209462132005,0
-0.58057892477692,48.209546751331,0

-0.58065583792592,48.20962720957,0
-0.58074298133485,48.209702894367,0
-0.58083969180774,48.209773229693,0
-0.58094523332558,48.209837680228,0
-0.58105880264763,48.209895755445,0
-0.58117953542477,48.209947013331,0
-0.58130651277828,48.209991063763,0
-0.58143876829392,48.210027571472,0
-0.5815752953782,48.210056258595,0
-0.5817150549206,48.210076906793,0
-0.58185698320357,48.21008935891,0
-0.58199999999998,48.210093520174,0
-0.58214301679642,48.21008935891,0
-0.58228494507939,48.210076906793,0
-0.5824247046218,48.210056258595,0
-0.58256123170607,48.210027571472,0
-0.58269348722172,48.209991063763,0
-0.58282046457522,48.209947013331,0
-0.58294119735236,48.209895755445,0
-0.58305476667439,48.209837680228,0
-0.58316030819223,48.209773229693,0
-0.58325701866514,48.209702894367,0
-0.58334416207408,48.20962720957,0
-0.58342107522307,48.209546751331,0
-0.58348717278604,48.209462132005,0
-0.5835419517605,48.209373995617,0
-0.58358499529455,48.209283012954,0
-0.5836159758579,48.209189876463,0
-0.58363465773276,48.209095294977,0
-0.58364089880601,48.208999988325,0
-0.58363465164854,48.208904681851,0
-0.58361596387431,48.208810100892,0
-0.5835849777757,48.20871696526,0
-0.58354192923869,48.208625983764,0
-0.58348714594559,48.208537848816,0
-0.58342104487951,48.208453231157,0
-0.58334412914941,48.208372774762,0
-0.58325698415974,48.208297091931,0
-0.58316027315453,48.208226758633,0
-0.58305473216901,48.208162310124,0
-0.5829411644277,48.208104236874,0
-0.58282043423169,48.208052980832,0
-0.58269346038127,48.208008932066,0
-0.58256120918428,48.207972425797,0
-0.58242468710294,48.207943739841,0
-0.5822849330958,48.207923092503,0
-0.5821430107122,48.207910640912,0
-0.58199999999998,48.207906479826,0
-0.58185698928779,48.207910640912,0
-0.58171506690419,48.207923092503,0
-0.58157531289705,48.207943739841,0
-0.58143879081571,48.207972425797,0
-0.5813065396187,48.208008932066,0
-0.58117956576831,48.208052980832,0
-0.5810588355723,48.208104236874,0
-0.58094526783098,48.208162310124,0
-0.58083972684544,48.208226758633,0

-0.58074301584026,48.208297091931,0
-0.58065587085059,48.208372774762,0
-0.58057895512045,48.208453231157,0
-0.5805128540544,48.208537848816,0
-0.58045807076131,48.208625983764,0
-0.58041502222429,48.20871696526,0
-0.58038403612569,48.208810100892,0
-0.58036534835146,48.208904681851,0
-0.58035910119399,48.208999988325,0
-0.58199999999999,48.209,0
</coordinates>
</LinearRing>
</outerBoundaryIs>
</Polygon>
</Placemark>
<Placemark>
<name>309</name>
<styleUrl>#stylehap2</styleUrl>
<Polygon>
<outerBoundaryIs>
<LinearRing>
<coordinates>
-0.58199999999999,48.381,0
-0.58035356409988,48.380999988254,0
-0.58035982621466,48.381095294907,0
-0.58037857111224,48.381189876394,0
-0.58040965620042,48.381283012888,0
-0.58045284496653,48.381373995555,0
-0.58050780877555,48.381462131947,0
-0.58057412936929,48.381546751277,0
-0.58065130204829,48.381627209523,0
-0.58073873951151,48.381702894326,0
-0.58083577632529,48.381773229657,0
-0.58094167398694,48.381837680199,0
-0.58105562654524,48.381895755421,0
-0.58117676673424,48.381947013313,0
-0.58130417257428,48.381991063751,0
-0.58143687438972,48.382027571464,0
-0.58157386218995,48.38205625859,0
-0.58171409335762,48.382076906791,0
-0.58185650058524,48.38208935891,0
-0.58199999999998,48.382093520174,0
-0.58214349941476,48.38208935891,0
-0.58228590664237,48.382076906791,0
-0.58242613781004,48.38205625859,0
-0.58256312561028,48.382027571464,0
-0.58269582742571,48.381991063751,0
-0.58282323326575,48.381947013313,0
-0.58294437345475,48.381895755421,0
-0.58305832601303,48.381837680199,0
-0.5831642236747,48.381773229657,0
-0.58326126048845,48.381702894326,0
-0.5833486979517,48.381627209523,0
-0.58342587063071,48.381546751277,0
-0.58349219122444,48.381462131947,0
-0.58354715503344,48.381373995555,0
-0.58359034379958,48.381283012888,0

-0.58362142888776,48.381189876394,0
-0.58364017378531,48.381095294907,0
-0.58364643590009,48.380999988254,0
-0.58364016764356,48.380904681781,0
-0.58362141679083,48.380810100823,0
-0.58359032611506,48.380716965194,0
-0.58354713229865,48.380625983702,0
-0.58349216413016,48.380537848757,0
-0.58342584000019,48.380453231104,0
-0.58334866471567,48.380372774715,0
-0.58326122565675,48.380297091889,0
-0.58316418830564,48.380226758597,0
-0.58305829118133,48.380162310095,0
-0.58294434021869,48.38010423685,0
-0.58282320263526,48.380052980814,0
-0.58269580033146,48.380008932054,0
-0.5825631028755,48.379972425788,0
-0.58242612012552,48.379943739836,0
-0.58228589454542,48.379923092501,0
-0.58214349327298,48.379910640912,0
-0.58199999999998,48.379906479826,0
-0.58185650672702,48.379910640912,0
-0.58171410545455,48.379923092501,0
-0.58157387987447,48.379943739836,0
-0.5814368971245,48.379972425788,0
-0.58130419966854,48.380008932054,0
-0.58117679736474,48.380052980814,0
-0.5810556597813,48.38010423685,0
-0.58094170881867,48.380162310095,0
-0.58083581169433,48.380226758597,0
-0.58073877434324,48.380297091889,0
-0.58065133528432,48.380372774715,0
-0.58057415999978,48.380453231104,0
-0.5805078358698,48.380537848757,0
-0.58045286770134,48.380625983702,0
-0.58040967388494,48.380716965194,0
-0.58037858320916,48.380810100823,0
-0.58035983235644,48.380904681781,0
-0.58035356409988,48.380999988254,0
-0.58199999999999,48.381,0
</coordinates>
</LinearRing>
</outerBoundaryIs>
</Polygon>
</Placemark>
<Placemark>
<name>310</name>
<styleUrl>#stylehap2</styleUrl>
<Polygon>
<outerBoundaryIs>
<LinearRing>
<coordinates>
-0.58199999999999,48.209,0
-0.58035910119399,48.208999988325,0
-0.58036534226721,48.209095294977,0
-0.5803840241421,48.209189876463,0
-0.58041500470544,48.209283012954,0

-0.58045804823949,48.209373995617,0
-0.58051282721396,48.209462132005,0
-0.58057892477692,48.209546751331,0
-0.58065583792592,48.20962720957,0
-0.58074298133485,48.209702894367,0
-0.58083969180774,48.209773229693,0
-0.58094523332558,48.209837680228,0
-0.58105880264763,48.209895755445,0
-0.58117953542477,48.209947013331,0
-0.58130651277828,48.209991063763,0
-0.58143876829392,48.210027571472,0
-0.5815752953782,48.210056258595,0
-0.5817150549206,48.210076906793,0
-0.58185698320357,48.21008935891,0
-0.58199999999998,48.210093520174,0
-0.58214301679642,48.21008935891,0
-0.58228494507939,48.210076906793,0
-0.5824247046218,48.210056258595,0
-0.58256123170607,48.210027571472,0
-0.58269348722172,48.209991063763,0
-0.58282046457522,48.209947013331,0
-0.58294119735236,48.209895755445,0
-0.58305476667439,48.209837680228,0
-0.58316030819223,48.209773229693,0
-0.58325701866514,48.209702894367,0
-0.58334416207408,48.20962720957,0
-0.58342107522307,48.209546751331,0
-0.58348717278604,48.209462132005,0
-0.5835419517605,48.209373995617,0
-0.58358499529455,48.209283012954,0
-0.5836159758579,48.209189876463,0
-0.58363465773276,48.209095294977,0
-0.58364089880601,48.208999988325,0
-0.58363465164854,48.208904681851,0
-0.58361596387431,48.208810100892,0
-0.5835849777757,48.20871696526,0
-0.58354192923869,48.208625983764,0
-0.58348714594559,48.208537848816,0
-0.58342104487951,48.208453231157,0
-0.58334412914941,48.208372774762,0
-0.58325698415974,48.208297091931,0
-0.58316027315453,48.208226758633,0
-0.58305473216901,48.208162310124,0
-0.5829411644277,48.208104236874,0
-0.58282043423169,48.208052980832,0
-0.58269346038127,48.208008932066,0
-0.58256120918428,48.207972425797,0
-0.58242468710294,48.207943739841,0
-0.5822849330958,48.207923092503,0
-0.5821430107122,48.207910640912,0
-0.58199999999998,48.207906479826,0
-0.58185698928779,48.207910640912,0
-0.58171506690419,48.207923092503,0
-0.58157531289705,48.207943739841,0
-0.58143879081571,48.207972425797,0
-0.5813065396187,48.208008932066,0
-0.58117956576831,48.208052980832,0

-0.5810588355723,48.208104236874,0
-0.58094526783098,48.208162310124,0
-0.58083972684544,48.208226758633,0
-0.58074301584026,48.208297091931,0
-0.58065587085059,48.208372774762,0
-0.58057895512045,48.208453231157,0
-0.5805128540544,48.208537848816,0
-0.58045807076131,48.208625983764,0
-0.58041502222429,48.20871696526,0
-0.58038403612569,48.208810100892,0
-0.58036534835146,48.208904681851,0
-0.58035910119399,48.208999988325,0
-0.58199999999999,48.209,0
</coordinates>
</LinearRing>
</outerBoundaryIs>
</Polygon>
</Placemark>
<Placemark>
<name>313</name>
<styleUrl>#stylehap2</styleUrl>
<Polygon>
<outerBoundaryIs>
<LinearRing>
<coordinates>
-0.58000000000004,48.183,0
-0.57835993367377,48.182999988336,0
-0.57836617158349,48.183095294987,0
-0.57838484398315,48.183189876473,0
-0.5784158088316,48.183283012964,0
-0.57845883053061,48.183373995627,0
-0.57851358171593,48.183462132014,0
-0.57857964574712,48.183546751338,0
-0.57865651987682,48.183627209577,0
-0.57874361907589,48.183702894374,0
-0.57884028048475,48.183773229698,0
-0.5789457684578,48.183837680233,0
-0.57905928016181,48.183895755448,0
-0.57917995168623,48.183947013334,0
-0.57930686461852,48.183991063765,0
-0.57943905303479,48.184027571473,0
-0.57957551085228,48.184056258596,0
-0.57971519948773,48.184076906793,0
-0.57985705576333,48.18408935891,0
-0.58000000000004,48.184093520173,0
-0.58014294423675,48.18408935891,0
-0.58028480051235,48.184076906793,0
-0.5804244891478,48.184056258596,0
-0.58056094696529,48.184027571473,0
-0.58069313538156,48.183991063765,0
-0.58082004831385,48.183947013334,0
-0.58094071983827,48.183895755448,0
-0.58105423154228,48.183837680233,0
-0.58115971951533,48.183773229698,0
-0.58125638092421,48.183702894374,0
-0.58134348012326,48.183627209577,0
-0.58142035425296,48.183546751338,0

-0.58148641828415,48.183462132014,0
-0.58154116946947,48.183373995627,0
-0.58158419116848,48.183283012964,0
-0.58161515601693,48.183189876473,0
-0.58163382841659,48.183095294987,0
-0.58164006632631,48.182999988336,0
-0.581633822341,48.182904681862,0
-0.58161514405034,48.182810100902,0
-0.58158417367449,48.18271696527,0
-0.58154114697964,48.182625983774,0
-0.58148639148182,48.182537848824,0
-0.5814203239525,48.182453231165,0
-0.58134344724533,48.182372774769,0
-0.58125634646779,48.182297091937,0
-0.58115968452737,48.182226758638,0
-0.58105419708588,48.182162310128,0
-0.58094068696034,48.182104236877,0
-0.58082001801339,48.182052980834,0
-0.58069310857923,48.182008932068,0
-0.58056092447546,48.181972425798,0
-0.5804244716538,48.181943739842,0
-0.58028478854575,48.181923092503,0
-0.58014293816116,48.181910640912,0
-0.58000000000004,48.181906479826,0
-0.57985706183892,48.181910640912,0
-0.57971521145433,48.181923092503,0
-0.57957552834628,48.181943739842,0
-0.57943907552462,48.181972425798,0
-0.57930689142085,48.182008932068,0
-0.57917998198669,48.182052980834,0
-0.57905931303974,48.182104236877,0
-0.5789458029142,48.182162310128,0
-0.57884031547271,48.182226758638,0
-0.57874365353229,48.182297091937,0
-0.57865655275475,48.182372774769,0
-0.57857967604758,48.182453231165,0
-0.57851360851826,48.182537848824,0
-0.57845885302044,48.182625983774,0
-0.57841582632559,48.18271696527,0
-0.57838485594974,48.182810100902,0
-0.57836617765908,48.182904681862,0
-0.57835993367377,48.182999988336,0
-0.58000000000004,48.183,0
</coordinates>
</LinearRing>
</outerBoundaryIs>
</Polygon>
</Placemark>
<Placemark>
<name>316</name>
<styleUrl>#stylehap2</styleUrl>
<Polygon>
<outerBoundaryIs>
<LinearRing>
<coordinates>
-0.57799999999997,48.247,0
-0.5763578823628,48.24699998831,0

-0.57636412806772,48.247095294961,0
-0.57638282381522,48.247189876447,0
-0.5764138273867,48.24728301294,0
-0.5764569028894,48.247373995603,0
-0.57651172254985,48.247462131992,0
-0.57657786920658,48.247546751319,0
-0.57665483948361,48.24762720956,0
-0.57674204762011,48.247702894358,0
-0.57683882992751,48.247773229685,0
-0.57694444984011,48.247837680222,0
-0.57705810352053,48.247895755439,0
-0.57717892597761,48.247947013327,0
-0.57730599765003,48.24799106376,0
-0.57743835140566,48.24802757147,0
-0.57757497990324,48.248056258594,0
-0.57771484326029,48.248076906792,0
-0.57785687696909,48.24808935891,0
-0.57799999999997,48.248093520173,0
-0.57814312303088,48.24808935891,0
-0.57828515673964,48.248076906792,0
-0.5784250200967,48.248056258594,0
-0.57856164859428,48.24802757147,0
-0.57869400234991,48.24799106376,0
-0.57882107402233,48.247947013327,0
-0.57894189647941,48.247895755439,0
-0.57905555015985,48.247837680222,0
-0.57916117007243,48.247773229685,0
-0.57925795237985,48.247702894358,0
-0.57934516051633,48.24762720956,0
-0.57942213079336,48.247546751319,0
-0.57948827745012,48.247462131992,0
-0.57954309711054,48.247373995603,0
-0.57958617261326,48.24728301294,0
-0.57961717618474,48.247189876447,0
-0.57963587193224,48.247095294961,0
-0.57964211763716,48.24699998831,0
-0.57963586583535,48.246904681835,0
-0.57961716417621,48.246810100877,0
-0.57958615505798,48.246716965246,0
-0.57954307454191,48.246625983751,0
-0.57948825055384,48.246537848803,0
-0.57942210038672,48.246453231146,0
-0.57934512752319,48.246372774752,0
-0.57925791780269,48.246297091921,0
-0.57916113496188,48.246226758625,0
-0.57905551558269,48.246162310118,0
-0.57894186348627,48.246104236868,0
-0.57882104361569,48.246052980828,0
-0.57869397545367,48.246008932064,0
-0.57856162602565,48.245972425795,0
-0.57842500254144,48.24594373984,0
-0.57828514473112,48.245923092503,0
-0.57814311693398,48.245910640912,0
-0.57799999999997,48.245906479826,0
-0.57785688306595,48.245910640912,0
-0.57771485526882,48.245923092503,0
-0.57757499745853,48.24594373984,0

-0.57743837397431,48.245972425795,0
-0.57730602454627,48.246008932064,0
-0.57717895638424,48.246052980828,0
-0.57705813651367,48.246104236868,0
-0.57694448441727,48.246162310118,0
-0.57683886503808,48.246226758625,0
-0.57674208219727,48.246297091921,0
-0.57665487247675,48.246372774752,0
-0.57657789961322,48.246453231146,0
-0.57651174944609,48.246537848803,0
-0.57645692545803,48.246625983751,0
-0.57641384494196,48.246716965246,0
-0.57638283582375,48.246810100877,0
-0.57636413416459,48.246904681835,0
-0.5763578823628,48.24699998831,0
-0.57799999999997,48.247,0
</coordinates>
</LinearRing>
</outerBoundaryIs>
</Polygon>
</Placemark>
<Placemark>
<name>324</name>
<styleUrl>#stylehap2</styleUrl>
<Polygon>
<outerBoundaryIs>
<LinearRing>
<coordinates>
-0.572,48.446,0
-0.57035145797593,48.445999988228,0
-0.57035772809418,48.44609529488,0
-0.57037649696343,48.446189876368,0
-0.5704076218093,48.446283012863,0
-0.57045086581682,48.446373995531,0
-0.57050589993062,48.446462131925,0
-0.57057230535776,48.446546751257,0
-0.5706495767532,48.446627209505,0
-0.5707371260648,48.44670289431,0
-0.57083428700761,48.446773229644,0
-0.57094032013437,48.446837680188,0
-0.57105441846284,48.446895755412,0
-0.57117571361757,48.446947013307,0
-0.57130328243947,48.446991063746,0
-0.5714361540123,48.447027571461,0
-0.57157331705339,48.447056258589,0
-0.57171372761144,48.44707690679,0
-0.57185631701349,48.44708935891,0
-0.57200000000001,48.447093520173,0
-0.57214368298653,48.44708935891,0
-0.57228627238855,48.44707690679,0
-0.5724266829466,48.447056258589,0
-0.5725638459877,48.447027571461,0
-0.57269671756055,48.446991063746,0
-0.57282428638242,48.446947013307,0
-0.57294558153715,48.446895755412,0
-0.57305967986562,48.446837680188,0
-0.57316571299238,48.446773229644,0

-0.57326287393522,48.44670289431,0
-0.5733504232468,48.446627209505,0
-0.57342769464226,48.446546751257,0
-0.57349410006937,48.446462131925,0
-0.5735491341832,48.446373995531,0
-0.57359237819069,48.446283012863,0
-0.57362350303657,48.446189876368,0
-0.57364227190584,48.44609529488,0
-0.57364854202409,48.445999988228,0
-0.57364226574213,48.445904681754,0
-0.57362349089647,48.445810100797,0
-0.57359236044302,48.445716965169,0
-0.57354911136725,48.445625983678,0
-0.57349407287838,48.445537848735,0
-0.5734276639024,48.445453231084,0
-0.57335038989212,48.445372774696,0
-0.57326283897914,48.445297091873,0
-0.57316567749706,48.445226758584,0
-0.57305964490955,48.445162310084,0
-0.57294554818247,48.445104236841,0
-0.57282425564256,48.445052980807,0
-0.57269669036956,48.445008932049,0
-0.57256382317175,48.444972425785,0
-0.57242666519896,48.444943739834,0
-0.57228626024845,48.4449230925,0
-0.57214367682282,48.444910640911,0
-0.57200000000001,48.444906479826,0
-0.57185632317718,48.444910640911,0
-0.57171373975157,48.4449230925,0
-0.57157333480106,48.444943739834,0
-0.57143617682825,48.444972425785,0
-0.57130330963046,48.445008932049,0
-0.57117574435743,48.445052980807,0
-0.57105445181752,48.445104236841,0
-0.57094035509045,48.445162310084,0
-0.57083432250293,48.445226758584,0
-0.57073716102085,48.445297091873,0
-0.5706496101079,48.445372774696,0
-0.5705723360976,48.445453231084,0
-0.57050592712162,48.445537848735,0
-0.57045088863277,48.445625983678,0
-0.57040763955697,48.445716965169,0
-0.57037650910355,48.445810100797,0
-0.57035773425787,48.445904681754,0
-0.57035145797593,48.445999988228,0
-0.572,48.446,0
</coordinates>
</LinearRing>
</outerBoundaryIs>
</Polygon>
</Placemark>
<Placemark>
<name>328</name>
<styleUrl>#stylehap2</styleUrl>
<Polygon>
<outerBoundaryIs>
<LinearRing>

<coordinates>
-0.56799999999998,48.186,0
-0.56635983767871,48.185999988335,0
-0.56636607595322,48.186095294986,0
-0.56638474944549,48.186189876472,0
-0.56641571610607,48.186283012963,0
-0.56645874032291,48.186373995626,0
-0.56651349471265,48.186462132013,0
-0.56657956261045,48.186546751338,0
-0.56665644123957,48.186627209576,0
-0.56674354553656,48.186702894373,0
-0.5668402126031,48.186773229697,0
-0.56694570675052,48.186837680232,0
-0.56705922509861,48.186895755448,0
-0.5671799036862,48.186947013333,0
-0.56730682404701,48.186991063765,0
-0.56743902020066,48.187027571473,0
-0.56757548600547,48.187056258596,0
-0.56771518281733,48.187076906793,0
-0.56785704739626,48.18708935891,0
-0.568,48.187093520173,0
-0.56814295260373,48.18708935891,0
-0.56828481718264,48.187076906793,0
-0.5684245139945,48.187056258596,0
-0.5685609797993,48.187027571473,0
-0.56869317595295,48.186991063765,0
-0.56882009631379,48.186947013333,0
-0.56894077490138,48.186895755448,0
-0.56905429324945,48.186837680232,0
-0.56915978739687,48.186773229697,0
-0.5692564544634,48.186702894373,0
-0.56934355876039,48.186627209576,0
-0.56942043738951,48.186546751338,0
-0.56948650528731,48.186462132013,0
-0.56954125967706,48.186373995626,0
-0.56958428389389,48.186283012963,0
-0.56961525055447,48.186189876472,0
-0.56963392404674,48.186095294986,0
-0.56964016232125,48.185999988335,0
-0.56963391797015,48.18590468186,0
-0.56961523858592,48.185810100901,0
-0.56958426639705,48.185716965269,0
-0.56954123718354,48.185625983773,0
-0.56948647848058,48.185537848823,0
-0.56942040708407,48.185453231164,0
-0.56934352587707,48.185372774768,0
-0.56925642000135,48.185297091936,0
-0.56915975240318,48.185226758637,0
-0.5690542587874,48.185162310128,0
-0.56894074201806,48.185104236877,0
-0.56882006600835,48.185052980834,0
-0.56869314914622,48.185008932068,0
-0.56856095730579,48.184972425798,0
-0.56842449649766,48.184943739841,0
-0.56828480521408,48.184923092503,0
-0.56814294652712,48.184910640912,0
-0.568,48.184906479826,0

-0.56785705347285,48.184910640912,0
-0.56771519478588,48.184923092503,0
-0.56757550350231,48.184943739841,0
-0.56743904269418,48.184972425798,0
-0.56730685085374,48.185008932068,0
-0.56717993399162,48.185052980834,0
-0.5670592579819,48.185104236877,0
-0.56694574121257,48.185162310128,0
-0.56684024759678,48.185226758637,0
-0.5667435799861,48.185297091936,0
-0.56665647412289,48.185372774768,0
-0.5665795929159,48.185453231164,0
-0.56651352151939,48.185537848823,0
-0.56645876281643,48.185625983773,0
-0.56641573360291,48.185716965269,0
-0.56638476141405,48.185810100901,0
-0.56636608202981,48.18590468186,0
-0.56635983767871,48.185999988335,0
-0.56799999999998,48.186,0
</coordinates>
</LinearRing>
</outerBoundaryIs>
</Polygon>
</Placemark>
<Placemark>
<name>330</name>
<styleUrl>#stylehap2</styleUrl>
<Polygon>
<outerBoundaryIs>
<LinearRing>
<coordinates>
-0.56799999999998,48.187,0
-0.56635980567689,48.186999988334,0
-0.56636604407299,48.187095294986,0
-0.5663847179295,48.187189876471,0
-0.56641568519419,48.187283012963,0
-0.56645871025041,48.187373995625,0
-0.56651346570841,48.187462132013,0
-0.56657953489523,48.187546751337,0
-0.56665641502429,48.187627209576,0
-0.5667435210208,48.187702894373,0
-0.56684018997343,48.187773229697,0
-0.5669456861792,48.187837680232,0
-0.5670592067422,48.187895755448,0
-0.56717988768445,48.187947013333,0
-0.56730681052174,48.187991063765,0
-0.56743900925479,48.188027571473,0
-0.56757547772231,48.188056258596,0
-0.56771517725996,48.188076906793,0
-0.56785704460694,48.18808935891,0
-0.568,48.188093520173,0
-0.56814295539303,48.18808935891,0
-0.56828482274001,48.188076906793,0
-0.56842452227766,48.188056258596,0
-0.5685609907452,48.188027571473,0
-0.56869318947825,48.187991063765,0
-0.56882011231552,48.187947013333,0

-0.56894079325776,48.187895755448,0
-0.56905431382077,48.187837680232,0
-0.56915981002654,48.187773229697,0
-0.56925647897917,48.187702894373,0
-0.56934358497567,48.187627209576,0
-0.56942046510474,48.187546751337,0
-0.56948653429155,48.187462132013,0
-0.56954128974956,48.187373995625,0
-0.56958431480578,48.187283012963,0
-0.56961528207046,48.187189876471,0
-0.56963395592697,48.187095294986,0
-0.56964019432308,48.186999988334,0
-0.56963394985005,48.18690468186,0
-0.56961527010125,48.186810100901,0
-0.56958429730797,48.186716965269,0
-0.56954126725482,48.186625983772,0
-0.56948650748337,48.186537848823,0
-0.56942043479764,48.186453231164,0
-0.56934355209054,48.186372774768,0
-0.56925644451524,48.186297091936,0
-0.56915977503092,48.186226758637,0
-0.56905427935683,48.186162310128,0
-0.56894076037266,48.186104236877,0
-0.56882008200845,48.186052980834,0
-0.56869316267004,48.186008932068,0
-0.56856096825043,48.185972425798,0
-0.56842450477985,48.185943739841,0
-0.56828481077082,48.185923092503,0
-0.56814294931611,48.185910640912,0
-0.568,48.185906479826,0
-0.56785705068386,48.185910640912,0
-0.56771518922915,48.185923092503,0
-0.56757549522012,48.185943739841,0
-0.56743903174953,48.185972425798,0
-0.56730683732992,48.186008932068,0
-0.56717991799152,48.186052980834,0
-0.5670592396273,48.186104236877,0
-0.56694572064313,48.186162310128,0
-0.56684022496904,48.186226758637,0
-0.56674355548473,48.186297091936,0
-0.56665644790942,48.186372774768,0
-0.56657956520233,48.186453231164,0
-0.5665134925166,48.186537848823,0
-0.56645873274515,48.186625983772,0
-0.56641570269199,48.186716965269,0
-0.56638472989872,48.186810100901,0
-0.56636605014991,48.18690468186,0
-0.56635980567689,48.186999988334,0
-0.56799999999998,48.187,0
</coordinates>
</LinearRing>
</outerBoundaryIs>
</Polygon>
</Placemark>
<Placemark>
<name>331</name>
<styleUrl>#stylehap2</styleUrl>

<Polygon>
<outerBoundaryIs>
<LinearRing>
<coordinates>
-0.56700000000001,48.186,0
-0.56535983767873,48.185999988335,0
-0.56536607595324,48.186095294986,0
-0.56538474944551,48.186189876472,0
-0.56541571610609,48.186283012963,0
-0.56545874032292,48.186373995626,0
-0.56551349471267,48.186462132013,0
-0.5655795626105,48.186546751338,0
-0.56565644123959,48.186627209576,0
-0.56574354553658,48.186702894373,0
-0.56584021260311,48.186773229697,0
-0.56594570675053,48.186837680232,0
-0.56605922509862,48.186895755448,0
-0.56617990368621,48.186947013333,0
-0.56630682404705,48.186991063765,0
-0.5664390202007,48.187027571473,0
-0.56657548600548,48.187056258596,0
-0.56671518281737,48.187076906793,0
-0.56685704739627,48.18708935891,0
-0.56700000000001,48.187093520173,0
-0.56714295260375,48.18708935891,0
-0.56728481718265,48.187076906793,0
-0.56742451399451,48.187056258596,0
-0.56756097979932,48.187027571473,0
-0.56769317595297,48.186991063765,0
-0.56782009631381,48.186947013333,0
-0.5679407749014,48.186895755448,0
-0.56805429324946,48.186837680232,0
-0.56815978739688,48.186773229697,0
-0.56825645446344,48.186702894373,0
-0.56834355876041,48.186627209576,0
-0.56842043738953,48.186546751338,0
-0.56848650528733,48.186462132013,0
-0.56854125967707,48.186373995626,0
-0.56858428389393,48.186283012963,0
-0.56861525055449,48.186189876472,0
-0.56863392404676,48.186095294986,0
-0.56864016232127,48.185999988335,0
-0.56863391797017,48.18590468186,0
-0.56861523858596,48.185810100901,0
-0.56858426639709,48.185716965269,0
-0.56854123718358,48.185625983773,0
-0.56848647848062,48.185537848823,0
-0.56842040708411,48.185453231164,0
-0.56834352587711,48.185372774768,0
-0.56825642000137,48.185297091936,0
-0.5681597524032,48.185226758637,0
-0.56805425878741,48.185162310128,0
-0.56794074201808,48.185104236877,0
-0.56782006600836,48.185052980834,0
-0.56769314914624,48.185008932068,0
-0.5675609573058,48.184972425798,0
-0.56742449649767,48.184943739841,0

-0.5672848052141,48.184923092503,0
-0.56714294652716,48.184910640912,0
-0.56700000000001,48.184906479826,0
-0.56685705347286,48.184910640912,0
-0.56671519478592,48.184923092503,0
-0.56657550350235,48.184943739841,0
-0.56643904269419,48.184972425798,0
-0.56630685085376,48.185008932068,0
-0.56617993399163,48.185052980834,0
-0.56605925798194,48.185104236877,0
-0.56594574121261,48.185162310128,0
-0.56584024759682,48.185226758637,0
-0.56574357999865,48.185297091936,0
-0.56565647412291,48.185372774768,0
-0.56557959291591,48.185453231164,0
-0.5655135215194,48.185537848823,0
-0.56545876281644,48.185625983773,0
-0.56541573360293,48.185716965269,0
-0.56538476141406,48.185810100901,0
-0.56536608202983,48.18590468186,0
-0.56535983767873,48.185999988335,0
-0.56700000000001,48.186,0
</coordinates>
</LinearRing>
</outerBoundaryIs>
</Polygon>
</Placemark>
<Placemark>
<name>332</name>
<styleUrl>#stylehap2</styleUrl>
<Polygon>
<outerBoundaryIs>
<LinearRing>
<coordinates>
-0.56700000000001,48.2,0
-0.56535938949392,48.199999988329,0
-0.56536562947158,48.20009529498,0
-0.56538430806505,48.200189876466,0
-0.56541528318611,48.200283012958,0
-0.56545831915835,48.200373995621,0
-0.56551308850904,48.200462132008,0
-0.56557917445944,48.200546751333,0
-0.56565607409551,48.200627209573,0
-0.56574320219389,48.200702894369,0
-0.56583989567522,48.200773229694,0
-0.56594541864971,48.20083768023,0
-0.5660589680178,48.200895755446,0
-0.56617967958219,48.200947013332,0
-0.56630663462571,48.200991063764,0
-0.56643886690384,48.201027571472,0
-0.56657537000002,48.201056258595,0
-0.5667151049863,48.201076906793,0
-0.56685700833207,48.20108935891,0
-0.56700000000001,48.201093520174,0
-0.56714299166796,48.20108935891,0
-0.5672848950137,48.201076906793,0
-0.56742462999998,48.201056258595,0

-0.56756113309615,48.201027571472,0
-0.56769336537431,48.200991063764,0
-0.5678203204178,48.200947013332,0
-0.56794103198222,48.200895755446,0
-0.56805458135031,48.20083768023,0
-0.56816010432481,48.200773229694,0
-0.5682567978061,48.200702894369,0
-0.56834392590451,48.200627209573,0
-0.56842082554055,48.200546751333,0
-0.56848691149098,48.200462132008,0
-0.56854168084164,48.200373995621,0
-0.56858471681391,48.200283012958,0
-0.56861569193497,48.200189876466,0
-0.56863437052845,48.20009529498,0
-0.5686406105061,48.199999988329,0
-0.5686343644472,48.199904681855,0
-0.56861567995726,48.199810100895,0
-0.56858469930369,48.199716965264,0
-0.56854165833092,48.199625983768,0
-0.56848688466374,48.199537848819,0
-0.56842079521195,48.19945323116,0
-0.56834389299602,48.199372774764,0
-0.56825676331767,48.199297091933,0
-0.56816006930433,48.199226758634,0
-0.56805454686188,48.199162310126,0
-0.56794099907374,48.199104236875,0
-0.56782029008918,48.199052980832,0
-0.56769333854707,48.199008932067,0
-0.56756111058543,48.198972425797,0
-0.56742461248975,48.198943739841,0
-0.56728488303601,48.198923092503,0
-0.56714298558671,48.198910640912,0
-0.56700000000001,48.198906479826,0
-0.56685701441331,48.198910640912,0
-0.56671511696401,48.198923092503,0
-0.56657538751027,48.198943739841,0
-0.56643888941459,48.198972425797,0
-0.56630666145292,48.199008932067,0
-0.56617970991082,48.199052980832,0
-0.56605900092626,48.199104236875,0
-0.56594545313814,48.199162310126,0
-0.56583993069569,48.199226758634,0
-0.56574323668233,48.199297091933,0
-0.56565610700398,48.199372774764,0
-0.56557920478807,48.19945323116,0
-0.56551311533626,48.199537848819,0
-0.5654583416691,48.199625983768,0
-0.56541530069633,48.199716965264,0
-0.56538432004276,48.199810100895,0
-0.56536563555282,48.199904681855,0
-0.56535938949392,48.199999988329,0
-0.56700000000001,48.2,0
</coordinates>
</LinearRing>
</outerBoundaryIs>
</Polygon>
</Placemark>

<Placemark>
<name>335</name>
<styleUrl>#stylehap2</styleUrl>
<Polygon>
<outerBoundaryIs>
<LinearRing>
<coordinates>
-0.56200000000001,48.2,0
-0.56035938949392,48.199999988329,0
-0.56036562947158,48.20009529498,0
-0.56038430806505,48.200189876466,0
-0.56041528318611,48.200283012958,0
-0.56045831915835,48.200373995621,0
-0.56051308850904,48.200462132008,0
-0.56057917445944,48.200546751333,0
-0.56065607409551,48.200627209573,0
-0.56074320219389,48.200702894369,0
-0.56083989567522,48.200773229694,0
-0.56094541864971,48.20083768023,0
-0.5610589680178,48.200895755446,0
-0.56117967958222,48.200947013332,0
-0.56130663462571,48.200991063764,0
-0.56143886690384,48.201027571472,0
-0.56157537000004,48.201056258595,0
-0.56171510498632,48.201076906793,0
-0.56185700833207,48.20108935891,0
-0.56200000000001,48.201093520174,0
-0.56214299166796,48.20108935891,0
-0.5622848950137,48.201076906793,0
-0.56242462999998,48.201056258595,0
-0.56256113309618,48.201027571472,0
-0.56269336537431,48.200991063764,0
-0.5628203204178,48.200947013332,0
-0.56294103198222,48.200895755446,0
-0.56305458135031,48.20083768023,0
-0.56316010432481,48.200773229694,0
-0.56325679780613,48.200702894369,0
-0.56334392590451,48.200627209573,0
-0.56342082554058,48.200546751333,0
-0.56348691149098,48.200462132008,0
-0.56354168084167,48.200373995621,0
-0.56358471681391,48.200283012958,0
-0.56361569193497,48.200189876466,0
-0.56363437052845,48.20009529498,0
-0.5636406105061,48.199999988329,0
-0.5636343644472,48.199904681855,0
-0.56361567995726,48.199810100895,0
-0.56358469930369,48.199716965264,0
-0.56354165833092,48.199625983768,0
-0.56348688466374,48.199537848819,0
-0.56342079521195,48.19945323116,0
-0.56334389299605,48.199372774764,0
-0.56325676331769,48.199297091933,0
-0.56316006930433,48.199226758634,0
-0.56305454686188,48.199162310126,0
-0.56294099907376,48.199104236875,0
-0.5628202900892,48.199052980832,0

-0.56269333854707,48.199008932067,0
-0.56256111058543,48.198972425797,0
-0.56242461248975,48.198943739841,0
-0.56228488303601,48.198923092503,0
-0.56214298558671,48.198910640912,0
-0.56200000000001,48.198906479826,0
-0.56185701441331,48.198910640912,0
-0.56171511696401,48.198923092503,0
-0.56157538751027,48.198943739841,0
-0.56143888941459,48.198972425797,0
-0.56130666145295,48.199008932067,0
-0.56117970991082,48.199052980832,0
-0.56105900092626,48.199104236875,0
-0.56094545313814,48.199162310126,0
-0.56083993069569,48.199226758634,0
-0.56074323668233,48.199297091933,0
-0.56065610700398,48.199372774764,0
-0.56057920478807,48.19945323116,0
-0.56051311533629,48.199537848819,0
-0.5604583416691,48.199625983768,0
-0.56041530069633,48.199716965264,0
-0.56038432004276,48.199810100895,0
-0.56036563555282,48.199904681855,0
-0.56035938949392,48.199999988329,0
-0.56200000000001,48.2,0
</coordinates>
</LinearRing>
</outerBoundaryIs>
</Polygon>
</Placemark>
<Placemark>
<name>338</name>
<styleUrl>#stylehap2</styleUrl>
<Polygon>
<outerBoundaryIs>
<LinearRing>
<coordinates>
-0.55899999999997,48.222,0
-0.5573586845103,48.22199998832,0
-0.55736492716698,48.222095294971,0
-0.55738361378451,48.222189876457,0
-0.55741460221367,48.222283012949,0
-0.5574576566769,48.222373995613,0
-0.55751244956074,48.222462132001,0
-0.55757856390747,48.222546751327,0
-0.55765549658693,48.222627209567,0
-0.55774266212436,48.222702894364,0
-0.55783939715543,48.22277322969,0
-0.5579449654742,48.222837680226,0
-0.55805856363599,48.222895755443,0
-0.55817932707213,48.22294701333,0
-0.55830633667058,48.222991063762,0
-0.55843862577169,48.223027571471,0
-0.55857518752627,48.223056258595,0
-0.55871498255995,48.223076906793,0
-0.55885694688499,48.22308935891,0
-0.55899999999998,48.223093520174,0

-0.55914305311494,48.22308935891,0
-0.55928501743999,48.223076906793,0
-0.55942481247366,48.223056258595,0
-0.55956137422825,48.223027571471,0
-0.55969366332936,48.222991063762,0
-0.55982067292781,48.22294701333,0
-0.55994143636397,48.222895755443,0
-0.56005503452574,48.222837680226,0
-0.56016060284451,48.22277322969,0
-0.56025733787558,48.222702894364,0
-0.56034450341301,48.222627209567,0
-0.56042143609247,48.222546751327,0
-0.56048755043919,48.222462132001,0
-0.56054234332304,48.222373995613,0
-0.56058539778626,48.222283012949,0
-0.56061638621543,48.222189876457,0
-0.56063507283295,48.222095294971,0
-0.56064131548963,48.22199998832,0
-0.56063506674441,48.221904681846,0
-0.56061637422331,48.221810100887,0
-0.56058538025494,48.221716965255,0
-0.56054232078522,48.22162598376,0
-0.56048752357969,48.221537848811,0
-0.56042140572736,48.221453231153,0
-0.56034447046495,48.221372774758,0
-0.56025730334565,48.221297091927,0
-0.5601605677819,48.22122675863,0
-0.56005499999581,48.221162310122,0
-0.55994140341589,48.221104236872,0
-0.5598206425627,48.22105298083,0
-0.55969363646983,48.221008932065,0
-0.55956135169046,48.220972425796,0
-0.55942479494234,48.22094373984,0
-0.55928500544787,48.220923092503,0
-0.55914304702637,48.220910640912,0
-0.55899999999998,48.220906479827,0
-0.55885695297357,48.220910640912,0
-0.55871499455206,48.220923092503,0
-0.55857520505759,48.22094373984,0
-0.55843864830948,48.220972425796,0
-0.55830636353011,48.221008932065,0
-0.55817935743724,48.22105298083,0
-0.55805859658404,48.221104236872,0
-0.55794500000413,48.221162310122,0
-0.55783943221804,48.22122675863,0
-0.55774269665429,48.221297091927,0
-0.55765552953501,48.221372774758,0
-0.55757859427258,48.221453231153,0
-0.55751247642027,48.221537848811,0
-0.55745767921472,48.22162598376,0
-0.55741461974499,48.221716965255,0
-0.55738362577663,48.221810100887,0
-0.55736493325553,48.221904681846,0
-0.5573586845103,48.22199998832,0
-0.55899999999997,48.222,0
</coordinates>
</LinearRing>

</outerBoundaryIs>
</Polygon>
</Placemark>
<Placemark>
<name>339</name>
<styleUrl>#stylehap2</styleUrl>
<Polygon>
<outerBoundaryIs>
<LinearRing>
<coordinates>
-0.55500000000006,48.243,0
-0.55335801078018,48.242999988311,0
-0.55336425599711,48.243095294963,0
-0.55338295028298,48.243189876449,0
-0.55341395143029,48.243283012941,0
-0.55345702356475,48.243373995605,0
-0.55351183893848,48.243462131994,0
-0.55357798042263,48.24354675132,0
-0.5536549446806,48.243627209561,0
-0.55374214599734,48.243702894359,0
-0.55383892073618,48.243773229686,0
-0.55394453238902,48.243837680223,0
-0.55405817718138,48.24389575544,0
-0.55417899018967,48.243947013328,0
-0.55430605192456,48.243991063761,0
-0.55443839532952,48.24402757147,0
-0.55457501314209,48.244056258594,0
-0.55471486556114,48.244076906792,0
-0.55485688816214,48.24408935891,0
-0.55500000000007,48.244093520174,0
-0.55514311183799,48.24408935891,0
-0.55528513443899,48.244076906792,0
-0.55542498685804,48.244056258594,0
-0.55556160467061,48.24402757147,0
-0.55569394807557,48.243991063761,0
-0.55582100981046,48.243947013328,0
-0.55594182281875,48.24389575544,0
-0.55605546761109,48.243837680223,0
-0.55616107926395,48.243773229686,0
-0.5562578540028,48.243702894359,0
-0.55634505531953,48.243627209561,0
-0.55642201957747,48.24354675132,0
-0.55648816106166,48.243462131994,0
-0.55654297643538,48.243373995605,0
-0.55658604856985,48.243283012941,0
-0.55661704971716,48.243189876449,0
-0.55663574400303,48.243095294963,0
-0.55664198921995,48.242999988311,0
-0.55663573790748,48.242904681837,0
-0.55661703771128,48.242810100878,0
-0.5565860310184,48.242716965247,0
-0.55654295387169,48.242625983752,0
-0.55648813417129,48.242537848804,0
-0.5564219891775,48.242453231147,0
-0.55634502233359,48.242372774753,0
-0.5562578194332,48.242297091922,0
-0.55616104416106,48.242226758626,0

-0.55605543304149,48.242162310118,0
-0.55594178983284,48.242104236869,0
-0.55582097941047,48.242052980828,0
-0.55569392118521,48.242008932064,0
-0.55556158210689,48.241972425795,0
-0.5554249693066,48.24194373984,0
-0.55528512243311,48.241923092503,0
-0.55514310574242,48.241910640912,0
-0.55500000000007,48.241906479826,0
-0.55485689425769,48.241910640912,0
-0.55471487756702,48.241923092503,0
-0.55457503069354,48.24194373984,0
-0.55443841789321,48.241972425795,0
-0.55430607881493,48.242008932064,0
-0.55417902058967,48.242052980828,0
-0.55405821016729,48.242104236869,0
-0.55394456695862,48.242162310118,0
-0.55383895583907,48.242226758626,0
-0.55374218056691,48.242297091922,0
-0.55365497766652,48.242372774753,0
-0.55357801082263,48.242453231147,0
-0.55351186582885,48.242537848804,0
-0.55345704612845,48.242625983752,0
-0.55341396898173,48.242716965247,0
-0.55338296228886,48.242810100878,0
-0.55336426209265,48.242904681837,0
-0.55335801078018,48.242999988311,0
-0.55500000000006,48.243,0
</coordinates>
</LinearRing>
</outerBoundaryIs>
</Polygon>
</Placemark>
<Placemark>
<name>341</name>
<styleUrl>#stylehap2</styleUrl>
<Polygon>
<outerBoundaryIs>
<LinearRing>
<coordinates>
-0.55500000000006,48.25,0
-0.55335778603148,48.249999988308,0
-0.55336403210247,48.25009529496,0
-0.5533827289464,48.250189876446,0
-0.55341373433634,48.250283012939,0
-0.55345681236572,48.250373995602,0
-0.55351163524181,48.250462131991,0
-0.55357778577874,48.250546751318,0
-0.55365476057094,48.250627209559,0
-0.55374197382322,48.250702894357,0
-0.55383876180811,48.250773229684,0
-0.55394438791672,48.250837680222,0
-0.55405804826449,48.250895755439,0
-0.5541788778095,48.250947013327,0
-0.55430595693651,48.25099106376,0
-0.55443831845663,48.25102757147,0
-0.55457495496949,48.251056258594,0

-0.55471482653164,48.251076906792,0
-0.55485686857282,48.25108935891,0
-0.55500000000007,48.251093520173,0
-0.55514313142728,48.25108935891,0
-0.55528517346849,48.251076906792,0
-0.55542504503064,48.251056258594,0
-0.5555616815435,48.25102757147,0
-0.55569404306363,48.25099106376,0
-0.55582112219063,48.250947013327,0
-0.55594195173564,48.250895755439,0
-0.55605561208339,48.250837680222,0
-0.556161238192,48.250773229684,0
-0.55625802617691,48.250702894357,0
-0.5563452394292,48.250627209559,0
-0.55642221422139,48.250546751318,0
-0.55648836475832,48.250462131991,0
-0.55654318763442,48.250373995602,0
-0.55658626566377,48.250283012939,0
-0.55661727105371,48.250189876446,0
-0.55663596789767,48.25009529496,0
-0.55664221396866,48.249999988308,0
-0.55663596179978,48.249904681834,0
-0.55661725904323,48.249810100876,0
-0.55658624810561,48.249716965245,0
-0.55654316506207,48.24962598375,0
-0.55648833785765,48.249537848802,0
-0.55642218380977,48.249453231145,0
-0.55634520643064,48.249372774751,0
-0.55625799159408,48.249297091921,0
-0.55616120307567,48.249226758624,0
-0.55605557750058,48.249162310117,0
-0.55594191873708,48.249104236868,0
-0.55582109177901,48.249052980827,0
-0.55569401616296,48.249008932063,0
-0.55556165897116,48.248972425795,0
-0.55542502747248,48.24894373984,0
-0.55528516145801,48.248923092503,0
-0.5551431253294,48.248910640912,0
-0.55500000000007,48.248906479826,0
-0.55485687467071,48.248910640912,0
-0.55471483854213,48.248923092503,0
-0.55457497252766,48.24894373984,0
-0.55443834102895,48.248972425795,0
-0.55430598383715,48.249008932063,0
-0.55417890822113,48.249052980827,0
-0.55405808126305,48.249104236868,0
-0.55394442249955,48.249162310117,0
-0.55383879692444,48.249226758624,0
-0.55374200840603,48.249297091921,0
-0.55365479356947,48.249372774751,0
-0.55357781619037,48.249453231145,0
-0.55351166214249,48.249537848802,0
-0.55345683493806,48.24962598375,0
-0.5534137518945,48.249716965245,0
-0.55338274095688,48.249810100876,0
-0.55336403820036,48.249904681834,0
-0.55335778603148,48.249999988308,0

-0.55500000000006,48.25,0
</coordinates>
</LinearRing>
</outerBoundaryIs>
</Polygon>
</Placemark>
<Placemark>
<name>345</name>
<styleUrl>#stylehap2</styleUrl>
<Polygon>
<outerBoundaryIs>
<LinearRing>
<coordinates>
-0.55099999999993,48.239,0
-0.54935813916926,48.238999988313,0
-0.54936438389829,48.239095294965,0
-0.54938307672284,48.239189876451,0
-0.54941407544654,48.239283012943,0
-0.54945714421348,48.239373995606,0
-0.54951195530142,48.239462131995,0
-0.54957809161415,48.239546751321,0
-0.54965504985436,48.239627209562,0
-0.54974224435282,48.23970289436,0
-0.54983901152477,48.239773229686,0
-0.54994461491966,48.239837680223,0
-0.55005825082586,48.239895755441,0
-0.55017905438747,48.239947013328,0
-0.550306106187,48.239991063761,0
-0.55043843924355,48.240027571471,0
-0.55057504637345,48.240056258594,0
-0.55071488785687,48.240076906793,0
-0.55085689935254,48.24008935891,0
-0.55099999999992,48.240093520174,0
-0.55114310064734,48.24008935891,0
-0.55128511214301,48.240076906793,0
-0.55142495362642,48.240056258594,0
-0.5515615607563,48.240027571471,0
-0.55169389381288,48.239991063761,0
-0.5518209456124,48.239947013328,0
-0.55194174917399,48.239895755441,0
-0.55205538508019,48.239837680223,0
-0.55216098847511,48.239773229686,0
-0.55225775564706,48.23970289436,0
-0.55234495014549,48.239627209562,0
-0.5524219083857,48.239546751321,0
-0.55248804469845,48.239462131995,0
-0.55254285578639,48.239373995606,0
-0.55258592455334,48.239283012943,0
-0.55261692327701,48.239189876451,0
-0.55263561610159,48.239095294965,0
-0.55264186083061,48.238999988313,0
-0.55263561000736,48.238904681839,0
-0.55261691127375,48.23881010088,0
-0.55258590700573,48.238716965249,0
-0.55254283322764,48.238625983754,0
-0.55248801781396,48.238537848805,0
-0.55242187799237,48.238453231148,0

-0.55234491716678,48.238372774754,0
-0.55225772108501,48.238297091923,0
-0.5521609533799,48.238226758626,0
-0.55205535051817,48.238162310119,0
-0.55194171619527,48.23810423687,0
-0.55182091521905,48.238052980828,0
-0.55169386692839,48.238008932064,0
-0.55156153819754,48.237972425795,0
-0.55142493607882,48.23794373984,0
-0.55128510013972,48.237923092503,0
-0.55114309455311,48.237910640912,0
-0.55099999999992,48.237906479826,0
-0.55085690544676,48.237910640912,0
-0.55071489986013,48.237923092503,0
-0.55057506392106,48.23794373984,0
-0.55043846180233,48.237972425795,0
-0.55030613307149,48.238008932064,0
-0.5501790847808,48.238052980828,0
-0.55005828380458,48.23810423687,0
-0.54994464948171,48.238162310119,0
-0.54983904661998,48.238226758626,0
-0.54974227891486,48.238297091923,0
-0.54965508283307,48.238372774754,0
-0.5495781220075,48.238453231148,0
-0.54951198218591,48.238537848805,0
-0.54945716677224,48.238625983754,0
-0.54941409299414,48.238716965249,0
-0.54938308872612,48.23881010088,0
-0.54936438999251,48.238904681839,0
-0.54935813916926,48.238999988313,0
-0.55099999999993,48.239,0
</coordinates>
</LinearRing>
</outerBoundaryIs>
</Polygon>
</Placemark>
<Placemark>
<name>353</name>
<styleUrl>#stylehap2</styleUrl>
<Polygon>
<outerBoundaryIs>
<LinearRing>
<coordinates>
-0.54499999999996,48.351,0
-0.54335453362944,48.350999988267,0
-0.54336079205989,48.351095294919,0
-0.54337952592237,48.351189876406,0
-0.54341059270854,48.3512830129,0
-0.54345375604495,48.351373995566,0
-0.54350868748997,48.351462131957,0
-0.54357496903166,48.351546751287,0
-0.54365209626769,48.351627209531,0
-0.54373948224284,48.351702894333,0
-0.54383646191523,48.351773229663,0
-0.54394229721712,48.351837680204,0
-0.54405618267182,48.351895755425,0
-0.54417725152414,48.351947013316,0

-0.54430458233739,48.351991063753,0
-0.54443720600697,48.352027571465,0
-0.54457411313715,48.352056258591,0
-0.54471426172457,48.352076906791,0
-0.54485658509032,48.35208935891,0
-0.54499999999997,48.352093520174,0
-0.54514341490959,48.35208935891,0
-0.54528573827534,48.352076906791,0
-0.54542588686276,48.352056258591,0
-0.54556279399293,48.352027571465,0
-0.54569541766254,48.351991063753,0
-0.54582274847577,48.351947013316,0
-0.54594381732811,48.351895755425,0
-0.54605770278282,48.351837680204,0
-0.5461635380847,48.351773229663,0
-0.54626051775709,48.351702894333,0
-0.54634790373225,48.351627209531,0
-0.54642503096827,48.351546751287,0
-0.54649131250996,48.351462131957,0
-0.54654624395495,48.351373995566,0
-0.54658940729136,48.3512830129,0
-0.54662047407754,48.351189876406,0
-0.54663920794002,48.351095294919,0
-0.54664546637049,48.350999988267,0
-0.54663920180834,48.350904681793,0
-0.54662046200049,48.350810100835,0
-0.54658938963588,48.350716965206,0
-0.5465462212575,48.350625983713,0
-0.54649128546019,48.350537848768,0
-0.54642500038808,48.350453231113,0
-0.54634787055079,48.350372774723,0
-0.54626048298256,48.350297091896,0
-0.54616350277372,48.350226758603,0
-0.54605766800828,48.3501623101,0
-0.54594378414663,48.350104236854,0
-0.54582271789558,48.350052980817,0
-0.54569539061277,48.350008932056,0
-0.54556277129548,48.34997242579,0
-0.54542586920727,48.349943739837,0
-0.54528572619829,48.349923092501,0
-0.54514340877791,48.349910640912,0
-0.54499999999997,48.349906479826,0
-0.54485659122202,48.349910640912,0
-0.54471427380164,48.349923092501,0
-0.54457413079264,48.349943739837,0
-0.54443722870443,48.34997242579,0
-0.54430460938717,48.350008932056,0
-0.54417728210433,48.350052980817,0
-0.54405621585328,48.350104236854,0
-0.54394233199162,48.3501623101,0
-0.54383649722621,48.350226758603,0
-0.54373951701735,48.350297091896,0
-0.54365212944914,48.350372774723,0
-0.54357499961185,48.350453231113,0
-0.54350871453975,48.350537848768,0
-0.54345377874241,48.350625983713,0
-0.54341061036403,48.350716965206,0

-0.54337953799944,48.350810100835,0
-0.54336079819159,48.350904681793,0
-0.54335453362944,48.350999988267,0
-0.54499999999996,48.351,0
</coordinates>
</LinearRing>
</outerBoundaryIs>
</Polygon>
</Placemark>
<Placemark>
<name>354</name>
<styleUrl>#stylehap2</styleUrl>
<Polygon>
<outerBoundaryIs>
<LinearRing>
<coordinates>
-0.54200000000003,47.817,0
-0.54037152801496,47.816999988485,0
-0.5403777218647,47.817095295135,0
-0.54039626229839,47.817189876617,0
-0.54042700827789,47.817283013103,0
-0.54046972586949,47.817373995758,0
-0.54052409002222,47.817462132136,0
-0.54058968704025,47.81754675145,0
-0.54066601772969,47.817627209677,0
-0.54075250119665,47.817702894461,0
-0.54084847926742,47.817773229772,0
-0.5409532214968,47.817837680294,0
-0.5410659307273,47.817895755497,0
-0.54118574915596,47.817947013371,0
-0.54131176486341,47.817991063792,0
-0.54144301875504,47.818027571491,0
-0.54157851186129,47.818056258606,0
-0.54171721294209,47.818076906798,0
-0.54185806633671,47.818089358912,0
-0.54200000000004,47.818093520173,0
-0.54214193366336,47.818089358912,0
-0.54228278705798,47.818076906798,0
-0.54242148813876,47.818056258606,0
-0.54255698124501,47.818027571491,0
-0.54268823513664,47.817991063792,0
-0.54281425084411,47.817947013371,0
-0.54293406927278,47.817895755497,0
-0.54304677850325,47.817837680294,0
-0.54315152073263,47.817773229772,0
-0.54324749880339,47.817702894461,0
-0.54333398227039,47.817627209677,0
-0.5434103129598,47.81754675145,0
-0.54347590997783,47.817462132136,0
-0.54353027413059,47.817373995758,0
-0.54357299172216,47.817283013103,0
-0.54360373770168,47.817189876617,0
-0.54362227813537,47.817095295135,0
-0.54362847198509,47.816999988485,0
-0.54362227217972,47.816904682009,0
-0.54360372597134,47.816810101047,0
-0.54357297457358,47.816716965409,0

-0.54353025208477,47.816625983905,0
-0.54347588370467,47.816537848947,0
-0.54341028325757,47.816453231277,0
-0.54333395004157,47.816372774869,0
-0.54324746502725,47.816297092024,0
-0.54315148643547,47.816226758712,0
-0.54304674472713,47.81616231019,0
-0.54293403704396,47.816104236926,0
-0.54281422114188,47.816052980871,0
-0.54268820886347,47.816008932095,0
-0.54255695919921,47.815972425815,0
-0.54242147099017,47.815943739851,0
-0.54228277532764,47.815923092508,0
-0.54214192770771,47.815910640913,0
-0.54200000000004,47.815906479826,0
-0.54185807229234,47.815910640913,0
-0.54171722467241,47.815923092508,0
-0.5415785290099,47.815943739851,0
-0.54144304080084,47.815972425815,0
-0.5413117911366,47.816008932095,0
-0.54118577885817,47.816052980871,0
-0.54106596295609,47.816104236926,0
-0.54095325527294,47.81616231019,0
-0.54084851356461,47.816226758712,0
-0.5407525349728,47.816297092024,0
-0.5406660499585,47.816372774869,0
-0.54058971674248,47.816453231277,0
-0.54052411629538,47.816537848947,0
-0.54046974791528,47.816625983905,0
-0.5404270254265,47.816716965409,0
-0.54039627402871,47.816810101047,0
-0.54037772782033,47.816904682009,0
-0.54037152801496,47.816999988485,0
-0.54200000000003,47.817,0
</coordinates>
</LinearRing>
</outerBoundaryIs>
</Polygon>
</Placemark>
<Placemark>
<name>355</name>
<styleUrl>#stylehap2</styleUrl>
<Polygon>
<outerBoundaryIs>
<LinearRing>
<coordinates>
-0.54099999999994,48.171,0
-0.53936031749643,48.170999988341,0
-0.53936655394757,48.171095294992,0
-0.5393852219786,48.171189876478,0
-0.53941617958155,48.171283012969,0
-0.53945919121328,48.171373995631,0
-0.53951392958616,48.171462132018,0
-0.53957997815721,48.171546751342,0
-0.5396568342967,48.171627209581,0
-0.53974391311236,48.171702894376,0
-0.53984055189979,48.1717732297,0

-0.5399460151855,48.171837680235,0
-0.54005950032417,48.17189575545,0
-0.54018014360743,48.171947013335,0
-0.54030702683771,48.171991063766,0
-0.54043918431719,48.172027571474,0
-0.54057561019858,48.172056258596,0
-0.54071526614169,48.172076906793,0
-0.54085708921756,48.172089358911,0
-0.54099999999995,48.172093520174,0
-0.54114291078232,48.172089358911,0
-0.54128473385819,48.172076906793,0
-0.54142438980133,48.172056258596,0
-0.54156081568272,48.172027571474,0
-0.54169297316217,48.171991063766,0
-0.54181985639245,48.171947013335,0
-0.5419404996757,48.17189575545,0
-0.54205398481437,48.171837680235,0
-0.54215944810011,48.1717732297,0
-0.54225608688752,48.171702894376,0
-0.54234316570318,48.171627209581,0
-0.54242002184267,48.171546751342,0
-0.54248607041371,48.171462132018,0
-0.54254080878659,48.171373995631,0
-0.54258382041833,48.171283012969,0
-0.54261477802128,48.171189876478,0
-0.54263344605231,48.171095294992,0
-0.54263968250344,48.170999988341,0
-0.54263343998068,48.170904681866,0
-0.54261476606255,48.170810100907,0
-0.54258380293581,48.170716965275,0
-0.5425407863115,48.170625983778,0
-0.54248604362894,48.170537848828,0
-0.54241999156208,48.170453231169,0
-0.5423431328468,48.170372774772,0
-0.54225605245369,48.17029709194,0
-0.54215941313508,48.17022675864,0
-0.54205395038054,48.17016231013,0
-0.54194046681933,48.170104236879,0
-0.54181982611184,48.170052980835,0
-0.54169294637742,48.170008932069,0
-0.54156079320762,48.169972425798,0
-0.54142437231881,48.169943739842,0
-0.54128472189946,48.169923092503,0
-0.54114290471069,48.169910640912,0
-0.54099999999995,48.169906479826,0
-0.54085709528919,48.169910640912,0
-0.54071527810042,48.169923092503,0
-0.54057562768109,48.169943739842,0
-0.54043920679226,48.169972425798,0
-0.54030705362248,48.170008932069,0
-0.54018017388804,48.170052980835,0
-0.54005953318058,48.170104236879,0
-0.53994604961933,48.17016231013,0
-0.5398405868648,48.17022675864,0
-0.53974394754619,48.17029709194,0
-0.53965686715308,48.170372774772,0
-0.53958000843782,48.170453231169,0

-0.53951395637094,48.170537848828,0
-0.53945921368838,48.170625983778,0
-0.53941619706406,48.170716965275,0
-0.53938523393736,48.170810100907,0
-0.5393665600192,48.170904681866,0
-0.53936031749643,48.170999988341,0
-0.54099999999994,48.171,0
</coordinates>
</LinearRing>
</outerBoundaryIs>
</Polygon>
</Placemark>
<Placemark>
<name>356</name>
<styleUrl>#stylehap2</styleUrl>
<Polygon>
<outerBoundaryIs>
<LinearRing>
<coordinates>
-0.54099999999994,48.24,0
-0.53935810707459,48.239999988312,0
-0.5393643519256,48.240095294964,0
-0.53938304511545,48.24018987645,0
-0.53941404444498,48.240283012942,0
-0.53945711405373,48.240373995606,0
-0.53951192621304,48.240462131995,0
-0.53957806381852,48.240546751321,0
-0.53965502356305,48.240627209562,0
-0.53974221976594,48.24070289436,0
-0.53983898882945,48.240773229686,0
-0.53994459428868,48.240837680223,0
-0.54005823241623,48.24089575544,0
-0.54017903833931,48.240947013328,0
-0.54030609262247,48.240991063761,0
-0.54043842826593,48.241027571471,0
-0.54057503806628,48.241056258594,0
-0.54071488228337,48.241076906793,0
-0.54085689655513,48.24108935891,0
-0.54099999999995,48.241093520174,0
-0.54114310344475,48.24108935891,0
-0.54128511771651,48.241076906793,0
-0.54142496193362,48.241056258594,0
-0.54156157173395,48.241027571471,0
-0.54169390737741,48.240991063761,0
-0.54182096166057,48.240947013328,0
-0.54194176758365,48.24089575544,0
-0.5420554057112,48.240837680223,0
-0.54216101117043,48.240773229686,0
-0.54225778023394,48.24070289436,0
-0.54234497643682,48.240627209562,0
-0.54242193618136,48.240546751321,0
-0.54248807378684,48.240462131995,0
-0.54254288594615,48.240373995606,0
-0.54258595555489,48.240283012942,0
-0.54261695488443,48.24018987645,0
-0.5426356480743,48.240095294964,0
-0.54264189292528,48.239999988312,0

-0.54263564197975,48.239904681838,0
-0.5426169428805,48.23981010088,0
-0.54258593800632,48.239716965248,0
-0.54254286338614,48.239625983753,0
-0.54248804690087,48.239537848805,0
-0.54242190578635,48.239453231148,0
-0.5423449434563,48.239372774753,0
-0.54225774567001,48.239297091923,0
-0.54216097607328,48.239226758626,0
-0.54205537114727,48.239162310119,0
-0.54194173460313,48.239104236869,0
-0.54182093126556,48.239052980828,0
-0.54169388049144,48.239008932064,0
-0.54156154917397,48.238972425795,0
-0.54142494438505,48.23894373984,0
-0.54128510571259,48.238923092503,0
-0.5411430973502,48.238910640912,0
-0.54099999999995,48.238906479827,0
-0.54085690264968,48.238910640912,0
-0.54071489428729,48.238923092503,0
-0.54057505561482,48.23894373984,0
-0.54043845082594,48.238972425795,0
-0.54030611950843,48.239008932064,0
-0.54017906873432,48.239052980828,0
-0.54005826539675,48.239104236869,0
-0.5399446288526,48.239162310119,0
-0.53983902392659,48.239226758626,0
-0.53974225432986,48.239297091923,0
-0.53965505654357,48.239372774753,0
-0.53957809421353,48.239453231148,0
-0.539511953099,48.239537848805,0
-0.53945713661374,48.239625983753,0
-0.53941406199356,48.239716965248,0
-0.53938305711937,48.23981010088,0
-0.53936435802015,48.239904681838,0
-0.53935810707459,48.239999988312,0
-0.54099999999994,48.24,0
</coordinates>
</LinearRing>
</outerBoundaryIs>
</Polygon>
</Placemark>
<Placemark>
<name>357</name>
<styleUrl>#stylehap2</styleUrl>
<Polygon>
<outerBoundaryIs>
<LinearRing>
<coordinates>
-0.53999999999996,48.172,0
-0.53836028552083,48.17199998834,0
-0.53836652209349,48.172095294992,0
-0.53838519048845,48.172189876477,0
-0.53841614869502,48.172283012968,0
-0.53845916116545,48.172373995631,0
-0.5385139006057,48.172462132018,0
-0.53857995046469,48.172546751342,0

-0.53865680810294,48.17262720958,0
-0.53874388861668,48.172702894376,0
-0.53884052928866,48.1727732297,0
-0.53894599463104,48.172837680235,0
-0.53905948198285,48.17289575545,0
-0.53918012761882,48.172947013335,0
-0.53930701332352,48.172991063766,0
-0.53943917338029,48.173027571474,0
-0.5395756019222,48.173056258596,0
-0.53971526058886,48.173076906793,0
-0.53985708643057,48.173089358911,0
-0.53999999999997,48.173093520174,0
-0.54014291356937,48.173089358911,0
-0.54028473941105,48.173076906793,0
-0.5404243980777,48.173056258596,0
-0.54056082661964,48.173027571474,0
-0.54069298667641,48.172991063766,0
-0.54081987238111,48.172947013335,0
-0.54094051801709,48.17289575545,0
-0.54105400536889,48.172837680235,0
-0.54115947071125,48.1727732297,0
-0.54125611138322,48.172702894376,0
-0.541343191897,48.17262720958,0
-0.54142004953524,48.172546751342,0
-0.54148609939423,48.172462132018,0
-0.54154083883448,48.172373995631,0
-0.54158385130492,48.172283012968,0
-0.54161480951146,48.172189876477,0
-0.54163347790644,48.172095294992,0
-0.5416397144791,48.17199998834,0
-0.54163347183448,48.171904681866,0
-0.54161479755206,48.171810100907,0
-0.54158383382143,48.171716965274,0
-0.54154081635816,48.171625983778,0
-0.54148607260798,48.171537848828,0
-0.54142001925297,48.171453231169,0
-0.54134315903881,48.171372774772,0
-0.54125607694751,48.171297091939,0
-0.54115943574431,48.17122675864,0
-0.54105397093315,48.17116231013,0
-0.5409404851589,48.171104236879,0
-0.54081984209885,48.171052980835,0
-0.54069295989019,48.171008932069,0
-0.54056080414332,48.170972425798,0
-0.54042438059425,48.170943739842,0
-0.54028472745165,48.170923092503,0
-0.54014290749741,48.170910640912,0
-0.53999999999997,48.170906479826,0
-0.5398570925025,48.170910640912,0
-0.53971527254828,48.170923092503,0
-0.53957561940569,48.170943739842,0
-0.53943919585659,48.170972425798,0
-0.53930704010975,48.171008932069,0
-0.53918015790109,48.171052980835,0
-0.53905951484103,48.171104236879,0
-0.53894602906676,48.17116231013,0
-0.5388405642556,48.17122675864,0

-0.5387439230524,48.171297091939,0
-0.5386568409611,48.171372774772,0
-0.53857998074696,48.171453231169,0
-0.53851392739193,48.171537848828,0
-0.53845918364175,48.171625983778,0
-0.5384161661785,48.171716965274,0
-0.53838520244785,48.171810100907,0
-0.53836652816545,48.171904681866,0
-0.53836028552083,48.17199998834,0
-0.53999999999996,48.172,0
</coordinates>
</LinearRing>
</outerBoundaryIs>
</Polygon>
</Placemark>
<Placemark>
<name>358</name>
<styleUrl>#stylehap2</styleUrl>
<Polygon>
<outerBoundaryIs>
<LinearRing>
<coordinates>
-0.53999999999996,48.118,0
-0.53836200970807,48.117999988362,0
-0.53836823972861,48.118095295014,0
-0.53838688849902,48.118189876499,0
-0.53841781415774,48.118283012989,0
-0.53846078140456,48.11837399565,0
-0.53851546328947,48.118462132036,0
-0.53858144369924,48.118546751358,0
-0.5386582205228,48.118627209595,0
-0.53874520947145,48.118702894389,0
-0.53884174852463,48.118773229711,0
-0.53894710296794,48.118837680244,0
-0.53906047098439,48.118895755457,0
-0.53918098975702,48.11894701334,0
-0.53930774203595,48.11899106377,0
-0.53943976312014,48.119027571476,0
-0.53957604820044,48.119056258598,0
-0.53971556000844,48.119076906794,0
-0.53985723671234,48.119089358911,0
-0.53999999999997,48.119093520174,0
-0.5401427632876,48.119089358911,0
-0.54028443999149,48.119076906794,0
-0.54042395179947,48.119056258598,0
-0.54056023687979,48.119027571476,0
-0.54069225796396,48.11899106377,0
-0.54081901024291,48.11894701334,0
-0.54093952901554,48.118895755457,0
-0.54105289703199,48.118837680244,0
-0.54115825147528,48.118773229711,0
-0.54125479052848,48.118702894389,0
-0.54134177947714,48.118627209595,0
-0.54141855630069,48.118546751358,0
-0.54148453671047,48.118462132036,0
-0.54153921859537,48.11837399565,0
-0.54158218584219,48.118283012989,0

-0.54161311150089,48.118189876499,0
-0.5416317602713,48.118095295014,0
-0.54163799029186,48.117999988362,0
-0.54163175421725,48.117904681888,0
-0.54161309957671,48.117810100928,0
-0.54158216841018,48.117716965295,0
-0.54153919618523,48.117625983797,0
-0.5414845100031,48.117537848846,0
-0.54141852610759,48.117453231185,0
-0.54134174671569,48.117372774787,0
-0.54125475619417,48.117297091952,0
-0.54115821661129,48.117226758651,0
-0.54105286269765,48.117162310139,0
-0.5409394962541,48.117104236886,0
-0.5408189800498,48.117052980841,0
-0.54069223125658,48.117008932073,0
-0.54056021446965,48.116972425801,0
-0.54042393436749,48.116943739843,0
-0.54028442806729,48.116923092504,0
-0.54014275723353,48.116910640913,0
-0.53999999999997,48.116906479827,0
-0.53985724276638,48.116910640913,0
-0.53971557193265,48.116923092504,0
-0.53957606563245,48.116943739843,0
-0.53943978553028,48.116972425801,0
-0.53930776874332,48.117008932073,0
-0.53918101995013,48.117052980841,0
-0.53906050374583,48.117104236886,0
-0.53894713730228,48.117162310139,0
-0.53884178338862,48.117226758651,0
-0.53874524380577,48.117297091952,0
-0.53865825328424,48.117372774787,0
-0.53858147389235,48.117453231185,0
-0.53851548999684,48.117537848846,0
-0.5384608038147,48.117625983797,0
-0.53841783158975,48.117716965295,0
-0.53838690042322,48.117810100928,0
-0.53836824578268,48.117904681888,0
-0.53836200970807,48.117999988362,0
-0.53999999999996,48.118,0
</coordinates>
</LinearRing>
</outerBoundaryIs>
</Polygon>
</Placemark>
<Placemark>
<name>359</name>
<styleUrl>#stylehap2</styleUrl>
<Polygon>
<outerBoundaryIs>
<LinearRing>
<coordinates>
-0.53899999999999,48.353,0
-0.53735446904371,48.352999988266,0
-0.53736072771962,48.353095294918,0
-0.53737946231719,48.353189876405,0
-0.53741053032256,48.353283012899,0

-0.53745369535299,48.353373995565,0
-0.53750862895394,48.353462131957,0
-0.53757491309709,48.353546751286,0
-0.53765204336034,48.35362720953,0
-0.53773943276541,48.353702894333,0
-0.53783641624432,48.353773229663,0
-0.53794225570031,48.353837680204,0
-0.53805614562517,48.353895755425,0
-0.53817721922961,48.353947013316,0
-0.53830455504081,48.353991063753,0
-0.53843718391613,48.354027571465,0
-0.5385740964202,48.354056258591,0
-0.53871425050873,48.354076906791,0
-0.53885657946101,48.35408935891,0
-0.53899999999998,48.354093520174,0
-0.53914342053898,48.35408935891,0
-0.53928574949124,48.354076906791,0
-0.53942590357979,48.354056258591,0
-0.53956281608383,48.354027571465,0
-0.53969544495915,48.353991063753,0
-0.53982278077035,48.353947013316,0
-0.53994385437482,48.353895755425,0
-0.54005774429965,48.353837680204,0
-0.54016358375567,48.353773229663,0
-0.54026056723458,48.353702894333,0
-0.54034795663964,48.35362720953,0
-0.54042508690287,48.353546751286,0
-0.54049137104603,48.353462131957,0
-0.54054630464698,48.353373995565,0
-0.5405894696774,48.353283012899,0
-0.54062053768277,48.353189876405,0
-0.54063927228036,48.353095294918,0
-0.54064553095627,48.352999988266,0
-0.540639266148,48.352904681792,0
-0.5406205256044,48.352810100834,0
-0.54058945201998,48.352716965205,0
-0.54054628194703,48.352625983712,0
-0.5404913439933,48.352537848767,0
-0.54042505631932,48.352453231113,0
-0.54034792345455,48.352372774722,0
-0.54026053245623,48.352297091896,0
-0.54016354844083,48.352226758603,0
-0.54005770952133,48.3521623101,0
-0.5399438211897,48.352104236854,0
-0.5398227501868,48.352052980817,0
-0.53969541790642,48.352008932056,0
-0.53956279338388,48.35197242579,0
-0.53942588592237,48.351943739837,0
-0.53928573741284,48.351923092501,0
-0.53914341440662,48.351910640912,0
-0.53899999999998,48.351906479827,0
-0.53885658559334,48.351910640912,0
-0.53871426258712,48.351923092501,0
-0.53857411407762,48.351943739837,0
-0.53843720661608,48.35197242579,0
-0.53830458209354,48.352008932056,0
-0.53817724981317,48.352052980817,0

-0.53805617881026,48.352104236854,0
-0.53794229047866,48.3521623101,0
-0.53783645155913,48.352226758603,0
-0.53773946754373,48.352297091896,0
-0.53765207654544,48.352372774722,0
-0.53757494368064,48.352453231113,0
-0.53750865600669,48.352537848767,0
-0.53745371805294,48.352625983712,0
-0.53741054797998,48.352716965205,0
-0.53737947439559,48.352810100834,0
-0.53736073385196,48.352904681792,0
-0.53735446904371,48.352999988266,0
-0.53899999999999,48.353,0
</coordinates>
</LinearRing>
</outerBoundaryIs>
</Polygon>
</Placemark>
<Placemark>
<name>364</name>
<styleUrl>#stylehap2</styleUrl>
<Polygon>
<outerBoundaryIs>
<LinearRing>
<coordinates>
-0.53700000000003,48.166,0
-0.53536047734843,48.165999988343,0
-0.53536671319213,48.166095294994,0
-0.53538537940372,48.16618987648,0
-0.53541633398911,48.166283012971,0
-0.53545934142812,48.166373995633,0
-0.53551407446494,48.16646213202,0
-0.53558011659725,48.166546751344,0
-0.53565696524429,48.166627209582,0
-0.53574403557082,48.166702894378,0
-0.53584066493702,48.166773229701,0
-0.53594611794112,48.166837680236,0
-0.53605959201604,48.16689575545,0
-0.53618022353763,48.166947013335,0
-0.53630709439779,48.166991063766,0
-0.53643923899292,48.167027571474,0
-0.53657565157376,48.167056258596,0
-0.5367152939014,48.167076906793,0
-0.53685710315051,48.167089358911,0
-0.53700000000004,48.167093520174,0
-0.53714289684957,48.167089358911,0
-0.53728470609868,48.167076906793,0
-0.53742434842631,48.167056258596,0
-0.53756076100716,48.167027571474,0
-0.53769290560229,48.166991063766,0
-0.53781977646245,48.166947013335,0
-0.53794040798401,48.16689575545,0
-0.53805388205896,48.166837680236,0
-0.53815933506305,48.166773229701,0
-0.53825596442925,48.166702894378,0
-0.53834303475578,48.166627209582,0
-0.53841988340283,48.166546751344,0

-0.53848592553513,48.16646213202,0
-0.53854065857196,48.166373995633,0
-0.53858366601094,48.166283012971,0
-0.53861462059635,48.16618987648,0
-0.53863328680795,48.166095294994,0
-0.53863952265162,48.165999988343,0
-0.538633280738,48.165904681868,0
-0.53861460864087,48.165810100909,0
-0.5385836485332,48.165716965277,0
-0.53854063610302,48.16562598378,0
-0.53848589875766,48.16553784883,0
-0.53841985313049,48.16545323117,0
-0.53834300190836,48.165372774774,0
-0.53825593000481,48.165297091941,0
-0.53815930010756,48.165226758641,0
-0.53805384763452,48.165162310131,0
-0.53794037513662,48.165104236879,0
-0.53781974619011,48.165052980836,0
-0.53769287882482,48.165008932069,0
-0.53756073853819,48.164972425799,0
-0.53742433094856,48.164943739842,0
-0.5372846941432,48.164923092504,0
-0.53714289077962,48.164910640912,0
-0.53700000000004,48.164906479826,0
-0.53685710922046,48.164910640912,0
-0.53671530585688,48.164923092504,0
-0.53657566905149,48.164943739842,0
-0.53643926146186,48.164972425799,0
-0.53630712117524,48.165008932069,0
-0.53618025380994,48.165052980836,0
-0.53605962486346,48.165104236879,0
-0.53594615236556,48.165162310131,0
-0.53584069989249,48.165226758641,0
-0.53574406999524,48.165297091941,0
-0.53565699809171,48.165372774774,0
-0.53558014686959,48.16545323117,0
-0.53551410124241,48.16553784883,0
-0.53545936389706,48.16562598378,0
-0.53541635146687,48.165716965277,0
-0.5353853913592,48.165810100909,0
-0.53536671926207,48.165904681868,0
-0.53536047734843,48.165999988343,0
-0.53700000000003,48.166,0
</coordinates>
</LinearRing>
</outerBoundaryIs>
</Polygon>
</Placemark>
<Placemark>
<name>365</name>
<styleUrl>#stylehap2</styleUrl>
<Polygon>
<outerBoundaryIs>
<LinearRing>
<coordinates>
-0.53700000000003,48.245,0
-0.53535794657501,48.24499998831,0

-0.53536419203592,48.245095294962,0
-0.53538288705255,48.245189876448,0
-0.53541388941189,48.24528301294,0
-0.53545696323039,48.245373995604,0
-0.53551178074735,48.245462131993,0
-0.53557792481768,48.245546751319,0
-0.53565489208501,48.24562720956,0
-0.53574209681143,48.245702894359,0
-0.53583887533434,48.245773229685,0
-0.53594449111684,48.245837680222,0
-0.53605814035297,48.24589575544,0
-0.5361789580854,48.245947013327,0
-0.5363060247888,48.245991063761,0
-0.5364383733688,48.24602757147,0
-0.53657499652359,48.246056258594,0
-0.53671485441134,48.246076906792,0
-0.53685688256594,48.24608935891,0
-0.53700000000004,48.246093520174,0
-0.53714311743414,48.24608935891,0
-0.53728514558874,48.246076906792,0
-0.53742500347649,48.246056258594,0
-0.53756162663125,48.24602757147,0
-0.53769397521127,48.245991063761,0
-0.53782104191465,48.245947013327,0
-0.53794185964708,48.24589575544,0
-0.53805550888324,48.245837680222,0
-0.53816112466573,48.245773229685,0
-0.53825790318865,48.245702894359,0
-0.53834510791506,48.24562720956,0
-0.5384220751824,48.245546751319,0
-0.53848821925273,48.245462131993,0
-0.53854303676969,48.245373995604,0
-0.53858611058816,48.24528301294,0
-0.5386171129475,48.245189876448,0
-0.53863580796416,48.245095294962,0
-0.53864205342507,48.24499998831,0
-0.53863580186792,48.244904681836,0
-0.53861710094029,48.244810100878,0
-0.53858609303481,48.244716965246,0
-0.53854301420352,48.244625983751,0
-0.53848819235941,48.244537848803,0
-0.53842204477909,48.244453231146,0
-0.53834507492554,48.244372774752,0
-0.53825786861528,48.244297091922,0
-0.538161089559,48.244226758625,0
-0.53805547430985,48.244162310118,0
-0.53794182665755,48.244104236869,0
-0.53782101151135,48.244052980828,0
-0.53769394831795,48.244008932064,0
-0.53756160406508,48.243972425795,0
-0.53742498592311,48.24394373984,0
-0.53728513358151,48.243923092503,0
-0.53714311133793,48.243910640912,0
-0.53700000000004,48.243906479826,0
-0.53685688866215,48.243910640912,0
-0.53671486641854,48.243923092503,0
-0.53657501407694,48.24394373984,0

-0.53643839593499,48.243972425795,0
-0.5363060516821,48.244008932064,0
-0.53617898848873,48.244052980828,0
-0.53605817334252,48.244104236869,0
-0.53594452569023,48.244162310118,0
-0.53583891044105,48.244226758625,0
-0.5357421313848,48.244297091922,0
-0.53565492507454,48.244372774752,0
-0.53557795522098,48.244453231146,0
-0.53551180764067,48.244537848803,0
-0.53545698579655,48.244625983751,0
-0.53541390696526,48.244716965246,0
-0.53538289905978,48.244810100878,0
-0.53536419813213,48.244904681836,0
-0.53535794657501,48.24499998831,0
-0.53700000000003,48.245,0
</coordinates>
</LinearRing>
</outerBoundaryIs>
</Polygon>
</Placemark>
<Placemark>
<name>366</name>
<styleUrl>#stylehap2</styleUrl>
<Polygon>
<outerBoundaryIs>
<LinearRing>
<coordinates>
-0.53600000000006,48.448,0
-0.53435139305305,48.447999988227,0
-0.53435766341803,48.448095294879,0
-0.53437643302623,48.448189876367,0
-0.53440755909766,48.448283012862,0
-0.53445080480802,48.44837399553,0
-0.53450584108903,48.448462131924,0
-0.53457224913122,48.448546751257,0
-0.53464952356968,48.448627209504,0
-0.53473707632908,48.448702894309,0
-0.53483424109828,48.448773229643,0
-0.53494027840086,48.448837680188,0
-0.53505438122281,48.448895755412,0
-0.53517568115448,48.448947013306,0
-0.53530325500041,48.448991063746,0
-0.53543613180617,48.44902757146,0
-0.53557330024919,48.449056258588,0
-0.53571371633708,48.44907690679,0
-0.5358563113548,48.44908935891,0
-0.53600000000005,48.449093520173,0
-0.53614368864531,48.44908935891,0
-0.53628628366302,48.44907690679,0
-0.53642669975094,48.449056258588,0
-0.53656386819397,48.44902757146,0
-0.5366967449997,48.448991063746,0
-0.53682431884562,48.448947013306,0
-0.53694561877732,48.448895755412,0
-0.53705972159925,48.448837680188,0
-0.53716575890183,48.448773229643,0

-0.53726292367105,48.448702894309,0
-0.53735047643042,48.448627209504,0
-0.53742775086891,48.448546751257,0
-0.53749415891108,48.448462131924,0
-0.53754919519209,48.44837399553,0
-0.53759244090245,48.448283012862,0
-0.5376235669739,48.448189876367,0
-0.5376423365821,48.448095294879,0
-0.53764860694706,48.447999988227,0
-0.53764233041773,48.447904681753,0
-0.53762355483245,48.447810100796,0
-0.53759242315284,48.447716965168,0
-0.53754917237364,48.447625983678,0
-0.53749413171711,48.447537848735,0
-0.53742772012567,48.447453231083,0
-0.53735044307208,48.447372774696,0
-0.53726288871113,48.447297091873,0
-0.53716572340261,48.447226758583,0
-0.53705968663936,48.447162310083,0
-0.53694558541895,48.447104236841,0
-0.53682428810241,48.447052980807,0
-0.53669671780573,48.447008932049,0
-0.5365638453755,48.446972425785,0
-0.53642668200134,48.446943739834,0
-0.53628627152157,48.4469230925,0
-0.53614368248094,48.446910640911,0
-0.53600000000005,48.446906479826,0
-0.53585631751917,48.446910640911,0
-0.53571372847853,48.4469230925,0
-0.5355733179988,48.446943739834,0
-0.53543615462461,48.446972425785,0
-0.53530328219438,48.447008932049,0
-0.5351757118977,48.447052980807,0
-0.53505441458116,48.447104236841,0
-0.53494031336078,48.447162310083,0
-0.5348342765975,48.447226758583,0
-0.53473711128898,48.447297091873,0
-0.53464955692803,48.447372774696,0
-0.53457227987444,48.447453231083,0
-0.534505868283,48.447537848735,0
-0.53445082762646,48.447625983678,0
-0.53440757684727,48.447716965168,0
-0.53437644516765,48.447810100796,0
-0.5343576695824,48.447904681753,0
-0.53435139305305,48.447999988227,0
-0.53600000000006,48.448,0
</coordinates>
</LinearRing>
</outerBoundaryIs>
</Polygon>
</Placemark>
<Placemark>
<name>369</name>
<styleUrl>#stylehap2</styleUrl>
<Polygon>
<outerBoundaryIs>
<LinearRing>

<coordinates>
-0.53399999999999,48.221,0
-0.53235871657343,48.22099998832,0
-0.53236495910825,48.221095294972,0
-0.53238364536085,48.221189876458,0
-0.53241463318477,48.22128301295,0
-0.53245768680701,48.221373995613,0
-0.53251247862053,48.221462132001,0
-0.5325785916758,48.221546751327,0
-0.53265552285241,48.221627209567,0
-0.53274268668709,48.221702894364,0
-0.53283941982846,48.22177322969,0
-0.53294498608493,48.221837680226,0
-0.53305858202754,48.221895755443,0
-0.53317934310454,48.22194701333,0
-0.5333063502218,48.221991063762,0
-0.53343863673855,48.222027571471,0
-0.53357519582533,48.222056258595,0
-0.53371498812801,48.222076906793,0
-0.53385694967968,48.22208935891,0
-0.53399999999998,48.222093520174,0
-0.53414305032031,48.22208935891,0
-0.53428501187198,48.222076906793,0
-0.53442480417466,48.222056258595,0
-0.53456136326144,48.222027571471,0
-0.53469364977819,48.221991063762,0
-0.53482065689545,48.22194701333,0
-0.53494141797245,48.221895755443,0
-0.53505501391503,48.221837680226,0
-0.53516058017153,48.22177322969,0
-0.5352573133129,48.221702894364,0
-0.53534447714758,48.221627209567,0
-0.53542140832419,48.221546751327,0
-0.53548752137943,48.221462132001,0
-0.53554231319298,48.221373995613,0
-0.53558536681522,48.22128301295,0
-0.53561635463913,48.221189876458,0
-0.53563504089171,48.221095294972,0
-0.53564128342656,48.22099998832,0
-0.5356350348035,48.220904681846,0
-0.53561634264768,48.220810100887,0
-0.53558534928487,48.220716965256,0
-0.53554229065639,48.22062598376,0
-0.5354874945214,48.220537848812,0
-0.53542137796073,48.220453231154,0
-0.53534444420131,48.220372774759,0
-0.53525727878484,48.220297091928,0
-0.53516054511085,48.22022675863,0
-0.53505497938701,48.220162310122,0
-0.53494138502616,48.220104236872,0
-0.53482062653199,48.22005298083,0
-0.53469362292013,48.220008932065,0
-0.53456134072485,48.219972425796,0
-0.5344247866443,48.21994373984,0
-0.53428499988053,48.219923092503,0
-0.53414304423209,48.219910640912,0
-0.53399999999998,48.219906479826,0

-0.5338569557679,48.219910640912,0
-0.53371500011946,48.219923092503,0
-0.53357521335566,48.21994373984,0
-0.53343865927512,48.219972425796,0
-0.53330637707983,48.220008932065,0
-0.53317937346798,48.22005298083,0
-0.53305861497381,48.220104236872,0
-0.53294502061298,48.220162310122,0
-0.53283945488914,48.22022675863,0
-0.53274272121512,48.220297091928,0
-0.53265555579868,48.220372774759,0
-0.53257862203923,48.220453231154,0
-0.53251250547859,48.220537848812,0
-0.53245770934358,48.22062598376,0
-0.5324146507151,48.220716965256,0
-0.53238365735231,48.220810100887,0
-0.53236496519649,48.220904681846,0
-0.53235871657343,48.22099998832,0
-0.53399999999999,48.221,0
</coordinates>
</LinearRing>
</outerBoundaryIs>
</Polygon>
</Placemark>
<Placemark>
<name>370</name>
<styleUrl>#stylehap2</styleUrl>
<Polygon>
<outerBoundaryIs>
<LinearRing>
<coordinates>
-0.53399999999999,48.246,0
-0.53235791446977,48.24599998831,0
-0.53236416005269,48.246095294962,0
-0.53238285543473,48.246189876448,0
-0.53241385840012,48.24628301294,0
-0.53245693306071,48.246373995604,0
-0.53251175164937,48.246462131993,0
-0.53257789701288,48.246546751319,0
-0.53265486578503,48.24662720956,0
-0.53274207221642,48.246702894358,0
-0.53283885263154,48.246773229685,0
-0.53294447047903,48.246837680222,0
-0.53305812193725,48.24689575544,0
-0.53317894203194,48.246947013327,0
-0.53330601121976,48.246991063761,0
-0.53343836238751,48.24702757147,0
-0.53357498821363,48.247056258594,0
-0.53371484883597,48.247076906792,0
-0.53385687976758,48.24708935891,0
-0.53399999999998,48.247093520174,0
-0.53414312023241,48.24708935891,0
-0.53428515116402,48.247076906792,0
-0.53442501178636,48.247056258594,0
-0.53456163761245,48.24702757147,0
-0.53469398878023,48.246991063761,0
-0.53482105796805,48.246947013327,0

-0.53494187806274,48.24689575544,0
-0.53505552952096,48.246837680222,0
-0.53516114736845,48.246773229685,0
-0.53525792778357,48.246702894358,0
-0.53534513421496,48.24662720956,0
-0.53542210298711,48.246546751319,0
-0.5354882483506,48.246462131993,0
-0.53554306693928,48.246373995604,0
-0.53558614159987,48.24628301294,0
-0.53561714456523,48.246189876448,0
-0.5356358399473,48.246095294962,0
-0.53564208553022,48.24599998831,0
-0.53563583385076,48.245904681836,0
-0.53561713255737,48.245810100877,0
-0.53558612404554,48.245716965246,0
-0.53554304437187,48.245625983751,0
-0.53548822145583,48.245537848803,0
-0.53542207258212,48.245453231146,0
-0.53534510122363,48.245372774752,0
-0.53525789320829,48.245297091922,0
-0.53516111225981,48.245226758625,0
-0.53505549494569,48.245162310118,0
-0.53494184507141,48.245104236869,0
-0.53482102756307,48.245052980828,0
-0.53469396188543,48.245008932064,0
-0.53456161504507,48.244972425795,0
-0.53442499423203,48.24494373984,0
-0.53428513915615,48.244923092503,0
-0.53414311413587,48.244910640912,0
-0.53399999999998,48.244906479826,0
-0.53385688586412,48.244910640912,0
-0.53371486084381,48.244923092503,0
-0.53357500576796,48.24494373984,0
-0.53343838495492,48.244972425795,0
-0.53330603811456,48.245008932064,0
-0.53317897243692,48.245052980828,0
-0.53305815492858,48.245104236869,0
-0.5329445050543,48.245162310118,0
-0.53283888774018,48.245226758625,0
-0.5327421067917,48.245297091922,0
-0.53265489877636,48.245372774752,0
-0.53257792741787,48.245453231146,0
-0.53251177854416,48.245537848803,0
-0.53245695562809,48.245625983751,0
-0.53241387595444,48.245716965246,0
-0.5323828674426,48.245810100877,0
-0.53236416614923,48.245904681836,0
-0.53235791446977,48.24599998831,0
-0.53399999999999,48.246,0
</coordinates>
</LinearRing>
</outerBoundaryIs>
</Polygon>
</Placemark>
<Placemark>
<name>373</name>
<styleUrl>#stylehap2</styleUrl>

<Polygon>
<outerBoundaryIs>
<LinearRing>
<coordinates>
-0.53200000000004,48.246,0
-0.5303579144698,48.24599998831,0
-0.53036416005272,48.246095294962,0
-0.53038285543479,48.246189876448,0
-0.53041385840018,48.24628301294,0
-0.53045693306074,48.246373995604,0
-0.53051175164942,48.246462131993,0
-0.53057789701291,48.246546751319,0
-0.53065486578506,48.24662720956,0
-0.53074207221648,48.246702894358,0
-0.53083885263157,48.246773229685,0
-0.53094447047908,48.246837680222,0
-0.5310581219373,48.24689575544,0
-0.53117894203197,48.246947013327,0
-0.53130601121982,48.246991063761,0
-0.53143836238757,48.24702757147,0
-0.53157498821366,48.247056258594,0
-0.531714848836,48.247076906792,0
-0.53185687976761,48.24708935891,0
-0.53200000000004,48.247093520174,0
-0.53214312023247,48.24708935891,0
-0.53228515116407,48.247076906792,0
-0.53242501178641,48.247056258594,0
-0.53256163761251,48.24702757147,0
-0.53269398878026,48.246991063761,0
-0.5328210579681,48.246947013327,0
-0.53294187806277,48.24689575544,0
-0.53305552952099,48.246837680222,0
-0.53316114736851,48.246773229685,0
-0.53325792778362,48.246702894358,0
-0.53334513421502,48.24662720956,0
-0.53342210298716,48.246546751319,0
-0.53348824835065,48.246462131993,0
-0.53354306693933,48.246373995604,0
-0.5335861415999,48.24628301294,0
-0.53361714456529,48.246189876448,0
-0.53363583994736,48.246095294962,0
-0.53364208553027,48.24599998831,0
-0.53363583385079,48.245904681836,0
-0.53361713255742,48.245810100877,0
-0.53358612404558,48.245716965246,0
-0.53354304437193,48.245625983751,0
-0.53348822145586,48.245537848803,0
-0.53342207258218,48.245453231146,0
-0.53334510122368,48.245372774752,0
-0.53325789320835,48.245297091922,0
-0.53316111225986,48.245226758625,0
-0.53305549494574,48.245162310118,0
-0.53294184507144,48.245104236869,0
-0.53282102756312,48.245052980828,0
-0.53269396188549,48.245008932064,0
-0.5325616150451,48.244972425795,0
-0.53242499423209,48.24494373984,0

-0.53228513915621,48.244923092503,0
-0.5321431141359,48.244910640912,0
-0.53200000000004,48.244906479826,0
-0.53185688586417,48.244910640912,0
-0.53171486084387,48.244923092503,0
-0.53157500576801,48.24494373984,0
-0.53143838495498,48.244972425795,0
-0.53130603811459,48.245008932064,0
-0.53117897243695,48.245052980828,0
-0.53105815492864,48.245104236869,0
-0.53094450505433,48.245162310118,0
-0.53083888774021,48.245226758625,0
-0.53074210679173,48.245297091922,0
-0.53065489877639,48.245372774752,0
-0.5305779274179,48.245453231146,0
-0.53051177854422,48.245537848803,0
-0.53045695562815,48.245625983751,0
-0.5304138759545,48.245716965246,0
-0.53038286744265,48.245810100877,0
-0.53036416614928,48.245904681836,0
-0.5303579144698,48.24599998831,0
-0.53200000000004,48.246,0
</coordinates>
</LinearRing>
</outerBoundaryIs>
</Polygon>
</Placemark>
<Placemark>
<name>374</name>
<styleUrl>#stylehap2</styleUrl>
<Polygon>
<outerBoundaryIs>
<LinearRing>
<coordinates>
-0.52999999999997,48.23,0
-0.52835842794237,48.229999988317,0
-0.52836467157403,48.230095294968,0
-0.52838336111179,48.230189876454,0
-0.52841435438425,48.230283012946,0
-0.528457415577,48.23037399561,0
-0.52851221702536,48.230462131998,0
-0.5285783417065,48.230546751324,0
-0.52865528641159,48.230627209564,0
-0.52874246557438,48.230702894362,0
-0.52883921572685,48.230773229688,0
-0.52894480054797,48.230837680225,0
-0.52905841646746,48.230895755442,0
-0.52917919878154,48.230947013329,0
-0.52930622823441,48.230991063762,0
-0.52943853801536,48.231027571471,0
-0.52957512111778,48.231056258594,0
-0.52971493800477,48.231076906793,0
-0.52985692452229,48.23108935891,0
-0.52999999999997,48.231093520174,0
-0.53014307547764,48.23108935891,0
-0.53028506199517,48.231076906793,0
-0.53042487888216,48.231056258594,0

-0.5305614619846,48.231027571471,0
-0.53069377176552,48.230991063762,0
-0.53082080121842,48.230947013329,0
-0.5309415835325,48.230895755442,0
-0.53105519945196,48.230837680225,0
-0.53116078427311,48.230773229688,0
-0.53125753442558,48.230702894362,0
-0.53134471358837,48.230627209564,0
-0.53142165829346,48.230546751324,0
-0.53148778297457,48.230462131998,0
-0.53154258442296,48.23037399561,0
-0.53158564561568,48.230283012946,0
-0.53161663888814,48.230189876454,0
-0.5316353284259,48.230095294968,0
-0.53164157205756,48.229999988317,0
-0.53163532233468,48.229904681842,0
-0.53161662689078,48.229810100884,0
-0.5315856280767,48.229716965252,0
-0.53154256187527,48.229625983757,0
-0.53148775610331,48.229537848808,0
-0.53142162791507,48.229453231151,0
-0.53134468062589,48.229372774756,0
-0.53125749988053,48.229297091926,0
-0.53116074919515,48.229226758628,0
-0.53105516490694,48.22916231012,0
-0.53094155056999,48.229104236871,0
-0.53082077084002,48.229052980829,0
-0.53069374489426,48.229008932065,0
-0.53056143943691,48.228972425796,0
-0.53042486134318,48.22894373984,0
-0.53028504999781,48.228923092503,0
-0.53014306938642,48.228910640912,0
-0.52999999999997,48.228906479826,0
-0.52985693061351,48.228910640912,0
-0.52971495000215,48.228923092503,0
-0.52957513865676,48.22894373984,0
-0.52943856056303,48.228972425796,0
-0.5293062551057,48.229008932065,0
-0.52917922915991,48.229052980829,0
-0.52905844942994,48.229104236871,0
-0.52894483509302,48.22916231012,0
-0.52883925080478,48.229226758628,0
-0.52874250011941,48.229297091926,0
-0.52865531937407,48.229372774756,0
-0.52857837208489,48.229453231151,0
-0.52851224389662,48.229537848808,0
-0.52845743812466,48.229625983757,0
-0.52841437192323,48.229716965252,0
-0.52838337310918,48.229810100884,0
-0.52836467766525,48.229904681842,0
-0.52835842794237,48.229999988317,0
-0.52999999999997,48.23,0
</coordinates>
</LinearRing>
</outerBoundaryIs>
</Polygon>
</Placemark>

<Placemark>
<name>375</name>
<styleUrl>#stylehap2</styleUrl>
<Polygon>
<outerBoundaryIs>
<LinearRing>
<coordinates>
-0.52800000000002,48.239,0
-0.52635813916934,48.238999988313,0
-0.52636438389839,48.239095294965,0
-0.52638307672293,48.239189876451,0
-0.52641407544661,48.239283012943,0
-0.52645714421355,48.239373995606,0
-0.52651195530149,48.239462131995,0
-0.52657809161425,48.239546751321,0
-0.52665504985445,48.239627209562,0
-0.52674224435292,48.23970289436,0
-0.52683901152484,48.239773229686,0
-0.52694461491976,48.239837680223,0
-0.52705825082596,48.239895755441,0
-0.52717905438757,48.239947013328,0
-0.52730610618707,48.239991063761,0
-0.52743843924365,48.240027571471,0
-0.52757504637355,48.240056258594,0
-0.52771488785697,48.240076906793,0
-0.52785689935261,48.24008935891,0
-0.52800000000002,48.240093520174,0
-0.52814310064741,48.24008935891,0
-0.52828511214308,48.240076906793,0
-0.52842495362649,48.240056258594,0
-0.5285615607564,48.240027571471,0
-0.52869389381295,48.239991063761,0
-0.52882094561248,48.239947013328,0
-0.52894174917409,48.239895755441,0
-0.52905538508029,48.239837680223,0
-0.52916098847518,48.239773229686,0
-0.52925775564713,48.23970289436,0
-0.52934495014559,48.239627209562,0
-0.5294219083858,48.239546751321,0
-0.52948804469853,48.239462131995,0
-0.52954285578649,48.239373995606,0
-0.52958592455344,48.239283012943,0
-0.52961692327711,48.239189876451,0
-0.52963561610166,48.239095294965,0
-0.52964186083071,48.238999988313,0
-0.52963561000744,48.238904681839,0
-0.52961691127385,48.23881010088,0
-0.52958590700583,48.238716965249,0
-0.52954283322771,48.238625983754,0
-0.52948801781404,48.238537848805,0
-0.52942187799244,48.238453231148,0
-0.52934491716688,48.238372774754,0
-0.52925772108511,48.238297091923,0
-0.52916095337997,48.238226758626,0
-0.52905535051824,48.238162310119,0
-0.52894171619537,48.23810423687,0
-0.52882091521915,48.238052980828,0

-0.52869386692846,48.238008932064,0
-0.52856153819764,48.237972425795,0
-0.52842493607889,48.23794373984,0
-0.52828510013982,48.237923092503,0
-0.52814309455321,48.237910640912,0
-0.52800000000002,48.237906479826,0
-0.52785690544683,48.237910640912,0
-0.52771489986023,48.237923092503,0
-0.52757506392116,48.23794373984,0
-0.52743846180241,48.237972425795,0
-0.52730613307156,48.238008932064,0
-0.5271790847809,48.238052980828,0
-0.52705828380467,48.23810423687,0
-0.52694464948181,48.238162310119,0
-0.52683904662007,48.238226758626,0
-0.52674227891494,48.238297091923,0
-0.52665508283317,48.238372774754,0
-0.52657812200758,48.238453231148,0
-0.52651198218598,48.238537848805,0
-0.52645716677234,48.238625983754,0
-0.52641409299421,48.238716965249,0
-0.52638308872619,48.23881010088,0
-0.52636438999258,48.238904681839,0
-0.52635813916934,48.238999988313,0
-0.52800000000002,48.239,0
</coordinates>
</LinearRing>
</outerBoundaryIs>
</Polygon>
</Placemark>
<Placemark>
<name>376</name>
<styleUrl>#stylehap2</styleUrl>
<Polygon>
<outerBoundaryIs>
<LinearRing>
<coordinates>
-0.52800000000002,48.238,0
-0.52635817126225,48.237999988313,0
-0.52636441586932,48.238095294965,0
-0.52638310832861,48.238189876451,0
-0.52641410644646,48.238283012943,0
-0.52645717437165,48.238373995607,0
-0.5265119843883,48.238462131995,0
-0.52657811940835,48.238546751322,0
-0.52665507614433,48.238627209562,0
-0.52674226893845,48.23870289436,0
-0.52683903421894,48.238773229687,0
-0.52694463554963,48.238837680224,0
-0.5270582692346,48.238895755441,0
-0.52717907043484,48.238947013328,0
-0.52730611975086,48.238991063761,0
-0.52743845022068,48.239027571471,0
-0.52757505468027,48.239056258594,0
-0.52771489343016,48.239076906793,0
-0.52785690214987,48.23908935891,0
-0.52800000000002,48.239093520174,0

-0.52814309785018,48.23908935891,0
-0.52828510656988,48.239076906793,0
-0.52842494531975,48.239056258594,0
-0.52856154977936,48.239027571471,0
-0.52869388024918,48.238991063761,0
-0.5288209295652,48.238947013328,0
-0.52894173076544,48.238895755441,0
-0.52905536445042,48.238837680224,0
-0.52916096578111,48.238773229687,0
-0.52925773106159,48.23870289436,0
-0.52934492385571,48.238627209562,0
-0.52942188059169,48.238546751322,0
-0.52948801561174,48.238462131995,0
-0.5295428256284,48.238373995607,0
-0.52958589355356,48.238283012943,0
-0.52961689167143,48.238189876451,0
-0.5296355841307,48.238095294965,0
-0.52964182873779,48.237999988313,0
-0.52963557803683,48.237904681839,0
-0.52961687966881,48.23781010088,0
-0.52958587600692,48.237716965249,0
-0.52954280307086,48.237625983754,0
-0.52948798872873,48.237537848806,0
-0.52942185019999,48.237453231148,0
-0.52934489087881,48.237372774754,0
-0.52925769650143,48.237297091924,0
-0.52916093068781,48.237226758627,0
-0.52905532989026,48.237162310119,0
-0.52894169778854,48.23710423687,0
-0.5288208991735,48.237052980829,0
-0.52869385336614,48.237008932064,0
-0.52856152722182,48.236972425795,0
-0.52842492777311,48.23694373984,0
-0.52828509456726,48.236923092503,0
-0.52814309175628,48.236910640912,0
-0.52800000000002,48.236906479827,0
-0.52785690824376,48.236910640912,0
-0.52771490543276,48.236923092503,0
-0.52757507222693,48.23694373984,0
-0.52743847277822,48.236972425795,0
-0.52730614663388,48.237008932064,0
-0.52717910082652,48.237052980829,0
-0.52705830221151,48.23710423687,0
-0.52694467010979,48.237162310119,0
-0.52683906931221,48.237226758627,0
-0.52674230349859,48.237297091924,0
-0.52665510912124,48.237372774754,0
-0.52657814980003,48.237453231148,0
-0.52651201127132,48.237537848806,0
-0.52645719692919,48.237625983754,0
-0.52641412399312,48.237716965249,0
-0.52638312033121,48.23781010088,0
-0.52636442196321,48.237904681839,0
-0.52635817126225,48.237999988313,0
-0.52800000000002,48.238,0
</coordinates>
</LinearRing>

</outerBoundaryIs>
</Polygon>
</Placemark>
<Placemark>
<name>378</name>
<styleUrl>#stylehap2</styleUrl>
<Polygon>
<outerBoundaryIs>
<LinearRing>
<coordinates>
-0.52700000000004,48.245,0
-0.52535794657501,48.24499998831,0
-0.52536419203592,48.245095294962,0
-0.52538288705258,48.245189876448,0
-0.52541388941191,48.24528301294,0
-0.52545696323039,48.245373995604,0
-0.52551178074735,48.245462131993,0
-0.52557792481768,48.245546751319,0
-0.52565489208501,48.24562720956,0
-0.52574209681143,48.245702894359,0
-0.52583887533435,48.245773229685,0
-0.52594449111686,48.245837680222,0
-0.526058140353,48.24589575544,0
-0.52617895808543,48.245947013327,0
-0.5263060247888,48.245991063761,0
-0.52643837336883,48.24602757147,0
-0.52657499652359,48.246056258594,0
-0.52671485441136,48.246076906792,0
-0.52685688256594,48.24608935891,0
-0.52700000000004,48.246093520174,0
-0.52714311743414,48.24608935891,0
-0.52728514558874,48.246076906792,0
-0.52742500347649,48.246056258594,0
-0.52756162663127,48.24602757147,0
-0.52769397521127,48.245991063761,0
-0.52782104191468,48.245947013327,0
-0.52794185964711,48.24589575544,0
-0.52805550888324,48.245837680222,0
-0.52816112466573,48.245773229685,0
-0.52825790318865,48.245702894359,0
-0.52834510791509,48.24562720956,0
-0.5284220751824,48.245546751319,0
-0.52848821925273,48.245462131993,0
-0.52854303676969,48.245373995604,0
-0.52858611058819,48.24528301294,0
-0.52861711294752,48.245189876448,0
-0.52863580796416,48.245095294962,0
-0.52864205342507,48.24499998831,0
-0.52863580186795,48.244904681836,0
-0.52861710094029,48.244810100878,0
-0.52858609303481,48.244716965246,0
-0.52854301420352,48.244625983751,0
-0.52848819235941,48.244537848803,0
-0.52842204477909,48.244453231146,0
-0.52834507492554,48.244372774752,0
-0.52825786861528,48.244297091922,0
-0.52816108955902,48.244226758625,0

-0.52805547430987,48.244162310118,0
-0.52794182665755,48.244104236869,0
-0.52782101151135,48.244052980828,0
-0.52769394831798,48.244008932064,0
-0.52756160406511,48.243972425795,0
-0.52742498592313,48.24394373984,0
-0.52728513358154,48.243923092503,0
-0.52714311133793,48.243910640912,0
-0.52700000000004,48.243906479826,0
-0.52685688866217,48.243910640912,0
-0.52671486641857,48.243923092503,0
-0.52657501407697,48.24394373984,0
-0.52643839593499,48.243972425795,0
-0.52630605168212,48.244008932064,0
-0.52617898848873,48.244052980828,0
-0.52605817334252,48.244104236869,0
-0.52594452569023,48.244162310118,0
-0.52583891044108,48.244226758625,0
-0.5257421313848,48.244297091922,0
-0.52565492507454,48.244372774752,0
-0.52557795522098,48.244453231146,0
-0.52551180764067,48.244537848803,0
-0.52545698579655,48.244625983751,0
-0.52541390696527,48.244716965246,0
-0.52538289905979,48.244810100878,0
-0.52536419813215,48.244904681836,0
-0.52535794657501,48.24499998831,0
-0.52700000000004,48.245,0
</coordinates>
</LinearRing>
</outerBoundaryIs>
</Polygon>
</Placemark>
<Placemark>
<name>379</name>
<styleUrl>#stylehap2</styleUrl>
<Polygon>
<outerBoundaryIs>
<LinearRing>
<coordinates>
-0.52700000000004,48.237,0
-0.52535820335343,48.236999988314,0
-0.52536444783857,48.237095294965,0
-0.52538313993258,48.237189876451,0
-0.52541413744464,48.237283012944,0
-0.52545720452811,48.237373995607,0
-0.52551201347352,48.237462131996,0
-0.52557814720097,48.237546751322,0
-0.5256551024328,48.237627209562,0
-0.52574229352268,48.237702894361,0
-0.52583905691178,48.237773229687,0
-0.52594465617841,48.237837680224,0
-0.52605828764227,48.237895755441,0
-0.52617908648129,48.237947013328,0
-0.52630613331393,48.237991063761,0
-0.52643846119715,48.238027571471,0
-0.5265750629866,48.238056258594,0

-0.52671489900307,48.238076906793,0
-0.52685690494699,48.23808935891,0
-0.52700000000004,48.238093520174,0
-0.52714309505311,48.23808935891,0
-0.52728510099701,48.238076906793,0
-0.52742493701351,48.238056258594,0
-0.52756153880293,48.238027571471,0
-0.52769386668614,48.237991063761,0
-0.52782091351881,48.237947013328,0
-0.52794171235783,48.237895755441,0
-0.52805534382169,48.237837680224,0
-0.5281609430883,48.237773229687,0
-0.52825770647742,48.237702894361,0
-0.5283448975673,48.237627209562,0
-0.52842185279911,48.237546751322,0
-0.52848798652655,48.237462131996,0
-0.52854279547197,48.237373995607,0
-0.52858586255543,48.237283012944,0
-0.5286168600675,48.237189876451,0
-0.52863555216151,48.237095294965,0
-0.52864179664665,48.236999988314,0
-0.52863554606797,48.23690468184,0
-0.52861684806553,48.236810100881,0
-0.52858584500974,48.236716965249,0
-0.52854277291568,48.236625983754,0
-0.52848795964501,48.236537848806,0
-0.52842182240911,48.236453231149,0
-0.5283448645922,48.236372774754,0
-0.52825767191917,48.236297091924,0
-0.52816090799693,48.236226758627,0
-0.52805530926343,48.236162310119,0
-0.52794167938273,48.23610423687,0
-0.52782088312879,48.236052980829,0
-0.5276938398046,48.236008932064,0
-0.52756151624664,48.235972425795,0
-0.52742491946781,48.23594373984,0
-0.52728508899504,48.235923092503,0
-0.52714308895955,48.235910640912,0
-0.52700000000004,48.235906479827,0
-0.52685691104053,48.235910640912,0
-0.52671491100503,48.235923092503,0
-0.52657508053227,48.23594373984,0
-0.52643848375344,48.235972425795,0
-0.52630616019547,48.236008932064,0
-0.52617911687131,48.236052980829,0
-0.52605832061737,48.23610423687,0
-0.52594469073667,48.236162310119,0
-0.52583909200317,48.236226758627,0
-0.52574232808093,48.236297091924,0
-0.5256551354079,48.236372774754,0
-0.52557817759099,48.236453231149,0
-0.52551204035509,48.236537848806,0
-0.52545722708442,48.236625983754,0
-0.52541415499034,48.236716965249,0
-0.52538315193454,48.236810100881,0
-0.52536445393213,48.23690468184,0
-0.52535820335343,48.236999988314,0

-0.52700000000004,48.237,0
</coordinates>
</LinearRing>
</outerBoundaryIs>
</Polygon>
</Placemark>
<Placemark>
<name>382</name>
<styleUrl>#stylehap2</styleUrl>
<Polygon>
<outerBoundaryIs>
<LinearRing>
<coordinates>
-0.52600000000007,48.234,0
-0.52435829961642,48.233999988315,0
-0.52436454373574,48.234095294967,0
-0.52438323473409,48.234189876453,0
-0.52441423042899,48.234283012945,0
-0.52445729498758,48.234373995608,0
-0.52451210071961,48.234462131997,0
-0.52457823056966,48.234546751323,0
-0.52465518128953,48.234627209563,0
-0.52474236726724,48.234702894361,0
-0.52483912498289,48.234773229687,0
-0.5249447180579,48.234837680224,0
-0.52505834285916,48.234895755441,0
-0.52517913461529,48.234947013328,0
-0.52530617399864,48.234991063761,0
-0.5254384941229,48.235027571471,0
-0.52557508790272,48.235056258594,0
-0.52571491571998,48.235076906793,0
-0.52585691333739,48.23508935891,0
-0.52600000000008,48.235093520174,0
-0.52614308666274,48.23508935891,0
-0.52628508428015,48.235076906793,0
-0.52642491209741,48.235056258594,0
-0.52656150587725,48.235027571471,0
-0.5266938260015,48.234991063761,0
-0.52682086538485,48.234947013328,0
-0.52694165714098,48.234895755441,0
-0.52705528194223,48.234837680224,0
-0.52716087501724,48.234773229687,0
-0.52725763273289,48.234702894361,0
-0.5273448187106,48.234627209563,0
-0.52742176943047,48.234546751323,0
-0.52748789928052,48.234462131997,0
-0.52754270501256,48.234373995608,0
-0.52758576957114,48.234283012945,0
-0.52761676526604,48.234189876453,0
-0.52763545626439,48.234095294967,0
-0.52764170038372,48.233999988315,0
-0.52763545017185,48.233904681841,0
-0.52761675326604,48.233810100882,0
-0.52758575202832,48.233716965251,0
-0.52754268245996,48.233625983755,0
-0.52748787240338,48.233537848807,0
-0.52742173904544,48.23345323115,0

-0.52734478574092,48.233372774755,0
-0.52725759818031,48.233297091925,0
-0.52716083993163,48.233226758628,0
-0.52705524738965,48.23316231012,0
-0.5269416241713,48.23310423687,0
-0.52682083499981,48.233052980829,0
-0.52669379912436,48.233008932064,0
-0.52656148332465,48.232972425795,0
-0.52642489455459,48.23294373984,0
-0.52628507228018,48.232923092503,0
-0.5261430805702,48.232910640912,0
-0.52600000000008,48.232906479827,0
-0.52585691942993,48.232910640912,0
-0.52571492771996,48.232923092503,0
-0.52557510544554,48.23294373984,0
-0.5254385166755,48.232972425795,0
-0.52530620087577,48.233008932064,0
-0.52517916500032,48.233052980829,0
-0.52505837582884,48.23310423687,0
-0.52494475261048,48.23316231012,0
-0.52483916006851,48.233226758628,0
-0.52474240181982,48.233297091925,0
-0.52465521425921,48.233372774755,0
-0.52457826095469,48.23345323115,0
-0.52451212759675,48.233537848807,0
-0.52445731754018,48.233625983755,0
-0.52441424797181,48.233716965251,0
-0.5243832467341,48.233810100882,0
-0.52436454982829,48.233904681841,0
-0.52435829961642,48.233999988315,0
-0.52600000000007,48.234,0
</coordinates>
</LinearRing>
</outerBoundaryIs>
</Polygon>
</Placemark>
<Placemark>
<name>384</name>
<styleUrl>#stylehap2</styleUrl>
<Polygon>
<outerBoundaryIs>
<LinearRing>
<coordinates>
-0.524,48.243,0
-0.5223580107801,48.242999988311,0
-0.52236425599705,48.243095294963,0
-0.52238295028292,48.243189876449,0
-0.52241395143023,48.243283012941,0
-0.52245702356467,48.243373995605,0
-0.52251183893839,48.243462131994,0
-0.52257798042258,48.24354675132,0
-0.52265494468054,48.243627209561,0
-0.52274214599728,48.243702894359,0
-0.52283892073612,48.243773229686,0
-0.52294453238896,48.243837680223,0
-0.5230581771813,48.24389575544,0
-0.52317899018961,48.243947013328,0

-0.5233060519245,48.243991063761,0
-0.52343839532946,48.24402757147,0
-0.52357501314203,48.244056258594,0
-0.52371486556106,48.244076906792,0
-0.52385688816208,48.24408935891,0
-0.52400000000001,48.244093520174,0
-0.52414311183793,48.24408935891,0
-0.52428513443893,48.244076906792,0
-0.52442498685798,48.244056258594,0
-0.52456160467053,48.24402757147,0
-0.52469394807552,48.243991063761,0
-0.52482100981038,48.243947013328,0
-0.5249418228187,48.24389575544,0
-0.52505546761103,48.243837680223,0
-0.52516107926389,48.243773229686,0
-0.52525785400274,48.243702894359,0
-0.52534505531947,48.243627209561,0
-0.52542201957742,48.24354675132,0
-0.5254881610616,48.243462131994,0
-0.52554297643532,48.243373995605,0
-0.52558604856979,48.243283012941,0
-0.5256170497171,48.243189876449,0
-0.52563574400297,48.243095294963,0
-0.52564198921989,48.242999988311,0
-0.52563573790742,48.242904681837,0
-0.52561703771119,48.242810100878,0
-0.52558603101834,48.242716965247,0
-0.52554295387163,48.242625983752,0
-0.52548813417123,48.242537848804,0
-0.52542198917742,48.242453231147,0
-0.52534502233353,48.242372774753,0
-0.52525781943314,48.242297091922,0
-0.525161044161,48.242226758626,0
-0.52505543304143,48.242162310118,0
-0.52494178983278,48.242104236869,0
-0.52482097941041,48.242052980828,0
-0.52469392118515,48.242008932064,0
-0.52456158210684,48.241972425795,0
-0.52442496930654,48.24194373984,0
-0.52428512243305,48.241923092503,0
-0.52414310574236,48.241910640912,0
-0.52400000000001,48.241906479826,0
-0.52385689425763,48.241910640912,0
-0.52371487756697,48.241923092503,0
-0.52357503069348,48.24194373984,0
-0.52343841789316,48.241972425795,0
-0.52330607881487,48.242008932064,0
-0.52317902058961,48.242052980828,0
-0.52305821016723,48.242104236869,0
-0.52294456695856,48.242162310118,0
-0.52283895583899,48.242226758626,0
-0.52274218056685,48.242297091922,0
-0.52265497766646,48.242372774753,0
-0.52257801082257,48.242453231147,0
-0.52251186582879,48.242537848804,0
-0.52245704612839,48.242625983752,0
-0.52241396898168,48.242716965247,0

-0.5223829622888,48.242810100878,0
-0.52236426209259,48.242904681837,0
-0.5223580107801,48.242999988311,0
-0.524,48.243,0
</coordinates>
</LinearRing>
</outerBoundaryIs>
</Polygon>
</Placemark>
<Placemark>
<name>385</name>
<styleUrl>#stylehap2</styleUrl>
<Polygon>
<outerBoundaryIs>
<LinearRing>
<coordinates>
-0.524,48.17,0
-0.52236034947038,48.169999988341,0
-0.52236658580002,48.170095294993,0
-0.52238525346711,48.170189876478,0
-0.52241621046649,48.170283012969,0
-0.52245922125958,48.170373995631,0
-0.52251395856514,48.170462132018,0
-0.52258000584827,48.170546751343,0
-0.52265686048911,48.170627209581,0
-0.52274393760676,48.170702894377,0
-0.52284057450975,48.170773229701,0
-0.52294603573891,48.170837680235,0
-0.52305951866461,48.17089575545,0
-0.52318015959527,48.170947013335,0
-0.52330704035123,48.170991063766,0
-0.52343919525357,48.171027571474,0
-0.52357561847454,48.171056258596,0
-0.52371527169428,48.171076906793,0
-0.52385709200449,48.171089358911,0
-0.52400000000001,48.171093520174,0
-0.5241429079955,48.171089358911,0
-0.52428472830574,48.171076906793,0
-0.52442438152545,48.171056258596,0
-0.52456080474645,48.171027571474,0
-0.52469295964876,48.170991063766,0
-0.52481984040475,48.170947013335,0
-0.52494048133541,48.17089575545,0
-0.5250539642611,48.170837680235,0
-0.52515942549027,48.170773229701,0
-0.52525606239323,48.170702894377,0
-0.52534313951088,48.170627209581,0
-0.52541999415172,48.170546751343,0
-0.52548604143487,48.170462132018,0
-0.52554077874043,48.170373995631,0
-0.52558378953352,48.170283012969,0
-0.5256147465329,48.170189876478,0
-0.5256334142,48.170095294993,0
-0.52563965052964,48.169999988341,0
-0.5256334081287,48.169904681867,0
-0.52561473457481,48.169810100907,0
-0.52558377205195,48.169716965275,0

-0.52554075626656,48.169625983779,0
-0.52548601465157,48.169537848829,0
-0.52541996387277,48.169453231169,0
-0.52534310665629,48.169372774773,0
-0.52525602796129,48.16929709194,0
-0.52515939052714,48.169226758641,0
-0.52505392982913,48.169162310131,0
-0.52494044848081,48.169104236879,0
-0.52481981012579,48.169052980836,0
-0.52469293286546,48.169008932069,0
-0.52456078227257,48.168972425798,0
-0.5244243640439,48.168943739842,0
-0.52428471634764,48.168923092504,0
-0.52414290192423,48.168910640912,0
-0.52400000000001,48.168906479827,0
-0.52385709807579,48.168910640912,0
-0.52371528365238,48.168923092504,0
-0.52357563595611,48.168943739842,0
-0.52343921772742,48.168972425798,0
-0.52330706713455,48.169008932069,0
-0.5231801898742,48.169052980836,0
-0.52305955151918,48.169104236879,0
-0.52294607017086,48.169162310131,0
-0.52284060947285,48.169226758641,0
-0.52274397203873,48.16929709194,0
-0.5226568933437,48.169372774773,0
-0.52258003612722,48.169453231169,0
-0.52251398534844,48.169537848829,0
-0.52245924373343,48.169625983779,0
-0.52241622794804,48.169716965275,0
-0.52238526542521,48.169810100907,0
-0.52236659187129,48.169904681867,0
-0.52236034947038,48.169999988341,0
-0.524,48.17,0
</coordinates>
</LinearRing>
</outerBoundaryIs>
</Polygon>
</Placemark>
<Placemark>
<name>387</name>
<styleUrl>#stylehap2</styleUrl>
<Polygon>
<outerBoundaryIs>
<LinearRing>
<coordinates>
-0.52300000000002,48.241,0
-0.52135807497824,48.240999988312,0
-0.52136431995122,48.241095294964,0
-0.52138301350638,48.24118987645,0
-0.52141401344182,48.241283012942,0
-0.52145708389243,48.241373995606,0
-0.52151189712314,48.241462131994,0
-0.52157803602146,48.241546751321,0
-0.52165499727039,48.241627209561,0
-0.52174219517778,48.24170289436,0
-0.52183896613298,48.241773229686,0

-0.52194457365664,48.241837680223,0
-0.52205821400567,48.24189575544,0
-0.52217902229035,48.241947013328,0
-0.52230607905729,48.241991063761,0
-0.52243841728777,48.24202757147,0
-0.52257502975872,48.242056258594,0
-0.52271487670963,48.242076906792,0
-0.52285689375766,48.24208935891,0
-0.52300000000002,48.242093520174,0
-0.52314310624239,48.24208935891,0
-0.52328512329042,48.242076906792,0
-0.52342497024133,48.242056258594,0
-0.52356158271228,48.24202757147,0
-0.52369392094278,48.241991063761,0
-0.52382097770969,48.241947013328,0
-0.5239417859944,48.24189575544,0
-0.52405542634343,48.241837680223,0
-0.52416103386706,48.241773229686,0
-0.52425780482227,48.24170289436,0
-0.52434500272965,48.241627209561,0
-0.52442196397859,48.241546751321,0
-0.5244881028769,48.241462131994,0
-0.52454291610762,48.241373995606,0
-0.52458598655822,48.241283012942,0
-0.52461698649367,48.24118987645,0
-0.52463568004882,48.241095294964,0
-0.52464192502181,48.240999988312,0
-0.52463567395394,48.240904681838,0
-0.52461697448908,48.240810100879,0
-0.52458596900871,48.240716965248,0
-0.52454289354639,48.240625983753,0
-0.52448807598946,48.240537848805,0
-0.52442193358192,48.240453231147,0
-0.52434496974735,48.240372774753,0
-0.52425777025643,48.240297091923,0
-0.52416099876801,48.240226758626,0
-0.52405539177759,48.240162310119,0
-0.52394175301207,48.240104236869,0
-0.52382094731303,48.240052980828,0
-0.52369389405534,48.240008932064,0
-0.52356156015105,48.239972425795,0
-0.52342495269182,48.23994373984,0
-0.52328511128583,48.239923092503,0
-0.5231431001475,48.239910640912,0
-0.52300000000002,48.239906479826,0
-0.52285689985254,48.239910640912,0
-0.52271488871421,48.239923092503,0
-0.52257504730823,48.23994373984,0
-0.522438439849,48.239972425795,0
-0.52230610594471,48.240008932064,0
-0.52217905268702,48.240052980828,0
-0.52205824698797,48.240104236869,0
-0.52194460822245,48.240162310119,0
-0.52183900123203,48.240226758626,0
-0.52174222974361,48.240297091923,0
-0.5216550302527,48.240372774753,0
-0.52157806641812,48.240453231147,0

-0.52151192401059,48.240537848805,0
-0.52145710645365,48.240625983753,0
-0.52141403099134,48.240716965248,0
-0.52138302551097,48.240810100879,0
-0.52136432604611,48.240904681838,0
-0.52135807497824,48.240999988312,0
-0.52300000000002,48.241,0
</coordinates>
</LinearRing>
</outerBoundaryIs>
</Polygon>
</Placemark>
<Placemark>
<name>390</name>
<styleUrl>#stylehap2</styleUrl>
<Polygon>
<outerBoundaryIs>
<LinearRing>
<coordinates>
-0.52199999999993,48.328,0
-0.52035527585727,48.327999988276,0
-0.5203615314672,48.328095294928,0
-0.52038025688169,48.328189876415,0
-0.52041130965671,48.328283012909,0
-0.52045445352525,48.328373995574,0
-0.52050936019384,48.328462131965,0
-0.52057561183906,48.328546751294,0
-0.520652704286,48.328627209537,0
-0.52074005084421,48.328702894339,0
-0.52083698677179,48.328773229668,0
-0.52094277433385,48.328837680208,0
-0.52105660841708,48.328895755429,0
-0.52117762265728,48.328947013319,0
-0.52130489603343,48.328991063755,0
-0.52143745987811,48.329027571466,0
-0.52157430525094,48.329056258592,0
-0.52171439061865,48.329076906791,0
-0.52185664978358,48.32908935891,0
-0.52199999999994,48.329093520174,0
-0.52214335021629,48.32908935891,0
-0.52228560938122,48.329076906791,0
-0.52242569474893,48.329056258592,0
-0.52256254012174,48.329027571466,0
-0.52269510396644,48.328991063755,0
-0.52282237734257,48.328947013319,0
-0.52294339158277,48.328895755429,0
-0.52305722566602,48.328837680208,0
-0.52316301322809,48.328773229668,0
-0.52325994915566,48.328702894339,0
-0.52334729571387,48.328627209537,0
-0.52342438816082,48.328546751294,0
-0.52349063980603,48.328462131965,0
-0.5235455464746,48.328373995574,0
-0.52358869034317,48.328283012909,0
-0.52361974311818,48.328189876415,0
-0.52363846853268,48.328095294928,0
-0.52364472414261,48.327999988276,0

-0.52363846240871,48.327904681802,0
-0.5236197310563,48.327810100844,0
-0.52358867270992,48.327716965215,0
-0.52354552380572,48.327625983721,0
-0.5234906127903,48.327537848775,0
-0.52342435761912,48.327453231121,0
-0.52334726257417,48.327372774729,0
-0.52325991442489,48.327297091902,0
-0.52316297796156,48.327226758608,0
-0.52305719093528,48.327162310104,0
-0.52294335844309,48.327104236857,0
-0.52282234680087,48.327052980819,0
-0.52269507695071,48.327008932058,0
-0.52256251745286,48.326972425791,0
-0.52242567711565,48.326943739838,0
-0.52228559731933,48.326923092502,0
-0.52214334409232,48.326910640912,0
-0.52199999999994,48.326906479826,0
-0.52185665590755,48.326910640912,0
-0.52171440268051,48.326923092502,0
-0.52157432288422,48.326943739838,0
-0.52143748254701,48.326972425791,0
-0.52130492304916,48.327008932058,0
-0.521177653199,48.327052980819,0
-0.52105664155678,48.327104236857,0
-0.52094280906459,48.327162310104,0
-0.52083702203831,48.327226758608,0
-0.52074008557498,48.327297091902,0
-0.5206527374257,48.327372774729,0
-0.52057564238075,48.327453231121,0
-0.52050938720958,48.327537848775,0
-0.52045447619416,48.327625983721,0
-0.52041132728996,48.327716965215,0
-0.52038026894355,48.327810100844,0
-0.52036153759116,48.327904681802,0
-0.52035527585727,48.327999988276,0
-0.52199999999993,48.328,0
</coordinates>
</LinearRing>
</outerBoundaryIs>
</Polygon>
</Placemark>
<Placemark>
<name>393</name>
<styleUrl>#stylehap2</styleUrl>
<Polygon>
<outerBoundaryIs>
<LinearRing>
<coordinates>
-0.52099999999996,48.249,0
-0.51935781814359,48.248999988309,0
-0.51936406409255,48.24909529496,0
-0.51938276057099,48.249189876447,0
-0.51941376535475,48.249283012939,0
-0.51945684254187,48.249373995603,0
-0.51951166434601,48.249462131992,0
-0.51957781358948,48.249546751318,0

-0.51965478687653,48.249627209559,0
-0.51974199842345,48.249702894358,0
-0.51983878451576,48.249773229684,0
-0.51994440855892,48.249837680222,0
-0.52005806668411,48.249895755439,0
-0.52017889386633,48.249947013327,0
-0.52030597050835,48.24999106376,0
-0.52043832944017,48.25002757147,0
-0.52057496328111,48.250056258594,0
-0.52071483210808,48.250076906792,0
-0.52085687137165,48.25008935891,0
-0.52099999999995,48.250093520174,0
-0.52114312862826,48.25008935891,0
-0.52128516789182,48.250076906792,0
-0.5214250367188,48.250056258594,0
-0.52156167055976,48.25002757147,0
-0.52169402949158,48.24999106376,0
-0.52182110613358,48.249947013327,0
-0.52194193331582,48.249895755439,0
-0.52205559144101,48.249837680222,0
-0.52216121548417,48.249773229684,0
-0.52225800157648,48.249702894358,0
-0.5223452131234,48.249627209559,0
-0.52242218641046,48.249546751318,0
-0.52248833565392,48.249462131992,0
-0.52254315745806,48.249373995603,0
-0.52258623464518,48.249283012939,0
-0.52261723942891,48.249189876447,0
-0.52263593590735,48.24909529496,0
-0.52264218185634,48.248999988309,0
-0.5226359298098,48.248904681835,0
-0.52261722741908,48.248810100876,0
-0.52258621708796,48.248716965245,0
-0.52254313488697,48.24862598375,0
-0.52248830875472,48.248537848802,0
-0.52242215600048,48.248453231145,0
-0.52234518012665,48.248372774751,0
-0.52225796699553,48.248297091921,0
-0.52216118036975,48.248226758624,0
-0.52205555686006,48.248162310117,0
-0.52194190031906,48.248104236868,0
-0.52182107572361,48.248052980827,0
-0.52169400259238,48.248008932063,0
-0.52156164798866,48.247972425795,0
-0.5214250191616,48.24794373984,0
-0.521285155882,48.247923092503,0
-0.5211431225307,48.247910640912,0
-0.52099999999995,48.247906479826,0
-0.5208568774692,48.247910640912,0
-0.52071484411791,48.247923092503,0
-0.52057498083833,48.24794373984,0
-0.52043835201127,48.247972425795,0
-0.52030599740755,48.248008932063,0
-0.5201789242763,48.248052980827,0
-0.52005809968084,48.248104236868,0
-0.51994444313984,48.248162310117,0
-0.51983881963015,48.248226758624,0

-0.51974203300438,48.248297091921,0
-0.51965481987326,48.248372774751,0
-0.51957784399942,48.248453231145,0
-0.51951169124521,48.248537848802,0
-0.51945686511296,48.24862598375,0
-0.51941378291194,48.248716965245,0
-0.51938277258082,48.248810100876,0
-0.51936407019011,48.248904681835,0
-0.51935781814359,48.248999988309,0
-0.52099999999996,48.249,0
</coordinates>
</LinearRing>
</outerBoundaryIs>
</Polygon>
</Placemark>
<Placemark>
<name>395</name>
<styleUrl>#stylehap2</styleUrl>
<Polygon>
<outerBoundaryIs>
<LinearRing>
<coordinates>
-0.52099999999996,48.327,0
-0.51935530810686,48.326999988277,0
-0.51936156359422,48.327095294929,0
-0.51938028864168,48.327189876415,0
-0.51941134080789,48.327283012909,0
-0.51945448383061,48.327373995574,0
-0.51950938942265,48.327462131965,0
-0.51957563976885,48.327546751294,0
-0.51965273070424,48.327627209538,0
-0.51974007554981,48.327702894339,0
-0.51983700957666,48.327773229668,0
-0.51994279506444,48.327837680208,0
-0.52005662691561,48.327895755429,0
-0.52017763878294,48.327947013319,0
-0.52030490966345,48.327991063755,0
-0.52043747090877,48.328027571466,0
-0.52057431359826,48.328056258592,0
-0.52071439621909,48.328076906791,0
-0.5208566525945,48.32808935891,0
-0.52099999999995,48.328093520173,0
-0.52114334740541,48.32808935891,0
-0.52128560378081,48.328076906791,0
-0.52142568640167,48.328056258592,0
-0.52156252909116,48.328027571466,0
-0.52169509033646,48.327991063755,0
-0.52182236121697,48.327947013319,0
-0.5219433730843,48.327895755429,0
-0.52205720493546,48.327837680208,0
-0.52216299042324,48.327773229668,0
-0.52225992445012,48.327702894339,0
-0.52234726929567,48.327627209538,0
-0.52242436023106,48.327546751294,0
-0.52249061057726,48.327462131965,0
-0.52254551616932,48.327373995574,0
-0.52258865919202,48.327283012909,0

-0.52261971135822,48.327189876415,0
-0.52263843640568,48.327095294929,0
-0.52264469189305,48.326999988277,0
-0.52263843028204,48.326904681803,0
-0.52261969929702,48.326810100845,0
-0.52258864155971,48.326716965215,0
-0.52254549350169,48.326625983722,0
-0.52249058356302,48.326537848776,0
-0.52242432969102,48.326453231121,0
-0.5223472361578,48.326372774729,0
-0.52225988972126,48.326297091902,0
-0.52216295515865,48.326226758608,0
-0.52205717020663,48.326162310104,0
-0.52194333994643,48.326104236858,0
-0.52182233067695,48.326052980819,0
-0.5216950633222,48.326008932058,0
-0.5215625064235,48.325972425791,0
-0.52142566876936,48.325943739838,0
-0.52128559171961,48.325923092502,0
-0.52114334128177,48.325910640912,0
-0.52099999999995,48.325906479826,0
-0.52085665871813,48.325910640912,0
-0.52071440828029,48.325923092502,0
-0.52057433123055,48.325943739838,0
-0.5204374935764,48.325972425791,0
-0.5203049366777,48.326008932058,0
-0.52017766932298,48.326052980819,0
-0.5200566600535,48.326104236858,0
-0.5199428297933,48.326162310104,0
-0.51983704484125,48.326226758608,0
-0.51974011027864,48.326297091902,0
-0.51965276384211,48.326372774729,0
-0.51957567030889,48.326453231121,0
-0.51950941643691,48.326537848776,0
-0.51945450649824,48.326625983722,0
-0.5194113584402,48.326716965215,0
-0.51938030070288,48.326810100845,0
-0.51936156971786,48.326904681803,0
-0.51935530810686,48.326999988277,0
-0.52099999999996,48.327,0
</coordinates>
</LinearRing>
</outerBoundaryIs>
</Polygon>
</Placemark>
<Placemark>
<name>396</name>
<styleUrl>#stylehap2</styleUrl>
<Polygon>
<outerBoundaryIs>
<LinearRing>
<coordinates>
-0.52099999999996,48.232,0
-0.51935836378284,48.231999988316,0
-0.51936460765833,48.232095294967,0
-0.51938329792634,48.232189876453,0
-0.51941429240996,48.232283012945,0

-0.51945735528552,48.232373995609,0
-0.5195121588756,48.232462131997,0
-0.51957828614105,48.232546751323,0
-0.51965523385336,48.232627209564,0
-0.51974241642343,48.232702894362,0
-0.51983917035727,48.232773229688,0
-0.51994475930512,48.232837680225,0
-0.52005837966525,48.232895755442,0
-0.52017916670009,48.232947013329,0
-0.52030620111796,48.232991063762,0
-0.52043851607026,48.233027571471,0
-0.5205751045111,48.233056258594,0
-0.52071492686291,48.233076906793,0
-0.52085691893008,48.23308935891,0
-0.52099999999995,48.233093520174,0
-0.52114308106983,48.23308935891,0
-0.52128507313699,48.233076906793,0
-0.52142489548881,48.233056258594,0
-0.52156148392965,48.233027571471,0
-0.52169379888197,48.232991063762,0
-0.52182083329982,48.232947013329,0
-0.52194162033466,48.232895755442,0
-0.52205524069479,48.232837680225,0
-0.52216082964264,48.232773229688,0
-0.52225758357648,48.232702894362,0
-0.52234476614657,48.232627209564,0
-0.52242171385886,48.232546751323,0
-0.52248784112431,48.232462131997,0
-0.52254264471438,48.232373995609,0
-0.52258570758997,48.232283012945,0
-0.52261670207356,48.232189876453,0
-0.5226353923416,48.232095294967,0
-0.52264163621707,48.231999988316,0
-0.5226353862497,48.231904681842,0
-0.5226166900749,48.231810100883,0
-0.52258569004908,48.231716965251,0
-0.52254262216425,48.231625983756,0
-0.52248781425009,48.231537848808,0
-0.52242168347715,48.23145323115,0
-0.52234473318047,48.231372774756,0
-0.52225754902767,48.231297091925,0
-0.52216079456086,48.231226758628,0
-0.52205520614597,48.23116231012,0
-0.52194158736856,48.23110423687,0
-0.52182080291812,48.231052980829,0
-0.52169377200776,48.231008932065,0
-0.52156146137951,48.230972425796,0
-0.52142487794792,48.23094373984,0
-0.52128506113831,48.230923092503,0
-0.52114307497795,48.230910640912,0
-0.52099999999995,48.230906479826,0
-0.52085692502198,48.230910640912,0
-0.52071493886159,48.230923092503,0
-0.52057512205201,48.23094373984,0
-0.52043853862039,48.230972425796,0
-0.52030622799217,48.231008932065,0
-0.52017919708181,48.231052980829,0

-0.52005841263134,48.23110423687,0
-0.51994479385393,48.23116231012,0
-0.51983920543907,48.231226758628,0
-0.51974245097224,48.231297091925,0
-0.51965526681946,48.231372774756,0
-0.51957831652275,48.23145323115,0
-0.51951218574981,48.231537848808,0
-0.51945737783566,48.231625983756,0
-0.51941430995085,48.231716965251,0
-0.51938330992503,48.231810100883,0
-0.51936461375021,48.231904681842,0
-0.51935836378284,48.231999988316,0
-0.52099999999996,48.232,0
</coordinates>
</LinearRing>
</outerBoundaryIs>
</Polygon>
</Placemark>
<Placemark>
<name>398</name>
<styleUrl>#stylehap2</styleUrl>
<Polygon>
<outerBoundaryIs>
<LinearRing>
<coordinates>
-0.51999999999998,48.233,0
-0.51835833170047,48.232999988315,0
-0.51836457569786,48.233095294967,0
-0.51838326633106,48.233189876453,0
-0.51841426142028,48.233283012945,0
-0.51845732513734,48.233373995609,0
-0.51851212979837,48.233462131997,0
-0.51857825835607,48.233546751323,0
-0.51865520757213,48.233627209564,0
-0.51874239184597,48.233702894361,0
-0.51883914767067,48.233773229688,0
-0.51894473868203,48.233837680224,0
-0.51905836126268,48.233895755441,0
-0.5191791506581,48.233947013329,0
-0.51930618755863,48.233991063762,0
-0.51943850509685,48.234027571471,0
-0.51957509620711,48.234056258594,0
-0.51971492129156,48.234076906793,0
-0.51985691613377,48.23408935891,0
-0.51999999999999,48.234093520174,0
-0.52014308386619,48.23408935891,0
-0.5202850787084,48.234076906793,0
-0.52042490379285,48.234056258594,0
-0.52056149490311,48.234027571471,0
-0.52069381244133,48.233991063762,0
-0.52082084934187,48.233947013329,0
-0.52094163873728,48.233895755441,0
-0.52105526131793,48.233837680224,0
-0.52116085232929,48.233773229688,0
-0.52125760815399,48.233702894361,0
-0.52134479242783,48.233627209564,0
-0.52142174164389,48.233546751323,0

-0.52148787020159,48.233462131997,0
-0.52154267486262,48.233373995609,0
-0.52158573857968,48.233283012945,0
-0.5216167336689,48.233189876453,0
-0.5216354243021,48.233095294967,0
-0.52164166829949,48.232999988315,0
-0.52163541820988,48.232904681841,0
-0.52161672166958,48.232810100882,0
-0.52158572103783,48.232716965251,0
-0.52154265231124,48.232625983756,0
-0.5214878433259,48.232537848807,0
-0.52142171126051,48.23245323115,0
-0.52134475945994,48.232372774755,0
-0.52125757360329,48.232297091925,0
-0.52116081724559,48.232226758628,0
-0.52105522676723,48.23216231012,0
-0.52094160576938,48.23210423687,0
-0.52082081895848,48.232052980829,0
-0.52069378556564,48.232008932065,0
-0.52056147235176,48.231972425795,0
-0.52042488625099,48.23194373984,0
-0.52028506670906,48.231923092503,0
-0.52014307777398,48.231910640912,0
-0.51999999999999,48.231906479826,0
-0.51985692222601,48.231910640912,0
-0.5197149332909,48.231923092503,0
-0.51957511374897,48.23194373984,0
-0.5194385276482,48.231972425795,0
-0.51930621443432,48.232008932065,0
-0.51917918104148,48.232052980829,0
-0.51905839423058,48.23210423687,0
-0.51894477323273,48.23216231012,0
-0.51883918275437,48.232226758628,0
-0.51874242639667,48.232297091925,0
-0.51865524054003,48.232372774755,0
-0.51857828873945,48.23245323115,0
-0.51851215667406,48.232537848807,0
-0.51845734768872,48.232625983756,0
-0.51841427896213,48.232716965251,0
-0.51838327833038,48.232810100882,0
-0.5183645817901,48.232904681841,0
-0.51835833170047,48.232999988315,0
-0.51999999999998,48.233,0
</coordinates>
</LinearRing>
</outerBoundaryIs>
</Polygon>
</Placemark>
<Placemark>
<name>403</name>
<styleUrl>#stylehap2</styleUrl>
<Polygon>
<outerBoundaryIs>
<LinearRing>
<coordinates>
-0.51800000000003,48.312,0
-0.51635579163877,48.311999988283,0

-0.51636204528867,48.312095294935,0
-0.51638076483259,48.312189876421,0
-0.51641180787109,48.312283012915,0
-0.51645493821126,48.31237399558,0
-0.51650982766247,48.31246213197,0
-0.51657605853232,48.312546751299,0
-0.516653126804,48.312627209542,0
-0.51674044597099,48.312702894342,0
-0.51683735149985,48.312773229671,0
-0.51694310588704,48.312837680211,0
-0.51705690427175,48.312895755431,0
-0.51717788056145,48.31294701332,0
-0.517305114024,48.312991063756,0
-0.51743763629578,48.313027571467,0
-0.5175744387528,48.313056258592,0
-0.51771448018853,48.313076906792,0
-0.51785669473967,48.31308935891,0
-0.51800000000002,48.313093520173,0
-0.51814330526041,48.31308935891,0
-0.51828551981152,48.313076906792,0
-0.51842556124724,48.313056258592,0
-0.5185623637043,48.313027571467,0
-0.51869488597607,48.312991063756,0
-0.5188221194386,48.31294701332,0
-0.5189430957283,48.312895755431,0
-0.51905689411303,48.312837680211,0
-0.5191626485002,48.312773229671,0
-0.51925955402905,48.312702894342,0
-0.51934687319605,48.312627209542,0
-0.51942394146775,48.312546751299,0
-0.51949017233758,48.31246213197,0
-0.51954506178879,48.31237399558,0
-0.51958819212896,48.312283012915,0
-0.51961923516745,48.312189876421,0
-0.51963795471138,48.312095294935,0
-0.5196442083613,48.311999988283,0
-0.51963794859278,48.311904681809,0
-0.51961922311615,48.311810100851,0
-0.51958817451115,48.311716965221,0
-0.51954503913975,48.311625983727,0
-0.51949014534551,48.311537848781,0
-0.51942391095277,48.311453231125,0
-0.51934684008538,48.311372774734,0
-0.51925951932872,48.311297091906,0
-0.51916261326457,48.311226758611,0
-0.51905685941267,48.311162310106,0
-0.51894306261763,48.31110423686,0
-0.51882208892362,48.311052980821,0
-0.51869485898397,48.311008932059,0
-0.51856234105526,48.310972425792,0
-0.51842554362941,48.310943739838,0
-0.51828550776022,48.310923092502,0
-0.51814329914178,48.310910640912,0
-0.51800000000002,48.310906479826,0
-0.51785670085827,48.310910640912,0
-0.51771449223983,48.310923092502,0
-0.51757445637063,48.310943739838,0

-0.51743765894481,48.310972425792,0
-0.51730514101607,48.311008932059,0
-0.51717791107643,48.311052980821,0
-0.51705693738245,48.31110423686,0
-0.51694314058738,48.311162310106,0
-0.51683738673551,48.311226758611,0
-0.51674048067135,48.311297091906,0
-0.51665315991469,48.311372774734,0
-0.51657608904728,48.311453231125,0
-0.51650985465454,48.311537848781,0
-0.51645496086029,48.311625983727,0
-0.51641182548892,48.311716965221,0
-0.51638077688389,48.311810100851,0
-0.51636205140726,48.311904681809,0
-0.51635579163877,48.311999988283,0
-0.51800000000003,48.312,0
</coordinates>
</LinearRing>
</outerBoundaryIs>
</Polygon>
</Placemark>
<Placemark>
<name>405</name>
<styleUrl>#stylehap2</styleUrl>
<Polygon>
<outerBoundaryIs>
<LinearRing>
<coordinates>
-0.51800000000003,48.321,0
-0.51635550156732,48.320999988279,0
-0.51636175631952,48.321095294931,0
-0.51638047916502,48.321189876418,0
-0.51641152767924,48.321283012911,0
-0.51645466562767,48.321373995577,0
-0.51650956476181,48.321462131967,0
-0.51657580731555,48.321546751296,0
-0.51665288918321,48.321627209539,0
-0.51674022375482,48.32170289434,0
-0.51683714637967,48.321773229669,0
-0.51694291942415,48.321837680209,0
-0.51705673788544,48.321895755429,0
-0.51717773551821,48.32194701332,0
-0.51730499142784,48.321991063755,0
-0.5174375370799,48.322027571467,0
-0.51757436367245,48.322056258592,0
-0.51771442981519,48.322076906791,0
-0.51785666945675,48.32208935891,0
-0.51800000000002,48.322093520173,0
-0.5181433305433,48.32208935891,0
-0.51828557018486,48.322076906791,0
-0.5184256363276,48.322056258592,0
-0.51856246292015,48.322027571467,0
-0.5186950085722,48.321991063755,0
-0.51882226448184,48.32194701332,0
-0.51894326211461,48.321895755429,0
-0.5190570805759,48.321837680209,0
-0.51916285362038,48.321773229669,0

-0.51925977624522,48.32170289434,0
-0.51934711081684,48.321627209539,0
-0.5194241926845,48.321546751296,0
-0.51949043523826,48.321462131967,0
-0.51954533437238,48.321373995577,0
-0.51958847232081,48.321283012911,0
-0.51961952083503,48.321189876418,0
-0.51963824368055,48.321095294931,0
-0.51964449843275,48.320999988279,0
-0.51963823755892,48.320904681805,0
-0.5196195087778,48.320810100847,0
-0.5195884546943,48.320716965217,0
-0.51954531171217,48.320625983724,0
-0.51949040823288,48.320537848778,0
-0.5194241621545,48.320453231123,0
-0.51934707768984,48.320372774731,0
-0.51925974152779,48.320297091904,0
-0.51916281836737,48.32022675861,0
-0.51905704585846,48.320162310105,0
-0.51894322898761,48.320104236858,0
-0.51882223395182,48.32005298082,0
-0.51869498156683,48.320008932058,0
-0.51856244025994,48.319972425791,0
-0.51842561870109,48.319943739838,0
-0.51828555812763,48.319923092502,0
-0.51814332442167,48.319910640912,0
-0.51800000000002,48.319906479826,0
-0.51785667557838,48.319910640912,0
-0.51771444187244,48.319923092502,0
-0.51757438129896,48.319943739838,0
-0.5174375597401,48.319972425791,0
-0.51730501843322,48.320008932058,0
-0.51717776604822,48.32005298082,0
-0.51705677101244,48.320104236858,0
-0.51694295414161,48.320162310105,0
-0.51683718163268,48.32022675861,0
-0.51674025847226,48.320297091904,0
-0.51665292231021,48.320372774731,0
-0.51657583784557,48.320453231123,0
-0.51650959176719,48.320537848778,0
-0.51645468828788,48.320625983724,0
-0.51641154530574,48.320716965217,0
-0.51638049122227,48.320810100847,0
-0.51636176244115,48.320904681805,0
-0.51635550156732,48.320999988279,0
-0.51800000000003,48.321,0
</coordinates>
</LinearRing>
</outerBoundaryIs>
</Polygon>
</Placemark>
<Placemark>
<name>407</name>
<styleUrl>#stylehap2</styleUrl>
<Polygon>
<outerBoundaryIs>
<LinearRing>

<coordinates>
-0.51700000000005,48.308,0
-0.51535592051356,48.307999988284,0
-0.51536217367373,48.308095294936,0
-0.51538089175084,48.308189876423,0
-0.51541193235652,48.308283012916,0
-0.51545505931644,48.308373995581,0
-0.51550994446566,48.308462131972,0
-0.51557617014452,48.3085467513,0
-0.51565323237568,48.308627209543,0
-0.51574054469863,48.308702894343,0
-0.51583744263196,48.308773229672,0
-0.51594318872997,48.308837680212,0
-0.51605697819493,48.308895755431,0
-0.51617794500223,48.308947013321,0
-0.51630516849181,48.308991063756,0
-0.51643768037604,48.309027571467,0
-0.51657447211002,48.309056258592,0
-0.51671450256876,48.309076906792,0
-0.51685670597254,48.30908935891,0
-0.51700000000006,48.309093520174,0
-0.51714329402757,48.30908935891,0
-0.51728549743134,48.309076906792,0
-0.51742552789008,48.309056258592,0
-0.51756231962406,48.309027571467,0
-0.5176948315083,48.308991063756,0
-0.51782205499788,48.308947013321,0
-0.51794302180517,48.308895755431,0
-0.51805681127013,48.308837680212,0
-0.51816255736814,48.308773229672,0
-0.51825945530147,48.308702894343,0
-0.51834676762442,48.308627209543,0
-0.51842382985559,48.3085467513,0
-0.51849005553445,48.308462131972,0
-0.51854494068366,48.308373995581,0
-0.51858806764359,48.308283012916,0
-0.51861910824927,48.308189876423,0
-0.51863782632637,48.308095294936,0
-0.51864407948654,48.307999988284,0
-0.5186378202091,48.307904681811,0
-0.51861909620061,48.307810100852,0
-0.5185880500296,48.307716965222,0
-0.51854491803956,48.307625983729,0
-0.51849002854828,48.307537848782,0
-0.5184237993473,48.307453231127,0
-0.51834673452098,48.307372774735,0
-0.51825942060872,48.307297091907,0
-0.51816252214021,48.307226758612,0
-0.51805677657738,48.307162310107,0
-0.51794298870173,48.30710423686,0
-0.51782202448959,48.307052980821,0
-0.51769480452213,48.307008932059,0
-0.51756229697999,48.306972425792,0
-0.51742551027612,48.306943739838,0
-0.51728548538269,48.306923092502,0
-0.51714328791029,48.306910640912,0
-0.51700000000006,48.306906479827,0

-0.51685671208981,48.306910640912,0
-0.51671451461741,48.306923092502,0
-0.51657448972398,48.306943739838,0
-0.51643770302011,48.306972425792,0
-0.51630519547797,48.307008932059,0
-0.51617797551051,48.307052980821,0
-0.51605701129837,48.30710423686,0
-0.51594322342273,48.307162310107,0
-0.51583747785989,48.307226758612,0
-0.51574057939139,48.307297091907,0
-0.51565326547912,48.307372774735,0
-0.5155762006528,48.307453231127,0
-0.51550997145182,48.307537848782,0
-0.51545508196054,48.307625983729,0
-0.5154119499705,48.307716965222,0
-0.51538090379949,48.307810100852,0
-0.51536217979101,48.307904681811,0
-0.51535592051356,48.307999988284,0
-0.51700000000005,48.308,0
</coordinates>
</LinearRing>
</outerBoundaryIs>
</Polygon>
</Placemark>
<Placemark>
<name>411</name>
<styleUrl>#stylehap2</styleUrl>
<Polygon>
<outerBoundaryIs>
<LinearRing>
<coordinates>
-0.51700000000005,48.312,0
-0.51535579163879,48.311999988283,0
-0.51536204528868,48.312095294935,0
-0.51538076483264,48.312189876421,0
-0.51541180787111,48.312283012915,0
-0.5154549382113,48.3123739958,0
-0.51550982766251,48.31246213197,0
-0.51557605853234,48.312546751299,0
-0.51565312680401,48.312627209542,0
-0.51574044597103,48.312702894342,0
-0.51583735149986,48.312773229671,0
-0.51594310588706,48.312837680211,0
-0.51605690427177,48.312895755431,0
-0.51617788056149,48.31294701332,0
-0.51630511402402,48.312991063756,0
-0.51643763629579,48.313027571467,0
-0.51657443875285,48.313056258592,0
-0.51671448018857,48.313076906792,0
-0.51685669473968,48.31308935891,0
-0.51700000000006,48.313093520173,0
-0.51714330526042,48.31308935891,0
-0.51728551981153,48.313076906792,0
-0.51742556124726,48.313056258592,0
-0.51756236370431,48.313027571467,0
-0.51769488597609,48.312991063756,0
-0.51782211943861,48.31294701332,0

-0.51794309572834,48.312895755431,0
-0.51805689411304,48.312837680211,0
-0.51816264850024,48.312773229671,0
-0.51825955402907,48.312702894342,0
-0.51834687319609,48.312627209542,0
-0.51842394146776,48.312546751299,0
-0.51849017233762,48.31246213197,0
-0.51854506178883,48.31237399558,0
-0.518588192129,48.312283012915,0
-0.51861923516747,48.312189876421,0
-0.51863795471142,48.312095294935,0
-0.51864420836132,48.311999988283,0
-0.5186379485928,48.311904681809,0
-0.51861922311617,48.311810100851,0
-0.51858817451117,48.311716965221,0
-0.51854503913977,48.311625983727,0
-0.51849014534552,48.311537848781,0
-0.51842391095281,48.311453231125,0
-0.5183468400854,48.311372774734,0
-0.51825951932873,48.311297091906,0
-0.51816261326458,48.311226758611,0
-0.51805685941271,48.311162310106,0
-0.51794306261764,48.31110423686,0
-0.51782208892366,48.311052980821,0
-0.51769485898402,48.311008932059,0
-0.51756234105528,48.310972425792,0
-0.51742554362945,48.310943739838,0
-0.51728550776023,48.310923092502,0
-0.51714329914182,48.310910640912,0
-0.51700000000006,48.310906479826,0
-0.51685670085831,48.310910640912,0
-0.51671449223987,48.310923092502,0
-0.51657445637068,48.310943739838,0
-0.51643765894483,48.310972425792,0
-0.51630514101609,48.311008932059,0
-0.51617791107644,48.311052980821,0
-0.51605693738246,48.31110423686,0
-0.51594314058739,48.311162310106,0
-0.51583738673552,48.311226758611,0
-0.51574048067137,48.311297091906,0
-0.51565315991471,48.311372774734,0
-0.51557608904732,48.311453231125,0
-0.51550985465458,48.311537848781,0
-0.51545496086033,48.311625983727,0
-0.51541182548894,48.311716965221,0
-0.51538077688394,48.311810100851,0
-0.51536205140731,48.311904681809,0
-0.51535579163879,48.311999988283,0
-0.51700000000005,48.312,0
</coordinates>
</LinearRing>
</outerBoundaryIs>
</Polygon>
</Placemark>
<Placemark>
<name>416</name>
<styleUrl>#stylehap2</styleUrl>

<Polygon>
<outerBoundaryIs>
<LinearRing>
<coordinates>
-0.51300000000003,48.312,0
-0.51135579163877,48.311999988283,0
-0.51136204528867,48.312095294935,0
-0.51138076483262,48.312189876421,0
-0.51141180787109,48.312283012915,0
-0.51145493821126,48.31237399558,0
-0.51150982766247,48.31246213197,0
-0.51157605853232,48.312546751299,0
-0.511653126804,48.312627209542,0
-0.51174044597099,48.312702894342,0
-0.51183735149985,48.312773229671,0
-0.51194310588704,48.312837680211,0
-0.51205690427175,48.312895755431,0
-0.51217788056148,48.31294701332,0
-0.512305114024,48.312991063756,0
-0.51243763629578,48.313027571467,0
-0.51257443875283,48.313056258592,0
-0.51271448018853,48.313076906792,0
-0.51285669473967,48.31308935891,0
-0.51300000000002,48.313093520173,0
-0.51314330526041,48.31308935891,0
-0.51328551981152,48.313076906792,0
-0.51342556124724,48.313056258592,0
-0.5135623637043,48.313027571467,0
-0.51369488597607,48.312991063756,0
-0.5138221194386,48.31294701332,0
-0.51394309572832,48.312895755431,0
-0.51405689411303,48.312837680211,0
-0.51416264850023,48.312773229671,0
-0.51425955402905,48.312702894342,0
-0.51434687319608,48.312627209542,0
-0.51442394146775,48.312546751299,0
-0.51449017233758,48.31246213197,0
-0.51454506178879,48.31237399558,0
-0.51458819212896,48.312283012915,0
-0.51461923516745,48.312189876421,0
-0.51463795471138,48.312095294935,0
-0.5146442083613,48.311999988283,0
-0.51463794859278,48.311904681809,0
-0.51461922311615,48.311810100851,0
-0.51458817451115,48.311716965221,0
-0.51454503913975,48.311625983727,0
-0.51449014534551,48.311537848781,0
-0.51442391095277,48.311453231125,0
-0.51434684008538,48.311372774734,0
-0.51425951932872,48.311297091906,0
-0.51416261326457,48.311226758611,0
-0.51405685941267,48.311162310106,0
-0.51394306261763,48.31110423686,0
-0.51382208892364,48.311052980821,0
-0.513694858984,48.311008932059,0
-0.51356234105526,48.310972425792,0
-0.51342554362941,48.310943739838,0

-0.51328550776022,48.310923092502,0
-0.51314329914178,48.310910640912,0
-0.51300000000002,48.310906479826,0
-0.51285670085827,48.310910640912,0
-0.51271449223986,48.310923092502,0
-0.51257445637063,48.310943739838,0
-0.51243765894481,48.310972425792,0
-0.51230514101607,48.311008932059,0
-0.51217791107643,48.311052980821,0
-0.51205693738245,48.31110423686,0
-0.51194314058738,48.311162310106,0
-0.51183738673551,48.311226758611,0
-0.51174048067136,48.311297091906,0
-0.51165315991469,48.311372774734,0
-0.51157608904728,48.311453231125,0
-0.51150985465456,48.311537848781,0
-0.51145496086032,48.311625983727,0
-0.51141182548892,48.311716965221,0
-0.51138077688392,48.311810100851,0
-0.51136205140729,48.311904681809,0
-0.51135579163877,48.311999988283,0
-0.51300000000003,48.312,0
</coordinates>
</LinearRing>
</outerBoundaryIs>
</Polygon>
</Placemark>
<Placemark>
<name>417</name>
<styleUrl>#stylehap2</styleUrl>
<Polygon>
<outerBoundaryIs>
<LinearRing>
<coordinates>
-0.51300000000003,48.312,0
-0.51135579163877,48.311999988283,0
-0.51136204528867,48.312095294935,0
-0.51138076483262,48.312189876421,0
-0.51141180787109,48.312283012915,0
-0.51145493821126,48.31237399558,0
-0.51150982766247,48.31246213197,0
-0.51157605853232,48.312546751299,0
-0.511653126804,48.312627209542,0
-0.51174044597099,48.312702894342,0
-0.51183735149985,48.312773229671,0
-0.51194310588704,48.312837680211,0
-0.51205690427175,48.312895755431,0
-0.51217788056148,48.31294701332,0
-0.512305114024,48.312991063756,0
-0.51243763629578,48.313027571467,0
-0.51257443875283,48.313056258592,0
-0.51271448018853,48.313076906792,0
-0.51285669473967,48.31308935891,0
-0.51300000000002,48.313093520173,0
-0.51314330526041,48.31308935891,0
-0.51328551981152,48.313076906792,0
-0.51342556124724,48.313056258592,0

-0.5135623637043,48.313027571467,0
-0.51369488597607,48.312991063756,0
-0.5138221194386,48.31294701332,0
-0.51394309572832,48.312895755431,0
-0.51405689411303,48.312837680211,0
-0.51416264850023,48.312773229671,0
-0.51425955402905,48.312702894342,0
-0.51434687319608,48.312627209542,0
-0.51442394146775,48.312546751299,0
-0.51449017233758,48.31246213197,0
-0.51454506178879,48.31237399558,0
-0.51458819212896,48.312283012915,0
-0.51461923516745,48.312189876421,0
-0.51463795471138,48.312095294935,0
-0.5146442083613,48.311999988283,0
-0.51463794859278,48.311904681809,0
-0.51461922311615,48.311810100851,0
-0.51458817451115,48.311716965221,0
-0.51454503913975,48.311625983727,0
-0.51449014534551,48.311537848781,0
-0.51442391095277,48.311453231125,0
-0.51434684008538,48.311372774734,0
-0.51425951932872,48.311297091906,0
-0.51416261326457,48.311226758611,0
-0.51405685941267,48.311162310106,0
-0.51394306261763,48.31110423686,0
-0.51382208892364,48.311052980821,0
-0.513694858984,48.311008932059,0
-0.51356234105526,48.310972425792,0
-0.51342554362941,48.310943739838,0
-0.51328550776022,48.310923092502,0
-0.51314329914178,48.310910640912,0
-0.51300000000002,48.310906479826,0
-0.51285670085827,48.310910640912,0
-0.51271449223986,48.310923092502,0
-0.51257445637063,48.310943739838,0
-0.51243765894481,48.310972425792,0
-0.51230514101607,48.311008932059,0
-0.51217791107643,48.311052980821,0
-0.51205693738245,48.31110423686,0
-0.51194314058738,48.311162310106,0
-0.51183738673551,48.311226758611,0
-0.51174048067136,48.311297091906,0
-0.51165315991469,48.311372774734,0
-0.51157608904728,48.311453231125,0
-0.51150985465456,48.311537848781,0
-0.51145496086032,48.311625983727,0
-0.51141182548892,48.311716965221,0
-0.51138077688392,48.311810100851,0
-0.51136205140729,48.311904681809,0
-0.51135579163877,48.311999988283,0
-0.51300000000003,48.312,0
</coordinates>
</LinearRing>
</outerBoundaryIs>
</Polygon>
</Placemark>

<Placemark>
<name>420</name>
<styleUrl>#stylehap2</styleUrl>
<Polygon>
<outerBoundaryIs>
<LinearRing>
<coordinates>
-0.51300000000003,48.251,0
-0.51135775391745,48.250999988308,0
-0.5113640001105,48.25109529496,0
-0.51138269731994,48.251189876446,0
-0.51141370331612,48.251283012938,0
-0.51145678218781,48.251373995602,0
-0.5115116061359,48.251462131991,0
-0.51157775796635,48.251546751318,0
-0.51165473426376,48.251627209559,0
-0.51174194922149,48.251702894357,0
-0.51183873909911,48.251773229684,0
-0.51194436727328,48.251837680221,0
-0.51205802984374,48.251895755439,0
-0.51217886175165,48.251947013327,0
-0.51230594336379,48.25199106376,0
-0.51243830747235,48.25202757147,0
-0.51257494665726,48.252056258594,0
-0.51271482095474,48.252076906792,0
-0.51285686577372,48.25208935891,0
-0.51300000000002,48.252093520174,0
-0.51314313422636,48.25208935891,0
-0.51328517904534,48.252076906792,0
-0.51342505334281,48.252056258594,0
-0.51356169252772,48.25202757147,0
-0.51369405663628,48.25199106376,0
-0.51382113824842,48.251947013327,0
-0.51394197015633,48.251895755439,0
-0.51405563272677,48.251837680221,0
-0.51416126090096,48.251773229684,0
-0.51425805077856,48.251702894357,0
-0.51434526573631,48.251627209559,0
-0.51442224203372,48.251546751318,0
-0.51448839386417,48.251462131991,0
-0.51454321781227,48.251373995602,0
-0.51458629668396,48.251283012938,0
-0.51461730268011,48.251189876446,0
-0.51463599988958,48.25109529496,0
-0.51464224608262,48.250999988308,0
-0.51463599379136,48.250904681834,0
-0.51461729066896,48.250810100875,0
-0.51458627912483,48.250716965244,0
-0.5145431952387,48.250625983749,0
-0.51448836696202,48.250537848801,0
-0.51442221162042,48.250453231144,0
-0.51434523273594,48.25037277475,0
-0.51425801619384,48.25029709192,0
-0.5141612257827,48.250226758624,0
-0.51405559814206,48.250162310117,0
-0.51394193715596,48.250104236868,0
-0.51382110783512,48.250052980827,0

-0.51369402973416,48.250008932063,0
-0.51356166995415,48.249972425795,0
-0.51342503578368,48.24994373984,0
-0.51328516703419,48.249923092502,0
-0.51314312812814,48.249910640912,0
-0.51300000000002,48.249906479826,0
-0.51285687187193,48.249910640912,0
-0.51271483296589,48.249923092502,0
-0.51257496421639,48.24994373984,0
-0.51243833004592,48.249972425795,0
-0.51230597026591,48.250008932063,0
-0.51217889216493,48.250052980827,0
-0.51205806284409,48.250104236868,0
-0.51194440185802,48.250162310117,0
-0.51183877421737,48.250226758624,0
-0.51174198380623,48.25029709192,0
-0.51165476726413,48.25037277475,0
-0.51157778837963,48.250453231144,0
-0.51151163303803,48.250537848801,0
-0.51145680476138,48.250625983749,0
-0.51141372087525,48.250716965244,0
-0.51138270933108,48.250810100875,0
-0.51136400620872,48.250904681834,0
-0.51135775391745,48.250999988308,0
-0.51300000000003,48.251,0
</coordinates>
</LinearRing>
</outerBoundaryIs>
</Polygon>
</Placemark>
<Placemark>
<name>421</name>
<styleUrl>#stylehap2</styleUrl>
<Polygon>
<outerBoundaryIs>
<LinearRing>
<coordinates>
-0.51199999999994,48.324,0
-0.51035540484499,48.323999988278,0
-0.51036165996474,48.32409529493,0
-0.51038038391111,48.324189876417,0
-0.5104114342512,48.32428301291,0
-0.51045457473654,48.324373995575,0
-0.51050947709938,48.324462131966,0
-0.51057572354904,48.324546751295,0
-0.51065280995018,48.324627209538,0
-0.51074014965835,48.32470289434,0
-0.51083707798374,48.324773229669,0
-0.51094285724936,48.324837680209,0
-0.51105668240503,48.324895755429,0
-0.5111776871545,48.324947013319,0
-0.51130495054895,48.324991063755,0
-0.51143750399701,48.325027571466,0
-0.51157433863736,48.325056258592,0
-0.51171441301846,48.325076906791,0
-0.51185666102629,48.32508935891,0
-0.51199999999994,48.325093520174,0

-0.51214333897361,48.32508935891,0
-0.51228558698141,48.325076906791,0
-0.51242566136252,48.325056258592,0
-0.51256249600289,48.325027571466,0
-0.51269504945092,48.324991063755,0
-0.5128223128454,48.324947013319,0
-0.51294331759485,48.324895755429,0
-0.51305714275054,48.324837680209,0
-0.51316292201616,48.324773229669,0
-0.51325985034153,48.32470289434,0
-0.51334719004969,48.324627209538,0
-0.51342427645086,48.324546751295,0
-0.51349052290052,48.324462131966,0
-0.51354542526333,48.324373995575,0
-0.51358856574868,48.32428301291,0
-0.51361961608876,48.324189876417,0
-0.51363834003514,48.32409529493,0
-0.51364459515491,48.323999988278,0
-0.51363833391252,48.323904681804,0
-0.51361960402955,48.323810100846,0
-0.5135885481193,48.323716965216,0
-0.51354540259941,48.323625983723,0
-0.51349049589069,48.323537848777,0
-0.51342424591583,48.323453231122,0
-0.51334715691727,48.32337277473,0
-0.51325981561839,48.323297091903,0
-0.51316288675735,48.323226758609,0
-0.51305710802738,48.323162310104,0
-0.5129432844624,48.323104236858,0
-0.51282228231037,48.32305298082,0
-0.51269502244112,48.323008932058,0
-0.51256247333895,48.322972425791,0
-0.51242564373311,48.322943739838,0
-0.5122855749222,48.322923092502,0
-0.512143332851,48.322910640912,0
-0.51199999999994,48.322906479826,0
-0.51185666714891,48.322910640912,0
-0.5117144250777,48.322923092502,0
-0.51157435626676,48.322943739838,0
-0.51143752666093,48.322972425791,0
-0.51130497755878,48.323008932058,0
-0.51117771768951,48.32305298082,0
-0.51105671553747,48.323104236858,0
-0.51094289197249,48.323162310104,0
-0.51083711324255,48.323226758609,0
-0.51074018438151,48.323297091903,0
-0.51065284308263,48.32337277473,0
-0.51057575408405,48.323453231122,0
-0.51050950410919,48.323537848777,0
-0.51045459740046,48.323625983723,0
-0.5104114518806,48.323716965216,0
-0.51038039597035,48.323810100846,0
-0.51036166608738,48.323904681804,0
-0.51035540484499,48.323999988278,0
-0.51199999999994,48.324,0
</coordinates>
</LinearRing>

</outerBoundaryIs>
</Polygon>
</Placemark>
<Placemark>
<name>422</name>
<styleUrl>#stylehap2</styleUrl>
<Polygon>
<outerBoundaryIs>
<LinearRing>
<coordinates>
-0.51099999999997,48.324,0
-0.50935540484501,48.323999988278,0
-0.50936165996478,48.32409529493,0
-0.50938038391115,48.324189876417,0
-0.50941143425121,48.32428301291,0
-0.50945457473658,48.324373995575,0
-0.5095094770994,48.324462131966,0
-0.50957572354906,48.324546751295,0
-0.5096528099502,48.324627209538,0
-0.50974014965836,48.32470289434,0
-0.50983707798375,48.324773229669,0
-0.50994285724938,48.324837680209,0
-0.51005668240507,48.324895755429,0
-0.51017768715452,48.324947013319,0
-0.51030495054899,48.324991063755,0
-0.51043750399702,48.325027571466,0
-0.5105743386374,48.325056258592,0
-0.5107144130185,48.325076906791,0
-0.5108566610263,48.32508935891,0
-0.51099999999998,48.325093520174,0
-0.51114333897363,48.32508935891,0
-0.51128558698143,48.325076906791,0
-0.51142566136254,48.325056258592,0
-0.51156249600291,48.325027571466,0
-0.51169504945094,48.324991063755,0
-0.51182231284541,48.324947013319,0
-0.51194331759487,48.324895755429,0
-0.51205714275056,48.324837680209,0
-0.51216292201618,48.324773229669,0
-0.51225985034157,48.32470289434,0
-0.51234719004973,48.324627209538,0
-0.51242427645087,48.324546751295,0
-0.51249052290053,48.324462131966,0
-0.51254542526335,48.324373995575,0
-0.51258856574872,48.32428301291,0
-0.51261961608878,48.324189876417,0
-0.51263834003518,48.32409529493,0
-0.51264459515492,48.323999988278,0
-0.51263833391254,48.323904681804,0
-0.51261960402957,48.323810100846,0
-0.51258854811931,48.323716965216,0
-0.51254540259943,48.323625983723,0
-0.51249049589073,48.323537848777,0
-0.51242424591584,48.323453231122,0
-0.51234715691728,48.32337277473,0
-0.51225981561841,48.323297091903,0
-0.51216288675737,48.323226758609,0

-0.5120571080274,48.323162310104,0
-0.51194328446244,48.323104236858,0
-0.51182228231038,48.32305298082,0
-0.51169502244113,48.323008932058,0
-0.51156247333899,48.322972425791,0
-0.51142564373315,48.322943739838,0
-0.51128557492222,48.322923092502,0
-0.51114333285101,48.322910640912,0
-0.51099999999998,48.322906479826,0
-0.51085666714892,48.322910640912,0
-0.51071442507772,48.322923092502,0
-0.51057435626678,48.322943739838,0
-0.51043752666094,48.322972425791,0
-0.5103049775588,48.323008932058,0
-0.51017771768955,48.32305298082,0
-0.51005671553749,48.323104236858,0
-0.50994289197253,48.323162310104,0
-0.50983711324257,48.323226758609,0
-0.50974018438152,48.323297091903,0
-0.50965284308265,48.32337277473,0
-0.50957575408409,48.323453231122,0
-0.5095095041092,48.323537848777,0
-0.5094545974005,48.323625983723,0
-0.50941145188062,48.323716965216,0
-0.50938039597037,48.323810100846,0
-0.5093616660874,48.323904681804,0
-0.50935540484501,48.323999988278,0
-0.51099999999997,48.324,0
</coordinates>
</LinearRing>
</outerBoundaryIs>
</Polygon>
</Placemark>
<Placemark>
<name>426</name>
<styleUrl>#stylehap2</styleUrl>
<Polygon>
<outerBoundaryIs>
<LinearRing>
<coordinates>
-0.50999999999999,48.313,0
-0.50835575941562,48.312999988282,0
-0.50836201318798,48.313095294934,0
-0.50838073309867,48.313189876421,0
-0.50841177674544,48.313283012914,0
-0.5084549079308,48.31337399558,0
-0.50850979845764,48.31346213197,0
-0.50857603062542,48.313546751298,0
-0.50865310040743,48.313627209541,0
-0.50874042128569,48.313702894342,0
-0.50883732871366,48.313773229671,0
-0.50894308517344,48.313837680211,0
-0.5090568857884,48.313895755431,0
-0.50917786444903,48.31394701332,0
-0.50930510040514,48.313991063756,0
-0.50943762527416,48.314027571467,0
-0.50957443041234,48.314056258592,0

-0.50971447459268,48.314076906792,0
-0.50985669193103,48.31408935891,0
-0.50999999999999,48.314093520174,0
-0.51014330806896,48.31408935891,0
-0.5102855254073,48.314076906792,0
-0.51042556958765,48.314056258592,0
-0.51056237472583,48.314027571467,0
-0.51069489959484,48.313991063756,0
-0.51082213555095,48.31394701332,0
-0.51094311421159,48.313895755431,0
-0.51105691482655,48.313837680211,0
-0.51116267128633,48.313773229671,0
-0.5112595787143,48.313702894342,0
-0.51134689959254,48.313627209541,0
-0.51142396937457,48.313546751298,0
-0.51149020154234,48.31346213197,0
-0.51154509206919,48.31337399558,0
-0.51158822325455,48.313283012914,0
-0.51161926690132,48.313189876421,0
-0.511637986812,48.313095294934,0
-0.51164424058436,48.312999988282,0
-0.51163798069307,48.312904681809,0
-0.51161925484936,48.31281010085,0
-0.51158820563575,48.31271696522,0
-0.51154506941891,48.312625983727,0
-0.51149017454877,48.31253784878,0
-0.51142393885793,48.312453231125,0
-0.51134686648006,48.312372774733,0
-0.51125954401206,48.312297091905,0
-0.51116263604873,48.312226758611,0
-0.51105688012431,48.312162310106,0
-0.51094308109909,48.31210423686,0
-0.51082210503432,48.312052980821,0
-0.51069487260127,48.312008932059,0
-0.51056235207555,48.311972425792,0
-0.51042555196885,48.311943739838,0
-0.51028551335534,48.311923092502,0
-0.51014330195,48.311910640912,0
-0.50999999999999,48.311906479826,0
-0.50985669804998,48.311910640912,0
-0.50971448664464,48.311923092502,0
-0.50957444803114,48.311943739838,0
-0.50943764792444,48.311972425792,0
-0.50930512739869,48.312008932059,0
-0.50917789496567,48.312052980821,0
-0.5090569189009,48.31210423686,0
-0.50894311987568,48.312162310106,0
-0.50883736395125,48.312226758611,0
-0.50874045598793,48.312297091905,0
-0.50865313351993,48.312372774733,0
-0.50857606114205,48.312453231125,0
-0.50850982545119,48.31253784878,0
-0.50845493058108,48.312625983727,0
-0.50841179436424,48.31271696522,0
-0.50838074515063,48.31281010085,0
-0.50836201930691,48.312904681809,0
-0.50835575941562,48.312999988282,0

-0.50999999999999,48.313,0
</coordinates>
</LinearRing>
</outerBoundaryIs>
</Polygon>
</Placemark>
<Placemark>
<name>427</name>
<styleUrl>#stylehap2</styleUrl>
<Polygon>
<outerBoundaryIs>
<LinearRing>
<coordinates>
-0.50999999999999,48.322,0
-0.5083554693283,48.321999988279,0
-0.50836172420302,48.322095294931,0
-0.50838044741545,48.322189876417,0
-0.50841149653827,48.322283012911,0
-0.5084546353323,48.322373995576,0
-0.50850953554259,48.322462131967,0
-0.50857577939491,48.322546751296,0
-0.50865286277363,48.322627209539,0
-0.50874019905735,48.32270289434,0
-0.50883712358226,48.322773229669,0
-0.50894289870034,48.322837680209,0
-0.50905671939297,48.322895755429,0
-0.50917771939785,48.322947013319,0
-0.50930497780229,48.322991063755,0
-0.50943752605286,48.323027571467,0
-0.50957435532787,48.323056258592,0
-0.50971442421659,48.323076906791,0
-0.50985666664674,48.32308935891,0
-0.50999999999999,48.323093520174,0
-0.51014333335325,48.32308935891,0
-0.5102855757834,48.323076906791,0
-0.51042564467212,48.323056258592,0
-0.51056247394713,48.323027571467,0
-0.5106950221977,48.322991063755,0
-0.51082228060213,48.322947013319,0
-0.51094328060701,48.322895755429,0
-0.51105710129962,48.322837680209,0
-0.51116287641773,48.322773229669,0
-0.51125980094263,48.32270289434,0
-0.51134713722633,48.322627209539,0
-0.51142422060508,48.322546751296,0
-0.5114904644574,48.322462131967,0
-0.51154536466769,48.322373995576,0
-0.51158850346172,48.322283012911,0
-0.51161955258454,48.322189876417,0
-0.51163827579697,48.322095294931,0
-0.51164453067169,48.321999988279,0
-0.51163826967502,48.321904681805,0
-0.51161954052663,48.321810100847,0
-0.51158848583424,48.321716965217,0
-0.51154534200626,48.321625983723,0
-0.51149043745055,48.321537848777,0
-0.51142419007341,48.321453231122,0

-0.51134710409752,48.321372774731,0
-0.51125976622329,48.321297091903,0
-0.51116284116278,48.321226758609,0
-0.51105706658028,48.321162310105,0
-0.5109432474782,48.321104236858,0
-0.51082225007046,48.32105298082,0
-0.51069499519084,48.321008932058,0
-0.51056245128567,48.320972425791,0
-0.51042562704465,48.320943739838,0
-0.51028556372551,48.320923092502,0
-0.51014332723129,48.320910640912,0
-0.50999999999999,48.320906479826,0
-0.5098566727687,48.320910640912,0
-0.50971443627448,48.320923092502,0
-0.50957437295534,48.320943739838,0
-0.50943754871431,48.320972425791,0
-0.50930500480915,48.321008932058,0
-0.50917774992952,48.32105298082,0
-0.50905675252178,48.321104236858,0
-0.50894293341968,48.321162310105,0
-0.50883715883721,48.321226758609,0
-0.5087402337767,48.321297091903,0
-0.50865289590247,48.321372774731,0
-0.50857580992658,48.321453231122,0
-0.50850956254944,48.321537848777,0
-0.50845465799373,48.321625983723,0
-0.50841151416574,48.321716965217,0
-0.50838045947336,48.321810100847,0
-0.50836173032497,48.321904681805,0
-0.5083554693283,48.321999988279,0
-0.50999999999999,48.322,0
</coordinates>
</LinearRing>
</outerBoundaryIs>
</Polygon>
</Placemark>
<Placemark>
<name>428</name>
<styleUrl>#stylehap2</styleUrl>
<Polygon>
<outerBoundaryIs>
<LinearRing>
<coordinates>
-0.50900000000001,48.251,0
-0.50735775391744,48.250999988308,0
-0.50736400011046,48.25109529496,0
-0.50738269731992,48.251189876446,0
-0.5074137033161,48.251283012938,0
-0.50745678218779,48.251373995602,0
-0.50751160613589,48.251462131991,0
-0.50757775796634,48.251546751318,0
-0.50765473426375,48.251627209559,0
-0.50774194922147,48.251702894357,0
-0.5078387390991,48.251773229684,0
-0.50794436727326,48.251837680221,0
-0.50805802984373,48.251895755439,0
-0.50817886175164,48.251947013327,0

-0.50830594336375,48.25199106376,0
-0.50843830747231,48.25202757147,0
-0.50857494665725,48.252056258594,0
-0.50871482095472,48.252076906792,0
-0.5088568657737,48.25208935891,0
-0.50900000000001,48.252093520174,0
-0.50914313422632,48.25208935891,0
-0.50928517904532,48.252076906792,0
-0.50942505334277,48.252056258594,0
-0.5095616925277,48.25202757147,0
-0.50969405663627,48.25199106376,0
-0.50982113824841,48.251947013327,0
-0.50994197015631,48.251895755439,0
-0.51005563272676,48.251837680221,0
-0.51016126090092,48.251773229684,0
-0.51025805077854,48.251702894357,0
-0.51034526573629,48.251627209559,0
-0.51042224203371,48.251546751318,0
-0.51048839386416,48.251462131991,0
-0.51054321781225,48.251373995602,0
-0.51058629668394,48.251283012938,0
-0.5106173026801,48.251189876446,0
-0.51063599988956,48.25109529496,0
-0.51064224608258,48.250999988308,0
-0.51063599379134,48.250904681834,0
-0.51061729066895,48.250810100875,0
-0.51058627912481,48.250716965244,0
-0.51054319523868,48.250625983749,0
-0.51048836696201,48.250537848801,0
-0.5104222116204,48.250453231144,0
-0.51034523273593,48.25037277475,0
-0.51025801619383,48.25029709192,0
-0.51016122578269,48.250226758624,0
-0.51005559814204,48.250162310117,0
-0.50994193715595,48.250104236868,0
-0.5098211078351,48.250052980827,0
-0.50969402973412,48.250008932063,0
-0.50956166995414,48.249972425795,0
-0.50942503578367,48.24994373984,0
-0.50928516703417,48.249923092502,0
-0.5091431281281,48.249910640912,0
-0.50900000000001,48.249906479826,0
-0.50885687187192,48.249910640912,0
-0.50871483296587,48.249923092502,0
-0.50857496421638,48.24994373984,0
-0.50843833004591,48.249972425795,0
-0.5083059702659,48.250008932063,0
-0.50817889216492,48.250052980827,0
-0.50805806284407,48.250104236868,0
-0.50794440185798,48.250162310117,0
-0.50783877421736,48.250226758624,0
-0.50774198380622,48.25029709192,0
-0.50765476726409,48.25037277475,0
-0.50757778837962,48.250453231144,0
-0.50751163303801,48.250537848801,0
-0.50745680476136,48.250625983749,0
-0.50741372087521,48.250716965244,0

-0.50738270933107,48.250810100875,0
-0.5073640062087,48.250904681834,0
-0.50735775391744,48.250999988308,0
-0.50900000000001,48.251,0
</coordinates>
</LinearRing>
</outerBoundaryIs>
</Polygon>
</Placemark>
<Placemark>
<name>434</name>
<styleUrl>#stylehap2</styleUrl>
<Polygon>
<outerBoundaryIs>
<LinearRing>
<coordinates>
-0.50900000000001,48.2,0
-0.50735938949392,48.199999988329,0
-0.5073656294716,48.20009529498,0
-0.50738430806505,48.200189876466,0
-0.50741528318611,48.200283012958,0
-0.50745831915838,48.200373995621,0
-0.50751308850904,48.200462132008,0
-0.50757917445947,48.200546751333,0
-0.50765607409551,48.200627209573,0
-0.50774320219392,48.200702894369,0
-0.50783989567521,48.200773229694,0
-0.50794541864971,48.20083768023,0
-0.5080589680178,48.200895755446,0
-0.50817967958222,48.200947013332,0
-0.50830663462571,48.200991063764,0
-0.50843886690387,48.201027571472,0
-0.50857537000004,48.201056258595,0
-0.50871510498632,48.201076906793,0
-0.50885700833206,48.20108935891,0
-0.50900000000001,48.201093520174,0
-0.50914299166795,48.20108935891,0
-0.50928489501372,48.201076906793,0
-0.50942463,48.201056258595,0
-0.50956113309618,48.201027571472,0
-0.50969336537434,48.200991063764,0
-0.50982032041783,48.200947013332,0
-0.50994103198225,48.200895755446,0
-0.51005458135031,48.20083768023,0
-0.5101601043248,48.200773229694,0
-0.51025679780613,48.200702894369,0
-0.51034392590453,48.200627209573,0
-0.51042082554058,48.200546751333,0
-0.510486911491,48.200462132008,0
-0.51054168084167,48.200373995621,0
-0.51058471681391,48.200283012958,0
-0.51061569193497,48.200189876466,0
-0.51063437052844,48.20009529498,0
-0.5106406105061,48.199999988329,0
-0.5106343644472,48.199904681855,0
-0.51061567995726,48.199810100895,0
-0.51058469930369,48.199716965264,0

-0.51054165833095,48.199625983768,0
-0.51048688466376,48.199537848819,0
-0.51042079521195,48.19945323116,0
-0.51034389299604,48.199372774764,0
-0.51025676331769,48.199297091933,0
-0.51016006930435,48.199226758634,0
-0.51005454686188,48.199162310126,0
-0.50994099907376,48.199104236875,0
-0.5098202900892,48.199052980832,0
-0.5096933385471,48.199008932067,0
-0.50956111058546,48.198972425797,0
-0.50942461248975,48.198943739841,0
-0.50928488303601,48.198923092503,0
-0.50914298558671,48.198910640912,0
-0.50900000000001,48.198906479826,0
-0.50885701441331,48.198910640912,0
-0.50871511696404,48.198923092503,0
-0.50857538751027,48.198943739841,0
-0.50843888941459,48.198972425797,0
-0.50830666145295,48.199008932067,0
-0.50817970991084,48.199052980832,0
-0.50805900092628,48.199104236875,0
-0.50794545313814,48.199162310126,0
-0.50783993069569,48.199226758634,0
-0.50774323668235,48.199297091933,0
-0.507656107004,48.199372774764,0
-0.50757920478807,48.19945323116,0
-0.50751311533628,48.199537848819,0
-0.5074583416691,48.199625983768,0
-0.50741530069633,48.199716965264,0
-0.50738432004276,48.199810100895,0
-0.50736563555282,48.199904681855,0
-0.50735938949392,48.199999988329,0
-0.50900000000001,48.2,0
</coordinates>
</LinearRing>
</outerBoundaryIs>
</Polygon>
</Placemark>
<Placemark>
<name>439</name>
<styleUrl>#stylehap2</styleUrl>
<Polygon>
<outerBoundaryIs>
<LinearRing>
<coordinates>
-0.50700000000006,48.229,0
-0.50535846001959,48.228999988317,0
-0.50536470352937,48.229095294969,0
-0.50538339270203,48.229189876455,0
-0.50541438536896,48.229283012947,0
-0.50545744572033,48.22937399561,0
-0.50551224609795,48.229462131998,0
-0.50557836948701,48.229546751324,0
-0.50565531268861,48.229627209565,0
-0.5057424901479,48.229702894362,0
-0.50583923840985,48.229773229688,0

-0.50594482116778,48.229837680225,0
-0.50605843486714,48.229895755442,0
-0.50617921482102,48.229947013329,0
-0.50630624179161,48.229991063762,0
-0.50643854898708,48.230027571471,0
-0.50657512942052,48.230056258595,0
-0.50671494357534,48.230076906793,0
-0.50685692731828,48.23008935891,0
-0.50700000000007,48.230093520174,0
-0.50714307268185,48.23008935891,0
-0.50728505642479,48.230076906793,0
-0.50742487057961,48.230056258595,0
-0.50756145101303,48.230027571471,0
-0.50769375820849,48.229991063762,0
-0.50782078517911,48.229947013329,0
-0.50794156513299,48.229895755442,0
-0.50805517883235,48.229837680225,0
-0.50816076159028,48.229773229688,0
-0.5082575098522,48.229702894362,0
-0.50834468731152,48.229627209565,0
-0.50842163051309,48.229546751324,0
-0.50848775390218,48.229462131998,0
-0.50854255427977,48.22937399561,0
-0.50858561463117,48.229283012947,0
-0.50861660729811,48.229189876455,0
-0.50863529647076,48.229095294969,0
-0.50864153998054,48.228999988317,0
-0.50863529037987,48.228904681843,0
-0.50861659530138,48.228810100884,0
-0.50858559709316,48.228716965253,0
-0.50854253173335,48.228625983757,0
-0.5084877270324,48.228537848809,0
-0.50842160013638,48.228453231151,0
-0.50834465435082,48.228372774756,0
-0.50825747530906,48.228297091926,0
-0.50816072651425,48.228226758629,0
-0.50805514428918,48.228162310121,0
-0.50794153217231,48.228104236871,0
-0.50782075480239,48.228052980829,0
-0.50769373133871,48.228008932065,0
-0.50756142846661,48.227972425796,0
-0.5074248530416,48.22794373984,0
-0.5072850444281,48.227923092503,0
-0.50714306659096,48.227910640912,0
-0.50700000000007,48.227906479826,0
-0.50685693340914,48.227910640912,0
-0.50671495557203,48.227923092503,0
-0.50657514695853,48.22794373984,0
-0.50643857153352,48.227972425796,0
-0.50630626866142,48.228008932065,0
-0.50617924519774,48.228052980829,0
-0.50605846782782,48.228104236871,0
-0.50594485571092,48.228162310121,0
-0.50583927348588,48.228226758629,0
-0.50574252469107,48.228297091926,0
-0.50565534564929,48.228372774756,0
-0.50557839986375,48.228453231151,0

-0.50551227296773,48.228537848809,0
-0.50545746826678,48.228625983757,0
-0.50541440290697,48.228716965253,0
-0.50538340469875,48.228810100884,0
-0.50536470962026,48.228904681843,0
-0.50535846001959,48.228999988317,0
-0.50700000000006,48.229,0
</coordinates>
</LinearRing>
</outerBoundaryIs>
</Polygon>
</Placemark>
<Placemark>
<name>448</name>
<styleUrl>#stylehap2</styleUrl>
<Polygon>
<outerBoundaryIs>
<LinearRing>
<coordinates>
-0.50199999999995,48.235,0
-0.50035826753041,48.234999988315,0
-0.50036451177166,48.235095294966,0
-0.50038320313522,48.235189876452,0
-0.50041419943579,48.235283012944,0
-0.50045726483598,48.235373995608,0
-0.50051207163908,48.235462131996,0
-0.5005782027815,48.235546751323,0
-0.50065515500529,48.235627209563,0
-0.50074234268696,48.235702894361,0
-0.50083910229364,48.235773229687,0
-0.50094469743242,48.235837680224,0
-0.50105832445442,48.235895755441,0
-0.50117911857138,48.235947013328,0
-0.50130616043771,48.235991063761,0
-0.50143848314814,48.236027571471,0
-0.50157507959769,48.236056258594,0
-0.50171491014789,48.236076906793,0
-0.50185691054062,48.23608935891,0
-0.50199999999996,48.236093520174,0
-0.50214308945928,48.23608935891,0
-0.50228508985204,48.236076906793,0
-0.50242492040221,48.236056258594,0
-0.50256151685176,48.236027571471,0
-0.5026938395622,48.235991063761,0
-0.50282088142852,48.235947013328,0
-0.50294167554548,48.235895755441,0
-0.50305530256748,48.235837680224,0
-0.50316089770626,48.235773229687,0
-0.50325765731294,48.235702894361,0
-0.50334484499464,48.235627209563,0
-0.50342179721841,48.235546751323,0
-0.50348792836083,48.235462131996,0
-0.50354273516395,48.235373995608,0
-0.50358580056411,48.235283012944,0
-0.50361679686469,48.235189876452,0
-0.50363548822824,48.235095294966,0
-0.50364173246949,48.234999988315,0

-0.50363548213536,48.23490468184,0
-0.50361678486405,48.234810100882,0
-0.50358578302035,48.23471696525,0
-0.5035427126101,48.234625983755,0
-0.50348790148221,48.234537848807,0
-0.50342176683172,48.234453231149,0
-0.50334481202313,48.234372774755,0
-0.50325762275848,48.234297091924,0
-0.50316086261871,48.234226758627,0
-0.50305526801302,48.23416231012,0
-0.502941642574,48.23410423687,0
-0.50282085104183,48.234052980829,0
-0.50269381268358,48.234008932064,0
-0.50256149429794,48.233972425795,0
-0.50242490285845,48.23394373984,0
-0.50228507785137,48.233923092503,0
-0.50214308336638,48.233910640912,0
-0.50199999999996,48.233906479827,0
-0.50185691663352,48.233910640912,0
-0.50171492214853,48.233923092503,0
-0.50157509714146,48.23394373984,0
-0.50143850570197,48.233972425795,0
-0.50130618731632,48.234008932064,0
-0.50117914895807,48.234052980829,0
-0.50105835742591,48.23410423687,0
-0.50094473198689,48.23416231012,0
-0.50083913738119,48.234226758627,0
-0.50074237724142,48.234297091924,0
-0.50065518797678,48.234372774755,0
-0.50057823316821,48.234453231149,0
-0.50051209851769,48.234537848807,0
-0.5004572873898,48.234625983755,0
-0.50041421697958,48.23471696525,0
-0.50038321513586,48.234810100882,0
-0.50036451786454,48.23490468184,0
-0.50035826753041,48.234999988315,0
-0.50199999999995,48.235,0
</coordinates>
</LinearRing>
</outerBoundaryIs>
</Polygon>
</Placemark>
<Placemark>
<name>453</name>
<styleUrl>#stylehap2</styleUrl>
<Polygon>
<outerBoundaryIs>
<LinearRing>
<coordinates>
-0.49900000000002,48.196,0
-0.49735951758172,48.19599998833,0
-0.49736575707263,48.196095294982,0
-0.49738443420824,48.196189876468,0
-0.49741540691134,48.196283012959,0
-0.497458439524,48.196373995622,0
-0.49751320459893,48.19646213201,0
-0.49757928539007,48.196546751335,0

-0.4976561790225,48.196627209574,0
-0.49774330031864,48.19670289437,0
-0.4978399862508,48.196773229695,0
-0.49794550098674,48.196837680231,0
-0.49805904148953,48.196895755446,0
-0.49817974362943,48.196947013332,0
-0.49830668876089,48.196991063764,0
-0.49843891071494,48.197027571473,0
-0.49857540315351,48.197056258595,0
-0.49871512722985,48.197076906793,0
-0.49885701949632,48.19708935891,0
-0.49900000000004,48.197093520174,0
-0.49914298050372,48.19708935891,0
-0.4992848727702,48.197076906793,0
-0.49942459684654,48.197056258595,0
-0.49956108928511,48.197027571473,0
-0.49969331123916,48.196991063764,0
-0.49982025637062,48.196947013332,0
-0.49994095851052,48.196895755446,0
-0.50005449901331,48.196837680231,0
-0.50016001374925,48.196773229695,0
-0.50025669968141,48.19670289437,0
-0.50034382097755,48.196627209574,0
-0.50042071460998,48.196546751335,0
-0.50048679540111,48.19646213201,0
-0.50054156047607,48.196373995622,0
-0.50058459308871,48.196283012959,0
-0.50061556579183,48.196189876468,0
-0.50063424292742,48.196095294982,0
-0.50064048241832,48.19599998833,0
-0.50063423684749,48.195904681856,0
-0.50061555381674,48.195810100897,0
-0.5005845755823,48.195716965265,0
-0.50054153797026,48.195625983769,0
-0.50048676857972,48.19553784882,0
-0.500420684288,48.195453231161,0
-0.50034378807626,48.195372774766,0
-0.50025666520053,48.195297091934,0
-0.50015997873643,48.195226758635,0
-0.50005446453241,48.195162310126,0
-0.49994092560923,48.195104236875,0
-0.49982022604863,48.195052980833,0
-0.4996932844178,48.195008932067,0
-0.49956106677932,48.194972425797,0
-0.49942457934013,48.194943739841,0
-0.4992848607951,48.194923092503,0
-0.4991429744238,48.194910640912,0
-0.49900000000004,48.194906479826,0
-0.49885702557624,48.194910640912,0
-0.49871513920494,48.194923092503,0
-0.49857542065994,48.194943739841,0
-0.49843893322075,48.194972425797,0
-0.49830671558225,48.195008932067,0
-0.49817977395141,48.195052980833,0
-0.49805907439084,48.195104236875,0
-0.49794553546764,48.195162310126,0
-0.49784002126361,48.195226758635,0

-0.49774333479951,48.195297091934,0
-0.49765621192378,48.195372774766,0
-0.49757931571205,48.195453231161,0
-0.49751323142032,48.19553784882,0
-0.49745846202978,48.195625983769,0
-0.49741542441775,48.195716965265,0
-0.49738444618331,48.195810100897,0
-0.49736576315255,48.195904681856,0
-0.49735951758172,48.19599998833,0
-0.49900000000002,48.196,0
</coordinates>
</LinearRing>
</outerBoundaryIs>
</Polygon>
</Placemark>
<Placemark>
<name>454</name>
<styleUrl>#stylehap2</styleUrl>
<Polygon>
<outerBoundaryIs>
<LinearRing>
<coordinates>
-0.49800000000005,48.257,0
-0.4963575611967,48.256999988305,0
-0.49636380812209,48.257095294957,0
-0.49638250752507,48.257189876443,0
-0.49641351715927,48.257283012936,0
-0.49645660108583,48.2573739956,0
-0.49651143146717,48.257462131989,0
-0.49657759106028,48.257546751316,0
-0.49665457639075,48.257627209557,0
-0.49674180158317,48.257702894356,0
-0.49683860281919,48.257773229683,0
-0.49694424338909,48.25783768022,0
-0.49705791929825,48.257895755438,0
-0.49717876538629,48.257947013326,0
-0.49730586191205,48.25799106376,0
-0.49743824155426,48.25802757147,0
-0.49757489677459,48.258056258594,0
-0.49771478748716,48.258076906792,0
-0.49785684897601,48.25808935891,0
-0.49800000000005,48.258093520174,0
-0.49814315102409,48.25808935891,0
-0.49828521251292,48.258076906792,0
-0.49842510322551,48.258056258594,0
-0.49856175844584,48.25802757147,0
-0.49869413808803,48.25799106376,0
-0.49882123461381,48.257947013326,0
-0.49894208070185,48.257895755438,0
-0.49905575661101,48.25783768022,0
-0.49916139718089,48.257773229683,0
-0.49925819841693,48.257702894356,0
-0.49934542360935,48.257627209557,0
-0.49942240893982,48.257546751316,0
-0.49948856853293,48.257462131989,0
-0.49954339891427,48.2573739956,0
-0.49958648284083,48.257283012936,0

-0.49961749247501,48.257189876443,0
-0.49963619187801,48.257095294957,0
-0.4996424388034,48.256999988305,0
-0.49963618577778,48.256904681831,0
-0.49961748045994,48.256810100873,0
-0.49958646527595,48.256716965242,0
-0.4995433763333,48.256625983747,0
-0.49948854162198,48.256537848799,0
-0.49942237851654,48.256453231142,0
-0.49934539059817,48.256372774749,0
-0.49925816382087,48.256297091919,0
-0.49916136205112,48.256226758623,0
-0.49905572201492,48.256162310116,0
-0.49894204769067,48.256104236867,0
-0.49882120419053,48.256052980827,0
-0.49869411117707,48.256008932063,0
-0.49856173586484,48.255972425794,0
-0.49842508566061,48.255943739839,0
-0.49828520049785,48.255923092502,0
-0.49814314492386,48.255910640912,0
-0.49800000000005,48.255906479826,0
-0.49785685507624,48.255910640912,0
-0.49771479950225,48.255923092502,0
-0.49757491433947,48.255943739839,0
-0.49743826413526,48.255972425794,0
-0.49730588882303,48.256008932063,0
-0.49717879580954,48.256052980827,0
-0.49705795230943,48.256104236867,0
-0.49694427798516,48.256162310116,0
-0.49683863794898,48.256226758623,0
-0.49674183617923,48.256297091919,0
-0.49665460940193,48.256372774749,0
-0.49657762148356,48.256453231142,0
-0.49651145837812,48.256537848799,0
-0.4964566236668,48.256625983747,0
-0.49641353472415,48.256716965242,0
-0.49638251954016,48.256810100873,0
-0.49636381422232,48.256904681831,0
-0.4963575611967,48.256999988305,0
-0.49800000000005,48.257,0
</coordinates>
</LinearRing>
</outerBoundaryIs>
</Polygon>
</Placemark>
<Placemark>
<name>455</name>
<styleUrl>#stylehap2</styleUrl>
<Polygon>
<outerBoundaryIs>
<LinearRing>
<coordinates>
-0.49800000000005,48.218,0
-0.49635881275227,48.217999988321,0
-0.49636505492161,48.218095294973,0
-0.49638374007952,48.218189876459,0
-0.49641472608782,48.218283012951,0

-0.49645777718742,48.218373995614,0
-0.49651256579039,48.218462132002,0
-0.49657867497162,48.218546751328,0
-0.49665560164021,48.218627209568,0
-0.49674276036721,48.218702894365,0
-0.49683948784006,48.218773229691,0
-0.49694504791035,48.218837680227,0
-0.49705863719618,48.218895755443,0
-0.49717939119648,48.21894701333,0
-0.49730639087096,48.218991063763,0
-0.49743866963553,48.219027571472,0
-0.49757522071973,48.219056258595,0
-0.49771500483031,48.219076906793,0
-0.49785695806275,48.21908935891,0
-0.49800000000005,48.219093520174,0
-0.49814304193732,48.21908935891,0
-0.49828499516979,48.219076906793,0
-0.49842477928037,48.219056258595,0
-0.49856133036455,48.219027571472,0
-0.49869360912914,48.218991063763,0
-0.49882060880362,48.21894701333,0
-0.49894136280392,48.218895755443,0
-0.49905495208973,48.218837680227,0
-0.49916051216004,48.218773229691,0
-0.4992572396329,48.218702894365,0
-0.49934439835989,48.218627209568,0
-0.49942132502849,48.218546751328,0
-0.49948743420971,48.218462132002,0
-0.49954222281268,48.218373995614,0
-0.49958527391225,48.218283012951,0
-0.49961625992058,48.218189876459,0
-0.49963494507849,48.218095294973,0
-0.49964118724783,48.217999988321,0
-0.49963493899126,48.217904681847,0
-0.49961624793108,48.217810100888,0
-0.4995852563848,48.217716965257,0
-0.4995422002798,48.217625983761,0
-0.49948740735606,48.217537848813,0
-0.49942129467001,48.217453231154,0
-0.49934436541901,48.21737277476,0
-0.49925720511052,48.217297091928,0
-0.49916047710509,48.217226758631,0
-0.49905491756735,48.217162310122,0
-0.49894132986304,48.217104236872,0
-0.49882057844515,48.217052980831,0
-0.49869358227549,48.217008932066,0
-0.49856130783167,48.216972425796,0
-0.49842476175289,48.216943739841,0
-0.49828498318029,48.216923092503,0
-0.4981430358501,48.216910640912,0
-0.49800000000005,48.216906479826,0
-0.49785696414998,48.216910640912,0
-0.49771501681981,48.216923092503,0
-0.49757523824718,48.216943739841,0
-0.49743869216841,48.216972425796,0
-0.49730641772462,48.217008932066,0
-0.49717942155495,48.217052980831,0

-0.49705867013706,48.217104236872,0
-0.49694508243273,48.217162310122,0
-0.49683952289498,48.217226758631,0
-0.49674279488958,48.217297091928,0
-0.49665563458109,48.21737277476,0
-0.49657870533009,48.217453231154,0
-0.49651259264402,48.217537848813,0
-0.4964577997203,48.217625983761,0
-0.4964147436153,48.217716965257,0
-0.49638375206902,48.217810100888,0
-0.49636506100884,48.217904681847,0
-0.49635881275227,48.217999988321,0
-0.49800000000005,48.218,0
</coordinates>
</LinearRing>
</outerBoundaryIs>
</Polygon>
</Placemark>
<Placemark>
<name>457</name>
<styleUrl>#stylehap2</styleUrl>
<Polygon>
<outerBoundaryIs>
<LinearRing>
<coordinates>
-0.49700000000007,48.256,0
-0.49535759332124,48.255999988306,0
-0.49536384012454,48.256095294958,0
-0.49538253916191,48.256189876444,0
-0.49541354818967,48.256283012936,0
-0.49545663127363,48.2563739956,0
-0.49551146058262,48.256462131989,0
-0.49557761888178,48.256546751316,0
-0.49565460270655,48.256627209557,0
-0.49574182619294,48.256702894356,0
-0.49583862553564,48.256773229683,0
-0.49594426403931,48.256837680221,0
-0.49605793772502,48.256895755438,0
-0.49617878144941,48.256947013326,0
-0.49630587548922,48.25699106376,0
-0.49643825254213,48.25702757147,0
-0.49657490508954,48.257056258594,0
-0.49671479306589,48.257076906792,0
-0.49685685177603,48.25708935891,0
-0.49700000000007,48.257093520173,0
-0.4971431482241,48.25708935891,0
-0.49728520693426,48.257076906792,0
-0.49742509491062,48.257056258594,0
-0.497561747458,48.25702757147,0
-0.49769412451092,48.25699106376,0
-0.49782121855075,48.256947013326,0
-0.49794206227511,48.256895755438,0
-0.49805573596083,48.256837680221,0
-0.49816137446449,48.256773229683,0
-0.49825817380721,48.256702894356,0
-0.49834539729361,48.256627209557,0
-0.49842238111835,48.256546751316,0

-0.49848853941751,48.256462131989,0
-0.4985433687265,48.2563739956,0
-0.49858645181046,48.256283012936,0
-0.49861746083825,48.256189876444,0
-0.49863615987559,48.256095294958,0
-0.4986424066789,48.255999988306,0
-0.49863615377569,48.255904681832,0
-0.49861744882379,48.255810100873,0
-0.49858643424655,48.255716965242,0
-0.49854334614675,48.255625983747,0
-0.49848851250804,48.2555378488,0
-0.49842235069675,48.255453231143,0
-0.49834536428423,48.255372774749,0
-0.49825813921303,48.255297091919,0
-0.49816133933664,48.255226758623,0
-0.49805570136664,48.255162310116,0
-0.49794202926573,48.255104236867,0
-0.49782118812913,48.255052980827,0
-0.49769409760141,48.255008932063,0
-0.49756172487825,48.254972425794,0
-0.49742507734668,48.25494373984,0
-0.49728519491983,48.254923092502,0
-0.49714314212423,48.254910640912,0
-0.49700000000007,48.254906479826,0
-0.49685685787593,48.254910640912,0
-0.49671480508032,48.254923092502,0
-0.49657492265345,48.25494373984,0
-0.49643827512188,48.254972425794,0
-0.49630590239872,48.255008932063,0
-0.496178811871,48.255052980827,0
-0.49605797073442,48.255104236867,0
-0.49594429863349,48.255162310116,0
-0.4958386606635,48.255226758623,0
-0.49574186078712,48.255297091919,0
-0.49565463571592,48.255372774749,0
-0.49557764930341,48.255453231143,0
-0.49551148749212,48.2555378488,0
-0.49545665385338,48.255625983747,0
-0.49541356575361,48.255716965242,0
-0.49538255117634,48.255810100873,0
-0.49536384622444,48.255904681832,0
-0.49535759332124,48.255999988306,0
-0.49700000000007,48.256,0
</coordinates>
</LinearRing>
</outerBoundaryIs>
</Polygon>
</Placemark>
<Placemark>
<name>460</name>
<styleUrl>#stylehap2</styleUrl>
<Polygon>
<outerBoundaryIs>
<LinearRing>
<coordinates>
-0.495,48.259,0
-0.49335749694234,48.258999988305,0

-0.4933637441119,48.259095294956,0
-0.49338244424622,48.259189876443,0
-0.49341345509336,48.259283012935,0
-0.49345654070523,48.259373995599,0
-0.49351137323146,48.259462131988,0
-0.49357753541271,48.259546751315,0
-0.49365452375483,48.259627209556,0
-0.49374175235955,48.259702894355,0
-0.49383855738257,48.259773229682,0
-0.49394420208529,48.25983768022,0
-0.49405788244162,48.259895755438,0
-0.49417873325742,48.259947013326,0
-0.4943058347555,48.25999106376,0
-0.49443821957673,48.26002757147,0
-0.49457488014333,48.260056258594,0
-0.49471477632883,48.260076906792,0
-0.49485684337551,48.26008935891,0
-0.49499999999999,48.260093520173,0
-0.49514315662451,48.26008935891,0
-0.49528522367119,48.260076906792,0
-0.49542511985665,48.260056258594,0
-0.49556178042329,48.26002757147,0
-0.49569416524451,48.25999106376,0
-0.4958212667426,48.259947013326,0
-0.49594211755837,48.259895755438,0
-0.49605579791473,48.25983768022,0
-0.49616144261744,48.259773229682,0
-0.49625824764044,48.259702894355,0
-0.49634547624516,48.259627209556,0
-0.49642246458731,48.259546751315,0
-0.49648862676855,48.259462131988,0
-0.49654345929478,48.259373995599,0
-0.49658654490665,48.259283012935,0
-0.49661755575379,48.259189876443,0
-0.49663625588812,48.259095294956,0
-0.49664250305767,48.258999988305,0
-0.49663624978723,48.258904681831,0
-0.49661754373738,48.258810100872,0
-0.49658652733984,48.258716965241,0
-0.49654343671132,48.258625983746,0
-0.49648859985465,48.258537848799,0
-0.49642243416069,48.258453231142,0
-0.49634544323037,48.258372774748,0
-0.49625821304058,48.258297091919,0
-0.49616140748382,48.258226758622,0
-0.49605576331487,48.258162310116,0
-0.49594208454358,48.258104236867,0
-0.49582123631599,48.258052980826,0
-0.49569413833061,48.258008932063,0
-0.49556175783985,48.257972425794,0
-0.49542510228987,48.257943739839,0
-0.49528521165477,48.257923092502,0
-0.49514315052362,48.257910640912,0
-0.49499999999999,48.257906479826,0
-0.4948568494764,48.257910640912,0
-0.49471478834522,48.257923092502,0
-0.49457489771015,48.257943739839,0

-0.49443824216017,48.257972425794,0
-0.49430586166941,48.258008932063,0
-0.49417876368403,48.258052980826,0
-0.49405791545644,48.258104236867,0
-0.49394423668514,48.258162310116,0
-0.4938385925162,48.258226758622,0
-0.49374178695943,48.258297091919,0
-0.49365455676965,48.258372774748,0
-0.49357756583932,48.258453231142,0
-0.49351140014537,48.258537848799,0
-0.4934565632887,48.258625983746,0
-0.49341347266017,48.258716965241,0
-0.49338245626264,48.258810100872,0
-0.49336375021279,48.258904681831,0
-0.49335749694234,48.258999988305,0
-0.495,48.259,0
</coordinates>
</LinearRing>
</outerBoundaryIs>
</Polygon>
</Placemark>
<Placemark>
<name>461</name>
<styleUrl>#stylehap2</styleUrl>
<Polygon>
<outerBoundaryIs>
<LinearRing>
<coordinates>
-0.495,48.261,0
-0.49335743268099,48.260999988304,0
-0.49336368009476,48.261095294956,0
-0.49338238096051,48.261189876442,0
-0.49341339302071,48.261283012934,0
-0.4934564803181,48.261373995598,0
-0.49351131498942,48.261462131988,0
-0.49357747975908,48.261546751315,0
-0.49365447111322,48.261627209556,0
-0.49374170313062,48.261702894355,0
-0.49383851194102,48.261773229682,0
-0.49394416077701,48.26183768022,0
-0.49405784558103,48.261895755438,0
-0.49417870112509,48.261947013326,0
-0.49430580759601,48.261991063759,0
-0.49443819759681,48.262027571469,0
-0.49457486351033,48.262056258594,0
-0.4947147651693,48.262076906792,0
-0.49485683777442,48.26208935891,0
-0.49499999999999,48.262093520174,0
-0.49514316222559,48.26208935891,0
-0.49528523483071,48.262076906792,0
-0.49542513648968,48.262056258594,0
-0.49556180240321,48.262027571469,0
-0.49569419240401,48.261991063759,0
-0.49582129887493,48.261947013326,0
-0.49594215441899,48.261895755438,0
-0.49605583922301,48.26183768022,0
-0.496161488059,48.261773229682,0

-0.49625829686939,48.261702894355,0
-0.49634552888677,48.261627209556,0
-0.49642252024093,48.261546751315,0
-0.49648868501059,48.261462131988,0
-0.49654351968191,48.261373995598,0
-0.4965866069793,48.261283012934,0
-0.4966176190395,48.261189876442,0
-0.49663631990525,48.261095294956,0
-0.49664256731902,48.260999988304,0
-0.4966363138037,48.26090468183,0
-0.49661760702179,48.260810100871,0
-0.49658658941058,48.26071696524,0
-0.496543497096,48.260625983746,0
-0.49648865809373,48.260537848798,0
-0.49642248981099,48.260453231141,0
-0.49634549586837,48.260372774748,0
-0.49625826226575,48.260297091918,0
-0.49616145292153,48.260226758622,0
-0.49605580461936,48.260162310115,0
-0.49594212140056,48.260104236867,0
-0.49582126844498,48.260052980826,0
-0.49569416548715,48.260008932063,0
-0.49556177981728,48.259972425794,0
-0.49542511892096,48.259943739839,0
-0.495285222813,48.259923092502,0
-0.49514315612404,48.259910640912,0
-0.49499999999999,48.259906479826,0
-0.49485684387597,48.259910640912,0
-0.49471477718702,48.259923092502,0
-0.49457488107905,48.259943739839,0
-0.49443822018274,48.259972425794,0
-0.49430583451286,48.260008932063,0
-0.49417873155501,48.260052980826,0
-0.49405787859943,48.260104236867,0
-0.49394419538063,48.260162310115,0
-0.49383854707849,48.260226758622,0
-0.49374173773427,48.260297091918,0
-0.49365450413165,48.260372774748,0
-0.49357751018903,48.260453231141,0
-0.49351134190628,48.260537848798,0
-0.49345650290401,48.260625983746,0
-0.49341341058944,48.26071696524,0
-0.49338239297823,48.260810100871,0
-0.49336368619631,48.26090468183,0
-0.49335743268099,48.260999988304,0
-0.495,48.261,0
</coordinates>
</LinearRing>
</outerBoundaryIs>
</Polygon>
</Placemark>
<Placemark>
<name>471</name>
<styleUrl>#stylehap2</styleUrl>
<Polygon>
<outerBoundaryIs>
<LinearRing>

<coordinates>
-0.48800000000006,48.28,0
-0.48635682184753,48.279999988296,0
-0.48636307158252,48.280095294948,0
-0.48638177940071,48.280189876434,0
-0.48641280299175,48.280283012927,0
-0.48645590631066,48.280373995591,0
-0.4865107613723,48.280462131981,0
-0.48657695074598,48.280546751309,0
-0.48665397073057,48.280627209551,0
-0.48674123518707,48.28070289435,0
-0.48683807999829,48.280773229678,0
-0.48694376812283,48.280837680216,0
-0.48705749520421,48.280895755435,0
-0.48717839569256,48.280947013324,0
-0.48730554943265,48.280991063758,0
-0.48743798866775,48.281027571469,0
-0.48757470540591,48.281056258593,0
-0.48771465909298,48.281076906792,0
-0.48785678453365,48.28108935891,0
-0.48800000000005,48.281093520174,0
-0.48814321546645,48.28108935891,0
-0.48828534090712,48.281076906792,0
-0.48842529459422,48.281056258593,0
-0.48856201133238,48.281027571469,0
-0.48869445056745,48.280991063758,0
-0.48882160430757,48.280947013324,0
-0.48894250479592,48.280895755435,0
-0.48905623187727,48.280837680216,0
-0.48916192000184,48.280773229678,0
-0.48925876481306,48.28070289435,0
-0.48934602926953,48.280627209551,0
-0.48942304925415,48.280546751309,0
-0.48948923862782,48.280462131981,0
-0.48954409368947,48.280373995591,0
-0.48958719700836,48.280283012927,0
-0.48961822059939,48.280189876434,0
-0.48963692841758,48.280095294948,0
-0.4896431781526,48.279999988296,0
-0.48963692230969,48.279904681822,0
-0.48961820856918,48.279810100864,0
-0.48958717942136,48.279716965233,0
-0.48954407108005,48.279625983739,0
-0.48948921168295,48.279537848792,0
-0.48942301879258,48.279453231135,0
-0.4893459962168,48.279372774742,0
-0.48925873017344,48.279297091913,0
-0.48916188482783,48.279226758618,0
-0.48905619723765,48.279162310112,0
-0.48894247174316,48.279104236864,0
-0.488821573846,48.279052980824,0
-0.4886944236226,48.279008932061,0
-0.48856198872296,48.278972425793,0
-0.48842527700723,48.278943739839,0
-0.48828532887692,48.278923092502,0
-0.48814320935857,48.278910640912,0
-0.48800000000005,48.278906479826,0

-0.48785679064156,48.278910640912,0
-0.48771467112318,48.278923092502,0
-0.4875747229929,48.278943739839,0
-0.48743801127717,48.278972425793,0
-0.4873055763775,48.279008932061,0
-0.48717842615413,48.279052980824,0
-0.48705752825694,48.279104236864,0
-0.48694380276245,48.279162310112,0
-0.48683811517227,48.279226758618,0
-0.48674126982669,48.279297091913,0
-0.48665400378333,48.279372774742,0
-0.48657698120755,48.279453231135,0
-0.48651078831716,48.279537848792,0
-0.48645592892005,48.279625983739,0
-0.48641282057876,48.27971696523,0
-0.48638179143094,48.279810100864,0
-0.48636307769043,48.279904681822,0
-0.48635682184753,48.279999988296,0
-0.48800000000006,48.28,0
</coordinates>
</LinearRing>
</outerBoundaryIs>
</Polygon>
</Placemark>
<Placemark>
<name>472</name>
<styleUrl>#stylehap2</styleUrl>
<Polygon>
<outerBoundaryIs>
<LinearRing>
<coordinates>
-0.48800000000006,48.219,0
-0.48635878069444,48.218999988321,0
-0.48636502298561,48.219095294973,0
-0.48638370850837,48.219189876459,0
-0.48641469512187,48.21928301295,0
-0.48645774706228,48.219373995614,0
-0.48651253673539,48.219462132002,0
-0.48657864720789,48.219546751327,0
-0.48665557537908,48.219627209567,0
-0.48674273580854,48.219702894365,0
-0.48683946517079,48.21977322969,0
-0.48694502730303,48.219837680227,0
-0.48705861880767,48.219895755443,0
-0.48717937516674,48.21994701333,0
-0.487306377322,48.219991063763,0
-0.4874386586705,48.220027571472,0
-0.48757521242207,48.220056258595,0
-0.4877149992632,48.220076906793,0
-0.48785695526857,48.22008935891,0
-0.48800000000005,48.220093520174,0
-0.48814304473153,48.22008935891,0
-0.4882850007369,48.220076906793,0
-0.48842478757806,48.220056258595,0
-0.4885613413296,48.220027571472,0
-0.4886936226781,48.219991063763,0
-0.48882062483339,48.21994701333,0

-0.48894138119244,48.219895755443,0
-0.48905497269707,48.219837680227,0
-0.48916053482933,48.21977322969,0
-0.48925726419159,48.219702894365,0
-0.48934442462104,48.219627209567,0
-0.48942135279224,48.219546751327,0
-0.48948746326471,48.219462132002,0
-0.48954225293783,48.219373995614,0
-0.48958530487826,48.21928301295,0
-0.48961629149173,48.219189876459,0
-0.48963497701452,48.219095294973,0
-0.48964121930568,48.218999988321,0
-0.48963497092696,48.218904681847,0
-0.48961627950158,48.218810100888,0
-0.48958528734982,48.218716965256,0
-0.48954223040372,48.218625983761,0
-0.48948743640961,48.218537848812,0
-0.48942132243211,48.218453231154,0
-0.48934439167836,48.218372774759,0
-0.4892572296673,48.218297091928,0
-0.48916049977247,48.218226758631,0
-0.48905493817281,48.218162310122,0
-0.48894134824978,48.218104236872,0
-0.48882059447326,48.218052980831,0
-0.488693595823,48.218008932066,0
-0.4885613187955,48.217972425796,0
-0.48842477004961,48.217943739841,0
-0.48828498874675,48.217923092503,0
-0.48814303864397,48.217910640912,0
-0.48800000000005,48.217906479827,0
-0.48785696135613,48.217910640912,0
-0.48771501125336,48.217923092503,0
-0.48757522995049,48.217943739841,0
-0.4874386812046,48.217972425796,0
-0.48730640417711,48.218008932066,0
-0.48717940552686,48.218052980831,0
-0.48705865175035,48.218104236872,0
-0.48694506182731,48.218162310122,0
-0.48683950022766,48.218226758631,0
-0.4867427703328,48.218297091928,0
-0.48665560832174,48.218372774759,0
-0.48657867756802,48.218453231154,0
-0.48651256359049,48.218537848812,0
-0.4864577695964,48.218625983761,0
-0.48641471265029,48.218716965256,0
-0.48638372049853,48.218810100888,0
-0.48636502907317,48.218904681847,0
-0.48635878069444,48.218999988321,0
-0.48800000000006,48.219,0
</coordinates>
</LinearRing>
</outerBoundaryIs>
</Polygon>
</Placemark>
<Placemark>
<name>473</name>
<styleUrl>#stylehap2</styleUrl>

<Polygon>
<outerBoundaryIs>
<LinearRing>
<coordinates>
-0.48800000000006,48.21,0
-0.48635906915198,48.209999988325,0
-0.48636531034697,48.210095294976,0
-0.48638399258653,48.210189876462,0
-0.48641497375474,48.210283012954,0
-0.48645801812925,48.210373995617,0
-0.4865127981733,48.210462132005,0
-0.4865788970269,48.21054675133,0
-0.48665581167776,48.21062720957,0
-0.48674295678831,48.210702894367,0
-0.48683966914969,48.210773229692,0
-0.48694521272845,48.210837680228,0
-0.48705878426821,48.210895755444,0
-0.48717951940296,48.210947013331,0
-0.48730649923602,48.210991063763,0
-0.48743875733433,48.211027571472,0
-0.48757528708468,48.211056258595,0
-0.48771504935627,48.211076906793,0
-0.48785698041081,48.21108935891,0
-0.48800000000005,48.211093520174,0
-0.4881430195893,48.21108935891,0
-0.48828495064383,48.211076906793,0
-0.48842471291543,48.211056258595,0
-0.48856124266579,48.211027571472,0
-0.48869350076408,48.210991063763,0
-0.48882048059714,48.210947013331,0
-0.48894121573189,48.210895755444,0
-0.48905478727165,48.210837680228,0
-0.48916033085044,48.210773229692,0
-0.48925704321179,48.210702894367,0
-0.48934418832237,48.21062720957,0
-0.4894211029732,48.21054675133,0
-0.48948720182681,48.210462132005,0
-0.48954198187088,48.210373995617,0
-0.48958502624536,48.210283012954,0
-0.48961600741357,48.210189876462,0
-0.48963468965316,48.210095294976,0
-0.48964093084815,48.209999988325,0
-0.48963468356858,48.209904681851,0
-0.48961599542932,48.209810100891,0
-0.48958500872554,48.20971696526,0
-0.48954195934785,48.209625983764,0
-0.48948717498491,48.209537848815,0
-0.48942107262801,48.209453231157,0
-0.48934415539589,48.209372774762,0
-0.4892570087045,48.20929709193,0
-0.4891602958108,48.209226758632,0
-0.48905475276436,48.209162310124,0
-0.48894118280544,48.209104236873,0
-0.48882045025195,48.209052980831,0
-0.48869347392218,48.209008932066,0
-0.48856122014276,48.208972425797,0
-0.48842469539563,48.208943739841,0

-0.48828493865958,48.208923092503,0
-0.48814301350474,48.208910640912,0
-0.48800000000005,48.208906479826,0
-0.48785698649538,48.208910640912,0
-0.48771506134053,48.208923092503,0
-0.48757530460449,48.208943739841,0
-0.48743877985737,48.208972425797,0
-0.48730652607795,48.209008932066,0
-0.48717954974815,48.209052980831,0
-0.48705881719469,48.209104236873,0
-0.48694524723574,48.209162310124,0
-0.4868397041893,48.209226758632,0
-0.4867429912956,48.20929709193,0
-0.48665584460421,48.209372774762,0
-0.48657892737209,48.209453231157,0
-0.48651282501519,48.209537848815,0
-0.48645804065228,48.209625983764,0
-0.48641499127456,48.20971696526,0
-0.48638400457078,48.209810100891,0
-0.48636531643153,48.209904681851,0
-0.48635906915198,48.209999988325,0
-0.48800000000006,48.21,0
</coordinates>
</LinearRing>
</outerBoundaryIs>
</Polygon>
</Placemark>
<Placemark>
<name>474</name>
<styleUrl>#stylehap2</styleUrl>
<Polygon>
<outerBoundaryIs>
<LinearRing>
<coordinates>
-0.48800000000006,48.108,0
-0.4863623284447,48.107999988367,0
-0.486368557254,48.108095295018,0
-0.48638720239659,48.108189876503,0
-0.48641812203845,48.108283012993,0
-0.48646108092514,48.108373995654,0
-0.48651575217025,48.108462132039,0
-0.4865817197415,48.108546751362,0
-0.4866584816255,48.108627209598,0
-0.48674545364725,48.108702894392,0
-0.486841973915,48.108773229713,0
-0.4869473078573,48.108837680246,0
-0.48706065381319,48.108895755458,0
-0.48718114913363,48.108947013341,0
-0.48730787674723,48.108991063771,0
-0.48743987214066,48.109027571477,0
-0.48757613070037,48.109056258598,0
-0.48771561535982,48.109076906794,0
-0.48785726449378,48.109089358911,0
-0.48800000000005,48.109093520174,0
-0.48814273550633,48.109089358911,0
-0.48828438464031,48.109076906794,0
-0.48842386929973,48.109056258598,0

-0.48856012785944,48.109027571477,0
-0.48869212325289,48.108991063771,0
-0.4888188508665,48.108947013341,0
-0.48893934618694,48.108895755458,0
-0.48905269214283,48.108837680246,0
-0.4891580260851,48.108773229713,0
-0.48925454635286,48.108702894392,0
-0.4893415183746,48.108627209598,0
-0.48941828025861,48.108546751362,0
-0.48948424782988,48.108462132039,0
-0.48953891907499,48.108373995654,0
-0.48958187796168,48.108283012993,0
-0.48961279760354,48.108189876503,0
-0.4896314427461,48.108095295018,0
-0.4896376715554,48.107999988367,0
-0.48963143669533,48.107904681892,0
-0.48961278568585,48.107810100932,0
-0.48958186053919,48.107716965299,0
-0.48953889667706,48.107625983801,0
-0.48948422113706,48.10753784885,0
-0.48941825008199,48.107453231188,0
-0.48934148563104,48.10737277479,0
-0.48925451203727,48.107297091955,0
-0.48915799124014,48.107226758653,0
-0.48905265782722,48.107162310141,0
-0.48893931344336,48.107104236887,0
-0.48881882068985,48.107052980842,0
-0.4886920965601,48.107008932074,0
-0.48856010546155,48.106972425801,0
-0.48842385187724,48.106943739844,0
-0.48828437272262,48.106923092504,0
-0.48814272945556,48.106910640913,0
-0.48800000000005,48.106906479826,0
-0.48785727054457,48.106910640913,0
-0.48771562727749,48.106923092504,0
-0.48757614812286,48.106943739844,0
-0.48743989453858,48.106972425801,0
-0.48730790344003,48.107008932074,0
-0.48718117931025,48.107052980842,0
-0.48706068655675,48.107104236887,0
-0.48694734217289,48.107162310141,0
-0.48684200875999,48.107226758653,0
-0.48674548796286,48.107297091955,0
-0.48665851436909,48.10737277479,0
-0.48658174991812,48.107453231188,0
-0.48651577886304,48.10753784885,0
-0.48646110332306,48.107625983801,0
-0.48641813946094,48.107716965299,0
-0.48638721431426,48.107810100932,0
-0.48636856330477,48.107904681892,0
-0.4863623284447,48.107999988367,0
-0.48800000000006,48.108,0
</coordinates>
</LinearRing>
</outerBoundaryIs>
</Polygon>
</Placemark>

<Placemark>
<name>475</name>
<styleUrl>#stylehap2</styleUrl>
<Polygon>
<outerBoundaryIs>
<LinearRing>
<coordinates>
-0.48699999999997,48.235,0
-0.48535826753043,48.234999988315,0
-0.48536451177166,48.235095294966,0
-0.48538320313524,48.235189876452,0
-0.48541419943582,48.235283012944,0
-0.48545726483598,48.235373995608,0
-0.48551207163908,48.235462131996,0
-0.48557820278152,48.235546751323,0
-0.48565515500529,48.235627209563,0
-0.48574234268696,48.235702894361,0
-0.48583910229367,48.235773229687,0
-0.48594469743243,48.235837680224,0
-0.48605832445444,48.235895755441,0
-0.48617911857138,48.235947013328,0
-0.48630616043773,48.235991063761,0
-0.48643848314815,48.236027571471,0
-0.48657507959772,48.236056258594,0
-0.48671491014789,48.236076906793,0
-0.48685691054065,48.23608935891,0
-0.48699999999996,48.236093520174,0
-0.48714308945928,48.23608935891,0
-0.48728508985204,48.236076906793,0
-0.48742492040221,48.236056258594,0
-0.48756151685178,48.236027571471,0
-0.48769383956222,48.235991063761,0
-0.48782088142854,48.235947013328,0
-0.48794167554551,48.235895755441,0
-0.4880553025675,48.235837680224,0
-0.48816089770626,48.235773229687,0
-0.48825765731297,48.235702894361,0
-0.48834484499464,48.235627209563,0
-0.48842179721841,48.235546751323,0
-0.48848792836085,48.235462131996,0
-0.48854273516395,48.235373995608,0
-0.48858580056411,48.235283012944,0
-0.48861679686471,48.235189876452,0
-0.48863548822826,48.235095294966,0
-0.48864173246952,48.234999988315,0
-0.48863548213537,48.23490468184,0
-0.48861678486405,48.234810100882,0
-0.48858578302035,48.23471696525,0
-0.48854271261013,48.234625983755,0
-0.48848790148224,48.234537848807,0
-0.48842176683172,48.234453231149,0
-0.48834481202315,48.234372774755,0
-0.48825762275848,48.234297091924,0
-0.48816086261874,48.234226758627,0
-0.48805526801302,48.23416231012,0
-0.487941642574,48.23410423687,0
-0.48782085104183,48.234052980829,0

-0.48769381268361,48.234008932064,0
-0.48756149429794,48.233972425795,0
-0.48742490285845,48.23394373984,0
-0.4872850778514,48.233923092503,0
-0.48714308336641,48.233910640912,0
-0.48699999999996,48.233906479827,0
-0.48685691663352,48.233910640912,0
-0.48671492214853,48.233923092503,0
-0.48657509714148,48.23394373984,0
-0.48643850570199,48.233972425795,0
-0.48630618731634,48.234008932064,0
-0.4861791489581,48.234052980829,0
-0.48605835742593,48.23410423687,0
-0.48594473198691,48.23416231012,0
-0.48583913738119,48.234226758627,0
-0.48574237724145,48.234297091924,0
-0.4856551879768,48.234372774755,0
-0.48557823316821,48.234453231149,0
-0.48551209851769,48.234537848807,0
-0.48545728738983,48.234625983755,0
-0.48541421697958,48.23471696525,0
-0.48538321513588,48.234810100882,0
-0.48536451786456,48.23490468184,0
-0.48535826753043,48.234999988315,0
-0.48699999999997,48.235,0
</coordinates>
</LinearRing>
</outerBoundaryIs>
</Polygon>
</Placemark>
<Placemark>
<name>478</name>
<styleUrl>#stylehap2</styleUrl>
<Polygon>
<outerBoundaryIs>
<LinearRing>
<coordinates>
-0.48599999999999,48.274,0
-0.48435701481085,48.273999988298,0
-0.48436326381255,48.27409529495,0
-0.48438196943446,48.274189876437,0
-0.48441298938289,48.274283012929,0
-0.48445608764057,48.274373995594,0
-0.48451093626089,48.274462131983,0
-0.4845771178621,48.274546751311,0
-0.48465412880229,48.274627209552,0
-0.48474138301121,48.274702894352,0
-0.48483821644971,48.274773229679,0
-0.48494389216294,48.274837680217,0
-0.48505760588881,48.274895755436,0
-0.48517849217917,48.274947013324,0
-0.48530563098686,48.274991063759,0
-0.48543805466877,48.275027571469,0
-0.48557475535131,48.275056258593,0
-0.48571469260262,48.275076906792,0
-0.48585680135245,48.27508935891,0
-0.48599999999998,48.275093520174,0

-0.48614319864753,48.27508935891,0
-0.48628530739737,48.275076906792,0
-0.48642524464865,48.275056258593,0
-0.48656194533122,48.275027571469,0
-0.48669436901312,48.274991063759,0
-0.48682150782079,48.274947013324,0
-0.48694239411117,48.274895755436,0
-0.48705610783705,48.274837680217,0
-0.48716178355027,48.274773229679,0
-0.48725861698877,48.274702894352,0
-0.4873458711977,48.274627209552,0
-0.48742288213789,48.274546751311,0
-0.48748906373909,48.274462131983,0
-0.48754391235941,48.274373995594,0
-0.48758701061709,48.274283012929,0
-0.4876180305655,48.274189876437,0
-0.48763673618741,48.27409529495,0
-0.48764298518914,48.273999988298,0
-0.48763673008153,48.273904681824,0
-0.48761801853924,48.273810100866,0
-0.48758699303585,48.273716965235,0
-0.48754388975742,48.273625983741,0
-0.48748903680308,48.273537848794,0
-0.48742285168629,48.273453231137,0
-0.48734583815578,48.273372774744,0
-0.48725858236052,48.273297091915,0
-0.48716174838781,48.273226758619,0
-0.48705607320879,48.273162310113,0
-0.48694236106928,48.273104236865,0
-0.48682147736922,48.273052980825,0
-0.48669434207711,48.273008932062,0
-0.48656192272923,48.272972425794,0
-0.48642522706744,48.272943739839,0
-0.48628529537108,48.272923092502,0
-0.48614319254163,48.272910640912,0
-0.48599999999998,48.272906479827,0
-0.48585680745835,48.272910640912,0
-0.48571470462888,48.272923092502,0
-0.48557477293255,48.272943739839,0
-0.48543807727076,48.272972425794,0
-0.48530565792288,48.273008932062,0
-0.48517852263077,48.273052980825,0
-0.48505763893071,48.273104236865,0
-0.48494392679119,48.273162310113,0
-0.48483825161217,48.273226758619,0
-0.48474141763946,48.273297091915,0
-0.48465416184418,48.273372774744,0
-0.48457714831367,48.273453231137,0
-0.48451096319688,48.273537848794,0
-0.48445611024256,48.273625983741,0
-0.48441300696413,48.273716965235,0
-0.48438198146074,48.273810100866,0
-0.48436326991846,48.273904681824,0
-0.48435701481085,48.273999988298,0
-0.48599999999999,48.274,0
</coordinates>
</LinearRing>

</outerBoundaryIs>
</Polygon>
</Placemark>
<Placemark>
<name>480</name>
<styleUrl>#stylehap2</styleUrl>
<Polygon>
<outerBoundaryIs>
<LinearRing>
<coordinates>
-0.485999999999999,48.209,0
-0.48435910119398,48.208999988325,0
-0.4843653422672,48.209095294977,0
-0.48438402414209,48.209189876463,0
-0.48441500470544,48.209283012954,0
-0.48445804823949,48.209373995617,0
-0.48451282721395,48.209462132005,0
-0.48457892477691,48.209546751331,0
-0.48465583792591,48.20962720957,0
-0.48474298133485,48.209702894367,0
-0.48483969180774,48.209773229693,0
-0.48494523332557,48.209837680228,0
-0.48505880264762,48.209895755445,0
-0.48517953542477,48.209947013331,0
-0.48530651277827,48.209991063763,0
-0.48543876829392,48.210027571472,0
-0.48557529537819,48.210056258595,0
-0.48571505492059,48.210076906793,0
-0.48585698320357,48.21008935891,0
-0.48599999999998,48.210093520174,0
-0.48614301679642,48.21008935891,0
-0.48628494507939,48.210076906793,0
-0.48642470462179,48.210056258595,0
-0.48656123170606,48.210027571472,0
-0.48669348722171,48.209991063763,0
-0.48682046457522,48.209947013331,0
-0.48694119735236,48.209895755445,0
-0.48705476667439,48.209837680228,0
-0.48716030819222,48.209773229693,0
-0.48725701866514,48.209702894367,0
-0.48734416207407,48.20962720957,0
-0.48742107522307,48.209546751331,0
-0.48748717278603,48.209462132005,0
-0.4875419517605,48.209373995617,0
-0.48758499529455,48.209283012954,0
-0.48761597585789,48.209189876463,0
-0.48763465773276,48.209095294977,0
-0.487640898806,48.208999988325,0
-0.48763465164853,48.208904681851,0
-0.4876159638743,48.208810100892,0
-0.4875849777757,48.20871696526,0
-0.48754192923868,48.208625983764,0
-0.48748714594559,48.208537848816,0
-0.48742104487951,48.208453231157,0
-0.4873441291494,48.208372774762,0
-0.48725698415973,48.208297091931,0
-0.48716027315452,48.208226758633,0

-0.48705473216901,48.208162310124,0
-0.48694116442769,48.208104236874,0
-0.48682043423168,48.208052980832,0
-0.48669346038127,48.208008932066,0
-0.48656120918428,48.207972425797,0
-0.48642468710294,48.207943739841,0
-0.4862849330958,48.207923092503,0
-0.4861430107122,48.207910640912,0
-0.48599999999998,48.207906479826,0
-0.48585698928779,48.207910640912,0
-0.48571506690419,48.207923092503,0
-0.48557531289705,48.207943739841,0
-0.48543879081571,48.207972425797,0
-0.48530653961869,48.208008932066,0
-0.4851795657683,48.208052980832,0
-0.48505883557229,48.208104236874,0
-0.48494526783098,48.208162310124,0
-0.48483972684544,48.208226758633,0
-0.48474301584025,48.208297091931,0
-0.48465587085058,48.208372774762,0
-0.48457895512045,48.208453231157,0
-0.4845128540544,48.208537848816,0
-0.4844580707613,48.208625983764,0
-0.48441502222429,48.20871696526,0
-0.48438403612568,48.208810100892,0
-0.48436534835145,48.208904681851,0
-0.48435910119398,48.208999988325,0
-0.48599999999999,48.209,0
</coordinates>
</LinearRing>
</outerBoundaryIs>
</Polygon>
</Placemark>
<Placemark>
<name>482</name>
<styleUrl>#stylehap2</styleUrl>
<Polygon>
<outerBoundaryIs>
<LinearRing>
<coordinates>
-0.48199999999997,48.243,0
-0.48035801078008,48.242999988311,0
-0.480364255997,48.243095294963,0
-0.48038295028288,48.243189876449,0
-0.48041395143019,48.243283012941,0
-0.48045702356465,48.243373995605,0
-0.48051183893837,48.243462131994,0
-0.48057798042256,48.24354675132,0
-0.4806549446805,48.243627209561,0
-0.48074214599723,48.243702894359,0
-0.48083892073608,48.243773229686,0
-0.48094453238894,48.243837680223,0
-0.48105817718128,48.24389575544,0
-0.48117899018957,48.243947013328,0
-0.48130605192446,48.243991063761,0
-0.48143839532944,48.24402757147,0
-0.48157501314199,48.244056258594,0

-0.48171486556104,48.244076906792,0
-0.48185688816204,48.24408935891,0
-0.48199999999996,48.244093520174,0
-0.48214311183789,48.24408935891,0
-0.48228513443891,48.244076906792,0
-0.48242498685794,48.244056258594,0
-0.48256160467051,48.24402757147,0
-0.48269394807547,48.243991063761,0
-0.48282100981036,48.243947013328,0
-0.48294182281868,48.24389575544,0
-0.48305546761102,48.243837680223,0
-0.48316107926385,48.243773229686,0
-0.4832578540027,48.243702894359,0
-0.48334505531943,48.243627209561,0
-0.4834220195774,48.24354675132,0
-0.48348816106158,48.243462131994,0
-0.48354297643531,48.243373995605,0
-0.48358604856974,48.243283012941,0
-0.48361704971705,48.243189876449,0
-0.48363574400293,48.243095294963,0
-0.48364198921987,48.242999988311,0
-0.48363573790738,48.242904681837,0
-0.48361703771117,48.242810100878,0
-0.4835860310183,48.242716965247,0
-0.48354295387159,48.242625983752,0
-0.48348813417119,48.242537848804,0
-0.4834219891774,48.242453231147,0
-0.48334502233351,48.242372774753,0
-0.48325781943312,48.242297091922,0
-0.48316104416096,48.242226758626,0
-0.48305543304141,48.242162310118,0
-0.48294178983274,48.242104236869,0
-0.48282097941037,48.242052980828,0
-0.4826939211851,48.242008932064,0
-0.48256158210682,48.241972425795,0
-0.48242496930649,48.24194373984,0
-0.48228512243301,48.241923092503,0
-0.48214310574235,48.241910640912,0
-0.48199999999996,48.241906479826,0
-0.48185689425761,48.241910640912,0
-0.48171487756692,48.241923092503,0
-0.48157503069344,48.24194373984,0
-0.48143841789314,48.241972425795,0
-0.48130607881483,48.242008932064,0
-0.48117902058956,48.242052980828,0
-0.48105821016719,48.242104236869,0
-0.48094456695854,48.242162310118,0
-0.48083895583897,48.242226758626,0
-0.48074218056684,48.242297091922,0
-0.48065497766644,48.242372774753,0
-0.48057801082255,48.242453231147,0
-0.48051186582874,48.242537848804,0
-0.48045704612834,48.242625983752,0
-0.48041396898163,48.242716965247,0
-0.48038296228878,48.242810100878,0
-0.48036426209255,48.242904681837,0
-0.48035801078008,48.242999988311,0

-0.48199999999997,48.243,0
</coordinates>
</LinearRing>
</outerBoundaryIs>
</Polygon>
</Placemark>
<Placemark>
<name>489</name>
<styleUrl>#stylehap2</styleUrl>
<Polygon>
<outerBoundaryIs>
<LinearRing>
<coordinates>
-0.476,48.191,0
-0.47435967765209,48.190999988333,0
-0.4743659165347,48.191095294984,0
-0.47438459184838,48.19118987647,0
-0.4744155615298,48.191283012961,0
-0.47445858994398,48.191373995624,0
-0.47451334967561,48.191462132011,0
-0.47457942401919,48.191546751336,0
-0.47465631014892,48.191627209575,0
-0.47474342294433,48.191702894372,0
-0.47484009944241,48.191773229696,0
-0.47494560388266,48.191837680232,0
-0.47505913330661,48.191895755447,0
-0.47517982366875,48.191947013333,0
-0.47530675641319,48.191991063765,0
-0.47543896546528,48.192027571473,0
-0.47557544458514,48.192056258596,0
-0.47571515502738,48.192076906793,0
-0.47585703344819,48.19208935891,0
-0.47600000000001,48.192093520174,0
-0.4761429665518,48.19208935891,0
-0.4762848449726,48.192076906793,0
-0.47642455541485,48.192056258596,0
-0.47656103453473,48.192027571473,0
-0.4766932435868,48.191991063765,0
-0.47682017633127,48.191947013333,0
-0.4769408666934,48.191895755447,0
-0.47705439611732,48.191837680232,0
-0.4771599005576,48.191773229696,0
-0.47725657705565,48.191702894372,0
-0.47734368985106,48.191627209575,0
-0.47742057598082,48.191546751336,0
-0.47748665032438,48.191462132011,0
-0.47754141005601,48.191373995624,0
-0.47758443847018,48.191283012961,0
-0.47761540815161,48.19118987647,0
-0.47763408346529,48.191095294984,0
-0.47764032234792,48.190999988333,0
-0.47763407738705,48.190904681858,0
-0.47761539617979,48.190810100899,0
-0.47758442096856,48.190716965267,0
-0.47754138755636,48.190625983771,0
-0.47748662351035,48.190537848822,0
-0.4774205456671,48.190453231163,0

-0.47734365695876,48.190372774767,0
-0.47725654258419,48.190297091935,0
-0.47715986555433,48.190226758636,0
-0.47705436164584,48.190162310127,0
-0.47694083380109,48.190104236876,0
-0.47682014601755,48.190052980833,0
-0.47669321677276,48.190008932068,0
-0.47656101203508,48.189972425797,0
-0.47642453791322,48.189943739841,0
-0.47628483300079,48.189923092503,0
-0.47614296047355,48.189910640912,0
-0.47600000000001,48.189906479827,0
-0.47585703952643,48.189910640912,0
-0.4757151669992,48.189923092503,0
-0.47557546208676,48.189943739841,0
-0.47543898796494,48.189972425797,0
-0.47530678322725,48.190008932068,0
-0.47517985398243,48.190052980833,0
-0.47505916619889,48.190104236876,0
-0.47494563835415,48.190162310127,0
-0.47484013444566,48.190226758636,0
-0.47474345741582,48.190297091935,0
-0.47465634304123,48.190372774767,0
-0.47457945433291,48.190453231163,0
-0.47451337648967,48.190537848822,0
-0.47445861244366,48.190625983771,0
-0.47441557903143,48.190716965267,0
-0.47438460382019,48.190810100899,0
-0.47436592261297,48.190904681858,0
-0.47435967765209,48.190999988333,0
-0.476,48.191,0
</coordinates>
</LinearRing>
</outerBoundaryIs>
</Polygon>
</Placemark>
<Placemark>
<name>491</name>
<styleUrl>#stylehap2</styleUrl>
<Polygon>
<outerBoundaryIs>
<LinearRing>
<coordinates>
-0.47399999999993,48.432,0
-0.472351912237,48.431999988233,0
-0.47235818062903,48.432095294886,0
-0.47237694432791,48.432189876373,0
-0.47240806059863,48.432283012869,0
-0.47245129269135,48.432373995536,0
-0.47250631164142,48.43246213193,0
-0.47257269877118,48.432546751262,0
-0.47264994887489,48.432627209509,0
-0.47273747406237,48.432702894313,0
-0.47283460823231,48.432773229647,0
-0.47294061214117,48.432837680191,0
-0.47305467902907,48.432895755414,0
-0.47317594075989,48.432947013308,0

-0.47330347442889,48.432991063747,0
-0.47343630938746,48.433027571461,0
-0.47357343463158,48.433056258589,0
-0.47371380649765,48.43307690679,0
-0.47385635660725,48.43308935891,0
-0.47399999999993,48.433093520174,0
-0.47414364339262,48.43308935891,0
-0.4742861935022,48.43307690679,0
-0.47442656536829,48.433056258589,0
-0.47456369061241,48.433027571461,0
-0.47469652557098,48.432991063747,0
-0.47482405923998,48.432947013308,0
-0.4749453209708,48.432895755414,0
-0.47505938785869,48.432837680191,0
-0.47516539176756,48.432773229647,0
-0.4752625259375,48.432702894313,0
-0.47535005112498,48.432627209509,0
-0.47542730122868,48.432546751262,0
-0.47549368835845,48.43246213193,0
-0.47554870730852,48.432373995536,0
-0.47559193940124,48.432283012869,0
-0.47562305567194,48.432189876373,0
-0.47564181937084,48.432095294886,0
-0.47564808776287,48.431999988233,0
-0.47564181321189,48.43190468176,0
-0.47562304354115,48.431810100803,0
-0.47559192166721,48.431716965175,0
-0.47554868451007,48.431625983683,0
-0.47549366118832,48.43153784874,0
-0.47542727051244,48.431453231088,0
-0.4753500177959,48.4313727747,0
-0.47526249100827,48.431297091877,0
-0.47516535629949,48.431226758587,0
-0.47505935292946,48.431162310086,0
-0.47494528764171,48.431104236843,0
-0.47482402852374,48.431052980809,0
-0.47469649840087,48.43100893205,0
-0.47456366781397,48.430972425786,0
-0.47442654763426,48.430943739835,0
-0.47428618137141,48.4309230925,0
-0.47414363723364,48.430910640912,0
-0.47399999999993,48.430906479826,0
-0.47385636276621,48.430910640912,0
-0.47371381862846,48.4309230925,0
-0.47357345236561,48.430943739835,0
-0.4734363321859,48.430972425786,0
-0.473303501599,48.43100893205,0
-0.47317597147613,48.431052980809,0
-0.47305471235816,48.431104236843,0
-0.47294064707041,48.431162310086,0
-0.47283464370038,48.431226758587,0
-0.4727375089916,48.431297091877,0
-0.47264998220397,48.4313727747,0
-0.47257272948743,48.431453231088,0
-0.47250633881152,48.43153784874,0
-0.47245131548977,48.431625983683,0
-0.47240807833266,48.431716965175,0

-0.47237695645872,48.431810100803,0
-0.47235818678798,48.43190468176,0
-0.472351912237,48.431999988233,0
-0.47399999999993,48.432,0
</coordinates>
</LinearRing>
</outerBoundaryIs>
</Polygon>
</Placemark>
<Placemark>
<name>492</name>
<styleUrl>#stylehap2</styleUrl>
<Polygon>
<outerBoundaryIs>
<LinearRing>
<coordinates>
-0.47399999999993,48.28,0
-0.47235682184739,48.279999988296,0
-0.4723630715824,48.280095294948,0
-0.4723817794006,48.280189876434,0
-0.47241280299163,48.280283012927,0
-0.47245590631052,48.280373995591,0
-0.47251076137219,48.280462131981,0
-0.47257695074584,48.280546751309,0
-0.47265397073046,48.280627209551,0
-0.47274123518692,48.28070289435,0
-0.47283807999815,48.280773229678,0
-0.47294376812271,48.280837680216,0
-0.47305749520407,48.280895755435,0
-0.47317839569242,48.280947013324,0
-0.47330554943254,48.280991063758,0
-0.47343798866764,48.281027571469,0
-0.47357470540576,48.281056258593,0
-0.47371465909286,48.281076906792,0
-0.47385678453353,48.28108935891,0
-0.47399999999993,48.281093520174,0
-0.47414321546634,48.28108935891,0
-0.47428534090701,48.281076906792,0
-0.47442529459411,48.281056258593,0
-0.47456201133223,48.281027571469,0
-0.47469445056733,48.280991063758,0
-0.47482160430745,48.280947013324,0
-0.47494250479578,48.280895755435,0
-0.47505623187716,48.280837680216,0
-0.47516192000172,48.280773229678,0
-0.47525876481292,48.28070289435,0
-0.47534602926941,48.280627209551,0
-0.47542304925401,48.280546751309,0
-0.47548923862768,48.280462131981,0
-0.47554409368935,48.280373995591,0
-0.47558719700824,48.280283012927,0
-0.47561822059927,48.280189876434,0
-0.47563692841747,48.280095294948,0
-0.47564317815245,48.279999988296,0
-0.47563692230955,48.279904681822,0
-0.47561820856907,48.279810100864,0
-0.47558717942125,48.279716965233,0

-0.47554407107994,48.279625983739,0
-0.47548921168283,48.279537848792,0
-0.47542301879244,48.279453231135,0
-0.47534599621666,48.279372774742,0
-0.47525873017329,48.279297091913,0
-0.47516188482771,48.279226758618,0
-0.47505619723753,48.279162310112,0
-0.47494247174305,48.279104236864,0
-0.47482157384585,48.279052980824,0
-0.47469442362249,48.279008932061,0
-0.47456198872284,48.278972425793,0
-0.47442527700709,48.278943739839,0
-0.4742853288768,48.278923092502,0
-0.47414320935843,48.278910640912,0
-0.47399999999993,48.278906479826,0
-0.47385679064142,48.278910640912,0
-0.47371467112307,48.278923092502,0
-0.47357472299278,48.278943739839,0
-0.47343801127703,48.278972425793,0
-0.47330557637738,48.279008932061,0
-0.47317842615399,48.279052980824,0
-0.47305752825682,48.279104236864,0
-0.47294380276234,48.279162310112,0
-0.47283811517216,48.279226758618,0
-0.47274126982655,48.279297091913,0
-0.47265400378321,48.279372774742,0
-0.47257698120743,48.279453231135,0
-0.47251078831704,48.279537848792,0
-0.47245592891993,48.279625983739,0
-0.47241282057862,48.279716965233,0
-0.4723817914308,48.279810100864,0
-0.47236307769029,48.279904681822,0
-0.47235682184739,48.279999988296,0
-0.47399999999993,48.28,0
</coordinates>
</LinearRing>
</outerBoundaryIs>
</Polygon>
</Placemark>
<Placemark>
<name>493</name>
<styleUrl>#stylehap2</styleUrl>
<Polygon>
<outerBoundaryIs>
<LinearRing>
<coordinates>
-0.47199999999998,48.222,0
-0.47035868451031,48.22199998832,0
-0.47036492716699,48.222095294971,0
-0.47038361378452,48.222189876457,0
-0.47041460221368,48.222283012949,0
-0.47045765667691,48.222373995613,0
-0.47051244956075,48.222462132001,0
-0.47057856390748,48.222546751327,0
-0.47065549658694,48.222627209567,0
-0.47074266212437,48.222702894364,0
-0.47083939715544,48.22277322969,0

-0.47094496547421,48.222837680226,0
-0.471058563636,48.222895755443,0
-0.47117932707214,48.22294701333,0
-0.47130633667059,48.222991063762,0
-0.4714386257717,48.223027571471,0
-0.47157518752628,48.223056258595,0
-0.47171498255996,48.223076906793,0
-0.471856946885,48.22308935891,0
-0.47199999999999,48.223093520174,0
-0.47214305311495,48.22308935891,0
-0.47228501744,48.223076906793,0
-0.47242481247367,48.223056258595,0
-0.47256137422826,48.223027571471,0
-0.47269366332937,48.222991063762,0
-0.47282067292782,48.22294701333,0
-0.47294143636398,48.222895755443,0
-0.47305503452575,48.222837680226,0
-0.47316060284452,48.22277322969,0
-0.47325733787559,48.222702894364,0
-0.47334450341302,48.222627209567,0
-0.47342143609248,48.222546751327,0
-0.4734875504392,48.222462132001,0
-0.47354234332305,48.222373995613,0
-0.47358539778627,48.222283012949,0
-0.47361638621544,48.222189876457,0
-0.47363507283296,48.222095294971,0
-0.47364131548964,48.22199998832,0
-0.47363506674442,48.221904681846,0
-0.47361637422332,48.221810100887,0
-0.47358538025495,48.221716965255,0
-0.47354232078523,48.22162598376,0
-0.4734875235797,48.221537848811,0
-0.47342140572737,48.221453231153,0
-0.47334447046496,48.221372774758,0
-0.47325730334566,48.221297091927,0
-0.47316056778191,48.22122675863,0
-0.47305499999582,48.221162310122,0
-0.4729414034159,48.221104236872,0
-0.47282064256271,48.22105298083,0
-0.47269363646984,48.221008932065,0
-0.47256135169047,48.220972425796,0
-0.47242479494235,48.22094373984,0
-0.47228500544788,48.220923092503,0
-0.47214304702638,48.220910640912,0
-0.47199999999999,48.220906479827,0
-0.47185695297358,48.220910640912,0
-0.47171499455208,48.220923092503,0
-0.4715752050576,48.22094373984,0
-0.47143864830949,48.220972425796,0
-0.47130636353012,48.221008932065,0
-0.47117935743725,48.22105298083,0
-0.47105859658405,48.221104236872,0
-0.47094500000414,48.221162310122,0
-0.47083943221805,48.22122675863,0
-0.4707426966543,48.221297091927,0
-0.47065552953502,48.221372774758,0
-0.47057859427259,48.221453231153,0

-0.47051247642028,48.221537848811,0
-0.47045767921473,48.22162598376,0
-0.470414619745,48.221716965255,0
-0.47038362577664,48.221810100887,0
-0.47036493325554,48.221904681846,0
-0.47035868451031,48.22199998832,0
-0.47199999999998,48.222,0
</coordinates>
</LinearRing>
</outerBoundaryIs>
</Polygon>
</Placemark>
<Placemark>
<name>494</name>
<styleUrl>#stylehap2</styleUrl>
<Polygon>
<outerBoundaryIs>
<LinearRing>
<coordinates>
-0.471,48.252,0
-0.46935772180169,48.251999988308,0
-0.46936396811677,48.252095294959,0
-0.46938266569177,48.252189876445,0
-0.46941367229419,48.252283012938,0
-0.46945675200822,48.252373995602,0
-0.46951157702839,48.252462131991,0
-0.46957773015244,48.252546751317,0
-0.46965470795517,48.252627209558,0
-0.46974192461843,48.252702894357,0
-0.46983871638887,48.252773229684,0
-0.46994434662871,48.252837680221,0
-0.47005801142198,48.252895755439,0
-0.47017884569291,48.252947013327,0
-0.47030592979031,48.25299106376,0
-0.47043829648746,48.25302757147,0
-0.4705749383446,48.253056258594,0
-0.47071481537753,48.253076906792,0
-0.47085686297445,48.25308935891,0
-0.47100000000001,48.253093520174,0
-0.47114313702556,48.25308935891,0
-0.47128518462246,48.253076906792,0
-0.47142506165542,48.253056258594,0
-0.47156170351255,48.25302757147,0
-0.4716940702097,48.25299106376,0
-0.4718211543071,48.252947013327,0
-0.47194198857803,48.252895755439,0
-0.4720556533713,48.252837680221,0
-0.47216128361115,48.252773229684,0
-0.47225807538156,48.252702894357,0
-0.47234529204485,48.252627209558,0
-0.47242226984758,48.252546751317,0
-0.47248842297162,48.252462131991,0
-0.47254324799177,48.252373995602,0
-0.47258632770582,48.252283012938,0
-0.47261733430824,48.252189876445,0
-0.47263603188324,48.252095294959,0
-0.47264227819832,48.251999988308,0

-0.47263602578469,48.251904681833,0
-0.47261732229643,48.251810100875,0
-0.47258631014575,48.251716965244,0
-0.47254322541698,48.251625983749,0
-0.472488396068,48.251537848801,0
-0.47242223943262,48.251453231144,0
-0.4723452590427,48.25137277475,0
-0.47225804079496,48.25129709192,0
-0.47216124849098,48.251226758624,0
-0.47205561878468,48.251162310117,0
-0.47194195557586,48.251104236868,0
-0.47182112389214,48.251052980827,0
-0.47169404330608,48.251008932063,0
-0.47156168093774,48.250972425795,0
-0.47142504409534,48.25094373984,0
-0.47128517261068,48.250923092503,0
-0.47114313092701,48.250910640912,0
-0.47100000000001,48.250906479826,0
-0.470856869073,48.250910640912,0
-0.47071482738934,48.250923092503,0
-0.47057495590467,48.25094373984,0
-0.47043831906228,48.250972425795,0
-0.47030595669393,48.251008932063,0
-0.47017887610787,48.251052980827,0
-0.47005804442413,48.251104236868,0
-0.46994438121533,48.251162310117,0
-0.46983875150903,48.251226758624,0
-0.46974195920505,48.25129709192,0
-0.46965474095731,48.25137277475,0
-0.46957776056739,48.251453231144,0
-0.46951160393199,48.251537848801,0
-0.46945677458303,48.251625983749,0
-0.46941368985426,48.251716965244,0
-0.46938267770355,48.251810100875,0
-0.46936397421532,48.251904681833,0
-0.46935772180169,48.251999988308,0
-0.471,48.252,0
</coordinates>
</LinearRing>
</outerBoundaryIs>
</Polygon>
</Placemark>
<Placemark>
<name>496</name>
<styleUrl>#stylehap2</styleUrl>
<Polygon>
<outerBoundaryIs>
<LinearRing>
<coordinates>
-0.46900000000005,48.187,0
-0.46735980567695,48.186999988334,0
-0.46736604407306,48.187095294986,0
-0.46738471792957,48.187189876471,0
-0.46741568519425,48.187283012963,0
-0.46745871025048,48.187373995625,0
-0.46751346570848,48.187462132013,0
-0.4675795348953,48.187546751337,0

-0.46765641502436,48.187627209576,0
-0.46774352102086,48.187702894373,0
-0.46784018997349,48.187773229697,0
-0.46794568617927,48.187837680232,0
-0.46805920674227,48.187895755448,0
-0.46817988768451,48.187947013333,0
-0.46830681052181,48.187991063765,0
-0.46843900925486,48.188027571473,0
-0.46857547772238,48.188056258596,0
-0.46871517726003,48.188076906793,0
-0.46885704460701,48.18808935891,0
-0.46900000000006,48.188093520173,0
-0.46914295539309,48.18808935891,0
-0.46928482274007,48.188076906793,0
-0.46942452227772,48.188056258596,0
-0.46956099074526,48.188027571473,0
-0.46969318947832,48.187991063765,0
-0.46982011231559,48.187947013333,0
-0.46994079325783,48.187895755448,0
-0.47005431382083,48.187837680232,0
-0.47015981002661,48.187773229697,0
-0.47025647897924,48.187702894373,0
-0.47034358497574,48.187627209576,0
-0.4704204651048,48.187546751337,0
-0.47048653429162,48.187462132013,0
-0.47054128974962,48.187373995625,0
-0.47058431480585,48.187283012963,0
-0.47061528207053,48.187189876471,0
-0.47063395592704,48.187095294986,0
-0.47064019432315,48.186999988334,0
-0.47063394985012,48.18690468186,0
-0.47061527010131,48.186810100901,0
-0.47058429730804,48.186716965269,0
-0.47054126725488,48.186625983772,0
-0.47048650748344,48.186537848823,0
-0.47042043479771,48.186453231164,0
-0.47034355209061,48.186372774768,0
-0.47025644451531,48.186297091936,0
-0.47015977503099,48.186226758637,0
-0.4700542793569,48.186162310128,0
-0.46994076037273,48.186104236877,0
-0.46982008200851,48.186052980834,0
-0.46969316267011,48.186008932068,0
-0.4695609682505,48.185972425798,0
-0.46942450477991,48.185943739841,0
-0.46928481077088,48.185923092503,0
-0.46914294931617,48.185910640912,0
-0.46900000000006,48.185906479826,0
-0.46885705068393,48.185910640912,0
-0.46871518922922,48.185923092503,0
-0.46857549522019,48.185943739841,0
-0.4684390317496,48.185972425798,0
-0.46830683732999,48.186008932068,0
-0.46817991799159,48.186052980834,0
-0.46805923962737,48.186104236877,0
-0.4679457206432,48.186162310128,0
-0.46784022496911,48.186226758637,0

-0.46774355548479,48.186297091936,0
-0.46765644790949,48.186372774768,0
-0.46757956520239,48.186453231164,0
-0.46751349251666,48.186537848823,0
-0.46745873274522,48.186625983772,0
-0.46741570269206,48.186716965269,0
-0.46738472989878,48.186810100901,0
-0.46736605014998,48.18690468186,0
-0.46735980567695,48.186999988334,0
-0.46900000000005,48.187,0
</coordinates>
</LinearRing>
</outerBoundaryIs>
</Polygon>
</Placemark>
<Placemark>
<name>497</name>
<styleUrl>#stylehap2</styleUrl>
<Polygon>
<outerBoundaryIs>
<LinearRing>
<coordinates>
-0.46900000000005,48.251,0
-0.46735775391746,48.250999988308,0
-0.46736400011051,48.25109529496,0
-0.46738269731997,48.251189876446,0
-0.46741370331613,48.251283012938,0
-0.46745678218782,48.251373995602,0
-0.46751160613591,48.251462131991,0
-0.46757775796637,48.251546751318,0
-0.46765473426378,48.251627209559,0
-0.46774194922153,48.251702894357,0
-0.46783873909913,48.251773229684,0
-0.46794436727329,48.251837680221,0
-0.46805802984376,48.251895755439,0
-0.46817886175167,48.251947013327,0
-0.4683059433638,48.25199106376,0
-0.46843830747237,48.25202757147,0
-0.46857494665727,48.252056258594,0
-0.46871482095475,48.252076906792,0
-0.46885686577373,48.25208935891,0
-0.46900000000006,48.252093520174,0
-0.46914313422637,48.25208935891,0
-0.46928517904535,48.252076906792,0
-0.46942505334283,48.252056258594,0
-0.46956169252773,48.25202757147,0
-0.4696940566363,48.25199106376,0
-0.46982113824843,48.251947013327,0
-0.46994197015634,48.251895755439,0
-0.47005563272681,48.251837680221,0
-0.47016126090097,48.251773229684,0
-0.4702580507786,48.251702894357,0
-0.47034526573632,48.251627209559,0
-0.47042224203373,48.251546751318,0
-0.47048839386418,48.251462131991,0
-0.47054321781228,48.251373995602,0
-0.47058629668397,48.251283012938,0

-0.47061730268013,48.251189876446,0
-0.47063599988959,48.25109529496,0
-0.47064224608264,48.250999988308,0
-0.47063599379137,48.250904681834,0
-0.47061729066898,48.250810100875,0
-0.47058627912484,48.250716965244,0
-0.47054319523871,48.250625983749,0
-0.47048836696204,48.250537848801,0
-0.47042221162043,48.250453231144,0
-0.47034523273596,48.25037277475,0
-0.47025801619386,48.25029709192,0
-0.47016122578271,48.250226758624,0
-0.47005559814207,48.250162310117,0
-0.469941937156,48.250104236868,0
-0.46982110783516,48.250052980827,0
-0.46969402973417,48.250008932063,0
-0.46956166995417,48.249972425795,0
-0.4694250357837,48.24994373984,0
-0.4692851670342,48.249923092502,0
-0.46914312812815,48.249910640912,0
-0.46900000000006,48.249906479826,0
-0.46885687187195,48.249910640912,0
-0.4687148329659,48.249923092502,0
-0.4685749642164,48.24994373984,0
-0.46843833004593,48.249972425795,0
-0.46830597026593,48.250008932063,0
-0.46817889216497,48.250052980827,0
-0.4680580628441,48.250104236868,0
-0.46794440185803,48.250162310117,0
-0.46783877421739,48.250226758624,0
-0.46774198380624,48.25029709192,0
-0.46765476726414,48.25037277475,0
-0.46757778837967,48.250453231144,0
-0.46751163303806,48.250537848801,0
-0.46745680476139,48.250625983749,0
-0.46741372087526,48.250716965244,0
-0.46738270933112,48.250810100875,0
-0.46736400620873,48.250904681834,0
-0.46735775391746,48.250999988308,0
-0.46900000000005,48.251,0
</coordinates>
</LinearRing>
</outerBoundaryIs>
</Polygon>
</Placemark>
<Placemark>
<name>499</name>
<styleUrl>#stylehap2</styleUrl>
<Polygon>
<outerBoundaryIs>
<LinearRing>
<coordinates>
-0.46799999999996,48.242,0
-0.46635804288001,48.241999988312,0
-0.46636428797495,48.242095294963,0
-0.46638298189546,48.242189876449,0
-0.46641398243681,48.242283012942,0

-0.46645705372932,48.242373995605,0
-0.46651186803153,48.242462131994,0
-0.46657800822273,48.24254675132,0
-0.46665497097614,48.242627209561,0
-0.46674217058815,48.242702894359,0
-0.46683894343512,48.242773229686,0
-0.4669445530232,48.242837680223,0
-0.46705819559393,48.24289575544,0
-0.46717900624036,48.242947013328,0
-0.46730606549121,48.242991063761,0
-0.46743840630886,48.24302757147,0
-0.46757502145055,48.243056258594,0
-0.46771487113544,48.243076906792,0
-0.46785689095989,48.24308935891,0
-0.46799999999995,48.243093520174,0
-0.46814310904003,48.24308935891,0
-0.46828512886448,48.243076906792,0
-0.46842497854937,48.243056258594,0
-0.46856159369107,48.24302757147,0
-0.46869393450871,48.242991063761,0
-0.46882099375956,48.242947013328,0
-0.468941804406,48.24289575544,0
-0.46905544697661,48.242837680223,0
-0.4691610565648,48.242773229686,0
-0.46925782941178,48.242702894359,0
-0.46934502902378,48.242627209561,0
-0.4694219917772,48.24254675132,0
-0.4694881319684,48.242462131994,0
-0.46954294627061,48.242373995605,0
-0.46958601756311,48.242283012942,0
-0.46961701810446,48.242189876449,0
-0.46963571202498,48.242095294963,0
-0.46964195711992,48.241999988312,0
-0.46963570592976,48.241904681838,0
-0.46961700609922,48.241810100879,0
-0.46958600001261,48.241716965248,0
-0.46954292370813,48.241625983753,0
-0.4694881050795,48.241537848804,0
-0.46942196137886,48.241453231147,0
-0.46934499603968,48.241372774753,0
-0.46925779484406,48.241297091923,0
-0.46916102146385,48.241226758626,0
-0.46905541240891,48.241162310118,0
-0.46894177142187,48.241104236869,0
-0.46882096336122,48.241052980828,0
-0.46869390761982,48.241008932064,0
-0.4685615711286,48.240972425795,0
-0.4684249609989,48.24094373984,0
-0.46828511685924,48.240923092503,0
-0.46814310294482,48.240910640912,0
-0.46799999999995,48.240906479826,0
-0.46785689705511,48.240910640912,0
-0.46771488314069,48.240923092503,0
-0.46757503900103,48.24094373984,0
-0.46743842887133,48.240972425795,0
-0.46730609238011,48.241008932064,0
-0.4671790366387,48.241052980828,0

-0.46705822857806,48.241104236869,0
-0.46694458759101,48.241162310118,0
-0.46683897853608,48.241226758626,0
-0.46674220515584,48.241297091923,0
-0.46665500396025,48.241372774753,0
-0.46657803862107,48.241453231147,0
-0.46651189492042,48.241537848804,0
-0.46645707629179,48.241625983753,0
-0.46641399998729,48.241716965248,0
-0.46638299390071,48.241810100879,0
-0.46636429407016,48.241904681838,0
-0.46635804288001,48.241999988312,0
-0.46799999999996,48.242,0
</coordinates>
</LinearRing>
</outerBoundaryIs>
</Polygon>
</Placemark>
<Placemark>
<name>501</name>
<styleUrl>#stylehap2</styleUrl>
<Polygon>
<outerBoundaryIs>
<LinearRing>
<coordinates>
-0.46500000000003,48.246,0
-0.46335791446981,48.24599998831,0
-0.46336416005273,48.246095294962,0
-0.46338285543477,48.246189876448,0
-0.46341385840016,48.24628301294,0
-0.46345693306075,48.246373995604,0
-0.46351175164941,48.246462131993,0
-0.46357789701292,48.246546751319,0
-0.46365486578507,48.24662720956,0
-0.46374207221646,48.246702894358,0
-0.46383885263158,48.246773229685,0
-0.46394447047907,48.246837680222,0
-0.46405812193729,48.24689575544,0
-0.46417894203198,48.246947013327,0
-0.4643060112198,48.246991063761,0
-0.46443836238755,48.24702757147,0
-0.46457498821367,48.247056258594,0
-0.46471484883601,48.247076906792,0
-0.46485687976762,48.24708935891,0
-0.46500000000002,48.247093520174,0
-0.46514312023245,48.24708935891,0
-0.46528515116406,48.247076906792,0
-0.4654250117864,48.247056258594,0
-0.46556163761249,48.24702757147,0
-0.46569398878027,48.246991063761,0
-0.46582105796809,48.246947013327,0
-0.46594187806278,48.24689575544,0
-0.466055529521,48.246837680222,0
-0.46616114736849,48.246773229685,0
-0.46625792778361,48.246702894358,0
-0.466345134215,48.24662720956,0
-0.46642210298715,48.246546751319,0

-0.46648824835064,48.246462131993,0
-0.46654306693932,48.246373995604,0
-0.46658614159991,48.24628301294,0
-0.46661714456527,48.246189876448,0
-0.46663583994734,48.246095294962,0
-0.46664208553026,48.24599998831,0
-0.4666358338508,48.245904681836,0
-0.46661713255741,48.245810100877,0
-0.46658612404558,48.245716965246,0
-0.46654304437191,48.245625983751,0
-0.46648822145587,48.245537848803,0
-0.46642207258216,48.245453231146,0
-0.46634510122367,48.245372774752,0
-0.46625789320833,48.245297091922,0
-0.46616111225985,48.245226758625,0
-0.46605549494573,48.245162310118,0
-0.46594184507145,48.245104236869,0
-0.46582102756311,48.245052980828,0
-0.46569396188547,48.245008932064,0
-0.46556161504511,48.244972425795,0
-0.46542499423207,48.24494373984,0
-0.46528513915619,48.244923092503,0
-0.46514311413591,48.244910640912,0
-0.46500000000002,48.244906479826,0
-0.46485688586416,48.244910640912,0
-0.46471486084385,48.244923092503,0
-0.46457500576799,48.24494373984,0
-0.46443838495496,48.244972425795,0
-0.4643060381146,48.245008932064,0
-0.46417897243696,48.245052980828,0
-0.46405815492862,48.245104236869,0
-0.46394450505434,48.245162310118,0
-0.46383888774022,48.245226758625,0
-0.46374210679174,48.245297091922,0
-0.4636548987764,48.245372774752,0
-0.46357792741791,48.245453231146,0
-0.4635117785442,48.245537848803,0
-0.46345695562813,48.245625983751,0
-0.46341387595448,48.245716965246,0
-0.46338286744264,48.245810100877,0
-0.46336416614927,48.245904681836,0
-0.46335791446981,48.24599998831,0
-0.46500000000003,48.246,0
</coordinates>
</LinearRing>
</outerBoundaryIs>
</Polygon>
</Placemark>
<Placemark>
<name>502</name>
<styleUrl>#stylehap2</styleUrl>
<Polygon>
<outerBoundaryIs>
<LinearRing>
<coordinates>
-0.46399999999994,48.204,0
-0.46235926137807,48.203999988327,0

-0.46236550184258,48.204095294979,0
-0.46238418189425,48.204189876464,0
-0.46241515943378,48.204283012956,0
-0.46245819876637,48.204373995619,0
-0.46251297239369,48.204462132007,0
-0.46257906350455,48.204546751332,0
-0.46265596914553,48.204627209572,0
-0.46274310404768,48.204702894368,0
-0.46283980507976,48.204773229694,0
-0.46294533629461,48.204837680229,0
-0.46305889452991,48.204895755445,0
-0.46317961552094,48.204947013332,0
-0.46330658047862,48.204991063764,0
-0.46343882308313,48.205027571472,0
-0.46357533683925,48.205056258595,0
-0.46371508273786,48.205076906793,0
-0.46385699716532,48.20508935891,0
-0.46399999999994,48.205093520173,0
-0.46414300283458,48.20508935891,0
-0.46428491726201,48.205076906793,0
-0.46442466316065,48.205056258595,0
-0.46456117691674,48.205027571472,0
-0.46469341952125,48.204991063764,0
-0.46482038447896,48.204947013332,0
-0.46494110546996,48.204895755445,0
-0.46505466370526,48.204837680229,0
-0.46516019492011,48.204773229694,0
-0.46525689595222,48.204702894368,0
-0.46534403085437,48.204627209572,0
-0.46542093649535,48.204546751332,0
-0.46548702760619,48.204462132007,0
-0.46554180123353,48.204373995619,0
-0.46558484056612,48.204283012956,0
-0.46561581810564,48.204189876464,0
-0.46563449815731,48.204095294979,0
-0.46564073862182,48.203999988327,0
-0.46563449207474,48.203904681853,0
-0.46561580612531,48.203810100894,0
-0.46558482305205,48.203716965262,0
-0.46554177871787,48.203625983766,0
-0.46548700077309,48.203537848817,0
-0.46542090616008,48.203453231159,0
-0.46534399793868,48.203372774763,0
-0.46525686145623,48.203297091932,0
-0.465160159892,48.203226758634,0
-0.46505462920929,48.203162310125,0
-0.4649410725543,48.203104236874,0
-0.46482035414372,48.203052980832,0
-0.46469339268815,48.203008932067,0
-0.46456115440109,48.202972425797,0
-0.46442464564658,48.202943739841,0
-0.4642849052817,48.202923092503,0
-0.46414299675201,48.202910640912,0
-0.46399999999994,48.202906479826,0
-0.46385700324789,48.202910640912,0
-0.4637150947182,48.202923092503,0
-0.46357535435332,48.202943739841,0

-0.46343884559878,48.202972425797,0
-0.46330660731172,48.203008932067,0
-0.46317964585618,48.203052980832,0
-0.4630589274456,48.203104236874,0
-0.4629453707906,48.203162310125,0
-0.4628398401079,48.203226758634,0
-0.46274313854364,48.203297091932,0
-0.46265600206119,48.203372774763,0
-0.46257909383979,48.203453231159,0
-0.4625129992268,48.203537848817,0
-0.46245822128202,48.203625983766,0
-0.46241517694782,48.203716965262,0
-0.46238419387459,48.203810100894,0
-0.46236550792515,48.203904681853,0
-0.46235926137807,48.203999988327,0
-0.46399999999994,48.204,0
</coordinates>
</LinearRing>
</outerBoundaryIs>
</Polygon>
</Placemark>
<Placemark>
<name>503</name>
<styleUrl>#stylehap2</styleUrl>
<Polygon>
<outerBoundaryIs>
<LinearRing>
<coordinates>
-0.46299999999997,48.223,0
-0.46135865244545,48.222999988319,0
-0.46136489522398,48.223095294971,0
-0.46138358220646,48.223189876457,0
-0.46141457124092,48.223283012949,0
-0.46145762654518,48.223373995612,0
-0.46151242049937,48.223462132,0
-0.46157853613766,48.223546751326,0
-0.46165547032002,48.223627209566,0
-0.46174263756032,48.223702894364,0
-0.4618393744812,48.22377322969,0
-0.46194494486237,48.223837680226,0
-0.46205854524342,48.223895755443,0
-0.46217931103887,48.22394701333,0
-0.46230632311866,48.223991063762,0
-0.46243861480424,48.224027571471,0
-0.4625751792268,48.224056258595,0
-0.46271497699161,48.224076906793,0
-0.4628569440902,48.22408935891,0
-0.46299999999998,48.224093520174,0
-0.46314305590973,48.22408935891,0
-0.46328502300832,48.224076906793,0
-0.46342482077313,48.224056258595,0
-0.46356138519569,48.224027571471,0
-0.46369367688129,48.223991063762,0
-0.46382068896106,48.22394701333,0
-0.4639414547565,48.223895755443,0
-0.46405505513756,48.223837680226,0
-0.46416062551873,48.22377322969,0

-0.46425736243964,48.223702894364,0
-0.46434452967991,48.223627209566,0
-0.46442146386227,48.223546751326,0
-0.46448757950055,48.223462132,0
-0.46454237345474,48.223373995612,0
-0.46458542875901,48.223283012949,0
-0.46461641779347,48.223189876457,0
-0.46463510477597,48.223095294971,0
-0.46464134755448,48.222999988319,0
-0.46463509868707,48.222904681845,0
-0.46461640580069,48.222810100886,0
-0.46458541122675,48.222716965255,0
-0.4645423509157,48.222625983759,0
-0.46448755263957,48.222537848811,0
-0.4644214334955,48.222453231153,0
-0.46434449673002,48.222372774758,0
-0.46425732790782,48.222297091927,0
-0.46416059045421,48.22222675863,0
-0.46405502060575,48.222162310122,0
-0.46394142180662,48.222104236872,0
-0.46382065859429,48.22205298083,0
-0.46369365002028,48.222008932065,0
-0.46356136265665,48.221972425796,0
-0.46342480324087,48.22194373984,0
-0.46328501101554,48.221923092503,0
-0.46314304982085,48.221910640912,0
-0.46299999999998,48.221906479827,0
-0.46285695017908,48.221910640912,0
-0.46271498898439,48.221923092503,0
-0.46257519675906,48.22194373984,0
-0.46243863734328,48.221972425796,0
-0.46230634997964,48.222008932065,0
-0.46217934140564,48.22205298083,0
-0.46205857819331,48.222104236872,0
-0.46194497939418,48.222162310122,0
-0.46183940954572,48.22222675863,0
-0.46174267209213,48.222297091927,0
-0.46165550326991,48.222372774758,0
-0.46157856650442,48.222453231153,0
-0.46151244736035,48.222537848811,0
-0.46145764908422,48.222625983759,0
-0.46141458877318,48.222716965255,0
-0.46138359419924,48.222810100886,0
-0.46136490131286,48.222904681845,0
-0.46135865244545,48.222999988319,0
-0.46299999999997,48.223,0
</coordinates>
</LinearRing>
</outerBoundaryIs>
</Polygon>
</Placemark>
<Placemark>
<name>505</name>
<styleUrl>#stylehap2</styleUrl>
<Polygon>
<outerBoundaryIs>
<LinearRing>

<coordinates>
-0.46199999999999,48.223,0
-0.46035865244546,48.222999988319,0
-0.46036489522399,48.223095294971,0
-0.46038358220647,48.223189876457,0
-0.46041457124096,48.223283012949,0
-0.4604576265452,48.223373995612,0
-0.46051242049942,48.223462132,0
-0.4605785361377,48.223546751326,0
-0.46065547032006,48.223627209566,0
-0.46074263756033,48.223702894364,0
-0.46083937448121,48.22377322969,0
-0.46094494486238,48.223837680226,0
-0.46105854524346,48.223895755443,0
-0.46117931103889,48.22394701333,0
-0.46130632311868,48.223991063762,0
-0.46143861480426,48.224027571471,0
-0.46157517922682,48.224056258595,0
-0.46171497699165,48.224076906793,0
-0.46185694409021,48.22408935891,0
-0.46199999999999,48.224093520174,0
-0.46214305590977,48.22408935891,0
-0.46228502300834,48.224076906793,0
-0.46242482077314,48.224056258595,0
-0.46256138519573,48.224027571471,0
-0.46269367688131,48.223991063762,0
-0.4628206889611,48.22394701333,0
-0.46294145475652,48.223895755443,0
-0.46305505513758,48.223837680226,0
-0.46316062551877,48.22377322969,0
-0.46325736243965,48.223702894364,0
-0.46334452967992,48.223627209566,0
-0.46342146386228,48.223546751326,0
-0.46348757950057,48.223462132,0
-0.46354237345478,48.223373995612,0
-0.46358542875902,48.223283012949,0
-0.46361641779348,48.223189876457,0
-0.46363510477599,48.223095294971,0
-0.46364134755452,48.222999988319,0
-0.46363509868708,48.222904681845,0
-0.46361640580073,48.222810100886,0
-0.46358541122676,48.222716965255,0
-0.46354235091572,48.222625983759,0
-0.46348755263959,48.222537848811,0
-0.46342143349552,48.222453231153,0
-0.46334449673006,48.222372774758,0
-0.46325732790783,48.222297091927,0
-0.46316059045425,48.22222675863,0
-0.46305502060576,48.222162310122,0
-0.46294142180666,48.222104236872,0
-0.46282065859431,48.22205298083,0
-0.46269365002033,48.222008932065,0
-0.46256136265669,48.221972425796,0
-0.46242480324088,48.22194373984,0
-0.46228501101556,48.221923092503,0
-0.46214304982087,48.221910640912,0
-0.46199999999999,48.221906479827,0

-0.46185695017912,48.221910640912,0
-0.4617149889844,48.221923092503,0
-0.46157519675908,48.22194373984,0
-0.4614386373433,48.221972425796,0
-0.46130634997966,48.222008932065,0
-0.46117934140565,48.22205298083,0
-0.46105857819332,48.222104236872,0
-0.4609449793942,48.222162310122,0
-0.46083940954573,48.22222675863,0
-0.46074267209215,48.222297091927,0
-0.46065550326992,48.222372774758,0
-0.46057856650446,48.222453231153,0
-0.4605124473604,48.222537848811,0
-0.46045764908424,48.222625983759,0
-0.46041458877322,48.222716965255,0
-0.46038359419925,48.222810100886,0
-0.46036490131287,48.222904681845,0
-0.46035865244546,48.222999988319,0
-0.46199999999999,48.223,0
</coordinates>
</LinearRing>
</outerBoundaryIs>
</Polygon>
</Placemark>
<Placemark>
<name>506</name>
<styleUrl>#stylehap2</styleUrl>
<Polygon>
<outerBoundaryIs>
<LinearRing>
<coordinates>
-0.46199999999999,47.782,0
-0.46037262471241,47.781999988499,0
-0.46037881439453,47.782095295149,0
-0.46039734234566,47.782189876631,0
-0.46042806762257,47.782283013116,0
-0.4604707564489,47.782373995771,0
-0.46052508399261,47.782462132148,0
-0.4605906368363,47.782546751461,0
-0.46066691612225,47.782627209687,0
-0.46075334134771,47.782702894469,0
-0.46084925478217,47.782773229779,0
-0.4609539264724,47.7828376803,0
-0.46106655979769,47.782895755502,0
-0.46118629753285,47.782947013374,0
-0.46131222837273,47.782991063794,0
-0.46144339386857,47.783027571492,0
-0.46157879572374,47.783056258607,0
-0.46171740339269,47.783076906798,0
-0.46185816192579,47.783089358912,0
-0.46199999999999,47.783093520174,0
-0.46214183807416,47.783089358912,0
-0.46228259660726,47.783076906798,0
-0.46242120427624,47.783056258607,0
-0.46255660613141,47.783027571492,0
-0.46268777162726,47.782991063794,0
-0.46281370246713,47.782947013374,0

-0.4629334402023,47.782895755502,0
-0.46304607352759,47.7828376803,0
-0.46315074521779,47.782773229779,0
-0.46324665865227,47.782702894469,0
-0.46333308387774,47.782627209687,0
-0.46340936316369,47.782546751461,0
-0.46347491600737,47.782462132148,0
-0.46352924355106,47.782373995771,0
-0.46357193237741,47.782283013116,0
-0.46360265765432,47.782189876631,0
-0.46362118560546,47.782095295149,0
-0.46362737528758,47.781999988499,0
-0.46362117966113,47.781904682023,0
-0.46360264594627,47.78181010106,0
-0.4635719152614,47.781716965422,0
-0.46352922154714,47.781625983918,0
-0.46347488978411,47.781537848958,0
-0.46340933351787,47.781453231287,0
-0.46333305171014,47.781372774879,0
-0.46324662494028,47.781297092032,0
-0.46315071098576,47.781226758719,0
-0.46304603981561,47.781162310196,0
-0.4629334080347,47.781104236931,0
-0.46281367282131,47.781052980875,0
-0.46268774540399,47.781008932097,0
-0.46255658412747,47.780972425817,0
-0.46242118716023,47.780943739852,0
-0.46228258489923,47.780923092508,0
-0.46214183212983,47.780910640914,0
-0.46199999999999,47.780906479826,0
-0.46185816787012,47.780910640914,0
-0.46171741510075,47.780923092508,0
-0.46157881283976,47.780943739852,0
-0.46144341587249,47.780972425817,0
-0.46131225459599,47.781008932097,0
-0.46118632717868,47.781052980875,0
-0.46106659196528,47.781104236931,0
-0.46095396018437,47.781162310196,0
-0.46084928901423,47.781226758719,0
-0.46075337505968,47.781297092032,0
-0.46066694828982,47.781372774879,0
-0.46059066648212,47.781453231287,0
-0.46052511021587,47.781537848958,0
-0.46047077845284,47.781625983918,0
-0.46042808473859,47.781716965422,0
-0.46039735405371,47.78181010106,0
-0.46037882033886,47.781904682023,0
-0.46037262471241,47.781999988499,0
-0.46199999999999,47.782,0
</coordinates>
</LinearRing>
</outerBoundaryIs>
</Polygon>
</Placemark>
<Placemark>
<name>507</name>
<styleUrl>#stylehap2</styleUrl>

<Polygon>
<outerBoundaryIs>
<LinearRing>
<coordinates>
-0.46000000000004,48.223,0
-0.45835865244552,48.222999988319,0
-0.45836489522405,48.223095294971,0
-0.45838358220653,48.223189876457,0
-0.45841457124099,48.223283012949,0
-0.45845762654526,48.223373995612,0
-0.45851242049945,48.223462132,0
-0.45857853613773,48.223546751326,0
-0.45865547032009,48.223627209566,0
-0.45874263756039,48.223702894364,0
-0.45883937448127,48.22377322969,0
-0.45894494486244,48.223837680226,0
-0.45905854524349,48.223895755443,0
-0.45917931103894,48.22394701333,0
-0.45930632311873,48.223991063762,0
-0.45943861480431,48.224027571471,0
-0.45957517922687,48.224056258595,0
-0.45971497699168,48.224076906793,0
-0.45985694409027,48.22408935891,0
-0.46000000000005,48.224093520174,0
-0.4601430559098,48.22408935891,0
-0.46028502300839,48.224076906793,0
-0.4604248207732,48.224056258595,0
-0.46056138519576,48.224027571471,0
-0.46069367688136,48.223991063762,0
-0.46082068896113,48.22394701333,0
-0.46094145475658,48.223895755443,0
-0.46105505513763,48.223837680226,0
-0.4611606255188,48.22377322969,0
-0.46125736243968,48.223702894364,0
-0.46134452967998,48.223627209566,0
-0.46142146386234,48.223546751326,0
-0.46148757950062,48.223462132,0
-0.46154237345481,48.223373995612,0
-0.46158542875908,48.223283012949,0
-0.46161641779354,48.223189876457,0
-0.46163510477604,48.223095294971,0
-0.46164134755455,48.222999988319,0
-0.46163509868714,48.222904681845,0
-0.46161640580076,48.222810100886,0
-0.46158541122682,48.222716965255,0
-0.46154235091578,48.222625983759,0
-0.46148755263964,48.222537848811,0
-0.46142143349558,48.222453231153,0
-0.46134449673009,48.222372774758,0
-0.46125732790789,48.222297091927,0
-0.46116059045428,48.22222675863,0
-0.46105502060582,48.222162310122,0
-0.46094142180669,48.222104236872,0
-0.46082065859436,48.22205298083,0
-0.46069365002036,48.222008932065,0
-0.46056136265672,48.221972425796,0
-0.46042480324094,48.22194373984,0

-0.46028501101561,48.221923092503,0
-0.46014304982092,48.221910640912,0
-0.46000000000005,48.221906479827,0
-0.45985695017915,48.221910640912,0
-0.45971498898446,48.221923092503,0
-0.45957519675913,48.22194373984,0
-0.45943863734335,48.221972425796,0
-0.45930634997971,48.222008932065,0
-0.45917934140571,48.22205298083,0
-0.45905857819338,48.222104236872,0
-0.45894497939425,48.222162310122,0
-0.45883940954579,48.22222675863,0
-0.45874267209221,48.222297091927,0
-0.45865550326998,48.222372774758,0
-0.45857856650449,48.222453231153,0
-0.45851244736043,48.222537848811,0
-0.45845764908429,48.222625983759,0
-0.45841458877325,48.222716965255,0
-0.45838359419931,48.222810100886,0
-0.45836490131293,48.222904681845,0
-0.45835865244552,48.222999988319,0
-0.46000000000004,48.223,0
</coordinates>
</LinearRing>
</outerBoundaryIs>
</Polygon>
</Placemark>
<Placemark>
<name>508</name>
<styleUrl>#stylehap2</styleUrl>
<Polygon>
<outerBoundaryIs>
<LinearRing>
<coordinates>
-0.46000000000004,48.229,0
-0.45835846001957,48.228999988317,0
-0.45836470352932,48.229095294969,0
-0.45838339270201,48.229189876455,0
-0.45841438536894,48.229283012947,0
-0.45845744572031,48.22937399561,0
-0.4585122460979,48.229462131998,0
-0.45857836948699,48.229546751324,0
-0.4586553126886,48.229627209565,0
-0.45874249014789,48.229702894362,0
-0.45883923840981,48.229773229688,0
-0.45894482116776,48.229837680225,0
-0.4590584348671,48.229895755442,0
-0.45917921482098,48.229947013329,0
-0.45930624179159,48.229991063762,0
-0.45943854898706,48.230027571471,0
-0.45957512942051,48.230056258595,0
-0.4597149435753,48.230076906793,0
-0.45985692731824,48.23008935891,0
-0.46000000000005,48.230093520174,0
-0.46014307268183,48.23008935891,0
-0.46028505642477,48.230076906793,0
-0.46042487057956,48.230056258595,0

-0.46056145101301,48.230027571471,0
-0.46069375820848,48.229991063762,0
-0.46082078517909,48.229947013329,0
-0.46094156513297,48.229895755442,0
-0.46105517883231,48.229837680225,0
-0.46116076159026,48.229773229688,0
-0.46125750985218,48.229702894362,0
-0.46134468731147,48.229627209565,0
-0.46142163051308,48.229546751324,0
-0.46148775390217,48.229462131998,0
-0.46154255427976,48.22937399561,0
-0.46158561463115,48.229283012947,0
-0.46161660729806,48.229189876455,0
-0.46163529647075,48.229095294969,0
-0.4616415399805,48.228999988317,0
-0.46163529037986,48.228904681843,0
-0.46161659530137,48.228810100884,0
-0.46158559709312,48.228716965253,0
-0.46154253173331,48.228625983757,0
-0.46148772703236,48.228537848809,0
-0.46142160013634,48.228453231151,0
-0.4613446543508,48.228372774756,0
-0.46125747530904,48.228297091926,0
-0.46116072651424,48.228226758629,0
-0.46105514428917,48.228162310121,0
-0.46094153217227,48.228104236871,0
-0.46082075480235,48.228052980829,0
-0.46069373133867,48.228008932065,0
-0.46056142846657,48.227972425796,0
-0.46042485304155,48.22794373984,0
-0.46028504442805,48.227923092503,0
-0.46014306659095,48.227910640912,0
-0.46000000000005,48.227906479826,0
-0.45985693340912,48.227910640912,0
-0.45971495557202,48.227923092503,0
-0.45957514695852,48.22794373984,0
-0.4594385715335,48.227972425796,0
-0.4593062686614,48.228008932065,0
-0.45917924519772,48.228052980829,0
-0.4590584678278,48.228104236871,0
-0.4589448557109,48.228162310121,0
-0.45883927348586,48.228226758629,0
-0.45874252469103,48.228297091926,0
-0.45865534564927,48.228372774756,0
-0.45857839986373,48.228453231151,0
-0.45851227296771,48.228537848809,0
-0.45845746826676,48.228625983757,0
-0.45841440290695,48.228716965253,0
-0.4583834046987,48.228810100884,0
-0.45836470962021,48.228904681843,0
-0.45835846001957,48.228999988317,0
-0.46000000000004,48.229,0
</coordinates>
</LinearRing>
</outerBoundaryIs>
</Polygon>
</Placemark>

<Placemark>
<name>509</name>
<styleUrl>#stylehap2</styleUrl>
<Polygon>
<outerBoundaryIs>
<LinearRing>
<coordinates>
-0.45299999999997,48.218,0
-0.4513588127522,48.217999988321,0
-0.45136505492154,48.218095294973,0
-0.45138374007945,48.218189876459,0
-0.45141472608775,48.218283012951,0
-0.45145777718735,48.218373995614,0
-0.45151256579032,48.218462132002,0
-0.45157867497154,48.218546751328,0
-0.45165560164014,48.218627209568,0
-0.45174276036713,48.218702894365,0
-0.45183948783998,48.218773229691,0
-0.45194504791028,48.218837680227,0
-0.45205863719611,48.218895755443,0
-0.45217939119641,48.21894701333,0
-0.45230639087089,48.218991063763,0
-0.45243866963545,48.219027571472,0
-0.45257522071966,48.219056258595,0
-0.45271500483024,48.219076906793,0
-0.45285695806268,48.21908935891,0
-0.45299999999998,48.219093520174,0
-0.45314304193725,48.21908935891,0
-0.45328499516971,48.219076906793,0
-0.4534247792803,48.219056258595,0
-0.45356133036448,48.219027571472,0
-0.45369360912906,48.218991063763,0
-0.45382060880355,48.21894701333,0
-0.45394136280385,48.218895755443,0
-0.45405495208965,48.218837680227,0
-0.45416051215997,48.218773229691,0
-0.45425723963282,48.218702894365,0
-0.45434439835982,48.218627209568,0
-0.45442132502841,48.218546751328,0
-0.45448743420963,48.218462132002,0
-0.45454222281261,48.218373995614,0
-0.45458527391218,48.218283012951,0
-0.45461625992051,48.218189876459,0
-0.45463494507842,48.218095294973,0
-0.45464118724776,48.217999988321,0
-0.45463493899119,48.217904681847,0
-0.45461624793101,48.217810100888,0
-0.45458525638473,48.217716965257,0
-0.45454220027972,48.217625983761,0
-0.45448740735598,48.217537848813,0
-0.45442129466994,48.217453231154,0
-0.45434436541894,48.21737277476,0
-0.45425720511045,48.217297091928,0
-0.45416047710502,48.217226758631,0
-0.45405491756728,48.217162310122,0
-0.45394132986297,48.217104236872,0
-0.45382057844508,48.217052980831,0

-0.45369358227541,48.217008932066,0
-0.45356130783159,48.216972425796,0
-0.45342476175282,48.216943739841,0
-0.45328498318022,48.216923092503,0
-0.45314303585002,48.216910640912,0
-0.45299999999998,48.216906479826,0
-0.4528569641499,48.216910640912,0
-0.45271501681974,48.216923092503,0
-0.45257523824711,48.216943739841,0
-0.45243869216833,48.216972425796,0
-0.45230641772454,48.217008932066,0
-0.45217942155488,48.217052980831,0
-0.45205867013699,48.217104236872,0
-0.45194508243268,48.217162310122,0
-0.45183952289491,48.217226758631,0
-0.45174279488951,48.217297091928,0
-0.45165563458102,48.2173727476,0
-0.45157870533001,48.217453231154,0
-0.45151259264395,48.217537848813,0
-0.45145779972023,48.217625983761,0
-0.45141474361523,48.217716965257,0
-0.45138375206894,48.217810100888,0
-0.45136506100876,48.217904681847,0
-0.4513588127522,48.217999988321,0
-0.45299999999997,48.218,0
</coordinates>
</LinearRing>
</outerBoundaryIs>
</Polygon>
</Placemark>
<Placemark>
<name>511</name>
<styleUrl>#stylehap2</styleUrl>
<Polygon>
<outerBoundaryIs>
<LinearRing>
<coordinates>
-0.452,48.218,0
-0.45035881275221,48.217999988321,0
-0.45036505492155,48.218095294973,0
-0.45038374007946,48.218189876459,0
-0.45041472608779,48.218283012951,0
-0.45045777718736,48.218373995614,0
-0.45051256579033,48.218462132002,0
-0.45057867497156,48.218546751328,0
-0.45065560164015,48.218627209568,0
-0.45074276036715,48.218702894365,0
-0.45083948784,48.218773229691,0
-0.45094504791032,48.218837680227,0
-0.45105863719615,48.218895755443,0
-0.45117939119642,48.21894701333,0
-0.45130639087091,48.218991063763,0
-0.45143866963549,48.219027571472,0
-0.45157522071967,48.219056258595,0
-0.45171500483026,48.219076906793,0
-0.45185695806272,48.21908935891,0
-0.45199999999999,48.219093520174,0

-0.45214304193729,48.21908935891,0
-0.45228499516973,48.219076906793,0
-0.45242477928034,48.219056258595,0
-0.45256133036452,48.219027571472,0
-0.45269360912908,48.218991063763,0
-0.45282060880356,48.21894701333,0
-0.45294136280386,48.218895755443,0
-0.45305495208969,48.218837680227,0
-0.45316051215999,48.218773229691,0
-0.45325723963284,48.218702894365,0
-0.45334439835983,48.218627209568,0
-0.45342132502843,48.218546751328,0
-0.45348743420965,48.218462132002,0
-0.45354222281262,48.218373995614,0
-0.45358527391222,48.218283012951,0
-0.45361625992055,48.218189876459,0
-0.45363494507843,48.218095294973,0
-0.45364118724777,48.217999988321,0
-0.4536349389912,48.217904681847,0
-0.45361624793105,48.217810100888,0
-0.45358525638474,48.217716965257,0
-0.45354220027974,48.217625983761,0
-0.45348740735602,48.217537848813,0
-0.45342129466996,48.217453231154,0
-0.45334436541895,48.21737277476,0
-0.45325720511046,48.217297091928,0
-0.45316047710506,48.217226758631,0
-0.45305491756732,48.217162310122,0
-0.45294132986298,48.217104236872,0
-0.45282057844509,48.217052980831,0
-0.45269358227543,48.217008932066,0
-0.45256130783164,48.216972425796,0
-0.45242476175286,48.216943739841,0
-0.45228498318023,48.216923092503,0
-0.45214303585007,48.216910640912,0
-0.45199999999999,48.216906479826,0
-0.45185696414994,48.216910640912,0
-0.45171501681975,48.216923092503,0
-0.45157523824715,48.216943739841,0
-0.45143869216837,48.216972425796,0
-0.45130641772456,48.217008932066,0
-0.45117942155489,48.217052980831,0
-0.451058670137,48.217104236872,0
-0.45094508243269,48.217162310122,0
-0.45083952289495,48.217226758631,0
-0.45074279488952,48.217297091928,0
-0.45065563458103,48.21737277476,0
-0.45057870533003,48.217453231154,0
-0.45051259264399,48.217537848813,0
-0.45045779972024,48.217625983761,0
-0.45041474361524,48.217716965257,0
-0.45038375206896,48.217810100888,0
-0.4503650610088,48.217904681847,0
-0.45035881275221,48.217999988321,0
-0.452,48.218,0
</coordinates>
</LinearRing>

</outerBoundaryIs>
</Polygon>
</Placemark>
<Placemark>
<name>512</name>
<styleUrl>#stylehap2</styleUrl>
<Polygon>
<outerBoundaryIs>
<LinearRing>
<coordinates>
-0.45100000000002,48.255,0
-0.44935762544394,48.254999988306,0
-0.44936387212519,48.255095294958,0
-0.44938257079692,48.255189876444,0
-0.44941357921831,48.255283012937,0
-0.44945666145974,48.255373995601,0
-0.44951148969641,48.25546213199,0
-0.4495776467017,48.255546751316,0
-0.44965462902082,48.255627209557,0
-0.44974185080129,48.255702894356,0
-0.44983864825078,48.255773229683,0
-0.44994428468832,48.255837680221,0
-0.45005795615076,48.255895755438,0
-0.45017879751156,48.255947013326,0
-0.45030588906557,48.25599106376,0
-0.45043826352934,48.25602757147,0
-0.45057491340393,48.256056258594,0
-0.45071479864422,48.256076906792,0
-0.45085685457583,48.25608935891,0
-0.45100000000003,48.256093520174,0
-0.45114314542421,48.25608935891,0
-0.45128520135582,48.256076906792,0
-0.45142508659611,48.256056258594,0
-0.45156173647071,48.25602757147,0
-0.45169411093447,48.25599106376,0
-0.45182120248848,48.255947013326,0
-0.45194204384931,48.255895755438,0
-0.45205571531172,48.255837680221,0
-0.45216135174926,48.255773229683,0
-0.45225814919875,48.255702894356,0
-0.45234537097922,48.255627209557,0
-0.45242235329834,48.255546751316,0
-0.45248851030363,48.25546213199,0
-0.4525433385403,48.255373995601,0
-0.45258642078173,48.255283012937,0
-0.45261742920312,48.255189876444,0
-0.45263612787485,48.255095294958,0
-0.4526423745561,48.254999988306,0
-0.45263612177528,48.254904681832,0
-0.45261741718933,48.254810100874,0
-0.45258640321876,48.254716965243,0
-0.4525433159618,48.254625983748,0
-0.4524884833956,48.2545378488,0
-0.45242232287839,48.254453231143,0
-0.45234533797165,48.254372774749,0
-0.45225811460645,48.254297091919,0
-0.45216131662332,48.254226758623,0

-0.45205568071944,48.254162310116,0
-0.45194201084171,48.254104236867,0
-0.45182117206854,48.254052980827,0
-0.45169408402644,48.254008932063,0
-0.4515617138922,48.253972425794,0
-0.45142506903313,48.25394373984,0
-0.45128518934205,48.253923092502,0
-0.45114313932464,48.253910640912,0
-0.45100000000003,48.253906479826,0
-0.4508568606754,48.253910640912,0
-0.45071481065799,48.253923092502,0
-0.45057493096691,48.25394373984,0
-0.45043828610786,48.253972425794,0
-0.4503059159736,48.254008932063,0
-0.4501788279315,48.254052980827,0
-0.45005798915833,48.254104236867,0
-0.4499443192806,48.254162310116,0
-0.44983868337672,48.254226758623,0
-0.44974188539359,48.254297091919,0
-0.44965466202839,48.254372774749,0
-0.44957767712165,48.254453231143,0
-0.44951151660444,48.2545378488,0
-0.44945668403824,48.254625983748,0
-0.44941359678128,48.254716965243,0
-0.44938258281071,48.254810100874,0
-0.44936387822476,48.254904681832,0
-0.44935762544394,48.254999988306,0
-0.45100000000002,48.255,0
</coordinates>
</LinearRing>
</outerBoundaryIs>
</Polygon>
</Placemark>
<Placemark>
<name>518</name>
<styleUrl>#stylehap2</styleUrl>
<Polygon>
<outerBoundaryIs>
<LinearRing>
<coordinates>
-0.44000000000005,48.128,0
-0.43836169079763,48.127999988358,0
-0.43836792203006,48.12809529501,0
-0.43838657443029,48.128189876495,0
-0.43841750610915,48.128283012985,0
-0.43846048172064,48.128373995647,0
-0.43851517425115,48.128462132032,0
-0.43858116750644,48.128546751355,0
-0.43865795927771,48.128627209592,0
-0.43874496516252,48.128702894387,0
-0.4388415230114,48.128773229709,0
-0.43894689796692,48.128837680242,0
-0.43906028805596,48.128895755456,0
-0.43918083029361,48.128947013339,0
-0.43930760725135,48.128991063769,0
-0.43943965404028,48.129027571476,0
-0.43957596565566,48.129056258597,0

-0.43971550462705,48.129076906794,0
-0.43985720891587,48.129089358911,0
-0.44000000000005,48.129093520173,0
-0.44014279108423,48.129089358911,0
-0.44028449537307,48.129076906794,0
-0.44042403434446,48.129056258597,0
-0.44056034595982,48.129027571476,0
-0.44069239274877,48.128991063769,0
-0.44081916970651,48.128947013339,0
-0.44093971194413,48.128895755456,0
-0.4410531020332,48.128837680242,0
-0.4411584769887,48.128773229709,0
-0.44125503483761,48.128702894387,0
-0.44134204072238,48.128627209592,0
-0.44141883249366,48.128546751355,0
-0.44148482574895,48.128462132032,0
-0.44153951827945,48.128373995647,0
-0.44158249389095,48.128283012985,0
-0.44161342556983,48.128189876495,0
-0.44163207797004,48.12809529501,0
-0.4416383092025,48.127999988358,0
-0.44163207191266,48.127904681884,0
-0.44161341363912,48.127810100924,0
-0.44158247644945,48.127716965291,0
-0.44153949585708,48.127625983794,0
-0.441484799027,48.127537848843,0
-0.44141880228409,48.127453231182,0
-0.44134200794305,48.127372774784,0
-0.44125500048451,48.12729709195,0
-0.44115844210567,48.127226758649,0
-0.44105306768013,48.127162310138,0
-0.44093967916483,48.127104236884,0
-0.44081913949692,48.12705298084,0
-0.44069236602682,48.127008932072,0
-0.44056032353745,48.1269724258,0
-0.44042401690294,48.126943739843,0
-0.44028448344235,48.126923092504,0
-0.44014278502685,48.126910640912,0
-0.44000000000005,48.126906479827,0
-0.43985721497325,48.126910640912,0
-0.43971551655774,48.126923092504,0
-0.43957598309716,48.126943739843,0
-0.43943967646265,48.1269724258,0
-0.4393076339733,48.127008932072,0
-0.43918086050318,48.12705298084,0
-0.43906032083529,48.127104236884,0
-0.43894693231999,48.127162310138,0
-0.43884155789443,48.127226758649,0
-0.43874499951559,48.12729709195,0
-0.43865799205705,48.127372774784,0
-0.43858119771604,48.127453231182,0
-0.4385152009731,48.127537848843,0
-0.43846050414302,48.127625983794,0
-0.43841752355067,48.127716965291,0
-0.43838658636098,48.127810100924,0
-0.43836792808744,48.127904681884,0
-0.43836169079763,48.127999988358,0

-0.44000000000005,48.128,0
</coordinates>
</LinearRing>
</outerBoundaryIs>
</Polygon>
</Placemark>
<Placemark>
<name>520</name>
<styleUrl>#stylehap2</styleUrl>
<Polygon>
<outerBoundaryIs>
<LinearRing>
<coordinates>
-0.437999999999999,48.302,0
-0.43635611377278,48.301999988287,0
-0.43636236619855,48.302095294939,0
-0.43638108207598,48.302189876425,0
-0.43641211903349,48.302283012919,0
-0.43645524092442,48.302373995584,0
-0.43651011962243,48.302462131974,0
-0.4365763375169,48.302546751302,0
-0.43665339068979,48.302627209545,0
-0.43674069274945,48.302702894345,0
-0.43683757929263,48.302773229673,0
-0.43694331296027,48.302837680213,0
-0.43705708904929,48.302895755432,0
-0.4371780416368,48.302947013321,0
-0.43730525017109,48.302991063756,0
-0.43743774647826,48.303027571468,0
-0.43757452213202,48.303056258593,0
-0.43771453612978,48.303076906792,0
-0.43785672281713,48.30308935891,0
-0.43799999999998,48.303093520174,0
-0.43814327718286,48.30308935891,0
-0.4382854638702,48.303076906792,0
-0.43842547786796,48.303056258593,0
-0.43856225352172,48.303027571468,0
-0.43869474982889,48.302991063756,0
-0.43882195836318,48.302947013321,0
-0.43894291095069,48.302895755432,0
-0.43905668703971,48.302837680213,0
-0.43916242070735,48.302773229673,0
-0.43925930725051,48.302702894345,0
-0.43934660931019,48.302627209545,0
-0.43942366248308,48.302546751302,0
-0.43948988037755,48.302462131974,0
-0.43954475907556,48.302373995584,0
-0.43958788096649,48.302283012919,0
-0.439618917924,48.302189876425,0
-0.43963763380143,48.302095294939,0
-0.4396438862272,48.301999988287,0
-0.43963762768617,48.301904681813,0
-0.43961890587929,48.301810100855,0
-0.4395878633583,48.301716965225,0
-0.43954473643891,48.301625983731,0
-0.43948985340026,48.301537848784,0
-0.4394236319848,48.301453231129,0

-0.43934657621764,48.301372774736,0
-0.43925927256915,48.301297091908,0
-0.43916238549098,48.301226758614,0
-0.43905665235836,48.301162310108,0
-0.43894287785814,48.301104236861,0
-0.43882192786491,48.301052980822,0
-0.43869472285161,48.301008932059,0
-0.43856223088508,48.300972425792,0
-0.43842546025978,48.300943739838,0
-0.43828545182549,48.300923092502,0
-0.43814327106759,48.300910640912,0
-0.43799999999998,48.300906479826,0
-0.43785672893239,48.300910640912,0
-0.43771454817449,48.300923092502,0
-0.4375745397402,48.300943739838,0
-0.4374377691149,48.300972425792,0
-0.43730527714837,48.301008932059,0
-0.43717807213507,48.301052980822,0
-0.43705712214184,48.301104236861,0
-0.43694334764162,48.301162310108,0
-0.436837614509,48.301226758614,0
-0.4367407274308,48.301297091908,0
-0.43665342378234,48.301372774736,0
-0.43657636801516,48.301453231129,0
-0.43651014659972,48.301537848784,0
-0.43645526356107,48.301625983731,0
-0.43641213664165,48.301716965225,0
-0.43638109412069,48.301810100855,0
-0.43636237231379,48.301904681813,0
-0.43635611377278,48.301999988287,0
-0.43799999999999,48.302,0
</coordinates>
</LinearRing>
</outerBoundaryIs>
</Polygon>
</Placemark>
<Placemark>
<name>524</name>
<styleUrl>#stylehap2</styleUrl>
<Polygon>
<outerBoundaryIs>
<LinearRing>
<coordinates>
-0.42899999999997,48.283,0
-0.42735672534199,48.282999988295,0
-0.42736297544373,48.283095294947,0
-0.42738168436033,48.283189876433,0
-0.42741270977311,48.283283012926,0
-0.42745581562324,48.28337399559,0
-0.42751067390638,48.28346213198,0
-0.42757686716722,48.283546751308,0
-0.42765389167515,48.28362720955,0
-0.42774116125666,48.283702894349,0
-0.42783801175563,48.283773229677,0
-0.4279437060874,48.283837680216,0
-0.42805743984814,48.283895755435,0
-0.42817834743725,48.283947013323,0

-0.42830550864541,48.283991063758,0
-0.428437955659,48.284027571468,0
-0.42857468042691,48.284056258593,0
-0.42871464233391,48.284076906792,0
-0.42885677612206,48.28408935891,0
-0.42899999999996,48.284093520174,0
-0.42914322387789,48.28408935891,0
-0.42928535766604,48.284076906792,0
-0.42942531957304,48.284056258593,0
-0.42956204434095,48.284027571468,0
-0.42969449135454,48.283991063758,0
-0.4298216525627,48.283947013323,0
-0.42994256015181,48.283895755435,0
-0.43005629391256,48.283837680216,0
-0.43016198824429,48.283773229677,0
-0.43025883874329,48.283702894349,0
-0.4303461083248,48.28362720955,0
-0.43042313283273,48.283546751308,0
-0.43048932609357,48.28346213198,0
-0.43054418437669,48.28337399559,0
-0.43058729022684,48.283283012926,0
-0.43061831563962,48.283189876433,0
-0.43063702455622,48.283095294947,0
-0.43064327465797,48.282999988295,0
-0.43063701844732,48.282904681821,0
-0.43061830360743,48.282810100862,0
-0.43058727263695,48.282716965232,0
-0.43054416176358,48.282625983738,0
-0.4304892991443,48.282537848791,0
-0.43042310236614,48.282453231134,0
-0.43034607526662,48.282372774742,0
-0.43025880409796,48.282297091913,0
-0.43016195306453,48.282226758617,0
-0.43005625926723,48.282162310111,0
-0.42994252709363,48.282104236864,0
-0.42982162209612,48.282052980824,0
-0.42969446440527,48.282008932061,0
-0.42956202172782,48.281972425793,0
-0.42942530198315,48.281943739839,0
-0.42928534563385,48.281923092502,0
-0.42914321776899,48.281910640912,0
-0.42899999999996,48.281906479827,0
-0.42885678223097,48.281910640912,0
-0.4287146543661,48.281923092502,0
-0.4285746980168,48.281943739839,0
-0.42843797827214,48.281972425793,0
-0.42830553559466,48.282008932061,0
-0.42817837790381,48.282052980824,0
-0.42805747290632,48.282104236864,0
-0.42794374073272,48.282162310111,0
-0.42783804693542,48.282226758617,0
-0.42774119590199,48.282297091913,0
-0.42765392473333,48.282372774742,0
-0.42757689763378,48.282453231134,0
-0.42751070085563,48.282537848791,0
-0.42745583823637,48.282625983738,0
-0.427412727363,48.282716965232,0

-0.42738169639252,48.282810100862,0
-0.42736298155263,48.282904681821,0
-0.42735672534199,48.282999988295,0
-0.42899999999997,48.283,0
</coordinates>
</LinearRing>
</outerBoundaryIs>
</Polygon>
</Placemark>
<Placemark>
<name>526</name>
<styleUrl>#stylehap2</styleUrl>
<Polygon>
<outerBoundaryIs>
<LinearRing>
<coordinates>
-0.428,48.253,0
-0.4263576896842,48.252999988307,0
-0.42636393612131,48.253095294959,0
-0.42638263406186,48.253189876445,0
-0.42641364127057,48.253283012937,0
-0.42645672182704,48.253373995601,0
-0.42651154791931,48.25346213199,0
-0.42657770233703,48.253546751317,0
-0.42665468164513,48.253627209558,0
-0.42674190001406,48.253702894357,0
-0.42683869367741,48.253773229684,0
-0.42694432598304,48.253837680221,0
-0.42705799299924,48.253895755439,0
-0.42717882963332,48.253947013327,0
-0.42730591621613,48.25399106376,0
-0.427438285502,48.25402757147,0
-0.42757493003149,48.254056258594,0
-0.42771480980005,48.254076906792,0
-0.42785686017505,48.25408935891,0
-0.428,48.254093520174,0
-0.42814313982493,48.25408935891,0
-0.42828519019993,48.254076906792,0
-0.42842506996852,48.254056258594,0
-0.42856171449798,48.25402757147,0
-0.42869408378385,48.25399106376,0
-0.42882117036666,48.253947013327,0
-0.42894200700077,48.253895755439,0
-0.42905567401697,48.253837680221,0
-0.42916130632259,48.253773229684,0
-0.42925809998594,48.253702894357,0
-0.42934531835485,48.253627209558,0
-0.42942229766295,48.253546751317,0
-0.42948845208069,48.25346213199,0
-0.42954327817296,48.253373995601,0
-0.42958635872941,48.253283012937,0
-0.42961736593812,48.253189876445,0
-0.42963606387868,48.253095294959,0
-0.42964231031581,48.252999988307,0
-0.4296360577798,48.252904681833,0
-0.42961735392567,48.252810100874,0
-0.42958634116837,48.252716965243,0

-0.4295432555969,48.252625983749,0
-0.42948842517559,48.252537848801,0
-0.42942226724634,48.252453231144,0
-0.4293452853509,48.25237277475,0
-0.42925806539744,48.25229709192,0
-0.42916127120049,48.252226758624,0
-0.42905563942846,48.252162310117,0
-0.42894197399679,48.252104236868,0
-0.42882113995005,48.252052980827,0
-0.42869405687878,48.252008932063,0
-0.42856169192194,48.251972425795,0
-0.42842505240745,48.25194373984,0
-0.42828517818748,48.251923092502,0
-0.42814313372605,48.251910640912,0
-0.428,48.251906479826,0
-0.42785686627393,48.251910640912,0
-0.42771482181252,48.251923092502,0
-0.42757494759253,48.25194373984,0
-0.42743830807806,48.251972425795,0
-0.42730594312121,48.252008932063,0
-0.42717886004996,48.252052980827,0
-0.42705802600319,48.252104236868,0
-0.42694436057152,48.252162310117,0
-0.42683872879949,48.252226758624,0
-0.42674193460257,48.25229709192,0
-0.42665471464911,48.25237277475,0
-0.42657773275367,48.252453231144,0
-0.42651157482439,48.252537848801,0
-0.42645674440308,48.252625983749,0
-0.42641365883161,48.252716965243,0
-0.42638264607433,48.252810100874,0
-0.42636394222021,48.252904681833,0
-0.4263576896842,48.252999988307,0
-0.428,48.253,0
</coordinates>
</LinearRing>
</outerBoundaryIs>
</Polygon>
</Placemark>
<Placemark>
<name>532</name>
<styleUrl>#stylehap2</styleUrl>
<Polygon>
<outerBoundaryIs>
<LinearRing>
<coordinates>
-0.404,48.358,0
-0.40235430754839,48.357999988264,0
-0.40236056683799,48.358095294916,0
-0.40237930327369,48.358189876403,0
-0.40241037432766,48.358283012897,0
-0.40245354359392,48.358373995563,0
-0.40250848258578,48.358462131955,0
-0.40257477323386,48.358546751285,0
-0.40265191106658,48.358627209529,0
-0.40273930904805,48.358702894331,0
-0.40283630204503,48.358773229662,0

-0.40294215188836,48.358837680203,0
-0.40305605299071,48.358895755424,0
-0.40317713847778,48.358947013316,0
-0.40330448678625,48.358991063752,0
-0.40343712867838,48.359027571465,0
-0.40357405461973,48.359056258591,0
-0.40371422246372,48.359076906791,0
-0.40385656538492,48.35908935891,0
-0.40399999999999,48.359093520174,0
-0.40414343461508,48.35908935891,0
-0.40428577753626,48.359076906791,0
-0.40442594538028,48.359056258591,0
-0.4045628713216,48.359027571465,0
-0.40469551321373,48.358991063752,0
-0.40482286152222,48.358947013316,0
-0.40494394700927,48.358895755424,0
-0.40505784811162,48.358837680203,0
-0.40516369795495,48.358773229662,0
-0.40526069095193,48.358702894331,0
-0.40534808893343,48.358627209529,0
-0.40542522676612,48.358546751285,0
-0.40549151741422,48.358462131955,0
-0.40554645640606,48.358373995563,0
-0.40558962567234,48.358283012897,0
-0.40562069672629,48.358189876403,0
-0.40563943316199,48.358095294916,0
-0.40564569245159,48.357999988264,0
-0.40563942702797,48.35790468179,0
-0.40562068464458,48.357810100832,0
-0.40558960801006,48.357716965203,0
-0.4055464336999,48.35762598371,0
-0.40549149035406,48.357537848765,0
-0.4054251961742,48.357453231111,0
-0.40534805573922,48.357372774721,0
-0.40526065616409,48.357297091895,0
-0.40516366263044,48.357226758602,0
-0.40505781332376,48.357162310099,0
-0.40494391381509,48.357104236853,0
-0.40482283093028,48.357052980816,0
-0.4046954861536,48.357008932055,0
-0.40456284861545,48.356972425789,0
-0.40442592771802,48.356943739837,0
-0.40428576545458,48.356923092501,0
-0.40414342848104,48.356910640912,0
-0.40399999999999,48.356906479826,0
-0.40385657151897,48.356910640912,0
-0.40371423454543,48.356923092501,0
-0.40357407228198,48.356943739837,0
-0.40343715138453,48.356972425789,0
-0.40330451384641,48.357008932055,0
-0.4031771690697,48.357052980816,0
-0.40305608618491,48.357104236853,0
-0.40294218667622,48.357162310099,0
-0.40283633736954,48.357226758602,0
-0.40273934383592,48.357297091895,0
-0.40265194426076,48.357372774721,0
-0.40257480382581,48.357453231111,0

-0.40250850964592,48.357537848765,0
-0.40245356630008,48.35762598371,0
-0.40241039198992,48.357716965203,0
-0.4023793153554,48.357810100832,0
-0.40236057297203,48.35790468179,0
-0.40235430754839,48.357999988264,0
-0.404,48.358,0
</coordinates>
</LinearRing>
</outerBoundaryIs>
</Polygon>
</Placemark>
<Placemark>
<name>535</name>
<styleUrl>#stylehap2</styleUrl>
<Polygon>
<outerBoundaryIs>
<LinearRing>
<coordinates>
-0.40100000000007,48.106,0
-0.39936239217114,48.105999988367,0
-0.39936862073829,48.106095295019,0
-0.39938726515554,48.106189876503,0
-0.39941818359443,48.106283012994,0
-0.39946114080963,48.106373995654,0
-0.39951580992748,48.10646213204,0
-0.39958177493187,48.106546751362,0
-0.39965853382895,48.106627209598,0
-0.39974550246641,48.106702894392,0
-0.39984201897831,48.106773229714,0
-0.39994734882173,48.106837680246,0
-0.40006069036696,48.106895755458,0
-0.4001811809985,48.106947013342,0
-0.40030790368064,48.106991063771,0
-0.40043989393762,48.107027571477,0
-0.40057614719496,48.107056258598,0
-0.40071562642646,48.107076906794,0
-0.40085727004826,48.107089358911,0
-0.40100000000006,48.107093520173,0
-0.40114272995189,48.107089358911,0
-0.40128437357369,48.107076906794,0
-0.40142385280518,48.107056258598,0
-0.40156010606253,48.107027571477,0
-0.40169209631948,48.106991063771,0
-0.40181881900165,48.106947013342,0
-0.40193930963316,48.106895755458,0
-0.40205265117839,48.106837680246,0
-0.40215798102182,48.106773229714,0
-0.40225449753372,48.106702894392,0
-0.40234146617117,48.106627209598,0
-0.40241822506826,48.106546751362,0
-0.40248419007267,48.10646213204,0
-0.4025388591905,48.106373995654,0
-0.40258181640572,48.106283012994,0
-0.40261273484461,48.106189876503,0
-0.40263137926186,48.106095295019,0
-0.40263760782898,48.105999988367,0

-0.40263137321173,48.105904681893,0
-0.40261272292822,48.105810100933,0
-0.40258179898513,48.1057169653,0
-0.40253883679504,48.105625983802,0
-0.40248416338278,48.10553784885,0
-0.40241819489492,48.105453231189,0
-0.40234143343118,48.10537277479,0
-0.40225446322187,48.105297091955,0
-0.40215794618065,48.105226758654,0
-0.40205261686654,48.105162310141,0
-0.40193927689317,48.105104236887,0
-0.40181878882831,48.105052980842,0
-0.40169206962962,48.105008932074,0
-0.40156008366705,48.104972425802,0
-0.4014238353846,48.104943739844,0
-0.4012843616573,48.104923092504,0
-0.40114272390176,48.104910640913,0
-0.40100000000006,48.104906479826,0
-0.40085727609836,48.104910640913,0
-0.40071563834282,48.104923092504,0
-0.40057616461555,48.104943739844,0
-0.40043991633308,48.104972425802,0
-0.40030793037053,48.105008932074,0
-0.40018121117184,48.105052980842,0
-0.40006072310695,48.105104236887,0
-0.39994738313361,48.105162310141,0
-0.39984205381948,48.105226758654,0
-0.39974553677828,48.105297091955,0
-0.39965856656894,48.10537277479,0
-0.3995818051052,48.105453231189,0
-0.39951583661737,48.10553784885,0
-0.39946116320511,48.105625983802,0
-0.39941820101501,48.1057169653,0
-0.3993872770719,48.105810100933,0
-0.39936862678839,48.105904681893,0
-0.39936239217114,48.105999988367,0
-0.40100000000007,48.106,0
</coordinates>
</LinearRing>
</outerBoundaryIs>
</Polygon>
</Placemark>
<Placemark>
<name>544</name>
<styleUrl>#stylehap2</styleUrl>
<Polygon>
<outerBoundaryIs>
<LinearRing>
<coordinates>
-0.39200000000005,48.282,0
-0.39035675751231,48.281999988295,0
-0.3903630074918,48.282095294947,0
-0.39038171604225,48.282189876433,0
-0.39041274084775,48.282283012926,0
-0.39045584585408,48.282373995591,0
-0.39051070306332,48.282462131981,0
-0.39057689502838,48.282546751308,0

-0.39065391802845,48.28262720955,0
-0.39074118590154,48.28270289435,0
-0.3908380345045,48.282773229678,0
-0.39094372676706,48.282837680216,0
-0.39105745830122,48.282895755435,0
-0.39117836352328,48.282947013324,0
-0.39130552224196,48.282991063758,0
-0.39143796666256,48.283027571468,0
-0.39157468875374,48.283056258593,0
-0.39171464792063,48.283076906792,0
-0.39185677892612,48.28308935891,0
-0.39200000000005,48.283093520174,0
-0.39214322107397,48.28308935891,0
-0.39228535207949,48.283076906792,0
-0.39242531124636,48.283056258593,0
-0.39256203333754,48.283027571468,0
-0.39269447775816,48.282991063758,0
-0.39282163647684,48.282947013324,0
-0.39294254169887,48.282895755435,0
-0.39305627323305,48.282837680216,0
-0.39316196549562,48.282773229678,0
-0.39325881409858,48.28270289435,0
-0.39334608197165,48.28262720955,0
-0.39342310497174,48.282546751308,0
-0.39348929693677,48.282462131981,0
-0.39354415414601,48.282373995591,0
-0.39358725915237,48.282283012926,0
-0.39361828395787,48.282189876433,0
-0.39363699250832,48.282095294947,0
-0.39364324248778,48.281999988295,0
-0.39363698639975,48.281904681821,0
-0.39361827192634,48.281810100863,0
-0.39358724156345,48.281716965232,0
-0.39354413153412,48.281625983738,0
-0.39348926998897,48.281537848791,0
-0.39342307450681,48.281453231135,0
-0.3933460489153,48.281372774742,0
-0.39325877945516,48.281297091913,0
-0.39316193031777,48.281226758618,0
-0.39305623858964,48.281162310112,0
-0.3929425086425,48.281104236864,0
-0.39282160601191,48.281052980824,0
-0.39269445081036,48.281008932061,0
-0.39256201072565,48.280972425793,0
-0.39242529365743,48.280943739839,0
-0.39228534004794,48.280923092502,0
-0.3921432149654,48.280910640912,0
-0.39200000000005,48.280906479826,0
-0.3918567850347,48.280910640912,0
-0.39171465995216,48.280923092502,0
-0.39157470634266,48.280943739839,0
-0.39143798927444,48.280972425793,0
-0.39130554918976,48.281008932061,0
-0.39117839398819,48.281052980824,0
-0.39105749135759,48.281104236864,0
-0.39094376141048,48.281162310112,0
-0.39083806968235,48.281226758618,0

-0.39074122054496,48.281297091913,0
-0.39065395108482,48.281372774742,0
-0.39057692549329,48.281453231135,0
-0.39051073001112,48.281537848791,0
-0.39045586846597,48.281625983738,0
-0.39041275843667,48.281716965232,0
-0.39038172807378,48.281810100863,0
-0.39036301360037,48.281904681821,0
-0.39035675751231,48.281999988295,0
-0.39200000000005,48.282,0
</coordinates>
</LinearRing>
</outerBoundaryIs>
</Polygon>
</Placemark>
<Placemark>
<name>545</name>
<styleUrl>#stylehap2</styleUrl>
<Polygon>
<outerBoundaryIs>
<LinearRing>
<coordinates>
-0.39200000000005,48.241,0
-0.39035807497829,48.240999988312,0
-0.39036431995125,48.241095294964,0
-0.39038301350641,48.24118987645,0
-0.39041401344185,48.241283012942,0
-0.39045708389245,48.241373995606,0
-0.39051189712317,48.241462131994,0
-0.39057803602148,48.241546751321,0
-0.39065499727042,48.241627209561,0
-0.39074219517783,48.24170289436,0
-0.39083896613301,48.241773229686,0
-0.39094457365667,48.241837680223,0
-0.39105821400569,48.24189575544,0
-0.39117902229038,48.241947013328,0
-0.39130607905732,48.241991063761,0
-0.39143841728779,48.24202757147,0
-0.39157502975874,48.242056258594,0
-0.39171487670965,48.242076906792,0
-0.39185689375771,48.24208935891,0
-0.39200000000005,48.242093520174,0
-0.39214310624241,48.24208935891,0
-0.39228512329044,48.242076906792,0
-0.39242497024138,48.242056258594,0
-0.3925615827123,48.24202757147,0
-0.3926939209428,48.241991063761,0
-0.39282097770972,48.241947013328,0
-0.39294178599442,48.24189575544,0
-0.39305542634345,48.241837680223,0
-0.39316103386709,48.241773229686,0
-0.39325780482229,48.24170289436,0
-0.3933450027297,48.241627209561,0
-0.39342196397861,48.241546751321,0
-0.39348810287693,48.241462131994,0
-0.39354291610764,48.241373995606,0
-0.39358598655825,48.241283012942,0

-0.39361698649369,48.24118987645,0
-0.39363568004887,48.241095294964,0
-0.39364192502183,48.240999988312,0
-0.39363567395396,48.240904681838,0
-0.3936169744891,48.240810100879,0
-0.39358596900873,48.240716965248,0
-0.39354289354642,48.240625983753,0
-0.39348807598951,48.240537848805,0
-0.39342193358195,48.240453231147,0
-0.39334496974737,48.240372774753,0
-0.39325777025648,48.240297091923,0
-0.39316099876804,48.240226758626,0
-0.39305539177764,48.240162310119,0
-0.3929417530121,48.240104236869,0
-0.39282094731305,48.240052980828,0
-0.39269389405536,48.240008932064,0
-0.39256156015107,48.239972425795,0
-0.39242495269184,48.23994373984,0
-0.39228511128586,48.239923092503,0
-0.39214310014753,48.239910640912,0
-0.39200000000005,48.239906479826,0
-0.39185689985259,48.239910640912,0
-0.39171488871424,48.239923092503,0
-0.39157504730825,48.23994373984,0
-0.39143843984902,48.239972425795,0
-0.39130610594473,48.240008932064,0
-0.39117905268704,48.240052980828,0
-0.391058246988,48.240104236869,0
-0.39094460822248,48.240162310119,0
-0.39083900123206,48.240226758626,0
-0.39074222974364,48.240297091923,0
-0.39065503025272,48.240372774753,0
-0.39057806641817,48.240453231147,0
-0.39051192401061,48.240537848805,0
-0.3904571064537,48.240625983753,0
-0.39041403099139,48.240716965248,0
-0.39038302551099,48.240810100879,0
-0.39036432604613,48.240904681838,0
-0.39035807497829,48.240999988312,0
-0.39200000000005,48.241,0
</coordinates>
</LinearRing>
</outerBoundaryIs>
</Polygon>
</Placemark>
<Placemark>
<name>546</name>
<styleUrl>#stylehap2</styleUrl>
<Polygon>
<outerBoundaryIs>
<LinearRing>
<coordinates>
-0.39200000000005,47.968,0
-0.39036677255271,47.967999988424,0
-0.39037298447383,47.968095295074,0
-0.39039157903389,47.968189876558,0
-0.39042241478335,47.968283013046,0

-0.39046525710577,47.968373995704,0
-0.39051978000141,47.968462132086,0
-0.39058556856664,47.968546751404,0
-0.39066212215014,47.968627209636,0
-0.39074885816193,47.968702894425,0
-0.39084511650637,47.968773229742,0
-0.3909501646053,47.968837680269,0
-0.39106320297329,47.968895755477,0
-0.39118337130233,47.968947013356,0
-0.39130975500988,47.968991063781,0
-0.39144139220025,47.969027571484,0
-0.39157728098639,47.969056258602,0
-0.39171638711637,47.969076906796,0
-0.39185765184633,47.969089358911,0
-0.39200000000005,47.969093520174,0
-0.39214234815379,47.969089358911,0
-0.39228361288375,47.969076906796,0
-0.39242271901371,47.969056258602,0
-0.39255860779984,47.969027571484,0
-0.39269024499022,47.968991063781,0
-0.39281662869777,47.968947013356,0
-0.39293679702681,47.968895755477,0
-0.39304983539479,47.968837680269,0
-0.39315488349375,47.968773229742,0
-0.39325114183817,47.968702894425,0
-0.39333787784998,47.968627209636,0
-0.39341443143348,47.968546751404,0
-0.39348021999871,47.968462132086,0
-0.39353474289433,47.968373995704,0
-0.39357758521674,47.968283013046,0
-0.3936084209662,47.968189876558,0
-0.39362701552626,47.968095295074,0
-0.39363322744741,47.967999988424,0
-0.3936270095215,47.967904681949,0
-0.39360840913914,47.967810100987,0
-0.39357756792671,47.967716965352,0
-0.3935347206667,47.967625983851,0
-0.39348019350886,47.967537848896,0
-0.39341440148627,47.967453231231,0
-0.39333784535534,47.967372774828,0
-0.39325110778346,47.967297091988,0
-0.39315484891369,47.967226758682,0
-0.39304980134011,47.967162310165,0
-0.3929367645322,47.967104236906,0
-0.39281659875055,47.967052980856,0
-0.39269021850037,47.967008932084,0
-0.39255858557222,47.966972425808,0
-0.39242270172368,47.966943739847,0
-0.39228360105666,47.966923092506,0
-0.39214234214903,47.966910640913,0
-0.39200000000005,47.966906479826,0
-0.39185765785109,47.966910640913,0
-0.39171639894345,47.966923092506,0
-0.39157729827642,47.966943739847,0
-0.39144141442788,47.966972425808,0
-0.39130978149975,47.967008932084,0
-0.39118340124954,47.967052980856,0

-0.39106323546792,47.967104236906,0
-0.39095019866001,47.967162310165,0
-0.39084515108643,47.967226758682,0
-0.39074889221664,47.967297091988,0
-0.39066215464475,47.967372774828,0
-0.39058559851383,47.967453231231,0
-0.39051980649126,47.967537848896,0
-0.39046527933339,47.967625983851,0
-0.39042243207338,47.967716965352,0
-0.39039159086098,47.967810100987,0
-0.39037299047859,47.967904681949,0
-0.39036677255271,47.967999988424,0
-0.39200000000005,47.968,0
</coordinates>
</LinearRing>
</outerBoundaryIs>
</Polygon>
</Placemark>
<Placemark>
<name>550</name>
<styleUrl>#stylehap2</styleUrl>
<Polygon>
<outerBoundaryIs>
<LinearRing>
<coordinates>
-0.38,48.186,0
-0.37835983767872,48.185999988335,0
-0.37836607595323,48.186095294986,0
-0.3783847494455,48.186189876472,0
-0.37841571610608,48.186283012963,0
-0.37845874032292,48.186373995626,0
-0.37851349471266,48.186462132013,0
-0.37857956261049,48.186546751338,0
-0.37865644123958,48.186627209576,0
-0.37874354553657,48.186702894373,0
-0.3788402126031,48.186773229697,0
-0.37894570675052,48.186837680232,0
-0.37905922509861,48.186895755448,0
-0.37917990368621,48.186947013333,0
-0.37930682404704,48.186991063765,0
-0.37943902020069,48.187027571473,0
-0.37957548600547,48.187056258596,0
-0.37971518281736,48.187076906793,0
-0.37985704739627,48.18708935891,0
-0.38,48.187093520173,0
-0.38014295260374,48.18708935891,0
-0.38028481718264,48.187076906793,0
-0.38042451399451,48.187056258596,0
-0.38056097979931,48.187027571473,0
-0.38069317595296,48.186991063765,0
-0.3808200963138,48.186947013333,0
-0.38094077490139,48.186895755448,0
-0.38105429324945,48.186837680232,0
-0.38115978739687,48.186773229697,0
-0.38125645446343,48.186702894373,0
-0.3813435587604,48.186627209576,0
-0.38142043738952,48.186546751338,0

-0.38148650528732,48.186462132013,0
-0.38154125967706,48.186373995626,0
-0.38158428389392,48.186283012963,0
-0.38161525055448,48.186189876472,0
-0.38163392404675,48.186095294986,0
-0.38164016232126,48.185999988335,0
-0.38163391797016,48.18590468186,0
-0.38161523858595,48.185810100901,0
-0.38158426639706,48.185716965269,0
-0.38154123718357,48.185625983773,0
-0.38148647848059,48.185537848823,0
-0.3814204070841,48.185453231164,0
-0.3813435258771,48.185372774768,0
-0.38125642000136,48.185297091936,0
-0.38115975240319,48.185226758637,0
-0.38105425878741,48.185162310128,0
-0.38094074201807,48.185104236877,0
-0.38082006600835,48.185052980834,0
-0.38069314914623,48.185008932068,0
-0.38056095730579,48.184972425798,0
-0.38042449649766,48.184943739841,0
-0.38028480521409,48.184923092503,0
-0.38014294652715,48.184910640912,0
-0.38,48.184906479826,0
-0.37985705347286,48.184910640912,0
-0.37971519478589,48.184923092503,0
-0.37957550350232,48.184943739841,0
-0.37943904269419,48.184972425798,0
-0.37930685085375,48.185008932068,0
-0.37917993399162,48.185052980834,0
-0.37905925798191,48.185104236877,0
-0.3789457412126,48.185162310128,0
-0.37884024759681,48.185226758637,0
-0.37874357999862,48.185297091936,0
-0.3786564741229,48.185372774768,0
-0.37857959291591,48.185453231164,0
-0.37851352151939,48.185537848823,0
-0.37845876281643,48.185625983773,0
-0.37841573360292,48.185716965269,0
-0.37838476141405,48.185810100901,0
-0.37836608202982,48.18590468186,0
-0.37835983767872,48.185999988335,0
-0.38,48.186,0
</coordinates>
</LinearRing>
</outerBoundaryIs>
</Polygon>
</Placemark>
<Placemark>
<name>554</name>
<styleUrl>#stylehap2</styleUrl>
<Polygon>
<outerBoundaryIs>
<LinearRing>
<coordinates>
-0.36699999999996,48.244,0
-0.36535797867837,48.243999988311,0

-0.3653642240173,48.244095294962,0
-0.36538291866855,48.244189876449,0
-0.36541392042185,48.244283012941,0
-0.36545699339829,48.244373995605,0
-0.36551180984361,48.244462131993,0
-0.36557795262083,48.24454675132,0
-0.36565491838343,48.24462720956,0
-0.36574212140496,48.244702894359,0
-0.36583889803578,48.244773229685,0
-0.36594451175341,48.244837680223,0
-0.3660581587676,48.24489575544,0
-0.3661789741379,48.244947013327,0
-0.36630603835696,48.244991063761,0
-0.36643838434939,48.24502757147,0
-0.36657500483299,48.245056258594,0
-0.3667148599863,48.245076906792,0
-0.36685688536402,48.24508935891,0
-0.36699999999997,48.245093520173,0
-0.3671431146359,48.24508935891,0
-0.36728514001362,48.245076906792,0
-0.36742499516695,48.245056258594,0
-0.36756161565053,48.24502757147,0
-0.36769396164296,48.244991063761,0
-0.36782102586202,48.244947013327,0
-0.36794184123234,48.24489575544,0
-0.36805548824651,48.244837680223,0
-0.36816110196414,48.244773229685,0
-0.36825787859496,48.244702894359,0
-0.36834508161649,48.24462720956,0
-0.36842204737909,48.24454675132,0
-0.36848819015631,48.244462131993,0
-0.36854300660163,48.244373995605,0
-0.36858607957807,48.244283012941,0
-0.36861708133137,48.244189876449,0
-0.36863577598262,48.244095294962,0
-0.36864202132155,48.243999988311,0
-0.36863576988675,48.243904681837,0
-0.36861706932482,48.243810100878,0
-0.36858606202566,48.243716965247,0
-0.36854298403669,48.243625983752,0
-0.36848816326447,48.243537848804,0
-0.36842201697744,48.243453231146,0
-0.36834504862877,48.243372774752,0
-0.36825784402347,48.243297091922,0
-0.36816106685934,48.243226758625,0
-0.36805545367503,48.243162310118,0
-0.36794180824459,48.243104236869,0
-0.36782099546037,48.243052980828,0
-0.36769393475112,48.243008932064,0
-0.36756159308562,48.242972425795,0
-0.36742497761454,48.24294373984,0
-0.36728512800708,48.242923092503,0
-0.36714310854002,48.242910640912,0
-0.36699999999997,48.242906479826,0
-0.3668568914599,48.242910640912,0
-0.36671487199284,48.242923092503,0
-0.36657502238538,48.24294373984,0

-0.3664384069143,48.242972425795,0
-0.3663060652488,48.243008932064,0
-0.36617900453955,48.243052980828,0
-0.36605819175532,48.243104236869,0
-0.36594454632489,48.243162310118,0
-0.36583893314061,48.243226758625,0
-0.36574215597645,48.243297091922,0
-0.36565495137117,48.243372774752,0
-0.36557798302248,48.243453231146,0
-0.36551183673545,48.243537848804,0
-0.36545701596323,48.243625983752,0
-0.36541393797426,48.243716965247,0
-0.36538293067509,48.243810100878,0
-0.36536423011317,48.243904681837,0
-0.36535797867837,48.243999988311,0
-0.36699999999996,48.244,0
</coordinates>
</LinearRing>
</outerBoundaryIs>
</Polygon>
</Placemark>
<Placemark>
<name>561</name>
<styleUrl>#stylehap2</styleUrl>
<Polygon>
<outerBoundaryIs>
<LinearRing>
<coordinates>
-0.36199999999997,48.375,0
-0.36035375813327,48.374999988257,0
-0.36036001951072,48.375095294909,0
-0.3603787621998,48.375189876396,0
-0.36040984362518,48.375283012891,0
-0.36045302730202,48.375373995557,0
-0.36050798463398,48.375462131949,0
-0.36057429741218,48.375546751279,0
-0.3606514609966,48.375627209524,0
-0.36073888815544,48.375702894327,0
-0.36083591353342,48.375773229658,0
-0.36094179871492,48.3758376802,0
-0.36105573784368,48.375895755422,0
-0.36117686375595,48.375947013314,0
-0.36130425458075,48.375991063751,0
-0.36143694075676,48.376027571464,0
-0.36157391241238,48.376056258591,0
-0.36171412705315,48.376076906791,0
-0.36185651749735,48.37608935891,0
-0.36199999999997,48.376093520174,0
-0.36214348250259,48.37608935891,0
-0.3622858729468,48.376076906791,0
-0.36242608758757,48.376056258591,0
-0.36256305924318,48.376027571464,0
-0.36269574541917,48.375991063751,0
-0.36282313624399,48.375947013314,0
-0.36294426215627,48.375895755422,0
-0.36305820128502,48.3758376802,0
-0.36316408646653,48.375773229658,0

-0.36326111184448,48.375702894327,0
-0.36334853900334,48.375627209524,0
-0.36342570258777,48.375546751279,0
-0.36349201536597,48.375462131949,0
-0.36354697269792,48.375373995557,0
-0.36359015637474,48.375283012891,0
-0.36362123780012,48.375189876396,0
-0.36363998048922,48.375095294909,0
-0.36364624186665,48.374999988257,0
-0.36363997434946,48.374904681783,0
-0.36362122570717,48.374810100826,0
-0.36359013869603,48.374716965196,0
-0.3635469499706,48.374625983704,0
-0.36349198828059,48.374537848759,0
-0.36342567196735,48.374453231106,0
-0.36334850577823,48.374372774716,0
-0.36326107702423,48.37429709189,0
-0.36316405110909,48.374226758598,0
-0.36305816646474,48.374162310096,0
-0.36294422893115,48.374104236851,0
-0.36282310562354,48.374052980814,0
-0.36269571833382,48.374008932054,0
-0.36256303651586,48.373972425789,0
-0.36242606990885,48.373943739836,0
-0.36228586085385,48.373923092501,0
-0.36214347636282,48.373910640912,0
-0.36199999999997,48.373906479826,0
-0.3618565236371,48.373910640912,0
-0.3617141391461,48.373923092501,0
-0.3615739300911,48.373943739836,0
-0.36143696348406,48.373972425789,0
-0.36130428166613,48.374008932054,0
-0.36117689437637,48.374052980814,0
-0.36105577106879,48.374104236851,0
-0.3609418335352,48.374162310096,0
-0.36083594889085,48.374226758598,0
-0.36073892297572,48.37429709189,0
-0.36065149422172,48.374372774716,0
-0.3605743280326,48.374453231106,0
-0.36050801171933,48.374537848759,0
-0.36045305002935,48.374625983704,0
-0.36040986130389,48.374716965196,0
-0.36037877429275,48.374810100826,0
-0.36036002565046,48.374904681783,0
-0.36035375813327,48.374999988257,0
-0.36199999999997,48.375,0
</coordinates>
</LinearRing>
</outerBoundaryIs>
</Polygon>
</Placemark>
<Placemark>
<name>569</name>
<styleUrl>#stylehap2</styleUrl>
<Polygon>
<outerBoundaryIs>
<LinearRing>

<coordinates>
-0.34400000000005,48.339,0
-0.34235492099548,48.338999988272,0
-0.34236117795392,48.339095294924,0
-0.34237990740741,48.339189876411,0
-0.34241096688121,48.339283012904,0
-0.34245412005743,48.33937399557,0
-0.34250903857171,48.339462131961,0
-0.34257530451054,48.33954675129,0
-0.34265241359028,48.339627209534,0
-0.34273977899389,48.339702894336,0
-0.342836735836,48.339773229666,0
-0.34294254622267,48.339837680206,0
-0.3430564048668,48.339895755427,0
-0.34317744521725,48.339947013318,0
-0.34330474605428,48.339991063754,0
-0.34343733850148,48.340027571466,0
-0.34357421340073,48.340056258592,0
-0.34371432899401,48.340076906791,0
-0.34385661885359,48.34008935891,0
-0.34400000000004,48.340093520174,0
-0.3441433811465,48.34008935891,0
-0.34428567100608,48.340076906791,0
-0.34442578659936,48.340056258592,0
-0.34456266149861,48.340027571466,0
-0.34469525394581,48.339991063754,0
-0.34482255478284,48.339947013318,0
-0.34494359513331,48.339895755427,0
-0.34505745377745,48.339837680206,0
-0.34516326416409,48.339773229666,0
-0.34526022100623,48.339702894336,0
-0.34534758640984,48.339627209534,0
-0.34542469548958,48.33954675129,0
-0.34549096142838,48.339462131961,0
-0.34554587994266,48.33937399557,0
-0.34558903311888,48.339283012904,0
-0.34562009259268,48.339189876411,0
-0.3456388220462,48.339095294924,0
-0.34564507900463,48.338999988272,0
-0.34563881591854,48.338904681798,0
-0.34562008052354,48.33881010084,0
-0.34558901547499,48.33871696521,0
-0.34554585726012,48.338625983717,0
-0.34549093439639,48.338537848772,0
-0.34542466492946,48.338453231117,0
-0.34534755325016,48.338372774726,0
-0.34526018625454,48.338297091899,0
-0.34516322887631,48.338226758606,0
-0.34505741902576,48.338162310102,0
-0.34494356197364,48.338104236856,0
-0.34482252422275,48.338052980818,0
-0.34469522691382,48.338008932057,0
-0.34456263881606,48.33797242579,0
-0.34442576895547,48.337943739837,0
-0.34428565893696,48.337923092501,0
-0.34414337501885,48.337910640912,0
-0.34400000000004,48.337906479827,0

-0.34385662498124,48.337910640912,0
-0.34371434106315,48.337923092501,0
-0.34357423104462,48.337943739837,0
-0.34343736118403,48.33797242579,0
-0.3433047730863,48.338008932057,0
-0.34317747577736,48.338052980818,0
-0.34305643802645,48.338104236856,0
-0.34294258097432,48.338162310102,0
-0.34283677112377,48.338226758606,0
-0.34273981374555,48.338297091899,0
-0.34265244674993,48.338372774726,0
-0.34257533507066,48.338453231117,0
-0.34250906560373,48.338537848772,0
-0.34245414273997,48.338625983717,0
-0.3424109845251,48.33871696521,0
-0.34237991947655,48.33881010084,0
-0.34236118408158,48.338904681798,0
-0.34235492099548,48.338999988272,0
-0.34400000000005,48.339,0
</coordinates>
</LinearRing>
</outerBoundaryIs>
</Polygon>
</Placemark>
<Placemark>
<name>570</name>
<styleUrl>#stylehap2</styleUrl>
<Polygon>
<outerBoundaryIs>
<LinearRing>
<coordinates>
-0.34400000000005,48.376,0
-0.34235372579888,48.375999988256,0
-0.3423599872992,48.376095294909,0
-0.34237873035631,48.376189876396,0
-0.34240981239207,48.37628301289,0
-0.34245299691701,48.376373995557,0
-0.34250795532832,48.376462131949,0
-0.34257426940894,48.376546751279,0
-0.34265143450891,48.376627209524,0
-0.34273886338494,48.376702894327,0
-0.34283589066861,48.376773229658,0
-0.34294177792986,48.3768376802,0
-0.34305571929656,48.376895755422,0
-0.34317684758797,48.376947013314,0
-0.34330424091497,48.376991063751,0
-0.34343692969718,48.377027571464,0
-0.34357390404319,48.37705625859,0
-0.34371412143806,48.377076906791,0
-0.34385651467912,48.37708935891,0
-0.34400000000004,48.377093520174,0
-0.34414348532097,48.37708935891,0
-0.34428587856203,48.377076906791,0
-0.3444260959569,48.37705625859,0
-0.34456307030294,48.377027571464,0
-0.34469575908514,48.376991063751,0
-0.34482315241215,48.376947013314,0

-0.34494428070355,48.376895755422,0
-0.34505822207025,48.3768376802,0
-0.34516410933151,48.376773229658,0
-0.34526113661517,48.376702894327,0
-0.34534856549118,48.376627209524,0
-0.34542573059118,48.376546751279,0
-0.34549204467177,48.376462131949,0
-0.34554700308308,48.376373995557,0
-0.34559018760802,48.37628301289,0
-0.34562126964378,48.376189876396,0
-0.34564001270091,48.376095294909,0
-0.34564627420121,48.375999988256,0
-0.34564000656081,48.375904681783,0
-0.34562125755016,48.375810100825,0
-0.34559016992833,48.375716965196,0
-0.34554698035453,48.375625983704,0
-0.34549201758492,48.375537848759,0
-0.34542569996905,48.375453231106,0
-0.34534853226425,48.375372774716,0
-0.34526110179298,48.37529709189,0
-0.34516407397214,48.375226758598,0
-0.34505818724806,48.375162310096,0
-0.34494424747661,48.375104236851,0
-0.34482312179002,48.375052980814,0
-0.34469573199829,48.375008932054,0
-0.34456304757436,48.374972425789,0
-0.34442607827722,48.374943739836,0
-0.34428586646841,48.374923092501,0
-0.34414347918087,48.374910640912,0
-0.34400000000004,48.374906479826,0
-0.34385652081922,48.374910640912,0
-0.34371413353168,48.374923092501,0
-0.34357392172287,48.374943739836,0
-0.34343695242573,48.374972425789,0
-0.34330426800182,48.375008932054,0
-0.34317687821007,48.375052980814,0
-0.34305575252348,48.375104236851,0
-0.34294181275203,48.375162310096,0
-0.34283592602798,48.375226758598,0
-0.34273889820711,48.37529709189,0
-0.34265146773586,48.375372774716,0
-0.34257430003104,48.375453231106,0
-0.34250798241517,48.375537848759,0
-0.34245301964558,48.375625983704,0
-0.34240983007176,48.375716965196,0
-0.34237874244995,48.375810100825,0
-0.34235999343928,48.375904681783,0
-0.34235372579888,48.375999988256,0
-0.34400000000005,48.376,0
</coordinates>
</LinearRing>
</outerBoundaryIs>
</Polygon>
</Placemark>
<Placemark>
<name>573</name>
<styleUrl>#stylehap2</styleUrl>

<Polygon>
<outerBoundaryIs>
<LinearRing>
<coordinates>
-0.34199999999998,48.423,0
-0.34035220407795,48.422999988237,0
-0.34035847136095,48.423095294889,0
-0.34037723173816,48.423189876377,0
-0.34040834249972,48.423283012872,0
-0.34045156693777,48.423373995539,0
-0.34050657614585,48.423462131933,0
-0.34057295152044,48.423546751264,0
-0.34065018794524,48.423627209511,0
-0.34073769763413,48.423702894316,0
-0.34083481460379,48.423773229648,0
-0.34094079974155,48.423837680192,0
-0.34105484643041,48.423895755416,0
-0.34117608668797,48.423947013309,0
-0.34130359777293,48.423991063748,0
-0.3414364092086,48.424027571462,0
-0.34157351016999,48.424056258589,0
-0.34171385717835,48.42407690679,0
-0.34185638204443,48.42408935891,0
-0.34199999999997,48.424093520174,0
-0.34214361795555,48.42408935891,0
-0.34228614282162,48.42407690679,0
-0.34242648982998,48.424056258589,0
-0.34256359079138,48.424027571462,0
-0.34269640222704,48.423991063748,0
-0.342823913312,48.423947013309,0
-0.34294515356957,48.423895755416,0
-0.34305920025842,48.423837680192,0
-0.34316518539619,48.423773229648,0
-0.34326230236584,48.423702894316,0
-0.34334981205473,48.423627209511,0
-0.34342704847953,48.423546751264,0
-0.34349342385413,48.423462131933,0
-0.3435484330622,48.423373995539,0
-0.34359165750025,48.423283012872,0
-0.34362276826181,48.423189876377,0
-0.34364152863903,48.423095294889,0
-0.343647795922,48.422999988237,0
-0.3436415224831,48.422904681763,0
-0.343622756137,48.422810100806,0
-0.34359163977497,48.422716965178,0
-0.343548410275,48.422625983687,0
-0.3434933966974,48.422537848743,0
-0.34342701777843,48.422453231091,0
-0.34334977874209,48.422372774703,0
-0.34326226745384,48.422297091879,0
-0.3431651499456,48.422226758589,0
-0.34305916534642,48.422162310088,0
-0.34294512025692,48.422104236845,0
-0.34282388261092,48.42205298081,0
-0.34269637507032,48.422008932051,0
-0.34256356800418,48.421972425786,0
-0.3424264721047,48.421943739835,0

-0.34228613069681,48.4219230925,0
-0.3421436117996,48.421910640912,0
-0.34199999999997,48.421906479827,0
-0.34185638820035,48.421910640912,0
-0.34171386930316,48.4219230925,0
-0.34157352789528,48.421943739835,0
-0.34143643199579,48.421972425786,0
-0.34130362492965,48.422008932051,0
-0.34117611738906,48.42205298081,0
-0.34105487974305,48.422104236845,0
-0.34094083465356,48.422162310088,0
-0.34083485005438,48.422226758589,0
-0.34073773254614,48.422297091879,0
-0.34065022125789,48.422372774703,0
-0.34057298222154,48.422453231091,0
-0.34050660330257,48.422537848743,0
-0.34045158972494,48.422625983687,0
-0.34040836022501,48.422716965178,0
-0.34037724386297,48.422810100806,0
-0.34035847751687,48.422904681763,0
-0.34035220407795,48.422999988237,0
-0.34199999999998,48.423,0
</coordinates>
</LinearRing>
</outerBoundaryIs>
</Polygon>
</Placemark>
<Placemark>
<name>575</name>
<styleUrl>#stylehap2</styleUrl>
<Polygon>
<outerBoundaryIs>
<LinearRing>
<coordinates>
-0.327,48.365,0
-0.32535408138063,48.364999988261,0
-0.3253603415297,48.365095294913,0
-0.32537908053961,48.3651898764,0
-0.32541015586298,48.365283012894,0
-0.32545333106138,48.365373995561,0
-0.32550827760296,48.365462131952,0
-0.32557457736096,48.365546751282,0
-0.32565172579442,48.365627209527,0
-0.32573913578681,48.36570289433,0
-0.3258361421135,48.36577322966,0
-0.32594200650387,48.365837680202,0
-0.32605592325984,48.365895755424,0
-0.32617702538803,48.365947013315,0
-0.32630439119843,48.365991063752,0
-0.32643705132009,48.366027571464,0
-0.3265739960798,48.366056258591,0
-0.32671418318778,48.366076906791,0
-0.32685654567193,48.36608935891,0
-0.327,48.366093520174,0
-0.32714345432807,48.36608935891,0
-0.3272858168122,48.366076906791,0
-0.32742600392018,48.366056258591,0

-0.32756294867991,48.366027571464,0
-0.32769560880157,48.365991063752,0
-0.32782297461197,48.365947013315,0
-0.32794407674014,48.365895755424,0
-0.32805799349613,48.365837680202,0
-0.3281638578865,48.36577322966,0
-0.32826086421319,48.36570289433,0
-0.32834827420558,48.365627209527,0
-0.32842542263904,48.365546751282,0
-0.32849172239704,48.365462131952,0
-0.32854666893862,48.365373995561,0
-0.32858984413702,48.365283012894,0
-0.32862091946039,48.3651898764,0
-0.3286396584703,48.365095294913,0
-0.32864591861937,48.364999988261,0
-0.32863965233392,48.364904681787,0
-0.32862090737405,48.36481010083,0
-0.32858982646797,48.3647169652,0
-0.32854664622373,48.364625983708,0
-0.3284916953265,48.364537848763,0
-0.32842539203536,48.364453231109,0
-0.32834824099866,48.364372774719,0
-0.32826082941199,48.364297091893,0
-0.32816382254843,48.364226758601,0
-0.32805795869494,48.364162310097,0
-0.32794404353324,48.364104236852,0
-0.32782294400832,48.364052980816,0
-0.32769558173103,48.364008932055,0
-0.32756292596502,48.363972425789,0
-0.32742598625115,48.363943739837,0
-0.32728580472589,48.363923092501,0
-0.32714344819169,48.363910640912,0
-0.327,48.363906479826,0
-0.32685655180831,48.363910640912,0
-0.32671419527411,48.363923092501,0
-0.32657401374885,48.363943739837,0
-0.32643707403495,48.363972425789,0
-0.32630441826897,48.364008932055,0
-0.32617705599168,48.364052980816,0
-0.32605595646676,48.364104236852,0
-0.32594204130506,48.364162310097,0
-0.32583617745157,48.364226758601,0
-0.32573917058801,48.364297091893,0
-0.32565175900134,48.364372774719,0
-0.32557460796462,48.364453231109,0
-0.3255083046735,48.364537848763,0
-0.32545335377627,48.364625983708,0
-0.325410173532,48.3647169652,0
-0.32537909262595,48.36481010083,0
-0.32536034766608,48.364904681787,0
-0.32535408138063,48.364999988261,0
-0.327,48.365,0
</coordinates>
</LinearRing>
</outerBoundaryIs>
</Polygon>
</Placemark>

<Placemark>
<name>576</name>
<styleUrl>#stylehap2</styleUrl>
<Polygon>
<outerBoundaryIs>
<LinearRing>
<coordinates>
-0.32500000000005,48.265,0
-0.32335730413726,48.264999988302,0
-0.32336355203951,48.265095294954,0
-0.32338225436832,48.26518987644,0
-0.32341326885508,48.265283012933,0
-0.32345635952403,48.265373995597,0
-0.3235111984863,48.265462131986,0
-0.32357736843362,48.265546751313,0
-0.32365436581274,48.265627209555,0
-0.32374160465664,48.265702894354,0
-0.32383842104304,48.265773229681,0
-0.32394407814689,48.265837680219,0
-0.32405777184777,48.265895755437,0
-0.32417863684991,48.265947013325,0
-0.32430575326815,48.265991063759,0
-0.32443815362982,48.266027571469,0
-0.32457483023886,48.266056258594,0
-0.32471474284663,48.266076906792,0
-0.32485682657046,48.26608935891,0
-0.32500000000006,48.266093520174,0
-0.32514317342963,48.26608935891,0
-0.32528525715346,48.266076906792,0
-0.32542516976122,48.266056258594,0
-0.32556184637027,48.266027571469,0
-0.32569424673194,48.265991063759,0
-0.32582136315018,48.265947013325,0
-0.32594222815232,48.265895755437,0
-0.32605592185319,48.265837680219,0
-0.32616157895705,48.265773229681,0
-0.32625839534345,48.265702894354,0
-0.32634563418734,48.265627209555,0
-0.32642263156646,48.265546751313,0
-0.32648880151379,48.265462131986,0
-0.32654364047606,48.265373995597,0
-0.32658673114501,48.265283012933,0
-0.32661774563177,48.26518987644,0
-0.32663644796058,48.265095294954,0
-0.32664269586283,48.264999988302,0
-0.32663644185771,48.264904681828,0
-0.32661773361141,48.26481010087,0
-0.32658671357245,48.264716965239,0
-0.32654361788521,48.264625983744,0
-0.32648877459105,48.264537848797,0
-0.32642260112988,48.26445323114,0
-0.32634560116171,48.264372774747,0
-0.32625836073224,48.264297091917,0
-0.32616154381189,48.264226758621,0
-0.32605588724197,48.264162310114,0
-0.32594219512669,48.264104236866,0
-0.3258213327136,48.264052980826,0

-0.32569421980918,48.264008932062,0
-0.3255618237794,48.263972425794,0
-0.32542515218866,48.263943739839,0
-0.3252852451331,48.263923092502,0
-0.32514316732673,48.263910640912,0
-0.32500000000006,48.263906479826,0
-0.32485683267336,48.263910640912,0
-0.32471475486699,48.263923092502,0
-0.32457484781143,48.263943739839,0
-0.32443817622069,48.263972425794,0
-0.32430578019091,48.264008932062,0
-0.32417866728649,48.264052980826,0
-0.32405780487342,48.264104236866,0
-0.32394411275812,48.264162310114,0
-0.3238384561882,48.264226758621,0
-0.32374163926784,48.264297091917,0
-0.3236543988384,48.264372774747,0
-0.32357739887021,48.26445323114,0
-0.32351122540904,48.264537848797,0
-0.3234563821149,48.264625983744,0
-0.32341328642764,48.264716965239,0
-0.32338226638867,48.26481010087,0
-0.32336355814241,48.264904681828,0
-0.32335730413726,48.264999988302,0
-0.32500000000005,48.265,0
</coordinates>
</LinearRing>
</outerBoundaryIs>
</Polygon>
</Placemark>
<Placemark>
<name>583</name>
<styleUrl>#stylehap2</styleUrl>
<Polygon>
<outerBoundaryIs>
<LinearRing>
<coordinates>
-0.31700000000001,48.21,0
-0.31535906915193,48.209999988325,0
-0.31536531034692,48.210095294976,0
-0.31538399258648,48.210189876462,0
-0.31541497375469,48.210283012954,0
-0.3154580181292,48.210373995617,0
-0.31551279817325,48.210462132005,0
-0.31557889702685,48.21054675133,0
-0.31565581167771,48.21062720957,0
-0.31574295678826,48.210702894367,0
-0.31583966914964,48.210773229692,0
-0.3159452127284,48.210837680228,0
-0.31605878426816,48.210895755444,0
-0.31617951940291,48.210947013331,0
-0.31630649923597,48.210991063763,0
-0.31643875733428,48.211027571472,0
-0.31657528708463,48.211056258595,0
-0.31671504935622,48.211076906793,0
-0.31685698041076,48.21108935891,0
-0.317,48.211093520174,0

-0.31714301958925,48.21108935891,0
-0.31728495064378,48.211076906793,0
-0.31742471291538,48.211056258595,0
-0.31756124266574,48.211027571472,0
-0.31769350076403,48.210991063763,0
-0.31782048059709,48.210947013331,0
-0.31794121573184,48.210895755444,0
-0.3180547872716,48.210837680228,0
-0.31816033085039,48.210773229692,0
-0.31825704321174,48.210702894367,0
-0.31834418832232,48.21062720957,0
-0.31842110297318,48.21054675133,0
-0.31848720182676,48.210462132005,0
-0.31854198187083,48.210373995617,0
-0.31858502624531,48.210283012954,0
-0.31861600741352,48.210189876462,0
-0.3186346896531,48.210095294976,0
-0.3186409308481,48.209999988325,0
-0.31863468356853,48.209904681851,0
-0.31861599542927,48.209810100891,0
-0.31858500872549,48.20971696526,0
-0.3185419593478,48.209625983764,0
-0.31848717498486,48.209537848815,0
-0.31842107262796,48.209453231157,0
-0.31834415539584,48.209372774762,0
-0.31825700870447,48.20929709193,0
-0.31816029581075,48.209226758632,0
-0.31805475276431,48.209162310124,0
-0.31794118280539,48.209104236873,0
-0.3178204502519,48.209052980831,0
-0.31769347392213,48.209008932066,0
-0.31756122014271,48.208972425797,0
-0.31742469539558,48.208943739841,0
-0.31728493865953,48.208923092503,0
-0.31714301350469,48.208910640912,0
-0.317,48.208906479826,0
-0.31685698649533,48.208910640912,0
-0.31671506134048,48.208923092503,0
-0.31657530460444,48.208943739841,0
-0.31643877985732,48.208972425797,0
-0.3163065260779,48.209008932066,0
-0.31617954974813,48.209052980831,0
-0.31605881719464,48.209104236873,0
-0.31594524723569,48.209162310124,0
-0.31583970418925,48.209226758632,0
-0.31574299129555,48.20929709193,0
-0.31565584460416,48.209372774762,0
-0.31557892737204,48.209453231157,0
-0.31551282501514,48.209537848815,0
-0.31545804065223,48.209625983764,0
-0.31541499127451,48.20971696526,0
-0.31538400457073,48.209810100891,0
-0.31536531643148,48.209904681851,0
-0.31535906915193,48.209999988325,0
-0.31700000000001,48.21,0
</coordinates>
</LinearRing>

</outerBoundaryIs>
</Polygon>
</Placemark>
<Placemark>
<name>585</name>
<styleUrl>#stylehap2</styleUrl>
<Polygon>
<outerBoundaryIs>
<LinearRing>
<coordinates>
-0.31600000000003,48.437,0
-0.31435175004101,48.436999988231,0
-0.3143580190494,48.437095294884,0
-0.3143767845944,48.437189876371,0
-0.3144079039269,48.437283012867,0
-0.31445114027386,48.437373995534,0
-0.31450616463819,48.437462131928,0
-0.31457255830113,48.43754675126,0
-0.31464981600715,48.437627209507,0
-0.31473734980825,48.437702894312,0
-0.3148344935376,48.437773229646,0
-0.31494050787883,48.43783768019,0
-0.31505458599274,48.437895755414,0
-0.31517585965773,48.437947013308,0
-0.31530340587822,48.437991063747,0
-0.31543625391009,48.438027571461,0
-0.31557339264981,48.438056258589,0
-0.31571377833104,48.43807690679,0
-0.31585634247018,48.43808935891,0
-0.31600000000004,48.438093520174,0
-0.31614365752988,48.43808935891,0
-0.31628622166901,48.43807690679,0
-0.31642660735027,48.438056258589,0
-0.31656374608997,48.438027571461,0
-0.31669659412183,48.437991063747,0
-0.31682414034235,48.437947013308,0
-0.31694541400732,48.437895755414,0
-0.31705949212123,48.43783768019,0
-0.31716550646249,48.437773229646,0
-0.3172626501918,48.437702894312,0
-0.31735018399291,48.437627209507,0
-0.31742744169893,48.43754675126,0
-0.31749383536186,48.437462131928,0
-0.31754885972622,48.437373995534,0
-0.31759209607316,48.437283012867,0
-0.31762321540568,48.437189876371,0
-0.31764198095069,48.437095294884,0
-0.31764824995905,48.436999988231,0
-0.31764197479003,48.436904681758,0
-0.31762320327156,48.436810100801,0
-0.31759207833426,48.436716965173,0
-0.31754883692152,48.436625983682,0
-0.31749380818428,48.436537848738,0
-0.31742741097426,48.436453231087,0
-0.31735015065467,48.436372774699,0
-0.31726261525298,48.436297091875,0
-0.31716547098467,48.436226758586,0

-0.3170594571824,48.436162310085,0
-0.3169453806691,48.436104236843,0
-0.31682410961766,48.436052980808,0
-0.31669656694427,48.43600893205,0
-0.31656372328526,48.435972425786,0
-0.31642658961135,48.435943739835,0
-0.31628620953489,48.4359230925,0
-0.31614365136922,48.435910640912,0
-0.31600000000004,48.435906479826,0
-0.31585634863083,48.435910640912,0
-0.31571379046516,48.4359230925,0
-0.31557341038871,48.435943739835,0
-0.3154362767148,48.435972425786,0
-0.31530343305581,48.43600893205,0
-0.3151758903824,48.436052980808,0
-0.31505461933096,48.436104236843,0
-0.31494054281766,48.436162310085,0
-0.31483452901539,48.436226758586,0
-0.31473738474708,48.436297091875,0
-0.31464984934539,48.436372774699,0
-0.31457258902582,48.436453231087,0
-0.31450619181578,48.436537848738,0
-0.31445116307854,48.436625983682,0
-0.31440792166579,48.436716965173,0
-0.31437679672852,48.436810100801,0
-0.31435802521006,48.436904681758,0
-0.31435175004101,48.436999988231,0
-0.31600000000003,48.437,0
</coordinates>
</LinearRing>
</outerBoundaryIs>
</Polygon>
</Placemark>
<Placemark>
<name>586</name>
<styleUrl>#stylehap2</styleUrl>
<Polygon>
<outerBoundaryIs>
<LinearRing>
<coordinates>
-0.31399999999996,48.337,0
-0.31235498553164,48.336999988273,0
-0.31236124224481,48.337095294925,0
-0.3123799709638,48.337189876411,0
-0.31241102921932,48.337283012905,0
-0.31245418070282,48.337373995571,0
-0.3125090970628,48.337462131962,0
-0.31257536040215,48.337546751291,0
-0.312652466457,48.337627209535,0
-0.31273982843332,48.337702894336,0
-0.31283678147188,48.337773229666,0
-0.31294258770759,48.337837680207,0
-0.313056441885,48.337895755427,0
-0.31317747748695,48.337947013318,0
-0.31330477332988,48.337991063754,0
-0.31343736057535,48.338027571466,0
-0.31357423010483,48.338056258592,0

-0.3137143402012,48.338076906791,0
-0.31385662447854,48.33808935891,0
-0.31399999999997,48.338093520174,0
-0.31414337552138,48.33808935891,0
-0.31428565979874,48.338076906791,0
-0.31442576989511,48.338056258592,0
-0.3145626394246,48.338027571466,0
-0.31469522667006,48.337991063754,0
-0.31482252251296,48.337947013318,0
-0.31494355811494,48.337895755427,0
-0.31505741229235,48.337837680207,0
-0.31516321852806,48.337773229666,0
-0.31526017156659,48.337702894336,0
-0.31534753354294,48.337627209535,0
-0.31542463959777,48.337546751291,0
-0.31549090293712,48.337462131962,0
-0.31554581929712,48.337373995571,0
-0.31558897078059,48.337283012905,0
-0.31562002903614,48.337189876411,0
-0.3156387577551,48.337095294925,0
-0.3156450144683,48.336999988273,0
-0.31563875162811,48.336904681799,0
-0.31562001696832,48.336810100841,0
-0.31558895313864,48.336716965211,0
-0.31554579661704,48.336625983718,0
-0.31549087590808,48.336537848772,0
-0.31542460904101,48.336453231118,0
-0.3153475003869,48.336372774727,0
-0.31526013681873,48.3362970919,0
-0.31516318324414,48.336226758606,0
-0.31505737754449,48.336162310102,0
-0.31494352495891,48.336104236856,0
-0.3148224919562,48.336052980818,0
-0.314695199641,48.336008932057,0
-0.31456261674454,48.33597242579,0
-0.31442575225316,48.335943739837,0
-0.31428564773093,48.335923092501,0
-0.31414336939441,48.335910640912,0
-0.31399999999997,48.335906479826,0
-0.31385663060553,48.335910640912,0
-0.31371435226901,48.335923092501,0
-0.31357424774678,48.335943739837,0
-0.3134373832554,48.33597242579,0
-0.31330480035892,48.336008932057,0
-0.31317750804374,48.336052980818,0
-0.31305647504104,48.336104236856,0
-0.31294262245545,48.336162310102,0
-0.31283681675578,48.336226758606,0
-0.31273986318119,48.3362970919,0
-0.31265249961304,48.336372774727,0
-0.31257539095891,48.336453231118,0
-0.31250912409186,48.336537848772,0
-0.31245420338287,48.336625983718,0
-0.31241104686127,48.336716965211,0
-0.31237998303159,48.336810100841,0
-0.31236124837181,48.336904681799,0
-0.31235498553164,48.336999988273,0

-0.31399999999996,48.337,0
</coordinates>
</LinearRing>
</outerBoundaryIs>
</Polygon>
</Placemark>
<Placemark>
<name>594</name>
<styleUrl>#stylehap2</styleUrl>
<Polygon>
<outerBoundaryIs>
<LinearRing>
<coordinates>
-0.28600000000006,48.388,0
-0.28435333764699,48.387999988252,0
-0.28435960062232,48.388095294904,0
-0.28437834809735,48.388189876391,0
-0.28440943746032,48.388283012886,0
-0.28445263216606,48.388373995552,0
-0.28450760353434,48.388462131945,0
-0.28457393324947,48.388546751275,0
-0.28465111654257,48.388627209521,0
-0.28473856603188,48.388702894324,0
-0.28483561619213,48.388773229656,0
-0.28494152841916,48.388837680198,0
-0.28505549665086,48.38889575542,0
-0.28517665350195,48.388947013313,0
-0.28530407686598,48.38899106375,0
-0.28543679693393,48.389027571463,0
-0.28557380357628,48.38905625859,0
-0.28571405403224,48.389076906791,0
-0.28585648084746,48.38908935891,0
-0.28600000000007,48.389093520174,0
-0.28614351915265,48.38908935891,0
-0.2862859459679,48.389076906791,0
-0.28642619642383,48.38905625859,0
-0.28656320306618,48.389027571463,0
-0.28669592313413,48.38899106375,0
-0.28682334649817,48.388947013313,0
-0.28694450334928,48.38889575542,0
-0.28705847158095,48.388837680198,0
-0.28716438380798,48.388773229656,0
-0.28726143396823,48.388702894324,0
-0.28734888345754,48.388627209521,0
-0.28742606675066,48.388546751275,0
-0.2874923964658,48.388462131945,0
-0.28754736783405,48.388373995552,0
-0.28759056253979,48.388283012886,0
-0.28762165190276,48.388189876391,0
-0.28764039937779,48.388095294904,0
-0.28764666235312,48.387999988252,0
-0.28764039323367,48.387904681778,0
-0.28762163980121,48.38781010082,0
-0.28759054484848,48.387716965191,0
-0.28754734509054,48.387625983699,0
-0.28749236936111,48.387537848755,0
-0.28742603610841,48.387453231102,0

-0.28734885020876,48.387372774713,0
-0.28726139912317,48.387297091887,0
-0.28716434842535,48.387226758596,0
-0.28705843673586,48.387162310094,0
-0.28694447010047,48.387104236849,0
-0.28682331585591,48.387052980813,0
-0.28669589602947,48.387008932053,0
-0.28656318032267,48.386972425788,0
-0.28642617873251,48.386943739836,0
-0.28628593386632,48.386923092501,0
-0.28614351300853,48.386910640912,0
-0.28600000000007,48.386906479826,0
-0.28585648699158,48.386910640912,0
-0.28571406613379,48.386923092501,0
-0.2855738212676,48.386943739836,0
-0.28543681967744,48.386972425788,0
-0.28530410397064,48.387008932053,0
-0.2851766841442,48.387052980813,0
-0.28505552989964,48.387104236849,0
-0.28494156326425,48.387162310094,0
-0.28483565157476,48.387226758596,0
-0.28473860087697,48.387297091887,0
-0.28465114979135,48.387372774713,0
-0.28457396389173,48.387453231102,0
-0.284507630639,48.387537848755,0
-0.28445265490957,48.387625983699,0
-0.28440945515164,48.387716965191,0
-0.28437836019893,48.38781010082,0
-0.28435960676644,48.387904681778,0
-0.28435333764699,48.387999988252,0
-0.28600000000006,48.388,0
</coordinates>
</LinearRing>
</outerBoundaryIs>
</Polygon>
</Placemark>
<Placemark>
<name>596</name>
<styleUrl>#stylehap2</styleUrl>
<Polygon>
<outerBoundaryIs>
<LinearRing>
<coordinates>
-0.28200000000004,48.429,0
-0.28035200953342,48.428999988235,0
-0.28035827755568,48.429095294887,0
-0.28037704014717,48.429189876375,0
-0.28040815458121,48.42928301287,0
-0.28045138412193,48.429373995537,0
-0.28050639982414,48.429462131931,0
-0.28057278303488,48.429546751263,0
-0.28065002857821,48.429627209509,0
-0.28073754859863,48.429702894314,0
-0.28083467703422,48.429773229647,0
-0.28094067468499,48.429837680191,0
-0.28105473483877,48.429895755415,0
-0.28117598941066,48.429947013308,0

-0.28130351555043,48.429991063747,0
-0.28143634266674,48.430027571461,0
-0.28157345981528,48.430056258589,0
-0.2817138233941,48.43007690679,0
-0.2818563650878,48.43008935891,0
-0.28200000000003,48.430093520174,0
-0.28214363491228,48.43008935891,0
-0.28228617660599,48.43007690679,0
-0.2824265401848,48.430056258589,0
-0.28256365733334,48.430027571461,0
-0.28269648444965,48.429991063747,0
-0.28282401058942,48.429947013308,0
-0.2829452651 6129,48.429895755415,0
-0.28305932531506,48.429837680191,0
-0.28316532296587,48.429773229647,0
-0.28326245140145,48.429702894314,0
-0.28334997142187,48.429627209509,0
-0.2834272169652,48.429546751263,0
-0.28349360017594,48.429462131931,0
-0.28354861587812,48.429373995537,0
-0.28359184541887,48.42928301287,0
-0.28362295985291,48.429189876375,0
-0.2836417224444,48.429095294887,0
-0.28364799046666,48.428999988235,0
-0.28364171628644,48.428904681761,0
-0.28362294772411,48.428810100804,0
-0.28359182768776,48.428716965176,0
-0.28354859308344,48.428625983684,0
-0.28349357301029,48.428537848741,0
-0.28342718625399,48.428453231089,0
-0.28334993809826,48.428372774701,0
-0.28326241647794,48.428297091877,0
-0.28316528750362,48.428226758587,0
-0.28305929039158,48.428162310087,0
-0.2829452318377,48.428104236844,0
-0.28282397987821,48.428052980809,0
-0.282696457284,48.42800893205,0
-0.28256363453866,48.427972425786,0
-0.2824265224537,48.427943739835,0
-0.28228616447718,48.4279230925,0
-0.28214362875432,48.427910640912,0
-0.28200000000003,48.427906479826,0
-0.28185637124576,48.427910640912,0
-0.2817138355229,48.4279230925,0
-0.28157347754639,48.427943739835,0
-0.28143636546142,48.427972425786,0
-0.28130354271608,48.42800893205,0
-0.28117602012187,48.428052980809,0
-0.28105476816238,48.428104236844,0
-0.2809407096085,48.428162310087,0
-0.28083471249643,48.428226758587,0
-0.28073758352211,48.428297091877,0
-0.2806500619018,48.428372774701,0
-0.28057281374609,48.428453231089,0
-0.2805064269898,48.428537848741,0
-0.28045140691664,48.428625983684,0
-0.28040817231232,48.428716965176,0

-0.28037705227598,48.428810100804,0
-0.28035828371364,48.428904681761,0
-0.28035200953342,48.428999988235,0
-0.28200000000004,48.429,0
</coordinates>
</LinearRing>
</outerBoundaryIs>
</Polygon>
</Placemark>
<Placemark>
<name>597</name>
<styleUrl>#stylehap2</styleUrl>
<Polygon>
<outerBoundaryIs>
<LinearRing>
<coordinates>
-0.28200000000004,48.389,0
-0.28035330528946,48.388999988251,0
-0.28035956838774,48.389095294903,0
-0.28037831623108,48.389189876391,0
-0.28040940620487,48.389283012885,0
-0.28045260175931,48.389373995552,0
-0.2805075742077,48.389462131944,0
-0.28057390522618,48.389546751275,0
-0.28065109003592,48.38962720952,0
-0.28073854124362,48.389702894324,0
-0.28083559331093,48.389773229655,0
-0.28094150761919,48.389837680198,0
-0.2810554780904,48.38989575542,0
-0.28117663732232,48.389947013313,0
-0.28130406319034,48.38999106375,0
-0.28143678586637,48.390027571463,0
-0.28157379520103,48.39005625859,0
-0.28171404841305,48.390076906791,0
-0.28185647802713,48.39008935891,0
-0.28200000000003,48.390093520174,0
-0.28214352197295,48.39008935891,0
-0.28228595158704,48.390076906791,0
-0.28242620479903,48.39005625859,0
-0.28256321413371,48.390027571463,0
-0.28269593680974,48.38999106375,0
-0.28282336267776,48.389947013313,0
-0.28294452190966,48.38989575542,0
-0.28305849238089,48.389837680198,0
-0.28316440668916,48.389773229655,0
-0.28326145875647,48.389702894324,0
-0.28334890996416,48.38962720952,0
-0.28342609477391,48.389546751275,0
-0.28349242579238,48.389462131944,0
-0.28354739824078,48.389373995552,0
-0.28359059379521,48.389283012885,0
-0.283621683769,48.389189876391,0
-0.28364043161234,48.389095294903,0
-0.28364669471062,48.388999988251,0
-0.28364042546786,48.388904681777,0
-0.28362167166679,48.38881010082,0
-0.28359057610293,48.388716965191,0

-0.28354737549602,48.388625983699,0
-0.28349239868622,48.388537848755,0
-0.28342606412997,48.388453231102,0
-0.28334887671355,48.388372774712,0
-0.28326142390947,48.388297091887,0
-0.28316437130457,48.388226758596,0
-0.2830584575339,48.388162310093,0
-0.28294448865904,48.388104236849,0
-0.28282333203383,48.388052980813,0
-0.28269590970358,48.388008932053,0
-0.28256319138895,48.387972425788,0
-0.28242618710675,48.387943739836,0
-0.2822859394848,48.387923092501,0
-0.2821435158285,48.387910640912,0
-0.28200000000003,48.387906479826,0
-0.28185648417158,48.387910640912,0
-0.28171406051529,48.387923092501,0
-0.28157381289331,48.387943739836,0
-0.28143680861113,48.387972425788,0
-0.28130409029648,48.388008932053,0
-0.28117666796625,48.388052980813,0
-0.28105551134104,48.388104236849,0
-0.28094154246618,48.388162310093,0
-0.28083562869549,48.388226758596,0
-0.28073857609061,48.388297091887,0
-0.28065112328653,48.388372774712,0
-0.28057393587011,48.388453231102,0
-0.28050760131384,48.388537848755,0
-0.28045262450406,48.388625983699,0
-0.28040942389715,48.388716965191,0
-0.28037832833329,48.38881010082,0
-0.28035957453222,48.388904681777,0
-0.28035330528946,48.388999988251,0
-0.28200000000004,48.389,0
</coordinates>
</LinearRing>
</outerBoundaryIs>
</Polygon>
</Placemark>
<Placemark>
<name>598</name>
<styleUrl>#stylehap2</styleUrl>
<Polygon>
<outerBoundaryIs>
<LinearRing>
<coordinates>
-0.28099999999995,48.429,0
-0.27935200953331,48.428999988235,0
-0.2793582775556,48.429095294887,0
-0.27937704014709,48.429189876375,0
-0.27940815458112,48.42928301287,0
-0.27945138412185,48.429373995537,0
-0.27950639982406,48.429462131931,0
-0.2795727830348,48.429546751263,0
-0.27965002857812,48.429627209509,0
-0.27973754859855,48.429702894314,0
-0.2798346770341,48.429773229647,0

-0.27994067468491,48.429837680191,0
-0.28005473483868,48.429895755415,0
-0.28017598941058,48.429947013308,0
-0.28030351555032,48.429991063747,0
-0.28043634266665,48.430027571461,0
-0.28057345981519,48.430056258589,0
-0.28071382339401,48.43007690679,0
-0.28085636508771,48.43008935891,0
-0.28099999999994,48.430093520174,0
-0.2811436349122,48.43008935891,0
-0.2812861766059,48.43007690679,0
-0.28142654018472,48.430056258589,0
-0.28156365733326,48.430027571461,0
-0.28169648444957,48.429991063747,0
-0.28182401058933,48.429947013308,0
-0.2819452651612,48.429895755415,0
-0.28205932531498,48.429837680191,0
-0.28216532296578,48.429773229647,0
-0.28226245140136,48.429702894314,0
-0.28234997142179,48.429627209509,0
-0.28242721696511,48.429546751263,0
-0.28249360017585,48.429462131931,0
-0.28254861587804,48.429373995537,0
-0.28259184541879,48.42928301287,0
-0.28262295985282,48.429189876375,0
-0.28264172244429,48.429095294887,0
-0.28264799046658,48.428999988235,0
-0.28264171628635,48.428904681761,0
-0.28262294772402,48.428810100804,0
-0.28259182768765,48.428716965176,0
-0.28254859308336,48.428625983684,0
-0.2824935730102,48.428537848741,0
-0.2824271862539,48.428453231089,0
-0.28234993809817,48.428372774701,0
-0.28226241647786,48.428297091877,0
-0.28216528750354,48.428226758587,0
-0.28205929039149,48.428162310087,0
-0.28194523183762,48.428104236844,0
-0.28182397987813,48.428052980809,0
-0.28169645728392,48.42800893205,0
-0.28156363453858,48.427972425786,0
-0.28142652245358,48.427943739835,0
-0.2812861644771,48.4279230925,0
-0.28114362875424,48.427910640912,0
-0.28099999999994,48.427906479826,0
-0.28085637124565,48.427910640912,0
-0.28071383552281,48.4279230925,0
-0.2805734775463,48.427943739835,0
-0.28043636546133,48.427972425786,0
-0.28030354271597,48.42800893205,0
-0.28017602012178,48.428052980809,0
-0.28005476816229,48.428104236844,0
-0.27994070960841,48.428162310087,0
-0.27983471249635,48.428226758587,0
-0.27973758352203,48.428297091877,0
-0.27965006190171,48.428372774701,0
-0.27957281374598,48.428453231089,0

-0.27950642698971,48.428537848741,0
-0.27945140691655,48.428625983684,0
-0.27940817231223,48.428716965176,0
-0.27937705227589,48.428810100804,0
-0.27935828371356,48.428904681761,0
-0.27935200953331,48.428999988235,0
-0.28099999999995,48.429,0
</coordinates>
</LinearRing>
</outerBoundaryIs>
</Polygon>
</Placemark>
<Placemark>
<name>600</name>
<styleUrl>#stylehap2</styleUrl>
<Polygon>
<outerBoundaryIs>
<LinearRing>
<coordinates>
-0.27300000000002,48.449,0
-0.27135136058888,48.448999988226,0
-0.27135763107722,48.449095294879,0
-0.27137640105492,48.449189876367,0
-0.27140752773918,48.449283012862,0
-0.27145077430104,48.44937399553,0
-0.27150581166575,48.449462131924,0
-0.27157222101556,48.449546751256,0
-0.27164949697568,48.449627209504,0
-0.27173705145912,48.449702894309,0
-0.27183421814166,48.449773229643,0
-0.27194025753233,48.449837680188,0
-0.27205436260119,48.449895755412,0
-0.27217566492155,48.449947013306,0
-0.27230324127968,48.449991063746,0
-0.27243612070212,48.45002757146,0
-0.27257329184633,48.450056258588,0
-0.27271371069939,48.45007690679,0
-0.27285630852516,48.45008935891,0
-0.27300000000001,48.450093520174,0
-0.2731436914749,48.45008935891,0
-0.27328628930066,48.45007690679,0
-0.27342670815372,48.450056258588,0
-0.27356387929793,48.45002757146,0
-0.27369675872034,48.449991063746,0
-0.2738243350785,48.449947013306,0
-0.27394563739884,48.449895755412,0
-0.27405974246772,48.449837680188,0
-0.27416578185839,48.449773229643,0
-0.27426294854094,48.449702894309,0
-0.27435050302437,48.449627209504,0
-0.27442777898446,48.449546751256,0
-0.2744941883343,48.449462131924,0
-0.27454922569901,48.44937399553,0
-0.27459247226084,48.449283012862,0
-0.27462359894513,48.449189876367,0
-0.27464236892283,48.449095294879,0
-0.27464863941117,48.448999988226,0

-0.27464236275813,48.448904681753,0
-0.27462358680302,48.448810100796,0
-0.27459245451027,48.448716965168,0
-0.27454920287929,48.448625983677,0
-0.27449416113883,48.448537848734,0
-0.27442774823957,48.448453231083,0
-0.2743504696642,48.448372774696,0
-0.27426291357911,48.448297091873,0
-0.27416574635722,48.448226758583,0
-0.27405970750589,48.448162310083,0
-0.27394560403867,48.448104236841,0
-0.2738243043336,48.448052980807,0
-0.2736967315249,48.448008932049,0
-0.27356385647824,48.447972425785,0
-0.27342669040315,48.447943739834,0
-0.27328627715855,48.4479230925,0
-0.27314368531017,48.447910640911,0
-0.27300000000001,48.447906479826,0
-0.27285631468989,48.447910640911,0
-0.27271372284151,48.4479230925,0
-0.2725733095969,48.447943739834,0
-0.27243614352181,48.447972425785,0
-0.27230326847516,48.448008932049,0
-0.27217569566645,48.448052980807,0
-0.27205439596136,48.448104236841,0
-0.27194029249416,48.448162310083,0
-0.27183425364283,48.448226758583,0
-0.27173708642094,48.448297091873,0
-0.27164953033585,48.448372774696,0
-0.27157225176049,48.448453231083,0
-0.27150583886123,48.448537848734,0
-0.27145079712076,48.448625983677,0
-0.27140754548978,48.448716965168,0
-0.27137641319703,48.448810100796,0
-0.27135763724192,48.448904681753,0
-0.27135136058888,48.448999988226,0
-0.27300000000002,48.449,0
</coordinates>
</LinearRing>
</outerBoundaryIs>
</Polygon>
</Placemark>
<Placemark>
<name>601</name>
<styleUrl>#stylehap2</styleUrl>
<Polygon>
<outerBoundaryIs>
<LinearRing>
<coordinates>
-0.27199999999993,48.448,0
-0.27035139305292,48.447999988227,0
-0.2703576634179,48.448095294879,0
-0.2703764330261,48.448189876367,0
-0.27040755909753,48.448283012862,0
-0.27045080480789,48.44837399553,0
-0.2705058410889,48.448462131924,0
-0.2705722491311,48.448546751257,0

-0.27064952356956,48.448627209504,0
-0.27073707632896,48.448702894309,0
-0.27083424109815,48.448773229643,0
-0.27094027840076,48.448837680188,0
-0.27105438122269,48.448895755412,0
-0.27117568115436,48.448947013306,0
-0.27130325500028,48.448991063746,0
-0.27143613180604,48.44902757146,0
-0.27157330024906,48.449056258588,0
-0.27171371633698,48.44907690679,0
-0.27185631135467,48.44908935891,0
-0.27199999999993,48.449093520173,0
-0.27214368864518,48.44908935891,0
-0.2722862836629,48.44907690679,0
-0.27242669975082,48.449056258588,0
-0.27256386819384,48.44902757146,0
-0.27269674499957,48.448991063746,0
-0.2728243188455,48.448947013306,0
-0.27294561877719,48.448895755412,0
-0.27305972159912,48.448837680188,0
-0.27316575890172,48.448773229643,0
-0.27326292367092,48.448702894309,0
-0.2733504764303,48.448627209504,0
-0.27342775086878,48.448546751257,0
-0.27349415891095,48.448462131924,0
-0.27354919519199,48.44837399553,0
-0.27359244090232,48.448283012862,0
-0.27362356697378,48.448189876367,0
-0.27364233658198,48.448095294879,0
-0.27364860694694,48.447999988227,0
-0.2736423304176,48.447904681753,0
-0.27362355483233,48.447810100796,0
-0.27359242315271,48.447716965168,0
-0.27354917237352,48.447625983678,0
-0.27349413171698,48.447537848735,0
-0.27342772012554,48.447453231083,0
-0.27335044307195,48.447372774696,0
-0.273262888711,48.447297091873,0
-0.27316572340251,48.447226758583,0
-0.27305968663923,48.447162310083,0
-0.27294558541885,48.447104236841,0
-0.27282428810228,48.447052980807,0
-0.2726967178056,48.447008932049,0
-0.27256384537537,48.446972425785,0
-0.27242668200121,48.446943739834,0
-0.27228627152145,48.4469230925,0
-0.27214368248081,48.446910640911,0
-0.27199999999993,48.446906479826,0
-0.27185631751904,48.446910640911,0
-0.27171372847841,48.4469230925,0
-0.27157331799867,48.446943739834,0
-0.27143615462448,48.446972425785,0
-0.27130328219425,48.447008932049,0
-0.27117571189757,48.447052980807,0
-0.27105441458103,48.447104236841,0
-0.27094031336065,48.447162310083,0
-0.27083427659737,48.447226758583,0

-0.27073711128885,48.447297091873,0
-0.2706495569279,48.447372774696,0
-0.27057227987431,48.447453231083,0
-0.2705058682829,48.447537848735,0
-0.27045082762634,48.447625983678,0
-0.27040757684714,48.447716965168,0
-0.27037644516753,48.447810100796,0
-0.27035766958227,48.447904681753,0
-0.27035139305292,48.447999988227,0
-0.27199999999993,48.448,0
</coordinates>
</LinearRing>
</outerBoundaryIs>
</Polygon>
</Placemark>
<Placemark>
<name>602</name>
<styleUrl>#stylehap2</styleUrl>
<Polygon>
<outerBoundaryIs>
<LinearRing>
<coordinates>
-0.27099999999996,48.448,0
-0.26935139305296,48.447999988227,0
-0.26935766341792,48.448095294879,0
-0.26937643302612,48.448189876367,0
-0.26940755909755,48.448283012862,0
-0.26945080480791,48.44837399553,0
-0.26950584108894,48.448462131924,0
-0.26957224913111,48.448546751257,0
-0.26964952356957,48.448627209504,0
-0.26973707632897,48.448702894309,0
-0.26983424109817,48.448773229643,0
-0.26994027840077,48.448837680188,0
-0.2700543812227,48.448895755412,0
-0.2701756811544,48.448947013306,0
-0.27030325500032,48.448991063746,0
-0.27043613180606,48.44902757146,0
-0.27057330024908,48.449056258588,0
-0.270713716337,48.44907690679,0
-0.27085631135471,48.44908935891,0
-0.27099999999997,48.449093520173,0
-0.27114368864523,48.44908935891,0
-0.27128628366291,48.44907690679,0
-0.27142669975083,48.449056258588,0
-0.27156386819385,48.44902757146,0
-0.27169674499961,48.448991063746,0
-0.27182431884554,48.448947013306,0
-0.27194561877721,48.448895755412,0
-0.27205972159914,48.448837680188,0
-0.27216575890174,48.448773229643,0
-0.27226292367094,48.448702894309,0
-0.27235047643034,48.448627209504,0
-0.2724277508688,48.448546751257,0
-0.27249415891097,48.448462131924,0
-0.272549195192,48.44837399553,0
-0.27259244090236,48.448283012862,0

-0.27262356697379,48.448189876367,0
-0.27264233658199,48.448095294879,0
-0.27264860694698,48.447999988227,0
-0.27264233041762,48.447904681753,0
-0.27262355483234,48.447810100796,0
-0.27259242315275,48.447716965168,0
-0.27254917237356,48.447625983678,0
-0.272494131717,48.447537848735,0
-0.27242772012558,48.447453231083,0
-0.27235044307199,48.447372774696,0
-0.27226288871104,48.447297091873,0
-0.27216572340253,48.447226758583,0
-0.27205968663925,48.447162310083,0
-0.27194558541886,48.447104236841,0
-0.2718242881023,48.447052980807,0
-0.27169671780564,48.447008932049,0
-0.27156384537541,48.446972425785,0
-0.27142668200122,48.446943739834,0
-0.27128627152149,48.4469230925,0
-0.27114368248085,48.446910640911,0
-0.27099999999997,48.446906479826,0
-0.27085631751908,48.446910640911,0
-0.27071372847845,48.4469230925,0
-0.27057331799869,48.446943739834,0
-0.27043615462453,48.446972425785,0
-0.27030328219429,48.447008932049,0
-0.27017571189761,48.447052980807,0
-0.27005441458105,48.447104236841,0
-0.26994031336066,48.447162310083,0
-0.26983427659738,48.447226758583,0
-0.26973711128889,48.447297091873,0
-0.26964955692794,48.447372774696,0
-0.26957227987433,48.447453231083,0
-0.26950586828291,48.447537848735,0
-0.26945082762638,48.447625983678,0
-0.26940757684716,48.447716965168,0
-0.26937644516757,48.447810100796,0
-0.26935766958229,48.447904681753,0
-0.26935139305296,48.447999988227,0
-0.27099999999996,48.448,0
</coordinates>
</LinearRing>
</outerBoundaryIs>
</Polygon>
</Placemark>
<Placemark>
<name>607</name>
<styleUrl>#stylehap2</styleUrl>
<Polygon>
<outerBoundaryIs>
<LinearRing>
<coordinates>
-0.26099999999997,48.433,0
-0.25935187980139,48.432999988233,0
-0.25935814831666,48.433095294885,0
-0.25937691238474,48.433189876373,0
-0.25940802926773,48.433283012868,0

-0.25945126221121,48.433373995536,0
-0.25950628224402,48.433462131929,0
-0.25957267068025,48.433546751261,0
-0.25964992230426,48.433627209508,0
-0.25973744921428,48.433702894313,0
-0.25983458529589,48.433773229646,0
-0.25994059129099,48.43383768019,0
-0.26005466042385,48.433895755414,0
-0.26017592454126,48.433947013308,0
-0.26030346072028,48.433991063747,0
-0.26043629829321,48.434027571461,0
-0.26057342623614,48.434056258589,0
-0.26071380086497,48.43407690679,0
-0.26085635378016,48.43408935891,0
-0.26099999999997,48.434093520174,0
-0.26114364621978,48.43408935891,0
-0.26128619913497,48.43407690679,0
-0.26142657376377,48.434056258589,0
-0.26156370170673,48.434027571461,0
-0.26169653927966,48.433991063747,0
-0.26182407545868,48.433947013308,0
-0.26194533957608,48.433895755414,0
-0.26205940870894,48.43383768019,0
-0.26216541470405,48.433773229646,0
-0.26226255078566,48.433702894313,0
-0.26235007769567,48.433627209508,0
-0.26242732931968,48.433546751261,0
-0.26249371775592,48.433462131929,0
-0.26254873778872,48.433373995536,0
-0.26259197073221,48.433283012868,0
-0.2626230876152,48.433189876373,0
-0.26264185168328,48.433095294885,0
-0.26264812019855,48.432999988233,0
-0.26264184552397,48.432904681759,0
-0.26262307548375,48.432810100802,0
-0.26259195299718,48.432716965174,0
-0.26254871498903,48.432625983683,0
-0.26249369058432,48.43253784874,0
-0.26242729860173,48.432453231088,0
-0.26235004436476,48.4323727747,0
-0.26226251585449,48.432297091876,0
-0.26216537923402,48.432226758587,0
-0.26205937377778,48.432162310086,0
-0.26194530624517,48.432104236843,0
-0.26182404474076,48.432052980808,0
-0.26169651210805,48.43200893205,0
-0.26156367890704,48.431972425786,0
-0.26142655602877,48.431943739835,0
-0.26128618700349,48.4319230925,0
-0.26114364006047,48.431910640912,0
-0.26099999999997,48.431906479826,0
-0.26085635993946,48.431910640912,0
-0.26071381299642,48.4319230925,0
-0.26057344397117,48.431943739835,0
-0.2604363210929,48.431972425786,0
-0.26030348789188,48.43200893205,0
-0.26017595525918,48.432052980808,0

-0.26005469375477,48.432104236843,0
-0.25994062622216,48.432162310086,0
-0.25983462076592,48.432226758587,0
-0.25973748414542,48.432297091876,0
-0.25964995563518,48.4323727747,0
-0.2595727013982,48.432453231088,0
-0.25950630941562,48.43253784874,0
-0.25945128501088,48.432625983683,0
-0.25940804700276,48.432716965174,0
-0.25937692451619,48.432810100802,0
-0.25935815447594,48.432904681759,0
-0.25935187980139,48.432999988233,0
-0.26099999999997,48.433,0
</coordinates>
</LinearRing>
</outerBoundaryIs>
</Polygon>
</Placemark>
<Placemark>
<name>616</name>
<styleUrl>#stylehap2</styleUrl>
<Polygon>
<outerBoundaryIs>
<LinearRing>
<coordinates>
-0.25199999999995,48.441,0
-0.25035162025203,48.44099998823,0
-0.25035788975363,48.441095294882,0
-0.25037665677586,48.44118987637,0
-0.25040777855843,48.441283012865,0
-0.2504510183096,48.441373995533,0
-0.25050604700646,48.441462131927,0
-0.25057244589721,48.441546751259,0
-0.25064970968659,48.441627209506,0
-0.25073725038029,48.441702894311,0
-0.25083440175902,48.441773229645,0
-0.25094042444824,48.441837680189,0
-0.25105451154515,48.441895755413,0
-0.25117579475983,48.441947013307,0
-0.251303351024,48.441991063746,0
-0.25143620951709,48.442027571461,0
-0.25157335905594,48.442056258589,0
-0.25171375579202,48.44207690679,0
-0.25185633115759,48.44208935891,0
-0.25199999999995,48.442093520174,0
-0.25214366884231,48.44208935891,0
-0.25228624420789,48.44207690679,0
-0.25242664094396,48.442056258589,0
-0.25256379048281,48.442027571461,0
-0.25269664897591,48.441991063746,0
-0.25282420524008,48.441947013307,0
-0.25294548845475,48.441895755413,0
-0.25305957555167,48.441837680189,0
-0.25316559824089,48.441773229645,0
-0.25326274961962,48.441702894311,0
-0.25335029031332,48.441627209506,0
-0.2534275541027,48.441546751259,0

-0.25349395299345,48.441462131927,0
-0.25354898169031,48.441373995533,0
-0.25359222144148,48.441283012865,0
-0.25362334322405,48.44118987637,0
-0.25364211024628,48.441095294882,0
-0.25364837974785,48.44099998823,0
-0.25364210408427,48.440904681756,0
-0.25362333108725,48.440810100799,0
-0.25359220369866,48.440716965171,0
-0.25354895888062,48.44062598368,0
-0.25349392580991,48.440537848737,0
-0.25342752337129,48.440453231086,0
-0.25335025696777,48.440372774698,0
-0.25326271467314,48.440297091874,0
-0.25316556275532,48.440226758585,0
-0.25305954060518,48.440162310084,0
-0.25294545510921,48.440104236842,0
-0.25282417450867,48.440052980808,0
-0.25269662179237,48.440008932049,0
-0.25256376767312,48.439972425785,0
-0.25242662320118,48.439943739834,0
-0.2522862320711,48.4399230925,0
-0.25214366268031,48.439910640911,0
-0.25199999999995,48.439906479826,0
-0.2518563373196,48.439910640911,0
-0.25171376792881,48.4399230925,0
-0.25157337679873,48.439943739834,0
-0.25143623232679,48.439972425785,0
-0.25130337820754,48.440008932049,0
-0.25117582549124,48.440052980808,0
-0.2510545448907,48.440104236842,0
-0.25094045939472,48.440162310084,0
-0.25083443724459,48.440226758585,0
-0.25073728532677,48.440297091874,0
-0.25064974303213,48.440372774698,0
-0.25057247662862,48.440453231086,0
-0.25050607419,48.440537848737,0
-0.25045104111929,48.44062598368,0
-0.25040779630122,48.440716965171,0
-0.25037666891265,48.440810100799,0
-0.25035789591564,48.440904681756,0
-0.25035162025203,48.44099998823,0
-0.25199999999995,48.441,0
</coordinates>
</LinearRing>
</outerBoundaryIs>
</Polygon>
</Placemark>
<Placemark>
<name>618</name>
<styleUrl>#stylehap2</styleUrl>
<Polygon>
<outerBoundaryIs>
<LinearRing>
<coordinates>
-0.25099999999998,48.4,0
-0.24935294923967,48.399999988247,0

-0.24935921369098,48.400095294899,0
-0.24937796558682,48.400189876386,0
-0.24940906228184,48.400283012881,0
-0.24945226717507,48.400373995548,0
-0.24950725150882,48.400462131941,0
-0.24957359686877,48.400546751272,0
-0.24965079836696,48.400627209517,0
-0.24973826848313,48.400702894321,0
-0.24983534153503,48.400773229653,0
-0.24994127874425,48.400837680196,0
-0.25005527385858,48.400895755419,0
-0.25017645928817,48.400947013311,0
-0.25030391270898,48.400991063749,0
-0.25043666408326,48.401027571463,0
-0.25057370304318,48.40105625859,0
-0.25071398658196,48.401076906791,0
-0.25085644699344,48.40108935891,0
-0.25099999999997,48.401093520174,0
-0.25114355300653,48.40108935891,0
-0.251286013418,48.401076906791,0
-0.25142629695676,48.40105625859,0
-0.25156333591668,48.401027571463,0
-0.25169608729096,48.400991063749,0
-0.25182354071179,48.400947013311,0
-0.25194472614136,48.400895755419,0
-0.25205872125569,48.400837680196,0
-0.25216465846493,48.400773229653,0
-0.25226173151683,48.400702894321,0
-0.25234920163298,48.400627209517,0
-0.25242640313119,48.400546751272,0
-0.25249274849115,48.400462131941,0
-0.25254773282487,48.400373995548,0
-0.25259093771812,48.400283012881,0
-0.25262203441314,48.400189876386,0
-0.25264078630898,48.400095294899,0
-0.25264705076027,48.399999988247,0
-0.25264078016082,48.399904681773,0
-0.2526220223036,48.399810100816,0
-0.25259092001516,48.399716965187,0
-0.25254771006639,48.399625983695,0
-0.25249272136865,48.399537848751,0
-0.25242637246879,48.399453231098,0
-0.25234916836232,48.399372774709,0
-0.25226169664882,48.399297091884,0
-0.25216462305903,48.399226758593,0
-0.25205868638767,48.399162310092,0
-0.25194469287069,48.399104236848,0
-0.25182351004939,48.399052980812,0
-0.25169606016846,48.399008932052,0
-0.25156331315821,48.398972425787,0
-0.25142627925382,48.398943739836,0
-0.25128600130846,48.398923092501,0
-0.25114354685836,48.398910640912,0
-0.25099999999997,48.398906479827,0
-0.2508564531416,48.398910640912,0
-0.25071399869148,48.398923092501,0
-0.25057372074614,48.398943739836,0

-0.25043668684175,48.398972425787,0
-0.25030393983148,48.399008932052,0
-0.25017648995057,48.399052980812,0
-0.25005530712924,48.399104236848,0
-0.24994131361226,48.399162310092,0
-0.24983537694094,48.399226758593,0
-0.24973830335112,48.399297091884,0
-0.24965083163762,48.399372774709,0
-0.24957362753117,48.399453231098,0
-0.24950727863132,48.399537848751,0
-0.24945228993357,48.399625983695,0
-0.24940907998478,48.399716965187,0
-0.24937797769634,48.399810100816,0
-0.24935921983915,48.399904681773,0
-0.24935294923967,48.399999988247,0
-0.25099999999998,48.4,0
</coordinates>
</LinearRing>
</outerBoundaryIs>
</Polygon>
</Placemark>
<Placemark>
<name>621</name>
<styleUrl>#stylehap2</styleUrl>
<Polygon>
<outerBoundaryIs>
<LinearRing>
<coordinates>
-0.245,48.352,0
-0.24335450133749,48.351999988266,0
-0.24336075989066,48.352095294918,0
-0.24337949412068,48.352189876405,0
-0.24341056151645,48.352283012899,0
-0.24345372569983,48.352373995565,0
-0.24350865822278,48.352462131957,0
-0.24357494106516,48.352546751286,0
-0.24365206981475,48.352627209531,0
-0.24373945750484,48.352702894333,0
-0.24383643908042,48.352773229663,0
-0.24394227645931,48.352837680204,0
-0.24405616414903,48.352895755425,0
-0.24417723537734,48.352947013316,0
-0.24430456868949,48.352991063753,0
-0.24443719496189,48.353027571465,0
-0.24457410477893,48.353056258591,0
-0.24471425611684,48.353076906791,0
-0.24485658227577,48.35308935891,0
-0.24500000000001,48.353093520174,0
-0.24514341772425,48.35308935891,0
-0.24528574388316,48.353076906791,0
-0.24542589522107,48.353056258591,0
-0.24556280503813,48.353027571465,0
-0.2456954313105,48.352991063753,0
-0.24582276462265,48.352947013316,0
-0.24594383585099,48.352895755425,0
-0.24605772354069,48.352837680204,0
-0.2461635609196,48.352773229663,0

-0.24626054249518,48.352702894333,0
-0.24634793018524,48.352627209531,0
-0.24642505893483,48.352546751286,0
-0.24649134177722,48.352462131957,0
-0.24654627430016,48.352373995565,0
-0.24658943848357,48.352283012899,0
-0.24662050587931,48.352189876405,0
-0.24663924010934,48.352095294918,0
-0.24664549866253,48.351999988266,0
-0.24663923397733,48.351904681793,0
-0.2466204938016,48.351810100835,0
-0.24658942082711,48.351716965205,0
-0.24654625160146,48.351625983713,0
-0.24649131472596,48.351537848767,0
-0.24642502835296,48.351453231113,0
-0.24634789700196,48.351372774723,0
-0.24626050771877,48.351297091896,0
-0.24616352560669,48.351226758603,0
-0.24605768876427,48.3511623101,0
-0.2459438026677,48.351104236854,0
-0.24582273404078,48.351052980817,0
-0.24569540425925,48.351008932056,0
-0.24556278233942,48.35097242579,0
-0.24542587756461,48.350943739837,0
-0.24528573180545,48.350923092501,0
-0.24514341159221,48.350910640912,0
-0.24500000000001,48.350906479826,0
-0.24485658840778,48.350910640912,0
-0.24471426819457,48.350923092501,0
-0.24457412243538,48.350943739837,0
-0.2444372176606,48.35097242579,0
-0.24430459574077,48.351008932056,0
-0.24417726595924,48.351052980817,0
-0.24405619733232,48.351104236854,0
-0.24394231123575,48.3511623101,0
-0.24383647439333,48.351226758603,0
-0.24373949228125,48.351297091896,0
-0.24365210299804,48.351372774723,0
-0.24357497164706,48.351453231113,0
-0.24350868527403,48.351537848767,0
-0.24345374839854,48.351625983713,0
-0.24341057917291,48.351716965205,0
-0.24337950619842,48.351810100835,0
-0.24336076602269,48.351904681793,0
-0.24335450133749,48.351999988266,0
-0.245,48.352,0
</coordinates>
</LinearRing>
</outerBoundaryIs>
</Polygon>
</Placemark>
<Placemark>
<name>622</name>
<styleUrl>#stylehap2</styleUrl>
<Polygon>
<outerBoundaryIs>
<LinearRing>

<coordinates>
-0.24400000000003,48.455,0
-0.24235116576666,48.454999988224,0
-0.24235743699536,48.455095294876,0
-0.24237620919049,48.455189876364,0
-0.24240733955247,48.45528301286,0
-0.24245059122431,48.455373995528,0
-0.24250563509239,48.455462131922,0
-0.24257205228954,48.455546751254,0
-0.24264933738118,48.455627209502,0
-0.24273690221089,48.455702894308,0
-0.24283408037572,48.455773229642,0
-0.24294013229727,48.455837680187,0
-0.24305425085027,48.455895755411,0
-0.24317556750537,48.455947013306,0
-0.24330315893979,48.455991063745,0
-0.24343605406524,48.45602757146,0
-0.24357324141971,48.456056258588,0
-0.24371367686688,48.45607690679,0
-0.24385629154429,48.45608935891,0
-0.24400000000003,48.456093520174,0
-0.24414370845579,48.45608935891,0
-0.2442863231332,48.45607690679,0
-0.24442675858034,48.456056258588,0
-0.24456394593481,48.45602757146,0
-0.24469684106026,48.455991063745,0
-0.24482443249468,48.455947013306,0
-0.24494574914978,48.455895755411,0
-0.24505986770279,48.455837680187,0
-0.24516591962433,48.455773229642,0
-0.24526309778916,48.455702894308,0
-0.2453506626189,48.455627209502,0
-0.24542794771051,48.455546751254,0
-0.24549436490766,48.455462131922,0
-0.24554940877575,48.455373995528,0
-0.24559266044758,48.45528301286,0
-0.24562379080956,48.455189876364,0
-0.2456425630047,48.455095294876,0
-0.24564883423339,48.454999988224,0
-0.24564255683796,48.45490468175,0
-0.24562377866345,48.454810100794,0
-0.24559264269116,48.454716965166,0
-0.24554938594855,48.454625983675,0
-0.24549433770323,48.454537848732,0
-0.24542791695549,48.454453231081,0
-0.24535062924773,48.454372774694,0
-0.24526306281583,48.454297091871,0
-0.24516588411148,48.454226758582,0
-0.24505983272946,48.454162310082,0
-0.24494571577861,48.45410423684,0
-0.24482440173965,48.454052980806,0
-0.24469681385583,48.454008932048,0
-0.24456392310759,48.453972425785,0
-0.24442674082391,48.453943739834,0
-0.24428631098707,48.4539230925,0
-0.24414370228905,48.453910640911,0
-0.24400000000003,48.453906479826,0

-0.24385629771103,48.453910640911,0
-0.24371368901299,48.4539230925,0
-0.24357325917614,48.453943739834,0
-0.24343607689246,48.453972425785,0
-0.24330318614422,48.454008932048,0
-0.2431755982604,48.454052980806,0
-0.24305428422144,48.45410423684,0
-0.24294016727059,48.454162310082,0
-0.24283411588857,48.454226758582,0
-0.24273693718422,48.454297091871,0
-0.24264937075232,48.454372774694,0
-0.24257208304457,48.454453231081,0
-0.24250566229682,48.454537848732,0
-0.24245061405153,48.454625983675,0
-0.24240735730889,48.454716965166,0
-0.2423762213366,48.454810100794,0
-0.2423574431621,48.45490468175,0
-0.24235116576666,48.454999988224,0
-0.24400000000003,48.455,0
</coordinates>
</LinearRing>
</outerBoundaryIs>
</Polygon>
</Placemark>
<Placemark>
<name>623</name>
<styleUrl>#stylehap2</styleUrl>
<Polygon>
<outerBoundaryIs>
<LinearRing>
<coordinates>
-0.24199999999996,48.408,0
-0.24035269015953,48.407999988243,0
-0.24035895559537,48.408095294895,0
-0.24037771044004,48.408189876383,0
-0.24040881202576,48.408283012878,0
-0.24045202371438,48.408373995545,0
-0.24050701669652,48.408462131938,0
-0.24057337249205,48.408546751269,0
-0.24065058613364,48.408627209515,0
-0.24073807000857,48.408702894319,0
-0.24083515832992,48.408773229652,0
-0.24094111220312,48.408837680195,0
-0.24105512524903,48.408895755418,0
-0.24117632974136,48.408947013311,0
-0.24130380321104,48.408991063749,0
-0.24143657546765,48.409027571462,0
-0.24157363598442,48.40905625859,0
-0.24171394159048,48.40907690679,0
-0.24185642441174,48.40908935891,0
-0.24199999999995,48.409093520174,0
-0.24214357558817,48.40908935891,0
-0.24228605840943,48.40907690679,0
-0.24242636401551,48.40905625859,0
-0.24256342453229,48.409027571462,0
-0.24269619678889,48.408991063749,0
-0.24282367025857,48.408947013311,0

-0.2429448747509,48.408895755418,0
-0.24305888779682,48.408837680195,0
-0.24316484166998,48.408773229652,0
-0.24326192999137,48.408702894319,0
-0.24334941386629,48.408627209515,0
-0.24342662750789,48.408546751269,0
-0.24349298330339,48.408462131938,0
-0.24354797628552,48.408373995545,0
-0.24359118797418,48.408283012878,0
-0.24362228955989,48.408189876383,0
-0.24364104440457,48.408095294895,0
-0.2436473098404,48.407999988243,0
-0.24364103825368,48.40790468177,0
-0.24362227744506,48.407810100812,0
-0.24359117026345,48.407716965184,0
-0.24354795351708,48.407625983692,0
-0.24349295616901,48.407537848748,0
-0.24342659683202,48.407453231096,0
-0.24334938058102,48.407372774707,0
-0.24326189510806,48.407297091883,0
-0.24316480624855,48.407226758592,0
-0.24305885291351,48.40716231009,0
-0.24294484146564,48.407104236847,0
-0.24282363958271,48.407052980811,0
-0.24269616965451,48.407008932052,0
-0.24256340176382,48.406972425787,0
-0.24242634630478,48.406943739835,0
-0.24228604629459,48.406923092501,0
-0.24214356943731,48.406910640912,0
-0.24199999999995,48.406906479827,0
-0.24185643056262,48.406910640912,0
-0.24171395370534,48.406923092501,0
-0.24157365369513,48.406943739835,0
-0.24143659823609,48.406972425787,0
-0.24130383034542,48.407008932052,0
-0.24117636041723,48.407052980811,0
-0.24105515853427,48.407104236847,0
-0.24094114708642,48.40716231009,0
-0.24083519375136,48.407226758592,0
-0.24073810489187,48.407297091883,0
-0.24065061941889,48.407372774707,0
-0.24057340316791,48.407453231096,0
-0.24050704383092,48.407537848748,0
-0.24045204648286,48.407625983692,0
-0.24040882973646,48.407716965184,0
-0.24037772255488,48.407810100812,0
-0.24035896174623,48.40790468177,0
-0.24035269015953,48.407999988243,0
-0.24199999999996,48.408,0
</coordinates>
</LinearRing>
</outerBoundaryIs>
</Polygon>
</Placemark>
<Placemark>
<name>625</name>
<styleUrl>#stylehap2</styleUrl>

<Polygon>
<outerBoundaryIs>
<LinearRing>
<coordinates>
-0.23199999999997,48.427,0
-0.23035207438865,48.426999988235,0
-0.23035834216448,48.427095294888,0
-0.23037710401778,48.427189876375,0
-0.23040821722753,48.42728301287,0
-0.23045144506719,48.427373995538,0
-0.23050645860444,48.427462131931,0
-0.23057283920285,48.427546751263,0
-0.23065008170632,48.42762720951,0
-0.23073759828254,48.427702894315,0
-0.23083472289571,48.427773229648,0
-0.23094071637505,48.427837680191,0
-0.23105477204001,48.427895755415,0
-0.23117602183993,48.427947013309,0
-0.23130354296088,48.427991063747,0
-0.23143636484974,48.428027571461,0
-0.23157347660197,48.428056258589,0
-0.2317138346567,48.42807690679,0
-0.23185637074059,48.42808935891,0
-0.23199999999998,48.428093520173,0
-0.23214362925937,48.42808935891,0
-0.23228616534324,48.42807690679,0
-0.23242652339797,48.428056258589,0
-0.2325636351502,48.428027571461,0
-0.23269645703905,48.427991063747,0
-0.23282397816,48.427947013309,0
-0.23294522795992,48.427895755415,0
-0.23305928362489,48.427837680191,0
-0.23316527710423,48.427773229648,0
-0.2332624017174,48.427702894315,0
-0.23334991829361,48.42762720951,0
-0.23342716079709,48.427546751263,0
-0.2334935413955,48.427462131931,0
-0.23354855493277,48.427373995538,0
-0.23359178277241,48.42728301287,0
-0.23362289598216,48.427189876375,0
-0.23364165783545,48.427095294888,0
-0.23364792561129,48.426999988235,0
-0.23364165167818,48.426904681762,0
-0.23362288385468,48.426810100805,0
-0.23359176504323,48.426716965176,0
-0.23354853214056,48.426625983685,0
-0.23349351423282,48.426537848742,0
-0.23342713008927,48.42645323109,0
-0.23334988497366,48.426372774702,0
-0.23326236679776,48.426297091878,0
-0.23316524164588,48.426226758588,0
-0.23305924870525,48.426162310087,0
-0.23294519463997,48.426104236844,0
-0.23282394745218,48.426052980809,0
-0.2326964298764,48.42600893205,0
-0.23256361235801,48.425972425786,0
-0.23242650566879,48.425943739835,0

-0.23228615321578,48.4259230925,0
-0.23214362310207,48.425910640912,0
-0.23199999999998,48.425906479826,0
-0.23185637689786,48.425910640912,0
-0.23171384678418,48.4259230925,0
-0.23157349433114,48.425943739835,0
-0.23143638764192,48.425972425786,0
-0.23130357012356,48.42600893205,0
-0.23117605254778,48.426052980809,0
-0.23105480535996,48.426104236844,0
-0.23094075129471,48.426162310087,0
-0.23083475835406,48.426226758588,0
-0.2307376332022,48.426297091878,0
-0.23065011502627,48.426372774702,0
-0.23057286991067,48.42645323109,0
-0.23050648576711,48.426537848742,0
-0.23045146785938,48.426625983685,0
-0.2304082349567,48.426716965176,0
-0.23037711614526,48.426810100805,0
-0.23035834832176,48.426904681762,0
-0.23035207438865,48.426999988235,0
-0.23199999999997,48.427,0
</coordinates>
</LinearRing>
</outerBoundaryIs>
</Polygon>
</Placemark>
<Placemark>
<name>626</name>
<styleUrl>#stylehap2</styleUrl>
<Polygon>
<outerBoundaryIs>
<LinearRing>
<coordinates>
-0.23099999999999,48.416,0
-0.22935243096579,48.41599998824,0
-0.22935869738658,48.416095294892,0
-0.22937745518138,48.41618987638,0
-0.22940856165994,48.416283012875,0
-0.22945178014696,48.416373995542,0
-0.22950678178127,48.416462131935,0
-0.22957314801696,48.416546751267,0
-0.22965037380726,48.416627209513,0
-0.22973787144701,48.416702894317,0
-0.22983497504452,48.41677322965,0
-0.22994094558893,48.416837680193,0
-0.23005497657432,48.416895755417,0
-0.23017620013777,48.41694701331,0
-0.2303036936651,48.416991063748,0
-0.23043648681319,48.417027571462,0
-0.23057356889629,48.417056258589,0
-0.23071389657934,48.41707690679,0
-0.2308564018202,48.41708935891,0
-0.231,48.417093520174,0
-0.2311435981798,48.41708935891,0
-0.23128610342065,48.41707690679,0
-0.23142643110371,48.417056258589,0

-0.23156351318681,48.417027571462,0
-0.23169630633489,48.416991063748,0
-0.23182379986222,48.41694701331,0
-0.23194502342567,48.416895755417,0
-0.23205905441106,48.416837680193,0
-0.23216502495547,48.41677322965,0
-0.23226212855298,48.416702894317,0
-0.23234962619273,48.416627209513,0
-0.23242685198303,48.416546751267,0
-0.23249321821872,48.416462131935,0
-0.23254821985303,48.416373995542,0
-0.23259143834005,48.416283012875,0
-0.23262254481861,48.41618987638,0
-0.23264130261341,48.416095294892,0
-0.2326475690342,48.41599998824,0
-0.23264129645983,48.415904681766,0
-0.23262253269845,48.415810100809,0
-0.23259142062156,48.415716965181,0
-0.23254819707458,48.415625983689,0
-0.23249319107243,48.415537848746,0
-0.23242682129371,48.415453231093,0
-0.23234959289286,48.415372774705,0
-0.23226209365438,48.415297091881,0
-0.23216498951849,48.41522675859,0
-0.23205901951243,48.415162310089,0
-0.23194499012582,48.415104236846,0
-0.23182376917289,48.41505298081,0
-0.23169627918861,48.415008932051,0
-0.23156349040836,48.414972425787,0
-0.23142641338522,48.414943739835,0
-0.2312860913005,48.4149230925,0
-0.23114359202621,48.414910640912,0
-0.231,48.414906479826,0
-0.23085640797376,48.414910640912,0
-0.23071390869949,48.4149230925,0
-0.23057358661477,48.414943739835,0
-0.23043650959163,48.414972425787,0
-0.23030372081139,48.415008932051,0
-0.2301762308271,48.41505298081,0
-0.23005500987417,48.415104236846,0
-0.22994098048756,48.415162310089,0
-0.2298350104815,48.41522675859,0
-0.22973790634561,48.415297091881,0
-0.22965040710713,48.415372774705,0
-0.22957317870629,48.415453231093,0
-0.22950680892756,48.415537848746,0
-0.22945180292541,48.415625983689,0
-0.22940857937843,48.415716965181,0
-0.22937746730154,48.415810100809,0
-0.22935870354016,48.415904681766,0
-0.22935243096579,48.41599998824,0
-0.23099999999999,48.416,0
</coordinates>
</LinearRing>
</outerBoundaryIs>
</Polygon>
</Placemark>

<Placemark>
<name>628</name>
<styleUrl>#stylehap2</styleUrl>
<Polygon>
<outerBoundaryIs>
<LinearRing>
<coordinates>
-0.22699999999998,48.418,0
-0.22535236614956,48.417999988239,0
-0.22535863281667,48.418095294891,0
-0.22537739134919,48.418189876379,0
-0.22540849905129,48.418283012874,0
-0.22545171923839,48.418373995541,0
-0.22550672303634,48.418462131934,0
-0.22557309188278,48.418546751266,0
-0.22565032071109,48.418627209512,0
-0.22573782179296,48.418702894317,0
-0.22583492921058,48.418773229649,0
-0.22594090392396,48.418837680193,0
-0.22605493939543,48.418895755416,0
-0.22617616772798,48.41894701331,0
-0.22630366627107,48.418991063748,0
-0.22643646464346,48.419027571462,0
-0.22657355211963,48.419056258589,0
-0.22671388532344,48.41907690679,0
-0.22685639617075,48.41908935891,0
-0.22699999999998,48.419093520174,0
-0.22714360382921,48.41908935891,0
-0.22728611467652,48.41907690679,0
-0.22742644788033,48.419056258589,0
-0.22756353535648,48.419027571462,0
-0.22769633372887,48.418991063748,0
-0.22782383227198,48.41894701331,0
-0.22794506060453,48.418895755416,0
-0.228059096076,48.418837680193,0
-0.22816507078938,48.418773229649,0
-0.22826217820697,48.418702894317,0
-0.22834967928887,48.418627209512,0
-0.22842690811718,48.418546751266,0
-0.22849327696362,48.418462131934,0
-0.22854828076158,48.418373995541,0
-0.22859150094865,48.418283012874,0
-0.22862260865077,48.418189876379,0
-0.22864136718329,48.418095294891,0
-0.22864763385038,48.417999988239,0
-0.22864136102904,48.417904681766,0
-0.22862259652927,48.417810100808,0
-0.22859148322822,48.41771696518,0
-0.22854825798064,48.417625983688,0
-0.22849324981436,48.417537848745,0
-0.2284268774245,48.417453231093,0
-0.22834964598536,48.417372774704,0
-0.22826214330456,48.41729709188,0
-0.22816503534851,48.41722675859,0
-0.22805906117356,48.417162310088,0
-0.22794502730102,48.417104236845,0
-0.2278238015793,48.41705298081,0

-0.2276963065796,48.417008932051,0
-0.22756351257554,48.416972425787,0
-0.22742643015991,48.416943739835,0
-0.22728610255502,48.4169230925,0
-0.22714359767496,48.416910640912,0
-0.22699999999998,48.416906479827,0
-0.22685640232497,48.416910640912,0
-0.22671389744492,48.4169230925,0
-0.22657356984006,48.416943739835,0
-0.22643648742442,48.416972425787,0
-0.22630369342033,48.417008932051,0
-0.22617619842066,48.41705298081,0
-0.22605497269894,48.417104236845,0
-0.2259409388264,48.417162310088,0
-0.22583496465145,48.41722675859,0
-0.2257378566954,48.41729709188,0
-0.2256503540146,48.417372774704,0
-0.22557312257546,48.417453231093,0
-0.2255067501856,48.417537848745,0
-0.22545174201932,48.417625983688,0
-0.22540851677174,48.41771696518,0
-0.22537740347066,48.417810100808,0
-0.22535863897092,48.417904681766,0
-0.22535236614956,48.417999988239,0
-0.22699999999998,48.418,0
</coordinates>
</LinearRing>
</outerBoundaryIs>
</Polygon>
</Placemark>
<Placemark>
<name>633</name>
<styleUrl>#stylehap2</styleUrl>
<Polygon>
<outerBoundaryIs>
<LinearRing>
<coordinates>
-0.19000000000005,48.377,0
-0.18835369346266,48.376999988256,0
-0.18835995508583,48.377095294908,0
-0.18837869851101,48.377189876395,0
-0.18840978115719,48.37728301289,0
-0.18845296653026,48.377373995556,0
-0.188507926021,48.377462131948,0
-0.1885742414041,48.377546751279,0
-0.18865140801972,48.377627209524,0
-0.18873883861297,48.377702894327,0
-0.18883586780247,48.377773229658,0
-0.18894175714356,48.3778376802,0
-0.18905570074835,48.377895755422,0
-0.18917683141902,48.377947013314,0
-0.18930422724833,48.377991063751,0
-0.18943691863691,48.378027571464,0
-0.18957389567349,48.37805625859,0
-0.1897141158226,48.378076906791,0
-0.18985651186069,48.37808935891,0
-0.19000000000006,48.378093520174,0

-0.19014348813944,48.37808935891,0
-0.19028588417751,48.378076906791,0
-0.19042610432664,48.37805625859,0
-0.19056308136319,48.378027571464,0
-0.19069577275178,48.377991063751,0
-0.19082316858111,48.377947013314,0
-0.19094429925175,48.377895755422,0
-0.19105824285654,48.3778376802,0
-0.19116413219765,48.377773229658,0
-0.19126116138713,48.377702894327,0
-0.19134859198041,48.377627209524,0
-0.19142575859603,48.377546751279,0
-0.19149207397913,48.377462131948,0
-0.19154703346984,48.377373995556,0
-0.19159021884292,48.37728301289,0
-0.19162130148909,48.377189876395,0
-0.19164004491428,48.377095294908,0
-0.19164630653747,48.376999988256,0
-0.19164003877385,48.376904681782,0
-0.19162128939481,48.376810100825,0
-0.19159020116226,48.376716965196,0
-0.19154701074004,48.376625983703,0
-0.19149204689077,48.376537848759,0
-0.19142572797222,48.376453231105,0
-0.19134855875165,48.376372774716,0
-0.19126112656304,48.37629709189,0
-0.19116409683635,48.376226758598,0
-0.19105820803245,48.376162310095,0
-0.190944266023,48.376104236851,0
-0.19082313795731,48.376052980814,0
-0.19069574566345,48.376008932054,0
-0.1905630586334,48.375972425789,0
-0.19042608664599,48.375943739836,0
-0.19028587208323,48.375923092501,0
-0.19014348199901,48.375910640912,0
-0.19000000000006,48.375906479826,0
-0.18985651800109,48.375910640912,0
-0.1897141279169,48.375923092501,0
-0.18957391335414,48.375943739836,0
-0.1894369413667,48.375972425789,0
-0.18930425433668,48.376008932054,0
-0.1891768620428,48.376052980814,0
-0.1890557339771,48.376104236851,0
-0.18894179196766,48.376162310095,0
-0.18883590316378,48.376226758598,0
-0.18873887343707,48.37629709189,0
-0.18865144124848,48.376372774716,0
-0.18857427202788,48.376453231105,0
-0.18850795310933,48.376537848759,0
-0.18845298926008,48.376625983703,0
-0.18840979883784,48.376716965196,0
-0.18837871060529,48.376810100825,0
-0.18835996122626,48.376904681782,0
-0.18835369346266,48.376999988256,0
-0.19000000000005,48.377,0
</coordinates>
</LinearRing>

</outerBoundaryIs>
</Polygon>
</Placemark>
<Placemark>
<name>634</name>
<styleUrl>#stylehap2</styleUrl>
<Polygon>
<outerBoundaryIs>
<LinearRing>
<coordinates>
-0.18700000000001,48.384,0
-0.18535346705927,48.383999988253,0
-0.18535972954282,48.384095294905,0
-0.18537847554489,48.384189876393,0
-0.18540956246492,48.384283012887,0
-0.18545275377631,48.384373995554,0
-0.18550772082466,48.384462131946,0
-0.18557404532715,48.384546751277,0
-0.18565122255455,48.384627209522,0
-0.18573866517124,48.384702894325,0
-0.18583570770431,48.384773229657,0
-0.18594161160758,48.384837680199,0
-0.1860555708823,48.384895755421,0
-0.18617671821142,48.384947013313,0
-0.18630413156093,48.38499106375,0
-0.18643684119801,48.385027571464,0
-0.18657383707255,48.38505625859,0
-0.18671407650572,48.385076906791,0
-0.1868564921271,48.38508935891,0
-0.18700000000001,48.385093520173,0
-0.18714350787292,48.38508935891,0
-0.1872859234943,48.385076906791,0
-0.18742616292746,48.38505625859,0
-0.18756315880201,48.385027571464,0
-0.18769586843912,48.38499106375,0
-0.1878232817886,48.384947013313,0
-0.18794442911772,48.384895755421,0
-0.18805838839244,48.384837680199,0
-0.1881642922957,48.384773229657,0
-0.18826133482878,48.384702894325,0
-0.18834877744549,48.384627209522,0
-0.1884259546729,48.384546751277,0
-0.18849227917538,48.384462131946,0
-0.1885472462237,48.384373995554,0
-0.1885904375351,48.384283012887,0
-0.18862152445513,48.384189876393,0
-0.1886402704572,48.384095294905,0
-0.18864653294074,48.383999988253,0
-0.18864026431443,48.383904681779,0
-0.18862151235622,48.383810100822,0
-0.18859041984766,48.383716965193,0
-0.18854722348518,48.383625983701,0
-0.18849225207665,48.383537848756,0
-0.18842592403736,48.383453231103,0
-0.18834874420397,48.383372774714,0
-0.18826129999132,48.383297091888,0
-0.18816425692084,48.383226758597,0

-0.18805835355498,48.383162310094,0
-0.18794439587621,48.38310423685,0
-0.18782325115307,48.383052980814,0
-0.18769584134038,48.383008932053,0
-0.18756313606349,48.382972425788,0
-0.18742614524004,48.382943739836,0
-0.18728591139539,48.382923092501,0
-0.18714350173012,48.382910640912,0
-0.18700000000001,48.382906479826,0
-0.18685649826989,48.382910640912,0
-0.18671408860463,48.382923092501,0
-0.18657385475997,48.382943739836,0
-0.18643686393653,48.382972425788,0
-0.18630415865963,48.383008932053,0
-0.18617674884695,48.383052980814,0
-0.18605560412381,48.38310423685,0
-0.18594164644504,48.383162310094,0
-0.18583574307918,48.383226758597,0
-0.18573870000869,48.383297091888,0
-0.18565125579605,48.383372774714,0
-0.18557407596268,48.383453231103,0
-0.18550774792337,48.383537848756,0
-0.18545277651486,48.383625983701,0
-0.18540958015236,48.383716965193,0
-0.18537848764382,48.383810100822,0
-0.18535973568561,48.383904681779,0
-0.18535346705927,48.383999988253,0
-0.18700000000001,48.384,0
</coordinates>
</LinearRing>
</outerBoundaryIs>
</Polygon>
</Placemark>
<Placemark>
<name>639</name>
<styleUrl>#stylehap2</styleUrl>
<Polygon>
<outerBoundaryIs>
<LinearRing>
<coordinates>
-0.13099999999997,48.443,0
-0.12935155534693,48.442999988229,0
-0.12935782509516,48.443095294881,0
-0.12937659285615,48.443189876369,0
-0.12940771586394,48.443283012864,0
-0.1294509573175,48.443373995532,0
-0.12950598818095,48.443462131926,0
-0.12957238968604,48.443546751258,0
-0.12964965651761,48.443627209505,0
-0.12973720065819,48.443702894311,0
-0.12983435586224,48.443773229644,0
-0.12994038272613,48.443837680189,0
-0.13005447431528,48.443895755413,0
-0.1301757623056,48.443947013307,0
-0.13030332359245,48.443991063746,0
-0.130436187317,48.444027571461,0
-0.1305733422563,48.444056258589,0

-0.13071374452071,48.44407690679,0
-0.1308563255004,48.44408935891,0
-0.13099999999998,48.444093520174,0
-0.13114367449953,48.44408935891,0
-0.13128625547921,48.44407690679,0
-0.13142665774365,48.444056258589,0
-0.13156381268293,48.444027571461,0
-0.1316966764075,48.443991063746,0
-0.13182423769436,48.443947013307,0
-0.13194552568465,48.443895755413,0
-0.13205961727383,48.443837680189,0
-0.13216564413772,48.443773229644,0
-0.13226279934177,48.443702894311,0
-0.13235034348235,48.443627209505,0
-0.13242761031389,48.443546751258,0
-0.13249401181897,48.443462131926,0
-0.13254904268243,48.443373995532,0
-0.13259228413601,48.443283012864,0
-0.13262340714378,48.443189876369,0
-0.13264217490479,48.443095294881,0
-0.13264844465302,48.442999988229,0
-0.1326421687421,48.442904681755,0
-0.13262339500566,48.442810100798,0
-0.13259226639126,48.44271696517,0
-0.13254901987024,48.442625983679,0
-0.13249398463246,48.442537848736,0
-0.13242757957912,48.442453231085,0
-0.13235031013314,48.442372774697,0
-0.13226276439144,48.442297091874,0
-0.13216560864825,48.442226758584,0
-0.1320595823235,48.442162310084,0
-0.13194549233546,48.442104236842,0
-0.13182420695956,48.442052980807,0
-0.13169664922099,48.442008932049,0
-0.13156378987074,48.441972425785,0
-0.1314266399989,48.441943739834,0
-0.1312862433411,48.4419230925,0
-0.13114366833686,48.441910640911,0
-0.13099999999998,48.441906479826,0
-0.13085633166309,48.441910640911,0
-0.13071375665883,48.4419230925,0
-0.13057336000105,48.441943739834,0
-0.13043621012921,48.441972425785,0
-0.13030335077897,48.442008932049,0
-0.13017579304039,48.442052980807,0
-0.13005450766449,48.442104236842,0
-0.12994041767643,48.442162310084,0
-0.1298343913517,48.442226758584,0
-0.12973723560849,48.442297091874,0
-0.12964968986682,48.442372774697,0
-0.12957242042084,48.442453231085,0
-0.1295060153675,48.442537848736,0
-0.12945098012971,48.442625983679,0
-0.12940773360869,48.44271696517,0
-0.12937660499426,48.442810100798,0
-0.12935783125786,48.442904681755,0
-0.12935155534693,48.442999988229,0

-0.13099999999997,48.443,0
</coordinates>
</LinearRing>
</outerBoundaryIs>
</Polygon>
</Placemark>
</Folder>
</Folder>
</Folder>
</Document>
</kml>

Supplementary Information II

Mayenne fire salamanders 14 microsatellite markers and 734 individuals in 41 populations
SalE8
G6
IIA6
E11
IA6
B11
SalE6
C3
Sal3
SalE7
SalE11
G9
SalE5
Sal29
POP
01_01 , 151163 229233 204204 299307 219223 185189 290294 224224 234234 229241 248252 237245 193193 175179
01_02 , 151163 229229 200224 243295 219219 189189 290294 220228 234234 241249 244248 237245 193193 175179
01_03 , 151163 229237 208220 243307 219219 189193 290290 224224 234234 229241 248252 245245 193193 179179
01_04 , 163163 229229 204204 239307 219219 185189 290290 224224 194234 229241 244248 245245 193193 171179
01_05 , 151159 229229 200204 299303 219219 185189 290290 220224 194246 229241 244248 245245 193193 171175
01_06 , 163167 229233 192220 239239 219223 185185 286294 216224 190234 237241 248248 241249 193193 171179
01_07 , 151163 229229 204220 239307 215219 173185 290290 224224 246246 229233 248248 237245 187187 175179
01_08 , 151163 229229 204220 243307 215219 185193 290290 224224 234246 229233 248248 237245 187193 175179
POP
02_01 , 151163 229233 204204 239307 219219 181185 290290 224224 246246 245245 244248 241245 193193 171171
02_02 , 163163 229229 200204 295307 219223 185189 290290 224224 234246 241241 244248 245245 187187 175179
02_03 , 163163 229229 192196 311311 219227 185189 290294 224224 194234 229241 244248 245245 193193 171175
02_04 , 163163 229229 196204 307311 219223 185189 290290 224224 234234 241241 244244 245245 193193 171171
02_05 , 151163 229233 200204 243295 223223 185193 290298 224228 194246 229245 248248 241249 187193 175179
02_06 , 151163 229233 204204 303311 219223 185189 290290 220224 194234 229241 248252 241245 193193 175183
02_07 , 151155 229229 204204 307307 219219 185189 290290 224224 234242 229241 244252 245245 187193 171179
02_08 , 151151 229229 204204 239307 219219 189193 290290 224224 234234 241241 244248 237245 193193 171183
02_09 , 163163 229229 204216 307315 219219 185185 290290 224224 234246 229229 244256 245245 187193 175179
02_10 , 163163 229229 196204 239307 219219 185189 290290 224224 234234 241241 244248 245253 187193 175179
02_11 , 163163 229229 196204 307311 223223 185189 290290 224224 234234 241241 244244 245245 193193 175179

02_12 , 163163 229229 192204 243307 219223 185193 290290 224224 234234 241241 244252 245245 193193 171175
02_13 , 151155 229229 204204 307307 219219 185189 290290 224224 234242 229237 244252 245245 187193 175183
02_14 , 163167 229229 200204 295315 219219 185189 290290 224224 234246 229229 248248 245245 187187 179179
02_15 , 159163 229233 204204 239307 215223 185189 290290 224224 194250 237241 248248 245253 193193 171179
POP
03_01 , 163167 221229 204204 239243 219219 185197 290294 224224 194234 229241 240248 241245 193193 179179
03_02 , 151163 225229 204204 299315 219219 181189 290290 220224 194246 229229 248252 245245 187193 175183
03_03 , 159163 225233 200204 243243 219219 181181 290290 220224 234234 225229 248252 245245 187193 175175
03_04 , 151159 229237 204224 303307 215223 181201 290290 224224 234234 229229 248248 245249 193193 171175
03_05 , 163163 233233 204208 239243 219219 189189 290290 224224 194234 237241 248248 241245 193193 171175
03_06 , 151167 229229 192192 243303 219223 177185 290290 224224 234234 229241 244248 241245 193193 175183
03_07 , 147163 229233 204208 239243 215219 185185 290294 224224 190234 241241 248248 249253 193193 155175
03_08 , 163163 233233 204204 239299 215219 185189 290290 224224 246250 237241 244248 245245 193193 171175
03_09 , 151151 233237 204204 239299 215219 185189 290290 224224 194246 229229 244244 245245 193193 171175
03_10 , 151151 233233 204204 239243 223223 189193 290290 224224 234234 229237 244248 245245 187187 171175
03_11 , 163163 229233 204208 303307 215223 185189 290290 224224 190194 241241 244248 245245 193193 171171
03_12 , 159187 225225 204204 239307 215215 189193 286286 224228 226250 241241 244252 245245 193193 179179
03_13 , 151159 229237 200208 299307 215219 185193 290294 224224 234246 229241 248252 245249 187193 171171
03_14 , 163167 229237 200204 239243 219227 185189 290290 224224 234234 241241 248248 245253 193193 175179
03_15 , 159163 229237 200208 243307 219219 189193 290298 228228 234234 241245 244248 245245 193197 171175
03_16 , 151163 229233 208220 239247 219223 181185 290290 220220 190234 237241 248248 245245 187187 179179
03_17 , 159167 229229 204204 243303 219219 185189 290294 224224 234234 241241 244248 245249 187193 179179
03_18 , 163163 221229 192192 239239 219219 185193 290294 220224 234234 229229 244244 241245 187193 179179
03_19 , 151163 229229 192192 239243 215219 185189 290294 224228 190234 233241 248248 245245 187193 179179
03_20 , 163163 225233 204204 239307 215219 185189 290290 224224 234234 241249 244248 237245 193193 175183
03_21 , 147163 233237 196200 247303 219219 185189 290290 220224 234234 229241 248248 245245 187193 171183
03_22 , 151163 229237 204204 295303 219219 185189 290290 220224 198250 229241 248248 245245 187193 171175
POP
04_01 , 151155 229233 192200 243307 219219 185193 290290 224224 190194 229241 244252 241245 193193 179179
04_02 , 163163 229233 192200 239243 219219 185189 290290 224224 234246 229241 244248 245249 193193

175183
04_03 , 163163 229229 192192 235299 215219 189189 290294 224224 194234 225241 248252 241245 193193
175179
04_04 , 163163 229233 204204 307307 219219 189193 286290 224224 234234 225241 248252 237245 187193
179179
04_05 , 163163 229229 192200 239243 219219 189197 290294 224224 234234 229241 248252 245249 193193
167179
04_06 , 151159 229229 204204 295303 219231 189189 290290 224224 234250 237241 248256 245249 193193
171179
04_07 , 151163 229233 200204 239239 215219 185193 290290 224228 234246 237241 244248 245249 193193
167179
04_08 , 151159 229229 204216 239239 219219 185193 286290 224228 234234 237241 244248 237249 193193
171179
04_09 , 151163 229229 192208 295299 215219 185189 290294 220220 234246 233241 248248 245249 193193
175179
04_10 , 163163 233233 192220 239303 215219 177193 290294 220224 194234 241241 248252 237245 193193
175175
04_11 , 151163 229229 204204 243295 219223 177189 290290 220224 234246 229229 244248 241245 193193
183183
04_12 , 151163 233233 204204 243303 219219 185189 286290 224224 234234 229229 248248 245245 187193
167175
04_13 , 151163 229233 204204 239243 219223 185189 290290 224224 194250 225229 244248 245245 193193
171175
04_14 , 159167 229229 204204 303303 215219 185189 290290 220224 234234 229241 248248 237249 187193
175175
04_15 , 163163 229233 192204 303315 219223 185193 290290 224224 234234 229245 244252 245245 187193
171171
04_16 , 151167 229229 192208 239243 223227 185189 290290 224224 234234 229241 244248 237249 193193
171175
POP
05_01 , 151163 229233 192200 239239 215223 189193 286290 224224 194234 229241 248248 245245 193193
175183
05_02 , 151163 229237 192204 243315 219219 185189 290290 224224 234250 229241 244248 245249 193193
167175
05_03 , 163167 229233 204204 239299 219223 185189 290294 220224 250250 229245 244248 237245 187193
171179
05_04 , 151163 229229 192204 239303 219223 181189 290290 224224 194234 229229 248248 241245 187193
179179
05_05 , 151163 229229 192204 239307 219223 181189 290294 224224 234250 229241 248252 237245 193193
175175
05_06 , 151163 229229 204220 239303 219219 189193 290290 224224 234234 241249 248252 245245 187193
175183
05_07 , 151163 229233 192204 243303 219231 185185 290290 220228 234234 241241 248248 245249 187193
175179
05_08 , 155167 225237 192204 299311 215219 189189 290290 216220 230234 237241 248248 245245 193193
179183
05_09 , 151167 233233 192204 239315 219219 189189 290290 224224 194234 241241 000000 245245 193193
175175
05_10 , 151163 229233 196208 243307 219231 185189 290290 224224 194234 241241 240244 245249 193193
171175
05_11 , 155163 229237 204220 299307 219219 189197 290294 224224 234250 241241 244248 245249 187193
175179
05_12 , 151171 225229 204204 239303 219219 185185 290290 224224 190234 237241 244252 241245 193193
171179
05_13 , 163163 229229 192204 299303 219231 189189 290290 224228 234234 229241 248248 245245 193193
171183
05_14 , 163163 229229 192200 239307 219219 189189 290294 220220 234234 241241 244252 249253 193193

175175
05_15 , 163163 229229 204220 239239 215219 185185 290290 224224 194234 241241 244248 249249 193193
175183
05_16 , 155163 229229 192204 303307 219219 185193 290294 224224 234246 241241 248256 237245 187193
175175
05_17 , 151151 229229 204220 239239 219223 189189 290294 224224 234234 241241 240252 245245 193193
175183
05_18 , 151167 229237 192220 303303 215219 185189 286294 224224 234250 241241 244248 245245 187193
171171
05_19 , 155163 229229 204204 295307 219219 185193 290294 224224 234234 241245 248256 237245 193193
175175
05_20 , 147155 229237 204204 239303 209227 189189 290290 224228 194194 241241 248252 249253 187193
167171
05_21 , 155163 229229 204204 243303 219223 177185 290290 224224 194194 229237 244248 245249 187193
171179
05_22 , 159163 229229 200208 243311 219219 185189 290294 224224 234234 229241 244248 253253 193193
167171
05_23 , 155163 229229 200204 247307 219219 189193 290290 220224 234234 225241 248252 245249 193193
175175
POP
06_01 , 151163 229229 200204 239307 215219 181185 290294 224224 234246 241245 248248 241245 193193
171179
06_02 , 163163 229229 192192 299299 219219 189193 290290 224224 234250 241241 248248 241249 193193
175183
06_03 , 151163 229229 192204 243299 215219 185189 290294 220224 234250 241241 248248 241249 187193
171183
06_04 , 159163 229229 212224 243303 219219 185185 290294 224224 234234 233241 244252 237245 193193
171179
06_05 , 163163 229229 204224 239299 219219 185197 290290 224228 234234 241241 244248 245245 193193
171179
06_06 , 163163 229233 192200 243311 219219 177197 290290 220228 234246 229229 244248 245249 193193
171179
06_07 , 159163 225229 192220 243303 219219 185189 290294 220224 194234 241241 248248 245245 187193
171183
06_08 , 163163 225225 204204 239303 223223 185189 290290 224224 234246 229241 248248 245245 193193
171171
06_09 , 147163 225233 192204 299303 215219 189189 290290 220224 246246 229241 240248 237245 193193
175175
06_10 , 151175 229229 192204 239307 215219 181189 290294 224224 234234 229245 244252 245245 193193
175175
06_11 , 151155 229229 192204 239239 215219 185189 290290 224224 234234 229229 248248 241245 193193
179183
06_12 , 159159 229229 192204 239243 219219 177185 290290 224224 230234 229233 248248 245249 193193
179183
06_13 , 151163 229229 208224 239299 219219 185197 286294 220224 234234 229241 244248 237249 187193
175175
06_14 , 163163 229233 204224 239299 215219 189197 290290 224228 234234 241241 244248 245245 187193
171179
06_15 , 163163 225237 192204 239239 215223 185193 290290 224224 234234 229229 248248 245245 193193
171183
06_16 , 163163 229229 192204 251299 219219 185193 286290 224224 190194 241241 252252 245245 187193
175183
06_17 , 151151 229233 192204 239239 219219 185185 290290 224224 234234 229241 248248 245245 193193
179179
06_18 , 155163 229237 200204 243303 215227 185189 290290 224224 190234 241241 240248 245249 193193
179183
POP

07_01 , 151163 229241 204204 239307 223223 185185 290290 228228 234234 229241 240248 245245 193193 175175
07_02 , 151163 229229 192204 239307 215219 189189 290290 224224 190234 229241 244248 245245 193193 163171
07_03 , 163163 225233 204204 239299 215219 185189 286290 224224 234246 241241 244248 237237 193193 171179
07_04 , 151163 229233 204208 307311 219219 185189 290290 224224 226234 229241 248248 245249 193193 171187
07_05 , 151163 229233 192204 239307 215219 185185 290290 224228 234246 229241 244248 241245 193193 175175
07_06 , 151163 229229 204204 307307 219219 185193 290290 224224 190234 241241 244248 245245 193193 163183
07_07 , 151167 233233 192204 239307 219219 185185 290290 224224 234246 229241 248248 241245 193193 175179
07_08 , 151155 229229 192204 239239 219231 189189 290290 224228 234234 241241 248248 237245 187193 171179
07_09 , 155167 229233 204208 239307 219223 185193 290298 224224 194234 229229 248264 245245 187187 163175
07_10 , 151163 233233 204220 243311 219231 185189 290290 224224 194234 229241 248248 245245 193193 179179
07_11 , 159167 229233 192220 239243 219219 189189 290290 224224 194234 241245 244248 237245 193193 179179
07_12 , 151163 229229 204204 239315 219223 173189 290290 224228 234234 241241 244248 245245 193193 175179
07_13 , 151163 233233 192204 243299 219231 189189 290290 224224 234246 237241 248248 245245 193193 175179
07_14 , 151155 229229 192200 239307 219223 193197 290290 224224 234238 225225 248248 245245 193193 171179
07_15 , 163163 229229 192204 303307 219231 181185 290298 224224 234234 229241 244248 245245 193193 171179
07_16 , 159163 229233 192208 295303 219219 185189 290290 224224 234234 241241 248248 245245 193193 171179
07_17 , 163163 229233 204204 239307 223231 185193 290290 224224 234234 229229 240248 245245 187193 179179
07_18 , 151159 229229 204204 239307 219227 189197 290290 224224 194234 229237 244248 245245 193193 175179
07_19 , 151167 225233 192204 239239 219231 177185 290290 220224 234250 241245 248248 245249 193193 171175
07_20 , 151163 225229 192204 247303 219223 189189 290294 224224 190234 241241 248252 245245 193193 175175
07_21 , 151159 229229 208216 247299 219219 185189 286290 224224 234234 229229 248252 237245 187187 183183
07_22 , 151163 229229 200200 239239 219223 185189 290298 220224 234234 229229 244248 245245 187193 171171
07_23 , 163163 229229 200204 239299 219219 185189 290290 220224 234234 225241 244248 245245 187193 171175
POP
08_01 , 159167 233233 204208 295303 215219 181189 286294 220224 234234 229241 244248 245245 193193 175187
08_02 , 159167 229229 192200 239239 215219 189189 290290 224224 194234 241241 248248 241245 187193 175175
08_03 , 163167 229233 192200 239307 219219 189193 290290 220224 234234 241241 248252 245245 193193 171175
08_04 , 151159 229233 204208 239307 219223 193197 290294 224228 234246 229241 248248 245245 193193 175179
08_05 , 163163 229233 200204 243307 223227 189193 290290 220224 194250 229241 248248 241245 193193 179179

08_06 , 155163 233237 192204 243295 219223 185193 290294 224224 234254 241249 244248 237245 187193
171175
08_07 , 155159 233233 192204 243295 223227 185189 290294 224224 234254 229241 244244 245245 187193
179183
08_08 , 147155 229233 204204 239307 219231 185197 290290 224224 234246 241245 248252 253253 193193
171175
08_09 , 167167 229233 192192 239239 215219 189189 290294 224224 234234 229245 248248 245249 193193
179179
08_10 , 151163 229233 192204 243307 215219 185189 290294 224224 190234 229241 244248 237245 193193
179179
08_11 , 151163 229229 192204 239315 219223 185189 290290 216224 234234 229241 248248 245245 187187
171175
08_12 , 163163 229229 192204 243307 223227 189189 290290 224224 234234 225241 248248 237249 187193
167175
08_13 , 155155 229237 200204 239303 215219 189189 290290 220224 194250 241241 248248 245245 193193
171175
08_14 , 163167 233233 200204 239299 219227 189189 290290 220224 234234 229241 248248 245245 193193
171179
08_15 , 155163 229233 204208 243299 219219 189193 290290 224224 194234 229229 244248 245245 193193
179179
08_16 , 163163 233237 204204 303307 215215 189189 290290 224224 234234 229245 248248 245245 187193
175183
08_17 , 159163 229233 192204 307307 223227 189193 290290 220224 234234 229229 248248 241245 193193
163179
POP
09_01 , 151155 229241 200204 239295 215219 185185 290294 220224 194246 225241 248256 245245 187193
179179
09_02 , 151159 229229 192192 239243 215219 189197 290290 224224 194234 225241 244248 245245 187193
171175
09_03 , 151159 225229 204204 239303 215219 185185 290294 224224 198234 229241 248248 237249 193193
179179
09_04 , 151163 237237 200204 239243 215219 185185 290290 224224 234234 229237 248248 237245 193193
179179
09_05 , 151163 229229 192200 239299 219219 189189 290290 220224 194234 229241 248252 245245 187193
163179
09_06 , 163167 229229 192204 243307 215223 185185 290290 224228 234246 225241 248248 237245 187193
171183
09_07 , 163163 229229 204220 239243 219223 189189 290294 224224 234234 241241 248260 241245 193193
179183
09_08 , 151159 229233 200204 239303 215219 177189 290290 224224 234246 233249 244248 237245 193193
175179
09_09 , 151163 229229 192204 239303 215219 185189 290290 224224 194234 229241 244248 245245 193193
171171
POP
10_01 , 147151 225229 200204 239303 219231 189189 290294 224224 190234 229229 244248 245249 193193
171175
10_02 , 151159 229233 200216 239303 219219 189197 290290 220224 234246 229237 240248 237245 193193
171171
10_03 , 143163 229229 192200 239307 219227 185189 286290 220224 194234 241249 244252 245245 193193
171171
10_04 , 151159 229229 200204 239239 215231 193193 290290 224224 234234 229241 244252 245253 187193
179183
10_05 , 151159 229233 200204 239243 219223 185193 290294 224224 234234 241241 248252 237245 187193
175183
10_06 , 163167 225229 204204 239295 219219 181189 290290 224224 194234 229241 248252 245245 193193
175179
10_07 , 147151 229233 200204 239243 219223 181189 290290 224228 234234 241241 248248 245249 187193

175179
10_08 , 155167 229237 204204 299307 215223 173189 290290 224224 194234 225241 244248 241245 193193
179179
10_09 , 151167 229237 192204 239303 215219 185189 294294 224224 194234 229229 248248 237245 187193
179179
10_10 , 151163 229229 192204 243307 219223 189189 290290 220228 194234 229241 248252 245245 193193
155175
10_11 , 163163 229229 192204 299311 219219 189193 290290 224224 234234 241245 248248 245245 193193
163179
10_12 , 151163 229233 192204 243307 215219 177181 290298 224228 234234 229241 248252 245245 193193
175179
10_13 , 151163 229229 192204 239303 219219 189193 290290 224224 190234 229241 248248 245249 187193
175175
10_14 , 155159 229233 204204 303311 219231 189193 290290 220228 194234 229229 244248 245245 187193
175183
POP
11_01 , 163163 225229 192204 243299 219219 185189 290298 228228 234246 229241 244244 237245 193193
175175
11_02 , 163163 229233 204208 239243 219227 181193 290294 220224 234238 241241 248252 245245 193193
179179
11_03 , 151163 225229 200204 243315 219219 189193 290294 224224 234250 241241 248252 245249 187193
175187
11_04 , 155163 229233 204204 239303 219219 177185 290290 224224 250250 217241 244248 245245 193193
179183
11_05 , 151151 229233 200204 243307 219223 189193 290290 224224 194234 229241 244248 245245 193193
171179
11_06 , 151163 225229 204208 243303 223223 189189 290290 220224 234234 241241 248248 245249 193193
175179
11_07 , 151151 229233 200204 243303 219223 185189 290290 220224 234234 229229 244252 249249 193193
175179
POP
12_01 , 163163 229233 208208 295307 219219 189189 290290 224224 234246 241241 244244 237245 193193
179183
12_02 , 151167 229229 200204 239307 219227 197197 290294 224224 234234 245245 248252 245245 187193
175179
12_03 , 151167 233233 200208 239239 215223 185189 290290 224224 234246 229241 244244 245245 187193
171179
12_04 , 151167 229233 192204 299307 215219 189189 290290 224224 194194 229241 248252 237237 187193
171175
12_05 , 151163 229229 200204 307311 219219 181193 290290 224224 234246 241245 248248 245245 193193
171179
12_06 , 159163 229233 204220 299303 219219 189189 290294 220224 234234 229241 244244 237245 193193
179179
12_07 , 151163 229229 204204 239307 215219 189189 286294 224224 194234 237241 244248 241245 193193
175183
12_08 , 151163 229233 208220 239247 219223 193201 290290 224224 234246 225229 248252 245245 193193
175179
12_09 , 163163 233237 200204 239239 219219 185189 290290 220224 234234 229241 244252 237245 187193
175175
12_10 , 151163 229229 204208 243303 219219 185189 290298 224224 190190 229241 248248 237237 193193
175179
12_11 , 159163 229229 204204 239307 219219 185189 290290 224224 234246 229241 248252 237249 187193
175179
12_12 , 163163 229229 200204 239307 219219 185189 286290 224224 234234 229241 248248 245249 193193
175179
12_13 , 163167 237237 192204 239307 219219 189193 290294 220224 234234 241241 244248 245245 193193
179179

12_14 , 155163 229229 192200 239303 215219 189189 290290 224224 194250 229245 248248 241245 187193 171171
12_15 , 163167 229229 204204 239295 219219 185189 286294 224224 234234 225229 248252 245245 193193 175175
POP
13_01 , 151155 229237 204204 299311 219219 189193 286290 224224 234234 245245 248248 245249 193193 171175
13_02 , 151167 229229 200204 239299 219219 189189 290290 224224 246246 229241 248248 245245 193193 175175
13_03 , 163163 233233 000000 299307 219219 185189 290290 224224 234234 241241 252252 245249 193193 171179
13_04 , 151167 229229 192204 299299 227227 185185 290290 224228 234234 229241 248248 245249 193193 171183
13_05 , 163163 233237 200204 239307 219223 185189 290290 220224 234234 237241 240248 237237 187187 171175
13_06 , 163163 229233 200204 307315 215219 189197 290290 220224 234246 241241 248248 237241 187193 171179
13_08 , 159163 229233 200208 243307 219219 189189 290290 224224 234246 229241 248248 237245 187193 171171
13_09 , 155163 229233 192204 307307 219227 185185 290290 220224 234250 241241 248252 237245 193193 167179
13_10 , 159163 229229 192204 307307 215219 185185 286294 224224 234246 241241 244252 249253 187193 175175
13_11 , 159163 225233 204208 299303 219227 185193 290290 224224 194234 241241 244248 237245 187193 175183
13_12 , 155155 233237 192200 299299 219219 185197 290294 220224 234246 237245 248248 245249 193193 167175
13_13 , 151151 229229 200200 243303 219219 177189 290294 220228 234234 229241 244252 245249 193193 171175
13_14 , 151163 233233 192204 239239 219219 189189 290290 224224 194234 229241 244248 237249 193193 179179
13_15 , 151151 229233 200204 299307 215219 185189 290290 220224 194234 229241 244248 245245 193193 175179
13_16 , 155163 229229 200204 243303 219223 181189 290294 224224 234234 225241 244248 237245 193193 163175
13_17 , 151155 229237 200208 239243 215231 185189 290290 224228 234250 229245 248248 241245 187193 171175
13_18 , 163163 229233 200204 307307 223231 185189 290290 224224 234234 233237 248248 237249 187187 171175
13_19 , 163163 225229 192208 303307 227227 189189 290290 220224 194250 241245 244248 241245 193193 171183
13_20 , 163163 229229 204204 307307 219223 189189 286290 224224 234234 229229 244252 245245 193193 167179
13_21 , 163163 229233 204208 239307 219223 189189 290290 224224 234234 237237 244248 245245 193193 171171
13_22 , 163167 229229 204204 243307 219223 189197 274290 224228 246250 229241 244244 245253 193193 171175
13_23 , 159163 229229 192204 299307 215219 181185 290290 220224 234234 241241 244248 245245 187193 175179
13_24 , 151163 229237 200204 243307 219219 185185 290290 224224 234250 233241 244248 245249 187193 171183
13_25 , 163163 229233 208208 299307 219231 185189 290290 224224 234234 229229 244248 245245 187193 179179
13_26 , 151151 229229 192200 239307 219219 181189 290290 224224 194250 229229 244248 245253 193193 175179
13_27 , 163163 229229 192208 295303 219227 185189 290290 220224 194250 229229 244244 237245 193193 179183

POP
14_01 , 151163 233233 204208 239239 219231 177181 290290 224224 234234 229241 244244 245245 187187 171171
14_02 , 151163 221229 192204 239307 215219 185189 290290 220224 194234 229241 244248 237245 187193 171179
14_03 , 163167 229229 192208 307307 219219 185189 290290 224224 194234 241245 248248 237245 193193 171175
14_04 , 163163 229233 192220 295303 215223 189193 290294 224228 234234 229241 244248 241245 187193 163171
14_05 , 143163 229229 200208 299315 215223 189189 290290 220224 234234 241245 244252 241245 193193 175179
14_06 , 163163 225229 200204 303307 215219 181189 290290 224224 190234 225245 252264 245245 187193 175179
14_07 , 151163 225229 200204 243299 219219 189189 290294 224224 234246 229241 248248 245245 193193 179183
14_08 , 155163 229233 204204 307311 215219 189189 290290 224228 194234 241245 244248 245245 187193 171175
14_09 , 155163 229229 200208 243315 219223 185189 278294 224224 234234 237245 248252 245245 187193 171179
14_10 , 151159 229229 192204 307307 219223 189197 290290 224224 234250 229229 248248 245245 187187 175175
14_11 , 163163 229229 204212 239247 219227 185189 290290 220224 234234 229245 248248 237245 193193 171175
14_12 , 151163 225229 192200 295307 219219 185189 290294 224224 234234 241249 248248 245245 193193 171179
14_13 , 151167 225229 192204 239307 219227 189189 286294 224224 190234 241241 244248 245249 193193 171183
14_14 , 151163 229229 200204 239307 219219 181185 290294 224224 234234 241245 244248 245245 193193 175183
14_15 , 151163 229229 204204 239303 219219 185189 290294 220224 234234 229241 244256 237241 187193 175175
14_16 , 159163 225229 192204 295295 219227 189189 286290 224228 234246 241245 248252 245245 187193 175179
14_17 , 151163 225229 204204 239307 219219 185193 290290 212228 234234 241245 248252 245245 187193 179179
14_18 , 151163 225229 192208 243307 219223 185193 290290 220224 190234 229241 244248 245245 193193 179179
14_19 , 163167 225229 200204 243307 219227 177185 290290 224224 234234 229241 244248 237245 193193 175183
14_20 , 155159 229229 192204 239307 215223 181185 290290 224224 234234 241241 244248 245245 187193 171175
14_21 , 151163 225229 192204 307307 219223 185189 290290 220224 234250 245257 248252 245245 193193 175187
14_22 , 151167 229233 204204 239243 219223 177189 290290 220224 234246 229241 248248 237241 187193 179183
14_23 , 151155 229233 204208 239311 219223 189197 290290 220224 234246 241241 240248 241245 193193 171179
14_24 , 163167 229233 204204 239243 219223 177197 290290 224224 234234 229241 248248 245245 187193 171179
14_25 , 163167 229229 204220 243307 219219 185189 290290 220224 234234 241245 244256 245245 193193 175179
POP
15_01 , 151163 225229 200204 239303 219231 185189 290298 224224 194234 229229 248248 245249 187193 179179
15_02 , 151159 225229 204204 239243 219223 189189 290294 220220 234234 241241 244244 245245 193193 175183
15_03 , 151163 229229 192212 247295 215219 189189 290290 220224 234234 229241 248248 245245 193193

175175
15_04 , 151163 229229 204208 239307 219219 185185 290294 224224 234234 229229 248252 245249 193193
155179
15_05 , 151163 229229 192204 000000 219223 185189 290290 224228 190234 229241 248248 237245 193193
171179
15_06 , 147167 229233 204204 239303 219223 189193 290290 224224 250250 229241 248248 245245 193193
179179
15_07 , 151159 229229 204208 239311 219223 185189 290290 224224 190234 229241 252252 245245 193193
175175
15_08 , 151155 229229 200204 239303 219219 189189 290290 220224 194250 229241 244248 241245 187193
179179
15_09 , 151163 229229 192204 239239 219219 189189 290290 224224 238246 229241 244248 245249 193193
175183
15_10 , 151159 229229 204208 247303 219223 185189 290290 224224 190234 229233 252252 245245 193193
175175
15_11 , 163163 225229 192200 243307 215223 189189 290290 224224 238250 229241 244244 237237 187193
171175
15_12 , 155163 225229 192208 239307 219219 189189 290290 224228 234234 237245 244248 241245 193197
175179
15_13 , 151167 229229 204208 303311 219223 185189 290294 224224 190234 233241 252252 245245 193193
175179
15_14 , 155163 229229 192200 239315 215219 189189 290294 220224 234234 229237 248252 241245 193193
175179
15_15 , 151163 229229 204204 243307 219219 185193 290290 220224 234234 241241 244248 245249 187187
175183
15_16 , 151163 233233 200204 239311 219219 177185 286290 220228 194234 241245 244248 245245 193193
179179
15_17 , 151167 229229 192200 239239 219219 189193 286290 224228 234234 229229 244256 245249 187193
175175
15_18 , 159163 229233 204204 311315 215219 193193 290294 224224 194234 229241 000000 245245 193193
175179
15_19 , 159167 225233 204204 239299 219219 189189 286294 224224 234246 241241 244248 249253 187193
179179
15_20 , 155159 225229 192212 239243 219219 181185 290290 220228 234234 241249 244244 245249 193193
179179
15_21 , 155167 225229 192204 299307 219223 181189 290290 224224 234238 241245 252252 245245 187193
179179
15_22 , 151163 225229 192204 243307 219219 189189 290290 224224 234234 229241 244248 245245 187193
175183
15_23 , 151151 225229 204204 239311 215219 189189 286290 220228 234258 241241 244248 237245 193193
175179
15_24 , 151163 225229 192204 243307 219219 189189 290290 220224 190234 229241 244248 245249 187193
175179
POP
16_01 , 163167 225225 204204 303311 219219 185189 290294 224224 186246 237241 248252 245245 193193
175179
16_02 , 151151 229237 204220 243303 219219 189189 290294 224224 234234 229241 248252 245245 187193
175179
16_03 , 151163 229229 200204 239307 219219 189193 290294 220224 194234 229241 244244 245249 187193
171179
16_04 , 151159 229229 192204 307311 215219 189193 290290 224224 234234 241245 252252 245245 193193
175183
16_05 , 163163 225229 192192 239303 219219 189189 290290 224224 234238 229241 244248 245245 193193
171179
16_06 , 151163 229229 200204 303303 219219 185189 290290 224228 234234 229241 240248 245245 187193
175179
16_07 , 151167 229237 204204 239299 219219 185193 290290 224228 194234 229245 248252 245245 187193

171179
16_08 , 159163 229233 192204 239307 223223 189193 290290 220224 194234 229241 244248 245245 187193
175179
16_09 , 159163 229229 204204 243311 215219 189189 290290 224224 190234 241245 252252 241245 187193
175179
16_10 , 151167 233237 192204 239299 223223 185189 290290 220224 234238 229237 248248 249249 193193
175175
16_11 , 163167 229229 200204 239303 223227 185189 290294 224224 234234 241245 244248 245245 193193
175179
16_12 , 151163 233233 200204 307315 219219 189193 290290 220224 234250 241245 244248 245245 187193
167175
16_13 , 151163 229229 204204 307307 215219 185189 290290 224224 194234 229241 248252 245245 193193
179179
16_14 , 163167 225229 192200 243307 219227 185189 290294 220224 194234 229237 244252 245245 187193
179179
16_15 , 163171 229229 204204 239299 219219 189189 290290 224228 234246 241245 248248 245249 187193
171175
16_16 , 151159 229229 204204 243311 219219 185189 290290 224224 190238 241245 252252 245245 187193
175179
16_17 , 151159 229229 204204 243299 215219 185189 290290 224224 194234 229241 248252 245245 193193
175179
16_18 , 163167 225229 204204 239299 219219 185189 290294 224224 194234 237241 248252 245249 193193
175179
16_19 , 151151 225225 204204 299303 215219 185185 290290 224224 234234 229241 244248 245245 193193
171175
POP
17_01 , 159163 229229 192204 239295 219223 177189 290298 224224 234246 241241 248248 241245 193193
179179
17_02 , 151163 229233 200204 243307 215219 185189 290290 224224 234234 229249 248252 237245 187193
175183
17_03 , 151167 229229 192204 239307 219219 189189 290290 224224 234234 229241 244248 245245 193193
171175
17_04 , 151159 229241 200204 239307 219231 177189 290294 224224 190234 229241 244248 249249 193193
179179
17_05 , 151163 229229 192204 243307 219219 185189 290290 224224 234234 241241 248248 245245 193193
175179
17_06 , 151163 229229 204204 239307 219219 185189 290290 224224 234234 229241 248248 245245 187193
000000
17_07 , 151163 229229 192204 239303 215219 185189 290290 224224 194234 229241 248248 237245 187193
163171
17_08 , 163163 229229 192192 303303 219223 189189 290294 224224 234234 241245 244248 245245 187187
175183
17_09 , 151151 229229 204204 243299 219231 189189 290290 216224 194234 241241 244248 237245 193193
171171
17_10 , 163167 229229 204220 239243 219231 189189 290290 216224 194234 229241 244248 245245 193193
175175
17_11 , 151171 229229 204212 303311 219223 185185 286290 224228 234234 225241 252252 245245 187187
179179
17_12 , 147151 225229 192204 239239 219219 189193 290290 220224 194234 245245 252252 237245 187187
163179
17_13 , 163167 229233 204204 239239 215219 189197 294294 224224 234234 229245 244248 245245 193193
179179
17_14 , 151163 229233 204204 243307 219219 185193 290290 224224 234234 229241 248248 245249 193193
171171
17_15 , 151163 225229 208224 239307 219227 189193 290290 224228 234262 233241 244248 245253 187187
175179
17_16 , 151163 229233 192204 239299 215219 185189 290290 224224 194246 229229 248248 245245 187193

175179
17_17 , 159163 229233 192204 239303 219219 189197 290290 220224 234234 241245 248252 245249 193193
171175
17_18 , 151163 225225 192204 239247 219219 185189 290290 224224 234234 229241 248248 241245 193193
175175
17_19 , 167167 229229 200204 243307 215219 189189 290298 224224 194234 241241 244252 237249 187193
171171
17_20 , 163163 229229 204204 239307 219219 185189 290290 224228 234234 241241 248248 245249 193193
175179
17_21 , 151163 221229 204204 243303 219219 185189 290290 224224 234246 241241 244244 237245 187193
175179
17_22 , 151167 229229 192204 303307 219219 189193 290290 220224 234234 241241 248248 241245 193193
179183
POP
18_01 , 163167 225229 192204 239239 219219 189193 290290 224224 234246 241241 248248 245245 187193
171175
18_02 , 163167 229229 204208 307307 219219 189193 290294 224228 234246 241245 244248 245245 187193
179183
18_03 , 163163 233237 204204 295307 215219 185193 290290 224228 194234 229229 248248 245249 193193
171175
18_04 , 155155 225229 204220 239311 215219 185189 290290 224224 234246 229241 248248 237245 193193
175175
18_05 , 151163 229233 192204 299311 219219 181189 290290 224228 234234 241241 248252 245245 187187
171179
18_06 , 163167 229229 200208 243311 219219 193193 290290 224224 234234 229241 248248 245249 193193
179179
18_07 , 151163 229229 200200 311319 219219 189193 290290 224228 234234 229241 244248 245245 193193
163171
18_08 , 163167 233237 200204 295307 219223 189193 290294 224224 194234 229241 248248 245245 193193
159175
18_09 , 151151 229237 192220 239243 219231 189193 286290 224228 234234 241245 248248 245245 187193
179183
18_10 , 163163 233237 200204 243311 219223 185189 290294 224224 234234 241241 248252 245245 193193
171175
18_11 , 151163 229237 192220 235239 209219 189193 290294 220228 234234 241241 248248 245249 187193
167183
18_12 , 159163 225233 192204 239303 219219 189197 290290 224224 194234 229237 248252 245245 193193
175179
18_13 , 163163 229229 192204 243307 215219 185193 290290 224224 234246 229229 244244 245245 187187
175179
18_14 , 155155 229229 204220 239303 215215 189193 290290 224224 194246 225229 248248 237245 193193
175175
18_15 , 151155 229229 200204 239307 219219 177185 286290 224224 194246 241245 248248 245249 193193
159175
POP
19_01 , 151163 229229 204204 239243 219223 189189 290290 224224 246250 229245 248248 237245 193193
171179
[truncated: 303,006 more chars]
